# The germline of the malaria mosquito produces abundant miRNAs, endo-siRNAs, piRNAs and 29-nt small RNAs

**DOI:** 10.1186/s12864-015-1257-2

**Published:** 2015-02-19

**Authors:** Leandro Castellano, Ermanno Rizzi, Jonathan Krell, Manlio Di Cristina, Roberto Galizi, Ayako Mori, Janis Tam, Gianluca De Bellis, Justin Stebbing, Andrea Crisanti, Tony Nolan

**Affiliations:** Department of Life Sciences, South Kensington Campus, Imperial College London, London, SW7 2AZ United Kingdom; Division of Oncology, Department of Surgery and Cancer, Imperial Centre for Translational and Experimental Medicine, Imperial College, London, UK; Istituto di Tecnologie Biomediche, Consiglio Nazionale delle Ricerche (ITB-CNR), Segrate, Milan, Italy; Department of Chemistry, Biology and Biotechnology, University of Perugia, Perugia, Italy; Dipartimento di Medicina Sperimentale Via Gambuli, Centro di Genomica Funzionale, University of Perugia, 06132 Perugia, Italy

**Keywords:** Mosquito, miRNAs, Small RNAs, piRNAs, Gametogenesis, Malaria, Genome defence, Transposons, Germline

## Abstract

**Background:**

Small RNAs include different classes essential for endogenous gene regulation and cellular defence against genomic parasites. However, a comprehensive analysis of the small RNA pathways in the germline of the mosquito *Anopheles gambiae* has never been performed despite their potential relevance to reproductive capacity in this malaria vector.

**Results:**

We performed small RNA deep sequencing during larval and adult gonadogenesis and find that they predominantly express four classes of regulatory small RNAs. We identified 45 novel miRNA precursors some of which were sex-biased and gonad-enriched , nearly doubling the number of previously known miRNA loci. We also determine multiple genomic clusters of 24-30 nt Piwi-interacting RNAs (piRNAs) that map to transposable elements (TEs) and 3’UTR of protein coding genes. Unusually, many TEs and the 3’UTR of some endogenous genes produce an abundant peak of 29-nt small RNAs with piRNA-like characteristics. Moreover, both sense and antisense piRNAs from TEs in both *Anopheles gambiae* and *Drosophila melanogaster* reveal novel features of piRNA sequence bias. We also discovered endogenous small interfering RNAs (endo-siRNAs) that map to overlapping transcripts and TEs.

**Conclusions:**

This is the first description of the germline miRNome in a mosquito species and should prove a valuable resource for understanding gene regulation that underlies gametogenesis and reproductive capacity. We also provide the first evidence of a piRNA pathway that is active against transposons in the germline and our findings suggest novel piRNA sequence bias. The contribution of small RNA pathways to germline TE regulation and genome defence in general is an important finding for approaches aimed at manipulating mosquito populations through the use of selfish genetic elements.

**Electronic supplementary material:**

The online version of this article (doi:10.1186/s12864-015-1257-2) contains supplementary material, which is available to authorized users.

## Background

In recent years additional layers of complexity have been revealed in the regulation of gene expression following the discovery in animals of several classes of small RNA molecules that can act at both the transcriptional and post-transcriptional level. Many of these small RNAs themselves show tissue-specific expression and have been shown to be essential for correct organogenesis and developmental progression. *Anopheles gambiae* mosquitoes are the major vectors of *Plasmodium* malaria parasites. Successful malaria control initiatives in the past have all relied on reducing the reproductive capacity of mosquito populations. Therefore a better understanding of the processes that regulate sexual development and, in particular, gonadogenesis and gametogenesis could provide novel targets for vector control. Small regulatory RNAs such as microRNAs (miRNAs) and other classes of small non-coding RNAs play a role in the germline of many organisms in germline stem cell maintenance and in restricting the expression of transposable elements yet little is known about the diversity of small RNAs and their contribution in the malaria mosquito [1-3].

Each of the different small RNA classes are characterised by their ability to interact with Argonaute (AGO) proteins, all of which are involved in gene silencing mechanisms [4]. Studies, principally performed with the fruitfly *Drosophila melanogaster*, indicate that in the fly AGO proteins can be divided into two different clades. One clade contains *AGO1* and *AGO2* that are expressed ubiquitously and function in gene silencing through binding with microRNAs (miRNAs) and small interfering RNAs (siRNAs), respectively [5]. The second clade contains the Piwi proteins, specifically expressed in the germline, composed of *AGO3*, *Piwi* and *Aubergine* (*Aub*) that bind to Piwi interacting RNAs (piRNAs).

miRNAs are a large class of ~21-24-nt small RNAs produced by *DICER1* processing of endogenously expressed RNA hairpin structures [6,7] that are involved in post-transcriptional gene repression [5,8]. After *DICER1* processing, the derived mature duplexes are unwound, loaded onto *AGO1* to guide it on the gene targets that are recognized through incomplete base pairing with the loaded single stranded, miRNA, enabling AGO1 to repress protein translation and/or destabilise the mRNA transcript [5,8]. On the other hand, siRNAs are exactly 21-nt long RNA molecules and produced by sequential *DICER2* cleavage of long double strand RNAs (dsRNAs) [9]. They are then unwound and loaded onto *AGO2* as a single stranded guide siRNA [4]. The complete complementarity between the loaded siRNA and the target permits AGO2 to mediate the cleavage of the target, that occurs opposite to the 10^th^ and the 11^th^ nt of the annealed siRNA [10]. piRNAs are, instead, 24-29–nt long and are particularly expressed in the germline, from discrete genomic loci and have been shown in several organisms to be involved in the silencing of genomic repeats and active transposable elements (TEs) [2,11,12]. Their biogenesis does not depend on *DICER* enzymes, but they are generated by a primary biogenesis pathway that is not completely understood and by a secondary biogenesis pathway called the ping-pong mechanism: piRNAs derived from one genomic strand generate the 5’ end of new piRNAs from the opposite strand due to the endonucleolytic activity of the *Piwi* proteins [2,13]. So far miRNAs have been exclusively involved in silencing of endogenous gene targets while siRNAs are involved in the repression of host genes, TEs and viruses. piRNAs are predominantly involved in the silencing of TEs, although a few examples of control of non-TE elements may exist (reviewed in [14]). Indeed in the mosquito *Aedes aegypti*, in addition to canonical RNAi-mediated silencing of viruses [15-17], piRNA-like molecules have also been implicated in this process.

Thus, while the diversity of small RNA populations and their function can be broadly conserved across a wide range of animals, species-specific differences are often revealed [18,19].

A few studies have attempted to identify computationally the microRNA pool in Anopheline mosquitoes yet only a small fraction of these microRNAs have been confirmed by sequencing or other approaches and the microRNA complement for *A. gambiae* is much smaller than that described for *Drosophila*, suggesting that it is far from completely described [20-25]. Moreover, two *Dicer* enzymes, *DCR1* and DCR2, and 5 *AGO* proteins *AGO1-*5 have been identified in *A. gambiae* suggesting that all the genetic machinery is there to process the full range of small regulatory RNAs [26]. Due to this lack of knowledge we set out to clone and analyse the small RNA populations present during the formation of the gonads in each sex to evaluate the contribution of each pathway to this essential process.

We identify a large number of novel miRNAs, some of which are sex-biased and/or enriched during gametogenesis, some of which represent new miRNA gene clusters, and others an expansion of existing clusters. We also identify endo-siRNAs derived from overlapping, convergently transcribed protein coding genes (cis-NAT-siRNAs) [27] and TEs and 5’-half-tRNAs that are 32-nt small RNAs formed by the processing of tRNA hairpin [28,29] whose expression was significantly downregulated in pre-vitellogenic ovaries. We finally identified in the gonads a large class of piRNAs, predominantly derived from TEs, for which we described novel sequence bias that may be relevant for piRNA recognition, loading and/or biogenesis.

This is the first report describing the complexity of the small RNA transcriptome during the essential processes of gonadogenesis and gametogonesis in the mosquito and the predominant role of piRNAs in limiting transposon proliferation in the germline is relevant to vector control approaches that propose these elements as agents to modify mosquito populations.

## Results and discussion

### Characteristics of the *Anopheles gambiae* small RNA population

**Figure 1:**
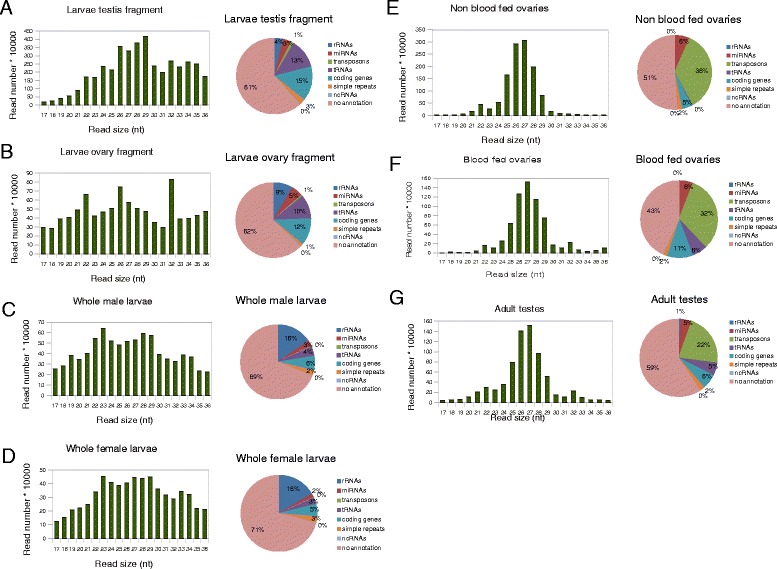
**Characteristics of** ***Anopheles gambiae*** **small RNAs.** The bar plot on the left shows size distribution of *Anopheles* small RNAs whereas the pie chart summarizes the annotation of *Anopheles* small RNA populations in **(A)** Larvae testis fragment, **(B)** Larvae ovary fragment **(C)** Whole male larvae, **(D)** Whole female larvae, **(E)** Non blood fed ovaries, **(F)** Blood fed ovaries and **(G)** Adult testes.

*Anopheles gambiae* adult female mosquitoes, in the absence of a bloodmeal remain in reproductive diapause in which the ovaries are arrested in the pre-vitellogenic phase of oogenesis. Post-bloodfeeding, oogenesis resumes and oocyte development is usually complete within 48 hours. By comparing ovaries in these two states we could examine global changes in small RNA profiles during female gametogenesis. To look at the process of gonadogenesis we compared in each sex adult gonads with larval gonads from L3 larval instars. Larval gonads at this developmental stage are rudimentary yet already sexually dimorphic ([30]; Nolan unpublished). We were unable to reliably dissect larval gonads so we enriched for this tissue by excising the larval segment that contained the gonad pair in each case. In total we isolated total RNA from non-blood fed ovaries, blood-fed ovaries, male testes, larval testes fraction, larval ovary fraction, whole male larvae and whole female larvae. We enriched for the small RNA fraction (16-40 nt) and made cDNA libraries that were subjected to high throughput sequencing on the Illumina platform. A total of approximately 120 million mapped reads, ranging from 17-36-nt, aligned perfectly to the *Anopheles gambiae* genome (*AgamP3.7,*https://www.vectorbase.org/organisms/anopheles-gambiae/pest/agamp3), with a minimum of 4 million mapped reads per condition (Additional file 1). We mapped these sequences to different categories of RNAs and performed a size distribution analysis in order to evaluate both the nature and size of the sequenced small RNAs in each class (Figure 1A-G). Most of the reads mapped to unannotated regions (40-70%) probably due the scarcity of annotated elements for *Anopheles gambiae* (Figure 1A-G). In whole larvae of both sexes we noticed a marked increase in the rRNA population and less clearly defined peaks of small RNA populations (Figure 1C-D) suggesting that there was a higher contribution of non-specific RNA degradation products to the total population in these samples. In the adult gonads of both sexes reads mapping to transposons dramatically increased relative to the larval conditions, concomitant with an increased average size of small RNA (most prominent in the range 24 to 30-nt) consistent with the known size distribution of piRNAs in other organisms, suggesting that this class represents the majority of the small RNAs produced in these tissues (Figure 1E, F and G). Interestingly, a 32-nt peak is clearly visible in vitellogenic ovaries as well as adult testes, but is absent in pre-vitellogenic ovaries (Figure 1E, F and G). The discrete 22-nt peak seen in the same tissues is consistent with representing the miRNA population of the gonads (Figure 1E, F and G). Each putative population of small RNAs was subsequently investigated further for their sequence composition and expression profile.

### Novel miRNAs discovered in the mosquito

In the analysed tissues we confirmed the expression of all the 67 precursors producing miRNAs from *Anopheles gambiae* annotated on the last release of miRBase (Release 20) database (Additional file 2). Apart from aga-miR-929 and aga-miR-219 that presented 2 and 5 total reads respectively, all the other miRNAs ranged from 28 to 974,600 reads and were expressed in multiple tissues (Additional files 3 and 4). The most expressed miRNA corresponded to aga-miR-263a (Additional file 4). In addition the top 20 most expressed miRNAs represent 96.3% of all the miRNA reads. Mapping the reads derived from the analysed samples we could also identify 62 previously unidentified “star” miRNA molecules that map on the opposite arm and with 2-nt 3’ overhang of the miRNA precursor respect to the known miRNA (Additional files 2 and 3).

**Figure 2:**
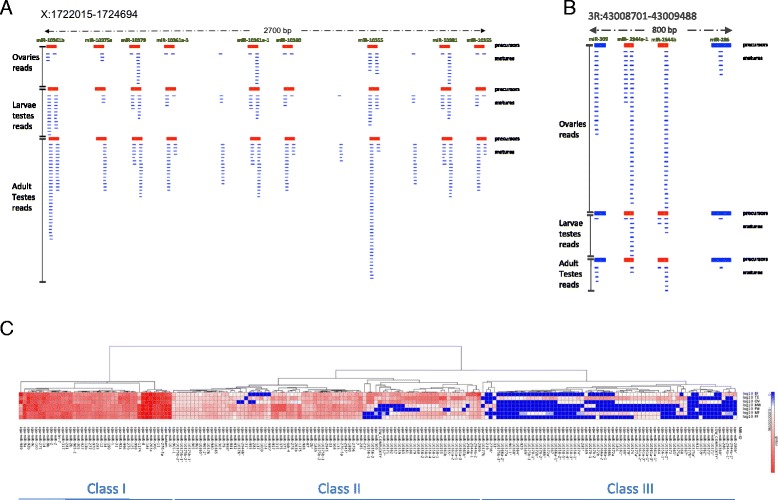
**Novel miRNAs are often localized in clusters and many show gonad-restricted or sex-biased expression. (A)** Cluster of 9 novel discovered miRNAs displayed as reads of small RNAs from ovaries, larvae testes and adult testes that map to novel miRNA precursor (shown in red). **(B)** An example of 2 novel discovered miRNAs that form a cluster with with 2 known miRNAs. Reads mapping to novel miRNA precursors areshown in red and reads from small RNA from ovaries, larvae testes and adult testes that map on while reads from known miRNA precursors areshown in blue. **(C)** Heatmap showing normalised mIRdeep read values (log10) in each sample.

The total percentage of reads deriving from annotated miRNAs in the samples ranged to 2 to 6% (Figure 1A-G). This small percentage encouraged us to search for novel miRNA molecules that may have escaped previous experimental analysis in *A. gambiae* or its relative *A. stephensi* [20,23]. In addition, increases in the power and availability of deep sequencing in the last few years and the development of new technologies for miRNA discovery [31,32] have helped to improve the discovery of new miRNA molecules. We could previously find several novel miRNAs in mice, using these technologies, simply reanalyzing publicly available datasets of small RNA sequences [33]. To annotate novel miRNAs with high confidence we used strict criteria previously used to discover authentic miRNAs [31,34,35]: (i) The mature miRNA molecule had to pair with 2-nt overhang, with the miRNA star on the stem of the predicted precursor; (ii) miRNA also contained a uniform 5′ terminus compared with the 3′ terminus iii) miRNA was expressed in multiple samples. Using these approaches we were able to identify 63 novel loci coding for miRNAs (Additional files 5 and 6). Amongst the novel miRNAs identified in this study, some were grouped in clusters. We classified one cluster composed of 9 novel miRNAs (Figure 2A), one composed of 4, one of 3 and one of 2. We also identified 3 additional loci where novel miRNAs are part of clusters containing known miRNAs (one representative example in Figure 2B). A further class of miRNA, known as mirtrons, have been described that derive from short introns and whose biogenesis derives from splicing, bypassing the canonical Drosha processing [36]. Mapping the reads onto all the introns from *Anopheles* that are shorter than 100 nt and performing RNA structure analysis of these introns, we also discovered two mirtrons (microRNAs whose accurate processing requires the spliceosome) (aga-mir-10377b and aga-mir-10378, Additional file 6). Although we could not find any star sequence for aga-mir-10378, we considered it authentic because their mature molecules, corresponding to the 5’ arm of the precursors, are terminally uridylated. Terminal uridylation for mirtrons has been amply described [33,37].

In mammals and plants a significant fraction of miRNAs derive from repetitive sequences [38-42]. To evaluate the extent of miRNAs that originate from repetitive sequences in the mosquito we used RepeatMasker (http://www.repeatmasker.org) on all the miRNA precursors, including both known and novel genes identified in this study. Surprisingly, miRNAs in the mosquito rarely derive from repetitive sequences, with only one novel miRNA derived from a low complexity (CGT)n repeat.

Overall the novel miRNAs identified were prevalently derived from intergenic regions (35%) or the sense strand of introns (62%) (Additional file 5). Just 2 novel miRNAs seem to derive from exonic regions.

A recent study using a similar sequencing approach applied to *A. gambiae* mosquitoes pre- and post-bloodmeal revealed 58 novel miRNAs, showing the utility of a targeted deep sequencing approach [43]. 18 of these miRNAs were also revealed by our analysis, ultimately reducing the number of truly novel miRNAs in our study to 45 (Additional file 5).

### Several miRNA genes are upregulated in the gonads and specifically during gametogenesis

We next performed a differential expression analysis of both the novel and known miRNAs between the different analysed tissues. The read counts for each miRNA were compared between conditions using the Bioconductor DESeq package, which uses a negative binomial distribution model to test for differential expression in deep sequencing datasets (Additional file 7) [44,45]. Quantification of relative expression by qPCR was used to confirm tissue-specific expression for a subset of these microRNAs that showed varying but significant differential expression according to DESeq (Additional file 8b). Hierarchical clustering according to expression profile identified 3 main classes according to their breadth and intensity of expression (Figure 2C). Unsurprisingly Class I, comprising miRNAs abundantly expressed across all samples, was predominantly made up of previously annotated miRNA, whereas classes II and III, comprising lowly-expressed miRNAs and tissue-restricted miRNAs, respectively, were enriched for novel miRNAs, reflecting the greater sensitivity afforded by dissecting gonadal tissues at various stages of development. Among the tissue restricted miRNAs we found 41 miRNAs that were differentially expressed (adjusted *P-* value < 0.01) between the testes and pre-vitellogenic ovaries, including two (aga-mir-2944a-2 and aga-mir-286b) that were also upregulated both in the testes and during oogenesis, suggesting a general role in gametogenesis (Additional files 7 and 8a). Validating our approach, microRNAs with known roles in oogenesis such as miR-989 were heavily ovary-enriched in our analysis [20,46]. Larval stages of the mosquito show virtually no sexual dimorphism in external features and consistent with this we failed to find microRNAs that were significantly differentially expressed between the two sexes. However mIR-989 and aga-mir-10361b, that were ovary and testes-enriched in the adult respectively, were similarly enriched in the immature larval gonad of each sex (Additional file 7: Table S4), indicating that each microRNA must play an early role in the formation of the gonad. Interestingly, testes-biased miRNAs showed a non-random chromosomal distribution – of the 31 miRNAs that showed strong testes-bias 20 (64.5%) mapped exclusively to the X chromosome, compared to only 23% for non-testes biased miRNAs (Additional file 7: Table S4; Fisher’s exact test, p < 2E-05). Protein coding genes on the X chromosome are usually silenced due to meiotic sex chromosome inactivation (MSCI) during spermatogenesis [47], however our findings are consistent with recent evidence that X-linked microRNAs can escape this inactivation [48]. Moreover the high number of testes-biased novel microRNAs we identified on the X chromosome suggests that for microRNA genes at least this chromosome represents a favourable environment for male-biased microRNAs to evolve, as others have suggested [49].

### Endo-siRNAs are preferentially expressed in adult testes of *Anopheles gambiae*

**Figure 3:**
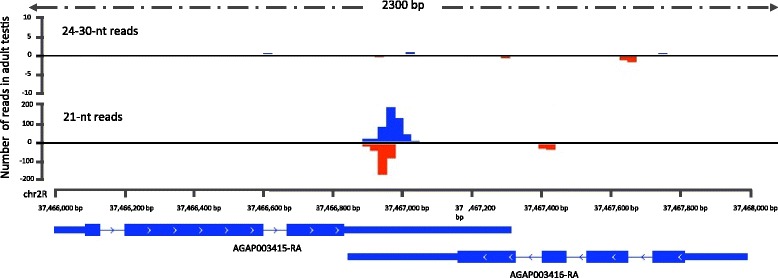
**cis-NAT-siRNAs derive from the overlap of convergently transcribed mRNAs.** Representation of a locus containing cis-NAT-siRNAs that derive from the overlap between AGAP003415-RA and AGAP003416-RA convergently transcribed transcripts.

Studies performed on the model organism *Drosophila melanogaster* indicated that flies express in abundance endo-siRNAs exactly 21-nt long [50-53]. endo-siRNAs usually derive from TEs, structured loci (that can produce folding dsRNA transcripts directly) and from the overlapping regions of convergently transcribed RNAs (cis-NAT-siRNAs). In contrast, miRNAs have a more heterogeneous length ranging from 20 to 24-nt. The existence of endo-siRNAs in the mosquito has not previously been confirmed. To look for this class of RNA, we first removed from our sequencing libraries all the reads that map onto known and novel miRNA precursors and investigated the nucleotide size distribution before and after miRNA removal (Additional file 9). As expected, the small RNA fraction that peaks from 20 to 23-nt was reduced (Additional file 9A-G). Interestingly, removing the miRNA fraction accentuated a significant 21-nt peak only in the adult testes, suggesting that only in this tissue did endo-siRNAs make up a significant proportion of this size class (Additional file 9F). Since endo-siRNAs can either derive from TEs or overlapping regions of two convergently transcribed transcripts, we systematically analyzed reads derived from regions of the genome predicted to contain overlapping transcripts on each strand, in all the various samples. This analysis did reveal distinct 21-nt peaks in all adult gonads indicating the expression of cis-NAT-siRNAs in these organs, that was again more evident in the adult testes (Figure 3 and Additional file 10). These analyses also indicated an additional distinct peak ranging from 24 to 30 nt in adult gonads of both sexes (Additional file 10E-F).

**Figure 4:**
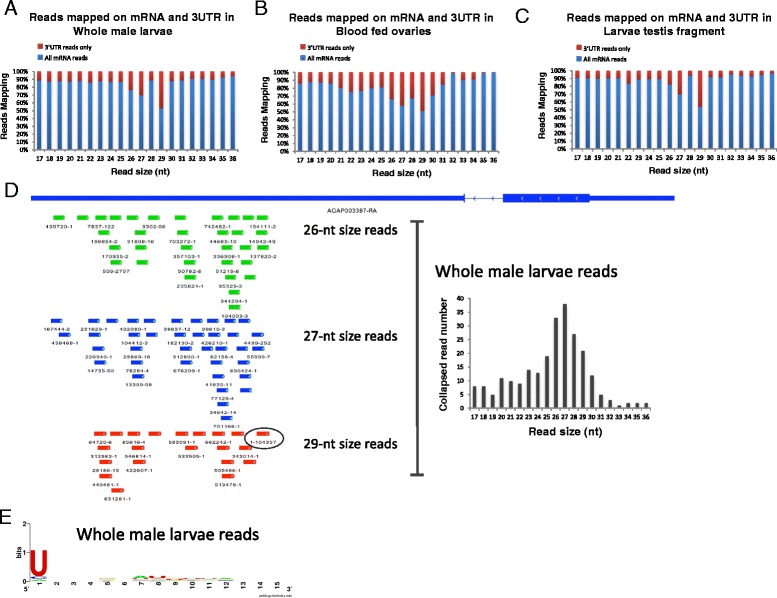
**mRNA-derived sense piRNAs predominantly arise from the 3’UTR regions.** Percentage of reads mapping on both the entire mRNA (blue) and the 3’UTR only (red) of *Anopheles* transcripts of the analyzed samples indicate that piRNAs predominantly derive from the 3’UTR part. The figure shows reads mapping and size distribution of **(A)** whole male larvae, **(B)** blood fed ovaries and **(C)** larvae testis fragment as representative examples. **(D)** (left) Mapping of 26, 27 and 29-nt sized piRNAs from whole male larvae on the 3’UTR of AGAP003387. The nomenclature under each small RNA refers to its unique but arbitrary ID followed with a post-hyphen suffix indicating the normalized number of reads corresponding to that sequence in the sample (e.g. 55599–7 was a small RNA given ID 55599 that was cloned 7 times) The most abundant read is 29-nt long, was represented in 104,357 reads and is highlighted with a circle. (right) Size distribution of all the collapsed reads from whole male larvae derived from this 3’UTR showing that small RNAs of between 25 and 29 nt predominate. **(E)** Nucleotide frequency for each position of the collapsed reads derived from the 3’UTR of AGAP003387 indicate a prevalence of U at the first position.

In *Drosophila melanogaster* there is a small but significant portion of piRNAs that derive from endogenous protein coding genes rather than transposons or repeat loci and these piRNAs preferentially derive from the 3’UTR of the transcript [54]. Supporting the hypothesis that the fraction of reads corresponding to 24-30 nt similarly represent piRNAs in the mosquito, this fraction shows a strong bias in mapping to the 3’UTR of mRNAs while in contrast the 17-24 nt fraction is more evenly distributed (Figure 4A-C and Additional file 11). We then evaluated the size distribution of the reads mapping on the entire mRNAs or the 3’UTR only. We identified a peak of reads that range from 24 to 27 in all the analyzed samples (Figure 4A-C). Surprisingly the curve of read size from all the samples presented a bimodal distribution not previously seen for the piRNA class, with a peak at 27 nt and a repeatedly larger peak at 29-nt. The 29-nt reads derive almost exclusively from 3’UTRs (Figure 4A-C and Additional file 12A-D). To investigate whether this abundant 29 nt RNA represented a novel class of piRNA we mapped all the 29-nt long reads from the various samples to examine from which mRNAs they derived.

### AGAP003387 locus produced high abundance of one 29-nt piRNA from its 3’UTR

The 29-nt fraction derived from mRNAs actually derived almost exclusively from the sense strand of the 3’UTR of the gene AGAP003387 and was dominated by a unique sequence that is the most abundant read in all the samples (about 1-4% of all the reads) (Figure 4D). All the sample tissues presented this identical pattern. The nuclear size distribution (26-29 nt) of each unique small RNA sequence (Figure 4D) that derives from this locus and the presence of a bias of uridine (U) as the first nucleotide in these sequences, suggests that they have some piRNA-like characteristics (Figure 4E).

### piRNAs map to discrete genomic loci and are produced abundantly from transposable elements

**Figure 5:**
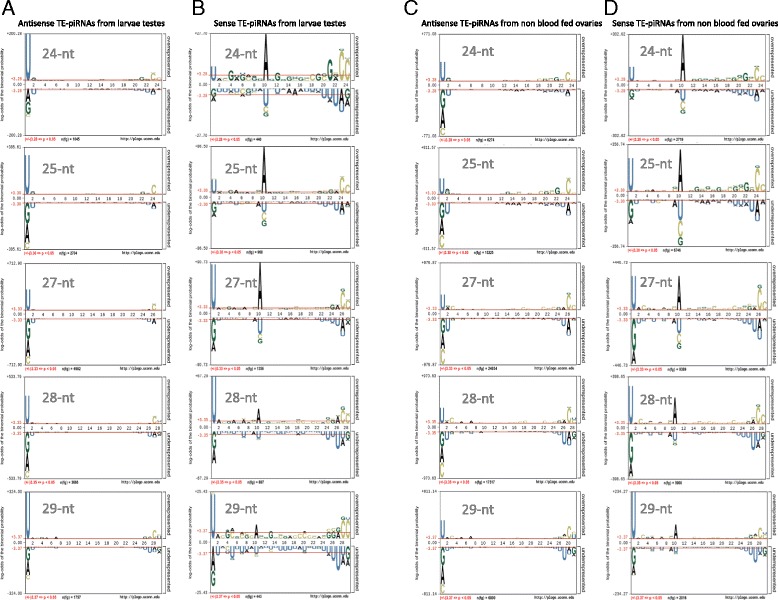
**piRNA sequence bias.** Small RNA reads in the 24-29 nt putative piRNA class were analysed for sequence bias. Shown are the reads mapping from larval testes and non-bloodfed ovaries as representative examples **(A)** Nucleotide bias of each position of antisense 24, 25, 27, 28 and 29-nt TE-piRNAs from larvae testes is computed and graphed using pLogo. **(B)** Nucleotide bias of each position of sense 24, 25, 27, 28 and 29-nt TE-piRNAs from larvae testes is computed and graphed using pLogo. **(C)** Nucleotide bias of each position of antisense 24, 25, 27, 28 and 29-nt TE-piRNAs from non-blood fed ovaries is computed and graphed using pLogo. **(D)** Nucleotide bias of each position of sense 24, 25, 27, 28 and 29-nt TE-piRNAs from non-blood fed ovaries is computed and graphed using pLogo.

We aimed to get an insight into the relative contributions in *Anopheles gambiae* of endo-siRNAs and piRNAs that derive from TEs, and hence may be involved in germline regulation/suppression of transposition. We mapped the larger classes of small RNAs against the transposable element complement of *A. gambiae* (Repbase, v19. [55]) and examined their sequence composition using the algorithm pLogo to display statistically significant over- or under- representations [56]. Small RNAs of between 26-29 nt that derived from the antisense strand of TEs generally (>70% of all sequences) showed a strong enrichment for U at their 5’ end (U1-bias) (Figure 5A and C), while those derived from the sense strand are enriched for A at position 10 (A-10 bias) (Figure 5B-D). These features strongly suggest that the majority of these molecules we identified in the mosquito are bona fide piRNAs since they are consistent with a ping-pong mechanism of piRNA generation described in *Drosophila* whereby antisense piRNAs (loaded onto *PIWI* or *Aubergine*), enriched for U at their 5’ end, direct the cleavage of a sense transcript to produce secondary piRNAs (loaded onto *AGO3*) that are consequently enriched for A at position 10.

In order to define piRNA expression clusters in the *Anopheles* genome we selected only reads containing a U1 or A10 that should represent a piRNA-enriched fraction from all samples. We than mapped these reads on the genome with perfect match at a unique position. This strategy has been successfully used to find the genomic origin of piRNAs in other organisms [2]. We considered loci as producing piRNAs if the average length of reads was 26-27-nt, abundance was more than 50 mean count of reads and loci larger than 100-bp. Using these criteria we revealed that *Anopheles* piRNAs map to more than 1500 discrete genomic loci (Additional file 13).

**Figure 6:**
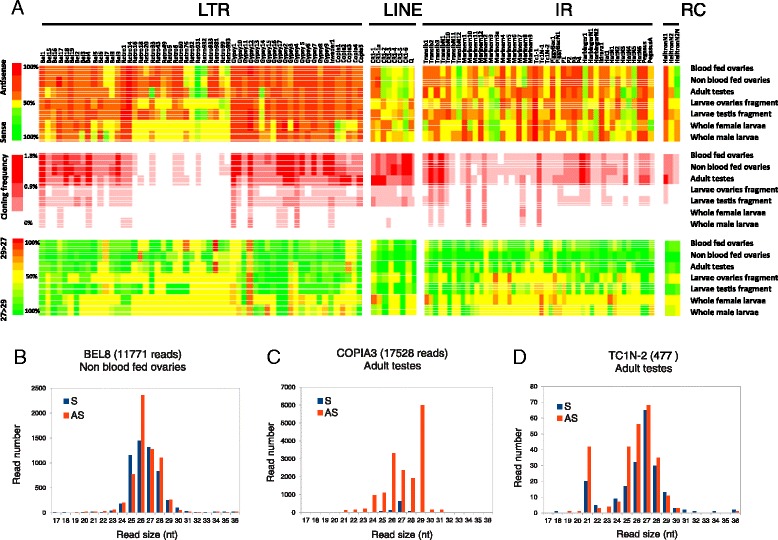
**Some TE loci produce a prevalence of 29-nt sized piRNAs. (A)** The upper panel is a heat map that indicates the strand bias of cloned piRNAs derived from the TEs. The bottom panel is a heat map that indicates the relative frequency of 29 vs 27-nt TE piRNAs as a ratio. Transposons are grouped into long terminal repeats (LTR), long interspersed nuclear elements (LINE), inverted repeats (IR) and rolling circles (RC) elements. **(B)** Read size distribution of sense and antisense piRNAs on the BEL8 family of LTR elements. **(C)** Read size distribution of sense and antisense piRNAs on the COPIA3 TE family of LTR elements. **(D)** Read size distribution of sense and antisense piRNAs on the TC1N-2 TE family of IR elements.

Putative *Anopheles* piRNAs deriving from TEs were recovered from both strands (Figure 6A and Additional file 14) but there was a clear bias towards antisense-derived piRNAs suggesting that the majority of piRNAs are produced through primary biogenesis pathway while the minority derive from the ping-pong pathway. In *Drosophila* piRNA biogenesis differs between the germline-derived ovary and the somatic part of the ovary (and the soma in general) - the somatic part of the ovary only expresses PIWI of the Piwi-class of proteins and primary piRNAs whose biogenesis is not controlled by a ping-pong mechanism [57]. Consistent with a similar soma-germline dichotomy operating in the mosquito piRNAs were produced almost exclusively from the antisense strand in the whole larvae (Additional file 14F and G) in which the vast majority of cells are of somatic origin, whereas the ratio of sense:antisense in TE-derived reads is much higher the larval fragment enriched for either the developing germline ovary or testis tissue (compare Additional file 14A-F and B-G) indicating that, as in other organisms, *Anopheles gambiae* only produces primary piRNAs in somatic tissues. On the other hand, in rare cases specific TE classes, such as *CR1-2*, *CR1-6* or *TransibN-12* predominantly produces piRNAs from the sense strand (Figure 6A top panel). The endo-siRNA production from TEs was quite low, judging by the relative amounts of 21-nt sized RNAs in each sample (Additional file 14). TEs predominantly produced piRNAs, although few examples, such as *TC1N-2* also produced endo-siRNAs (Figure 6D). We could not see any cases of TEs exclusively producing endo-siRNA, in contrast to the situation in *Drosophila* [53], suggesting that this pathway has only a minor role to play in TE control in the mosquito (Additional file 14B).

### A subgroup of TEs produce predominantly 29-nt sized small RNAs

**Figure 7:**
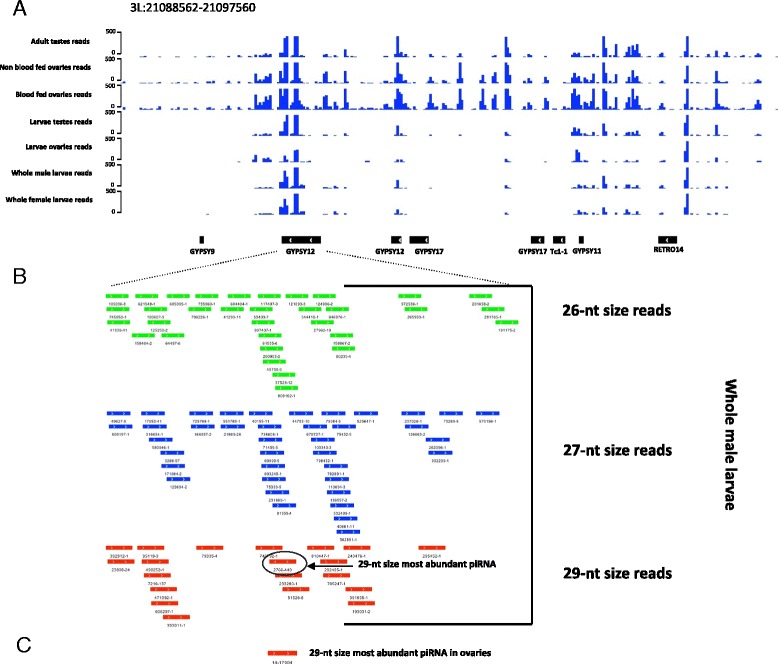
**GYPSY12 TE produces an abundant 29-nt piRNA. (A)** Density of the antisense reads belonging to a cluster of piRNAs located at chromosomal 3 L:21088562–21097560 locus. **(B)** Mapping of 26-, 27- and 29-nt sized piRNAs to the GYPSY12-LTR element. Shown here are the reads mapping from whole male larvae as a representative example. The most abundant piRNA (2766 reads) is 29-nt long and highlighted with a circle. **(C)** The same 29-nt piRNA was consistently the most abundant in the various tissues examined. An example is shown from the ovaries (17944 normalised reads).

Interestingly, the size distribution of putative TE piRNAs from the whole larvae did not show a normal distribution but showed a bimodal distribution with a wide peak that ranges from 24 to 28 and a second peak at 29-nt that was seen among reads that mapped uniquely to the genome(Additional file 14F and G) and accentuated among reads that mapped to TEs at multiple genomic locations (Additional file 15), indicating that abundant 29-nt small RNAs are not exclusively expressed from the 3’UTR of endogenous protein coding genes (Figure 4A-C and Additional file 12A-D). Moreover a systematic analysis comparing the prominence and orientation of these two size classes of small RNAs showed that though most TEs predominantly produced RNAs of 27-nt (Figure 6A lower panel), there are few cases of TEs such as *GYPSY12* and *COPIA3* classes that predominantly produced 29-nt RNAs (Figure 6A lower panel and 6C). Interestingly these loci highly producing 29-nt RNAs only formed these from the antisense strand in all tissues (Figure 6A lower panel). *GYPSY12* is located in a genomic location that produced a highly conserved cluster of primary piRNAs (Figure 7A). This cluster covers a genomic region containing 7 LTR transposons. The highest peak of small RNA production is located at the middle of the GYPSY12 sequence and corresponds to a unique 29-nt RNA that is highly conserved among the tissues analyzed and in each of the various tissues is expressed at least two orders of magnitude more than other 29-nt RNAs or smaller putative piRNAs derived from this same locus (Figure 7B and C). This huge over-representation of a single 29-nt RNA at a piRNA locus is similar to the situation observed earlier in the 3’UTR of a protein coding gene (Figure 4). At both loci there were fewer species of 29-nt piRNAs than of the 27-nt variety, despite the very high expression of some individuals of the 29 nt class (Figures 4 and 7). Similarly to antisense piRNAs this class of longer 29 nt small RNA molecule has prominent U1 nucleotide bias but in contrast to classical piRNAs whose population is quite complex with most RNAs being cloned only very rarely in both *D. melanogaster* [2] and *A. gambiae* (this study) this group of antisense 29-nt small RNA are represented several times by the same molecule.

### Anopheles piRNAs show novel sequence bias in addition to the classical characteristics of A10 and U1 bias

Further analysis of the sequence characteristics of putative *Anopheles* piRNAs that derive from both the sense and the antisense strand of TEs revealed other nucleotide biases and further complexity at positions of the piRNA in addition to the described A10 bias for sense piRNAs and U1 bias for antisense piRNAs. In particular piRNAs derived from the sense strand, in addition to showing a predominance of A10, are also highly enriched for U1. To exclude the possibility that the sense piRNA population was a mixture of primary (U1) and secondary (A10) piRNAs, we analyzed just the reads derived from the TEs that contain A10 and we observed that they also preferentially contained a U at position 1, confirming that these sequence biases frequently manifest in the same piRNA molecule (data not shown). Interestingly, the ratio A10 vs U1 decreased with increasing piRNA size in the various tissues, suggesting qualitative differences between the species of piRNA that may be related to their mode of biogenesis (Figure 5B and D). To our knowledge this is the first report of such sequence bias and was revealed by the additional sensitivity in detection afforded by separating piRNAs into different size classes. To confirm if this phenomenon held true across different organisms we re-analysed the dataset of *Drosophila* piRNAs that were specifically bound by the Piwi proteins PIWI, AUB and AGO3 [2], split these by size class and used pLogo to check for sequence bias (Additional file 16). As expected all populations displayed the known signatures yet strikingly the population of piRNAs derived from AGO3, that are heavily enriched for sense strand piRNAs, additionally contained a sequence bias of U (40%) at the 5’ terminal position that was statistically significant and similar to what we observed in the mosquito (Additional file 16C). This U1 enrichment also for sense piRNAs suggests that AGO3 preferentially loads U1 enriched small RNAs as for other argonaute family members in worm, fly and fly and human [58-61]. We also noticed a significant enrichment of cytosines in the last 2 nt of the piRNAs of each size class that was present in each of the two species regardless of piRNA size (Figure 5 and Additional file 16).

### *Anopheles gambiae* produces an abundant class of 32-nt half tRNA that is down-regulated in pre-vitellogenic ovaries

A distinct population of longer small RNAs, 32 nucleotides in length, were among the most abundant class of RNA revealed in all samples that mapped exclusively to tRNA genes, and specifically the 5’ end. These 5’ half tRNAs have been previously described in other organisms and have recently been shown to have wide ranging yet fully elucidated roles in a wide range of host processes ranging from translational inhibition, stress response and signalling [29,62,63]. Though we cannot speculate a role for these half tRNAs in the mosquito it is interesting to note that they were abundantly expressed in all samples but showed a 30-fold upregulation in ovaries specifically during vitellogenesis (Additional file 7).

## Conclusions

In this study we have greatly increased the known complement of small regulatory RNAs in the mosquito *A. gambiae*, identifying a large number of novel microRNAs, revealing the extent of endogenous siRNA production and describing for the first time the presence of piRNAs with likely roles in transposon control in the germline and possibly also roles in the control of a limited number of endogenous genes.

By focusing on the gonads at various stages of development we were able to sample the microRNAs expressed during the development and maintenance of these tissues at a much higher sensitivity than would have been afforded by examining the whole animal. This is the case even more so for the testes, given the relatively small size of this tissue and may in part explain the higher proportion of novel microRNAs recovered from the testis-enriched pool. The microRNA profile of both the ovary and testes through development and at different stages of gametogenesis described here should provide a valuable resource for better understanding the regulation of gene expression during this crucial process. In the future further functional analysis through mutagenesis or mis-expression of those microRNAs specifically enriched during the process of male or female gametogenesis should reveal candidate genes whose disruption could block mosquito reproduction.

The piRNA pathway has been shown in several organisms to be essential in the germline for repressing the expression of transposable elements, whose unregulated transposition would cause deleterious effects and loss of reproductive fitness. This report is the first description of the piRNA landscape in the reproductive tissues of the mosquito. We detected abundant piRNAs expressed from numerous clusters around the genome with sequence homology to all transposable element families identified in the *A. gambiae* genome. Different TE species produce distinct piRNA pools that were indicative of primary piRNA silencing only or a combination of primary and ping-pong produced piRNAs. The three Piwi proteins PIWI, AGO3 and Aub have different roles in the two mechanisms of piRNA generation and mutations in any of them lead to a de-repression of TE activity in the model insect *Drosophila*, indicating that they have non-redundant roles [64,65]. Interestingly our results show that different transposable elements can produce piRNA pools with markedly different characteristics, consistent with a route of biogenesis by specific members of the Piwi family. In *Drosophila*, members of the gypsy family of retrotransposons are expressed as virion-like particles in the somatic follicle cells of the gonad that surround the oocyte. In the follicle cells only PIWI is expressed of the 3 Piwi proteins, leading to silencing by primary piRNAs generated from transcripts of *flamenco*, a locus rich in degenerate copies of gypsy [2]. Similarly we saw that mosquito piRNAs mapping to the various gypsy elements were almost exclusively anti-sense, consistent with a primary biogenesis by PIWI, though as yet we do not know if there is a functional equivalent of *flamenco* as a piRNA master locus in the mosquito, or if in the somatic follicle only PIWI is expressed. Orthologues of each of the 3 Piwi genes are present in the *A. gambiae* genome and expression analysis in the closely related mosquito *A. stephensi* reveals that each is ovary-enriched and upregulated during the process of oogenesis [66] As the genetic tools available in the mosquito improve it should be possible to dissect the role of various AGO and Piwi proteins in small RNA biogenesis and activity, and to confirm Piwi-association of some of the putative piRNA classes that we have revealed.

In terms of the characteristics of the piRNA populations we discovered, many features are conserved with other organisms such as size (24-30 nt), the sequence bias at positions 1 and 10 that are signatures of a ping pong mechanism of biogenesis. On the other hand, we reveal some novelties in the generation of piRNAs such as a population of piRNAs recognizing the 3’ UTR of endogenous genes, previously undocumented sequence biases in piRNAs corresponding to the sense strand of TEs and an abundant 29 nt class of piRNA.

Despite the wide interest in piRNAs in recent years, many questions remain unresolved. For example, although the mechanism by which transposable elements are repressed in most cases is likely a combination of mRNA degradation through the Slicer activity of piRNA-loaded Piwi members and piRNA-guided heterochromatic silencing of expression, there are also reports of piRNA-directed translational repression [67,68]. Moreover, how the initial trigger RNA from the piRNA cluster is produced, recognized and loaded onto a Piwi protein is still not clearly resolved. The novel characteristics of the piRNA pool in the mosquito adds clues to their origin may help in resolving the mechanism of piRNA biogenesis in general. Furthermore, there are several instances of piRNAs and other small RNAs derived from repetitive elements on the sex chromosomes that have been co-opted to mediate interactions between the chromosomes and can play a role in sex determination [69,70]. It remains to be seen whether such a role exists in the mosquito, where much of the Y chromosome is made of repetitive sequences including several transposon relics [71].

Certainly the extensive characterization of the piRNA pool provided here and the demonstration of extensive TE-derived piRNA pools that are abundantly expressed in the germline provides an answer as to how the mosquito manages to control the proliferation of TEs in its genome. Attempts to introduce into a mosquito population anti-pathogen or otherwise beneficial constructs through the use of TEs designed to spread at super-Mendelian frequency, as has been proposed [72], will have to make contingency for this genome defence mechanism.

## Methods

### Ethics statement

All animal work was conducted according to UK Home Office Regulations and approved under Home Office License PPL 70/6453.

### RNA extraction

Gonads were dissected in PBS from 3–4 day old adult male and female mosquitoes (G3 strain) that had been reared under standard insectary conditions (adults reared at 27°C, 80% humidity, fed ad libitum on 5% glucose). To obtain vitellogenic ovaries females were dissected 24 hours after a bloodmeal from an anaesthetised mouse. In order to sex larval stages that are otherwise morphologically indistinguishable we used a mosquito line containing a sex-linked insertion of the visible marker gene RFP (Nolan, unpublished) that could allow us to unambiguously separate male and female larvae in the progeny of a cross between RFP-positive males and wild type females. Each sample contained tissue from a minimum of 10 individuals. The small size and delicate nature of the larval gonads prevented their removal intact from the larval body. In order to enrich for the gonadal tissue we dissected segments 5 to 7 from L4 larvae using fine 30 gauge needles. We separately confirmed under microscopy that this section consistently contained both male and female developing gonads. RNA was then isolated from this tissues using TRIzol Reagent (Life Technologies), following the manufactured instructions. RNA quality and integrity was assessed using a Bioanalyzer instrument (Agilent Technologies Genomics).

### Small RNA libraries preparation and sequencing

One μg of purified RNA from two biological replicate per condition was used to prepare small RNA libraries according to the TruSeq Small RNA Sample preparation kit (Illumina) instructions. Fifty base pair (bp), single end sequencing was performed using the HiSeq 2000 instrument (Illumina).

### Reads preprocessing and mapping

We clipped out the 3’ adaptors form the reads obtained at the end of the sequencing run using the FASX-toolkit from Hannon lab (http://hannonlab.cshl.edu/fastx_toolkit/) before further analyses. The processed reads from each sample were then mapped to the AgamP3 genome assembly using Bowtie version 0.12.7 allowing for 0 mismatches. Annotation of small RNAs was usually done mapping the reads on data downloaded from VectorBase (https://www.vectorbase.org/downloads) with the exception of transposable elements that were instead obtained from the University of California at Santa Cruz (USCS) Genome Browser (http://genome.ucsc.edu).

### miRNA discovery and miRNA expression profile

To discover novel miRNAs we used both miRCat from the UEA small RNA Workbench (https://srna-workbench.cmp.uea.ac.uk/) and miRDeep2 algorithms [31,32] maintaining default setting and filtering reads by size ≥17. Among the novel candidates discovered using this approaches, high-confidence miRNAs containing both mature and star sequences complementary with 2-nt 3′ overhang detected in multiple samples were considered. To discover mirtrons we mapped all the reads coming from the various samples on introns smaller than 100-nt and manually inspected them. Newly discovered miRNAs were then quantified among the various tissue samples using the quantifier module from the miRDeep2 package. Differential expression profile of both novel and known miRNAs across the samples was performed using DESeq from the Bioconductor project (http://www.bioconductor.org).

Normalised expression values from the mIRDeep analysis were used to create a heatmap with the software GENE-E (www.broadinstitute.org) using log10 values. Only mIRs with a read count >20 in at least one condition were included in the heatmap and were hierarchically clustered by Euclidean distance.

### Evaluation of miRNAs located on genomic repeats

To evaluate whether annotated miRNAs are located on genomic repeats and to discover the nature of these repeats, novel and downloaded miRNA precursors from the miRBase release 20 were analyzed using the RepeatMasker script, version 3.2.8 (http://www.repeatmasker.org/).

### qPCR validation of miRNA expression

Reverse transcription was primed using a stem-loop primer with 8 nucleotides of complementarity to the target mIR of interest and cDNA synthesized using Superscript III reverse transcriptase, followed by PCR amplification using a mIR-specific primer and a primer specific to the stem loop [73]. Reactions were performed in a StepOne Plus RT-PCR machine (Invitrogen) and PCR product was quantified by measuring SYBR green incorporation. The broadly expressed and non-sex-specific microRNA *bantam* was used as an internal control and the comparative Ct method was used to compare miRNA amounts between samples. 2 biological replicates were performed for each sample (TE, BF or OV) and a mimimum of 10 individuals were included per sample.

### piRNA cluster analysis

To discover piRNA clusters, few clusters of small RNA reads that map to TEs were first selected and verified that the reads that map on the antisense strand predominantly contained an uridine (U) as the first nucleotide whereas the reads that map on the sense strand contained an adenosine (A) at the 10^th^ position. This indicated that also piRNAs that derive from TE in mosquito are produced through a secondary ping-pong pathway as in *Drosophila* [2]. Next, only reads containing a U as first nucleotide or an adenosine A at the 10^th^ position were bioinformatically selected from each sample in order to enrich the reads of piRNAs. Clusters of piRNAs were than identified using the SiLoCo implementation from the UEA small RNA Workbench [32], considering chromosomal regions no larger than 100 base pairs (bp), loci containing average read size that range from 26 to 27 and containing more than 50 reads.

### piRNA motif analysis

To analyse TE-piRNA motif either WebLogo [74] on trimmed reads or pLogo [56] on size-selected reads were used.

### Analysis of *Drosophila* publicly available samples

Analysis of *Drosophila* derived sequence reads was performed on fastq files downloaded from NCBI Gene Expression Omnibus (GEO), (GSE6734, GSE15378, GSE11086) using the same tools and procedures described above for *Anopheles*.

### Availability of supporting data

The datasets supporting the results of this article have been deposited at the European Nucleotide Archive with submission number PRJEB7896.

## Additional files

Additional file 1:
**Genomic mapped reads of small RNAs from each tissue.**
Additional file 2:
**Small RNA reads distribution and predicted RNA secondary structure of the known miRNAs in the analyzed tissues.** OV adult non-bloodfed ovary, TE adult testis, BF adult bloodfed ovary, MF larval testis-enriched fragment, FF larval ovary-enriched fragment, MW whole male larvae, FW whole female larvae. Number suffix indicates replicate number e.g. OV2: adult non-bloodfed ovary, replicate 2. Additional file 3:
**On the top, sequence and predicted RNA secondary structure of annotated aga-mir-133 precursor.** On the bottom sequence and predicted RNA secondary structure of aga-mir-133 precursor identified in this study. Additional file 4:
**Raw read count of known** ***Anopheles*** **miRNAs from each sample.** OV adult non-bloodfed ovary, TE adult testis, BF adult bloodfed ovary, MF larval testis-enriched fragment, FF larval ovary-enriched fragment, MW whole male larvae, FW whole female larvae. Number suffix indicates replicate number e.g. OV2: adult non-bloodfed ovary, replicate 2. Additional file 5:
**Summary of novel identified miRNAs detailing genomic location, mIRbase ID deposition numbers, number of times mature or star molecule was cloned.** miRNAs also cloned in a recent study by Biryukova et al. are also indicated. Additional file 6:
**Small RNA reads distribution and predicted RNA secondary structure of the novel, identified miRNAs in the analysed tissues.**
Additional file 7:
**DESeq output from differential expression analysis between samples.** Samples compared are described in row 1 (eg. “OV_vs_TE”) Mean read intensity (from two biological replicates) was compared between samples using DESeq and adjusted p-value (padj). micoRNAs with significant differential expression at padj < 0.01 are highlighted in yellow. OV adult non-bloodfed ovary, TE adult testis, BF adult bloodfed ovary, MF larval testis-enriched fragment, FF larval ovary-enriched fragment, MW whole male larvae, FW whole female larvae. Additional file 8:
**a) comparison of fold changes in mIR expression between male and female gonads (Testes (TE), non-bloodfed ovaries (OV) and bloodfed ovaries (BF)) as calculated from RNA-seq data (DESeq) and qRT-PCR.** Two classes of gonad-enriched mIRs were identified, those enriched in the testes only and those enriched in the testes and bloodfed vitellogenic ovaries, but not pre-vitellogenic ovaries, that likely have a role in gametogenesis. (b) Fold changes calculated by the two methods generally showed good correlation. (c) Fold change (log2) increase in testis expression vs carcass expression is shown for two testis-specific miRNAs. Additional file 9:
**Read size distribution of small RNAs in total (green) and with the miRNA population removed (red) in each of the various samples (A-G) with and without (red) miRNAs.**
Additional file 10:
**cis-NAT-siRNAs size distribution on the various samples (A-G).**
Additional file 11:
**(A) Distribution of the reads from 21 to 24-nt long on the 5UTR, CDS and 3UTR of mRNAs normalized for their genomic coverage.** (B) Distribution of the reads from 24 to 30-nt long on the 5UTR, CDS and 3UTR of mRNAs normalized for their genomic coverage. Additional file 12:
**Size distribution small RNAs mapping to coding transcripts and the relative distribution in the 3’ UTR (red) compared to the entire mRNA (blue) in the following representative samples: (A) whole female larvae, (B) non blood-fed ovaries, (C) larvae ovary fragment and (D) adult testes.**
Additional file 13:
**Chromosomal distribution of piRNA clusters from the various** ***Anopheles*** **tissues analyzed in this study.** Each tab represents the clusters from a biological sample replicate. Some clusters map to Y chromosome sequence (“Y_unplaced”) even in female samples presumably because of incomplete genome annotation of repetitive regions across all the chromosomes. Strand bias is given as figure between 1 (positive strand) and 0 (negative strand). ‘Unique sRNAs’ refers to the number of sequence reads from the locus that only have a single match to the genome. ‘Mean count’ refers to the abundance of reads that derived from each locus, normalized per million of genome-mapping reads. Additional file 14:
**Nucleotide size distribution of reads from** ***Anopheles*** **antisense (grey) and sense (red) TEs using reads from the various samples (A-G).**
Additional file 15:
**Nucleotide size distribution of reads that map on more than 10 locations from** ***Anopheles*** **antisense (grey) and sense (red) TEs, from whole male (left) and female (right) larvae.**
Additional file 16:
**Nucleotide bias of each position of 24 and 25-nt long piRNAs obtained from (A) PIWI, (B) AUB and (C) AGO3** ***Drosophila*** **proteins.**
