# Supplementary material for: The germline of the malaria mosquito produces abundant miRNAs, endo-siRNAs, piRNAs and 29-nt small RNAs
Source: BMC Genomics. 2015 Feb 19;16(1):100. doi: 10.1186/s12864-015-1257-2 (PMC4345017; doi:10.1186/s12864-015-1257-2)
Supplement: Additional file 1: — Genomic mapped reads of small RNAs from each tissue. [file 12864_2015_1257_MOESM1_ESM.pdf]

Tissue	Genomic mapped reads
Adult testes #1	5,053,835
Adult testes #2	6,040,976
Adult ovaries #1	10,025,380
Adult ovaries #2	5,956,747
Adult ovaries from blood fed females #1	5,420,509
Adult ovaries from blood fed females #2	6,229,521
Larvae fragment testes #1	4,084,245
Larvae fragment testes #2	35,808,114
Larvae fragment ovaries #1	8,277,528
Larvae fragment ovaries #2	4,191,267
Male whole larvae #1	7,731,867
Male whole larvae #2	5,320,380
Female whole larvae #1	5,918,991
Female whole larvae #2	10,168,373
