# Supplementary material for: The germline of the malaria mosquito produces abundant miRNAs, endo-siRNAs, piRNAs and 29-nt small RNAs
Source: BMC Genomics. 2015 Feb 19;16(1):100. doi: 10.1186/s12864-015-1257-2 (PMC4345017; doi:10.1186/s12864-015-1257-2)
Supplement: Additional file 2: — Small RNA reads distribution and predicted RNA secondary structure of the known miRNAs in the analyzed tissues. OV adult non-bloodfed ovary, TE adult testis, BF adult bloodfed ovary, MF larval testis-enriched fragment, FF larval ovary-enriched fragment, MW whole male larvae, FW whole female larvae. Number suffix indicates replicate number e.g. OV2: adult non-bloodfed ovary, replicate 2. [file 12864_2015_1257_MOESM2_ESM.pdf]

miRBase precursor : aga-bantam
Total read count : 160609
aga-bantam read count : 141649
aga-bantam* read count : 18955
remaining reads : 5

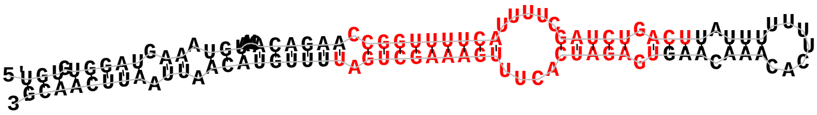

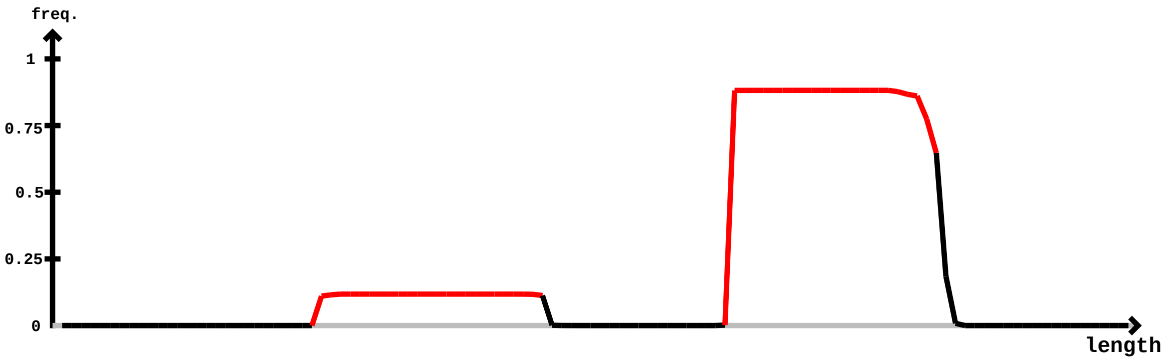


```
                              aga-bantam*
                                                                          aga-bantam
5'- ugugugggaugaaauguaaucacagaaccgguuuucauuuucgaucugacuuauuuuuuucacaaacaagugagaucacuuugaaagcugauuuuguacaauuaauucaacg  -3'   exp
    .((.(((((.((.(((....((((((.(((((((((.....(((((.((((.(((.......))).)))).)))))....))))))))).))))))))).)).))))))).   reads   mm   sample
    ..........................ccgguuuucauuuucgauc..................................................................   1       0    OV2
    ..........................ccgguuuucauuuucgaucuga...............................................................   15      0    OV2
    ..........................ccgguuuucauuuucgaucugac..............................................................   50      0    OV2
    ..........................ccgguuuucauuuucgaucugacC.............................................................   1       1    OV2
    ..........................ccgAuuuucauuuucgaucugacu.............................................................   1       1    OV2
    ..........................ccgguuuucauuuucgauUugacu.............................................................   1       1    OV2
    ..........................ccgguuuucGuuuucgaucugacu.............................................................   1       1    OV2
    ..........................ccgguuuucauuuucUaucugacu.............................................................   1       1    OV2
    ..........................Acgguuuucauuuucgaucugacu.............................................................   1       1    OV2
    ..........................ccggAuuucauuuucgaucugacu.............................................................   1       1    OV2
    ..........................ccgguuuucauuuucgaucugacA.............................................................   14      1    OV2
    ..........................ccgguuuucauuuucgaucugacu.............................................................   855     0    OV2
    ..........................ccgguuuuUauuuucgaucugacu.............................................................   1       1    OV2
    ..........................ccggGuuucauuuucgaucugacu.............................................................   1       1    OV2
    ..........................ccgguuCucauuuucgaucugacu.............................................................   1       1    OV2
    ..........................ccgguuuucauuuuAgaucugacu.............................................................   1       1    OV2
    ..........................ccgguuuucauCuucgaucugacu.............................................................   1       1    OV2
    ..........................ccgguuuucauuuucgaucugacG.............................................................   1       1    OV2
    ..........................ccgguuuucauuuucgaucuAacu.............................................................   1       1    OV2
    ..........................ccggCuuucauuuucgaucugacu.............................................................   1       1    OV2
    ..........................ccgguuuucauuuucgaCcugacu.............................................................   1       1    OV2
    ..........................ccgguuuucauuuucgaucugacuA............................................................   2       1    OV2
    ..........................ccgguuuucauuuucgaucugacuua...........................................................   3       0    OV2
    ..........................ccgguuuucauuuucgaucugacuuauuuuuuucacaaacaag..........................................   1       0    OV2
    ...........................cgguuuuUauuuucgaucugacu.............................................................   1       1    OV2
    ...........................cgguuuucauuuucgaucugacu.............................................................   23      0    OV2
    ............................gguuuucauuuucgaucugacu.............................................................   34      0    OV2
    ............................gguuuucauuuucgaucugacuu............................................................   4       0    OV2
    ............................gguuuucauuuucgaucugacuuU...........................................................   1       1    OV2
    ....................................................................gugagaucacuuugaaagcu.......................   1       0    OV2
    ....................................................................gugagaucacuuugaaagcug......................   3       0    OV2
    ....................................................................gugagaucacuuugaaagcuga.....................   3       0    OV2
    ....................................................................Cugagaucacuuugaaagcuga.....................   3       1    OV2
    ....................................................................gugagaucacuuugaaagcugau....................   3       0    OV2
```

```
                          aga-bantam*
                                                                     aga-bantam
ugugugggaugaaauguaaucacagaaccgguuuucauuuucgaucugacuuauuuuuuucacaaacaagugagaucacuuugaaagcugauuuuguacaauuaauucaacg

....................................................................Cugagaucacuuugaaagcugau....................	2	1	OV2
....................................................................Cugagaucacuuugaaagcugauu...................	2	1	OV2
....................................................................gugagaucacuuugaaagcugauuu..................	1	0	OV2
.....................................................................ugagaucacuuugaaA..........................	1	1	OV2
.....................................................................ugagaucacuuugaaag.........................	60	0	OV2
.....................................................................ugagaucacuuugaaagc........................	93	0	OV2
.....................................................................ugagaucacuuugaaagcu.......................	16	0	OV2
.....................................................................ugagaucacuuugaaagcug......................	755	0	OV2
.....................................................................ugagaucacuuuAaaagcug......................	1	1	OV2
.....................................................................uAagaucacuuugaaagcug......................	1	1	OV2
.....................................................................ugagaucacuuugGaagcug......................	1	1	OV2
.....................................................................ugagaucacuuugaaagcGg......................	2	1	OV2
.....................................................................ugagaucacuAugaaagcug......................	1	1	OV2
.....................................................................Cgagaucacuuugaaagcug......................	1	1	OV2
.....................................................................ugagaucacuuCgaaagcug......................	1	1	OV2
.....................................................................ugagaCcacuuugaaagcuga.....................	1	1	OV2
.....................................................................ugagaucacuuugaaagcGga.....................	1	1	OV2
.....................................................................uAagaucacuuugaaagcuga.....................	1	1	OV2
.....................................................................ugagaucacuuugaaaCcuga.....................	1	1	OV2
.....................................................................ugagaucacuuCgaaagcuga.....................	1	1	OV2
.....................................................................ugagaucacuuugaaagcCga.....................	2	1	OV2
.....................................................................ugagGucacuuugaaagcuga.....................	1	1	OV2
.....................................................................ugagaucacuuugUaagcuga.....................	1	1	OV2
.....................................................................ugagaucacuuugaaagcuga.....................	1355	0	OV2
.....................................................................ugagaucacuuugaaagGuga.....................	1	1	OV2
.....................................................................ugagaucacuuugaaagcugG.....................	8	1	OV2
.....................................................................ugagaucacuuugCaagcuga.....................	1	1	OV2
.....................................................................ugagaucacuuuAaaagcuga.....................	2	1	OV2
.....................................................................ugagaucacuuugaaagcugGu....................	1	1	OV2
.....................................................................ugagaucacuuugaaagcugaC....................	3	1	OV2
.....................................................................ugagaucacuuugaaagcugaG....................	1	1	OV2
.....................................................................ugUgaucacuuugaaagcugau....................	1	1	OV2
.....................................................................ugaCaucacuuugaaagcugau....................	1	1	OV2
.....................................................................ugGgaucacuuugaaagcugau....................	2	1	OV2
.....................................................................ugagaucacuuCgaaagcugau....................	2	1	OV2
.....................................................................ugagaucacuuugaaagcuAau....................	1	1	OV2
.....................................................................ugagaucacuuuAaaagcugau....................	3	1	OV2
.....................................................................ugagaucacuuugaaagcugau....................	3231	0	OV2
.....................................................................ugagaucaAuuugaaagcugau....................	1	1	OV2
.....................................................................ugagaucacuuugaaaCcugau....................	1	1	OV2
.....................................................................Ggagaucacuuugaaagcugau....................	2	1	OV2
.....................................................................Agagaucacuuugaaagcugau....................	1	1	OV2
.....................................................................uAagaucacuuugaaagcugau....................	3	1	OV2
.....................................................................ugaUaucacuuugaaagcugau....................	1	1	OV2
.....................................................................ugagaAcacuuugaaagcugau....................	1	1	OV2
.....................................................................ugagauUacuuugaaagcugau....................	3	1	OV2
.....................................................................ugagaucacuuuUaaagcugau....................	1	1	OV2
.....................................................................ugagaucacuCugaaagcugau....................	1	1	OV2
.....................................................................ugagaucacuuugaaagcugaA....................	8	1	OV2
.....................................................................Cgagaucacuuugaaagcugau....................	5	1	OV2
.....................................................................ugagaucacuuugaaagcCgau....................	1	1	OV2
.....................................................................ugagaucacuAugaaagcugauu...................	2	1	OV2
.....................................................................ugagaucacuuugGaagcugauu...................	1	1	OV2
.....................................................................Cgagaucacuuugaaagcugauu...................	1	1	OV2
.....................................................................ugagaucacuuugaaagUugauu...................	1	1	OV2
.....................................................................ugagaucacuuugaaagcuAauu...................	1	1	OV2
.....................................................................ugagaucacuuugaaagcugauu...................	1009	0	OV2
.....................................................................ugagaucacuuugaaagcugaAu...................	1	1	OV2
.....................................................................ugagaucacuuugaaagcugauG...................	2	1	OV2
.....................................................................ugagaucacuuugaaagcugauA...................	16	1	OV2
.....................................................................ugagaucacuuugaaagcCgauu...................	1	1	OV2
.....................................................................ugGgaucacuuugaaagcugauuu..................	1	1	OV2
.....................................................................ugagaucacuuugaaagcugauuC..................	2	1	OV2
.....................................................................ugagaucacuuugaaagcugauuu..................	20	0	OV2
.....................................................................ugagaucacuuugaaagcugauuA..................	4	1	OV2
......................................................................gagaucacuuugaaagcugau....................	1	0	OV2

..........................ccgguuuucauuuucgau...................................................................	1	0	TE1
..........................ccgguuuucauuuucgaucuga...............................................................	4	0	TE1
```

```
                          aga-bantam*
                                                                     aga-bantam
uguguggaugaaauguaaucacagaaccgguuuucauuuucgaucugacuuauuuuuuucacaaacaagugagaucacuuugaaagcugauuuuguacaauuaauucaacg

..........................ccgguuuucauuuucgaucugac..............................................................  19    0  TE1
..........................ccgguuuucauuuucgaucugacu.............................................................  501   0  TE1
..........................ccgguuuucauuuucgaucugacA.............................................................  1     1  TE1
..........................ccgguuuucauuuucAaucugacu.............................................................  1     1  TE1
..........................ccgguuuucauuuucgaucugGcu.............................................................  1     1  TE1
..........................ccgAuuuucauuuucgaucugacu.............................................................  1     1  TE1
..........................ccgguuuucauGuucgaucugacu.............................................................  1     1  TE1
..........................ccgguuuucauuuucgaucugacuA............................................................  4     1  TE1
..........................ccgguuuucauuuucgaucugacuu............................................................  4     0  TE1
...........................cgguuuucauuuucgaucugacu.............................................................  14    0  TE1
...........................cgguuuucauuuucgaucugacuu............................................................  1     0  TE1
............................gguuuucauuuucgaucugacu.............................................................  14    0  TE1
............................gguuuucauuuucgaucugacuu............................................................  1     0  TE1
............................gguuuucauuuucgaucugacuuU..........................................................  1     1  TE1
...................................................................agugagaucacuuugaaagcu.......................  1     0  TE1
....................................................................gugagaucacuuugaaag.........................  1     0  TE1
....................................................................gugagaucacuuugaaagcuga.....................  3     0  TE1
....................................................................Cugagaucacuuugaaagcuga.....................  1     1  TE1
....................................................................gugagaucacuuugaaagcugau....................  4     0  TE1
....................................................................Cugagaucacuuugaaagcugau....................  1     1  TE1
.....................................................................ugagaucacuuugaaag.........................  41    0  TE1
.....................................................................ugagaucacuuugaaagc........................  69    0  TE1
.....................................................................ugagaucacuuugaaagA........................  1     1  TE1
.....................................................................ugagaucacuCugaaagcu.......................  1     1  TE1
.....................................................................ugagaucacuuAgaaagcu.......................  1     1  TE1
.....................................................................ugagaucacuuugaaagcu.......................  69    0  TE1
.....................................................................ugagaucacuuugaaagcGg......................  3     1  TE1
.....................................................................uAagaucacuuugaaagcug......................  1     1  TE1
.....................................................................ugagaucacuuugaaaUcug......................  1     1  TE1
.....................................................................ugagaucacuuugaaagcug......................  633   0  TE1
.....................................................................ugagaucacuuugaaagcuA......................  2     1  TE1
.....................................................................ugagaucacuuugaaCgcug......................  1     1  TE1
.....................................................................ugagaucacuuGgaaagcug......................  1     1  TE1
.....................................................................ugagaCcacuuugaaagcug......................  1     1  TE1
.....................................................................ugagaucacuuuUaaagcuga.....................  1     1  TE1
.....................................................................ugagaucacuuugaaagcuga.....................  544   0  TE1
.....................................................................ugagaucacuuugaaagcugG.....................  6     1  TE1
.....................................................................ugagaucacuuugaaagcCga.....................  1     1  TE1
.....................................................................ugagaucacuuugaaagcugU.....................  1     1  TE1
.....................................................................ugagaCcacuuugaaagcuga.....................  1     1  TE1
.....................................................................ugagaucacuuugaaagcuAau....................  3     1  TE1
.....................................................................ugagaucacuuugaaagcugau....................  1805  0  TE1
.....................................................................ugagaucacuuugaCagcugau....................  1     1  TE1
.....................................................................ugagaucacuuCgaaagcugau....................  1     1  TE1
.....................................................................Cgagaucacuuugaaagcugau....................  2     1  TE1
.....................................................................ugagaucacuuugaaagcugaG....................  1     1  TE1
.....................................................................ugagaucacuuugaaaCcugau....................  2     1  TE1
.....................................................................ugaUaucacuuugaaagcugau....................  1     1  TE1
.....................................................................ugGgaucacuuugaaagcugau....................  1     1  TE1
.....................................................................ugagaucacuuugaaagAugau....................  2     1  TE1
.....................................................................uAagaucacuuugaaagcugau....................  2     1  TE1
.....................................................................ugagaucacuuugaaaAcugau....................  2     1  TE1
.....................................................................ugagaucacuuuAaaagcugau....................  3     1  TE1
.....................................................................ugagaucacuuugaaagcugaA....................  1     1  TE1
.....................................................................ugagauUacuuugaaagcugau....................  3     1  TE1
.....................................................................ugagaucacuuugaaCgcugau....................  1     1  TE1
.....................................................................ugagaucacuuugaaGgcugau....................  1     1  TE1
.....................................................................ugagaucacuuugaaaUcugau....................  3     1  TE1
.....................................................................ugagaucacuuugaaagcugauu...................  556   0  TE1
.....................................................................ugagaucacuuugaaagcCgauu...................  1     1  TE1
.....................................................................ugagaucacuuugaaagcugaGu...................  2     1  TE1
.....................................................................ugagaucacuuugaaagcugauC...................  1     1  TE1
.....................................................................ugagaucacuuugaaagcugauA...................  11    1  TE1
.....................................................................ugagauUacuuugaaagcugauu...................  1     1  TE1
.....................................................................ugagaucacuuCgaaagcugauu...................  1     1  TE1
.....................................................................ugagaucacuuugaaagcugauuC..................  1     1  TE1
.....................................................................ugagaucacuuugaaagcugauuu..................  25    0  TE1
.....................................................................ugagaucacuuugaaagcugauuA..................  12    1  TE1
.....................................................................ugaCaucacuuugaaagcugauuu..................  1     1  TE1
```

```
                         aga-bantam*
                                                                     aga-bantam
uguguggaugaaauguaaucacagaaccgguuuucauuuucgaucugacuuauuuuuuucacaaacaagugagaucacuuugaaagcugauuuuguacaauuaauucaacg

.........................Cccgguuuucauuuucgaucugacu.............................................................  1     1     FF2
..........................ccgguuuucauuuucgaucuga...............................................................  2     0     FF2
..........................ccgguuuucauuuucgaucugac..............................................................  27    0     FF2
..........................ccgguuuucauCuucgaucugacu.............................................................  1     1     FF2
..........................ccgguuuucauuuucgaucugGcu.............................................................  1     1     FF2
..........................ccAguuuucauuuucgaucugacu.............................................................  1     1     FF2
..........................ccgguuuucauuuucAaucugacu.............................................................  1     1     FF2
..........................ccgguuuucauuuucgaucugacA.............................................................  2     1     FF2
..........................ccgguuuucauuuucgaucugacu.............................................................  608   0     FF2
..........................ccgguuuucauuuucgUucugacu.............................................................  1     1     FF2
..........................ccgguuuucauuuucgaucugacG.............................................................  1     1     FF2
..........................ccgguuuucauuuucgaCcugacu.............................................................  1     1     FF2
..........................ccgguuCucauuuucgaucugacu.............................................................  1     1     FF2
..........................ccgguuuucauuuucgaucugacuu............................................................  2     0     FF2
..........................ccgguuuucauuuucgaucugacuA............................................................  1     1     FF2
..........................ccgguuuucauuuucgaucugacuua...........................................................  2     0     FF2
..........................ccgguuuucauuuucgaucugacuuU...........................................................  2     1     FF2
...........................cgguuuucauuuucgaucugacu.............................................................  8     0     FF2
............................gguuuucauuuucgaucugacu.............................................................  6     0     FF2
....................................................................Cugagaucacuuugaaag.........................  1     1     FF2
....................................................................gugagaucacuuugaaagcug......................  2     0     FF2
....................................................................gugagaucacuuugaaagcuga.....................  1     0     FF2
....................................................................gugagaucacuuugaaagcugau....................  1     0     FF2
....................................................................Cugagaucacuuugaaagcugau....................  2     1     FF2
....................................................................Cugagaucacuuugaaagcugauu...................  1     1     FF2
.....................................................................ugagaucacuuugaaag.........................  23    0     FF2
.....................................................................ugaAaucacuuugaaagc........................  1     1     FF2
.....................................................................ugagaucacuuugaaagc........................  64    0     FF2
.....................................................................ugagaucacuuugaaagcA.......................  1     1     FF2
.....................................................................Cgagaucacuuugaaagcu.......................  1     1     FF2
.....................................................................ugagaucacuuugaaagcu.......................  31    0     FF2
.....................................................................ugagaucacuuugGaagcug......................  1     1     FF2
.....................................................................ugagaucacuuugaaagcuA......................  1     1     FF2
.....................................................................ugagaucacuuugaaagcug......................  530   0     FF2
.....................................................................ugagaucacuuuAaaagcug......................  1     1     FF2
.....................................................................ugagaucacuuugaaagcGg......................  2     1     FF2
.....................................................................ugagUucacuuugaaagcuga.....................  1     1     FF2
.....................................................................ugagaucacuuugaaagcugG.....................  7     1     FF2
.....................................................................ugagaucacuuugaaagcuAa.....................  1     1     FF2
.....................................................................ugagaucacuuugaaaAcuga.....................  1     1     FF2
.....................................................................ugagaCcacuuugaaagcuga.....................  1     1     FF2
.....................................................................ugagaucacuCugaaagcuga.....................  1     1     FF2
.....................................................................ugagaucacuuugaaagcuga.....................  964   0     FF2
.....................................................................ugagaucaUuuugaaagcuga.....................  1     1     FF2
.....................................................................ugagauAacuuugaaagcuga.....................  1     1     FF2
.....................................................................uAagaucacuuugaaagcuga.....................  1     1     FF2
.....................................................................ugagaucacuuCgaaagcuga.....................  1     1     FF2
.....................................................................ugagaucacuuAgaaagcuga.....................  1     1     FF2
.....................................................................Ggagaucacuuugaaagcugau....................  1     1     FF2
.....................................................................ugagaucacCuugaaagcugau....................  1     1     FF2
.....................................................................ugagaucacuuugaaagcugau....................  2352  0     FF2
.....................................................................ugagaucacuuugaGagcugau....................  1     1     FF2
.....................................................................ugagaucacuuugaaagcugaC....................  2     1     FF2
.....................................................................ugagGucacuuugaaagcugau....................  1     1     FF2
.....................................................................ugagauUacuuugaaagcugau....................  1     1     FF2
.....................................................................Cgagaucacuuugaaagcugau....................  1     1     FF2
.....................................................................ugagaucUcuuugaaagcugau....................  1     1     FF2
.....................................................................ugagaucacuuugaaagGugau....................  1     1     FF2
.....................................................................uAagaucacuuugaaagcugau....................  1     1     FF2
.....................................................................ugagaucacuuugGaagcugau....................  1     1     FF2
.....................................................................ugagaucacuuugaaagcuAau....................  1     1     FF2
.....................................................................ugagaucacuuCgaaagcugau....................  2     1     FF2
.....................................................................ugagaucacuuugaaagcugaA....................  7     1     FF2
.....................................................................ugagaCcacuuugaaagcugau....................  1     1     FF2
.....................................................................ugagaucacuuugaaagcCgau....................  2     1     FF2
.....................................................................ugagaucacuuuAaaagcugau....................  2     1     FF2
.....................................................................ugagaucacuuugaaagcugaG....................  4     1     FF2
.....................................................................ugagaucacuuugaaagcCgauu...................  1     1     FF2
```

```
                          aga-bantam*
                                                                     aga-bantam
uguguggaugaaauguaaucacagaaccgguuuucauuuucgaucugacuuauuuuuuucacaaacaagugagaucacuuugaaagcugauuuuguacaauuaauucaacg

.....................................................................ugagaucacuuugaaagcugauA...................   14     1   FF2
.....................................................................ugagaucacuuugaaaAcugauu...................   1      1   FF2
.....................................................................Agagaucacuuugaaagcugauu...................   1      1   FF2
.....................................................................ugagaucacuuugaaagcugGuu...................   1      1   FF2
.....................................................................ugagaucacuuugaaagcugUuu...................   1      1   FF2
.....................................................................ugagaucacuuugGaagcugauu...................   1      1   FF2
.....................................................................ugagaucacuuugaaagcugauu...................   683    0   FF2
.....................................................................uAagaucacuuugaaagcugauu...................   1      1   FF2
.....................................................................ugagauAacuuugaaagcugauu...................   1      1   FF2
.....................................................................ugagaucacuAugaaagcugauu...................   1      1   FF2
.....................................................................ugagaCcacuuugaaagcugauu...................   1      1   FF2
.....................................................................ugagaucacuCugaaagcugauuu..................   1      1   FF2
.....................................................................ugagaucacuuugaaagcugauuA..................   7      1   FF2
.....................................................................ugagaucacuuugaaagcugauuu..................   38     0   FF2
......................................................................gagaucacuuugaaagcugauuu..................   1      0   FF2

.........................Cccgguuuucauuuucgaucuga...............................................................   1      1   MF2
.........................Cccgguuuucauuuucgaucugacu.............................................................   3      1   MF2
..........................ccgguuuucauuuucgaucug................................................................   7      0   MF2
..........................ccgguuuucauuuucgaucuga...............................................................   30     0   MF2
..........................ccgguuuucauuuucgaucugac..............................................................   222    0   MF2
..........................ccgguuuucauuuucgaucugaA..............................................................   4      1   MF2
..........................ccgguuuucaAuuucgaucugac..............................................................   1      1   MF2
..........................ccgguuuucauuCucgaucugac..............................................................   1      1   MF2
..........................ccgguuuucauCuucgaucugac..............................................................   1      1   MF2
..........................ccgguuuucaCuuucgaucugacu.............................................................   3      1   MF2
..........................ccgguuuucauuuucgaucuAacu.............................................................   3      1   MF2
..........................ccgguuuucauuuucgaucAgacu.............................................................   1      1   MF2
..........................ccggAuuucauuuucgaucugacu.............................................................   2      1   MF2
..........................ccgguuuucauuuucgaucugGcu.............................................................   3      1   MF2
..........................ccggCuuucauuuucgaucugacu.............................................................   4      1   MF2
..........................Acgguuuucauuuucgaucugacu.............................................................   1      1   MF2
..........................ccgguuuucauuuuUgaucugacu.............................................................   2      1   MF2
..........................ccgguuuucauuuucgaucugacu.............................................................   5097   0   MF2
..........................ccgguuuucauuuucgaucugacA.............................................................   23     1   MF2
..........................ccgguuuucauGuucgaucugacu.............................................................   2      1   MF2
..........................ccgguuuucauuuucgaucCgacu.............................................................   2      1   MF2
..........................ccgguuuuUauuuucgaucugacu.............................................................   4      1   MF2
..........................ccgguGuucauuuucgaucugacu.............................................................   1      1   MF2
..........................ccgguuuucauuuucgaucugacG.............................................................   7      1   MF2
..........................ccgguuuucauuuucgaCcugacu.............................................................   4      1   MF2
..........................ccgguuuucauuuucgaucugaAu.............................................................   2      1   MF2
..........................ccgguuuucauuCucgaucugacu.............................................................   5      1   MF2
..........................ccgguuuucauuuucgauUugacu.............................................................   1      1   MF2
..........................ccggGuuucauuuucgaucugacu.............................................................   1      1   MF2
..........................ccgguuCucauuuucgaucugacu.............................................................   4      1   MF2
..........................ccgAuuuucauuuucgaucugacu.............................................................   1      1   MF2
..........................Ucgguuuucauuuucgaucugacu.............................................................   2      1   MF2
..........................ccgguCuucauuuucgaucugacu.............................................................   2      1   MF2
..........................ccgguuuucauuuucgaucugCcu.............................................................   1      1   MF2
..........................ccgguuAucauuuucgaucugacu.............................................................   1      1   MF2
..........................ccgguuuucauuuucgaucugUcu.............................................................   1      1   MF2
..........................ccCguuuucauuuucgaucugacu.............................................................   1      1   MF2
..........................ccgguuuucauuuucgUucugacu.............................................................   2      1   MF2
..........................ccgguuuucGuuuucgaucugacu.............................................................   1      1   MF2
..........................ccAguuuucauuuucgaucugacu.............................................................   3      1   MF2
..........................ccgguuuucauuuucgaucugacC.............................................................   6      1   MF2
..........................ccgguuuucauuuucgaucugacuA............................................................   1      1   MF2
..........................ccgguuuucauuuucgaucugacAu............................................................   2      1   MF2
..........................ccgguuuucauuuucgaucugacuu............................................................   23     0   MF2
..........................ccgguuuucauuuucgaucugacuuauu.........................................................   2      0   MF2
..........................ccgguuuucauuuucgaucugacuuauuuuuuucacaa...............................................   1      0   MF2
..........................ccgguuuucauuuucgaucugacuuauuuuuuucacaaacaagA.........................................   1      1   MF2
...........................cgguuuucauuuucgaucugac..............................................................   2      0   MF2
...........................cggCuuucauuuucgaucugacu.............................................................   1      1   MF2
...........................cgguuuucauuuucgauAugacu.............................................................   1      1   MF2
...........................cgguuuucauuuucgaucugacu.............................................................   156    0   MF2
...........................cgguuuucauuuucgaucugacuu............................................................   1      0   MF2
...........................cgguuuucauuuucgaucugacuuU...........................................................   1      1   MF2
```

```
                          aga-bantam*
                                                                     aga-bantam
uguguggaugaaauguaaucacagaaccgguuuucauuuucgaucugacuuauuuuuuucacaaacaagugagaucacuuugaaagcugauuuuguacaauuaauucaacg

...........................cgguuuucauuuucgaucugacuuCu..........................................................  2     1   MF2
............................gguuuucauuuucgaucugac..............................................................  1     0   MF2
............................gguuuucauuuucgaucugacu.............................................................  74    0   MF2
............................gguuuCcauuuucgaucugacu.............................................................  1     1   MF2
............................gguuuucauuuucgaucugacuu............................................................  11    0   MF2
............................gguuuucauuuucgaucugacuua...........................................................  1     0   MF2
............................gguuuucauuuucgaucugacuuU...........................................................  3     1   MF2
.............................guuuucauuuucgaucugacu.............................................................  1     0   MF2
..............................uuuucauuuucgaucugacu.............................................................  1     0   MF2
................................uucauuuucgaucugacu.............................................................  1     0   MF2
....................................................................gugagaucacuuugaaagc........................  1     0   MF2
....................................................................gugagaucacuuugaaagcu.......................  7     0   MF2
....................................................................gugagauAacuuugaaagcug......................  1     1   MF2
....................................................................Cugagaucacuuugaaagcug......................  1     1   MF2
....................................................................gugagaucacuuugaaagcug......................  13    0   MF2
....................................................................Cugagaucacuuugaaagcuga.....................  3     1   MF2
....................................................................gugagaucacuuugaaagcuga.....................  13    0   MF2
....................................................................gugagaucacuuugaaagcugau....................  11    0   MF2
....................................................................Cugagaucacuuugaaagcugau....................  19    1   MF2
....................................................................gugagaucacuuugaaagcugaA....................  2     1   MF2
....................................................................Cugagaucacuuugaaagcugauu...................  2     1   MF2
.....................................................................ugagaucacuuugaaag.........................  139   0   MF2
.....................................................................ugagaucacuuugaaaA.........................  3     1   MF2
.....................................................................ugagaucacuuGgaaagc........................  1     1   MF2
.....................................................................ugagaucCcuuugaaagc........................  1     1   MF2
.....................................................................ugagauUacuuugaaagc........................  1     1   MF2
.....................................................................ugagaucacuuugaaagA........................  1     1   MF2
.....................................................................ugagaucacuuugaaagc........................  401   0   MF2
.....................................................................ugagaucacuuugUaagc........................  1     1   MF2
.....................................................................ugagaucacuuugaaagcC.......................  1     1   MF2
.....................................................................ugagaucacuuugaaagcA.......................  2     1   MF2
.....................................................................ugagaucacuuugaaagcu.......................  248   0   MF2
.....................................................................ugagaucacuuugaaagcG.......................  2     1   MF2
.....................................................................Ggagaucacuuugaaagcug......................  1     1   MF2
.....................................................................ugagaCcacuuugaaagcug......................  4     1   MF2
.....................................................................ugagaucacuuugaaagcug......................  4707  0   MF2
.....................................................................ugagaucacuuugaaagcuA......................  2     1   MF2
.....................................................................uAagaucacuuugaaagcug......................  4     1   MF2
.....................................................................ugagaucacuuugaaGgcug......................  1     1   MF2
.....................................................................ugagauUacuuugaaagcug......................  1     1   MF2
.....................................................................ugagaucacuuAgaaagcug......................  1     1   MF2
.....................................................................ugagaucacuuugaaCgcug......................  1     1   MF2
.....................................................................ugGgaucacuuugaaagcug......................  2     1   MF2
.....................................................................ugagaucacuuCgaaagcug......................  4     1   MF2
.....................................................................ugagaucaAuuugaaagcug......................  1     1   MF2
.....................................................................ugagaucacuuugaaagcCg......................  1     1   MF2
.....................................................................ugagauAacuuugaaagcug......................  3     1   MF2
.....................................................................ugagaucacuuugaaagcGg......................  9     1   MF2
.....................................................................ugagaGcacuuugaaagcug......................  2     1   MF2
.....................................................................ugagaucacuAugaaagcug......................  1     1   MF2
.....................................................................ugagaucacuuuAaaagcug......................  1     1   MF2
.....................................................................ugagaucacuuugaaaUcug......................  2     1   MF2
.....................................................................ugagaucacuCugaaagcug......................  3     1   MF2
.....................................................................ugagaAcacuuugaaagcug......................  1     1   MF2
.....................................................................ugagaucacCuugaaagcug......................  1     1   MF2
.....................................................................ugagaucacuuugaaaAcug......................  1     1   MF2
.....................................................................uUagaucacuuugaaagcug......................  1     1   MF2
.....................................................................ugaAaucacuuugaaagcug......................  2     1   MF2
.....................................................................ugagaucacuuugaaagcuUa.....................  1     1   MF2
.....................................................................ugagaucacuuugaaagGuga.....................  1     1   MF2
.....................................................................ugagaucacCuugaaagcuga.....................  5     1   MF2
.....................................................................ugagaucaAuuugaaagcuga.....................  1     1   MF2
.....................................................................ugagCucacuuugaaagcuga.....................  1     1   MF2
.....................................................................ugGgaucacuuugaaagcuga.....................  2     1   MF2
.....................................................................ugagaucacuuugaaagcCga.....................  3     1   MF2
.....................................................................ugagaucacuuugaaagcuga.....................  6885  0   MF2
.....................................................................ugagaGcacuuugaaagcuga.....................  1     1   MF2
.....................................................................ugagaucacuuugaaagUuga.....................  1     1   MF2
.....................................................................Ggagaucacuuugaaagcuga.....................  1     1   MF2
```

aga-bantam*

aga-bantam

uguguggaugaaauguaaucacagaaccgguuuucauuuucgaucugacuuauuuuuuucacaaacaagugagaucacuuugaaagcugauuuuguacaauuaauucaacg

....ugagaucacuuCgaaagcuga.....................	2	1	MF2
....ugagaucacuuugaaagcugU.....................	3	1	MF2
....ugagaCcacuuugaaagcuga.....................	3	1	MF2
....ugagaucacuuugaaagcugC.....................	1	1	MF2
....ugagaucacuuugaaagcuAa.....................	8	1	MF2
....ugagaucacuuugaaagcugG.....................	53	1	MF2
....ugagaucacuuugaaaCcuga.....................	4	1	MF2
....ugagaucacGuugaaagcuga.....................	1	1	MF2
....ugagaucacuuuAaaagcuga.....................	4	1	MF2
....ugagUucacuuugaaagcuga.....................	1	1	MF2
....ugagaucacuGugaaagcuga.....................	1	1	MF2
....ugagaucaGuuugaaagcuga.....................	1	1	MF2
....ugagauUacuuugaaagcuga.....................	5	1	MF2
....ugagaucGcuuugaaagcuga.....................	2	1	MF2
....ugagaucacuuGgaaagcuga.....................	2	1	MF2
....ugagauAacuuugaaagcuga.....................	1	1	MF2
....ugagauGacuuugaaagcuga.....................	1	1	MF2
....ugagaucacuCugaaagcuga.....................	3	1	MF2
....Cgagaucacuuugaaagcuga.....................	1	1	MF2
....ugagaucacuuuCaaagcuga.....................	1	1	MF2
....ugaAaucacuuugaaagcuga.....................	3	1	MF2
....ugagaucacuuugaaaUcuga.....................	1	1	MF2
....ugagaucacuuAgaaagcuga.....................	3	1	MF2
....ugaUaucacuuugaaagcuga.....................	1	1	MF2
....uAagaucacuuugaaagcuga.....................	3	1	MF2
....ugagaucUcuuugaaagcuga.....................	1	1	MF2
....ugagaucacuuugaaaAcuga.....................	2	1	MF2
....Agagaucacuuugaaagcuga.....................	1	1	MF2
....uUagaucacuuugaaagcugau....................	1	1	MF2
....ugagaucacuuugaaagcugaC....................	11	1	MF2
....ugagaucacuuugaaagcCgau....................	15	1	MF2
....ugagaucacCuugaaagcugau....................	13	1	MF2
....ugagaucacuuugaaagcugaG....................	20	1	MF2
....ugagaucacuuuUaaagcugau....................	2	1	MF2
....ugagaucCcuuugaaagcugau....................	1	1	MF2
....ugagaucacuuugaUagcugau....................	1	1	MF2
....ugagaucacuuugaaagGugau....................	1	1	MF2
....ugagaucacuuugaaaCcugau....................	6	1	MF2
....uAagaucacuuugaaagcugau....................	16	1	MF2
....ugagaucacuuugaaaAcugau....................	10	1	MF2
....ugagaucacuuugaaagcugau....................	21211	0	MF2
....ugagaucacuuugaaagUugau....................	1	1	MF2
....ugagaucacuuuAaaagcugau....................	26	1	MF2
....ugagaucacuuugaaGgcugau....................	2	1	MF2
....ugagaucGcuuugaaagcugau....................	4	1	MF2
....ugaAaucacuuugaaagcugau....................	2	1	MF2
....ugagaucacuuugaaagcugGu....................	4	1	MF2
....Cgagaucacuuugaaagcugau....................	7	1	MF2
....ugagaucacuuugaaagAugau....................	8	1	MF2
....ugagaucacuuugaaaUcugau....................	2	1	MF2
....ugagaucacuuugaaagcugCu....................	2	1	MF2
....uCagaucacuuugaaagcugau....................	2	1	MF2
....ugagaucacuuugaaagcuAau....................	15	1	MF2
....ugagaCcacuuugaaagcugau....................	13	1	MF2
....ugagaucacuuugUaagcugau....................	1	1	MF2
....ugagaucacuuuGCaagcugau....................	1	1	MF2
....ugagaucacuCugaaagcugau....................	7	1	MF2
....ugagaucacuuugaaagcAgau....................	1	1	MF2
....ugagaucacAuugaaagcugau....................	7	1	MF2
....ugagaucacuuugaaCgcugau....................	1	1	MF2
....ugagaucacuGugaaagcugau....................	1	1	MF2
....Ggagaucacuuugaaagcugau....................	2	1	MF2
....ugagaucacuuCgaaagcugau....................	12	1	MF2
....ugaCaucacuuugaaagcugau....................	2	1	MF2
....ugGgaucacuuugaaagcugau....................	1	1	MF2
....ugagaAcacuuugaaagcugau....................	2	1	MF2
....ugagaucaUuuugaaagcugau....................	4	1	MF2
....ugagauUacuuugaaagcugau....................	9	1	MF2
....ugagaucacuuGgaaagcugau....................	4	1	MF2

```
                          aga-bantam*
                                                                     aga-bantam
ugugugaugaaauguaaucacagaaccgguuuucauuuucgaucugacuuauuuuuuucacaaacaagugagaucacuuugaaagcugauuuuguacaauuaauucaacg

.....................................................................ugagaucacuAugaaagcugau....................   1      1      MF2
.....................................................................ugagaucacuuugaaagcugUu....................   1      1      MF2
.....................................................................ugagaucacuuugGaagcugau....................   1      1      MF2
.....................................................................ugagaucaAuuugaaagcugau....................   2      1      MF2
.....................................................................Agagaucacuuugaaagcugau....................   2      1      MF2
.....................................................................ugagaucUcuuugaaagcugau....................   1      1      MF2
.....................................................................ugagaucacuuugaaagcugaA....................   77     1      MF2
.....................................................................ugagauAacuuugaaagcugau....................   2      1      MF2
.....................................................................ugagCucacuuugaaagcugau....................   1      1      MF2
.....................................................................ugagGucacuuugaaagcugau....................   3      1      MF2
.....................................................................ugagaucacuuAgaaagcugau....................   12     1      MF2
.....................................................................ugGgaucacuuugaaagcugauu...................   2      1      MF2
.....................................................................ugagaucacuCugaaagcugauu...................   6      1      MF2
.....................................................................ugagUucacuuugaaagcugauu...................   1      1      MF2
.....................................................................ugagaucacuuugaaagcugaCu...................   12     1      MF2
.....................................................................ugagaucacuuAgaaagcugauu...................   4      1      MF2
.....................................................................ugagaucacuuugaaagcugaGu...................   4      1      MF2
.....................................................................ugUgaucacuuugaaagcugauu...................   4      1      MF2
.....................................................................ugagaucacuuugaaagcugaAu...................   10     1      MF2
.....................................................................Agagaucacuuugaaagcugauu...................   1      1      MF2
.....................................................................uAagaucacuuugaaagcugauu...................   6      1      MF2
.....................................................................ugagaucacuuugaaagcugauA...................   131    1      MF2
.....................................................................ugagaucaUuuugaaagcugauu...................   4      1      MF2
.....................................................................ugagaucacCuugaaagcugauu...................   2      1      MF2
.....................................................................ugagaucGcuuugaaagcugauu...................   1      1      MF2
.....................................................................ugagaucacuuugaaaCcugauu...................   2      1      MF2
.....................................................................ugagaucacuuugaGagcugauu...................   1      1      MF2
.....................................................................ugagaucacuuugaaagcugauG...................   3      1      MF2
.....................................................................ugagaucacuuugaaagcuAauu...................   11     1      MF2
.....................................................................ugagaucacuuugaaaAcugauu...................   4      1      MF2
.....................................................................ugagaucacuuuAaaagcugauu...................   9      1      MF2
.....................................................................Cgagaucacuuugaaagcugauu...................   3      1      MF2
.....................................................................ugagaucacuuugaaagcugCuu...................   1      1      MF2
.....................................................................ugagaucacAuugaaagcugauu...................   2      1      MF2
.....................................................................ugagaucCcuuugaaagcugauu...................   1      1      MF2
.....................................................................ugagauUacuuugaaagcugauu...................   8      1      MF2
.....................................................................ugagGucacuuugaaagcugauu...................   1      1      MF2
.....................................................................ugagaucacuuugaaaUcugauu...................   2      1      MF2
.....................................................................ugagaAcacuuugaaagcugauu...................   1      1      MF2
.....................................................................ugagaucacuuugaaagAugauu...................   1      1      MF2
.....................................................................ugagaucacuuugaaagcuUauu...................   1      1      MF2
.....................................................................ugagaucacuuugaaagcugauu...................   9141   0      MF2
.....................................................................Ggagaucacuuugaaagcugauu...................   1      1      MF2
.....................................................................ugagaCcacuuugaaagcugauu...................   4      1      MF2
.....................................................................ugagaucacuuugaaagcugauC...................   1      1      MF2
.....................................................................ugagaucacuuugaaagcCgauu...................   2      1      MF2
.....................................................................ugagaucacuuugaaagGugauu...................   1      1      MF2
.....................................................................ugagaucacuuCgaaagcugauu...................   9      1      MF2
.....................................................................ugagaucacuuugaaagcugGuu...................   1      1      MF2
.....................................................................ugagaucacuuugaaagcugauuG..................   4      1      MF2
.....................................................................ugaUaucacuuugaaagcugauuu..................   1      1      MF2
.....................................................................ugagaAcacuuugaaagcugauuu..................   1      1      MF2
.....................................................................ugagaucacuuuAaaagcugauuu..................   1      1      MF2
.....................................................................ugagaucacuuugaaaAcugauuu..................   1      1      MF2
.....................................................................ugagaucacuuugaaagcugauuA..................   89     1      MF2
.....................................................................Agagaucacuuugaaagcugauuu..................   1      1      MF2
.....................................................................Cgagaucacuuugaaagcugauuu..................   1      1      MF2
.....................................................................ugagaucacuuugaaagcugauuC..................   8      1      MF2
.....................................................................ugagaucacuuugaaagcugaAuu..................   1      1      MF2
.....................................................................ugagaucacuuCgaaagcugauuu..................   1      1      MF2
.....................................................................ugagaucacuuugaaagcugauuu..................   563    0      MF2
.....................................................................ugagaucacuuugaaagcugauAu..................   2      1      MF2
.....................................................................uAagaucacuuugaaagcugauuu..................   1      1      MF2
.....................................................................ugagaucaUuuugaaagcugauuu..................   2      1      MF2
.....................................................................ugagaucacuuugaaagcugauAuu.................   1      1      MF2
.....................................................................ugagaucacuuugaaagcugauuCu.................   2      1      MF2
.....................................................................ugagaucacuuugaaagcugauuuuU................   2      1      MF2
..........................................................................ucacuuugaaagcugau....................   1      0      MF2

..........................ccgguuuucauuuucgauc..................................................................   2      0      FW2
```

```
                          aga-bantam*
                                                                     aga-bantam
uguguggaugaaauguaaucacagaaccgguuuucauuuucgaucugacuuauuuuuuucacaaacaagugagaucacuuugaaagcugauuuuguacaauuaauucaacg

..........................ccgguuuucauuuucgaucuga...............................................................    2     0    FW2
..........................ccgguuuucauuuucgaucugac..............................................................    23    0    FW2
..........................ccgAuuucauuuucgaucugacu.............................................................    2     1    FW2
..........................ccgguuuucauuuucgauAugacu.............................................................    1     1    FW2
..........................ccgguuuucauuuuUgaucugacu.............................................................    1     1    FW2
..........................ccgguuuucauuuucgaucugacA.............................................................    7     1    FW2
..........................ccggCuuucauuuucgaucugacu.............................................................    2     1    FW2
..........................ccgguCuucauuuucgaucugacu.............................................................    1     1    FW2
..........................Acgguuuucauuuucgaucugacu.............................................................    1     1    FW2
..........................ccgguuuucauuuucgaucCgacu.............................................................    1     1    FW2
..........................ccgguuuucauuuucgGucugacu.............................................................    1     1    FW2
..........................Ucgguuuucauuuucgaucugacu.............................................................    1     1    FW2
..........................ccgguuuucaCuuucgaucugacu.............................................................    1     1    FW2
..........................ccgguuuucauuuucgaCcugacu.............................................................    1     1    FW2
..........................ccgguuuucauuuucgaucugGcu.............................................................    1     1    FW2
..........................ccAguuuucauuuucgaucugacu.............................................................    1     1    FW2
..........................ccgguuuucauuuucgaucugacu.............................................................    826   0    FW2
..........................ccgguuuucauuuucgaucugacuu............................................................    5     0    FW2
..........................ccgguuuucauuuucgaucugacuA............................................................    3     1    FW2
...........................cgguuuucauuuucgaucugac..............................................................    1     0    FW2
...........................cgguuuucauuuucUaucugacu.............................................................    1     1    FW2
...........................cgguuCucauuuucgaucugacu.............................................................    1     1    FW2
...........................cgguuuucauuuucgaucugacu.............................................................    128   0    FW2
...........................cgguuuucauuuucgaucugacuu............................................................    4     0    FW2
............................gguuuucauuuucgaucugacu.............................................................    30    0    FW2
............................gguuuuAauuuucgaucugacu.............................................................    1     1    FW2
............................gguuuucauuuucgaucugacuu............................................................    12    0    FW2
.................................ucauuuucgaucugacu.............................................................    1     0    FW2
...................................................................Ggugagaucacuuugaaagcugau....................    1     1    FW2
...................................................................agugagaucacuuugaaagcugauu...................    2     0    FW2
....................................................................gugagaucaAuuugaaagc........................    1     1    FW2
....................................................................gugagaucacuuugaaagcug......................    1     0    FW2
....................................................................gugagaucacuuugaaagcuga.....................    5     0    FW2
....................................................................Cugagaucacuuugaaagcugau....................    9     1    FW2
....................................................................gugagaucacuuugaaagcugau....................    6     0    FW2
....................................................................Cugagaucacuuugaaagcugauu...................    1     1    FW2
.....................................................................ugagaucacuuugaaag.........................    45    0    FW2
.....................................................................ugagaucacuuugaaagc........................    104   0    FW2
.....................................................................ugagaucacuuugaaagcu.......................    25    0    FW2
.....................................................................ugagaucacuuugaaagcug......................    353   0    FW2
.....................................................................ugagaucacuCugaaagcug......................    1     1    FW2
.....................................................................ugagaucacuuuAaaagcuga.....................    1     1    FW2
.....................................................................ugagaucacuuuUaaagcuga.....................    1     1    FW2
.....................................................................ugagaucacuuugaaagcugG.....................    3     1    FW2
.....................................................................ugagaucacuuugaaagcuAa.....................    1     1    FW2
.....................................................................ugagaucacuuugaaagcuga.....................    959   0    FW2
.....................................................................uAagaucacuuugaaagcuga.....................    1     1    FW2
.....................................................................ugagaucacuuGgaaagcuga.....................    1     1    FW2
.....................................................................ugagaucacuuugaaagcugaC....................    2     1    FW2
.....................................................................Agagaucacuuugaaagcugau....................    1     1    FW2
.....................................................................ugagaCcacuuugaaagcugau....................    2     1    FW2
.....................................................................ugagauAacuuugaaagcugau....................    1     1    FW2
.....................................................................ugagauUacuuugaaagcugau....................    4     1    FW2
.....................................................................ugagaucacAuugaaagcugau....................    1     1    FW2
.....................................................................ugagaucacuGugaaagcugau....................    1     1    FW2
.....................................................................ugaAaucacuuugaaagcugau....................    1     1    FW2
.....................................................................ugagaucacCuugaaagcugau....................    6     1    FW2
.....................................................................ugagaucacuuugaaagcCgau....................    1     1    FW2
.....................................................................ugagaucacuuugaaaUcugau....................    1     1    FW2
.....................................................................ugGgaucacuuugaaagcugau....................    2     1    FW2
.....................................................................ugagaucacuuugaaGgcugau....................    1     1    FW2
.....................................................................ugagaucacuuugaGagcugau....................    2     1    FW2
.....................................................................ugagaucacuuugaaagcugau....................    8137  0    FW2
.....................................................................ugagaucGcuuugaaagcugau....................    2     1    FW2
.....................................................................ugagaucaAuuugaaagcugau....................    1     1    FW2
.....................................................................ugagaucacuuuAaaagcugau....................    2     1    FW2
.....................................................................ugagaucacuCugaaagcugau....................    4     1    FW2
.....................................................................ugUgaucacuuugaaagcugau....................    1     1    FW2
.....................................................................ugagaucacuuugaaagcugaG....................    1     1    FW2
```

```
                          aga-bantam*
                                                                     aga-bantam
uguguggaugaaauguaaucacagaaccgguuuucauuuucgaucugacuuauuuuuuucacaaacaagugagaucacuuugaaagcugauuuuguacaauuaauucaacg

.....................................................................ugagaucacuuugaaagcuCau....................  1     1  FW2
.....................................................................ugagaucacuuugaaagcugGu....................  2     1  FW2
.....................................................................ugagaucaUuuugaaagcugau....................  1     1  FW2
.....................................................................ugaUaucacuuugaaagcugau....................  1     1  FW2
.....................................................................Cgagaucacuuugaaagcugau....................  4     1  FW2
.....................................................................ugagaucacuuugaaagcugUu....................  3     1  FW2
.....................................................................ugagaucacuAugaaagcugau....................  1     1  FW2
.....................................................................ugagaucacuuugaaagcuAau....................  5     1  FW2
.....................................................................uAagaucacuuugaaagcugau....................  6     1  FW2
.....................................................................ugagaucacuuAgaaagcugau....................  2     1  FW2
.....................................................................ugagaucacuuugaaaAcugau....................  2     1  FW2
.....................................................................ugagaucacuuugaaaCcugau....................  4     1  FW2
.....................................................................ugagaucacGuugaaagcugau....................  1     1  FW2
.....................................................................ugagGucacuuugaaagcugau....................  1     1  FW2
.....................................................................ugagaucacuuCgaaagcugau....................  6     1  FW2
.....................................................................ugagaucacuuugaaagAugau....................  2     1  FW2
.....................................................................ugagaAcacuuugaaagcugau....................  2     1  FW2
.....................................................................ugagaucacuuugaaagcuAauu...................  2     1  FW2
.....................................................................ugagaucCcuuugaaagcugauu...................  1     1  FW2
.....................................................................ugagaucacuuugaaagcugUuu...................  2     1  FW2
.....................................................................ugagaucacuuugaaagcugauA...................  31    1  FW2
.....................................................................ugGgaucacuuugaaagcugauu...................  1     1  FW2
.....................................................................ugagaucacuuugaaagcugCuu...................  1     1  FW2
.....................................................................ugagGucacuuugaaagcugauu...................  3     1  FW2
.....................................................................ugagaucacuuugaaagcugGuu...................  3     1  FW2
.....................................................................ugagaucacGuugaaagcugauu...................  2     1  FW2
.....................................................................uAagaucacuuugaaagcugauu...................  1     1  FW2
.....................................................................ugagaucacuuugaaagcugaCu...................  2     1  FW2
.....................................................................ugagaucacuCugaaagcugauu...................  1     1  FW2
.....................................................................ugagaCcacuuugaaagcugauu...................  1     1  FW2
.....................................................................ugagaucacuuugaaagcCgauu...................  2     1  FW2
.....................................................................uUagaucacuuugaaagcugauu...................  1     1  FW2
.....................................................................ugagaucaGuuugaaagcugauu...................  1     1  FW2
.....................................................................ugagaucacuuCgaaagcugauu...................  1     1  FW2
.....................................................................ugagUucacuuugaaagcugauu...................  1     1  FW2
.....................................................................ugagaucacuuugaaagcugauu...................  4004  0  FW2
.....................................................................ugagaucacuuugaaagUugauu...................  1     1  FW2
.....................................................................ugagaucaUuuugaaagcugauu...................  1     1  FW2
.....................................................................ugagauUacuuugaaagcugauu...................  3     1  FW2
.....................................................................Cgagaucacuuugaaagcugauu...................  1     1  FW2
.....................................................................ugagaucacuuugaaagcugaAu...................  1     1  FW2
.....................................................................ugagaucacuuugaaaCcugauu...................  1     1  FW2
.....................................................................ugagaucacuuuAaaagcugauu...................  3     1  FW2
.....................................................................ugagaucacuuugaaagAugauu...................  2     1  FW2
.....................................................................ugagaucacCuugaaagcugauu...................  1     1  FW2
.....................................................................ugagaucacuuugaaagcugauuC..................  9     1  FW2
.....................................................................Cgagaucacuuugaaagcugauuu..................  1     1  FW2
.....................................................................ugagaucacuuugaaagcugauuA..................  15    1  FW2
.....................................................................ugagaucacuuugaaagcugauuu..................  134   0  FW2
.....................................................................ugagaucacuuugaaagcuAauuu..................  1     1  FW2
.....................................................................ugagaucacuuGgaaagcugauuu..................  1     1  FW2
.....................................................................uAagaucacuuugaaagcugauuu..................  1     1  FW2
......................................................................Uagaucacuuugaaagcugau....................  1     1  FW2
.......................................................................agaucacuuugaaagcu.......................  1     0  FW2
.......................................................................agaucacuuugaaagcugauu...................  1     0  FW2
........................................................................gaucacuuugaaagcuga.....................  1     0  FW2
.........................................................................aucacuuugaaagcugauG...................  2     1  FW2
...........................................................................cacuuugaaagcugauu...................  1     0  FW2

..........................ccgguuuucauuuucgauc..................................................................  1     0  OV1
..........................ccgguuuucauuuucgaucug................................................................  2     0  OV1
..........................ccgguuuucauuuucgaucugG...............................................................  1     1  OV1
..........................ccgguuuucauuuucgaucuga...............................................................  13    0  OV1
..........................ccgguuuucauuuucgaucugac..............................................................  57    0  OV1
..........................ccgguuuucauuuucgaucugaA..............................................................  1     1  OV1
..........................ccgguuuuUauuuucgaucugacu.............................................................  2     1  OV1
..........................ccgguuuucauuuucAaucugacu.............................................................  1     1  OV1
..........................ccgguuuucauuuucgUucugacu.............................................................  2     1  OV1
..........................ccCguuuucauuuucgaucugacu.............................................................  1     1  OV1
```

```
                          aga-bantam*
                                                                     aga-bantam
uguguggaugaaauguaaucacagaaccgguuuucauuuucgaucugacuuauuuuuuucacaaacaagugagaucacuuugaaagcugauuuuguacaauuaauucaacg

..........................ccggCuuucauuuucgaucugacu.............................................................   2      1      OV1
..........................ccgguuuucCuuuucgaucugacu.............................................................   1      1      OV1
..........................ccgguuCucauuuucgaucugacu.............................................................   1      1      OV1
..........................ccggGuuucauuuucgaucugacu.............................................................   1      1      OV1
..........................ccgguuuucUuuuucgaucugacu.............................................................   1      1      OV1
..........................ccgguuuucauuuucgaucugaAu.............................................................   1      1      OV1
..........................ccgguuuucauCuucgaucugacu.............................................................   3      1      OV1
..........................ccgguuuucauuCucgaucugacu.............................................................   1      1      OV1
..........................cAgguuuucauuuucgaucugacu.............................................................   1      1      OV1
..........................ccAguuuucauuuucgaucugacu.............................................................   2      1      OV1
..........................ccgguuuucauGuucgaucugacu.............................................................   1      1      OV1
..........................ccgguuuucauuuucgaucugacA.............................................................   17     1      OV1
..........................ccgguuuucauuuucgaucGgacu.............................................................   1      1      OV1
..........................ccUguuuucauuuucgaucugacu.............................................................   1      1      OV1
..........................ccgguuuucauuuucgaucugacu.............................................................   1219   0      OV1
..........................ccgguuuucauuuucgaucugacuA............................................................   1      1      OV1
..........................ccgguuuucauuuucgaucugacuu............................................................   5      0      OV1
..........................ccgguuuucauuuucgaucugacCu............................................................   1      1      OV1
..........................ccgguuuucauuuucgaucugacuua...........................................................   4      0      OV1
..........................ccgguuuucauuuucgaucugacuuauu.........................................................   1      0      OV1
...........................cgguuuucauuuucgaucugacu.............................................................   24     0      OV1
...........................cgguuuucauuuucgaucugacuu............................................................   1      0      OV1
............................gguuuucauuuucgaucugac..............................................................   1      0      OV1
............................gguuuucauuuucgaucugacu.............................................................   53     0      OV1
............................gguuuucauuuucgaucugacuu............................................................   2      0      OV1
....................................................................gugagaucacuuugaaagcu.......................   1      0      OV1
....................................................................gugagaucacuuugaaagcug......................   7      0      OV1
....................................................................Cugagaucacuuugaaagcug......................   1      1      OV1
....................................................................Cugagaucacuuugaaagcuga.....................   1      1      OV1
....................................................................gugagaucacuuugaaagcuga.....................   10     0      OV1
....................................................................gugagaucacuuugaaagcugau....................   2      0      OV1
....................................................................Cugagaucacuuugaaagcugau....................   4      1      OV1
....................................................................gugagaucacuuugaaagcugaA....................   1      1      OV1
.....................................................................ugagaucacuuuAaaag.........................   1      1      OV1
.....................................................................ugagaucacuuugaaag.........................   89     0      OV1
.....................................................................ugagaCcacuuugaaagc........................   1      1      OV1
.....................................................................ugagaucacuuugaaagc........................   139    0      OV1
.....................................................................ugagaucacuuugaaagcu.......................   29     0      OV1
.....................................................................ugagaucacuuAgaaagcug......................   1      1      OV1
.....................................................................ugagaucacuuuAaaagcug......................   1      1      OV1
.....................................................................ugagaucacuuugaaagcGg......................   1      1      OV1
.....................................................................Cgagaucacuuugaaagcug......................   1      1      OV1
.....................................................................ugGgaucacuuugaaagcug......................   1      1      OV1
.....................................................................ugagaucacuCugaaagcug......................   1      1      OV1
.....................................................................ugagaucacuuugaaCgcug......................   1      1      OV1
.....................................................................ugagaucacuuugaGagcug......................   2      1      OV1
.....................................................................ugagauUacuuugaaagcug......................   1      1      OV1
.....................................................................ugagaucacuuugaaaAcug......................   1      1      OV1
.....................................................................Ggagaucacuuugaaagcug......................   1      1      OV1
.....................................................................ugagaucacCuugaaagcug......................   1      1      OV1
.....................................................................ugagaucaAuuugaaagcug......................   1      1      OV1
.....................................................................ugagaucCcuuugaaagcug......................   1      1      OV1
.....................................................................ugaAaucacuuugaaagcug......................   1      1      OV1
.....................................................................ugagaucacGuugaaagcug......................   1      1      OV1
.....................................................................ugagaucacuuugaaagcug......................   1163   0      OV1
.....................................................................Agagaucacuuugaaagcug......................   1      1      OV1
.....................................................................ugagaAcacuuugaaagcuga.....................   1      1      OV1
.....................................................................ugagaucacCuugaaagcuga.....................   1      1      OV1
.....................................................................uAagaucacuuugaaagcuga.....................   2      1      OV1
.....................................................................ugagaCcacuuugaaagcuga.....................   1      1      OV1
.....................................................................ugagaucacuuugaaagcugU.....................   2      1      OV1
.....................................................................ugagaucacuCugaaagcuga.....................   1      1      OV1
.....................................................................ugagaucacuuugaaagcuga.....................   1676   0      OV1
.....................................................................ugagauAacuuugaaagcuga.....................   1      1      OV1
.....................................................................ugagaucacuuugaaagcCga.....................   2      1      OV1
.....................................................................ugagaucacuuugaGagcuga.....................   1      1      OV1
.....................................................................ugagaucacuuugaaagcuAa.....................   1      1      OV1
.....................................................................ugagaucacuuGgaaagcuga.....................   1      1      OV1
.....................................................................ugagaucacuuugaaagcugG.....................   2      1      OV1
```

```
                          aga-bantam*
                                                                     aga-bantam
ugugugg augaaauguaaucacagaaccgguuuucauuuucgaucugacuuauuuuuuucacaaacaagugagaucacuuugaaagcugauuuuguacaauuaauucaacg

.....................................................................ugagauUacuuugaaagcuga.....................	1	1	OV1
.....................................................................ugagaucacuuuAaaagcuga.....................	1	1	OV1
.....................................................................ugagaucacAuugaaagcugau....................	1	1	OV1
.....................................................................ugagaucacCuugaaagcugau....................	2	1	OV1
.....................................................................ugagaucacuuugaaagcugaC....................	1	1	OV1
.....................................................................ugagaucacuuCgaaagcugau....................	3	1	OV1
.....................................................................ugagaucacuuuAaaagcugau....................	5	1	OV1
.....................................................................uAagaucacuuugaaagcugau....................	3	1	OV1
.....................................................................ugagaucaUuuugaaagcugau....................	2	1	OV1
.....................................................................ugagaucacuuugaaagcugau....................	3209	0	OV1
.....................................................................ugagaucacuuugaaagcuAau....................	2	1	OV1
.....................................................................ugagaucacuuugaaagcugaG....................	1	1	OV1
.....................................................................ugagaucacuuugaaagcugaA....................	13	1	OV1
.....................................................................ugagGucacuuugaaagcugau....................	1	1	OV1
.....................................................................ugagaucacuuugaaagcugCu....................	1	1	OV1
.....................................................................ugagaucacuuGgaaagcugau....................	1	1	OV1
.....................................................................ugagaucacuuugaaagUugau....................	1	1	OV1
.....................................................................ugagauUacuuugaaagcugau....................	1	1	OV1
.....................................................................Ggagaucacuuugaaagcugauu...................	1	1	OV1
.....................................................................uAagaucacuuugaaagcugauu...................	1	1	OV1
.....................................................................ugagaucacuuugaaagcugaCu...................	3	1	OV1
.....................................................................ugagaucacuuugaaagcugauA...................	20	1	OV1
.....................................................................ugagaucacuuugaGagcugauu...................	1	1	OV1
.....................................................................ugagaucacuuugaaagcugaGu...................	1	1	OV1
.....................................................................Agagaucacuuugaaagcugauu...................	1	1	OV1
.....................................................................ugagauUacuuugaaagcugauu...................	1	1	OV1
.....................................................................ugagaucacuuugaaagcugauu...................	879	0	OV1
.....................................................................ugagaucacuCugaaagcugauu...................	1	1	OV1
.....................................................................ugagaucaGuuugaaagcugauu...................	1	1	OV1
.....................................................................ugGgaucacuuugaaagcugauu...................	1	1	OV1
.....................................................................ugagaucaAuuugaaagcugauu...................	1	1	OV1
.....................................................................Cgagaucacuuugaaagcugauu...................	1	1	OV1
.....................................................................ugagaucacuuuAaaagcugauu...................	1	1	OV1
.....................................................................ugagaucacuuugaaagcugauuu..................	19	0	OV1
.....................................................................ugagaucacuuugaaagcugauuA..................	2	1	OV1
......................................................................gagaucacuuugaaagcug......................	1	0	OV1

........................Cccgguuuucauuuucgaucugacu..............................................................	1	1	FF1
.........................ccgguuuucauuuucgaucuga................................................................	2	0	FF1
.........................ccgguuuucauuuucgaucugac...............................................................	39	0	FF1
.........................ccgguuuuUauuuucgaucugacu..............................................................	1	1	FF1
.........................ccgguuuucauuuucgaucugacG..............................................................	1	1	FF1
.........................ccgguuuCcauuuucgaucugacu..............................................................	1	1	FF1
.........................ccgguuuucauuuucgaucugacu..............................................................	865	0	FF1
.........................ccgguuuucauuuucgaucugGcu..............................................................	1	1	FF1
.........................ccgguuuucauuuucgCucugacu..............................................................	1	1	FF1
.........................ccgguuuucauuuuUgaucugacu..............................................................	1	1	FF1
.........................ccgguuuucauuuucgaucugacA..............................................................	6	1	FF1
.........................ccgguuuucaCuuucgaucugacu..............................................................	1	1	FF1
.........................ccgguCuucauuuucgaucugacu..............................................................	1	1	FF1
.........................ccgguuuucauuuucCaucugacu..............................................................	1	1	FF1
.........................ccgguuuucauuuucgaucugacC..............................................................	1	1	FF1
.........................ccgguuuucauuuucgaucugacuu.............................................................	7	0	FF1
.........................ccgguuuucauuuucgaucugacuA.............................................................	2	1	FF1
.........................ccgguuuucauuuucgaucugacuC.............................................................	1	1	FF1
.........................ccgguuuucauuuucgaucugacuua............................................................	3	0	FF1
..........................cgguuuucauuGucgaucugacu..............................................................	1	1	FF1
..........................cgguuuucauuuucgaucugacu..............................................................	4	0	FF1
...........................gguuuucauuuucgaucugacu..............................................................	12	0	FF1
....................................................................gugagaucacuuugaaagcug......................	5	0	FF1
....................................................................Cugagaucacuuugaaagcug......................	1	1	FF1
....................................................................gugagaucacuuugaaagcuga.....................	1	0	FF1
....................................................................Cugagaucacuuugaaagcuga.....................	2	1	FF1
....................................................................gugagaucacuuugaaagcugau....................	1	0	FF1
....................................................................Cugagaucacuuugaaagcugau....................	1	1	FF1
....................................................................Cugagaucacuuugaaagcugauu...................	1	1	FF1
.....................................................................ugagaucacuuugaaag.........................	8	0	FF1
.....................................................................ugagaucacuuugaaagc........................	28	0	FF1
.....................................................................ugagaucacuuugaaagcu.......................	4	0	FF1
```

```
                          aga-bantam*
                                                                     aga-bantam
ugugugaugaaauguaaucacagaaccgguuuucauuuucgaucugacuuauuuuuuucacaaacaagugagaucacuuugaaagcugauuuuguacaauuaauucaacg

.....................................................................ugagaucacuuugaaagcGg......................   1     1   FF1
.....................................................................uAagaucacuuugaaagcug......................   1     1   FF1
.....................................................................ugagaucacuuAgaaagcug......................   1     1   FF1
.....................................................................ugagaucacuuugaaagcug......................   284   0   FF1
.....................................................................ugagaucacuuugaaagcCga.....................   1     1   FF1
.....................................................................uAagaucacuuugaaagcuga.....................   1     1   FF1
.....................................................................ugagaucacuuugaaagcuga.....................   418   0   FF1
.....................................................................ugagauUacuuugaaagcuga.....................   1     1   FF1
.....................................................................ugagaucacuuugaaagcugG.....................   3     1   FF1
.....................................................................ugagaucacuuugaaagcugaC....................   1     1   FF1
.....................................................................ugagaucacuuugaaagcugau....................   1201  0   FF1
.....................................................................ugagaucacuuugaaagcCgau....................   1     1   FF1
.....................................................................ugagaucacuuugaaagcuUau....................   1     1   FF1
.....................................................................ugagaucacuuugaaagcugaA....................   6     1   FF1
.....................................................................ugagaucacuuugaaagcAgau....................   1     1   FF1
.....................................................................ugagaucacuGugaaagcugau....................   1     1   FF1
.....................................................................ugagaucacuuuAaaagcugau....................   1     1   FF1
.....................................................................ugagaucacuuuAaaagcugauu...................   2     1   FF1
.....................................................................ugagaucacuuugaaagcugauA...................   7     1   FF1
.....................................................................ugagaucacuuugaaagcugauu...................   394   0   FF1
.....................................................................ugagaucacuuugaaagcugauuA..................   1     1   FF1
.....................................................................ugagUucacuuugaaagcugauuu..................   1     1   FF1
.....................................................................ugagaucacuuugaaagcugauuu..................   4     0   FF1
......................................................................gagaucacuuugaaagcugau....................   1     0   FF1

..........................ccgguuuucauuuucgauc..................................................................   1     0   MF1
..........................ccgguuuucauuuucgaucuga...............................................................   6     0   MF1
..........................ccgguuuucauuuucgaucugac..............................................................   27    0   MF1
..........................ccgguuuucauuuucgaucugacu.............................................................   608   0   MF1
..........................ccgguuuuUauuuucgaucugacu.............................................................   1     1   MF1
..........................ccgguCuucauuuucgaucugacu.............................................................   1     1   MF1
..........................ccgguuCucauuuucgaucugacu.............................................................   1     1   MF1
..........................ccgguuuucauuuucgaucuAacu.............................................................   1     1   MF1
..........................ccgguuuucauuuucgaucugGcu.............................................................   1     1   MF1
..........................ccgguuuucauuuucgaucugacA.............................................................   4     1   MF1
..........................ccgguuuucauuuucgaucugacG.............................................................   2     1   MF1
..........................ccgguuuucauGuucgaucugacu.............................................................   1     1   MF1
..........................ccgguuuucauuuucgaucugaUu.............................................................   1     1   MF1
..........................ccgguuuucauuCucgaucugacu.............................................................   1     1   MF1
..........................ccgguuuucauuuucgaucugacuu............................................................   2     0   MF1
..........................ccgguuuucauuuucgaucugacuA............................................................   2     1   MF1
...........................cgguuuucauuuucgaucugacu.............................................................   27    0   MF1
............................gguuuucauuuucgaucugacu.............................................................   18    0   MF1
............................gguuuucauuuucgaucugacuu............................................................   3     0   MF1
....................................................................gugagaucacuuugaaagcug......................   4     0   MF1
....................................................................gugagaucacuuugaaagcuga.....................   4     0   MF1
....................................................................Cugagaucacuuugaaagcugau....................   2     1   MF1
....................................................................gugagaucacuuugaaagcugau....................   2     0   MF1
....................................................................gugagaucacuuugaaagcugauA...................   1     1   MF1
....................................................................Cugagaucacuuugaaagcugauu...................   1     1   MF1
.....................................................................Cgagaucacuuugaaag.........................   1     1   MF1
.....................................................................ugagaucacuuugaaag.........................   94    0   MF1
.....................................................................ugagaucacuCugaaag.........................   1     1   MF1
.....................................................................ugagaucacuuugaaaA.........................   1     1   MF1
.....................................................................ugagaucacuuugaaagc........................   169   0   MF1
.....................................................................ugagaucacuuugaGagc........................   1     1   MF1
.....................................................................ugagaucacuuuAaaagc........................   1     1   MF1
.....................................................................ugagaucacuuugaaagcu.......................   55    0   MF1
.....................................................................uUagaucacuuugaaagcug......................   1     1   MF1
.....................................................................uAagaucacuuugaaagcug......................   1     1   MF1
.....................................................................ugagaucacuuugaaagcGg......................   1     1   MF1
.....................................................................ugGgaucacuuugaaagcug......................   1     1   MF1
.....................................................................ugagaCcacuuugaaagcug......................   1     1   MF1
.....................................................................ugagaucacuuugaaaCcug......................   1     1   MF1
.....................................................................ugagaucacCuugaaagcug......................   1     1   MF1
.....................................................................ugagaucacuuAgaaagcug......................   1     1   MF1
.....................................................................ugagaucacuuugaaagUug......................   1     1   MF1
.....................................................................ugagaucacuuuAaaagcug......................   1     1   MF1
.....................................................................Cgagaucacuuugaaagcug......................   1     1   MF1
```

```
                          aga-bantam*
                                                                     aga-bantam
ugugugaugaaauguaaucacagaaccgguuuucauuuucgaucugacuuauuuuuuucacaaacaagugagaucacuuugaaagcugauuuuguacaauuaauucaacg

.....................................................................ugagaucacuuCgaaagcug......................    1          1          MF1
.....................................................................ugagaucacuuugaaagcuA......................    1          1          MF1
.....................................................................ugagaucacuuugaGagcug......................    1          1          MF1
.....................................................................ugagaucacuuugaaagcCg......................    1          1          MF1
.....................................................................ugagaucacuuugaaagcug......................    1099       0          MF1
.....................................................................ugagaucacuuuAaaagcuga.....................    2          1          MF1
.....................................................................ugagaucacuuAgaaagcuga.....................    1          1          MF1
.....................................................................ugagaucacuuGgaaagcuga.....................    1          1          MF1
.....................................................................ugagaucacuuugaaagcuga.....................    1804       0          MF1
.....................................................................ugagaucacuuugaaagcugG.....................    9          1          MF1
.....................................................................ugagaucacuuCgaaagcuga.....................    3          1          MF1
.....................................................................ugagaucacuuugaaagcCga.....................    1          1          MF1
.....................................................................ugagaucacuuugaaaCcuga.....................    1          1          MF1
.....................................................................ugagaucacuuugaaagcuAa.....................    1          1          MF1
.....................................................................uAagaucacuuugaaagcuga.....................    1          1          MF1
.....................................................................Cgagaucacuuugaaagcuga.....................    2          1          MF1
.....................................................................ugagaucacuuugaaaAcuga.....................    2          1          MF1
.....................................................................ugagauUacuuugaaagcugau....................    3          1          MF1
.....................................................................ugagaucacuuugaaaAcugau....................    2          1          MF1
.....................................................................uCagaucacuuugaaagcugau....................    1          1          MF1
.....................................................................uUagaucacuuugaaagcugau....................    1          1          MF1
.....................................................................ugagaucacuuugaaagcugaG....................    5          1          MF1
.....................................................................Cgagaucacuuugaaagcugau....................    2          1          MF1
.....................................................................uAagaucacuuugaaagcugau....................    6          1          MF1
.....................................................................ugagaucacuCugaaagcugau....................    2          1          MF1
.....................................................................ugagaucUcuuugaaagcugau....................    1          1          MF1
.....................................................................ugagaucacuuugaaagcuAau....................    3          1          MF1
.....................................................................ugagaucacuuAgaaagcugau....................    2          1          MF1
.....................................................................ugagaucacuuuAaaagcugau....................    3          1          MF1
.....................................................................ugagaucacuuugaaagcugaC....................    3          1          MF1
.....................................................................ugagaucaAuuugaaagcugau....................    1          1          MF1
.....................................................................ugagaucaUuuugaaagcugau....................    1          1          MF1
.....................................................................ugagaucacuuugaaagcCgau....................    1          1          MF1
.....................................................................ugagaucacuuugaaagAugau....................    2          1          MF1
.....................................................................ugagauGacuuugaaagcugau....................    1          1          MF1
.....................................................................ugagaucacuuGgaaagcugau....................    1          1          MF1
.....................................................................ugagaucacCuugaaagcugau....................    5          1          MF1
.....................................................................ugagaucacuuugaaaCcugau....................    5          1          MF1
.....................................................................ugGgaucacuuugaaagcugau....................    2          1          MF1
.....................................................................ugagaucacuuugaaaUcugau....................    5          1          MF1
.....................................................................ugagGucacuuugaaagcugau....................    2          1          MF1
.....................................................................ugagaucacuuugaaGgcugau....................    1          1          MF1
.....................................................................ugagaucacuuugaaagcugau....................    5124       0          MF1
.....................................................................ugagaucacuuuUaaagcugau....................    1          1          MF1
.....................................................................ugagaucacAuugaaagcugau....................    2          1          MF1
.....................................................................ugagaucacuuugaaagGugau....................    1          1          MF1
.....................................................................ugagUucacuuugaaagcugau....................    2          1          MF1
.....................................................................ugagaucacuGugaaagcugau....................    1          1          MF1
.....................................................................ugagaucacuuugaaagcugaA....................    16         1          MF1
.....................................................................ugagaucacuuCgaaagcugau....................    6          1          MF1
.....................................................................ugagaucacuuugaGagcugauu...................    2          1          MF1
.....................................................................ugagaucacuCugaaagcugauu...................    1          1          MF1
.....................................................................ugagaucacuuCgaaagcugauu...................    1          1          MF1
.....................................................................ugagaucacuuugaaagcugauu...................    1560       0          MF1
.....................................................................ugagauUacuuugaaagcugauu...................    2          1          MF1
.....................................................................ugagaucacuuugaaagcugaCu...................    3          1          MF1
.....................................................................uAagaucacuuugaaagcugauu...................    1          1          MF1
.....................................................................ugagaucacuuugaaagcugauA...................    19         1          MF1
.....................................................................ugagaCcacuuugaaagcugauu...................    1          1          MF1
.....................................................................ugagaucacuuugaaagcugaAu...................    1          1          MF1
.....................................................................Ggagaucacuuugaaagcugauu...................    2          1          MF1
.....................................................................ugagCucacuuugaaagcugauu...................    1          1          MF1
.....................................................................ugagaucacuuuAaaagcugauu...................    1          1          MF1
.....................................................................ugagaucacuuugaaagcugCuu...................    1          1          MF1
.....................................................................ugagaucacuuugaaagcugaGu...................    1          1          MF1
.....................................................................ugagaucacuuugaaagcCgauu...................    1          1          MF1
.....................................................................ugagaucacuuugaaGgcugauu...................    1          1          MF1
.....................................................................ugagaucacuuugaaagcugauuA..................    8          1          MF1
.....................................................................ugagaucacuuugaaagcugauuC..................    1          1          MF1
```

```
                          aga-bantam*
                                                                     aga-bantam
ugugugGaugaaauguaaucacagaaccgguuuucauuuucgaucugacuuauuuuuuucacaaacaagugagaucacuuugaaagcugauuuuguacaauuaauucaacg

.....................................................................ugagaucacuuugaaagcugauuu..................   60     0   MF1

.........................Cccgguuuucauuuucgaucugacu.............................................................   3      1   BF2
..........................ccgguuuucauuuucgaucuga...............................................................   10     0   BF2
..........................ccgguuuucauuuucgaucugac..............................................................   94     0   BF2
..........................ccgguuuucauuuucgaucugaA..............................................................   1      1   BF2
..........................ccgguuuucauuuucgaCcugacu.............................................................   1      1   BF2
..........................ccgguuuucauuCucgaucugacu.............................................................   2      1   BF2
..........................ccgguuuucauuuucgaucugacu.............................................................   1992   0   BF2
..........................ccgguuuucauuuucgaucuUacu.............................................................   1      1   BF2
..........................ccgguuuucauAuucgaucugacu.............................................................   1      1   BF2
..........................ccgAuuuucauuuucgaucugacu.............................................................   2      1   BF2
..........................ccUguuuucauuuucgaucugacu.............................................................   1      1   BF2
..........................ccgguuuucauCuucgaucugacu.............................................................   1      1   BF2
..........................ccgguuuuCUuuucgaucugacu..............................................................   1      1   BF2
..........................ccgguuuucauuuucgaucugacA.............................................................   15     1   BF2
..........................ccgguuuucauuGcgaucugacu..............................................................   1      1   BF2
..........................ccgguuCucauuuucgaucugacu.............................................................   1      1   BF2
..........................ccgguuuucauuuuUgaucugacu.............................................................   1      1   BF2
..........................ccgguuuucauuuucgaucuAacu.............................................................   1      1   BF2
..........................ccgguuuucauuuucgaucugacC.............................................................   3      1   BF2
..........................ccgguuuucauuuucAaucugacu.............................................................   1      1   BF2
..........................ccgguuuucauuCcgaucugacu..............................................................   2      1   BF2
..........................ccgguuuucauuAucgaucugacu.............................................................   1      1   BF2
..........................ccgguuuucaCuuucgaucugacu.............................................................   1      1   BF2
..........................ccgguuuUauuuucgaucugacu..............................................................   2      1   BF2
..........................ccgguuuucauuuucgaucCgacu.............................................................   1      1   BF2
..........................ccgguuuucauuuucgaucugacuA............................................................   2      1   BF2
..........................ccgguuuucauuuucgaucugacuu............................................................   3      0   BF2
..........................ccgguuuucauuuucgaucugacuua...........................................................   2      0   BF2
..........................ccgguuuucauuuucgaucugacuuauu.........................................................   1      0   BF2
...........................cgguuuucauuuucgaucugacG.............................................................   1      1   BF2
...........................cgguuuucauuuucgauUgacu..............................................................   1      1   BF2
...........................cgguuuucauuuucgaucugacu.............................................................   33     0   BF2
............................gguuuucauuuucgaucugacu.............................................................   53     0   BF2
............................gguuuucauuuucgaucugacuu............................................................   12     0   BF2
............................gguuuucauuuucgaucugacCu............................................................   1      1   BF2
..............................uuuucauuuucgaucugacu.............................................................   1      0   BF2
...............................uuucauuuucgaucugacu.............................................................   1      0   BF2
....................................................................gugagaucacuuugaaagcug......................   1      0   BF2
....................................................................gugagaucacuuugaaagcuga.....................   3      0   BF2
....................................................................Cugagaucacuuugaaagcugau....................   3      1   BF2
....................................................................gugagaucacuuugaaagcugau....................   10     0   BF2
.....................................................................ugagaucacuuugaaag.........................   35     0   BF2
.....................................................................ugagaucacuuugaaagA........................   1      1   BF2
.....................................................................ugagaucacuuugaaagc........................   52     0   BF2
.....................................................................ugagaucacuuugaaagcu.......................   21     0   BF2
.....................................................................ugagaCcacuuugaaagcug......................   1      1   BF2
.....................................................................ugagaucacuuugaaagcug......................   697    0   BF2
.....................................................................ugagaucacuuugaaagcuA......................   1      1   BF2
.....................................................................ugagaucacuuCgaaagcug......................   1      1   BF2
.....................................................................ugaCaucacuuugaaagcug......................   1      1   BF2
.....................................................................ugagaucacuuugaaaCcug......................   1      1   BF2
.....................................................................ugagaucacuuugaaagcugU.....................   1      1   BF2
.....................................................................ugagaucacuuugaaaAcuga.....................   1      1   BF2
.....................................................................ugagauAacuuugaaagcuga.....................   1      1   BF2
.....................................................................ugagaucacuuugaGagcuga.....................   1      1   BF2
.....................................................................ugagaucacuuugaaagcuAa.....................   1      1   BF2
.....................................................................ugagaucacuuugaaaUcuga.....................   2      1   BF2
.....................................................................ugagaucacuuugaaagcugG.....................   8      1   BF2
.....................................................................Cgagaucacuuugaaagcuga.....................   1      1   BF2
.....................................................................uAagaucacuuugaaagcuga.....................   1      1   BF2
.....................................................................ugagaucGcuuugaaagcuga.....................   1      1   BF2
.....................................................................ugagaucacuuugaaagUuga.....................   1      1   BF2
.....................................................................Agagaucacuuugaaagcuga.....................   1      1   BF2
.....................................................................ugagaucacuuugaaagcuga.....................   1233   0   BF2
.....................................................................ugaAaucacuuugaaagcuga.....................   1      1   BF2
.....................................................................ugagaucacuuugaaagcCga.....................   1      1   BF2
.....................................................................ugagaucacuuugaaGgcugau....................   1      1   BF2
```

aga-bantam*

aga-bantam

uguguggaugaaauguaaucacagaaccgguuuucauuuucgaucugacuuauuuuuuucacaaacaagugagaucacuuugaaagcugauuuuguacaauuaauucaacg

....ugGgaucacuuugaaagcugau....................	1	1	BF2
....ugagaucacuuugaaaUcugau....................	1	1	BF2
....ugagaucacuAugaaagcugau....................	1	1	BF2
....ugagaucGcuuugaaagcugau....................	1	1	BF2
....ugagaucacuuugaaagcugaC....................	2	1	BF2
....ugagaucacuuugaaagcugaA....................	6	1	BF2
....Cgagaucacuuugaaagcugau....................	3	1	BF2
....ugagaucacuuugGaagcugau....................	1	1	BF2
....uAagaucacuuugaaagcugau....................	3	1	BF2
....ugagaucacGuugaaagcugau....................	1	1	BF2
....ugagaucacuuCgaaagcugau....................	1	1	BF2
....ugagaucacuuuAaaagcugau....................	2	1	BF2
....ugagauUacuuugaaagcugau....................	2	1	BF2
....ugagaucacuuugaaagcugau....................	4444	0	BF2
....ugagaucacuuugaaagcuAau....................	2	1	BF2
....ugagauAacuuugaaagcugau....................	1	1	BF2
....ugagaucacuuugaGagcugau....................	1	1	BF2
....ugagaucacCuugaaagcugau....................	2	1	BF2
....ugagGucacuuugaaagcugau....................	1	1	BF2
....ugUgaucacuuugaaagcugau....................	1	1	BF2
....ugagaucacuuugaaaAcugau....................	2	1	BF2
....ugaCaucacuuugaaagcugau....................	1	1	BF2
....ugagaucacuuAgaaagcugau....................	2	1	BF2
....ugagaucaUuuugaaagcugau....................	1	1	BF2
....ugagaucacuuugaaagcugaCu...................	1	1	BF2
....ugagaucacuuugaaagcugauu...................	1263	0	BF2
....ugagaucacuuugaaagcugauC...................	1	1	BF2
....ugagaucacuuAgaaagcugauu...................	1	1	BF2
....ugagaucacuuugaaagcugaAu...................	1	1	BF2
....ugUgaucacuuugaaagcugauu...................	1	1	BF2
....ugagaucacuuuAaaagcugauu...................	1	1	BF2
....ugagaucacuuugaaagcugauA...................	44	1	BF2
....ugagaucacuuugaaagcuAauu...................	2	1	BF2
....ugaCaucacuuugaaagcugauu...................	1	1	BF2
....ugagaucacCuugaaagcugauuu..................	1	1	BF2
....ugagaucacuCugaaagcugauuu..................	2	1	BF2
....ugagaucacuuugaaagcugauuu..................	21	0	BF2
....ugagaucacuuugaaagcugauuA..................	3	1	BF2
....ugagaucacuuugaaagcugauuG..................	1	1	BF2
....ugagaucacuuugaaagcugauuC..................	1	1	BF2
....gagaucacuuugaaagcugau....................	1	0	BF2
....gagaucacuuugaaagcugauu...................	1	0	BF2
....cacuuugaaagcugauu...................	1	0	BF2
.........................Cccgguuuucauuuucgaucugacu...	1	1	BF1
..........................ccgguuuucauuuucgaucuga...	2	0	BF1
..........................ccgguuuucauuuucgaucugac..	39	0	BF1
..........................ccgguuuucauuuucgaucugacG...	1	1	BF1
..........................ccgguuuucauuuucgaucugGcu...	1	1	BF1
..........................ccgguuuucaCuuucgaucugacu...	1	1	BF1
..........................ccgguuuucauuuucgCucugacu...	1	1	BF1
..........................ccgguCuucauuuucgaucugacu...	1	1	BF1
..........................ccgguuuucauuuuUgaucugacu...	1	1	BF1
..........................ccgguuuucauuuucgaucugacC...	1	1	BF1
..........................ccgguuuucauuuucgaucugacu...	860	0	BF1
..........................ccgguuuucauuuucgaucugacA...	6	1	BF1
..........................ccgguuuuUauuuucgaucugacu...	1	1	BF1
..........................ccgguuuCcauuuucgaucugacu...	1	1	BF1
..........................ccgguuuucauuuucCaucugacu...	1	1	BF1
..........................ccgguuuucauuuucgaucugacuC..	1	1	BF1
..........................ccgguuuucauuuucgaucugacuA..	2	1	BF1
..........................ccgguuuucauuuucgaucugacuu..	7	0	BF1
..........................ccgguuuucauuuucgaucugacuua...	3	0	BF1
...........................cgguuuucauuuucgaucugacu...	4	0	BF1
...........................cgguuuucauuGucgaucugacu...	1	1	BF1
............................gguuuucauuuucgaucugacu...	12	0	BF1
....gugagaucacuuugaaagcug......................	5	0	BF1
....Cugagaucacuuugaaagcug......................	1	1	BF1
....gugagaucacuuugaaagcuga.....................	1	0	BF1

```
                          aga-bantam*
                                                                     aga-bantam
ugugugguaugaaauguaaucacagaaccgguuuucauuuucgaucugacuuauuuuuuucacaaacaagugagaucacuuugaaagcugauuuuguacaauuaauucaacg

....................................................................Cugagaucacuuugaaagcuga.....................   2      1   BF1
....................................................................Cugagaucacuuugaaagcugau....................   1      1   BF1
....................................................................gugagaucacuuugaaagcugau....................   1      0   BF1
....................................................................Cugagaucacuuugaaagcugauu...................   1      1   BF1
.....................................................................ugagaucacuuugaaag.........................   8      0   BF1
.....................................................................ugagaucacuuugaaagc........................   28     0   BF1
.....................................................................ugagaucacuuugaaagcu.......................   4      0   BF1
.....................................................................ugagaucacuuAgaaagcug......................   1      1   BF1
.....................................................................ugagaucacuuugaaagcGg......................   1      1   BF1
.....................................................................uAagaucacuuugaaagcug......................   1      1   BF1
.....................................................................ugagaucacuuugaaagcug......................   282    0   BF1
.....................................................................ugagauUacuuugaaagcuga.....................   1      1   BF1
.....................................................................ugagaucacuuugaaagcugG.....................   3      1   BF1
.....................................................................ugagaucacuuugaaagcCga.....................   1      1   BF1
.....................................................................ugagaucacuuugaaagcuga.....................   418    0   BF1
.....................................................................uAagaucacuuugaaagcuga.....................   1      1   BF1
.....................................................................ugagaucacuuugaaagcCgau....................   1      1   BF1
.....................................................................ugagaucacuuugaaagcAgau....................   1      1   BF1
.....................................................................ugagaucacuuuAaaagcugau....................   1      1   BF1
.....................................................................ugagaucacuuugaaagcugaA....................   6      1   BF1
.....................................................................ugagaucacuuugaaagcugaC....................   1      1   BF1
.....................................................................ugagaucacuuugaaagcugau....................   1201   0   BF1
.....................................................................ugagaucacuGugaaagcugau....................   1      1   BF1
.....................................................................ugagaucacuuuAaaagcugauu...................   2      1   BF1
.....................................................................ugagaucacuuugaaagcugauu...................   393    0   BF1
.....................................................................ugagaucacuuugaaagcugauA...................   7      1   BF1
.....................................................................ugagaucacuuugaaagcugauuu..................   4      0   BF1
.....................................................................ugagaucacuuugaaagcugauuA..................   1      1   BF1
.....................................................................ugagUucacuuugaaagcugauuu..................   1      1   BF1
......................................................................gagaucacuuugaaagcugau....................   1      0   BF1

..........................ccgguuuucauuuucgauc..................................................................   1      0   MW1
..........................ccgguuuucauuuucgaucug................................................................   1      0   MW1
..........................ccgguuuucauuuucgaucuga...............................................................   4      0   MW1
..........................ccgguuuucauuuucgaucugac..............................................................   32     0   MW1
..........................ccUguuuucauuuucgaucugacu.............................................................   1      1   MW1
..........................ccgguuuucauuuucgaucugGcu.............................................................   1      1   MW1
..........................ccgguuuucauuuucgaucugacu.............................................................   947    0   MW1
..........................Gcgguuuucauuuucgaucugacu.............................................................   1      1   MW1
..........................cAgguuuucauuuucgaucugacu.............................................................   1      1   MW1
..........................ccgguuuucauuuucgaucugaGu.............................................................   1      1   MW1
..........................ccgguuuuUauuuucgaucugacu.............................................................   1      1   MW1
..........................ccgguuuucauuuucgauUugacu.............................................................   1      1   MW1
..........................ccgguuuucauuuucgaucugacuu............................................................   2      0   MW1
..........................ccgguuuucauuuucgaucugacuuauuuuuuucacaaaca............................................   1      0   MW1
...........................cgguuuucauuuucgaucugacC.............................................................   1      1   MW1
...........................cgguuuucauuuucgaucugacu.............................................................   157    0   MW1
............................gguuuucauuuucgaucugacu.............................................................   27     0   MW1
............................gguuuucauuuucgaucugacuu............................................................   9      0   MW1
............................gguuuucauuuucgaucugacuuU...........................................................   1      1   MW1
..............................uuuucauuuucgaucugacu.............................................................   2      0   MW1
...............................uuucauuuucgaucugacu.............................................................   1      0   MW1
...............................uuucauuuucgaucugacuu............................................................   1      0   MW1
.................................................................caagugagaucacuuugaaagcuga.....................   1      0   MW1
....................................................................gugagaucacuuugaaagcu.......................   1      0   MW1
....................................................................gugagaucacuuugaaagcug......................   2      0   MW1
....................................................................gugagaucacuuugaaagcuga.....................   2      0   MW1
....................................................................Cugagaucacuuugaaagcuga.....................   1      1   MW1
....................................................................Cugagaucacuuugaaagcugau....................   4      1   MW1
....................................................................gugagaucacuuugaaagcugau....................   6      0   MW1
....................................................................Cugagaucacuuugaaagcugauu...................   1      1   MW1
....................................................................gugagaucacuuugaaagcugauuu..................   1      0   MW1
.....................................................................ugagaucacuuugaaag.........................   60     0   MW1
.....................................................................ugagaucacuuugaaagc........................   101    0   MW1
.....................................................................ugagaucacuuugaaagcu.......................   81     0   MW1
.....................................................................ugagaucacuuugGaagcug......................   1      1   MW1
.....................................................................ugagaucacuuugaUagcug......................   1      1   MW1
.....................................................................ugagaucacuuugaaagcug......................   445    0   MW1
.....................................................................ugagaucaUuuugaaagcug......................   1      1   MW1
```

```
                          aga-bantam*
                                                                     aga-bantam
uguguggaugaaauguaaucacagaaccgguuuucauuuucgaucugacuuauuuuuuucacaaacaagugagaucacuuugaaagcugauuuuguacaauuaauucaacg

.....................................................................ugagaucacuuugaaagcugG.....................    4      1     MW1
.....................................................................uAagaucacuuugaaagcuga.....................    1      1     MW1
.....................................................................ugagaucacCuugaaagcuga.....................    1      1     MW1
.....................................................................ugagaucacuuugaaagcuga.....................    1211   0     MW1
.....................................................................ugagaucacuuCgaaagcuga.....................    1      1     MW1
.....................................................................ugagaucacuuugaaagcCga.....................    2      1     MW1
.....................................................................ugagaucacuuuAaaagcuga.....................    1      1     MW1
.....................................................................ugagaucacuuAgaaagcuga.....................    1      1     MW1
.....................................................................Cgagaucacuuugaaagcuga.....................    3      1     MW1
.....................................................................ugagaucacuuugaCagcuga.....................    1      1     MW1
.....................................................................ugagaucacuuAgaaagcugau....................    1      1     MW1
.....................................................................ugagaucacuuCgaaagcugau....................    2      1     MW1
.....................................................................ugagaucacuuugaaagcugaC....................    7      1     MW1
.....................................................................ugagaucacuAugaaagcugau....................    1      1     MW1
.....................................................................ugagGucacuuugaaagcugau....................    1      1     MW1
.....................................................................uAagaucacuuugaaagcugau....................    4      1     MW1
.....................................................................ugagaucacCuugaaagcugau....................    3      1     MW1
.....................................................................ugagaucacuuugaCagcugau....................    1      1     MW1
.....................................................................ugagaucacGuugaaagcugau....................    1      1     MW1
.....................................................................ugagaucacuuugaaagcuUau....................    2      1     MW1
.....................................................................ugagaCcacuuugaaagcugau....................    3      1     MW1
.....................................................................Cgagaucacuuugaaagcugau....................    4      1     MW1
.....................................................................ugagaucacuuugaaagcCgau....................    3      1     MW1
.....................................................................ugagaAcacuuugaaagcugau....................    1      1     MW1
.....................................................................ugagaucGcuuugaaagcugau....................    1      1     MW1
.....................................................................ugaCaucacuuugaaagcugau....................    1      1     MW1
.....................................................................uUagaucacuuugaaagcugau....................    1      1     MW1
.....................................................................ugagaucacuuGgaaagcugau....................    3      1     MW1
.....................................................................ugagUucacuuugaaagcugau....................    1      1     MW1
.....................................................................ugagaucacuuugaaGgcugau....................    1      1     MW1
.....................................................................ugagauUacuuugaaagcugau....................    3      1     MW1
.....................................................................ugaAaucacuuugaaagcugau....................    2      1     MW1
.....................................................................ugagaucacuuugaaagcugaA....................    6      1     MW1
.....................................................................ugagaucacuuugaaagcuCau....................    1      1     MW1
.....................................................................ugagaucacuuuAaaagcugau....................    3      1     MW1
.....................................................................ugagaucacuuugaaagAugau....................    1      1     MW1
.....................................................................ugagaucacuCugaaagcugau....................    1      1     MW1
.....................................................................ugagaucacuuugaGagcugau....................    1      1     MW1
.....................................................................ugagaucacuuugaaagcugGu....................    3      1     MW1
.....................................................................ugagaucacuuugaaagcugaG....................    1      1     MW1
.....................................................................ugagaucacuuugaaaCcugau....................    1      1     MW1
.....................................................................ugagaucacuuugaaaAcugau....................    2      1     MW1
.....................................................................ugagaucacuuugaaagcugau....................    9776   0     MW1
.....................................................................ugagaucacuuugaaaUcugau....................    2      1     MW1
.....................................................................Agagaucacuuugaaagcugau....................    1      1     MW1
.....................................................................Ggagaucacuuugaaagcugau....................    2      1     MW1
.....................................................................ugagaucacAuugaaagcugau....................    1      1     MW1
.....................................................................ugagaucacuuugaaagcuAau....................    5      1     MW1
.....................................................................ugagaucacuuugaaagGugau....................    1      1     MW1
.....................................................................ugagaucaAuugaaagcugau.....................    1      1     MW1
.....................................................................ugagaCcacuuugaaagcugauu...................    2      1     MW1
.....................................................................uAagaucacuuugaaagcugauu...................    6      1     MW1
.....................................................................ugagaucacuuugaaagUugauu...................    1      1     MW1
.....................................................................ugagaucUcuuugaaagcugauu...................    1      1     MW1
.....................................................................ugagaucacuuugaaagcugaCu...................    2      1     MW1
.....................................................................Ggagaucacuuugaaagcugauu...................    1      1     MW1
.....................................................................ugagaucacuCugaaagcugauu...................    1      1     MW1
.....................................................................ugagaucacuuugaaagcugauu...................    3758   0     MW1
.....................................................................ugaAaucacuuugaaagcugauu...................    1      1     MW1
.....................................................................ugagaucacuuugaaagcugauC...................    1      1     MW1
.....................................................................ugagaucacAuugaaagcugauu...................    1      1     MW1
.....................................................................ugagaucacuuuGGaagcugauu...................    1      1     MW1
.....................................................................ugagaucacuuugaaagcugauG...................    1      1     MW1
.....................................................................ugagaucacCuugaaagcugauu...................    3      1     MW1
.....................................................................ugagUucacuuugaaagcugauu...................    1      1     MW1
.....................................................................ugagaucacuuugaaagcugauA...................    27     1     MW1
.....................................................................Agagaucacuuugaaagcugauu...................    1      1     MW1
.....................................................................ugagaucacuuugaaagcugaAu...................    1      1     MW1
.....................................................................ugagGucacuuugaaagcugauu...................    1      1     MW1
```

```
                          aga-bantam*
                                                                     aga-bantam
uguguggaugaaauguaaucacagaaccgguuuucauuuucgaucugacuuauuuuuuucacaaacaagugagaucacuuugaaagcugauuuuguacaauuaauucaacg

.....................................................................ugagaucacuuugaaagcuAauu...................  8     1  MW1
.....................................................................ugagauUacuuugaaagcugauu...................  1     1  MW1
.....................................................................ugagaucacuuuAaaagcugauu...................  3     1  MW1
.....................................................................ugagaucacuuCgaaagcugauu...................  2     1  MW1
.....................................................................ugagaucacuuugaaagcCgauu...................  1     1  MW1
.....................................................................ugagaucacuuugaaagcugauuA..................  13    1  MW1
.....................................................................ugagaucacuuugaaagcugauuu..................  101   0  MW1
.....................................................................ugagaucacuuugaaagcugauuC..................  2     1  MW1
......................................................................gagaucacuuugaaagcuga.....................  1     0  MW1
......................................................................gagaucacuuugaaagcugau....................  1     0  MW1
......................................................................Uagaucacuuugaaagcugau....................  1     1  MW1
......................................................................gagaucacuuugaaagcugauu...................  1     0  MW1
...........................................................................cacuuugaaagcugauu...................  4     0  MW1

.........................Cccgguuuucauuuucgaucugacu.............................................................  1     1  FW1
..........................ccgguuuucauuuucgaucuga...............................................................  1     0  FW1
..........................ccgguuuucauuuucgaucugac..............................................................  10    0  FW1
..........................ccggCuuucauuuucgaucugacu.............................................................  1     1  FW1
..........................Ucgguuuucauuuucgaucugacu.............................................................  1     1  FW1
..........................ccgguuuucauuuucgaCcugacu.............................................................  1     1  FW1
..........................ccgguuuuAauuuucgaucugacu.............................................................  1     1  FW1
..........................ccgguuuucauuuucgaucuAacu.............................................................  1     1  FW1
..........................ccgguuuucauuuucgaucugacu.............................................................  478   0  FW1
..........................ccgguuuucauuuucgaucugacA.............................................................  1     1  FW1
..........................ccgguuuucauuuCcgaucugacu.............................................................  1     1  FW1
..........................ccgguuuucauuuucgaucugacuA............................................................  1     1  FW1
..........................ccgguuuucauuuucgaucugacuu............................................................  3     0  FW1
...........................cgguuuucauuuucgaucugacu.............................................................  69    0  FW1
...........................cgguuuucauuuucgaCcugacu.............................................................  1     1  FW1
...........................cgguuuucauuuucgaucugacuu............................................................  1     0  FW1
............................gguuuucauuuucgaucugacu.............................................................  10    0  FW1
............................gguuuucauuuucgaucugacuu............................................................  2     0  FW1
.............................guuuucauuuucgaucugacu.............................................................  1     0  FW1
....................................................................Cugagaucacuuugaaa..........................  1     1  FW1
....................................................................gugagaucacuuugaaagc........................  1     0  FW1
....................................................................Cugagaucacuuugaaagcugau....................  5     1  FW1
....................................................................gugagaucacuuugaaagcugau....................  8     0  FW1
.....................................................................ugagaucacuuugaaag..........................  27    0  FW1
.....................................................................ugagaucacuuugaaagc.........................  41    0  FW1
.....................................................................ugagaucaUuuugaaagcu........................  1     1  FW1
.....................................................................ugagaucacuuugaaagcu........................  43    0  FW1
.....................................................................ugagaucacuuugaaagcuA.......................  1     1  FW1
.....................................................................ugaAaucacuuugaaagcug.......................  1     1  FW1
.....................................................................ugagaucacuuugaaagcug.......................  164   0  FW1
.....................................................................ugagaucacuuugaaagcugG......................  1     1  FW1
.....................................................................ugagaucacuAugaaagcuga......................  1     1  FW1
.....................................................................uAagaucacuuugaaagcuga......................  1     1  FW1
.....................................................................ugagaucacuuugaaagcuga......................  480   0  FW1
.....................................................................ugagaucGcuuugaaagcugau.....................  1     1  FW1
.....................................................................uAagaucacuuugaaagcugau.....................  1     1  FW1
.....................................................................ugagaucacuuugaaagcugGu.....................  1     1  FW1
.....................................................................Agagaucacuuugaaagcugau.....................  1     1  FW1
.....................................................................ugagaucacCuugaaagcugau.....................  1     1  FW1
.....................................................................Cgagaucacuuugaaagcugau.....................  1     1  FW1
.....................................................................ugagauUacuuugaaagcugau.....................  4     1  FW1
.....................................................................ugagaucacuuugaaagcugaC.....................  1     1  FW1
.....................................................................ugagaucacuuGgaaagcugau.....................  1     1  FW1
.....................................................................ugagaCcacuuugaaagcugau.....................  1     1  FW1
.....................................................................ugagaucacuuugaaagcuAau.....................  1     1  FW1
.....................................................................ugagaucaUuuugaaagcugau.....................  2     1  FW1
.....................................................................ugagaucacuuuAaaagcugau.....................  3     1  FW1
.....................................................................ugaCaucacuuugaaagcugau.....................  1     1  FW1
.....................................................................ugagaucacuuugaaagcugaA.....................  4     1  FW1
.....................................................................ugagaucacuuugaaagcugau.....................  4189  0  FW1
.....................................................................ugagaucacuuugaaaAcugau.....................  1     1  FW1
.....................................................................ugaAaucacuuugaaagcugau.....................  1     1  FW1
.....................................................................ugagaucacuuCgaaagcugau.....................  2     1  FW1
.....................................................................ugagaucacuuuCaaagcugau.....................  2     1  FW1
.....................................................................ugagaucacuGugaaagcugau.....................  1     1  FW1
```

```
                          aga-bantam*
                                                                     aga-bantam
ugugugggaugaaauguaaucacagaaccgguuuucauuuucgaucugacuuauuuuuuucacaaacaagugagaucacuuugaaagcugauuuuguacaauuaauucaacg
```

....ugagaucacuuugaaagcCgau....................	2	1	FW1
....ugagaucacuuugaaagUugau....................	1	1	FW1
....uAagaucacuuugaaagcugauu...................	3	1	FW1
....ugagaucacuuugaaaAcugauu...................	2	1	FW1
....ugagaucacuuugaaagcugauu...................	1588	0	FW1
....ugGgaucacuuugaaagcugauu...................	1	1	FW1
....ugagaucacuuugaaagAugauu...................	1	1	FW1
....ugagaucacuAugaaagcugauu...................	2	1	FW1
....ugagaucacuuugaaagcugaCu...................	1	1	FW1
....ugagaucacuuugaaagcugauG...................	1	1	FW1
....ugagaucacuuugaaagcuCauu...................	1	1	FW1
....ugagaucacuuugaaagcuAauu...................	2	1	FW1
....ugagauAacuuugaaagcugauu...................	1	1	FW1
....ugagaucacuuuCaaagcugauu...................	1	1	FW1
....ugagaucacuuugaaagcCgauu...................	1	1	FW1
....ugagaucacuuugaaGgcugauu...................	2	1	FW1
....ugagaucacuuugaaagcugauA...................	11	1	FW1
....ugagaucacuuCgaaagcugauu...................	3	1	FW1
....ugagaucaUuuugaaagcugauu...................	1	1	FW1
....ugagGucacuuugaaagcugauu...................	1	1	FW1
....ugagaucacuuugaaagcugauC...................	3	1	FW1
....ugagaucacuuuAaaagcugauu...................	1	1	FW1
....ugagaucacuuugaaagcugauuA..................	5	1	FW1
....ugagaucacuuugaaagcugauuu..................	38	0	FW1
....Cgagaucacuuugaaagcugauuu..................	1	1	FW1
....ugagaucacuuugaaagcugaAuu..................	1	1	FW1
....gagaucacuuugaaagcugau....................	1	0	FW1
....cacuuugaaagcugauu...................	2	0	FW1
..........................ccgguuuucauuuucgaucuga...	1	0	MW2
..........................ccgguuuucauuuucgaucugac..	9	0	MW2
..........................ccgguuuucauuuucgaucugacu...	315	0	MW2
..........................ccgguuuucauuuuUgaucugacu...	1	1	MW2
..........................ccgguuuucauuuucgaucugacA...	3	1	MW2
...........................cgguuuucauuuucgaCcugacu...	1	1	MW2
...........................cgguuuucauuuucgaucugacu...	43	0	MW2
............................gguuuucauuuucgaucugac..	1	0	MW2
............................gguuuucauuuucgaucugacu...	7	0	MW2
............................gguuuucauuuucgaucugacuu..	3	0	MW2
....agugagaucacuuugaaagcugau....................	1	0	MW2
....gugagaucacuuugaaagcug......................	1	0	MW2
....gugagaucacuuugaaagcugau....................	3	0	MW2
....Cugagaucacuuugaaagcugau....................	1	1	MW2
....Cugagaucacuuugaaagcugauu...................	1	1	MW2
....ugagaucacuuugaaag.........................	15	0	MW2
....ugagaucacuuugaaagc........................	24	0	MW2
....ugagaucacuuugaaagcu.......................	6	0	MW2
....ugagaucacuuugaaagcug......................	140	0	MW2
....ugagaucacuuugaaagcuga.....................	332	0	MW2
....ugagaucacuuugaaagcCga.....................	1	1	MW2
....ugagaucacuuCgaaagcuga.....................	1	1	MW2
....ugagaucacuuugGaagcuga.....................	1	1	MW2
....ugagaucacuuugaaagcugG.....................	3	1	MW2
....ugaAaucacuuugaaagcugau....................	1	1	MW2
....ugagaucacuuGgaaagcugau....................	2	1	MW2
....Cgagaucacuuugaaagcugau....................	1	1	MW2
....ugagauUacuuugaaagcugau....................	1	1	MW2
....ugagaucacuuugaaagAugau....................	1	1	MW2
....ugagaucacuuugaaaAcugau....................	1	1	MW2
....ugagaucacuuugaaagcugaC....................	1	1	MW2
....ugagaucaUuuugaaagcugau....................	1	1	MW2
....ugagaucacuuugGaagcugau....................	1	1	MW2
....uAagaucacuuugaaagcugau....................	1	1	MW2
....uUagaucacuuugaaagcugau....................	1	1	MW2
....ugagaucacuuugaaagcuAau....................	2	1	MW2
....ugagaucacuuugaaagcugau....................	2362	0	MW2
....ugagaCcacuuugaaagcugau....................	2	1	MW2
....ugagaucacAuugaaagcugau....................	1	1	MW2
....ugagaucacuuugaaaUcugau....................	1	1	MW2

```
                          aga-bantam*
                                                                           aga-bantam
uguguggaugaaauguaaucacagaaccgguuuucauuuucgaucugacuuauuuuuuucacaaacaagugagaucacuuugaaagcugauuuuguacaauuaauucaacg

.....................................................................ugagaucacuuCgaaagcugau....................  1     1   MW2
.....................................................................ugagaucacuuugaGagcugau....................  1     1   MW2
.....................................................................ugagaucacuuugaaagcugauA...................  11    1   MW2
.....................................................................ugagaucacuuugaaagcuAauu...................  1     1   MW2
.....................................................................ugagauAacuuugaaagcugauu...................  1     1   MW2
.....................................................................ugagaucacuCugaaagcugauu...................  1     1   MW2
.....................................................................ugagaucacuuugaaagcugauu...................  973   0   MW2
.....................................................................uAagaucacuuugaaagcugauu...................  1     1   MW2
.....................................................................ugagaucacuuuAaaagcugauu...................  1     1   MW2
.....................................................................ugagaucacuuugaaaCcugauu...................  1     1   MW2
.....................................................................ugagaucacuuugaaagcAgauu...................  1     1   MW2
.....................................................................Ggagaucacuuugaaagcugauu...................  1     1   MW2
.....................................................................ugagaucacuuugaaagcugaAu...................  2     1   MW2
.....................................................................ugagaucacuuugaaagcugauuA..................  8     1   MW2
.....................................................................ugagaucacuuugaaagcugauuu..................  37    0   MW2
......................................................................gagaucacuuugaaagcugau....................  1     0   MW2
........................................................................gaucacuuugaaagcugau....................  1     0   MW2
.........................................................................aucacuuugaaagcugau....................  1     0   MW2
.........................................................................aucacuuugaaagcugauu...................  1     0   MW2
...........................................................................cacuuugaaagcugauu...................  1     0   MW2

..........................ccgguuuucauuuucgaucug................................................................  2     0   TE2
..........................ccgguuuucauuuucgaucuga...............................................................  14    0   TE2
..........................ccgguuuucauAuucgaucugac..............................................................  1     1   TE2
..........................ccgguuuucauuuucgaucugac..............................................................  62    0   TE2
..........................ccgguuuucauuuucgaucugacG.............................................................  2     1   TE2
..........................ccgguuuucUuuucgaucugacu..............................................................  1     1   TE2
..........................ccgguuuucauuuucgaCcugacu.............................................................  2     1   TE2
..........................ccgguuuucauuuucgaucugacC.............................................................  1     1   TE2
..........................ccgguuuucauuuucgaucugacu.............................................................  1275  0   TE2
..........................ccgguuuucauuuucgaucugacA.............................................................  4     1   TE2
..........................ccgguuuucauuuucgCucugacu.............................................................  1     1   TE2
..........................ccgguuuucauCuucgaucugacu.............................................................  1     1   TE2
..........................ccgguuuucauuCucgaucugacu.............................................................  2     1   TE2
..........................ccgguuuucauuuucgaucCgacu.............................................................  2     1   TE2
..........................ccgguuuucauuuCcgaucugacu.............................................................  1     1   TE2
..........................ccgguuuucaCuuucgaucugacu.............................................................  1     1   TE2
..........................ccgguuuucauuuucgaucugGcu.............................................................  1     1   TE2
..........................ccgguuuucauuuucgaucugaUu.............................................................  1     1   TE2
..........................ccgUuuuucauuuucgaucugacu.............................................................  1     1   TE2
..........................ccgguuuucauuuucgaucugacuu............................................................  3     0   TE2
..........................ccgguuuucauuuucgaucugacuA............................................................  2     1   TE2
...........................cgguuuucauuuucgaucugac..............................................................  1     0   TE2
...........................cgguuuucauuuucgaucugacu.............................................................  28    0   TE2
............................gguuuucauuuucgaucuga...............................................................  1     0   TE2
............................gguuuucauuuucgaucugacu.............................................................  21    0   TE2
............................gguuuucauuuucgaucuAacu.............................................................  2     1   TE2
............................gguuuucauuuucgaucugacuu............................................................  5     0   TE2
....................................................................gugagaucacuuugaaagcu.......................  1     0   TE2
....................................................................gugagaucacuuugaaagcug......................  2     0   TE2
....................................................................gugagaucacuuugaaagcuga.....................  1     0   TE2
....................................................................gugagaucacuuugaaagcugau....................  10    0   TE2
....................................................................Cugagaucacuuugaaagcugau....................  6     1   TE2
....................................................................Cugagaucacuuugaaagcugauu...................  1     1   TE2
.....................................................................ugagaucacuuugaaag.........................  122   0   TE2
.....................................................................uAagaucacuuugaaag.........................  1     1   TE2
.....................................................................ugagaucacuuuAaaagc........................  1     1   TE2
.....................................................................ugagaucacAuugaaagc........................  1     1   TE2
.....................................................................ugagaucacuuugaaaCc........................  1     1   TE2
.....................................................................ugagaucacuuugaaaAc........................  1     1   TE2
.....................................................................ugagaucacuuugaaagA........................  2     1   TE2
.....................................................................ugagaucacuuugaaagc........................  278   0   TE2
.....................................................................ugagaucacuuugaaagcG.......................  4     1   TE2
.....................................................................ugagaucacuuugaaagcu.......................  331   0   TE2
.....................................................................ugagaucacuuugaaagcA.......................  4     1   TE2
.....................................................................ugagaucacuuCgaaagcug......................  1     1   TE2
.....................................................................ugagaucacuuugaaaUcug......................  2     1   TE2
.....................................................................ugagaucacuuugaaagAug......................  1     1   TE2
.....................................................................ugagaucacuuuAaaagcug......................  4     1   TE2
```

```
                          aga-bantam*
                                                                     aga-bantam
uguguggaugaaauguaaucacagaaccgguuuucauuuucgaucugacuuauuuuuuucacaaacaagugagaucacuuugaaagcugauuuuguacaauuaauucaacg

.....................................................................ugagaucacuuGgaaagcug......................  1     1   TE2
.....................................................................ugagaucacuuugaaaCcug......................  1     1   TE2
.....................................................................ugagaucacCuugaaagcug......................  1     1   TE2
.....................................................................ugaCaucacuuugaaagcug......................  1     1   TE2
.....................................................................ugagaucacuuugaGagcug......................  2     1   TE2
.....................................................................uAagaucacuuugaaagcug......................  3     1   TE2
.....................................................................ugagaucacuCugaaagcug......................  1     1   TE2
.....................................................................ugagaucacuuugaaagcuA......................  4     1   TE2
.....................................................................Cgagaucacuuugaaagcug......................  1     1   TE2
.....................................................................ugagaucacuuugaaagcGg......................  8     1   TE2
.....................................................................ugagaucacuuAgaaagcug......................  3     1   TE2
.....................................................................ugagaucacuuugaaagcug......................  2459  0   TE2
.....................................................................ugagaucacuuugaaagUug......................  1     1   TE2
.....................................................................ugagaucacuAugaaagcug......................  1     1   TE2
.....................................................................ugUgaucacuuugaaagcug......................  1     1   TE2
.....................................................................ugagauUacuuugaaagcug......................  2     1   TE2
.....................................................................ugagauUacuuugaaagcuga.....................  1     1   TE2
.....................................................................ugagaucacuCugaaagcuga.....................  1     1   TE2
.....................................................................ugagaucacuuCgaaagcuga.....................  2     1   TE2
.....................................................................uCagaucacuuugaaagcuga.....................  1     1   TE2
.....................................................................ugagaucacuuugaaaAcuga.....................  2     1   TE2
.....................................................................uAagaucacuuugaaagcuga.....................  1     1   TE2
.....................................................................ugagaucaUuuugaaagcuga.....................  2     1   TE2
.....................................................................ugagaucacAuugaaagcuga.....................  4     1   TE2
.....................................................................ugUgaucacuuugaaagcuga.....................  1     1   TE2
.....................................................................ugagaCcacuuugaaagcuga.....................  1     1   TE2
.....................................................................ugagaucacuuugaaagcuga.....................  1723  0   TE2
.....................................................................Cgagaucacuuugaaagcuga.....................  2     1   TE2
.....................................................................ugagaucacCuugaaagcuga.....................  1     1   TE2
.....................................................................ugagaucacGuugaaagcuga.....................  1     1   TE2
.....................................................................ugagaucacuuugaaagcugG.....................  26    1   TE2
.....................................................................ugagaCcacuuugaaagcugau....................  4     1   TE2
.....................................................................ugagaucacuuugaaagcuAau....................  2     1   TE2
.....................................................................ugagaucacuuGgaaagcugau....................  1     1   TE2
.....................................................................ugGgaucacuuugaaagcugau....................  1     1   TE2
.....................................................................ugagaucacCuugaaagcugau....................  2     1   TE2
.....................................................................ugagaucUcuuugaaagcugau....................  1     1   TE2
.....................................................................ugaAaucacuuugaaagcugau....................  3     1   TE2
.....................................................................ugagaucacuuugUaagcugau....................  1     1   TE2
.....................................................................ugagauUacuuugaaagcugau....................  2     1   TE2
.....................................................................ugagaucacuuugaaagcugau....................  4929  0   TE2
.....................................................................ugagaucaUuuugaaagcugau....................  1     1   TE2
.....................................................................ugagaucacuuugaaagcugaC....................  1     1   TE2
.....................................................................ugagaucacuuugaCagcugau....................  1     1   TE2
.....................................................................ugagaucacuuugaaagcugGu....................  2     1   TE2
.....................................................................ugagaucaAuuugaaagcugau....................  2     1   TE2
.....................................................................uUagaucacuuugaaagcugau....................  1     1   TE2
.....................................................................ugagaucacuuugaaagcugaA....................  3     1   TE2
.....................................................................uAagaucacuuugaaagcugau....................  2     1   TE2
.....................................................................ugagauAacuuugaaagcugau....................  2     1   TE2
.....................................................................ugagaucacuuugaaaCcugau....................  3     1   TE2
.....................................................................Cgagaucacuuugaaagcugau....................  2     1   TE2
.....................................................................uCagaucacuuugaaagcugau....................  1     1   TE2
.....................................................................ugagaucacuuugaaaAcugau....................  3     1   TE2
.....................................................................ugagaucacuuugaGagcugau....................  1     1   TE2
.....................................................................ugagaucacuuugaaagcCgau....................  4     1   TE2
.....................................................................ugagaucacuGugaaagcugau....................  1     1   TE2
.....................................................................Ggagaucacuuugaaagcugau....................  1     1   TE2
.....................................................................ugagaucacuuugaaagcugaG....................  3     1   TE2
.....................................................................ugagaucacuCugaaagcugau....................  1     1   TE2
.....................................................................ugagaucacuuCgaaagcugau....................  5     1   TE2
.....................................................................ugagaucacuuuAaaagcugau....................  5     1   TE2
.....................................................................ugagaucacGuugaaagcugau....................  1     1   TE2
.....................................................................ugagaucacuuugaaagcugauA...................  16    1   TE2
.....................................................................Cgagaucacuuugaaagcugauu...................  1     1   TE2
.....................................................................ugagaucacuuugaaaCcugauu...................  2     1   TE2
.....................................................................ugagaucacuuuAaaagcugauu...................  1     1   TE2
.....................................................................ugagaucacuuugaaagcugUuu...................  1     1   TE2
.....................................................................ugUgaucacuuugaaagcugauu...................  1     1   TE2
```

```
                          aga-bantam*
                                                                     aga-bantam
uguguggaugaaauguaaucacagaaccgguuuucauuuucgaucugacuuauuuuuuucacaaacaagugagaucacuuugaaagcugauuuuguacaauuaauucaacg

.....................................................................ugagaucacuuAgaaagcugauu...................   1      1      TE2
.....................................................................ugagaucacuuugaaagcugaGu...................   2      1      TE2
.....................................................................ugagaCcacuuugaaagcugauu...................   1      1      TE2
.....................................................................ugagaucacuuCgaaagcugauu...................   1      1      TE2
.....................................................................ugagauUacuuugaaagcugauu...................   1      1      TE2
.....................................................................ugagaucacuuugaaagcugauu...................   1404   0      TE2
.....................................................................ugagaucaUuuugaaagcugauu...................   1      1      TE2
.....................................................................ugagaucacuuugaaagcugaCu...................   1      1      TE2
.....................................................................ugagaucacuuGgaaagcugauu...................   2      1      TE2
.....................................................................ugagaucacuuugaaagcugauG...................   2      1      TE2
.....................................................................ugagaucacuuugaaaUcugauu...................   1      1      TE2
.....................................................................ugagaucacuuugaaagcCgauu...................   1      1      TE2
.....................................................................ugagGucacuuugaaagcugauu...................   1      1      TE2
.....................................................................ugagaucacuuugaaagcugGuu...................   1      1      TE2
.....................................................................uAagaucacuuugaaagcugauu...................   3      1      TE2
.....................................................................ugagaucacuuGgaaagcugauuu..................   1      1      TE2
.....................................................................ugagaucacuuugaaagcugauuA..................   16     1      TE2
.....................................................................ugagaucacuuugaaagcugauuu..................   48     0      TE2
.....................................................................ugagaucacuuugaaagcugauuC..................   2      1      TE2
......................................................................gagaucacuuugaaagcug......................   3      0      TE2
......................................................................gagaucacuuugaaagcugauu...................   1      0      TE2
```

miRBase precursor : aga-let-7
Total read count : 11938
aga-let-7 read count : 11707
aga-let-7* read count : 217
remaining reads : 14

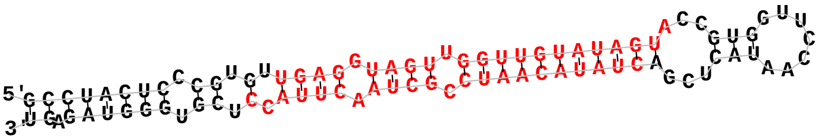

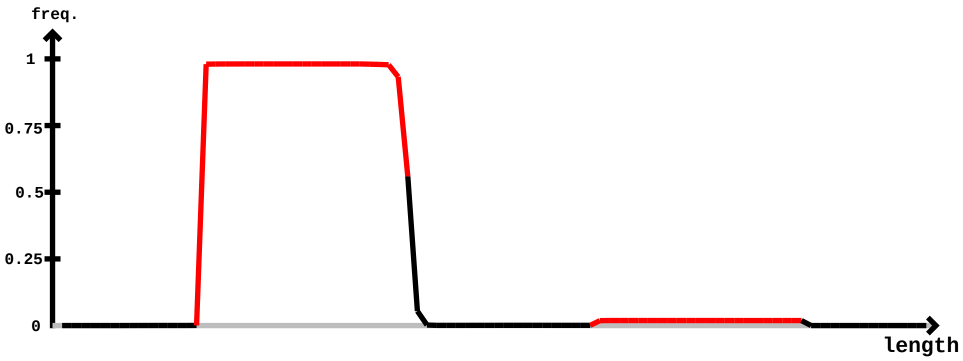


```
                                                              aga-let-7*
              aga-let-7
5'- gccuacucccgugu ugagguaguugguuguauagua ccgugguuccaauacucga cuauacaauccgcuaacuuacc ucgugggguagagu  -3'    exp
    ((((((((.((.(.((((.((((.((((((((((...(((.......)))...)))))))))).)))).)))).).)).)))))).))   reads   mm   sample
    ..............ugagguaguugguuguau..........................................................   1       0    FF2
    ..............ugagguaguugguuguauag........................................................   4       0    FF2
    ..............ugagguaguugguuguauagu.......................................................   70      0    FF2
    ..............ugagguaguugguuguauaAu......................................................   1       1    FF2
    ..............ugagguaguugguugCauagu.......................................................   1       1    FF2
    ..............ugagguaguugguuguauagua......................................................   160     0    FF2
    ..............ugagAuaguugguuguauagua......................................................   1       1    FF2
    ..............ugagguaguugguuguauaguU......................................................   2       1    FF2
    ..............uAagguaguugguuguauagua......................................................   1       1    FF2
    ..............ugagguaguuggCuguauagua......................................................   1       1    FF2
    ..............ugagguaguCgguuguauagua......................................................   1       1    FF2
    ..............ugagguaguugguuguauaguaU.....................................................   4       1    FF2
    ..............ugagguaguugguuguauaguaA.....................................................   14      1    FF2
    .......................................................cuauacaauccgcuaacuuacc.............   5       0    FF2

    ..............ugagguaguugguuguau..........................................................   2       0    OV2
    ..............ugagguaguugguuguaua.........................................................   3       0    OV2
    ..............ugagguaguugguuguauag........................................................   80      0    OV2
    ..............ugagguaguugguuguauaU........................................................   1       1    OV2
    ..............ugagguaguugguuAuauag........................................................   1       1    OV2
    ..............ugagguaguugguuguauagC.......................................................   1       1    OV2
    ..............Cgagguaguugguuguauagu.......................................................   1       1    OV2
    ..............ugagguaguugguuguauagu.......................................................   493     0    OV2
    ..............ugagguaguugguuguauagA.......................................................   2       1    OV2
    ..............ugagAuaguugguuguauagu.......................................................   1       1    OV2
    ..............ugagguaguuggGuguauagu.......................................................   1       1    OV2
    ..............ugagguaguugguugCauagu.......................................................   1       1    OV2
    ..............ugagguaguugguuguauaAua......................................................   1       1    OV2
    ..............ugagguaguugguuguauaguC......................................................   1       1    OV2
    ..............ugagguaguugguuguauagua......................................................   192     0    OV2
    ..............ugagguaguugguuguauaguU......................................................   10      1    OV2
    ..............ugagguaguugguuguauagAa......................................................   1       1    OV2
    ..............ugagguaguugguuguaAagua......................................................   1       1    OV2
    ..............ugagguaguugguuguauaguac.....................................................   1       0    OV2
```

```
                                                       aga-let-7*
              aga-let-7
gccuacucccguguugagguaguugguuguauaguaccgugguuccaauacucgacuauacaauccgcuaacuuaccucguggguagagu

..............ugagguaguugguuguauaguaU.....................................................   13    1    OV2
..............ugagguaguugguuguauaguaA.....................................................   49    1    OV2
..............ugagguaguugguuguauaguaAc....................................................   1     1    OV2
..............ugagguaguugguuguauaguacc....................................................   1     0    OV2
.......................................................cuauacaauccgcuaacuuacc.............   3     0    OV2
........................................................uauacaauccgcuaacuuacc.............   1     0    OV2

.............uugagguaguugguuguauagu.......................................................   1     0    TE1
..............ugagguaguugguugua...........................................................   1     0    TE1
..............ugagguaguugguuguauag........................................................   11    0    TE1
..............ugagguaguugguuguauagu.......................................................   173   0    TE1
..............ugagguaguugguuguauaguG......................................................   1     1    TE1
..............ugagguaguugguuguauagua......................................................   220   0    TE1
..............ugagguaguugguuguauaguU......................................................   1     1    TE1
..............ugagguaguugguuguauaguaU.....................................................   8     1    TE1
..............ugagguaguugguuguauaguac.....................................................   2     0    TE1
..............ugagguaguugguuguauaguaA.....................................................   22    1    TE1
...............gagguaguugguuguauagua......................................................   1     0    TE1
....................................ccgugguuccGauacucgacuauacaauccgcuaacuuacc.............   1     1    TE1
.......................................................cuauacaauccgcuaacuuacc.............   4     0    TE1

..............uAagguaguugguuguauag........................................................   1     1    MF2
..............ugagguaguugguuguauag........................................................   13    0    MF2
..............ugagguagCugguuguauagu.......................................................   2     1    MF2
..............ugagguaguugguuguauagC.......................................................   1     1    MF2
..............ugagguGguugguuguauagu.......................................................   1     1    MF2
..............uAagguaguugguuguauagu.......................................................   1     1    MF2
..............ugagguaguuAguuguauagu.......................................................   1     1    MF2
..............ugagguaguuggAuguauagu.......................................................   1     1    MF2
..............ugagguaguugguuguauagu.......................................................   341   0    MF2
..............ugagguaguugguugGauagu.......................................................   1     1    MF2
..............ugaggCaguugguuguauagua......................................................   2     1    MF2
..............uAagguaguugguuguauagua......................................................   1     1    MF2
..............ugagguaguugguuguauaguU......................................................   12    1    MF2
..............ugagguaguugguuguauaguG......................................................   4     1    MF2
..............ugagguagCugguuguauagua......................................................   1     1    MF2
..............ugagguaguugguuguauUgua......................................................   1     1    MF2
..............ugagAuaguugguuguauagua......................................................   1     1    MF2
..............ugagguaguugguuguauaguC......................................................   1     1    MF2
..............ugaggGaguugguuguauagua......................................................   1     1    MF2
..............ugagguaguugguuguauagua......................................................   984   0    MF2
..............ugagguaguugguuguauaguaA.....................................................   76    1    MF2
..............ugagguaguugguuguauaguac.....................................................   2     0    MF2
..............ugagguaguugguuguauaguaU.....................................................   23    1    MF2
..............ugagguaguugguuguauaguacc....................................................   2     0    MF2
..............ugagguaguugguuguauaguaccgugguuccaaua........................................   1     0    MF2
.............................................caauacucgacuauacaauccgcuaacuuacc.............   2     0    MF2
.......................................................cuauacaauccgcuaacuuacc.............   22    0    MF2
.......................................................cuauacaauccgcuaacuuaGc.............   1     1    MF2

..............ugagguaguugguugua...........................................................   1     0    FW2
..............ugagguaguugguuguauag........................................................   13    0    FW2
..............ugCgguaguugguuguauagu.......................................................   1     1    FW2
..............ugagguaguugguuguauagu.......................................................   182   0    FW2
..............ugagguaguugguuguauagG.......................................................   1     1    FW2
..............ugagguaguugguuguauaguU......................................................   6     1    FW2
..............ugagguaguugguuguaAagua......................................................   1     1    FW2
..............ugaggCaguugguuguauagua......................................................   1     1    FW2
..............ugagguaguugguugCauagua......................................................   2     1    FW2
..............ugagguaguuggCuguauagua......................................................   1     1    FW2
..............Cgagguaguugguuguauagua......................................................   1     1    FW2
..............ugagguaguugguuguauagua......................................................   499   0    FW2
..............ugagguaguugguuguauaguaA.....................................................   22    1    FW2
..............ugagguaguugguuguauaguaU.....................................................   6     1    FW2
.......................................................cuauacaauccgcuaacuuacc.............   33    0    FW2
........................................................uauacaauccgcuaacuuacc.............   2     0    FW2

..............ugagguaguugguuguauag........................................................   21    0    FF1
..............ugagguaguugguuguauagu.......................................................   184   0    FF1
```

```
                                                       aga-let-7*
              aga-let-7
gccuacucccguguugagguaguugguuguauaguaccgugguuccaauacucgacuauacaauccgcuaacuuaccucguggguagagu

..............ugagguaguugguugCauagu.......................................................   1     1     FF1
..............ugagguaguugCuuguauagu.......................................................   1     1     FF1
..............ugagguaguugguuguauagA.......................................................   1     1     FF1
..............ugagguaguuggCuguauagu.......................................................   1     1     FF1
..............ugagguaguuggCuguauagua......................................................   1     1     FF1
..............ugagguaguugguCguauagua......................................................   1     1     FF1
..............ugagguaguugguuguauagua......................................................   296   0     FF1
..............ugaggGaguugguuguauagua......................................................   1     1     FF1
..............ugagguaguugguuguauaguU......................................................   5     1     FF1
..............ugagguaguugguuguauaguC......................................................   1     1     FF1
..............ugagguaguugguuguauaguaU.....................................................   12    1     FF1
..............ugagguaguugguuguauaguaA.....................................................   23    1     FF1
..............ugagguaguugguuguauaguacc....................................................   1     0     FF1
..............ugagguaguugguuguauaguaAc....................................................   1     1     FF1
....................................ccgugguuccaauacucgacuauacaauccgcuaacuuacc.............   1     0     FF1
.......................................................cuauacaauccgcuaacuuacc.............   17    0     FF1

..............ugagguaguugguugua...........................................................   5     0     OV1
..............ugagguaguugguuguau..........................................................   4     0     OV1
..............ugagguaguugguuguaua.........................................................   7     0     OV1
..............ugagguaguugguuguauag........................................................   227   0     OV1
..............ugagguaguugguuguauaA........................................................   1     1     OV1
..............ugagguaguugguuguauaU........................................................   1     1     OV1
..............ugagguaguugguCguauag........................................................   1     1     OV1
..............Cgagguaguugguuguauag........................................................   1     1     OV1
..............ugagguaguugguuguauGg........................................................   1     1     OV1
..............ugagUuaguugguuguauagu.......................................................   1     1     OV1
..............ugaggCaguugguuguauagu.......................................................   1     1     OV1
..............ugagguaguugguuAuauagu.......................................................   1     1     OV1
..............ugagguaguuggCuguauagu.......................................................   2     1     OV1
..............ugagguaguuAguuguauagu.......................................................   1     1     OV1
..............ugagguaguugguuguauagu.......................................................   959   0     OV1
..............ugGgguaguugguuguauagu.......................................................   1     1     OV1
..............uAagguaguugguuguauagu.......................................................   1     1     OV1
..............ugagguaguugguuguauagA.......................................................   4     1     OV1
..............ugagguaguugguuguauagCa......................................................   1     1     OV1
..............ugagguaguugguuguaCagua......................................................   1     1     OV1
..............ugagguaguuggGuguauagua......................................................   1     1     OV1
..............ugagguaguugguuguauaguC......................................................   1     1     OV1
..............ugagguaguugguuguauaguG......................................................   1     1     OV1
..............ugaggGaguugguuguauagua......................................................   1     1     OV1
..............ugagguaguugguuguauagua......................................................   366   0     OV1
..............ugagguaguugguuguauaguU......................................................   24    1     OV1
..............ugagguaguugguuguauaguaU.....................................................   38    1     OV1
..............ugagguaguugguuguauaguaA.....................................................   68    1     OV1
..............ugagguaguugguuguauaguac.....................................................   3     0     OV1
..............ugagguaguugguuguauaguacc....................................................   1     0     OV1
.......................................................cuauacaauccgcuaacuuacc.............   12    0     OV1
........................................................uauacaauccgcuaacuuacc.............   1     0     OV1

..............ugagguaguugguuguauag........................................................   3     0     MF1
..............ugagguaguugguuguauagu.......................................................   99    0     MF1
..............ugagguaguugguuguaAagu.......................................................   1     1     MF1
..............uAagguaguugguuguauagu.......................................................   1     1     MF1
..............ugagguaguugguuguauagua......................................................   153   0     MF1
..............ugagguaguugguuguauaguC......................................................   1     1     MF1
..............ugagguaguugguuguauaguU......................................................   3     1     MF1
..............ugagguaguugguuguauaguaU.....................................................   3     1     MF1
..............ugagguaguugguuguauaguaA.....................................................   8     1     MF1
.......................................................cuauacaauccgcuaacuuacc.............   3     0     MF1

......ucccgugGugagguaguugguuguauagu.......................................................   1     1     BF2
.......cccgugGugagguaguugguuguauagua......................................................   1     1     BF2
..............ugagguaguugguuguau..........................................................   1     0     BF2
..............ugagguaguugguuguaua.........................................................   1     0     BF2
..............Agagguaguugguuguauag........................................................   1     1     BF2
..............ugagguGguugguuguauag........................................................   1     1     BF2
..............ugagguaguugguuguauag........................................................   83    0     BF2
..............ugagguaguugguuguauagA.......................................................   1     1     BF2
```

```
                                                       aga-let-7*
              aga-let-7
gccuacucccguguugagguaguugguuguauaguaccgugguuccaauacucgacuauacaauccgcuaacuuaccucgugggguagagu

..............ugaggGaguugguuguauagu.......................................................   2     1   BF2
..............ugagguaguuUguuguauagu.......................................................   1     1   BF2
..............ugagguaguugguuguauagu.......................................................   569   0   BF2
..............ugagguaguugguuguauagG.......................................................   1     1   BF2
..............ugagguaguuggAuguauagua......................................................   1     1   BF2
..............ugagguaguugguuguauagua......................................................   704   0   BF2
..............ugagguaguCgguuguauagua......................................................   2     1   BF2
..............ugagguaguugguuguauaguU......................................................   13    1   BF2
..............ugagguaguugguuguauaguG......................................................   3     1   BF2
..............ugagguaguugUuuguauagua......................................................   1     1   BF2
..............ugagguaguugguCguauagua......................................................   1     1   BF2
..............ugagguaguugguuguaCagua......................................................   1     1   BF2
..............ugagguaUuugguuguauagua......................................................   1     1   BF2
..............uAgguaguugguuguauagua.......................................................   1     1   BF2
..............ugagguaguugguuguauaguC......................................................   1     1   BF2
..............ugaggGaguugguuguauagua......................................................   1     1   BF2
..............ugagguaguugguuguauaguaU.....................................................   14    1   BF2
..............ugagguaguugguuguauaguaA.....................................................   56    1   BF2
..............ugagguaguugguuguauaguac.....................................................   2     0   BF2
..............ugagguaguugguuguauaguaAc....................................................   1     1   BF2
..............ugagguaguugguuguauaguacc....................................................   2     0   BF2
...............gagguaguugguuguauag........................................................   1     0   BF2
................agguaguugguuguauagua......................................................   1     0   BF2
..................guaguugguuguauagu.......................................................   1     0   BF2
....................................ccgugguuccaauacucgacuauacaauccgcuaacuuacc.............   1     0   BF2
..........................................uuccaauacucgacuauacaauccgcu.....................   1     0   BF2
.......................................................cuauacaauccgcuaacuuU...............   1     1   BF2
.......................................................cuauacaauccgcuaacuuacc.............   21    0   BF2

..............ugagguaguugguuguauag........................................................   21    0   BF1
..............ugagguaguugguuguauagA.......................................................   1     1   BF1
..............ugagguaguugguugCauagu.......................................................   1     1   BF1
..............ugagguaguugguuguauagu.......................................................   183   0   BF1
..............ugagguaguugCuuguauagu.......................................................   1     1   BF1
..............ugagguaguuggCuguauagu.......................................................   1     1   BF1
..............ugagguaguugguuguauagua......................................................   296   0   BF1
..............ugagguaguugguuguauaguU......................................................   5     1   BF1
..............ugaggGaguugguuguauagua......................................................   1     1   BF1
..............ugagguaguugguCguauagua......................................................   1     1   BF1
..............ugagguaguuggCuguauagua......................................................   1     1   BF1
..............ugagguaguugguuguauaguC......................................................   1     1   BF1
..............ugagguaguugguuguauaguaA.....................................................   23    1   BF1
..............ugagguaguugguuguauaguaU.....................................................   12    1   BF1
..............ugagguaguugguuguauaguacc....................................................   1     0   BF1
..............ugagguaguugguuguauaguaAc....................................................   1     1   BF1
....................................ccgugguuccaauacucgacuauacaauccgcuaacuuacc.............   1     0   BF1
.......................................................cuauacaauccgcuaacuuacc.............   17    0   BF1

.........cgugGugagguaguugguuguauagua......................................................   1     1   MW1
.............Cugagguaguugguuguauagua......................................................   1     1   MW1
..............ugagguaguugguugua...........................................................   1     0   MW1
..............ugagguaguugguuguau..........................................................   1     0   MW1
..............ugagguaguugguuguaua.........................................................   1     0   MW1
..............ugagguaguugguuguauag........................................................   16    0   MW1
..............ugagguaguugguugCauagu.......................................................   1     1   MW1
..............ugagguaguugAuuguauagu.......................................................   1     1   MW1
..............ugagguaguuggGuguauagu.......................................................   1     1   MW1
..............ugagguaguugguuguauagu.......................................................   397   0   MW1
..............ugaggCaguugguuguauagu.......................................................   1     1   MW1
..............ugaggAaguugguuguauagu.......................................................   1     1   MW1
..............ugagguaguuggCuguauagu.......................................................   1     1   MW1
..............ugagguaguAgguuguauagua......................................................   1     1   MW1
..............ugaggCaguugguuguauagua......................................................   2     1   MW1
..............ugagguaguugguuguaCagua......................................................   1     1   MW1
..............ugagguaguCgguuguauagua......................................................   1     1   MW1
..............ugagguaguugguuguauagua......................................................   869   0   MW1
..............ugagguaguugguAguauagua......................................................   1     1   MW1
..............ugagguaguugguuguauaguU......................................................   6     1   MW1
..............ugagguaguuggAuguauagua......................................................   1     1   MW1
```

```
                                                     aga-let-7*
               aga-let-7
gccuacucccguguugagguaguugguuguauaguaccgugguuccaauacucgacuauacaauccgcuaacuuaccucguggguagagu

..............Cgagguaguugguuguauagua......................................................   1     1     MW1
..............ugagguaguugguuguauGgua......................................................   1     1     MW1
..............ugagguaguugguuguauaguac.....................................................   3     0     MW1
..............ugagguaguugguuguauaguaU.....................................................   11    1     MW1
..............ugagguaguugguuguauaguaG.....................................................   1     1     MW1
..............ugagguaguugguuguauaguaA.....................................................   30    1     MW1
..............ugagguaguugguuguauaguacc....................................................   1     0     MW1
..............ugagguaguugguuguauaguaAc....................................................   1     1     MW1
...............gagguaguugguuguauagu.......................................................   1     0     MW1
....................................ccgugguuccGauacucga...................................   1     1     MW1
....................................ccgugguuccaauacucgacuauacaauccgcuaacuuacc.............   1     0     MW1
.......................................................cuauacaauccgcuaacuuacc.............   27    0     MW1

.............Cugagguaguugguuguauagu.......................................................   1     1     FW1
.............Cugagguaguugguuguauagua......................................................   1     1     FW1
..............ugagguaguugguuguaua.........................................................   1     0     FW1
..............ugagguaguugguuguauag........................................................   4     0     FW1
..............ugagguaguugguuguauagu.......................................................   142   0     FW1
..............ugagguaguugAuuguauagua......................................................   1     1     FW1
..............ugagguaguugguuguauaguU......................................................   2     1     FW1
..............ugaggGaguugguuguauagua......................................................   1     1     FW1
..............ugagguagCugguuguauagua......................................................   1     1     FW1
..............ugagguaguugguuguauagua......................................................   392   0     FW1
..............ugagguaguugguuguauaguaA.....................................................   4     1     FW1
..............ugagguaguugguuguauaguac.....................................................   2     0     FW1
..............ugagguaguugguuguauaguaU.....................................................   2     1     FW1
....................................ccgugguuccaauacucgacuauacaauccgcuaacuuacc.............   1     0     FW1
.......................................................cuauacaauccgcuaacuuacc.............   24    0     FW1

..............ugagguaguugguuguauag........................................................   4     0     MW2
..............ugagguaguugguuguauagu.......................................................   59    0     MW2
..............ugagguaguuggAuguauagua......................................................   1     1     MW2
..............ugagguaguugguuAuauagua......................................................   1     1     MW2
..............ugagguaguuggCuguauagua......................................................   1     1     MW2
..............ugagguGguugguuguauagua......................................................   1     1     MW2
..............ugagguaguugguuguauagua......................................................   177   0     MW2
..............ugagguaguugguuguauaguG......................................................   1     1     MW2
..............ugagguaguugUuuguauagua......................................................   2     1     MW2
..............ugagguaguugguCguauagua......................................................   1     1     MW2
..............ugagguaguugguuguauaguU......................................................   1     1     MW2
..............ugagguaguugguuguauaguaU.....................................................   1     1     MW2
..............ugagguaguugguuguauaguaA.....................................................   3     1     MW2
..............ugagguaguugguuguauaguacc....................................................   1     0     MW2
.......................................................cuauacaauccgcuaacuuac..............   1     0     MW2
.......................................................cuauacaauccgcuaacuuacc.............   10    0     MW2

..............ugagguaguugguugua...........................................................   3     0     TE2
..............ugagguaguugguuguau..........................................................   1     0     TE2
..............ugagguaguugguuguauU.........................................................   2     1     TE2
..............ugagguaguugguuguaua.........................................................   1     0     TE2
..............ugagguaguugguuguauag........................................................   30    0     TE2
..............ugagguaguuggAuguauagu.......................................................   1     1     TE2
..............ugagguaguugguGguauagu.......................................................   1     1     TE2
..............Cgagguaguugguuguauagu.......................................................   1     1     TE2
..............ugaggCaguugguuguauagu.......................................................   1     1     TE2
..............ugagguaguugguuguauagu.......................................................   542   0     TE2
..............ugaggCaguugguuguauagua......................................................   2     1     TE2
..............ugagguaguugguugCauagua......................................................   1     1     TE2
..............ugagguaguugAuuguauagua......................................................   1     1     TE2
..............ugagguaguuggCuguauagua......................................................   4     1     TE2
..............ugagguaguugguuguauaguU......................................................   8     1     TE2
..............ugaggGaguugguuguauagua......................................................   2     1     TE2
..............ugagguagCugguuguauagua......................................................   1     1     TE2
..............ugagguaguugguuAuauagua......................................................   1     1     TE2
..............ugagguaguugguuguauaguG......................................................   1     1     TE2
..............uAagguaguugguuguauagua......................................................   1     1     TE2
..............ugagguaguugguuguauagua......................................................   533   0     TE2
..............ugagguaguugguuguauaguac.....................................................   5     0     TE2
..............ugagguaguugguuguauaguaA.....................................................   37    1     TE2
```

```
                                                      aga-let-7*
              aga-let-7
gccuacucccguguugagguaguugguuguauaguaccgugguuccaauacucgacuauacaauccgcuaacuuaccucgugguagagu

..............ugagguaguugguuguauaguaU.....................................................    19     1     TE2
..............ugagguaguugguuguauaguacc....................................................    1      0     TE2
...............gagguaguugguuguauag........................................................    1      0     TE2
...............gagguaguugguuguauagu.......................................................    2      0     TE2
...............gagguaguugguuguauagua......................................................    2      0     TE2
................agguaguugguuguauaguac.....................................................    1      0     TE2
.......................................................cuauacaauccgcuaacuuacc.............    10     0     TE2
........................................................uauacaauccgcuaacuuacc.............    2      0     TE2
```

miRBase precursor : aga-mir-1000
Total read count : 429
aga-miR-1000 read count : 411
aga-miR-1000* read count : 13
remaining reads : 5

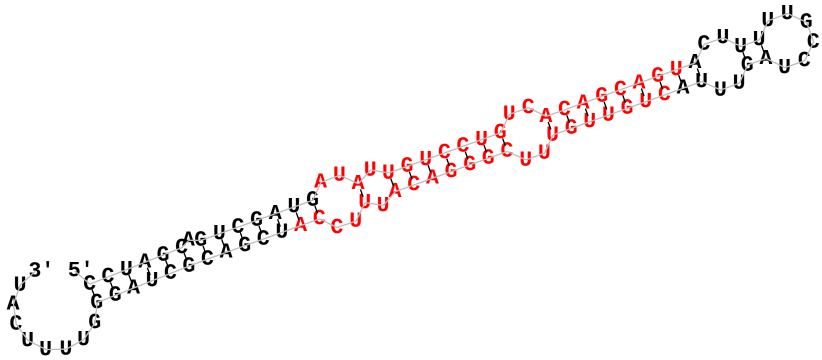

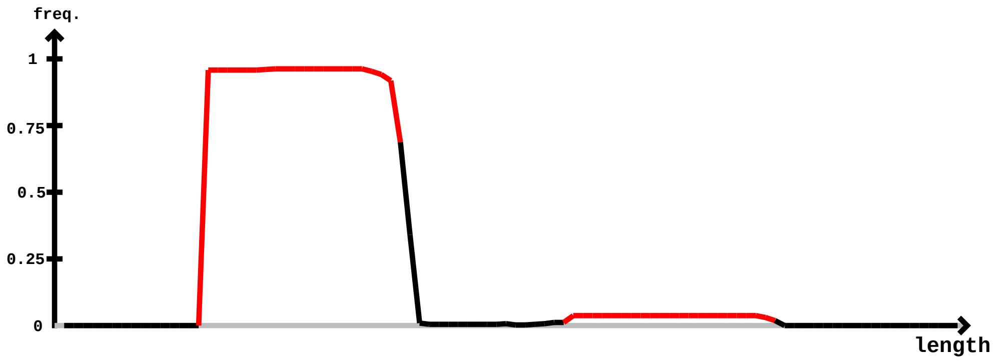


```
                                   aga-miR-1000
                                                                 aga-miR-1000*
5'- ccuagcagucgaugauauuguccugucacagcaguacuuuuugccuaguuuacuguuguuucgggacauuuccaucgacgcuagguuuucau   -3'    exp
    ((((((.(((((((..(.(((((((..(((((((((..((......))..)))))))))..))))))).)..))))))))))))).......    reads      mm      sample
    ..............auauuguccugucacagcagu.........................................................    2          0       OV2

    ..............auauuguccugucacagcag..........................................................    4          0       FF2
    ..............auauuguccugucacagcagu.........................................................    4          0       FF2
    ..............auauuguccugucacagcagua........................................................    1          0       FF2

    ..............auauuguccugucacagca...........................................................    3          0       TE1
    ..............auauuguccugucacagcag..........................................................    5          0       TE1
    ..............auauuguccugucacagUag..........................................................    1          1       TE1
    ..............auauuguccugucacagcagu.........................................................    11         0       TE1
    ..............auauuguccugucacagcagua........................................................    6          0       TE1
    .............................................uaguuuacuguuguuucgggacauuuc....................    1          0       TE1
    ....................................................cuguuguuucgggacauuucc...................    1          0       TE1

    ..............auauuguccugucacagca...........................................................    1          0       MF2
    ..............auauuguccugucacagcag..........................................................    28         0       MF2
    ..............auauuguccugucacagcagu.........................................................    29         0       MF2
    ..............auauuguccugucacagcagua........................................................    15         0       MF2
    ..............auauuguccugucacagcaguaU.......................................................    1          1       MF2
    ................................................uuuacuguuguuucgggacauuucc...................    1          0       MF2
    ..................................................uacuguuguuucgggacauuuc....................    1          0       MF2
    ....................................................cuguuguuucgggacauuucc...................    1          0       MF2
    ....................................................cuguuguuucgggacauuucca..................    7          0       MF2

    ..............auauuguccugucacag.............................................................    1          0       FW2
    ..............auauuguccugucacagc............................................................    1          0       FW2
    ..............auauuguccugucacagca...........................................................    1          0       FW2
    ..............auauuguccugucacagcag..........................................................    7          0       FW2
    ..............auauuguccugucacagcagu.........................................................    25         0       FW2
    ..............auauuguccugucacagcagua........................................................    25         0       FW2
    ....................uccugucacagcaguacuuuuugccu..............................................    1          0       FW2
    .....................ccugucacagcaguacuuuuugccu..............................................    1          0       FW2
    ....................................................cuguuguuucgggacauuucc...................    1          0       FW2

    ..............auauuguccugucacagcag..........................................................    8          0       MF1
```

```
              aga-miR-1000
                                                  aga-miR-1000*
ccuagcagucgaugauauuguccugucacagcaguacuuuuugccuaguuuacuguuguuucgggacauuuccaucgacgcuaggguuuucau

..............auauuguccugucacagcagu..........................................................    6     0     MF1
..............auauuguccugucacagcagua.........................................................    4     0     MF1
....................................................uacuguuguuucgggacauuuc.....................    1     0     MF1

..............auauuguccugucacag..............................................................    1     0     MW1
..............auauuguccugucacagc.............................................................    2     0     MW1
..............auauuguccugucacagca............................................................    1     0     MW1
..............auauuguccugucacagcag...........................................................    8     0     MW1
..............auauuguccugGcacagcagu..........................................................    1     1     MW1
..............auauuguccugucacagcagu..........................................................    29    0     MW1
..............auauuguccugucacagcagua.........................................................    46    0     MW1

..............auauuguccugucacagc.............................................................    1     0     FW1
..............auauuguccugucacagcGg...........................................................    1     1     FW1
..............auauuguccugucacagcag...........................................................    7     0     FW1
..............auauuguccugucacagcagu..........................................................    9     0     FW1
..............auauuguccugucacagcagua.........................................................    13    0     FW1
..............auauuguccugucacagcaguU.........................................................    1     1     FW1
.................................................uuacuguuguuucgggacauuucc....................    1     0     FW1

..............auauuguccugucacagcag...........................................................    2     0     MW2
..............auauuguccugucacagcagu..........................................................    7     0     MW2
..............auauuguccugucacagcagua.........................................................    10    0     MW2

..............auauuguccugucacag..............................................................    2     0     TE2
..............auauuguccugucacagc.............................................................    1     0     TE2
..............auauuguccugucacagca............................................................    4     0     TE2
..............auauuguccugucacagcag...........................................................    28    0     TE2
..............auauuguccugucacagcagu..........................................................    25    0     TE2
..............auauuguccugucacagcagG..........................................................    1     1     TE2
..............auauugucUugucacagcagua.........................................................    1     1     TE2
..............auauuguccugucacagcagua.........................................................    20    0     TE2
..............auauuguccugucacagcaguac........................................................    1     0     TE2
....................................................cuguuguuucgggacauuucca...................    1     0     TE2
```

miRBase precursor : aga-mir-100
Total read count : 56924
aga-miR-100 read count : 52997
aga-miR-100* read count : 3820
remaining reads : 107

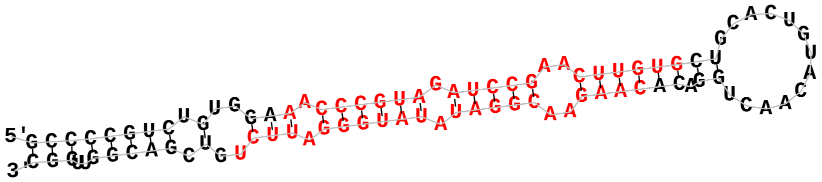

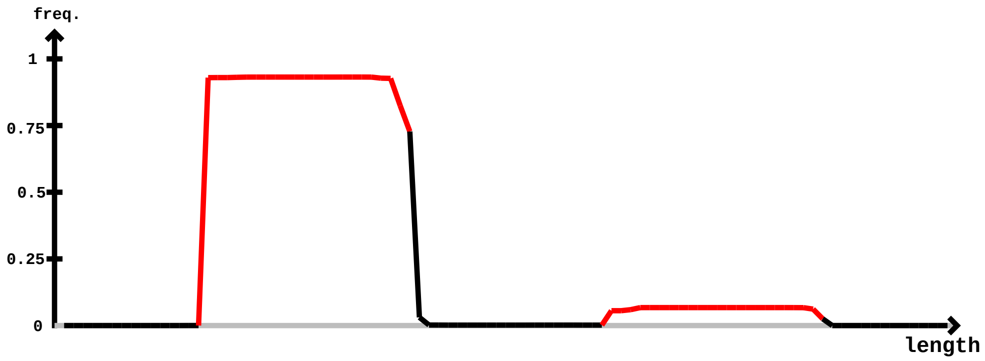


```
                    aga-miR-100
                                                   aga-miR-100*
5'- gccccgucuguggaaacccguagauccgaacuugugcugcacuguacaacuggacacaagaacggauauaugggauucugucgacgguuggc  -3'    exp
    ((((((((.(..(((.((((((.(((((..((((((((............)).))))))..))))).))))))).)))..).)))))..)))    reads      mm         sample
    ..............aacccguagauccgaacu............................................................    5          0          FF2
    ..............aacccguagauccgaacuug..........................................................    94         0          FF2
    ..............aacccguagauccgaacuugu.........................................................    47         0          FF2
    ..............aacccguagauccgaacuGgug........................................................    1          1          FF2
    ..............aacccguagaucUgaacuugug........................................................    1          1          FF2
    ..............aacccguagauccgaacuugug........................................................    582        0          FF2
    ..............aacccgCagauccgaacuugug........................................................    1          1          FF2
    ..............aacccguagauccgaacuCgug........................................................    1          1          FF2
    ..............aacccguagauAcgaacuugug........................................................    1          1          FF2
    ..............Uacccguagauccgaacuugug........................................................    1          1          FF2
    ..............aacccguagauGcgaacuugug........................................................    1          1          FF2
    ..............aacccguagauccgaacuugugA.......................................................    9          1          FF2
    ..............aacccguagauccgaacuugugc.......................................................    1          0          FF2
    ..............aacccguagauccgaacuugugU.......................................................    1          1          FF2
    ..............aacccguagauccgaacuugugG.......................................................    2          1          FF2
    ...............acccguagauccgaacuugug........................................................    1          0          FF2
    .................ccguagauccgaacuugug........................................................    1          0          FF2
    ...................................gcugcacuguacaacuggacacaagaacggaua........................    1          0          FF2
    ....................................cugcacuguacaacuggaca....................................    1          0          FF2
    ......................................gcacuguacaacuggacacaagaacggauauaugggauucu.............    2          0          FF2
    ........................................................caagaacggauauauggga.................    1          0          FF2
    ........................................................caagaacggauauaugggauu...............    5          0          FF2
    ........................................................caagaacggauauaugggauuc..............    39         0          FF2
    ........................................................caagaacggauauaCgggauucu.............    1          1          FF2
    ........................................................caagaacggauauaugggauucu.............    38         0          FF2
    ..........................................................agaacggauauaugggauuc..............    1          0          FF2
    ...........................................................gaacggauauaugggauuc..............    1          0          FF2

    ..............aacccguagauccgaac.............................................................    3          0          OV2
    ..............aacccguagauccgaacu............................................................    24         0          OV2
    ..............aacccguagauccgaacuu...........................................................    6          0          OV2
    ..............aacccguagauccAaacuug..........................................................    1          1          OV2
    ..............aacccguagauAcgaacuug..........................................................    1          1          OV2
    ..............aacccguagauccgaacuug..........................................................    637        0          OV2
```

```
              aga-miR-100
                                                        aga-miR-100*
gccccgucuguggaaacccguagauccgaacuugugcugcacuguacaacuggacacaagaacggauauaugggauucugucgacgguuggc

..............aacccAuagauccgaacuug..........................................................    2     1    OV2
..............aacccguagauccgaacuuA..........................................................    1     1    OV2
..............aacccguagauccAaacuugu.........................................................    2     1    OV2
..............aacccAuagauccgaacuugu.........................................................    2     1    OV2
..............aacccguagauGcgaacuugu.........................................................    1     1    OV2
..............aacccguagauccgaacuuAu.........................................................    2     1    OV2
..............aacccguagauAcgaacuugu.........................................................    1     1    OV2
..............aacccguagauccgaacuugG.........................................................    2     1    OV2
..............aacccguagaGccgaacuugu.........................................................    1     1    OV2
..............aacccguagauUcgaacuugu.........................................................    1     1    OV2
..............aacccguagauccgaacCugu.........................................................    1     1    OV2
..............aacccguagauccgaacuugC.........................................................    1     1    OV2
..............aaccUguagauccgaacuugu.........................................................    6     1    OV2
..............aacccgCagauccgaacuugu.........................................................    1     1    OV2
..............aacccguagauccgaacuugu.........................................................    1381  0    OV2
..............aacccguagaucUgaacuugu.........................................................    2     1    OV2
..............aacccguagaAccgaacuugug........................................................    1     1    OV2
..............aaccUguagauccgaacuugug........................................................    6     1    OV2
..............aacccguagaGccgaacuugug........................................................    2     1    OV2
..............aacccguagaucUgaacuugug........................................................    4     1    OV2
..............aacccguagauccgaacuCgug........................................................    1     1    OV2
..............aacccguaAauccgaacuugug........................................................    1     1    OV2
..............aacccguagauccgaacuuguA........................................................    16    1    OV2
..............aacccguGgauccgaacuugug........................................................    1     1    OV2
..............aacccguagauccgaacuugug........................................................    3495  0    OV2
..............aaUccguagauccgaacuugug........................................................    1     1    OV2
..............aacccgCagauccgaacuugug........................................................    1     1    OV2
..............aacUcguagauccgaacuugug........................................................    1     1    OV2
..............aacccguagauccgGacuugug........................................................    1     1    OV2
..............aacccguagauccgaacuuAug........................................................    1     1    OV2
..............Gacccguagauccgaacuugug........................................................    1     1    OV2
..............aacccguagaCccgaacuugug........................................................    2     1    OV2
..............aacccguagauccAaacuugug........................................................    1     1    OV2
..............aacccguagauUcgaacuugug........................................................    1     1    OV2
..............aacccguagauccgaacCugug........................................................    2     1    OV2
..............aacccguagauccgaacuugAg........................................................    1     1    OV2
..............aacccguagauccgaacuugCg........................................................    2     1    OV2
..............aacccUuagauccgaacuugug........................................................    1     1    OV2
..............aacccguagauccgaacuuguU........................................................    2     1    OV2
..............aacccguagauAcgaacuugug........................................................    2     1    OV2
..............aacccguagauccgaacuugugG.......................................................    22    1    OV2
..............aacccguagauccgaacuugugA.......................................................    360   1    OV2
..............aacccguagauccgaacuugugU.......................................................    19    1    OV2
..............aacccguagauccgaacuugugc.......................................................    11    0    OV2
..............aacccguagauccgaacuugugcA......................................................    2     1    OV2
..............aacccguagauccgaacuugugAu......................................................    1     1    OV2
..............aacccguagauccgaacuugugcug.....................................................    1     0    OV2
..............aacccguagauccgaacuugugcugcacuguacaacuggaA.....................................    1     1    OV2
...............acccguagauccgaacuug..........................................................    1     0    OV2
...............acccguagauccgaacuugug........................................................    1     0    OV2
.................ccguagauccgaacuugu.........................................................    2     0    OV2
.................ccguagauccgaacuugug........................................................    3     0    OV2
..................cguagauccgaacuugu.........................................................    1     0    OV2
..................cguagauccgaacuugug........................................................    1     0    OV2
....................................cugcacuguacaacuggaca....................................    1     0    OV2
........................................................caagaacggauauaugggau................    2     0    OV2
........................................................caagaacggauauaugggauu...............    32    0    OV2
........................................................caagaacggauauaugggauuU..............    4     1    OV2
........................................................Uaagaacggauauaugggauuc..............    1     1    OV2
........................................................caagaacggauauaugggauuc..............    74    0    OV2
........................................................Aaagaacggauauaugggauucu.............    1     1    OV2
........................................................caagaacggauauaugggauucC.............    1     1    OV2
........................................................caagaacggauauaugggauucu.............    102   0    OV2
........................................................caagaacggauauaugggauucuA............    1     1    OV2
..........................................................agaacggauauaugggauuc..............    8     0    OV2
...........................................................gaacggauauaugggauuc..............    8     0    OV2

.............Caacccguagauccgaacuugug........................................................    1     1    TE1
..............aacccguagauccgaacu............................................................    3     0    TE1
```

```
              aga-miR-100
                                                          aga-miR-100*
gccccgucuguggaaacccguagauccgaacuugugcugcacuguacaacuggacacaagaacggauauaugggauucugucgacgguuggc

..............aacccguagauccgaacuu...........................................................    7      0    TE1
..............aacccguagGuccgaacuug..........................................................    1      1    TE1
..............aacccguagauGcgaacuug..........................................................    1      1    TE1
..............aaUccguagauccgaacuug..........................................................    1      1    TE1
..............aCcccguagauccgaacuug..........................................................    1      1    TE1
..............aacccguagauccgaacuug..........................................................    219    0    TE1
..............aacccguagauUcgaacuugu.........................................................    1      1    TE1
..............aacccguagauccgaaUuugu.........................................................    1      1    TE1
..............aacccguagauccgaacuugu.........................................................    133    0    TE1
..............aacUcguagauccgaacuugu.........................................................    1      1    TE1
..............aacccguagauccgaacuuguU........................................................    1      1    TE1
..............aacccguagauccgaacuuguA........................................................    2      1    TE1
..............Uacccguagauccgaacuugug........................................................    1      1    TE1
..............aaccUguagauccgaacuugug........................................................    1      1    TE1
..............aacccguagauccgaGcuugug........................................................    1      1    TE1
..............aacccguagauUcgaacuugug........................................................    3      1    TE1
..............aacccguagauccgaacuugGg........................................................    1      1    TE1
..............aacccguagauAcgaacuugug........................................................    1      1    TE1
..............aacccguagaCccgaacuugug........................................................    1      1    TE1
..............aacccguagauccgaacuugCg........................................................    1      1    TE1
..............aacccAuagauccgaacuugug........................................................    2      1    TE1
..............aacUcguagauccgaacuugug........................................................    1      1    TE1
..............aacccguagauccgaacuCgug........................................................    1      1    TE1
..............aacccguagauccgaacuugug........................................................    1152   0    TE1
..............aaAccguagauccgaacuugug........................................................    1      1    TE1
..............aacccguagauccgGacuugug........................................................    1      1    TE1
..............aacccgCagauccgaacuugug........................................................    1      1    TE1
..............aacccguagauccgaacuugugG.......................................................    3      1    TE1
..............aacccguagauccgaacuugugU.......................................................    2      1    TE1
..............aacccguagauccgaacuugugA.......................................................    43     1    TE1
..............aacccguagauccgaacuugugcug.....................................................    1      0    TE1
..............aacccguagauccgaacuugugcugc....................................................    1      0    TE1
..............aacccguagauccgaacuugugcugcacug................................................    1      0    TE1
..............aacccguagauccgaacuugugcugcacuguacaacugg.......................................    2      0    TE1
...............acccguagauccgaacuugug........................................................    1      0    TE1
..................cguagauccgaacuugu.........................................................    1      0    TE1
..................cguagauccgaacuugug........................................................    1      0    TE1
............................aacuugugcugcacuguacaacuggacacaagaacggauauaugggauuc..............    1      0    TE1
........................................................caagaacggauauauggga.................    1      0    TE1
........................................................caagaacggauauaugggau................    1      0    TE1
........................................................caagaacggauauaugggauu...............    23     0    TE1
........................................................caagaacggauauaugggauuc..............    80     0    TE1
........................................................caagaacggauauaugggauucC.............    1      1    TE1
........................................................caagaacggauauaugggauucu.............    32     0    TE1
........................................................caagaacggauauaugggaCucu.............    1      1    TE1
........................................................caagaacggauauauggUauucu.............    1      1    TE1
........................................................caagaacggauGuaugggauucu.............    1      1    TE1
..........................................................agaacggauauaugggauuc..............    15     0    TE1
..........................................................Cgaacggauauaugggauuc..............    1      1    TE1
...........................................................gaacggauauaugggauuc..............    53     0    TE1

.............aaacccguagauccgaacuugug........................................................    1      0    MF2
..............aacccguagauccgaacu............................................................    4      0    MF2
..............aacccguaCauccgaacuug..........................................................    1      1    MF2
..............aacccguagauUcgaacuug..........................................................    1      1    MF2
..............aacccguagauccgaacuug..........................................................    416    0    MF2
..............aacccguagauccAaacuug..........................................................    1      1    MF2
..............aacccguagauccgaacuGg..........................................................    1      1    MF2
..............aacccguagauccgaacuugu.........................................................    212    0    MF2
..............aacccguagaCccgaacuugu.........................................................    1      1    MF2
..............aacccguagaucUgaacuugu.........................................................    1      1    MF2
..............aaccUguagauccgaacuugu.........................................................    2      1    MF2
..............aacccguagauccgaCcuugu.........................................................    1      1    MF2
..............aaccUguagauccgaacuugug........................................................    3      1    MF2
..............aacccguagauccgaacuCgug........................................................    3      1    MF2
..............aacccguagaucUgaacuugug........................................................    2      1    MF2
..............aacccguagaCccgaacuugug........................................................    2      1    MF2
..............aacccguagauccgaacuugGg........................................................    1      1    MF2
..............aacccguagauccgaacuuguU........................................................    1      1    MF2
```

```
              aga-miR-100
                                                        aga-miR-100*
gccccgucuguggaaacccguagauccgaacuugugcugcacuguacaacuggacacaagaacggauauaugggauucugucgacgguuggc

..............aacccguagauccgaacuuAug........................................................ 1     1  MF2
..............Gacccguagauccgaacuugug........................................................ 1     1  MF2
..............aacccguagaucAgaacuugug........................................................ 1     1  MF2
..............aacccguagauccgaacuuguA........................................................ 4     1  MF2
..............aacccguagauccAaacuugug........................................................ 1     1  MF2
..............aacccguGgauccgaacuugug........................................................ 1     1  MF2
..............aacccguagauccUaacuugug........................................................ 1     1  MF2
..............aacccguagauccgaaAuugug........................................................ 1     1  MF2
..............aacccAuagauccgaacuugug........................................................ 2     1  MF2
..............aacccguagauccgaaGuugug........................................................ 1     1  MF2
..............aacccguagauccgaacuugug........................................................ 2661  0  MF2
..............aacccguagauccgaGcuugug........................................................ 1     1  MF2
..............aacccgCagauccgaacuugug........................................................ 3     1  MF2
..............aacccguagauUcgaacuugug........................................................ 2     1  MF2
..............aacccguagauccgaacuugugG....................................................... 11    1  MF2
..............aacccguagauccgaacuugugU....................................................... 5     1  MF2
..............aacccguagauccgaacuugugA....................................................... 66    1  MF2
..............aacccguagauccgaacuugugc....................................................... 4     0  MF2
..............aacccguagauccgaacuugugAu...................................................... 1     1  MF2
..............aacccguagauccgaacuugugcug..................................................... 1     0  MF2
..............aacccguagauccgaacuugugcugcGcug................................................ 1     1  MF2
..............aacccguagauccgaacuugugcugcacuguacaacugg....................................... 1     0  MF2
..............aacccguagauccgaacuugugcugcacuguacaacuggaA.................................... 2     1  MF2
..............aacccguagauccgaacuugugcugcacuguacaacuggaca.................................... 5     0  MF2
.................ccguagauccgaacuugug........................................................ 1     0  MF2
..................cguagauccgaacuugug........................................................ 1     0  MF2
...................guagauccgaacuugug........................................................ 1     0  MF2
....................................cugcacuguacaacuggaca.................................... 4     0  MF2
....................................cugcacuguacaacuggacacaagaacggauauaugggauuc.............. 1     0  MF2
................................................acuggacacaagaacggauauaugggauuc.............. 2     0  MF2
........................................................caagaacggauauaugggauu............... 27    0  MF2
........................................................caagaacggauauaugggauuc.............. 182   0  MF2
........................................................caagaacggauauaugggauuGu............. 1     1  MF2
........................................................caagaacggauauaugAgauucu............. 1     1  MF2
........................................................caagaaUggauauaugggauucu............. 1     1  MF2
........................................................caagaacggauauaugggauucu............. 254   0  MF2
..........................................................agaacggauauaugggauu............... 1     0  MF2
..........................................................agaacggauauaugggauuc.............. 19    0  MF2
...........................................................gaacggauauaugggauu............... 1     0  MF2
...........................................................gaacggauauaugggauuc.............. 26    0  MF2
.............................................................acggauauaCgggauuc.............. 1     1  MF2

...........ggGaacccguagauccgaacuugug........................................................ 1     1  FW2
.............Caacccguagauccgaacuugug........................................................ 1     1  FW2
..............aacccguagauAcgaacu............................................................ 1     1  FW2
..............aacccguagauccgaacu............................................................ 37    0  FW2
..............aacccguagauccgaacuu........................................................... 5     0  FW2
..............aacccguagaGccgaacuug.......................................................... 1     1  FW2
..............aacccUuagauccgaacuug.......................................................... 1     1  FW2
..............aacccguagauUcgaacuug.......................................................... 1     1  FW2
..............aaccUguagauccgaacuug.......................................................... 1     1  FW2
..............aacccguagauccgaaUuug.......................................................... 1     1  FW2
..............aacccguagauccgaacuug.......................................................... 775   0  FW2
..............aacccguagauccgaacuCgu......................................................... 1     1  FW2
..............aacccguagauccgaacuugA......................................................... 1     1  FW2
..............aacccguagauccgaGcuugu......................................................... 1     1  FW2
..............aacccguagauccgaacuugG......................................................... 2     1  FW2
..............aacccguagauccgaacuugu......................................................... 418   0  FW2
..............aacccgGagauccgaacuugug........................................................ 2     1  FW2
..............aacccguagauAcgaacuugug........................................................ 2     1  FW2
..............aaccUguagauccgaacuugug........................................................ 1     1  FW2
..............aacccguGgauccgaacuugug........................................................ 1     1  FW2
..............aacccguagaucAgaacuugug........................................................ 1     1  FW2
..............aacccguagauccgaacuugug........................................................ 7084  0  FW2
..............aacccgCagauccgaacuugug........................................................ 3     1  FW2
..............aacccguagauccgaacCugug........................................................ 3     1  FW2
..............aacccguagauccgaacuugGg........................................................ 4     1  FW2
..............aacccguagauUcgaacuugug........................................................ 6     1  FW2
..............aacccguagauccgaacuugAg........................................................ 2     1  FW2
```

```
              aga-miR-100
                                                        aga-miR-100*
gccccgucuguggaaacccguagauccgaacuugugcugcacuguacaacuggacacaagaacggauauaugggauucugucgacgguuggc

..............aacccguagauccgaacuuguA........................................................    7     1    FW2
..............aacGcguagauccgaacuugug........................................................    1     1    FW2
..............aacccguagGuccgaacuugug........................................................    1     1    FW2
..............aaGccguagauccgaacuugug........................................................    1     1    FW2
..............aacccguagauccAaacuugug........................................................    2     1    FW2
..............aacccguagauccgaacuugCg........................................................    4     1    FW2
..............aacccguagauccCaacuugug........................................................    1     1    FW2
..............aacccguagaCccgaacuugug........................................................    4     1    FW2
..............aacccguaAauccgaacuugug........................................................    1     1    FW2
..............aacccguagauccgGacuugug........................................................    1     1    FW2
..............aacccCuagauccgaacuugug........................................................    1     1    FW2
..............aacccguagauccgaacuuAug........................................................    2     1    FW2
..............aacccguagaGccgaacuugug........................................................    1     1    FW2
..............aacccAuagauccgaacuugug........................................................    1     1    FW2
..............aacUcguagauccgaacuugug........................................................    2     1    FW2
..............aacccUuagauccgaacuugug........................................................    1     1    FW2
..............aacccguagauccgaacuCgug........................................................    2     1    FW2
..............aacccguagauccgaaAuugug........................................................    1     1    FW2
..............aacccguagaucUgaacuugug........................................................    1     1    FW2
..............aacccguagauccgaacuugugG.......................................................    10    1    FW2
..............aacccguagauccgaacuugugA.......................................................    62    1    FW2
..............aacccguagauccgaacuugugU.......................................................    6     1    FW2
..............aacccguagauccgaacuugugc.......................................................    6     0    FW2
..............aacccguagauccgaacuugugcA......................................................    1     1    FW2
..............aacccguagauccgaacuugugcugcacuguacaacuggaca....................................    2     0    FW2
..............aacccguagauccgaacuugugcugcacuguacaacuggacacaa.................................    1     0    FW2
...............acccguagauccgaacuugug........................................................    2     0    FW2
...............aAccguagauccgaacuugug........................................................    1     1    FW2
.................ccguagauccgaacuugug........................................................    5     0    FW2
..................cguagauccgaacuugug........................................................    3     0    FW2
...................guagauccgaacuugug........................................................    1     0    FW2
....................................cugcacuguacaacuggaca....................................    7     0    FW2
....................................cugcacuguacaacuggacacaagaacggauauaugggauuc..............    1     0    FW2
..........................................uguacaacuggacacaagaacggauauaugggauuc..............    1     0    FW2
...............................................aacuggacacaagaacggauauaugggauuc..............    1     0    FW2
.......................................................Ccaagaacggauauaugggauu...............    1     1    FW2
........................................................caagaacggauauaugggauu...............    17    0    FW2
........................................................caagaacggauauaugggauuc..............    142   0    FW2
........................................................caagaacggauauaugggauucu.............    97    0    FW2
..........................................................agaacggauauaugggauuc..............    14    0    FW2
...........................................................gaacggauauaugggauuc..............    16    0    FW2

..............aacccguagauccgaacu............................................................    2     0    FF1
..............aacccguagauccgaacuu...........................................................    1     0    FF1
..............aacccguagauccgaacuug..........................................................    178   0    FF1
..............aacccguagauccgaacuAg..........................................................    1     1    FF1
..............aacccguagauccgaacuugG.........................................................    1     1    FF1
..............aacccguagauGcgaacuugu.........................................................    1     1    FF1
..............aacccguagauccgaacuuAu.........................................................    1     1    FF1
..............aacccguagauccgaacuugu.........................................................    281   0    FF1
..............aacccguagaucUgaacuugu.........................................................    1     1    FF1
..............aacccguagauccgaUcuugu.........................................................    1     1    FF1
..............aacccguagauccgaGcuugug........................................................    1     1    FF1
..............aaUccguagauccgaacuugug........................................................    1     1    FF1
..............aacccguagauccgaacuuguU........................................................    1     1    FF1
..............Gacccguagauccgaacuugug........................................................    1     1    FF1
..............aacccguagaucUgaacuugug........................................................    1     1    FF1
..............aacccguagauccgaacuuguA........................................................    4     1    FF1
..............aacccguagauGcgaacuugug........................................................    1     1    FF1
..............aacccguagauccgaacuugGg........................................................    3     1    FF1
..............aacccguagauccgaacuCgug........................................................    2     1    FF1
..............aacccguagaCccgaacuugug........................................................    1     1    FF1
..............aacccguagauccgaacCugug........................................................    1     1    FF1
..............aacccguagauccAaacuugug........................................................    1     1    FF1
..............aaccUguagauccgaacuugug........................................................    1     1    FF1
..............aacccguagUuccgaacuugug........................................................    1     1    FF1
..............aacccguagauccgaacuugug........................................................    1660  0    FF1
..............aacccguagauccgUacuugug........................................................    1     1    FF1
..............aacccguGgauccgaacuugug........................................................    2     1    FF1
```

```
              aga-miR-100
                                                                                aga-miR-100*
gccccgucuguggaaacccguagauccgaacuugugcugcacuguacaacuggacacaagaacggauauaugggauucugucgacgguuggc

..............aacccguagaAccgaacuugug........................................................   1      1      FF1
..............aacccguaAauccgaacuugug........................................................   1      1      FF1
..............aacccguagauccgaacuugugU.......................................................   4      1      FF1
..............aacccguagauccgaacuugugA.......................................................   104    1      FF1
..............aacccguagauccgaacuugugc.......................................................   6      0      FF1
..............aacccguagauccgaacuugugG.......................................................   17     1      FF1
..............aacccguagauccgaacuugugAu......................................................   1      1      FF1
..............aacccguagauccgaacuugugcug.....................................................   1      0      FF1
..............aacccguagauccgaacuugugcugcacuguacaacugg.......................................   1      0      FF1
..............aacccguagauccgaacuugugcugcacuguacaacuggaca....................................   5      0      FF1
..............aacccguagauccgaacuugugcugcacuguacaaAuggaca....................................   1      1      FF1
..............aacccguagauccgaacuugugcugcacuguacaacuggacaAa..................................   1      1      FF1
...............acccguagauccgaacuugug........................................................   1      0      FF1
.................ccguagauccgaacuugug........................................................   2      0      FF1
..................cguagauccgaacuugu.........................................................   1      0      FF1
..................cguagauccgaacuugugA.......................................................   1      1      FF1
........................................................caagaacggauauaugggauu...............   12     0      FF1
........................................................caagaacggauauaugggauuc..............   35     0      FF1
........................................................caagaacggaAauaugggauuc..............   1      1      FF1
........................................................caagaacggauauaugUgauucu.............   1      1      FF1
........................................................caagaacggauauaugggauucu.............   69     0      FF1
..........................................................agaacggauauaugggauuc..............   6      0      FF1
..........................................................agaacggauauaugggauucu.............   1      0      FF1
...........................................................gaacggauauaugggauuc..............   3      0      FF1

.............Caacccguagauccgaacuug..........................................................   1      1      OV1
..............aacccguagauccgaac.............................................................   2      0      OV1
..............aacccguagauccgaacu............................................................   30     0      OV1
..............aacUcguagauccgaacu............................................................   1      1      OV1
..............aacccguagauccgaacuu...........................................................   7      0      OV1
..............aacccguagauccgaacuuU..........................................................   1      1      OV1
..............aacccguaAauccgaacuug..........................................................   1      1      OV1
..............aacccguagauccgaacuug..........................................................   893    0      OV1
..............aacGcguagauccgaacuug..........................................................   1      1      OV1
..............aacccguagauUcgaacuug..........................................................   1      1      OV1
..............aacccguagauccAaacuug..........................................................   1      1      OV1
..............aacccguagauccgaacuCg..........................................................   1      1      OV1
..............aacccguagauccgaacCug..........................................................   1      1      OV1
..............aacccguagauGcgaacuug..........................................................   1      1      OV1
..............aacccUuagauccgaacuugu.........................................................   1      1      OV1
..............aacccguagauccgaacuugC.........................................................   2      1      OV1
..............aacccguagauGcgaacuugu.........................................................   1      1      OV1
..............aacccguagauccgaacuugu.........................................................   1184   0      OV1
..............aacccguagauccAaacuugu.........................................................   1      1      OV1
..............aacccguagaucAgaacuugu.........................................................   1      1      OV1
..............aacccguagauccgaacuuAu.........................................................   4      1      OV1
..............aaccUguagauccgaacuugu.........................................................   2      1      OV1
..............aacUcguagauccgaacuugu.........................................................   1      1      OV1
..............aacccguagauAcgaacuugu.........................................................   1      1      OV1
..............aacccguagaAccgaacuugu.........................................................   1      1      OV1
..............aacccguagauccgGacuugu.........................................................   1      1      OV1
..............aacccguagauccgaUcuugu.........................................................   1      1      OV1
..............aacccguagaucUgaacuugu.........................................................   4      1      OV1
..............aacccguagauUcgaacuugu.........................................................   1      1      OV1
..............aacccAuagauccgaacuugug........................................................   2      1      OV1
..............aacccguagauccgaacuuAug........................................................   2      1      OV1
..............aacccguagauAcgaacuugug........................................................   4      1      OV1
..............aacccguagaAccgaacuugug........................................................   1      1      OV1
..............aacccguagauccgaacuuUug........................................................   1      1      OV1
..............aacccguagauccgaacuugug........................................................   3588   0      OV1
..............aacccguagauUcgaacuugug........................................................   3      1      OV1
..............aacccguagauccCaacuugug........................................................   1      1      OV1
..............aacccgCagauccgaacuugug........................................................   1      1      OV1
..............aacccguagaucUgaacuugug........................................................   1      1      OV1
..............aacccguagauccgaacuuguA........................................................   10     1      OV1
..............aacccguagaGccgaacuugug........................................................   1      1      OV1
..............aacccguagauccgaacuugCg........................................................   1      1      OV1
..............aacccguagauccgaacuCgug........................................................   1      1      OV1
..............aacccguagauccgaacuuguU........................................................   3      1      OV1
```

```
              aga-miR-100
                                                        aga-miR-100*
gccccgucuguggaaacccguagauccgaacuugugcugcacuguacaacuggacacaagaacggauauaugggauucugucgacgguuggc

..............aacccguaAauccgaacuugug........................................................    1     1    OV1
..............aacccguagaCccgaacuugug........................................................    1     1    OV1
..............aaUccguagauccgaacuugug........................................................    2     1    OV1
..............aacccguaUauccgaacuugug........................................................    1     1    OV1
..............aaccUguagauccgaacuugug........................................................    2     1    OV1
..............aacccguagauccAaacuugug........................................................    3     1    OV1
..............Uacccguagauccgaacuugug........................................................    2     1    OV1
..............aacccguagauccgaacuugugc.......................................................    8     0    OV1
..............aacccguagauccgaacuugugG.......................................................    29    1    OV1
..............aacccguagauccgaacuugugU.......................................................    14    1    OV1
..............aacccguagauccgaacuugugA.......................................................    359   1    OV1
..............aacccguagauccgaacuugugAu......................................................    2     1    OV1
..............aacccguagauccgaacuugugcug.....................................................    1     0    OV1
..............aacccguagauccgaacuugugcugcacuguacaacuggaca....................................    2     0    OV1
..............aacccguagauccgaacuugugcugcacuguacaacuggacaU...................................    1     1    OV1
...............acccgGagauccgaacuugug........................................................    1     1    OV1
.................ccguagauccgaacuugu.........................................................    3     0    OV1
.................ccguagauccgaacuugug........................................................    2     0    OV1
.................ccguagauccgaacuugugA.......................................................    2     1    OV1
..................cguagauccgaacuugu.........................................................    4     0    OV1
..................cguagauccgaacuugug........................................................    1     0    OV1
........................................................caagaacggauauauggga.................    2     0    OV1
........................................................caagaacggauauaugggau................    4     0    OV1
........................................................caagaacggauauaugggauu...............    64    0    OV1
........................................................caagaacggaAauaugggauuc..............    1     1    OV1
........................................................caagaacggGuauaugggauuc..............    1     1    OV1
........................................................caagaacggauauaugggauuc..............    107   0    OV1
........................................................caagaacggauauaugggauuGu.............    1     1    OV1
........................................................caagaacggauauaGgggauucu.............    1     1    OV1
........................................................caagaacggauauaugggauucu.............    136   0    OV1
........................................................caagaacggauauauggAauucu.............    1     1    OV1
........................................................caagaacUgauauaugggauucu.............    1     1    OV1
........................................................caagaacggauauaugggauucA.............    1     1    OV1
..........................................................agaacggauauaugggauuc..............    7     0    OV1
...........................................................gaacggauauaugggauuc..............    21    0    OV1

..............aacccguagauccgaacu............................................................    5     0    MF1
..............aacccguagauccgaacuug..........................................................    136   0    MF1
..............Cacccguagauccgaacuug..........................................................    2     1    MF1
..............aacccguagauccgaacuugu.........................................................    69    0    MF1
..............aacccguagauccgaacuCgu.........................................................    1     1    MF1
..............aacccAuagauccgaacuugug........................................................    2     1    MF1
..............aacccguagauUcgaacuugug........................................................    2     1    MF1
..............Uacccguagauccgaacuugug........................................................    1     1    MF1
..............aacccguagauccgaacuuguU........................................................    1     1    MF1
..............aacccguagauccgaacuuAug........................................................    1     1    MF1
..............aacccguagauccgaacuugug........................................................    688   0    MF1
..............aaccAguagauccgaacuugug........................................................    1     1    MF1
..............aacccguagauAcgaacuugug........................................................    1     1    MF1
..............aacccguagauccgaacuugugU.......................................................    1     1    MF1
..............aacccguagauccgaacuugugG.......................................................    2     1    MF1
..............aacccguagauccgaacuugugA.......................................................    14    1    MF1
..................cguagauccgaacuugug........................................................    1     0    MF1
...................guagauccgaacuugug........................................................    1     0    MF1
....................................cugcacuguacaacuggaca....................................    1     0    MF1
........................................................caagaacggauauaugggauu...............    2     0    MF1
........................................................Uaagaacggauauaugggauuc..............    1     1    MF1
........................................................caagaacggauauaugggauuc..............    29    0    MF1
........................................................caagaacggauauaugggauuG..............    1     1    MF1
........................................................caagaaAggauauaugggauucu.............    1     1    MF1
........................................................caagaacggauauaugggauucu.............    13    0    MF1
..........................................................agaacggauauaugggauuc..............    4     0    MF1
...........................................................gaacggauauaugggauuc..............    5     0    MF1

..............aacccguagauccgaacu............................................................    7     0    BF2
..............aacccguagauccgaacuu...........................................................    1     0    BF2
..............aacccguaUauccgaacuug..........................................................    1     1    BF2
..............aacccguagauccgaacuug..........................................................    172   0    BF2
..............aacccAuagauccgaacuug..........................................................    1     1    BF2
```

```
              aga-miR-100
                                          aga-miR-100*
gccccgucuguggaaacccguagauccgaacuugugcugcacuguacaacuggacacaagaacggauauaugggauucugucgacgguuggc

..............aacccguagaCccgaacuug..........................................................   1      1    BF2
..............aacccguagauccAaacuugu.........................................................   1      1    BF2
..............aacccguagaucUgaacuugu.........................................................   1      1    BF2
..............aacccguagauccgaacuugu.........................................................   247    0    BF2
..............aaccUguagauccgaacuugu.........................................................   1      1    BF2
..............aaUccguagauccgaacuugu.........................................................   1      1    BF2
..............aacccguagaCccgaacuugug........................................................   1      1    BF2
..............aacccguagauccgaacuugAg........................................................   1      1    BF2
..............aaccUguagauccgaacuugug........................................................   3      1    BF2
..............aacccguagauccAaacuugug........................................................   1      1    BF2
..............aacccAuagauccgaacuugug........................................................   1      1    BF2
..............aacccguagauUcgaacuugug........................................................   4      1    BF2
..............aacccguagaucUgaacuugug........................................................   1      1    BF2
..............aacccguaAauccgaacuugug........................................................   1      1    BF2
..............aacccguagauccgaacuuguA........................................................   1      1    BF2
..............aacccguagUuccgaacuugug........................................................   1      1    BF2
..............aacccguagauccgaacuuAug........................................................   1      1    BF2
..............aacccguagauccgaacuuguU........................................................   2      1    BF2
..............aacccguagauccgaacuugug........................................................   1749   0    BF2
..............aacccguagauccUaacuugug........................................................   2      1    BF2
..............aacUcguagauccgaacuugug........................................................   2      1    BF2
..............aaAccguagauccgaacuugug........................................................   1      1    BF2
..............aacccguagauccgaacCugug........................................................   1      1    BF2
..............aacccCuagauccgaacuugug........................................................   1      1    BF2
..............aacccguagauccgaacuugugU.......................................................   2      1    BF2
..............aacccguagauccgaacuugugc.......................................................   2      0    BF2
..............aacccguagauccgaacuugugA.......................................................   57     1    BF2
..............aacccguagauccgaacuugugG.......................................................   5      1    BF2
..............aacccguagauccgaacuugugcug.....................................................   1      0    BF2
...............acccguagauccgaacuugug........................................................   1      0    BF2
................cccguagauccgaacuugu.........................................................   1      0    BF2
................cccguagauccgaacuugugA.......................................................   1      1    BF2
.................ccguagauccgaacuugu.........................................................   1      0    BF2
.................ccguagauccgaacuugug........................................................   6      0    BF2
..................cguagauccgaacuugu.........................................................   3      0    BF2
..................cguagauccgaacuugug........................................................   13     0    BF2
....................................cugcacuguacaacuggaca....................................   7      0    BF2
....................................cugcacuguacaacuggacacaagaacggauauaugggauuc..............   1      0    BF2
........................................................caagaacggauauaugggauu...............   15     0    BF2
........................................................caagaacggauauaugggauuc..............   110    0    BF2
........................................................caagaaAggauauaugggauuc..............   1      1    BF2
........................................................caagaacggaAauaugggauuc..............   1      1    BF2
........................................................caagaacggaGauaugggauuc..............   1      1    BF2
........................................................Aaagaacggauauaugggauucu.............   1      1    BF2
........................................................caagaacggauauaugggauucC.............   2      1    BF2
........................................................caagaacggauauaugggauucG.............   1      1    BF2
........................................................caagaacggauauaugggauucu.............   207    0    BF2
.........................................................aagaacggauauaugggauuc..............   1      0    BF2
..........................................................agaacggauauaugggauuc..............   28     0    BF2
..........................................................agaacggauauaugggauucu.............   3      0    BF2
...........................................................gaacggauauaugggauuc..............   13     0    BF2
............................................................aacggauauaugggauuc..............   2      0    BF2
.............................................................acggauauaugggauuc..............   1      0    BF2

..............aacccguagauccgaacu............................................................   2      0    BF1
..............aacccguagauccgaacuu...........................................................   1      0    BF1
..............aacccguagauccgaacuug..........................................................   177    0    BF1
..............aacccguagauccgaacuAg..........................................................   1      1    BF1
..............aacccguagauccgaacuuAu.........................................................   1      1    BF1
..............aacccguagauccgaUcuugu.........................................................   1      1    BF1
..............aacccguagauGcgaacuugu.........................................................   1      1    BF1
..............aacccguagauccgaacuugG.........................................................   1      1    BF1
..............aacccguagauccgaacuugu.........................................................   281    0    BF1
..............aacccguagaucUgaacuugu.........................................................   1      1    BF1
..............aacccguaAauccgaacuugug........................................................   1      1    BF1
..............aacccguagauccgaacCugug........................................................   1      1    BF1
..............aacccguagauccgaacuuguU........................................................   1      1    BF1
..............aacccguagauccgaGcuugug........................................................   1      1    BF1
..............aacccguagauGcgaacuugug........................................................   1      1    BF1
```

```
              aga-miR-100
                                                        aga-miR-100*
gccccgucuguggaaacccguagauccgaacuugugcugcacuguacaacuggacacaagaacggauauaugggauucugucgacgguuggc

..............aacccguagauccgaacuugug........................................................  1653  0  BF1
..............aacccguagaAccgaacuugug........................................................  1     1  BF1
..............aacccguagauccAaacuugug........................................................  1     1  BF1
..............aacccguagauccgUacuugug........................................................  1     1  BF1
..............aacccguagUuccgaacuugug........................................................  1     1  BF1
..............aacccguagaucUgaacuugug........................................................  1     1  BF1
..............aaUccguagauccgaacuugug........................................................  1     1  BF1
..............aacccguagauccgaacuugGg........................................................  3     1  BF1
..............Gacccguagauccgaacuugug........................................................  1     1  BF1
..............aacccguagauccgaacuuguA........................................................  4     1  BF1
..............aacccguagaCccgaacuugug........................................................  1     1  BF1
..............aacccguGgauccgaacuugug........................................................  2     1  BF1
..............aacccguagauccgaacuCgug........................................................  2     1  BF1
..............aaccUguagauccgaacuugug........................................................  1     1  BF1
..............aacccguagauccgaacuugugG.......................................................  17    1  BF1
..............aacccguagauccgaacuugugc.......................................................  6     0  BF1
..............aacccguagauccgaacuugugU.......................................................  4     1  BF1
..............aacccguagauccgaacuugugA.......................................................  104   1  BF1
..............aacccguagauccgaacuugugAu......................................................  1     1  BF1
..............aacccguagauccgaacuugugcug.....................................................  1     0  BF1
..............aacccguagauccgaacuugugcugcacuguacaacugg.......................................  1     0  BF1
..............aacccguagauccgaacuugugcugcacuguacaaAuggaca....................................  1     1  BF1
..............aacccguagauccgaacuugugcugcacuguacaacuggaca....................................  5     0  BF1
..............aacccguagauccgaacuugugcugcacuguacaacuggacaAa..................................  1     1  BF1
...............acccguagauccgaacuugug........................................................  1     0  BF1
.................ccguagauccgaacuugug........................................................  2     0  BF1
..................cguagauccgaacuugu.........................................................  1     0  BF1
..................cguagauccgaacuugugA.......................................................  1     1  BF1
........................................................caagaacggauauaugggauu...............  12    0  BF1
........................................................caagaacggauauaugggauuc..............  35    0  BF1
........................................................caagaacggaAauaugggauuc..............  1     1  BF1
........................................................caagaacggauauaugggauucu.............  69    0  BF1
........................................................caagaacggauauaugUgauucu.............  1     1  BF1
..........................................................agaacggauauaugggauuc..............  6     0  BF1
..........................................................agaacggauauaugggauucu.............  1     0  BF1
...........................................................gaacggauauaugggauuc..............  3     0  BF1

..............aacccguagauccgaacu............................................................  49    0  MW1
..............aacccAuagauccgaacu............................................................  1     1  MW1
..............aacccguagauccgaacuu...........................................................  7     0  MW1
..............aacccguagauGcgaacuug..........................................................  1     1  MW1
..............aacccgCagauccgaacuug..........................................................  1     1  MW1
..............aacccguagauccgaacAug..........................................................  1     1  MW1
..............aaccUguagauccgaacuug..........................................................  1     1  MW1
..............Gacccguagauccgaacuug..........................................................  1     1  MW1
..............aacccguagauccgaacuug..........................................................  700   0  MW1
..............Gacccguagauccgaacuugu.........................................................  1     1  MW1
..............aaccAguagauccgaacuugu.........................................................  1     1  MW1
..............aacccguagauccgaacuugu.........................................................  487   0  MW1
..............aGcccguagauccgaacuugug........................................................  1     1  MW1
..............aacccguaAauccgaacuugug........................................................  1     1  MW1
..............aacccguagauccgaacuugug........................................................  7277  0  MW1
..............aacccguagauccgaacuugCg........................................................  4     1  MW1
..............aacccguagauccgaacuAgug........................................................  1     1  MW1
..............aacccAuagauccgaacuugug........................................................  2     1  MW1
..............aacccgCagauccgaacuugug........................................................  4     1  MW1
..............aacccguagaCccgaacuugug........................................................  2     1  MW1
..............aacccguagauUcgaacuugug........................................................  7     1  MW1
..............aacccguagCuccgaacuugug........................................................  1     1  MW1
..............aaccUguagauccgaacuugug........................................................  1     1  MW1
..............aacccCuagauccgaacuugug........................................................  1     1  MW1
..............aacccguagauccgaacuuguA........................................................  6     1  MW1
..............aacccguagauGcgaacuugug........................................................  1     1  MW1
..............aacccguagauccAaacuugug........................................................  4     1  MW1
..............aacccguagauccgaacuCgug........................................................  2     1  MW1
..............aacUcguagauccgaacuugug........................................................  2     1  MW1
..............aacccguagGuccgaacuugug........................................................  3     1  MW1
..............aacccguagauccgaacuugGg........................................................  3     1  MW1
..............Uacccguagauccgaacuugug........................................................  2     1  MW1
```

```
              aga-miR-100
                                                        aga-miR-100*
gccccgucuguggaaacccguagauccgaacuugugcugcacuguacaacuggacacaagaacggauauaugggauucugucgacgguuggc

..............aacccguagauccgaacuuAug........................................................  5     1   MW1
..............aaAccguagauccgaacuugug........................................................  1     1   MW1
..............aacccguagauccgaacAugug........................................................  1     1   MW1
..............aacccguagauccgaacCugug........................................................  3     1   MW1
..............aacccguaUauccgaacuugug........................................................  1     1   MW1
..............aacccguagaucUgaacuugug........................................................  2     1   MW1
..............aacccguagauAcgaacuugug........................................................  1     1   MW1
..............aacccguagauccgaacuugugc.......................................................  4     0   MW1
..............aacccguagauccgaacuugugU.......................................................  12    1   MW1
..............aacccguagauccgaacuugugA.......................................................  80    1   MW1
..............aacccguagauccgaacuugugG.......................................................  7     1   MW1
..............aacccguagauccgaacuugugcugcacuguacaac..........................................  1     0   MW1
..............aacccguagauccgaacuugugcugcacuguacaacuggaca....................................  3     0   MW1
...............aAccguagauccgaacuug..........................................................  1     1   MW1
...............acccguagauccgaacuugug........................................................  1     0   MW1
................cccguagauccgaacuugug........................................................  1     0   MW1
.................ccguagauccgaacuugu.........................................................  4     0   MW1
.................ccguagauccgaacuugug........................................................  8     0   MW1
.................ccguagauccgaacuugugA.......................................................  1     1   MW1
..................cguagauccgaacuugu.........................................................  3     0   MW1
..................cguagauccgaacuugug........................................................  4     0   MW1
..................cguagauccgaacuugugA.......................................................  1     1   MW1
...................guagauccgaacuugug........................................................  2     0   MW1
...................guagauccgaacuugugA.......................................................  1     1   MW1
...................guagauccgaacuugugcugcacuguacaacuggaca....................................  1     0   MW1
....................................cugcacuguacaacuggaca....................................  15    0   MW1
................................................acuggacacaagaacggauauaugggauuc..............  1     0   MW1
.................................................cuggacacaagaacggauauaugggauuc..............  2     0   MW1
........................................................caagaacggauauaugggauu...............  19    0   MW1
........................................................caagaacggaCauaugggauuc..............  1     1   MW1
........................................................caagaacggauauaugggauuc..............  238   0   MW1
........................................................caagaacggauauaugggauucA.............  2     1   MW1
........................................................caagaacggauauaugggauucC.............  3     1   MW1
........................................................caagaacggauauaugUgauucu.............  1     1   MW1
........................................................caagaacgAauauaugggauucu.............  2     1   MW1
........................................................caagaacggauUuaugggauucu.............  1     1   MW1
........................................................caagaacggauauaugggauucu.............  186   0   MW1
..........................................................agaacggauauaugggauu...............  1     0   MW1
..........................................................agaacggauauaugggauuc..............  54    0   MW1
..........................................................agaacggauauaugggauucu.............  1     0   MW1
...........................................................gaacggauauaugggauuc..............  83    0   MW1
...........................................................gaGcggauauaugggauuc..............  1     1   MW1
...........................................................gaacggauauaugggauucu.............  2     0   MW1

............gGaacccguagauccgaacuugug........................................................  1     1   FW1
.............Caacccguagauccgaacuugu.........................................................  1     1   FW1
..............aacccguagauccgaacu............................................................  19    0   FW1
..............aacccguagauccgaacuu...........................................................  1     0   FW1
..............aacccguagauccgaacuug..........................................................  372   0   FW1
..............aacccguagauUcgaacuug..........................................................  3     1   FW1
..............aacccguagauccgaacuCg..........................................................  1     1   FW1
..............aacccAuagauccgaacuug..........................................................  1     1   FW1
..............aacccguagauccgaacuugu.........................................................  198   0   FW1
..............aacccguagauccgaacuugug........................................................  3420  0   FW1
..............aacccguagauccgaacuGgug........................................................  2     1   FW1
..............aacccguagaucUgaacuugug........................................................  2     1   FW1
..............aacAcguagauccgaacuugug........................................................  2     1   FW1
..............Gacccguagauccgaacuugug........................................................  1     1   FW1
..............aacccguagaCccgaacuugug........................................................  1     1   FW1
..............aacccguaCauccgaacuugug........................................................  1     1   FW1
..............aacccguagauAcgaacuugug........................................................  1     1   FW1
..............aaUccguagauccgaacuugug........................................................  1     1   FW1
..............aacccguagauccgaacCugug........................................................  1     1   FW1
..............aacccguagauccAaacuugug........................................................  3     1   FW1
..............aacccguagauUcgaacuugug........................................................  2     1   FW1
..............aacccguagauccgaacAugug........................................................  1     1   FW1
..............aacccguagauccgaacuuguU........................................................  2     1   FW1
..............aacccguagauccgaacuuAug........................................................  2     1   FW1
..............aacccguagauccgaacuugCg........................................................  1     1   FW1
```

```
              aga-miR-100
                                          aga-miR-100*
gccccgucuguggaaacccguagauccgaacuugugcugcacuguacaacuggacacaagaacggauauaugggauucugucgacgguuggc

..............aacccguagaGccgaacuugug........................................................   1     1   FW1
..............aGcccguagauccgaacuugug........................................................   1     1   FW1
..............aacccguagauGcgaacuugug........................................................   2     1   FW1
..............aacccguagauccUaacuugug........................................................   1     1   FW1
..............aacccguagauccgaacuugugU.......................................................   5     1   FW1
..............aacccguagauccgaacuugugA.......................................................   26    1   FW1
..............aacccguagauccgaacuugugG.......................................................   6     1   FW1
..............aacccguagauccgaacuugugc.......................................................   2     0   FW1
..............aacccguagauccgaacuugugcugcacuguacaacuggaca....................................   3     0   FW1
...............acccguagauccgaacuugug........................................................   3     0   FW1
.................ccguagauccgaacuugug........................................................   3     0   FW1
..................cguagauccgaacuugu.........................................................   1     0   FW1
..................cguagauccgaacuugug........................................................   1     0   FW1
...................guagauccgaacuugug........................................................   1     0   FW1
....................................cugcacuguacaacuggaca....................................   5     0   FW1
...............................................aacuggacacaagaacggauauaugggauuc..............   1     0   FW1
................................................acuggacacaagaacggauauaugggauuc..............   1     0   FW1
........................................................caagaacggauauaugggauu...............   5     0   FW1
........................................................caagaacggauauaugggauuc..............   119   0   FW1
........................................................caagaacggauauaugggauucu.............   83    0   FW1
........................................................caagaaUggauauaugggauucu.............   1     1   FW1
.........................................................aagaacggauauaugggauuc..............   1     0   FW1
..........................................................agaacggauauaugggauuc..............   12    0   FW1
..........................................................agaacggauauaugggauucu.............   2     0   FW1
...........................................................gaacggauauaugggauuc..............   18    0   FW1
...........................................................gaacggauauaugggauucu.............   2     0   FW1

............gGaacccguagauccgaacuugug........................................................   1     1   MW2
..............aacccguagauccgaacu............................................................   21    0   MW2
..............aaccUguagauccgaacuug..........................................................   1     1   MW2
..............aacccguagauccgaacuug..........................................................   356   0   MW2
..............aacUcguagauccgaacuug..........................................................   1     1   MW2
..............aacccguagauccgaaAuug..........................................................   1     1   MW2
..............Gacccguagauccgaacuug..........................................................   2     1   MW2
..............aacccguagauccgaacuuAu.........................................................   2     1   MW2
..............aacccguagauccgaacuugu.........................................................   128   0   MW2
..............aacccguagaCccgaacuugug........................................................   2     1   MW2
..............aacccgCagauccgaacuugug........................................................   1     1   MW2
..............aacccguagauccAaacuugug........................................................   1     1   MW2
..............aacccguagauccgaacuuUug........................................................   2     1   MW2
..............aacccguagauGcgaacuugug........................................................   1     1   MW2
..............aacccguagauccgaacuuguA........................................................   2     1   MW2
..............aacccAuagauccgaacuugug........................................................   1     1   MW2
..............aacccguagaAccgaacuugug........................................................   1     1   MW2
..............aacUcguagauccgaacuugug........................................................   1     1   MW2
..............aGcccguagauccgaacuugug........................................................   1     1   MW2
..............aacccguagauccgaacuugug........................................................   2009  0   MW2
..............aacccguagauccgaacuuAug........................................................   1     1   MW2
..............aacccguagauAcgaacuugug........................................................   2     1   MW2
..............aacccguagauccgaacuugGg........................................................   1     1   MW2
..............aacccguagauUcgaacuugug........................................................   3     1   MW2
..............aacccguagauccgaacuGgug........................................................   1     1   MW2
..............aacccguagauccgaacuugugU.......................................................   1     1   MW2
..............aacccguagauccgaacuugugA.......................................................   25    1   MW2
..............aacccguagauccgaacuugugcugcacuguacaacuggaca....................................   2     0   MW2
...............acccguagauccgaacuug..........................................................   1     0   MW2
...............aAccguagauccgaacuugug........................................................   1     1   MW2
.................ccguagauccgaacuugu.........................................................   1     0   MW2
.................ccguagauccgaacuugGg........................................................   1     1   MW2
.................ccguagauccgaacuugug........................................................   2     0   MW2
.................ccguagauccgaacuugugcu......................................................   1     0   MW2
....................................cugcacuguacaacuggaca....................................   3     0   MW2
........................................................caagaacggauauaugggauu...............   2     0   MW2
........................................................caagaacggauauaugggauuc..............   62    0   MW2
........................................................caagaacgAauauaugggauuc..............   1     1   MW2
........................................................caagaacggauauaugggauucu.............   43    0   MW2
..........................................................agaacggauauaugggauuc..............   7     0   MW2
...........................................................gaacggauauaugggauuc..............   8     0   MW2

..............aacccguagauccgaac.............................................................   2     0   TE2
```

```
              aga-miR-100
                                                        aga-miR-100*
gccccgucuguggaaacccguagauccgaacuugugcugcacuguacaacuggacacaagaacggauauaugggauucugucgacgguuggc

..............aacccguagauccgaacu............................................................    21      0       TE2
..............aacccguagauccgaacuu...........................................................    14      0       TE2
..............aacccguagauccgaacuug..........................................................    633     0       TE2
..............aacccguagauGcgaacuug..........................................................    1       1       TE2
..............aacccguagauccgaacuuA..........................................................    1       1       TE2
..............aacccguagaCccgaacuug..........................................................    1       1       TE2
..............aacccguagauccgaacuCg..........................................................    1       1       TE2
..............aacccguagauccgaacuuU..........................................................    1       1       TE2
..............aacccguagauAcgaacuug..........................................................    1       1       TE2
..............aaccUguagauccgaacuugu.........................................................    2       1       TE2
..............aacccguagauccgaacuugu.........................................................    340     0       TE2
..............aacccguagauccAaacuugu.........................................................    1       1       TE2
..............aacccguagauccgaacuCgu.........................................................    1       1       TE2
..............aacccguagauccgaacCugug........................................................    2       1       TE2
..............aacccguagauGcgaacuugug........................................................    3       1       TE2
..............aaUccguagauccgaacuugug........................................................    1       1       TE2
..............aacccguagauccgCacuugug........................................................    1       1       TE2
..............aacccguagaucUgaacuugug........................................................    3       1       TE2
..............aacccguagaucGgaacuugug........................................................    1       1       TE2
..............aacccguagauccgaacuuguU........................................................    1       1       TE2
..............aacccAuagauccgaacuugug........................................................    2       1       TE2
..............aacccguagauccgaacuuAug........................................................    2       1       TE2
..............aacccUuagauccgaacuugug........................................................    1       1       TE2
..............aacAcguagauccgaacuugug........................................................    2       1       TE2
..............aacccguagauccgaacuugug........................................................    2157    0       TE2
..............aacccguaAauccgaacuugug........................................................    1       1       TE2
..............aacccguagaCccgaacuugug........................................................    2       1       TE2
..............aacccguagauAcgaacuugug........................................................    1       1       TE2
..............aacccguagauccgaacuCgug........................................................    1       1       TE2
..............aacccguagauUcgaacuugug........................................................    2       1       TE2
..............aacccguagauccgaacuuguA........................................................    9       1       TE2
..............aacccguagauccgaGcuugug........................................................    1       1       TE2
..............aacccguagauccgaacuugugc.......................................................    4       0       TE2
..............aacccguagauccgaacuugugU.......................................................    8       1       TE2
..............aacccguagauccgaacuugugA.......................................................    36      1       TE2
..............aacccguagauccgaacuugugG.......................................................    18      1       TE2
..............aacccguagauccgaacuugugcug.....................................................    2       0       TE2
..............aacccguagauccgaacuugugcugc....................................................    1       0       TE2
..............aacccguagauccgaacuugugcugcac..................................................    1       0       TE2
................cccguagauccgaacuugu.........................................................    1       0       TE2
.................ccguagauccgaacuugu.........................................................    2       0       TE2
.................ccguagauccgaacuugug........................................................    1       0       TE2
..................cguagauccgaacuugug........................................................    2       0       TE2
........................................................caagaacggauauaugggau................    2       0       TE2
........................................................Aaagaacggauauaugggauu...............    1       1       TE2
........................................................caagaacggauauaugggauu...............    66      0       TE2
........................................................caagaacggauauaugUgauuc..............    1       1       TE2
........................................................caagaacggauauaugggauuU..............    1       1       TE2
........................................................caagaacggauauaugggauuc..............    134     0       TE2
........................................................caagaacggauauaugggauucA.............    1       1       TE2
........................................................caagaacggauauaugggauucu.............    68      0       TE2
.........................................................aagaacggauauaugggauu...............    1       0       TE2
..........................................................agaacggauauaugggauuc..............    32      0       TE2
...........................................................gaacggauauauggAauuc..............    1       1       TE2
...........................................................gaacggauauaugggaCuc..............    1       1       TE2
...........................................................gaacgAauauaugggauuc..............    1       1       TE2
...........................................................gaacggauauaugggauuc..............    169     0       TE2
............................................................aacggauauaugggauuc..............    2       0       TE2
```

miRBase precursor	: aga-mir-10
Total read count	: 201101
aga-miR-10 read count	: 197804
aga-miR-10* read count	: 3293
remaining reads	: 4

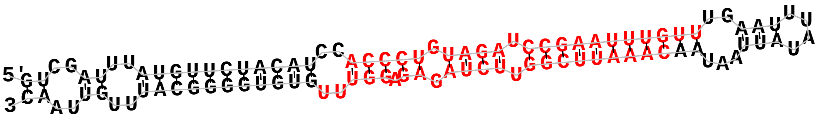

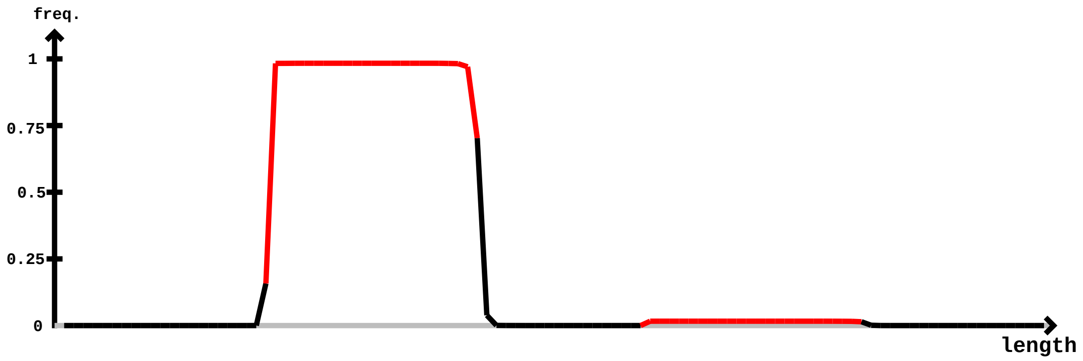


```
                                aga-miR-10
                                                         aga-miR-10*
5'- gucgauuuauguucuacaucaacccuguagauccgaauuuguuugaauuuauauuaauaacaaauucgguucuagagagguuugugugggcauuuguuaac   -3'    exp
    ((..((..((((((((((..(((((.((((.((((((((((..(((....)))...))))))))))).)))).)).)))..))))))))))..))..))   reads      mm        sample
    ....................cacccuguagauccgaauuug............................................................   9          0         TE1
    ....................cacccuguagauccgaauuugC...........................................................   1          1         TE1
    ....................Uacccuguagauccgaauuugu...........................................................   1          1         TE1
    ....................cacccuguagauccgaauuugu...........................................................   275        0         TE1
    ....................cacccuguagauccAaauuugu...........................................................   2          1         TE1
    ....................cacccuguagaCccgaauuugu...........................................................   2          1         TE1
    ....................cacccuguagauccgaauuuguu..........................................................   84         0         TE1
    .....................acccuguagauccgaauu..............................................................   1          0         TE1
    .....................acccuguagauccgaauuug............................................................   18         0         TE1
    .....................acccuguagauccUaauuugu...........................................................   1          1         TE1
    .....................acccuguagauccgaauuugu...........................................................   332        0         TE1
    .....................Ucccuguagauccgaauuugu...........................................................   1          1         TE1
    .....................Ccccuguagauccgaauuugu...........................................................   1          1         TE1
    .....................acccuguagauccgaauuuguG..........................................................   2          1         TE1
    .....................acccuguagauUcgaauuuguu..........................................................   1          1         TE1
    .....................acccuguagauAcgaauuuguu..........................................................   1          1         TE1
    .....................acccuguagauccgaauuugCu..........................................................   1          1         TE1
    .....................acccuguGgauccgaauuuguu..........................................................   1          1         TE1
    .....................acccuguagauGcgaauuuguu..........................................................   3          1         TE1
    .....................acccuguagaCccgaauuuguu..........................................................   2          1         TE1
    .....................acccuguagauccgaauuuUuu..........................................................   1          1         TE1
    .....................acccuguagauccgaauuuguu..........................................................   1604       0         TE1
    .....................aUccuguagauccgaauuuguu..........................................................   1          1         TE1
    .....................acccuguagauccgaauuuguA..........................................................   2          1         TE1
    .....................acccuguagaAccgaauuuguu..........................................................   1          1         TE1
    .....................acccuguagauccgaauuuguC..........................................................   1          1         TE1
    .....................acccuguagGuccgaauuuguu..........................................................   1          1         TE1
    .....................acccCguagauccgaauuuguu..........................................................   1          1         TE1
    .....................acccuguagauccgaauuuguuG.........................................................   3          1         TE1
    .....................acccuguagauccgaauuuguuu.........................................................   4          0         TE1
    .....................acccuguagauccgaauuuUuuu.........................................................   1          1         TE1
    .....................acccuguagauccgaauuuguuA.........................................................   67         1         TE1
    .....................acccuguagauccgaauuuguuuA........................................................   2          1         TE1
    ............................................................caaauucgguucuagagagguuu..................   14         0         TE1
```

```
                     aga-miR-10
                                                            aga-miR-10*
gucgauuuauguucuacauccacccuguagauccgaauuuguuugaauuuauauuaauaacaaauucgguucuagagagguuuguguggggcauuuguuaac

............................................................caaauucgguucuagagagguuuU..................    1       1       TE1

....................cacccuguagauccgaauuu..............................................................    1       0       OV2
....................cacccuguagauccgaauuug.............................................................    15      0       OV2
....................cacccuguagauccgGauuugu............................................................    1       1       OV2
....................cacccCguagauccgaauuugu............................................................    1       1       OV2
....................cacccuguagauccgaauuugu............................................................    253     0       OV2
....................cGcccuguagauccgaauuuguu...........................................................    1       1       OV2
....................cacccuguagauccgaauCuguu...........................................................    1       1       OV2
....................cacccuguagauccgaauuuguu...........................................................    102     0       OV2
....................cacccuguagauccgaauuuguuA..........................................................    4       1       OV2
....................cacccuguagauccgaauuuguuu..........................................................    3       0       OV2
.....................acccuguagauccgaau................................................................    1       0       OV2
.....................acccuguagauccgaauu...............................................................    1       0       OV2
.....................acccuguagauccgaauuu..............................................................    2       0       OV2
.....................acccuguagauccgaauuug.............................................................    29      0       OV2
.....................acccuguagauccAaauuugu............................................................    1       1       OV2
.....................acccuguagauccUaauuugu............................................................    1       1       OV2
.....................Gcccuguagauccgaauuugu............................................................    1       1       OV2
.....................acccuguagauccgaauuugu............................................................    529     0       OV2
.....................Ucccuguagauccgaauuuguu...........................................................    1       1       OV2
.....................acccuguagauGcgaauuuguu...........................................................    1       1       OV2
.....................acccuguagaGccgaauuuguu...........................................................    1       1       OV2
.....................Ccccuguagauccgaauuuguu...........................................................    1       1       OV2
.....................acccuguagauccgaGuuuguu...........................................................    1       1       OV2
.....................acccuguagauccgaaCuuguu...........................................................    1       1       OV2
.....................acccuguagauccAaauuuguu...........................................................    1       1       OV2
.....................acccuguagauccgaauuuguu...........................................................    1814    0       OV2
.....................acccuguagauccgaauuuAuu...........................................................    1       1       OV2
.....................acccugCagauccgaauuuguu...........................................................    1       1       OV2
.....................acccuguagauccgaauuuguA...........................................................    1       1       OV2
.....................acccuguagauUcgaauuuguu...........................................................    1       1       OV2
.....................acccuguagaAccgaauuuguu...........................................................    1       1       OV2
.....................acccuguagauAcgaauuuguu...........................................................    1       1       OV2
.....................aUccuguagauccgaauuuguu...........................................................    1       1       OV2
.....................acccuguagaCccgaauuuguu...........................................................    2       1       OV2
.....................acccuguagauccgaauuugCu...........................................................    2       1       OV2
.....................acccuguagauccgaauuuguuG..........................................................    3       1       OV2
.....................acccuguagauccgaauuuguuA..........................................................    119     1       OV2
.....................acccuguagauccgaauuuguuC..........................................................    1       1       OV2
.....................acccuguagauccgaauuuguuu..........................................................    20      0       OV2
.....................acccuguagauccgaauuuguuuA.........................................................    1       1       OV2
.....................acccuguagauccgaauuuguuuAaa.......................................................    1       1       OV2
.......................ccuguagauccgaauuuguu...........................................................    1       0       OV2
............................................................caaauucgguucuagagagguu....................    4       0       OV2
............................................................caaauucgguucuagagagguuu...................    33      0       OV2
............................................................caaauuUgguucuagagagguuu...................    1       1       OV2
............................................................caaauucgguucuagagagguuuU..................    3       1       OV2

...................Acacccuguagauccgaauuugu............................................................    1       1       FF2
....................cacccuguagauccgaau................................................................    1       0       FF2
....................cacccuguagauccgaauu...............................................................    7       0       FF2
....................cacccuguagauccgaauuu..............................................................    6       0       FF2
....................cacccuguaCauccgaauuug.............................................................    1       1       FF2
....................cacccuguagauccgaauuug.............................................................    66      0       FF2
....................cacccuguagauccAaauuug.............................................................    1       1       FF2
....................caAccuguagauccgaauuugu............................................................    3       1       FF2
....................Uacccuguagauccgaauuugu............................................................    1       1       FF2
....................cacccuguagauccUaauuugu............................................................    1       1       FF2
....................cacccuguagauccgCauuugu............................................................    1       1       FF2
....................cacccuguagauAcgaauuugu............................................................    1       1       FF2
....................cacccuguagCuccgaauuugu............................................................    1       1       FF2
....................cacccugGagauccgaauuugu............................................................    1       1       FF2
....................cacccuguagauccgaauuugG............................................................    6       1       FF2
....................caccAuguagauccgaauuugu............................................................    1       1       FF2
....................cacccuguagauccgaauCugu............................................................    1       1       FF2
....................caGccuguagauccgaauuugu............................................................    1       1       FF2
....................cacccuguagauccgaauuugA............................................................    1       1       FF2
....................cacccugCagauccgaauuugu............................................................    2       1       FF2
```

aga-miR-10

aga-miR-10*

gucgauuuauguucuacauccacccuguagauccgaauuuguuugaauuuauauuaauaacaaauucgguucuagagagguuugugugggcauuuguuaac

....................cacUcuguagauccgaauuugu...	1	1	FF2
....................cacccuUuagauccgaauuugu...	1	1	FF2
....................cacccuguCgauccgaauuugu...	1	1	FF2
....................cacccuguagauccgaauuugu...	1432	0	FF2
....................cacccuguagaCccgaauuuguu..	1	1	FF2
....................cacccuguagauccgaaCuuguu..	1	1	FF2
....................cacccuAuagauccgaauuuguu..	2	1	FF2
....................cacccuguagauccgaauuuguu..	466	0	FF2
....................cacccuguagauccgaauuugCu..	1	1	FF2
....................cacccuguagauccUaauuuguu..	1	1	FF2
....................cacccuguagauccgaauuugGu..	2	1	FF2
....................cacccuguagaucUgaauuuguu..	1	1	FF2
....................cacccuguagaAccgaauuuguu..	1	1	FF2
....................cacccuguagauccgaauuuguG..	1	1	FF2
....................cacccuguagauccgaauAuguu..	1	1	FF2
....................cacccuguaCauccgaauuuguu..	1	1	FF2
....................cUcccuguagauccgaauuuguu..	1	1	FF2
....................cacccuguagauccgaauuuguA..	4	1	FF2
....................cacccuguagauccgaauuuguuA...	11	1	FF2
....................cacccuguagauccgaauuuguuu...	9	0	FF2
.....................acccuguagauccAaau...	1	1	FF2
.....................acccuguagauccgaau...	13	0	FF2
.....................acccuguagauccgaauu..	16	0	FF2
.....................acccuguagaCccgaauuug..	1	1	FF2
.....................acccuguagauccgaauuug..	160	0	FF2
.....................acccuguagCuccgaauuug..	1	1	FF2
.....................acAcuguagauccgaauuug..	1	1	FF2
.....................acccugCagauccgaauuug..	1	1	FF2
.....................acccuguagauccgaauuuAu...	4	1	FF2
.....................acccuguagauccgaauuugC...	1	1	FF2
.....................acccuguagauAcgaauuugu...	2	1	FF2
.....................acccuguagauccgaauuugu...	2440	0	FF2
.....................acccuguagauGcgaauuugu...	1	1	FF2
.....................aUccuguagauccgaauuugu...	1	1	FF2
.....................acccuAuagauccgaauuugu...	1	1	FF2
.....................acccugCagauccgaauuugu...	1	1	FF2
.....................acccAguagauccgaauuugu...	1	1	FF2
.....................accUuguagauccgaauuugu...	1	1	FF2
.....................acccuguagauccgaauCugu...	2	1	FF2
.....................acccuguagaucUgaauuugu...	2	1	FF2
.....................acccuguagaGccgaauuugu...	1	1	FF2
.....................acccuguagaCccgaauuugu...	3	1	FF2
.....................acccuguagauccgGauuugu...	1	1	FF2
.....................acccuguagauccgCauuugu...	1	1	FF2
.....................acccuguagauUcgaauuugu...	1	1	FF2
.....................acccuguGgauccgaauuugu...	1	1	FF2
.....................acccuguagauccCaauuugu...	1	1	FF2
.....................acccCguagauccgaauuugu...	2	1	FF2
.....................aAccuguagauccgaauuuguu..	2	1	FF2
.....................acccuguGgauccgaauuuguu..	1	1	FF2
.....................acccuguagauccgCauuuguu..	1	1	FF2
.....................acccCguagauccgaauuuguu..	4	1	FF2
.....................acccuguagauccgaauuuguA..	8	1	FF2
.....................acccuUuagauccgaauuuguu..	2	1	FF2
.....................Ccccuguagauccgaauuuguu..	1	1	FF2
.....................acccuguagaAccgaauuuguu..	1	1	FF2
.....................acccuAuagauccgaauuuguu..	3	1	FF2
.....................acccuguagauccgaauuugCu..	4	1	FF2
.....................acccuguagauccgaauuuguG..	15	1	FF2
.....................acccuguagauccAaauuuguu..	9	1	FF2
.....................acAcuguagauccgaauuuguu..	1	1	FF2
.....................acccuguagauccgaaAuuguu..	1	1	FF2
.....................aUccuguagauccgaauuuguu..	2	1	FF2
.....................acccuguagUuccgaauuuguu..	1	1	FF2
.....................acccuguagauccgGauuuguu..	1	1	FF2
.....................acccuguaUauccgaauuuguu..	1	1	FF2
.....................acccuguagauccgaGuuuguu..	2	1	FF2
.....................acccuguagaCccgaauuuguu..	4	1	FF2
.....................acccuguagauccgaaGuuguu..	1	1	FF2

```
                    aga-miR-10
                                        aga-miR-10*
gucgauuuauguucuacauccacccuguagauccgaauuuguuugaauuuauauuaauaacaaauucgguucuagagagguuuguguggggcauuuguuaac

.....................acccuguagauAcgaauuuguu...........................................................  5     1   FF2
.....................acUcuguagauccgaauuuguu...........................................................  5     1   FF2
.....................acccuguagauccgaauCuguu...........................................................  3     1   FF2
.....................acccuguagauccgaauuuguC...........................................................  5     1   FF2
.....................acccugAagauccgaauuuguu...........................................................  1     1   FF2
.....................accUuguagauccgaauuuguu...........................................................  3     1   FF2
.....................acccuguagauccgaauuuAuu...........................................................  6     1   FF2
.....................acccuguUgauccgaauuuguu...........................................................  1     1   FF2
.....................accAuguagauccgaauuuguu...........................................................  1     1   FF2
.....................acccuguagaucUgaauuuguu...........................................................  2     1   FF2
.....................acccuguagaucAgaauuuguu...........................................................  3     1   FF2
.....................Ucccuguagauccgaauuuguu...........................................................  2     1   FF2
.....................acccuguagauccgaauuCguu...........................................................  2     1   FF2
.....................acccuguagauUcgaauuuguu...........................................................  6     1   FF2
.....................acccugCagauccgaauuuguu...........................................................  3     1   FF2
.....................acccuguagauGcgaauuuguu...........................................................  3     1   FF2
.....................acccuguagauccgaCuuuguu...........................................................  1     1   FF2
.....................acccuguagauccgaaCuuguu...........................................................  3     1   FF2
.....................acccuguagauccgaauuuguu...........................................................  8181  0   FF2
.....................acccuguagauccgaauuugGu...........................................................  1     1   FF2
.....................acccuguagaucAgaauuuguuu..........................................................  1     1   FF2
.....................acccuguagauccgaauCuguuu..........................................................  1     1   FF2
.....................acccuguagauccgaauuuguuu..........................................................  83    0   FF2
.....................acccuguagauccgaauuuguuA..........................................................  384   1   FF2
.....................acccuguagauccgaauuuguuC..........................................................  2     1   FF2
.....................acccuguagauccgaauuuguuG..........................................................  4     1   FF2
.....................acccuguagauccgaauuuguuuA.........................................................  1     1   FF2
.....................acccuguagauccgaauuuguuug.........................................................  1     0   FF2
.....................acccuguagauccgaauuuguuAg.........................................................  1     1   FF2
.....................acccuguagauccgaauuuguuuC.........................................................  1     1   FF2
.....................acccuguagauccgaauuuguuuAa........................................................  2     1   FF2
......................cccuguagauccgaauuuguu...........................................................  2     0   FF2
.......................ccuguagauccgaauuugu............................................................  1     0   FF2
.......................ccuguagauccgaauuuguu...........................................................  5     0   FF2
.........................uguagauccgaauuuguu...........................................................  2     0   FF2
............................................................caaauucgguucuagagag.......................  1     0   FF2
............................................................caaauucgguucuagagagg......................  2     0   FF2
............................................................caaauucgguucuagagaggu.....................  4     0   FF2
............................................................caaauucggCucuagagagguu....................  1     1   FF2
............................................................caaauucgguucuagagagguu....................  17    0   FF2
............................................................caaauuUgguucuagagagguu....................  1     1   FF2
............................................................caaauucgguucuagagagguuu...................  169   0   FF2
............................................................caaauucgguucuagagagguuuC..................  3     1   FF2
............................................................caaauucgguucuagagagguuuU..................  8     1   FF2
............................................................caaauucgguucuagagagguuuA..................  2     1   FF2
............................................................caaauucgguucuagagagguuug..................  1     0   FF2
............................................................caaauucgguucuagagagguuuUu.................  1     1   FF2
............................................................caaauucgguucuagagagguuuAu.................  1     1   FF2
.............................................................aaauucgguucuagagagguuu...................  1     0   FF2

...................ccacccuguagauccgaauuugu............................................................  3     0   MF2
...................Ucacccuguagauccgaauuugu............................................................  1     1   MF2
...................ccacccuguagauccgaauuuguu...........................................................  1     0   MF2
...................Ucacccuguagauccgaauuuguu...........................................................  2     1   MF2
....................cacccuguagauccgaa.................................................................  1     0   MF2
....................cacccuguagauccgaau................................................................  3     0   MF2
....................cacccuguagauccgaauu...............................................................  12    0   MF2
....................cacccuguagauccgaauuu..............................................................  26    0   MF2
....................cacccuguagauccgaauuug.............................................................  492   0   MF2
....................cacccuguagauccgaGuuug.............................................................  1     1   MF2
....................Uacccuguagauccgaauuug.............................................................  1     1   MF2
....................cacccuguagauccAaauuug.............................................................  2     1   MF2
....................cacccuguagauccgGauuug.............................................................  1     1   MF2
....................caGccuguagauccgaauuugu............................................................  2     1   MF2
....................cacccuguagauccgaauuugG............................................................  20    1   MF2
....................cacGcuguagauccgaauuugu............................................................  1     1   MF2
....................cacccuguagauccgUauuugu............................................................  2     1   MF2
....................cacccuguagauccAaauuugu............................................................  6     1   MF2
....................cacccGguagauccgaauuugu............................................................  1     1   MF2
```

aga-miR-10

aga-miR-10*

gucgauuuauguucuacauccacccuguagauccgaauuuguuugaauuuauauuaauaacaaauucgguucuagagagguuugugugggg cauuuguuaac

...................cacccuguCgauccgaauuugu...	1	1	MF2
...................cacccuguagauccgaGuuugu...	2	1	MF2
...................cacccuguagaCccgaauuugu...	12	1	MF2
...................cacccuguagauccgaauAugu...	1	1	MF2
...................cacccuguagUuccgaauuugu...	4	1	MF2
...................cGcccuguagauccgaauuugu...	2	1	MF2
...................cacccuguagauccgaauuCgu...	3	1	MF2
...................cacccuguagauccgGauuugu...	2	1	MF2
...................cacccuguagauccgaCuuugu...	2	1	MF2
...................cacccuguagauccgaauuugu...	14930	0	MF2
...................cacccuguagCuccgaauuugu...	2	1	MF2
...................cacccuguagauccgaauuuUu...	1	1	MF2
...................cacccuguagauccgaauuuAu...	6	1	MF2
...................cacccuguagauccgaauuugC...	10	1	MF2
...................cacccCguagauccgaauuugu...	5	1	MF2
...................AacccuguagauccgaauuuGu...	2	1	MF2
...................cacccuguaCauccgaauuugu...	1	1	MF2
...................cacccuguagaAccgaauuugu...	3	1	MF2
...................caccUuguagauccgaauuugu...	2	1	MF2
...................cacUcuguagauccgaauuugu...	5	1	MF2
...................cacccuguagaucUgaauuugu...	7	1	MF2
...................Gacccuguagauccgaauuugu...	2	1	MF2
...................cacccuguagauccgaauCugu...	5	1	MF2
...................cacccuguagauccgCauuugu...	2	1	MF2
...................cacccuUuagauccgaauuugu...	3	1	MF2
...................cacccugCagauccgaauuugu...	11	1	MF2
...................cacccuguagauUcgaauuugu...	10	1	MF2
...................cacccuAuagauccgaauuugu...	13	1	MF2
...................caccAuguagauccgaauuugu...	1	1	MF2
...................cacccuguagauccgaauuGgu...	1	1	MF2
...................cacccuguagaGccgaauuugu...	3	1	MF2
...................cacccuguagaucAgaauuugu...	3	1	MF2
...................cacccuguagauccgaaCuugu...	1	1	MF2
...................cacccugAagauccgaauuugu...	2	1	MF2
...................cacccuguagGuccgaauuugu...	2	1	MF2
...................Uacccuguagauccgaauuugu...	3	1	MF2
...................caUccuguagauccgaauuugu...	6	1	MF2
...................cacccuguaAauccgaauuugu...	3	1	MF2
...................cacccuguagauccgaauuugA...	11	1	MF2
...................cacccuguagauccgaauuuguA..	40	1	MF2
...................cacccuguGgauccgaauuuguu..	1	1	MF2
...................cacccuguagauccgaGuuuguu..	1	1	MF2
...................cacccugCagauccgaauuuguu..	3	1	MF2
...................cacccuguagCuccgaauuuguu..	2	1	MF2
...................cacccCguagauccgaauuuguu..	2	1	MF2
...................cacccuguagaGccgaauuuguu..	3	1	MF2
...................cacccuguagaucGgaauuuguu..	1	1	MF2
...................cacccuguagauccgaauuugGu..	3	1	MF2
...................cacccuguagauccgaauuuUuu..	1	1	MF2
...................cacccuCuagauccgaauuuguu..	1	1	MF2
...................cacccuguagauccgCauuuguu..	1	1	MF2
...................cacccuguagauccgaauuuguu..	4971	0	MF2
...................cacccuguagaAccgaauuuguu..	2	1	MF2
...................cGcccuguagauccgaauuuguu..	2	1	MF2
...................cacccGguagauccgaauuuguu..	1	1	MF2
...................caUccuguagauccgaauuuguu..	3	1	MF2
...................caccUuguagauccgaauuuguu..	1	1	MF2
...................cacccuAuagauccgaauuuguu..	2	1	MF2
...................cacUcuguagauccgaauuuguu..	1	1	MF2
...................Uacccuguagauccgaauuuguu..	4	1	MF2
...................cacccuguagauccAaauuuguu..	1	1	MF2
...................cacccuguagaucUgaauuuguu..	2	1	MF2
...................cacccuguagauccgaaCuuguu..	2	1	MF2
...................cacccuguagauccgaauCuguu..	1	1	MF2
...................cacccuguagauUcgaauuuguu..	1	1	MF2
...................cacccuguagauccgaauuugCu..	4	1	MF2
...................cacccugGagauccgaauuuguu..	1	1	MF2
...................cacccuguagUuccgaauuuguu..	1	1	MF2
...................cacccuguagauccgaauuuguG..	3	1	MF2

aga-miR-10

aga-miR-10*

gucgauuuauguucuacauccacccuguagauccgaauuuguuugaauuuauauuaauaacaaauucgguucuagagagguuuguguggggcauuuguuaac

....................cacccuguagaCccgaauuuguu...	1	1	MF2
....................cacccuguagauccAaauuuguuu..	1	1	MF2
....................cacccuguagauccgaauuuguuu..	94	0	MF2
....................cacccuguagauccgaauuuUuuu..	1	1	MF2
....................cacccuguagauccgaauuuguAu..	2	1	MF2
....................cacccuguagauccgaauuuguuA..	192	1	MF2
....................cacccuguagauccgaauuuguuuU...	2	1	MF2
....................cacccuguagauccgaauuuguuAg...	1	1	MF2
....................cacccuguagauccgaauuuguuuA...	1	1	MF2
....................cacccuguagauccgaauuuguuugaauuuau..	1	0	MF2
.....................acccuguagauccgaau..	1	0	MF2
.....................acccuguagauccgaauu...	24	0	MF2
.....................acccuguagauccgaauuu..	48	0	MF2
.....................acccuguagUuccgaauuug...	1	1	MF2
.....................acccugGagauccgaauuug...	1	1	MF2
.....................acccuguagauccgaauuug...	932	0	MF2
.....................acccuguagauGcgaauuug...	1	1	MF2
.....................acccuAuagauccgaauuug...	1	1	MF2
.....................acccuguGgauccgaauuug...	1	1	MF2
.....................acccAguagauccgaauuug...	1	1	MF2
.....................acccuguagauAcgaauuug...	2	1	MF2
.....................acccuguagauccgaaCuug...	1	1	MF2
.....................Ccccuguagauccgaauuug...	1	1	MF2
.....................acccuguagaucGgaauuug...	1	1	MF2
.....................acccuguagaucUgaauuugu..	10	1	MF2
.....................acccugAagauccgaauuugu..	6	1	MF2
.....................acccuguagauccgaauCugu..	11	1	MF2
.....................acccuguGgauccgaauuugu..	4	1	MF2
.....................acccuguagGuccgaauuugu..	7	1	MF2
.....................acccuguagauccgaauuAgu..	2	1	MF2
.....................acccuCuagauccgaauuugu..	1	1	MF2
.....................aUccuguagauccgaauuugu..	6	1	MF2
.....................acccuguagauccgaauuugu..	19839	0	MF2
.....................acccugCagauccgaauuugu..	9	1	MF2
.....................acccuguagauccAaauuugu..	12	1	MF2
.....................acccuUuagauccgaauuugu..	2	1	MF2
.....................acccugGagauccgaauuugu..	1	1	MF2
.....................acccuguagauccgaauuugG..	9	1	MF2
.....................acccuguaAauccgaauuugu..	4	1	MF2
.....................Ccccuguagauccgaauuugu..	1	1	MF2
.....................acccuguagauccgaUuuugu..	3	1	MF2
.....................acccuguagauccgaaCuugu..	5	1	MF2
.....................acccuguagCuccgaauuugu..	5	1	MF2
.....................acccuguagauccgaauuugC..	10	1	MF2
.....................acccuguaUauccgaauuugu..	3	1	MF2
.....................acccuguagauccgUauuugu..	3	1	MF2
.....................acccuguagaAccgaauuugu..	1	1	MF2
.....................acccuAuagauccgaauuugu..	13	1	MF2
.....................acccAguagauccgaauuugu..	2	1	MF2
.....................acccuguagauccgaGuuugu..	2	1	MF2
.....................acccuguagauccgCauuugu..	3	1	MF2
.....................acccuguagauccgaaGuugu..	1	1	MF2
.....................Gcccuguagauccgaauuugu..	5	1	MF2
.....................acAcuguagauccgaauuugu..	3	1	MF2
.....................acccuguagauccgaauuuUu..	1	1	MF2
.....................acccCguagauccgaauuugu..	5	1	MF2
.....................acccuguagauccgaauuCgu..	8	1	MF2
.....................acccuguagauccgaCuuugu..	3	1	MF2
.....................aAccuguagauccgaauuugu..	1	1	MF2
.....................acccuguagauccgaaAuugu..	1	1	MF2
.....................accGuguagauccgaauuugu..	1	1	MF2
.....................acccuguagauccgaauuugA..	3	1	MF2
.....................acccuguagauccgaauuuAu..	6	1	MF2
.....................acUcuguagauccgaauuugu..	7	1	MF2
.....................acccuguagauAcgaauuugu..	15	1	MF2
.....................aGccuguagauccgaauuugu..	2	1	MF2
.....................acccuguagUuccgaauuugu..	2	1	MF2
.....................accUuguagauccgaauuugu..	1	1	MF2
.....................acccuguagauccgaauuuCu..	1	1	MF2

```
                    aga-miR-10
                                        aga-miR-10*
gucgauuuauguucuacauccacccuguagauccgaauuuguuugaauuuauauuaauaacaaauucgguucuagagagguuugugugggcauuuguuaac

.....................acccuguagaucAgaauuugu...........................................................    6       1    MF2
.....................acccuguagauccgGauuugu...........................................................    4       1    MF2
.....................acccuguagaCccgaauuugu...........................................................    13      1    MF2
.....................acccuguagauccCaauuugu...........................................................    1       1    MF2
.....................acccuguagauccUaauuugu...........................................................    1       1    MF2
.....................acccuguagauGcgaauuugu...........................................................    8       1    MF2
.....................acccuguagauccgaauAugu...........................................................    2       1    MF2
.....................acccuguagaGccgaauuugu...........................................................    2       1    MF2
.....................acccuguagauUcgaauuugu...........................................................    18      1    MF2
.....................acccuguagauGcgaauuuguu..........................................................    42      1    MF2
.....................acccAguagauccgaauuuguu..........................................................    4       1    MF2
.....................acccuguagauccgUauuuguu..........................................................    4       1    MF2
.....................acccuguagaCccgaauuuguu..........................................................    24      1    MF2
.....................acccuUuagauccgaauuuguu..........................................................    7       1    MF2
.....................acccuguagauccCaauuuguu..........................................................    3       1    MF2
.....................acccuguagauAcgaauuuguu..........................................................    51      1    MF2
.....................acccuguagauccgGauuuguu..........................................................    6       1    MF2
.....................acccuAuagauccgaauuuguu..........................................................    35      1    MF2
.....................accAuguagauccgaauuuguu..........................................................    5       1    MF2
.....................acccuguagauccgaauuuAuu..........................................................    20      1    MF2
.....................acccuCuagauccgaauuuguu..........................................................    3       1    MF2
.....................acccuguagauccgaCuuuguu..........................................................    7       1    MF2
.....................acccuguagaucAgaauuuguu..........................................................    20      1    MF2
.....................acccuguaAauccgaauuuguu..........................................................    8       1    MF2
.....................acccuguagGuccgaauuuguu..........................................................    7       1    MF2
.....................acccCguagauccgaauuuguu..........................................................    28      1    MF2
.....................accGuguagauccgaauuuguu..........................................................    2       1    MF2
.....................acccuguagauccgaGuuuguu..........................................................    15      1    MF2
.....................acccuguagauccgCauuuguu..........................................................    3       1    MF2
.....................acccuguagauccgaauuuguC..........................................................    31      1    MF2
.....................acccuguagauccgaauuugGu..........................................................    10      1    MF2
.....................accUuguagauccgaauuuguu..........................................................    15      1    MF2
.....................acGcuguagauccgaauuuguu..........................................................    2       1    MF2
.....................acccuguagUuccgaauuuguu..........................................................    4       1    MF2
.....................acccuguagauccgaauuCguu..........................................................    14      1    MF2
.....................acccuguagaucGgaauuuguu..........................................................    3       1    MF2
.....................aAccuguagauccgaauuuguu..........................................................    2       1    MF2
.....................acccuguagauccgaauuugCu..........................................................    39      1    MF2
.....................Ucccuguagauccgaauuuguu..........................................................    8       1    MF2
.....................acccuguagauccgaauuuguA..........................................................    116     1    MF2
.....................acccugAagauccgaauuuguu..........................................................    8       1    MF2
.....................acccuguaCauccgaauuuguu..........................................................    1       1    MF2
.....................acccuguagauccgaauGuguu..........................................................    1       1    MF2
.....................aUccuguagauccgaauuuguu..........................................................    24      1    MF2
.....................acccuguagauccAaauuuguu..........................................................    52      1    MF2
.....................acccuguagauUcgaauuuguu..........................................................    57      1    MF2
.....................acccuguagauccgaUuuuguu..........................................................    7       1    MF2
.....................Gcccuguagauccgaauuuguu..........................................................    20      1    MF2
.....................acccuguagaAccgaauuuguu..........................................................    12      1    MF2
.....................acccugGagauccgaauuuguu..........................................................    4       1    MF2
.....................acccuguagauccgaaCuuguu..........................................................    21      1    MF2
.....................Ccccuguagauccgaauuuguu..........................................................    2       1    MF2
.....................acccugCagauccgaauuuguu..........................................................    15      1    MF2
.....................acccuguagauccgaauAuguu..........................................................    1       1    MF2
.....................acccuguaUauccgaauuuguu..........................................................    4       1    MF2
.....................acccuguagauccgaauCuguu..........................................................    17      1    MF2
.....................acccuguagauccgaauuuguG..........................................................    87      1    MF2
.....................acccuguagauccgaauuuCuu..........................................................    1       1    MF2
.....................acccuguGgauccgaauuuguu..........................................................    9       1    MF2
.....................acccGguagauccgaauuuguu..........................................................    3       1    MF2
.....................aGccuguagauccgaauuuguu..........................................................    2       1    MF2
.....................acUcuguagauccgaauuuguu..........................................................    17      1    MF2
.....................acccuguagauccgaaGuuguu..........................................................    1       1    MF2
.....................acccuguagauccgaauuuUuu..........................................................    5       1    MF2
.....................acccuguagauccgaaAuuguu..........................................................    5       1    MF2
.....................acccuguagCuccgaauuuguu..........................................................    4       1    MF2
.....................acccuguagauccgaauuuguu..........................................................    69911   0    MF2
.....................acccuguagauccgaauGguu...........................................................    3       1    MF2
.....................acccuguagauccgaauuugAu..........................................................    2       1    MF2
```

aga-miR-10

aga-miR-10*

gucgauuuauguucuacauccacccuguagauccgaauuuguuugaauuuauauuaauaacaaauucgguucuagagagguuugugugggcauuuguuaac

.....................acccuguagaGccgaauuuguu..	9	1	MF2
.....................acccuguagauccgaauuAguu..	1	1	MF2
.....................acccuguagaucUgaauuuguu..	40	1	MF2
.....................acccuguagauccUaauuuguu..	5	1	MF2
.....................acAcuguagauccgaauuuguu..	7	1	MF2
.....................acccuguagauccgaauuuguuG...	73	1	MF2
.....................acUcuguagauccgaauuuguuu...	1	1	MF2
.....................acccuguagauUcgaauuuguuu...	1	1	MF2
.....................acccuguagauccgaauuuguuA...	3891	1	MF2
.....................acccuguagauccgaauuuguuC...	29	1	MF2
.....................acccuguagauccgaauuuguuu...	603	0	MF2
.....................acccuguagauccgaauuuguAu...	2	1	MF2
.....................acccuguagaucUgaauuuguuu...	1	1	MF2
.....................acccuguagaCccgaauuuguuu...	1	1	MF2
.....................acccuguagauccgaauuuUuuu...	5	1	MF2
.....................acccuguagauccgaauuuguCu...	1	1	MF2
.....................acccuguagauccgaauuuguuuU..	3	1	MF2
.....................acccuguagauccgaauuuguuug..	2	0	MF2
.....................acccuguagauccgaauuuguuuA..	36	1	MF2
.....................acccuguagauccgaauuuguuAg..	14	1	MF2
.....................acccuguagauccgaauuuguuuAa...	15	1	MF2
.....................acccuguagauccgaauuuguuuAaa..	4	1	MF2
.....................acccuguagauccgaauuuguuugaau...	1	0	MF2
......................cccuguagauccgaauuugu...	5	0	MF2
......................Accuguagauccgaauuuguu..	1	1	MF2
......................cccuguagauccgaauuuguu..	16	0	MF2
......................cccuguagauccgaauuuguuA...	3	1	MF2
......................cccuguagauccgaauuuguuu...	3	0	MF2
.......................ccuguagauccgaauuugu...	7	0	MF2
.......................ccuguagauccgaauuuguu..	23	0	MF2
.......................ccuguagauccgaauuuguuu...	1	0	MF2
........................cuguagauccgaauuugu...	1	0	MF2
........................cuguagauccgaauuuguu..	2	0	MF2
.........................uguagauccgaauuuguu..	3	0	MF2
....caaauucgguucuagagag......................	7	0	MF2
....caaauucgguucuagagagA.....................	4	1	MF2
....caaauucgguucuagagagg.....................	21	0	MF2
....caaauucgguucuagagagUu....................	1	1	MF2
....caaauucggCucuagagaggu....................	1	1	MF2
....caaauucgguucuagagaggu....................	48	0	MF2
....caaauucgguucuagagagguC...................	1	1	MF2
....caaauucgguucuagagaggGu...................	1	1	MF2
....caaauucgguucuagagagguu...................	199	0	MF2
....Uaaauucgguucuagagagguuu..................	1	1	MF2
....caaauucgUuucuagagagguuu..................	1	1	MF2
....caaauucgguucuagagagguuG..................	1	1	MF2
....caGauucgguucuagagagguuu..................	1	1	MF2
....caaauucgguAcuagagagguuu..................	1	1	MF2
....caaauucgguucuagagagguuu..................	1783	0	MF2
....caaauucgguGcuagagagguuu..................	1	1	MF2
....caaauuAgguucuagagagguuu..................	1	1	MF2
....caaauucgguucCagagagguuu..................	2	1	MF2
....caaauucgguucuagagagguCu..................	1	1	MF2
....caaauucgguucuagagGgguuu..................	1	1	MF2
....caaauucgguucuagagagguAu..................	1	1	MF2
....caaauucgguuAuagagagguuu..................	1	1	MF2
....caaGuucgguucuagagagguuu..................	1	1	MF2
....caaauucggGucuagagagguuu..................	1	1	MF2
....caaauucgguucuagagaggCuu..................	2	1	MF2
....caaauucgguucuagagagguGu..................	1	1	MF2
....caaauucgguucuagaUagguuu..................	2	1	MF2
....caaaCucgguucuagagagguuu..................	2	1	MF2
....caaauucgguucuagagagguuC..................	1	1	MF2
....caaauucgguucuagagagUuuu..................	1	1	MF2
....caaauucgguucuagagaggGuu..................	2	1	MF2
....caaauucgguucuagaAagguuu..................	1	1	MF2
....caaauucgguucuaAagagguuu..................	1	1	MF2
....caaauucgguucuagagagguuuC.................	10	1	MF2
....caaauucgAuucuagagagguuug.................	1	1	MF2

```
                     aga-miR-10
                                                            aga-miR-10*
gucgauuuauguucuacauccacccuguagauccgaauuuguuugaauuuauauuaauaacaaauucgguucuagagagguuugugugggcauuuguuaac

............................................................caaauucgguucuagagagguuuA.................    8      1     MF2
............................................................caaauucgguucuagagagguuug.................    10     0     MF2
............................................................caaauucgguucuagagagguuuU.................    121    1     MF2
............................................................caaauucgguucuagagagguuuCu................    2      1     MF2
............................................................caaauucgguucuagagagguuuAu................    8      1     MF2
............................................................caaauucgguucuagagagguuuUu................    5      1     MF2
.............................................................Caauucgguucuagagagguuu..................    1      1     MF2
.............................................................aaauucgguucuagagagguuu..................    5      0     MF2
.............................................................aaauucgguucuagagagguuuU.................    2      1     MF2
.............................................................aaauucgguucuagagagguuug.................    1      0     MF2
.............................................................aaauucgguucuagagagguuugu................    1      0     MF2
..............................................................aauucgguucuagagagguuu..................    1      0     MF2

...................ccacccuguagauccgaauuugu...........................................................    1      0     FW2
...................Ucacccuguagauccgaauuuguu..........................................................    1      1     FW2
....................cacccuguagauccgaau...............................................................    1      0     FW2
....................cacccuguagauccgaauu..............................................................    1      0     FW2
....................cacccuguagauccgaauuug............................................................    24     0     FW2
....................cacccuguagauccgaauuugG...........................................................    1      1     FW2
....................cacccuCuagauccgaauuugu...........................................................    1      1     FW2
....................cacccuguagauccgaGuuugu...........................................................    1      1     FW2
....................cacccuguagauccUaauuugu...........................................................    1      1     FW2
....................cacccuguagauccgaauuugu...........................................................    970    0     FW2
....................cacccugGagauccgaauuugu...........................................................    1      1     FW2
....................cacccuguagaCccgaauuugu...........................................................    1      1     FW2
....................cacccuguagauccgaauuuguA..........................................................    1      1     FW2
....................cacccuguagauUcgaauuuguu..........................................................    1      1     FW2
....................cacccuguagauccgaauuuguu..........................................................    433    0     FW2
....................cacccuguagauccgaauuuguC..........................................................    1      1     FW2
....................cacccuguagauccAaauuuguu..........................................................    1      1     FW2
....................cacccuAuagauccgaauuuguu..........................................................    2      1     FW2
....................cacccuguagauccgaauuuguuu.........................................................    7      0     FW2
....................cacccuguagauccgaauuuguuA.........................................................    6      1     FW2
.....................acccuguagauccgaau...............................................................    1      0     FW2
.....................acccuguagauccgaauu..............................................................    5      0     FW2
.....................acccuguagauccgaauuu.............................................................    4      0     FW2
.....................acccuguagauccgaauuug............................................................    47     0     FW2
.....................acccuguagauccgaauuugG...........................................................    1      1     FW2
.....................acccuguagaCccgaauuugu...........................................................    2      1     FW2
.....................acccuguagaGccgaauuugu...........................................................    1      1     FW2
.....................acccuguagauccgaauuugu...........................................................    1188   0     FW2
.....................acccuguagauccgaaCuugu...........................................................    1      1     FW2
.....................acccuguagauUcgaauuugu...........................................................    1      1     FW2
.....................acccuAuagauccgaauuugu...........................................................    1      1     FW2
.....................acccuguagauccgaauuGgu...........................................................    1      1     FW2
.....................acccuguagauccgaauuuguC..........................................................    3      1     FW2
.....................acccuguagauccgaauuugAu..........................................................    1      1     FW2
.....................acccuguUgauccgaauuuguu..........................................................    1      1     FW2
.....................acccuguagauccgaauCuguu..........................................................    5      1     FW2
.....................acccuguagaucGgaauuuguu..........................................................    1      1     FW2
.....................accUuguagauccgaauuuguu..........................................................    1      1     FW2
.....................acccuguagauccgaauuugGu..........................................................    1      1     FW2
.....................acccuguagauccgaauuugCu..........................................................    2      1     FW2
.....................acccuguagauccAaauuuguu..........................................................    9      1     FW2
.....................aUccuguagauccgaauuuguu..........................................................    2      1     FW2
.....................acccuguagauUcgaauuuguu..........................................................    9      1     FW2
.....................acccuUuagauccgaauuuguu..........................................................    1      1     FW2
.....................acccCguagauccgaauuuguu..........................................................    4      1     FW2
.....................Gcccuguagauccgaauuuguu..........................................................    4      1     FW2
.....................acccuguagauAcgaauuuguu..........................................................    2      1     FW2
.....................acccuguagauccgaaCuuguu..........................................................    5      1     FW2
.....................acccuguagauccgaauuuAuu..........................................................    1      1     FW2
.....................acccuguagGuccgaauuuguu..........................................................    2      1     FW2
.....................acccuguagauccgaGuuuguu..........................................................    1      1     FW2
.....................acccuguagauccUaauuuguu..........................................................    1      1     FW2
.....................acccuguagauccgaauuuguA..........................................................    6      1     FW2
.....................acccuguagauccgaauuuguu..........................................................    10556  0     FW2
.....................acccuAuagauccgaauuuguu..........................................................    7      1     FW2
.....................acccuguagauccgaaAuuguu..........................................................    3      1     FW2
```

```
                     aga-miR-10
                                                            aga-miR-10*
gucgauuuauguucuacauccacccuguagauccgaauuuguuugaauuuauauuaauaacaaauucgguucuagagagguuuguguggggcauuuguuaac

.....................acccugCagauccgaauuuguu...........................................................    2     1     FW2
.....................acccuguaUauccgaauuuguu...........................................................    1     1     FW2
.....................acccuguagauccgaauuuguG...........................................................    2     1     FW2
.....................acccuguagauGcgaauuuguu...........................................................    4     1     FW2
.....................acccuguagauccgGauuuguu...........................................................    2     1     FW2
.....................acccuguagaucUgaauuuguu...........................................................    1     1     FW2
.....................acccuguagaCccgaauuuguu...........................................................    3     1     FW2
.....................acUcuguagauccgaauuuguu...........................................................    2     1     FW2
.....................acccuguaAauccgaauuuguu...........................................................    2     1     FW2
.....................acccuguagUuccgaauuuguu...........................................................    1     1     FW2
.....................acccuguagaAccgaauuuguu...........................................................    2     1     FW2
.....................acccAguagauccgaauuuguu...........................................................    1     1     FW2
.....................acccuguagauccgaauCuguuu..........................................................    1     1     FW2
.....................acccuguagauccgaauuuguuC..........................................................    3     1     FW2
.....................acccuguagauccgaauuuguuG..........................................................    3     1     FW2
.....................acccuguagauccgaauuuguuA..........................................................    325   1     FW2
.....................acccuguagGuccgaauuuguuu..........................................................    1     1     FW2
.....................acccuguagauccgaauuuguuu..........................................................    72    0     FW2
.....................acccuguagauccgaauuuguuuA.........................................................    2     1     FW2
.....................acccuguagauccgaauuuguuuga........................................................    1     0     FW2
.....................acccuguagauccgaauuuguuuAa........................................................    1     1     FW2
.......................ccuguagauccgaauuugu............................................................    2     0     FW2
.......................ccuguagauccgaauuuguu...........................................................    4     0     FW2
.........................uguagauccgaauuuguu...........................................................    1     0     FW2
............................................................caaauucgguucuagaga........................    1     0     FW2
............................................................caaauucgguucuagagagguu....................    3     0     FW2
............................................................caaauucgguucuagagagguuu...................    64    0     FW2
............................................................caaauucgguucuagagagguuuU..................    3     1     FW2

....................cacccuguagauccgaauu...............................................................    2     0     FF1
....................cacccuguagauccgaauuug.............................................................    11    0     FF1
....................cacccuguagauccgaauuugu............................................................    214   0     FF1
....................cacccuCuagauccgaauuugu............................................................    1     1     FF1
....................cacUcuguagauccgaauuugu............................................................    2     1     FF1
....................caccUuguagauccgaauuugu............................................................    1     1     FF1
....................cacccugAagauccgaauuugu............................................................    1     1     FF1
....................cacccuguagauccgaauuugC............................................................    1     1     FF1
....................cacccuguagauccgaauuuguA...........................................................    1     1     FF1
....................cacccuguagauccgaauuuguu...........................................................    110   0     FF1
....................cacccuguagauccgaauuuguuu..........................................................    3     0     FF1
....................cacccuguagauccgaauuuguuA..........................................................    1     1     FF1
....................cacccuguagauccgaauuuguuAg.........................................................    1     1     FF1
.....................acccuguagauccgaauuug.............................................................    15    0     FF1
.....................acccuguagauccgaauCugu............................................................    1     1     FF1
.....................aUccuguagauccgaauuugu............................................................    1     1     FF1
.....................acccuguagauccAaauuugu............................................................    1     1     FF1
.....................acccuguagauccgaauuugu............................................................    315   0     FF1
.....................Gcccuguagauccgaauuuguu...........................................................    1     1     FF1
.....................acccuguagauccgaauuuguA...........................................................    3     1     FF1
.....................acccuguagauccgaauuuguu...........................................................    1377  0     FF1
.....................acccuguagaCccgaauuuguu...........................................................    2     1     FF1
.....................acccuguagaucUgaauuuguu...........................................................    1     1     FF1
.....................acccuguagauccgaauuuguG...........................................................    1     1     FF1
.....................Ucccuguagauccgaauuuguu...........................................................    1     1     FF1
.....................acccuguagaucAgaauuuguu...........................................................    1     1     FF1
.....................acccuguagauccgaauuugCu...........................................................    1     1     FF1
.....................acccuguagauccgaauuuguuu..........................................................    3     0     FF1
.....................acccuguagauccgaauuuguuC..........................................................    1     1     FF1
.....................acccuguagauccgaauuuguuG..........................................................    1     1     FF1
.....................acccuguagauccgaauuuguuA..........................................................    70    1     FF1
.....................acccuguagauccgaauuuguuuAa........................................................    1     1     FF1
......................cccuguagauccgaauuuguu...........................................................    1     0     FF1
.......................ccuguagauccgaauuugu............................................................    1     0     FF1
.......................ccuguagauccgaauuuguu...........................................................    1     0     FF1
............................................................caaauucgguucuagaAa........................    1     1     FF1
............................................................caaauucgguucuagagagguu....................    3     0     FF1
............................................................caaauucgguucuagagaggGuu...................    1     1     FF1
............................................................caaauucgguucuagagagguuu...................    24    0     FF1
............................................................caaauucgguucuagagagguuuU..................    2     1     FF1
```

aga-miR-10

aga-miR-10*

gucgauuuauguucuacauccacccuguagauccgaauuuguuugaauuuauauuaauaacaaauucgguucuagagagguuugugugggcauuuguuaac

....................cacccuguagauccgaa..	1	0	OV1
....................cacccuguagauccgaauu..	5	0	OV1
....................cacccuguagauccgaauuu...	1	0	OV1
....................cacccuguagauccgaauuug..	21	0	OV1
....................cacccuguagauccgaauCugu...	1	1	OV1
....................cacccuguagauccgaauuugu...	353	0	OV1
....................cacccuguagauccgaauuugA...	1	1	OV1
....................caUccuguagauccgaauuugu...	1	1	OV1
....................cacccuguagGuccgaauuuguu..	1	1	OV1
....................cacccuguagauccgaauuuguu..	131	0	OV1
....................caccUuguagauccgaauuuguu..	1	1	OV1
....................cacccuguagauccgaauuuguA..	1	1	OV1
....................Uacccuguagauccgaauuuguu..	1	1	OV1
....................cacccuguagauccgaauuuguAu...	1	1	OV1
....................cacccuguagauccgaauuuguuA...	2	1	OV1
....................cacccuguagauccgaauuuguuu...	1	0	OV1
.....................acccuguagauccgaau...	1	0	OV1
.....................acccuguagauccgaauu..	3	0	OV1
.....................acccuguagauccgaauuu...	5	0	OV1
.....................acccuguagaGccgaauuug..	1	1	OV1
.....................acccuguagauccgaauuug..	48	0	OV1
.....................acccuguagauGcgaauuugu...	1	1	OV1
.....................acccuguagauccgaauCugu...	1	1	OV1
.....................acccuguaAauccgaauuugu...	1	1	OV1
.....................acUcuguagauccgaauuugu...	1	1	OV1
.....................acccuguagauccgaauuugu...	623	0	OV1
.....................acccuguagauUcgaauuugu...	1	1	OV1
.....................acccuguagUuccgaauuugu...	1	1	OV1
.....................acccuguagaucAgaauuuguu..	1	1	OV1
.....................acccuguagauccgaGuuuguu..	1	1	OV1
.....................acccuguagauccgaauuCguu..	1	1	OV1
.....................acccuguagauccgaauuuguu..	2007	0	OV1
.....................acccuguagauccgaauCuguu..	3	1	OV1
.....................acccuguagauccgaauuuguA..	1	1	OV1
.....................acccuguagauccgaaCuuguu..	2	1	OV1
.....................acccuguagauUcgaauuuguu..	1	1	OV1
.....................acccugCagauccgaauuuguu..	1	1	OV1
.....................acccCguagauccgaauuuguu..	2	1	OV1
.....................Ucccuguagauccgaauuuguu..	2	1	OV1
.....................acccuguagauccgaauuuguuA...	80	1	OV1
.....................acccuguagauccgaauuuguuC...	1	1	OV1
.....................acccCguagauccgaauuuguuu...	1	1	OV1
.....................acccuguagauccgaauuuguuu...	28	0	OV1
.....................acccuguagauccgaauuuguuuA..	1	1	OV1
.....................acccuguagauccgaauuuguuuAa...	1	1	OV1
.....................acccuguagauccgaauuuguuugaauu..	1	0	OV1
......................Accuguagauccgaauuuguu..	1	1	OV1
....caaauucgguucuagagagg.....................	1	0	OV1
....caaauucgguucuagagaggu....................	2	0	OV1
....caaauucgguucuagagagguu...................	9	0	OV1
....caaauucgguAcuagagagguu...................	1	1	OV1
....caaauucgguucuagagagguuu..................	70	0	OV1
....caaauucgguucuagagagguuuU.................	8	1	OV1
....caaauucgguucuagagagguuug.................	1	0	OV1
....caaauucgguucuagagagguuuUu................	1	1	OV1
....................cacccuguagauccgaau...	1	0	MF1
....................cacccuguagauccgaauu..	3	0	MF1
....................cacccuguagauccgaauuu...	3	0	MF1
....................cacccuguagauccgaauuug..	97	0	MF1
....................cacGcuguagauccgaauuug..	1	1	MF1
....................cacccuguaAauccgaauuugu...	1	1	MF1
....................Uacccuguagauccgaauuugu...	8	1	MF1
....................cacccuguaCauccgaauuugu...	1	1	MF1
....................caccUuguagauccgaauuugu...	1	1	MF1
....................cacccuAuagauccgaauuugu...	1	1	MF1
....................caUccuguagauccgaauuugu...	1	1	MF1
....................cacccuguagauccgaauuugA...	3	1	MF1

aga-miR-10

aga-miR-10*

gucgauuuauguucuacauccacccuguagauccgaauuuguuugaauuuauauuaauaacaaauucgguucuagagagguuugugugggcauuuguuaac

....................cacccuguagauccgaGuuugu...	1	1	MF1
....................cGcccuguagauccgaauuugu...	1	1	MF1
....................cacccuguagauccgaauuugC...	1	1	MF1
....................Aacccuguagauccgaauuugu...	1	1	MF1
....................cacccuguagauccCaauuugu...	1	1	MF1
....................cacccuguagauccgaauuugu...	1639	0	MF1
....................cacccuguagauccgaauuugG...	3	1	MF1
....................cacccuguagaucAgaauuugu...	1	1	MF1
....................cacccugAagauccgaauuugu...	1	1	MF1
....................cacccuguagaAccgaauuugu...	1	1	MF1
....................cacUcuguagauccgaauuuguu..	1	1	MF1
....................cacccuguagauccgaauuuUuu..	1	1	MF1
....................cacccuguaAauccgaauuuguu..	1	1	MF1
....................cacccuguagauccgaauuuguA..	10	1	MF1
....................cacccuguagauccgaauuuguG..	1	1	MF1
....................cacccuguagaCccgaauuuguu..	1	1	MF1
....................cacccuguagauccgaauuugCu..	1	1	MF1
....................cacccuguagauccgaauuuguu..	603	0	MF1
....................cacccuguagauccgaauuugAu..	1	1	MF1
....................cacccuguagCuccgaauuuguu..	1	1	MF1
....................cacccuguagauccgaauuuguuu...	15	0	MF1
....................cacccuguagauccgaauuuguuA...	13	1	MF1
.....................acccuguagauccgaau...	1	0	MF1
.....................acccuguagauccgaauu..	19	0	MF1
.....................acccuguagauccgaauuu...	18	0	MF1
.....................acccuguagauccgaauuug..	141	0	MF1
.....................acccGguagauccgaauuug..	1	1	MF1
.....................acccuguagauccUaauuug..	1	1	MF1
.....................acccuguagauUcgaauuugu...	5	1	MF1
.....................acccuguagauccgaauuugG...	2	1	MF1
.....................acccuguagauccgaauCugu...	3	1	MF1
.....................acccuguagauccgaauuugC...	1	1	MF1
.....................acccuguagaucUgaauuugu...	1	1	MF1
.....................acccuguagauccgaauuugA...	2	1	MF1
.....................acccugGagauccgaauuugu...	1	1	MF1
.....................acccuguagauccgaaCuugu...	1	1	MF1
.....................acccuguagauccgaauuCgu...	1	1	MF1
.....................acccuguagauccAaauuugu...	2	1	MF1
.....................acccuguagauccgaauuuAu...	3	1	MF1
.....................acccuguagauccgaauuugu...	2699	0	MF1
.....................acccCguagauccgaauuugu...	1	1	MF1
.....................acAcuguagauccgaauuugu...	1	1	MF1
.....................acccuguagauAcgaauuugu...	1	1	MF1
.....................acccuguagaCccgaauuugu...	1	1	MF1
.....................acccuguagauccgaauuAgu...	1	1	MF1
.....................acccuguagauGcgaauuugu...	2	1	MF1
.....................acccugAagauccgaauuugu...	1	1	MF1
.....................Ccccuguagauccgaauuugu...	1	1	MF1
.....................acccuguagauccUaauuuguu..	1	1	MF1
.....................acccuguagauccgaaCuuguu..	5	1	MF1
.....................acccuAuagauccgaauuuguu..	3	1	MF1
.....................acccuguagauccgaUuuuguu..	1	1	MF1
.....................acccuguagauccgaauCuguu..	5	1	MF1
.....................acccuguagauUcgaauuuguu..	9	1	MF1
.....................aAccuguagauccgaauuuguu..	1	1	MF1
.....................acccuguagaucUgaauuuguu..	3	1	MF1
.....................acccuguagauccgaauuuAuu..	3	1	MF1
.....................Ucccuguagauccgaauuuguu..	1	1	MF1
.....................acccuguagauccgaaGuuguu..	3	1	MF1
.....................acccuguagaGccgaauuuguu..	1	1	MF1
.....................accGuguagauccgaauuuguu..	1	1	MF1
.....................acccuguagauccgaauuuguG..	9	1	MF1
.....................acccuguagaucGgaauuuguu..	1	1	MF1
.....................acccuguagauGcgaauuuguu..	4	1	MF1
.....................acccuguagauccgGauuuguu..	1	1	MF1
.....................acccugCagauccgaauuuguu..	5	1	MF1
.....................acccuguagauAcgaauuuguu..	10	1	MF1
.....................acccuguagCuccgaauuuguu..	1	1	MF1
.....................acccuguagauccgaauuugCu..	6	1	MF1

aga-miR-10

aga-miR-10*

gucgauuuauguucuacauccacccuguagauccgaauuuguuugaauuuauauuaauaacaaauucgguucuagagagguuuguguggggcauuuguuaac

.....................acccuguagauccAaauuuguu...	4	1	MF1
.....................aUccuguagauccgaauuuguu...	1	1	MF1
.....................acccuguagauccgaauuuguC...	4	1	MF1
.....................acccuguagaAccgaauuuguu...	2	1	MF1
.....................acUcuguagauccgaauuuguu...	4	1	MF1
.....................acccuguagauccgCauuuguu...	1	1	MF1
.....................acccuguagauccgaauuuguu...	10015	0	MF1
.....................acccuguagauccgaauuuguA...	9	1	MF1
.....................acccuguagGuccgaauuuguu...	2	1	MF1
.....................acccuguagaCccgaauuuguu...	1	1	MF1
.....................Gcccuguagauccgaauuuguu...	1	1	MF1
.....................acccuguagUuccgaauuuguu...	1	1	MF1
.....................acccugGagauccgaauuuguu...	2	1	MF1
.....................acccCguagauccgaauuuguu...	7	1	MF1
.....................acccuguaAauccgaauuuguu...	4	1	MF1
.....................acAcuguagauccgaauuuguu...	3	1	MF1
.....................acccuguagaucAgaauuuguu...	2	1	MF1
.....................acccuguagauccgaauuCguu...	3	1	MF1
.....................acccuguaUauccgaauuuguu...	1	1	MF1
.....................accUuguagauccgaauuuguu...	1	1	MF1
.....................acccuguGgauccgaauuuguu...	2	1	MF1
.....................acccuguagauccgaGuuuguu...	3	1	MF1
.....................aUccuguagauccgaauuuguuu..	1	1	MF1
.....................acccuguagauccgaauuuguuu..	116	0	MF1
.....................acccuguagauccgaauuuguuA..	425	1	MF1
.....................acccuguagauUcgaauuuguuu..	1	1	MF1
.....................acccuguagauccgaauuuguuG..	3	1	MF1
.....................acccuguagauccgaauuuguuC..	3	1	MF1
.....................acccuguagauccgaauuuguuuA...	1	1	MF1
.....................acccuguagauccgaauuuguuAg...	2	1	MF1
.....................acccuguagauccgaauuuguuuU...	1	1	MF1
.....................acccuguagauccgaauuuguuuAa..	1	1	MF1
.....................acccuguagauccgaauuuguuugaauuuauauuaauaa..	1	0	MF1
......................cccuguagauccgaauuuguu...	1	0	MF1
.......................ccuguagauccgaauuugu..	1	0	MF1
.......................ccuguagauccgaauuuguu...	3	0	MF1
.......................ccuguagauccgaauuuguuA..	1	1	MF1
....caaauucgguucuagagag.......................	2	0	MF1
....caaauucgguucuagagagg......................	4	0	MF1
....caaauucgguucuagagaggu.....................	4	0	MF1
....caaauucgguucuagagagguG....................	1	1	MF1
....caaauucgguucuagagagguu....................	31	0	MF1
....caaauuUgguucuagagagguu....................	1	1	MF1
....caaauucgguucuagagagguuu...................	229	0	MF1
....caaauucgguucuagagagguCu...................	1	1	MF1
....caaauucgguucuagagagguuuC..................	3	1	MF1
....caaauucgguucuagagagguuuU..................	12	1	MF1
....caaauucgguucuagagagguuug..................	2	0	MF1
....caaauucgguucuagagagguuuUu.................	1	1	MF1
....caaauucgguucuagagagguuuAu.................	1	1	MF1
....aaauucgguucuagagagguuuU..................	1	1	MF1
....................cacccuguagauccgaauuug...	4	0	BF2
....................cacccugAagauccgaauuugu..	1	1	BF2
....................Uacccuguagauccgaauuugu..	2	1	BF2
....................cacccuguagauccgaauuugu..	160	0	BF2
....................cacccuguagauccgaauuuguu...	51	0	BF2
....................cacccuguagauccgaauuuguA...	1	1	BF2
....................cacccuguagauccgaauuuguuA..	3	1	BF2
....................cacccuguagauccgaauuuguuu..	2	0	BF2
.....................acccuguagauccgaauuu..	3	0	BF2
.....................acccuguagauccgaauuug...	11	0	BF2
.....................acccuguagauAcgaauuugu..	1	1	BF2
.....................acccuguagauccAaauuugu..	1	1	BF2
.....................acccuguagauccgaauuugu..	261	0	BF2
.....................acccuguagauccgGauuuguu...	1	1	BF2
.....................acccuguGgauccgaauuuguu...	1	1	BF2
.....................acccuguagauccAaauuuguu...	1	1	BF2
.....................acccugCagauccgaauuuguu...	1	1	BF2

```
                    aga-miR-10
                                        aga-miR-10*
gucgauuuauguucuacauccacccuguagauccgaauuuguuugaauuuauauuaauaacaaauucgguucuagagagguuuguguggggcauuuguuaac

.....................acccuguagaGccgaauuuguu...........................................................   1     1   BF2
.....................aUccuguagauccgaauuuguu...........................................................   1     1   BF2
.....................acccuUuagauccgaauuuguu...........................................................   1     1   BF2
.....................acccuguagauccgaauuuguu...........................................................   1178  0   BF2
.....................acccCguagauccgaauuuguu...........................................................   1     1   BF2
.....................acccuguagauccgaauuugAu...........................................................   1     1   BF2
.....................accAuguagauccgaauuuguu...........................................................   1     1   BF2
.....................acccuguagauGcgaauuuguu...........................................................   1     1   BF2
.....................acccuguagauccgaauuuguuG..........................................................   1     1   BF2
.....................acccuguagauccgaauuuguuC..........................................................   1     1   BF2
.....................acccuguagauccgaauuuguuu..........................................................   6     0   BF2
.....................acccuguagauccgaauuuguuA..........................................................   66    1   BF2
......................cccuguagauccgaauuuguu...........................................................   1     0   BF2
.......................ccuguagauccgaauuuguu...........................................................   1     0   BF2
........................cuguagauccgaauuuguu...........................................................   2     0   BF2
.........................uguagauccgaauuuguu...........................................................   1     0   BF2
............................................................caaauucgguucuagagaggu.....................   1     0   BF2
............................................................caaauucgguucuagagagguu....................   9     0   BF2
............................................................caaauucgguucuagagagguuu...................   51    0   BF2
............................................................caaauucgguucuGgagagguuu...................   1     1   BF2
............................................................caaauucgguucuagagagguuuU..................   2     1   BF2
............................................................caaauucgguucuagagagguuug..................   1     0   BF2

....................cacccuguagauccgaauu...............................................................   2     0   BF1
....................cacccuguagauccgaauuug.............................................................   11    0   BF1
....................cacccuguagauccgaauuugu............................................................   214   0   BF1
....................caccUuguagauccgaauuugu............................................................   1     1   BF1
....................cacccugAagauccgaauuugu............................................................   1     1   BF1
....................cacccuCuagauccgaauuugu............................................................   1     1   BF1
....................cacccuguagauccgaauuugC............................................................   1     1   BF1
....................cacUcuguagauccgaauuugu............................................................   2     1   BF1
....................cacccuguagauccgaauuuguA...........................................................   1     1   BF1
....................cacccuguagauccgaauuuguu...........................................................   109   0   BF1
....................cacccuguagauccgaauuuguuu..........................................................   3     0   BF1
....................cacccuguagauccgaauuuguuA..........................................................   1     1   BF1
....................cacccuguagauccgaauuuguuAg.........................................................   1     1   BF1
.....................acccuguagauccgaauuug.............................................................   15    0   BF1
.....................acccuguagauccgaauCugu............................................................   1     1   BF1
.....................aUccuguagauccgaauuugu............................................................   1     1   BF1
.....................acccuguagauccAaauuugu............................................................   1     1   BF1
.....................acccuguagauccgaauuugu............................................................   313   0   BF1
.....................acccuguagaCccgaauuuguu...........................................................   2     1   BF1
.....................Gcccuguagauccgaauuuguu...........................................................   1     1   BF1
.....................acccuguagauccgaauuugCu...........................................................   1     1   BF1
.....................acccuguagaucUgaauuuguu...........................................................   1     1   BF1
.....................acccuguagauccgaauuuguG...........................................................   1     1   BF1
.....................acccuguagauccgaauuuguA...........................................................   3     1   BF1
.....................acccuguagauccgaauuuguu...........................................................   1375  0   BF1
.....................acccuguagaucAgaauuuguu...........................................................   1     1   BF1
.....................acccuguagauccgaauuuguuu..........................................................   3     0   BF1
.....................acccuguagauccgaauuuguuA..........................................................   70    1   BF1
.....................acccuguagauccgaauuuguuC..........................................................   1     1   BF1
.....................acccuguagauccgaauuuguuG..........................................................   1     1   BF1
.....................acccuguagauccgaauuuguuAa.........................................................   1     1   BF1
......................cccuguagauccgaauuuguu...........................................................   1     0   BF1
.......................ccuguagauccgaauuugu............................................................   1     0   BF1
.......................ccuguagauccgaauuuguu...........................................................   1     0   BF1
............................................................caaauucgguucuagaAa........................   1     1   BF1
............................................................caaauucgguucuagagagguu....................   3     0   BF1
............................................................caaauucgguucuagagaggGuu...................   1     1   BF1
............................................................caaauucgguucuagagagguuu...................   24    0   BF1
............................................................caaauucgguucuagagagguuuU..................   2     1   BF1

...................ccacccuguagauccgaauuugu............................................................   1     0   MW1
....................cacccuguagauccgaau................................................................   3     0   MW1
....................cacccuguagauccgaauuug.............................................................   18    0   MW1
....................cacccuguagauccgaauuuCu............................................................   1     1   MW1
....................cacccuguagaGccgaauuugu............................................................   1     1   MW1
....................cacccuguagauccgaauuugu............................................................   639   0   MW1
```

```
                     aga-miR-10
                                             aga-miR-10*
gucgauuuauguucuacauccacccuguagauccgaauuuguuugaauuuauauuaauaacaaauucgguucuagagagguuuguguggggcauuuguuaac

....................cacccuguagCuccgaauuugu............................................................    1      1     MW1
....................cacccuguagauccgaauuugC............................................................    1      1     MW1
....................cacccuAuagauccgaauuugu............................................................    1      1     MW1
....................cacccCguagauccgaauuuguu...........................................................    1      1     MW1
....................cacccuguagauccgaauAuguu...........................................................    1      1     MW1
....................cacccuguagauccgaaCuuguu...........................................................    1      1     MW1
....................cacccuguagauccgaauuuguu...........................................................    241    0     MW1
....................cacccuguagauccgaauuuguuA..........................................................    5      1     MW1
....................cacccuguagauccgaauuuUuuu..........................................................    1      1     MW1
....................cacccuguagauccgaauuuguuu..........................................................    2      0     MW1
.....................acccuguagauccgaau................................................................    2      0     MW1
.....................acccuguagauccgaauu...............................................................    8      0     MW1
.....................acccuguagauccgaauuu..............................................................    14     0     MW1
.....................acccuguagauccgaauuug.............................................................    48     0     MW1
.....................acccuguagaCccgaauuugu............................................................    1      1     MW1
.....................acccuguagaucUgaauuugu............................................................    1      1     MW1
.....................acccuguagaAccgaauuugu............................................................    1      1     MW1
.....................acccuguagauccgaauuuAu............................................................    1      1     MW1
.....................acccuguagUuccgaauuugu............................................................    1      1     MW1
.....................aUccuguagauccgaauuugu............................................................    1      1     MW1
.....................acccuguagauccgaauuCgu............................................................    1      1     MW1
.....................acccuguagauccgaauuugu............................................................    829    0     MW1
.....................acccuguagauccgaauuugCu...........................................................    5      1     MW1
.....................aUccuguagauccgaauuuguu...........................................................    1      1     MW1
.....................acAcuguagauccgaauuuguu...........................................................    1      1     MW1
.....................acccuguagauccgaauuuAuu...........................................................    1      1     MW1
.....................acccuguagaCccgaauuuguu...........................................................    3      1     MW1
.....................accUuguagauccgaauuuguu...........................................................    1      1     MW1
.....................acccugCagauccgaauuuguu...........................................................    1      1     MW1
.....................acccuguagauccgGauuuguu...........................................................    1      1     MW1
.....................acccuguagGuccgaauuuguu...........................................................    1      1     MW1
.....................acccuguagauccgaauCuguu...........................................................    3      1     MW1
.....................acccuguUgauccgaauuuguu...........................................................    1      1     MW1
.....................acccuguagauccgaauuCguu...........................................................    1      1     MW1
.....................acGcuguagauccgaauuuguu...........................................................    1      1     MW1
.....................acccuguagauccgaauuuguu...........................................................    6268   0     MW1
.....................aAccuguagauccgaauuuguu...........................................................    2      1     MW1
.....................acccuguagaucUgaauuuguu...........................................................    1      1     MW1
.....................acccuguagauccgaauuugGu...........................................................    1      1     MW1
.....................acccuguagauccgaaCuuguu...........................................................    3      1     MW1
.....................acccCguagauccgaauuuguu...........................................................    6      1     MW1
.....................acccuguagauccgaGuuuguu...........................................................    1      1     MW1
.....................acccuUuagauccgaauuuguu...........................................................    2      1     MW1
.....................acccuguagauGcgaauuuguu...........................................................    3      1     MW1
.....................acccuguagaGccgaauuuguu...........................................................    1      1     MW1
.....................acccuguagauccgaauuuguC...........................................................    3      1     MW1
.....................acccuAuagauccgaauuuguu...........................................................    1      1     MW1
.....................acccuguagauccgaauuuguA...........................................................    3      1     MW1
.....................acccuguagauUcgaauuuguu...........................................................    1      1     MW1
.....................acccuguagauccgaauuuguG...........................................................    2      1     MW1
.....................acccuguagauccAaauuuguu...........................................................    5      1     MW1
.....................acccuguagauccgaauuuguuu..........................................................    44     0     MW1
.....................acccuguagauccgaauuuguuA..........................................................    204    1     MW1
.....................acccuguagauccgaauuuguuC..........................................................    2      1     MW1
.....................acccuguagauccgaauuuguuG..........................................................    3      1     MW1
.....................acccuguagauccgaauuuguuAg.........................................................    1      1     MW1
.....................acccuguagauccgaauuuguuuA.........................................................    1      1     MW1
.....................acccuguagauccgaauuuguuuU.........................................................    1      1     MW1
.....................acccuguagauccgaauuuguuuAaa.......................................................    1      1     MW1
.....................acccuguagauccgaauuuguuugaauuuauauuaauaa..........................................    1      0     MW1
......................cccuguagauccgaauuuguu...........................................................    2      0     MW1
.......................ccuguagauccgaauuuguu...........................................................    1      0     MW1
.........................uguagauccgaauuugu............................................................    3      0     MW1
..........................guagauccgaauuuguu...........................................................    1      0     MW1
............................................................caaauucgguucuagagagguu....................    11     0     MW1
............................................................caaauucgguucuagagagguuu...................    74     0     MW1
............................................................caaauucgguucuagagagguuC...................    1      1     MW1
............................................................caaauucgguucuagagagguuuU..................    1      1     MW1

....................cacccuguagauccgaauu...............................................................    1      0     FW1
```

aga-miR-10

aga-miR-10*

```
gucgauuuauguucuacauccacccuguagauccgaauuuguuugaauuuauauuaauaacaaauucgguucuagagagguuugugugggcauuuguuaac

....................cacccuguagauccgaauuu.............................................................    1      0    FW1
....................cacccuguagauccgaauuug............................................................    19     0    FW1
....................Gacccuguagauccgaauuugu...........................................................    1      1    FW1
....................cacccuguagaCccgaauuugu...........................................................    1      1    FW1
....................cacccuguagauccgaauuugu...........................................................    487    0    FW1
....................cacccuguagCuccgaauuugu...........................................................    1      1    FW1
....................cacccuguagauccgaauuugG...........................................................    1      1    FW1
....................cacccuguagauccAaauuugu...........................................................    1      1    FW1
....................cacccuguagauccgaauuuguA..........................................................    1      1    FW1
....................cacccuguagauccgaauuuguu..........................................................    195    0    FW1
....................cacccuguagauUcgaauuuguu..........................................................    1      1    FW1
....................cacccuguagauccgaauuuguuA.........................................................    6      1    FW1
.....................acccuguagauccgaau...............................................................    1      0    FW1
.....................acccuguagauccgaauu..............................................................    2      0    FW1
.....................acccuguagauccgaauuu.............................................................    6      0    FW1
.....................acccuguagauccgaauuug............................................................    19     0    FW1
.....................acccuguagauccgaauuugu...........................................................    535    0    FW1
.....................acccuguagauccgaauuugA...........................................................    1      1    FW1
.....................acccuguagauccgaaCuugu...........................................................    1      1    FW1
.....................acccuguagauAcgaauuuguu..........................................................    5      1    FW1
.....................acccuguagaucAgaauuuguu..........................................................    1      1    FW1
.....................acccuAuagauccgaauuuguu..........................................................    4      1    FW1
.....................acccuguagaAccgaauuuguu..........................................................    1      1    FW1
.....................acccuguagauccgaauuugCu..........................................................    5      1    FW1
.....................acccuguagauccgaGuuuguu..........................................................    1      1    FW1
.....................acccuguagauccgaauuCguu..........................................................    3      1    FW1
.....................acccugAagauccgaauuuguu..........................................................    1      1    FW1
.....................acccuguagauUcgaauuuguu..........................................................    3      1    FW1
.....................acccugCagauccgaauuuguu..........................................................    2      1    FW1
.....................Gcccuguagauccgaauuuguu..........................................................    2      1    FW1
.....................acccuguGgauccgaauuuguu..........................................................    1      1    FW1
.....................acccuguagauccgaaCuuguu..........................................................    1      1    FW1
.....................acccuguagauccgaauuuguC..........................................................    3      1    FW1
.....................acccuguagauccAaauuuguu..........................................................    2      1    FW1
.....................acccCguagauccgaauuuguu..........................................................    2      1    FW1
.....................acccuguagauccUaauuuguu..........................................................    1      1    FW1
.....................acccuguagauccgaauuugGu..........................................................    1      1    FW1
.....................acccuguagauccgaauuuguG..........................................................    4      1    FW1
.....................acccuguagauccgaauuuguu..........................................................    4229   0    FW1
.....................acccuguagaGccgaauuuguu..........................................................    2      1    FW1
.....................acccuguagauccgaauuuguA..........................................................    2      1    FW1
.....................acccuUuagauccgaauuuguu..........................................................    1      1    FW1
.....................acccuguagauGcgaauuuguu..........................................................    1      1    FW1
.....................acccuguagaCccgaauuuguu..........................................................    3      1    FW1
.....................acccuguagauccgaauuuguuu.........................................................    26     0    FW1
.....................acccuguagauccgaauuuguuG.........................................................    4      1    FW1
.....................acccuguagauccgaauuuguuA.........................................................    115    1    FW1
.....................acccuguagauccgaauuuguuC.........................................................    1      1    FW1
.....................acccuguagauccgaauuuguuuA........................................................    1      1    FW1
.....................acccuguagauccgaauuuguuugaa......................................................    1      0    FW1
.......................ccuguagauccgaauuuguu..........................................................    4      0    FW1
............................................................caaauucgguucuagagaggu....................    2      0    FW1
............................................................caaauucgguucuagagagguu...................    2      0    FW1
............................................................caaauucgguucuagagagguuu..................    28     0    FW1
............................................................caaauucgguucuagagagguuuU.................    1      1    FW1

....................cacccuguagauccgaauu..............................................................    2      0    MW2
....................cacccuguagauccgaauuu.............................................................    1      0    MW2
....................cacccuguagauccgaauuug............................................................    7      0    MW2
....................cacccuguagauccgaauuugG...........................................................    1      1    MW2
....................cacccuguagauccgaauGugu...........................................................    1      1    MW2
....................cacccuguagauccgaauuugu...........................................................    301    0    MW2
....................cacccuguagGuccgaauuugu...........................................................    2      1    MW2
....................cacccuguagauccgaauuuguA..........................................................    1      1    MW2
....................cacccuguagauccgaauuuguG..........................................................    1      1    MW2
....................cacAcuguagauccgaauuuguu..........................................................    1      1    MW2
....................cacccuguagauccgaauuuguu..........................................................    99     0    MW2
....................cacccuguagauccgaauuuguuA.........................................................    6      1    MW2
.....................acccuguagauccgaau...............................................................    1      0    MW2
```

```
                     aga-miR-10
                                                            aga-miR-10*
gucgauuuauguucuacauccacccuguagauccgaauuuguuugaauuuauauuaauaacaaauucgguucuagagagguuugugugggcauuuguuaac

.....................acccuguagauccgaauu..............................................................   4      0     MW2
.....................acccuguagauccgaauuu.............................................................   3      0     MW2
.....................Ucccuguagauccgaauuug............................................................   1      1     MW2
.....................acccuguagauccgaauuug............................................................   12     0     MW2
.....................acccugCagauccgaauuugu...........................................................   1      1     MW2
.....................acccuguagauccgaGuuugu...........................................................   1      1     MW2
.....................Gcccuguagauccgaauuugu...........................................................   1      1     MW2
.....................acccuguagauccAaauuugu...........................................................   1      1     MW2
.....................acccuguagauccgaauCugu...........................................................   4      1     MW2
.....................acccuguagauccgaauuugu...........................................................   382    0     MW2
.....................acccuguagauccgaauCuguu..........................................................   1      1     MW2
.....................acccuguagauUcgaauuuguu..........................................................   2      1     MW2
.....................acccuguagauccgaaCuuguu..........................................................   2      1     MW2
.....................acccuguagauccgaauuuguA..........................................................   1      1     MW2
.....................acccugAagauccgaauuuguu..........................................................   1      1     MW2
.....................Ucccuguagauccgaauuuguu..........................................................   1      1     MW2
.....................acccuguagauccgaauuuguu..........................................................   2902   0     MW2
.....................acccugGagauccgaauuuguu..........................................................   1      1     MW2
.....................acccuguagaucUgaauuuguu..........................................................   2      1     MW2
.....................acccuguagauccAaauuuguu..........................................................   2      1     MW2
.....................acccuguagauccgaauuuguG..........................................................   2      1     MW2
.....................acccuguagauccgaUuuuguu..........................................................   1      1     MW2
.....................acccuguGgauccgaauuuguu..........................................................   2      1     MW2
.....................acccuguagaucAgaauuuguu..........................................................   1      1     MW2
.....................acccCguagauccgaauuuguu..........................................................   1      1     MW2
.....................acccuguagauccUaauuuguu..........................................................   1      1     MW2
.....................acUcuguagauccgaauuuguu..........................................................   1      1     MW2
.....................acccuguagaCccgaauuuguu..........................................................   2      1     MW2
.....................acccuguagauccgaauGuguu..........................................................   1      1     MW2
.....................acccuAuagauccgaauuuguu..........................................................   3      1     MW2
.....................acccuguagauccgaauuuguuA.........................................................   105    1     MW2
.....................acccuguagauccgaauuuguuC.........................................................   1      1     MW2
.....................acccuguagauccgaauuuguuu.........................................................   17     0     MW2
.........................uguagauccgaauuugu...........................................................   1      0     MW2
............................................................caaauucgguucuagagaggu....................   1      0     MW2
............................................................caaauucgguucuagagagguu...................   3      0     MW2
............................................................caaauucgguucuagagagguuu..................   14     0     MW2
............................................................caaauucgguucuagagagguuuU.................   1      1     MW2
............................................................caaauucgguucuagagagguuug.................   1      0     MW2

....................cacccuguagauccgaauuug............................................................   18     0     TE2
....................cacccuguagauccgaauuugu...........................................................   417    0     TE2
....................cacccuguagauccgaauuugG...........................................................   1      1     TE2
....................cacccCguagauccgaauuugu...........................................................   1      1     TE2
....................cacccuguagCuccgaauuugu...........................................................   1      1     TE2
....................cacccuguagauccgaauuuguA..........................................................   1      1     TE2
....................cacccuguagauccgaauuuguu..........................................................   122    0     TE2
....................cacccuguagauccgaauuuguuA.........................................................   2      1     TE2
.....................acccuguagauccgaauu..............................................................   4      0     TE2
.....................acccuguagauccgaauuu.............................................................   5      0     TE2
.....................acccuAuagauccgaauuug............................................................   1      1     TE2
.....................acccuguagauccgaauuug............................................................   31     0     TE2
.....................acccuguagauccgaauuugu...........................................................   580    0     TE2
.....................acccuguagauccAaauuugu...........................................................   1      1     TE2
.....................acccuguagauccgaaCuugu...........................................................   1      1     TE2
.....................acccuguagauccgaauCugu...........................................................   1      1     TE2
.....................acccuguagauccgaauuugCu..........................................................   1      1     TE2
.....................acccuAuagauccgaauuuguu..........................................................   1      1     TE2
.....................acccuguagauccgaauuuguC..........................................................   2      1     TE2
.....................acccuguagaCccgaauuuguu..........................................................   3      1     TE2
.....................acccuguagauccgaUuuuguu..........................................................   1      1     TE2
.....................acccuguagauccgaaGuuguu..........................................................   1      1     TE2
.....................acccuguagauccgaauuuguu..........................................................   2600   0     TE2
.....................acccuguagauAcgaauuuguu..........................................................   2      1     TE2
.....................acccuguagauccgaaCuuguu..........................................................   2      1     TE2
.....................aUccuguagauccgaauuuguu..........................................................   2      1     TE2
.....................acccuguagauUcgaauuuguu..........................................................   2      1     TE2
.....................acccuguagauccgGauuuguu..........................................................   1      1     TE2
.....................acccuguagauccAaauuuguu..........................................................   1      1     TE2
```

```
                     aga-miR-10
                                                            aga-miR-10*
gucgauuuauguucuacauccacccuguagauccgaauuuguuugaauuuauauuaauaacaaauucgguucuagagagguuugugugggcauuuguuaac

.....................acccCguagauccgaauuuguu..........................................................    2      1      TE2
.....................acccuguagauccgaauCuguu..........................................................    1      1      TE2
.....................acccuguagauccgaauuuguA..........................................................    1      1      TE2
.....................acccugCagauccgaauuuguu..........................................................    2      1      TE2
.....................acccuguagaucUgaauuuguu..........................................................    2      1      TE2
.....................acccuguagauccgaauuuguuu.........................................................    15     0      TE2
.....................acccuguagauccgaauuuguuA.........................................................    109    1      TE2
.....................acccuguagauccgaauuuguuG.........................................................    2      1      TE2
.......................ccuguagauccgaauuugu...........................................................    1      0      TE2
.........................uguagauccgaauuuguu..........................................................    1      0      TE2
............................................................caaauucgguucuagagagg.....................    2      0      TE2
............................................................caaauucgguucuagagagguu...................    3      0      TE2
............................................................Uaaauucgguucuagagagguuu..................    1      1      TE2
............................................................caaauucgguucuagagagguuu..................    24     0      TE2
............................................................caaauucgguucuagagagguuuU.................    1      1      TE2
```

miRBase precursor : aga-mir-1174
Total read count : 67795
aga-miR-1174 read count : 64177
remaining reads : 3618

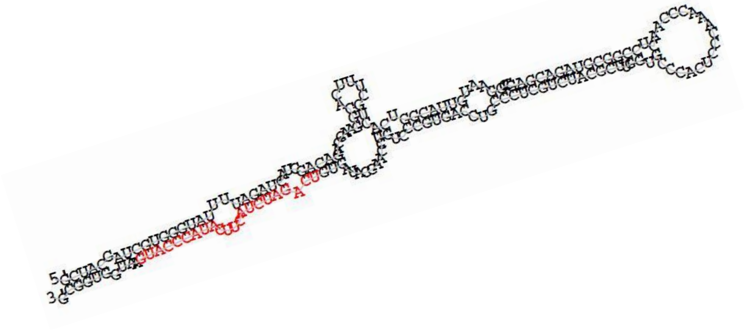

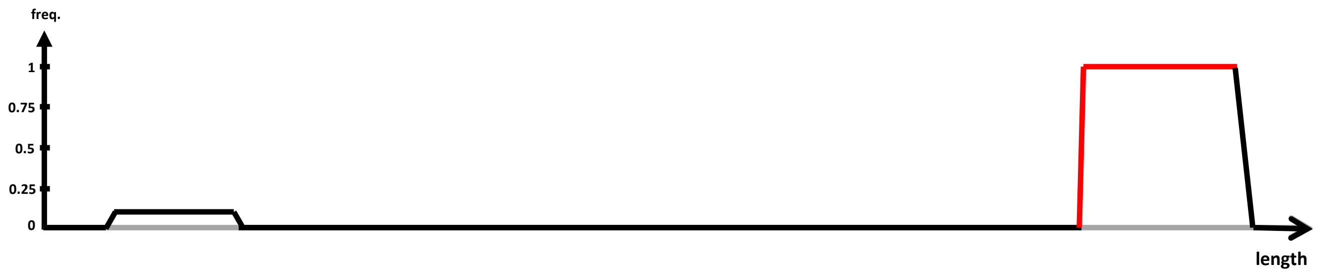

aga-mir-1174

```
5'-gcuacgaucguggguauuuuagaucaucgacagaccaaggcacuuucgucacuggcauuguaaggccgagcagaugcggccucaacccaaacccucacccgugguucgcaucugcucccguccagugcccuguccaaccauacugucagaucuacuucauacccaugaauggugggcg-3`
(((((.(((((((((((..((((((...(((((.....(((......)))...(((((((...((..((((((((((((((.(.................).)).))))))))))))))...)))))))............)))))).))))))....))))))))).)).))))).   reads   mm   sample
.................................................................................................................................................ucagaucuacuucauacccaug..........   1   0   BF1
.................................................................................................................................................ucagaucuacuucauacccaCga.........   1   1   BF1
.................................................................................................................................................ucagaucuacuucauacccauCa.........   1   1   BF1
.................................................................................................................................................ucagaucuaUuucauacccaugaa........   1   1   BF1
.................................................................................................................................................ucagaucuacuucauacUcaugaau.......   1   1   BF1
.................................................................................................................................................ucagaucuacuucauacccauAa.........   2   1   BF2
.................................................................................................................................................ucagaucuacuCcauacccaugaa........   2   1   BF2
.................................................................................................................................................ucagaucuacuucauacccauA..........   1   1   FF1
.................................................................................................................................................ucagaucuacCucauacccauga.........   1   1   FF1
.................................................................................................................................................ucagaucuacuuUauacccauga.........   1   1   FF1
.................................................................................................................................................ucagaCcuacuucauacccaugaa........   1   1   FF1
.................................................................................................................................................ucagaucuacuCcauacccaugaau.......   1   1   FF1
.........guggguauuuuagaucauc.....................................................................................................................................................   2   0   FF2
.........guggguauuuuagaucaucgac..................................................................................................................................................   1   0   FF2
........guggguauuuuagaucaucgaUa.................................................................................................................................................   112   1   FF2
.........guggguauuuuagaucaucgacC.................................................................................................................................................   1   1   FF2
.........guggguauuuuagaucaucgacU.................................................................................................................................................   1   1   FF2
.........guggguauuuuagaucaucgaca.................................................................................................................................................   1   0   FF2
.........guggguauuuuagaucaucgacagacc.............................................................................................................................................   1   0   FF2
.........guggguauuuuagaucaucgacagacc.............................................................................................................................................   1   0   FF2
....................agaucaucgacagaccaaggcacuuucgucacugg..........................................................................................................................   1   0   FF2
...................................caaggcacuuucgucacuggcaCu......................................................................................................................   1   1   FF2
...........................................uuucgucacuggcaCuguaaggc...............................................................................................................   1   1   FF2
..............................................cgucacuggcaCuguaaggccgagcagaugcggccucaacccaaac.....................................................................................   1   1   FF2
.................................................cacuggcaCuguaaggccgagcag........................................................................................................   1   1   FF2
..........................................................................................................................cagugcccuguccaaccauacugucagaucu........................   1   0   FF2
.............................................................................................................................................acugucagaucuacuucauacccaugaaA.......   1   1   FF2
..............................................................................................................................................cugucagaucuacuucauacccauga.........   1   0   FF2
................................................................................................................................................Cucagaucuacuucauacccaugaa........   3   1   FF2
.................................................................................................................................................ucagaucuacuucauaccc.............   10   0   FF2
.................................................................................................................................................ucagaucuacuucauaccca............   15   0   FF2
```

5′-gcuacgaucguggguauuuuagaucaucgacagaccaaggcacuuucgucacuggcauuguaaggccgagcagaugcggccucaacccaaacccucacccgugguucgcaucugcucccguccagugcccuguccaaccauacugucagaucuacuucauacccaugaauggugggcg-3'	reads	mm	sample
....ucagaucuacuucauaccca............	1	0	FF2
....ucagaucuacuucauacccau...........	6	0	FF2
....ucagaucuacuucauacccaug..........	1	0	FF2
....ucagaucuacuuUauacccaug..........	1	1	FF2
....ucagaucuacuuUauacccaug..........	1	1	FF2
....ucagaucuacuucauacccaug..........	1	0	FF2
....uUagaucuacuucauacccaug..........	317	1	FF2
....ucagaucuacuucauacccaug..........	1	0	FF2
....ucGgaucuacuucauacccaug..........	1	1	FF2
....ucagaucuacuAcauacccauga.........	1	1	FF2
....ucagaucuacuucauacccaAga.........	1	1	FF2
....ucagaucuacuucauaGccauga.........	1	1	FF2
....ucagaucuacuucauacccauga.........	1	0	FF2
....ucagCucuacuucauacccauga.........	1	1	FF2
....ucagaucuacuGcauacccauga.........	1	1	FF2
....ucagaucuacuucauacAcauga.........	1	1	FF2
....ucagaucuCcuucauacccauga.........	1	1	FF2
....ucagaucuacuucauaAccauga.........	1574	1	FF2
....ucGgaucuacuucauacccauga.........	2	1	FF2
....ucagaucuacuucauacccauAa.........	4	1	FF2
....ucagaucuacuucauaccUauga.........	1	1	FF2
....ucagaucuacuucauaUccauga.........	1	1	FF2
....ucagaucuaGuucauacccauga.........	1	1	FF2
....uAagaucuacuucauacccauga.........	1	1	FF2
....ucagaucuacuCcauacccauga.........	1	1	FF2
....ucagaucuacuucauacAcauga.........	1	1	FF2
....ucagaucuacuucauacccaCga.........	2	1	FF2
....ucagaucuacuGcauacccauga.........	2	1	FF2
....ucagaucuacuucauacccaugU.........	1	1	FF2
....uUagaucuacuucauacccauga.........	2	1	FF2
....ucagaucuacuucauaccAauga.........	2	1	FF2
....ucagaucuacAucauacccaugaa........	1	1	FF2
....ucagaucuacuucauacAcaugaa........	1	1	FF2
....ucagaucuacuucauacccaugaa........	2	0	FF2
....ucagaucAacuucauacccaugaa........	1	1	FF2
....uGagaucuacuucauacccaugaa........	2	1	FF2
....ucagaucuacuucauaUccaugaa........	1	1	FF2
....uUagaucuacuucauacccaugaa........	2	1	FF2
....ucagaucuaUuucauacccaugaa........	1646	1	FF2
....ucagaucuacuucauacccaugaa........	1	0	FF2
....ucagaucuacuucUuacccaugaa........	10	1	FF2
....ucagaucuacuucauacccaCgaa........	1	1	FF2
....ucagaucuacuucauacccaGgaa........	1	1	FF2
....ucagaucuacuucauacccaCgaa........	2	1	FF2
....ucagaucuacuucauaAccaugaa........	1	1	FF2
....ucagaucuacuucauacccaugaaA.......	4	1	FF2
....ucagaucuacuucauacccaugaCu.......	507	1	FF2
....ucagaucuacuucauacccaugaaC.......	4	1	FF2
....ucagaucuacuucauacccaugaaC.......	139	1	FF2
....ucagaucuacuucauacccaugaauA......	1	1	FF2
....cagaucuacuucauacccauga.........	1	0	FF2
....cagaucuacuucauacccaugaa........	2	0	FF2
....agaucuacuucauacccauga.........	1	0	FF2

5′- gcuacgaucguggguauuuuagaucaucgacagaccaaggcacuuucgucacuggcauuguaaggccgagcagaugcggccucaacccaaacccucacccgugguucgcaucugcucccguccagugcccuguccaaccauacugucagaucuacuucauacccaugaaugguggcg-3'	reads	mm	sample
.........guggguauuuuagaucaucgaca...	60	0	FW1
.........guggguauuuuagaucauc...	1	0	FW1
.........guggguauuuuagaucauc...	2	0	FW1
.........guggguauuuuagaucaucga...	3	0	FW1
.........gugAguauuuuagaucaucgaca...	1	1	FW1
.........guggguauuuuagaucaucgacagaccaCggcacuuucg...	1	1	FW1
.........guggguauuuuagaucaucgacagaccaaggcacuuucguca..	1	0	FW1
.........guggguauuuuagaucaucgacagaccaCggcacuuucgucacuggc...	1	1	FW1
...........ggguauuuuagaucaucgaca...	1	0	FW1
...............auuuuagaucaucgacagaccaCggcacu...	1	1	FW1
...............auuuuagaucaucgacagaccaaggcacuuucgucacuggca..	1	0	FW1
................uuuuagaucaucgacagaccaaggcacu...	1	0	FW1
.......................ucaucgacagaccaaggcacuuucgucacuggcaCugu..	1	1	FW1
........................caucgacagaccaaggcacuuucgucacu..	1	0	FW1
...................................caCggcacuuucgucacuggca..	1	1	FW1
......................................ggcacuuucgucacuggcaCug...	1	1	FW1
......................................ggcacuuucgucacuggcaCuguaag...	2	1	FW1
......................................ggcacuuucgucacuggcaCuguaaggc..	1	1	FW1
......................................ggcacuuucgucacuggcaCuguaaggccga...	1	1	FW1
....gcacuuucgucacuggcaCuguaaggccgagca...	1	1	FW1
....acuuucgucacuggcaCuguaaggccgagcagau..	1	1	FW1
....uggcaCuguaaggccgagcagaugcggccuca...	1	1	FW1
....gaugcggccucaacccaaacccucacccgugguucgc..	1	0	FW1
....cggccucaacccaaacccucacccgugguucgcaucugc..	1	0	FW1
....cucccguccagugcccuguccaaccauacug................................	1	0	FW1
....cuguccaaccauacugucagaucuacuucauacccaugaaA.......	1	1	FW1
....ccaaccauacugucagaucuacuucauacccaugaa........	1	0	FW1
....cauacugucagaucuacuucauacccauga.........	1	0	FW1
....auacugucagaucuacuucauacccaugaa........	1	0	FW1
....ugucagaucuacuucauacccaugaaA.......	1	1	FW1
....ucagaucuacuucauac...............	1	0	FW1
....ucagaucuacuucauacc..............	6	0	FW1
....ucagaucuacuucauaccc.............	9	0	FW1
....ucagaucuacuucauaccca............	2	0	FW1
....ucagaucuacuucauacccauA..........	50	1	FW1
....ucagaucuaGuucauacccaug..........	1	1	FW1
....ucagaucuacuucauacccaugG.........	731	1	FW1
....ucagaucuacuucauacccGuga.........	2	1	FW1
....ucagaucuacuucauaUccauga.........	1	1	FW1
....ucagaucuacuucauacccauga.........	1	0	FW1
....ucagaucuacuucauacccaugG.........	1	1	FW1
....ucagauAuacuucauacccauga.........	1	1	FW1
....ucagaucuacuucauacccaugaa........	1	0	FW1
....ucagaucuacuucaAacccaugaa........	1	1	FW1
....ucagaucuacuucauacccauAaa........	1	1	FW1
....ucagaucuacuucauacccaugaG........	1	1	FW1
....ucagaucuacuucauGcccaugaa........	721	1	FW1
....ucagaucuaUuucauacccaugaa........	5	1	FW1
....ucagaucuacuucaAacccaugaa........	2	1	FW1
....uAagaucuacuucauacccaugaa........	1	1	FW1
....ucagaucAacuucauacccaugaa........	3	1	FW1
....ucagaucuacuGcauacccaugaa........	1	1	FW1

5′-gcuacgaucguggguauuuuagaucaucgacagaccaaggcacuuucgucacuggcauuguaaggccgagcagaugcggccucaacccaaacccucacccgugguucgcaucugcucccguccagugcccuguccaaccauacugucagaucuacuucauacccaugaaugguggcg-3'	reads	mm	sample
....ucagaucuacuucaCacccaugaau.......	2	1	FW1
....ucagaucuacuucauacccaugaaA.......	5	1	FW1
....ucagaucuacuucauacccaugaau.......	1	0	FW1
....ucagaucuacuucauacccaugaaA.......	71	1	FW1
....uUagaucuacuucauacccaugaau.......	213	1	FW1
....ucagaucuacuucauacccaugaauU......	1	1	FW1
....ucagaucuacuucauacccaugaauU......	1	1	FW1
....cagaucuacuucauacccauga.........	2	0	FW1
....cagaucuacuucauacccaugaa........	4	0	FW1
....cagaucuacuucauacccaugaaA.......	1	1	FW1
....agaucuacuucauacccauga.........	1	0	FW1
....uacuucauacccaugaa........	1	0	FW1
.........guggguauuuuagaucauc...	3	0	FW2
.........guggguaCuuuagaucaucgac..	5	1	FW2
.........guggguauuuuGgaucaucgaca...	1	1	FW2
.........guggguauuuuagaucaucgaca...	143	0	FW2
.........guggguauuuuagaucaucgacagaccaaggcacu...	1	0	FW2
.........guggguauuuuagaucaucgacagaccaCggcacuuuc..	1	1	FW2
............gguauuuuagaucaucga...	1	0	FW2
..................uuagaucaucgacagaccaaggcac..	1	0	FW2
.....................gaucaucgacagaccaaggcacuuucgucacuggcaCug...	1	1	FW2
........................caucgacagaccaCggcac..	1	1	FW2
........................caucgacagaccaCggcacuuucgucac...	1	1	FW2
........................caucgacagaccaCggcacuuucgucacu..	1	1	FW2
........................caucgacagaccaaggcacuuucgucacugg..	1	0	FW2
........................caucgacagaccaaggcacuuucgucacuggcaCugu..	1	1	FW2
.........................aucgacagaccaaggcacuuucgucacuggcaCu..	1	1	FW2
................................gaccaCggcacuuucgucacu..	1	1	FW2
....................................aaggcacuuucgucacuggcaCug...	1	1	FW2
......................................ggcacuuucgucacuggcaCugua...	2	1	FW2
......................................ggcacuuucgucacuggcaCuguaaggccgagcaga...	1	1	FW2
....cuuucgucacuggcaCuguaa..	1	1	FW2
....cgucacuggcaCuguaaggccgagcaga...	1	1	FW2
....cacuggcaCuguaaggccgagcagaugcggccucaacccaaacc..	1	1	FW2
....acuggcaCuguaaggccgagcagaugcggccucaacccaaacc..	2	1	FW2
....caCuguaaggccgagcagaugcggccucaacccaaacccucacccgu...	1	1	FW2
....ugcggccucaacccaaacccucacccgugguucgc..	1	0	FW2
....ugcggccucaacccaaacccucacccgugguucgc..	1	0	FW2
....cggccucaacccaaacccucacccgugguucgca...	1	0	FW2
....cugcucccguccagugcccuguccaaccauacugucagaucuac......................	1	0	FW2
....ugcucccguccagugcccuguccaaccauacug................................	1	0	FW2
....gcucccguccagugcccuguccaaccauacugucagaucuacuucauaccc.............	1	0	FW2
....cucccguccagugcccuguccaaccauacug................................	1	0	FW2
....cccguccagugcccuguccaaccauacu.................................	1	0	FW2
....cagugcccuguccaaccauacugucagaucuacuucauacccaugaa........	1	0	FW2
....cccuguccaaccauacugucagaucuacuucauacccaugaa........	1	0	FW2
....aaccauacugucagaucuacuucauacccaugaaA.......	1	1	FW2
....acugucagaucuacuucauacccaugaa........	1	0	FW2
....Ggucagaucuacuucauacccaugaa........	1	1	FW2
....Cucagaucuacuucauacccaugaa........	2	1	FW2
....Cucagaucuacuucauacccaugaau.......	1	1	FW2

5'- gcuacgaucgugggguauuuuagaucaucgacagaccaaggcacuuucgucacuggcauuguaaggccgagcagaugcggccucaacccaaacccucacccgugguucgcaucugcucccguccagugcccuguccaaccauacugucagaucuacuucauacccaugaaugguggcg-3'	reads	mm	sample
....ucagaucuacuucauac...............	13	0	FW2
....ucagaucuacuuUauaccc.............	11	1	FW2
....ucagaucuacuucauCccca............	10	1	FW2
....ucagaucuacCucauaccca............	2	1	FW2
....ucagaucuacuucauacccaGg..........	90	1	FW2
....ucagaucuacuucauaAccaug..........	1	1	FW2
....ucagaucuacuucaCacccauga.........	1	1	FW2
....ucagaucuaUuucauacccauga.........	1	1	FW2
....ucagaucuacuucauacccaugG.........	1	1	FW2
....ucagauUuacuucauacccauga.........	1	1	FW2
....ucagaucuacuucauacAcauga.........	1	1	FW2
....ucagaucuGcuucauacccauga.........	1	1	FW2
....ucagaucuacCucauacccauga.........	1	1	FW2
....ucagaucuaAuucauacccauga.........	2	1	FW2
....Ccagaucuacuucauacccauga.........	1	1	FW2
....ucagaucuacuCcauacccauga.........	2	1	FW2
....ucagaucuacuucauacccUuga.........	2141	1	FW2
....ucagaucuacuucauGcccauga.........	1	1	FW2
....ucagaucuacuCcauacccauga.........	1	1	FW2
....ucagaCcuacuucauacccauga.........	3	1	FW2
....ucagaucuacuucauacccauCa.........	1	1	FW2
....ucagaucuacuCcauacccauga.........	3	1	FW2
....ucagaucuacuucauaAccauga.........	1	1	FW2
....ucagaucuacuucauacccaugG.........	1	1	FW2
....ucGgaucuacuucauacccauga.........	1	1	FW2
....ucagaucuacuucauacccaugaC........	1	1	FW2
....ucagaucuacuuUauacccaugaa........	9	1	FW2
....ucagaucuacuucauacccaugaU........	1	1	FW2
....ucagaucuacuuGauacccaugaa........	1	1	FW2
....ucagaucuacuucauacccGugaa........	2	1	FW2
....ucaAaucuacuucauacccaugaa........	2	1	FW2
....ucagGucuacuucauacccaugaa........	1	1	FW2
....ucagaucuacuucauacccaGgaa........	1	1	FW2
....ucagaucuacuucauCcccaugaa........	1	1	FW2
....ucagaucuacuucauacccaugUa........	4	1	FW2
....ucagaucuacuucaCacccaugaa........	1	1	FW2
....ucagaucuaUuucauacccaugaa........	2	1	FW2
....ucagaucuacuucauaccAaugaa........	1	1	FW2
....ucagaucuacuucauacccaugaU........	2	1	FW2
....Ccagaucuacuucauacccaugaa........	1	1	FW2
....ucagaucuacuucauaccUaugaa........	1	1	FW2
....ucagaucuaGuucauacccaugaa........	1	1	FW2
....ucagaucuacuucauacccaugaa........	2195	0	FW2
....uUagaucuacuucauacccaugaa........	1	1	FW2
....ucagaucuacuucauacccauAaa........	1	1	FW2
....uUagaucuacuucauacccaugaa........	4	1	FW2
....ucagaucuacuucauacccaugaC........	1	1	FW2
....ucagaucuacuucauacccaugaa........	1	0	FW2
....ucagaucuacuucauacccaugaaC.......	1	1	FW2
....ucagaucuacuucauacccaugaUu.......	1	1	FW2
....ucagaucuacuucauacccaugaaA.......	1	1	FW2

5′- gcuacgaucguggguauuuuagaucaucgacagaccaaggcacuuucgucacuggcauuguaaggccgagcagaugcggccucaacccaaacccucacccgugguucgcaucugcucccguccagugcccuguccaaccauacugucagaucuacuucauacccaugaauggugg cg-3'	reads	mm	sample
....ucagaucuacuucauacccaugaaC.......	245	1FW2	
....ucagaucuacuucauacccaugaCu.......	2	1	FW2
....ucGgaucuacuucauacccaugaau.......	681	1	FW2
....ucagaucuacuucauacccaugaau.......	9	0	FW2
....ucagaucuacuucauacccaugaauU......	3	1	FW2
....ucagaucuacuucauacccaugaaug......	1	0	FW2
....ucagaucuacuucauacccaugaauU......	1	1	FW2
....cagaucuacuucauacccauga.........	3	0	FW2
....cagaucuacuucauacccaugaa........	1	0	FW2
....agaucuacuucauacccaugaa........	4	0	FW2
....agaucuacuucauacccaugaa........	1	0	FW2
....agaucuacuucauacccaugaau.......	1	0	FW2
....agaucuacuucauacccaugaaA.......	1	1	FW2
....aucuacuucauacccauga.........	1	0	FW2
....ucuacuucauacccauga.........	2	0	FW2
....uacuucauacccaugaa........	1	0	FW2
.........guggguauuuuagauUaucgac..	7	1	MF1
.........guggguauuuuagaucaucgaAa...	1	1	MF1
.........gugAguauuuuagaucaucgaca...	1	1	MF1
.........guggguaCuuuagaucaucgaca...	210	1	MF1
.........guggguauuuuagaucaucgacagaccaaggcacu...	1	0	MF1
.........guggguauuuuagaucaucgacagaccaaggcacuuucguc...	1	0	MF1
....cUcuggcauuguaaggc...	1	1	MF1
....gcucccguccagugcccuguccaaccauacugucCgaucuacuucauaccc.............	1	1	MF1
....ugcccuguccaaccauacug................................	1	0	MF1
....cugucagaucuacuucauacccauga.........	1	0	MF1
....Cucagaucuacuucauacccauga.........	1	1	MF1
....Cucagaucuacuucauacccaugaa........	3	1	MF1
....ucagaucuacuucauacc..............	1	0	MF1
....ucagaucuacuucauaccc.............	13	0	MF1
....Acagaucuacuucauaccca............	30	1	MF1
....ucagaucuacuucauacccau...........	8	0	MF1
....ucagaucuacuucauacccauU..........	1	1	F1
....ucagaucuacuucauCcccaug..........	443	1	MF1
....ucagaucuacuucUuacccaug..........	1	1	MF1
....ucagaucuacuucaCacccaug..........	1	1	MF1
....ucagaucuacuucauaGccaug..........	2	1	MF1
....ucagaucuacuucauacccaug..........	1	0	MF1
....ucagaucuacuucauacccaugU.........	1	1	MF1
....ucagaucuacCucauacccauga.........	1	1	MF1
....uGagaucuacuucauacccauga.........	1	1	MF1
....ucagaucuacuucauaUccauga.........	1	1	MF1
....ucagaucuacuucauaGccauga.........	1	1	MF1
....ucagaucuacAucauacccauga.........	1	1	MF1
....ucagaucuacCucauacccauga.........	3	1	MF1
....ucagaucuacuucauacccauga.........	1	0	MF1
....ucagaucuacuucauacccauga.........	1	0	MF1
....Gcagaucuacuucauacccauga.........	2671	1	MF1
....ucagGucuacuucauacccauga.........	1	1	MF1
....ucagaucuaAuucauacccauga.........	2	1	MF1
....ucagaucuacuucauacccaCga.........	3	1	MF1

```
5'- gcuacgaucguggguauuuuagaucaucgacagaccaaggcacuuucgucacuggcauuguaaggccgagcagaugcggccucaacccaaacccucacccgugguucgcaucugcucccguccagugcccuguccaaccauacugucagaucuacuucauacccaugaaugguggcg-3'    reads   mm   sample
    .................................................................................................................................................ucagaucuacuucauaAccaugaa........    3       1    MF1
    .................................................................................................................................................ucagaucuacuGcauacccaugaa........    1       1    MF1
    .................................................................................................................................................uAagaucuacuucauacccaugaa........    1       1    MF1
    .................................................................................................................................................Acagaucuacuucauacccaugaa........    2       1    MF1
    .................................................................................................................................................ucagaucuacuuAauacccaugaa........    1       1    MF1
    .................................................................................................................................................ucagauAuacuucauacccaugaa........    1       1    MF1
    .................................................................................................................................................ucagaucuacuucauacccaugaU........    2       1    MF1
    .................................................................................................................................................ucagaucuaGuucauacccaugaa........    1       1    MF1
    .................................................................................................................................................ucUgaucuacuucauacccaugaa........    2225    1    MF1
    .................................................................................................................................................ucagaucuacuucauacccaugaa........    11      0    MF1
    .................................................................................................................................................ucagaucuacuucauacccaugaaC.......    666     1    MF1
    .................................................................................................................................................ucUgaucuacuucauacccaugaau.......    170     1    MF1
    .................................................................................................................................................ucagaucuacuucauacccaugaau.......    5       0    MF1
    .................................................................................................................................................ucagaucuacuucauaAccaugaau.......    2       1    MF1
    .................................................................................................................................................ucagaucuacuucauaccUaugaau.......    2       1    MF1
    .................................................................................................................................................ucagaucuacuucauacccaugaaA.......    12      1    MF1
    .................................................................................................................................................ucagaucuacuucauacccaugaauA......    1       1    MF1
    .................................................................................................................................................ucagaucuacuucauacccaugaaAg......    1       1    MF1
    ..................................................................................................................................................Uagaucuacuucauaccc.............    1       1    MF1
    ..................................................................................................................................................Uagaucuacuucauacccaugaa........    5       1    MF1
    ...................................................................................................................................................agaucuacuucauacccaugaa........    1       0    MF1
    .......................................................................................................................................................cuacuucauacccauga.........    4       0    MF1
    ........cguggguauuuuagauc........................................................................................................................................................    3       0    MF2
    .........guggguauuuuagaucaucga...................................................................................................................................................    4       0    MF2
    .........guggguauuuuagaucaucgac..................................................................................................................................................    1       0    MF2
    .........guggguauuuuagaucaucgac..................................................................................................................................................    1       0    MF2
    .........guggguauuuuagaucaucgac..................................................................................................................................................    1       0    MF2
    .........guggguauuuuagaucauUgac..................................................................................................................................................    31      1    MF2
    .........guggguauuuuagaucaucgac..................................................................................................................................................    2       0    MF2
    .........guggguauuuCagaucaucgaca.................................................................................................................................................    1       1    MF2
    .........guggguauuuuagaCcaucgaca.................................................................................................................................................    2       1    MF2
    .........Augggguauuuuagaucaucgaca................................................................................................................................................    1       1    MF2
    .........guggguauuuuagaucaucgaca.................................................................................................................................................    1       0    MF2
    .........guggguauuuuagaucaucgacU.................................................................................................................................................    1       1    MF2
    .........gugggAauuuuagaucaucgaca.................................................................................................................................................    1955    1    MF2
    .........guggguauuuuagaucaucgaca.................................................................................................................................................    1       0    MF2
    .........guggguauuuuagaucaucgacU.................................................................................................................................................    1       1    MF2
    .........gGggguauuuuagaucaucgaca.................................................................................................................................................    2       1    MF2
    .........gugggGauuuuagaucaucgaca.................................................................................................................................................    1       1    MF2
    .........guggguauuuuagGucaucgaca.................................................................................................................................................    1       1    MF2
    .........guggguauuuuCgaucaucgaca.................................................................................................................................................    2       1    MF2
    .........gCggguauuuuagaucaucgaca.................................................................................................................................................    1       1    MF2
    .........guggguauuuuagaucaucgacC.................................................................................................................................................    1       1    MF2
    .........guggguauuuuagaucaucgaGa.................................................................................................................................................    2       1    MF2
    .........guAgguauuuuagaucaucgaca.................................................................................................................................................    17      1    MF2
    .........gugggguGuuuuagaucaucgaca................................................................................................................................................    2       1    MF2
    .........guggguauuuuagaucaucgacC.................................................................................................................................................    1       1    MF2
    .........guggguauuuuagaucaucUaca.................................................................................................................................................    1       1    MF2
    .........guggguauuuuagaucaucgacaga...............................................................................................................................................    1       0    MF2
    .........guggguauuuuagaucaucgacagac..............................................................................................................................................    1       0    MF2
    .........guggguauuuuagaucaucgacagaccaag..........................................................................................................................................    1       0    MF2
    .........guggguauuuuagaucaucgacagaccaagg.........................................................................................................................................    1       0    MF2
    .........guggguauuuuagaucaucgacagaccaaggc........................................................................................................................................    4       0    MF2
    .........guggguauuuuagaucaucgacagaccaaggca.......................................................................................................................................    4       0    MF2
    .........guggguauuuuagaucaucgacagaccaaggcacu.....................................................................................................................................    2       0    MF2
```

5'- gcuacgaucguggguauuuuagaucaucgacagaccaaggcacuuucgucacuggcauuguaaggccgagcagaugcggccucaacccaaacccucacccgugguucgcaucugcucccguccagugcccuguccaaccauacugucagaucuacuucauacccaugaaugguggcg-3'	reads	mm	sample
....ucagaucuacuuUauacccauga.........	2	1	MF1
....ucagaucuacuucauacccCuga.........	1	1	MF1
....ucagaucuacuCcauacccauga.........	1	1	MF1
....ucagaucuacuucauacccaGga.........	3	1	MF1
....ucagaucuacuucauaUccauga.........	2	1	MF1
....ucagaucuacuucauacccauga.........	4	0	MF1
....ucagaucuacuucCuacccauga.........	1	1	MF1
....ucagaucuacuucauacUcauga.........	1	1	MF1
....ucagaucuacuucauaccUaugaa........	3	1	MF1
....ucagaucuacuucauacccaugaG........	5	1	MF1
....ucagGucuacuucauacccaugaa........	1	1	MF1
....ucagaucuacuucauacccGugaa........	1	1	MF1
....ucagaucuacuuGauacccaugaa........	1	1	MF1
....ucagauGuacuucauacccaugaa........	2	1	MF1
....ucagaucuacuCcauacccaugaa........	1	1	MF1
....ucaCaucuacuucauacccaugaa........	1	1	MF1
....ucagaucuacuucauacccaugGa........	1	1	MF1
....ucagaucuacuuUauacccaugaa........	1	1	MF1
....ucagaucuacuucauaccGaugaa........	1	1	MF1
....ucagaucuacuucauacccaugaU........	1	1	MF1
....ucagaucuaGuucauacccaugaa........	2	1	MF1
....ucagaucuacuCcauacccaugaa........	1	1	MF1
....uUagaucuacuucauacccaugaa........	1	1	MF1
....ucagaucuacuucauacccaugaC........	1	1	MF1
....ucagaucuacuuUauacccauga.........	2	1	MF1
....ucagaucuacuucauacccCuga.........	1	1	MF1
....ucagaucuacuCcauacccauga.........	1	1	MF1
....ucagaucuacuucauacccaGga.........	3	1	MF1
....ucagaucuacuucauaUccauga.........	2	1	MF1
....ucagaucuacuucauacccauga.........	4	0	MF1
....ucagaucuacuucCuacccauga.........	1	1	MF1
....ucagaucuacuucauacUcauga.........	1	1	MF1
....ucagaucuacuucauaccUaugaa........	3	1	MF1
....ucagaucuacuucauacccaugaG........	5	1	MF1
....ucagGucuacuucauacccaugaa........	1	1	MF1
....ucagaucuacuucauacccGugaa........	1	1	MF1
....ucagaucuacuuGauacccaugaa........	1	1	MF1
....ucagauGuacuucauacccaugaa........	2	1	MF1
....ucagaucuacuCcauacccaugaa........	1	1	MF1
....ucaCaucuacuucauacccaugaa........	1	1	MF1
....ucagaucuacuucauacccaugGa........	1	1	MF1
....ucagaucuacuuUauacccaugaa........	1	1	MF1
....ucagaucuacuucauaccGaugaa........	1	1	MF1
....ucagaucuacuucauacccaugaU........	1	1	MF1
....ucagaucuaGuucauacccaugaa........	2	1	MF1
....ucagaucuacuCcauacccaugaa........	1	1	MF1
....uUagaucuacuucauacccaugaa........	1	1	MF1
....ucagaucuacuucauacccaugaC........	1	1	MF1
.........guggguauuuuagaucaucgacagaccaaggcacuu..	1	0	MF2
.........guggguauuuuagaucaucgacagaccaaggcacuu..	4	0	MF2
.........guggguauuuuagaucaucgacagaccaaggcacuuuc..	1	0	MF2
.........guggguauuuuagaucaucgacagaccaaggcacuuucgu..	1	0	MF2
.........guggguauuuuagaucaucgacagaccaaggcacuuucguU...	1	1	MF2
.........guggguauuuuagaucaucgacagaccaCggcacuuucgucac...	1	1	MF2
.........guggguauuuuagaucaucgacagaccaCggcacuuucgucacuggca..	1	1	MF2
...........ggguauuuuagaucaucgaca...	1	0	MF2
...........ggguauuuuagaucaucgacagaccaaggcacu...	1	0	MF2
............gguauuuuagaucaucgaca...	1	0	MF2
............gguauuuuagaucaucgacagaccaaggcacuuucgucacuggcaCuguaa..	1	1	MF2
.............guauuuuagaucaucgacaga...	2	0	MF2

5'- gcuacgaucguggguauuuuagaucaucgacagaccaaggcacuuucgucacuggcauuguaaggccgagcagaugcggccucaacccaaacccucacccgugguucgcaucugcucccguccagugcccuguccaaccauacugucagaucuacuucauacccaugaauggugcg-3'	reads	mm	sample
....gucacuggcaCuguaaggccgagcagaugcggccucaaccca...	1	1	MF2
....cacuggcaCuguaaggc..	1	1	MF2
....cacuggcaCuguaaggccgagcagau...	1	1	MF2
....cacuggcaCuguaaggccgagcagaugcgg...	1	1	MF2
....cacuggcaCuguaaggccgagcagaugcggccucaacccaaacccucaccc..	1	1	MF2
....acuggcaCuguaaggccgagcagaugc...	1	1	MF2
....acuggcaCuguaaggccgagcagaugcggccucaaccca...	1	1	MF2
....uggcaCuguaaggccgagcagau...	2	1	MF2
....gagcagaugcggccucaacccaaacccu...	2	0	MF2
....gcagaugcggccucaacccaaacccucacccgug...	2	0	MF2
....gcagaugcggccucaacccaaacccucacccgugguucgc...	1	0	MF2
....cagaugcggccucaacccaaacc...	1	0	MF2
....cagaugcggccucaacccaaacccucacc...	2	0	MF2
....cagaugcggccucaacccaaacccucacccgu..	2	0	MF2
....cagaugcggccucaacccaaacccucacccgugguucgc...	1	0	MF2
....gaugcggccucaacccaaacccucacc...	1	0	MF2
....gaugcggccucaacccaaacccucacccgugguucgc...	1	0	MF2
....augcggccucaacccaaacccucacccgugguucgA...	1	1	MF2
....ugcggccucaacccaaacccucacccgu..	1	0	MF2
....ugcggccucaacccaaacccucacccgugguucgcaucugcu..	1	0	MF2
....gcggccucaacccaaacccucacccgugguucgcau...	2	0	MF2
....cggccucaacccaaacccucacccgugguucgcaucu...	1	0	MF2
....cggccucaacccaaacccuGacccgugguucgcaucug..	1	1	MF2
....cggccucaacccaaacccucacccgugguucgcaucug..	1	0	MF2
....cucacccgugguucgcaucugcu..	1	0	MF2
....cacccgugguucgcaucugcucccguccagugcccuguccU..	1	1	MF2
....cgugguucgcaucugcu..	1	0	MF2
....uucgcaucugcucccguccagugcccuguccaaccauacug...............................	2	0	MF2
....caucugcucccguccagugcccuguccaaccauacug...............................	1	0	MF2
....aucugcucccguccagugcccuguccaacc.....................................	1	0	MF2
....aucugcucccguccagugcccuguccaaccauacugu..............................	2	0	MF2
....gcucccguccagugcccug..	1	0	MF2
....gcucccguccagugcccuguccaaccauacug...............................	1	0	MF2
....cucccguccagugcccu...	1	0	MF2
....cucccguccagugcccuguccaaccauacugucagaucuacuucauaccca...........	2	0	MF2
....cagugcccuguccaaccauacugucagau.........................	1	0	MF2
....cagugcccuguccaaccauacugucagaucuacuucauacccaugaa.......	1	0	MF2
....cagugcccuguccaaccauacugucagaucuacuucauacccaugaau......	1	0	MF2
....agugcccuguccaaccauacugucagaucu.......................	1	0	MF2
....ugcccuguccaaccauacug...............................	2	0	MF2
....cccuguccaaccauacugucagaucuacuucauacccaugaa.......	1	0	MF2
....cccuguccaaccauacugucagaucuacuucauacccaugaa.......	1	0	MF2
....ccuguccaaccauacugucagaucuacuucauacccaugaa.......	1	0	MF2
....ccauacugucagaucuacuucauacccauga........	5	0	MF2
....cauacugucagaucuacuucauacccauga........	1	0	MF2
....cauacugucagaucuacuucauacccaugaa.......	1	0	MF2
....cauacugucagaucuacuucauacccaugaaA......	1	1	MF2
....auacugucagaucuacuucauacccauga........	3	0	MF2
....auacugucagaucuacuucauacccaugaa.......	2	0	MF2

5'- gcuacgaucguggguauuuuagaucaucgacagaccaaggcacuuucgucacuggcauuguaaggccgagcagaugcggccucaacccaaacccucacccgugguucgcaucugcucccguccagugcccuguccaaccauacugucagaucuacuucauacccaugaaugguggcg-3'	reads	mm	sample
....auacugucagaucuacuucauacccaugaau.......	1	0	MF2
....uacugucagaucuacuucauacccauga.........	1	0	MF2
....uacugucagaucuacuucauacccaugaa........	1	0	MF2
....ugucagaucuacuucauaccca............	1	0	MF2
....gucagaucuacuucauacccauga.........	4	0	MF2
....Cucagaucuacuucauacccaugaa........	9	1	MF2
....Cucagaucuacuucauacccaugaau.......	1	1	MF2
....ucagaucuacuucauaccc.............	1	0	MF2
....ucagaucuacuucauaccc.............	41	0	MF2
....ucagaucuacuucauaccc.............	1	0	MF2
....ucagaucuacuucauaccca............	1	0	MF2
....ucagaucuacuucauaccca............	1	0	MF2
....ucagaucuacuucauaccca............	90	0	MF2
....ucagaucuacuucauaccca............	1	0	MF2
....ucUgaucuacuucauaccca............	1	1	MF2
....ucagaucuacuucauacccau...........	30	0	MF2
....ucagaucuacuucauacccau...........	2477	0	MF2
....ucagaucuacuucauacccaug..........	3	0	MF2
....ucagaucuacuucauacccaCg..........	4	1	MF2
....ucagaucuacuucauacccaug..........	1	0	MF2
....ucagaucuacuucauacccaGg..........	2	1	MF2
....ucagaucuacuucauacccaug..........	1	0	MF2
....ucagaucuacuucauacAcaug..........	1	1	MF2
....ucagaCcuacuucauacccaug..........	1	1	MF2
....ucagaucuacuucauacUcaug..........	1	1	MF2
....ucagaucuacuucauaUccaug..........	1	1	MF2
....ucagaucuacuucauacccaug..........	2	0	MF2
....ucagaucAacuucauacccaug..........	1	1	MF2
....ucagaucuacuAcauacccaug..........	1	1	MF2
....ucagCucuacuucauacccaug..........	4	1	MF2
....ucagaucuacuucauacccaug..........	1	0	MF2
....ucagaucuacuucauacccCug..........	2	1	MF2
....ucagaucuacuCcauacccaug..........	2	1	MF2
....ucagaucuacuucauaccGaug..........	2	1	MF2
....ucagaCcuacuucauacccauga.........	5	1	MF2
....ucagaucuacuuAauacccauga.........	2	1	MF2
....ucagaucuacuucauacccauga.........	39	0	MF2
....ucagaucuacuucaCacccauga.........	2	1	MF2
....ucagaucuacuucauacccaCga.........	5	1	MF2
....ucaAaucuacuucauacccauga.........	2	1	MF2
....ucagaucuacuucauacccaCga.........	1	1	MF2
....ucagaucuacuucauacGcauga.........	8	1	MF2
....ucagaucuacuucauacccauUa.........	1	1	MF2
....ucagaucuacuucGuacccauga.........	4	1	MF2
....ucagaucuacuucaCacccauga.........	1	1	MF2
....ucagaucuacuucauacccauAa.........	1	1	MF2
....uAagaucuacuucauacccauga.........	5	1	MF2
....ucagaucuacuucauaccUauga.........	3	1	MF2
....ucagaucuacGucauacccauga.........	12345	1	MF2
....ucagaucuacuucauacccaugG.........	1	1	MF2

```
5'- gcuacgaucguggguauuuuagaucaucgacagaccaaggcacuuucgucacuggcauuguaaggccgagcagaugcggccucaacccaaacccucacccgugguucgcaucugcucccguccagugcccuguccaaccauacugucagaucuacuucauacccaugaaugguggcg-3'    reads    mm    sample
    .................................................................................................................................................ucagaucuGcuucauacccauga.........    1    1    MF2
    .................................................................................................................................................ucagaucuacuucauacccauga.........    1    0    MF2
    .................................................................................................................................................ucagaucuacuucauaccAauga.........    1    1    MF2
    .................................................................................................................................................ucagaucuacuucauacccauga.........    1    0    MF2
    .................................................................................................................................................ucagaucuacuucauacccaugG.........    11    1    MF2
    .................................................................................................................................................uUagaucuacuucauacccauga.........    3    1    MF2
    .................................................................................................................................................ucagaucuacuucauaAccauga.........    1    1    MF2
    .................................................................................................................................................ucagaucuacuucauaccAauga.........    2    1    MF2
    .................................................................................................................................................ucagaucuacuucauacccauga.........    3    0    MF2
    .................................................................................................................................................ucagaCcuacuucauacccauga.........    1    1    MF2
    .................................................................................................................................................ucagaGcuacuucauacccauga.........    14    1    MF2
    .................................................................................................................................................ucagaucuacuucauacccaugaa........    5    0    MF2
    .................................................................................................................................................ucagaucuacuucauaGccaugaa........    16    1    MF2
    .................................................................................................................................................ucagaucuacuucauaccAaugaa........    8    1    MF2
    .................................................................................................................................................ucagaucuacuucauacccaCgaa........    1    1    MF2
    .................................................................................................................................................ucagaucuacuucauacccaAgaa........    3    1    MF2
    .................................................................................................................................................ucagUucuacuucauacccaugaa........    1    1    MF2
    .................................................................................................................................................ucagaucuacuucauacccaugCa........    12868    1    MF2
    .................................................................................................................................................ucagaucuacuucauacccaugaU........    1    1    MF2
    .................................................................................................................................................ucagaAcuacuucauacccaugaa........    2    1    MF2
    .................................................................................................................................................ucagaucuacuucauacccaugaa........    2    0    MF2
    .................................................................................................................................................ucGgaucuacuucauacccaugaa........    2    1    MF2
    .................................................................................................................................................ucagaucuacuucauacccaugaCu.......    1    1    MF2
    .................................................................................................................................................ucagaucuacuucauacccGugaau.......    1    1    MF2
    .................................................................................................................................................ucagaucuacuucauacccaugaUu.......    94    1    MF2
    .................................................................................................................................................ucagaucuacuucauacccaugaaC.......    26    1    MF2
    .................................................................................................................................................ucagaucuacuucauacccaugaaG.......    5728    1    MF2
    .................................................................................................................................................ucagaucuacuucauacccaugaUu.......    1    1    MF2
    .................................................................................................................................................ucagauAuacuucauacccaugaau.......    1    1    MF2
    .................................................................................................................................................ucagaucuacuucauacccaugaau.......    1355    0    MF2
    .................................................................................................................................................ucagaucuacuucauacccaugaUu.......    1    1    MF2
    .................................................................................................................................................ucagaucuacuucauacccaugaaC.......    2    1    MF2
    .................................................................................................................................................ucagaucuacuucauacccaugaaA.......    1    1    MF2
    .................................................................................................................................................ucagaucuacuucauacccaugaau.......    1    0    MF2
    .................................................................................................................................................ucagaucuacuucauacccaugaau.......    1    0    MF2
    .................................................................................................................................................ucagaucuacuucauacccaugaaA.......    1    1    MF2
    .................................................................................................................................................ucagaucuaUuucauacccaugaau.......    13    1    MF2
    .................................................................................................................................................ucagaucuacuucauacccaugaCu.......    1    1    MF2
    .................................................................................................................................................ucagaucuacuucauGcccaugaau.......    2    1    MF2
    .................................................................................................................................................ucagaucuacuucauacccaugaaC.......    2    1    MF2
    .................................................................................................................................................ucagaucuacuucauacccaugaUu.......    1    1    MF2
    .................................................................................................................................................ucagaucuacuucauacccauAaau.......    2    1    MF2
    .................................................................................................................................................ucagaucuacCucauacccaugaau.......    1    1    MF2
    .................................................................................................................................................ucagaucuacuGcauacccaugaau.......    1    1    MF2
    .................................................................................................................................................ucagaucuacuucauacccaugaaC.......    2    1    MF2
    .................................................................................................................................................ucagaucCacuucauacccaugaau.......    1    1    MF2
    .................................................................................................................................................ucagaucuacuucauacccaugaauU......    5    1    MF2
    .................................................................................................................................................ucagaucuacuucauacccaugaauA......    2    1    MF2
    .................................................................................................................................................ucagaucuacuucauacccaugaauU......    2    1    MF2
    .................................................................................................................................................ucagaucuacuucauacccaugaaAg......    3    1    MF2
    .................................................................................................................................................ucagaucuacuucauacccaugaaCg......    1    1    MF2
    ..................................................................................................................................................cagaucuacuucauacccauga.........    2    0    MF2
```

```
5′- gcuacgaucguggguauuuuagaucaucgacagaccaaggcacuuucgucacuggcauuguaaggccgagcagaugcggccucaacccaaacccucacccgugguucgcaucugcucccguccagugcccuguccaaccauacugucagaucuacuucauacccaugaauggugcg-3'    reads    mm    sample
    ..................................................................................................................................................cagaucuacuucauacccauga........    1    0    MF2
    ..................................................................................................................................................cagaucuacuucauacccaugaa.......    4    0    MF2
    ..................................................................................................................................................cagaucuacuucauacccaugaa.......    2    0    MF2
    ..................................................................................................................................................cagaucuacuucauacccaugaaA......    2    1    MF2
    ...................................................................................................................................................agaucuacuucauacccaugaa.......    2    0    MF2
    ...................................................................................................................................................agaucuacuucauacccaugaau......    3    0    MF2
    ...................................................................................................................................................agaucuacuucauacccaugaaA......    1    1    MF2
    .........guggguauuuuagaucauc....................................................................................................................................................    3    0    MW1
    .........guggguauuuuagaucaucgac.................................................................................................................................................    8    0    MW1
    .........guggguauuuuagaucaucgacG................................................................................................................................................    1    1    MW1
    .........guggguauuuuagaucaucgacU................................................................................................................................................    138    1    MW1
    .........guggguauuuuagaucaucgacU................................................................................................................................................    138    1    MW1
    .........guggguauuuuagaucaucgacagaccaCggcacuuucgucacugg.........................................................................................................................    1    1    MW1
    ...........ggguauuuuagaucaucgacagaccaCggcacuuucguc..............................................................................................................................    1    1    MW1
    ............gguauuuuagaucaucgacagaccaCggcacuuucgucacu...........................................................................................................................    1    1    MW1
    ..............uauuuuagaucaucgacagaccaaggcacuuucgu...............................................................................................................................    1    0    MW1
    ...............auuuuagaucaucgacagaccaCggcacuuuc.................................................................................................................................    1    1    MW1
    ...............auuuuagaucaucgacagaccaCggcacuuucgu...............................................................................................................................    1    1    MW1
    ................uuuuagaucaucgacagaccaaggcacuuucgucacuggcaC......................................................................................................................    1    1    MW1
    .................uuuagaucaucgacagaccaaggcacuuucg................................................................................................................................    1    0    MW1
    .................uuuagaucaucgacagaccaaggcacuuucgucacuggc........................................................................................................................    1    0    MW1
    ....................agaucaucgacagaccaaggcacuuucgucacuggcaC......................................................................................................................    1    1    MW1
    ........................caucgacagaccaCggcacuuucguca.............................................................................................................................    1    1    MW1
    ........................caucgacagaccaaggcacuuucgucacuggcaCuguaaggccga...........................................................................................................    1    1    MW1
    ..........................ucgacagaccaaggcacuuucgucacuggcaCug....................................................................................................................    1    1    MW1
    ...................................caaggcacuuucgucacuggcaCug....................................................................................................................    3    1    MW1
    ......................................ggcacuuucgucacuggcaCuguaag................................................................................................................    1    1    MW1
    ......................................ggcacuuucgucacuggcaCuguaaggcc.............................................................................................................    1    1    MW1
    ......................................ggcacuuucgucacuggcaCuguaaggccgagcagaug....................................................................................................    1    1    MW1
    .........................................acuuucgucacuggcaCuguaaggccgagcag.......................................................................................................    1    1    MW1
    ..............................................cgucacuggcaCuguaaggcc.............................................................................................................    1    1    MW1
    ................................................ucacuggcaCuguaaggccgagcagaugcggccucaaccc........................................................................................    1    1    MW1
    ...................................................cuggcaCuguaaggccgagcagaugcggccucaacc.........................................................................................    2    1    MW1
    ...................................................cuggcaCuguaaggccgagcagaugcggccucaacccaaacccucacccg...........................................................................    1    1    MW1
    .......................................................caCuguaaggccgagcagaugc...................................................................................................    1    1    MW1
    .......................................................................agaugcggccucaacccaaacccucaccc............................................................................    1    0    MW1
    ........................................................................gaugcggccucaacccaaacccucacccgugguucg....................................................................    1    0    MW1
    ............................................................................cggccucaacccaaacccucacccgugguucgcau.................................................................    1    0    MW1
    ..................................................................................................................cucccguccagugcccuguccaaccauacug...............................    1    0    MW1
    ............................................................................................................................gugcccuguccaaccauacugGcagauc........................    1    1    MW1
    ..............................................................................................................................gcccuguccaaccauacugucagaucu.......................    1    0    MW1
    ..........................................................................................................................................cauacugucagaucuacuucauacccauga........    2    0    MW1
    ..............................................................................................................................................cugucagaucuacuucauacccaug.........    1    0    MW1
    ...............................................................................................................................................uCucagaucuacuucauacccaugaa.......    1    1    MW1
    ................................................................................................................................................Cucagaucuacuucauacccauga........    1    1    MW1
    .................................................................................................................................................ucagaucuacuucauacc.............    7    0    MW1
    .................................................................................................................................................ucagaucuacuucauaccc............    6    0    MW1
    .................................................................................................................................................ucagaucuacuucauacccG...........    9    1    MW1
    .................................................................................................................................................ucagaucuacuucauacccau..........    1    0    MW1
    .................................................................................................................................................ucagaucuacCucauacccaug.........    130    1    MW1
    .................................................................................................................................................ucagaucuGcuucauacccaug.........    1    1    MW1
    .................................................................................................................................................ucagaucuacGucauacccauga........    1    1    MW1
    .................................................................................................................................................ucagaucuacuucauacccaugU........    1412    1    MW1
    .................................................................................................................................................ucagaucuacuucauacccauga........    1    0    MW1
    .................................................................................................................................................ucagaucuacCucauacccauga........    1    1    MW1
```

5'- gcuacgaucguggguauuuuagaucaucgacagaccaaggcacuuucgucacuggcauuguaaggccgagcagaugcggccucaacccaaacccucacccgugguucgcaucugcucccguccagugcccuguccaaccauacugucagaucuacuucauacccaugaaugguggcg-3'	reads	mm	sample
....ucUgaucuacuucauacccauga.........	3	1	MW1
....ucagaucuacuCcauacccauga.........	1	1	MW1
....ucagaucuacGucauacccauga.........	1	1	MW1
....ucagaucAacuucauacccauga.........	1	1	MW1
....ucagaucuacuucauacccGuga.........	1	1	MW1
....ucagaucuacuuUauacccauga.........	1	1	MW1
....ucagaucuacuucaCacccauga.........	2	1	MW1
....ucagaucuacuGcauacccauga.........	1	1	MW1
....Acagaucuacuucauacccauga.........	3	1	MW1
....ucagaucuacuucauacccauga.........	1	0	MW1
....ucagaAcuacuucauacccauga.........	1127	1	MW1
....ucagaucuacuucauacccauAaa........	1	1	MW1
....ucagaucuacuucauacccaugaa........	1	0	MW1
....ucagaucuacuucauacccaugCa........	1	1	MW1
....ucagaucuacuucauaUccaugaa........	1	1	MW1
....ucagaucuacuucauaccGaugaa........	4	1	MW1
....ucagaucuGcuucauacccaugaa........	2	1	MW1
....ucaCaucuacuucauacccaugaa........	1	1	MW1
....ucagaucuacuucauacccauAaa........	1	1	MW1
....ucagaucuacuucauacccaugaa........	1	0	MW1
....ucagUucuacuucauacccaugaa........	1	1	MW1
....ucagaucuacuucauacccaugaUu.......	1	1	MW1
....ucagaucuacuucauacccaugaau.......	3	0	MW1
....ucagaucuacCucauacccaugaau.......	266	1	MW1
....ucagaucuacuucauacccaugaaA.......	6	1	MW1
....ucagaucuacuucauacccaCgaau.......	105	1	MW1
....ucagaucuacuucauUcccaugaau.......	1	1	MW1
....ucagaucuacuucauacccaugaauA......	1	1	MW1
....cagaucuacuucauacccauga.........	3	0	MW1
....cagaucuacuucauacccaugaa........	4	0	MW1
....agaucuacuucauacccaugaa........	5	0	MW1
....agaucuacuucauacccaugaaA.......	1	1	MW1
....gaucuacuucauacccauga.........	1	0	MW1
....gaucuacuucauacccauga.........	1	0	MW1
....aucuacuucauacccauga.........	1	0	MW1
....aucuacuucauacccaugaaA.......	1	1	MW1
....cuacuucauacccauga.........	3	0	MW1
..uacgaucguggguauuuuagaucaucgacagac..	1	0	MW2
.........guggguauuuuagaucaucga...	1	0	MW2
.........guggguauuuuagaucaucgac..	1	0	MW2
.........guggguauuuuagaucaucgaca...	2	0	MW2
.........guggguauuuuagaucaucgaca...	1	0	MW2
.........guggguauuuuagaucaucgaca...	56	0	MW2
.........guggguauuuuagaucaucgacagaccaaggcac..	1	0	MW2
..........uggguauuuuagaucaucgaca...	1	0	MW2
...........ggguauuuuagaucaucgaca...	1	0	MW2
............gguauuuuagaucaucgacagaccaaggcac..	2	0	MW2
...............auuuuagaucaucgacagaccaaggcacuuucguca..	1	0	MW2
........................caucgacagaccaaggcacuuuc..	1	0	MW2
........................caucgacagaccaaggcacuuucgucacuggcaC...	1	1	MW2
............................gacagaccaCggcacuuucgucacu..	1	1	MW2

```
5'-gcuacgaucguggguauuuuagaucaucgacagaccaaggcacuuucgucacuggcauuguaaggccgagcagaugcggccucaacccaaacccucacccgugguucgcaucugcucccguccagugcccuguccaaccauacugucagaucuacuucauacccaugaaugguggcg-3'   reads   mm   sample
............................gacagaccaCggcacuuucgucacuggc.........................................................................................................................   1   1   MW2
...............................agaccaaggcacuuucgucacuggcaCuguaaggccgag...........................................................................................................   1   1   MW2
................................gaccaaggcacuuucgucacuggcaCuguaaggccgagcaga.......................................................................................................   1   1   MW2
.................................................cacuggcaCuguaaggc...............................................................................................................   1   1   MW2
.................................................cacuggcaCuguaaggccgagc..........................................................................................................   1   1   MW2
...................................................cuggcaCuguaaggccgagcagau......................................................................................................   1   1   MW2
.............................................................................................................aucugcucccguccagugcccuguccaaccau....................................   1   0   MW2
.............................................................................................................aucugcucccguccagugcccuguccaaccauacug................................   1   0   MW2
....................................................................................................................cccguccagugcccuguccaaccauacugucagaucuacuu....................   1   0   MW2
..........................................................................................................................................cauacugucagaucuacuucauacccaugaa........   1   0   MW2
................................................................................................................................................Cucagaucuacuucauacccauga.........   1   1   MW2
................................................................................................................................................gucagaucuacuucauacccaugaaA.......   1   1   MW2
.................................................................................................................................................ucagaucuacuucauac...............   3   0   MW2
.................................................................................................................................................ucagaucuacuucauacc..............   12   0   MW2
.................................................................................................................................................ucagaucuacuucauacc..............   2   0   MW2
.................................................................................................................................................ucagaucuacuucauaccUa............   8   1   MW2
.................................................................................................................................................ucagaucuacuucauacccau...........   2   0   MW2
.................................................................................................................................................ucagaucuacuucauacccaGg..........   31   1   MW2
.................................................................................................................................................ucagaucuacuuUauacccauga.........   1   1   MW2
.................................................................................................................................................ucagaucuacuucCuacccauga.........   1   1   MW2
.................................................................................................................................................ucagaucuaAuucauacccauga.........   1   1   MW2
.................................................................................................................................................ucagaucuacuucCuacccauga.........   1   1   MW2
.................................................................................................................................................ucagaucuacuucauacccaugC.........   1   1   MW2
.................................................................................................................................................ucagaucuacuucauacccauAa.........   1   1   MW2
.................................................................................................................................................ucagCucuacuucauacccauga.........   1   1   MW2
.................................................................................................................................................ucUgaucuacuucauacccauga.........   2   1   MW2
.................................................................................................................................................ucagaucuacuucauacccauAa.........   593   1   MW2
.................................................................................................................................................ucagaucuaAuucauacccaugaa........   1   1   MW2
.................................................................................................................................................ucagaucuacCucauacccaugaa........   2   1   MW2
.................................................................................................................................................ucagGucuacuucauacccaugaa........   1   1   MW2
.................................................................................................................................................ucagaucuacuucauacccaugaa........   1   0   MW2
.................................................................................................................................................ucagaucuacuucauacUcaugaa........   1   1   MW2
.................................................................................................................................................ucagaucuacuucauacccaugUa........   1   1   MW2
.................................................................................................................................................ucagaucuacuucauacccaugaC........   1   1   MW2
.................................................................................................................................................ucagaucuacuucauacccaugaa........   1   0   MW2
.................................................................................................................................................ucagaCcuacuucauacccaugaa........   1   1   MW2
.................................................................................................................................................ucagaucuacuucauacccaGgaa........   1   1   MW2
.................................................................................................................................................ucagaucuacuCcauacccaugaa........   684   1   MW2
.................................................................................................................................................ucagaucuacuucauacccaugaaA.......   214   1   MW2
.................................................................................................................................................ucagaucuacuucauacccaugaau.......   63   0   MW2
.................................................................................................................................................ucagaucuacuucauacccaugaaG.......   2   1   MW2
..................................................................................................................................................cagaucuacuucauacccauga.........   1   0   MW2
..................................................................................................................................................cagaucuacuucauacccaugaa........   1   0   MW2
...................................................................................................................................................agaucuacuucauacccauga.........   2   0   MW2
...................................................................................................................................................agaucuacuucauacccauga.........   2   0   MW2
...................................................................................................................................................agaucuacuucauacccaugaau.......   1   0   MW2
............................................................................................................................................uacugucagaucuacuucauacccaugaau.......   1   0   OV1
.................................................................................................................................................ucagaucuacuAcauacccauga.........   2   1   OV1
.................................................................................................................................................ucagaucuacuucauaUccaugaa........   3   1   OV1
.................................................................................................................................................uUagaucuacuucauacccauga.........   5   1   OV2
.................................................................................................................................................ucaCaucuacuucauacccaugaa........   3   1   OV2
.................................................................................................................................................ucagaCcuacuucauacccaugaau.......   1   1   OV2
.................................................................................................................................................ucagaucuacuucauacccaugaaA.......   1   1   OV2
```

5′- gcuacgaucguggguauuuuagaucaucgacagaccaaggcacuuucgucacuggcauuguaaggccgagcagaugcggccucaacccaaacccucacccgugguucgcaucugcucccguccagugcccuguccaaccauacugucagaucuacuucauacccaugaauggugggcg-3'	reads	mm	sample
.........guggguauuuuagaucaucgaca...	1	0	TE1
....ucagaucuacuucauacccaug..........	2	0	TE1
....uAagaucuacuucauacccauga.........	30	1	TE1
....ucagaucuacuucauaAccaugaa........	35	1	TE1
....ucagaucuacAucauacccaugaa........	1	1	TE1
....ucagaucuacuucauacccaugaUu.......	7	1	TE1
....ucagaucuacuuUauacccaugaau.......	5	1	TE1
....ucagaucuacuucauacccaugaaA.......	1	1	TE1
....ucagaucuacuucauacccaugaUu.......	1	1	TE1
.........guggguauuuuagauAaucgaca...	3	1	TE2
....ucagaucuacuucauaccca............	2	0	TE2
....ucagaucuacuGcauacccaug..........	2	1	TE2
....ucagaucuacuucauacccauga.........	1	0	TE2
....ucagaucuacuAcauacccauga.........	1	1	TE2
....ucagaucuacuucaAacccauga.........	39	1	TE2
....ucagaucuacuucauacUcaugaa........	34	1	TE2
....ucagaucuacuucauacccaugaaA.......	7	1	TE2
....ucagaucuacuCcauacccaugaau.......	1	1	TE2
....ucagaucuacuucauacccaugaau.......	6	0	TE2

miRBase precursor : aga-mir-1175
Total read count : 11567
aga-miR-1175 read count : 7844
aga-miR-1175* read count : 3720
remaining reads : 3

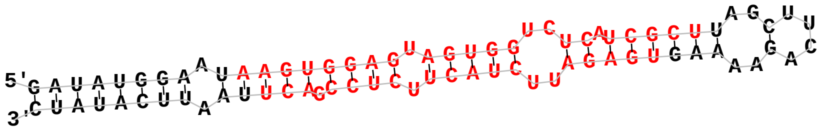

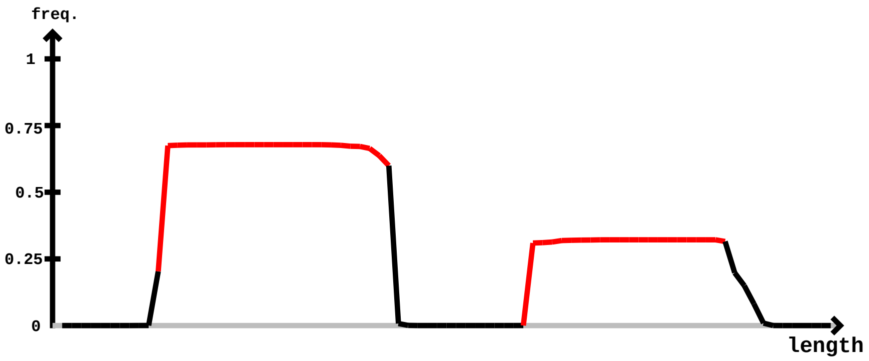

aga-miR-1175

aga-miR-1175*

```
5'- gauauggaauaaguggaguaguggucucaucgcuuagcuucagaaaagugagauucuacuucuccgacuuaauucauauc   -3'    exp
    ((((((((.((((((((.(((((..((.((((((..(....)..))))))))..))))).)))).))))).))))))))    reads      mm         sample
    .........uaaguggaguaguggucucaucgc...............................................    1          0          TE1
    .........uaaguggaguaguggucucaucgcu..............................................    1          0          TE1
    ..........aaguggaguaguggucucaucgcu..............................................    3          0          TE1
    ..........aaguggaguaguggucucaucgcG..............................................    1          1          TE1
    ................................................ugagauucuacuucuccgacuua.........    2          0          TE1

    .......aauaaguggaguaguggucucaucg................................................    2          0          FF2
    .........uaaguggaguaguggucu.....................................................    2          0          FF2
    .........uaaguggaguaguggucuc....................................................    1          0          FF2
    .........uaaguggaguaguggucuca...................................................    1          0          FF2
    .........uaaguggaguaguggucucau..................................................    1          0          FF2
    .........uaaguggaguaguggucucauc.................................................    2          0          FF2
    .........uaaguggaguaguggucucaucg................................................    5          0          FF2
    .........uaaguggaguaguggucucaucU................................................    2          1          FF2
    .........uaaguggaguaguggucucaucgc...............................................    12         0          FF2
    .........uaaguggaguaguggucucaucgcu..............................................    93         0          FF2
    .........uaagAggaguaguggucucaucgcu..............................................    1          1          FF2
    .........uaaguggaguagugguAucaucgcu..............................................    1          1          FF2
    .........uaaguggaguaguggucucaucgcG..............................................    1          1          FF2
    .........uaaguggaguaguggucucaucgcuu.............................................    1          0          FF2
    ..........aaguggaguaguggucucauc.................................................    2          0          FF2
    ..........aaguggaguaguggucucaucU................................................    2          1          FF2
    ..........aaguggaguaguggucucaucg................................................    7          0          FF2
    ..........aaguggaguaguggucucaucgc...............................................    11         0          FF2
    ..........aaguggaguaguggucucaucgcu..............................................    240        0          FF2
    ..........aaguggagCaguggucucaucgcu..............................................    1          1          FF2
    ..........aaguggaguaguggucucaucgcG..............................................    3          1          FF2
    ..........aaguggaguaguggucucaucgcA..............................................    1          1          FF2
    ..........aaguggaguaguggucucaucgcC..............................................    1          1          FF2
    ..........aaguggaguaguggucucaucgcuu.............................................    1          0          FF2
    ...........aguggaguaguggucucaucgcu..............................................    1          0          FF2
    ............guggaguaguggucucaucgcu..............................................    1          0          FF2
    ...............................gcuuagcuucagaaaag................................    1          0          FF2
    ................................................ugagauucuacuucuccgac............    6          0          FF2
```

```
          aga-miR-1175
                                          aga-miR-1175*
gauauggaauaaguggaguaguggucucaucgcuuagcuucagaaaagugagauucuacuucuccgacuuaauucauauc

................................................ugagaAucuacuucuccgac............   1      1    FF2
................................................ugagauucuacuucuccgacC...........   1      1    FF2
................................................ugagauucuacuucuccgacu...........   60     0    FF2
................................................ugagauucuacuucuccgacuu..........   25     0    FF2
................................................ugagauucuacuucuccgacuuG.........   1      1    FF2
................................................ugagauucuacuucuccgacuuU.........   1      1    FF2
................................................ugagauucuacuucuccgacuua.........   50     0    FF2
................................................ugagauucuacuucuccgacuuaC........   1      1    FF2
................................................ugagauucuacuucuccgacuuaa........   42     0    FF2
................................................ugagauucuacuucuccgacuuaU........   5      1    FF2
................................................ugagauucuacuucuccgacuuaaA.......   2      1    FF2
................................................ugagauucuacuucuccgacuuaau.......   2      0    FF2
.................................................gagauucuacuucuccgacuua.........   1      0    FF2
..................................................agauucuacuucuccgacuuaau.......   1      0    FF2

.........uaaguggaguagugguc......................................................   1      0    MF2
.........uaaguggaguaguggucu.....................................................   3      0    MF2
.........uaaguggaguaguggucuc....................................................   7      0    MF2
.........uaaguggaguaguggucuca...................................................   15     0    MF2
.........uaaguggaguaguggucucau..................................................   3      0    MF2
.........uaaguggaguaguggucucauU.................................................   4      1    MF2
.........uaaguggaguaguggucucauc.................................................   23     0    MF2
.........uaaguggaguaguggucucaucU................................................   23     1    MF2
.........uaaguggaguaguggucucaucg................................................   43     0    MF2
.........uaagugAaguaguggucucaucgc...............................................   1      1    MF2
.........Aaguggaguaguggucucaucgc...............................................   1      1    MF2
.........uaaguggaguaguggucucaucgc...............................................   163    0    MF2
.........uGaguggaguaguggucucaucgcu..............................................   1      1    MF2
.........uaaguggaguaguggucucauGgcu..............................................   2      1    MF2
.........uaaguggagAaguggucucaucgcu..............................................   1      1    MF2
.........uaaguggaguaguggucucaucgcC..............................................   1      1    MF2
.........uaaguggaguaguggucucaCcgcu..............................................   1      1    MF2
.........uaaguggaguagGggucucaucgcu..............................................   1      1    MF2
.........uCaguggaguaguggucucaucgcu..............................................   1      1    MF2
.........uaaguggaguaguggCcucaucgcu..............................................   2      1    MF2
.........Caaguggaguaguggucucaucgcu..............................................   1      1    MF2
.........uaaguggaguaguggucCcaucgcu..............................................   1      1    MF2
.........uaaguggaguaguggucucauAgcu..............................................   1      1    MF2
.........uaagugAaguaguggucucaucgcu..............................................   1      1    MF2
.........uaaguggaguGguggucucaucgcu..............................................   2      1    MF2
.........uaaguggaguaguggucucaucgcu..............................................   1477   0    MF2
.........uaaguggaguaguggucucaucgcA..............................................   2      1    MF2
.........uaaguggaguaguggucucaucgcG..............................................   12     1    MF2
.........uaaguggaguaguggucucaucUcu..............................................   1      1    MF2
.........uaaguggaguaguggucucauUgcu..............................................   2      1    MF2
.........uaaguggaguaguggucucaucgcAu.............................................   1      1    MF2
.........uaaguggaguaguggucucaucgcCu.............................................   1      1    MF2
.........uaaguggaguaguggucucaucgcuA.............................................   1      1    MF2
.........uaaguggagCaguggucucaucgcuu.............................................   1      1    MF2
.........uaaguggaguaguggucucaucgcuu.............................................   25     0    MF2
.........uaaguggaguaguggucucaucgcuua............................................   1      0    MF2
..........aaguggaguaguggucu.....................................................   1      0    MF2
..........aaguggaguaguggucuca...................................................   5      0    MF2
..........aaguggaguaguggucucauU.................................................   1      1    MF2
..........aaguggaguaguggucucauc.................................................   11     0    MF2
..........aaguggaguaguggucucaucg................................................   90     0    MF2
..........aaguggaguagAggucucaucg................................................   1      1    MF2
..........aaguggaguaguggucucaucA................................................   2      1    MF2
..........aaguggaguaguggucucaucU................................................   15     1    MF2
..........aaguggaguaguggucucaucgc...............................................   135    0    MF2
..........aagGggaguaguggucucaucgc...............................................   1      1    MF2
..........aaguggaguaguggucucaucgU...............................................   1      1    MF2
..........aaguggaguaguggucucaCcgcu..............................................   1      1    MF2
..........aaguggaguagCggucucaucgcu..............................................   3      1    MF2
..........aagCggaguaguggucucaucgcu..............................................   2      1    MF2
..........aaguggaAuaguggucucaucgcu..............................................   1      1    MF2
..........aaguggaguaguggucucaucgUu..............................................   1      1    MF2
..........aaguggaguaguggucucaucAcu..............................................   3      1    MF2
..........aaguggaguaguggucuUaucgcu..............................................   3      1    MF2
```

```
          aga-miR-1175
                                                aga-miR-1175*
gauauggaauaaguggaguaguggucucaucgcuuagcuucagaaaagugagauucuacuucuccgacuuaauucauauc

..........aaguggaguaguggucucaucgcG..............................................    32      1    MF2
..........aaguggagCaguggucucaucgcu..............................................    2       1    MF2
..........aUguggaguaguggucucaucgcu..............................................    1       1    MF2
..........aaguggaguagugAucucaucgcu..............................................    2       1    MF2
..........aaguggaguaguggucuAaucgcu..............................................    1       1    MF2
..........aaguggaguaguggucucaucgcu..............................................    3269    0    MF2
..........aaguggaguagAggucucaucgcu..............................................    3       1    MF2
..........aagGggaguaguggucucaucgcu..............................................    3       1    MF2
..........aaguggaguaguggucucauUgcu..............................................    3       1    MF2
..........aaguggaguGguggucucaucgcu..............................................    2       1    MF2
..........Uaguggaguaguggucucaucgcu..............................................    1       1    MF2
..........aaguggaguaguggCcucaucgcu..............................................    2       1    MF2
..........aaguggaguagugguGucaucgcu..............................................    1       1    MF2
..........Caguggaguaguggucucaucgcu..............................................    1       1    MF2
..........aaguggaguaAuggucucaucgcu..............................................    2       1    MF2
..........aaguggaguagugguUucaucgcu..............................................    1       1    MF2
..........aaguggaguaguggucucaucCcu..............................................    2       1    MF2
..........aaguggaguaguAgucucaucgcu..............................................    1       1    MF2
..........aaguggaguaguggucucaucgcC..............................................    3       1    MF2
..........aaguggagGaguggucucaucgcu..............................................    2       1    MF2
..........aaguggaguagGggucucaucgcu..............................................    1       1    MF2
..........aaguggaguaguggucucaucgcA..............................................    5       1    MF2
..........aaguggaguaguggucucaucgcuu.............................................    24      0    MF2
..........aaguggaguaguggucucaucgcAu.............................................    1       1    MF2
..........aaguggaguaguggucucaucgcuC.............................................    1       1    MF2
..........aaguggaguaguggucucaucgcuuU............................................    3       1    MF2
..........aaguggaguaguggucucaucgcuua............................................    1       0    MF2
...........aguggaguaguggucucaucgcu..............................................    8       0    MF2
............guggaguaguggucucaucgcG..............................................    1       1    MF2
............guggaguaguggucucaucgcu..............................................    1       0    MF2
...............................................Uugagauucuacuucuccgacu...........    1       1    MF2
................................................ugagauucuacuuAuccgac............    1       1    MF2
................................................ugagauucuacuucuccgac............    40      0    MF2
................................................ugagauucuacuucucGgacu...........    1       1    MF2
................................................ugagauucuacuucuccgacu...........    847     0    MF2
................................................ugagauucAacuucuccgacu...........    1       1    MF2
................................................ugagauucuacuucuccgacG...........    3       1    MF2
................................................ugagauucuacuucucUgacu...........    1       1    MF2
................................................ugagauucuacuucuccAacu...........    1       1    MF2
................................................ugagauucuacuucuccgacC...........    2       1    MF2
................................................ugagauucuaAuucuccgacu...........    1       1    MF2
................................................Cgagauucuacuucuccgacu...........    1       1    MF2
................................................ugagauucuacuuUccgacu...........    1       1    MF2
................................................ugagauucuCcuucuccgacu...........    1       1    MF2
................................................uAagauucuacuucuccgacu...........    1       1    MF2
................................................ugagaCucuacuucuccgacu...........    1       1    MF2
................................................ugagauucuacuucucAgacu...........    1       1    MF2
................................................ugagauucuacuucuccgacCu..........    6       1    MF2
................................................ugagauucuacuucuUcgacuu..........    1       1    MF2
................................................uAagauucuacuucuccgacuu..........    2       1    MF2
................................................ugagauucuacuucuccgacuu..........    360     0    MF2
................................................ugagauucuaGuucuccgacuua.........    1       1    MF2
................................................ugagauucuacuucuccgCcuua.........    1       1    MF2
................................................Cgagauucuacuucuccgacuua.........    1       1    MF2
................................................ugUgauucuacuucuccgacuua.........    1       1    MF2
................................................ugagauucuacuucucUgacuua.........    2       1    MF2
................................................ugagauucuacuucuccgacuua.........    506     0    MF2
................................................ugagauucuacuucuccgacuuG.........    1       1    MF2
................................................ugagauucuacuucuccgacuuU.........    5       1    MF2
................................................ugagauucuacuucuccCacuuaa........    1       1    MF2
................................................ugagauucuacuCcuccgacuuaa........    1       1    MF2
................................................ugagauucuacuucuccgacuGaa........    2       1    MF2
................................................ugagauucuacuAcuccgacuuaa........    1       1    MF2
................................................ugagauucuacCucuccgacuuaa........    1       1    MF2
................................................ugagauucuacuucuccgacuuaU........    44      1    MF2
................................................ugagauucuacuucuccgacuuaG........    1       1    MF2
................................................ugagauucuacuucuccgacuuaa........    528     0    MF2
................................................ugagauucuacuucuccgacuuaau.......    19      0    MF2
................................................ugagauucuacuucuccgacuuaaA.......    42      1    MF2
```

```
          aga-miR-1175
                                      aga-miR-1175*
gauauggaauaaguggaguaguggucucaucgcuuagcuucagaaaagugagauucuacuucuccgacuuaauucauauc

................................................ugagauucuacuucuccgacuuaaAu......   1     1     MF2
................................................ugagauucuacuucuccgacuuaauA......   1     1     MF2
.................................................gagauucuacuucuccgacuu..........   1     0     MF2
.................................................gagauucuacuucuccgacuua.........   4     0     MF2
.................................................gagauucuacuucuccgacuuaa........   4     0     MF2
.................................................gagauucuacuucuccgacuuaau.......   1     0     MF2
..................................................agauucCacuucuccgacu...........   1     1     MF2
..................................................agauucuacuucuccgacuua.........   1     0     MF2
..................................................agauucuacuucuccgacuuaa........   2     0     MF2
..................................................agauucuacuucuccgacuuaau.......   6     0     MF2
...................................................gauucuacuucuccgacuu..........   1     0     MF2
...................................................gauucuacuucuccgacuuaau.......   1     0     MF2

........Cuaaguggaguaguggucucaucgc...............................................   1     1     FW2
.........uaaguggaguaguggucucaucg................................................   1     0     FW2
.........uaaguggaguaguggucucaucU................................................   2     1     FW2
.........uaaguggaguaguggucucaucgc...............................................   9     0     FW2
.........uaaguAgaguaguggucucaucgcu..............................................   1     1     FW2
.........uaaguggaguaguggucAcaucgcu..............................................   1     1     FW2
.........uaaguggaguaguggucucaucgcu..............................................   53    0     FW2
..........aaguggaguaguggucu.....................................................   1     0     FW2
..........aaguggaguaguggucucau..................................................   1     0     FW2
..........aaguggaguaguggucucauc.................................................   8     0     FW2
..........aaguggaguaguggucucaucg................................................   14    0     FW2
..........aaguggaguaguggucucaucU................................................   2     1     FW2
..........aaguggaguaguggucucaucgc...............................................   17    0     FW2
..........aaguggaguagGgucucaucgcu...............................................   1     1     FW2
..........aaguggaguaguggucucaucgcG..............................................   1     1     FW2
..........aaguggaguaguggucucaucgcu..............................................   299   0     FW2
..........aaguggaguagAggucucaucgcu..............................................   1     1     FW2
..........aaguggaguaguggucucaucgcuu.............................................   2     0     FW2
..........aaguggaguaguggucucaucgcuuU............................................   2     1     FW2
...........aguggaguaguggucucaucgcu..............................................   3     0     FW2
............guggaguaguggucucaucgcu..............................................   1     0     FW2
...............gaguaguggucucaucgcu..............................................   1     0     FW2
................aguaguggucucaucgcu..............................................   2     0     FW2
................................................ugagauucuacuucuccgac............   5     0     FW2
................................................ugagauucuacuucuccgacu...........   80    0     FW2
................................................ugagaGucuacuucuccgacu...........   1     1     FW2
................................................ugagauucuacuucuccgacA...........   1     1     FW2
................................................ugagauucuacuucuccgacuu..........   20    0     FW2
................................................ugagauucuacuucuccgacuua.........   29    0     FW2
................................................ugagaCucuacuucuccgacuuaa........   1     1     FW2
................................................ugagauucuacuucuccgacuuaa........   56    0     FW2
................................................ugagauucuacuucuccgacuuaau.......   1     0     FW2
.................................................gagauucuacuucuccgacuuaa........   2     0     FW2
..................................................agauucuacuucuccgacu...........   4     0     FW2
..................................................agauucuacuucuccgacuuaa........   1     0     FW2
..................................................agauucuacuucuccgacuuaau.......   2     0     FW2
...................................................gauucuacuuGuccgacu...........   1     1     FW2
...................................................gauucuacuucuccgacu...........   7     0     FW2
...................................................gauucuacuucuccgacuu..........   1     0     FW2
...................................................gauucuacuucuccgacuua.........   2     0     FW2
...................................................gauucuacuucuccgacuuaa........   1     0     FW2
...................................................gauucuacuucuccgacuuaau.......   1     0     FW2
....................................................auucuacuucuccgacu...........   1     0     FW2
....................................................auucuacuucuccgacuuaaA.......   1     1     FW2
.....................................................uucuacuucuccgacuuaa........   1     0     FW2
......................................................ucuacuucuccgacuuaa........   1     0     FW2
.......................................................cuacuucuccgacuuaa........   3     0     FW2

...................................................gauucuacuucuccgacu...........   1     0     OV1
....................................................auucuacuucuccgacu...........   1     0     OV1

..........aaguggaguaguggucucaucgcu..............................................   1     0     FF1
...................................................Uauucuacuucuccgacuu..........   1     1     FF1

.........uaaguggaguaguggucuc....................................................   5     0     MF1
.........uaaguggaguaguggucucU...................................................   1     1     MF1
```

```
          aga-miR-1175
                                                aga-miR-1175*
gauauggaauaaguggaguaguggucucaucgcuuagcuucagaaaagugagauucuacuucuccgacuuaauucauauc

.........uaaguggaguaguggucuca...................................................    10      0       MF1
.........uaaguggaguaguggucucau..................................................    1       0       MF1
.........uaaguggaguaguggucucauc.................................................    5       0       MF1
.........uaaguggaguaguggucucauU.................................................    1       1       MF1
.........uaaguggaguaguggucucaucU................................................    2       1       MF1
.........uaaguggaguaguggucucaucg................................................    7       0       MF1
.........uaaguggaguaguggucucaucgc...............................................    19      0       MF1
.........uaaguggaguaguggucucaucgcu..............................................    121     0       MF1
.........uaaguggaguaguggucucaucgcC..............................................    1       1       MF1
.........uaaguggaguaguggCcucaucgcu..............................................    1       1       MF1
.........uaaguggaguaguggucucaucgcG..............................................    2       1       MF1
.........uaaguggaguaguggucucaucgcA..............................................    4       1       MF1
.........uaaguggaguaguggucucaucgcuu.............................................    1       0       MF1
.........uaaguggaguaguggucucaucgcCu.............................................    1       1       MF1
..........aaguggaguaguggucu.....................................................    1       0       MF1
..........aaguggaguaguggucuc....................................................    1       0       MF1
..........aaguggaguaguggucuca...................................................    3       0       MF1
..........aaguggaguaguggucucau..................................................    2       0       MF1
..........aaguggaguaguggucucauc.................................................    8       0       MF1
..........aaguggaguaguggucucaucU................................................    4       1       MF1
..........aaguggaguaguggucucaucg................................................    17      0       MF1
..........aaguggaguaguggucucaucgc...............................................    23      0       MF1
..........aaguggaguaguggucucauUgcu..............................................    1       1       MF1
..........aaguggaguaguggucucaucgcC..............................................    1       1       MF1
..........aaguggaguaguggucucaucgcu..............................................    417     0       MF1
..........aaguggaguaguggucucaucgcG..............................................    2       1       MF1
..........aagugAaguaguggucucaucgcu..............................................    1       1       MF1
..........aaguggaguaguggucuAaucgcu..............................................    1       1       MF1
..........aaguggaguaguggucucaucAcuu.............................................    1       1       MF1
..........aaguggaguaguggucucaucgcuA.............................................    1       1       MF1
..........aaguggaguaguggucucaucgcuu.............................................    7       0       MF1
................................................ugagauucuacuucuccga.............    1       0       MF1
................................................ugagauucuacuucuccgac............    4       0       MF1
................................................ugagauuGuacuucuccgacu...........    1       1       MF1
................................................uAagauucuacuucuccgacu...........    1       1       MF1
................................................ugagauucuacuucuccgacG...........    2       1       MF1
................................................ugagauucuacuucuccgacu...........    174     0       MF1
................................................ugagauucuacCucuccgacu...........    1       1       MF1
................................................ugagauucuacuucuccgacuu..........    34      0       MF1
................................................ugagauucuacuucuccAacuua.........    1       1       MF1
................................................ugagauucuacuucuccgacuuU.........    1       1       MF1
................................................ugagauucuacuucuccgacuua.........    61      0       MF1
................................................ugagauucuacuucuccgacuuaU........    4       1       MF1
................................................ugagauucuacuucuccgacuuaa........    35      0       MF1
................................................ugagauucuacuucuccgacuuaaA.......    3       1       MF1
................................................ugagauucuacuucuccgacuuaau.......    4       0       MF1
.................................................gagauucuacuuUuccgacuuaa........    1       1       MF1
.................................................gagauucuacuucuccgacuuaa........    2       0       MF1
..................................................agauucuacuucuccgacuuaau.......    2       0       MF1

................................................ugagauucuacuucuccgac............    1       0       BF2

..........aaguggaguaguggucucaucgcu..............................................    1       0       BF1
...................................................Uauucuacuucuccgacuu..........    1       1       BF1

.........uaaguggaguaguggucu.....................................................    2       0       MW1
.........uaaguggaguaguggucuc....................................................    1       0       MW1
.........uaaguggaguaguggucucau..................................................    1       0       MW1
.........uaaguggaguaguggucucauc.................................................    2       0       MW1
.........uaaguggaguaguggucucaucg................................................    5       0       MW1
.........uaaguggaguaguggucucaucU................................................    2       1       MW1
.........uaaguggaguaguggucucaucgc...............................................    8       0       MW1
.........uaaguggaguagCggucucaucgcu..............................................    1       1       MW1
.........uaaguggaguaguggucucaucgcu..............................................    71      0       MW1
.........uaaguggaguaguggucucaucgcG..............................................    1       1       MW1
..........aaguggaguaguggucuc....................................................    1       0       MW1
..........aaguggaguaguggucuca...................................................    1       0       MW1
..........aaguggaguaguggucucau..................................................    2       0       MW1
..........aaguggaguaguggucucauc.................................................    10      0       MW1
```

```
          aga-miR-1175
                                                aga-miR-1175*
gauauggaauaaguggaguaguggucucaucgcuuagcuucagaaaagugagauucuacuucuccgacuuaauucauauc

..........aaguggaguaguggucucaucU................................................    4     1     MW1
..........aaguggaguaguggucucaucg................................................    30    0     MW1
..........aaguggaguaguggucucaucgc...............................................    14    0     MW1
..........aaguggaguaguggucucaucgcu..............................................    304   0     MW1
..........aaguggaguaguggucucauUgcu..............................................    1     1     MW1
..........aaguggaguaguggucucaucgcG.............................................    3     1     MW1
..........aaguggaguaguggucuUaucgcu..............................................    1     1     MW1
..........aaguggaguaguggucucaucgcuu............................................    5     0     MW1
..........aaguggaguagGggucucaucgcuu............................................    1     1     MW1
...........aguggaguaguggucucaucgcu..............................................    2     0     MW1
............guggaguaguggucucaucgcu..............................................    3     0     MW1
............guggaguaguggucucaucgcuu.............................................    1     0     MW1
.............uggaguaguggucucaucgcu..............................................    1     0     MW1
...............gaguaguggucucaucgcu..............................................    2     0     MW1
.................guaguggucucaucgcu..............................................    1     0     MW1
................................................ugagauucuacuucuccgac............    6     0     MW1
................................................ugagauucuacuucuccgacu...........    87    0     MW1
................................................ugagauucuacuucuccgacG...........    2     1     MW1
................................................ugagauucuacuucuccgacuu..........    56    0     MW1
................................................ugagauucuacuucuccgacuC..........    1     1     MW1
................................................ugagauucuacuucuccgacCu..........    1     1     MW1
................................................ugagauucuacuucuccgacuua.........    70    0     MW1
................................................ugagauucuacuucuccgacuuU.........    1     1     MW1
................................................ugagauucuacuucuccgacuuaa........    45    0     MW1
................................................ugagauucuacuucuccgacuuaU........    1     1     MW1
................................................ugagauucuacuucuccgacuuaaA.......    2     1     MW1
................................................ugagauucuacuucuccgacuuaau.......    4     0     MW1
..................................................agauucuacuucuccgacu...........    4     0     MW1
..................................................agauucuacuucuccgacuuaa........    1     0     MW1
...................................................gauucuacuucuccgacu...........    6     0     MW1
...................................................gauucuacuucuccgacuu..........    11    0     MW1
...................................................gauucuacuucuccgacuua.........    6     0     MW1
...................................................gauucuacuucuccgacuuaa........    5     0     MW1
...................................................gauucuacuucuccgacuuaaA.......    1     1     MW1
....................................................auucuacuucuccgacu...........    1     0     MW1
....................................................auucuacuucuccgacuua.........    1     0     MW1
....................................................auucuacuucuccgacuuaa........    4     0     MW1
.....................................................uucuacuucuccgacuu..........    1     0     MW1
.....................................................uucuacuucuccgacuua.........    1     0     MW1
.....................................................uucuacuucuccgacuuaa........    1     0     MW1
.......................................................cuacuucuccgacuuaa........    3     0     MW1
........................................................uacuucuccgacuuaau.......    1     0     MW1

.........uaaguggaguaguggucucaucg................................................    3     0     FW1
.........uaaguggaguaguggucucaucgc...............................................    2     0     FW1
.........uaaguggaguaguggucucaucgcu..............................................    25    0     FW1
..........aaguggaguaguggucuca...................................................    1     0     FW1
..........aaguggaguaguggucucauc.................................................    5     0     FW1
..........aaguggaguaguggucucaucg................................................    12    0     FW1
..........aaguggaguaguggucucaucU................................................    4     1     FW1
..........aaguggaguaguggucucaucgc...............................................    7     0     FW1
..........aaguggaguaguggucucaucgcu..............................................    145   0     FW1
..........aaguAgaguaguggucucaucgcu..............................................    1     1     FW1
..........aagCggaguaguggucucaucgcu..............................................    1     1     FW1
..........aaguggaguaguggCcucaucgcu..............................................    1     1     FW1
..........Uaguggaguaguggucucaucgcu..............................................    1     1     FW1
..........aaguggaguaguggucucaucgcuu............................................    1     0     FW1
...........aguggaguaguggucucaucg................................................    1     0     FW1
...........aguggaguaguggucucaucgcu..............................................    2     0     FW1
............guggaguaguggucucaucgcu..............................................    3     0     FW1
..............ggaguaguggucucaucgcu..............................................    1     0     FW1
...............gaguaguggucucaucgcu..............................................    2     0     FW1
................aguaguggucucaucgcu..............................................    1     0     FW1
................................................ugagauucuacuucuccgac............    3     0     FW1
................................................ugagauucuacuucuccgacC...........    1     1     FW1
................................................ugagauucuacuucuccgacu...........    26    0     FW1
................................................ugagauucuacuucuccgacuu..........    23    0     FW1
................................................ugagauucuacuucuccgacuua.........    25    0     FW1
................................................ugagauucuacuucuccgacuuaa........    27    0     FW1
```

```
          aga-miR-1175
                                                  aga-miR-1175*
gauauggaauaaguggaguaguggucucaucgcuuagcuucagaaaagugagauucuacuucuccgacuuaauucauauc

................................................ugagauucuacuucuccgacuuaaA.......   5    1   FW1
..................................................agauucuacuucuccgac............   1    0   FW1
..................................................agauucuacuucuccgacuuaa........   2    0   FW1
...................................................gauucuacuucuccgac............   1    0   FW1
...................................................gauucuacuucuccgacu...........   2    0   FW1
...................................................gauucuacuucuccgacuu..........   5    0   FW1
...................................................gauucuacuucuccgacuua.........   3    0   FW1
....................................................auucuacuucuccgacu...........   1    0   FW1
.....................................................uucuacuucuccgacuua.........   1    0   FW1
.....................................................uucuacuucuccgacuuaU........   1    1   FW1
......................................................ucuacuucuccgacuua.........   1    0   FW1
......................................................ucuacuucuccgacuuaaA.......   1    1   FW1
.......................................................cuacuucuccgacuuaa........   2    0   FW1

.........uaaguggaguaguggucuc....................................................   1    0   MW2
.........uaaguggaguaguggucuca...................................................   1    0   MW2
.........uaaguggaguaguggucucauc.................................................   1    0   MW2
.........uaaguggaguaguggucucaucgc...............................................   2    0   MW2
.........uaaguggaguaguggucucaucgcu..............................................   19   0   MW2
..........aaguggaguaguggucuca...................................................   1    0   MW2
..........aaguggaguaguggucucauc.................................................   3    0   MW2
..........aaguggaguaguggucucaucU................................................   3    1   MW2
..........aaguggaguaguggucucaucg................................................   7    0   MW2
..........aaguggaguaguggucucaucgc...............................................   6    0   MW2
..........aagGggaguaguggucucaucgcu..............................................   1    1   MW2
..........aaguggaguaguggucucaucgcG..............................................   2    1   MW2
..........aaguggaguaguggucucaucgcu..............................................   109  0   MW2
..........aaguggaguaguggucucaucgcuu.............................................   2    0   MW2
...........aguggaguaguggucucaucgc...............................................   1    0   MW2
................aguaguggucucaucgcu..............................................   1    0   MW2
................................................ugagauucuacuucuccgac............   2    0   MW2
................................................ugagauucuacuucuccgacu...........   18   0   MW2
................................................ugagauucuacuucuccgacuu..........   5    0   MW2
................................................ugagauucuacuucuccgacuua.........   5    0   MW2
................................................ugagauucuacuucuccgacuuaa........   4    0   MW2
...................................................gauucuacuucuccgacu...........   2    0   MW2
...................................................gauucuacuucuccgacuu..........   2    0   MW2
...................................................gauucuacuucuccgacuuaa........   1    0   MW2
....................................................auucuacuucuccgacu...........   2    0   MW2
.....................................................uucuacuucuccgacuuaa........   1    0   MW2
.....................................................uucuacuucuccgacuuaau.......   1    0   MW2
......................................................ucuacuucuccgacuuaa........   1    0   MW2

..........aaguggaguaguggucucaucgcu..............................................   1    0   TE2
................................................ugagauucuacuucuccgacu...........   4    0   TE2
................................................ugaUauucuacuucuccgacu...........   1    1   TE2
```

miRBase precursor : aga-mir-11
Total read count : 129411
aga-miR-11 read count : 126248
remaining reads : 3163

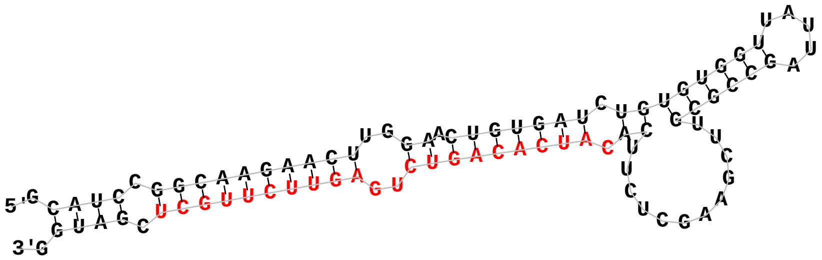

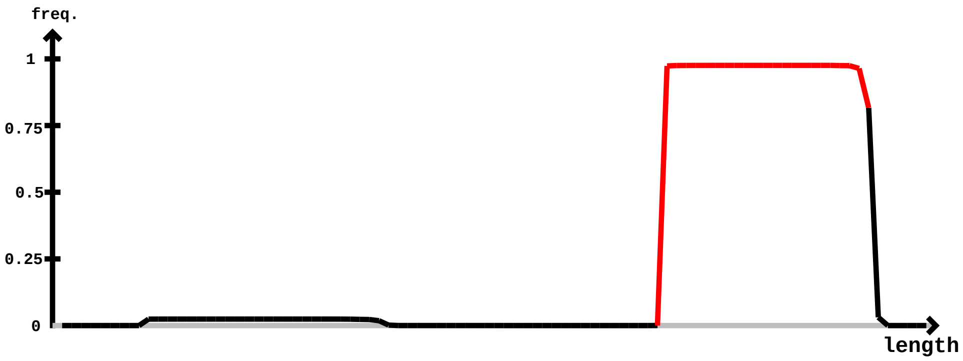

aga-miR-11

```
5'- gcauccggcaagaacuuggaacugugaucugugugguuauuagccgcguucgaagcucuucacaucacagucugaguucuugcucgaugg  -3'    exp
    .((((.((((((((((..((.(((((((....(((((.....)))))....(((....)))..)))))))))..)))))))))).)))).   reads   mm   sample
    ........caagaacuuggaacugugaucu............................................................   3       0    TE1
    ........caagaacuuggaacugugaucug...........................................................   3       0    TE1
    ........caagaacuuggaacugugaucugA..........................................................   1       1    TE1
    ........caagaacuuggaacugugaucugu..........................................................   12      0    TE1
    ........caagaacuuggaacugugaucuguU.........................................................   1       1    TE1
    ........caagaacuuggaacugugaucugug.........................................................   91      0    TE1
    ........caagaacCuggaacugugaucugug.........................................................   1       1    TE1
    ........caagaacuuggaacugugaucuguA.........................................................   1       1    TE1
    ........caagaacuuggaacugugaucugugu........................................................   1       0    TE1
    ........caagaacuuggaacugugaucugugA........................................................   4       1    TE1
    ..........agaacuuggaacugugaucugug.........................................................   1       0    TE1
    .............................................................Ccaucacagucugaguucuugcu......   2       1    TE1
    ..............................................................caucacagucugaguucu..........   5       0    TE1
    ..............................................................caucacagucugaguucuu.........   10      0    TE1
    ..............................................................caucacagucugaguucuug........   114     0    TE1
    ..............................................................cauUacagucugaguucuugc.......   1       1    TE1
    ..............................................................caucacagCcugaguucuugc.......   1       1    TE1
    ..............................................................caucacagucuAaguucuugc.......   3       1    TE1
    ..............................................................caucaUagucugaguucuugc.......   1       1    TE1
    ..............................................................caucacagucugaguuUuugc.......   1       1    TE1
    ..............................................................caucacagucugaguucuugA.......   2       1    TE1
    ..............................................................caucacagucugaguCcuugc.......   1       1    TE1
    ..............................................................caucacagucugaguucuuAc.......   1       1    TE1
    ..............................................................caucacagucugaguucuugc.......   1418    0    TE1
    ..............................................................caucacagucugaguucuCgc.......   1       1    TE1
    ..............................................................caucacagucugaAuucuugc.......   1       1    TE1
    ..............................................................caucacagucugaguuGuugc.......   1       1    TE1
    ..............................................................caucacagucCgaguucuugc.......   1       1    TE1
    ..............................................................caucacagucugaguGcuugcu......   1       1    TE1
    ..............................................................caucacagucuCaguucuugcu......   2       1    TE1
    ..............................................................caucacagCcugaguucuugcu......   4       1    TE1
    ..............................................................caucacagucugagGucuugcu......   1       1    TE1
    ..............................................................caucacaguGugaguucuugcu......   1       1    TE1
    ..............................................................caucacagucuAaguucuugcu......   5       1    TE1
    ..............................................................caucacaguUugaguucuugcu......   1       1    TE1
```

aga-miR-11

```
gcauccggcaagaacuuggaacugugaucugugugguuauuagccgcguucgaagcucuucacaucacagucugaguucuugcucgaugg
..............................................................caucacaUucugaguucuugcu......   2      1   TE1
..............................................................caucacagucugaguuUuugcu......   1      1   TE1
..............................................................caucacagucugagCucuugcu......   2      1   TE1
..............................................................cauUacagucugaguucuugcu......   2      1   TE1
..............................................................caucacagucugaguucuCgcu......   1      1   TE1
..............................................................caucacagucugaguuAuugcu......   2      1   TE1
..............................................................caucacagucugaguucuuAcu......   3      1   TE1
..............................................................caucacagucugaguucuugcu......   4692   0   TE1
..............................................................caucacagucugaguucuugcC......   11     1   TE1
..............................................................caucacUgucugaguucuugcu......   1      1   TE1
..............................................................caucaUagucugaguucuugcu......   2      1   TE1
..............................................................caucacaguAugaguucuugcu......   4      1   TE1
..............................................................caucacagucugaguucuuCcu......   1      1   TE1
..............................................................caucacGgucugaguucuugcu......   2      1   TE1
..............................................................cGucacagucugaguucuugcu......   1      1   TE1
..............................................................caucacagucCgaguucuugcu......   5      1   TE1
..............................................................Uaucacagucugaguucuugcu......   3      1   TE1
..............................................................caucacagucugaguucCugcu......   1      1   TE1
..............................................................caucGcagucugaguucuugcu......   2      1   TE1
..............................................................caucacagucugaguucuugcG......   9      1   TE1
..............................................................caucaAagucugaguucuugcu......   1      1   TE1
..............................................................caucacagucugaguucAugcu......   1      1   TE1
..............................................................caucacagucuUaguucuugcu......   5      1   TE1
..............................................................caucacagucugagAucuugcu......   2      1   TE1
..............................................................caucacagucugaguCcuugcu......   1      1   TE1
..............................................................caucacagucugaguucuugcA......   25     1   TE1
..............................................................caucacagucugCguucuugcu......   1      1   TE1
..............................................................caucacagucugaguucuugcuA.....   35     1   TE1
..............................................................caucacagucugaguucuugcuG.....   4      1   TE1
..............................................................caucacagucugaguucuugcuU.....   72     1   TE1
...............................................................aucacagucugaguucuugc.......   2      0   TE1
...............................................................aucacagucugaguucuugcu......   6      0   TE1

........caagaacuuggaacugugauc.............................................................   3      0   OV2
........caagaacuuggaacugugaucu............................................................   1      0   OV2
........caagaacuuggaacugugaucug...........................................................   4      0   OV2
........caagaacuuCgaacugugaucugu..........................................................   1      1   OV2
........caagaacuuggaacugugaucugu..........................................................   12     0   OV2
........caagaacuuggaacugugaucuguU.........................................................   1      1   OV2
........caagaacuuggaacugugaucugug.........................................................   104    0   OV2
........caagaacuuggaacugugaucugugA........................................................   10     1   OV2
........caagaacuuggaacugugaucugugG........................................................   4      1   OV2
.........aagaacuuggaacugugaucugug.........................................................   1      0   OV2
..........agaacuuggaacugugaucugu..........................................................   1      0   OV2
..........agaacuuggaacugugaucugug.........................................................   2      0   OV2
.............................................................Ucaucacagucugaguucuugcu......   1      1   OV2
.............................................................acaucacagucugaguucuugcu......   2      0   OV2
.............................................................Ccaucacagucugaguucuugcu......   4      1   OV2
..............................................................caucacagucugaguucu..........   8      0   OV2
..............................................................caucacagucugaguucCu.........   1      1   OV2
..............................................................caucacagucugaguucuu.........   1      0   OV2
..............................................................caucacagucugaguucuuU........   1      1   OV2
..............................................................caucacagucugaguucuug........   103    0   OV2
..............................................................caucacagucuUaguucuugc.......   1      1   OV2
..............................................................caucacagucCgaguucuugc.......   3      1   OV2
..............................................................caucacagucugaguucuugA.......   3      1   OV2
..............................................................caucacagucugaguucuugG.......   2      1   OV2
..............................................................caucacagucugaguCcuugc.......   1      1   OV2
..............................................................caucacagucugaguucuugc.......   2162   0   OV2
..............................................................caucacagucugagCucuugc.......   1      1   OV2
..............................................................caucacaguUugaguucuugc.......   1      1   OV2
..............................................................caucacGgucugaguucuugc.......   1      1   OV2
..............................................................caucacaAucugaguucuugc.......   1      1   OV2
..............................................................cauUacagucugaguucuugc.......   1      1   OV2
..............................................................caucacagucuAaguucuugc.......   1      1   OV2
..............................................................caucacagucGgaguucuugc.......   1      1   OV2
..............................................................caucacagGcugaguucuugc.......   1      1   OV2
..............................................................caucacagucugagAucuugcu......   1      1   OV2
..............................................................Uaucacagucugaguucuugcu......   7      1   OV2
..............................................................caucacagucugagCucuugcu......   10     1   OV2
```

aga-miR-11

gcauccggcaagaacuuggaacugugaucugugugguuauuagccgcguucgaagcucuucacaucacagucugaguucuugcucgaugg			
....caucacagucugaguucuAgcu......	1	1	OV2
....caucaAagucugaguucuugcu......	1	1	OV2
....caucacagucuCaguucuugcu......	5	1	OV2
....caucacagucugaguucuugGu......	1	1	OV2
....caucacagCcugaguucuugcu......	3	1	OV2
....caucacaguUugaguucuugcu......	1	1	OV2
....caucacagucugaAuucuugcu......	1	1	OV2
....Gaucacagucugaguucuugcu......	1	1	OV2
....caucacagucugaguucCugcu......	7	1	OV2
....caucacagucugaguucuugcA......	36	1	OV2
....caucacagucuUaguucuugcu......	6	1	OV2
....caucacagucugaguucuuAcu......	2	1	OV2
....cauUacagucugaguucuugcu......	11	1	OV2
....Aaucacagucugaguucuugcu......	1	1	OV2
....caucacagucugaguucuGgcu......	2	1	OV2
....caucacagucCgaguucuugcu......	5	1	OV2
....caucacagucGgaguucuugcu......	2	1	OV2
....caucCcagucugaguucuugcu......	1	1	OV2
....caucacagucugaguucuugcu......	11717	0	OV2
....caAcacagucugaguucuugcu......	1	1	OV2
....caucacagucugaguuAuugcu......	3	1	OV2
....caucacagucugaUuucuugcu......	2	1	OV2
....caucacagucugaguucuugcG......	15	1	OV2
....caucacaAucugaguucuugcu......	3	1	OV2
....caucacagucugaguCcuugcu......	3	1	OV2
....caucacagucuAaguucuugcu......	14	1	OV2
....caucacaUucugaguucuugcu......	1	1	OV2
....caucacagucugaguucuCgcu......	5	1	OV2
....caucacagucugaguucuugcC......	36	1	OV2
....caucacagGcugaguucuugcu......	1	1	OV2
....caucacagAcugaguucuugcu......	1	1	OV2
....caucaUagucugaguucuugcu......	3	1	OV2
....caucacagucugGguucuugcu......	2	1	OV2
....caucGcagucugaguucuugcu......	1	1	OV2
....caCcacagucugaguucuugcu......	3	1	OV2
....caucUcagucugaguucuugcu......	1	1	OV2
....caucacagucugaguucuugcuA.....	144	1	OV2
....caucacagucugaguucuugcuG.....	17	1	OV2
....caucacagucugaguucuugcuU.....	377	1	OV2
....caucacagucugaguucuugcuc.....	26	0	OV2
....caucacagucugaguucuugcucU....	1	1	OV2
....aucacagucugaguucuugc.......	5	0	OV2
....aucacagucugaguucuugcu......	24	0	OV2
....acagucugaguucuugcu......	1	0	OV2
.....cggcaagaacuuggaacugugau..	1	0	FF2
........caagaacuuggaacugugauc...	1	0	FF2
........caagaacuuggaacugugaucu..	3	0	FF2
........caagaacuuggaacugugaucug...	2	0	FF2
........caagaacuuggaacugugaucugu..	8	0	FF2
........caagaacuuggaacugugaucugug...	57	0	FF2
........caagaacuuggaacugugaucuguU...	1	1	FF2
........caagaacuuggaacugugaucuguA...	1	1	FF2
........caagaacuuggaacugugaucugugA..	1	1	FF2
........caagaacuuggaacugugaucugugG..	1	1	FF2
........caagaacuuggaacugugaucugugC..	1	1	FF2
........caagaacuuggaacugugaucugugu..	2	0	FF2
........caagaacuuggaacugugaucugugugguuauuagccg..	1	0	FF2
....caucacagucugaguuc...........	1	0	FF2
....caucacagucugaguucu..........	1	0	FF2
....caucacagucugaguucuug........	20	0	FF2
....caucacagucugagCucuugc.......	1	1	FF2
....caucacagucuAaguucuugc.......	2	1	FF2
....cUucacagucugaguucuugc.......	1	1	FF2
....caucacagucugaguucuugU.......	1	1	FF2
....caucacagucugaguucuugc.......	320	0	FF2
....caucaUagucugaguucuugc.......	1	1	FF2
....caucacagucugaguucuugA.......	1	1	FF2
....caucacaUucugaguucuugc.......	1	1	FF2
....caucacUgucugaguucuugc.......	1	1	FF2

aga-miR-11

```
gcauccggcaagaacuuggaacugugaucugugugguuauuagccgcguucgaagcucuucacaucacagucugaguucuugcucgaugg
..............................................................caucacagucugaguuGuugc.......  1     1     FF2
..............................................................caucacagucugaguucuuAcu......  1     1     FF2
..............................................................caucacagucugaguuGuugcu......  1     1     FF2
..............................................................caucacagucugaguucuugcG......  9     1     FF2
..............................................................caucacagucugaguucuugcu......  1871  0     FF2
..............................................................caucacagucugaguucuugcA......  1     1     FF2
..............................................................Uaucacagucugaguucuugcu......  1     1     FF2
..............................................................caucacagucuUaguucuugcu......  2     1     FF2
..............................................................caucacagucugaguucuCgcu......  1     1     FF2
..............................................................caucacagucuAaguucuugcu......  1     1     FF2
..............................................................cUucacagucugaguucuugcu......  1     1     FF2
..............................................................cGucacagucugaguucuugcu......  1     1     FF2
..............................................................caucacagucugaguucuugUu......  3     1     FF2
..............................................................caucacagucugaAuucuugcu......  1     1     FF2
..............................................................caucacagucugaguucuuCcu......  1     1     FF2
..............................................................caucacagucugagCucuugcu......  1     1     FF2
..............................................................caucacagucugaguucuugcC......  4     1     FF2
..............................................................caucUcagucugaguucuugcu......  1     1     FF2
..............................................................caucacagucuCaguucuugcu......  1     1     FF2
..............................................................caucacagucugaguCcuugcu......  1     1     FF2
..............................................................caucacagCcugaguucuugcu......  2     1     FF2
..............................................................caucacaguUugaguucuugcu......  1     1     FF2
..............................................................caucacaguGugaguucuugcu......  1     1     FF2
..............................................................caucacagucugaguucuugcuA.....  5     1     FF2
..............................................................caucacagucugaguucuugcuc.....  4     0     FF2
..............................................................caucacagucugaguucuugcCc.....  1     1     FF2
..............................................................caucacagucugaguucuugcuG.....  3     1     FF2
..............................................................caucacagucugaguucuugcuU.....  49    1     FF2
...............................................................aucacagucugaguucuugcu......  3     0     FF2
...................................................................cagucugaguucuugcu......  1     0     FF2

........caagaacuuggaacugugau..............................................................  1     0     MF2
........caagaacuuggaacugugauc.............................................................  7     0     MF2
........caagaacuuggaacugugaucu............................................................  15    0     MF2
........caagaacuuggaacugugaucug...........................................................  15    0     MF2
........caagaacuuggaacugugaucugu..........................................................  123   0     MF2
........caaAacuuggaacugugaucugu..........................................................  1     1     MF2
........caagaacuuggaacugugaucugG..........................................................  1     1     MF2
........caagaacuuggaacugugaucugGg.........................................................  1     1     MF2
........caagaacuuggaacugugaucuguU.........................................................  4     1     MF2
........caagaacuuggaacugugaucGgug.........................................................  1     1     MF2
........caagaacuugUaacugugaucugug.........................................................  2     1     MF2
........caagaacuuggaacugugaucugug.........................................................  610   0     MF2
........caagaacuuggGacugugaucugug.........................................................  2     1     MF2
........caagaacuuggaacugugaucuguA.........................................................  3     1     MF2
........caagaacuuggaacugugaucuAug.........................................................  2     1     MF2
........caagaacuuggaacugugaucugugu........................................................  9     0     MF2
........caagaacuuggaacugugaucugugG........................................................  3     1     MF2
........caagaacuuggaacugugaucugugA........................................................  36    1     MF2
........caagaacuuggaacugugaucuguAu........................................................  2     1     MF2
........caagaacuuggaacugugaucugugC........................................................  7     1     MF2
........caagaacuuggaacugugaucuguguU.......................................................  2     1     MF2
........caagaacuuggaacugugaucuguguA.......................................................  1     1     MF2
........caagaacuuggaacugugaucugugugguuauua................................................  1     0     MF2
........caagaacuuggaacugugaucugugugguuauuagcc.............................................  1     0     MF2
........caagaacuuggaacugugaucugugugguuauuagccgcgu.........................................  2     0     MF2
.................................ugguuauuagccgcguucgaagcucuuca............................  1     0     MF2
.............................................................acaucacagucugaguucuugc.......  1     0     MF2
.............................................................acaucacagucugaguucuugcu......  2     0     MF2
.............................................................Ccaucacagucugaguucuugcu......  4     1     MF2
..............................................................caucacagucugaguucu..........  8     0     MF2
..............................................................caucacagucugaguucuu.........  6     0     MF2
..............................................................caucUcagucugaguucuug........  1     1     MF2
..............................................................caucacagucugaguucuug........  230   0     MF2
..............................................................caucacagucugaguucCugc.......  1     1     MF2
..............................................................cGucacagucugaguucuugc.......  1     1     MF2
..............................................................caucacagucugaguucuugU.......  1     1     MF2
..............................................................caCcacagucugaguucuugc.......  2     1     MF2
..............................................................caucacaguGugaguucuugc.......  1     1     MF2
..............................................................caucacagucugagCucuugc.......  2     1     MF2
```

aga-miR-11

```
gcauccggcaagaacuuggaacugugaucugugugguuauuagccgcguucgaagcucuucacaucacagucugaguucuugcucgaugg
..............................................................caucacagucugaguuUuugc.......  2      1  MF2
..............................................................caucacagucugaguucuCgc.......  1      1  MF2
..............................................................caucacagucGgaguucuugc.......  1      1  MF2
..............................................................caucaUagucugaguucuugc.......  1      1  MF2
..............................................................caucacagCcugaguucuugc.......  5      1  MF2
..............................................................caucacagucuCaguucuugc.......  1      1  MF2
..............................................................caucacagucuUaguucuugc.......  3      1  MF2
..............................................................caucacagucuAaguucuugc.......  2      1  MF2
..............................................................caucacagucugaguAcuugc.......  1      1  MF2
..............................................................cauUacagucugaguucuugc.......  3      1  MF2
..............................................................caucacagucugaguucuugc.......  3135   0  MF2
..............................................................caucacagucugagGucuugc.......  1      1  MF2
..............................................................Uaucacagucugaguucuugc.......  1      1  MF2
..............................................................caucacagucugaguucuGgc.......  1      1  MF2
..............................................................caucacagucugaguuAuugc.......  1      1  MF2
..............................................................caucacagucugaguucuugA.......  4      1  MF2
..............................................................caucacagucAgaguucuugc.......  1      1  MF2
..............................................................caucacagucugaguuGuugc.......  1      1  MF2
..............................................................caucacagucugaAuucuugc.......  1      1  MF2
..............................................................caucaGagucugaguucuugcu......  1      1  MF2
..............................................................caucacaguGugaguucuugcu......  2      1  MF2
..............................................................caucacagucugaCuucuugcu......  2      1  MF2
..............................................................caucacagucugaguucuugcC......  67     1  MF2
..............................................................caucacagucuCaguucuugcu......  4      1  MF2
..............................................................caucacaAucugaguucuugcu......  5      1  MF2
..............................................................caucacagGcugaguucuugcu......  2      1  MF2
..............................................................caucacagCcugaguucuugcu......  12     1  MF2
..............................................................caucGcagucugaguucuugcu......  1      1  MF2
..............................................................caucacaguAugaguucuugcu......  4      1  MF2
..............................................................Aaucacagucugaguucuugcu......  5      1  MF2
..............................................................caucaUagucugaguucuugcu......  10     1  MF2
..............................................................caucacagucugUguucuugcu......  1      1  MF2
..............................................................caucacagucGgaguucuugcu......  3      1  MF2
..............................................................caucacagucugaguucuuAcu......  8      1  MF2
..............................................................caucacagucugaguuAuugcu......  3      1  MF2
..............................................................Uaucacagucugaguucuugcu......  4      1  MF2
..............................................................caucacagucugaguucuugGu......  1      1  MF2
..............................................................caucacagucugaguucuuUcu......  1      1  MF2
..............................................................caucacGgucugaguucuugcu......  4      1  MF2
..............................................................caucacagucugaguucCugcu......  8      1  MF2
..............................................................caAcacagucugaguucuugcu......  1      1  MF2
..............................................................cauUacagucugaguucuugcu......  11     1  MF2
..............................................................caucacagucugaguucuCgcu......  7      1  MF2
..............................................................caucUcagucugaguucuugcu......  2      1  MF2
..............................................................caucacagucugagCucuugcu......  7      1  MF2
..............................................................cauGacagucugaguucuugcu......  1      1  MF2
..............................................................caucacagucugaguucuugcG......  51     1  MF2
..............................................................caucacaUucugaguucuugcu......  1      1  MF2
..............................................................Gaucacagucugaguucuugcu......  3      1  MF2
..............................................................caucacagucugaguucuugcA......  49     1  MF2
..............................................................cGucacagucugaguucuugcu......  4      1  MF2
..............................................................caucacagucugaguucuAgcu......  1      1  MF2
..............................................................caucacagucugaguuUuugcu......  11     1  MF2
..............................................................caCcacagucugaguucuugcu......  6      1  MF2
..............................................................caucacagucugCguucuugcu......  1      1  MF2
..............................................................caucacagucugaguucuugAu......  1      1  MF2
..............................................................caucacagucCgaguucuugcu......  7      1  MF2
..............................................................caucacagucugaAuucuugcu......  1      1  MF2
..............................................................caucacagucuUaguucuugcu......  8      1  MF2
..............................................................caucacagucugaguucuugUu......  2      1  MF2
..............................................................cCucacagucugaguucuugcu......  1      1  MF2
..............................................................caucacagucuAaguucuugcu......  19     1  MF2
..............................................................caucacagucugGguucuugcu......  3      1  MF2
..............................................................caucacagucugaguucuugcu......  18618  0  MF2
..............................................................caucacagucugaguCcuugcu......  4      1  MF2
..............................................................caucacaguUugaguucuugcu......  7      1  MF2
..............................................................cUucacagucugaguucuugcu......  2      1  MF2
..............................................................caucacagucugagGucuugcu......  6      1  MF2
..............................................................caucacagucugaguucuugcuA.....  54     1  MF2
..............................................................caucacagucugaguucuugcuU.....  400    1  MF2
```

aga-miR-11

gcauccggcaagaacuuggaacugugaucugugugguuauuagccgcguucgaagcucuucacaucacagucugaguucuugcucgaugg			
....caucacagucugaguucuugcuc.....	29	0	MF2
....caucacagucugaguucuugcuG.....	25	1	MF2
....aucacagucugaguucuugc.......	11	0	MF2
....aucacagucugaguucuugcu......	28	0	MF2
....aucacagucugaguucuugcuU.....	2	1	MF2
....acagucugaguucuugcu......	1	0	MF2
........caagaacuuggaacugugauc...	3	0	FW2
........caagaacuuggaacugugaucu..	8	0	FW2
........caagaacuuggaacugugaucug...	13	0	FW2
........caagaacuuggaacugugaucugu..	50	0	FW2
........caagaacuuggaacugugaucugug...	73	0	FW2
........caagaacuuggaacuAugaucugug...	1	1	FW2
........caagaacuuggaacugugaucuguU...	1	1	FW2
........caagaacuuggaacugugaucuguA...	3	1	FW2
........caagaacuuggaacugugaucugugA..	3	1	FW2
........caagaacuuggaacugugaucugugG..	1	1	FW2
.........aagaacuuggaacugugaucu..	1	0	FW2
..........agaacuuggaacugugaucugu..	1	0	FW2
....caucacagucugaguucu..........	3	0	FW2
....caucacagucugaguucuu.........	1	0	FW2
....caucacagucugaguucuug........	20	0	FW2
....caucacagucugaguucuugc.......	361	0	FW2
....caucacagucAgaguucuugc.......	1	1	FW2
....caucacagucugagCucuugc.......	1	1	FW2
....cGucacagucugaguucuugc.......	1	1	FW2
....caucacagucuUaguucuugcu......	1	1	FW2
....caCcacagucugaguucuugcu......	2	1	FW2
....Aaucacagucugaguucuugcu......	1	1	FW2
....caucacagucugaguucuuUcu......	1	1	FW2
....caucacagucugaguuAuugcu......	1	1	FW2
....caucacagucuAaguucuugcu......	2	1	FW2
....caucacagucugaguucCugcu......	1	1	FW2
....caucacagucGgaguucuugcu......	1	1	FW2
....caucacagucugaUuucuugcu......	1	1	FW2
....caucacagCcugaguucuugcu......	1	1	FW2
....caucacagucugaguucuugcC......	5	1	FW2
....cauUacagucugaguucuugcu......	2	1	FW2
....caucacagucugaguucuugcA......	1	1	FW2
....cGucacagucugaguucuugcu......	1	1	FW2
....caAcacagucugaguucuugcu......	1	1	FW2
....caucacagucCgaguucuugcu......	1	1	FW2
....caucacagucugaguucuugcu......	2674	0	FW2
....caucacagucuCaguucuugcu......	1	1	FW2
....caucacagucugaguucuugcG......	1	1	FW2
....caucacagucugaguucuugGu......	1	1	FW2
....caucacagucugGguucuugcu......	1	1	FW2
....caucacagucugaguucuugAu......	1	1	FW2
....caucacaAucugaguucuugcu......	1	1	FW2
....caucacagucugaguucuCgcu......	1	1	FW2
....caucaUagucugaguucuugcu......	1	1	FW2
....caucacagucugagGucuugcu......	1	1	FW2
....caucacagucugaguucuugcuG.....	3	1	FW2
....caucacagucugaguucuugcuA.....	6	1	FW2
....caucacagucugaguucuugcuc.....	5	0	FW2
....caucacagucugaguucuugcuU.....	53	1	FW2
....aucacagucugaguucuugcu......	4	0	FW2
....ucacagucugaguucuugc.......	1	0	FW2
....ucacagucugaguucuugcu......	1	0	FW2
....cacagucugaguucuugcu......	3	0	FW2
....cagucugaguucuugcu......	2	0	FW2
........caagaacuuggaacugugauc...	5	0	OV1
........caagaacuuggaacugugauA...	2	1	OV1
........caagaacuuggaacuguAauc...	1	1	OV1
........caagaacuuggaacugugaucu..	15	0	OV1
........caagaacuuggaacugugaucug...	2	0	OV1
........caagaacuuggaaGugugaucug...	1	1	OV1
........caagaacuuggaacugugaucugu..	39	0	OV1
........caagaacuuUgaacugugaucugug...	1	1	OV1

aga-miR-11

```
gcauccggcaagaacuuggaacugugaucugugugguuauuagccgcguucgaagcucuucacaucacagucugaguucuugcucgaugg
........caagaacuuggaacugugaucuguA.........................................................  2      1   OV1
........caagaacuuggaacugugaucuAug.........................................................  1      1   OV1
........caagaacuuggaacCgugaucugug.........................................................  1      1   OV1
........caagaacuuggaacugugaucugug.........................................................  175    0   OV1
........caagaacuuggaacugugaucugugu........................................................  8      0   OV1
........caagaacuuggaacugugaucugugC........................................................  1      1   OV1
........caagaacuuggaacugugaucugugG........................................................  2      1   OV1
........caagaacuuggaacugugaucugugA........................................................  13     1   OV1
........caagaacuuggaacugugaucuguguggu.....................................................  1      0   OV1
.........aagaacuuggaacugugauc.............................................................  1      0   OV1
.........aagaacuuggaacugugaucugug.........................................................  1      0   OV1
..........agaacuuggaacugugaucuguguggu.....................................................  1      0   OV1
.............................................................acaucacagucugaguucuugc.......  1      0   OV1
.............................................................Ccaucacagucugaguucuugcu......  1      1   OV1
..............................................................caucacagucugaguucu..........  15     0   OV1
..............................................................caucacagucugaguucuu.........  5      0   OV1
..............................................................caucacagucugagGucuug........  1      1   OV1
..............................................................caucacagucugaguucuug........  94     0   OV1
..............................................................caucacaAucugaguucuugc.......  1      1   OV1
..............................................................caucacagucugaguuAuugc.......  1      1   OV1
..............................................................caucacagucugaguucuugc.......  2658   0   OV1
..............................................................caucacagucuCaguucuugc.......  2      1   OV1
..............................................................caucacagucugagCucuugc.......  2      1   OV1
..............................................................caucacagucugCguucuugc.......  1      1   OV1
..............................................................caucacagCcugaguucuugc.......  2      1   OV1
..............................................................Aaucacagucugaguucuugc.......  1      1   OV1
..............................................................caucaAagucugaguucuugc.......  2      1   OV1
..............................................................caucacagucugaguucGugc.......  1      1   OV1
..............................................................cauUacagucugaguucuugc.......  1      1   OV1
..............................................................Uaucacagucugaguucuugc.......  2      1   OV1
..............................................................caucacagucCgaguucuugc.......  2      1   OV1
..............................................................caucacaUucugaguucuugc.......  1      1   OV1
..............................................................caucaUagucugaguucuugc.......  1      1   OV1
..............................................................caucacagucugaguucuugU.......  1      1   OV1
..............................................................caucacagucuAaguucuugc.......  3      1   OV1
..............................................................caucacagucugaguuGuugc.......  1      1   OV1
..............................................................caucacaguAugaguucuugc.......  1      1   OV1
..............................................................caucacagucugagGucuugc.......  1      1   OV1
..............................................................caucacagucugaguucuGgc.......  1      1   OV1
..............................................................caucacagucugaguCcuugc.......  2      1   OV1
..............................................................caucacagucugaguucuuAcu......  8      1   OV1
..............................................................caucacagucugaguucuugUu......  2      1   OV1
..............................................................caGcacagucugaguucuugcu......  1      1   OV1
..............................................................caucaUagucugaguucuugcu......  4      1   OV1
..............................................................caucacaguAugaguucuugcu......  1      1   OV1
..............................................................caucacagAcugaguucuugcu......  2      1   OV1
..............................................................caucacaAucugaguucuugcu......  1      1   OV1
..............................................................cauUacagucugaguucuugcu......  8      1   OV1
..............................................................caucacagucugCguucuugcu......  1      1   OV1
..............................................................caucacagucugaAuucuugcu......  3      1   OV1
..............................................................caucacagucugaguuUuugcu......  9      1   OV1
..............................................................caucacagucugaguucuugAu......  2      1   OV1
..............................................................caucacagucugaUuucuugcu......  1      1   OV1
..............................................................caucacagucugaguucuCgcu......  1      1   OV1
..............................................................caucacagucugaguuGuugcu......  4      1   OV1
..............................................................caucacagucugaguucuugcu......  15631  0   OV1
..............................................................caucacagucugaguuAuugcu......  7      1   OV1
..............................................................caucacaguGugaguucuugcu......  2      1   OV1
..............................................................caucacagucugagGucuugcu......  5      1   OV1
..............................................................Uaucacagucugaguucuugcu......  2      1   OV1
..............................................................caAcacagucugaguucuugcu......  1      1   OV1
..............................................................caucacaCucugaguucuugcu......  1      1   OV1
..............................................................caucacagucugUguucuugcu......  1      1   OV1
..............................................................caucacagucuAaguucuugcu......  21     1   OV1
..............................................................caucacagucugagCucuugcu......  7      1   OV1
..............................................................caucacagucugGguucuugcu......  1      1   OV1
..............................................................cauAacagucugaguucuugcu......  3      1   OV1
..............................................................cUucacagucugaguucuugcu......  1      1   OV1
..............................................................caucacagucugaguGcuugcu......  1      1   OV1
..............................................................caucacagucugaguAcuugcu......  1      1   OV1
```

aga-miR-11

```
gcauccggcaagaacuuggaacugugaucugugugguuauuagccgcguucgaagcucuucacaucacagucugaguucuugcucgaugg
..............................................................caucGcagucugaguucuugcu......    2    1    OV1
..............................................................caucacaguUugaguucuugcu......    7    1    OV1
..............................................................caucacagucugaguucuugcC......    49   1    OV1
..............................................................caCcacagucugaguucuugcu......    6    1    OV1
..............................................................caucacCgucugaguucuugcu......    1    1    OV1
..............................................................caucacagucuUaguucuugcu......    7    1    OV1
..............................................................caucacagGcugaguucuugcu......    1    1    OV1
..............................................................cGucacagucugaguucuugcu......    3    1    OV1
..............................................................caucacagucugaguucuugcG......    26   1    OV1
..............................................................caucacGgucugaguucuugcu......    3    1    OV1
..............................................................caucacagucuCaguucuugcu......    2    1    OV1
..............................................................caucacagucugaguCcuugcu......    3    1    OV1
..............................................................caucacagucCgaguucuugcu......    11   1    OV1
..............................................................caucacagucugaguucCugcu......    5    1    OV1
..............................................................caucacagucugaguucuuCcu......    1    1    OV1
..............................................................caucacagCcugaguucuugcu......    3    1    OV1
..............................................................caucacUgucugaguucuugcu......    2    1    OV1
..............................................................caucacagucugaguucuugcA......    47   1    OV1
..............................................................caucacagucugaguucuugcuc.....    30   0    OV1
..............................................................caucacagucugaguucuugcuU.....    470  1    OV1
..............................................................caucacagCcugaguucuugcuc.....    1    1    OV1
..............................................................caucacagucugaguucuugcuG.....    18   1    OV1
..............................................................caucacagucugaguucuugcuA.....    230  1    OV1
..............................................................caucacagucugaguucuugcucU....    1    1    OV1
...............................................................aucacagucugaguucuugc.......    9    0    OV1
...............................................................aucacagucugaguucuugcu......    27   0    OV1
................................................................ucacagucugaguucuugc.......    1    0    OV1
................................................................ucacagucugaguucuugcu......    2    0    OV1
.................................................................cacagucugaguucuugcu......    1    0    OV1
..................................................................acagucugaguucuugc.......    2    0    OV1
..................................................................acagucugaguucuugcu......    1    0    OV1

........caagaacuuggaacugugauc.............................................................    4    0    FF1
........caagaacuuggaacugugaucu............................................................    6    0    FF1
........caagaacuuggaacugugaucug...........................................................    7    0    FF1
........caagaacuuggaacugugaucugu..........................................................    39   0    FF1
........caagaacuuggaacugugaucuguU.........................................................    1    1    FF1
........caagaacuuggaacuguAaucugug.........................................................    1    1    FF1
........caagaacuuggaacugugaucugug.........................................................    215  0    FF1
........caagaacuuggUacugugaucugug.........................................................    1    1    FF1
........caagaacuuggaacugugaucuguA.........................................................    2    1    FF1
........caagaacuuggaacugugaucugugA........................................................    20   1    FF1
........caagaacuuggaacugugaucugugG........................................................    3    1    FF1
........caagaacuuggaacugugaucuguCu........................................................    1    1    FF1
........caagaacuuggaacugugaucugugu........................................................    2    0    FF1
........caagaacuuggaacugugaucugugugg......................................................    1    0    FF1
........caagaacuuggaacugugaucuguguggu.....................................................    1    0    FF1
.........aagaacuuggaacugugaucugug.........................................................    2    0    FF1
..........agaacuuggaacugugaucugu..........................................................    1    0    FF1
..........agaacuuggaacugugaucugug.........................................................    1    0    FF1
..........agaacuuggaacugugaucugugA........................................................    1    1    FF1
.............................................................Ccaucacagucugaguucuugcu......    3    1    FF1
..............................................................caucacagucugaguucu..........    2    0    FF1
..............................................................caucacaguAugaguucuug........    1    1    FF1
..............................................................caucacagucugaguucuug........    26   0    FF1
..............................................................caucacagucugaguucuuA........    2    1    FF1
..............................................................caucacagucuAaguucuugc.......    2    1    FF1
..............................................................caucacagucuCaguucuugc.......    1    1    FF1
..............................................................caucacagucugaguucuCgc.......    3    1    FF1
..............................................................caCcacagucugaguucuugc.......    1    1    FF1
..............................................................caucacagucugaguucuuAc.......    1    1    FF1
..............................................................caucacagucugaguucuugc.......    765  0    FF1
..............................................................caucacagAcugaguucuugc.......    1    1    FF1
..............................................................caucacagucugaguucuugA.......    1    1    FF1
..............................................................caucacGgucugaguucuugc.......    1    1    FF1
..............................................................caucacagucugaguucuAgc.......    1    1    FF1
..............................................................caucacagucugagCucuugcu......    4    1    FF1
..............................................................caucacagucugaguucuuAcu......    3    1    FF1
..............................................................caucGcagucugaguucuugcu......    1    1    FF1
..............................................................caucacagucugaguucuGgcu......    1    1    FF1
```

aga-miR-11

```
gcauccggcaagaacuuggaacugugaucugugugguuauuagccgcguucgaagcucuucacaucacagucugaguucuugcucgaugg
..............................................................caucacagucuAaguucuugcu......   6     1   FF1
..............................................................caucacCgucugaguucuugcu......   1     1   FF1
..............................................................caucacaAucugaguucuugcu......   2     1   FF1
..............................................................caucaUagucugaguucuugcu......   3     1   FF1
..............................................................caucacagucugaguucuugAu......   1     1   FF1
..............................................................cauUacagucugaguucuugcu......   7     1   FF1
..............................................................caucacagucugaguucuugcC......   29    1   FF1
..............................................................caucacagucugaguucCugcu......   1     1   FF1
..............................................................caucacagucAgaguucuugcu......   1     1   FF1
..............................................................caucacagucugaguuUuugcu......   3     1   FF1
..............................................................caucacagucGgaguucuugcu......   2     1   FF1
..............................................................caucacagucugaguucuCgcu......   1     1   FF1
..............................................................Uaucacagucugaguucuugcu......   1     1   FF1
..............................................................caucacagCcugaguucuugcu......   1     1   FF1
..............................................................Gaucacagucugaguucuugcu......   1     1   FF1
..............................................................caucacagucuCaguucuugcu......   2     1   FF1
..............................................................caucacagucugaguucuugcu......   7282  0   FF1
..............................................................caucaAagucugaguucuugcu......   2     1   FF1
..............................................................caucacagucugaguucuugcG......   11    1   FF1
..............................................................caucacGgucugaguucuugcu......   2     1   FF1
..............................................................caucacaguUugaguucuugcu......   2     1   FF1
..............................................................caucacagucugGguucuugcu......   2     1   FF1
..............................................................caucacagucugaguucuugcA......   14    1   FF1
..............................................................cGucacagucugaguucuugcu......   2     1   FF1
..............................................................caCcacagucugaguucuugcu......   3     1   FF1
..............................................................caucacagucCgaguucuugcu......   8     1   FF1
..............................................................caucacagucuUaguucuugcu......   6     1   FF1
..............................................................caucacagucugaguucuugcuc.....   10    0   FF1
..............................................................caucacagucugaguucuugcuG.....   6     1   FF1
..............................................................caucacagucugaguucuugcuA.....   103   1   FF1
..............................................................caucacagucugaguucuugcuU.....   224   1   FF1
...............................................................aucacagucugaguucuugcu......   11    0   FF1
................................................................ucacagucugaguucuugcu......   2     0   FF1
.................................................................cacagucugaguucuugcu......   1     0   FF1

........caagaacuuggaacugugauc.............................................................   2     0   MF1
........caagaacuuggaacugugaucu............................................................   4     0   MF1
........caagaacuuggaacugugaucug...........................................................   3     0   MF1
........caagaacuuggCacugugaucugu..........................................................   1     1   MF1
........caagaacuuggaacugugaucugu..........................................................   17    0   MF1
........caagaacuuggaacugAgaucugug.........................................................   1     1   MF1
........caagaacuuggaacugugaucugug.........................................................   84    0   MF1
........caagaacuugAaacugugaucugug.........................................................   1     1   MF1
........caagaacuuggaacugugaucugugA........................................................   4     1   MF1
........caagaacuuggaacugugaucugugG........................................................   1     1   MF1
..............................................................caucacagucugaguucu..........   5     0   MF1
..............................................................caucacagucugaguucuu.........   4     0   MF1
..............................................................caucacagucugaUuucuug........   1     1   MF1
..............................................................Gaucacagucugaguucuug........   1     1   MF1
..............................................................caucacagucugaguucuug........   62    0   MF1
..............................................................caucacagucugaguuAuugc.......   1     1   MF1
..............................................................caucacagucugaguucuugc.......   741   0   MF1
..............................................................caucacagucuAaguucuugc.......   2     1   MF1
..............................................................caucacagucuUaguucuugc.......   1     1   MF1
..............................................................cauUacagucugaguucuugc.......   1     1   MF1
..............................................................caucaUagucugaguucuugc.......   1     1   MF1
..............................................................caucacagucugaguucuugA.......   4     1   MF1
..............................................................caCcacagucugaguucuugc.......   1     1   MF1
..............................................................cauGacagucugaguucuugc.......   1     1   MF1
..............................................................caAcacagucugaguucuugc.......   2     1   MF1
..............................................................cauUacagucugaguucuugcu......   4     1   MF1
..............................................................caucacagucugagCucuugcu......   1     1   MF1
..............................................................caucacagucugaguucuCgcu......   5     1   MF1
..............................................................caucacagucugaguucuugcG......   12    1   MF1
..............................................................caucacagGcugaguucuugcu......   2     1   MF1
..............................................................caucaGagucugaguucuugcu......   1     1   MF1
..............................................................caucacagucugaguCcuugcu......   1     1   MF1
..............................................................caucacagucugaguuUuugcu......   2     1   MF1
..............................................................caucacagAcugaguucuugcu......   1     1   MF1
..............................................................caucacagucugaguuGuugcu......   2     1   MF1
```

aga-miR-11

```
gcauccggcaagaacuuggaacugugaucugugugguuauuagccgcguucgaagcucuucacaucacagucugaguucuugcucgaugg
..............................................................caucacagucugaguucuuUcu......    1       1       MF1
..............................................................caucacagucugaguucuugcC......    13      1       MF1
..............................................................caucacagucugaguucuugcu......    3939    0       MF1
..............................................................caAcacagucugaguucuugcu......    1       1       MF1
..............................................................caucacagucugaguucuugAu......    1       1       MF1
..............................................................caucacagucugaguucuugcA......    4       1       MF1
..............................................................caucacaUucugaguucuugcu......    2       1       MF1
..............................................................caucaUagucugaguucuugcu......    2       1       MF1
..............................................................caucacagucugGguucuugcu......    1       1       MF1
..............................................................caucacagCcugaguucuugcu......    1       1       MF1
..............................................................caucacagucuUaguucuugcu......    8       1       MF1
..............................................................caucacagucCgaguucuugcu......    2       1       MF1
..............................................................caucacaguAugaguucuugcu......    1       1       MF1
..............................................................cGucacagucugaguucuugcu......    1       1       MF1
..............................................................caucacagucugaguuAuugcu......    1       1       MF1
..............................................................caucacaguUugaguucuugcu......    2       1       MF1
..............................................................Uaucacagucugaguucuugcu......    1       1       MF1
..............................................................caCcacagucugaguucuugcu......    2       1       MF1
..............................................................caucacagucuAaguucuugcu......    5       1       MF1
..............................................................caucacagucuCaguucuugcu......    3       1       MF1
..............................................................caucacagucugaguucuuAcu......    4       1       MF1
..............................................................caucacagucugaAuucuugcu......    2       1       MF1
..............................................................caucacagucugaguucuugcuc.....    6       0       MF1
..............................................................caucacagucugaguucuugcuA.....    8       1       MF1
..............................................................caucacagucugaguucuugcuG.....    2       1       MF1
..............................................................caucacagucugaguucuugcuU.....    93      1       MF1
...............................................................aucacagucugaguucuugcu......    8       0       MF1
...............................................................aucacagucugaguCcuugcu......    1       1       MF1
..................................................................acagucugaguucuugcu......    1       0       MF1

........caagaacuuggaacugugauc.............................................................    2       0       BF2
........caagaacuuggaacugugaucu............................................................    11      0       BF2
........caagaacuuggaacugugaucug...........................................................    4       0       BF2
........caagaacuuggaacugugaucugu..........................................................    90      0       BF2
........caagaacuuggaacugGgaucugu..........................................................    1       1       BF2
........caagaacuuggaaUugugaucugug.........................................................    1       1       BF2
........caagaacuuggaacugugaucuguA.........................................................    4       1       BF2
........caagaacuuggaacugugaucugug.........................................................    181     0       BF2
........caagaacuuggaacugugaucugugA........................................................    19      1       BF2
........caagaacuuggaacugugaucugugG........................................................    2       1       BF2
........caagaacuuggaacugugaucugugu........................................................    1       0       BF2
.........aagaacuuggaacugugaucuguguggu.....................................................    1       0       BF2
..........agaacuuggaacugugaucugu..........................................................    2       0       BF2
..........agaacuuggaacugugaucugug.........................................................    2       0       BF2
......................................auuagccgcguucgaagcucuucacaucacagucugaguucuug........    1       0       BF2
...........................................................ucacaucacagucugaguucuugc.......    1       0       BF2
.............................................................acaucacagucugaguucuugcu......    1       0       BF2
.............................................................Ccaucacagucugaguucuugcu......    1       1       BF2
..............................................................caucacagucugaguucu..........    4       0       BF2
..............................................................caucacagucugaguucuug........    122     0       BF2
..............................................................caucacagCcugaguucuug........    1       1       BF2
..............................................................caucacagAcugaguucuug........    1       1       BF2
..............................................................caucacagucuAaguucuug........    1       1       BF2
..............................................................caucacagucugaguucuuAc.......    1       1       BF2
..............................................................Uaucacagucugaguucuugc.......    1       1       BF2
..............................................................caucGcagucugaguucuugc.......    1       1       BF2
..............................................................Gaucacagucugaguucuugc.......    1       1       BF2
..............................................................caucacagucuAaguucuugc.......    1       1       BF2
..............................................................caucacagucugaguucAugc.......    1       1       BF2
..............................................................caucacagucugaguucuugc.......    1285    0       BF2
..............................................................cauUacagucugaguucuugc.......    2       1       BF2
..............................................................caucacagucugagCucuugc.......    3       1       BF2
..............................................................caucacagucugaguucCugc.......    1       1       BF2
..............................................................caucacagucugaguucuugU.......    1       1       BF2
..............................................................caucacagucugaAuucuugc.......    1       1       BF2
..............................................................caucacagucugaguucuugGu......    1       1       BF2
..............................................................caucacagucugagGucuugcu......    1       1       BF2
..............................................................caucUcagucugaguucuugcu......    1       1       BF2
..............................................................caucacagucugaguucuugcu......    6243    0       BF2
..............................................................caucacagucugaUuucuugcu......    1       1       BF2
```

aga-miR-11

```
gcauccggcaagaacuuggaacugugaucugugugguuauuagccgcguucgaagcucuucacaucacagucugaguucuugcucgaugg
..............................................................caucacagCcugaguucuugcu......    3     1     BF2
..............................................................caucacagucugaguucuuAcu......    1     1     BF2
..............................................................caucacagucugaguucuugUu......    1     1     BF2
..............................................................caucacagucugaguucuuUcu......    2     1     BF2
..............................................................caucacagucGgaguucuugcu......    1     1     BF2
..............................................................caucacagucugaguucuugcC......    48    1     BF2
..............................................................caucacagucugUguucuugcu......    1     1     BF2
..............................................................caucacagucugaguCcuugcu......    4     1     BF2
..............................................................caucacagucugaguuAuugcu......    1     1     BF2
..............................................................caucacagucAgaguucuugcu......    2     1     BF2
..............................................................caucGcagucugaguucuugcu......    1     1     BF2
..............................................................caucacagucuAaguucuugcu......    7     1     BF2
..............................................................caucacaguUugaguucuugcu......    1     1     BF2
..............................................................Uaucacagucugaguucuugcu......    5     1     BF2
..............................................................caucacagucugagCucuugcu......    4     1     BF2
..............................................................caucacagucugaguucuugcA......    18    1     BF2
..............................................................caucacagucuUaguucuugcu......    6     1     BF2
..............................................................caAcacagucugaguucuugcu......    1     1     BF2
..............................................................Gaucacagucugaguucuugcu......    1     1     BF2
..............................................................caucacagucCgaguucuugcu......    3     1     BF2
..............................................................caucacagucugaguucuugcG......    17    1     BF2
..............................................................cauUacagucugaguucuugcu......    2     1     BF2
..............................................................Aaucacagucugaguucuugcu......    1     1     BF2
..............................................................caucaUagucugaguucuugcu......    1     1     BF2
..............................................................caucacagucugaguuUuugcu......    1     1     BF2
..............................................................caCcacagucugaguucuugcu......    3     1     BF2
..............................................................caucacagucugaguucuugcuG.....    10    1     BF2
..............................................................caucacagucugaguucuugcuc.....    34    0     BF2
..............................................................caucacagucugaguucuugcuA.....    127   1     BF2
..............................................................caucacagucugaguucuugcuU.....    453   1     BF2
..............................................................caucacagucugaguucuugcuUg....    1     1     BF2
...............................................................aucacagucugaguucuugc.......    1     0     BF2
...............................................................aucacagucugaguucuugcu......    8     0     BF2
...............................................................aucacagucugaguucuugcuU.....    3     1     BF2
................................................................ucacagucugaguucuugcu......    2     0     BF2
..................................................................acagucugaguucuugcu......    1     0     BF2
..................................................................acagucugaguucuugcuA.....    1     1     BF2

........caagaacuuggaacugugauc.............................................................    4     0     BF1
........caagaacuuggaacugugaucu............................................................    6     0     BF1
........caagaacuuggaacugugaucug...........................................................    6     0     BF1
........caagaacuuggaacugugaucugu..........................................................    40    0     BF1
........caagaacuuggUacugugaucugug.........................................................    1     1     BF1
........caagaacuuggaacugugaucugug.........................................................    215   0     BF1
........caagaacuuggaacugugaucuguA.........................................................    2     1     BF1
........caagaacuuggaacuguAaucugug.........................................................    1     1     BF1
........caagaacuuggaacugugaucuguU.........................................................    1     1     BF1
........caagaacuuggaacugugaucugugG........................................................    3     1     BF1
........caagaacuuggaacugugaucuguCu........................................................    1     1     BF1
........caagaacuuggaacugugaucugugA........................................................    20    1     BF1
........caagaacuuggaacugugaucugugu........................................................    2     0     BF1
........caagaacuuggaacugugaucugugugg......................................................    1     0     BF1
........caagaacuuggaacugugaucuguguggu.....................................................    1     0     BF1
.........aagaacuuggaacugugaucugug.........................................................    2     0     BF1
..........agaacuuggaacugugaucugu..........................................................    1     0     BF1
..........agaacuuggaacugugaucugug.........................................................    1     0     BF1
..........agaacuuggaacugugaucugugA........................................................    1     1     BF1
.............................................................Ccaucacagucugaguucuugcu......    3     1     BF1
..............................................................caucacagucugaguucu..........    2     0     BF1
..............................................................caucacagucugaguucuug........    23    0     BF1
..............................................................caucacagucugaguucuuA........    2     1     BF1
..............................................................caucacaguAugaguucuug........    1     1     BF1
..............................................................caucacagucugaguucuAgc.......    1     1     BF1
..............................................................caucacagAcugaguucuugc.......    1     1     BF1
..............................................................caCcacagucugaguucuugc.......    1     1     BF1
..............................................................caucacagucugaguucuugc.......    758   0     BF1
..............................................................caucacagucugaguucuugA.......    1     1     BF1
..............................................................caucacagucugaguucuuAc.......    1     1     BF1
..............................................................caucacagucugaguucuCgc.......    3     1     BF1
..............................................................caucacGgucugaguucuugc.......    1     1     BF1
```

```
gcauccggcaagaacuuggaacugugaucugugugguuauuagccgcguucgaagcucuucacaucacagucugaguucuugcucgaugg
..............................................................caucacagucuCaguucuugc.......    1      1      BF1
..............................................................caucacagucuAaguucuugc.......    2      1      BF1
..............................................................caucacaguUugaguucuugcu......    2      1      BF1
..............................................................cGucacagucugaguucuugcu......    2      1      BF1
..............................................................caucacagucuAaguucuugcu......    6      1      BF1
..............................................................caucacagucugaguucuugcC......    29     1      BF1
..............................................................caucacagucugaguucuCgcu......    1      1      BF1
..............................................................caucacagucugaguucuugAu......    1      1      BF1
..............................................................caucacagucugaguucuGgcu......    1      1      BF1
..............................................................caucacagucAgaguucuugcu......    1      1      BF1
..............................................................caucacagucGgaguucuugcu......    2      1      BF1
..............................................................caucacagucugaguucuugcG......    11     1      BF1
..............................................................caucaAagucugaguucuugcu......    2      1      BF1
..............................................................caucacaAucugaguucuugcu......    2      1      BF1
..............................................................caucacagucugaguuUuugcu......    3      1      BF1
..............................................................caucacagucugaguucuugcu......    7273   0      BF1
..............................................................caucacagucugaguucuuAcu......    3      1      BF1
..............................................................cauUacagucugaguucuugcu......    7      1      BF1
..............................................................caucacGgucugaguucuugcu......    2      1      BF1
..............................................................caucacagucCgaguucuugcu......    8      1      BF1
..............................................................Uaucacagucugaguucuugcu......    1      1      BF1
..............................................................caucacCgucugaguucuugcu......    1      1      BF1
..............................................................caucacagucuCaguucuugcu......    2      1      BF1
..............................................................caucacagucuUaguucuugcu......    6      1      BF1
..............................................................caucacagucugagCucuugcu......    4      1      BF1
..............................................................caucacagCcugaguucuugcu......    1      1      BF1
..............................................................Gaucacagucugaguucuugcu......    1      1      BF1
..............................................................caucacagucugaguucuugcA......    14     1      BF1
..............................................................caucacagucugGguucuugcu......    2      1      BF1
..............................................................caCcacagucugaguucuugcu......    3      1      BF1
..............................................................caucaUagucugaguucuugcu......    3      1      BF1
..............................................................caucacagucugaguucCugcu......    1      1      BF1
..............................................................caucGcagucugaguucuugcu......    1      1      BF1
..............................................................caucacagucugaguucuugcuG.....    6      1      BF1
..............................................................caucacagucugaguucuugcuA.....    103    1      BF1
..............................................................caucacagucugaguucuugcuU.....    223    1      BF1
..............................................................caucacagucugaguucuugcuc.....    10     0      BF1
...............................................................aucacagucugaguucuugcu......    11     0      BF1
..................................................................ucagucugaguucuugcu......    2      0      BF1
...................................................................cagucugaguucuugcu......    1      0      BF1

........caagaacuuggaacugugaucu............................................................    7      0      FW1
........caagaacuuggaacugugaucug...........................................................    2      0      FW1
........caagaacuuggaacugugaucugu..........................................................    24     0      FW1
........caagaacuuggaacCgugaucugu..........................................................    1      1      FW1
........caagaacuuggaacugugaucugug.........................................................    36     0      FW1
........caagaacuuggaacugugaucugugA........................................................    3      1      FW1
.................................ugguuauuagccgcguucgaagcu.................................    1      0      FW1
..............................................................caucacagucugaguucu..........    1      0      FW1
..............................................................caucacagucugaguucuu.........    1      0      FW1
..............................................................caucacagucugaguCcuug........    1      1      FW1
..............................................................caucacagucugaguucuug........    15     0      FW1
..............................................................caucacagucugaguuAuugc.......    1      1      FW1
..............................................................caucacagucugaguucuugA.......    1      1      FW1
..............................................................caucacagucugaguucuugc.......    192    0      FW1
..............................................................Uaucacagucugaguucuugcu......    1      1      FW1
..............................................................caucacagucugaguucuugcC......    5      1      FW1
..............................................................caucacagucAgaguucuugcu......    1      1      FW1
..............................................................caucacagucugaguucuugcu......    1331   0      FW1
..............................................................caCcacagucugaguucuugcu......    3      1      FW1
..............................................................caucacagucugaguucuugcA......    3      1      FW1
..............................................................caucacagucugaguucuugAu......    1      1      FW1
..............................................................cauAacagucugaguucuugcu......    1      1      FW1
..............................................................caucacagucCgaguucuugcu......    1      1      FW1
..............................................................caucacagucugaguucuugcG......    2      1      FW1
..............................................................caucacagucugagCucuugcu......    1      1      FW1
..............................................................caucaUagucugaguucuugcu......    1      1      FW1
..............................................................caucacagucugaguucuugcuA.....    5      1      FW1
..............................................................caucacagucugaguucuugcuc.....    2      0      FW1
..............................................................caucacagucugaguucuugcuG.....    4      1      FW1
```

aga-miR-11

gcauccggcaagaacuuggaacugugaucugugugguuauuagccgcguucgaagcucuucacaucacagucugaguucuugcucgaugg			
....caucacagucugaguucuugcuU.....	25	1	FW1
....cacagucugaguucuugc.......	1	0	FW1
....cacagucugaguucuugcu......	4	0	FW1
....acagucugaguucuugcu......	1	0	FW1
........caagaacuuggaacugugau..	1	0	MW1
........caagaacuuggaacugugauc...	3	0	MW1
........caagaacuuggaacugugaucu..	6	0	MW1
........caagaacuuggaacugugaucug...	7	0	MW1
........caagaacuCggaacugugaucugu..	1	1	MW1
........caagaacuuggaacugugaucugu..	45	0	MW1
........caagaacuuggaacugugaucugug..	66	0	MW1
........caagGacuuggaacugugaucugug..	1	1	MW1
........caagaacuuggaacugCgaucugug..	1	1	MW1
........caagaacuuggaacugugaucuguA...	2	1	MW1
........caagaacuuggaacugugaucugugu...	1	0	MW1
........caagaacuuggaacugugaucugugA...	2	1	MW1
..........agaacuuggaacugugaucugug...	1	0	MW1
....caucacagucugaguucu..........	4	0	MW1
....caucacagucugaguucuu.........	3	0	MW1
....caucacagucugaguucuug........	31	0	MW1
....caucacagucugaguucuugc.......	373	0	MW1
....caucacagucugaguucuugA.......	1	1	MW1
....caucacagucugaCuucuugc.......	1	1	MW1
....caucacagAcugaguucuugc.......	1	1	MW1
....caucacagucuCaguucuugc.......	1	1	MW1
....caucacagucugGguucuugcu......	1	1	MW1
....caucacagucugaguuGuugcu......	1	1	MW1
....caucacagucugaguucuugcA......	1	1	MW1
....caAcacagucugaguucuugcu......	1	1	MW1
....caucacagucuAaguucuugcu......	3	1	MW1
....caucacagucugaguucuugcC......	3	1	MW1
....caucacagucugaguucuugcu......	2262	0	MW1
....caucacagucugaguucuugcG......	5	1	MW1
....caucacagucugagCucuugcu......	2	1	MW1
....caucacagucugaguucCugcu......	3	1	MW1
....caucacagucugaguucuuAcu......	1	1	MW1
....caucacagAcugaguucuugcu......	1	1	MW1
....caucacagucugUguucuugcu......	1	1	MW1
....caucacagucuCaguucuugcu......	1	1	MW1
....caCcacagucugaguucuugcu......	2	1	MW1
....caucacagucCgaguucuugcu......	2	1	MW1
....caucacaguUugaguucuugcu......	1	1	MW1
....caucacagucugaguucuugGu......	1	1	MW1
....caucacagucugaguucuAgcu......	1	1	MW1
....Uaucacagucugaguucuugcu......	3	1	MW1
....caucacagucugagGucuugcu......	1	1	MW1
....caucacagucugaguucuugcuc.....	3	0	MW1
....caucacagucugaguucuugcuU.....	69	1	MW1
....caucacagucugaguucuugcuA.....	5	1	MW1
....caucacagucugaguucuugcuG.....	2	1	MW1
....aucacagucugaguucuugc.......	2	0	MW1
....aucacagucugaguucuugcu......	4	0	MW1
....ucacagucugaguucuugcu......	2	0	MW1
....cacagucugaguucuugc.......	1	0	MW1
....cacagucugaguucuugcC......	1	1	MW1
....cacagucugaguucuugcu......	5	0	MW1
....cagucugaguucuugcu......	2	0	MW1
........caagaacuuggaacugugaucu..	8	0	MW2
........caagaacuuggaacugugaucug...	1	0	MW2
........caagaacuuggaacugCgaucugu..	1	1	MW2
........caagaacuuggaacugugaucugG..	1	1	MW2
........caagaacuuggaacugugaucugu..	25	0	MW2
........caagaacuuggaacugugaucugug..	23	0	MW2
........caagaacuuggaacugugaucuguA...	1	1	MW2
........caagaacuuggaacugugaucugugA...	1	1	MW2
....caucacagucugaguucu..........	1	0	MW2
....caucacagucugaguucuu.........	1	0	MW2
....caucacagucugaguucuug........	6	0	MW2

aga-miR-11

gcauccggcaagaacuuggaacugugaucugugugguuauuagccgcguucgaagcucuucacaucacagucugaguucuugcucgaugg			
....caucacagucugaguucuugc.......	122	0	MW2
....caucacagucugaguucuuAc.......	1	1	MW2
....caucacagucuAaguucuugcu......	3	1	MW2
....caucacagucugaguucuugcA......	1	1	MW2
....caucacagucugaguucuugcu......	750	0	MW2
....caucacagucugaguucuugcC......	1	1	MW2
....cauUacagucugaguucuugcu......	2	1	MW2
....caucacagucCgaguucuugcu......	2	1	MW2
....caucacagucugaguucuugcG......	1	1	MW2
....caucacagucugaguuAuugcu......	1	1	MW2
....caucacagucugaguucuugcuA.....	1	1	MW2
....caucacagucugaguucuugcuG.....	1	1	MW2
....caucacagucugaguucuugcuU.....	18	1	MW2
....caucacagucugaguucuugcuc.....	2	0	MW2
....ucacagucugaguucuugcu......	1	0	MW2
....cacagucugaguucuugcu......	1	0	MW2
.....cggcaagaacuuggaacuguga...	1	0	TE2
........caagaacuuggaacugug..	2	0	TE2
........caagaacuuggaacugugau..	2	0	TE2
........caagaacuuggaacugugauc...	4	0	TE2
........caagaacuuggaacugugaucu..	13	0	TE2
........caagaacuuggaacugugaucug...	3	0	TE2
........caagaacuuggGacugugaucug...	1	1	TE2
........caagaacuCggaacugugaucugu..	1	1	TE2
........caagaacuuggaacugugaucugu..	32	0	TE2
........caagaacuuggaacugugaucuguA...	1	1	TE2
........caagaacuuggaacugugaucugGg...	1	1	TE2
........caagaacuuggaacugugaCcugug...	1	1	TE2
........caagaacuuggGacugugaucugug...	1	1	TE2
........caagaacuuggaacugugaucuguU...	1	1	TE2
........caagaacuuggaacugugaucugug...	127	0	TE2
........caagaacuuggaacugugaucugugu..	5	0	TE2
........caagaacuuggaacugugaucugugA..	8	1	TE2
..........agaacuuggaacugugaucugu..	1	0	TE2
....Ccaucacagucugaguucuugcu......	3	1	TE2
....caucacagucugaguuc...........	1	0	TE2
....caucacagucugaguucu..........	21	0	TE2
....caucacagucugagCucu..........	1	1	TE2
....caucacagucugaguucuu.........	19	0	TE2
....caucacagucugaguucuuA........	1	1	TE2
....caucacagucugUguucuug........	1	1	TE2
....caucacagucugaguucuCg........	1	1	TE2
....caucacagucugaguucuug........	442	0	TE2
....cauUacagucugaguucuug........	1	1	TE2
....caucacagucugaguucCug........	1	1	TE2
....caucacaguUugaguucuug........	2	1	TE2
....caCcacagucugaguucuugc.......	2	1	TE2
....caucacagucugagGucuugc.......	1	1	TE2
....caucacagucugCguucuugc.......	1	1	TE2
....caucacagucugaguuUuugc.......	1	1	TE2
....Uaucacagucugaguucuugc.......	1	1	TE2
....caucacagucuCaguucuugc.......	2	1	TE2
....caucacagAcugaguucuugc.......	1	1	TE2
....caucacagucCgaguucuugc.......	1	1	TE2
....caucacagGcugaguucuugc.......	1	1	TE2
....caucacagucugaguCcuugc.......	2	1	TE2
....caucaGagucugaguucuugc.......	1	1	TE2
....caucacagucugaguucuugc.......	4577	0	TE2
....caucacagucugagCucuugc.......	1	1	TE2
....caucacagucugaguucCugc.......	1	1	TE2
....caucacagucugaguucuuUc.......	1	1	TE2
....caucacagucugaguucuCgc.......	1	1	TE2
....caucacagucugaguuGuugc.......	3	1	TE2
....caucacagucugaguucuugA.......	1	1	TE2
....caucacagucugaguucuuAc.......	5	1	TE2
....caucacagucugaAuucuugc.......	1	1	TE2
....caucacagucuUaguucuugc.......	1	1	TE2
....caucacaCucugaguucuugc.......	1	1	TE2
....cGucacagucugaguucuugc.......	3	1	TE2

aga-miR-11

```
gcauccggcaagaacuuggaacugugaucugugugguuauuagccgcguucgaagcucuucacaucacagucugaguucuugcucgaugg
..............................................................caucacagucugaguucuugU.......    1      1      TE2
..............................................................caucacaAucugaguucuugc.......    1      1      TE2
..............................................................caucaAagucugaguucuugc.......    1      1      TE2
..............................................................caucacagucuAaguucuugc.......    4      1      TE2
..............................................................caucacagucugaguuAuugc.......    3      1      TE2
..............................................................caGcacagucugaguucuugc.......    1      1      TE2
..............................................................caucacUgucugaguucuugc.......    1      1      TE2
..............................................................caucacaguUugaguucuugc.......    1      1      TE2
..............................................................caucacaguGugaguucuugc.......    3      1      TE2
..............................................................cauUacagucugaguucuugc.......    3      1      TE2
..............................................................caucacagucugaguucuuAcu......    6      1      TE2
..............................................................caucacagucugaguCcuugcu......    3      1      TE2
..............................................................caucacagucugUguucuugcu......    2      1      TE2
..............................................................caucGcagucugaguucuugcu......    1      1      TE2
..............................................................Aaucacagucugaguucuugcu......    1      1      TE2
..............................................................caucacaUucugaguucuugcu......    1      1      TE2
..............................................................caucacagCcugaguucuugcu......    12     1      TE2
..............................................................caucacagucAgaguucuugcu......    1      1      TE2
..............................................................caucacagucugagGucuugcu......    4      1      TE2
..............................................................caucacaguAugaguucuugcu......    4      1      TE2
..............................................................caucacUgucugaguucuugcu......    2      1      TE2
..............................................................caucacagucugCguucuugcu......    1      1      TE2
..............................................................Gaucacagucugaguucuugcu......    1      1      TE2
..............................................................caucUcagucugaguucuugcu......    1      1      TE2
..............................................................caucacagucugaguuGuugcu......    6      1      TE2
..............................................................caucacagucugaguucuugcC......    33     1      TE2
..............................................................caucacagucugaguuAuugcu......    4      1      TE2
..............................................................caucacagucugaguucuugcA......    58     1      TE2
..............................................................caucacagucCgaguucuugcu......    8      1      TE2
..............................................................caucacagucuAaguucuugcu......    21     1      TE2
..............................................................caucacagucugaguuUuugcu......    4      1      TE2
..............................................................caucacagucugGguucuugcu......    1      1      TE2
..............................................................cauUacagucugaguucuugcu......    9      1      TE2
..............................................................caucacagucugaguucuuUcu......    4      1      TE2
..............................................................caucacagucugagAucuugcu......    2      1      TE2
..............................................................caucacagucugagCucuugcu......    6      1      TE2
..............................................................caucacagucugaguAcuugcu......    1      1      TE2
..............................................................cUucacagucugaguucuugcu......    2      1      TE2
..............................................................caucacagAcugaguucuugcu......    1      1      TE2
..............................................................caucacagucugaUuucuugcu......    1      1      TE2
..............................................................caucacagucugaguucuugcu......    15251  0      TE2
..............................................................caucacagucugaguucuCgcu......    4      1      TE2
..............................................................caucacagucugaAuucuugcu......    2      1      TE2
..............................................................caucacaCucugaguucuugcu......    1      1      TE2
..............................................................caucacagucGgaguucuugcu......    3      1      TE2
..............................................................caCcacagucugaguucuugcu......    6      1      TE2
..............................................................caucacGgucugaguucuugcu......    2      1      TE2
..............................................................caucacagucugaguucuAgcu......    3      1      TE2
..............................................................caucacagGcugaguucuugcu......    1      1      TE2
..............................................................Uaucacagucugaguucuugcu......    10     1      TE2
..............................................................caucacaAucugaguucuugcu......    1      1      TE2
..............................................................caucacagucugaguucCugcu......    1      1      TE2
..............................................................caucacagucugaguucuugUu......    7      1      TE2
..............................................................caucacagucuCaguucuugcu......    3      1      TE2
..............................................................caucacagucugaguucuugcG......    32     1      TE2
..............................................................cGucacagucugaguucuugcu......    1      1      TE2
..............................................................caucacaguUugaguucuugcu......    2      1      TE2
..............................................................caucacaguGugaguucuugcu......    4      1      TE2
..............................................................caucacagucugaguucuugAu......    1      1      TE2
..............................................................caucaUagucugaguucuugcu......    9      1      TE2
..............................................................caucacagucuUaguucuugcu......    1      1      TE2
..............................................................caAcacagucugaguucuugcu......    1      1      TE2
..............................................................caucacagucugaguucuugcuU.....    222    1      TE2
..............................................................caucacagucugaguucuugcuA.....    149    1      TE2
..............................................................caucacagucugaguucuugcuc.....    7      0      TE2
..............................................................caucacagucugaguucuugcuG.....    5      1      TE2
...............................................................aucacagucugaguucuug........    2      0      TE2
...............................................................aucacagucugaguucuugc.......    15     0      TE2
...............................................................aucacGgucugaguucuugc.......    1      1      TE2
...............................................................aucacagucugaguucuugcu......    17     0      TE2
```

aga-miR-11

```
gcauccggcaagaacuuggaacugugaucugugugguuauuagccgcguucgaagcucuucacaucacagucugaguucuugcucgaugg
...............................................................aucacagucugaguucuugcuA.....    1    1    TE2
................................................................ucacagucugaguucuug........    1    0    TE2
................................................................ucacagucugaguucuugc.......    3    0    TE2
................................................................ucacagucugaguucuugcu......    8    0    TE2
.................................................................cacagucugaguucuugcu......    1    0    TE2
..................................................................acagucugaguucuugcu......    2    0    TE2
```

miRBase precursor : aga-mir-124
Total read count : 115
aga-miR-124 read count : 79
aga-miR-124* read count : 34
remaining reads : 2

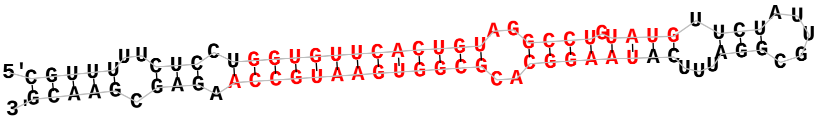

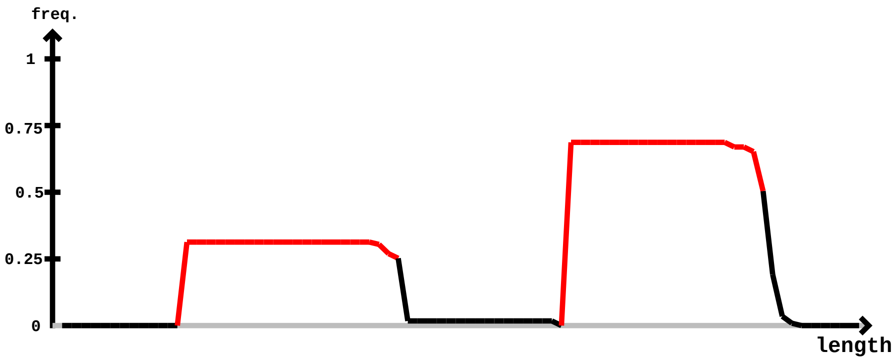

aga-miR-124*

aga-miR-124

5'- cguuuuucuccu**gguguucacuguaggccuguaug**uucuauugcggauuuca**uaaggcacgcggugaaugcca**agagcgaacg -3'	exp		
(((((..(((.(((((((((((((..((((.((((.(((.....)))...))))))))..))))))))))))).))).)))))	reads	mm	sample
....uaaggcacgcggugaaugcc...........	2	0	OV2
............gguguucacuguaggccuguauguucuauugcggauuuc................................	1	0	TE1
............gguguucacuguaggccuguaug..	3	0	FF2
............gguguucacuguaggccuguauguucuauugcggauuuc................................	1	0	FF2
....uaaggcacgcggugaaugccaaAa......	1	1	FF2
............gguguucacuguaggccugua...	1	0	MF2
............gguguucacuguaggccuguaug..	9	0	MF2
....uaaggcacgcggugaaugcc...........	3	0	MF2
....uaaggcacgcggugaaugcca..........	3	0	MF2
....uaaggcacgcgguAaaugcca..........	1	1	MF2
....uaaggcacgcggugaaugccaaA.......	1	1	MF2
............gguguucacuguaggccugua...	1	0	FW2
............gguguucacuguaggccuguaug..	7	0	FW2
....uaaggcacgcggugaaugcc...........	1	0	FW2
....uaaggcacgcggugaaugcca..........	9	0	FW2
....uaaggcacgcggugaaugccaa.........	5	0	FW2
....uaaggcacgcggugaaugccaaU.......	1	1	FW2
....uaaggcacgcggugaau..............	1	0	MF1
....uaaggcacgcggugaaugc............	2	0	MF1
....uaaggcacgcggugaaugcc...........	1	0	MF1
....uaaggcacgcggugaaugcca..........	1	0	MF1
....uaaggcacgcggugaaugccaa.........	1	0	MF1
............gguguucacuguaggccugua...	2	0	MW1
............gguguucacuguaggccuguaug..	4	0	MW1
....uaaggcacgcggugaaugcc...........	5	0	MW1
....uaaggcacgcggugaaugcca..........	12	0	MW1
....uaaggcacgcggugaaugccaa.........	6	0	MW1
....uaaggcacgcggugaaugccaaA.......	1	1	MW1

```
            aga-miR-124*
                                                    aga-miR-124
cguuuuucuccugguguucacuguaggccuguauguucuauugcggauuucauaaggcacgcggugaaugccaagagcgaacg

............gguguucacuguaggccuguau.................................................   1   0   FW1
............gguguucacuguaggccuguaug................................................   2   0   FW1
....................................................uaaggcacgcggugaaugcc...........   2   0   FW1
....................................................uaaggcacgcggugaaugcca..........   6   0   FW1
....................................................uaaggcacgcggugaaugccaa.........   2   0   FW1

............gguguucacuguaggccugu...................................................   1   0   MW2
............gguguucacuguaggccuguau.................................................   1   0   MW2
............gguguucacuguaggccuguaug................................................   2   0   MW2
....................................................uaaggcacgcggugaau..............   1   0   MW2
....................................................uaaggcacgcggugaaugcc...........   3   0   MW2
....................................................uaaggcacgcggugaaugcca..........   3   0   MW2
....................................................uaaggcacgcggugaaugccaa.........   3   0   MW2

....................................................uaaggcacgcggugaaugcca..........   1   0   TE2
....................................................uaaggcacgcggugaaugccaa.........   1   0   TE2
```

miRBase precursor	: aga-mir-125
Total read count	: 5044
aga-miR-125 read count	: 4707
aga-miR-125* read count	: 164
remaining reads	: 173

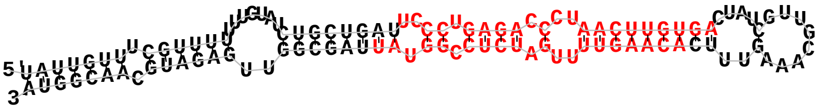

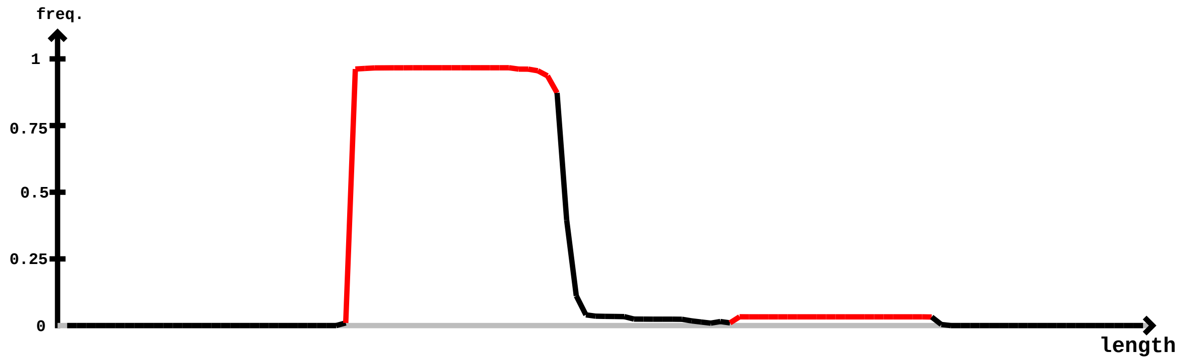


```
                                                                          aga-miR-125*
                                  aga-miR-125
5'- uauuguuucguuuuuuuguaucugcugauucccugagacccuaacuugugacuaucguugcaaaguuucacaaguuuugaucuccgguauuagcgguugagaugcaacggua   -3'    exp
    (((((((.((((((.......((((((((..((.((((.(..(((((((((....(........)..)))))))))..).)))).)).))))))))..)))))).)))))))  reads      mm          sample
    .............................ucccugagacccuaacuug................................................................  2          0           FF2
    .............................ucccugagacccuaacuugug..............................................................  4          0           FF2
    .............................Gcccugagacccuaacuugug..............................................................  1          1           FF2
    .............................ucccugagacccuaacuuguga.............................................................  33         0           FF2
    .............................ucccugagacccuaacuugugG.............................................................  4          1           FF2
    .............................ucccugagacccuaacuugugac............................................................  10         0           FF2
    .............................ucccugagacccuaacuugugaA............................................................  3          1           FF2
    .............................ucccugagacccuaacuugugacu...........................................................  2          0           FF2
    .............................ucccugagacccuaacuugugacuau.........................................................  1          0           FF2
    .............................ucccugagacccuaacuugugacuaucguugcaaa................................................  1          0           FF2
    .............................ucccugagacccuaacuugugacuaucguugcaaaguuu............................................  1          0           FF2
    .....................................................................acaaguuuugaucuccgguau......................  1          0           FF2

    ............................uucccugagacccuaacuugug..............................................................  2          0           TE1
    ............................uucccugagacccuaacuuguga.............................................................  1          0           TE1
    .............................ucccugagacccuaacu..................................................................  1          0           TE1
    .............................ucccugagacccuaacuug................................................................  1          0           TE1
    .............................ucccugagacccuaacuugA...............................................................  1          1           TE1
    .............................ucccugagacccuaacuugug..............................................................  11         0           TE1
    .............................ucccugagacccuaacuugugG.............................................................  11         1           TE1
    .............................ucccugagacccuaacuuguga.............................................................  130        0           TE1
    .............................uUccugagacccuaacuuguga.............................................................  1          1           TE1
    .............................ucccugagacccuaacuugugac............................................................  61         0           TE1
    .............................uGccugagacccuaacuugugac............................................................  1          1           TE1
    .............................ucccugagacccuaacuugugaA............................................................  11         1           TE1
    .............................ucccugagacccuaacuugugacA...........................................................  1          1           TE1
    .............................ucccugagacccuaacuugugacu...........................................................  11         0           TE1
    .............................ucccugagacccuaacuugugacuUu.........................................................  1          1           TE1
    .............................ucccugagacccuaacuugugacuaucgu......................................................  2          0           TE1
    .............................ucccugagacccuaacuugugacuaucguugcaaag...............................................  1          0           TE1
    .............................ucccugagacccuaacuugugacuaucguugcaaagu..............................................  1          0           TE1
    .............................ucccugagacccuaacuugugacuaucguCgcaaagu..............................................  1          1           TE1
    .............................ucccugagacccuaacuugugacuaucguugcaaaguA.............................................  1          1           TE1
    .............................ucccugagacccuaacuugugacuaucguugcaaaguuuA...........................................  1          1           TE1
```

```
                                                                   aga-miR-125*
                             aga-miR-125
uauuguuucguuuuuuuguaucugcugauucccugagacccuaacuugugacuaucguugcaaaguuucacaaguuuugaucuccgguauuagcgguugagaugcaacggua

...............................ccugagacccuaacuuguga.............................................................    1     0     TE1
...................................................................ucacaaguuuugaucuccgguau......................    5     0     TE1
.....................................................................acaaguuuugaucuccgguau......................    8     0     TE1

............................uucccugagacccuaacuugu...............................................................    1     0     OV2
.............................ucccugagacccuaacu..................................................................    2     0     OV2
.............................ucccugagacccuaacuu.................................................................    1     0     OV2
.............................ucccugagacccuaacuug................................................................    3     0     OV2
.............................ucccugagacccuaacuugu...............................................................    30    0     OV2
.............................ucccugagacccuaaUuugu...............................................................    1     1     OV2
.............................ucccugagacccuaacuugGg..............................................................    1     1     OV2
.............................ucccugagacccuaacuugug..............................................................    58    0     OV2
.............................ucccugagacUcuaacuuguga.............................................................    1     1     OV2
.............................ucccugagacccuaacuuguga.............................................................    266   0     OV2
.............................ucccugagacccuaacuugAga.............................................................    1     1     OV2
.............................ucccugagacccuaacuugugG.............................................................    29    1     OV2
.............................ucccuAagacccuaacuugugac............................................................    1     1     OV2
.............................ucccugagacccuaacuugugaA............................................................    91    1     OV2
.............................ucccugagacccuaacuugugac............................................................    72    0     OV2
.............................ucccugagacccuaacuugugaG............................................................    1     1     OV2
.............................ucccugagacccuaacuugugaU............................................................    1     1     OV2
.............................ucccugagacccuaacuugugacA...........................................................    1     1     OV2
.............................ucccugagacccuaacuugugacu...........................................................    28    0     OV2
.............................ucccugagacccuaacuugugaAu...........................................................    1     1     OV2
.............................ucccugagacccuaacuugugacua..........................................................    1     0     OV2
.............................ucccugagacccuaacuugugacuau.........................................................    1     0     OV2
.............................ucccugagacccuaacuugugacuaucgu......................................................    9     0     OV2
.............................ucccugagacccuaacuugugacuaucguugcaaa................................................    7     0     OV2
.............................ucccugagacccuaacuugugacuaucguugcaaaU...............................................    1     1     OV2
.............................ucccugagacccuaacuugugacuaucguugcaaag...............................................    3     0     OV2
.............................ucccugagacccuaacuugugacuaucguugcaaagu..............................................    3     0     OV2
.............................ucccugagacccuaacuugugacuaucguugcaaaguu.............................................    1     0     OV2
.............................ucccugagacccuaacuugugacuaucguugcaaaguuu............................................    2     0     OV2
..............................cccugagacccuaacuuUuga.............................................................    1     1     OV2
....................................................................cacaaguuuugaucuccgguau......................    1     0     OV2
.....................................................................acaaguuuugaucuccggua.......................    1     0     OV2
.....................................................................acaaguuuugaucuccgguau......................    2     0     OV2

............................uucccugagacccuaacuug................................................................    1     0     MF2
............................uucccugagacccuaacuuguga.............................................................    1     0     MF2
.............................ucccugagacccuaacu..................................................................    1     0     MF2
.............................ucccugagacccuaacuug................................................................    1     0     MF2
.............................ucccugagacccuaacuugug..............................................................    8     0     MF2
.............................ucccugagacccuaacuugugG.............................................................    18    1     MF2
.............................ucccugagacccuaacuuguga.............................................................    150   0     MF2
.............................ucccugagacccuaacuugugac............................................................    53    0     MF2
.............................ucccugagacccuaacuugugaU............................................................    1     1     MF2
.............................ucccugagacccuaacuugAgac............................................................    1     1     MF2
.............................ucccugagacccuaacuugugaA............................................................    17    1     MF2
.............................ucccugagacccuaacuugugacC...........................................................    2     1     MF2
.............................ucccugagacccuaacuugugacu...........................................................    20    0     MF2
.............................ucccugagacccuaacuugugacuU..........................................................    1     1     MF2
.............................ucccugagacccuaacuugugacuaucguugcaU.................................................    1     1     MF2
.............................ucccugagacccuaacuugugacuaucguugcaaa................................................    1     0     MF2
.............................ucccugagacccuaacuugugacuaucguugcaaag...............................................    1     0     MF2
.............................ucccugagacccuaacuugugacuaucguugcaaaguuu............................................    5     0     MF2
..............................cccugagacccuaacuugugacu...........................................................    1     0     MF2
................................cugagacccuaacuuguga.............................................................    1     0     MF2
...................................................................ucacaaguuuugaucuccgguau......................    1     0     MF2
.....................................................................acaaguuuugaucuccgguau......................    12    0     MF2
.....................................................................acaaguuuugaucuccgguauu.....................    2     0     MF2

............................uucccugagacccuaacuuguga.............................................................    3     0     FW2
............................uucccugagacccuaacuugugaA............................................................    2     1     FW2
.............................ucccugagacccuaacu..................................................................    2     0     FW2
.............................ucccugagacccuaacuu.................................................................    1     0     FW2
.............................ucccugagacccuaacuug................................................................    2     0     FW2
.............................ucccugagacccuaacuugu...............................................................    1     0     FW2
.............................ucccugagacccuaacuugug..............................................................    11    0     FW2
```

```
                                                                     aga-miR-125*
                             aga-miR-125
uauuguuucguuuuuuuguaucugcugauucccugagacccuaacuugugacuaucguugcaaaguuucacaaguuuugaucuccgguauuagcgguugagaugcaacggua

.............................ucccugagacccuaacuuguga.............................................................   143   0   FW2
.............................ucccCgagacccuaacuuguga.............................................................   1     1   FW2
.............................ucccugagacccuaacuugugG.............................................................   11    1   FW2
.............................ucccugagacccuaacuugugaG............................................................   1     1   FW2
.............................ucccugagacccuaacuugugaA............................................................   11    1   FW2
.............................ucccugagaccAuaacuugugac............................................................   1     1   FW2
.............................ucccugagGcccuaacuugugac............................................................   1     1   FW2
.............................ucccugagacccuaacuugugac............................................................   134   0   FW2
.............................ucccugagacccuaacuugugacC...........................................................   1     1   FW2
.............................ucccugagacccuaacuugugacA...........................................................   1     1   FW2
.............................ucccugagacccuaacuugugacu...........................................................   40    0   FW2
.............................ucccugagacccuaacuugugacua..........................................................   1     0   FW2
.............................ucccugagacccuaacuugugacuaucguugcaaa................................................   1     0   FW2
.............................ucccugagacccuaacuugugacuaucguugcaaagu..............................................   2     0   FW2
.............................ucccugagacccuaacuugugacuaucguugcaaaguu.............................................   1     0   FW2
.............................ucccugagacccuaacuugugacuaucguugcaaaguuu............................................   6     0   FW2
..............................cccugagacccuaacuugugacu...........................................................   1     0   FW2
...............................ccugagacccuaacuugugacu...........................................................   2     0   FW2
...................................agacccuaacuugugac............................................................   1     0   FW2
...............................................gugacuaucguugcaaaguuucU..........................................   1     1   FW2
.................................................................uuucacaaguuuugaucuccggua.......................   1     0   FW2
...................................................................ucacaaguuuugaucuccgguau......................   3     0   FW2
.....................................................................acaaguuuugaucuccgguau......................   7     0   FW2
.....................................................................acaaguuuugaucuccgguauu.....................   2     0   FW2

............................uucccugagacccuaacuuguga.............................................................   4     0   FF1
............................uucccugagacccuaacuugugaA............................................................   2     1   FF1
.............................ucccugagacccuaacuugu...............................................................   9     0   FF1
.............................ucccugagaccUuaacuugug..............................................................   1     1   FF1
.............................ucccugagacccAaacuugug..............................................................   1     1   FF1
.............................ucccugagacccuaacuugug..............................................................   22    0   FF1
.............................ucccugagacccuaacuugugG.............................................................   18    1   FF1
.............................ucccugGgacccuaacuuguga.............................................................   1     1   FF1
.............................ucccGgagacccuaacuuguga.............................................................   1     1   FF1
.............................ucccugagacccuaacuuguga.............................................................   138   0   FF1
.............................ucccugagacGcuaacuuguga.............................................................   1     1   FF1
.............................ucccugagacccuaacuugugaA............................................................   25    1   FF1
.............................ucccugagacccuaacuugugaU............................................................   2     1   FF1
.............................Acccugagacccuaacuugugac............................................................   1     1   FF1
.............................ucccugagacccuaacuugugac............................................................   80    0   FF1
.............................ucccugagacccuaacuugugacu...........................................................   28    0   FF1
.............................ucccugagacccuaacuugugacC...........................................................   1     1   FF1
.............................ucccugagacccuaacuugugacA...........................................................   2     1   FF1
.............................ucccugagacccuaacuugugacua..........................................................   3     0   FF1
.............................ucccugagacccuaacuugugacuaucg.......................................................   1     0   FF1
.............................ucccugagacccuaacuugugacuaucgu......................................................   6     0   FF1
.............................ucccugagacccuaacuugugacuaucguugcaaa................................................   4     0   FF1
.............................ucccugagacccuaacuugugacuaucguugcaaU................................................   1     1   FF1
.............................ucccugagacccuaacuugugacuaucguugcaaaU...............................................   2     1   FF1
.............................ucccugagacccuaacuugugacuaucguugcaaag...............................................   5     0   FF1
.............................ucccugagacccuaacuugugacuaucguugcaaagu..............................................   2     0   FF1
.............................ucccugagacccuaacuugugacuaucguugcaaaguu.............................................   2     0   FF1
.............................ucccugagacccuaacuugugacuaucguugcaaaguuu............................................   5     0   FF1
.............................ucccugagacccuaacuugugacuaucguugcaaaguuuc...........................................   1     0   FF1
..............................cccugagacccuaacuugugacu...........................................................   1     0   FF1
...................................................................ucacaaguuuugaucuccgguau......................   5     0   FF1
.....................................................................acaaguuuugaucuccgguau......................   9     0   FF1

............................uucccugagacccuaacuugu...............................................................   2     0   OV1
............................uucccugagacccuaacuugug..............................................................   3     0   OV1
............................uucccugagacccuaacuuguga.............................................................   5     0   OV1
............................uucccugagacccuaacuugugaA............................................................   1     1   OV1
.............................ucccugagacccuaacu..................................................................   7     0   OV1
.............................ucccugagacccuaacuug................................................................   5     0   OV1
.............................ucccugagacccuaacuugu...............................................................   29    0   OV1
.............................ucccugagacccuaacuugug..............................................................   116   0   OV1
.............................ucccugagaUccuaacuuguga.............................................................   1     1   OV1
.............................ucccugagacccuaacuugugU.............................................................   1     1   OV1
.............................ucccugCgacccuaacuuguga.............................................................   1     1   OV1
```

```
                                                                   aga-miR-125*
                              aga-miR-125
uauuguuucguuuuuuuguaucugcugauucccugagacccuaacuugugacuaucguugcaaaguuucacaaguuuugaucuccgguauuagcgguugagaugcaacggua

.............................ucccugagacccuaacuugugG.............................................................    37    1    OV1
.............................ucccuAagacccuaacuuguga.............................................................    1     1    OV1
.............................ucccugagacccuaacuuguga.............................................................    418   0    OV1
.............................ucccugagacccCaacuuguga.............................................................    1     1    OV1
.............................ucccugagacUcuaacuuguga.............................................................    2     1    OV1
.............................ucccugagaccUuaacuuguga.............................................................    1     1    OV1
.............................ucccugagacccuaacuugugaA............................................................    125   1    OV1
.............................ucccugagacccCaacuugugac............................................................    1     1    OV1
.............................Ccccugagacccuaacuugugac............................................................    1     1    OV1
.............................ucccugagacccuaacuugugac............................................................    111   0    OV1
.............................ucccugagaAccuaacuugugac............................................................    1     1    OV1
.............................ucccugagacccuaacuugugaU............................................................    4     1    OV1
.............................ucccugagacccuaacCugugac............................................................    1     1    OV1
.............................ucccugagacccuaacuugugacu...........................................................    60    0    OV1
.............................ucccugagacccuaacuugugaAu...........................................................    2     1    OV1
.............................ucccugagacccuaacuugugacA...........................................................    3     1    OV1
.............................ucccugagacccuaacuugugacuU..........................................................    1     1    OV1
.............................ucccugagacccuaacuugugacua..........................................................    4     0    OV1
.............................ucccugagacccuaacuugugacuau.........................................................    1     0    OV1
.............................ucccugagacccuaacuugugacuauc........................................................    2     0    OV1
.............................ucccugagacccuaacuugugacuaucg.......................................................    1     0    OV1
.............................ucccugagacccuaacuugugacuaucgu......................................................    9     0    OV1
.............................ucccugagacccuaacuugugacuaucguu.....................................................    1     0    OV1
.............................ucccugagacccuaacuugugacuaucguug....................................................    1     0    OV1
.............................ucccugagacccuaacuugugacuaucguugcaa.................................................    1     0    OV1
.............................ucccugagacccuaacuugugacuaucguugcaaU................................................    2     1    OV1
.............................ucccugagacccuaacuugugacuaucguugcaaa................................................    7     0    OV1
.............................ucccugagacccuaacuugugacuaucguugcaaaU...............................................    1     1    OV1
.............................ucccugagacccuaacuugugacuaucguugcaaagu..............................................    8     0    OV1
.............................ucccugagacccuaacuugugacuaucguugcaaaguu.............................................    1     0    OV1
..............................cccugagacccuaacuugugacu...........................................................    1     0    OV1
...............................ccugagacccuaacuuguga.............................................................    2     0    OV1
...................................................................ucacaaguuuugaucuccgguau......................    4     0    OV1
....................................................................cacaaguuuugaucuccgguau......................    1     0    OV1
.....................................................................acaaguuuugaucuccgguau......................    8     0    OV1
.....................................................................acaaguuuugaucuccgguauu.....................    1     0    OV1

............................uucccugagacccuaacuuguga.............................................................    1     0    MF1
.............................ucccugagacccuaacuug................................................................    1     0    MF1
.............................ucccugagacccuaacuugug..............................................................    2     0    MF1
.............................ucccugagacccuaacuuguga.............................................................    44    0    MF1
.............................ucccugagacccuaacuugugG.............................................................    5     1    MF1
.............................ucccugagacccuaacuugugac............................................................    11    0    MF1
.............................ucccugagacccuaacuugugaA............................................................    5     1    MF1
...................................................................ucacaaguuuugaucuccgguau......................    1     0    MF1
.....................................................................acaaguuuugaucuccgguau......................    4     0    MF1
.....................................................................acaaguuuugaucuccgguauu.....................    1     0    MF1

............................uucccugagacccuaacuugugG.............................................................    1     1    BF2
............................uucccugagacccuaacuuguga.............................................................    4     0    BF2
.............................ucccugagacccuaacu..................................................................    3     0    BF2
.............................ucccugagacccuaacuug................................................................    3     0    BF2
.............................ucccugagacccuaacuugu...............................................................    5     0    BF2
.............................ucccugagacccuaacuugug..............................................................    15    0    BF2
.............................uccGugagacccuaacuuguga.............................................................    1     1    BF2
.............................ucccugagacUcuaacuuguga.............................................................    2     1    BF2
.............................ucccugagacccuaacuuguga.............................................................    193   0    BF2
.............................ucccCgagacccuaacuuguga.............................................................    1     1    BF2
.............................ucccugagacccuaacuuUuga.............................................................    1     1    BF2
.............................ucccugagacccuaacuugugG.............................................................    27    1    BF2
.............................ucccugagacccuaacuugugaA............................................................    44    1    BF2
.............................ucccugagacccuaacuugugac............................................................    98    0    BF2
.............................ucccugagacccuaacuugugaU............................................................    2     1    BF2
.............................ucccugagacccuaacuugugacu...........................................................    33    0    BF2
.............................ucccugagacccuaacuugugaAu...........................................................    1     1    BF2
.............................ucccugagacccuaacuugugacA...........................................................    3     1    BF2
.............................ucccugagacccuaacuugugacua..........................................................    4     0    BF2
.............................ucccugagacccuaacuugugacuaucgu......................................................    12    0    BF2
.............................ucccugagacccuaacuugugacuaucguugcaaa................................................    1     0    BF2
```

```
                                                                 aga-miR-125*
                                         aga-miR-125
uauuguuucguuuuuuuguaucugcugauucccugagacccuaacuugugacuaucguugcaaaguuucacaaguuuugaucuccgguauuagcgguugagaugcaacggua

.............................ucccugagacccuaacuugugacuaucguugcaaag...............................................   2     0     BF2
.............................ucccugagacccuaacuugugacuaucguugcaaaguuu............................................   1     0     BF2
.............................ucccugagacccuaacuugugacuaucguugcaaaguuuU...........................................   1     1     BF2
...............................ccugagacccuaacuugug..............................................................   1     0     BF2
...............................ccugagacccuaacuugugac............................................................   1     0     BF2
.................................ugagacccuaacuuguga.............................................................   1     0     BF2
..............................................ugugacuaucguugcaaaguu.............................................   1     0     BF2
..............................................ugugacuaucguugcaaaguuuc...........................................   1     0     BF2
...................................................................ucacaaguuuugaucuccgguau......................   3     0     BF2
.....................................................................acaaguuuugaucuccgguau......................   10    0     BF2
.....................................................................acaaguuuugaucuccgguauu.....................   2     0     BF2

............................uucccugagacccuaacuuguga.............................................................   4     0     BF1
............................uucccugagacccuaacuugugaA............................................................   2     1     BF1
.............................ucccugagacccuaacuugu...............................................................   9     0     BF1
.............................ucccugagacccAaacuugug..............................................................   1     1     BF1
.............................ucccugagacccuaacuugug..............................................................   22    0     BF1
.............................ucccugagaccUuaacuugug..............................................................   1     1     BF1
.............................ucccugagacccuaacuuguga.............................................................   138   0     BF1
.............................ucccGgagacccuaacuuguga.............................................................   1     1     BF1
.............................ucccugagacGcuaacuuguga.............................................................   1     1     BF1
.............................ucccugGgacccuaacuuguga.............................................................   1     1     BF1
.............................ucccugagacccuaacuugugG.............................................................   18    1     BF1
.............................ucccugagacccuaacuugugaA............................................................   25    1     BF1
.............................ucccugagacccuaacuugugaU............................................................   2     1     BF1
.............................ucccugagacccuaacuugugac............................................................   79    0     BF1
.............................Acccugagacccuaacuugugac............................................................   1     1     BF1
.............................ucccugagacccuaacuugugacC...........................................................   1     1     BF1
.............................ucccugagacccuaacuugugacA...........................................................   2     1     BF1
.............................ucccugagacccuaacuugugacu...........................................................   28    0     BF1
.............................ucccugagacccuaacuugugacua..........................................................   3     0     BF1
.............................ucccugagacccuaacuugugacuaucg.......................................................   1     0     BF1
.............................ucccugagacccuaacuugugacuaucgu......................................................   6     0     BF1
.............................ucccugagacccuaacuugugacuaucguugcaaa................................................   4     0     BF1
.............................ucccugagacccuaacuugugacuaucguugcaaU................................................   1     1     BF1
.............................ucccugagacccuaacuugugacuaucguugcaaag...............................................   5     0     BF1
.............................ucccugagacccuaacuugugacuaucguugcaaaU...............................................   2     1     BF1
.............................ucccugagacccuaacuugugacuaucguugcaaagu..............................................   2     0     BF1
.............................ucccugagacccuaacuugugacuaucguugcaaaguu.............................................   2     0     BF1
.............................ucccugagacccuaacuugugacuaucguugcaaaguuu............................................   5     0     BF1
.............................ucccugagacccuaacuugugacuaucguugcaaaguuuc...........................................   1     0     BF1
..............................cccugagacccuaacuugugacu...........................................................   1     0     BF1
...................................................................ucacaaguuuugaucuccgguau......................   5     0     BF1
.....................................................................acaaguuuugaucuccgguau......................   9     0     BF1

............................uucccugagacccuaacuuguga.............................................................   2     0     FW1
.............................ucccugagacccuaacuug................................................................   2     0     FW1
.............................ucccugagacccuaacuugu...............................................................   1     0     FW1
.............................ucccugagacccuaacuugug..............................................................   4     0     FW1
.............................ucccugagacccuaacuugugG.............................................................   12    1     FW1
.............................ucccugagacccuaacuuguga.............................................................   78    0     FW1
.............................ucccugagacccuaacuugugaA............................................................   7     1     FW1
.............................ucccugagacccuaacuugugac............................................................   57    0     FW1
.............................ucccugagacccuaacuuAugac............................................................   1     1     FW1
.............................ucccugagacccuaacuugugacu...........................................................   16    0     FW1
.............................ucccugagacccuaacuugugacuaucguugcaaaguu.............................................   1     0     FW1
..............................cccugagacccuaacuugugacu...........................................................   2     0     FW1
...................................................................ucacaaguuuugaucuccgguau......................   1     0     FW1
.....................................................................acaaguuuugaucuccggua.......................   1     0     FW1
.....................................................................acaaguuuugaucuccgguau......................   8     0     FW1
.....................................................................acaaguuuugaucuccgguauu.....................   1     0     FW1

............................uucccugagacccuaacuugug..............................................................   1     0     MW1
............................uucccugagacccuaacuuguga.............................................................   2     0     MW1
............................Cucccugagacccuaacuugugac............................................................   1     1     MW1
.............................ucccugagacccuaacC..................................................................   1     1     MW1
.............................ucccugagacccuaacu..................................................................   7     0     MW1
.............................ucccugagacccuaacuug................................................................   3     0     MW1
.............................ucccugagacccuaacuugu...............................................................   2     0     MW1
```

```
                                                                              aga-miR-125*
                                       aga-miR-125
uauuguuucguuuuuuuguaucugcugauucccugagacccuaacuugugacuaucguugcaaaguuucacaaguuuugaucuccgguauuagcgguugagaugcaacggua

.............................ucccugagacccuaacuugug..............................................................    12     0     MW1
.............................ucccugagacccuaacuuguga.............................................................    145    0     MW1
.............................ucccugagacccuaacuugugG.............................................................    18     1     MW1
.............................ucccugagacccuaacuGguga.............................................................    1      1     MW1
.............................ucccugagacccuCacuuguga.............................................................    1      1     MW1
.............................uUccugagacccuaacuuguga.............................................................    1      1     MW1
.............................ucccugagacccuaacuugugaA............................................................    5      1     MW1
.............................ucccugagacccuaacuugugaU............................................................    1      1     MW1
.............................ucccugagacccuaacuuAugac............................................................    1      1     MW1
.............................ucccugagacccuaacuugugac............................................................    103    0     MW1
.............................ucccugagacccuaacuugugacu...........................................................    27     0     MW1
.............................ucccugagacccuaacuugugacuG..........................................................    1      1     MW1
.............................ucccugagacccuaacuugugacua..........................................................    2      0     MW1
.............................ucccugagacccuaacuugugacuaucgu......................................................    1      0     MW1
.............................ucccugagacccuaacuugugacuaucguugcaaagu..............................................    2      0     MW1
.............................ucccugagacccuaacuugugacuaucguugcaaaguu.............................................    1      0     MW1
.............................ucccugagacccuaacuugugacuaucguugcaaaguCuc...........................................    1      1     MW1
...............................ccugagacccuaacuugugaA............................................................    1      1     MW1
...................................................................ucacaaguuuugaucuccgguau......................    4      0     MW1
.....................................................................acaaguuuugaucuccgguau......................    7      0     MW1
.....................................................................acaaguuuugaucuccgguauu.....................    5      0     MW1
.....................................................................acaaguuuugaucuccgguauA.....................    1      1     MW1

............................uucccugagacccuaacuuguga.............................................................    1      0     MW2
.............................ucccugagacccuaacuug................................................................    1      0     MW2
.............................ucccugagacccuaacuugug..............................................................    1      0     MW2
.............................ucccugagacccuaacuuguga.............................................................    33     0     MW2
.............................ucccugagacccuaacuugugG.............................................................    7      1     MW2
.............................ucccugagacccuaacuuCugac............................................................    1      1     MW2
.............................ucccugagacccuaacuugugac............................................................    27     0     MW2
.............................ucccugagacccuaacuugugaA............................................................    3      1     MW2
.............................ucccugagacccuaacuugugaU............................................................    1      1     MW2
.............................ucccugagacccuaacuugugacu...........................................................    9      0     MW2
.............................ucccugagacccuaacuugugacuaucguugcaaaguu.............................................    2      0     MW2
..............................cccugagacccuaacuugugacu...........................................................    1      0     MW2
.....................................................................acaaguuuugaucuccgguau......................    1      0     MW2
.....................................................................acaaguuuugaucuccgguauC.....................    1      1     MW2

............................uucccugagacccuaacuuguga.............................................................    2      0     TE2
............................Cucccugagacccuaacuugugac............................................................    1      1     TE2
.............................ucccugagacccuaacu..................................................................    3      0     TE2
.............................ucccugagacccuaacuug................................................................    5      0     TE2
.............................ucccugagacccuaacuugu...............................................................    5      0     TE2
.............................ucccugagacccuaacuugug..............................................................    23     0     TE2
.............................ucccugagacccuaacuuguga.............................................................    181    0     TE2
.............................ucccugagacccuaacuugugG.............................................................    35     1     TE2
.............................Ccccugagacccuaacuuguga.............................................................    1      1     TE2
.............................uUccugagacccuaacuuguga.............................................................    1      1     TE2
.............................ucccugagacccuaacuugugaG............................................................    1      1     TE2
.............................ucccugagacccuaacuugugac............................................................    100    0     TE2
.............................ucccugagacccuaacuugugaU............................................................    1      1     TE2
.............................uccCgagacccuaacuugugac............................................................    1      1     TE2
.............................ucccugagacccuaacuugugaA............................................................    20     1     TE2
.............................uccCgagacccuaacuugugacu...........................................................    1      1     TE2
.............................ucccugagacccuaacuugugacu...........................................................    22     0     TE2
.............................ucccugagacccuaacuugugacuU..........................................................    1      1     TE2
.............................ucccugagacccuaacuugugacua..........................................................    2      0     TE2
.............................ucccugagacccuaacuugugacAa..........................................................    1      1     TE2
.............................ucccugagacccuaacuugugacuaucgu......................................................    1      0     TE2
..............................cccugagacccuaacuugugacu...........................................................    2      0     TE2
...............................ccugagacccuaacuug................................................................    1      0     TE2
...............................ccugagacccuaacuuguga.............................................................    1      0     TE2
...................................................................ucacaaguuuugaucuccgguau......................    7      0     TE2
...................................................................ucacaaguuuugaucuccgguauu.....................    1      0     TE2
....................................................................cacaaguuuugaucuccgAuau......................    1      1     TE2
.....................................................................acaaguuuugaGcuccgguau......................    1      1     TE2
.....................................................................acaaguuuugaucuccgguau......................    14     0     TE2
.....................................................................acaaguuuugaucuccgguauu.....................    2      0     TE2
```

miRBase precursor : aga-mir-12
Total read count : 8449
aga-mir-12 read count : 7325
aga-mir-12* read count : 1097
remaining reads : 27

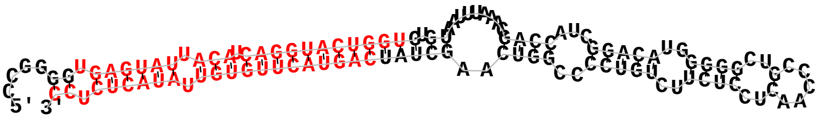

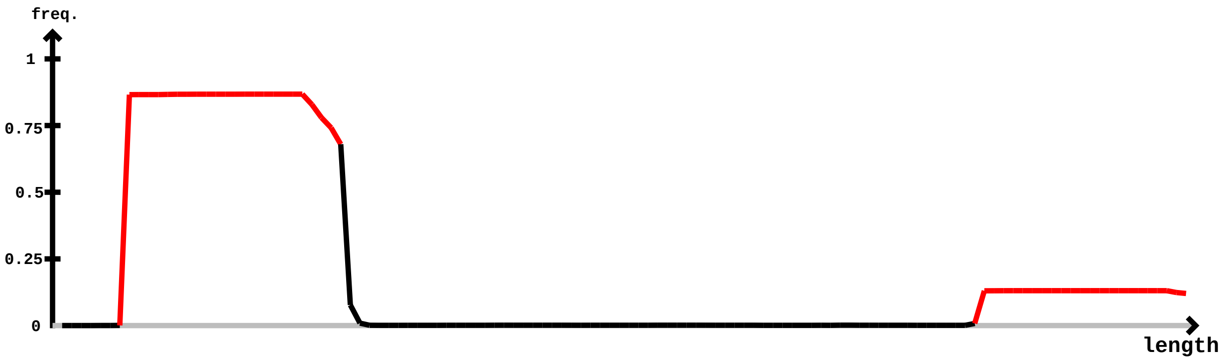


```
                                                                                                 aga-mir-12*
      aga-mir-12
5'- ccggggugaguauuacaucagguacugguguguaauuuaaacgaccaucggacaugggggcugcccaacuccucuucuguccccggucaagcuaucaguacuuguguuauacucucc   -3'   exp
    ....((.((((((.(((.((((((((((((.((........((.....))(((.(((((((................))))))))))..)))))))))))))))).)))))).))   reads   mm   sampl
    ......ugaguauuacaucagguac............................................................................................   6       0    TE1
    ......ugaguauuacaucagguacu...........................................................................................   5       0    TE1
    ......ugaguauuacaucagguacug..........................................................................................   3       0    TE1
    ......ugaguauuacaucagguacugg.........................................................................................   7       0    TE1
    ......ugaguauuacaucagguacuggG........................................................................................   1       1    TE1
    ......ugaguauuacaucagguacuggA........................................................................................   2       1    TE1
    ......ugaguauuacaucaggCacuggu........................................................................................   1       1    TE1
    ......ugaguauuacaucagguacuggu........................................................................................   65      0    TE1
    ......ugaguauuacaucagguacugguU.......................................................................................   6       1    TE1
    ......ugaguauuacaucagguacuggug.......................................................................................   1       0    TE1
    ......ugaguauuacaucagguacugguA.......................................................................................   1       1    TE1
    ......ugaguauuacaucagguacugguUu......................................................................................   1       1    TE1
    ..............................................................................................ucaguacuuguguuauacucu..   2       0    TE1
    ...............................................................................................caguacuuguguuauacucucc   13      0    TE1

    ......ugaguauuacaucagguac............................................................................................   23      0    OV2
    ......ugaguauuacaucagguacC...........................................................................................   1       1    OV2
    ......ugaguauuacaucagguacu...........................................................................................   9       0    OV2
    ......ugaguauuacaucagguacug..........................................................................................   19      0    OV2
    ......ugaguauuacaucagguacugg.........................................................................................   13      0    OV2
    ......Cgaguauuacaucagguacuggu........................................................................................   1       1    OV2
    ......ugaguauuacaucagguacuggu........................................................................................   231     0    OV2
    ......ugaguauuacaucagguacuggA........................................................................................   3       1    OV2
    ......ugaguauuacauGagguacuggu........................................................................................   1       1    OV2
    ......ugaguauuacaucagguUcuggu........................................................................................   1       1    OV2
    ......ugaguauuacCucagguacuggu........................................................................................   1       1    OV2
    ......ugaguauuacaucagguacugAu........................................................................................   1       1    OV2
    ......ugaguauuacaucagguacugguU.......................................................................................   10      1    OV2
    ......ugaguauuacaucagguacugguA.......................................................................................   11      1    OV2
    ......ugaguauuacaucagguacugguCu......................................................................................   4       1    OV2
    ......ugaguauuacaucagguacugguUu......................................................................................   1       1    OV2
    .............................................caucggacauggggUcugcccaacuccucuucuguccccgguc.............................   1       1    OV2
    ..............................................................................................ucaguacuuguguuauacucu..   1       0    OV2
    ...............................................................................................caguacuuguguuauacucu..   1       0    OV2
```

```
                                                                                               aga-mir-12*
      aga-mir-12
ccggggugaguauuacaucagguacugguguguaauuuaaacgaccaucggacaugggggcugcccaacuccucuucuguccccggucaagcuaucaguacuuguguuauacucucc

...............................................................................................caguacuuguguuauacucucU  2     1     OV2
...............................................................................................caguacuuguguuauacucucc  30    0     OV2

......ugaguauuacaucagguac............................................................................................  1     0     FF2
......ugaguauuacaucagguacu...........................................................................................  26    0     FF2
......ugaguauuacCucagguacug..........................................................................................  1     1     FF2
......ugaguauuacaucagguacug..........................................................................................  26    0     FF2
......ugaguauuacaucagguUcugg.........................................................................................  1     1     FF2
......ugaguauuacaucagguacugA.........................................................................................  1     1     FF2
......ugaguauuacaucagguacugg.........................................................................................  25    0     FF2
......Cgaguauuacaucagguacuggu........................................................................................  1     1     FF2
......ugaguauuacaucagguacuggC........................................................................................  1     1     FF2
......ugaguauuacaucaggGacuggu........................................................................................  2     1     FF2
......ugaguauuacaucagguacuggA........................................................................................  4     1     FF2
......ugaguauuacaucagguacuggu........................................................................................  179   0     FF2
......ugagCauuacaucagguacuggu........................................................................................  1     1     FF2
......ugaguauuacaucagguacugguU.......................................................................................  16    1     FF2
......ugaguauuacaucagguacugguA.......................................................................................  2     1     FF2
......ugaguauuacaucagguacuggug.......................................................................................  1     0     FF2
.................................................................................cccggucaagcuaucaguacuuguguuauacucucc  1     0     FF2
..............................................................................................ucaguacuuguguuauacucu..  1     0     FF2
...............................................................................................caguacuuguguuauacucucc  31    0     FF2
...............................................................................................caguacuuguguuauacucucU  2     1     FF2

...Uggugaguauuacaucagguacuggu........................................................................................  2     1     MF2
.....Cugaguauuacaucagguacuggu........................................................................................  2     1     MF2
......ugaguauuacaucaggGac............................................................................................  1     1     MF2
......ugaguauuacaucagguac............................................................................................  95    0     MF2
......uCaguauuacaucagguacu...........................................................................................  1     1     MF2
......ugaguauuacaucagguacC...........................................................................................  2     1     MF2
......ugaguauuacaucagguacu...........................................................................................  192   0     MF2
......ugagCauuacaucagguacug..........................................................................................  1     1     MF2
......Cgaguauuacaucagguacug..........................................................................................  1     1     MF2
......ugaguauuacaucagAuacug..........................................................................................  1     1     MF2
......ugaguauuacaucagguacug..........................................................................................  160   0     MF2
......ugaguauuacaucaggCacugg.........................................................................................  1     1     MF2
......ugaguauuacaucagguacugg.........................................................................................  249   0     MF2
......ugaguauuacaucagguacGgg.........................................................................................  1     1     MF2
......ugaguauuacaucagguacugU.........................................................................................  27    1     MF2
......ugaguauuacaucagguacugA.........................................................................................  1     1     MF2
......ugaguauuacaucagguacugC.........................................................................................  2     1     MF2
......ugaguauuacaAcagguacuggu........................................................................................  1     1     MF2
......uAaguauuacaucagguacuggu........................................................................................  4     1     MF2
......ugaguauuacauUagguacuggu........................................................................................  1     1     MF2
......ugaguauuacaucagguacuggA........................................................................................  61    1     MF2
......ugaguauuacaucagguacuggC........................................................................................  4     1     MF2
......ugagCauuacaucagguacuggu........................................................................................  1     1     MF2
......ugaguauuacaucagguacugAu........................................................................................  4     1     MF2
......Cgaguauuacaucagguacuggu........................................................................................  3     1     MF2
......ugaguauuaGaucagguacuggu........................................................................................  1     1     MF2
......ugaguauuacaucagguacuggG........................................................................................  10    1     MF2
......ugaguaCuacaucagguacuggu........................................................................................  1     1     MF2
......ugaguauuaUaucagguacuggu........................................................................................  1     1     MF2
......ugaguauuacaucagguacugCu........................................................................................  1     1     MF2
......ugaguauuacaucagguacugUu........................................................................................  4     1     MF2
......ugaguaAuacaucagguacuggu........................................................................................  1     1     MF2
......ugaguauuacaucagguacuAgu........................................................................................  1     1     MF2
......ugaguauuacaucagguaUuggu........................................................................................  1     1     MF2
......ugaguauuacaucagAuacuggu........................................................................................  1     1     MF2
......ugaguauuacaucaggCacuggu........................................................................................  2     1     MF2
......ugaguauuacaCcagguacuggu........................................................................................  2     1     MF2
......ugaguauuacaucagguacuggu........................................................................................  2449  0     MF2
......ugaguauuacaGcagguacuggu........................................................................................  1     1     MF2
......ugagAauuacaucagguacuggu........................................................................................  1     1     MF2
......ugaguauuacaucagUuacuggu........................................................................................  1     1     MF2
......ugaguauuacaucagguacugguC.......................................................................................  6     1     MF2
......ugaguauuacaucagguacugguA.......................................................................................  45    1     MF2
......ugaguauuacaucagguacugguU.......................................................................................  290   1     MF2
......ugaguauuacaucagguacuggug.......................................................................................  4     0     MF2
```

```
                                                                                               aga-mir-12*
      aga-mir-12
ccggggugaguauuacaucagguacugguguguaauuuaaacgaccaucggacaugggggcugcccaacuccucuucuguccccggucaagcuaucaguacuuguguuauacucucc

......ugaguauuacaucagguacugguAu......................................................................................  17   1   MF2
......ugaguauuacaucagguacugguUu......................................................................................  12   1   MF2
......ugaguauuacaucagguacugguCu......................................................................................  13   1   MF2
......ugaguauuacaucagguacugguUug.....................................................................................  2    1   MF2
......ugaguauuacaucagguacugguguguaauuuaaacgGccaucgga.................................................................  2    1   MF2
.............................guguaauuuaaacgGccaucggacaugggggcugccca..................................................  1    1   MF2
.............................guguaauuuaaacgGccaucggacaugggggcugcccaacuccucuucug......................................  1    1   MF2
...................................uuuaaacgaccaucggacaugggggcugcccaacucc.............................................  1    0   MF2
............................................ccaucggacaugggggcugcccaacuccucuucuguc....................................  1    0   MF2
....................................................caugggggcugcccaacuccucuucugucc...................................  1    0   MF2
..............................................................................guccccggucaagcuaucaguacuuguguuauacucucc  1    0   MF2
................................................................................ccccggucaagcuaucaguacuuguguuauacucuc.  2    0   MF2
................................................................................ccccggucaagcuaucaguacuuguguuauacucucc  4    0   MF2
..............................................................................................ucaguacuuguguuauacucu..  9    0   MF2
..............................................................................................ucaguacuuguguuauacucuc.  1    0   MF2
..............................................................................................ucaguacuuguguuauacucucU  3    1   MF2
...............................................................................................caguacuuguguuauacucu..  4    0   MF2
...............................................................................................caguacuuguguuauacucuc.  11   0   MF2
...............................................................................................caguacuuguguuauacucucG  1    1   MF2
...............................................................................................caguacGuguguuauacucucc  1    1   MF2
...............................................................................................caUuacuuguguuauacucucc  1    1   MF2
...............................................................................................caguacuuguguCauacucucc  1    1   MF2
...............................................................................................caguacuugugGuauacucucc  1    1   MF2
...............................................................................................caguacuuguguuauacucucc  445  0   MF2
...............................................................................................cagGacuuguguuauacucucc  1    1   MF2
...............................................................................................caguacuuguguuauacucucU  16   1   MF2
...............................................................................................caguacuuguguuauacuGucc  1    1   MF2
...............................................................................................caguacuugugAuauacucucc  1    1   MF2
................................................................................................aguacuuguguuauacucucc  1    0   MF2
.................................................................................................guacuuguguuauacucucc  1    0   MF2
..................................................................................................uacuuguguuauacucucc  1    0   MF2

......ugagGauuacaucagguac............................................................................................  1    1   FW2
......ugaguauuacaucagguac............................................................................................  14   0   FW2
......ugagGauuacaucagguacu...........................................................................................  1    1   FW2
......ugaguauuacaucagguacu...........................................................................................  31   0   FW2
......ugaguauuacaucagguacug..........................................................................................  11   0   FW2
......ugaguauuacaucagguacugC.........................................................................................  1    1   FW2
......ugaguauuacaucagguacugg.........................................................................................  17   0   FW2
......ugaguauuacaucagguacugU.........................................................................................  2    1   FW2
......ugaguauuacCucagguacuggu........................................................................................  1    1   FW2
......ugaguauuacaucagguacuggu........................................................................................  120  0   FW2
......ugaguauuacauUagguacuggu........................................................................................  1    1   FW2
......ugaguauuacaucagguacuggA........................................................................................  3    1   FW2
......ugaguauuacaucagguacuggG........................................................................................  1    1   FW2
......ugaguauuacaucagguacugguU.......................................................................................  5    1   FW2
......ugaguauuacaucagguacugguA.......................................................................................  2    1   FW2
......ugaguauuacaucagguacugguAu......................................................................................  1    1   FW2
..........uauuacaucagguacug..........................................................................................  1    0   FW2
..........uauuacaucagguacuggu........................................................................................  1    0   FW2
......................................................ugggggcugcccaacucc.............................................  1    0   FW2
..............................................................................................ucaguacuuguguuauacucu..  3    0   FW2
...............................................................................................caguacuuguguuauacucuc.  1    0   FW2
...............................................................................................caguacuuguguuauacucucc  28   0   FW2

......ugaguauuacaucagguac............................................................................................  29   0   OV1
......uAaguauuacaucagguac............................................................................................  1    1   OV1
......ugaguauuacaucagguacu...........................................................................................  12   0   OV1
......ugaguauuacaucagguacug..........................................................................................  7    0   OV1
......ugaguauuacaucagguacugg.........................................................................................  11   0   OV1
......ugaguauuacaucagguacuggA........................................................................................  5    1   OV1
......ugaguauuacaucagguacuggG........................................................................................  1    1   OV1
......ugaguauuacaucagguacuggu........................................................................................  307  0   OV1
......ugaguauuaAaucagguacuggu........................................................................................  1    1   OV1
......ugaguaCuacaucagguacuggu........................................................................................  1    1   OV1
......ugaguauuacaucagguacugguU.......................................................................................  25   1   OV1
......ugaguauuacaucagguacugguC.......................................................................................  1    1   OV1
......ugaguauuacaucagguacugguA.......................................................................................  8    1   OV1
......ugaguauuacaucagguacugguCu......................................................................................  4    1   OV1
```

```
                                                                                             aga-mir-12*
      aga-mir-12
ccggggugaguauuacaucagguacugguguguaauuuaaacgaccaucggacaugggggcugcccaacuccucuucugucccccggucaagcuaucaguacuuguguuauacucucc

......ugaguauuacaucagguacugguUu.......................................................................................    2    1    OV1
.............................................................ugcccaacuccucuucugucc....................................    2    0    OV1
...............................................................................................ucaguacuuguguuauacucu..    4    0    OV1
................................................................................................caguacuuguguuauacucu..    1    0    OV1
................................................................................................caguacuuguguuauacucuc.    5    0    OV1
................................................................................................caguacuuguguuauacucucU    2    1    OV1
................................................................................................caguacuuguguuauacucucc    41   0    OV1

......ugaguauuacaucagguac.............................................................................................    3    0    FF1
......ugaguauuacaucagguacu............................................................................................    4    0    FF1
......ugaguauuacaucagguacug...........................................................................................    1    0    FF1
......ugaguauuacaucagguacugg..........................................................................................    11   0    FF1
......ugaguauuacaucagguacuggA.........................................................................................    1    1    FF1
......ugaguauuacaucagguacuggu.........................................................................................    150  0    FF1
......ugaguauuacaucagguacuggug........................................................................................    2    0    FF1
......ugaguauuacaucagguacugguU........................................................................................    6    1    FF1
......ugaguauuacaucagguacugguA........................................................................................    2    1    FF1
...............................................................................................ucaguacuuguguuauacucu..    3    0    FF1
...............................................................................................ucaguacuuguguuauacucuc.    1    0    FF1
................................................................................................caguacuugugAuauacucucc    1    1    FF1
................................................................................................caguacuuguguuauacucucc    37   0    FF1

......ugaguauuacaucagguac.............................................................................................    52   0    MF1
......ugaguauuacaucagguacu............................................................................................    63   0    MF1
......ugaguauuacaucagguacug...........................................................................................    29   0    MF1
......ugaguauCacaucagguacug...........................................................................................    1    1    MF1
......ugaguauuacaucagguacugU..........................................................................................    6    1    MF1
......ugaguauuacaucagguacugC..........................................................................................    1    1    MF1
......ugaguauuacaucagguacugg..........................................................................................    25   0    MF1
......ugaguauuacaucagguacuggC.........................................................................................    2    1    MF1
......ugaguauuacaucagguacuggu.........................................................................................    171  0    MF1
......ugaguauuacaucagguacuggA.........................................................................................    4    1    MF1
......ugaguauuacaucagguacuggug........................................................................................    1    0    MF1
......ugaguauuacaucagguacugguA........................................................................................    8    1    MF1
......ugaguauuacaucagguacugguU........................................................................................    22   1    MF1
......ugaguauuacaucagguacugguC........................................................................................    1    1    MF1
......ugaguauuacaucagguacugguCu.......................................................................................    1    1    MF1
...............................................................................................ucaguacuuguguuauacucu..    1    0    MF1
...............................................................................................ucaguacuuguguuauacucuc.    1    0    MF1
................................................................................................caguacuuguguuauacucu..    3    0    MF1
................................................................................................caguacuuguguuauacucuc.    6    0    MF1
................................................................................................caguacuuguguuauacucucc    115  0    MF1
................................................................................................caguacuuguguuauacucucA    1    1    MF1
.................................................................................................aguacuuguguuauacucucc    1    0    MF1

.....Cugaguauuacaucagguacuggu.........................................................................................    1    1    BF2
......ugaguauuacaucagguac.............................................................................................    35   0    BF2
......ugaguauuacaucagCuacu............................................................................................    1    1    BF2
......ugaguauuacaucagguacu............................................................................................    15   0    BF2
......ugaguauuacaucagguacug...........................................................................................    13   0    BF2
......ugaguauuacaucagguacGgg..........................................................................................    1    1    BF2
......ugaguauuacaucagguacugU..........................................................................................    1    1    BF2
......ugaguauuacaucagguacugg..........................................................................................    37   0    BF2
......ugaguauuacaucagguaUuggu.........................................................................................    1    1    BF2
......ugaguauuacaucaAguacuggu.........................................................................................    1    1    BF2
......ugaguauuacaucagguacuggA.........................................................................................    2    1    BF2
......ugaguauuacaucagguacuggG.........................................................................................    4    1    BF2
......uAaguauuacaucagguacuggu.........................................................................................    1    1    BF2
......ugagCauuacaucagguacuggu.........................................................................................    1    1    BF2
......ugaguauuacaAcagguacuggu.........................................................................................    2    1    BF2
......ugaguauuacaucagguacuggu.........................................................................................    553  0    BF2
......ugaguauuacauUagguacuggu.........................................................................................    1    1    BF2
......ugaguauuacaucagUuacuggu.........................................................................................    1    1    BF2
......ugaguauuacaucagguacugguU........................................................................................    30   1    BF2
......ugaguauuacaucagguacugguA........................................................................................    9    1    BF2
......ugaguauuacaucagguacugguC........................................................................................    1    1    BF2
......ugaguauuacaucagguacugguAu.......................................................................................    2    1    BF2
......ugaguauuacaucagguacugguCu.......................................................................................    1    1    BF2
.......gaguauuacaucagguacuggu.........................................................................................    1    0    BF2
```

```
                                                                                               aga-mir-12*
      aga-mir-12
ccggggugaguauuacaucagguacugguguguaauuuaaacgaccaucggacaugggggcugcccaacuccucuucuguccccggucaagcuaucaguacuuguguuauacucucc

..............................................................................................ucaguacuuguguuauacuc...   1     0     BF2
..............................................................................................ucaguacuuguguuauacucu..   11    0     BF2
..............................................................................................ucaguacuuguguuauacucucU   1     1     BF2
...............................................................................................caguacuugugAuauacucucc   1     1     BF2
...............................................................................................caguacuuguguuauacucucc   91    0     BF2
...............................................................................................caguacuuguguuauacucucU   2     1     BF2

......ugaguauuacaucagguac............................................................................................   3     0     BF1
......ugaguauuacaucagguacu...........................................................................................   4     0     BF1
......ugaguauuacaucagguacug..........................................................................................   1     0     BF1
......ugaguauuacaucagguacugg.........................................................................................   11    0     BF1
......ugaguauuacaucagguacuggu........................................................................................   150   0     BF1
......ugaguauuacaucagguacuggA........................................................................................   1     1     BF1
......ugaguauuacaucagguacugguA.......................................................................................   2     1     BF1
......ugaguauuacaucagguacugguU.......................................................................................   6     1     BF1
......ugaguauuacaucagguacuggug.......................................................................................   2     0     BF1
..............................................................................................ucaguacuuguguuauacucu..   3     0     BF1
..............................................................................................ucaguacuuguguuauacucuc.   1     0     BF1
...............................................................................................caguacuuguguuauacucucc   37    0     BF1
...............................................................................................caguacuugugAuauacucucc   1     1     BF1

......ugaguauuacaucagguac............................................................................................   8     0     FW1
......ugaguauuacaucagguacu...........................................................................................   4     0     FW1
......ugaguauuacaucagguacug..........................................................................................   4     0     FW1
......ugaguauuacaucagguacugg.........................................................................................   12    0     FW1
......ugaguaCuacaucagguacuggu........................................................................................   1     1     FW1
......ugaguauuaUaucagguacuggu........................................................................................   1     1     FW1
......ugaguauuacaucagguacuggu........................................................................................   84    0     FW1
......ugaguauuacaCcagguacuggu........................................................................................   1     1     FW1
......ugaguauuacaucagguacugguA.......................................................................................   2     1     FW1
......ugaguauuacaucagguacugguU.......................................................................................   5     1     FW1
......ugaguauuacaucagguacugguCu......................................................................................   2     1     FW1
......ugaguauuacaucagguacugguUu......................................................................................   1     1     FW1
...........auuacaucagguacuggu........................................................................................   1     0     FW1
..................cagguacugguguguaauuuaaacgac........................................................................   1     0     FW1
..................cagguacugguguguaauuuaaacgGccaucggac................................................................   1     1     FW1
.............................................caucggacauggggUcugcccaacuccucuucugucccc.................................   1     1     FW1
..............................................................................................ucaguacuuguguuauacucu..   1     0     FW1
...............................................................................................caguacuuguguuauacucucc   15    0     FW1

......ugaguauuacaucaggCac............................................................................................   1     1     MW1
......ugaguauuacaucagguac............................................................................................   21    0     MW1
......ugaguauuacaucagguacu...........................................................................................   17    0     MW1
......ugaguauuacaucagguacug..........................................................................................   11    0     MW1
......ugaguauuacaucagguacugg.........................................................................................   34    0     MW1
......ugaguauuacaucagguacugU.........................................................................................   1     1     MW1
......ugaguauuacaucagguacuggu........................................................................................   189   0     MW1
......ugaguauuacauUagguacuggu........................................................................................   1     1     MW1
......ugaguauuacaucagguacuggA........................................................................................   2     1     MW1
......ugaguauuacaucagguacuggug.......................................................................................   1     0     MW1
......ugaguauuacaucagguacugguA.......................................................................................   3     1     MW1
......ugaguauuacaucagguacugguU.......................................................................................   10    1     MW1
......ugaguauuacaucagguacugguUu......................................................................................   1     1     MW1
......ugaguauuacaucagguacugguCu......................................................................................   2     1     MW1
.......gaguauuacaucagguacuggu........................................................................................   1     0     MW1
..........uauuacaucagguacug..........................................................................................   1     0     MW1
..........uauuacaucagguacugg.........................................................................................   1     0     MW1
...........auuacaucagguacuggu........................................................................................   3     0     MW1
.............uacaucagguacugguguguaauuuaaacgacc.......................................................................   1     0     MW1
..............................................................................................ucaguacuuguguuauacucu..   5     0     MW1
...............................................................................................caguacuuguguuauacucucU   2     1     MW1
...............................................................................................caguacuuguguuauacucucc   30    0     MW1

......ugaguauuacaucagguac............................................................................................   11    0     MW2
......ugaguauuacaucagguacu...........................................................................................   7     0     MW2
......ugaguauuacaucagguacug..........................................................................................   2     0     MW2
......ugaguauuacaucagguacugg.........................................................................................   5     0     MW2
......ugaguauuacaucagguacugU.........................................................................................   1     1     MW2
......ugaguauuacaucagguacuggu........................................................................................   28    0     MW2
```

```
                                                                                               aga-mir-12*
      aga-mir-12
ccggggugaguauuacaucagguacugguguguaauuuaaacgaccaucggacaugggggcugcccaacuccucuucuguccccggucaagcuaucaguacuuguguuauacucucc

......ugaguauuacaucagguacuggA........................................................................................   1   1   MW2
......ugaguauuacaucagguacugguU.......................................................................................   3   1   MW2
......ugaguauuacaucagguacugguUu......................................................................................   1   1   MW2
..........uauuacaucagguacuggu........................................................................................   2   0   MW2
...........auuacaucagguacuggu........................................................................................   1   0   MW2
............................................ccaucggacaugggggcugcccaacuccucuucugu.....................................   1   0   MW2
..............................................................................................ucaguacuuguguuauacucu..   1   0   MW2
...............................................................................................caguacuuguguuauacucucc   12  0   MW2

......ugaguauuacaucagguac............................................................................................   25  0   TE2
......ugaguauuacaucagguacu...........................................................................................   21  0   TE2
......ugaguauuacaucagguacug..........................................................................................   26  0   TE2
......ugaguauuacaucagguacugU.........................................................................................   3   1   TE2
......ugaguauuacaucagguacugg.........................................................................................   12  0   TE2
......ugaguauuacaucagguacuggu........................................................................................   229 0   TE2
......ugaguauuacaucagguacuggC........................................................................................   1   1   TE2
......ugaguauuacaucagUuacuggu........................................................................................   1   1   TE2
......ugaguauuacaucagguacugguU.......................................................................................   18  1   TE2
......ugaguauuacaucagguacugguA.......................................................................................   6   1   TE2
......ugaguauuacaucagguacugguC.......................................................................................   1   1   TE2
......ugaguauuacaucagguacugguCu......................................................................................   1   1   TE2
......ugaguauuacaucagguacugguAu......................................................................................   1   1   TE2
......ugaguauuacaucagguacugguUu......................................................................................   1   1   TE2
.......................................aacgaccaucggacauggggUcugccc...................................................   1   1   TE2
..............................................................................................ucaguacuuguguuauacucu..   2   0   TE2
..............................................................................................ucaguacuuguguuauacucuc.   1   0   TE2
..............................................................................................ucaguacuuguguuauacucucc   2   0   TE2
...............................................................................................caguacuuguguuauacucu..   1   0   TE2
...............................................................................................caguacuuguguuauacucucc   37  0   TE2
...............................................................................................caguacuuguguuaCacucucc   1   1   TE2
```

miRBase precursor : aga-mir-133
Total read count : 1518
aga-miR-133 read count : 742
aga-miR-133* read count : 17
aga-miR-133 read count : 742
aga-miR-133* read count : 17
remaining reads : 0

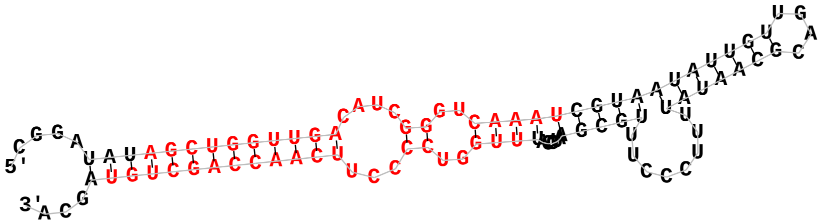

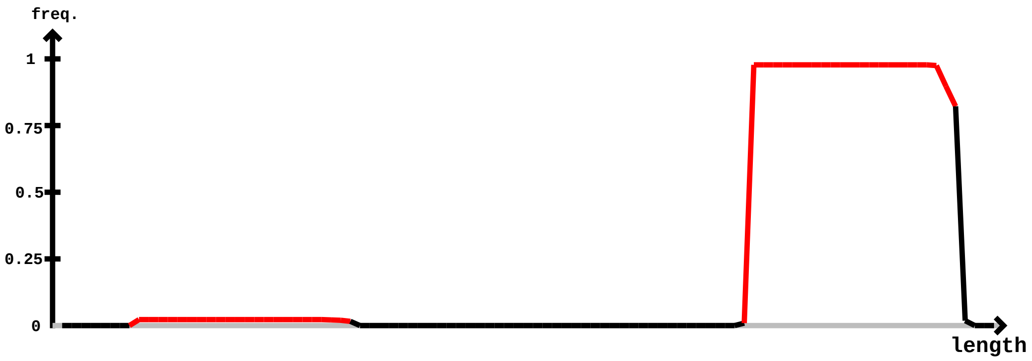

aga-miR-133*

aga-miR-133

```
5'- cggauauagcugguugacaucgggucaaaucguaauauuguugacgcaauauuuucccuuugcgaaugcauuuggucccuucaaccagcuguagca  -3'   exp
    ....(((((((((((((....((..(((((((((((((((....))))))).........))))).....))))..))...))))))))))))...   reads   mm   sample
    .......................................................................uuggucccuucaaccagcugu....   18      0    OV2

    .......................................................................uuggucccuucaaccagcug.....   3       0    TE1
    .......................................................................uuggucccuucaaccagcugu....   31      0    TE1

    .......................................................................uuggucccuucaaccagcu......   6       0    FF2
    .......................................................................uuggucccuucaaccagcug.....   3       0    FF2
    .......................................................................uuggucccuucaaccagcugu....   20      0    FF2
    .......................................................................uuggucccuucaaccagcuguU...   1       1    FF2

    .......agcugguugacaucggguca.....................................................................   1       0    MF2
    .......agcugguugacaucgggucaaau..................................................................   4       0    MF2
    ......................................................................uuuggucccuucaaccagcug.....   2       0    MF2
    ......................................................................uuuggucccuucaaccagcugu....   2       0    MF2
    .......................................................................uuggucccuucaaccagc.......   1       0    MF2
    .......................................................................uuggucccuucaaccagcu......   22      0    MF2
    .......................................................................uuggucccuucaaccagcug.....   21      0    MF2
    .......................................................................uuggucccuucaaccagcugu....   131     0    MF2
    .......................................................................uuggucccuucaacUagcugu....   1       1    MF2
    .......................................................................uuggucccuucaaccaAcugu....   2       1    MF2
    .......................................................................uuggucccuucaaccagcugua...   2       0    MF2
    .......................................................................uuggucccuucaaccagcuguU...   4       1    MF2
    .......................................................................uuggucccuucaaccagcuguC...   1       1    MF2

    .......agcugguugacaucgggucaa....................................................................   1       0    FW2
    .......agcugguugacaucgggucaaau..................................................................   1       0    FW2
    .......................................................................uuggucccuucaaccagcu......   6       0    FW2
    .......................................................................uuggucccuucaaccagcug.....   3       0    FW2
    .......................................................................uuggucccuucaaccagcCgu....   1       1    FW2
    .......................................................................uuggucccuucaaccagcugu....   72      0    FW2
    .......................................................................uuggucccuucaaccagcuguU...   1       1    FW2

    .......................................................................uuggucccuucaaccagc.......   1       0    OV1
    .......................................................................uuggucccuucaaccagcug.....   1       0    OV1
```

```
       aga-miR-133*
                                                                      aga-miR-133
cggauauagcugguugacaucgggucaaaucguaauauuguugacgcaauauuuucccuuugcgaaugcauuugguccccuucaaccagcuguagca

......................................................................uugguccccuucaaccagcugu....    3      0      OV1

.......agcugguugacaucgggucaaau...................................................................    1      0      FF1
......................................................................uugguccccuucaaccagcug.....    1      0      FF1
......................................................................uugguccccuucaaccagcugu....    4      0      FF1

.....................................................................uuugguccccuucaaccagcugu....    1      0      MF1
......................................................................uugguccccuucaaccagcu......    11     0      MF1
......................................................................uugguccccuucaaccagcug.....    10     0      MF1
......................................................................uugguccccuucaaccagcugu....    45     0      MF1
......................................................................uugguccccuucaaccagcuguU...    1      1      MF1

.......agcugguugacaucgggucaaau...................................................................    1      0      BF2
......................................................................uugguccccuucaaccagcugu....    15     0      BF2

.......agcugguugacaucgggucaaau...................................................................    1      0      BF1
......................................................................uugguccccuucaaccagcug.....    1      0      BF1
......................................................................uugguccccuucaaccagcugu....    4      0      BF1

.......agcugguugacaucgggucaaa....................................................................    1      0      FW1
.......agcugguugacaucgggucaaau...................................................................    1      0      FW1
......................................................................uugguccccuucaaccagcu......    2      0      FW1
......................................................................uugguccccuucaaccagcug.....    1      0      FW1
......................................................................uugguccccuucaaccagcugu....    43     0      FW1
......................................................................uAgguccccuucaaccagcugu....    1      1      FW1
......................................................................uugguccccuucaaccagcuguU...    1      1      FW1

.......agcugguugacaucgggucaaa....................................................................    2      0      MW1
.......agcuAguugacaucgggucaaau...................................................................    1      1      MW1
.....................................................................uuugguccccuucaaccagcugu....    1      0      MW1
......................................................................uugguccccuucaaccagcu......    5      0      MW1
......................................................................uugguccccuucaacUagcug.....    1      1      MW1
......................................................................uugguccccuucaaccagcug.....    3      0      MW1
......................................................................uugguccccuucaaccagcugu....    115    0      MW1
......................................................................uugguccccuucaaccagcuguU...    1      1      MW1

.......agcugguugacaucgggucaaau...................................................................    1      0      MW2
......................................................................uugguccccuucaaccagcu......    3      0      MW2
......................................................................uugguccccuucaaccagcug.....    2      0      MW2
......................................................................uugguccccuuGaaccagcugu....    1      1      MW2
......................................................................uugguccccuucaaccagcugu....    26     0      MW2
......................................................................uugguccccuucaaccagcuguU...    1      1      MW2

.......agcugguugacaucgggucaaau...................................................................    1      0      TE2
.....................................................................uuugguccccuucaaccagcugu....    1      0      TE2
......................................................................uugguccccuucaaccagcu......    4      0      TE2
......................................................................uugguccccuucaaccagcug.....    5      0      TE2
......................................................................uugguccccuucaaccagcugu....    71     0      TE2
......................................................................uuggucUcuucaaccagcugu....    1      1      TE2
......................................................................uugguccccuucaaccagcuguU...    1      1      TE2
```

miRBase precursor : aga-mir-137
Total read count : 195
aga-miR-137 read count : 174
aga-miR-137* read count : 20
remaining reads : 1

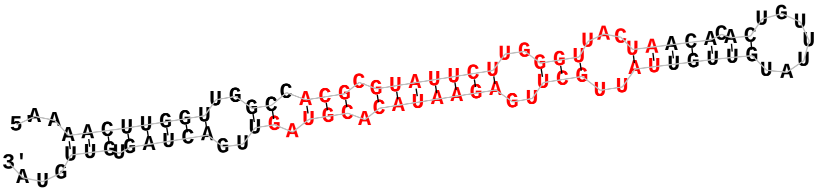

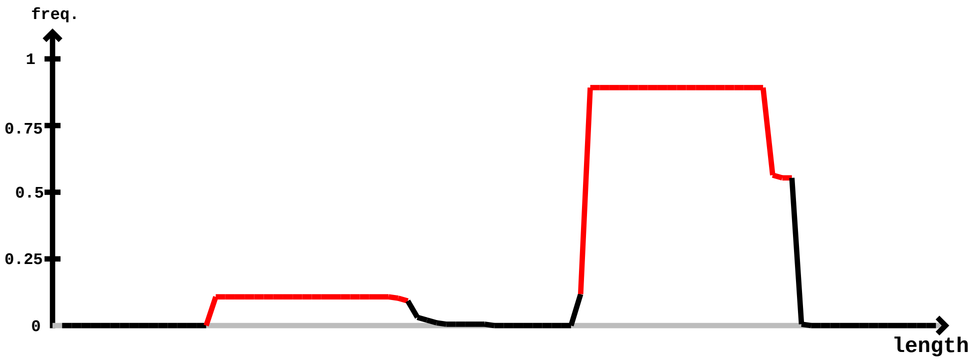


```
                                                      aga-miR-137
              aga-miR-137*
5'- aaaacuugguuggccacgcguauucuuggguuacuaacacacuguuuauguuguuauugcuugagaauacacguaguugacuaguguugua   -3'    exp
    ..((((((((..((.(((.(((((((..(((...(((((.((.......)))))))..)))..))))))).))).))..))))).)))...   reads      mm         sample
    ......................................................uauugcuugagaauacacguag...............   1          0          OV2

    ...............acgcguauucuuggguuacua.......................................................   1          0          TE1
    ...............acgcguauucuuggguuacuaa......................................................   1          0          TE1
    ...............acgcguauucuuggguuacuaacacacug...............................................   1          0          TE1
    ......................................................uauugcuugagaauacacg..................   1          0          TE1
    ......................................................uauugcuugagaauacacguag...............   3          0          TE1

    ...............acgcguauucuuggguuacua.......................................................   2          0          FF2
    ......................................................uauugcuugagaauacacg..................   4          0          FF2
    ......................................................uauugcuugagaauacacguag...............   3          0          FF2

    ...............acgcguauucuuggguuacu........................................................   2          0          MF2
    ...............acgcguauucuuggguuacua.......................................................   7          0          MF2
    ...............acAcguauucuuggguuacua.......................................................   1          1          MF2
    ...............acgcguauucuuggguuacuaa......................................................   1          0          MF2
    .....................................................uuauugcuugagaauacacg..................   3          0          MF2
    .....................................................uuauugcuugagaauacacguag...............   4          0          MF2
    .....................................................uuauugcuugagaauacacguagA..............   1          1          MF2
    ......................................................uauugcuugagaauacacg..................   20         0          MF2
    ......................................................uauugcuugagaauacacguag...............   27         0          MF2

    .....................................................uuauugcuugagaauacacg..................   2          0          FW2
    .....................................................uuauugcuugagaauacacguag...............   2          0          FW2
    ......................................................uauugcuugagaauacacg..................   7          0          FW2
    ......................................................uauugcuugagaauacacguag...............   13         0          FW2

    ...............acgcguauucuuggguuac.........................................................   1          0          MF1
    ...............acgcguauucuuggguuacuaac.....................................................   1          0          MF1
    .....................................................uuauugcuugagaauacacg..................   1          0          MF1
    .....................................................uuauugcuugagaauacacgu.................   1          0          MF1
    .....................................................uuauugcuugagaauacacguag...............   3          0          MF1
    ......................................................uauugcuugagaauacacg..................   11         0          MF1
    ......................................................uauugcuugagaauacacguag...............   16         0          MF1
```

```
                                                      aga-miR-137
               aga-miR-137*
aaaacuugguuggccacgcguauucuuggguuacuaacacacuguuuauguuguuauugcuugagaauacacguaguugacuaguguugua

......................................................uauugcuugagaauacacg..................  1   0   FW1
......................................................uauugcuugagaauacacguag...............  11  0   FW1

...............acgcguauucuuggguuacuaac.....................................................  1   0   MW1
.....................................................uuauugcuugagaauacacg..................  2   0   MW1
.....................................................uuauugcuugagaauacacguag...............  3   0   MW1
......................................................uauugcuugagaauacacg..................  2   0   MW1
......................................................uauugcuugagaauacacguag...............  13  0   MW1

...............acgcguauucuuggguuacuaaca....................................................  1   0   MW2
......................................................uauugcuugagaauacacg..................  4   0   MW2
......................................................uauugcuugagaauacacguag...............  4   0   MW2

...............acgcguauucuuggguuacua.......................................................  1   0   TE2
.....................................................uuauugcuugagaauacacgu.................  1   0   TE2
......................................................uauugcuugagaauacacg..................  6   0   TE2
......................................................uauugcuugagaauacacguag...............  4   0   TE2
```

miRBase precursor : aga-mir-13b
Total read count : 4478
aga-miR-13b read count : 4036
remaining reads : 442

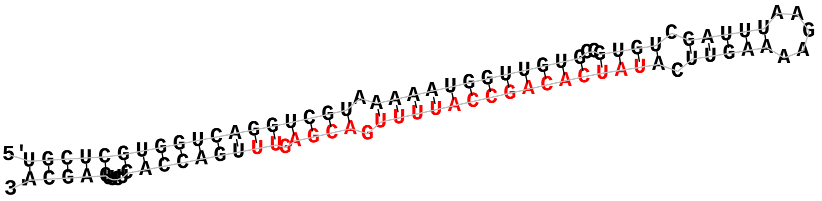

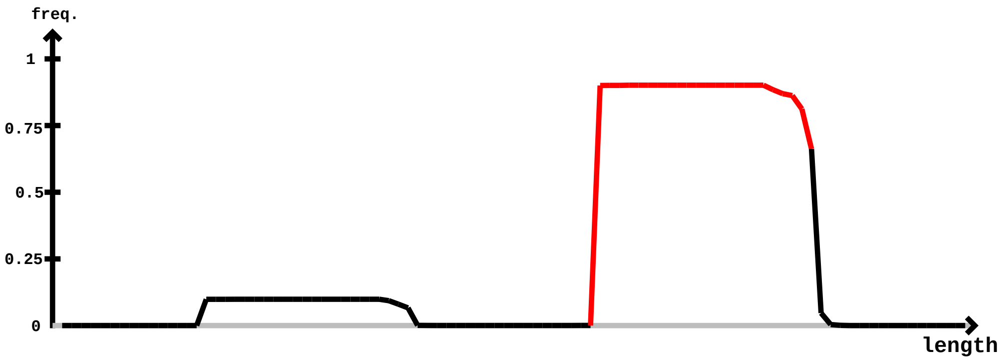

aga-miR-13b

```
5'- ugcucguggucaggucguaaaaaugguugugccgugucgauuuaagaaaaguucauaucacagccauuuugacgaguuugaccacucuugagca   -3'   exp
    .((((((((((((((((.(((((((((((..((((.(((((.....))))).))))))))))))))).)))).)))))))))....)))).    reads   mm   sample
    ..............ucguaaaaaugguugugccg............................................................    4   0   TE1
    ..............ucguaaaaaugguugugccgug..........................................................    6   0   TE1
    .......................................................uaucacagccauuuugacg....................    1   0   TE1
    .......................................................uaucacagccauuuugacgaU..................    1   1   TE1
    .......................................................uaucacagccauuuugacgag..................    6   0   TE1
    .......................................................uaucacagccauuuugacgagu.................    19   0   TE1
    .......................................................uaucacagccauuuugacgaguG................    1   1   TE1
    .......................................................uaucacagccauuuugacgaguu................    98   0   TE1
    .......................................................uaucacagccauuuugacgaguuu...............    6   0   TE1
    .......................................................uaucacagccauuuugacgaguuA...............    1   1   TE1

    ..............ucguaaaaaugguugugccg............................................................    1   0   FF2
    ..............ucguaaaaaugguugugccgu...........................................................    4   0   FF2
    ..............ucguaaaaaugguugugccgug..........................................................    10   0   FF2
    .......................................................uaucacagccauuuugac.....................    5   0   FF2
    .......................................................uaucacagccauuuugacg....................    1   0   FF2
    .......................................................uaucacagccauuuugaUgag..................    2   1   FF2
    .......................................................uaucacagccauuuugacgag..................    10   0   FF2
    .......................................................uauUacagccauuuugacgagu.................    1   1   FF2
    .......................................................uaucacagccauuuugacgagu.................    33   0   FF2
    .......................................................uaucacagccauuuugacgaguu................    112   0   FF2
    .......................................................uaucacagccaCuuugacgaguu................    1   1   FF2
    .......................................................uaucacagccauuuugacgaguuA...............    3   1   FF2
    .......................................................uaucacagccauuuugacgaguuu...............    7   0   FF2

    ..............ucguaaaaaugguugugccg............................................................    4   0   OV2
    ..............ucguaaaaaugguugugccgu...........................................................    6   0   OV2
    ..............ucguaaaaaugguugugccgug..........................................................    8   0   OV2
    ..............ucguaaaaaugguugugccguU..........................................................    1   1   OV2
    .......................................................uaucacagccauuuugacg....................    1   0   OV2
    .......................................................uaucacagccauuuugacga...................    3   0   OV2
    .......................................................uaucacagccauuuugacgag..................    9   0   OV2
    .......................................................uaucacagccauuuugaUgag..................    2   1   OV2
    .......................................................uaucacagccauuuugacgagu.................    46   0   OV2
    .......................................................uaucacagccauuuugacgagA.................    1   1   OV2
```

aga-miR-13b

```
ugcucguggucaggucguaaaaaugguugugccgugucgauuuaagaaaaguucauaucacagccauuuugacgaguuugaccacucuugagca
.......................................................uaucacagccauuuugacgaguA................   1     1   OV2
.......................................................uaucacaCccauuuugacgaguu................   1     1   OV2
.......................................................uaucacagccauuuugacgaguu................   163   0   OV2
.......................................................uaucacagccauuuugacgaguuG...............   1     1   OV2
.......................................................uaucacagccauuuugacgaguuu...............   7     0   OV2
.......................................................uaucacagccauuuugacgaguuA...............   3     1   OV2
.......................................................uaucacagccauuuugacgaguuuU..............   2     1   OV2

..............ucguaaaaaugguugugcc.............................................................   12    0   MF2
..............uUguaaaaaugguugugccg............................................................   1     1   MF2
..............ucguaaaaaugguugugccg............................................................   27    0   MF2
..............ucguaaaaaugguugugccU............................................................   5     1   MF2
..............ucguaaaaaugguugugccgu...........................................................   28    0   MF2
..............ucguaaaaaugguugugccgG...........................................................   1     1   MF2
..............ucguaaaaaugguugugccgug..........................................................   119   0   MF2
..............ucguaaaaaugguugugccguU..........................................................   2     1   MF2
..............ucguaaaaaugguugugccgugG.........................................................   1     1   MF2
..............ucguaaaaaugguugugccguguA........................................................   1     1   MF2
..............ucguaaaaaugguugugccgugucgauuuaagaaaaguuca.......................................   1     0   MF2
.................uaaaaaugguugugccgug..........................................................   1     0   MF2
.......................................................uaucacagccauuuugac.....................   18    0   MF2
.......................................................uaucacagccauuuugacg....................   28    0   MF2
.......................................................uaucacagccauuuugacA....................   1     1   MF2
.......................................................uaucacagccauuuugaUg....................   1     1   MF2
.......................................................uaucacagccauuuugacga...................   14    0   MF2
.......................................................uaucacagccauuuugaUga...................   1     1   MF2
.......................................................uaucacagccauuuugacgag..................   70    0   MF2
.......................................................uaucacagccauuuugaUgag..................   10    1   MF2
.......................................................uaucacagccauuuugaUgagu.................   1     1   MF2
.......................................................uaucaGagccauuuugacgagu.................   1     1   MF2
.......................................................uaucacagccauuuugacgagu.................   222   0   MF2
.......................................................uaucacagAcauuuugacgagu.................   1     1   MF2
.......................................................uaucacagccauuuugacgCgu.................   1     1   MF2
.......................................................uaucacagccauuuugacgagC.................   1     1   MF2
.......................................................uaucacagccauuuugGcgagu.................   1     1   MF2
.......................................................uaucacagccauuuCgacgaguu................   1     1   MF2
.......................................................uaucacagccauCuugacgaguu................   1     1   MF2
.......................................................uaucacagccCuuuugacgaguu................   1     1   MF2
.......................................................uaucacagccauuuugacgaguG................   1     1   MF2
.......................................................Caucacagccauuuugacgaguu................   2     1   MF2
.......................................................uaucacagccauuuugacgaguA................   2     1   MF2
.......................................................uaucacagccGuuuugacgaguu................   1     1   MF2
.......................................................uaucacagccauuuugacgGguu................   1     1   MF2
.......................................................uauUacagccauuuugacgaguu................   2     1   MF2
.......................................................uaucaAagccauuuugacgaguu................   1     1   MF2
.......................................................uaucacagccauuCugacgaguu................   1     1   MF2
.......................................................uaucacagccauuuugacgagCu................   1     1   MF2
.......................................................uaucacagccauuuugacgaguu................   907   0   MF2
.......................................................uaucacagccauuuugacgaguuG...............   3     1   MF2
.......................................................uaucacagccauuuugacgaguuC...............   1     1   MF2
.......................................................uaucacagccauuuugacgaguuu...............   67    0   MF2
.......................................................uaucacagccauuuugacgaguuA...............   16    1   MF2
.......................................................uaucacagccauuuugacgaguAu...............   1     1   MF2
.......................................................uaucacagccauuuugacgaguuuU..............   3     1   MF2
.......................................................uaucacagccauuuugacgaguuuA..............   2     1   MF2
.......................................................uaucacagccauuuugacgaguuug..............   1     0   MF2
.......................................................uaucacagccauuuugacgaguuuAa.............   2     1   MF2
.......................................................uaucacagccauuuugacgaguuuCa.............   2     1   MF2

..............ucguaaaGaugguugugcc.............................................................   1     1   FW2
..............ucguaaaaaugguugugccgu...........................................................   5     0   FW2
..............ucguaaaaaugguugugccgug..........................................................   11    0   FW2
.......................................................uaucacagccauuuugaU.....................   1     1   FW2
.......................................................uaucacagccauuuugac.....................   13    0   FW2
.......................................................uaucacagccauuuugacg....................   3     0   FW2
.......................................................uaucacagccauuuugaUg....................   1     1   FW2
.......................................................uaucacagccauuuugacga...................   1     0   FW2
.......................................................uaucacagccauuuugacgag..................   6     0   FW2
.......................................................uaucacagccauuuugacgagu.................   25    0   FW2
.......................................................uaucacagccauCuugacgaguu................   1     1   FW2
```

aga-miR-13b

```
ugcucguggucaggucguaaaaaugguugugccgugucgauuuaagaaaaguucauaucacagccauuuugacgaguuugaccacucuugagca
.......................................................uaucacagccauuuugacgaguC................    1     1     FW2
.......................................................uaucacagccauuuugacgaguu................    193   0     FW2
.......................................................uaucacagccauuCugacgaguu................    1     1     FW2
.......................................................uaucacagccauuuugacgaguuu...............    3     0     FW2
.......................................................uaucacagccauuuugaAgaguuu...............    1     1     FW2
.......................................................uaucacagccauuuugacgaguuA...............    3     1     FW2
.......................................................uaucacagccauuuugacgaguuuU..............    1     1     FW2
..........................................................cacagccauuuugacgagu.................    1     0     FW2

..............ucguaaaaaugguugugccg............................................................    1     0     FF1
..............ucguaaaaaugguugugccgug..........................................................    16    0     FF1
.......................................................uaucacagccauuuugac.....................    1     0     FF1
.......................................................uaucacagccauuuugacg....................    3     0     FF1
.......................................................uaucacagccauuuugacgag..................    9     0     FF1
.......................................................uaucacagccauuuugacgagG.................    1     1     FF1
.......................................................uaucacagccauuuugacgagu.................    16    0     FF1
.......................................................Caucacagccauuuugacgaguu................    1     1     FF1
.......................................................uaucacagccauuuugaAgaguu................    1     1     FF1
.......................................................uaucacagccauuuugacgaguu................    62    0     FF1
.......................................................uaucacagccauuuugacgaguuG...............    1     1     FF1
.......................................................uaucacagccauuuugacgaguuA...............    3     1     FF1
.......................................................uaucacagccauuuugacgaguuu...............    1     0     FF1
.......................................................uaucacagccauuuugacgaguuuU..............    1     1     FF1

..............ucguaaaaaugguugugcc.............................................................    4     0     OV1
..............ucguaaaaaugguugugccg............................................................    6     0     OV1
..............ucguaaaaaugguugugccgu...........................................................    2     0     OV1
..............ucguaaaaaugguugugccgug..........................................................    19    0     OV1
..............ucgGaaaaaugguugugccgug..........................................................    1     1     OV1
.......................................................uaucacagccauuuugacg....................    1     0     OV1
.......................................................uaucacagccauuuugaUg....................    1     1     OV1
.......................................................uaucacagccauuuugacga...................    1     0     OV1
.......................................................uaucacagccauuuugacgag..................    17    0     OV1
.......................................................uaucacagccauuuugacgaA..................    1     1     OV1
.......................................................uaucacagccauuuugaUgag..................    2     1     OV1
.......................................................uaucacagccauuuugacgagu.................    55    0     OV1
.......................................................uaucacagccauCuugacgaguu................    1     1     OV1
.......................................................uaucacagccauuuugacgaguu................    170   0     OV1
.......................................................uaucacagccauuuugacgaguA................    1     1     OV1
.......................................................uaucacagccauuuuAacgaguu................    1     1     OV1
.......................................................uaucacagccauuuugacgaguuu...............    7     0     OV1
.......................................................uaucacagccauuuugacgaguuA...............    5     1     OV1
.......................................................uaucacagccauuuugacgaguuuU..............    1     1     OV1
........................................................aucacagccauuuugacgag..................    1     0     OV1

..............ucguaaaaaugguugugcc.............................................................    4     0     MF1
..............ucguaaaaaugguugugccU............................................................    3     1     MF1
..............ucguaaaaaugguugugccg............................................................    2     0     MF1
..............ucguaaaaaugguugugccgu...........................................................    2     0     MF1
..............ucguaaaaaugAuugugccgug..........................................................    1     1     MF1
..............ucguaaaaaugguugugccgug..........................................................    15    0     MF1
.......................................................uaucacagccauuuugac.....................    1     0     MF1
.......................................................uaucacagccauuuugacg....................    3     0     MF1
.......................................................uaucacagccauuuugacga...................    1     0     MF1
.......................................................uaucacagccauuuugacgag..................    19    0     MF1
.......................................................uaucacagccauuuugaUgag..................    1     1     MF1
.......................................................uaucacagccauuuugacgagu.................    50    0     MF1
.......................................................uaucacagccauuuugacgaguu................    168   0     MF1
.......................................................uaucacagccaGuuugacgaguuu...............    1     1     MF1
.......................................................uaucacagccauuuugacgaguuA...............    6     1     MF1
.......................................................uaucacagccauuuugacgaguuu...............    7     0     MF1
.......................................................uaucacagccauuuugacgaguuuA..............    1     1     MF1

..............ucguaaaaaugguugugcc.............................................................    2     0     BF2
..............ucguaaaaaugguugugccgu...........................................................    3     0     BF2
..............ucguaaaaaugguuguAccgug..........................................................    1     1     BF2
..............ucguaaaaaugguugugccgug..........................................................    23    0     BF2
.......................................................uaucacagccauuuugac.....................    3     0     BF2
.......................................................uaucacagccauuuugacg....................    4     0     BF2
.......................................................uaucacagccauuuugaUg....................    1     1     BF2
```

aga-miR-13b

```
ugcucguggucaggucguaaaaaugguugugccgugucgauuuaagaaaaguucauaucacagccauuuugacgaguuugaccacucuugagca
.......................................................uaucacagccauuuugacga...................   2    0   BF2
.......................................................uaucacagccauuuugaUgag..................   6    1   BF2
.......................................................uaucacagccauuuugacgag..................   11   0   BF2
.......................................................uaucacagccauuuugacgagu.................   35   0   BF2
.......................................................uauUacagccauuuugacgaguu................   1    1   BF2
.......................................................uaucacagccauuuugacgaguu................   129  0   BF2
.......................................................uaucacagccauuuugacgaguuA...............   3    1   BF2
.......................................................uaucacagccauuuugacgaguuu...............   6    0   BF2

..............ucguaaaaaugguugugccg............................................................   1    0   BF1
..............ucguaaaaaugguugugccgug..........................................................   16   0   BF1
.......................................................uaucacagccauuuugac.....................   1    0   BF1
.......................................................uaucacagccauuuugacg....................   3    0   BF1
.......................................................uaucacagccauuuugacgag..................   9    0   BF1
.......................................................uaucacagccauuuugacgagG.................   1    1   BF1
.......................................................uaucacagccauuuugacgagu.................   16   0   BF1
.......................................................Caucacagccauuuugacgaguu................   1    1   BF1
.......................................................uaucacagccauuuugaAgaguu................   1    1   BF1
.......................................................uaucacagccauuuugacgaguu................   62   0   BF1
.......................................................uaucacagccauuuugacgaguuG...............   1    1   BF1
.......................................................uaucacagccauuuugacgaguuA...............   3    1   BF1
.......................................................uaucacagccauuuugacgaguuu...............   1    0   BF1
.......................................................uaucacagccauuuugacgaguuuU..............   1    1   BF1

..............ucguaaaaaugguugugccgu...........................................................   3    0   MW1
..............ucguaaaaaugguugugccgug..........................................................   27   0   MW1
.....................................................cauaucacagccauuuugac.....................   1    0   MW1
.......................................................uaucacagccauuuugac.....................   13   0   MW1
.......................................................uaucacagccauuuugacg....................   4    0   MW1
.......................................................uaucacagccauuuugacga...................   2    0   MW1
.......................................................uaucacagccauuuugacgag..................   5    0   MW1
.......................................................uaucacagccauuuugacgagu.................   37   0   MW1
.......................................................uaAcacagccauuuugacgaguu................   1    1   MW1
.......................................................uaucacagccauuuugacgaguu................   230  0   MW1
.......................................................uaucacagccauuuugacgaguC................   1    1   MW1
.......................................................uGucacagccauuuugacgaguu................   2    1   MW1
.......................................................uaucacagccauuuugacgaguA................   1    1   MW1
.......................................................uaucacagccauuuugacgGguu................   1    1   MW1
.......................................................uaucacagccauuuugacgaguuC...............   1    1   MW1
.......................................................uaucacagccauuuugacgaguuu...............   1    0   MW1
.......................................................uaucacagccauuuugacgaguuA...............   5    1   MW1

..............ucguaaaaaugguugugccg............................................................   2    0   FW1
..............ucguaaaaaugguugugccgu...........................................................   3    0   FW1
..............ucguaaaaaugguugugccgug..........................................................   9    0   FW1
.......................................................uaucacagccauuuugac.....................   11   0   FW1
.......................................................uaucacagccauuuugacg....................   4    0   FW1
.......................................................uaucacagccauuuugacga...................   1    0   FW1
.......................................................uaucacagccauuuugaUga...................   1    1   FW1
.......................................................uaucacagccauuuugaUgag..................   2    1   FW1
.......................................................uaucacagccauuuugacgagu.................   19   0   FW1
.......................................................uaucacagccauuuugacgaguu................   122  0   FW1
.......................................................uaucacagccauuuugacgaguuG...............   1    1   FW1
.......................................................uaucacagccauuuugacgaguuA...............   4    1   FW1
........................................................aucacagccauuuugacgaguu................   1    0   FW1
..........................................................cacagccauuuugacgaguu................   2    0   FW1

..............ucguaaaaaugguugugccgu...........................................................   2    0   MW2
..............ucguaaaaaugguugugccgug..........................................................   1    0   MW2
.......................................................uaucacagccauuuugac.....................   6    0   MW2
.......................................................uaucacagccauuuugacg....................   1    0   MW2
.......................................................uaucacagccauuuugacgag..................   5    0   MW2
.......................................................uaucacagccauuuugacgagu.................   15   0   MW2
.......................................................uauAacagccauuuugacgaguu................   1    1   MW2
.......................................................uaucacagccauuuugacgaguu................   85   0   MW2
.......................................................uaucacagccauuuugacgaguuA...............   1    1   MW2

..............ucguaaaaaugguugugcc.............................................................   1    0   TE2
..............ucguaaaaaugguugugccU............................................................   1    1   TE2
..............ucguaaaaaugguugugccg............................................................   1    0   TE2
```

aga-miR-13b

```
ugcucguggucaggucguaaaaaugguugugccgugucgauuuaagaaaaguucauaucacagccauuuugacgaguuugaccacucuugagca
..............ucguaaaaaugguugugccgu...........................................................    3      0      TE2
..............ucguaaaaaugguugugccgug..........................................................    7      0      TE2
.......................................................uaucacagccauuuugac.....................    2      0      TE2
.......................................................uaucacagccauuuugacg....................    2      0      TE2
.......................................................uaucacagccauuuugaUg....................    2      1      TE2
.......................................................uaucacagccauuuugacga...................    5      0      TE2
.......................................................uaucacagccauuuugaUgag..................    1      1      TE2
.......................................................uaucacagccauuuugacgag..................    21     0      TE2
.......................................................uaucacagccauuuugacgagu.................    68     0      TE2
.......................................................uaCcacagccauuuugacgagu.................    1      1      TE2
.......................................................uaucacagccauuuugacgagG.................    2      1      TE2
.......................................................uaucacagccauuuCgacgaguu................    1      1      TE2
.......................................................uaucacagccaCuuugacgaguu................    1      1      TE2
.......................................................uaucacagccauuuugacgaguu................    207    0      TE2
.......................................................uauUacagccauuuugacgaguu................    1      1      TE2
.......................................................uaucacagccauuuugacgagGu................    2      1      TE2
.......................................................uaucacagccauuuugacgaguA................    2      1      TE2
.......................................................uaucacagccauuuugacAaguu................    1      1      TE2
.......................................................uaucacagccauuuugacgaguuA...............    5      1      TE2
.......................................................uaucacagccauuuugacgaguuu...............    7      0      TE2
```

miRBase precursor : aga-mir-14
Total read count : 55928
aga-miR-14 read count : 55860
aga-miR-14* read count : 33
remaining reads : 35

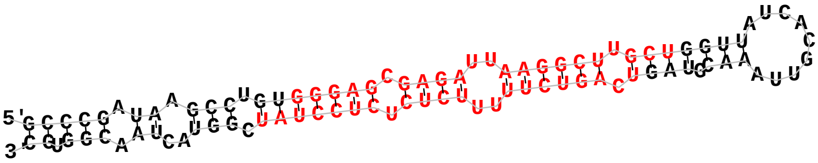

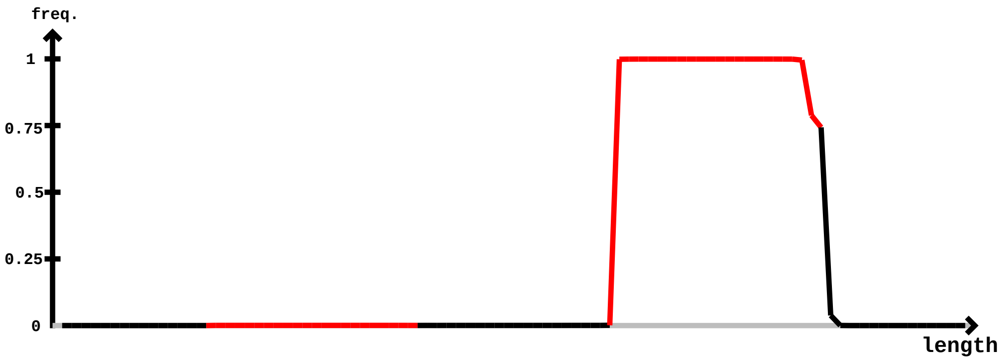

aga-miR-14*

aga-miR-14

```
5'- gcccgauaagccugugggagcgagauuaaggcuugcugguuaucacguuaaacguagucagucuuuuucucucuccuaucgguacuaacggugc   -3'    exp
    (((((.((.(((.(((((((.((((..((((((.(((((((.........))).)))).))))))..)))).))))))).)))..)).))).))    reads    mm    sample
    ........................................uaucacguuaaacguagucagucu..............................    1        0     TE1
    ........................................................Cucagucuuuuucucucuccu.................    1        1     TE1
    ........................................................gucagucuuuuucucucuccua................    1        0     TE1
    ........................................................Aucagucuuuuucucucuccuau...............    1        1     TE1
    ........................................................Cucagucuuuuucucucuccuau...............    1        1     TE1
    .........................................................ucagucuuuuucucucucc..................    12       0     TE1
    .........................................................ucagucCuuuucucucuccu.................    1        1     TE1
    .........................................................ucagucuuuuucucCcuccu.................    1        1     TE1
    .........................................................Ccagucuuuuucucucuccu.................    1        1     TE1
    .........................................................ucagucuuuuucucucuccG.................    1        1     TE1
    .........................................................ucagucuuuuucucucuccu.................    592      0     TE1
    .........................................................ucagucuuuuucucucCccu.................    1        1     TE1
    .........................................................ucagucuuuuucuUucuccu.................    1        1     TE1
    .........................................................ucagucuuuuucucucuccua................    167      0     TE1
    .........................................................Ccagucuuuuucucucuccua................    1        1     TE1
    .........................................................ucagucCuuuucucucuccua................    2        1     TE1
    .........................................................ucagucuuuuuGucucuccuau...............    1        1     TE1
    .........................................................ucaguAuuuuucucucuccuau...............    1        1     TE1
    .........................................................ucagCcuuuuucucucuccuau...............    1        1     TE1
    .........................................................ucagucuuuuucuGucuccuau...............    1        1     TE1
    .........................................................ucagucuuuuucucucuccuaC...............    10       1     TE1
    .........................................................Ccagucuuuuucucucuccuau...............    1        1     TE1
    .........................................................ucagucuuuuuAucucuccuau...............    1        1     TE1
    .........................................................ucagucuuuuucucucuccuau...............    2853     0     TE1
    .........................................................ucagucuuuuucucucucAuau...............    1        1     TE1
    .........................................................ucagAcuuuuucucucuccuau...............    1        1     TE1
    .........................................................ucagucuuuuucucucuccuaG...............    5        1     TE1
    .........................................................ucagucCuuuucucucuccuau...............    3        1     TE1
    .........................................................ucagucuuuuucucucuccuaA...............    4        1     TE1
    .........................................................ucaguUuuuuucucucuccuau...............    3        1     TE1
    .........................................................uAagucuuuuucucucuccuau...............    1        1     TE1
    .........................................................ucagucuCuuucucucuccuau...............    2        1     TE1
    .........................................................ucagucuuuuuUucucuccuau...............    3        1     TE1
    .........................................................ucagucuuuCucucucuccuau...............    1        1     TE1
```

```
               aga-miR-14*
                                                         aga-miR-14
gcccgauaagccugugggagcgagauuaaggcuugcugguuaucacguuaaacguagucagucuuuuucucucuccuaucgguacuaacggugc

.........................................................ucaUucuuuuucucucuccuau...............   1      1      TE1
.........................................................Acagucuuuuucucucuccuau...............   1      1      TE1
.........................................................ucagGcuuuuucucucuccuau...............   1      1      TE1
.........................................................ucagucuuuuucucuGuccuau...............   1      1      TE1
.........................................................uUagucuuuuucucucuccuau...............   1      1      TE1
.........................................................ucagucuuuuucCcucuccuau...............   1      1      TE1
.........................................................ucagucuuuuucucucuccuGu...............   1      1      TE1
.........................................................ucagucuuuuucucCcuccuau...............   2      1      TE1
.........................................................ucagucuuuuucucucuccuauA..............   64     1      TE1
.........................................................ucagucuuuuucucucuccuauU..............   101    1      TE1
.........................................................ucagucuuuuucucucuccuauc..............   1      0      TE1
.........................................................ucagucuuuuucucucuccuauG..............   1      1      TE1
.........................................................ucagucuuuuucucucuccuaucA.............   1      1      TE1
.........................................................ucagucuuuuucucucuccuaucU.............   1      1      TE1
..........................................................cagucuuuuucucucuccuau...............   2      0      TE1
..........................................................cagucuuuuucucucuccuauc..............   1      0      TE1

...............gggagcgagauuaaggcuugc..........................................................   1      0      OV2
...............gggagcgagauuaaggcuugcu.........................................................   1      0      OV2
.....................................gguuaucacguuaaacguagucagucu..............................   1      0      OV2
.....................................gguuaucacguuaaacguagucagucuuuuucucucuccuau...............   1      0      OV2
........................................................Cucagucuuuuucucucuccu.................   2      1      OV2
........................................................Cucagucuuuuucucucuccuau...............   1      1      OV2
.........................................................ucagucuuuuucucucuU...................   1      1      OV2
.........................................................ucagucuuuuucucucuc...................   1      0      OV2
.........................................................ucagucuuuuucucucucc..................   19     0      OV2
.........................................................ucagucuuuuucucucAccu.................   1      1      OV2
.........................................................ucagucuuuuuGucucuccu.................   1      1      OV2
.........................................................ucaAucuuuuucucucuccu.................   1      1      OV2
.........................................................ucagucuuuuuUucucuccu.................   1      1      OV2
.........................................................uUagucuuuuucucucuccu.................   1      1      OV2
.........................................................ucagucuuuuucCcucuccu.................   2      1      OV2
.........................................................ucagucuuuuucucucuccu.................   1467   0      OV2
.........................................................ucagucuuuuucucuGuccu.................   1      1      OV2
.........................................................ucagucuuuuucucucuccG.................   1      1      OV2
.........................................................ucaguAuuuuucucucuccu.................   1      1      OV2
.........................................................ucagucuuuuucucucuccC.................   1      1      OV2
.........................................................ucagucCuuuucucucuccu.................   2      1      OV2
.........................................................ucagucuCuuucucucuccu.................   1      1      OV2
.........................................................ucagucuuuuucucucuccua................   245    0      OV2
.........................................................ucagucuuuuucucucuccuG................   1      1      OV2
.........................................................ucagucuuuuucucucuccuU................   2      1      OV2
.........................................................ucagucuuuuucucCcuccua................   2      1      OV2
.........................................................ucagucuuuuuAucucuccua................   2      1      OV2
.........................................................ucagucCuuuucucucuccuau...............   5      1      OV2
.........................................................ucagucuuuuuAucucuccuau...............   1      1      OV2
.........................................................ucagucuuuCucucucuccuau...............   1      1      OV2
.........................................................ucagucuuuuucucucuGcuau...............   1      1      OV2
.........................................................ucagucuuuuuGucucuccuau...............   1      1      OV2
.........................................................ucagucuuuuucucucuccuaC...............   13     1      OV2
.........................................................ucUgucuuuuucucucuccuau...............   1      1      OV2
.........................................................ucagucuuuuucucucucAuau...............   1      1      OV2
.........................................................uUagucuuuuucucucuccuau...............   2      1      OV2
.........................................................ucagucuuuuucucucuccuaA...............   4      1      OV2
.........................................................ucagucuuuuAcucucuccuau...............   1      1      OV2
.........................................................ucagucuuuuucucucuccCau...............   4      1      OV2
.........................................................ucagucuuuuucucucuccuau...............   2480   0      OV2
.........................................................ucagucuuuuucucucuccuaG...............   1      1      OV2
.........................................................ucagucuuuuucucucCccuau...............   1      1      OV2
.........................................................ucagucuuuuucucucuccuauA..............   54     1      OV2
.........................................................ucagucuuuuucucucuccuauc..............   3      0      OV2
.........................................................ucagucuuuuucucucuccuauU..............   76     1      OV2
.........................................................ucagucuuuuucucucuccuaucU.............   1      1      OV2
..........................................................cagucuuuuucucucuccu.................   1      0      OV2
..........................................................cagucuuuuucucucuccuauU..............   2      1      OV2
...........................................................agucuuuuucucucuccu.................   2      0      OV2
...........................................................agucuuuuucucucuccuau...............   1      0      OV2

...............gggagcgagauuaaggcuugc..........................................................   1      0      FF2
```

```
                aga-miR-14*
                                          aga-miR-14
gcccgauaagccugugggagcgagauuaaggcuugcugguuaucacguuaaacguagucagucuuuuucucucuccuaucgguacuaacggugc

........................................................gucagucuuuuucucucuccua................    1      0      FF2
.........................................................ucagucuuuuucucucucc..................    1      0      FF2
.........................................................ucagucuuuuucAcucuccu.................    1      1      FF2
.........................................................ucagucuuuuuGucucuccu.................    1      1      FF2
.........................................................ucagucCuuuucucucuccu.................    1      1      FF2
.........................................................ucagucuuuuucucucuccu.................    181    0      FF2
.........................................................ucagucuuuuuAucucuccu.................    1      1      FF2
.........................................................ucagucuuuuCcucucuccu.................    1      1      FF2
.........................................................ucagucuuuuucucucuccua................    43     0      FF2
.........................................................ucagucuuuuucucucCccuau...............    1      1      FF2
.........................................................ucagucuuuuucucucuccuau...............    1075   0      FF2
.........................................................ucagucCuuuucucucuccuau...............    2      1      FF2
.........................................................ucagucuuuuuGucucuccuau...............    2      1      FF2
.........................................................ucagucuuuuucucucuccuaC...............    5      1      FF2
.........................................................ucagucuuuuucucucuccAau...............    1      1      FF2
.........................................................ucagCcuuuuucucucuccuau...............    2      1      FF2
.........................................................ucagucuuuuucucucuccuaG...............    2      1      FF2
.........................................................ucagucuuuAucucucuccuau...............    1      1      FF2
.........................................................ucagucuuuuucucucucGuau...............    1      1      FF2
.........................................................ucagucuuuuucucucuccCau...............    1      1      FF2
.........................................................ucagucuuuuucucucuccuauU..............    28     1      FF2
.........................................................ucagucuuuuucucucuccuauc..............    5      0      FF2
.........................................................ucagucuuuuucucucuccuauA..............    22     1      FF2
.........................................................ucagucuuuuucucucuccuaucU.............    2      1      FF2
...........................................................agucuuuuucucucuccuau...............    1      0      FF2

...............gggagcgagauuaaggcuug...........................................................    1      0      MF2
...............gggagcgagauuaaggcuugcu.........................................................    2      0      MF2
....................cgagauuaaggcuugcuU........................................................    1      1      MF2
.....................................gguuaucacguuaaacguagucagucuuuuucucucuccuau...............    8      0      MF2
.........................................aucacguuaaacguagucagucuu.............................    1      0      MF2
....................................................cguGgucagucuuuuucucucuccuau...............    1      1      MF2
........................................................gucagucuuuuucucucuccua................    2      0      MF2
........................................................Cucagucuuuuucucucuccua................    1      1      MF2
........................................................Cucagucuuuuucucucuccuau...............    3      1      MF2
........................................................gucagucuuuuucucucuccuau...............    1      0      MF2
.........................................................ucagucuuuuucucucucc..................    27     0      MF2
.........................................................ucagucuuuuucucucucU..................    1      1      MF2
.........................................................ucagucuuuuucucucuccu.................    1431   0      MF2
.........................................................ucagucuuuuucucuUuccu.................    1      1      MF2
.........................................................ucagucuuCuucucucuccu.................    1      1      MF2
.........................................................Ccagucuuuuucucucuccu.................    1      1      MF2
.........................................................ucagucuuuuCcucucuccu.................    2      1      MF2
.........................................................ucagucuuuuucucuAuccu.................    1      1      MF2
.........................................................uUagucuuuuucucucuccu.................    2      1      MF2
.........................................................ucagucuuuuuUucucuccu.................    1      1      MF2
.........................................................ucagucuuuuucucucuccG.................    2      1      MF2
.........................................................ucaUucuuuuucucucuccu.................    1      1      MF2
.........................................................ucagucuuuuuAucucuccu.................    1      1      MF2
.........................................................ucagucuCuuucucucuccu.................    1      1      MF2
.........................................................ucagucuuuAucucucuccu.................    1      1      MF2
.........................................................ucagucCuuuucucucuccu.................    3      1      MF2
.........................................................ucagCcuuuuucucucuccu.................    2      1      MF2
.........................................................ucagucuuuuucucucuccC.................    3      1      MF2
.........................................................ucagucuuuuucucucuUcua................    1      1      MF2
.........................................................ucagucuuuuGcucucuccua................    2      1      MF2
.........................................................ucagucCuuuucucucuccua................    3      1      MF2
.........................................................ucagucuuuuucucucuccua................    607    0      MF2
.........................................................ucagucuuuuucucuUuccua................    2      1      MF2
.........................................................ucagucuuuuucucucuccuU................    3      1      MF2
.........................................................ucagucuuuAucucucuccua................    1      1      MF2
.........................................................ucagucuuuuucucucuccuUu...............    3      1      MF2
.........................................................ucagucuuuuucucucucGuau...............    1      1      MF2
.........................................................ucagCcuuuuucucucuccuau...............    3      1      MF2
.........................................................ucagucuuuuucucucuAcuau...............    1      1      MF2
.........................................................ucagucuuuuucucucuccGau...............    1      1      MF2
.........................................................ucagucuuuuucucAcuccuau...............    2      1      MF2
.........................................................ucagucuuuuucucucuccAau...............    1      1      MF2
.........................................................ucagucuuuuucucucuccuaC...............    61     1      MF2
```

```
               aga-miR-14*
                                          aga-miR-14
gcccgauaagccugugggagcgagauuaaggcuugcugguuaucacguuaaacguagucagucuuuuucucucuccuaucgguacuaacggugc

.........................................................ucagucuuuuucucuAuccuau...............    1      1    MF2
.........................................................ucagucuuuuucucucuccuaA...............    22     1    MF2
.........................................................ucagucuuuuucucucAuau...............    1      1    MF2
.........................................................ucagucuuuAucucucuccuau...............    5      1    MF2
.........................................................ucagucuuuuucucucucUuau...............    1      1    MF2
.........................................................ucagGcuuuuucucucuccuau...............    1      1    MF2
.........................................................ucagucuuuCucucucuccuau...............    3      1    MF2
.........................................................ucagucuuAuucucucuccuau...............    1      1    MF2
.........................................................ucagucuuuuucucucGccuau...............    1      1    MF2
.........................................................ucagucuuCuucucucuccuau...............    4      1    MF2
.........................................................ucGgucuuuuucucucuccuau...............    1      1    MF2
.........................................................ucagucuuuuucucucuccuCu...............    1      1    MF2
.........................................................ucagucuuuuucucucuccuaG...............    13     1    MF2
.........................................................ucagucuuuuucucucAccuau...............    3      1    MF2
.........................................................ucaguAuuuuucucucuccuau...............    1      1    MF2
.........................................................ucagucuuuuuGucucuccuau...............    5      1    MF2
.........................................................ucagucuuuuucucucuUcuau...............    2      1    MF2
.........................................................ucaguGuuuuucucucuccuau...............    1      1    MF2
.........................................................ucagucAuuuucucucuccuau...............    1      1    MF2
.........................................................uUagucuuuuucucucuccuau...............    6      1    MF2
.........................................................Ccagucuuuuucucucuccuau...............    4      1    MF2
.........................................................ucagucuuuuuCcucuccuau...............    3      1    MF2
.........................................................uGagucuuuuucucucuccuau...............    1      1    MF2
.........................................................ucagucuuuuucucucuGcuau...............    1      1    MF2
.........................................................ucagucuuuuuUucucuccuau...............    4      1    MF2
.........................................................ucagucuuuuucuUucuccuau...............    4      1    MF2
.........................................................ucagAcuuuuucucucuccuau...............    1      1    MF2
.........................................................ucagucuuuuucucucuccCau...............    8      1    MF2
.........................................................ucagucCuuuucucucuccuau...............    15     1    MF2
.........................................................ucagucuuuuuAucucuccuau...............    8      1    MF2
.........................................................ucagucuuuuucucuGuccuau...............    2      1    MF2
.........................................................ucagucuuuuucucucCccuau...............    2      1    MF2
.........................................................ucaguUuuuuucucucuccuau...............    3      1    MF2
.........................................................ucagucuuuuucucucuccuGu...............    4      1    MF2
.........................................................ucagucuuuuucucucuccuau...............    13743  0    MF2
.........................................................ucagucuuuuucucuUuccuau...............    7      1    MF2
.........................................................ucUgucuuuuucucucuccuau...............    2      1    MF2
.........................................................ucagucuuuuucucucuccuauA..............    214    1    MF2
.........................................................ucagucuuuuucucucuccuauG..............    1      1    MF2
.........................................................ucagucuuuuucucucuccuauc..............    16     0    MF2
.........................................................ucagucuuuuucucucuccuauU..............    563    1    MF2
.........................................................ucagucuuuuucucucuccuaucU.............    5      1    MF2
..........................................................cagucuuuuucucucuccuau...............    2      0    MF2
..........................................................cagucuuuuucucucuccuauA..............    2      1    MF2
..........................................................cagucuuuuucucucuccuauc..............    1      0    MF2
...........................................................agucuuuuucucucuccu.................    1      0    MF2
...........................................................agucuuuuucucucuccuauU..............    1      1    MF2

...............gggagcgGgauuaaggcuugcu.........................................................    1      1    FW2
...............gggagcgagauuaaggcuugcu.........................................................    4      0    FW2
....................cgagauuaaggcuugcu.........................................................    1      0    FW2
.........................................................ucagucuuuuucucucu....................    1      0    FW2
.........................................................ucagucuuuuucucucuc...................    2      0    FW2
.........................................................ucagucuuuuucucucuccu.................    134    0    FW2
.........................................................uUagucuuuuucucucuccu.................    1      1    FW2
.........................................................ucagucCuuuucucucuccu.................    1      1    FW2
.........................................................ucagucuuuCucucucuccu.................    1      1    FW2
.........................................................ucagucuuuuucucucuccua................    28     0    FW2
.........................................................ucaguUuuuuucucucuccuau...............    1      1    FW2
.........................................................ucagucuuuuucucucuccuau...............    594    0    FW2
.........................................................ucagucuuuuuAucucuccuau...............    1      1    FW2
.........................................................ucagucuuuuuUucucuccuau...............    1      1    FW2
.........................................................ucagucuuuuAcucucuccuau...............    1      1    FW2
.........................................................ucagucuuuuucucucuccuaA...............    2      1    FW2
.........................................................uUagucuuuuucucucuccuau...............    1      1    FW2
.........................................................ucagucuuuuucuGucuccuau...............    1      1    FW2
.........................................................ucagucCuuuucucucuccuau...............    1      1    FW2
.........................................................ucagucAuuuucucucuccuau...............    1      1    FW2
.........................................................ucagucuuuuucucucuccuauU..............    17     1    FW2
```

```
               aga-miR-14*
                                                         aga-miR-14
gcccgauaagccugugggagcgagauuaaggcuugcugguuaucacguuaaacguagucagucuuuuucucucuccuaucgguacuaacggugc

.........................................................ucagucuuuuucucucuccuauA..............    7      1      FW2
.........................................................ucagucuuuuucucucuccuauGgg............    1      1      FW2
...........................................................agucuuuuucucucuccuau...............    1      0      FW2

...............gggagcgagauuaaggcuugcu.........................................................    1      0      FF1
.....................................gguuaucacguuaaacg........................................    1      0      FF1
.....................................gguuaucacguuaaacguagucagucuuuuucucucuccuau...............    2      0      FF1
.....................................gguuaucacguuaaacguagucagucuuCuucucucuccuau...............    1      1      FF1
........................................................gucagucuuuuucucucuccuau...............    1      0      FF1
.........................................................ucagucuuuuucucucucc..................    3      0      FF1
.........................................................ucagucuuuuucucucuUcu.................    1      1      FF1
.........................................................ucagucuuuuucucucuccu.................    263    0      FF1
.........................................................ucagucuuuuucucucuccua................    73     0      FF1
.........................................................ucagucuuuuucucucuccuaA...............    1      1      FF1
.........................................................ucagucuuuuucucucuccuau...............    1447   0      FF1
.........................................................ucagucuuuuuGucucuccuau...............    3      1      FF1
.........................................................ucagucuuuuuUucucuccuau...............    1      1      FF1
.........................................................ucagucuuuuCcucucuccuau...............    1      1      FF1
.........................................................ucagucuuuuucucucuccuaC...............    2      1      FF1
.........................................................ucagucuuuuucucucuccCau...............    1      1      FF1
.........................................................ucagucuuuuucucucucUuau...............    1      1      FF1
.........................................................uUagucuuuuucucucuccuau...............    1      1      FF1
.........................................................ucagucuuuuucuUucuccuau...............    1      1      FF1
.........................................................ucaguUuuuuucucucuccuau...............    1      1      FF1
.........................................................ucUgucuuuuucucucuccuau...............    1      1      FF1
.........................................................ucagucuuuuucucucuccuUu...............    1      1      FF1
.........................................................ucagucCuuuucucucuccuau...............    1      1      FF1
.........................................................ucagucuuuuucucuAuccuau...............    1      1      FF1
.........................................................ucagucuuuuucCcucuccuau...............    2      1      FF1
.........................................................ucagucuuuuucucucuccuauG..............    1      1      FF1
.........................................................ucagucuuuuucucucuccAauc..............    1      1      FF1
.........................................................ucagucuuuuucucucuccuauU..............    39     1      FF1
.........................................................ucagucuuuuucucucuccuauA..............    30     1      FF1
..........................................................cagucuuuuucucucuccua................    1      0      FF1
..........................................................cagucuuuuucucucuccuaucg.............    1      0      FF1
...........................................................agucuuuuucucucuccua................    1      0      FF1

...............gggagcgagauuaaggcuug...........................................................    1      0      OV1
...............gggagcgagauuaaggcuugc..........................................................    1      0      OV1
.....................................gguuaucacguuaaacguagucagucuuuuucucucuccuau...............    1      0      OV1
....................................................cguGgucagucuuuuucucucuccuau...............    1      1      OV1
........................................................Cucagucuuuuucucucuccu.................    1      1      OV1
........................................................gucagucuuuuucucucuccu.................    1      0      OV1
........................................................Cucagucuuuuucucucuccuau...............    1      1      OV1
.........................................................ucagucuuuuucucucuc...................    3      0      OV1
.........................................................ucagucuuuuucucucucU..................    1      1      OV1
.........................................................ucagucuuuuucucucucc..................    45     0      OV1
.........................................................ucagucuuuuuUucucucc..................    1      1      OV1
.........................................................ucagucuuuuucucucCccu.................    2      1      OV1
.........................................................ucagucuuuuuUucucuccu.................    2      1      OV1
.........................................................ucaguUuuuuucucucuccu.................    1      1      OV1
.........................................................ucagucuuuuucuUucuccu.................    2      1      OV1
.........................................................ucagAcuuuuucucucuccu.................    1      1      OV1
.........................................................ucagucuuuuucuGucuccu.................    2      1      OV1
.........................................................ucagucCuuuucucucuccu.................    11     1      OV1
.........................................................ucagucuuuuucucucucAu.................    1      1      OV1
.........................................................ucUgucuuuuucucucuccu.................    1      1      OV1
.........................................................ucagucuuuuucucucuccG.................    5      1      OV1
.........................................................ucagucuuuuucucCcuccu.................    3      1      OV1
.........................................................ucagucuuuuucCcucuccu.................    2      1      OV1
.........................................................ucagucuuuuucucuUuccu.................    1      1      OV1
.........................................................ucagucuuuuucucucuAcu.................    1      1      OV1
.........................................................ucagucuuuAucucucuccu.................    1      1      OV1
.........................................................ucagucuuuuucucucuccu.................    3190   0      OV1
.........................................................ucagucuuuuuAucucuccu.................    1      1      OV1
.........................................................ucagucuuuuucAcucuccu.................    1      1      OV1
.........................................................ucagucuuuuucucucuUcu.................    3      1      OV1
.........................................................ucagucuuuuucucAcuccu.................    1      1      OV1
.........................................................ucagucuuuuucucucuccC.................    3      1      OV1
```

```
               aga-miR-14*
                                                         aga-miR-14
gcccgauaagccugugggagcgagauuaaggcuugcugguuaucacguuaaacguagucagucuuuuucucucuccuaucgguacuaacggugc

.........................................................ucagucuuuuuGucucuccu..................   2      1      OV1
.........................................................uUagucuuuuucucucuccu.................   4      1      OV1
.........................................................ucagucuCuuucucucuccu.................   2      1      OV1
.........................................................ucagucuuuuucucucuccua................   503    0      OV1
.........................................................ucagucuuuuucucucuccuU................   1      1      OV1
.........................................................ucagucuuuuuAucucuccua................   1      1      OV1
.........................................................ucagucCuuuucucucuccua................   3      1      OV1
.........................................................ucagucuuuuCcucucuccua................   1      1      OV1
.........................................................ucagucuuuuucucucAccua................   1      1      OV1
.........................................................ucagucuuuGucucucuccuau...............   1      1      OV1
.........................................................ucagucuuuuucucucuUcuau...............   1      1      OV1
.........................................................ucagucuuuuucucucuccuaG...............   2      1      OV1
.........................................................ucaAucuuuuucucucuccuau...............   1      1      OV1
.........................................................ucagucuuuuucucuUuccuau...............   1      1      OV1
.........................................................ucagucuuuuucucucuccuCu...............   1      1      OV1
.........................................................ucagucuuuuucucucuccuUu...............   1      1      OV1
.........................................................ucagucuuuuucuGucuccuau...............   1      1      OV1
.........................................................ucagucuuuuucucucuccCau...............   3      1      OV1
.........................................................ucagucuuuuucucucuccuau...............   4307   0      OV1
.........................................................ucaUucuuuuucucucuccuau...............   2      1      OV1
.........................................................uUagucuuuuucucucuccuau...............   4      1      OV1
.........................................................ucagucuuuuCcucucuccuau...............   2      1      OV1
.........................................................ucagucCuuuucucucuccuau...............   8      1      OV1
.........................................................ucGgucuuuuucucucuccuau...............   1      1      OV1
.........................................................ucagucuuCuucucucuccuau...............   1      1      OV1
.........................................................ucagucuuuuuAucucuccuau...............   3      1      OV1
.........................................................ucagucuuuuucuAucuccuau...............   1      1      OV1
.........................................................ucagucuuuuuGucucuccuau...............   1      1      OV1
.........................................................ucagucuuuuucucucuccuaC...............   19     1      OV1
.........................................................ucagucuuuuucucAcuccuau...............   1      1      OV1
.........................................................ucagucuuuuucucucCccuau...............   2      1      OV1
.........................................................ucagCcuuuuucucucuccuau...............   1      1      OV1
.........................................................Acagucuuuuucucucuccuau...............   1      1      OV1
.........................................................Ccagucuuuuucucucuccuau...............   2      1      OV1
.........................................................ucagucuuuuuUucucuccuau...............   3      1      OV1
.........................................................ucagucuuuuucucucAccuau...............   1      1      OV1
.........................................................ucagucuuuCucucucuccuau...............   2      1      OV1
.........................................................ucagucuuuuucucucuccuaA...............   7      1      OV1
.........................................................ucagucuuuuucucuAuccuau...............   1      1      OV1
.........................................................ucUgucuuuuucucucuccuau...............   1      1      OV1
.........................................................ucagucuuuuucucucuccuauc..............   5      0      OV1
.........................................................ucagucuuuuucucucuccuauA..............   79     1      OV1
.........................................................ucagucuuuuucucucuccuauG..............   1      1      OV1
.........................................................ucagucuuuuucucucuccuauU..............   163    1      OV1
.........................................................ucagucuuuuucucucuccuaucU.............   2      1      OV1
..........................................................cagucuuuuucucucuccuau...............   2      0      OV1
..................................................................uucucucuccuaucgguacuaacgg...   1      0      OV1

...............gggagcgagauuaaggcuugc..........................................................   2      0      MF1
...............gggagcgagauuaaggcuugcu.........................................................   2      0      MF1
....................cgagauuaaggcuugcu.........................................................   1      0      MF1
.....................................gguuaucacguAaaacguagucagucuuuuucucucuccuau...............   1      1      MF1
.......................................uuaucacguuaaacguagucagucuuuuucucucuccua................   1      0      MF1
........................................................Aucagucuuuuucucucuccuau...............   1      1      MF1
.........................................................ucagucuuuuucucucu....................   1      0      MF1
.........................................................ucagucuuuuucucucucc..................   11     0      MF1
.........................................................ucagucCuuuucucucuccu.................   1      1      MF1
.........................................................ucaAucuuuuucucucuccu.................   1      1      MF1
.........................................................ucagucuuuuucucucuccu.................   570    0      MF1
.........................................................ucagucuuuuucucucuccG.................   6      1      MF1
.........................................................uUagucuuuuucucucuccu.................   1      1      MF1
.........................................................ucagucuCuuucucucuccu.................   1      1      MF1
.........................................................ucagucuuuuucucucuUcu.................   1      1      MF1
.........................................................Ccagucuuuuucucucuccua................   1      1      MF1
.........................................................ucagucCuuuucucucuccua................   1      1      MF1
.........................................................ucagucuuuuucucucuccua................   121    0      MF1
.........................................................ucagucuuuuucucucAccua................   1      1      MF1
.........................................................ucagucuuuuucucucuUcuau...............   1      1      MF1
.........................................................ucagucuuuuucucucCccuau...............   3      1      MF1
```

```
               aga-miR-14*
                                                         aga-miR-14
gcccgauaagccugugggagcgagauuaaggcuugcugguuaucacguuaaacguagucagucuuuuucucucuccuaucgguacuaacggugc

.........................................................ucagucuuuuucucucuccuGu...............    1       1       MF1
.........................................................ucagAcuuuuucucucuccuau...............    1       1       MF1
.........................................................ucagucuuuuucucucuccuaA...............    7       1       MF1
.........................................................ucagucuuuuucucucuccuaC...............    10      1       MF1
.........................................................ucagucuuCuucucucuccuau...............    1       1       MF1
.........................................................ucagucuuuuuUucucuccuau...............    1       1       MF1
.........................................................ucagucuuuuucucucuccuaG...............    1       1       MF1
.........................................................ucagucAuuuucucucuccuau...............    1       1       MF1
.........................................................ucagucuuuuucucucuccuau...............    1518    0       MF1
.........................................................ucagucuuuuucucucuAcuau...............    2       1       MF1
.........................................................ucagucuuuuucCcucuccuau...............    1       1       MF1
.........................................................Ccagucuuuuucucucuccuau...............    2       1       MF1
.........................................................ucagucuuuuuGucucuccuau...............    1       1       MF1
.........................................................ucagucuuuuuAucucuccuau...............    1       1       MF1
.........................................................ucagucuuuuucucucuccuauA..............    14      1       MF1
.........................................................ucagucuuuuucucucuccuauU..............    57      1       MF1
.........................................................ucagucuuuuucucucuccuaucU.............    1       1       MF1
..........................................................cagucuuuuucucucuccuau...............    1       0       MF1

...............gggagcgagauuaaggcuug...........................................................    1       0       BF2
...............gggagcgagauuaaggcuugc..........................................................    3       0       BF2
...............gggagcgagauuaaggcuugcu.........................................................    1       0       BF2
.....................................gguuaucacguuaaacguagucagucuuuuucucucuccuau...............    4       0       BF2
....................................................cguGgucagucuuuuucucucuccu.................    1       1       BF2
........................................................gucagucuuuuucucucuccu.................    1       0       BF2
........................................................Cucagucuuuuucucucuccuau...............    2       1       BF2
........................................................gucagucuuuuucucucuccuau...............    1       0       BF2
.........................................................ucagucuuuuucucucu....................    1       0       BF2
.........................................................ucagucuuuuucucucucc..................    22      0       BF2
.........................................................ucagucuuuuucucucuccG.................    3       1       BF2
.........................................................ucagucuuuuucucucuccC.................    2       1       BF2
.........................................................uUagucuuuuucucucuccu.................    2       1       BF2
.........................................................ucagucuuuuucucucuccu.................    835     0       BF2
.........................................................ucagucCuuuucucucuccu.................    1       1       BF2
.........................................................ucagucuuuuucucuUuccu.................    3       1       BF2
.........................................................ucagucuuuuucucucuccCa................    1       1       BF2
.........................................................ucagucuuuuucucucuccua................    159     0       BF2
.........................................................ucagucuuuuucucucuccuG................    1       1       BF2
.........................................................ucagucuuuuuAucucuccua................    1       1       BF2
.........................................................ucagucuuuCucucucuccua................    1       1       BF2
.........................................................ucagucuuuuucucucuccuU................    4       1       BF2
.........................................................ucagucuuuuucucucuccCau...............    1       1       BF2
.........................................................ucagucuuuuucucucucUuau...............    3       1       BF2
.........................................................ucagucuuuCucucucuccuau...............    4       1       BF2
.........................................................ucagucuuuuCcucucuccuau...............    2       1       BF2
.........................................................ucagucuuuuucucucuccuau...............    3784    0       BF2
.........................................................ucagCcuuuuucucucuccuau...............    1       1       BF2
.........................................................ucagucCuuuucucucuccuau...............    7       1       BF2
.........................................................ucagucuuuuuGucucuccuau...............    1       1       BF2
.........................................................ucagucuuuuucucucuccuaA...............    4       1       BF2
.........................................................ucagucuuuuucucCcuccuau...............    1       1       BF2
.........................................................ucagAcuuuuucucucuccuau...............    1       1       BF2
.........................................................Acagucuuuuucucucuccuau...............    1       1       BF2
.........................................................ucagucuuCuucucucuccuau...............    3       1       BF2
.........................................................ucagucuuuuucucucuUcuau...............    2       1       BF2
.........................................................ucagucuuAuucucucuccuau...............    1       1       BF2
.........................................................ucagucuuuuucucucuccuGu...............    1       1       BF2
.........................................................ucagucuuuuuAucucuccuau...............    2       1       BF2
.........................................................Gcagucuuuuucucucuccuau...............    1       1       BF2
.........................................................ucagucuuuuucuAucuccuau...............    1       1       BF2
.........................................................ucagucuuuuucucucuccuaC...............    17      1       BF2
.........................................................ucagucuuuuucCcucuccuau...............    1       1       BF2
.........................................................uUagucuuuuucucucuccuau...............    1       1       BF2
.........................................................ucagucuuuuucucucucAuau...............    1       1       BF2
.........................................................ucagucuuuuucucuUuccuau...............    1       1       BF2
.........................................................ucagucuuuuucucucuccuauU..............    86      1       BF2
.........................................................ucagucuuuuucucucuccuauA..............    66      1       BF2
.........................................................ucagucuuuuucucucuccuauc..............    3       0       BF2
.........................................................ucagucuuuuucucucuccuaucU.............    1       1       BF2
```

aga-miR-14*

aga-miR-14

gcccgauaagccugugggagcgagauuaaggcuugcugguuaucacguuaaacguagucagucuuuuucucucuccuaucgguacuaacggugc

....cagucuuuuucucucuccuau...............	2	0	BF2
....cagucuuuuucucucuccuauc..............	1	0	BF2
....agucuuuuucucucuccuau...............	1	0	BF2
...............gggagcgagauuaaggcuugcu...	1	0	BF1
.....................................gguuaucacguuaaacg..	1	0	BF1
.....................................gguuaucacguuaaacguagucagucuuCuucucucuccuau...............	1	1	BF1
.....................................gguuaucacguuaaacguagucagucuuuuucucucuccuau...............	2	0	BF1
....gucagucuuuuucucucuccuau...............	1	0	BF1
....ucagucuuuuucucucucc..................	3	0	BF1
....ucagucuuuuucucucuUcu.................	1	1	BF1
....ucagucuuuuucucucuccu.................	263	0	BF1
....ucagucuuuuucucucuccua................	72	0	BF1
....ucagucuuuuucuUucuccuau...............	1	1	BF1
....ucagucuuuuucucuAuccuau...............	1	1	BF1
....ucaguUuuuuucucucuccuau...............	1	1	BF1
....ucagucuuuuucCcucuccuau...............	2	1	BF1
....uUagucuuuuucucucuccuau...............	1	1	BF1
....ucagucCuuuucucucuccuau...............	1	1	BF1
....ucagucuuuuucucucuccuaC...............	2	1	BF1
....ucagucuuuuuUucucuccuau...............	1	1	BF1
....ucagucuuuuuGucucuccuau...............	3	1	BF1
....ucagucuuuuucucucuccCau...............	1	1	BF1
....ucUgucuuuuucucucuccuau...............	1	1	BF1
....ucagucuuuuucucucuccuau...............	1440	0	BF1
....ucagucuuuuucucucuccuaA...............	1	1	BF1
....ucagucuuuuCcucucuccuau...............	1	1	BF1
....ucagucuuuuucucucuccuUu...............	1	1	BF1
....ucagucuuuuucucucucUuau...............	1	1	BF1
....ucagucuuuuucucucuccuauG..............	1	1	BF1
....ucagucuuuuucucucuccuauU..............	39	1	BF1
....ucagucuuuuucucucuccuauA..............	30	1	BF1
....ucagucuuuuucucucuccAauc..............	1	1	BF1
....cagucuuuuucucucuccua................	1	0	BF1
....cagucuuuuucucucuccuaucg.............	1	0	BF1
....agucuuuuucucucuccua................	1	0	BF1
...............gggagcgagauuaaggcuugcu...	1	0	MW1
.....................................gguuaucacguuaaacguagucagucuuuuucucucuccuau...............	1	0	MW1
....ucagucuuuuucucucu....................	2	0	MW1
....ucagucuuuuucucucucc..................	3	0	MW1
....ucagucuuuuucucucuccu.................	152	0	MW1
....ucagucCuuuucucucuccu.................	1	1	MW1
....uUagucuuuuucucucuccu.................	1	1	MW1
....ucagCcuuuuucucucuccu.................	1	1	MW1
....ucagucuuuuucucucuccua................	20	0	MW1
....Acagucuuuuucucucuccuau...............	2	1	MW1
....ucagCcuuuuucucucuccuau...............	1	1	MW1
....ucagucCuuuucucucuccuau...............	1	1	MW1
....ucagucuuuuucucucuccCau...............	1	1	MW1
....ucagucuCuuucucucuccuau...............	1	1	MW1
....ucagucuuuuucucucuccuau...............	545	0	MW1
....ucagucuuCuucucucuccuau...............	1	1	MW1
....ucagucuuuuucucucuccuaA...............	1	1	MW1
....ucagucuuuuucucucuccuaC...............	4	1	MW1
....ucagucuuuuucucucuccuauc..............	2	0	MW1
....ucagucuuuuucucucuccuaGc..............	1	1	MW1
....ucagucuuuuucucucuccuauA..............	12	1	MW1
....ucagucuuuuucucucuccuauU..............	14	1	MW1
....ucagucuuuuucucucuccuauG..............	1	1	MW1
....ucagucuuuuucucucuccuaucU.............	2	1	MW1
....agucuuuuucucucuccuau...............	1	0	MW1
....cuuuuucucucuccuau...............	1	0	MW1
...............gggagcgagauuaaggcuugcu...	1	0	FW1
....ucagucuuuuucucucuccu.................	43	0	FW1
....ucagucuuuuucucucuccua................	9	0	FW1
....ucagucuuuuucucucuccuau...............	260	0	FW1
....Ccagucuuuuucucucuccuau...............	1	1	FW1

```
               aga-miR-14*
                                                         aga-miR-14
gcccgauaagccugugggagcgagauuaaggcuugcugguuaucacguuaaacguagucagucuuuuucucucuccuaucgguacuaacggugc

.........................................................ucagucuuuuucucucuccuauA..............    6     1     FW1
.........................................................ucagucuuuuucucucuccuauc..............    1     0     FW1
.........................................................ucagucuuuuucucucuccuauU..............    8     1     FW1
............................................................gucuuuuucucucuccuau...............    1     0     FW1

...............gggagcgagauuaaggcuugcu.........................................................    2     0     MW2
....................................................cguCgucagucuuuuucucucuccuau...............    1     1     MW2
.....................................................guCgucagucuuuuucucucuccuau...............    1     1     MW2
.........................................................ucagucuuuuucucucucc..................    1     0     MW2
.........................................................ucagucuuuuucucucuccu.................    46    0     MW2
.........................................................ucagucuuuuucucucuccua................    8     0     MW2
.........................................................ucagucuuuuucucucuccuau...............    219   0     MW2
.........................................................ucagCcuuuuucucucuccuau...............    1     1     MW2
.........................................................ucagucuuuuuGucucuccuau...............    1     1     MW2
.........................................................ucagucuuuuucucucuccuaC...............    1     1     MW2
.........................................................ucagucuuuuucucucuccuauA..............    4     1     MW2
.........................................................ucagucuuuuucucucuccuauU..............    6     1     MW2

............ugugggagcgagauuaaggcuug...........................................................    1     0     TE2
...............gggagcgagauuaaggcuugcu.........................................................    2     0     TE2
.......................................................agucagucuuuuucucucuccu.................    2     0     TE2
........................................................Cucagucuuuuucucucuccu.................    1     1     TE2
........................................................Cucagucuuuuucucucuccuau...............    1     1     TE2
.........................................................ucagucuuuuucucucu....................    1     0     TE2
.........................................................ucagucuuuuucucucuc...................    2     0     TE2
.........................................................ucagucuuuuucucucucU..................    1     1     TE2
.........................................................ucagucuuuuucucucucc..................    45    0     TE2
.........................................................ucagucuuCuucucucuccu.................    1     1     TE2
.........................................................ucagucuuuuucucuUuccu.................    1     1     TE2
.........................................................ucagucuuuuucAcucuccu.................    1     1     TE2
.........................................................ucagucuuuuucucucCccu.................    1     1     TE2
.........................................................ucagucuuuuucucucuccG.................    9     1     TE2
.........................................................ucagucuuuuucucucuccu.................    2253  0     TE2
.........................................................uUagucuuuuucucucuccu.................    1     1     TE2
.........................................................ucagucuuuuucucucuccA.................    1     1     TE2
.........................................................ucagucuuuuuAucucuccu.................    1     1     TE2
.........................................................ucagucuuuuucCcucuccu.................    1     1     TE2
.........................................................ucaAucuuuuucucucuccu.................    1     1     TE2
.........................................................ucagucuuuuCcucucuccu.................    2     1     TE2
.........................................................ucagucuuuuuGucucuccu.................    3     1     TE2
.........................................................ucagucuuuuucucucuccC.................    2     1     TE2
.........................................................ucagucCuuuucucucuccu.................    2     1     TE2
.........................................................ucagCcuuuuucucucuccua................    1     1     TE2
.........................................................ucagucuuuuucucucuccua................    410   0     TE2
.........................................................ucagucuuuuucCcucuccua................    1     1     TE2
.........................................................ucagucuuuuucucucuccuG................    1     1     TE2
.........................................................ucagucCuuuucucucuccua................    1     1     TE2
.........................................................ucagucuuuuucucucuccuU................    2     1     TE2
.........................................................ucaguGuuuuucucucuccua................    1     1     TE2
.........................................................uUagucuuuuucucucuccuau...............    4     1     TE2
.........................................................ucagucuuuuucucucuUcuau...............    1     1     TE2
.........................................................ucagucuuuuucucucAccuau...............    2     1     TE2
.........................................................Acagucuuuuucucucuccuau...............    1     1     TE2
.........................................................ucagucuuuuucuAucuccuau...............    1     1     TE2
.........................................................ucaAucuuuuucucucuccuau...............    3     1     TE2
.........................................................ucagucuuuuucucucCccuau...............    3     1     TE2
.........................................................ucagucuuuCucucucuccuau...............    1     1     TE2
.........................................................ucagucuuuuucucAcuccuau...............    1     1     TE2
.........................................................ucagucuuuuucucucuccuaC...............    20    1     TE2
.........................................................ucagucuuCuucucucuccuau...............    3     1     TE2
.........................................................ucagucuuuuuGucucuccuau...............    3     1     TE2
.........................................................ucagucuuuuuAucucuccuau...............    1     1     TE2
.........................................................ucagucuCuuucucucuccuau...............    2     1     TE2
.........................................................ucagucuuuuuUucucuccuau...............    3     1     TE2
.........................................................ucagucuuuuucucucuccuUu...............    1     1     TE2
.........................................................ucagucuuuuucucuUuccuau...............    2     1     TE2
.........................................................ucagucuuuuucucucuccCau...............    3     1     TE2
.........................................................ucagucuuuuucucucuccuaG...............    2     1     TE2
.........................................................ucagucuuuuCcucucuccuau...............    1     1     TE2
```

```
               aga-miR-14*
                                                        aga-miR-14
gcccgauaagccugugggagcgagauuaaggcuugcugguuaucacguuaaacguagucagucuuuuucucucuccuaucgguacuaacggugc

........................................................ucagucuuuuucAcucuccuau...............    1     1    TE2
........................................................ucGgucuuuuucucucuccuau...............    1     1    TE2
........................................................ucagucuuuuucucucuccuaA...............    7     1    TE2
........................................................ucagucuuuuucCcucuccuau...............    2     1    TE2
........................................................ucagCcuuuuucucucuccuau...............    2     1    TE2
........................................................ucagucuuuuucucucucGuau...............    1     1    TE2
........................................................ucagucCuuuucucucuccuau...............    16    1    TE2
........................................................ucagucuuuuucucucucUuau...............    1     1    TE2
........................................................ucaguAuuuuucucucuccuau...............    1     1    TE2
........................................................ucagucuuuGucucucuccuau...............    1     1    TE2
........................................................Ccagucuuuuucucucuccuau...............    2     1    TE2
........................................................ucaguUuuuuucucucuccuau...............    1     1    TE2
........................................................ucagucuuuuucucCcuccuau...............    1     1    TE2
........................................................ucagucuuuuucucucuccuau...............    4460  0    TE2
........................................................ucagucuuuuucucucuccuauc..............    4     0    TE2
........................................................ucagucuuuuucucucuccuauU..............    132   1    TE2
........................................................ucagucuuuuucucucuccuauA..............    115   1    TE2
........................................................ucagucuuuuucucucuccuaucU.............    3     1    TE2
.........................................................cagucuuuuucucucuccu..................    2     0    TE2
.........................................................cagucuuuuucucucuccuau................    4     0    TE2
.........................................................cagucuuuuucucucuccuauA...............    1     1    TE2
..........................................................agucuuuuucucucuccuau................    1     0    TE2
..........................................................agucuuuuucucucuccuauA...............    1     1    TE2
```

miRBase precursor : aga-mir-184
Total read count : 611464
aga-miR-184 read count : 611330
aga-miR-184* read count : 69
remaining reads : 65

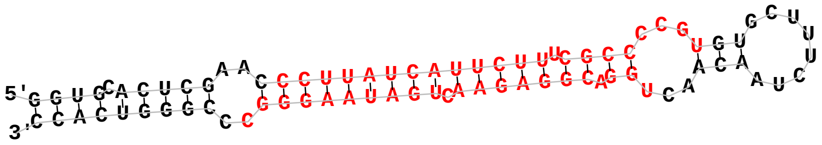

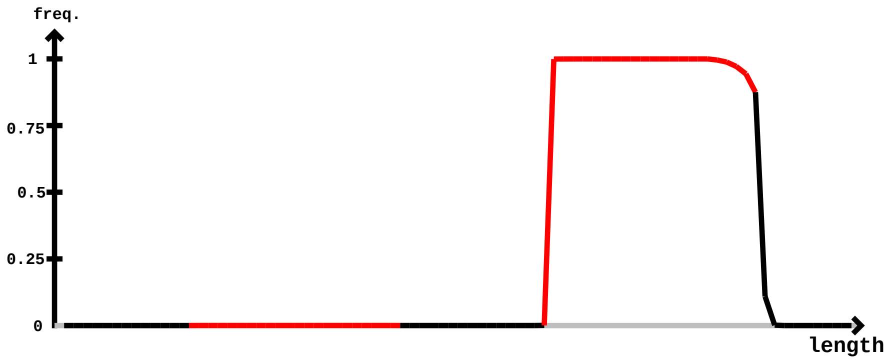


```
                                        aga-miR-184*
                                                                        aga-miR-184
5'- ggugcacucgaacccuuaucauucuuucgccccgugugcuuucuaacaacuggacggagaacugauaagggcccgggucacc   -3'    exp
    ((((.(((((..(((((((((((((.((((...(((........)))...)).)))))))).)))))))))..)))))))))   reads      mm         sample
    .............ccuuaucauucuuucgccccgugugcuuucuaac...................................   1          0          FF2
    ..............................................caacuggacggagaacugauaagggc..........   1          0          FF2
    .................................................cuggacggagaacugauaa..............   1          0          FF2
    .................................................cuggacggagaacugauaag.............   2          0          FF2
    .................................................cuggacggagaacugauaaggg...........   1          0          FF2
    .................................................cuggacggagaacugauaagggc..........   11         0          FF2
    ..................................................uggacggagaacugaua...............   164        0          FF2
    ..................................................ugAacggagaacugaua...............   1          1          FF2
    ..................................................uggacggagaacugauU...............   2          1          FF2
    ..................................................uggacggagaacugauaG..............   1          1          FF2
    ..................................................uggacggagaacuAauaa..............   1          1          FF2
    ..................................................ugAacggagaacugauaa..............   1          1          FF2
    ..................................................uggacgAagaacugauaa..............   1          1          FF2
    ..................................................uggacggagCacugauaa..............   2          1          FF2
    ..................................................uggacggagaacugauaU..............   5          1          FF2
    ..................................................uggacggagaacugauaa..............   361        0          FF2
    ..................................................uggacggagaacugauaag.............   810        0          FF2
    ..................................................uggacggagaacugauaaC.............   1          1          FF2
    ..................................................uggacggagGacugauaag.............   1          1          FF2
    ..................................................uggacgAagaacugauaag.............   1          1          FF2
    ..................................................uggacggagaGcugauaag.............   1          1          FF2
    ..................................................uggacggagaacugauaaU.............   5          1          FF2
    ..................................................uggacggagaacugauaaA.............   73         1          FF2
    ..................................................uggacggagaaGugauaag.............   1          1          FF2
    ..................................................uggacggagaacugauaagU............   3          1          FF2
    ..................................................uggacggagaCcugauaagg............   2          1          FF2
    ..................................................uggacggagaacugauaagg............   1132       0          FF2
    ..................................................ugAacggagaacugauaagg............   2          1          FF2
    ..................................................uUgacggagaacugauaagg............   1          1          FF2
    ..................................................uggacggagaacugauaagA............   54         1          FF2
    ..................................................uggacggagaacugaCaagg............   1          1          FF2
    ..................................................uggacggagGacugauaagg............   1          1          FF2
    ..................................................uggacggagaaAugauaagg............   1          1          FF2
    ..................................................uggacgAagaacugauaagg............   1          1          FF2
```

```
             aga-miR-184*
                                                  aga-miR-184
ggugcacucgaacccuuaucauucuuucgccccgugugcuuucuaacaacuggacggagaacugauaagggcccgggucacc

..................................................uggaUggagaacugauaagg............    2       1       FF2
..................................................uggacggagaacugaCaaggg...........    1       1       FF2
..................................................ugAacggagaacugauaaggg...........    2       1       FF2
..................................................uggacggagaacugauaaAgg...........    7       1       FF2
..................................................uggacggagaaUugauaaggg...........    1       1       FF2
..................................................uggaUggagaacugauaaggg...........    1       1       FF2
..................................................uggacggagaacugauaaggU...........    13      1       FF2
..................................................uggacggagaacugauaaggA...........    136     1       FF2
..................................................uggacggagGacugauaaggg...........    1       1       FF2
..................................................uggacggagaacugauUaggg...........    1       1       FF2
..................................................uggacggagaacugauaaggC...........    1       1       FF2
..................................................uggacggagaacugauGaggg...........    2       1       FF2
..................................................Cggacggagaacugauaaggg...........    1       1       FF2
..................................................uggacggUgaacugauaaggg...........    1       1       FF2
..................................................uggacggagaacuAauaaggg...........    1       1       FF2
..................................................uggacggagaacugauaaggg...........    1928    0       FF2
..................................................uggacggGgaacugauaaggg...........    1       1       FF2
..................................................uggacggagaacugauaagAg...........    1       1       FF2
..................................................uggacggUgaacugauaagggc..........    1       1       FF2
..................................................uggacggaAaacugauaagggc..........    5       1       FF2
..................................................Cggacggagaacugauaagggc..........    10      1       FF2
..................................................uggacggagaacugauGagggc..........    3       1       FF2
..................................................uggacggagaUcugauaagggc..........    2       1       FF2
..................................................uggacggagaacugUuaagggc..........    2       1       FF2
..................................................uggacggagCacugauaagggc..........    4       1       FF2
..................................................uggacUgagaacugauaagggc..........    2       1       FF2
..................................................uAgacggagaacugauaagggc..........    5       1       FF2
..................................................uggacgCagaacugauaagggc..........    1       1       FF2
..................................................uggacggagGacugauaagggc..........    7       1       FF2
..................................................uggaAggagaacugauaagggc..........    1       1       FF2
..................................................uggacggagaacGgauaagggc..........    2       1       FF2
..................................................uggacggagaacugauUagggc..........    4       1       FF2
..................................................uggacggagaacuAauaagggc..........    12      1       FF2
..................................................uggacggagaacuUauaagggc..........    1       1       FF2
..................................................uggacggaUaacugauaagggc..........    1       1       FF2
..................................................uggacggagaGcugauaagggc..........    2       1       FF2
..................................................ugUacggagaacugauaagggc..........    1       1       FF2
..................................................uggacggagaCcugauaagggc..........    2       1       FF2
..................................................uggacggagaacugaCaagggc..........    3       1       FF2
..................................................Aggacggagaacugauaagggc..........    4       1       FF2
..................................................uggacggagaacAgauaagggc..........    2       1       FF2
..................................................uUgacggagaacugauaagggc..........    1       1       FF2
..................................................uggGcggagaacugauaagggc..........    4       1       FF2
..................................................uggacggagUacugauaagggc..........    3       1       FF2
..................................................uggacggagaacCgauaagggc..........    8       1       FF2
..................................................uggacggagaacugauaagggA..........    318     1       FF2
..................................................uggacggagaacugauaagUgc..........    1       1       FF2
..................................................uggacggagaacugauaagggG..........    19      1       FF2
..................................................uggacggagaacugGuaagggc..........    1       1       FF2
..................................................uggacggagaacugauaagggU..........    53      1       FF2
..................................................uggacggagaacugauaaUggc..........    4       1       FF2
..................................................uggacggagaaAugauaagggc..........    12      1       FF2
..................................................uggacggagaaGugauaagggc..........    8       1       FF2
..................................................uggacggagaacugauaaCggc..........    1       1       FF2
..................................................uggacgAagaacugauaagggc..........    17      1       FF2
..................................................uggaGggagaacugauaagggc..........    1       1       FF2
..................................................uggacggagaaUugauaagggc..........    6       1       FF2
..................................................uggacggaCaacugauaagggc..........    3       1       FF2
..................................................uggaUggagaacugauaagggc..........    8       1       FF2
..................................................uggacggGgaacugauaagggc..........    3       1       FF2
..................................................uggacAgagaacugauaagggc..........    10      1       FF2
..................................................ugAacggagaacugauaagggc..........    9       1       FF2
..................................................uggacggagaacugauaaggAc..........    7       1       FF2
..................................................uggacgUagaacugauaagggc..........    2       1       FF2
..................................................uggacggagaacugauaaAggc..........    2       1       FF2
..................................................uggacggagaacugauCagggc..........    2       1       FF2
..................................................uggacggagaacugauaagggc..........    20147   0       FF2
..................................................uggacggagaacugauaGgggc..........    6       1       FF2
..................................................Gggacggagaacugauaagggc..........    2       1       FF2
```

```
             aga-miR-184*
                                                  aga-miR-184
ggugcacucgaacccuuaucauucuuucgccccgugugcuuucuaacaacuggacggagaacugauaagggcccgggucacc

..................................................uggacggagaacugauaagggcU.........   1732   1   FF2
..................................................uggacggagaacugauaagggcA.........   795    1   FF2
..................................................uggacggagaacugauaagggcc.........   56     0   FF2
..................................................uggacggagaacugauaagggcG.........   16     1   FF2
..................................................uggacggagaacugauaagggccA........   5      1   FF2
..................................................uggacggagaacugauaagggccc........   1      0   FF2
..................................................uggacggagaacugauaagggcUc........   5      1   FF2
..................................................uggacggagaacugauaagggccU........   17     1   FF2
..................................................uggacggagaacugauaagggcAc........   4      1   FF2
...................................................ggacggagaacugauaagA............   1      1   FF2
...................................................ggacggagaacugauaaggg...........   2      0   FF2
...................................................ggacggagaacugauaagggc..........   7      0   FF2
...................................................ggacggagaacugauaagggcU.........   1      1   FF2
....................................................gacggagaacugauaagggcc.........   1      0   FF2

.............ccuuaucauucuuucgUcccgu...............................................   1      1   OV2
.............ccuuaucauucuuucgccccgu...............................................   1      0   OV2
.............ccuuaucauucuuucgccccgugugcu..........................................   1      0   OV2
.............ccuuaucauucuuucgccccgugugcuuucuaacaac................................   3      0   OV2
...............................................aacuggacggagaacugauaagggc..........   1      0   OV2
.................................................cuggacggagaacugauaagg............   4      0   OV2
.................................................cuggacggagaacugauaaggg...........   2      0   OV2
.................................................Guggacggagaacugauaagggc..........   1      1   OV2
.................................................cuggacggagaacugauaagggc..........   12     0   OV2
.................................................cuggacggagaacugauaagggcU.........   1      1   OV2
.................................................cuggacggagaacugauaagggcA.........   3      1   OV2
..................................................uggacggUgaacugaua...............   1      1   OV2
..................................................uggacggagaCcugaua...............   2      1   OV2
..................................................uggacggagaacugaua...............   203    0   OV2
..................................................uggacggagaacugauaa..............   309    0   OV2
..................................................uggacggagaacugauaU..............   2      1   OV2
..................................................uAgacggagaacugauaa..............   1      1   OV2
..................................................uggacggagaacugauaag.............   779    0   OV2
..................................................uggaAggagaacugauaag.............   1      1   OV2
..................................................uggaUggagaacugauaag.............   1      1   OV2
..................................................uggacggagaacugauaaU.............   1      1   OV2
..................................................uggacggagaacugauaaA.............   43     1   OV2
..................................................uggacUgagaacugauaag.............   1      1   OV2
..................................................ugUacggagaacugauaag.............   1      1   OV2
..................................................uggacggagaCcugauaagg............   1      1   OV2
..................................................uggacggagaacugauaagA............   57     1   OV2
..................................................uggaUggagaacugauaagg............   1      1   OV2
..................................................uggacUgagaacugauaagg............   1      1   OV2
..................................................uggacggCgaacugauaagg............   1      1   OV2
..................................................uggacggagaacugauaagg............   1445   0   OV2
..................................................uggacggagaacCgauaagg............   1      1   OV2
..................................................uggacAgagaacugauaagg............   1      1   OV2
..................................................uggacggagaaGugauaagg............   1      1   OV2
..................................................uggacggagaacuUauaagg............   1      1   OV2
..................................................uAgacggagaacugauaaggg...........   1      1   OV2
..................................................uggUcggagaacugauaaggg...........   1      1   OV2
..................................................uggacggagaacugauaaggA...........   161    1   OV2
..................................................uUgacggagaacugauaaggg...........   1      1   OV2
..................................................uggacggagaaGugauaaggg...........   2      1   OV2
..................................................ugUacggagaacugauaaggg...........   1      1   OV2
..................................................uggacggagaacugauaaggU...........   5      1   OV2
..................................................uggacggGgaacugauaaggg...........   4      1   OV2
..................................................uggacggagaacugauaagUg...........   1      1   OV2
..................................................uggacgAagaacugauaaggg...........   3      1   OV2
..................................................uggacggagaacuAauaaggg...........   3      1   OV2
..................................................uggacggagaacugaAaaggg...........   1      1   OV2
..................................................uggacggagaacugaGaaggg...........   1      1   OV2
..................................................uggacUgagaacugauaaggg...........   2      1   OV2
..................................................uggGcggagaacugauaaggg...........   1      1   OV2
..................................................uggacggagaacugaCaaggg...........   1      1   OV2
..................................................uggacggagaaUugauaaggg...........   2      1   OV2
..................................................Aggacggagaacugauaaggg...........   2      1   OV2
..................................................uggacggagaacugauaGggg...........   1      1   OV2
..................................................Cggacggagaacugauaaggg...........   1      1   OV2
```

```
                aga-miR-184*
                                                  aga-miR-184
ggugcacucgaacccuuaucauucuuucgccccgugugcuuucuaacaacuggacggagaacugauaagggcccgggucacc

..................................................uggacggagCacugauaaggg...........   1      1    OV2
..................................................uggacggagaUcugauaaggg...........   1      1    OV2
..................................................uggacggagaaAugauaaggg...........   2      1    OV2
..................................................uggacggagaCcugauaaggg...........   3      1    OV2
..................................................uggacAgagaacugauaaggg...........   1      1    OV2
..................................................uggacggagaacCgauaaggg...........   3      1    OV2
..................................................uggacggagaacugauaaggC...........   2      1    OV2
..................................................ugAacggagaacugauaaggg...........   2      1    OV2
..................................................uggacggagaacugauaaAgg...........   17     1    OV2
..................................................uggacggagaacugauaaggg...........   3909   0    OV2
..................................................uggacggagaacugauGagggc..........   6      1    OV2
..................................................ugAacggagaacugauaagggc..........   20     1    OV2
..................................................uUgacggagaacugauaagggc..........   4      1    OV2
..................................................uggacggGgaacugauaagggc..........   7      1    OV2
..................................................uggacggagaacugauaaggUc..........   1      1    OV2
..................................................uggacggagaacGgauaagggc..........   2      1    OV2
..................................................uggacggagaacugauaagggA..........   698    1    OV2
..................................................uggGcggagaacugauaagggc..........   9      1    OV2
..................................................uggacggagaaUugauaagggc..........   7      1    OV2
..................................................uggaUggagaacugauaagggc..........   8      1    OV2
..................................................ugUacggagaacugauaagggc..........   4      1    OV2
..................................................uggacggagaacugauaaAggc..........   5      1    OV2
..................................................uggacggagaacugGuaagggc..........   2      1    OV2
..................................................ugCacggagaacugauaagggc..........   2      1    OV2
..................................................uggacggagaacugauaagCgc..........   1      1    OV2
..................................................uggacggagaGcugauaagggc..........   5      1    OV2
..................................................uggacggagaacugauaCgggc..........   2      1    OV2
..................................................uggUcggagaacugauaagggc..........   3      1    OV2
..................................................uggacggagaacuUauaagggc..........   1      1    OV2
..................................................uggaAggagaacugauaagggc..........   2      1    OV2
..................................................uggacggagaacugauaagggG..........   4      1    OV2
..................................................uggacggagaacCgauaagggc..........   22     1    OV2
..................................................uCgacggagaacugauaagggc..........   1      1    OV2
..................................................uggacggagaacuAauaagggc..........   19     1    OV2
..................................................uggacggagaaGugauaagggc..........   7      1    OV2
..................................................uggacggagaacugauaaCggc..........   1      1    OV2
..................................................uggacggCgaacugauaagggc..........   1      1    OV2
..................................................uggacggagaacugauaaggAc..........   17     1    OV2
..................................................uggacggagaacugauCagggc..........   1      1    OV2
..................................................uggacgCagaacugauaagggc..........   4      1    OV2
..................................................uggacggagaacugauaagggU..........   46     1    OV2
..................................................uggacggagaUcugauaagggc..........   13     1    OV2
..................................................uggacggUgaacugauaagggc..........   2      1    OV2
..................................................uggacggagaacugauaagAgc..........   5      1    OV2
..................................................uggaGggagaacugauaagggc..........   1      1    OV2
..................................................uggacggagaacugauaagUgc..........   3      1    OV2
..................................................uggacgAagaacugauaagggc..........   29     1    OV2
..................................................Aggacggagaacugauaagggc..........   3      1    OV2
..................................................uggacggagaaAugauaagggc..........   15     1    OV2
..................................................uAgacggagaacugauaagggc..........   13     1    OV2
..................................................uggacggagGacugauaagggc..........   8      1    OV2
..................................................uggacggagUacugauaagggc..........   6      1    OV2
..................................................uggacggaCaacugauaagggc..........   3      1    OV2
..................................................uggacggaAaacugauaagggc..........   2      1    OV2
..................................................uggacggagaacugCuaagggc..........   1      1    OV2
..................................................uggCcggagaacugauaagggc..........   1      1    OV2
..................................................uggacggagaacugauaaggCc..........   1      1    OV2
..................................................uggacggagaacugauaGgggc..........   3      1    OV2
..................................................uggacggagCacugauaagggc..........   1      1    OV2
..................................................uggacggagaacugauaagggc..........   38046  0    OV2
..................................................uggacUgagaacugauaagggc..........   5      1    OV2
..................................................Gggacggagaacugauaagggc..........   5      1    OV2
..................................................uggacggagaacugaCaagggc..........   13     1    OV2
..................................................Cggacggagaacugauaagggc..........   12     1    OV2
..................................................uggacggagaCcugauaagggc..........   12     1    OV2
..................................................uggacgUagaacugauaagggc..........   1      1    OV2
..................................................uggacggaUaacugauaagggc..........   3      1    OV2
..................................................uggacggagaacugUuaagggc..........   3      1    OV2
..................................................uggacAgagaacugauaagggc..........   4      1    OV2
```

```
             aga-miR-184*
                                                  aga-miR-184
ggugcacucgaacccuuaucauucuuucgccccgugugcuuucuaacaacuggacggagaacugauaagggcccgggucacc

..................................................uggacggagaacugaAaagggc..........    1      1     OV2
..................................................uggacggagaacugauaagggUc.........    1      1     OV2
..................................................ugUacggagaacugauaagggcc.........    1      1     OV2
..................................................uggacggagaacugauaagggAc.........    3      1     OV2
..................................................uggacggagaacugauaagggcG.........    29     1     OV2
..................................................uggacggagaacugauaagggcU.........    1989   1     OV2
..................................................uggacggagaacugauaagggcA.........    3814   1     OV2
..................................................uggacggagaacugauaagggcc.........    81     0     OV2
..................................................uggacggagaacugauaagggccA........    9      1     OV2
..................................................uggacggagaacugauaagggcAc........    15     1     OV2
..................................................uggacggagaacugauaagggcUc........    8      1     OV2
..................................................uggacggagaacugauaagggccU........    16     1     OV2
...................................................ggacggagaacugauaagg............    4      0     OV2
...................................................ggacggagaacugauaaggg...........    2      0     OV2
...................................................ggacggagaacugauaagggc..........    18     0     OV2
...................................................ggacggagaacugauaagggcU.........    1      1     OV2
....................................................gacggagaacugauaagggc..........    3      0     OV2
.....................................................acggagaacugauaagggc..........    2      0     OV2

.............ccuuaucauucuuucgcc...................................................    1      0     TE1
.............ccuuaucauucuuucgccccg................................................    1      0     TE1
.............ccuuaucauucuuucgccccgu...............................................    1      0     TE1
.............ccuuaucauucuuucgccccgugugcuuucuaaca..................................    1      0     TE1
.................................................cuggacggagaacugauaag.............    1      0     TE1
.................................................cuggacggagaacugauaaggg...........    3      0     TE1
.................................................cuggacggagaacugauaagggc..........    5      0     TE1
.................................................cuggacggagaacugauaaggCc..........    1      1     TE1
.................................................cuggacggagaacugauaagggcU.........    1      1     TE1
..................................................uggacggagCacugaua...............    1      1     TE1
..................................................uggacggagaacugaua...............    77     0     TE1
..................................................uggacggagaacugauaa..............    132    0     TE1
..................................................uggacggagaacugauaaA.............    3      1     TE1
..................................................uggacgAagaacugauaag.............    1      1     TE1
..................................................uggacggGgaacugauaag.............    1      1     TE1
..................................................uggacggagaacugauaag.............    169    0     TE1
..................................................uggacggagaacugauaagU............    1      1     TE1
..................................................uggacggagaCcugauaagg............    1      1     TE1
..................................................uggacggagaacugauaagg............    384    0     TE1
..................................................uggacggagaacugauaagA............    2      1     TE1
..................................................uAgacggagaacugauaagg............    1      1     TE1
..................................................uggacgAagaacugauaagg............    1      1     TE1
..................................................uggacggagaacugauaaAgg...........    3      1     TE1
..................................................uggacggagaacugauaaggC...........    1      1     TE1
..................................................uggacggagaacugauCaggg...........    1      1     TE1
..................................................uggGcggagaacugauaaggg...........    1      1     TE1
..................................................uggacggagaacugauUaggg...........    1      1     TE1
..................................................Cggacggagaacugauaaggg...........    1      1     TE1
..................................................uggacggagaaGugauaaggg...........    1      1     TE1
..................................................uggacggagaCcugauaaggg...........    1      1     TE1
..................................................uggacggagaacuUauaaggg...........    1      1     TE1
..................................................uggacggagaacugauaaggg...........    1462   0     TE1
..................................................uggacggagaacuAauaaggg...........    1      1     TE1
..................................................uggacggaAaacugauaaggg...........    1      1     TE1
..................................................ugAacggagaacugauaaggg...........    1      1     TE1
..................................................uggacggagaacugauaaggA...........    18     1     TE1
..................................................uggacgCagaacugauaagggc..........    1      1     TE1
..................................................Gggacggagaacugauaagggc..........    1      1     TE1
..................................................uggacggagaaGugauaagggc..........    6      1     TE1
..................................................uggacggagaacugauaaCggc..........    1      1     TE1
..................................................uggacggagaacugauaagggA..........    160    1     TE1
..................................................uggacggUgaacugauaagggc..........    3      1     TE1
..................................................uggacggagaacuAauaagggc..........    9      1     TE1
..................................................uggacggaUaacugauaagggc..........    1      1     TE1
..................................................uggGcggagaacugauaagggc..........    4      1     TE1
..................................................uggacggagaacugauaaggUc..........    2      1     TE1
..................................................uggacggagaacugauaagUgc..........    3      1     TE1
..................................................uggacggagaacugauUagggc..........    1      1     TE1
..................................................uggacggagaacugauaaggAc..........    7      1     TE1
..................................................uggacggagGacugauaagggc..........    5      1     TE1
```

```
              aga-miR-184*
                                                   aga-miR-184
ggugcacucgaacccuuaucauucuuucgccccgugugcuuucuaacaacuggacggagaacugauaagggcccgggucacc

..................................................uggacggagaacugGuaagggc..........   2       1    TE1
..................................................uggacggagaacugauaagggc..........   13718   0    TE1
..................................................uggacgAagaacugauaagggc..........   8       1    TE1
..................................................uggacggagaaUugauaagggc..........   3       1    TE1
..................................................uggacggagaacugauaagAgc..........   6       1    TE1
..................................................uggacggagaacugaCaagggc..........   5       1    TE1
..................................................uggacggagaacugauaaAggc..........   2       1    TE1
..................................................uggaAggagaacugauaagggc..........   1       1    TE1
..................................................uggacggagaacAgauaagggc..........   2       1    TE1
..................................................uggacggagCacugauaagggc..........   4       1    TE1
..................................................Aggacggagaacugauaagggc..........   3       1    TE1
..................................................uCgacggagaacugauaagggc..........   1       1    TE1
..................................................uggacggagaGcugauaagggc..........   4       1    TE1
..................................................ugAacggagaacugauaagggc..........   8       1    TE1
..................................................uggacggagaacugauaagggU..........   18      1    TE1
..................................................uggacggagaacugauaagCgc..........   1       1    TE1
..................................................uggacggCgaacugauaagggc..........   1       1    TE1
..................................................uggacggaAaacugauaagggc..........   2       1    TE1
..................................................uggacggagaacuUauaagggc..........   1       1    TE1
..................................................uggacggagaacugauGagggc..........   2       1    TE1
..................................................uggacggagaacugaAaagggc..........   1       1    TE1
..................................................uggacggagaacugauaUgggc..........   2       1    TE1
..................................................uAgacggagaacugauaagggc..........   6       1    TE1
..................................................ugCacggagaacugauaagggc..........   1       1    TE1
..................................................uggacggGgaacugauaagggc..........   2       1    TE1
..................................................uggacggagaacugauaGgggc..........   2       1    TE1
..................................................uggacggagaacugauaagggG..........   8       1    TE1
..................................................uggaUggagaacugauaagggc..........   8       1    TE1
..................................................uggacggagaacCgauaagggc..........   6       1    TE1
..................................................ugUacggagaacugauaagggc..........   2       1    TE1
..................................................uggacggagaUcugauaagggc..........   4       1    TE1
..................................................uggacCgagaacugauaagggc..........   1       1    TE1
..................................................uggacggagaacugauaaUggc..........   2       1    TE1
..................................................uggCcggagaacugauaagggc..........   1       1    TE1
..................................................uggacggagaaAugauaagggc..........   4       1    TE1
..................................................uggacAgagaacugauaagggc..........   9       1    TE1
..................................................uggacgUagaacugauaagggc..........   2       1    TE1
..................................................uggacggagaCcugauaagggc..........   1       1    TE1
..................................................uggacggagaacugauaagggcA.........   939     1    TE1
..................................................uggacggagaCcugauaagggcc.........   1       1    TE1
..................................................uggacggagaacugauaagggcU.........   881     1    TE1
..................................................uggacggagaacugauaagggAc.........   2       1    TE1
..................................................uggacggagaaGugauaagggcc.........   1       1    TE1
..................................................uggacggagaacugauaagggcc.........   35      0    TE1
..................................................uggacggagaacugauaagggcG.........   7       1    TE1
..................................................uggacggagaacugauaagggcUc........   1       1    TE1
..................................................uggacggagaacugauaagggccA........   2       1    TE1
..................................................uggacggagaacugauaagggcAc........   1       1    TE1
..................................................uggacggagaacugauaagggccU........   6       1    TE1
...................................................ggacggagaacugauaaggg...........   1       0    TE1
...................................................ggacAgagaacugauaagggc..........   1       1    TE1
...................................................ggacggagaacugauaagggc..........   1       0    TE1
....................................................gacggagaacCgauaagggc..........   1       1    TE1
....................................................gacggagaacugauaagggc..........   1       0    TE1
....................................................gacggagaacugauaagggccc........   2       0    TE1
....................................................gacggagaacugauaagggcccU.......   1       1    TE1

.............ccuuaucauucuuucgccc..................................................   1       0    MF2
.............ccuuaucauucuuucgccccg................................................   3       0    MF2
.............ccuuaucauucuuucgccccgu...............................................   14      0    MF2
.............ccuuaucauucuuucgccccgugugcuuucuaacaa.................................   1       0    MF2
.............ccuuaucauucuuucgccccgugugcuuucuaacaac................................   7       0    MF2
..............cuuaucauucuuucgccccgugugcuuucuaacaaA................................   1       1    MF2
............................gccccgugugcuuucuaacaacug..............................   1       0    MF2
..............................cccgugugcuuucuaacaacuggacggagaacugauaag.............   1       0    MF2
................................cgugugcuuucuaacaacuggacggagaacugauaagggc..........   1       0    MF2
.................................gugugcuuucuaacaacuggacggagaacugauaagggc..........   1       0    MF2
...................................gugcuuucuaacaacuggacggagaacugauaagggc..........   5       0    MF2
..............................................caacuggacggagaacugauaagggc..........   7       0    MF2
```

aga-miR-184*

aga-miR-184

ggugcacucgaacccuuaucauucuuucgccccgugugcuuucuaacaacuggacggagaacugauaagggcccgggucacc

....Ucuggacggagaacugauaagggc..........	2	1	MF2
....cuggacggagaacugauaa..............	3	0	MF2
....cuggacggagaacugauaag.............	5	0	MF2
....cuggacggagaacugauaaA.............	1	1	MF2
....cuggacggagaacugauaagg............	8	0	MF2
....cuggacggagaacugauaaggg...........	19	0	MF2
....cuggacggagaacugauaaggA...........	1	1	MF2
....Auggacggagaacugauaagggc..........	1	1	MF2
....cuggacggagaacugauaagggc..........	59	0	MF2
....cuggacggagaacugauaagggA..........	2	1	MF2
....cuggacggagaacugauaagggcU.........	4	1	MF2
....cuggacggagaacugauaagggcA.........	1	1	MF2
....cuggacggagaacugauaagggcAc........	1	1	MF2
....cuggacggagaacugauaagggccA........	1	1	MF2
....uggacggagGacugaua...............	1	1	MF2
....uggacggagaacugaua...............	588	0	MF2
....uggacggagaacugauU...............	4	1	MF2
....uggacggagaacuAaua...............	1	1	MF2
....uggacggagaaAugaua...............	1	1	MF2
....uggacggagaacugauaC..............	1	1	MF2
....uggacggagaacugauUa..............	1	1	MF2
....uggacUgagaacugauaa..............	1	1	MF2
....uggacggagaacugauaa..............	1125	0	MF2
....uggacggagaaGugauaa..............	1	1	MF2
....uggacggagaaAugauaa..............	2	1	MF2
....uggacggagaacugauaU..............	20	1	MF2
....uggacggagaaAugauaag.............	1	1	MF2
....uggacggagaacuAauaag.............	1	1	MF2
....uggaUggagaacugauaag.............	2	1	MF2
....ugAacggagaacugauaag.............	1	1	MF2
....uggacggagaacCgauaag.............	3	1	MF2
....uggacggagaacugauaaC.............	3	1	MF2
....Aggacggagaacugauaag.............	1	1	MF2
....uggacggagaacugauaaA.............	330	1	MF2
....uggaAggagaacugauaag.............	1	1	MF2
....uggacgUagaacugauaag.............	1	1	MF2
....uUgacggagaacugauaag.............	1	1	MF2
....uggacggagaacugauCag.............	1	1	MF2
....uggacggagaacugauaag.............	2908	0	MF2
....uggacggagaacugauaaU.............	21	1	MF2
....Cggacggagaacugauaag.............	1	1	MF2
....uggacggagaacugaCaagg............	3	1	MF2
....uggGcggagaacugauaagg............	2	1	MF2
....uggaUggagaacugauaagg............	2	1	MF2
....uggaAggagaacugauaagg............	1	1	MF2
....Aggacggagaacugauaagg............	4	1	MF2
....uggacggagaacugauaagC............	2	1	MF2
....uggacggUgaacugauaagg............	1	1	MF2
....uggacggagaacugauGagg............	1	1	MF2
....ugUacggagaacugauaagg............	2	1	MF2
....ugAacggagaacugauaagg............	1	1	MF2
....uggacggagaacugauaagA............	158	1	MF2
....uggacgUagaacugauaagg............	1	1	MF2
....uggacggagaacugauUagg............	1	1	MF2
....Cggacggagaacugauaagg............	4	1	MF2
....uggacggagaacugauaagg............	5251	0	MF2
....uCgacggagaacugauaagg............	1	1	MF2
....uggacggagaacuUauaagg............	2	1	MF2
....uggacggagaacugauaGgg............	2	1	MF2
....uggacggagaacuAauaagg............	2	1	MF2
....uggacggagaacugGuaagg............	2	1	MF2
....uggacggagaaUugauaagg............	1	1	MF2
....uggacggaAaacugauaagg............	1	1	MF2
....uggacggagaacCgauaagg............	4	1	MF2
....uggacggagaaAugauaagg............	2	1	MF2
....uggacggagaacugauCagg............	2	1	MF2
....uggacggagCacugauaagg............	1	1	MF2
....uggacgAagaacugauaagg............	5	1	MF2
....uggacggagaacugauaagU............	16	1	MF2

```
             aga-miR-184*
                                                  aga-miR-184
ggugcacucgaacccuuaucauucuuucgccccgugugcuuucuaacaacuggacggagaacugauaagggcccgggucacc

..................................................uggacggagaaAugauaaggg...........   6       1   MF2
..................................................Cggacggagaacugauaaggg...........   3       1   MF2
..................................................uggacggagaacugauCaggg...........   4       1   MF2
..................................................uggacggagaacuAauaaggg...........   5       1   MF2
..................................................uggacggagaacugauaaggU...........   86      1   MF2
..................................................uAgacggagaacugauaaggg...........   7       1   MF2
..................................................uCgacggagaacugauaaggg...........   2       1   MF2
..................................................uggacggagaacugaAaaggg...........   1       1   MF2
..................................................uggacggagaacugauaaCgg...........   1       1   MF2
..................................................uggacggagaacugauaaAgg...........   30      1   MF2
..................................................uggaUggagaacugauaaggg...........   3       1   MF2
..................................................uggacggagaacugauGaggg...........   1       1   MF2
..................................................uggacAgagaacugauaaggg...........   2       1   MF2
..................................................uggGcggagaacugauaaggg...........   6       1   MF2
..................................................uggacggagaacugauaaggg...........   9948    0   MF2
..................................................uggUcggagaacugauaaggg...........   2       1   MF2
..................................................uggacggagUacugauaaggg...........   2       1   MF2
..................................................uggacggagaGcugauaaggg...........   1       1   MF2
..................................................ugAacggagaacugauaaggg...........   2       1   MF2
..................................................uggacggagaacugauUaggg...........   1       1   MF2
..................................................uUgacggagaacugauaaggg...........   2       1   MF2
..................................................uggacggagCacugauaaggg...........   1       1   MF2
..................................................uggacggagaacugauaaggA...........   757     1   MF2
..................................................uggacggagaacugauaagAg...........   4       1   MF2
..................................................uggacggagGacugauaaggg...........   3       1   MF2
..................................................uggacggGgaacugauaaggg...........   2       1   MF2
..................................................uggacgAagaacugauaaggg...........   9       1   MF2
..................................................uggacggagaacugauaaggC...........   16      1   MF2
..................................................uggacggagaaGugauaaggg...........   2       1   MF2
..................................................uggacggagaacGgauaaggg...........   1       1   MF2
..................................................uggacggagaacCgauaaggg...........   9       1   MF2
..................................................uggaAggagaacugauaagggc..........   11      1   MF2
..................................................uggacggagaacuAauaagggc..........   109     1   MF2
..................................................uggacggagaGcugauaagggc..........   25      1   MF2
..................................................uggacggaUaacugauaagggc..........   3       1   MF2
..................................................uggacggagaacugauUagggc..........   3       1   MF2
..................................................uggacggGgaacugauaagggc..........   31      1   MF2
..................................................uggacggagaacugauaaAggc..........   21      1   MF2
..................................................uggacggagaacCgauaagggc..........   55      1   MF2
..................................................uggacggagUacugauaagggc..........   14      1   MF2
..................................................uggacggagaacugUuaagggc..........   5       1   MF2
..................................................uggCcggagaacugauaagggc..........   1       1   MF2
..................................................Gggacggagaacugauaagggc..........   6       1   MF2
..................................................uggacggagaacugauaaUggc..........   7       1   MF2
..................................................ugCacggagaacugauaagggc..........   5       1   MF2
..................................................uggacggagaacuCauaagggc..........   2       1   MF2
..................................................uggacggUgaacugauaagggc..........   5       1   MF2
..................................................uUgacggagaacugauaagggc..........   8       1   MF2
..................................................uggacggagaacugauGagggc..........   13      1   MF2
..................................................uggacggagaacAgauaagggc..........   4       1   MF2
..................................................uggaUggagaacugauaagggc..........   30      1   MF2
..................................................uggacggagaacugauaagCgc..........   4       1   MF2
..................................................uggaGggagaacugauaagggc..........   3       1   MF2
..................................................Cggacggagaacugauaagggc..........   44      1   MF2
..................................................uggacggagGacugauaagggc..........   44      1   MF2
..................................................uggacgUagaacugauaagggc..........   6       1   MF2
..................................................uggacggagaacugauaagggG..........   126     1   MF2
..................................................uggacUgagaacugauaagggc..........   7       1   MF2
..................................................uAgacggagaacugauaagggc..........   28      1   MF2
..................................................uCgacggagaacugauaagggc..........   9       1   MF2
..................................................uggacggagaacugCuaagggc..........   1       1   MF2
..................................................uggUcggagaacugauaagggc..........   6       1   MF2
..................................................uggacggagaacugauCagggc..........   22      1   MF2
..................................................uggacggagaacGgauaagggc..........   6       1   MF2
..................................................uggacggagaacugauaaggAc..........   78      1   MF2
..................................................uggacggagaacugauaaCggc..........   2       1   MF2
..................................................uggacggagaacugauaagggc..........   114108  0   MF2
..................................................uggacgAagaacugauaagggc..........   80      1   MF2
..................................................uggacggagaacugauaagggA..........   1954    1   MF2
```

```
             aga-miR-184*
                                                  aga-miR-184
ggugcacucgaacccuuaucauucuuucgccccgugugcuuucuaacaacuggacggagaacugauaagggcccgggucacc

..................................................uggacggagaacugGuaagggc..........   7      1   MF2
..................................................uggacggagaaAugauaagggc..........   61     1   MF2
..................................................uggacggagaaUugauaagggc..........   29     1   MF2
..................................................uggacggagaacugauaagAgc..........   32     1   MF2
..................................................uggacggagaacugauaagggU..........   286    1   MF2
..................................................uggGcggagaacugauaagggc..........   22     1   MF2
..................................................uggacggagaacuUauaagggc..........   5      1   MF2
..................................................uggacggagaacugauaagUgc..........   7      1   MF2
..................................................uggacggagCacugauaagggc..........   22     1   MF2
..................................................uggacggagaacugauaaggUc..........   10     1   MF2
..................................................ugUacggagaacugauaagggc..........   11     1   MF2
..................................................uggacAgagaacugauaagggc..........   37     1   MF2
..................................................uggacCgagaacugauaagggc..........   2      1   MF2
..................................................uggacggagaacugauaUgggc..........   2      1   MF2
..................................................uggacggaAaacugauaagggc..........   8      1   MF2
..................................................uggacggagaacugaAaagggc..........   4      1   MF2
..................................................ugAacggagaacugauaagggc..........   69     1   MF2
..................................................uggacggagaCcugauaagggc..........   11     1   MF2
..................................................uggacggagaacugauaGgggc..........   11     1   MF2
..................................................uggacggagaacugauaaggCc..........   3      1   MF2
..................................................uggacgCagaacugauaagggc..........   4      1   MF2
..................................................uggacggagaacugaCaagggc..........   33     1   MF2
..................................................uggacggCgaacugauaagggc..........   2      1   MF2
..................................................Aggacggagaacugauaagggc..........   15     1   MF2
..................................................uggacggagaacugaGaagggc..........   4      1   MF2
..................................................uggacggagaUcugauaagggc..........   19     1   MF2
..................................................uggacggagaacugauaCgggc..........   2      1   MF2
..................................................uggacggaCaacugauaagggc..........   7      1   MF2
..................................................uggacggagaaGugauaagggc..........   35     1   MF2
..................................................uggacggagaacugauaagggAc.........   2      1   MF2
..................................................uggacggagaacugauaagggUc.........   5      1   MF2
..................................................uggacggagaaUugauaagggcc.........   1      1   MF2
..................................................uggacggagaacugauaagggcU.........   12250  1   MF2
..................................................uggacggagaacugauaagggcA.........   6294   1   MF2
..................................................uggacggagaacugauaagggcG.........   129    1   MF2
..................................................uggacggagaacugauaagggcc.........   391    0   MF2
..................................................uggacggagaacugauaagggccc........   5      0   MF2
..................................................uggacggagaacugauaagggccA........   30     1   MF2
..................................................uggacggagaacugauaagggcAc........   17     1   MF2
..................................................uggacggagaacugauaagggccU........   231    1   MF2
..................................................uggacggagaacugauaagggccG........   5      1   MF2
..................................................uggacggagaacugauaagggcUc........   69     1   MF2
..................................................uggacggagaacugauaagggUccggg.....   1      1   MF2
...................................................ggacggagaacugauaag.............   1      0   MF2
...................................................ggacggagaacugauaagg............   2      0   MF2
...................................................Ugacggagaacugauaagggc..........   2      1   MF2
...................................................ggacggagaacugauaagggc..........   41     0   MF2
...................................................ggacggagaacugauaagggcU.........   4      1   MF2
....................................................gacggagaacugauaagggc..........   3      0   MF2
....................................................gacggagaacugauaagggcc.........   3      0   MF2
....................................................gacggagaacugauaagggccc........   13     0   MF2
.....................................................acggagaacugauaagggcc.........   2      0   MF2
.....................................................acggagaacugauaagggccc........   3      0   MF2

.............ccuuaucauucuuucgccccgugugcuuucuaac...................................   1      0   FW2
...............................................aacuggacggagaacugauaagggc..........   1      0   FW2
.................................................cuggacggagaacugauaa..............   1      0   FW2
.................................................cuggacggagaacugauaag.............   1      0   FW2
.................................................cuggacggagaacugauaagg............   1      0   FW2
.................................................cuggacggagaacugauaaggg...........   3      0   FW2
.................................................cuggacggagaacugauaagggc..........   7      0   FW2
..................................................uggacggagaacugaua...............   62     0   FW2
..................................................uggacggagaacugauaa..............   115    0   FW2
..................................................uggacAgagaacugauaa..............   1      1   FW2
..................................................uggacggagaacugauaag.............   157    0   FW2
..................................................uggacggagaacugauaaA.............   4      1   FW2
..................................................uggacAgagaacugauaagg............   1      1   FW2
..................................................uggacggagaacugauaagg............   284    0   FW2
..................................................ugAacggagaacugauaaggg...........   2      1   FW2
```

```
             aga-miR-184*
                                                  aga-miR-184
ggugcacucgaacccuuaucauucuuucgccccgugugcuuucuaacaacuggacggagaacugauaagggcccgggucacc

..................................................uggacgAagaacugauaaggg...........   1          1          FW2
..................................................uggacggagaacuAauaaggg...........   1          1          FW2
..................................................uggacggagaaAugauaaggg...........   1          1          FW2
..................................................uggacggagaacugauaaggA...........   9          1          FW2
..................................................uggacggagaacugauaaggU...........   1          1          FW2
..................................................uggacggagaacugauaaAgg...........   15         1          FW2
..................................................ugUacggagaacugauaaggg...........   1          1          FW2
..................................................uggacggagaacugauaaggg...........   868        0          FW2
..................................................uggacggagaacugauaaAggc..........   1          1          FW2
..................................................uggacggagaacGgauaagggc..........   1          1          FW2
..................................................uggacggagaacugauaagAgc..........   2          1          FW2
..................................................uggacggagaacugauaaggUc..........   1          1          FW2
..................................................Cggacggagaacugauaagggc..........   4          1          FW2
..................................................uggacggagaacugaGaagggc..........   1          1          FW2
..................................................uggacggagaacugauaagCgc..........   2          1          FW2
..................................................uggacggagaacuAauaagggc..........   9          1          FW2
..................................................uggacggGgaacugauaagggc..........   3          1          FW2
..................................................uggacgAagaacugauaagggc..........   10         1          FW2
..................................................uggacAgagaacugauaagggc..........   1          1          FW2
..................................................uggacggagUacugauaagggc..........   2          1          FW2
..................................................uggacggagaCcugauaagggc..........   1          1          FW2
..................................................uggacggagaUcugauaagggc..........   3          1          FW2
..................................................uggacggagaaUugauaagggc..........   1          1          FW2
..................................................ugAacggagaacugauaagggc..........   5          1          FW2
..................................................uggacggagGacugauaagggc..........   2          1          FW2
..................................................uggacggagaacugauaaggAc..........   10         1          FW2
..................................................uggacggagaacugCuaagggc..........   1          1          FW2
..................................................uggacggagaacugauaaggCc..........   1          1          FW2
..................................................uggacggagCacugauaagggc..........   1          1          FW2
..................................................uggacggagaacugauaUgggc..........   1          1          FW2
..................................................uggacggagaacuCauaagggc..........   1          1          FW2
..................................................uggacggagaacugauaagggU..........   6          1          FW2
..................................................uggacggagaacugauaagggc..........   11300      0          FW2
..................................................uggacggagaacugauaagggA..........   43         1          FW2
..................................................uggacggagaaAugauaagggc..........   4          1          FW2
..................................................uggacUgagaacugauaagggc..........   1          1          FW2
..................................................uggUcggagaacugauaagggc..........   1          1          FW2
..................................................uggacggagaacCgauaagggc..........   5          1          FW2
..................................................uAgacggagaacugauaagggc..........   2          1          FW2
..................................................uggacggagaGcugauaagggc..........   2          1          FW2
..................................................uggGcggagaacugauaagggc..........   4          1          FW2
..................................................uggacgUagaacugauaagggc..........   2          1          FW2
..................................................uUgacggagaacugauaagggc..........   1          1          FW2
..................................................uggaUggagaacugauaagggc..........   3          1          FW2
..................................................uggacggCgaacugauaagggc..........   1          1          FW2
..................................................uggacggagaacugaCaagggc..........   5          1          FW2
..................................................uggacggagaacugauaagggcG.........   2          1          FW2
..................................................uggacggagaacugauaagggcc.........   21         0          FW2
..................................................uggacggagaacugauaagggcA.........   265        1          FW2
..................................................uggacggagaacugauaagggcU.........   438        1          FW2
..................................................uggacggagaacugauaagggccU........   12         1          FW2
..................................................uggacggagaacugauaagggcUc........   2          1          FW2
...................................................ggacggagaacugauaagggc..........   4          0          FW2
....................................................gacggagaacugauaaggg...........   2          0          FW2
....................................................gacggagaacugauaagggc..........   3          0          FW2
....................................................gacggagaacugauaagggcc.........   2          0          FW2
....................................................gacggagaacugauaagggccc........   4          0          FW2
.....................................................acggagaacugauaagggc..........   1          0          FW2
......................................................cggagaacugauaagggc..........   3          0          FW2
.......................................................ggagaacugauaagggc..........   3          0          FW2
.......................................................ggagaacugauaagggcU.........   1          1          FW2
........................................................gagaacugauaagggcU.........   1          1          FW2

.............ccuuaucauucuuucgcccc.................................................   1          0          FF1
.............ccuuaucauucuuucgccccgu...............................................   5          0          FF1
.............ccuuaucauucuuucgccccgugugcuuucuaacaa.................................   1          0          FF1
.............ccuuaucauucuuucgccccgugugcuuucuaacaac................................   1          0          FF1
...................................gugcuuucuaacaacuggacggagaacugauaagggc..........   2          0          FF1
...........................................uaacaacuggacggagaacugauaagggc..........   1          0          FF1
```

aga-miR-184*

aga-miR-184

ggugcacucgaacccuuaucauucuuucgccccgugugcuuucuaacaacuggacggagaacugauaagggcccgggucacc

....Ucuggacggagaacugauaagggc..........	1	1	FF1
....cuggacggagaacugauaa..............	1	0	FF1
....cuggacggagaacugauaagg............	3	0	FF1
....cuggacggagaacugauaaggg...........	2	0	FF1
....cuggacggagaacugauaagggc..........	15	0	FF1
....cuAgacggagaacugauaagggc..........	1	1	FF1
....cuggacggagaacugauaagggU..........	1	1	FF1
....cuggacggagaacugauaagggcU.........	2	1	FF1
....cuggacggagaacugauaagggcA.........	1	1	FF1
....uggacggagaCcugaua...............	1	1	FF1
....uggacggagaacugaua...............	31	0	FF1
....uggacggagaacugauaU..............	1	1	FF1
....uggacggagaacugauaG..............	1	1	FF1
....uggacggagaacugauaa..............	81	0	FF1
....uggacgAagaacugauaag.............	1	1	FF1
....uggaUggagaacugauaag.............	1	1	FF1
....uggacggagaacuAauaag.............	1	1	FF1
....uggacggagaacugauaag.............	172	0	FF1
....uggacggagaacugauCag.............	1	1	FF1
....uggacggagaacugauaaA.............	8	1	FF1
....uggacggagaacugauaagA............	16	1	FF1
....uggacggagaacugauaagU............	3	1	FF1
....uggacggagaacugauaagg............	381	0	FF1
....uggacggGgaacugauaaggg...........	1	1	FF1
....uAgacggagaacugauaaggg...........	3	1	FF1
....uggacggagaacugGuaaggg...........	1	1	FF1
....uggacggagaacugauaaAgg...........	5	1	FF1
....uggacggagaacugauaaggA...........	35	1	FF1
....ugAacggagaacugauaaggg...........	2	1	FF1
....uggacggagaacugCuaaggg...........	1	1	FF1
....uggacggagaCcugauaaggg...........	1	1	FF1
....uggacggagaacugauaaggg...........	1396	0	FF1
....uggacggagaacuAauaaggg...........	2	1	FF1
....uggacggagaacuUauaaggg...........	1	1	FF1
....uggacggagaacugauaaggU...........	1	1	FF1
....uggacggagaacugauaCgggc..........	1	1	FF1
....uggacggagaGcugauaagggc..........	5	1	FF1
....uggacggagaacugauaagggc..........	25961	0	FF1
....uggaUggagaacugauaagggc..........	5	1	FF1
....uCgacggagaacugauaagggc..........	3	1	FF1
....uUgacggagaacugauaagggc..........	1	1	FF1
....uggacggagaacugauaGgggc..........	3	1	FF1
....uggacggagaUcugauaagggc..........	4	1	FF1
....uggacggaAaacugauaagggc..........	2	1	FF1
....uggacggagaacugUuaagggc..........	3	1	FF1
....uggacggagGacugauaagggc..........	4	1	FF1
....uggacggaCaacugauaagggc..........	1	1	FF1
....uAgacggagaacugauaagggc..........	5	1	FF1
....uggacggagCacugauaagggc..........	1	1	FF1
....uggUcggagaacugauaagggc..........	3	1	FF1
....uggacggagaacugaCaagggc..........	9	1	FF1
....uggacggagaacugaAaagggc..........	3	1	FF1
....Cggacggagaacugauaagggc..........	10	1	FF1
....uggacggagaacugGuaagggc..........	5	1	FF1
....uggacggagaaAugauaagggc..........	14	1	FF1
....uggacggagaacugauaagUgc..........	2	1	FF1
....uggacAgagaacugauaagggc..........	10	1	FF1
....uggacggagaacuCauaagggc..........	1	1	FF1
....uggacggagaacugauaagggG..........	3	1	FF1
....uggGcggagaacugauaagggc..........	7	1	FF1
....uggacggagaacCgauaagggc..........	16	1	FF1
....uggacggagaacugauaagggU..........	21	1	FF1
....uggacggagaacugauaaAggc..........	7	1	FF1
....uggaAggagaacugauaagggc..........	1	1	FF1
....uggacggagaacugauUagggc..........	2	1	FF1
....uggacggagaacuAauaagggc..........	15	1	FF1
....uggacggagaacugauaagAgc..........	2	1	FF1
....uggacggagUacugauaagggc..........	6	1	FF1
....uggacgCagaacugauaagggc..........	1	1	FF1

```
             aga-miR-184*
                                                  aga-miR-184
ggugcacucgaacccuuaucauucuuucgccccgugugcuuucuaacaacuggacggagaacugauaagggcccgggucacc

..................................................uggacUgagaacugauaagggc..........  2     1  FF1
..................................................Aggacggagaacugauaagggc..........  7     1  FF1
..................................................uggacggGgaacugauaagggc..........  3     1  FF1
..................................................uggacggagaacugauaagggA..........  325   1  FF1
..................................................uggacggagaaUugauaagggc..........  4     1  FF1
..................................................uggacggagaacugauaaggAc..........  8     1  FF1
..................................................uggacgUagaacugauaagggc..........  3     1  FF1
..................................................ugAacggagaacugauaagggc..........  15    1  FF1
..................................................uggacgAagaacugauaagggc..........  14    1  FF1
..................................................uggacggagaCcugauaagggc..........  5     1  FF1
..................................................uggacggagaacugauGagggc..........  3     1  FF1
..................................................uggacggagaaGugauaagggc..........  8     1  FF1
..................................................uggCcggagaacugauaagggc..........  1     1  FF1
..................................................uggacggagaacugauaaggUc..........  3     1  FF1
..................................................uggacggUgaacugauaagggc..........  1     1  FF1
..................................................uggacggagaacugauaagCgc..........  1     1  FF1
..................................................uggacggagaacugauaaggCc..........  2     1  FF1
..................................................ugUacggagaacugauaagggc..........  3     1  FF1
..................................................uggacggagaacugaAaagggcc.........  1     1  FF1
..................................................uggacggagaacugauaagggcG.........  16    1  FF1
..................................................uggacggagaacugauaaggUcc.........  1     1  FF1
..................................................uAgacggagaacugauaagggcc.........  1     1  FF1
..................................................uggacggagaacugauaagggcA.........  2870  1  FF1
..................................................uggacggagaacugauaagggcU.........  866   1  FF1
..................................................uggacggagaacugauaagggcc.........  142   0  FF1
..................................................uggacggagaacugauaagggcUc........  6     1  FF1
..................................................uggacggagaacugauaagggcAc........  14    1  FF1
..................................................uggacggagaacugauaagggccU........  10    1  FF1
..................................................uggacggagaacugauaagggccA........  14    1  FF1
..................................................uggacggagaacugauaagggccc........  2     0  FF1
..................................................uggacggagaacugauaagggcccC.......  1     1  FF1
...................................................Ugacggagaacugauaagggc..........  1     1  FF1
...................................................ggacggagaacugauaagggc..........  6     0  FF1
....................................................gacggagaacugauaagggccc........  4     0  FF1

.............ccuuaucauucuuucgccc..................................................  1     0  OV1
.............ccuuaucauucuuucgccccAu...............................................  1     1  OV1
.............ccuuaucauucuuucgccccgu...............................................  10    0  OV1
.............ccuuaucauucuuucgccccguU..............................................  1     1  OV1
.............ccuuaucauucuuucgccccgugugcu..........................................  2     0  OV1
.............ccuuaucauucuuucgccccgugugcuuucuaac...................................  1     0  OV1
.............ccuuaucauucuuucgccccgugugcuuucuaaca..................................  1     0  OV1
...............uuaucauucuuucgccccg................................................  1     0  OV1
...........................................uaacaacuggacggagaacugauaagggc..........  1     0  OV1
............................................aacaacuggacggagaacugauaaggg...........  1     0  OV1
...............................................aUcuggacggagaacugauaagggc..........  2     1  OV1
................................................Ucuggacggagaacugauaaggg...........  1     1  OV1
.................................................cuggacggagaacugauaa..............  2     0  OV1
.................................................cuggacggagaacugauaaA.............  1     1  OV1
.................................................cuggacggagaacugauaag.............  4     0  OV1
.................................................cuggacggagaacugauaagg............  6     0  OV1
.................................................cuggacggagaacugauaaggg...........  5     0  OV1
.................................................cuggacggagaacugauaagggA..........  1     1  OV1
.................................................Uuggacggagaacugauaagggc..........  3     1  OV1
.................................................cuggacggagaacugauaagggc..........  28    0  OV1
.................................................cuggacggagaacugauaagggcU.........  1     1  OV1
..................................................uggacggagaaAugaua...............  1     1  OV1
..................................................uggacggagaacugaua...............  447   0  OV1
..................................................uggacggagaacugauG...............  1     1  OV1
..................................................uggacggagaacugGua...............  1     1  OV1
..................................................uggacggagaacuAaua...............  1     1  OV1
..................................................uggacggagaacugUua...............  1     1  OV1
..................................................uggacggagaCcugauaa..............  1     1  OV1
..................................................uggacgAagaacugauaa..............  1     1  OV1
..................................................uggacggagaacugauaG..............  1     1  OV1
..................................................uggacggagaacugauaU..............  2     1  OV1
..................................................uggacggagaacugauaa..............  739   0  OV1
..................................................uggacggagGacugauaa..............  1     1  OV1
..................................................uggacggagaacugauaaC.............  1     1  OV1
```

```
             aga-miR-184*
                                                  aga-miR-184
ggugcacucgaacccuuaucauucuuucgccccgugugcuuucuaacaacuggacggagaacugauaagggcccgggucacc

..................................................uggacggagCacugauaag.............   1          1          OV1
..................................................uggacggagaacCgauaag.............   3          1          OV1
..................................................uggacggagaaUugauaag.............   1          1          OV1
..................................................uggacggagaacugauaaU.............   2          1          OV1
..................................................ugCacggagaacugauaag.............   1          1          OV1
..................................................uggacggagaacugauaag.............   1502       0          OV1
..................................................uggaGggagaacugauaag.............   1          1          OV1
..................................................uggacAgagaacugauaag.............   1          1          OV1
..................................................ugAacggagaacugauaag.............   2          1          OV1
..................................................Aggacggagaacugauaag.............   1          1          OV1
..................................................uggacggagaacugauaaA.............   124        1          OV1
..................................................uggacggagaacuAauaag.............   1          1          OV1
..................................................uggacggagaacugaCaag.............   1          1          OV1
..................................................uggacggagaacGgauaag.............   1          1          OV1
..................................................ugAacggagaacugauaagg............   2          1          OV1
..................................................uggacggagaacugauaagU............   5          1          OV1
..................................................uggacggagaacugauUagg............   1          1          OV1
..................................................uggacggagaaAugauaagg............   1          1          OV1
..................................................uggacggagaacugGuaagg............   1          1          OV1
..................................................uggacggaAaacugauaagg............   4          1          OV1
..................................................uggacggagaacugauaaAg............   2          1          OV1
..................................................uggacggagaacuAauaagg............   1          1          OV1
..................................................uggacggagaacugaCaagg............   1          1          OV1
..................................................uggacggagaacCgauaagg............   1          1          OV1
..................................................uggacggagaacugauaagg............   2968       0          OV1
..................................................uggacAgagaacugauaagg............   1          1          OV1
..................................................Cggacggagaacugauaagg............   1          1          OV1
..................................................uggacgAagaacugauaagg............   2          1          OV1
..................................................uggacggagaacugauaagA............   86         1          OV1
..................................................uggacggGgaacugauaagg............   1          1          OV1
..................................................uggacggagaacugaAaagg............   1          1          OV1
..................................................uggacgUagaacugauaagg............   1          1          OV1
..................................................uggacggagaacugauCagg............   1          1          OV1
..................................................uggacggagaacGgauaagg............   1          1          OV1
..................................................uggacggagaaUugauaagg............   3          1          OV1
..................................................uggacggagaacugauaagC............   2          1          OV1
..................................................uggacggagaGcugauaaggg...........   1          1          OV1
..................................................ugUacggagaacugauaaggg...........   1          1          OV1
..................................................uggacggagaacugauaaAgg...........   27         1          OV1
..................................................uggacggagaacCgauaaggg...........   5          1          OV1
..................................................uggacggagaacugauaaggg...........   8397       0          OV1
..................................................uggacggagaacuUauaaggg...........   2          1          OV1
..................................................uggUcggagaacugauaaggg...........   1          1          OV1
..................................................uggacggagaacugauaaggA...........   356        1          OV1
..................................................ugAacggagaacugauaaggg...........   4          1          OV1
..................................................uggacAgagaacugauaaggg...........   5          1          OV1
..................................................uggacggagaaAugauaaggg...........   2          1          OV1
..................................................uAgacggagaacugauaaggg...........   6          1          OV1
..................................................uggacggagaCcugauaaggg...........   1          1          OV1
..................................................uggacggGgaacugauaaggg...........   1          1          OV1
..................................................uggacggagaacuAauaaggg...........   6          1          OV1
..................................................uggacggagaUcugauaaggg...........   1          1          OV1
..................................................uggaUggagaacugauaaggg...........   3          1          OV1
..................................................uggacggagaacugaCaaggg...........   2          1          OV1
..................................................uggacggagUacugauaaggg...........   1          1          OV1
..................................................uggacggaCaacugauaaggg...........   1          1          OV1
..................................................uggGcggagaacugauaaggg...........   1          1          OV1
..................................................Cggacggagaacugauaaggg...........   3          1          OV1
..................................................uggacggagaacugauGaggg...........   1          1          OV1
..................................................uggacggagaaGugauaaggg...........   3          1          OV1
..................................................uggacggagaacugauaaggU...........   12         1          OV1
..................................................uggacggagGacugauaaggg...........   4          1          OV1
..................................................uggacggagaacugauaaggC...........   8          1          OV1
..................................................uggacgAagaacugauaaggg...........   7          1          OV1
..................................................uggacggagaacugauaagAg...........   6          1          OV1
..................................................uggacggagaacugauUaggg...........   2          1          OV1
..................................................uggacggagaacugUuaaggg...........   1          1          OV1
..................................................uggacggagaacugGuaaggg...........   2          1          OV1
..................................................uUgacggagaacugauaaggg...........   1          1          OV1
```

```
             aga-miR-184*
                                                  aga-miR-184
ggugcacucgaacccuuaucauucuuucgccccgugugcuuucuaacaacuggacggagaacugauaagggcccgggucacc

..................................................uggacggagaUcugauaagggc..........   17      1     OV1
..................................................ugUacggagaacugauaagggc..........   9       1     OV1
..................................................uggacggGgaacugauaagggc..........   7       1     OV1
..................................................uggacggagaacugauaaggAc..........   53      1     OV1
..................................................uggUcggagaacugauaagggc..........   6       1     OV1
..................................................uggacggagaacugauaUgggc..........   5       1     OV1
..................................................uCgacggagaacugauaagggc..........   2       1     OV1
..................................................uggacggaAaacugauaagggc..........   8       1     OV1
..................................................uggacggagaaAugauaagggc..........   40      1     OV1
..................................................uggacCgagaacugauaagggc..........   5       1     OV1
..................................................uggacggagaacGgauaagggc..........   7       1     OV1
..................................................uggacggagaacugauaagggG..........   103     1     OV1
..................................................uggacggagaacugaAaagggc..........   3       1     OV1
..................................................uggaUggagaacugauaagggc..........   32      1     OV1
..................................................uggacggagaacAgauaagggc..........   2       1     OV1
..................................................uggacggagaacugauUagggc..........   4       1     OV1
..................................................uggacggUgaacugauaagggc..........   5       1     OV1
..................................................uggacggCgaacugauaagggc..........   2       1     OV1
..................................................uggacggagaacugUuaagggc..........   5       1     OV1
..................................................uggacggagaacugauaGgggc..........   12      1     OV1
..................................................uggCcggagaacugauaagggc..........   2       1     OV1
..................................................uggacggagaaGugauaagggc..........   37      1     OV1
..................................................uggacggagaacugauaagUgc..........   9       1     OV1
..................................................uggacggagaacugauaagggc..........   87493   0     OV1
..................................................Cggacggagaacugauaagggc..........   27      1     OV1
..................................................uggacggagaacugauaaUggc..........   3       1     OV1
..................................................uggacggaUaacugauaagggc..........   2       1     OV1
..................................................uggacgAagaacugauaagggc..........   74      1     OV1
..................................................ugCacggagaacugauaagggc..........   2       1     OV1
..................................................uggacggagaacugauGagggc..........   9       1     OV1
..................................................uggacggagaacugauaagggU..........   112     1     OV1
..................................................uggacggagaaUugauaagggc..........   34      1     OV1
..................................................uUgacggagaacugauaagggc..........   12      1     OV1
..................................................uggacggagaacugauaaAggc..........   12      1     OV1
..................................................uggacggagaacCgauaagggc..........   40      1     OV1
..................................................uAgacggagaacugauaagggc..........   29      1     OV1
..................................................uggGcggagaacugauaagggc..........   15      1     OV1
..................................................uggacggagaacuUauaagggc..........   7       1     OV1
..................................................uggacUgagaacugauaagggc..........   9       1     OV1
..................................................uggacggagaacugGuaagggc..........   6       1     OV1
..................................................uggacggagaacuCauaagggc..........   4       1     OV1
..................................................uggacggagaacugauaagAgc..........   27      1     OV1
..................................................uggacggagaGcugauaagggc..........   19      1     OV1
..................................................uggacggagaacugaCaagggc..........   22      1     OV1
..................................................uggacggagaacugauaagggA..........   1886    1     OV1
..................................................uggaAggagaacugauaagggc..........   4       1     OV1
..................................................uggacggagaCcugauaagggc..........   15      1     OV1
..................................................Aggacggagaacugauaagggc..........   10      1     OV1
..................................................uggacAgagaacugauaagggc..........   23      1     OV1
..................................................uggacggagaacugauaaggUc..........   12      1     OV1
..................................................uggacggagUacugauaagggc..........   17      1     OV1
..................................................uggacgCagaacugauaagggc..........   8       1     OV1
..................................................uggacggagCacugauaagggc..........   15      1     OV1
..................................................uggacggagaacugaGaagggc..........   2       1     OV1
..................................................uggacgUagaacugauaagggc..........   2       1     OV1
..................................................uggacggagGacugauaagggc..........   19      1     OV1
..................................................uggacggagaacugauaagCgc..........   3       1     OV1
..................................................uggacggagaacugCuaagggc..........   1       1     OV1
..................................................ugAacggagaacugauaagggc..........   50      1     OV1
..................................................uggacggagaacugauaCgggc..........   2       1     OV1
..................................................uggacggagaacuAauaagggc..........   80      1     OV1
..................................................uggacggagaacugauCagggc..........   6       1     OV1
..................................................uggaGggagaacugauaagggc..........   1       1     OV1
..................................................uggacggaCaacugauaagggc..........   4       1     OV1
..................................................Gggacggagaacugauaagggc..........   7       1     OV1
..................................................uggacggagaacugauaaCggc..........   1       1     OV1
..................................................uggacggagaacugauaagggcG.........   56      1     OV1
..................................................uggacggagaacugauaagggcU.........   5122    1     OV1
..................................................uggacggagaacugauaagggGc.........   1       1     OV1
```

```
             aga-miR-184*
                                                  aga-miR-184
ggugcacucgaacccuuaucauucuuucgccccgugugcuuucuaacaacuggacggagaacugauaagggcccgggucacc

..................................................uggacggagaacugauaagggAc.........   7      1   OV1
..................................................uggacggagCacugauaagggcc.........   1      1   OV1
..................................................uAgacggagaacugauaagggcc.........   1      1   OV1
..................................................uggacggagaacugauaagggcc.........   206    0   OV1
..................................................uggacggagaacugauaagggcA.........   9113   1   OV1
..................................................uggacggagaacugauaagggcAc........   60     1   OV1
..................................................uggacggagaacugauaagggccc........   2      0   OV1
..................................................uggacggagaacugauaagggccA........   23     1   OV1
..................................................uggacggagaacugauaagggccU........   47     1   OV1
..................................................uggacggagaacugauaagggcUc........   10     1   OV1
...................................................Ugacggagaacugauaagg............   1      1   OV1
...................................................ggacggagaacugauaagg............   1      0   OV1
...................................................ggacggagaacugauaaggg...........   4      0   OV1
...................................................ggacggagaacugauaaAgg...........   1      1   OV1
...................................................ggacggagaacugauaagggc..........   25     0   OV1
...................................................Ugacggagaacugauaagggc..........   1      1   OV1
...................................................ggacggagaacugauaagggcG.........   1      1   OV1
...................................................ggacggagaacugauaagggcA.........   5      1   OV1
...................................................ggacggagaacugauaagggcU.........   4      1   OV1
....................................................gacggagaacugauaagggc..........   1      0   OV1
....................................................gacggagaacugauaagggcc.........   3      0   OV1
.....................................................acggagaacugauaagggcc.........   1      0   OV1
.....................................................acggagaacugauaagggccc........   1      0   OV1

.............ccuuaucauucuuucgccccU................................................   1      1   MF1
.............ccuuaucauucuuucgccccgu...............................................   4      0   MF1
................................................Ucuggacggagaacugauaagggc..........   1      1   MF1
.................................................cuggacggagaacugauaa..............   1      0   MF1
.................................................cuggacggagaacugauaag.............   3      0   MF1
.................................................cuggacggagaacugauaagg............   3      0   MF1
.................................................cuggacggagaacugauaaggg...........   7      0   MF1
.................................................cuggacggagaacugauaagggc..........   5      0   MF1
.................................................cuggacggagaacugauaagggU..........   2      1   MF1
..................................................uggacggagaacugaua...............   331    0   MF1
..................................................uggacggagaacugauU...............   3      1   MF1
..................................................uggacggagaacugauC...............   1      1   MF1
..................................................uggaUggagaacugauaa..............   1      1   MF1
..................................................uggacggagaacugaAaa..............   1      1   MF1
..................................................uggacggagaacuAauaa..............   1      1   MF1
..................................................uggacgAagaacugauaa..............   2      1   MF1
..................................................uAgacggagaacugauaa..............   1      1   MF1
..................................................uggacggagaacugauaa..............   568    0   MF1
..................................................uggacggagaacugauaU..............   4      1   MF1
..................................................uggacggagaacCgauaag.............   1      1   MF1
..................................................uggacggagaacugaCaag.............   1      1   MF1
..................................................Cggacggagaacugauaag.............   2      1   MF1
..................................................uggacggagCacugauaag.............   1      1   MF1
..................................................uggacAgagaacugauaag.............   1      1   MF1
..................................................uggacggagaacugauaag.............   1051   0   MF1
..................................................uggacggagaaAugauaag.............   4      1   MF1
..................................................uggacggagaacugauaaA.............   105    1   MF1
..................................................uggacggagaacuAauaag.............   1      1   MF1
..................................................ugAacggagaacugauaag.............   1      1   MF1
..................................................uggacggagaacugauaaU.............   11     1   MF1
..................................................uggacgAagaacugauaagg............   1      1   MF1
..................................................uggacggagGacugauaagg............   1      1   MF1
..................................................uggaGggagaacugauaagg............   1      1   MF1
..................................................uggacggagaCcugauaagg............   2      1   MF1
..................................................uggacggagaaAugauaagg............   1      1   MF1
..................................................uggacggagaacuAauaagg............   1      1   MF1
..................................................uggacggagaacGgauaagg............   1      1   MF1
..................................................uggacgCagaacugauaagg............   1      1   MF1
..................................................uggacggagaacugauaagC............   1      1   MF1
..................................................uggacggagaacugauaagg............   1481   0   MF1
..................................................ugUacggagaacugauaagg............   1      1   MF1
..................................................uggacggagaacugauaagA............   49     1   MF1
..................................................uggacggagaacugauUagg............   1      1   MF1
..................................................ugAacggagaacugauaagg............   1      1   MF1
..................................................uggacggagaacugauaagU............   8      1   MF1
```

```
             aga-miR-184*
                                                  aga-miR-184
ggugcacucgaacccuuaucauucuuucgccccgugugcuuucuaacaacuggacggagaacugauaagggcccgggucacc

..................................................uggacggagaaUugauaagg............   2      1   MF1
..................................................uggacggagaacugauaagAg...........   1      1   MF1
..................................................uggacggagaGcugauaaggg...........   1      1   MF1
..................................................uggacgAagaacugauaaggg...........   1      1   MF1
..................................................ugAacggagaacugauaaggg...........   2      1   MF1
..................................................uggacggagaacugauaaUgg...........   1      1   MF1
..................................................Aggacggagaacugauaaggg...........   1      1   MF1
..................................................uggacggagaacugauaaggU...........   13     1   MF1
..................................................uggacggagaacugauaaggC...........   4      1   MF1
..................................................uggacggagaacugauaUggg...........   1      1   MF1
..................................................uggacggagaacugauaaggA...........   147    1   MF1
..................................................uggacAgagaacugauaaggg...........   3      1   MF1
..................................................uggacggagaacugauaaAgg...........   6      1   MF1
..................................................uggacggagaacugauCaggg...........   1      1   MF1
..................................................uggacggCgaacugauaaggg...........   1      1   MF1
..................................................uggacggagGacugauaaggg...........   1      1   MF1
..................................................uggacggagaCcugauaaggg...........   1      1   MF1
..................................................uggacggagaacugauaaggg...........   2333   0   MF1
..................................................uggacggagaaAugauaaggg...........   1      1   MF1
..................................................Aggacggagaacugauaagggc..........   6      1   MF1
..................................................uggacggagaacugaAaagggc..........   1      1   MF1
..................................................Gggacggagaacugauaagggc..........   1      1   MF1
..................................................uggacggagaacugauaagggc..........   21961  0   MF1
..................................................Cggacggagaacugauaagggc..........   5      1   MF1
..................................................uggGcggagaacugauaagggc..........   7      1   MF1
..................................................uggacggagaCcugauaagggc..........   4      1   MF1
..................................................uggacggGgaacugauaagggc..........   7      1   MF1
..................................................ugAacggagaacugauaagggc..........   16     1   MF1
..................................................uggacggCgaacugauaagggc..........   1      1   MF1
..................................................uggacggagaaAugauaagggc..........   16     1   MF1
..................................................uggacggagaacugauaagggU..........   29     1   MF1
..................................................uggacggagaacuCauaagggc..........   3      1   MF1
..................................................uggacggagaacugauGagggc..........   2      1   MF1
..................................................uggacggagaacugauaagggG..........   22     1   MF1
..................................................uggacggagaacugauaaggUc..........   1      1   MF1
..................................................uggacggaUaacugauaagggc..........   2      1   MF1
..................................................ugUacggagaacugauaagggc..........   2      1   MF1
..................................................uggacgAagaacugauaagggc..........   17     1   MF1
..................................................uggacAgagaacugauaagggc..........   10     1   MF1
..................................................uggacggagCacugauaagggc..........   1      1   MF1
..................................................uggacggagaacAgauaagggc..........   1      1   MF1
..................................................uggacggagaacugauaaggAc..........   16     1   MF1
..................................................uggacggagaacugCuaagggc..........   1      1   MF1
..................................................uggacggagaacugauUagggc..........   2      1   MF1
..................................................uggacggagaacuAauaagggc..........   17     1   MF1
..................................................ugCacggagaacugauaagggc..........   1      1   MF1
..................................................uggaAggagaacugauaagggc..........   3      1   MF1
..................................................uAgacggagaacugauaagggc..........   6      1   MF1
..................................................uggacgCagaacugauaagggc..........   1      1   MF1
..................................................uggacggagaacugauaagggA..........   350    1   MF1
..................................................uggacggagGacugauaagggc..........   6      1   MF1
..................................................uggacggagaacugauaGgggc..........   1      1   MF1
..................................................uggacggagaacCgauaagggc..........   16     1   MF1
..................................................uggacggagaaUugauaagggc..........   12     1   MF1
..................................................uggacggagaacugauaUgggc..........   1      1   MF1
..................................................uggacggagaacugauaaAggc..........   2      1   MF1
..................................................uggacggagaacugauaaggCc..........   1      1   MF1
..................................................uggacggagaacugGuaagggc..........   3      1   MF1
..................................................uggacggaCaacugauaagggc..........   3      1   MF1
..................................................uggacggagaUcugauaagggc..........   4      1   MF1
..................................................uUgacggagaacugauaagggc..........   1      1   MF1
..................................................uggacggagaaGugauaagggc..........   11     1   MF1
..................................................uggacggUgaacugauaagggc..........   2      1   MF1
..................................................uggaUggagaacugauaagggc..........   8      1   MF1
..................................................uggacggagaacugaCaagggc..........   7      1   MF1
..................................................uggacggagUacugauaagggc..........   1      1   MF1
..................................................uggUcggagaacugauaagggc..........   1      1   MF1
..................................................uggacggaAaacugauaagggc..........   5      1   MF1
..................................................uggacggagaacugauaagAgc..........   5      1   MF1
```

```
             aga-miR-184*
                                                  aga-miR-184
ggugcacucgaacccuuaucauucuuucgccccgugugcuuucuaacaacuggacggagaacugauaagggcccgggucacc

..................................................uggacggagaGcugauaagggc..........   5      1    MF1
..................................................uggacggagaacugauaaggAcc.........   1      1    MF1
..................................................uggacggagaacugauaagggcA.........   910    1    MF1
..................................................uggacggagaacugauaagggcU.........   1824   1    MF1
..................................................uggacggagaacugauaagggcc.........   53     0    MF1
..................................................uggacggagaacugauaagggcG.........   21     1    MF1
..................................................uggacggagaacugauaagggcAc........   1      1    MF1
..................................................uggacggagaacugauaagggccA........   8      1    MF1
..................................................uggacggagaacugauaagggccU........   37     1    MF1
..................................................uggacggagaacugauaagggcUc........   9      1    MF1
..................................................uggacggagaacugauaagggcccCgg.....   1      1    MF1
...................................................ggacggagaacugauaaggg...........   2      0    MF1
...................................................ggacggagaacugauaagggc..........   6      0    MF1
...................................................ggacggagaacugauaagggcA.........   1      1    MF1
...................................................ggacggagaacugauaagggcG.........   1      1    MF1
...................................................ggacggagaacugauaagggcU.........   2      1    MF1
....................................................gacggagaacugauaagggcc.........   2      0    MF1
....................................................gacggagaacugauaagggccc........   1      0    MF1
.....................................................acggagaacugauaagggcc.........   1      0    MF1
.....................................................acggagaacugauaagggccc........   3      0    MF1

.............ccuuaucauucuuucgccccgugugcuuucuaacaac................................   3      0    BF2
...................................gugcuuucuaacaacuggacggagaacugauaagggc..........   4      0    BF2
.................................................cuggacggagaacugauaagg............   2      0    BF2
.................................................Guggacggagaacugauaaggg...........   1      1    BF2
.................................................cuggacggagaacugauaagggc..........   17     0    BF2
.................................................Auggacggagaacugauaagggc..........   3      1    BF2
.................................................Guggacggagaacugauaagggc..........   11     1    BF2
.................................................cuggacggagaacugauaagggcA.........   1      1    BF2
..................................................uggacggagaacugaua...............   88     0    BF2
..................................................uggacgAagaacugaua...............   1      1    BF2
..................................................uggacggagaacugauaU..............   2      1    BF2
..................................................uggacggagaacugauaa..............   154    0    BF2
..................................................uggacggagaacugaCaa..............   1      1    BF2
..................................................uggacggagaacugauaag.............   253    0    BF2
..................................................uggacggagaacugauaaA.............   27     1    BF2
..................................................uggacggagaacugauaagA............   16     1    BF2
..................................................ugAacggagaacugauaagg............   1      1    BF2
..................................................uggacgAagaacugauaagg............   1      1    BF2
..................................................uggacggagaacugauaagg............   396    0    BF2
..................................................Cggacggagaacugauaagg............   2      1    BF2
..................................................uggacggagaacugauaagU............   1      1    BF2
..................................................uggacggagaacCgauaaggg...........   1      1    BF2
..................................................uggacggagaacugauaaggU...........   3      1    BF2
..................................................uggacggagaacugauaaggA...........   35     1    BF2
..................................................Cggacggagaacugauaaggg...........   1      1    BF2
..................................................uggacggagaaAugauaaggg...........   1      1    BF2
..................................................uggacgAagaacugauaaggg...........   1      1    BF2
..................................................uggacggagaaUugauaaggg...........   1      1    BF2
..................................................uUgacggagaacugauaaggg...........   1      1    BF2
..................................................uggacggagaacugauaagUg...........   1      1    BF2
..................................................uggacggagaacugauaaAgg...........   9      1    BF2
..................................................uggacggagaacugauaaggg...........   1305   0    BF2
..................................................uggacggagaacuAauaaggg...........   1      1    BF2
..................................................ugUacggagaacugauaagggc..........   2      1    BF2
..................................................uggacggagaacugauaagggG..........   3      1    BF2
..................................................Gggacggagaacugauaagggc..........   2      1    BF2
..................................................uggacggagaacugaGaagggc..........   2      1    BF2
..................................................uggacggagaUcugauaagggc..........   6      1    BF2
..................................................Cggacggagaacugauaagggc..........   6      1    BF2
..................................................uggacggagaacugauaGgggc..........   6      1    BF2
..................................................uggacggagaGcugauaagggc..........   7      1    BF2
..................................................Aggacggagaacugauaagggc..........   3      1    BF2
..................................................uggacggagaacugauaaggCc..........   1      1    BF2
..................................................uggacggagaacugauaagggU..........   21     1    BF2
..................................................uggacggagaacugauaaCggc..........   2      1    BF2
..................................................uggacggUgaacugauaagggc..........   1      1    BF2
..................................................uggacggagaacugauaagUgc..........   2      1    BF2
..................................................uggacggagaaGugauaagggc..........   11     1    BF2
```

```
             aga-miR-184*
                                                  aga-miR-184
ggugcacucgaacccuuaucauucuuucgccccgugugcuuucuaacaacuggacggagaacugauaagggcccgggucacc

..................................................uggacggagaacugauaaggAc..........   17      1   BF2
..................................................uggacggagaacuAauaagggc..........   24      1   BF2
..................................................uggacggaUaacugauaagggc..........   2       1   BF2
..................................................uggacggagaacCgauaagggc..........   16      1   BF2
..................................................uggacggagaacugauaCgggc..........   2       1   BF2
..................................................uggacggagGacugauaagggc..........   5       1   BF2
..................................................uggacggagaacugauGagggc..........   3       1   BF2
..................................................uCgacggagaacugauaagggc..........   3       1   BF2
..................................................uggacggagaacugauaagCgc..........   2       1   BF2
..................................................uggacggagaaUugauaagggc..........   7       1   BF2
..................................................uggacggagaacGgauaagggc..........   1       1   BF2
..................................................uggacUgagaacugauaagggc..........   8       1   BF2
..................................................uggacgUagaacugauaagggc..........   6       1   BF2
..................................................uggacggagaacAgauaagggc..........   3       1   BF2
..................................................uggaAggagaacugauaagggc..........   3       1   BF2
..................................................uggacggagaacugaCaagggc..........   9       1   BF2
..................................................uggacggagaacugauaagAgc..........   6       1   BF2
..................................................uggacggagaacugauaaAggc..........   4       1   BF2
..................................................uggacggagaacugUuaagggc..........   1       1   BF2
..................................................uggGcggagaacugauaagggc..........   7       1   BF2
..................................................uggaUggagaacugauaagggc..........   5       1   BF2
..................................................uUgacggagaacugauaagggc..........   4       1   BF2
..................................................uggacggagUacugauaagggc..........   2       1   BF2
..................................................uAgacggagaacugauaagggc..........   4       1   BF2
..................................................uggacggagaacugauaagggc..........   29592   0   BF2
..................................................uggacggaCaacugauaagggc..........   1       1   BF2
..................................................uggacAgagaacugauaagggc..........   6       1   BF2
..................................................uggacgAagaacugauaagggc..........   18      1   BF2
..................................................uggacggagaCcugauaagggc..........   7       1   BF2
..................................................uggacggagCacugauaagggc..........   1       1   BF2
..................................................uggacggagaacugGuaagggc..........   2       1   BF2
..................................................uggacggaAaacugauaagggc..........   1       1   BF2
..................................................uggacggagaaAugauaagggc..........   14      1   BF2
..................................................ugAacggagaacugauaagggc..........   19      1   BF2
..................................................uggacggagaacugauaaUggc..........   5       1   BF2
..................................................uggUcggagaacugauaagggc..........   1       1   BF2
..................................................uggacggagaacugauaaggUc..........   1       1   BF2
..................................................uggacggagaacugauaagggA..........   283     1   BF2
..................................................uggacgCagaacugauaagggc..........   1       1   BF2
..................................................ugCacggagaacugauaagggc..........   1       1   BF2
..................................................uggacggGgaacugauaagggc..........   4       1   BF2
..................................................uggacggagaacugauaagggcG.........   29      1   BF2
..................................................uggacggagaacugauaagggcU.........   743     1   BF2
..................................................uggacggagaacugauaagggcc.........   146     0   BF2
..................................................uggacggagaacugauaagggcA.........   2478    1   BF2
..................................................uggacggagaacugauaagggccG........   1       1   BF2
..................................................uggacggagaacugauaagggcUc........   3       1   BF2
..................................................uggacggagaacugauaagggccU........   16      1   BF2
..................................................uggacggagaacugauaagggccA........   16      1   BF2
..................................................uggacggagaacugauaagggcAc........   14      1   BF2
...................................................ggacggagaacugauaagggc..........   17      0   BF2
...................................................ggacggagaacugauaagggcA.........   1       1   BF2
....................................................gacggagaacugauaagggc..........   4       0   BF2
....................................................gacggagaacugauaagggccc........   2       0   BF2
.....................................................acggagaacugauaagggcA.........   2       1   BF2
.....................................................acggagaacugauaagggccc........   2       0   BF2
.......................................................ggagaacugauaagggc..........   3       0   BF2
........................................................gagaacugauaagggcA.........   2       1   BF2

.............ccuuaucauucuuucgcccc.................................................   1       0   BF1
.............ccuuaucauucuuucgccccgu...............................................   5       0   BF1
.............ccuuaucauucuuucgccccgugugcuuucuaacaa.................................   1       0   BF1
.............ccuuaucauucuuucgccccgugugcuuucuaacaac................................   1       0   BF1
...................................gugcuuucuaacaacuggacggagaacugauaagggc..........   2       0   BF1
...........................................uaacaacuggacggagaacugauaagggc..........   1       0   BF1
................................................Ucuggacggagaacugauaagggc..........   1       1   BF1
.................................................cuggacggagaacugauaa..............   1       0   BF1
.................................................cuggacggagaacugauaagg............   3       0   BF1
.................................................cuggacggagaacugauaaggg...........   2       0   BF1
```

```
             aga-miR-184*
                                                    aga-miR-184
ggugcacucgaacccuuaucauucuuucgccccgugugcuuucuaacaacuggacggagaacugauaagggcccgggucacc

.................................................cuggacggagaacugauaagggU..........   1      1      BF1
.................................................cuAgacggagaacugauaagggc..........   1      1      BF1
.................................................cuggacggagaacugauaagggc..........   15     0      BF1
.................................................cuggacggagaacugauaagggcU.........   2      1      BF1
.................................................cuggacggagaacugauaagggcA.........   1      1      BF1
..................................................uggacggagaacugaua...............   31     0      BF1
..................................................uggacggagaCcugaua...............   1      1      BF1
..................................................uggacggagaacugauaG..............   1      1      BF1
..................................................uggacggagaacugauaa..............   78     0      BF1
..................................................uggacggagaacugauaU..............   1      1      BF1
..................................................uggacggagaacugauCag.............   1      1      BF1
..................................................uggacggagaacuAauaag.............   1      1      BF1
..................................................uggacggagaacugauaaA.............   8      1      BF1
..................................................uggacgAagaacugauaag.............   1      1      BF1
..................................................uggaUggagaacugauaag.............   1      1      BF1
..................................................uggacggagaacugauaag.............   171    0      BF1
..................................................uggacggagaacugauaagA............   16     1      BF1
..................................................uggacggagaacugauaagg............   378    0      BF1
..................................................uggacggagaacugauaagU............   1      1      BF1
..................................................uAgacggagaacugauaaggg...........   3      1      BF1
..................................................uggacggGgaacugauaaggg...........   1      1      BF1
..................................................ugAacggagaacugauaaggg...........   2      1      BF1
..................................................uggacggagaacugGuaaggg...........   1      1      BF1
..................................................uggacggagaacugauaaAgg...........   5      1      BF1
..................................................uggacggagaacuUauaaggg...........   1      1      BF1
..................................................uggacggagaacugCuaaggg...........   1      1      BF1
..................................................uggacggagaacugauaaggg...........   1363   0      BF1
..................................................uggacggagaacuAauaaggg...........   2      1      BF1
..................................................uggacggagaCcugauaaggg...........   1      1      BF1
..................................................uggacggagaacugauaaggA...........   35     1      BF1
..................................................uggacggagaacugauaaggU...........   1      1      BF1
..................................................uggacggaCaacugauaagggc..........   1      1      BF1
..................................................Aggacggagaacugauaagggc..........   7      1      BF1
..................................................uggCcggagaacugauaagggc..........   1      1      BF1
..................................................uggacggagaacugauaaggAc..........   7      1      BF1
..................................................uggacggagaGcugauaagggc..........   5      1      BF1
..................................................uggacggagCacugauaagggc..........   1      1      BF1
..................................................uggacggagaacugauaaggUc..........   3      1      BF1
..................................................uggacggagaacuAauaagggc..........   15     1      BF1
..................................................uggacggagaacugauaagggG..........   3      1      BF1
..................................................uggacggagaacugauaagAgc..........   2      1      BF1
..................................................uggacgUagaacugauaagggc..........   3      1      BF1
..................................................uggacggagaacugauaagUgc..........   2      1      BF1
..................................................uggacggagaacugGuaagggc..........   5      1      BF1
..................................................uggacAgagaacugauaagggc..........   9      1      BF1
..................................................uggacggaAaacugauaagggc..........   2      1      BF1
..................................................uggacUgagaacugauaagggc..........   2      1      BF1
..................................................uggacggagaacugauaagggU..........   21     1      BF1
..................................................uggacgCagaacugauaagggc..........   1      1      BF1
..................................................uggGcggagaacugauaagggc..........   7      1      BF1
..................................................Cggacggagaacugauaagggc..........   10     1      BF1
..................................................uggacggagaUcugauaagggc..........   4      1      BF1
..................................................uAgacggagaacugauaagggc..........   5      1      BF1
..................................................uggacggagaacugauaCgggc..........   1      1      BF1
..................................................uggaUggagaacugauaagggc..........   5      1      BF1
..................................................uggaAggagaacugauaagggc..........   1      1      BF1
..................................................uggacggagaCcugauaagggc..........   5      1      BF1
..................................................uggacggagaaUugauaagggc..........   4      1      BF1
..................................................ugAacggagaacugauaagggc..........   15     1      BF1
..................................................uCgacggagaacugauaagggc..........   3      1      BF1
..................................................uggacggagaacugauaagCgc..........   1      1      BF1
..................................................uggacggagaacugauaaAggc..........   7      1      BF1
..................................................uggacggagGacugauaagggc..........   4      1      BF1
..................................................uggacgAagaacugauaagggc..........   14     1      BF1
..................................................uggacggagaacugUuaagggc..........   3      1      BF1
..................................................uggacggagaacugaAaagggc..........   3      1      BF1
..................................................uggacggagaacugaCaagggc..........   9      1      BF1
..................................................uggacggagaaAugauaagggc..........   14     1      BF1
..................................................ugUacggagaacugauaagggc..........   3      1      BF1
```

```
             aga-miR-184*
                                                  aga-miR-184
ggugcacucgaacccuuaucauucuuucgccccgugugcuuucuaacaacuggacggagaacugauaagggcccgggucacc

..................................................uggacggagaacugauaaggCc..........    2      1    BF1
..................................................uggacggGgaacugauaagggc..........    3      1    BF1
..................................................uggacggagaacugauaagggc..........    25910  0    BF1
..................................................uggUcggagaacugauaagggc..........    3      1    BF1
..................................................uggacggagaacugauaagggA..........    325    1    BF1
..................................................uggacggagaacCgauaagggc..........    16     1    BF1
..................................................uggacggagaacugauGagggc..........    3      1    BF1
..................................................uggacggagaacugauUagggc..........    2      1    BF1
..................................................uggacggUgaacugauaagggc..........    1      1    BF1
..................................................uggacggagaacuCauaagggc..........    1      1    BF1
..................................................uggacggagaaGugauaagggc..........    8      1    BF1
..................................................uggacggagaacugauaGgggc..........    3      1    BF1
..................................................uUgacggagaacugauaagggc..........    1      1    BF1
..................................................uggacggagUacugauaagggc..........    6      1    BF1
..................................................uggacggagaacugauaagggcG.........    16     1    BF1
..................................................uggacggagaacugauaagggcU.........    857    1    BF1
..................................................uggacggagaacugauaagggcA.........    2864   1    BF1
..................................................uAgacggagaacugauaagggcc.........    1      1    BF1
..................................................uggacggagaacugauaaggUcc.........    1      1    BF1
..................................................uggacggagaacugauaagggcc.........    141    0    BF1
..................................................uggacggagaacugaAaagggcc.........    1      1    BF1
..................................................uggacggagaacugauaagggcAc........    14     1    BF1
..................................................uggacggagaacugauaagggccU........    10     1    BF1
..................................................uggacggagaacugauaagggccA........    14     1    BF1
..................................................uggacggagaacugauaagggccc........    2      0    BF1
..................................................uggacggagaacugauaagggcUc........    6      1    BF1
..................................................uggacggagaacugauaagggcccC.......    1      1    BF1
...................................................Ugacggagaacugauaagggc..........    1      1    BF1
...................................................ggacggagaacugauaagggc..........    6      0    BF1
....................................................gacggagaacugauaagggccc........    4      0    BF1

.............ccuuaucauucuuucgccccg................................................    1      0    MW1
................................cgugugcuuucuaacaacuggac...........................    1      0    MW1
................................cgugugcuuucuaacaacuggacggagaacugauaagg............    1      0    MW1
..............................................caacuggacggagaacugauaagggc..........    1      0    MW1
...............................................aacuggacggagaacugauaa..............    1      0    MW1
...............................................aacuggacggagaacugauaagggc..........    1      0    MW1
.................................................cuggacggagaacugauaaggg...........    3      0    MW1
.................................................cuggacggagaacugauaagggc..........    4      0    MW1
.................................................Guggacggagaacugauaagggc..........    1      1    MW1
..................................................uggacggagaacugaua...............    78     0    MW1
..................................................uggacggagaacugauaG..............    1      1    MW1
..................................................uggacggagaacugauaa..............    231    0    MW1
..................................................uggacggagaaAugauaa..............    1      1    MW1
..................................................Cggacggagaacugauaa..............    1      1    MW1
..................................................uggacggagaacugaCaag.............    1      1    MW1
..................................................uggacggagaacugauaag.............    205    0    MW1
..................................................uggacggagaacugauaaA.............    5      1    MW1
..................................................uggacggagaacugauaagA............    5      1    MW1
..................................................uggacggagaacugauaagg............    328    0    MW1
..................................................ugAacggagaacugauaagg............    1      1    MW1
..................................................uggacggagaacugauUagg............    1      1    MW1
..................................................uggacggagaacugGuaagg............    1      1    MW1
..................................................uggacUgagaacugauaaggg...........    1      1    MW1
..................................................uggacggagaacugauaaggg...........    944    0    MW1
..................................................uggacggagaacugauaaggU...........    3      1    MW1
..................................................uAgacggagaacugauaaggg...........    1      1    MW1
..................................................uggacggagaacugaCaaggg...........    1      1    MW1
..................................................uggacggagaacugauaaAgg...........    5      1    MW1
..................................................uggacggagUacugauaaggg...........    1      1    MW1
..................................................uggacggagaacCgauaaggg...........    1      1    MW1
..................................................uggacggagaacugauaaggA...........    10     1    MW1
..................................................uggacggagaacugauaagCg...........    1      1    MW1
..................................................uggacggagaaGugauaaggg...........    1      1    MW1
..................................................uggacggagaaUugauaagggc..........    2      1    MW1
..................................................uggacgAagaacugauaagggc..........    13     1    MW1
..................................................ugUacggagaacugauaagggc..........    1      1    MW1
..................................................uggacggagaacuUauaagggc..........    2      1    MW1
..................................................uggacUgagaacugauaagggc..........    1      1    MW1
```

```
             aga-miR-184*
                                                  aga-miR-184
ggugcacucgaacccuuaucauucuuucgccccgugugcuuucuaacaacuggacggagaacugauaagggcccgggucacc

..................................................uggacggGgaacugauaagggc..........   1       1   MW1
..................................................uggacggagaacugauaaggCc..........   1       1   MW1
..................................................uggacggagaacugauaagggG..........   1       1   MW1
..................................................uggacggagaacugaCaagggc..........   1       1   MW1
..................................................uggaUggagaacugauaagggc..........   3       1   MW1
..................................................uggacggagaacugauaagUgc..........   1       1   MW1
..................................................uggacggagaacugauaaggAc..........   5       1   MW1
..................................................uggacggagaacGgauaagggc..........   1       1   MW1
..................................................uggacggagaacugauaagAgc..........   2       1   MW1
..................................................uUgacggagaacugauaagggc..........   1       1   MW1
..................................................uggacggagaacugGuaagggc..........   2       1   MW1
..................................................uggacAgagaacugauaagggc..........   2       1   MW1
..................................................uggacggagaaAugauaagggc..........   3       1   MW1
..................................................uggGcggagaacugauaagggc..........   2       1   MW1
..................................................uggacggagaacugauaaAggc..........   2       1   MW1
..................................................uggacggUgaacugauaagggc..........   1       1   MW1
..................................................uggacggagaaGugauaagggc..........   1       1   MW1
..................................................uAgacggagaacugauaagggc..........   3       1   MW1
..................................................Cggacggagaacugauaagggc..........   3       1   MW1
..................................................Aggacggagaacugauaagggc..........   2       1   MW1
..................................................uggacggagaCcugauaagggc..........   2       1   MW1
..................................................uggacggagaUcugauaagggc..........   2       1   MW1
..................................................uggacgCagaacugauaagggc..........   1       1   MW1
..................................................uggacggCgaacugauaagggc..........   1       1   MW1
..................................................uggacggagaacCgauaagggc..........   6       1   MW1
..................................................uggacggagaacugauaagggc..........   10593   0   MW1
..................................................uggacggagaacugauaagggU..........   8       1   MW1
..................................................uggacggagaGcugauaagggc..........   2       1   MW1
..................................................uggacggagCacugauaagggc..........   1       1   MW1
..................................................uggacggagGacugauaagggc..........   2       1   MW1
..................................................ugAacggagaacugauaagggc..........   4       1   MW1
..................................................uggacggagaacuAauaagggc..........   7       1   MW1
..................................................ugCacggagaacugauaagggc..........   1       1   MW1
..................................................uggacggagaacugauaagggA..........   44      1   MW1
..................................................uggacggagaacugauaagggcG.........   6       1   MW1
..................................................uggacggagaacugauaagggcc.........   16      0   MW1
..................................................uggacggagaacugauaagggcA.........   305     1   MW1
..................................................uggacggagaacugauaagggcU.........   422     1   MW1
..................................................uggacggagaacugauaagggcUc........   3       1   MW1
..................................................uggacggagaacugauaagggccU........   8       1   MW1
..................................................uggacggagaacugauaagggccA........   2       1   MW1
...................................................ggacggagaacugauaaggg...........   1       0   MW1
...................................................ggacggagaacugauaagggc..........   3       0   MW1
...................................................ggacggagaacugauaagggcU.........   1       1   MW1
....................................................gacggagaacugauaagggc..........   7       0   MW1
....................................................gacggagaacugauaagggcc.........   1       0   MW1
....................................................gacggagaacugauaagggcU.........   2       1   MW1
....................................................gacggagaacugauaagggccc........   1       0   MW1
.....................................................acggagaacugauaagg............   2       0   MW1
.....................................................acggagaacugauaagggc..........   1       0   MW1
......................................................cggagaacugauaagggc..........   2       0   MW1
......................................................cggagaacugauaagggcU.........   3       1   MW1
.......................................................ggagaacugauaagggc..........   5       0   MW1
.......................................................ggagaacugauaagggcU.........   2       1   MW1

.............ccuuaucauucuuucgcc...................................................   1       0   FW1
.............ccuuaucauucuuucgccccgu...............................................   1       0   FW1
..............................................caacuggacggagaacugauaagggc..........   1       0   FW1
...............................................aacuggacggagaacugauaagggc..........   1       0   FW1
.................................................cuggacggagaacugauaagg............   1       0   FW1
.................................................cuggacggagaacugauaagggc..........   4       0   FW1
.................................................cuggacggagaacugauaagggcU.........   2       1   FW1
..................................................uggacggagaacugauU...............   1       1   FW1
..................................................uggacggagaacugaua...............   35      0   FW1
..................................................uggacggagaacugauaa..............   90      0   FW1
..................................................uggacggagaacugauaag.............   86      0   FW1
..................................................uggacggagaaUugauaag.............   1       1   FW1
..................................................uggacggagaacugauaaA.............   4       1   FW1
..................................................uggacggagaacugauaagg............   169     0   FW1
```

```
             aga-miR-184*
                                                  aga-miR-184
ggugcacucgaacccuuaucauucuuucgccccgugugcuuucuaacaacuggacggagaacugauaagggcccgggucacc

..................................................uggacggagaacCgauaagg............    1       1       FW1
..................................................uggacggagaCcugauaaggg...........    1       1       FW1
..................................................uggacggagaacugauaaggU...........    2       1       FW1
..................................................Cggacggagaacugauaaggg...........    1       1       FW1
..................................................uggacggagaacugauaaggg...........    515     0       FW1
..................................................uggacgAagaacugauaaggg...........    1       1       FW1
..................................................uggacggagaacugauaaAgg...........    4       1       FW1
..................................................uggacggagaGcugauaaggg...........    1       1       FW1
..................................................uggacggagaacugauaaggA...........    5       1       FW1
..................................................uggacggagUacugauaagggc..........    1       1       FW1
..................................................Gggacggagaacugauaagggc..........    1       1       FW1
..................................................uggacggagaGcugauaagggc..........    2       1       FW1
..................................................uggacggagaacugauaaAggc..........    2       1       FW1
..................................................ugAacggagaacugauaagggc..........    3       1       FW1
..................................................uggCcggagaacugauaagggc..........    1       1       FW1
..................................................uggacggagaacuAauaagggc..........    2       1       FW1
..................................................ugUacggagaacugauaagggc..........    2       1       FW1
..................................................uggacggagaacugauaaggUc..........    1       1       FW1
..................................................Cggacggagaacugauaagggc..........    3       1       FW1
..................................................uggacUgagaacugauaagggc..........    2       1       FW1
..................................................uggacggGgaacugauaagggc..........    1       1       FW1
..................................................uggGcggagaacugauaagggc..........    1       1       FW1
..................................................Aggacggagaacugauaagggc..........    1       1       FW1
..................................................uggacggaAaacugauaagggc..........    1       1       FW1
..................................................uAgacggagaacugauaagggc..........    1       1       FW1
..................................................uggacggagaacugauaagggc..........    6029    0       FW1
..................................................uggacggagaacugUuaagggc..........    1       1       FW1
..................................................uggacggagaCcugauaagggc..........    7       1       FW1
..................................................uggacggagaacugauaagggA..........    16      1       FW1
..................................................uggacggagaacugaCaagggc..........    3       1       FW1
..................................................uggacggagCacugauaagggc..........    1       1       FW1
..................................................uggacgAagaacugauaagggc..........    6       1       FW1
..................................................uggacggagaacugauGagggc..........    1       1       FW1
..................................................uggacAgagaacugauaagggc..........    2       1       FW1
..................................................uggaGggagaacugauaagggc..........    1       1       FW1
..................................................uggacggagaacCgauaagggc..........    3       1       FW1
..................................................uggacggUgaacugauaagggc..........    2       1       FW1
..................................................uggacggagaacugauaagggU..........    5       1       FW1
..................................................uggaUggagaacugauaagggc..........    4       1       FW1
..................................................uggacggagaacugauaaggAc..........    2       1       FW1
..................................................uggacggagGacugauaagggc..........    3       1       FW1
..................................................uggacggaCaacugauaagggc..........    1       1       FW1
..................................................uggacggagaacugauaUgggc..........    1       1       FW1
..................................................uggacggagaaAugauaagggc..........    3       1       FW1
..................................................uggacggagaacugauaaggCc..........    1       1       FW1
..................................................uggacggagaUcugauaagggc..........    1       1       FW1
..................................................uggacggagaacugauaagggcU.........    237     1       FW1
..................................................uggacggagaacugauaagggcA.........    157     1       FW1
..................................................uggacggagaacugauaagggcc.........    11      0       FW1
..................................................uggacggagaacugauaagggcG.........    4       1       FW1
..................................................uggacggagaacugauaagggcUc........    2       1       FW1
..................................................uggacggagaacugauaagggccU........    5       1       FW1
..................................................uggacggagaacugauaagggcccU.......    1       1       FW1
...................................................ggacggagaacugauaagggc..........    4       0       FW1
....................................................gacggagaacugauaagggc..........    3       0       FW1
....................................................gacggagaacugauaagggcc.........    1       0       FW1
.....................................................acggagaacugauaagggcU.........    1       1       FW1
......................................................cggagaacugauaagggc..........    1       0       FW1
.......................................................ggagaacugauaagggc..........    2       0       FW1
.......................................................ggagaacugauaagggcU.........    1       1       FW1

.............ccuuaucauucuuucgccccg................................................    1       0       MW2
.............ccuuaucauucuuucgccccgu...............................................    2       0       MW2
................................................acuggacggagaacugauaagg............    1       0       MW2
.................................................cuggacggagaacugauaaggg...........    1       0       MW2
.................................................cuggacggagaacugauaagggc..........    2       0       MW2
..................................................uggacggagaacugaua...............    25      0       MW2
..................................................uggacggagaacugauaa..............    64      0       MW2
..................................................uggacggagaCcugauaag.............    1       1       MW2
```

```
             aga-miR-184*
                                                  aga-miR-184
ggugcacucgaacccuuaucauucuuucgccccgugugcuuucuaacaacuggacggagaacugauaagggcccgggucacc

..................................................uggacAgagaacugauaag.............    1     1   MW2
..................................................uggacggagaacugauaag.............    58    0   MW2
..................................................uggacggagaacugauaaAg............    1     1   MW2
..................................................uggacggagaacugauaagg............    69    0   MW2
..................................................ugAacggagaacugauaagg............    1     1   MW2
..................................................uggacggagaacugauaaggA...........    1     1   MW2
..................................................uggacggagaacugauaaAgg...........    2     1   MW2
..................................................uggacggagaaUugauaaggg...........    1     1   MW2
..................................................uggacggagaacugauaaggg...........    232   0   MW2
..................................................uUgacggagaacugauaaggg...........    1     1   MW2
..................................................uggacgAagaacugauaaggg...........    1     1   MW2
..................................................uggUcggagaacugauaagggc..........    1     1   MW2
..................................................uggacggCgaacugauaagggc..........    1     1   MW2
..................................................uggacggagaacugauaaUggc..........    1     1   MW2
..................................................ugAacggagaacugauaagggc..........    1     1   MW2
..................................................uggacggagaacuCauaagggc..........    1     1   MW2
..................................................uggacggagaaAugauaagggc..........    2     1   MW2
..................................................uUgacggagaacugauaagggc..........    1     1   MW2
..................................................uggacggagaacugauaagggc..........    3172  0   MW2
..................................................uggacggagaacugauaagggA..........    9     1   MW2
..................................................Aggacggagaacugauaagggc..........    1     1   MW2
..................................................uggacggagaacugauaagggU..........    1     1   MW2
..................................................uggacUgagaacugauaagggc..........    1     1   MW2
..................................................uggacggagGacugauaagggc..........    2     1   MW2
..................................................Cggacggagaacugauaagggc..........    1     1   MW2
..................................................uggacgAagaacugauaagggc..........    3     1   MW2
..................................................uggacggagaacugauaaggAc..........    1     1   MW2
..................................................uggacggagaacugauaagggcU.........    96    1   MW2
..................................................uggacggagaacugauaagggcc.........    6     0   MW2
..................................................uggacggagaacugauaagggcG.........    1     1   MW2
..................................................uggacggagaacugauaagggcA.........    77    1   MW2
..................................................uggacggagaacugauaagggcUc........    1     1   MW2
..................................................uggacggagaacugauaagggcAc........    1     1   MW2
..................................................uggacggagaacugauaagggccU........    2     1   MW2
...................................................ggacggagaacugauaagggc..........    1     0   MW2
....................................................gacggagaacugauaagg............    1     0   MW2
....................................................gacggagaacugauaagggc..........    1     0   MW2
....................................................gacggagaacugauaagggccc........    3     0   MW2
.....................................................acggagaacugauaagggc..........    1     0   MW2
......................................................cggagaacugauaagggc..........    1     0   MW2
......................................................cggagaacugauaagggcU.........    1     1   MW2
.......................................................ggagaacugauaagggc..........    6     0   MW2
.......................................................ggagaacugauaagggcA.........    1     1   MW2

.............ccuuaucauucuuucgccccg................................................    4     0   TE2
.............ccuuaucauucuuucgccccgu...............................................    1     0   TE2
.............ccuuaucauucuuucgccccguA..............................................    1     1   TE2
.............ccuuaucauucuuucgccccgugugcuuucuaacaac................................    1     0   TE2
.................................................cuggacggagaacugauaagg............    2     0   TE2
.................................................cuggacggagaacugauaaggg...........    7     0   TE2
.................................................cuggacggagaacugauaagggA..........    1     1   TE2
.................................................cuggacggagaacugauaagggc..........    14    0   TE2
.................................................cuggacggagaacugauaagggcU.........    1     1   TE2
..................................................uggacAgagaacugaua...............    1     1   TE2
..................................................uggacggagaacugauC...............    1     1   TE2
..................................................uggacggagaacugauU...............    1     1   TE2
..................................................uggacggagaacugCua...............    1     1   TE2
..................................................uggacggagaGcugaua...............    1     1   TE2
..................................................uggacggagaacugaua...............    305   0   TE2
..................................................Cggacggagaacugaua...............    1     1   TE2
..................................................uggacggagaacugauaa..............    544   0   TE2
..................................................uggacUgagaacugauaa..............    1     1   TE2
..................................................uggacggagaacugauaU..............    2     1   TE2
..................................................uggacggagaacugauaG..............    1     1   TE2
..................................................uggacggagaCcugauaa..............    1     1   TE2
..................................................uggacgAagaacugauaag.............    1     1   TE2
..................................................uggacggagaacugaCaag.............    1     1   TE2
..................................................uggacggagaacugGuaag.............    2     1   TE2
..................................................uggacgCagaacugauaag.............    1     1   TE2
```

```
                aga-miR-184*
                                                     aga-miR-184
ggugcacucgaacccuuaucauucuuucgccccgugugcuuucuaacaacuggacggagaacugauaagggcccgggucacc

..................................................Cggacggagaacugauaag.............   1       1     TE2
..................................................ugAacggagaacugauaag.............   1       1     TE2
..................................................uggacggagaacugauaaU.............   2       1     TE2
..................................................uggacggagaaUugauaag.............   3       1     TE2
..................................................uggacggagaUcugauaag.............   2       1     TE2
..................................................uggacggagaacugauaag.............   827     0     TE2
..................................................uggaUggagaacugauaag.............   2       1     TE2
..................................................uggacggagaacugauaaA.............   17      1     TE2
..................................................uggacggagaacugauaagU............   2       1     TE2
..................................................uggGcggagaacugauaagg............   1       1     TE2
..................................................uggacggagGacugauaagg............   2       1     TE2
..................................................uggacggagaUcugauaagg............   1       1     TE2
..................................................uggacggagaacugauaagg............   1670    0     TE2
..................................................uggacggagaacugauGagg............   2       1     TE2
..................................................uggacggagUacugauaagg............   2       1     TE2
..................................................uggacCgagaacugauaagg............   1       1     TE2
..................................................uggacggagaaAugauaagg............   2       1     TE2
..................................................uggacggagaacugGuaagg............   1       1     TE2
..................................................uggacggagaacugauaGgg............   2       1     TE2
..................................................uggacgAagaacugauaagg............   2       1     TE2
..................................................uggacggagaaGugauaagg............   1       1     TE2
..................................................uggaUggagaacugauaagg............   1       1     TE2
..................................................uggacggagaacugauaagA............   8       1     TE2
..................................................uggacggaCaacugauaagg............   1       1     TE2
..................................................ugAacggagaacugauaagg............   1       1     TE2
..................................................uggacggagGacugauaaggg...........   3       1     TE2
..................................................uggacggagaacugauCaggg...........   2       1     TE2
..................................................uggacggagaacuAauaaggg...........   3       1     TE2
..................................................uggacgAagaacugauaaggg...........   4       1     TE2
..................................................uggacggagaacugauaaggg...........   4894    0     TE2
..................................................uggacggagaacugauaaUgg...........   2       1     TE2
..................................................uggacggagaacugauaaggC...........   4       1     TE2
..................................................uggacggagaacugauaaggA...........   76      1     TE2
..................................................uggacggagaaUugauaaggg...........   1       1     TE2
..................................................uggacggGgaacugauaaggg...........   1       1     TE2
..................................................uggGcggagaacugauaaggg...........   1       1     TE2
..................................................uggacggagaacAgauaaggg...........   1       1     TE2
..................................................uggacggagaacugauGaggg...........   1       1     TE2
..................................................uggacggagaacugauaaggU...........   10      1     TE2
..................................................uggacggCgaacugauaaggg...........   1       1     TE2
..................................................uUgacggagaacugauaaggg...........   1       1     TE2
..................................................uggacggagaaAugauaaggg...........   3       1     TE2
..................................................Gggacggagaacugauaaggg...........   1       1     TE2
..................................................uggacggagaacugaCaaggg...........   2       1     TE2
..................................................ugAacggagaacugauaaggg...........   2       1     TE2
..................................................uggacggagCacugauaaggg...........   2       1     TE2
..................................................uggaUggagaacugauaaggg...........   2       1     TE2
..................................................uggacAgagaacugauaaggg...........   2       1     TE2
..................................................uggacggagaacCgauaaggg...........   1       1     TE2
..................................................uggacggagaacugauaagAg...........   2       1     TE2
..................................................uggaAggagaacugauaaggg...........   1       1     TE2
..................................................uggacggagaacugauaaAgg...........   11      1     TE2
..................................................uggacggagaaGugauaaggg...........   3       1     TE2
..................................................uggacggagaacugauaagUgc..........   6       1     TE2
..................................................uggCcggagaacugauaagggc..........   2       1     TE2
..................................................uggacggagaacugauaagggG..........   45      1     TE2
..................................................uUgacggagaacugauaagggc..........   3       1     TE2
..................................................uggacggagaacugauaagggA..........   568     1     TE2
..................................................uggacgUagaacugauaagggc..........   3       1     TE2
..................................................uggacAgagaacugauaagggc..........   15      1     TE2
..................................................uggacggagaacugauaagggc..........   47470   0     TE2
..................................................uggacggagaaAugauaagggc..........   36      1     TE2
..................................................uggacggagaacugauaagCgc..........   2       1     TE2
..................................................uggacggagaGcugauaagggc..........   3       1     TE2
..................................................uggacggagaacugaCaagggc..........   11      1     TE2
..................................................uggacggagaacugauaaggCc..........   2       1     TE2
..................................................uggacggagaacugauCagggc..........   10      1     TE2
..................................................uggacggagaacugauaaCggc..........   3       1     TE2
..................................................uggacggagaacugauaagggU..........   58      1     TE2
```

aga-miR-184*

aga-miR-184

ggugcacucgaacccuuaucauucuuucgccccgugugcuuucuaacaacuggacggagaacugauaagggcccgggucacc

....Cggacggagaacugauaagggc..........	19	1	TE2
....Gggacggagaacugauaagggc..........	6	1	TE2
....uggacggagaacuCauaagggc..........	1	1	TE2
....uggaUggagaacugauaagggc..........	16	1	TE2
....uggacggagaacugauaaAggc..........	4	1	TE2
....uggacggagaacugauaGgggc..........	5	1	TE2
....uggacggCgaacugauaagggc..........	1	1	TE2
....uggacggagaacugaAaagggc..........	1	1	TE2
....uggacggagaacugCuaagggc..........	1	1	TE2
....uggacggagaacugauaaggUc..........	5	1	TE2
....uggacggagaaGugauaagggc..........	12	1	TE2
....uggacggagaacugauGagggc..........	8	1	TE2
....uggacggagCacugauaagggc..........	10	1	TE2
....uggacggaAaacugauaagggc..........	7	1	TE2
....Aggacggagaacugauaagggc..........	10	1	TE2
....uggacggagaacuUauaagggc..........	8	1	TE2
....ugAacggagaacugauaagggc..........	23	1	TE2
....uggacggagGacugauaagggc..........	19	1	TE2
....uggacggagaacuAauaagggc..........	40	1	TE2
....uggacggagaacugauaUgggc..........	1	1	TE2
....uggacggagaacugGuaagggc..........	9	1	TE2
....uggacggagaacugUuaagggc..........	5	1	TE2
....uggacggagaacugauaaUggc..........	6	1	TE2
....uggacggagaacugauaagAgc..........	15	1	TE2
....uggacgAagaacugauaagggc..........	34	1	TE2
....uggacgCagaacugauaagggc..........	8	1	TE2
....uggacggagaCcugauaagggc..........	7	1	TE2
....uggacggagaUcugauaagggc..........	10	1	TE2
....uggGcggagaacugauaagggc..........	10	1	TE2
....uggacUgagaacugauaagggc..........	5	1	TE2
....uAgacggagaacugauaagggc..........	16	1	TE2
....uggaAggagaacugauaagggc..........	1	1	TE2
....uggacCgagaacugauaagggc..........	1	1	TE2
....uggacggagaacCgauaagggc..........	12	1	TE2
....uggacggagaacugauUagggc..........	1	1	TE2
....uCgacggagaacugauaagggc..........	2	1	TE2
....uggacggagUacugauaagggc..........	6	1	TE2
....uggacggagaaUugauaagggc..........	14	1	TE2
....ugCacggagaacugauaagggc..........	2	1	TE2
....ugUacggagaacugauaagggc..........	4	1	TE2
....uggacggagaacGgauaagggc..........	2	1	TE2
....uggacggagaacugauaaggAc..........	39	1	TE2
....uggacggaCaacugauaagggc..........	2	1	TE2
....uggacggGgaacugauaagggc..........	10	1	TE2
....uggUcggagaacugauaagggc..........	2	1	TE2
....uggacggagaacugauaagggAc.........	1	1	TE2
....uggacggGgaacugauaagggcc.........	1	1	TE2
....uggacggagaacugauaagggcc.........	87	0	TE2
....uggacggagaacugauaagggcU.........	2948	1	TE2
....uggacggagaacugauaagggcA.........	2487	1	TE2
....uggacggagaacugauaagggGc.........	1	1	TE2
....uggacggagaacugauaagUgcc.........	1	1	TE2
....uggacggagaacugauaagggcG.........	29	1	TE2
....Aggacggagaacugauaagggcc.........	1	1	TE2
....uggacggagaacugauaagggccA........	7	1	TE2
....uggacggagaacugauaagggcAc........	4	1	TE2
....uggacggagaacugauaagggccU........	23	1	TE2
....uggacggagaacugauaagggccG........	1	1	TE2
....uggacggagaacugauaagggccc........	2	0	TE2
....uggacggagaacugauaagggcUc........	6	1	TE2
....ggacggagaacugauaaggg...........	6	0	TE2
....ggacggagaacugauaagggc..........	15	0	TE2
....Ugacggagaacugauaagggc..........	2	1	TE2
....ggacggagaacugauaagggcA.........	1	1	TE2
....gacggagaacugauaagggc..........	6	0	TE2
....Aacggagaacugauaagggc..........	1	1	TE2
....gacggagaacugauaagggcc.........	1	0	TE2
....gacggagaacugauaagggccc........	2	0	TE2
....acggagaacugauaagggc..........	2	0	TE2

aga-miR-184*

aga-miR-184

ggugcacucgaacccuuaucauucuuucgccccgugugcuuucuaacaacuggacggagaacugauaagggcccgggucacc

....acggagaacugauaagggccc........ 2 0 TE2

miRBase precursor : aga-mir-1889
Total read count : 1359
aga-miR-1889 read count : 1049
remaining reads : 310

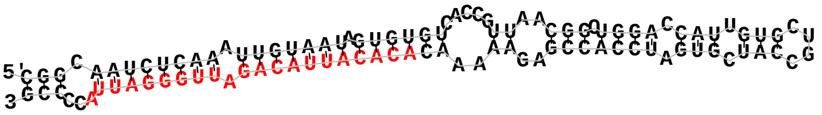

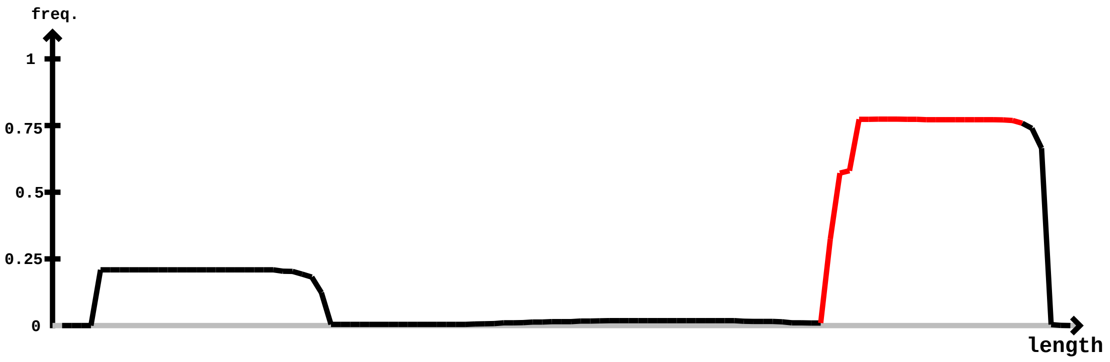


```
                                                                             aga-miR-1889
5'- cggcaaucucaaauuguaauagugugucaccguuaacggcuggaccauugugcugccaucgugauccaccgagaaaaacacacauuacagauugggauuaccccg   -3'    exp
    .((.((((((((.(((((((.((((((.....((..(((.((((.(((.(((....))).))).)))))))..))..))))))))))))).)))))))).))...   reads    mm    sample
    ...caaucucaaauuguaauagugu................................................................................   1        0     FF2
    ...caaucucaaauuguaauagugug...............................................................................   1        0     FF2
    ...caaucucaaauuguaauagugugu..............................................................................   4        0     FF2
    .............................................cauugugcugccaucgugauccaccgagaaaaaca.........................   1        0     FF2
    ...............................................................................acacauuacagauugggauuac....   3        0     FF2
    ...............................................................................acacauuacagauugggauuacc...   13       0     FF2
    ................................................................................cacauuacagauugggauuac....   2        0     FF2
    ................................................................................cacauuacagauugggauuacc...   18       0     FF2
    .................................................................................acauuacagauugggauuacc...   1        0     FF2
    ..................................................................................cauuacagauugggauuaU....   1        1     FF2
    ..................................................................................cauuacagauugggauuacc...   4        0     FF2

    ...caaucucaaauuguaauag...................................................................................   3        0     OV2
    ...caaucucaaauuguaauagug.................................................................................   2        0     OV2
    ...caaucucaaauuguaauagugu................................................................................   3        0     OV2
    ...caaucucaaauuguaauagugug...............................................................................   12       0     OV2
    ...caaucucaaauuguaauaguguG...............................................................................   2        1     OV2
    ...caaucucaaauuguaauaguguC...............................................................................   1        1     OV2
    ...caaucucaaauuguaauagugugu..............................................................................   16       0     OV2
    ..........................ucaccguuaacggcuggaccauugugc....................................................   1        0     OV2
    ................................................ugugcugccaucgugauccaccgaga...............................   1        0     OV2
    ..................................................ugcugccaucgugauccaccgagaa..............................   1        0     OV2
    ...............................................................................acacauuacagauugggauu......   2        0     OV2
    ...............................................................................acacauuacagauugggauua.....   3        0     OV2
    ...............................................................................acacauuacagauugggauuaA....   1        1     OV2
    ...............................................................................acacauuacagauugggauuaU....   1        1     OV2
    ...............................................................................acacauuacagauugggauuac....   3        0     OV2
    ...............................................................................acacauuacagauugggauuacU...   2        1     OV2
    ...............................................................................acacauuacagauugggauuacc...   23       0     OV2
    ................................................................................cacauuacagauugggauua.....   1        0     OV2
    ................................................................................cacauuacagauugggauuac....   2        0     OV2
    ................................................................................cacauuacagUuugggauuacc...   1        1     OV2
    ................................................................................cacauuacagauugggauuacU...   2        1     OV2
    ................................................................................cacauuacagauugggauuacc...   25       0     OV2
    ................................................................................cacaAuacagauugggauuacc...   1        1     OV2
```

aga-miR-1889

```
cggcaaucucaaauuguaauagugugucaccguuaacggcuggaccauugugcugccaucgugauccaccgagaaaaacacacauuacagauugggauuaccccg
.................................................................................acauuacagauugggauuacc...   1    0    OV2
..................................................................................cauuacagauugggauuac....   1    0    OV2
..................................................................................cauuacagauugggauuacc...   24   0    OV2
..................................................................................cauuacagauugggauuacU...   4    1    OV2

...caaucucaaauuguaauag...................................................................................   1    0    TE1
...caaucucaaauuguaauagugug...............................................................................   1    0    TE1
...caaucucaaauuguaauagugugu..............................................................................   3    0    TE1
..........................ucaccguuaacggcuggaccauugugcugccaucgugauccaccgagaaaa............................   1    0    TE1
...........................caccguuaacggcuggaccauugugcugccaucgugauccaccgagaaaaa...........................   2    0    TE1
...............................................................................acacauuacagauugggauua.....   1    0    TE1
...............................................................................acacauuacagauugggauuaU....   2    1    TE1
...............................................................................acacauuacagauugggauuac....   1    0    TE1
...............................................................................acacauuacagauugggauuacc...   15   0    TE1
................................................................................cacauuacagauugggauuacc...   10   0    TE1
..................................................................................cauuacagauugggauuacc...   10   0    TE1

...caaucucaaauuguaauagug.................................................................................   1    0    MF2
...caaucucaaauuguaauagugu................................................................................   1    0    MF2
...caaucucaaauuguaauagugug...............................................................................   21   0    MF2
...caaucucaaauuguaauagugugA..............................................................................   5    1    MF2
...caaucucaaauuguaauagugugu..............................................................................   34   0    MF2
...caaucucaaauuguaauagugugG..............................................................................   3    1    MF2
..........................................gaccauugugcugccaucgugauccaccgagaaaaacacacauua..................   1    0    MF2
............................................ccauugugcugccaucgugauccaccgagaaaaaca.........................   1    0    MF2
.............................................cauugugcugccaucgugauccacc...................................   3    0    MF2
..................................................ugcugccaucgugauccaccgagaaaaacac........................   1    0    MF2
...............................................................................acacauuacagauugggauu......   2    0    MF2
...............................................................................acacauuacagauugggauua.....   4    0    MF2
...............................................................................acacauuacagauugggauuaU....   4    1    MF2
...............................................................................acacauuacagauugggauuac....   18   0    MF2
...............................................................................acacauuacagauugggauuaA....   2    1    MF2
...............................................................................acacauuacagauugAgauuacc...   1    1    MF2
...............................................................................acacauuacagauugggauuacc...   74   0    MF2
...............................................................................acacauuacagauugggauuacA...   1    1    MF2
...............................................................................acacauuacagauugggauuaGc...   1    1    MF2
...............................................................................acacauuacagauugggauuacU...   1    1    MF2
................................................................................cacauuacagauugggauuac....   1    0    MF2
................................................................................cacauuacagauugggauuacc...   89   0    MF2
................................................................................cacauuacagauugggauuacU...   1    1    MF2
................................................................................cacauuacagauugggauuaccc..   1    0    MF2
................................................................................cacauuacagauugggauuacccc.   1    0    MF2
.................................................................................acauuacagauugggauuac....   1    0    MF2
.................................................................................acauuacagauugggauuacc...   3    0    MF2
..................................................................................cauuacagauugggauuacc...   18   0    MF2

...caaucucaaauuguaauagugu................................................................................   1    0    FW2
...caaucucaaauuguaauagugug...............................................................................   1    0    FW2
...caaucucaaauuguaauagugugu..............................................................................   6    0    FW2
...............................................................................acacauuacagauugggauu......   1    0    FW2
...............................................................................acacauuacagauugggauuaA....   1    1    FW2
...............................................................................acacauuacagauugggauuacc...   2    0    FW2
................................................................................cacauuacagauugggauuacc...   4    0    FW2
.................................................................................acauuacagauugggauuacc...   1    0    FW2
..................................................................................cauuacagauugggauuacU...   1    1    FW2
..................................................................................cauuacagauugggauuacc...   6    0    FW2

...caaucucaaauuguaauagugu................................................................................   2    0    FF1
...caaucucaaauuguaauagugug...............................................................................   8    0    FF1
...caaucucaaauuguaauagugugG..............................................................................   3    1    FF1
...caaucucaaauuguaauagugugA..............................................................................   1    1    FF1
...caaucucaaauuguaauagugugu..............................................................................   19   0    FF1
..........................................gaccauugugcugccaucgugauccaccgagaaaaacacacauuaca................   1    0    FF1
...............................................................................acacauuacagauugggauu......   1    0    FF1
...............................................................................acacauuacagauugggauua.....   1    0    FF1
...............................................................................acacauuacagauugggauuac....   2    0    FF1
...............................................................................acacGuuacagauugggauuacc...   2    1    FF1
...............................................................................acacauuacagauugggauuacc...   7    0    FF1
................................................................................cacauuacagauugggauu......   1    0    FF1
................................................................................cacauuacagauugggauuacc...   19   0    FF1
```

aga-miR-1889

```
cggcaaucucaaauuguaauagugugucaccguuaacggcuggaccauugugcugccaucgugauccaccgagaaaaacacacauuacagauugggauuaccccg
................................................................................cacauuacagauugggUuacc...   1   1   FF1
................................................................................cacauuacagauugggauuacU...  1   1   FF1
.................................................................................acauuacagauugggauuacc...  2   0   FF1
..................................................................................cauuacagauugggauuacc...  6   0   FF1

...caaucucaaauuguaauag...................................................................................  2   0   OV1
...caaucucaaauuguaauagug.................................................................................  4   0   OV1
...caaucucaaauuguaauagugug...............................................................................  8   0   OV1
...caaucucaaauuguaauagugugG..............................................................................  2   1   OV1
...caaucucaaauuguaauagugugA..............................................................................  3   1   OV1
...caaucucaaauuguaauagugugu..............................................................................  8   0   OV1
...caaucucaaauGguaauagugugu..............................................................................  1   1   OV1
...........................................accauugugcugccaucgugauccaccg..................................  1   0   OV1
................................................ugugcugccaucgugauccaccgaga...............................  1   0   OV1
................................................ugugcugccaucgugauccaccgagaa..............................  1   0   OV1
.....................................................ugccaucgugauccaccgagaa..............................  1   0   OV1
.......................................................ccaucgugauccaccgagaa..............................  1   0   OV1
...............................................................................acacauuacagauugggauu......  1   0   OV1
...............................................................................acacauuacagauugggauua.....  4   0   OV1
...............................................................................acacauuacagauugggauuac....  12  0   OV1
...............................................................................acacauuacagauugggauuaU....  1   1   OV1
...............................................................................acacauuacagauugggauuaA....  2   1   OV1
...............................................................................acacauuacagauugggauuacU...  2   1   OV1
...............................................................................acacCuuacagauugggauuacc...  1   1   OV1
...............................................................................acacauuacagauugggauuacc...  38  0   OV1
................................................................................cacauuacagauugggauuac....  2   0   OV1
................................................................................cacauuacagauugggauuacU...  1   1   OV1
................................................................................cacauuacagauugggauuacc...  32  0   OV1
..................................................................................cauuacagauugggauuacc...  41  0   OV1
..................................................................................Gauuacagauugggauuacc...  1   1   OV1
..................................................................................cauuacagauugggauuacU...  3   1   OV1

...caaucucaaauuguaauagu..................................................................................  1   0   MF1
...caaucucaaauuguaauagugug...............................................................................  3   0   MF1
...caaucucaaauuguaauagugugu..............................................................................  3   0   MF1
...............................................................................acacauuacagauugggauu......  2   0   MF1
...............................................................................acacauuacagauugggauuac....  3   0   MF1
...............................................................................acacauuacagauugggauuacc...  5   0   MF1
...............................................................................acacauuacagauugggauuacA...  1   1   MF1
................................................................................cacauuacagauugggauuacc...  11  0   MF1
................................................................................cacauuacagauugggauuacU...  2   1   MF1
..................................................................................cauuacagauugggauuacc...  6   0   MF1
....................................................................................uuacagauugggauuacc...  1   0   MF1

...caaucucaaauuguaauagug.................................................................................  5   0   BF2
...caaucucaaauuguaauagugu................................................................................  3   0   BF2
...caaucucaaauuguaauagAgug...............................................................................  1   1   BF2
...caaucucaaauuguaauagugug...............................................................................  7   0   BF2
...caaucucaaauuguaauagugugu..............................................................................  24  0   BF2
...caaucucaaauuguaauagugugG..............................................................................  3   1   BF2
...caaucucaaauuguaauagugugA..............................................................................  3   1   BF2
...............................................uugugcugccaucgugauccaccgagaaaaaca.........................  1   0   BF2
.....................................................ugccaucgugauccaccgagaaaaaca.........................  3   0   BF2
...............................................................................acacauuacagauugggau.......  2   0   BF2
...............................................................................acacauuacagauugggauu......  1   0   BF2
...............................................................................acacauuacagauugggauua.....  5   0   BF2
...............................................................................aUacauuacagauugggauua.....  1   1   BF2
...............................................................................acacauuacagauugggauuaU....  1   1   BF2
...............................................................................acacauuacagauugggauuac....  17  0   BF2
...............................................................................acacauuacagauugggauuacc...  34  0   BF2
...............................................................................acacauuacagauugggauuacU...  2   1   BF2
...............................................................................acacauuacagauugggauuaccA..  1   1   BF2
................................................................................cacauuacagauugggauuac....  1   0   BF2
................................................................................cacauuacagauugggauuacU...  6   1   BF2
................................................................................cacauuacagauugggauuacc...  38  0   BF2
.................................................................................acauuacagauugggauuaccc..  1   0   BF2
..................................................................................cauuacagauugggauuacU...  2   1   BF2
..................................................................................cauuacagauugggauuacc...  17  0   BF2

...caaucucaaauuguaauag...................................................................................  1   0   BF1
```

aga-miR-1889

```
cggcaaucucaaauuguaauagugugucaccguuaacggcuggaccauugugcugccaucgugauccaccgagaaaaacacacauuacagauugggauuaccccg
...caaucucaaauuguaauagug.................................................................................    1    0    BF1
...caaucucaaauuguaauagugu................................................................................    1    0    BF1
...caaucucaaauuguaauagugug...............................................................................    8    0    BF1
...caaucucaaauuguaauagugugG..............................................................................    1    1    BF1
...caaucucaaauuguaauagugugu..............................................................................    8    0    BF1
...........................caccguuaacggcuggaccauugugcugccaucgugauccaccgagaa..............................    1    0    BF1
..............................................................................cacacauuacagauugggauua.....    2    0    BF1
...............................................................................acacauuacagauugggauua.....    2    0    BF1
...............................................................................acacauuacagauugggauuaU....    2    1    BF1
...............................................................................acacauuacagauugggauuac....    7    0    BF1
...............................................................................acacauuacagauugggauuacc...    31   0    BF1
...............................................................................acacauuacagauugggauuacU...    2    1    BF1
................................................................................cacauuacagauugggauuGcc...    1    1    BF1
................................................................................cacauuacagauugggauuacc...    24   0    BF1
................................................................................cacauuacagauugggauuacU...    1    1    BF1
..................................................................................cauuacagauugggauuacc...    26   0    BF1

...caaucucaaauuguaauagugu................................................................................    1    0    FW1
...caaucucaaauuguaauagugugu..............................................................................    2    0    FW1
...caaucucaaauuguaauagugugucaccguuaacggcuggac............................................................    1    0    FW1
.............................................cauugugcugccaucgugauccaccgagaaaaaca.........................    1    0    FW1
...............................................................................acacauuacagauugggauuacU...    1    1    FW1
...............................................................................acacauuacagauugggauuacc...    1    0    FW1
................................................................................cacauuacagauugggauuacc...    2    0    FW1
..................................................................................cauuacagauugggauuacc...    2    0    FW1

...caaucucaaauuguaauagug.................................................................................    1    0    MW1
...caaucucaaauuguaauagugu................................................................................    2    0    MW1
...caaucucaaauuguaauaguUug...............................................................................    1    1    MW1
...caaucucaaauuguaauagugug...............................................................................    3    0    MW1
...caaucucaaauuguaauagugugA..............................................................................    1    1    MW1
...caaucucaaauuguaauagugugu..............................................................................    3    0    MW1
...............................................................................acacauuacagauugggau.......    1    0    MW1
...............................................................................acacauuacagauugggauuac....    1    0    MW1
...............................................................................acacauuacagauugggauuaU....    1    1    MW1
...............................................................................acacauuacagauugggauuacU...    4    1    MW1
...............................................................................acacauuacagauugggauuacc...    1    0    MW1
................................................................................cacauuacagauugggauuacU...    1    1    MW1
................................................................................cacauuacagauugggauuacc...    3    0    MW1
..................................................................................cauuacagauugggauuacc...    3    0    MW1

...caaucucaaauuguaauagugug...............................................................................    1    0    MW2
........................................................caucgugauccaccgagaaaaacacacauuaca................    1    0    MW2
...............................................................................acacauuacagauuggga........    1    0    MW2
...............................................................................acacauuacagauugggauuac....    1    0    MW2
...............................................................................acacauuacagauugggauuacc...    2    0    MW2
...............................................................................acacauuacagauugggauuacU...    2    1    MW2
................................................................................cacauuacagauugggauuacc...    3    0    MW2
..................................................................................cauuacagauugggauuacc...    1    0    MW2

...caaucucaaauuguaauagugug...............................................................................    4    0    TE2
...caaucucaaauuguaauaguguC...............................................................................    1    1    TE2
...caaucucaaauuguaauagugugG..............................................................................    1    1    TE2
...caaucucaaauuguaauagugugu..............................................................................    5    0    TE2
...............................................................................acacauuacagauugggauu......    3    0    TE2
...............................................................................acacauuacagauugggauua.....    2    0    TE2
...............................................................................acacauuacagauugggauuac....    3    0    TE2
...............................................................................acacauuacagauugggauuacc...    29   0    TE2
................................................................................cacauuacagauugggauuac....    1    0    TE2
................................................................................cacauuacagauugggauuacc...    32   0    TE2
................................................................................cacauuacagauuAggauuacc...    1    1    TE2
.................................................................................acauuacagauugggauuacc...    2    0    TE2
.................................................................................acauuacagauugggauuaccc..    1    0    TE2
..................................................................................cauuaUagauugggauuacc...    1    1    TE2
..................................................................................cauuacagauugggauuacc...    84   0    TE2
```

miRBase precursor : aga-mir-1890
Total read count : 496
aga-miR-1890 read count : 451
aga-miR-1890* read count : 37
remaining reads : 8

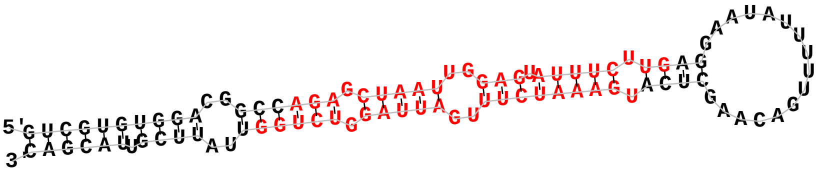

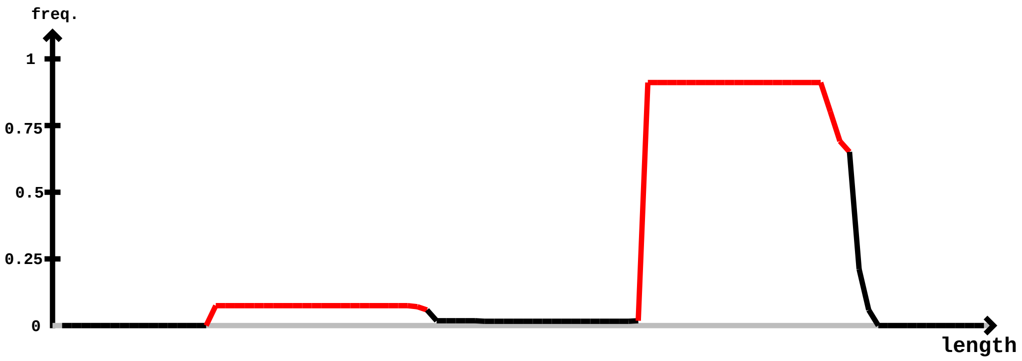

aga-miR-1890*

aga-miR-1890

```
5'- gucguguggacggccagagcuaauuggaguauuucuugaggaauauuuuugacaagcucaugaaaucuuugauuaggucugguuauucguuacgac  -3'   exp
    ((((((((((..((((((.(((((..(((.(((((.((((...............)))).)))))))))..))))).))))))..)))).))))))    reads   mm   sample
    ...............agagcuaauuggaguauuucuug..........................................................    1       0    OV2
    ...............agagcuaauuggaguauuucuugaggaa.....................................................    1       0    OV2
    ............................................................ugaaaucuuugauuagguc.................    2       0    OV2
    ............................................................ugaaaucuuugauuaggucugg..............    1       0    OV2
    ............................................................ugaaaucuuugauuaggucuggCu............    4       1    OV2

    ...............agagcuaauuggaguauuucuu...........................................................    1       0    FF2
    ...............agagcuaauuggaguauuucuug..........................................................    1       0    FF2
    ............................................................ugaaaucuuugauuaggucu................    1       0    FF2
    ............................................................ugaaaucuuugauuaggucugg..............    2       0    FF2
    ............................................................ugaaaucuuugauuaggucuggC.............    1       1    FF2

    ...............agagcuaauuggaguauuucuu...........................................................    1       0    TE1
    ...............agagcuaauuggaguauuucuug..........................................................    5       0    TE1
    ......................................aggaauauuGuugacaagcuca....................................    1       1    TE1
    ............................................................ugaaaucuuugauuagguc.................    1       0    TE1
    ............................................................ugaaaucuuugauuaggucu................    4       0    TE1
    ............................................................ugaaaucuuugauuaggucug...............    3       0    TE1
    ............................................................ugaaaucuuugauuaggucugg..............    19      0    TE1
    ............................................................ugaaaucuuugauuaggucuggA.............    2       1    TE1
    ............................................................ugaaaucuuugauuaggucugguu............    1       0    TE1
    ............................................................ugaaaucuuugauuaggucuggCu............    2       1    TE1

    ...............agagcuaauuggaguauuucu............................................................    1       0    MF2
    ...............agagcuaauuggaguauuucuug..........................................................    9       0    MF2
    ............................................................ugaaaucuuugauuagguc.................    7       0    MF2
    ............................................................ugaaaucuuugauuaggucu................    7       0    MF2
    ............................................................ugaaaucuuugauuaggucug...............    2       0    MF2
    ............................................................Cgaaaucuuugauuaggucugg..............    1       1    MF2
    ............................................................ugaaaucuuuAauuaggucugg..............    1       1    MF2
    ............................................................ugaaaucuuugauuaggucugA..............    1       1    MF2
    ............................................................ugaaaucuuugauuaggucugg..............    45      0    MF2
    ............................................................ugaaaucuuugauuaggucugU..............    1       1    MF2
    ............................................................uAaaaucuuugauuaggucugg..............    1       1    MF2
```

```
               aga-miR-1890*
                                                            aga-miR-1890
gucguguggacggccagagcuaauuggaguauuucuugaggaauauuuuugacaagcucaugaaaucuuugauuaggucugguuauucguuacgac

...........................................................ugaaaucuuugauuaggucuggC..............    3    1    MF2
...........................................................ugaaaucuuugauuaggucuggu..............    1    0    MF2
...........................................................ugaaaucuuugauuaggucugCu..............    1    1    MF2
...........................................................ugaaaucuuugauuaggucuggA..............    12   1    MF2
...........................................................ugaaaucuuugauuaggucuggCu.............    4    1    MF2
...........................................................ugaaaucuuugauuaggucugguA.............    2    1    MF2
...........................................................ugaaaucuuugauuaggucugguu.............    1    0    MF2

...........................................................ugaaaucuuugauuagguc..................    1    0    FW2
...........................................................ugaaaucuuugauuaggucu.................    9    0    FW2
...........................................................ugaaaucuuugauuaggucug................    1    0    FW2
...........................................................ugaaaucuuugauuaggucugg...............    15   0    FW2
...........................................................ugaaaucuuugauuaggucuggu..............    1    0    FW2
...........................................................ugaaaucuuugauuaggucugguu.............    1    0    FW2

...........................................................ugaaaucuuugauuagguc..................    2    0    FF1
...........................................................ugaaaucuuugauuaggucugg...............    7    0    FF1
...........................................................ugaaaucuuugauuaggucuggA..............    1    1    FF1
...........................................................ugaaaucuuugauuaggucuggC..............    4    1    FF1

...............agagcuaauuggaguauuucuu...........................................................    2    0    OV1
......................................aggaauauuGuugacaagcuca....................................    1    1    OV1
...........................................................ugaaaucuuugauuagguc..................    1    0    OV1
...........................................................ugaaaucuuugauuaggucu.................    2    0    OV1
...........................................................ugaaaucuuugauuaggucugg...............    11   0    OV1
...........................................................ugaaaucuuugauuaggucuggA..............    1    1    OV1
...........................................................ugaaaucuuugauuaggucuggC..............    2    1    OV1
...........................................................ugaaaucuuugauuaggucuggu..............    3    0    OV1
...........................................................ugaaaucuuugauuaggucuggCu.............    6    1    OV1
...........................................................ugaaaucuuugauuaggucugguA.............    1    1    OV1
...........................................................ugaaaucuuugauuaggucugguu.............    1    0    OV1

......................................aggaauauuGuugacaagcuca....................................    1    1    MF1
...........................................................ugaaaucuuugauuagguc..................    1    0    MF1
...........................................................ugaaaucuuugauuaggucu.................    2    0    MF1
...........................................................ugaaaucuuugauuaggucug................    2    0    MF1
...........................................................ugaaaucuuugauuaggucugg...............    3    0    MF1
...........................................................ugaaaucuuugauuaggucuggC..............    3    1    MF1
...........................................................ugaaaucuuugauuaggucuggCu.............    1    1    MF1

...............agagcuaauuggaguauuucuu...........................................................    1    0    BF2
...............agagcuaauuggaguauuucuug..........................................................    2    0    BF2
......................................aggaauauuGuugacaagcuca....................................    1    1    BF2
......................................aggaauauuGuugacaagcucaugaaaucuuugauuaggucugg..............    1    1    BF2
..........................................................Cugaaaucuuugauuaggucugg...............    1    1    BF2
...........................................................ugaaaucuuugauuagguc..................    12   0    BF2
...........................................................ugaaaucuuugauuaggucu.................    1    0    BF2
...........................................................ugaaaucuuugauuaggucug................    1    0    BF2
...........................................................uAaaaucuuugauuaggucugg...............    1    1    BF2
...........................................................ugaaaucuuugUuuaggucugg...............    1    1    BF2
...........................................................ugaaaucuuugauuaggucugg...............    22   0    BF2
...........................................................ugaaaucuuugauuaggucuggu..............    3    0    BF2
...........................................................ugaaaucuuugauuaggucuggA..............    6    1    BF2
...........................................................ugaaaucuuugauuaggucuggC..............    1    1    BF2

...........................................................ugaaaucuuugauuagguc..................    2    0    BF1
...........................................................ugaaaucuuugauuaggucugg...............    7    0    BF1
...........................................................ugaaaucuuugauuaggucuggA..............    1    1    BF1
...........................................................ugaaaucuuugauuaggucuggC..............    4    1    BF1

...........................................................ugaaaucuuugauuagguc..................    2    0    FW1
...........................................................ugaaaucuuugauuaggucu.................    4    0    FW1
...........................................................ugaaaucuuugauuaggucugg...............    14   0    FW1
...........................................................ugaaaCcuuugauuaggucugg...............    1    1    FW1
...........................................................ugaaaucuuugauuaggucuggA..............    1    1    FW1
...........................................................ugaaaucuuugauuaggucuggC..............    1    1    FW1
...........................................................ugaaaucuuugauuaggucugguA.............    1    1    FW1

...............agagcuaauuggaguauuucuug..........................................................    1    0    MW1
```

```
               aga-miR-1890*
                                                            aga-miR-1890
gucguguggacggccagagcuaauuggaguauuucuugaggaauauuuuugacaagcucaugaaaucuuugauuaggucugguuauucguuacgac

......................................aggaauauuGuugacaagcuca....................................  1     1     MW1
............................................................ugaaaucuuugauuagguc.................  12    0     MW1
............................................................ugaaaucuuugauuaggucu................  1     0     MW1
............................................................ugaaaucuuugauuaggucug...............  1     0     MW1
............................................................ugaaaucuuugauuaggucugg..............  25    0     MW1
............................................................ugaaaucuuugauuaggucuggC.............  1     1     MW1
............................................................ugaaaucuuugauuaggucuggA.............  2     1     MW1
............................................................ugaaaucuuugauuaggucuggu.............  1     0     MW1

............................................................ugaaaucuuugauuagguc.................  2     0     MW2
............................................................ugaaaucuuugauuaggucu................  2     0     MW2
............................................................ugaaaucuuugauuaggucugg..............  3     0     MW2

...............agagcuaauuggaguauuucu............................................................  1     0     TE2
...............agagcuaauuggaguauuucuu...........................................................  1     0     TE2
...............agagcuaauuggaguauuucuuA..........................................................  1     1     TE2
...............agagcuaauuggaguauuucuug..........................................................  8     0     TE2
......................................aggaauauuGuugacaagcuca....................................  2     1     TE2
............................................................ugaaaucuuugauuagguc.................  9     0     TE2
............................................................ugaaaucuuugauuaggucu................  22    0     TE2
............................................................ugaaaucuuugauuaggucug...............  10    0     TE2
............................................................ugaaaucuuugauuaggucugg..............  34    0     TE2
............................................................ugaaaucuuugauuaggucugCu............  1     1     TE2
............................................................ugaaaucuuugauuaggucuggA.............  11    1     TE2
............................................................ugaaaucuuugauuaggucuggu.............  4     0     TE2
............................................................ugaaaucuuugauuaggucuggC.............  4     1     TE2
............................................................ugaaaucuuugauuaggucuggCu............  2     1     TE2
............................................................ugaaaucuuugauuaggucugguu............  2     0     TE2
```

miRBase precursor : aga-mir-1891
Total read count : 402
aga-miR-1891 read count : 316
aga-miR-1891* read count : 86
remaining reads : 0

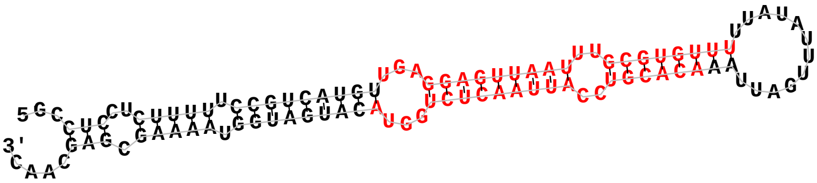

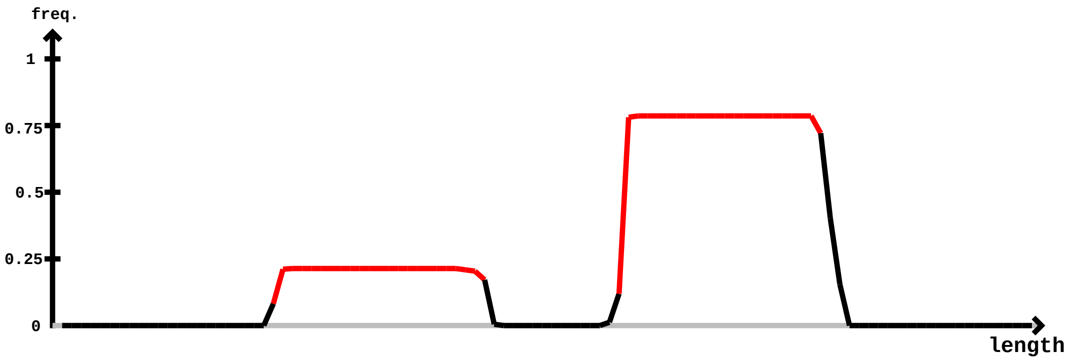


```
                    aga-miR-1891*
                                              aga-miR-1891
5'- gccuccucuuuuuccgucauguugaggaguuaauuugcguguuuuuauauuugauuaaacacguccauuaacucugguacaugaugguaaaagcgagcaac   -3'    exp
    ((.((..(((((.(((((((((...(((((((((..((((((((............))))))))..)))))))))...))))))))).))))).))))...    reads    mm    sample
    ..........................................................acacguccauuaacucugguac.....................    1        0     OV2
    ..........................................................acacguccauuaacucugguaca....................    1        0     OV2

    .....................uugaggaguuaauuugcgCg............................................................    1        1     TE1
    .....................uugaggaguuaauuugcguguu..........................................................    3        0     TE1
    .....................uugaggaguuaauuugcguguuu.........................................................    1        0     TE1
    .....................uugaggagCuaauuugcguguuu.........................................................    1        1     TE1
    ......................ugaggaguuaauuugcgugG...........................................................    1        1     TE1
    ......................ugaggaguuaauuugcguguuu.........................................................    13       0     TE1
    ........................................................aaacacguccauuaacucuggu.......................    1        0     TE1
    .........................................................aacacguccauuaacucuggu.......................    2        0     TE1
    .........................................................aacacguccauuaacucuggua......................    11       0     TE1
    .........................................................aacacguccauuaacucugguaA.....................    1        1     TE1
    ..........................................................acacguccauuaacucuggu.......................    3        0     TE1
    ..........................................................acacguccauuaacucuggua......................    29       0     TE1
    ..........................................................acacguccauuaacucugguac.....................    36       0     TE1
    ..........................................................acacguccauuaacucugguaA.....................    1        1     TE1
    ..........................................................acacguccauuaacucugguaca....................    18       0     TE1
    ..........................................................acacguccauuaacucugguaGa....................    1        1     TE1
    ...........................................................cacguccauuaacucuggua......................    1        0     TE1
    ...........................................................cacguccauuaacucugguaca....................    1        0     TE1

    .....................uugaggaguuaauuugcguguuu.........................................................    2        0     MF2
    ......................ugaggaguuaauuugcguguuG.........................................................    1        1     MF2
    ......................ugaggaguuaauuugcguguuu.........................................................    8        0     MF2
    .........................................................aacacguccauuaacucuggua......................    3        0     MF2
    .........................................................aacacguccauuaacucugguaA.....................    2        1     MF2
    ..........................................................acacguccauuaacucuggu.......................    1        0     MF2
    ..........................................................acacguccauuaacucuggua......................    8        0     MF2
    ..........................................................acacguccauuaacucugguac.....................    6        0     MF2
    ..........................................................acacguccauuaacucugguaU.....................    2        1     MF2
    ..........................................................acacguccauuaacucugguaca....................    8        0     MF2

    .....................uugaggaguuaauuugcguguuu.........................................................    1        0     OV1
```

```
                      aga-miR-1891*
                                                          aga-miR-1891
gccuccucuuuuuccgucauguugaggaguuaauuugcguguuuuuauauuugauuaaacacguccauuaacucugguacaugaugguaaaagcgagcaac

.....................uugaggaguuaauuugcguguuu.........................................................    1    0    OV1
..........................................................acacguccauuaacucuggua......................    2    0    OV1
..........................................................acacguccauuaacucugguaA.....................    1    1    OV1
..........................................................acacguccauuaacucugguaca....................    1    0    OV1

.....................uugaggaguuaauuugcguguu..........................................................    1    0    FF1
.....................uugaggaguuaauuugcguguuu.........................................................    2    0    FF1
.........................................................aacacguccauuaacucuggua......................    1    0    FF1
..........................................................acacguccauuaacucugguac.....................    1    0    FF1
..........................................................acacguccauuaacucugguaca....................    2    0    FF1

.........................................................aacacguccauuaacucuggua......................    2    0    MF1
..........................................................acacguccauuaacucuggua......................    1    0    MF1
..........................................................acacguccauuaacucugguac.....................    1    0    MF1

.....................uugaggaguuaauuugcguguuu.........................................................    1    0    BF2
.....................uugaggaguuaauuugcguguuAu........................................................    1    1    BF2
......................ugaggaguuaauuugcguguuu.........................................................    2    0    BF2
..........................................................acacguccauuaacucuggua......................    1    0    BF2
..........................................................acacguccauuaacucugguac.....................    2    0    BF2
..........................................................acacguccauuaacucugguaca....................    1    0    BF2

.....................uugaggaguuaauuugcguguu..........................................................    1    0    BF1
.....................uugaggaguuaauuugcguguuu.........................................................    2    0    BF1
.........................................................aacacguccauuaacucuggua......................    1    0    BF1
..........................................................acacguccauuaacucugguac.....................    1    0    BF1
..........................................................acacguccauuaacucugguaca....................    2    0    BF1

.....................uugaggaguuaauuugcgug............................................................    1    0    TE2
.....................uugaggaguuaauuugcgugu...........................................................    1    0    TE2
.....................uugaggaguuaauuugcguguu..........................................................    8    0    TE2
.....................uugaggaguuaauuugcguguuu.........................................................    5    0    TE2
......................ugaggaguuaauuugcguguuu.........................................................    27   0    TE2
.......................gaggaguuaauuugcguguuu.........................................................    1    0    TE2
........................................................aaacacguccauuaacucuggu.......................    1    0    TE2
........................................................aaacacguccauuaacucuggua......................    1    0    TE2
........................................................aaacacguccauuaacucugguac.....................    2    0    TE2
.........................................................aacacguccauuaacucuggu.......................    5    0    TE2
.........................................................aacacguccauuaacucuggua......................    14   0    TE2
.........................................................aacacguccauuaacucugguac.....................    1    0    TE2
..........................................................acacguccauuaacucuggu.......................    13   0    TE2
..........................................................acacguccauAaacucuggua......................    1    1    TE2
..........................................................acacguccauuaacucuggua......................    51   0    TE2
..........................................................acacguccauuGacucuggua......................    1    1    TE2
..........................................................acacguccauuaacucugUuac.....................    1    1    TE2
..........................................................acacguccauuaacucugguac.....................    41   0    TE2
..........................................................acacguccauuaacucugguaca....................    26   0    TE2
..........................................................acacguccauuaacucugguacU...................    1    1    TE2
```

miRBase precursor : aga-mir-190
Total read count : 2571
aga-miR-190 read count : 2551
aga-miR-190* read count : 19
remaining reads : 1

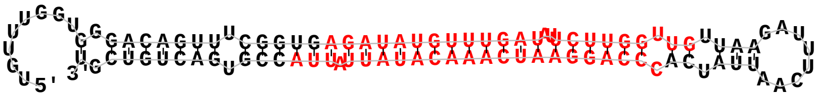

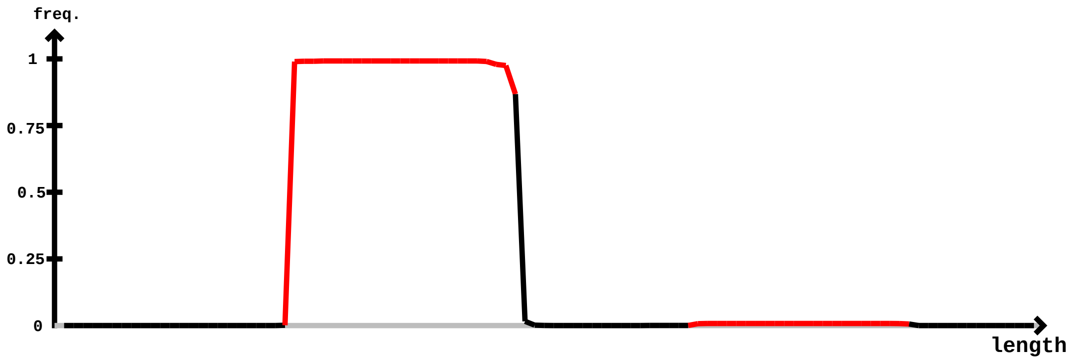


```
                                                                             aga-miR-190*
                        aga-miR-190
5'- uguuuggugggacaguuucggugagauauguuugauauucuugguuguuaagauuucaauuaucacccaggaaucaaacauauuauuaccgugacugucgu  -3'    exp
    ..........(((((((.((((((((((((((((((..((((((.((.(((........))).)).))))))))))))))))))).)))))).)))))))..    reads     mm        sample
    .......................agauauguuugauauucuugguu........................................................    5         0         OV2
    .......................agauauguuugauaCucuugguu........................................................    1         1         OV2
    .......................agauauguuugCuauucuugguug.......................................................    1         1         OV2
    .......................agauauguuugauauucuuCguug.......................................................    1         1         OV2
    .......................agauauguuugauauucuugguug.......................................................    94        0         OV2
    .......................agauauguuugauauucuugguugu......................................................    1         0         OV2
    .................................................................cccaggaaucaaacauauuau...............    1         0         OV2

    .......................agauauguuugauauucuugg..........................................................    1         0         FF2
    .......................agauauguuugauauucuugguu........................................................    2         0         FF2
    .......................agauauguuugauauucuugguu........................................................    12        0         FF2
    .......................agauaugCuugauauucuugguu........................................................    1         1         FF2
    .......................Ugauauguuugauauucuugguug.......................................................    1         1         FF2
    .......................agauauguuugauauucuugguug.......................................................    70        0         FF2
    .................................................................cccaggaaucaaacauauuauu..............    1         0         FF2
    .................................................................cccaggaaucaaacauauuauua.............    1         0         FF2

    .......................agauauguuugauauucuugg..........................................................    1         0         TE1
    .......................agauauguuugauauucuugguu........................................................    11        0         TE1
    .......................agauauguuugauauucuugguug.......................................................    48        0         TE1
    .......................agauauguuugauauucuugguugu......................................................    1         0         TE1
    .................................................................cccaggaaucaaacauauuauuU.............    2         1         TE1
    ..................................................................ccaggaaucaaacauauuauua.............    2         0         TE1

    ......................Cagauauguuugauauucuugguu........................................................    1         1         MF2
    ......................Cagauauguuugauauucuugguug.......................................................    2         1         MF2
    .......................agauauguuugauauucuug...........................................................    4         0         MF2
    .......................agauauguuugauauucuugg..........................................................    4         0         MF2
    .......................agauauguuugauauucuugguu........................................................    4         0         MF2
    .......................agauauguuugauauucuugguG........................................................    2         1         MF2
    .......................agauauguuugauauucuugguu........................................................    125       0         MF2
    .......................agauauguuugauauCcuugguug.......................................................    1         1         MF2
    .......................agauauguuugauaCucuugguug.......................................................    1         1         MF2
    .......................agauaugCuugauauucuugguug.......................................................    2         1         MF2
```

```
                                                                 aga-miR-190*
                       aga-miR-190
uguuuggugggacaguuucggugagauauguuugauauucuugguuguuaagauuucaauuaucacccaggaaucaaacauauuauuaccgugacugucgu

.......................agauauguuugUuauucuugguug......................................................  1    1  MF2
.......................agauauguuuUauauucuugguug......................................................  1    1  MF2
.......................agauauguuugauauucuugguug......................................................  866  0  MF2
.......................agauauguuugauauucuuggAug......................................................  2    1  MF2
.......................agauauguCugauauucuugguug......................................................  1    1  MF2
.......................agauauguGugauauucuugguug......................................................  1    1  MF2
.......................agauauguuugaCauucuugguug......................................................  1    1  MF2
.......................agaCauguuugauauucuugguug......................................................  1    1  MF2
.......................agauauguuugauauucuugguugC.....................................................  2    1  MF2
.......................agauauguuugUuauucuugguugu.....................................................  1    1  MF2
.......................agauauguuugauauucuugguugu.....................................................  15   0  MF2
.......................agauauguuugauauucuugguuguu....................................................  1    0  MF2
.......................agauauguuugauauucuugguugCu....................................................  1    1  MF2
.......................agauauguuugauauucuugguuguCa...................................................  1    1  MF2
..........................uauguuugauauucuugguug......................................................  1    0  MF2
.................................................................cccaggaaucaaacauauuauu..............  1    0  MF2
.................................................................cccaggaaucaaacauauuauua.............  7    0  MF2

.......................agauauguuugauauucuugg.........................................................  9    0  FW2
.......................agauauguuugauauucuuggu........................................................  1    0  FW2
.......................agauauguuugauauucuugguu.......................................................  32   0  FW2
.......................agauauguuugCuauucuugguu.......................................................  1    1  FW2
.......................agauauguuugauauucuugguug......................................................  70   0  FW2
.......................agauauguuugauauucuugguugu.....................................................  2    0  FW2

.......................agauauguuugauauucuugguu.......................................................  2    0  FF1
.......................agauauguuugauauucuugguug......................................................  169  0  FF1
........................gauauguuugauauucuugguug......................................................  1    0  FF1

.......................agauauguuugauauucuuggu........................................................  1    0  OV1
.......................agauauguuugauauucuugguu.......................................................  4    0  OV1
.......................agauauguuugauauucuugguug......................................................  149  0  OV1
.......................agGuauguuugauauucuugguug......................................................  1    1  OV1
.......................agauauguuCgauauucuugguug......................................................  1    1  OV1
.......................agauauguuugauauucuuggAug......................................................  1    1  OV1
.......................agauauguuugCuauucuugguug......................................................  1    1  OV1
.......................agauauguuugauauucuuggGug......................................................  2    1  OV1
.......................agauauguuugauauucuugguugA.....................................................  1    1  OV1
.......................agauauguuugauauucuugguugu.....................................................  3    0  OV1

.......................agauauguuugauauucuugg.........................................................  2    0  MF1
.......................agauauguuugauauucuuggu........................................................  1    0  MF1
.......................agauauguuugauauucuugguG.......................................................  1    1  MF1
.......................agauauguuugauauucuugguu.......................................................  24   0  MF1
.......................agauauguGugauauucuugguug......................................................  1    1  MF1
.......................agauauguuugauauucuugguug......................................................  124  0  MF1
.......................agauauguuugauauucuugguugu.....................................................  2    0  MF1
.................................................................cccaggaaucaaacauauuauu..............  1    0  MF1

.......................agauauguuugauauucuugguu.......................................................  2    0  BF2
.......................agauauguuugauaCucuugguug......................................................  2    1  BF2
.......................agauauguuugauauucuuAguug......................................................  1    1  BF2
.......................agauauguuugauauucuugguug......................................................  120  0  BF2
.......................agauauguuugauauucuugguugu.....................................................  3    0  BF2
..........................uauguuugauauucuugguug......................................................  2    0  BF2

.......................agauauguuugauauucuugguu.......................................................  2    0  BF1
.......................agauauguuugauauucuugguug......................................................  169  0  BF1
........................gauauguuugauauucuugguug......................................................  1    0  BF1

.......................agauauguuugauauucuugg.........................................................  6    0  MW1
.......................agauauguuugauauucuuggu........................................................  2    0  MW1
.......................agauauguuugauauucuugguu.......................................................  7    0  MW1
.......................agauaAguuugauauucuugguu.......................................................  1    1  MW1
.......................agauauguuugauauucuugguug......................................................  51   0  MW1
.......................agauauguuugauauucuugguugu.....................................................  1    0  MW1

.......................agauauguuugauauucuug..........................................................  1    0  FW1
.......................agauauguuugauauucuugg.........................................................  1    0  FW1
```

```
                                                                 aga-miR-190*
                       aga-miR-190
uguuuggugggacaguuucggugagauauguuugauauucuugguuguuaagauuucaauuaucacccaggaaucaaacauauuauuaccgugacugucgu

.......................agauauguuugauauucuugguu.......................................................   9     0   FW1
.......................agauauguCugauauucuugguug......................................................   1     1   FW1
.......................agauauguuugauauucuugguug......................................................   45    0   FW1
.......................agauauguuugauauucuugguuguu....................................................   1     0   FW1

.......................agauauguuugauauucuugg.........................................................   1     0   MW2
.......................agauauguuugauauucuugguu.......................................................   2     0   MW2
.......................agauauguuugauauucuugguug......................................................   13    0   MW2
.......................agauauguuugauauucuugguugu.....................................................   1     0   MW2
........................gauauguuugauauucuugguug......................................................   1     0   MW2
............................................................uaucacccaggaaucaaacauauuauua.............   1     0   MW2

.......................agauauguuugauauucuugg.........................................................   3     0   TE2
.......................agauauguuugauauucuugguu.......................................................   27    0   TE2
.......................agauauguuugauauucuugguA.......................................................   3     1   TE2
.......................agauauguuugGuauucuugguu.......................................................   1     1   TE2
.......................agauauguuugauauuAuugguug......................................................   1     1   TE2
.......................agauauguuugauauucuugguug......................................................   166   0   TE2
.......................agauauUuuugauauucuugguug......................................................   1     1   TE2
.......................agauauguuugauauucuugguugA.....................................................   1     1   TE2
.......................agauauguuugauauucuugguugu.....................................................   3     0   TE2
.................................................................cccaggaaucaaacauauuauu..............   1     0   TE2
.................................................................cccaggaaucaaacauauuauua.............   2     0   TE2
```

miRBase precursor : aga-mir-1
Total read count : 2401
aga-miR-1 read count : 2396
aga-miR-1* read count : 4
remaining reads : 1

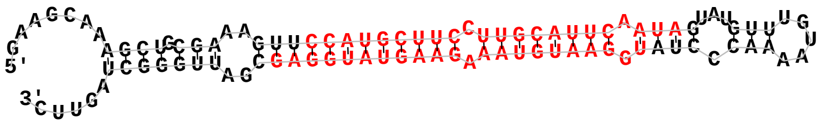

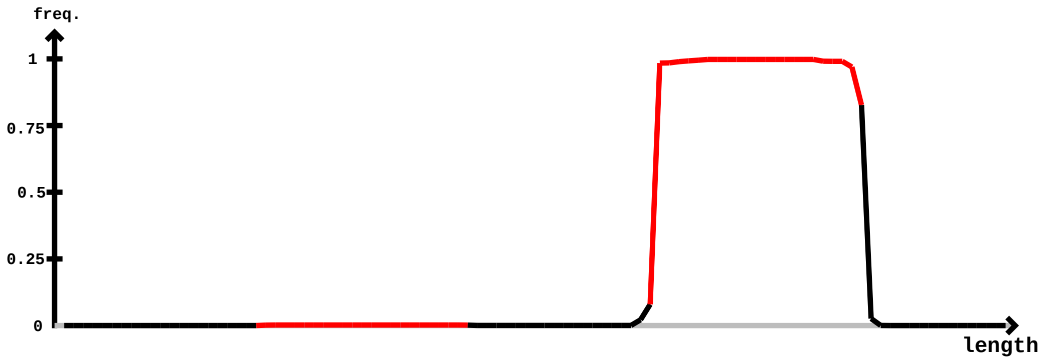


```
aga-miR-1*
aga-miR-1
5'- gaagcaaagcugcgaaaguuccaugcuuccuugcauucaauaguauguuuguaaaacccuauggaauguaaagaaguauggagcgauugggcuaguuc -3'   exp
    .......((((.(((..(((((((((((.((((((((.((((...(((.....))).)))).)))))))).)))))))))))..))))))).......   reads   mm   sample
    ...........................................................uauggaauguaaagaaguauggagA..............   1   1   OV2
    ............................................................auggaauguaaagaaguaugg.................   1   0   OV2
    ............................................................auggaauguaaagaaguaugga................   4   0   OV2
    ............................................................auggaauguaaagaaguauggag...............   1   0   OV2
    .............................................................uggaauguaaagaagua....................   2   0   OV2
    .............................................................uggaauguaaagaaguaugg.................   1   0   OV2
    .............................................................uggaauguaaagaaguaugga................   19   0   OV2
    .............................................................uggaauguaaagaagCauggag...............   1   1   OV2
    .............................................................uggaauguaaagaaguauggaA...............   1   1   OV2
    .............................................................uggaauguaaagaaguauggag...............   51   0   OV2
    .............................................................uggaauguaaagaaguauggagA..............   1   1   OV2
    .............................................................uggaauguaaagaaguauggagU..............   1   1   OV2

    ...........................................................uauggaauguaaagaaguauggaU...............   1   1   TE1
    ............................................................auggaauguaaagaaguaugga................   1   0   TE1
    ............................................................auggaauguaaagaaguauggag...............   1   0   TE1
    .............................................................uggaauguaaagaaguaugg.................   1   0   TE1
    .............................................................uggaauguaaagaaguaugga................   2   0   TE1
    .............................................................uAgaauguaaagaaguauggag...............   1   1   TE1
    .............................................................uggaauguaaagaaguauggag...............   11   0   TE1

    ...........................................................uauggaauguaaagaaguaugg.................   1   0   FF2
    ...........................................................uauggaauguaaagaaguaugga................   2   0   FF2
    ...........................................................uauggaauguaaagaaguauggaA...............   1   1   FF2
    ............................................................auggaauguaaagaagua....................   1   0   FF2
    ............................................................Cuggaauguaaagaaguaugga................   1   1   FF2
    ............................................................auggaauguaaagaaguaugga................   11   0   FF2
    ............................................................Cuggaauguaaagaaguauggag...............   1   1   FF2
    ............................................................auggaauguaaagaaguauggaU...............   1   1   FF2
    ............................................................auggaauguaaagaaguauggag...............   2   0   FF2
    .............................................................uggaauguaaagaaguaugg.................   5   0   FF2
    .............................................................uggaauguaaagaaguaugga................   12   0   FF2
    .............................................................uggaauguaaagaaguauggag...............   136   0   FF2
    .............................................................uggaauguaaagaaguauggagA..............   3   1   FF2
```

```
                    aga-miR-1*
                                                             aga-miR-1
gaagcaaagcugcgaaaguuccaugcuuccuugcauucaauaguauguuuguaaaacccuauggaauguaaagaaguauggagcgauugggcuaguuc

.............................................................uggaauguaaagaaguauggagU..............   3     1     FF2
...............................................................gaauguaaagaaguauggag...............   1     0     FF2

....................ccaugcuuccuugcauucaaua........................................................   1     0     MF2
...........................................................uauggaauguaaagaagua....................   2     0     MF2
...........................................................uauggaauguaaagaaguaugg.................   2     0     MF2
...........................................................uauggaauguaaagaaguaugga................   19    0     MF2
...........................................................uauggaauguaaagaaguauggag...............   1     0     MF2
...........................................................uauggaauguaaagaaguauggaU...............   1     1     MF2
............................................................auggaauguaaagaagua....................   1     0     MF2
............................................................auggaauguaaagaaguaugg.................   2     0     MF2
............................................................auggaaugAaaagaaguaugg.................   1     1     MF2
............................................................auggaauguaaagaaguaugga................   33    0     MF2
............................................................auggaauguaaagaaguauggaA...............   3     1     MF2
............................................................auggaauguaaagaaguauggag...............   20    0     MF2
............................................................Cuggaauguaaagaaguauggag...............   1     1     MF2
............................................................auggaauguaaagaaguauggaU...............   1     1     MF2
............................................................auggaauguaaagaaguauggagA..............   2     1     MF2
.............................................................uggaauguaaagaagua....................   4     0     MF2
.............................................................uggaauguaaagaaguau...................   1     0     MF2
.............................................................uggaauguaaagaaguaugg.................   9     0     MF2
.............................................................uggaauguaaagaaguaugga................   73    0     MF2
.............................................................ugAaauguaaagaaguaugga................   1     1     MF2
.............................................................uggaauguaaagaaguaugAa................   1     1     MF2
.............................................................uggaauguaaagaagGauggag...............   1     1     MF2
.............................................................uggaauguaaagaaguauggag...............   837   0     MF2
.............................................................uggaauguaaagaaguauggaC...............   2     1     MF2
.............................................................uggaaugCaaagaaguauggag...............   1     1     MF2
.............................................................ugAaauguaaagaaguauggag...............   1     1     MF2
.............................................................uggaauAuaaagaaguauggag...............   1     1     MF2
.............................................................uggaauCuaaagaaguauggag...............   1     1     MF2
.............................................................uggaauguaaagaaguaugAag...............   1     1     MF2
.............................................................uggaauguaaagaaguauggGg...............   1     1     MF2
.............................................................uggaauguGaagaaguauggag...............   1     1     MF2
.............................................................uggaauguaaagaaguauggaA...............   1     1     MF2
.............................................................uggUauguaaagaaguauggag...............   1     1     MF2
.............................................................uggaauguaaagaaguauAgag...............   1     1     MF2
.............................................................uggaauguaaagaaguauggagU..............   7     1     MF2
.............................................................uggaauguaaagaaguauggagc..............   2     0     MF2
.............................................................uggaauguaaagaaguauggagA..............   21    1     MF2
..............................................................ggaauguaaagaaguauggag...............   1     0     MF2
...............................................................gaauguaaagaaguauggag...............   1     0     MF2
................................................................aauguaaagaaguauggag...............   2     0     MF2

....................ccaugcuuccuugcauucaau.........................................................   1     0     FW2
...........................................................uauggaauguaaagaaguaugga................   1     0     FW2
...........................................................uauggaauguaaagaaguauggaA...............   2     1     FW2
............................................................auggaauguaaagaaguaugga................   7     0     FW2
............................................................auggaauguaaagaaguauggag...............   1     0     FW2
............................................................auggaauguaaagaaguauggagA..............   1     1     FW2
.............................................................uggaauguaaagaaguaugga................   9     0     FW2
.............................................................uggaauguaaaUaaguauggag...............   1     1     FW2
.............................................................uggaauguaaagaaguauggag...............   97    0     FW2
.............................................................uggaauguaaagaaguauggagA..............   1     1     FW2
...............................................................gaauguaaagaaguauggag...............   2     0     FW2
...............................................................gaauguaaagaaguauggaA...............   1     1     FW2
................................................................aauguaaagaaguauggag...............   2     0     FW2
.................................................................auguaaagaaguauggag...............   1     0     FW2
..................................................................uguaaagaaguauggag...............   2     0     FW2

...........................................................uauggaauguaaagaaguaugg.................   1     0     FF1
............................................................auggaauguaaagaaguaugg.................   1     0     FF1
............................................................auggaauguaaagaaguaugga................   2     0     FF1
............................................................auggaauguaaagaaguauggag...............   2     0     FF1
.............................................................uggaauguaaagaaguaugg.................   3     0     FF1
.............................................................uggaauguaaagaaguaugga................   8     0     FF1
.............................................................uggaauguaaagaaguauggag...............   38    0     FF1
.............................................................uggaauguaaagaaguauggagU..............   1     1     FF1
.............................................................uggaauguaaagaaguauggagA..............   1     1     FF1
```

```
                    aga-miR-1*
                                                                    aga-miR-1
gaagcaaagcugcgaaaguuccaugcuuccuugcauucaauaguauguuuguaaaacccuauggaauguaaagaaguauggagcgauugggcuaguuc


...........................................................uauggaauguaaagaaguaugg.................   1     0     OV1
...........................................................uauggaauguaaagaaguaugga................   1     0     OV1
............................................................auggaauguaaagaagua....................   1     0     OV1
............................................................auggaauguaaagaaguaugg.................   1     0     OV1
............................................................auggaauguaaagaaguaugga................   1     0     OV1
............................................................auggaauguaaagaaguauggag...............   1     0     OV1
.............................................................uggaauguaaagaaguaugga................   18    0     OV1
.............................................................uggaauguaaagaaguauggag...............   70    0     OV1
.............................................................uggaauguaaagaaguauggaU...............   1     1     OV1
.............................................................uggaauguaaagaaguauggagA..............   2     1     OV1
.............................................................uggaauguaaagaaguauggagU..............   2     1     OV1

....................ccaugcuuccuugcauucaauaguauguuuguaaaaccc.......................................   1     0     MF1
...........................................................uauggaauguaaagaaguaugga................   4     0     MF1
............................................................auggaauguaaagaaguaugga................   4     0     MF1
............................................................auggaauguaaagaaguauggag...............   1     0     MF1
.............................................................uggaauguaaagaaguaugg.................   2     0     MF1
.............................................................uggaauguaaagaaguaugga................   9     0     MF1
.............................................................uggaauguaaagaaguauggaC...............   1     1     MF1
.............................................................uggaauguaaagaaguauggag...............   141   0     MF1
.............................................................uggaauguaaagaaguauggagA..............   2     1     MF1
.............................................................uggaauguaaagaaguauggagU..............   2     1     MF1
..................................................................uguaaagaaguauggag...............   1     0     MF1

...........................................................uauggaauguaaagaaguaugga................   4     0     BF2
............................................................auggaauguaaagaagua....................   1     0     BF2
............................................................auggaauguaaagaaguaugga................   3     0     BF2
.............................................................uggaauguaaagaagua....................   1     0     BF2
.............................................................uggaauguaaagaaguaugg.................   11    0     BF2
.............................................................uggaauguaaagaaguaugga................   39    0     BF2
.............................................................Cggaauguaaagaaguauggag...............   1     1     BF2
.............................................................uggaauguaaagaaguauggaU...............   1     1     BF2
.............................................................uggaauguaaagaaguauggag...............   136   0     BF2
.............................................................uggaauguaaagaaguauggagU..............   1     1     BF2
.............................................................uggaauguaaagaaguauggagcA.............   1     1     BF2
..............................................................ggaauguaaagaaguauggag...............   1     0     BF2
................................................................aauguaaagaaguauggag...............   3     0     BF2
.................................................................auguaaagaaguaugga................   1     0     BF2
.................................................................auguaaagaaguauggag...............   1     0     BF2
..................................................................uguaaagaaguauggag...............   2     0     BF2

...........................................................uauggaauguaaagaaguaugg.................   1     0     BF1
............................................................auggaauguaaagaaguaugg.................   1     0     BF1
............................................................auggaauguaaagaaguaugga................   2     0     BF1
............................................................auggaauguaaagaaguauggag...............   2     0     BF1
.............................................................uggaauguaaagaaguaugg.................   3     0     BF1
.............................................................uggaauguaaagaaguaugga................   8     0     BF1
.............................................................uggaauguaaagaaguauggag...............   38    0     BF1
.............................................................uggaauguaaagaaguauggagU..............   1     1     BF1
.............................................................uggaauguaaagaaguauggagA..............   1     1     BF1

...........................................................uauggaauguaaagaaguaugga................   1     0     FW1
............................................................auggaauguaaagaaguaugga................   1     0     FW1
............................................................auggaauguaaagaaguauggag...............   2     0     FW1
.............................................................uggaauguaaagaagua....................   1     0     FW1
.............................................................uggaauguaaagaaguaugg.................   1     0     FW1
.............................................................uggaauguaaagaaguaugga................   6     0     FW1
.............................................................uggaauguaaagaaguauggag...............   63    0     FW1
.............................................................uggaauguaaagaaguauggagU..............   2     1     FW1

.....................caugcuuccuugcauucaaua........................................................   1     0     MW1
...........................................................uauggaauguaaagaaguaugga................   3     0     MW1
............................................................auggaauguaaagaaguaugga................   3     0     MW1
............................................................auggaauguaaagaaguauggag...............   3     0     MW1
.............................................................uggaauguaaagaaguaugga................   17    0     MW1
.............................................................uggaaugCaaagaaguauggag...............   1     1     MW1
.............................................................uggaauguaaagaaguauggag...............   131   0     MW1
.............................................................uggaauguaaagaaguauggaA...............   1     1     MW1
```

```
                    aga-miR-1*
                                                             aga-miR-1
gaagcaaagcugcgaaaguuccaugcuuccuugcauucaauaguauguuuguaaaacccuauggaauguaaagaaguauggagcgauugggcuaguuc

.............................................................uggaauguaaagaaguauggagU..............   2   1   MW1
.............................................................uggaauguaaagaaguauggagA..............   2   1   MW1
...............................................................gaauguaaagaaguauggag...............   4   0   MW1
.................................................................auguaaagaaguauggag...............   3   0   MW1

....................ccaugcuuccuugcauucaaua........................................................   1   0   MW2
...........................................................uauggaauguaaagaaguaugga................   2   0   MW2
............................................................auggaauguaaagaaguaugg.................   1   0   MW2
............................................................auggaauguaaagaaguaugga................   3   0   MW2
............................................................auggaauguaaagaaguauggag...............   1   0   MW2
.............................................................uggaauguaaagaagua....................   2   0   MW2
.............................................................uggaauguaaagaaguaugga................   4   0   MW2
.............................................................uggaauguaaagaaguauggag...............   32  0   MW2
...............................................................gaauguaaagaaguaugga................   1   0   MW2
...............................................................gaauguaaagaaguauggag...............   1   0   MW2
..................................................................uguaaagaaguauggag...............   2   0   MW2

............................................................auggaauguaaagaaguaugg.................   1   0   TE2
............................................................auggaauguaaagaaguaugga................   1   0   TE2
............................................................auggaauguaaagaaguauggag...............   2   0   TE2
.............................................................uggaauguaaagaaguaugga................   1   0   TE2
.............................................................uggaauguaaagaaguauggag...............   35  0   TE2
```

miRBase precursor : aga-mir-210
Total read count : 88
aga-miR-210 read count : 87
aga-miR-210* read count : 0
remaining reads : 1

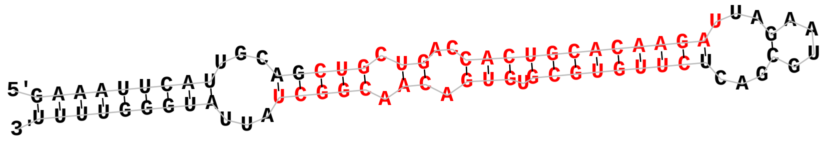

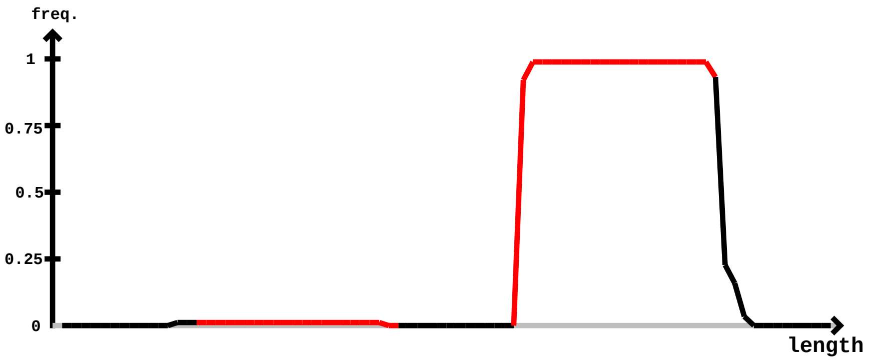


```
                                                  aga-miR-210
                      aga-miR-210*
5'- gaaauucauugcagcugcugaccacugcacaagauuagaaugcgacucuugugcgugugacaacggcuauuauggguuuu   -3'    exp
    ((((((((...(((((.((..((((((((((((...(....)...))))))))).))).)).)))))...))))))))   reads      mm         sample
    ...............................................cuugugcgugugacaacggcu............   2          0          TE1

    ...............................................cuugugcgugugacaacggcu............   7          0          OV2

    ...............................................cuugugcgugugacaacggc.............   2          0          FW2
    ...............................................cuugugcgugugacaacggcu............   12         0          FW2
    ...............................................cuugugcgugugacaacggcuau..........   1          0          FW2
    ...............................................cuugugcgugugacaacggcuauu.........   1          0          FW2
    ................................................uugugcgugugacaacggcuauu.........   1          0          FW2

    ...............................................cuugugcgugugacaacggcu............   3          0          FF1
    ...............................................cuugugcgugugacaacggcua...........   1          0          FF1
    ...............................................cuugugcgugugacaacggcuau..........   1          0          FF1

    ...............................................cuugugcgugugacaacggcu............   3          0          OV1

    ...............................................cuugugcgugugacaacggcu............   2          0          BF2
    ...............................................cuugugcgugugacaacggcuau..........   1          0          BF2
    ................................................uugugcgugugacaacggcu............   1          0          BF2

    ...............................................cuugugcgugugacaacggcu............   3          0          BF1
    ...............................................cuugugcgugugacaacggcua...........   1          0          BF1
    ...............................................cuugugcgugugacaacggcuau..........   1          0          BF1

    ...........cagcugcugaccacugcacaag...............................................   1          0          MW1
    ...............................................cuugugcgugugacaacggcu............   12         0          MW1
    ...............................................cuugugcgugugacaacggcua...........   2          0          MW1
    ...............................................cuugugcgugugacaacggcuau..........   5          0          MW1
    ...............................................cuugugcgugugacaacggcuauu.........   1          0          MW1

    ...............................................cuugugcgugugacaacggcu............   7          0          FW1
    ...............................................cuugugcgugugacaacggcua...........   1          0          FW1
    ................................................uugugcgugugacaacggcu............   2          0          FW1
    ................................................uugugcgugugacaacggcua...........   1          0          FW1
```

```
                                               aga-miR-210
              aga-miR-210*
gaaauucauugcagcugcugaccacugcacaagauuagaaugcgacucuugugcgugugacaacggcuauuauggguuuu


...............................................cuugugcgugugacaacggcu............    4          0          MW2
...............................................cuugugcgugugacaacggcG............    1          1          MW2
...............................................cuugugcgugugacaacggcuau..........    2          0          MW2
................................................uugugcgugugacaacggcu............    1          0          MW2

...............................................cuugugcgugugacaacggc.............    3          0          TE2
...............................................cuugugcgugugacaacggcu............    2          0          TE2
```

miRBase precursor : aga-mir-219
Total read count : 43
aga-miR-219 read count : 38
aga-miR-219* read count : 5
remaining reads : 0

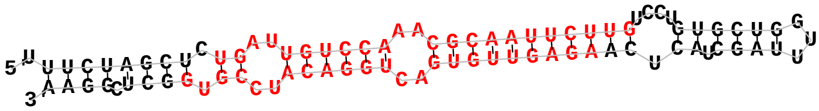

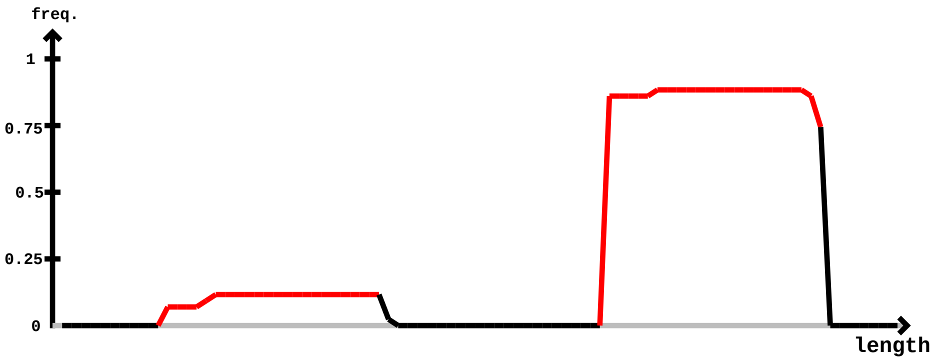

aga-miR-219*

aga-miR-219

```
5'- uuucuagcucugauuguccaaacgcaauucuuguccugugcugguuuagcuacucaagaguugugacuggacauccguggcucggaa  -3'    exp
    .((((((((.((..((((((..(((((((((((....((((((...)))).)).)))))))))))..))))))..)).)))).))))   reads      mm         sample
    ........................................................agaguugugacuggacauccgug........   2          0          MF1

    ..........ugauuguccaaacgcaauucuug......................................................   2          0          MW1
    ...............guccaaacgcaauucuug......................................................   1          0          MW1
    ........................................................agaguugugacuggacauccg..........   1          0          MW1
    ........................................................agaguugugacuggacauccgu.........   3          0          MW1
    ........................................................agaguugugacGggacauccgug........   1          1          MW1
    ........................................................agaguugugacuggacauccgug........   18         0          MW1
    .............................................................ugugacuggacauccgug........   1          0          MW1

    ..........ugauuguccaaacgcaauucuugC.....................................................   1          1          MW2
    ........................................................agaguugugacuggacauccgug........   2          0          MW2

    ........................................................agaguugugacuggacauccgug........   4          0          MF2

    ........................................................agaguugugacuggacauccgu.........   1          0          TE2

    ..............uguccaaacgcaauucuug......................................................   1          0          FW2
    ........................................................agaguugugacuggacauccgu.........   1          0          FW2
    ........................................................agaguugugacuggacauccgug........   4          0          FW2
```

miRBase precursor	: aga-mir-2-1
Total read count	: 29270
aga-miR-2 read count	: 2721
aga-miR-2* read count	: 26546
remaining reads	: 3

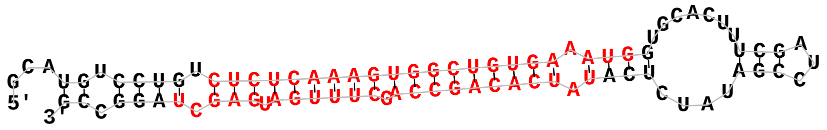

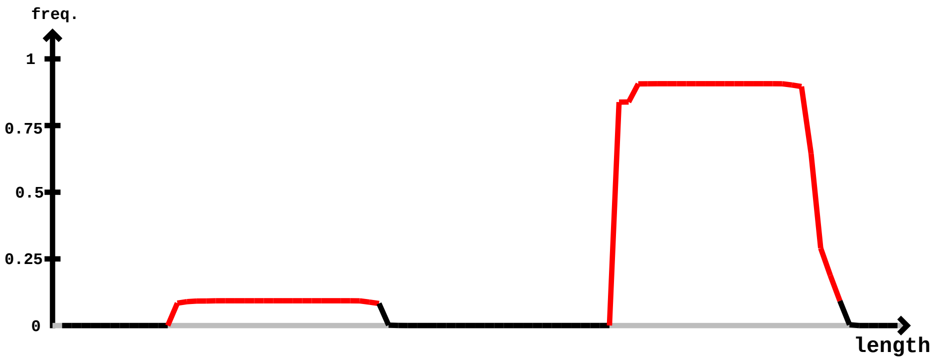


```
                        aga-miR-2
                                                                aga-miR-2*
5'- gcauguccugucucucaaaguggcugugaaauggugcacuuucgauccgauaucucauaucacagccagcuuugaugagcuaggccg   -3'    exp
    ....(.((((.((((((((((((((((((.((((.......(((...)))....)))).))))))))).)))))).))).)))).).    reads     mm      sample
    ...........cucucaaaguggcugugaaau.......................................................    1         0       TE1
    ...........cucucaaaguggcugugaaaug......................................................    12        0       TE1
    ............ucucaaaguUgcugugaaaug......................................................    1         1       TE1
    ............ucucaaaguggcugugaaaug......................................................    3         0       TE1
    ...............caaaguggcugugaaauA......................................................    2         1       TE1
    .........................................................uaucacagccagcuuuga............    4         0       TE1
    .........................................................uaucacagccagcuuugaA...........    1         1       TE1
    .........................................................uaucacagccagcuuugaAg..........    77        1       TE1
    .........................................................uaucacagccagcuuugaug..........    7         0       TE1
    .........................................................uaucacagccagcuuugauga.........    10        0       TE1
    .........................................................uaucacagccagcuuugaAga.........    196       1       TE1
    .........................................................uaucacagccagcuuugaugag........    15        0       TE1
    .........................................................uaucacagccagcuuugaAgag........    132       1       TE1
    .........................................................uaucacagccagcuuugaAgagc.......    76        1       TE1
    .........................................................uaucacagccagcuuugaugagc.......    45        0       TE1
    .........................................................uaucacagccagcuuugaugagcu......    106       0       TE1
    .........................................................uaucacagccagcuuuAaugagcu......    1         1       TE1
    .........................................................uaucacagccagcuuugaugaUcu......    1         1       TE1
    .........................................................uaucacagccagcuuugaAgagcu......    2         1       TE1
    .........................................................uaucacagccagcuuugaugagcG......    1         1       TE1
    .........................................................uaucacagccagcuuugaugagcua.....    1         0       TE1
    .........................................................uaucacagccagcuuugaugagcuC.....    2         1       TE1
    .........................................................uaucacagccagcuuugaugagcuU.....    2         1       TE1
    ..........................................................aucacagccagcuuugaAga.........    1         1       TE1
    ...........................................................ucacagccagcuuugaug..........    1         0       TE1
    ...........................................................ucacagccagcuuugauga.........    1         0       TE1
    ...........................................................ucacagccagcuuugaugag........    29        0       TE1
    ...........................................................ucacagccagcuuugaugagc.......    72        0       TE1

    ...........cucucaaaguggcugugaaa........................................................    8         0       FF2
    ...........cucucaaaguggcugugaaau.......................................................    11        0       FF2
    ...........cucucaaaguggcugugaaaug......................................................    161       0       FF2
    ...........cucucaaaguggcugugGaaug......................................................    1         1       FF2
    ...........cucucaaaguUgcugugaaaug......................................................    1         1       FF2
```

```
           aga-miR-2
                                                         aga-miR-2*
gcauguccugucucucaaaguggcugugaaauggugcacuuucgauccgauaucucauaucacagccagcuuugaugagcuaggccg

...........cucucaaaguggcugugaaauU......................................................  2     1    FF2
...........cucucaaaguggcuguAaaaug......................................................  1     1    FF2
...........cucucaaaguggcugugaaauA......................................................  1     1    FF2
...........cucucaaaguggcugugaaaugA.....................................................  2     1    FF2
...........cucucaaaguggcugugaaaugU.....................................................  3     1    FF2
............ucucaaaguggcugugaaaug......................................................  2     0    FF2
.............cucaaaguggcugugaaaug......................................................  2     0    FF2
..............ucaaaguggcugugaaau.......................................................  2     0    FF2
.........................................................uaucacagccagcuuuga............  4     0    FF2
.........................................................uaucacagccagcuuugau...........  1     0    FF2
.........................................................uaucacagccagcuuugaA...........  13    1    FF2
.........................................................uaucacagccagcuuugaug..........  15    0    FF2
.........................................................uaucacagccagcuuugaAg..........  395   1    FF2
.........................................................uaucacagccagcuuugauAa.........  1     1    FF2
.........................................................uaucacagccagcuuugauga.........  35    0    FF2
.........................................................uaucacagccagcuuugaAga.........  328   1    FF2
.........................................................uaucacagccagcuuugaAgag........  47    1    FF2
.........................................................uaucacagccagcuuugaugag........  22    0    FF2
.........................................................uaucacagccagcuuugaugaA........  1     1    FF2
.........................................................uauUacagccagcuuugaugag........  1     1    FF2
.........................................................uaucacagccagcuuugaAgagc.......  17    1    FF2
.........................................................uaucacagccagcuuugaugagc.......  19    0    FF2
.........................................................uaucacagccagcuuugaugagcu......  61    0    FF2
.........................................................uaucacagccagcuuugaAgagcu......  1     1    FF2
.........................................................uaucacagccagcuuugaugagcuC.....  1     1    FF2
..........................................................aucacagccagcuuugaAg..........  1     1    FF2
...........................................................ucacagccagcuuugaugag........  21    0    FF2
...........................................................ucacagccagcuuugaugagc.......  42    0    FF2
...........................................................ucacagccagcGuugaugagc.......  1     1    FF2
...........................................................ucacagccagcuuugaugagA.......  1     1    FF2
...........................................................ucacagccagcuuugaugagcG......  2     1    FF2

...........cucucaaaguggcugugaa.........................................................  1     0    OV2
...........cucucaaaguggcugugaaa........................................................  6     0    OV2
...........cucucaaaguggcugugaaaA.......................................................  1     1    OV2
...........cucucaaaguggcugugaaau.......................................................  1     0    OV2
...........cucucaaaguggcugugaaaug......................................................  53    0    OV2
............ucucaaaguggcugugaaa........................................................  1     0    OV2
............ucucaaaguggcugugaaau.......................................................  2     0    OV2
............ucucaaaguggcugugaaaug......................................................  8     0    OV2
.............cucaaaguggcugugaaa........................................................  1     0    OV2
.............cucaaaguggcugugaaaug......................................................  8     0    OV2
.........................................................uaucacagccagcuuuga............  10    0    OV2
.........................................................uaucacagccagcuuugaA...........  19    1    OV2
.........................................................uaucacagccagcuuugau...........  1     0    OV2
.........................................................uaucacagccagUuuugaug..........  1     1    OV2
.........................................................uaucacagccagcuuugaAg..........  784   1    OV2
.........................................................uaucacagccagcuuugauA..........  2     1    OV2
.........................................................uaucacagccagcuuugaug..........  57    0    OV2
.........................................................uaucacagccUgcuuugauga.........  1     1    OV2
.........................................................uaucacaAccagcuuugauga.........  2     1    OV2
.........................................................uaucacagccagcuuugauga.........  67    0    OV2
.........................................................uaucacagccagcuuugaAga.........  1641  1    OV2
.........................................................uaucacagccagcuuugaugag........  42    0    OV2
.........................................................uaucacagccagcuuugaAgag........  99    1    OV2
.........................................................uaucacagccagcuuugaugagc.......  35    0    OV2
.........................................................uaucacagccagcuuugaAgagc.......  23    1    OV2
.........................................................uaucacagccagcuuugaugagA.......  4     1    OV2
.........................................................uaucacagccagcuuugaugagcu......  56    0    OV2
.........................................................uaucacagccagcuuugaugaUcu......  1     1    OV2
.........................................................uaucacagccagcuuugaAgagcu......  1     1    OV2
.........................................................uaucacagccagcuuugaugagcuU.....  3     1    OV2
..........................................................aucacagccagcuuugaugagc.......  2     0    OV2
...........................................................ucacagccagcuuugaug..........  1     0    OV2
...........................................................ucacagccagcuuugaAga.........  1     1    OV2
...........................................................ucacagccagcuuugauga.........  1     0    OV2
...........................................................ucacagccagcuuugaugag........  59    0    OV2
...........................................................ucacagccagcuuugaugagA.......  2     1    OV2
...........................................................ucacagccagcuuugauAagc.......  1     1    OV2
```

```
           aga-miR-2
                                                         aga-miR-2*
gcauguccugucucucaaaguggcugugaaauggugcacuuucgauccgauaucucauaucacagccagcuuugaugagcuaggccg

...........................................................ucacagccagcuuugaugagc.......    169        0          OV2
...........................................................ucacagccagcuuugaugagcG......    2          1          OV2
...........................................................ucacagccagcuuugaugagcu......    2          0          OV2
.............................................................acagccagcuuugaAga.........    2          1          OV2
.............................................................acagccagcuuugaugagc.......    1          0          OV2

...........cucucaaaguggcuguAaaa........................................................    1          1          MF2
...........cucucaaaguggcugugaaa........................................................    60         0          MF2
...........cucucaaaguggcugugaaau.......................................................    69         0          MF2
...........cucucaaaguggcugugaaauA......................................................    1          1          MF2
...........cucucaaagGggcugugaaaug......................................................    3          1          MF2
...........cucucUaaguggcugugaaaug......................................................    1          1          MF2
...........cucucaaaguggcugugaaaug......................................................    1195       0          MF2
...........cucuUaaaguggcugugaaaug......................................................    1          1          MF2
...........cucucaaagCggcugugaaaug......................................................    1          1          MF2
...........cucucaaaguggcugugaaauU......................................................    14         1          MF2
...........cuGucaaaguggcugugaaaug......................................................    1          1          MF2
...........cucCcaaaguggcugugaaaug......................................................    2          1          MF2
...........cucucaaUguggcugugaaaug......................................................    1          1          MF2
...........cucucaaagugCcugugaaaug......................................................    2          1          MF2
...........cucucaaaguggcugugaaauC......................................................    1          1          MF2
...........cucucaaaguggcugugaaaugU.....................................................    9          1          MF2
...........cucucaaaguggcugugaaaugA.....................................................    8          1          MF2
...........cucucaaaguggcugugaaaugC.....................................................    3          1          MF2
...........cucucaaaguggcugugaaaugUu....................................................    6          1          MF2
...........cucucaaaguggcugugaaaugAu....................................................    1          1          MF2
............ucucaaaguggcuguga..........................................................    1          0          MF2
............ucucaaaguggcugugaaau.......................................................    4          0          MF2
............ucCcaaaguggcugugaaaug......................................................    1          1          MF2
............ucucaaaguggcugugaaaug......................................................    63         0          MF2
............ucucaaaguggcugugaaaugU.....................................................    1          1          MF2
.............cucaaaguggcugugaaaug......................................................    17         0          MF2
.............cucaaaguggcugugaaaugA.....................................................    1          1          MF2
..............ucaaaguggcugugaaaug......................................................    3          0          MF2
...............caaaguggcugugaaaug......................................................    3          0          MF2
...............caaaguggcugugaaaugCu....................................................    1          1          MF2
........................................................auaucacagccagcuuugauga.........    1          0          MF2
........................................................Cuaucacagccagcuuugaugagcu......    1          1          MF2
........................................................auaucacagccagcuuugaAgagcu......    1          1          MF2
.........................................................uaucacagccagcuuug.............    1          0          MF2
.........................................................uaucacagccagcuuuga............    34         0          MF2
.........................................................uaucacagccagcuuugaA...........    35         1          MF2
.........................................................uaucacagccagcuuugaC...........    1          1          MF2
.........................................................uaucacagccagcuuugau...........    11         0          MF2
.........................................................uaucacagccagcuuugauA..........    1          1          MF2
.........................................................uaucacagccagcuuugaug..........    111        0          MF2
.........................................................uaucacagccagcuuugaAg..........    1894       1          MF2
.........................................................uaucacagccagcuuugauAa.........    1          1          MF2
.........................................................uaucacagccagcuuugGuga.........    1          1          MF2
.........................................................uaucacagccagcuuugauga.........    207        0          MF2
.........................................................uaucacagccagcuuugaCga.........    1          1          MF2
.........................................................uaucacagccagcuuugaAga.........    1683       1          MF2
.........................................................uaucacagccagcuuugaAgag........    329        1          MF2
.........................................................uaucacagccagcuuugaugGg........    1          1          MF2
.........................................................uaucacagccagcuuugaugag........    127        0          MF2
.........................................................uaucacagccagcuuugaugaA........    1          1          MF2
.........................................................uaucacagccagcuuugaugaU........    1          1          MF2
.........................................................uaucacagccagcuuugaugagc.......    150        0          MF2
.........................................................uaucacagccagcuuugaugagU.......    1          1          MF2
.........................................................uaucacagccagcuuugaugagA.......    8          1          MF2
.........................................................uauAacagccagcuuugaugagc.......    1          1          MF2
.........................................................uaucaUagccagcuuugaugagc.......    1          1          MF2
.........................................................uUucacagccagcuuugaugagc.......    1          1          MF2
.........................................................uaucacagccagcuuGgaugagc.......    1          1          MF2
.........................................................uaucacagccagcuuugaAgagc.......    141        1          MF2
.........................................................uaucacagccagcuuugaAgagcu......    13         1          MF2
.........................................................uaucacagccGgcuuugaugagcu......    1          1          MF2
.........................................................uaucacagccagcuuugaugaCcu......    1          1          MF2
.........................................................uaucacagccagcuuugaugagcu......    490        0          MF2
```

```
           aga-miR-2
                                                         aga-miR-2*
gcauguccugucucucaaaguggcugugaaauggugcacuuucgauccgauaucucauaucacagccagcuuugaugagcuaggccg

.........................................................uaucacagccagcuuugaugagcG......    3     1     MF2
.........................................................uaucacagccagcuuugaugagcuC.....    6     1     MF2
.........................................................uaucacagccagcuuugaugagcuU.....    13    1     MF2
..........................................................aucacagccagcuuugaAga.........    1     1     MF2
..........................................................aucacagccagcuuugaugagc.......    3     0     MF2
...........................................................ucacagccagcuuugaug..........    1     0     MF2
...........................................................ucacagccagcuuugaAg..........    1     1     MF2
...........................................................ucacagccagcuuugauga.........    2     0     MF2
...........................................................ucacagccagcuuugaugag........    108   0     MF2
...........................................................ucacagccagcuuugauAagc.......    1     1     MF2
...........................................................ucacagccagcuuugaugagc.......    244   0     MF2
...........................................................ucacagccagcuuugaugagA.......    1     1     MF2
...........................................................ucacagccagcuuugaugagcG......    3     1     MF2
...........................................................ucacagccagcuuugaugagcu......    1     0     MF2
............................................................cacagccagcuuugaugagc.......    1     0     MF2
............................................................Uacagccagcuuugaugagc.......    1     1     MF2

...........cucucaaaguggcugugaa.........................................................    1     0     FW2
...........cucucaaaguggcugugaaa........................................................    3     0     FW2
...........cucucaaaguggcugugaaau.......................................................    6     0     FW2
...........cucucaaaguggcugugaaaug......................................................    93    0     FW2
...........cucucaaagugAcugugaaaug......................................................    1     1     FW2
...........cucucaaaguggcugugaaauA......................................................    1     1     FW2
...........cucucaaaguggcugugaaauggugcacuu..............................................    1     0     FW2
............ucucaaaguggcugugaaaug......................................................    1     0     FW2
.............cucaaaguggcugugaaa........................................................    1     0     FW2
.............cucaaaguggcugugaaau.......................................................    2     0     FW2
.............cucaaaguggcugugaaaug......................................................    1     0     FW2
.........................................................uaucacagccagcuuuga............    4     0     FW2
.........................................................uaucacagccagcuuugau...........    2     0     FW2
.........................................................uaucacagccagcuuugaA...........    2     1     FW2
.........................................................uaucacagccagcuuugaug..........    16    0     FW2
.........................................................uaucacagccagcuuugaAg..........    167   1     FW2
.........................................................uaucacagccagcuuugauga.........    14    0     FW2
.........................................................uaucacagccagcuuugaAga.........    216   1     FW2
.........................................................uaucaUagccagcuuugauga.........    1     1     FW2
.........................................................uaucacagccagcuuugaugag........    23    0     FW2
.........................................................uaucacagccagcuuugaAgag........    148   1     FW2
.........................................................uaucacagccagcuuugaugagc.......    67    0     FW2
.........................................................uaucacagccagcuuugauAagc.......    1     1     FW2
.........................................................uaucacagccagcuuugaAgagc.......    88    1     FW2
.........................................................uaucacagccagcuCugaugagcu......    1     1     FW2
.........................................................uaucacagccagcuuugaugagcu......    366   0     FW2
.........................................................uaucacagccaUcuuugaugagcu......    1     1     FW2
.........................................................uaucacagccagcuuugaugagcG......    3     1     FW2
.........................................................uaucacagccagcuuugaAgagcu......    1     1     FW2
.........................................................uauGacagccagcuuugaugagcu......    1     1     FW2
.........................................................uaucacaAccagcuuugaugagcu......    1     1     FW2
.........................................................uaucacGgccagcuuugaugagcu......    1     1     FW2
.........................................................uaucacagccagcuuugaugagcuC.....    2     1     FW2
.........................................................uaucacagccagcuuugaugagcuU.....    5     1     FW2
..........................................................aucacagccagcuuugaAga.........    1     1     FW2
...........................................................ucacagccagcuuugaug..........    1     0     FW2
...........................................................ucacagccagcuuugauga.........    5     0     FW2
...........................................................ucacagccagcuuugaugag........    13    0     FW2
...........................................................ucacagccagcuuugaugagc.......    69    0     FW2
...........................................................ucacagccagcuuugaugagcG......    1     1     FW2
............................................................cacagccagcuuugauga.........    1     0     FW2
............................................................cacagccagcuuugaAgag........    1     1     FW2
............................................................cacagccagcuuugaugagcu......    1     0     FW2

...........cucucaaaguggcugugaa.........................................................    1     0     OV1
...........cucucaaaguggcugugaaa........................................................    16    0     OV1
...........cucucaaaguggcugugaaau.......................................................    11    0     OV1
...........cucucaaaguggcugugUaaug......................................................    1     1     OV1
...........cucucaaaguggcugugaaauU......................................................    2     1     OV1
...........cucucaaaguggcugugaaaug......................................................    67    0     OV1
...........cucucaaaguggcugugaaaugU.....................................................    1     1     OV1
...........cucucaaaguggcugugaaaugA.....................................................    1     1     OV1
```

```
           aga-miR-2
                                                         aga-miR-2*
gcauguccugucucucaaaguggcugugaaauggugcacuuucgauccgauaucucauaucacagccagcuuugaugagcuaggccg

............ucucaaaguggcugugaaa........................................................   3      0    OV1
............ucucaaaguggcugugaaau.......................................................   1      0    OV1
............ucucaaaguggcugugaaaug......................................................   24     0    OV1
............ucucaaaguggcugugaaaugA.....................................................   1      1    OV1
.............cucaaaguggcugugaaa........................................................   1      0    OV1
.............cucaaaguggcugugaaau.......................................................   1      0    OV1
.............cCcaaaguggcugugaaaug......................................................   1      1    OV1
.............cucaaaguggcugugaaaug......................................................   12     0    OV1
...............caaaguggcugugaaaug......................................................   7      0    OV1
................aaaguggcugugaaaug......................................................   2      0    OV1
.........................................................uaucacagccagcuuug.............   4      0    OV1
.........................................................uaucacagccagcuuuga............   25     0    OV1
.........................................................uaucacagccagcuuugaA...........   20     1    OV1
.........................................................uaucacagccagcuuugau...........   4      0    OV1
.........................................................uaucacagccagcuuugaG...........   1      1    OV1
.........................................................uaucacagccagcuuugaug..........   75     0    OV1
.........................................................uaucacagccagcuuugaAg..........   1277   1    OV1
.........................................................uaucacagccagcuuugaCg..........   1      1    OV1
.........................................................uaCcacagccagcuuugaug..........   1      1    OV1
.........................................................uaucacagccagcuuugaAga.........   2082   1    OV1
.........................................................uaucacagccagcuuugaugG.........   1      1    OV1
.........................................................uaucacagccagcuuugauga.........   80     0    OV1
.........................................................uaucacagccagcuuugaugU.........   1      1    OV1
.........................................................uaucacagccagcuuugaugaU........   1      1    OV1
.........................................................uaucacagccagcuuugaugag........   50     0    OV1
.........................................................uaucacagccagcuuugaugaA........   2      1    OV1
.........................................................uaucacagccagcuuugaAgag........   157    1    OV1
.........................................................uaucacagccagcuuugaugagA.......   10     1    OV1
.........................................................uaucacagccagcuuugaAgagc.......   13     1    OV1
.........................................................uaucacagccagcuuugaugagc.......   40     0    OV1
.........................................................uaucacagccagcuuugaugagcu......   45     0    OV1
.........................................................uaucacagccagcuuugaAgagcu......   1      1    OV1
.........................................................uaucacagUcagcuuugaugagcu......   1      1    OV1
.........................................................uauUacagccagcuuugaugagcu......   1      1    OV1
.........................................................uaucacagccagcuuugaugagcuC.....   2      1    OV1
.........................................................uaucacagccagcuuugaugagcuU.....   1      1    OV1
..........................................................aucacagccagcuuugaAgag........   1      1    OV1
...........................................................ucacagccagcuuugaA...........   1      1    OV1
...........................................................ucacagccagcuuugaug..........   3      0    OV1
...........................................................ucacagccagcuuugauga.........   7      0    OV1
...........................................................ucacagccagcuuugaugag........   104    0    OV1
...........................................................ucacagccagcuuugaugagc.......   247    0    OV1
...........................................................ucacagccagcAuugaugagc.......   1      1    OV1
...........................................................ucacagccagcuuugaugagA.......   7      1    OV1
...........................................................ucacagccagcuuugaugagcu......   1      0    OV1
.............................................................acagccagcuuugaAga.........   2      1    OV1
.............................................................acagccagcuuugaugagc.......   2      0    OV1

...........cucucaaaguggcugugaa.........................................................   1      0    FF1
...........cucucaaaguggcugugaaa........................................................   5      0    FF1
...........cucucaaaguggcugugaaau.......................................................   2      0    FF1
...........cucucaaaguCgcugugaaau.......................................................   1      1    FF1
...........cucucaaaguggcugugaaauA......................................................   1      1    FF1
...........cucucaaaguggcugugaaaug......................................................   63     0    FF1
............ucucaaaguggcugugaaa........................................................   1      0    FF1
............ucucaaaguggcugugaaau.......................................................   1      0    FF1
............ucucaaaguggcugugaaaug......................................................   4      0    FF1
.............cucaaaguggcugugaaaug......................................................   4      0    FF1
...............caaaguggcugugaaaug......................................................   1      0    FF1
.........................................................uaucacagccagcuuuga............   2      0    FF1
.........................................................uaucacagccagcuuugau...........   2      0    FF1
.........................................................uaucacagccagcuuugaA...........   1      1    FF1
.........................................................uaucacagccagcuuugaAg..........   210    1    FF1
.........................................................uaucacagccagcuuugaug..........   9      0    FF1
.........................................................uaucacagccagcuuugaAga.........   437    1    FF1
.........................................................uaucacagccagcuuugauga.........   12     0    FF1
.........................................................uaucacagccagcuuugaugaA........   1      1    FF1
.........................................................uaucacagccagcuuugaAgag........   87     1    FF1
.........................................................uaucacagccagcuuugaugag........   26     0    FF1
```

```
           aga-miR-2
                                                         aga-miR-2*
gcauguccugucucucaaaguggcugugaaauggugcacuuucgauccgauaucucauaucacagccagcuuugaugagcuaggccg

.........................................................uaucacagccagcuuugaAgagc.......    25     1     FF1
.........................................................uaucacagccagcuuugaugagA.......    2      1     FF1
.........................................................uaucacagccagcuuugaugagc.......    26     0     FF1
.........................................................uaucacagccagcuuugauAagc.......    1      1     FF1
.........................................................uaucacagccagcuuugaugagcG......    1      1     FF1
.........................................................uaucacagccagcuuugaAgagcu......    4      1     FF1
.........................................................uaucacagccagcuuugaugagcu......    105    0     FF1
.........................................................uaucacagccagcuuugaugagcuC.....    4      1     FF1
.........................................................uaucacagccagcuuugaugagcuU.....    2      1     FF1
...........................................................ucacagccagcuuugauga.........    4      0     FF1
...........................................................ucacagccagcuuugaugag........    23     0     FF1
...........................................................ucacagccagcuuugaugagA.......    1      1     FF1
...........................................................ucacagccagcCuugaugagc.......    1      1     FF1
...........................................................ucacagccagcuuugaugagc.......    59     0     FF1

..........Acucucaaaguggcugugaaaug......................................................    1      1     MF1
...........cucucaaaguggcugugaaa........................................................    9      0     MF1
...........cucucaaaguggcugugaaau.......................................................    5      0     MF1
...........cucucaaagGggcugugaaaug......................................................    1      1     MF1
...........cucucaaagugUcugugaaaug......................................................    1      1     MF1
...........cucucaaaguggcugugaaaug......................................................    114    0     MF1
...........cucucaaaguggcugugaaaugU.....................................................    2      1     MF1
............ucucaaaguggcugugaaa........................................................    2      0     MF1
............ucucaaaguggcugugaaau.......................................................    4      0     MF1
............ucucaaaguggcugugaaaug......................................................    6      0     MF1
.............cucaaaguggcugugaaaug......................................................    2      0     MF1
................aaaguggcugugaaaug......................................................    1      0     MF1
.................................gugcacuuucgauccgauaucuca..............................    1      0     MF1
.........................................................uaucacagccagcuuug.............    2      0     MF1
.........................................................uaucacagccagcuuuga............    27     0     MF1
.........................................................uaucGcagccagcuuugau...........    1      1     MF1
.........................................................uaucacagccagcuuugau...........    4      0     MF1
.........................................................uaucacagccagcuuugaA...........    20     1     MF1
.........................................................uaucacagccagcuuugaug..........    40     0     MF1
.........................................................uaucacagccaAcuuugaug..........    1      1     MF1
.........................................................uaucacagccagcuuugaAg..........    578    1     MF1
.........................................................uaucacagccagcuuuAauga.........    1      1     MF1
.........................................................uaucacagccagcuuugaAga.........    544    1     MF1
.........................................................uaucacagccagcuuugauga.........    64     0     MF1
.........................................................uaucacagccagcuuugaAgag........    91     1     MF1
.........................................................uaucacagccagcuuugaugag........    35     0     MF1
.........................................................uaucacagccagcuuugaugagc.......    24     0     MF1
.........................................................uaucacagccagcuuugaAgagc.......    22     1     MF1
.........................................................uaucacagccagcuuugaugaUcu......    1      1     MF1
.........................................................uaucacagccagcuuugaAgagcu......    3      1     MF1
.........................................................uaucacagccagcuuugaugagcu......    96     0     MF1
.........................................................uaucacagccagcuuugaugagcuC.....    1      1     MF1
.........................................................uaucacagccagcuuugaugagcuU.....    1      1     MF1
...........................................................ucacagccagcuuugaug..........    2      0     MF1
...........................................................ucacagccagcuuugauga.........    2      0     MF1
...........................................................ucacagccagcuuugaugag........    40     0     MF1
...........................................................ucacagccagcuuugaugagc.......    68     0     MF1
...........................................................ucacagccagcuuugaugagcC......    1      1     MF1
...........................................................ucacagccagcuuugaugagcG......    3      1     MF1

...........cucucaaaguggcugugaaa........................................................    2      0     BF2
...........cucucaaaguggcugugaaau.......................................................    2      0     BF2
...........cucucaaaguggcugugaaaug......................................................    30     0     BF2
............ucucaaaguggcugugaaa........................................................    1      0     BF2
............ucucaaaguggcugugaaaug......................................................    10     0     BF2
.............cucaaaguggcugugaaaug......................................................    5      0     BF2
..............ucaaaguggcugugaaaug......................................................    2      0     BF2
...............caaaguggcugugaaaug......................................................    5      0     BF2
.................aaguggcugugaaaugC.....................................................    1      1     BF2
.........................................................uaucacagccagcuuuga............    4      0     BF2
.........................................................uaucacagccagcuuugaA...........    6      1     BF2
.........................................................uaucacagccagcuuugaug..........    43     0     BF2
.........................................................uaucacagccagcuuugaAg..........    737    1     BF2
.........................................................uaucacagccagcuuugaGg..........    1      1     BF2
```

```
           aga-miR-2
                                                         aga-miR-2*
gcauguccugucucucaaaguggcugugaaauggugcacuuucgauccgauaucucauaucacagccagcuuugaugagcuaggccg

.........................................................uaucacagccagcuuugauga.........    53     0    BF2
.........................................................uaucacagccagcuuugaAga.........    1214   1    BF2
.........................................................uaucacagccagcuuugaugag........    64     0    BF2
.........................................................uaucacagccagcuuugaAgag........    220    1    BF2
.........................................................uaucacagccagcuuugaugagc.......    41     0    BF2
.........................................................uaucacagccagcuuugaAgagc.......    44     1    BF2
.........................................................uaucacagccagcuuugaugagA.......    13     1    BF2
.........................................................uaucacagUcagcuuugaugagcu......    1      1    BF2
.........................................................uaucacagccagcuuugaugagcG......    1      1    BF2
.........................................................uaucacagccagcuuugaugagcu......    213    0    BF2
.........................................................uaucacagccagcuuugaAgagcu......    1      1    BF2
.........................................................uaucacagccaAcuuugaugagcu......    1      1    BF2
.........................................................uaucacagccagcuuugaugagcuC.....    13     1    BF2
.........................................................uaucacagccagcuuugaugagcuU.....    1      1    BF2
.........................................................uaucacagccagcuuugaugagcua.....    2      0    BF2
..........................................................aucacagccagcuuugaugagc.......    1      0    BF2
...........................................................ucacagccagcuuugaug..........    2      0    BF2
...........................................................ucacagccagcuuugauga.........    4      0    BF2
...........................................................ucacagccagcuuugaugag........    48     0    BF2
...........................................................ucacagccagcuuugaugagA.......    3      1    BF2
...........................................................ucacagccagcuuugaugagc.......    63     0    BF2
...........................................................ucacagccagcuuugaugagcG......    1      1    BF2

...........cucucaaaguggcugugaa.........................................................    1      0    BF1
...........cucucaaaguggcugugaaa........................................................    4      0    BF1
...........cucucaaaguCgcugugaaau.......................................................    1      1    BF1
...........cucucaaaguggcugugaaau.......................................................    2      0    BF1
...........cucucaaaguggcugugaaauA......................................................    1      1    BF1
...........cucucaaaguggcugugaaaug......................................................    63     0    BF1
............ucucaaaguggcugugaaa........................................................    1      0    BF1
............ucucaaaguggcugugaaau.......................................................    1      0    BF1
............ucucaaaguggcugugaaaug......................................................    4      0    BF1
.............cucaaaguggcugugaaaug......................................................    4      0    BF1
...............caaaguggcugugaaaug......................................................    1      0    BF1
.........................................................uaucacagccagcuuuga............    2      0    BF1
.........................................................uaucacagccagcuuugaA...........    1      1    BF1
.........................................................uaucacagccagcuuugau...........    2      0    BF1
.........................................................uaucacagccagcuuugaAg..........    209    1    BF1
.........................................................uaucacagccagcuuugaug..........    9      0    BF1
.........................................................uaucacagccagcuuugaAga.........    437    1    BF1
.........................................................uaucacagccagcuuugauga.........    12     0    BF1
.........................................................uaucacagccagcuuugaugag........    26     0    BF1
.........................................................uaucacagccagcuuugaAgag........    87     1    BF1
.........................................................uaucacagccagcuuugaugaA........    1      1    BF1
.........................................................uaucacagccagcuuugauAagc.......    1      1    BF1
.........................................................uaucacagccagcuuugaAgagc.......    25     1    BF1
.........................................................uaucacagccagcuuugaugagc.......    26     0    BF1
.........................................................uaucacagccagcuuugaugagA.......    2      1    BF1
.........................................................uaucacagccagcuuugaugagcu......    105    0    BF1
.........................................................uaucacagccagcuuugaugagcG......    1      1    BF1
.........................................................uaucacagccagcuuugaAgagcu......    4      1    BF1
.........................................................uaucacagccagcuuugaugagcuC.....    4      1    BF1
.........................................................uaucacagccagcuuugaugagcuU.....    2      1    BF1
...........................................................ucacagccagcuuugauga.........    4      0    BF1
...........................................................ucacagccagcuuugaugag........    23     0    BF1
...........................................................ucacagccagcuuugaugagc.......    59     0    BF1
...........................................................ucacagccagcCuugaugagc.......    1      1    BF1
...........................................................ucacagccagcuuugaugagA.......    1      1    BF1

...........cucucaaaguggcugugaa.........................................................    3      0    MW1
...........cucucaaaguggcugugaaa........................................................    3      0    MW1
...........cucucaaaguggcugugaaau.......................................................    7      0    MW1
...........cucucaaaguggcugugaaaug......................................................    128    0    MW1
...........cucuUaaaguggcugugaaaug......................................................    1      1    MW1
............ucucaaaguggcugugaaaug......................................................    7      0    MW1
.............cucaaaguggcugugaaaug......................................................    1      0    MW1
.........................................................uaucacagccagcuuug.............    2      0    MW1
.........................................................uaucacagccagcuuuga............    1      0    MW1
.........................................................uaucacagccagcuuugaA...........    4      1    MW1
```

```
          aga-miR-2
                                              aga-miR-2*
gcauguccugucucucaaaguggcugugaaauggugcacuuucgauccgauaucucauaucacagccagcuuugaugagcuaggccg

.........................................................uaucacagccagcuuugaug..........   22    0    MW1
.........................................................uaucacagccagcuuugaAg..........   233   1    MW1
.........................................................uaucacagccagcuuugauAa.........   1     1    MW1
.........................................................uaucacagccagcuuugaAga.........   248   1    MW1
.........................................................uaucacagccagcuuugauga.........   25    0    MW1
.........................................................uaucacagccagcuuugaAgag........   114   1    MW1
.........................................................uaucacagccagcuuugaugag........   31    0    MW1
.........................................................uaucacagccagcuuugaugagU.......   1     1    MW1
.........................................................uaucacagccagcuuugaAgagc.......   65    1    MW1
.........................................................uaucacagccagcuuugaugagc.......   39    0    MW1
.........................................................uaucacagccagcuuCgaugagcu......   1     1    MW1
.........................................................uaucacGgccagcuuugaugagcu......   1     1    MW1
.........................................................uaucacagccagcuuugaugagcG......   1     1    MW1
.........................................................uauUacagccagcuuugaugagcu......   1     1    MW1
.........................................................uaucacagccagcuuugaAgagcu......   2     1    MW1
.........................................................uaucacagccagcuuugaCgagcu......   1     1    MW1
.........................................................uaucacagccagcuuugaugagcu......   344   0    MW1
.........................................................uaucacagccagcuuugaugagcC......   1     1    MW1
.........................................................uaucacagUcagcuuugaugagcu......   1     1    MW1
.........................................................uaucacagccagcuuugaugagcuU.....   5     1    MW1
.........................................................uaucacagccagcuuugaugagcuC.....   8     1    MW1
...........................................................ucacagccagcuuugaug..........   3     0    MW1
...........................................................ucacagccagcuuugauga.........   2     0    MW1
...........................................................ucacagccagcuuugaugag........   11    0    MW1
...........................................................ucacagccagcuuugaugagc.......   39    0    MW1
...........................................................ucacagccagcuuugaugagcG......   1     1    MW1
............................................................cacagccagcuuugaug..........   2     0    MW1
............................................................cacagccagcuuugauga.........   2     0    MW1
.............................................................acagccagcuuugaAgag........   1     1    MW1

...........cucucaaaguggcugugaa.........................................................   1     0    FW1
...........cucucaaaguggcugugaaa........................................................   4     0    FW1
...........cucucaaaguggcugugaaau.......................................................   2     0    FW1
...........cucucaaaguggcugugaaaA.......................................................   1     1    FW1
...........cucucaaaguggcugugaaaug......................................................   81    0    FW1
.............cucaaaguggcugugaaau.......................................................   1     0    FW1
.................................gugcacuuucgauccgauaucuca..............................   1     0    FW1
........................................................auaucacagccagcuuugaAga.........   1     1    FW1
.........................................................uaucacagccagcuuugaA...........   1     1    FW1
.........................................................uaucacagccagcuuugaug..........   9     0    FW1
.........................................................uaucacagccagcuuugaAg..........   79    1    FW1
.........................................................uaucacagccagcuuugaAga.........   119   1    FW1
.........................................................uaucacagccagcuuugauga.........   11    0    FW1
.........................................................uaucacagccagcuuugaAgag........   51    1    FW1
.........................................................uaucacagccagcuuugaugag........   5     0    FW1
.........................................................uaucacagccagcuuugaugagA.......   3     1    FW1
.........................................................uaucacagccagcuuugaAgagc.......   25    1    FW1
.........................................................uaucacagccagcuuugaugagc.......   29    0    FW1
.........................................................uaucacagccagcuuugauAagcu......   1     1    FW1
.........................................................uaucacagccagcuuugaAgagcu......   2     1    FW1
.........................................................uaucacagccagcuuugaugagcu......   223   0    FW1
.........................................................uaucGcagccagcuuugaugagcu......   1     1    FW1
.........................................................uauUacagccagcuuugaugagcu......   1     1    FW1
.........................................................uaucacagccagcuuugaugagcG......   4     1    FW1
.........................................................uaucacagccagcuuugaugagcuU.....   2     1    FW1
.........................................................uaucacagccagcuuugaugagcuC.....   3     1    FW1
...........................................................ucacagccagcuuugaug..........   4     0    FW1
...........................................................ucacagccagcuuugaugag........   7     0    FW1
...........................................................ucaUagccagcuuugaugagc.......   1     1    FW1
...........................................................ucacagccagcuuugaugagc.......   30    0    FW1

...........cucucaaaguggcugugaaau.......................................................   4     0    MW2
...........cucucaaaguggcugugaaaug......................................................   38    0    MW2
.........................................................uaucacagccagcuuuga............   1     0    MW2
.........................................................uaucacagccagcuuugaA...........   2     1    MW2
.........................................................uaucacagccagcuuugaug..........   3     0    MW2
.........................................................uaucacagccagcuuugaAg..........   47    1    MW2
.........................................................uaucacagccagcuuugauga.........   3     0    MW2
.........................................................uaucacagccagcuuugaAga.........   65    1    MW2
```

```
           aga-miR-2
                                                         aga-miR-2*
gcauguccugucucucaaaguggcugugaaauggugcacuuucgauccgauaucucauaucacagccagcuuugaugagcuaggccg

.........................................................uaucacagccagcuuugaugag........    7     0     MW2
.........................................................uaucacagccagcuuugaAgag........    36    1     MW2
.........................................................uaucacagccagcuuugaAgagc.......    22    1     MW2
.........................................................uaucacagccagcuuugaugagc.......    20    0     MW2
.........................................................uaucGcagccagcuuugaugagcu......    1     1     MW2
.........................................................uaucacagccagcCuugaugagcu......    1     1     MW2
.........................................................uaucacagccagcuuugaCgagcu......    1     1     MW2
.........................................................uaucacagccaAcuuugaugagcu......    1     1     MW2
.........................................................uaucacagccagcuuugaugagcu......    84    0     MW2
.........................................................uaucacagccagcuuugaugagcuU.....    1     1     MW2
.........................................................uaucacagccagcuuugaugagcuC.....    3     1     MW2
...........................................................ucacagccagcuuCgaugag........    1     1     MW2
...........................................................ucacagccagcuuugaugag........    4     0     MW2
...........................................................ucacagccagcuuugaugaAc.......    1     1     MW2
...........................................................ucacagccagcuuugaugagc.......    31    0     MW2
............................................................cacagccagcuuugauga.........    2     0     MW2

...........cucucaaaguggcugugaa.........................................................    2     0     TE2
...........cucucaaaguggcugugaaa........................................................    3     0     TE2
...........cucucaaaguggcugugaaau.......................................................    3     0     TE2
...........cucucaaaguggcugugaaauA......................................................    2     1     TE2
...........cucucaaaguggcugugaaaug......................................................    13    0     TE2
...........cucucaaaguggcugugaaaugA.....................................................    1     1     TE2
............ucucaaaguggcugugaaau.......................................................    1     0     TE2
............ucucaaaguggcugugaaaug......................................................    2     0     TE2
............ucucaaaguggcugugaaaugA.....................................................    1     1     TE2
.............cucaaaguggcugugaaa........................................................    1     0     TE2
...............caaaguggcugugaaaug......................................................    1     0     TE2
.........................................................uaucacagccagcuuug.............    2     0     TE2
.........................................................uaucacagccagcuuuga............    13    0     TE2
.........................................................uaucacagccagcuuugau...........    1     0     TE2
.........................................................uaucacagccagcuuugaA...........    12    1     TE2
.........................................................uaucacagccagcuuugaAg..........    212   1     TE2
.........................................................uaucacagccagcuuugaug..........    18    0     TE2
.........................................................uaucacagccagcuuugaAga.........    477   1     TE2
.........................................................uaucacagccagcuuugauga.........    23    0     TE2
.........................................................uaucacagccagcuuugaAgag........    323   1     TE2
.........................................................uaucacagccagcuuugaugag........    41    0     TE2
.........................................................uaucacagccagcuCugaugag........    1     1     TE2
.........................................................uaucacagccCgcuuugaugagc.......    1     1     TE2
.........................................................uaucacagccagcuuugaAgagc.......    105   1     TE2
.........................................................uaucacagccagcuuugaugagc.......    124   0     TE2
.........................................................uaucacagccagcuuugaAgagcu......    8     1     TE2
.........................................................uaucacagccagcuuugaugagcG......    4     1     TE2
.........................................................uaucacagccagcuuugaugagcu......    203   0     TE2
.........................................................uauAacagccagcuuugaugagcu......    1     1     TE2
.........................................................uaucacagccagcuuugaugagcuU.....    4     1     TE2
..........................................................aucacagccagcuuugaugagcuU.....    1     1     TE2
...........................................................ucacagccagcuuugaug..........    5     0     TE2
...........................................................ucacagccagcuuugauga.........    9     0     TE2
...........................................................ucacagccagcuuugaugU.........    1     1     TE2
...........................................................ucacagccagcuuugaugag........    48    0     TE2
...........................................................ucacagccagcuuugaugaAc.......    1     1     TE2
...........................................................ucacagccagcuuuAaugagc.......    2     1     TE2
...........................................................ucacagccagcuuugaugagA.......    1     1     TE2
...........................................................ucacagccagcuuugaugagU.......    1     1     TE2
...........................................................ucacagccagcuuugaugagc.......    141   0     TE2
...........................................................ucacagccagcuuugaugagcG......    1     1     TE2
.............................................................acagccagcuuugaAgag........    1     1     TE2
```

miRBase precursor	: aga-mir-2-2
Total read count	: 29491
aga-miR-2 read count	: 29324
aga-miR-2* read count	: 157
remaining reads	: 10

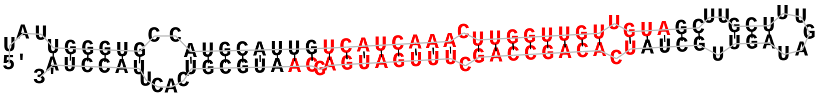

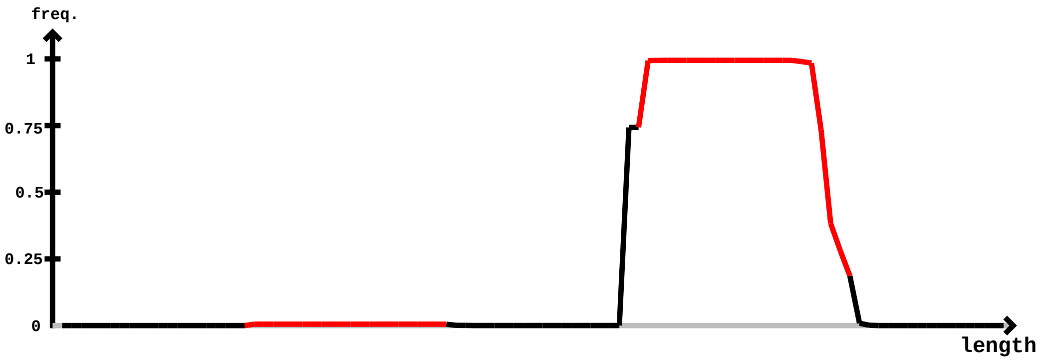


```
                                                                  aga-miR-2
                      aga-miR-2*
5'- uauugggugccaugcauugucaucaaacuugguuguuguagcuugcuuugauaguugcuaucacagccagcuuugaugagcaaugcgucacuuaccua   -3'    exp
    ...((((((..((((((((((((((((.((((((((.(((((..(((.....))).))))).)))))))).)))))))).))))))))....))))))    reads      mm         sample
    ...................ucaucaaacuugguuguugu............................................................    1          0          OV2
    ...................ucaucaaacuugguuguugua...........................................................    6          0          OV2
    ...................ucaucaaacuugguuguuguagc.........................................................    3          0          OV2
    ...................ucaucaaacuugguuguuguagcu........................................................    1          0          OV2
    ..........................................................cuaucacagccagcuuugaAg....................    2          1          OV2
    ..........................................................cuaucacagccagcuuugaAga...................    1          1          OV2
    ...........................................................uaucacagccagcuuuga......................    10         0          OV2
    ...........................................................uaucacagccagcuuugaA.....................    19         1          OV2
    ...........................................................uaucacagccagcuuugau.....................    1          0          OV2
    ...........................................................uaucacagccagUuuugaug....................    1          1          OV2
    ...........................................................uaucacagccagcuuugaug....................    57         0          OV2
    ...........................................................uaucacagccagcuuugauA....................    2          1          OV2
    ...........................................................uaucacagccagcuuugaAg....................    784        1          OV2
    ...........................................................uaucacagccUgcuuugauga...................    1          1          OV2
    ...........................................................uaucacagccagcuuugaAga...................    1641       1          OV2
    ...........................................................uaucacagccagcuuugauga...................    67         0          OV2
    ...........................................................uaucacaAccagcuuugauga...................    2          1          OV2
    ...........................................................uaucacagccagcuuugaugag..................    42         0          OV2
    ...........................................................uaucacagccagcuuugaAgag..................    99         1          OV2
    ...........................................................uaucacagccagcuuugaAgagc.................    23         1          OV2
    ...........................................................uaucacagccagcuuugaugagA.................    4          1          OV2
    ...........................................................uaucacagccagcuuugaugagc.................    35         0          OV2
    ...........................................................uaucacagccagcuuugaugagca................    1          0          OV2
    ............................................................aucacagccagcuuugaugagc.................    2          0          OV2
    .............................................................ucacagccagcuuugaug....................    1          0          OV2
    .............................................................ucacagccagcuuugaAga...................    1          1          OV2
    .............................................................ucacagccagcuuugauga...................    1          0          OV2
    .............................................................ucacagccagcuuugaugag..................    59         0          OV2
    .............................................................ucacagccagcuuugaugagc.................    169        0          OV2
    .............................................................ucacagccagcuuugaugagA.................    2          1          OV2
    .............................................................ucacagccagcuuugauAagc.................    1          1          OV2
    .............................................................ucacagccagcuuugaGgagca................    1          1          OV2
    .............................................................ucacagccaAcuuugaugagca................    1          1          OV2
    .............................................................ucacagccagcuuugaugagca................    427        0          OV2
```

```
                                                            aga-miR-2
                   aga-miR-2*
uauugggugccaugcauugucaucaaacuugguuguuguagcuugcuuugauaguugcuaucacagccagcuuugaugagcaaugcgucacuuaccua

............................................................ucacagccagcuuugaugagcG................    2          1          OV2
............................................................ucacagccagcuCugaugagca................    1          1          OV2
............................................................ucacagccagcuuugaugagcaa...............    4          0          OV2
............................................................ucacagccagcuuugaugagcaU...............    17         1          OV2
............................................................ucacagccagcuuugaugagcaC...............    6          1          OV2
............................................................ucacagccagcuuugaugagcaUu..............    3          1          OV2
............................................................ucacagccagcuuugaugagcaCu..............    1          1          OV2
..............................................................acagccagcuuugaAga...................    2          1          OV2
..............................................................acagccagcuuugaugagc.................    1          0          OV2

...................ucaucaaacuugguuguugu...........................................................    1          0          FF2
...................ucaucaaacuugguuguugua..........................................................    4          0          FF2
.........................................................cuaucacagccagcuuugaAg....................    1          1          FF2
..........................................................uaucacagccagcuuuga......................    4          0          FF2
..........................................................uaucacagccagcuuugau.....................    1          0          FF2
..........................................................uaucacagccagcuuugaA.....................    13         1          FF2
..........................................................uaucacagccagcuuugaug....................    15         0          FF2
..........................................................uaucacagccagcuuugaAg....................    395        1          FF2
..........................................................uaucacagccagcuuugauAa...................    1          1          FF2
..........................................................uaucacagccagcuuugaAga...................    328        1          FF2
..........................................................uaucacagccagcuuugauga...................    35         0          FF2
..........................................................uauUacagccagcuuugaugag..................    1          1          FF2
..........................................................uaucacagccagcuuugaAgag..................    47         1          FF2
..........................................................uaucacagccagcuuugaugag..................    22         0          FF2
..........................................................uaucacagccagcuuugaugaA..................    1          1          FF2
..........................................................uaucacagccagcuuugaAgagc.................    17         1          FF2
..........................................................uaucacagccagcuuugaugagc.................    19         0          FF2
...........................................................aucacagccagcuuugaAg....................    1          1          FF2
............................................................ucacagccagcuuugaugag..................    21         0          FF2
............................................................ucacagccagcuuugaugagA.................    1          1          FF2
............................................................ucacagccagcuuugaugagc.................    42         0          FF2
............................................................ucacagccagcGuugaugagc.................    1          1          FF2
............................................................ucacagccagcuuugaugagca................    230        0          FF2
............................................................ucacagccagcuuugaugagcG................    2          1          FF2
............................................................ucacagccagcuuugaugagcaC...............    2          1          FF2
............................................................ucacagccagcuuugaugagcaa...............    2          0          FF2
............................................................ucacagccagcuuugaugagcaU...............    6          1          FF2

...................ucaucaaacuugguuguug............................................................    1          0          TE1
...................ucaucaaacuugguuguugua..........................................................    1          0          TE1
..........................................................uaucacagccagcuuuga......................    4          0          TE1
..........................................................uaucacagccagcuuugaA.....................    1          1          TE1
..........................................................uaucacagccagcuuugaAg....................    77         1          TE1
..........................................................uaucacagccagcuuugaug....................    7          0          TE1
..........................................................uaucacagccagcuuugauga...................    10         0          TE1
..........................................................uaucacagccagcuuugaAga...................    196        1          TE1
..........................................................uaucacagccagcuuugaAgag..................    132        1          TE1
..........................................................uaucacagccagcuuugaugag..................    15         0          TE1
..........................................................uaucacagccagcuuugaAgagc.................    76         1          TE1
..........................................................uaucacagccagcuuugaugagc.................    45         0          TE1
..........................................................uaucacagccagcuuugaugagcG................    1          1          TE1
..........................................................uaucacagccagcuuugaAgagca................    2          1          TE1
...........................................................aucacagccagcuuugaAga...................    1          1          TE1
............................................................ucacagccagcuuugaug....................    1          0          TE1
............................................................ucacagccagcuuugauga...................    1          0          TE1
............................................................ucacagccagcuuugaugag..................    29         0          TE1
............................................................ucacagccagcuuugaugagc.................    72         0          TE1
............................................................ucacagccagcuuugaugagca................    142        0          TE1
............................................................ucacagccagcuuugauUagca................    1          1          TE1
............................................................ucacagccagUuuugaugagca................    1          1          TE1
............................................................ucacagccagcuuugaugagcaC...............    1          1          TE1
............................................................ucacagccagcuuugaugagcaU...............    2          1          TE1
............................................................ucacagccagcuuugaugagcaa...............    2          0          TE1

.................ugucaucaaacuugguuguugu...........................................................    1          0          MF2
...................ucaucaaacuugguuguugua..........................................................    33         0          MF2
...................uUaucaaacuugguuguugua..........................................................    1          1          MF2
...................ucaucaaacuugguuguuguaU.........................................................    2          1          MF2
...................ucaucaaacuugguuguuguaC.........................................................    1          1          MF2
```

```
                                                            aga-miR-2
                   aga-miR-2*
uauugggugccaugcauugucaucaaacuugguuguuguagcuugcuuugauaguugcuaucacagccagcuuugaugagcaaugcgucacuuaccua

...................ucaucaaacuugguuguuguagc........................................................    1      0    MF2
.........................................................cuaucacagccagcuuugaAg....................    1      1    MF2
.........................................................cuaucacagccagcuuugaAga...................    1      1    MF2
.........................................................cuaucacagccagcuuugaugagcU................    1      1    MF2
..........................................................uaucacagccagcuuug.......................    1      0    MF2
..........................................................uaucacagccagcuuuga......................    34     0    MF2
..........................................................uaucacagccagcuuugaC.....................    1      1    MF2
..........................................................uaucacagccagcuuugaA.....................    35     1    MF2
..........................................................uaucacagccagcuuugau.....................    11     0    MF2
..........................................................uaucacagccagcuuugaug....................    111    0    MF2
..........................................................uaucacagccagcuuugaAg....................    1894   1    MF2
..........................................................uaucacagccagcuuugauA....................    1      1    MF2
..........................................................uaucacagccagcuuugGuga...................    1      1    MF2
..........................................................uaucacagccagcuuugauAa...................    1      1    MF2
..........................................................uaucacagccagcuuugaCga...................    1      1    MF2
..........................................................uaucacagccagcuuugaAga...................    1683   1    MF2
..........................................................uaucacagccagcuuugauga...................    207    0    MF2
..........................................................uaucacagccagcuuugaugaU..................    1      1    MF2
..........................................................uaucacagccagcuuugaAgag..................    329    1    MF2
..........................................................uaucacagccagcuuugaugag..................    127    0    MF2
..........................................................uaucacagccagcuuugaugGg..................    1      1    MF2
..........................................................uaucacagccagcuuugaugaA..................    1      1    MF2
..........................................................uaucacagccagcuuugaugagc.................    150    0    MF2
..........................................................uaucacagccagcuuGgaugagc.................    1      1    MF2
..........................................................uUucacagccagcuuugaugagc.................    1      1    MF2
..........................................................uaucacagccagcuuugaugagU.................    1      1    MF2
..........................................................uauAacagccagcuuugaugagc.................    1      1    MF2
..........................................................uaucaUagccagcuuugaugagc.................    1      1    MF2
..........................................................uaucacagccagcuuugaugagA.................    8      1    MF2
..........................................................uaucacagccagcuuugaAgagc.................    141    1    MF2
..........................................................uaucacagccagcuuugaugagcG................    3      1    MF2
..........................................................uaucacagccagcuuugaugagAa................    1      1    MF2
..........................................................uaucacagccagcuuugaugagca................    6      0    MF2
..........................................................uaucacagccagcuuugaAgagca................    4      1    MF2
...........................................................aucacagccagcuuugaAga...................    1      1    MF2
...........................................................aucacagccagcuuugaugagc.................    3      0    MF2
...........................................................Cucacagccagcuuugaugagca................    1      1    MF2
............................................................ucacagccagcuuugaAg....................    1      1    MF2
............................................................ucacagccagcuuugaug....................    1      0    MF2
............................................................ucacagccagcuuugauga...................    2      0    MF2
............................................................ucacagccagcuuugaugag..................    108    0    MF2
............................................................ucacagccagcuuugaugagA.................    1      1    MF2
............................................................ucacagccagcuuugaugagc.................    244    0    MF2
............................................................ucacagccagcuuugauAagc.................    1      1    MF2
............................................................ucacagccagcGuugaugagca................    1      1    MF2
............................................................ucacagccagcuAugaugagca................    1      1    MF2
............................................................ucacagccagcCuugaugagca................    1      1    MF2
............................................................ucacagccagcuuuAaugagca................    2      1    MF2
............................................................ucacagccaUcuuugaugagca................    1      1    MF2
............................................................ucacagccagcuuCgaugagca................    1      1    MF2
............................................................ucaUagccagcuuugaugagca................    1      1    MF2
............................................................ucacagccagcuuugaugagca................    1334   0    MF2
............................................................ucacagccGgcuuugaugagca................    2      1    MF2
............................................................ucacagccagcAuugaugagca................    1      1    MF2
............................................................ucacagccagcuuugauAagca................    1      1    MF2
............................................................ucacagccagcuuugaugagcG................    3      1    MF2
............................................................ucacagccagcuuugaAgagca................    1      1    MF2
............................................................ucacagccagcuuugauCagca................    1      1    MF2
............................................................ucGcagccagcuuugaugagca................    1      1    MF2
............................................................ucacagcUagcuuugaugagca................    1      1    MF2
............................................................ucacagccagcuuugaugagcaC...............    18     1    MF2
............................................................ucacagccagcuuugaugagcaa...............    11     0    MF2
............................................................ucacagccagcuuugaugagcaU...............    36     1    MF2
............................................................ucacagccagcuuugaugagcaCu..............    8      1    MF2
............................................................ucacagccagcuuugaugagcaaA..............    5      1    MF2
............................................................ucacagccagcuuugaugagcaUu..............    6      1    MF2
.............................................................cacagccagcuuugaugagc.................    1      0    MF2
.............................................................Uacagccagcuuugaugagc.................    1      1    MF2
..............................................................acagccagcuuugaugagca................    1      0    MF2
```

```
                                                              aga-miR-2
                   aga-miR-2*
uauugggugccaugcauugucaucaaacuugguuguuguagcuugcuuugauaguugcuaucacagccagcuuugaugagcaaugcgucacuuaccua


...................ucaucaaacuugguuguug............................................................   1     0    FW2
...................ucaucaaacuugguuguugu...........................................................   1     0    FW2
...................ucaucaaacuugguuguugua..........................................................   14    0    FW2
...................ucaucaaacuugguuguuguaU.........................................................   1     1    FW2
.................................uguuguagcuugcuuugauaguugcuaucacagc...............................   1     0    FW2
..........................................................uaucacagccagcuuuga......................   4     0    FW2
..........................................................uaucacagccagcuuugaA.....................   2     1    FW2
..........................................................uaucacagccagcuuugau.....................   2     0    FW2
..........................................................uaucacagccagcuuugaug....................   16    0    FW2
..........................................................uaucacagccagcuuugaAg....................   167   1    FW2
..........................................................uaucacagccagcuuugauga...................   14    0    FW2
..........................................................uaucaUagccagcuuugauga...................   1     1    FW2
..........................................................uaucacagccagcuuugaAga...................   216   1    FW2
..........................................................uaucacagccagcuuugaugag..................   23    0    FW2
..........................................................uaucacagccagcuuugaAgag..................   148   1    FW2
..........................................................uaucacagccagcuuugaAgagc.................   88    1    FW2
..........................................................uaucacagccagcuuugauAagc.................   1     1    FW2
..........................................................uaucacagccagcuuugaugagc.................   67    0    FW2
..........................................................uaucacagccagcuuugaugagcG................   3     1    FW2
..........................................................uaucacagccagcuuugaugagca................   3     0    FW2
..........................................................uaucacagccagcuuugaAgagca................   1     1    FW2
...........................................................aucacagccagcuuugaAga...................   1     1    FW2
............................................................ucacagccagcuuugaug....................   1     0    FW2
............................................................ucacagccagcuuugauga...................   5     0    FW2
............................................................ucacagccagcuuugaugag..................   13    0    FW2
............................................................ucacagccagcuuugaugagc.................   69    0    FW2
............................................................ucacagccagcuuugaugagca................   329   0    FW2
............................................................ucacagccagcuuugaugCgca................   1     1    FW2
............................................................ucacagccGgcuuugaugagca................   1     1    FW2
............................................................ucacagccagcuuugaugagcG................   1     1    FW2
............................................................ucacagccagcuuugaugagcaa...............   3     0    FW2
............................................................ucacagccagcuuugaugagcaU...............   2     1    FW2
............................................................ucacagccagcuuugaugagcaC...............   4     1    FW2
............................................................ucacagccagcuuugaugagcaUu..............   3     1    FW2
.............................................................cacagccagcuuugauga...................   1     0    FW2
.............................................................cacagccagcuuugaAgag..................   1     1    FW2
.............................................................cacagccagcuuugaugagca................   2     0    FW2
..............................................................acagccagcuuugaugagca................   1     0    FW2

...................ucaucaaacuugguuguugua..........................................................   1     0    FF1
...................ucaucaaacuugguuguuguagc........................................................   3     0    FF1
..........................................................uaucacagccagcuuuga......................   2     0    FF1
..........................................................uaucacagccagcuuugaA.....................   1     1    FF1
..........................................................uaucacagccagcuuugau.....................   2     0    FF1
..........................................................uaucacagccagcuuugaug....................   9     0    FF1
..........................................................uaucacagccagcuuugaAg....................   210   1    FF1
..........................................................uaucacagccagcuuugaAga...................   437   1    FF1
..........................................................uaucacagccagcuuugauga...................   12    0    FF1
..........................................................uaucacagccagcuuugaAgag..................   87    1    FF1
..........................................................uaucacagccagcuuugaugaA..................   1     1    FF1
..........................................................uaucacagccagcuuugaugag..................   26    0    FF1
..........................................................uaucacagccagcuuugauAagc.................   1     1    FF1
..........................................................uaucacagccagcuuugaugagA.................   2     1    FF1
..........................................................uaucacagccagcuuugaugagc.................   26    0    FF1
..........................................................uaucacagccagcuuugaAgagc.................   25    1    FF1
..........................................................uaucacagccagcuuugaugagcG................   1     1    FF1
............................................................ucacagccagcuuugauga...................   4     0    FF1
............................................................ucacagccagcuuugaugag..................   23    0    FF1
............................................................ucacagccagcuuugaugagA.................   1     1    FF1
............................................................ucacagccagcCuugaugagc.................   1     1    FF1
............................................................ucacagccagcuuugaugagc.................   59    0    FF1
............................................................ucacagccagcuuugUugagca................   1     1    FF1
............................................................ucacagccagcuuugaugagAa................   1     1    FF1
............................................................uUacagccagcuuugaugagca................   1     1    FF1
............................................................ucacagccagcuuugaugagca................   172   0    FF1
............................................................ucacagccagcAuugaugagca................   1     1    FF1
............................................................ucacagccagcuuugaugagcaU...............   3     1    FF1
............................................................ucacagccagcuuugaugagcaC...............   1     1    FF1
```

aga-miR-2

aga-miR-2*

```
uauugggugccaugcauugucaucaaacuugguuguuguagcuugcuuugauaguugcuaucacagccagcuuugaugagcaaugcgucacuuaccua

............................................................ucacagccagcuuugaugagcaG...............  1     1  FF1
............................................................ucacagccagcuuugaugagcaa...............  4     0  FF1
............................................................ucacagccagcuuugaugagcaUu..............  1     1  FF1

...................ucaucaaacuugguuguugu...........................................................  1     0  OV1
...................ucaucaaacuugguuguugua..........................................................  7     0  OV1
...................ucaucaaacuugguuguuguagc........................................................  2     0  OV1
.........................................................cuaucacagccagcuuugaAga...................  1     1  OV1
..........................................................uaucacagccagcuuug.......................  4     0  OV1
..........................................................uaucacagccagcuuuga......................  25    0  OV1
..........................................................uaucacagccagcuuugau.....................  4     0  OV1
..........................................................uaucacagccagcuuugaG.....................  1     1  OV1
..........................................................uaucacagccagcuuugaA.....................  20    1  OV1
..........................................................uaucacagccagcuuugaAg....................  1277  1  OV1
..........................................................uaucacagccagcuuugaug....................  75    0  OV1
..........................................................uaCcacagccagcuuugaug....................  1     1  OV1
..........................................................uaucacagccagcuuugaCg....................  1     1  OV1
..........................................................uaucacagccagcuuugaugU...................  1     1  OV1
..........................................................uaucacagccagcuuugaugG...................  1     1  OV1
..........................................................uaucacagccagcuuugaAga...................  2082  1  OV1
..........................................................uaucacagccagcuuugauga...................  80    0  OV1
..........................................................uaucacagccagcuuugaugaA..................  2     1  OV1
..........................................................uaucacagccagcuuugaugaU..................  1     1  OV1
..........................................................uaucacagccagcuuugaAgag..................  157   1  OV1
..........................................................uaucacagccagcuuugaugag..................  50    0  OV1
..........................................................uaucacagccagcuuugaugagc.................  40    0  OV1
..........................................................uaucacagccagcuuugaAgagc.................  13    1  OV1
..........................................................uaucacagccagcuuugaugagA.................  10    1  OV1
..........................................................uaucacagccagcuuugaugagca................  1     0  OV1
...........................................................aucacagccagcuuugaAgag..................  1     1  OV1
............................................................ucacagccagcuuugaA.....................  1     1  OV1
............................................................ucacagccagcuuugaug....................  3     0  OV1
............................................................ucacagccagcuuugauga...................  7     0  OV1
............................................................ucacagccagcuuugaugag..................  104   0  OV1
............................................................ucacagccagcuuugaugagA.................  7     1  OV1
............................................................ucacagccagcuuugaugagc.................  247   0  OV1
............................................................ucacagccagcAuugaugagc.................  1     1  OV1
............................................................ucacagccagcuuuUaugagca................  1     1  OV1
............................................................ucacagccagcuuuAaugagca................  1     1  OV1
............................................................ucacagUcagcuuugaugagca................  1     1  OV1
............................................................ucacagccagcuuugaugagca................  600   0  OV1
............................................................ucacagccagcuuugaugagcaU...............  14    1  OV1
............................................................ucacagccagcuuugaugagcaa...............  12    0  OV1
............................................................ucacagccagcuuugaugagcaC...............  18    1  OV1
............................................................ucacagccagcuuugaugagcaUu..............  1     1  OV1
.............................................................cacagccagcuuugaugagca................  1     0  OV1
..............................................................acagccagcuuugaAga...................  2     1  OV1
..............................................................acagccagcuuugaugagc.................  2     0  OV1
..............................................................acagccagcuuugaugagca................  1     0  OV1

...................ucaucaaacuugguuguugua..........................................................  7     0  MF1
...................ucaucaaacCugguuguugua..........................................................  1     1  MF1
...................ucaucaaacuugguuguuguaU.........................................................  1     1  MF1
..........................................................uaucacagccagcuuug.......................  2     0  MF1
..........................................................uaucacagccagcuuuga......................  27    0  MF1
..........................................................uaucacagccagcuuugaA.....................  20    1  MF1
..........................................................uaucGcagccagcuuugau.....................  1     1  MF1
..........................................................uaucacagccagcuuugau.....................  4     0  MF1
..........................................................uaucacagccagcuuugaug....................  40    0  MF1
..........................................................uaucacagccagcuuugaAg....................  578   1  MF1
..........................................................uaucacagccaAcuuugaug....................  1     1  MF1
..........................................................uaucacagccagcuuugauga...................  64    0  MF1
..........................................................uaucacagccagcuuugaAga...................  544   1  MF1
..........................................................uaucacagccagcuuuAauga...................  1     1  MF1
..........................................................uaucacagccagcuuugaAgag..................  91    1  MF1
..........................................................uaucacagccagcuuugaugag..................  35    0  MF1
..........................................................uaucacagccagcuuugaugagc.................  24    0  MF1
..........................................................uaucacagccagcuuugaAgagc.................  22    1  MF1
............................................................ucacagccagcuuugaug....................  2     0  MF1
```

```
                                                            aga-miR-2
                   aga-miR-2*
uauugggugccaugcauugucaucaaacuugguuguuguagcuugcuuugauaguugcuaucacagccagcuuugaugagcaaugcgucacuuaccua

............................................................ucacagccagcuuugauga...................   2      0    MF1
............................................................ucacagccagcuuugaugag..................   40     0    MF1
............................................................ucacagccagcuuugaugagc.................   68     0    MF1
............................................................ucacagccagcuuugaugagcG................   3      1    MF1
............................................................ucacagccagcuuugaugagcC................   1      1    MF1
............................................................ucacagccagcuAugaugagca................   1      1    MF1
............................................................ucacagccagcuuuAaugagca................   1      1    MF1
............................................................Ccacagccagcuuugaugagca................   1      1    MF1
............................................................ucacagccagcuuugaugagca................   411    0    MF1
............................................................ucacagccagcuuAgaugagca................   1      1    MF1
............................................................ucacagccaAcuuugaugagca................   1      1    MF1
............................................................ucacagccagcuuugaugagcaa...............   3      0    MF1
............................................................ucacagccagcuuugaugagcaC...............   3      1    MF1
............................................................ucacagccagcuuugaugagcaU...............   10     1    MF1
............................................................ucacagccagcuuugaugagcaUu..............   2      1    MF1
.............................................................cacagccagcuuugaugagca................   1      0    MF1

...................ucaucaaacuugguugu..............................................................   1      0    BF2
...................ucaucaaacuugguuguugua..........................................................   9      0    BF2
...................ucaucaaacuugguuguuguagc........................................................   1      0    BF2
..........................................................uaucacagccagcuuuga......................   4      0    BF2
..........................................................uaucacagccagcuuugaA.....................   6      1    BF2
..........................................................uaucacagccagcuuugaGg....................   1      1    BF2
..........................................................uaucacagccagcuuugaug....................   43     0    BF2
..........................................................uaucacagccagcuuugaAg....................   737    1    BF2
..........................................................uaucacagccagcuuugaAga...................   1214   1    BF2
..........................................................uaucacagccagcuuugauga...................   53     0    BF2
..........................................................uaucacagccagcuuugaAgag..................   220    1    BF2
..........................................................uaucacagccagcuuugaugag..................   64     0    BF2
..........................................................uaucacagccagcuuugaugagc.................   41     0    BF2
..........................................................uaucacagccagcuuugaugagA.................   13     1    BF2
..........................................................uaucacagccagcuuugaAgagc.................   44     1    BF2
..........................................................uaucacagccagcuuugaugagcG................   1      1    BF2
..........................................................uaucacagccagcuuugaugagAa................   1      1    BF2
..........................................................uaucacagccagcuuugaugagca................   1      0    BF2
..........................................................uaucacagccagcuuugaAgagcaa...............   1      1    BF2
...........................................................aucacagccagcuuugaugagc.................   1      0    BF2
...........................................................aucacagccagcuuugaugagca................   1      0    BF2
............................................................ucacagccagcuuugaug....................   2      0    BF2
............................................................ucacagccagcuuugauga...................   4      0    BF2
............................................................ucacagccagcuuugaugag..................   48     0    BF2
............................................................ucacagccagcuuugaugagA.................   3      1    BF2
............................................................ucacagccagcuuugaugagc.................   63     0    BF2
............................................................ucacagccagcuuGgaugagca................   1      1    BF2
............................................................ucacagccagcuuugaugagca................   251    0    BF2
............................................................ucacagccagcuuugaugagcG................   1      1    BF2
............................................................ucacagccagcuuuAaugagca................   1      1    BF2
............................................................ucacagccCgcuuugaugagca................   1      1    BF2
............................................................ucacagccagcuuugaugagcaC...............   5      1    BF2
............................................................ucacagccagcuuugaugagcaa...............   6      0    BF2
............................................................ucacagccagcuuugaugagcaU...............   5      1    BF2
............................................................ucacagccagcuuugaugagcaCu..............   1      1    BF2
............................................................ucacagccagcuuugaugagcaaA..............   1      1    BF2

...................ucaucaaacuugguuguugua..........................................................   1      0    BF1
...................ucaucaaacuugguuguuguagc........................................................   3      0    BF1
..........................................................uaucacagccagcuuuga......................   2      0    BF1
..........................................................uaucacagccagcuuugau.....................   2      0    BF1
..........................................................uaucacagccagcuuugaA.....................   1      1    BF1
..........................................................uaucacagccagcuuugaAg....................   209    1    BF1
..........................................................uaucacagccagcuuugaug....................   9      0    BF1
..........................................................uaucacagccagcuuugauga...................   12     0    BF1
..........................................................uaucacagccagcuuugaAga...................   437    1    BF1
..........................................................uaucacagccagcuuugaugaA..................   1      1    BF1
..........................................................uaucacagccagcuuugaugag..................   26     0    BF1
..........................................................uaucacagccagcuuugaAgag..................   87     1    BF1
..........................................................uaucacagccagcuuugaugagA.................   2      1    BF1
..........................................................uaucacagccagcuuugauAagc.................   1      1    BF1
..........................................................uaucacagccagcuuugaAgagc.................   25     1    BF1
```

aga-miR-2

aga-miR-2*

uauugggugccaugcauugucaucaaacuugguuguuguagcuugcuuugauaguugcuaucacagccagcuuugaugagcaaugcgucacuuaccua

....uaucacagccagcuuugaugagc.................	26	0	BF1
....uaucacagccagcuuugaugagcG................	1	1	BF1
....ucacagccagcuuugauga...................	4	0	BF1
....ucacagccagcuuugaugag..................	23	0	BF1
....ucacagccagcCuugaugagc.................	1	1	BF1
....ucacagccagcuuugaugagc.................	59	0	BF1
....ucacagccagcuuugaugagA.................	1	1	BF1
....uUacagccagcuuugaugagca................	1	1	BF1
....ucacagccagcAuugaugagca................	1	1	BF1
....ucacagccagcuuugUugagca................	1	1	BF1
....ucacagccagcuuugaugagAa................	1	1	BF1
....ucacagccagcuuugaugagca................	172	0	BF1
....ucacagccagcuuugaugagcaa...............	4	0	BF1
....ucacagccagcuuugaugagcaU...............	3	1	BF1
....ucacagccagcuuugaugagcaG...............	1	1	BF1
....ucacagccagcuuugaugagcaC...............	1	1	BF1
....ucacagccagcuuugaugagcaUu..............	1	1	BF1
...................ucaucaaacuugguuguugu...	1	0	FW1
...................ucaucaaacuugguuguugua..	10	0	FW1
...................ucaucaaacuugguuguuguagc..	1	0	FW1
....uaucacagccagcuuugaA.....................	1	1	FW1
....uaucacagccagcuuugaug....................	9	0	FW1
....uaucacagccagcuuugaAg....................	79	1	FW1
....uaucacagccagcuuugaAga...................	119	1	FW1
....uaucacagccagcuuugauga...................	11	0	FW1
....uaucacagccagcuuugaAgag..................	51	1	FW1
....uaucacagccagcuuugaugag..................	5	0	FW1
....uaucacagccagcuuugaugagc.................	29	0	FW1
....uaucacagccagcuuugaAgagc.................	25	1	FW1
....uaucacagccagcuuugaugagA.................	3	1	FW1
....uaucacagccagcuuugaugagca................	1	0	FW1
....uaucacagccagcuuugaAgagca................	1	1	FW1
....uaucacagccagcuuugaugagcG................	4	1	FW1
....ucacagccagcuuugaug....................	4	0	FW1
....ucacagccagcuuugaugag..................	7	0	FW1
....ucaUagccagcuuugaugagc.................	1	1	FW1
....ucacagccagcuuugaugagc.................	30	0	FW1
....ucacagccagcuuugaugagca................	219	0	FW1
....ucacagccagcuuugauAagca................	1	1	FW1
....ucacagccagcuuugaugagcaU...............	3	1	FW1
....ucacagccagcuuugaugagcaC...............	1	1	FW1
....ucacagccagcuuugaugagcaUu..............	1	1	FW1
...................ucaucaaacuugguuguugu...	2	0	MW1
...................ucaucaaacuuAguuguugua..	1	1	MW1
...................ucaucaaacuugguuguugua..	20	0	MW1
...................ucaucaaacuugguuguuguaU...	2	1	MW1
...................ucaucaaacuugguuguuguagc..	1	0	MW1
....uaucacagccagcuuug.......................	2	0	MW1
....uaucacagccagcuuuga......................	1	0	MW1
....uaucacagccagcuuugaA.....................	4	1	MW1
....uaucacagccagcuuugaug....................	22	0	MW1
....uaucacagccagcuuugaAg....................	233	1	MW1
....uaucacagccagcuuugauAa...................	1	1	MW1
....uaucacagccagcuuugauga...................	25	0	MW1
....uaucacagccagcuuugaAga...................	248	1	MW1
....uaucacagccagcuuugaAgag..................	114	1	MW1
....uaucacagccagcuuugaugag..................	31	0	MW1
....uaucacagccagcuuugaAgagc.................	65	1	MW1
....uaucacagccagcuuugaugagU.................	1	1	MW1
....uaucacagccagcuuugaugagc.................	39	0	MW1
....uaucacagccagcuuugaAgagca................	4	1	MW1
....uaucacagccagcuuugaugagcG................	1	1	MW1
....uaucacagccagcuuugaugagcC................	1	1	MW1
....ucacagccagcuuugaug....................	3	0	MW1
....ucacagccagcuuugauga...................	2	0	MW1
....ucacagccagcuuugaugag..................	11	0	MW1
....ucacagccagcuuugaugagc.................	39	0	MW1

aga-miR-2

aga-miR-2*

```
uauugggugccaugcauugucaucaaacuugguuguuguagcuugcuuugauaguugcuaucacagccagcuuugaugagcaaugcgucacuuaccua

............................................................ucacagccagcuuugaugagcG................   1     1    MW1
............................................................uUacagccagcuuugaugagca................   1     1    MW1
............................................................ucacagccagcuuugauAagca................   1     1    MW1
............................................................ucacagccagcuuCgaugagca................   1     1    MW1
............................................................ucacagccagcuuugaCgagca................   2     1    MW1
............................................................ucacagccagcuuugaugagca................   371   0    MW1
............................................................ucacagccagcAuugaugagca................   1     1    MW1
............................................................ucacagccagcuuugaugagcaC...............   5     1    MW1
.............................................................cacagccagcuuugaug....................   2     0    MW1
.............................................................cacagccagcuuugauga...................   2     0    MW1
.............................................................cacagccagcuuugaugagca................   1     0    MW1
..............................................................acagccagcuuugaAgag..................   1     1    MW1
..............................................................acagccagcuuugaugagca................   1     0    MW1

...................ucaucaaacuugguuguugua..........................................................   4     0    MW2
...................ucaucaaacuugguuguuguagc........................................................   1     0    MW2
........................................gcuugcuuugauaguugcua......................................   1     0    MW2
..........................................................uaucacagccagcuuuga......................   1     0    MW2
..........................................................uaucacagccagcuuugaA.....................   2     1    MW2
..........................................................uaucacagccagcuuugaug....................   3     0    MW2
..........................................................uaucacagccagcuuugaAg....................   47    1    MW2
..........................................................uaucacagccagcuuugaAga...................   65    1    MW2
..........................................................uaucacagccagcuuugauga...................   3     0    MW2
..........................................................uaucacagccagcuuugaugag..................   7     0    MW2
..........................................................uaucacagccagcuuugaAgag..................   36    1    MW2
..........................................................uaucacagccagcuuugaugagc.................   20    0    MW2
..........................................................uaucacagccagcuuugaAgagc.................   22    1    MW2
..........................................................uaucacagccagcuuugaAgagca................   1     1    MW2
............................................................ucacagccagcuuCgaugag..................   1     1    MW2
............................................................ucacagccagcuuugaugag..................   4     0    MW2
............................................................ucacagccagcuuugaugaAc.................   1     1    MW2
............................................................ucacagccagcuuugaugagc.................   31    0    MW2
............................................................ucacagccagcuuugaugagca................   100   0    MW2
............................................................ucacagccGgcuuugaugagca................   1     1    MW2
............................................................ucaUagccagcuuugaugagca................   1     1    MW2
............................................................ucacagccagcuuugaugagcaa...............   1     0    MW2
............................................................ucacagccagcuuugaugagcaU...............   1     1    MW2
.............................................................cacagccagcuuugauga...................   2     0    MW2

...................ucaucaaacuugguuguugua..........................................................   1     0    TE2
...................ucaucaaacuugguuguuguaU.........................................................   1     1    TE2
..........................................................uaucacagccagcuuug.......................   2     0    TE2
..........................................................uaucacagccagcuuuga......................   13    0    TE2
..........................................................uaucacagccagcuuugau.....................   1     0    TE2
..........................................................uaucacagccagcuuugaA.....................   12    1    TE2
..........................................................uaucacagccagcuuugaAg....................   212   1    TE2
..........................................................uaucacagccagcuuugaug....................   18    0    TE2
..........................................................uaucacagccagcuuugauga...................   23    0    TE2
..........................................................uaucacagccagcuuugaAga...................   477   1    TE2
..........................................................uaucacagccagcuCugaugag..................   1     1    TE2
..........................................................uaucacagccagcuuugaugag..................   41    0    TE2
..........................................................uaucacagccagcuuugaAgag..................   323   1    TE2
..........................................................uaucacagccCgcuuugaugagc.................   1     1    TE2
..........................................................uaucacagccagcuuugaAgagc.................   105   1    TE2
..........................................................uaucacagccagcuuugaugagc.................   124   0    TE2
..........................................................uaucacagccagcuuugaAgagca................   8     1    TE2
..........................................................uaucacagccagcuuugaugagcG................   4     1    TE2
............................................................ucacagccagcuuugaug....................   5     0    TE2
............................................................ucacagccagcuuugauga...................   9     0    TE2
............................................................ucacagccagcuuugaugU...................   1     1    TE2
............................................................ucacagccagcuuugaugag..................   48    0    TE2
............................................................ucacagccagcuuugaugagA.................   1     1    TE2
............................................................ucacagccagcuuugaugagU.................   1     1    TE2
............................................................ucacagccagcuuugaugagc.................   141   0    TE2
............................................................ucacagccagcuuugaugaAc.................   1     1    TE2
............................................................ucacagccagcuuuAaugagc.................   2     1    TE2
............................................................ucacagccagcuuugaugagcG................   1     1    TE2
............................................................ucacagccagcuuugaugagca................   331   0    TE2
............................................................ucacagccagcuuugaCgagca................   1     1    TE2
```

```
                                                            aga-miR-2
                   aga-miR-2*
uauugggugccaugcauugucaucaaacuugguuguuguagcuugcuuugauaguugcuaucacagccagcuuugaugagcaaugcgucacuuaccua

............................................................ucacagccagcuuugaugagcaC...............   1   1   TE2
............................................................ucacagccagcuuugaugagcaa...............   1   0   TE2
............................................................ucacagccagcuuugaugagcaU...............   4   1   TE2
..............................................................acagccagcuuugaAgag..................   1   1   TE2
```

miRBase precursor	: aga-mir-263a
Total read count	: 978624
aga-miR-263a read count	: 976987
aga-miR-263a* read count	: 1503
remaining reads	: 134

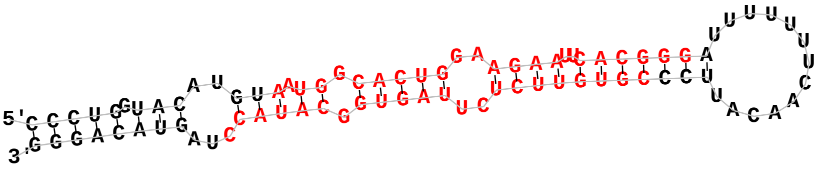

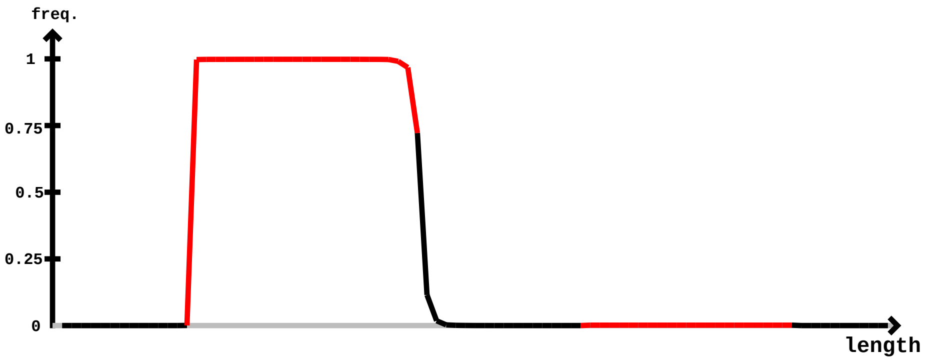

aga-miR-263a

aga-miR-263a*

5'- cccugguacauguaauggcacuggaagaauucacgggauuuuuuucaacauucccguguucucuuaguggcauaccuaguacaggg -3'	exp		
(((((.(((..(((.((.(((((..((((..(((((((.............)))))))))))..))))).)))))...))))))))	reads	mm	sample
............Caauggcacuggaagaauucacgg..	4	1	FF2
............Aaauggcacuggaagaauucacgg..	1	1	FF2
............Caauggcacuggaagaauucacggg...	8	1	FF2
.............aauggcacuggaagaauu...	2	0	FF2
.............aauggcacuggaagaauuca...	20	0	FF2
.............aauggcacuggaagaauucac..	329	0	FF2
.............aauggUacuggaagaauucac..	1	1	FF2
.............aauAgcacuggaagaauucac..	1	1	FF2
.............aaugAcacuggaagaauucac..	1	1	FF2
.............aauggcacuggaagaauucaA..	1	1	FF2
.............aauggcacuggUagaauucac..	1	1	FF2
.............Gauggcacuggaagaauucac..	1	1	FF2
.............aauggcacuggaagaauCcac..	1	1	FF2
.............aauggcacugAaagaauucacg...	2	1	FF2
.............aauggcacCggaagaauucacg...	1	1	FF2
.............aauggcacuggCagaauucacg...	1	1	FF2
.............aauggcacuggaagaauucGcg...	1	1	FF2
.............aauggcacuggGagaauucacg...	1	1	FF2
.............aauggcacuggaagaauuUacg...	1	1	FF2
.............aauggUacuggaagaauucacg...	1	1	FF2
.............aauggcacuggaagaauucacU...	1	1	FF2
.............aauggcacuggaagaauucacg...	877	0	FF2
.............aauggcacuggaagaauucacA...	2	1	FF2
.............aaugAcacuggaagaauucacg...	1	1	FF2
.............aauggcacuggaagaGuucacgg..	1	1	FF2
.............aauggcacuAgaagaauucacgg..	1	1	FF2
.............aauggcacuggaagaaAucacgg..	1	1	FF2
.............aauggcacuggaagaaCucacgg..	3	1	FF2
.............aauggcacuggaagaauucGcgg..	1	1	FF2
.............aauggGacuggaagaauucacgg..	1	1	FF2
.............aauggcaUuggaagaauucacgg..	4	1	FF2
.............aauggcacuggaagaauucacgA..	8	1	FF2
.............aauggcacuggCagaauucacgg..	3	1	FF2
.............Gauggcacuggaagaauucacgg..	1	1	FF2

```
            aga-miR-263a
                                                      aga-miR-263a*
cccugguacauguaauggcacuggaagaauucacgggauuuuuuucaacauucccguguucucuuaguggcauaccuaguacaggg

.............aauggcacuggaaAaauucacgg..................................................   1       1   FF2
.............aauggUacuggaagaauucacgg..................................................   1       1   FF2
.............aauggcacuggaagaauucacgU..................................................   3       1   FF2
.............aauggcacuggaagGauucacgg..................................................   1       1   FF2
.............aauggcacuggaagaauucaUgg..................................................   4       1   FF2
.............aauggcacuggaagaauuAacgg..................................................   1       1   FF2
.............aauggcacuUgaagaauucacgg..................................................   1       1   FF2
.............aaugCcacuggaagaauucacgg..................................................   2       1   FF2
.............aauggcacuggaagaauucacAg..................................................   2       1   FF2
.............aauggcacuggaagaauucacgg..................................................   5492    0   FF2
.............aauAgcacuggaagaauucacgg..................................................   3       1   FF2
.............aUuggcacuggaagaauucacgg..................................................   1       1   FF2
.............Cauggcacuggaagaauucacgg..................................................   1       1   FF2
.............aauggAacuggaagaauucacgg..................................................   2       1   FF2
.............aauggcacugCaagaauucacgg..................................................   1       1   FF2
.............aauggcacugAaagaauucacgg..................................................   5       1   FF2
.............aauggcacugUaagaauucacgg..................................................   1       1   FF2
.............aauggcacuggUagaauucacgg..................................................   3       1   FF2
.............aauggcaGuggaagaauucacgg..................................................   1       1   FF2
.............aauggcacuggGagaauucacgg..................................................   1       1   FF2
.............aauggcacCggaagaauucacgg..................................................   2       1   FF2
.............aauggcacuCgaagaauucacgg..................................................   1       1   FF2
.............aauggcacuggaagaauucacUg..................................................   3       1   FF2
.............aauggcacuggaagaauucacCg..................................................   1       1   FF2
.............aauggcacuggaagaauCcacgg..................................................   1       1   FF2
.............aauggcacuggaagaauuUacgg..................................................   2       1   FF2
.............aauggcCcuggaagaauucacggg.................................................   1       1   FF2
.............aauggcacuggaagaauAcacggg.................................................   1       1   FF2
.............aauggcacCggaagaauucacggg.................................................   4       1   FF2
.............aauggcacuggaagaauucacggA.................................................   27      1   FF2
.............aaugAcacuggaagaauucacggg.................................................   6       1   FF2
.............aauggcacuggaagaaCucacggg.................................................   7       1   FF2
.............Gauggcacuggaagaauucacggg.................................................   3       1   FF2
.............aauggcacuggaaCaauucacggg.................................................   1       1   FF2
.............aauggcacuggaagaauuAacggg.................................................   1       1   FF2
.............aauggcacuggaagaCuucacggg.................................................   4       1   FF2
.............Uauggcacuggaagaauucacggg.................................................   4       1   FF2
.............aaGggcacuggaagaauucacggg.................................................   2       1   FF2
.............aauggcacuggaagaauucacAgg.................................................   17      1   FF2
.............aauggcacuggaagaauucacggC.................................................   15      1   FF2
.............aauggcacuggaagaauucacgAg.................................................   10      1   FF2
.............aauCgcacuggaagaauucacggg.................................................   4       1   FF2
.............aauggcacuggaagaauucUcggg.................................................   2       1   FF2
.............aauggcacuggGagaauucacggg.................................................   4       1   FF2
.............aauggcacuggaagaUuucacggg.................................................   1       1   FF2
.............aauggcGcuggaagaauucacggg.................................................   2       1   FF2
.............aauggcacuggaagaauucacCgg.................................................   5       1   FF2
.............aauggcacuCgaagaauucacggg.................................................   4       1   FF2
.............aauggcacuggaagaGuucacggg.................................................   9       1   FF2
.............aauAgcacuggaagaauucacggg.................................................   8       1   FF2
.............aauggcacugCaagaauucacggg.................................................   4       1   FF2
.............aauggcacuggaagaaAucacggg.................................................   4       1   FF2
.............aauggcacugAaagaauucacggg.................................................   12      1   FF2
.............aauggcacuggaagaauucacggU.................................................   11      1   FF2
.............aaCggcacuggaagaauucacggg.................................................   3       1   FF2
.............aauggcacuggaagaauuUacggg.................................................   20      1   FF2
.............aauggUacuggaagaauucacggg.................................................   8       1   FF2
.............aauggcacuggaagaauCcacggg.................................................   5       1   FF2
.............aauggcacuUgaagaauucacggg.................................................   4       1   FF2
.............aauggcacuggCagaauucacggg.................................................   10      1   FF2
.............aauggcacuggaagaauucacggg.................................................   16578   0   FF2
.............aauggcacuggaagaauucacUgg.................................................   4       1   FF2
.............aauUgcacuggaagaauucacggg.................................................   1       1   FF2
.............aauggcacuggaaAaauucacggg.................................................   4       1   FF2
.............aauggAacuggaagaauucacggg.................................................   1       1   FF2
.............aauggcacugUaagaauucacggg.................................................   7       1   FF2
.............aauggcacuggUagaauucacggg.................................................   4       1   FF2
.............aauggcacuggaagaauucaUggg.................................................   11      1   FF2
.............aaugUcacuggaagaauucacggg.................................................   2       1   FF2
```

```
             aga-miR-263a
                                                     aga-miR-263a*
cccugguacauguaauggcacuggaagaauucacgggauuuuuuucaacauucccguguucucuuaguggcauaccuaguacaggg

.............aUuggcacuggaagaauucacggg.................................................    1      1     FF2
.............aaAggcacuggaagaauucacggg.................................................    1      1     FF2
.............aauggcacuAgaagaauucacggg.................................................    6      1     FF2
.............aauggcaAuggaagaauucacggg.................................................    1      1     FF2
.............aauggcaGuggaagaauucacggg.................................................    1      1     FF2
.............aauggcacuggaUgaauucacggg.................................................    1      1     FF2
.............aauggcacuggaagGauucacggg.................................................    1      1     FF2
.............aauggcacuggaagaauucGcggg.................................................    4      1     FF2
.............aauggcacuggaagaauucGcggga................................................    1      1     FF2
.............aauggcacuggaagaauucacgCga................................................    1      1     FF2
.............aauggcacuggaagaauucacggga................................................    2199   0     FF2
.............aauggcacuCgaagaauucacggga................................................    2      1     FF2
.............aauggcacuggaagaauuUacggga................................................    1      1     FF2
.............aauggcacuggaaUaauucacggga................................................    1      1     FF2
.............aauggcacuggCagaauucacggga................................................    2      1     FF2
.............aauggcacuggaagaaAucacggga................................................    1      1     FF2
.............aauggcacuggaagaauucacggAa................................................    12     1     FF2
.............aauggcacuggaagaauucacgggU................................................    186    1     FF2
.............aauggcacuggaagaauAcacggga................................................    1      1     FF2
.............aauggGacuggaagaauucacggga................................................    1      1     FF2
.............aauggcacuggGagaauucacggga................................................    1      1     FF2
.............aauggcacuggaagaauucacggUa................................................    2      1     FF2
.............aaAggcacuggaagaauucacggga................................................    1      1     FF2
.............aauggcacugAaagaauucacggga................................................    2      1     FF2
.............aauggcacuggaagaauucacgAga................................................    1      1     FF2
.............aauggcacuggaagaauucacgUga................................................    1      1     FF2
.............aauggcacuggaagaauucacgggG................................................    12     1     FF2
.............aauggcacCggaagaauucacggga................................................    2      1     FF2
.............aaugUcacuggaagaauucacggga................................................    1      1     FF2
.............aauggcacuggaagaauucaUggga................................................    2      1     FF2
.............aauggcacuAgaagaauucacggga................................................    2      1     FF2
.............aauggcacugUaagaauucacggga................................................    1      1     FF2
.............aauggcacuggaagaauucacgggC................................................    10     1     FF2
.............aauggcacuggaagaaCucacggga................................................    1      1     FF2
.............aaCggcacuggaagaauucacggga................................................    4      1     FF2
.............aauggcaUuggaagaauucacggga................................................    1      1     FF2
.............aauggcacuggaagaauucacgggau...............................................    184    0     FF2
.............aauggcacuggaagaauucacggAau...............................................    1      1     FF2
.............aauggcacuggaagaauucacgggUu...............................................    15     1     FF2
.............aauggcacuggaaAaauucacgggau...............................................    1      1     FF2
.............aauggcacuggaagaauucacgggCu...............................................    2      1     FF2
.............aauggcacuggaagaauucacgggaC...............................................    2      1     FF2
.............aauggcacuggaagaauucacgggaA...............................................    207    1     FF2
.............aauggcacuggaagaauucacgggauu..............................................    14     0     FF2
.............aauggcacuggaagaauucacgggaAu..............................................    45     1     FF2
.............aauggcacuggaagaauucacgggGuu..............................................    1      1     FF2
.............aauggcacuggaagaauucacgggauA..............................................    3      1     FF2
.............aauggcacuggaagaaCucacgggauu..............................................    1      1     FF2
.............aauggcacuggaagaauucacgggUuu..............................................    5      1     FF2
.............aauggcacuggaagaauucacgggUuuu.............................................    5      1     FF2
.............aauggcacuggaagaauucacgggauCu.............................................    1      1     FF2
.............aauggcacuggaagaauucacgggaAuu.............................................    2      1     FF2
.............aauggcacuggaagaauucacgggauAu.............................................    7      1     FF2
.............aauggcacuggaagaauucacgggauuu.............................................    4      0     FF2
.............aauggcacuggaagaauucacgggauuA.............................................    1      1     FF2
.............aauggcacuggaagaauucacgggUuuuu............................................    2      1     FF2
.............aauggcacuggaagaauucacgggauuAu............................................    3      1     FF2
.............aauggcacuggaagaauucacgggauuuu............................................    2      0     FF2
.............aauggcacuggaagaauucacgggaAuuu............................................    4      1     FF2
.............aauggcacuggaagaauucacgggUuuuuu...........................................    1      1     FF2
.............aauggcacuggaagaauucacgggauAuuu...........................................    1      1     FF2
.............aauggcacuggaagaauucacgggauuuAu...........................................    2      1     FF2
.............aauggcacuggaagaauucacgggauuuCu...........................................    1      1     FF2
.............aauggcacuggaagaauucacgggauuuuu...........................................    1      0     FF2
.............aauggcacuggaagaauucacgggauuuAuu..........................................    1      1     FF2
..............auggcacuggaagaauucacggg.................................................    2      0     FF2
..............auggcacuggaagaauucacggga................................................    4      0     FF2
...............uggcacuggaagaauucacggg.................................................    1      0     FF2
..................cacuggaagaauucacgg..................................................    1      0     FF2
```

aga-miR-263a

aga-miR-263a*

cccugguacauguaauggcacuggaagaauucacgggauuuuuuucaacauucccguguucucuuaguggcauaccuaguacaggg

....................cuggaagaauucacggg...	2	0	FF2
....cguguucucuuaguggcauac...........	1	0	FF2
....cguguucucuuaguggcauacc..........	13	0	FF2
............Caauggcacuggaagaauucac..	1	1	OV2
............Caauggcacuggaagaauucacg...	1	1	OV2
............Caauggcacuggaagaauucacgg..	5	1	OV2
............uaauggcacuggaagaauucacgg..	1	0	OV2
............Caauggcacuggaagaauucacggg...	7	1	OV2
............Caauggcacuggaagaauucacggga..	2	1	OV2
.............aauggcacuggaagaau..	1	0	OV2
.............aauggcacuggaagaauu...	10	0	OV2
.............aauggcacuggaagaauuca...	54	0	OV2
.............aauggcacugAaagaauucac..	1	1	OV2
.............aauggcacuggaagaauucac..	466	0	OV2
.............aauggcacuggaagaauucaA..	1	1	OV2
.............Gauggcacuggaagaauucac..	2	1	OV2
.............aauAgcacuggaagaauucac..	1	1	OV2
.............aauggcaAuggaagaauucac..	1	1	OV2
.............aauggcacuggaagaauuUacg..	2	1	OV2
.............aauggcacuggGagaauucacg...	1	1	OV2
.............aauggcacCggaagaauucacg...	1	1	OV2
.............Uauggcacuggaagaauucacg...	2	1	OV2
.............aauggcacAggaagaauucacg...	1	1	OV2
.............aauggcacugCaagaauucacg...	3	1	OV2
.............aauggcacuggaagaauucacU...	1	1	OV2
.............aauggcacuggaagaauucacg...	2490	0	OV2
.............aauggcacugUaagaauucacg...	1	1	OV2
.............aaAggcacuggaagaauucacg...	1	1	OV2
.............aauggcacuggaaUaauucacg...	1	1	OV2
.............aauggcacuggaagaauucacA...	6	1	OV2
.............aauAgcacuggaagaauucacg...	1	1	OV2
.............aaugAcacuggaagaauucacg...	1	1	OV2
.............aauggcacuggaCgaauucacg...	1	1	OV2
.............aaCggcacuggaagaauucacg...	2	1	OV2
.............aauggcacuggaagaauucGcg...	1	1	OV2
.............aauggcacuggaagaauucCcgg..	1	1	OV2
.............aauggcacugUaagaauucacgg..	10	1	OV2
.............aaCggcacuggaagaauucacgg..	7	1	OV2
.............Gauggcacuggaagaauucacgg..	4	1	OV2
.............aaGggcacuggaagaauucacgg..	2	1	OV2
.............aauggcaUuggaagaauucacgg..	3	1	OV2
.............aauggcacuggaagaauGcacgg..	1	1	OV2
.............aauggcacuggaaCaauucacgg..	1	1	OV2
.............aauggcacuggaagaauuAacgg..	1	1	OV2
.............aaugCcacuggaagaauucacgg..	1	1	OV2
.............aauggcacCggaagaauucacgg..	10	1	OV2
.............aauggcacuggaagaauucGcgg..	4	1	OV2
.............aauggcacuggaagaauuUacgg..	22	1	OV2
.............aauggcacuggaagaaCucacgg..	7	1	OV2
.............aauggcacuggaagaauucaGgg..	1	1	OV2
.............aauggGacuggaagaauucacgg..	1	1	OV2
.............aauggcacuggaagaauCcacgg..	6	1	OV2
.............aauggcacuggaagaauucacUg..	1	1	OV2
.............aauggcacuggCagaauucacgg..	10	1	OV2
.............aaugUcacuggaagaauucacgg..	5	1	OV2
.............aauggcacuggaagaGuucacgg..	3	1	OV2
.............aauggcacuggaaUaauucacgg..	1	1	OV2
.............aauggcacuggaagaauucacgU..	3	1	OV2
.............aauggAacuggaagaauucacgg..	6	1	OV2
.............aaAggcacuggaagaauucacgg..	2	1	OV2
.............aauggcacuAgaagaauucacgg..	10	1	OV2
.............aauggcacuggaagaUuucacgg..	2	1	OV2
.............Cauggcacuggaagaauucacgg..	1	1	OV2
.............aauggcacuggaagaaAucacgg..	1	1	OV2
.............aauggcacuggGagaauucacgg..	7	1	OV2
.............aauggcGcuggaagaauucacgg..	3	1	OV2
.............Uauggcacuggaagaauucacgg..	5	1	OV2
.............aauggcacuggaagaauucacAg..	10	1	OV2

```
             aga-miR-263a
                                                        aga-miR-263a*
cccugguacauguaauggcacuggaagaauucacgggauuuuuuucaacauucccguguucucuuaguggcauaccuaguacaggg

.............aauggcacuggaaAaauucacgg..................................................   3       1   OV2
.............aaugAcacuggaagaauucacgg..................................................   12      1   OV2
.............aauggcacuggaGgaauucacgg..................................................   3       1   OV2
.............aauggcacugAaagaauucacgg..................................................   20      1   OV2
.............aauCgcacuggaagaauucacgg..................................................   1       1   OV2
.............aauggcacuggaagaauucacgg..................................................   26456   0   OV2
.............aauggcacGggaagaauucacgg..................................................   1       1   OV2
.............aauggcacuggaagaauucacgC..................................................   4       1   OV2
.............aauggcacuUgaagaauucacgg..................................................   5       1   OV2
.............aauggUacuggaagaauucacgg..................................................   9       1   OV2
.............aauggcacuCgaagaauucacgg..................................................   1       1   OV2
.............aUuggcacuggaagaauucacgg..................................................   2       1   OV2
.............aauggcacuggaagaauucacgA..................................................   40      1   OV2
.............aauggcacugCaagaauucacgg..................................................   6       1   OV2
.............aauggcacuggUagaauucacgg..................................................   5       1   OV2
.............aauggcacuggaagaauuGacgg..................................................   4       1   OV2
.............aauggcacuggaagaauucaUgg..................................................   9       1   OV2
.............aauggcacuggaagUauucacgg..................................................   1       1   OV2
.............aauggcacuggaagaauucaAgg..................................................   1       1   OV2
.............aauggcacuggaagaauAcacgg..................................................   1       1   OV2
.............aauggcacuggaagCauucacgg..................................................   1       1   OV2
.............aGuggcacuggaagaauucacgg..................................................   2       1   OV2
.............aauAgcacuggaagaauucacgg..................................................   9       1   OV2
.............aauUgcacuggaagaauucacgg..................................................   1       1   OV2
.............aauggcacuggaaCaauucacggg.................................................   4       1   OV2
.............Gauggcacuggaagaauucacggg.................................................   1       1   OV2
.............aauggcacuggaagaauucacggC.................................................   6       1   OV2
.............aauggcacuggaagaGuucacggg.................................................   3       1   OV2
.............aauggcacuggaagaauCcacggg.................................................   7       1   OV2
.............aauggcacuggUagaauucacggg.................................................   5       1   OV2
.............aauggcaUuggaagaauucacggg.................................................   1       1   OV2
.............aauggcacAggaagaauucacggg.................................................   1       1   OV2
.............aauggcacuggaagaauuAacggg.................................................   1       1   OV2
.............aauggcacuggaagaauucacggg.................................................   24848   0   OV2
.............aauggcacCggaagaauucacggg.................................................   9       1   OV2
.............aauggcacuggaGgaauucacggg.................................................   1       1   OV2
.............aauggcacuUgaagaauucacggg.................................................   2       1   OV2
.............aauggAacuggaagaauucacggg.................................................   3       1   OV2
.............aauggcacuggaaUaauucacggg.................................................   2       1   OV2
.............aauggcacuggaagaauucacgAg.................................................   5       1   OV2
.............aauggcacuggaagaauucacAgg.................................................   6       1   OV2
.............aauggcacuggaagaauucaGggg.................................................   2       1   OV2
.............Uauggcacuggaagaauucacggg.................................................   1       1   OV2
.............aUuggcacuggaagaauucacggg.................................................   2       1   OV2
.............aauggcacuCgaagaauucacggg.................................................   3       1   OV2
.............aauggcacuggaagaauucaUggg.................................................   9       1   OV2
.............aauggcacGggaagaauucacggg.................................................   2       1   OV2
.............aauggcacuggaagaauAcacggg.................................................   2       1   OV2
.............aauggcacuggaCgaauucacggg.................................................   2       1   OV2
.............aauggcacugCaagaauucacggg.................................................   10      1   OV2
.............aauggcGcuggaagaauucacggg.................................................   3       1   OV2
.............aauggcacuggaagaaCucacggg.................................................   22      1   OV2
.............aauggcacuggaagGauucacggg.................................................   4       1   OV2
.............aauggUacuggaagaauucacggg.................................................   6       1   OV2
.............aauAgcacuggaagaauucacggg.................................................   8       1   OV2
.............aauCgcacuggaagaauucacggg.................................................   1       1   OV2
.............aaugAcacuggaagaauucacggg.................................................   4       1   OV2
.............aauggcacugUaagaauucacggg.................................................   11      1   OV2
.............aauggcacuggaagaauucaAggg.................................................   1       1   OV2
.............aauggcacuggCagaauucacggg.................................................   5       1   OV2
.............aauUgcacuggaagaauucacggg.................................................   2       1   OV2
.............aauggcaAuggaagaauucacggg.................................................   2       1   OV2
.............aauggcacuggaagaauucacgCg.................................................   2       1   OV2
.............aauggcacuggaagaauucacggA.................................................   151     1   OV2
.............aauggcacuggaaAaauucacggg.................................................   6       1   OV2
.............aaAggcacuggaagaauucacggg.................................................   3       1   OV2
.............aauggcacuAgaagaauucacggg.................................................   5       1   OV2
.............aauggcacuggaagaauucacCgg.................................................   2       1   OV2
.............aaCggcacuggaagaauucacggg.................................................   6       1   OV2
```

```
             aga-miR-263a
                                         aga-miR-263a*
cccugguacauguaauggcacuggaagaauucacgggauuuuuuucaacauucccguguucucuuaguggcauaccuaguacaggg

.............aauggcacuggGagaauucacggg.................................................    8      1    OV2
.............aauggcacuggaagaauuGacggg.................................................    1      1    OV2
.............aauggcacuggaagaaAucacggg.................................................    2      1    OV2
.............aauggcacuggaagaauucacggU.................................................    16     1    OV2
.............aauggcacugAaagaauucacggg.................................................    24     1    OV2
.............aauggcacuggaagaauucCcggg.................................................    1      1    OV2
.............aauggcacuggaagaauuUacggg.................................................    18     1    OV2
.............aaGggcacuggaagaauucacggg.................................................    3      1    OV2
.............aGuggcacuggaagaauucacggg.................................................    3      1    OV2
.............aauggcacuggaagaauucGcggg.................................................    4      1    OV2
.............aauggcacuggaagaauucacgUg.................................................    3      1    OV2
.............aaugCcacuggaagaauucacggg.................................................    2      1    OV2
.............aauggcacuggaagaauucacggAa................................................    119    1    OV2
.............aauggcacuggUagaauucacggga................................................    2      1    OV2
.............aauggcacuggaaAaauucacggga................................................    1      1    OV2
.............aauggcacuggaagaaCucacggga................................................    6      1    OV2
.............aauggcacuggaagaauucGcggga................................................    1      1    OV2
.............aauggUacuggaagaauucacggga................................................    4      1    OV2
.............aauggcacuggaUgaauucacggga................................................    1      1    OV2
.............aauCgcacuggaagaauucacggga................................................    1      1    OV2
.............aauggcacuggaagaauucaUggga................................................    2      1    OV2
.............aauggcacugCaagaauucacggga................................................    4      1    OV2
.............aauggAacuggaagaauucacggga................................................    1      1    OV2
.............aauggcacugAaagaauucacggga................................................    6      1    OV2
.............aauggcacuggaagaauuUacggga................................................    4      1    OV2
.............aauAgcacuggaagaauucacggga................................................    2      1    OV2
.............aauggcacuggaagaauCcacggga................................................    3      1    OV2
.............aauggcacuggCagaauucacggga................................................    4      1    OV2
.............aauggcacuggaagaauAcacggga................................................    3      1    OV2
.............aauggcacuAgaagaauucacggga................................................    5      1    OV2
.............aauggcacugUaagaauucacggga................................................    5      1    OV2
.............aaAggcacuggaagaauucacggga................................................    1      1    OV2
.............aauggcacuggaagaauucacgggG................................................    22     1    OV2
.............aauggcacuggaagaauucacAgga................................................    3      1    OV2
.............aauggcacuCgaagaauucacggga................................................    1      1    OV2
.............aauggcacuggaagaauucacgggC................................................    18     1    OV2
.............aauggcacuggaagGauucacggga................................................    1      1    OV2
.............aauggcacuggaagaauucacgAga................................................    1      1    OV2
.............aauggcacuggaagaauucacgggU................................................    385    1    OV2
.............aauggcacuggaGgaauucacggga................................................    1      1    OV2
.............aauggcacCggaagaauucacggga................................................    3      1    OV2
.............aauggcacuggaagaauucacggga................................................    7462   0    OV2
.............aaCggcacuggaagaauucacggga................................................    1      1    OV2
.............Uauggcacuggaagaauucacggga................................................    2      1    OV2
.............aauggcacuggaagaauucacggUa................................................    4      1    OV2
.............aauggcacuggGagaauucacggga................................................    3      1    OV2
.............aauggcacGggaagaauucacggga................................................    1      1    OV2
.............aauggcacuggaagaauuAacggga................................................    1      1    OV2
.............aauggcacuggaagaaGucacggga................................................    1      1    OV2
.............aauggcacuggaagaauucaAggga................................................    1      1    OV2
.............aauggcacuggaagaauucacgggCu...............................................    3      1    OV2
.............aauggcacuggaagaauucacgggUu...............................................    17     1    OV2
.............aauAgcacuggaagaauucacgggau...............................................    1      1    OV2
.............aauggcacuggaagaauucacgggaA...............................................    1068   1    OV2
.............aauggcacuggaagaauucacgggau...............................................    286    0    OV2
.............aauggcacuggaagaaCucacgggau...............................................    1      1    OV2
.............aauggcacuggaagaauucacgAgau...............................................    1      1    OV2
.............aauggcacuggaagaauucacgggaC...............................................    21     1    OV2
.............aauggcacuggaagaauucacggAau...............................................    1      1    OV2
.............aauggcacuggaagaauucacgggUuu..............................................    7      1    OV2
.............aauggcacuggaagaauucacgggaAu..............................................    62     1    OV2
.............aauggcacuggaagaauucacgggauA..............................................    8      1    OV2
.............aauggcacuggaagaauucacgggauu..............................................    17     0    OV2
.............aauggcacuggaagaauucacgggauC..............................................    1      1    OV2
.............aauggcacuggaagaauucacgggaCu..............................................    3      1    OV2
.............aauggcacuggaagaauucacgggauCu.............................................    2      1    OV2
.............aauggcacuggaagaauucacgggauAu.............................................    3      1    OV2
.............aauggcacuggaagaauucacgggUuuu.............................................    2      1    OV2
.............aauggcacuggaagaauucacgggaCuu.............................................    1      1    OV2
```

```
             aga-miR-263a
                                                      aga-miR-263a*
cccugguacauguaauggcacuggaagaauucacgggauuuuuuucaacauucccguguucucuuaguggcauaccuaguacaggg

.............aauggcacuggaagaauucacgggauuu.............................................  6     0  OV2
.............aauggcacuggaagaauucacgggaCuuu............................................  1     1  OV2
.............aauggcacuggaagaauucacgggauuuu............................................  5     0  OV2
.............aauggcacuggaagaauucacgggaAuuu............................................  2     1  OV2
.............aauggcacuggaagaauucacgggauuuA............................................  1     1  OV2
.............aauggcacuggaagaauucacgggUuuuu............................................  1     1  OV2
.............aauggcacuggaagaauucacgggauuuuuu..........................................  1     0  OV2
..............auggcacuggaagaauucacg...................................................  2     0  OV2
..............auggcacuggaagaauucacgg..................................................  13    0  OV2
..............auggcacuggaagaauucacggg.................................................  12    0  OV2
..............auggcacuggaagaauucacgggG................................................  1     1  OV2
..............auggcacuggaagaauucacggga................................................  22    0  OV2
..............auggcacuggaagaauucacgggaA...............................................  2     1  OV2
..............auggcacuggaagaauucacgggaAuuu............................................  1     1  OV2
...............uggcacuggaagaauucacggg.................................................  1     0  OV2
...............uggcacuggaagaauucacggga................................................  1     0  OV2
..................cacuggaagaauucacggA.................................................  1     1  OV2
...................acuggaagaauucacggU.................................................  1     1  OV2
....................cuggaagaauucacggg.................................................  1     0  OV2
..................................................uucccguguucucuuaguggcauacc..........  1     0  OV2
......................................................cguguucucuuaguggcauacc..........  15    0  OV2

...........gGaauggcacuggaagaauucacgg..................................................  1     1  TE1
............Caauggcacuggaagaauucacgg..................................................  6     1  TE1
............uaauggcacuggaagaauucacgg..................................................  1     0  TE1
............Caauggcacuggaagaauucacggg.................................................  1     1  TE1
............Caauggcacuggaagaauucacggga................................................  1     1  TE1
.............aauggcacuggaagaau........................................................  1     0  TE1
.............aauggcacuggaagaauu.......................................................  8     0  TE1
.............aauggcacuggaagaauuca.....................................................  8     0  TE1
.............aauggcacugAaagaauucac....................................................  1     1  TE1
.............aauggcacuggaagaauucac....................................................  78    0  TE1
.............aauggcacuggaagaUuucac....................................................  1     1  TE1
.............aauggcacuggGagaauucacg...................................................  1     1  TE1
.............aauggcacuggaagaauucacA...................................................  1     1  TE1
.............aauggcacuggaagaauucacU...................................................  1     1  TE1
.............aauggcacuggaagaauucacg...................................................  350   0  TE1
.............aauggcaAuggaagaauucacg...................................................  1     1  TE1
.............aaCggcacuggaagaauucacgg..................................................  1     1  TE1
.............aauggcacuggaagaauucacUg..................................................  2     1  TE1
.............aaAggcacuggaagaauucacgg..................................................  1     1  TE1
.............aauggcacuggaaUaauucacgg..................................................  1     1  TE1
.............aaGggcacuggaagaauucacgg..................................................  1     1  TE1
.............Gauggcacuggaagaauucacgg..................................................  1     1  TE1
.............aaugAcacuggaagaauucacgg..................................................  2     1  TE1
.............aauggcacugUaagaauucacgg..................................................  1     1  TE1
.............aauggcacuggaagaauucGcgg..................................................  2     1  TE1
.............aauAgcacuggaagaauucacgg..................................................  2     1  TE1
.............aauggcacuggaaCaauucacgg..................................................  1     1  TE1
.............aauggcacuggGagaauucacgg..................................................  2     1  TE1
.............aauggcacuggaagaauuUacgg..................................................  3     1  TE1
.............aauggcacuggaagaauucacgC..................................................  1     1  TE1
.............aauggGacuggaagaauucacgg..................................................  1     1  TE1
.............aauggcaGuggaagaauucacgg..................................................  1     1  TE1
.............aauggcacuggaagaaCucacgg..................................................  1     1  TE1
.............aauggcacuUgaagaauucacgg..................................................  1     1  TE1
.............aauggcacuggaagaauCcacgg..................................................  2     1  TE1
.............aauggcacugCaagaauucacgg..................................................  2     1  TE1
.............aauggcacuggCagaauucacgg..................................................  2     1  TE1
.............aauggcacuggaagaauucacAg..................................................  1     1  TE1
.............aauggcacuggaagaGuucacgg..................................................  1     1  TE1
.............aauggcacuggaGgaauucacgg..................................................  2     1  TE1
.............aauggUacuggaagaauucacgg..................................................  1     1  TE1
.............aauggcacuggaagaauucacgA..................................................  3     1  TE1
.............aauggcacugAaagaauucacgg..................................................  4     1  TE1
.............aauggcacuggaagaauucaUgg..................................................  3     1  TE1
.............aauggcacuAgaagaauucacgg..................................................  3     1  TE1
.............aauggcacuggaagaauucacgg..................................................  3796  0  TE1
.............aauggcacuggaagaauucacgUg.................................................  1     1  TE1
```

```
             aga-miR-263a
                                                 aga-miR-263a*
cccugguacauguaauggcacuggaagaauucacgggauuuuuuucaacauucccguguucucuuaguggcauaccuaguacaggg

.............aauggcacuggaagaauucacggU.................................................   4      1    TE1
.............aauggcacuggaagaaCucacggg.................................................   1      1    TE1
.............aauggcacuggGagaauucacggg.................................................   2      1    TE1
.............aaCggcacuggaagaauucacggg.................................................   2      1    TE1
.............aauggcacCggaagaauucacggg.................................................   2      1    TE1
.............aaugUcacuggaagaauucacggg.................................................   1      1    TE1
.............aauggUacuggaagaauucacggg.................................................   3      1    TE1
.............aauAgcacuggaagaauucacggg.................................................   1      1    TE1
.............aauggcacuggaagaauucacUgg.................................................   1      1    TE1
.............aauggcacuggaagaauucacggg.................................................   1918   0    TE1
.............aauggcacuggaagaauucacgAg.................................................   3      1    TE1
.............aauggcacuggaagaauuUacggg.................................................   2      1    TE1
.............aauggcacugUaagaauucacggg.................................................   3      1    TE1
.............aauggcacuggaagaauucacggA.................................................   4      1    TE1
.............aauggcacuggUagaauucacggg.................................................   1      1    TE1
.............aauggcacuggaagaauucaUggg.................................................   3      1    TE1
.............aauggcacugAaagaauucacggg.................................................   2      1    TE1
.............aauggcacuggaagaaAucacggg.................................................   1      1    TE1
.............aauggcacugCaagaauucacggg.................................................   1      1    TE1
.............aauggcacuggaagaauucacAgg.................................................   1      1    TE1
.............aauggcacuUgaagaauucacggg.................................................   2      1    TE1
.............aauggcacuggaagaGuucacggg.................................................   1      1    TE1
.............aauggcacugCaagaauucacggga................................................   1      1    TE1
.............aauggcacuggaagaauucacggga................................................   458    0    TE1
.............aauggcacuggaagaauucacgggC................................................   4      1    TE1
.............aauggUacuggaagaauucacggga................................................   1      1    TE1
.............aauggcacuggaagaaCucacggga................................................   1      1    TE1
.............aauggcacugAaagaauucacggga................................................   2      1    TE1
.............aauggcacuggaagaauucacggAa................................................   5      1    TE1
.............aauggcacuggaagaauucacgggU................................................   46     1    TE1
.............aauggcacuggaagaauucacgggaA...............................................   49     1    TE1
.............aauggcacuggaagaauucacgggCu...............................................   1      1    TE1
.............aauggcacuggaagaauucacgggau...............................................   42     0    TE1
.............aauggcacuggaagaauucacgggaC...............................................   2      1    TE1
.............aauggcacuggaagaauucacgggUu...............................................   1      1    TE1
.............aauggcacuggaagaauucacgggaAu..............................................   11     1    TE1
.............aauggcacuggaagaauucacgggauu..............................................   3      0    TE1
.............aauggcacuggaagaauucacgggaCu..............................................   1      1    TE1
.............aauggcacuggaagaauucacgggaAuu.............................................   3      1    TE1
.............aauggcacuggaagaauucacgggauuu.............................................   1      0    TE1
.............aauggcacuggaagaauucacgggauuuu............................................   2      0    TE1
.............aauggcacuggaagaauucacgggaAuuu............................................   2      1    TE1
.............aauggcacuggaagaauucacgggauuuuu...........................................   1      0    TE1
.............aauggcacuggaagaauucacgggauAuuu...........................................   1      1    TE1
.............aauggcacuggaagaauucacgggauuuuuu..........................................   1      0    TE1
..............auggcacuggaagaauucacgg..................................................   1      0    TE1
...............uggcacuggaagaauucacgg..................................................   2      0    TE1
.................gcacuggaagaauucacggg.................................................   1      0    TE1

............Caauggcacuggaagaauucacg...................................................   1      1    MF2
............Caauggcacuggaagaauucacgg..................................................   27     1    MF2
............uaauggcacuggaagaauucacgg..................................................   1      0    MF2
............uaauggcacuggaagaauucacggg.................................................   4      0    MF2
............Caauggcacuggaagaauucacggg.................................................   55     1    MF2
............uaauggcacuggaagaauucacggga................................................   2      0    MF2
............Caauggcacuggaagaauucacggga................................................   7      1    MF2
............Caauggcacuggaagaauucacgggau...............................................   1      1    MF2
............uaauggcacuggaagaauucacgggauuuuuu..........................................   1      0    MF2
.............aauggcacuggaagaau........................................................   1      0    MF2
.............aauggcacuggaagaauu.......................................................   4      0    MF2
.............aauggcacuggaagaauuc......................................................   4      0    MF2
.............aGuggcacuggaagaauuca.....................................................   1      1    MF2
.............aauggUacuggaagaauuca.....................................................   1      1    MF2
.............aauggcGcuggaagaauuca.....................................................   1      1    MF2
.............aauggcacuggaagaauuca.....................................................   128    0    MF2
.............aauggcacugAaagaauuca.....................................................   1      1    MF2
.............aauggcacuggaagaauucaA....................................................   8      1    MF2
.............aauggcaUuggaagaauucac....................................................   1      1    MF2
.............aauggcacuggUagaauucac....................................................   2      1    MF2
```

```
             aga-miR-263a
                                                     aga-miR-263a*
cccugguacauguaauggcacuggaagaauucacgggauuuuuuucaacauucccguguucucuuaguggcauaccuaguacaggg

.............aauggcacCggaagaauucac....................................................   1      1   MF2
.............aauggcacuggGagaauucac....................................................   1      1   MF2
.............aauggcacuggaagaauCcac....................................................   1      1   MF2
.............aauggcacuggaagaaCucac....................................................   2      1   MF2
.............Uauggcacuggaagaauucac....................................................   1      1   MF2
.............aaCggcacuggaagaauucac....................................................   2      1   MF2
.............aauggcacuggaagaauucaU....................................................   1      1   MF2
.............aauggUacuggaagaauucac....................................................   2      1   MF2
.............aauggcacugUaagaauucac....................................................   2      1   MF2
.............aauggcacuAgaagaauucac....................................................   2      1   MF2
.............aauggcacuggaagaauucac....................................................   2339   0   MF2
.............aauAgcacuggaagaauucac....................................................   2      1   MF2
.............aauggcacuggaagaauAcac....................................................   2      1   MF2
.............aUuggcacuggaagaauucac....................................................   1      1   MF2
.............aaugAcacuggaagaauucac....................................................   4      1   MF2
.............aauggcacuggCagaauucac....................................................   1      1   MF2
.............aauggcacuggaagaauuUac....................................................   3      1   MF2
.............aauggcacAggaagaauucac....................................................   1      1   MF2
.............aauggcacugAaagaauucac....................................................   1      1   MF2
.............aaugUcacuggaagaauucac....................................................   1      1   MF2
.............aauggcacuggUagaauucacg...................................................   1      1   MF2
.............aauggcacugCaagaauucacg...................................................   1      1   MF2
.............Uauggcacuggaagaauucacg...................................................   1      1   MF2
.............aauggcGcuggaagaauucacg...................................................   3      1   MF2
.............aaCggcacuggaagaauucacg...................................................   4      1   MF2
.............aauggcacuggaagaauucacU...................................................   17     1   MF2
.............aauggcaGuggaagaauucacg...................................................   1      1   MF2
.............aaugUcacuggaagaauucacg...................................................   2      1   MF2
.............aauggcacuggaagaauuAacg...................................................   2      1   MF2
.............aauggcacugUaagaauucacg...................................................   5      1   MF2
.............aauggcacCggaagaauucacg...................................................   2      1   MF2
.............aauggcacuggaagaauucacA...................................................   31     1   MF2
.............aaugAcacuggaagaauucacg...................................................   1      1   MF2
.............aauggAacuggaagaauucacg...................................................   1      1   MF2
.............aauggcacuggaagGauucacg...................................................   3      1   MF2
.............aauggcacugAaagaauucacg...................................................   5      1   MF2
.............aauggcacuggCagaauucacg...................................................   3      1   MF2
.............aauggcacuUgaagaauucacg...................................................   3      1   MF2
.............aauggcacuggaaUaauucacg...................................................   1      1   MF2
.............aauggcacuggGagaauucacg...................................................   3      1   MF2
.............aauggcacuggaaAaauucacg...................................................   1      1   MF2
.............aauAgcacuggaagaauucacg...................................................   10     1   MF2
.............aauggcCcuggaagaauucacg...................................................   1      1   MF2
.............aauggcacuggaagaauucacg...................................................   6482   0   MF2
.............aauggUacuggaagaauucacg...................................................   3      1   MF2
.............aauggcacuggaagaauuGacg...................................................   1      1   MF2
.............aauggcacuggaagaauAcacg...................................................   2      1   MF2
.............aauggcacuggaagaauuUacg...................................................   9      1   MF2
.............aauggcacuggaagaaAucacg...................................................   2      1   MF2
.............aauggcacuggaagaauCcacg...................................................   3      1   MF2
.............aauggcacuggaagaauucacC...................................................   3      1   MF2
.............aauggcacuAgaagaauucacg...................................................   1      1   MF2
.............aauggcacuggaagaaCucacg...................................................   5      1   MF2
.............aauggcacAggaagaauucacg...................................................   1      1   MF2
.............aaugCcacuggaagaauucacgg..................................................   4      1   MF2
.............aauggcacuCgaagaauucacgg..................................................   4      1   MF2
.............aauggcGcuggaagaauucacgg..................................................   6      1   MF2
.............Gauggcacuggaagaauucacgg..................................................   7      1   MF2
.............aauggcacuggaagaGuucacgg..................................................   9      1   MF2
.............aaGggcacuggaagaauucacgg..................................................   2      1   MF2
.............aauggcacuggaagaaCucacgg..................................................   16     1   MF2
.............Cauggcacuggaagaauucacgg..................................................   2      1   MF2
.............aauggcacuUgaagaauucacgg..................................................   14     1   MF2
.............aCuggcacuggaagaauucacgg..................................................   1      1   MF2
.............aauggcacuggaagaauucacUg..................................................   7      1   MF2
.............aauggGacuggaagaauucacgg..................................................   3      1   MF2
.............aauggcacuggGagaauucacgg..................................................   25     1   MF2
.............aaugAcacuggaagaauucacgg..................................................   22     1   MF2
.............aGuggcacuggaagaauucacgg..................................................   4      1   MF2
```

```
             aga-miR-263a
                                                       aga-miR-263a*
cccugguacauguaauggcacuggaagaauucacgggauuuuuuucaacauucccguguucucuuaguggcauaccuaguacaggg

.............aauggcacuggaaCaauucacgg..................................................	2	1	MF2
.............aauggcacugUaagaauucacgg..................................................	15	1	MF2
.............aauCgcacuggaagaauucacgg..................................................	1	1	MF2
.............aauggcacuggaagaauucacAg..................................................	29	1	MF2
.............aauUgcacuggaagaauucacgg..................................................	2	1	MF2
.............aauggcacuggaagaaAucacgg..................................................	4	1	MF2
.............aauggcacuggaagaauucGcgg..................................................	3	1	MF2
.............aauggcaAuggaagaauucacgg..................................................	2	1	MF2
.............aauggcacuggaagaauucacgC..................................................	8	1	MF2
.............aauggcUcuggaagaauucacgg..................................................	7	1	MF2
.............aaCggcacuggaagaauucacgg..................................................	9	1	MF2
.............aauggcacuggaagaauucaUgg..................................................	24	1	MF2
.............aauggcacuggaagaauucacCg..................................................	13	1	MF2
.............aauggcaUuggaagaauucacgg..................................................	9	1	MF2
.............aauggcacuggaagaUuucacgg..................................................	2	1	MF2
.............aauggcacAggaagaauucacgg..................................................	9	1	MF2
.............aaAggcacuggaagaauucacgg..................................................	7	1	MF2
.............aauggcacuggaagaaGucacgg..................................................	1	1	MF2
.............aauggcacuggaagaauuUacgg..................................................	37	1	MF2
.............aauggcacuggaagaauuGacgg..................................................	4	1	MF2
.............aauggcacuggaagaauucacgA..................................................	65	1	MF2
.............aauggcacuggaagaauAcacgg..................................................	2	1	MF2
.............aauggUacuggaagaauucacgg..................................................	13	1	MF2
.............aauggcacugCaagaauucacgg..................................................	9	1	MF2
.............aUuggcacuggaagaauucacgg..................................................	3	1	MF2
.............aauggcacuggaagGauucacgg..................................................	4	1	MF2
.............aauggcacuggaagaauucacgU..................................................	10	1	MF2
.............aauAgcacuggaagaauucacgg..................................................	34	1	MF2
.............aauggcacuggaUgaauucacgg..................................................	3	1	MF2
.............aauggcacuggaaAaauucacgg..................................................	6	1	MF2
.............aauggcacuggaagaauCcacgg..................................................	11	1	MF2
.............Uauggcacuggaagaauucacgg..................................................	6	1	MF2
.............aauggcacuggaagaauucUcgg..................................................	1	1	MF2
.............aauggcaGuggaagaauucacgg..................................................	2	1	MF2
.............aauggcacCggaagaauucacgg..................................................	17	1	MF2
.............aauggAacuggaagaauucacgg..................................................	3	1	MF2
.............aauggcacuggaagaauucaAgg..................................................	3	1	MF2
.............aaugUcacuggaagaauucacgg..................................................	1	1	MF2
.............aauggcacuAgaagaauucacgg..................................................	20	1	MF2
.............aauggcacuggaagaauucCcgg..................................................	3	1	MF2
.............aauggcacugAaagaauucacgg..................................................	47	1	MF2
.............aauggcacuggaagaauucacgg..................................................	45717	0	MF2
.............aauggcacGggaagaauucacgg..................................................	4	1	MF2
.............aauggcacuggaagaauucaGgg..................................................	2	1	MF2
.............aauggcacuggaagaauuAacgg..................................................	8	1	MF2
.............aauggcacuggCagaauucacgg..................................................	13	1	MF2
.............aauggcacuggUagaauucacgg..................................................	12	1	MF2
.............aauggcacuggaGgaauucacgg..................................................	3	1	MF2
.............aauggcacuggaagaaCucacggg.................................................	49	1	MF2
.............aauggcacuggaagaauucacgAg.................................................	40	1	MF2
.............aaugUcacuggaagaauucacggg.................................................	9	1	MF2
.............aauggcacuggaagaauucaUggg.................................................	50	1	MF2
.............aauggcacuggaagaauucacCgg.................................................	30	1	MF2
.............aauCgcacuggaagaauucacggg.................................................	4	1	MF2
.............aauggcacuggaagaauAcacggg.................................................	11	1	MF2
.............aauggcacuggaagaauucUcggg.................................................	2	1	MF2
.............aauggcacCggaagaauucacggg.................................................	38	1	MF2
.............aaAggcacuggaagaauucacggg.................................................	13	1	MF2
.............aauggcacuggaagaaAucacggg.................................................	12	1	MF2
.............aaugCcacuggaagaauucacggg.................................................	6	1	MF2
.............aauggcacAggaagaauucacggg.................................................	6	1	MF2
.............aauggcacuggaagaauuGacggg.................................................	6	1	MF2
.............aauggcacuggaagaCuucacggg.................................................	5	1	MF2
.............aauggcacuggaagaaGucacggg.................................................	6	1	MF2
.............aauggcacuggaagaauCcacggg.................................................	30	1	MF2
.............aauggGacuggaagaauucacggg.................................................	6	1	MF2
.............aauggcacuggCagaauucacggg.................................................	46	1	MF2
.............aauggcacGggaagaauucacggg.................................................	11	1	MF2
.............aauggcacuggaagaUuucacggg.................................................	6	1	MF2
```

```
             aga-miR-263a
                                                     aga-miR-263a*
cccugguacauguaauggcacuggaagaauucacgggauuuuuuucaacauucccguguucucuuaguggcauaccuaguacaggg

.............aauAgcacuggaagaauucacggg.................................................    74       1    MF2
.............aauUgcacuggaagaauucacggg.................................................    5        1    MF2
.............aauggcacuUgaagaauucacggg.................................................    13       1    MF2
.............aauggcacuggaagaauucaGggg.................................................    2        1    MF2
.............aUuggcacuggaagaauucacggg.................................................    8        1    MF2
.............aauggcacugAaagaauucacggg.................................................    108      1    MF2
.............aauggcacuggaagaauucacgCg.................................................    5        1    MF2
.............aauggcacuggaagaauucacUgg.................................................    15       1    MF2
.............aauggcacuggaagaGuucacggg.................................................    8        1    MF2
.............aauggcacuggaagaauuAacggg.................................................    10       1    MF2
.............aauggUacuggaagaauucacggg.................................................    21       1    MF2
.............aauggcacuggaagaauucacggg.................................................    104490   0    MF2
.............aauggcacuAgaagaauucacggg.................................................    39       1    MF2
.............aauggcacuCgaagaauucacggg.................................................    13       1    MF2
.............aauggcacuggaUgaauucacggg.................................................    7        1    MF2
.............aauggcaGuggaagaauucacggg.................................................    4        1    MF2
.............aCuggcacuggaagaauucacggg.................................................    2        1    MF2
.............aauggcacuggaaUaauucacggg.................................................    4        1    MF2
.............aauggcacuggaagaauucacggU.................................................    64       1    MF2
.............aauggcCcuggaagaauucacggg.................................................    2        1    MF2
.............aauggcacuggaagaauucCcggg.................................................    4        1    MF2
.............aauggcacuggaagaauucGcggg.................................................    17       1    MF2
.............aauggcaAuggaagaauucacggg.................................................    6        1    MF2
.............aauggcacuggaagaauucacgUg.................................................    4        1    MF2
.............aauggAacuggaagaauucacggg.................................................    15       1    MF2
.............aauggcacuggGagaauucacggg.................................................    39       1    MF2
.............aaugAcacuggaagaauucacggg.................................................    41       1    MF2
.............aGuggcacuggaagaauucacggg.................................................    8        1    MF2
.............aauggcGcuggaagaauucacggg.................................................    8        1    MF2
.............aaGggcacuggaagaauucacggg.................................................    5        1    MF2
.............Gauggcacuggaagaauucacggg.................................................    16       1    MF2
.............aauggcacuggaagaauucacggA.................................................    190      1    MF2
.............aauggcacuggaagaauGcacggg.................................................    1        1    MF2
.............aauggcacugCaagaauucacggg.................................................    20       1    MF2
.............aauggcacuggaaCaauucacggg.................................................    5        1    MF2
.............aauggcacuggaagCauucacggg.................................................    1        1    MF2
.............Uauggcacuggaagaauucacggg.................................................    11       1    MF2
.............aauggcUcuggaagaauucacggg.................................................    5        1    MF2
.............aauggcacuggaagaauucaAggg.................................................    6        1    MF2
.............aauggcacuggaagUauucacggg.................................................    4        1    MF2
.............aauggcacuggaagaauucacAgg.................................................    55       1    MF2
.............aauggcacugUaagaauucacggg.................................................    45       1    MF2
.............aauggcacuggaaAaauucacggg.................................................    11       1    MF2
.............aauggcacuggaagGauucacggg.................................................    6        1    MF2
.............aaCggcacuggaagaauucacggg.................................................    28       1    MF2
.............aauggcaUuggaagaauucacggg.................................................    22       1    MF2
.............aauggcacuggUagaauucacggg.................................................    33       1    MF2
.............aauggcacuggaGgaauucacggg.................................................    13       1    MF2
.............aauggcacuggaagaauUacggg..................................................    77       1    MF2
.............Cauggcacuggaagaauucacggg.................................................    4        1    MF2
.............aauggcacuggaagaauucacggC.................................................    64       1    MF2
.............Cauggcacuggaagaauucacggga................................................    1        1    MF2
.............aauggcacuggaaCaauucacggga................................................    1        1    MF2
.............aauggcacuggaagaauucacgUga................................................    1        1    MF2
.............aauggcacuggCagaauucacggga................................................    8        1    MF2
.............aauCgcacuggaagaauucacggga................................................    2        1    MF2
.............aauggcacCggaagaauucacggga................................................    4        1    MF2
.............aaugAcacuggaagaauucacggga................................................    10       1    MF2
.............aauggGacuggaagaauucacggga................................................    3        1    MF2
.............aUuggcacuggaagaauucacggga................................................    1        1    MF2
.............aauggUacuggaagaauucacggga................................................    7        1    MF2
.............aauggcacuggaagaCuucacggga................................................    1        1    MF2
.............aauggcacuggaagaauucaGggga................................................    3        1    MF2
.............aauggcacAggaagaauucacggga................................................    2        1    MF2
.............aGuggcacuggaagaauucacggga................................................    3        1    MF2
.............aauggcacuggaagaauucacgggC................................................    113      1    MF2
.............aauggcacugUaagaauucacggga................................................    4        1    MF2
.............aaugUcacuggaagaauucacggga................................................    3        1    MF2
.............aauggcacuggaagaauCcacggga................................................    9        1    MF2
```

```
             aga-miR-263a
                                                      aga-miR-263a*
cccugguacauguaauggcacuggaagaauucacgggauuuuuuucaacauucccguguucucuuaguggcauaccuaguacaggg

.............aaCggcacuggaagaauucacggga................................................    6          1          MF2
.............aauggcacuggaagaauucacCgga................................................    1          1          MF2
.............aauggcacuggaagaauucacgggU................................................    1616       1          MF2
.............aauggcacuggaagaaCucacggga................................................    11         1          MF2
.............aauggcGcuggaagaauucacggga................................................    3          1          MF2
.............aauggAacuggaagaauucacggga................................................    5          1          MF2
.............aauggcacuggaagaGuucacggga................................................    1          1          MF2
.............aauggcacuggaagaauuGacggga................................................    2          1          MF2
.............aauggcaAuggaagaauucacggga................................................    1          1          MF2
.............aauggcacuggaaAaauucacggga................................................    3          1          MF2
.............aauggcacuggaagaauucaUggga................................................    3          1          MF2
.............aauggcacugCaagaauucacggga................................................    6          1          MF2
.............aauggcacuggaagaauucacAgga................................................    7          1          MF2
.............aauggcacuggaGgaauucacggga................................................    2          1          MF2
.............aauggcacuggaagaauucacggAa................................................    98         1          MF2
.............aaAggcacuggaagaauucacggga................................................    1          1          MF2
.............aauggcacuggaagaauucacggga................................................    17502      0          MF2
.............aauggcacuggaCgaauucacggga................................................    2          1          MF2
.............aauggcacuCgaagaauucacggga................................................    3          1          MF2
.............aauggcacuggGagaauucacggga................................................    7          1          MF2
.............aauggcacugAaagaauucacggga................................................    16         1          MF2
.............aaGggcacuggaagaauucacggga................................................    1          1          MF2
.............aauggcacuggaagGauucacggga................................................    2          1          MF2
.............aauAgcacuggaagaauucacggga................................................    11         1          MF2
.............aauggcacuggaagaauucGcggga................................................    3          1          MF2
.............aauggcacuggaagaauuUacggga................................................    12         1          MF2
.............aauggcacuUgaagaauucacggga................................................    5          1          MF2
.............aauggcacuggaagaauucacgggG................................................    96         1          MF2
.............aauggcacuggaUgaauucacggga................................................    3          1          MF2
.............aauggcacuggaagaauucacUgga................................................    3          1          MF2
.............aauggcUcuggaagaauucacggga................................................    1          1          MF2
.............aauggcacuggaagaauucaAggga................................................    4          1          MF2
.............aauggcacuggaagaaAucacggga................................................    4          1          MF2
.............Uauggcacuggaagaauucacggga................................................    1          1          MF2
.............aauggcacuggUagaauucacggga................................................    3          1          MF2
.............aauggcacuggaagaauuAacggga................................................    3          1          MF2
.............aauggcacuggaagaauucacggUa................................................    6          1          MF2
.............aauggcacuAgaagaauucacggga................................................    7          1          MF2
.............aauggcacuggaagaauucacggCa................................................    6          1          MF2
.............Gauggcacuggaagaauucacggga................................................    2          1          MF2
.............aauggcacuggaagaauucacgggGu...............................................    1          1          MF2
.............aauggcacuggaagaauuUacgggau...............................................    2          1          MF2
.............aauggUacuggaagaauucacgggau...............................................    1          1          MF2
.............aauggcacuggaagaaCucacgggau...............................................    1          1          MF2
.............aaugAcacuggaagaauucacgggau...............................................    1          1          MF2
.............aauggcacuggaagaauucacggAau...............................................    6          1          MF2
.............aauggcacCggaagaauucacgggau...............................................    3          1          MF2
.............aauggcacuggaagaauCcacgggau...............................................    2          1          MF2
.............aauggcacuggaagaauuGacgggau...............................................    2          1          MF2
.............aauggcacuggaagaauucacgggUu...............................................    171        1          MF2
.............aauggcacuggaagaauucacgggaG...............................................    9          1          MF2
.............aauggcacugUaagaauucacgggau...............................................    1          1          MF2
.............aauggcacuggaagaauucacgggau...............................................    1924       0          MF2
.............aauggcacuggaagaauucacgggaA...............................................    2424       1          MF2
.............aauggcacuggaagaauucacgggaC...............................................    22         1          MF2
.............aauggcacuggCagaauucacgggau...............................................    1          1          MF2
.............aauggcacugAaagaauucacgggau...............................................    2          1          MF2
.............aauggcacuggaagaauucacggUau...............................................    7          1          MF2
.............aauggcacuAgaagaauucacgggau...............................................    1          1          MF2
.............Uauggcacuggaagaauucacgggau...............................................    1          1          MF2
.............aauggcacuggaagaauucacUggau...............................................    1          1          MF2
.............aauggcacuggGagaauucacgggau...............................................    1          1          MF2
.............aauggcacuggaagaauucacgggCu...............................................    27         1          MF2
.............aauggcacuggaagaauucacgggauu..............................................    127        0          MF2
.............aauggcacuggaagaauucacgggauC..............................................    4          1          MF2
.............aauggcacuggaagaauucacgggauA..............................................    33         1          MF2
.............aauggcacuggaagaauucacgggaAu..............................................    520        1          MF2
.............aauggcacuggaagaauucCcgggauu..............................................    1          1          MF2
.............aauAgcacuggaagaauucacgggauu..............................................    1          1          MF2
```

```
             aga-miR-263a
                                                     aga-miR-263a*
cccugguacauguaauggcacuggaagaauucacgggauuuuuuucaacauucccguguucucuuaguggcauaccuaguacaggg

.............aauggcacuggaagaauCcacgggauu..............................................    1     1     MF2
.............aauggcacuggaagaauucacgggaCu...............................................   12    1     MF2
.............aauggcacuggaagaauucacgggUuu..............................................    96    1     MF2
.............aauggcacugAaagaauucacgggauu..............................................    1     1     MF2
.............aauggcacuggaagaauucacgggCuu..............................................    3     1     MF2
.............aauggcacuggaagaauucacgggaGuu.............................................    2     1     MF2
.............aauggcacuggaagaauucacgggauuu.............................................    82    0     MF2
.............aauggcacuggaagaauucacgggauAu.............................................    71    1     MF2
.............aauggcacuggaagaauucacgggUuuu.............................................    23    1     MF2
.............aauggcacuggaagaauucacgggCuuu.............................................    1     1     MF2
.............aauggcacuggaagaauucacgggaCuu.............................................    5     1     MF2
.............aauggcacuggaagaauucacgggauuA.............................................    6     1     MF2
.............aauggcacuggaagaauucacgggaAuu.............................................    37    1     MF2
.............aauggcacuggaagaauucacgggauuG.............................................    3     1     MF2
.............aauggcacuggaagaauucacgggauCu.............................................    7     1     MF2
.............aauggcacuggaagaauucacgggaCuuu............................................    1     1     MF2
.............aauggcacuggaagaauucacgggauuAu............................................    29    1     MF2
.............aauggcacuggaagaauCcacgggauuuu............................................    1     1     MF2
.............aauggcacuggaagaauucacgggauuuA............................................    8     1     MF2
.............aauggcacuggaagaauucacgggaAuuu............................................    47    1     MF2
.............aauggcacuggaagaauucacgggauuCu............................................    3     1     MF2
.............aauggcacuggaagaauucacgggauuuu............................................    71    0     MF2
.............aauggcacuggaagaauucacgggauAuu............................................    10    1     MF2
.............aauggcacuggaagaauucacgggauuuC............................................    1     1     MF2
.............aauggcacuggaagaauucacgggauuuG............................................    2     1     MF2
.............aauggcacuggaagaauucacgggUuuuu............................................    10    1     MF2
.............aaugAcacuggaagaauucacgggauuuu............................................    1     1     MF2
.............aauggcacuggaagaauucacgggauuuuu...........................................    20    0     MF2
.............aauggcacuggaagaauucacgggaCuuuu...........................................    1     1     MF2
.............aauggcacuggaagaauucacgggUuuuuu...........................................    5     1     MF2
.............aauggcacuggaagaauucacgggauAuuu...........................................    2     1     MF2
.............aauggcacuggaagaauucacgggauuuAu...........................................    10    1     MF2
.............aauggcacuggaagaauucacgggauuuuA...........................................    1     1     MF2
.............aauggcacuggaagaauucacgggauCuuu...........................................    1     1     MF2
.............aauggcacuggaagaauucacgggauuGuu...........................................    1     1     MF2
.............aauggcacuggaagaauucacgggaAuuuu...........................................    10    1     MF2
.............aauggcacuggaagaauucacgggauuuCu...........................................    2     1     MF2
.............aauggcacuggaagaauucacgggauuAuu...........................................    5     1     MF2
.............aauggcacuggaagaauucacgggauuuuuA..........................................    2     1     MF2
.............aauggcacuggaagaauucacgggauuuCuu..........................................    1     1     MF2
.............aauggcacuggaagaauucacgggauuuuAu..........................................    2     1     MF2
.............aauggcacuggaagaauucacgggauuAuuu..........................................    2     1     MF2
.............aauggcacuggaagaauucacgggaAuuuuu..........................................    7     1     MF2
.............aauggcacuggaagaauucacgggauuuuuu..........................................    1     0     MF2
.............aauggcacuggaagaauucacgggUuuuuuu..........................................    2     1     MF2
.............aauggcacuggaagaauucacgggauuuuuuu.........................................    2     0     MF2
.............aauggcacuggaagaauucacgggaAuuuuuu.........................................    1     1     MF2
.............aauggcacuggaagaauucacgggauuuuuuucaaca....................................    2     0     MF2
.............aauggcacuggaagaauucacgggauuuuuuucaacau...................................    1     0     MF2
..............auggcacuggaagaauucacg...................................................    1     0     MF2
..............auggcacuggaagaauucacgg..................................................    2     0     MF2
..............auggcacuggaagaauucaUggg.................................................    1     1     MF2
..............auggcacuggaagaauucacggg.................................................    14    0     MF2
..............auggcacuggaagaauucacggga................................................    20    0     MF2
..............auggcacuggaagaauucacgggau...............................................    3     0     MF2
..............auggcacuggaagaauucacgggaA...............................................    1     1     MF2
..............auggcacuggaagaauucacgggauuu.............................................    1     0     MF2
...............uggcacuggaagaauucacggg.................................................    1     0     MF2
...............uggcacuggaagaauucacgggau...............................................    1     0     MF2
.................gcacuggaagaauucacggg.................................................    1     0     MF2
..................cacuggaagaauucacg...................................................    1     0     MF2
..................cacuggaagaauucacgg..................................................    2     0     MF2
..................cacuggaagaauucacggg.................................................    1     0     MF2
....................cuggaagaauucacggg.................................................    1     0     MF2
....................cuggaagaauucacggC.................................................    1     1     MF2
....................cuggaagaauucacggga................................................    1     0     MF2
.....................................................ccguguucucuuaguggcauacc..........    2     0     MF2
......................................................cguguucucuuaguAgcauacc..........    1     1     MF2
......................................................cguguucucuuaguggcauacc..........    92    0     MF2
```

```
               aga-miR-263a
                                                      aga-miR-263a*
cccugguacauguaauggcacuggaagaauucacgggauuuuuuucaacauucccguguucucuuaguggcauaccuaguacaggg

.......................................................guguucucuuaguggcauacc..........    1        0        MF2

............Caauggcacuggaagaauucac....................................................    2        1        FW2
............Caauggcacuggaagaauucacgg..................................................    1        1        FW2
............Caauggcacuggaagaauucacggg.................................................    17       1        FW2
............Caauggcacuggaagaauucacggga................................................    1        1        FW2
.............aauggcacuggaagaau........................................................    6        0        FW2
.............aauggcacuggaagaauu.......................................................    7        0        FW2
.............aauggcacuggaagaauuc......................................................    2        0        FW2
.............aauggcaUuggaagaauuca.....................................................    1        1        FW2
.............aauggcacuggaagaauuca.....................................................    38       0        FW2
.............aauggcacugAaagaauucac....................................................    2        1        FW2
.............aauggcacuAgaagaauucac....................................................    1        1        FW2
.............aauggcacuggaagaauucac....................................................    429      0        FW2
.............aauggcacuggaGgaauucac....................................................    1        1        FW2
.............aauggcacuggaagaaAucac....................................................    1        1        FW2
.............aauggcacuggaagaauucUc....................................................    1        1        FW2
.............aauggcacuggaagaauCcac....................................................    1        1        FW2
.............aauggcacuggaagaauucacU...................................................    1        1        FW2
.............aauggcacuggaagaauCcacg...................................................    1        1        FW2
.............aauggcacuggGagaauucacg...................................................    1        1        FW2
.............aauggcacuggaagaauucacg...................................................    1310     0        FW2
.............aauggcacugUaagaauucacg...................................................    2        1        FW2
.............aaAggcacuggaagaauucacg...................................................    1        1        FW2
.............aauggcacCggaagaauucacg...................................................    1        1        FW2
.............aaCggcacuggaagaauucacg...................................................    1        1        FW2
.............aauggcacuggaagaauucacA...................................................    2        1        FW2
.............Gauggcacuggaagaauucacg...................................................    2        1        FW2
.............aauggcaUuggaagaauucacgg..................................................    2        1        FW2
.............aauggcacugAaagaauucacgg..................................................    21       1        FW2
.............aauggcacuUgaagaauucacgg..................................................    3        1        FW2
.............aauggcacuggaagaauucacAg..................................................    4        1        FW2
.............aauggcacuggaagaGuucacgg..................................................    2        1        FW2
.............aaGggcacuggaagaauucacgg..................................................    2        1        FW2
.............aauggcGcuggaagaauucacgg..................................................    2        1        FW2
.............aauggcacuCgaagaauucacgg..................................................    1        1        FW2
.............aauggcacuggaagaauuUacgg..................................................    7        1        FW2
.............aaugAcacuggaagaauucacgg..................................................    1        1        FW2
.............aauggcUcuggaagaauucacgg..................................................    1        1        FW2
.............Cauggcacuggaagaauucacgg..................................................    1        1        FW2
.............aauggcacuggaagaauucacgC..................................................    2        1        FW2
.............aauggcaGuggaagaauucacgg..................................................    1        1        FW2
.............Gauggcacuggaagaauucacgg..................................................    6        1        FW2
.............aUuggcacuggaagaauucacgg..................................................    2        1        FW2
.............aauggcacuggCagaauucacgg..................................................    3        1        FW2
.............aauggcacuggaagaauucaAgg..................................................    1        1        FW2
.............aauggcacuggGagaauucacgg..................................................    1        1        FW2
.............aauggcacuAgaagaauucacgg..................................................    4        1        FW2
.............aauggcacuggaagGauucacgg..................................................    2        1        FW2
.............aaCggcacuggaagaauucacgg..................................................    5        1        FW2
.............aauggcacuggaagaCuucacgg..................................................    1        1        FW2
.............aauggAacuggaagaauucacgg..................................................    2        1        FW2
.............aauggcacuggaagaauucacgg..................................................    12718    0        FW2
.............aauggcacuggaagaauucGcgg..................................................    2        1        FW2
.............aauggcacuggaaAaauucacgg..................................................    6        1        FW2
.............aauggcacuggaagaaCucacgg..................................................    6        1        FW2
.............aauggcacugUaagaauucacgg..................................................    5        1        FW2
.............aauggcacuggaagaauAcacgg..................................................    1        1        FW2
.............aauggcacuggaagaauucacgU..................................................    5        1        FW2
.............aauggcacuggaagaauCcacgg..................................................    6        1        FW2
.............aauggcacGggaagaauucacgg..................................................    1        1        FW2
.............aauggcaAuggaagaauucacgg..................................................    1        1        FW2
.............aauAgcacuggaagaauucacgg..................................................    2        1        FW2
.............Uauggcacuggaagaauucacgg..................................................    2        1        FW2
.............aauggcacCggaagaauucacgg..................................................    1        1        FW2
.............aauggcacuggaUgaauucacgg..................................................    1        1        FW2
.............aauggcacugCaagaauucacgg..................................................    2        1        FW2
.............aauggcacuggUagaauucacgg..................................................    10       1        FW2
.............aauggcacuggaagaauucacgA..................................................    10       1        FW2
```

```
             aga-miR-263a
                                                           aga-miR-263a*
cccugguacauguaauggcacuggaagaauucacgggauuuuuuucaacauucccguguucucuuaguggcauaccuaguacaggg

.............aauggUacuggaagaauucacgg..................................................    2       1       FW2
.............aauggcacuggaagaauucaUgg..................................................    3       1       FW2
.............aUuggcacuggaagaauucacggg.................................................    1       1       FW2
.............aauAgcacuggaagaauucacggg.................................................    18      1       FW2
.............aauggcaUuggaagaauucacggg.................................................    5       1       FW2
.............aauggcacAggaagaauucacggg.................................................    4       1       FW2
.............aaAggcacuggaagaauucacggg.................................................    1       1       FW2
.............aauggcacuggGagaauucacggg.................................................    9       1       FW2
.............aauggAacuggaagaauucacggg.................................................    5       1       FW2
.............aauggcacuggaagaauucacggA.................................................    41      1       FW2
.............aaugUcacuggaagaauucacggg.................................................    1       1       FW2
.............aauggcacuggaagaauGcacggg.................................................    1       1       FW2
.............aauggcacuggaagaauucaGggg.................................................    2       1       FW2
.............aauggcacuggaagaauAcacggg.................................................    1       1       FW2
.............aauggcacuggaagaauCcacggg.................................................    7       1       FW2
.............aauggcacuggaagaauuUacggg.................................................    16      1       FW2
.............aauggcacuggaagaauuGacggg.................................................    4       1       FW2
.............aauggcacuggaagaauucUcggg.................................................    2       1       FW2
.............aauggcacugUaagaauucacggg.................................................    16      1       FW2
.............aauggcacuggaGgaauucacggg.................................................    5       1       FW2
.............aauggcacuggaagaauucacggC.................................................    30      1       FW2
.............aauggcacuggaagaauucacgAg.................................................    7       1       FW2
.............Gauggcacuggaagaauucacggg.................................................    2       1       FW2
.............aauggcacuggaaAaauucacggg.................................................    4       1       FW2
.............aauggcacuggaaCaauucacggg.................................................    2       1       FW2
.............aauggcacGggaagaauucacggg.................................................    2       1       FW2
.............aauggcacuggaagaauucacCgg.................................................    2       1       FW2
.............aauggcUcuggaagaauucacggg.................................................    1       1       FW2
.............aauggcacuggaagaauucacgCg.................................................    1       1       FW2
.............aauUgcacuggaagaauucacggg.................................................    1       1       FW2
.............aauggcacuggUagaauucacggg.................................................    7       1       FW2
.............aauggcacuCgaagaauucacggg.................................................    1       1       FW2
.............aauggcacuggaagGauucacggg.................................................    2       1       FW2
.............aauggcacugCaagaauucacggg.................................................    10      1       FW2
.............aauggcacuggaagaauucaAggg.................................................    3       1       FW2
.............aaugAcacuggaagaauucacggg.................................................    7       1       FW2
.............aauggcacuggaagaauucacUgg.................................................    3       1       FW2
.............aauggcacuUgaagaauucacggg.................................................    2       1       FW2
.............aauggcacuggaagaauucacggg.................................................    25818   0       FW2
.............aauggcacugAaagaauucacggg.................................................    23      1       FW2
.............aauggcacuggaagaauucGcggg.................................................    6       1       FW2
.............aauggcacuAgaagaauucacggg.................................................    12      1       FW2
.............aaGggcacuggaagaauucacggg.................................................    3       1       FW2
.............aauggcacuggaagaaCucacggg.................................................    10      1       FW2
.............aauggcacuggaagaGuucacggg.................................................    4       1       FW2
.............aauggcacuggaUgaauucacggg.................................................    1       1       FW2
.............Uauggcacuggaagaauucacggg.................................................    4       1       FW2
.............aauggcacCggaagaauucacggg.................................................    6       1       FW2
.............aauggcaAuggaagaauucacggg.................................................    2       1       FW2
.............Cauggcacuggaagaauucacggg.................................................    2       1       FW2
.............aauggcacuggaagaauuAacggg.................................................    3       1       FW2
.............aCuggcacuggaagaauucacggg.................................................    1       1       FW2
.............aauggUacuggaagaauucacggg.................................................    2       1       FW2
.............aauggcacuggaagaauucacgUg.................................................    3       1       FW2
.............aauggcGcuggaagaauucacggg.................................................    4       1       FW2
.............aaugCcacuggaagaauucacggg.................................................    1       1       FW2
.............aauggcacuggaagaauucacAgg.................................................    3       1       FW2
.............aaCggcacuggaagaauucacggg.................................................    9       1       FW2
.............aauggcacuggaagaauucacggU.................................................    11      1       FW2
.............aauggcacuggaagaaAucacggg.................................................    1       1       FW2
.............aauggcacuggaaUaauucacggg.................................................    2       1       FW2
.............aauggcacuggaagaauucaUggg.................................................    5       1       FW2
.............aauggcacuggCagaauucacggg.................................................    7       1       FW2
.............aauggcacuggaagaauucacgggG................................................    15      1       FW2
.............aauggcacuggaagaauucacgAga................................................    1       1       FW2
.............aauggcacuggCagaauucacggga................................................    1       1       FW2
.............aauggcacugAaagaauucacggga................................................    1       1       FW2
.............Cauggcacuggaagaauucacggga................................................    1       1       FW2
.............aauAgcacuggaagaauucacggga................................................    2       1       FW2
```

```
             aga-miR-263a
                                         aga-miR-263a*
cccugguacauguaauggcacuggaagaauucacgggauuuuuucaacauucccguguucucuuaguggcauaccuaguacaggg

.............aauggcacuggaagaauCcacggga...............................................  1     1   FW2
.............aauggcacuggGagaauucacggga...............................................  6     1   FW2
.............aauggcacugUaagaauucacggga...............................................  2     1   FW2
.............aauggcacCggaagaauucacggga...............................................  1     1   FW2
.............aauggcacuggaagaauucaUggga...............................................  2     1   FW2
.............aauggcacuggaagCauucacggga...............................................  1     1   FW2
.............aauggcacugCaagaauucacggga...............................................  2     1   FW2
.............aauggcacuggaagaauucacggAa...............................................  12    1   FW2
.............aauggcacuggaagGauucacggga...............................................  1     1   FW2
.............Uauggcacuggaagaauucacggga...............................................  1     1   FW2
.............aauggcacuggaagaauucacgggU...............................................  314   1   FW2
.............aaCggcacuggaagaauucacggga...............................................  2     1   FW2
.............aauggcacuggaagaauucacgggC...............................................  30    1   FW2
.............aauggcacuggaagaauucacggga...............................................  2786  0   FW2
.............aauggcacuggaagaauuUacggga...............................................  3     1   FW2
.............aauggcacuggaagaauucacgggau..............................................  89    0   FW2
.............aauggcacuggaagaauucacgggaA..............................................  124   1   FW2
.............aauggcacuggaagaauucacgggUu..............................................  20    1   FW2
.............aauggcacuggaagaauucacgggaG..............................................  2     1   FW2
.............aauggcacuggaagaauucacgggCu..............................................  2     1   FW2
.............aauggcacuUgaagaauucacgggau..............................................  1     1   FW2
.............aauggcacuggaagaauucacAggau..............................................  1     1   FW2
.............aauggcacuggaagaauucacgggauu.............................................  3     0   FW2
.............aauggcacuggaagaauucacgggauA.............................................  1     1   FW2
.............aauggcacuggaagaauucacgggUuu.............................................  6     1   FW2
.............aauggcacuggaagaauucacgggaCu.............................................  1     1   FW2
.............aauggcacuggaagaauucacgggaAu.............................................  18    1   FW2
.............aauggcacuggaagaauucacgggUuuu............................................  1     1   FW2
.............aauggcacuggaagaauucacgggauAu............................................  2     1   FW2
.............aauggcacuggaagaauucacgggauuu............................................  4     0   FW2
.............aauggcacuggaagaauucacgggaAuuu...........................................  1     1   FW2
.............aauggcacuggaagaauucacgggauAuu...........................................  1     1   FW2
.............aauggcacuggaagaauucacgggauuAu...........................................  1     1   FW2
.............aauggcacuggaagaauucacgggauuuu...........................................  1     0   FW2
..............auggcacuggaagaauucac...................................................  1     0   FW2
..............auggcacuggaagaauucacgg.................................................  3     0   FW2
..............auggcacuggaagaauucacggg................................................  10    0   FW2
..............auggcacuggaagaauucacggga...............................................  4     0   FW2
..............auggcacuggaagaauucacgggau..............................................  1     0   FW2
...............uggcacuggaagaauucacggg................................................  1     0   FW2
...............uggcacuggaagaauucacggga...............................................  1     0   FW2
...............uggcacuggaagaauucacgggau..............................................  1     0   FW2
..................cacuggaagaauucacgg.................................................  2     0   FW2
....................cuggaagaauucacggg................................................  5     0   FW2
....................................................ccguguucucuuaguggcauac...........  3     0   FW2
....................................................ccguguucucuuaguggcauacc..........  3     0   FW2
.....................................................cguguucucuuaguggcauacc..........  204   0   FW2
.....................................................cguguucucuAaguggcauacc..........  1     1   FW2
.....................................................cguguucucuuaguggcauaccu.........  1     0   FW2

............Caauggcacuggaagaauucac...................................................  1     1   OV1
............Caauggcacuggaagaauucacg..................................................  1     1   OV1
............uaauggcacuggaagaauucacgg.................................................  1     0   OV1
............Caauggcacuggaagaauucacgg.................................................  26    1   OV1
............Gaauggcacuggaagaauucacgg.................................................  1     1   OV1
............uaauggcacuggaagaauucaGggg................................................  1     1   OV1
............Caauggcacuggaagaauucacggg................................................  32    1   OV1
............Gaauggcacuggaagaauucacggg................................................  1     1   OV1
............Caauggcacuggaagaauucacggga...............................................  10    1   OV1
.............aauggcacuggaagaau.......................................................  7     0   OV1
.............aauggcacuggaagaauu......................................................  9     0   OV1
.............aauggcacuggaagaauuc.....................................................  3     0   OV1
.............aauggcacuggaagaauuca....................................................  73    0   OV1
.............aauggcacuAgaagaauucac...................................................  2     1   OV1
.............aauggcacuggaagaauucGc...................................................  1     1   OV1
.............aauggUacuggaagaauucac...................................................  1     1   OV1
.............aauggcacuggaagaauucac...................................................  537   0   OV1
.............aauggcacuggGagaauucac...................................................  1     1   OV1
.............aaCggcacuggaagaauucac...................................................  1     1   OV1
```

aga-miR-263a

aga-miR-263a*

```
cccugguacauguaauggcacuggaagaauucacgggauuuuuuucaacauucccguguucucuuaguggcauaccuaguacaggg

.............aauggcacuggaagaauucaA....................................................  2      1  OV1
.............aauggcacuggaaCaauucacg...................................................  1      1  OV1
.............aauggcacuggaagaaCucacg...................................................  5      1  OV1
.............aauggcacuggGagaauucacg...................................................  1      1  OV1
.............aaCggcacuggaagaauucacg...................................................  2      1  OV1
.............aauggcacuggaagaauucacg...................................................  3079   0  OV1
.............aauggcacuggaagaauucaAg...................................................  1      1  OV1
.............aauggcacuggUagaauucacg...................................................  1      1  OV1
.............aauggcacuggaagaCuucacg...................................................  1      1  OV1
.............aauggcacuggaagaauucacA...................................................  11     1  OV1
.............aauggcacuggCagaauucacg...................................................  3      1  OV1
.............aauggcUcuggaagaauucacg...................................................  1      1  OV1
.............aauggcaUuggaagaauucacg...................................................  1      1  OV1
.............aaugCcacuggaagaauucacg...................................................  1      1  OV1
.............aaugUcacuggaagaauucacg...................................................  2      1  OV1
.............aauggGacuggaagaauucacg...................................................  1      1  OV1
.............aauAgcacuggaagaauucacg...................................................  1      1  OV1
.............aauggcacuggaagaauucacU...................................................  1      1  OV1
.............aauggcacuggaagaauucaUg...................................................  1      1  OV1
.............aauggcacCggaagaauucacg...................................................  1      1  OV1
.............aaAggcacuggaagaauucacg...................................................  1      1  OV1
.............aauggcacuggaagaauuAacg...................................................  1      1  OV1
.............aauggcacugAaagaauucacg...................................................  3      1  OV1
.............aauggcacuggaagaauuGacg...................................................  1      1  OV1
.............aauggcaAuggaagaauucacg...................................................  1      1  OV1
.............aauggcacuggaagaauuUacg...................................................  4      1  OV1
.............aauggcacuAgaagaauucacg...................................................  2      1  OV1
.............aauggUacuggaagaauucacg...................................................  1      1  OV1
.............aauggcacuggaaAaauucacg...................................................  1      1  OV1
.............aauggcacuggUagaauucacgg..................................................  13     1  OV1
.............aauggcacugAaagaauucacgg..................................................  42     1  OV1
.............aauggcacuggaagaauucacAg..................................................  24     1  OV1
.............aauggcacuggaUgaauucacgg..................................................  1      1  OV1
.............aauggcacuggaagaGuucacgg..................................................  6      1  OV1
.............aauggcGcuggaagaauucacgg..................................................  8      1  OV1
.............aauggcacuUgaagaauucacgg..................................................  10     1  OV1
.............aaGggcacuggaagaauucacgg..................................................  3      1  OV1
.............aauggcacuggaagaauucacgg..................................................  55531  0  OV1
.............aauggcacuggaagaauucacCg..................................................  7      1  OV1
.............aauggcacuggaagaauucUcgg..................................................  4      1  OV1
.............aauggcaUuggaagaauucacgg..................................................  11     1  OV1
.............aauggcacuggGagaauucacgg..................................................  17     1  OV1
.............aauggcacuggCagaauucacgg..................................................  24     1  OV1
.............aauggcCcuggaagaauucacgg..................................................  3      1  OV1
.............aauggcacuggaagaauucacgU..................................................  7      1  OV1
.............aauggcacuAgaagaauucacgg..................................................  25     1  OV1
.............aauggcacuggaagaauucacUg..................................................  14     1  OV1
.............aauggcacuggaaUaauucacgg..................................................  1      1  OV1
.............aUuggcacuggaagaauucacgg..................................................  3      1  OV1
.............aauggcacCggaagaauucacgg..................................................  13     1  OV1
.............aauggcacuggaagaaCucacgg..................................................  27     1  OV1
.............aauggcacuggaagCauucacgg..................................................  1      1  OV1
.............aauggcacuggaagaauucaGgg..................................................  1      1  OV1
.............aauggcaAuggaagaauucacgg..................................................  2      1  OV1
.............aauggcacuCgaagaauucacgg..................................................  6      1  OV1
.............aauggcUcuggaagaauucacgg..................................................  4      1  OV1
.............aauggcacAggaagaauucacgg..................................................  5      1  OV1
.............aauggcacuggaagaaGucacgg..................................................  4      1  OV1
.............aauggcacuggaagaauucaUgg..................................................  31     1  OV1
.............aauggcacuggaagaauucCcgg..................................................  2      1  OV1
.............aauggcacuggaagGauucacgg..................................................  2      1  OV1
.............aauggcacuggaagaCuucacgg..................................................  3      1  OV1
.............aauggcacugUaagaauucacgg..................................................  26     1  OV1
.............aauggcacuggaagaauuAacgg..................................................  11     1  OV1
.............aauCgcacuggaagaauucacgg..................................................  1      1  OV1
.............Gauggcacuggaagaauucacgg..................................................  8      1  OV1
.............Cauggcacuggaagaauucacgg..................................................  1      1  OV1
.............aauggcacuggaagaauuGacgg..................................................  2      1  OV1
.............aauAgcacuggaagaauucacgg..................................................  26     1  OV1
```

```
             aga-miR-263a
                                                       aga-miR-263a*
cccugguacauguaauggcacuggaagaauucacgggauuuuuuucaacauucccguguucucuuaguggcauaccuaguacaggg

.............aauggGacuggaagaauucacgg..................................................    4        1    OV1
.............aauggcacuggaaAaauucacgg..................................................    9        1    OV1
.............aauggcacuggaagaauuUacgg..................................................    36       1    OV1
.............aaugCcacuggaagaauucacgg..................................................    5        1    OV1
.............aauUgcacuggaagaauucacgg..................................................    2        1    OV1
.............aauggcacuggaagaaAucacgg..................................................    3        1    OV1
.............aauggcacuggaagaUuucacgg..................................................    2        1    OV1
.............Uauggcacuggaagaauucacgg..................................................    9        1    OV1
.............aauggcacuggaagaauucacgA..................................................    61       1    OV1
.............aauggcacuggaaCaauucacgg..................................................    5        1    OV1
.............aauggcacuggaagaauucaAgg..................................................    5        1    OV1
.............aaCggcacuggaagaauucacgg..................................................    13       1    OV1
.............aaAggcacuggaagaauucacgg..................................................    4        1    OV1
.............aaugUcacuggaagaauucacgg..................................................    5        1    OV1
.............aauggUacuggaagaauucacgg..................................................    26       1    OV1
.............aauggcacugCaagaauucacgg..................................................    12       1    OV1
.............aGuggcacuggaagaauucacgg..................................................    11       1    OV1
.............aaugAcacuggaagaauucacgg..................................................    15       1    OV1
.............aauggcacuggaagaauucGcgg..................................................    13       1    OV1
.............aauggcacuggaagaauAcacgg..................................................    3        1    OV1
.............aauggcacGggaagaauucacgg..................................................    3        1    OV1
.............aauggAacuggaagaauucacgg..................................................    9        1    OV1
.............aauggcacuggaagaauCcacgg..................................................    16       1    OV1
.............aauggcacuggaagaauucacgC..................................................    3        1    OV1
.............aauggcacuggaGgaauucacgg..................................................    6        1    OV1
.............aauggcacuggaagaauucCcggg.................................................    1        1    OV1
.............aGuggcacuggaagaauucacggg.................................................    9        1    OV1
.............aauggcacuggaagaaCucacggg.................................................    35       1    OV1
.............aaugAcacuggaagaauucacggg.................................................    26       1    OV1
.............aauggcacuggaagGauucacggg.................................................    4        1    OV1
.............aauggcacuggaagaauAcacggg.................................................    7        1    OV1
.............Uauggcacuggaagaauucacggg.................................................    5        1    OV1
.............aauggcacuggaagaUuucacggg.................................................    4        1    OV1
.............aauggcacuggaagaauucacgAg.................................................    33       1    OV1
.............Gauggcacuggaagaauucacggg.................................................    13       1    OV1
.............aauggcaAuggaagaauucacggg.................................................    4        1    OV1
.............aauggcaUuggaagaauucacggg.................................................    11       1    OV1
.............aaAggcacuggaagaauucacggg.................................................    5        1    OV1
.............aauggcacuggaagaauucacgUg.................................................    1        1    OV1
.............aauggcGcuggaagaauucacggg.................................................    4        1    OV1
.............aaugUcacuggaagaauucacggg.................................................    3        1    OV1
.............aauggcacuAgaagaauucacggg.................................................    17       1    OV1
.............aauCgcacuggaagaauucacggg.................................................    1        1    OV1
.............aauggcacuggaagaauuGacggg.................................................    5        1    OV1
.............aaugCcacuggaagaauucacggg.................................................    5        1    OV1
.............aauggcacuggaagaauucacggC.................................................    20       1    OV1
.............aauggcacuggGagaauucacggg.................................................    20       1    OV1
.............aauggcacuggaagaauucacggg.................................................    60205    0    OV1
.............aUuggcacuggaagaauucacggg.................................................    5        1    OV1
.............aauggcacuggaagaauCcacggg.................................................    16       1    OV1
.............aauggcacuggaagaauucGcggg.................................................    13       1    OV1
.............aauggcUcuggaagaauucacggg.................................................    8        1    OV1
.............aauggcacuggaagaauucacgCg.................................................    1        1    OV1
.............aauggcacugUaagaauucacggg.................................................    24       1    OV1
.............aauggcacuggaagaauucacAgg.................................................    31       1    OV1
.............aauggcacuggaagaaAucacggg.................................................    3        1    OV1
.............aauggcacuggaagaGuucacggg.................................................    4        1    OV1
.............aauggcacuggaaUaauucacggg.................................................    3        1    OV1
.............aauggUacuggaagaauucacggg.................................................    24       1    OV1
.............aauggcacuggaagaauuAacggg.................................................    16       1    OV1
.............aauggcacuggCagaauucacggg.................................................    20       1    OV1
.............aauggcacAggaagaauucacggg.................................................    5        1    OV1
.............aauggcacuggaagaauucaUggg.................................................    32       1    OV1
.............aauggcacuCgaagaauucacggg.................................................    9        1    OV1
.............aaCggcacuggaagaauucacggg.................................................    23       1    OV1
.............aauggcacugAaagaauucacggg.................................................    64       1    OV1
.............aauggcacCggaagaauucacggg.................................................    21       1    OV1
.............aauggcacGggaagaauucacggg.................................................    5        1    OV1
.............aauggcacuggaagaauucacggA.................................................    342      1    OV1
```

```
                        aga-miR-263a
                                                                          aga-miR-263a*
cccugguacauguaauggcacuggaagaauucacgggauuuuuuucaacauucccguguucucuuaguggcauaccuaguacaggg

.............aauggcacuggaUgaauucacggg.................................................   5      1   OV1
.............aauggcacuggaagaauucacCgg.................................................   17     1   OV1
.............aauAgcacuggaagaauucacggg.................................................   33     1   OV1
.............aauUgcacuggaagaauucacggg.................................................   1      1   OV1
.............aauggcacuggaagCauucacggg.................................................   2      1   OV1
.............aauggcacuggaagaauucaGggg.................................................   3      1   OV1
.............aauggGacuggaagaauucacggg.................................................   2      1   OV1
.............aauggcacugCaagaauucacggg.................................................   13     1   OV1
.............aauggcacuggaGgaauucacggg.................................................   6      1   OV1
.............aauggcacuggaagaauucacUgg.................................................   9      1   OV1
.............aauggcacuggUagaauucacggg.................................................   16     1   OV1
.............aauggcacuggaaAaauucacggg.................................................   11     1   OV1
.............aauggcacuggaagaauuCUcggg.................................................   3      1   OV1
.............aauggcacuggaagaauuUacggg.................................................   32     1   OV1
.............aauggAacuggaagaauucacggg.................................................   5      1   OV1
.............Cauggcacuggaagaauucacggg.................................................   2      1   OV1
.............aauggcaGuggaagaauucacggg.................................................   1      1   OV1
.............aauggcacuggaagaauucacggU.................................................   25     1   OV1
.............aauggcacuUgaagaauucacggg.................................................   11     1   OV1
.............aauggcacuggaagaCuucacggg.................................................   5      1   OV1
.............aauggcacuggaagaauucaAggg.................................................   4      1   OV1
.............aauggcCcuggaagaauucacggg.................................................   2      1   OV1
.............aauggcacuggaagaauucaGggga................................................   1      1   OV1
.............aauggcacuggUagaauucacggga................................................   2      1   OV1
.............aauggcacuggaaAaauucacggga................................................   1      1   OV1
.............aaugAcacuggaagaauucacggga................................................   8      1   OV1
.............aauggcaUuggaagaauucacggga................................................   2      1   OV1
.............aauggcacAggaagaauucacggga................................................   1      1   OV1
.............aauggcacCggaagaauucacggga................................................   6      1   OV1
.............aauggcacuggaagaauucaAggga................................................   3      1   OV1
.............aauggcacuggaagaauuGacggga................................................   1      1   OV1
.............aauggcacugAaagaauucacggga................................................   14     1   OV1
.............aauggcacuggaUgaauucacggga................................................   2      1   OV1
.............aauggAacuggaagaauucacggga................................................   4      1   OV1
.............aauggcacGggaagaauucacggga................................................   3      1   OV1
.............aauggcacuggaagaauucacAgga................................................   11     1   OV1
.............aaAggcacuggaagaauucacggga................................................   5      1   OV1
.............aauggcacuggaagaauucaUggga................................................   7      1   OV1
.............aauggcacuggCagaauucacggga................................................   12     1   OV1
.............aauAgcacuggaagaauucacggga................................................   5      1   OV1
.............Gauggcacuggaagaauucacggga................................................   1      1   OV1
.............aauggcGcuggaagaauucacggga................................................   1      1   OV1
.............aauggcacuggaagUauucacggga................................................   1      1   OV1
.............aaugUcacuggaagaauucacggga................................................   3      1   OV1
.............aauggcacuggaagaauucacggAa................................................   247    1   OV1
.............aauggcacuggaagaauucGcggga................................................   4      1   OV1
.............aauggcacuggaagaauucacgAga................................................   6      1   OV1
.............aauggcacuggaagaauucacgggC................................................   74     1   OV1
.............aauggcacugUaagaauucacggga................................................   7      1   OV1
.............aauggcacuAgaagaauucacggga................................................   9      1   OV1
.............aGuggcacuggaagaauucacggga................................................   3      1   OV1
.............aauggcacuggaagaCuucacggga................................................   2      1   OV1
.............aauggcacugCaagaauucacggga................................................   1      1   OV1
.............aauggcacuggaagaGuucacggga................................................   1      1   OV1
.............aauggcacuggaagaauucacggCa................................................   4      1   OV1
.............aauggcacuggaagaauucCcggga................................................   1      1   OV1
.............aauggcacuggaagaaAucacggga................................................   4      1   OV1
.............aauggcaAuggaagaauucacggga................................................   1      1   OV1
.............aauCgcacuggaagaauucacggga................................................   2      1   OV1
.............aauggGacuggaagaauucacggga................................................   1      1   OV1
.............aauggcacuggaagaauCcacggga................................................   6      1   OV1
.............aauggcacuggaagaauucacgggG................................................   40     1   OV1
.............aaCggcacuggaagaauucacggga................................................   6      1   OV1
.............aauggcacuggaagaauAcacggga................................................   1      1   OV1
.............aCuggcacuggaagaauucacggga................................................   2      1   OV1
.............aauggUacuggaagaauucacggga................................................   5      1   OV1
.............aauggcacuggaagaauucacggga................................................   16668  0   OV1
.............Uauggcacuggaagaauucacggga................................................   2      1   OV1
.............aauggcacuCgaagaauucacggga................................................   3      1   OV1
```

aga-miR-263a

aga-miR-263a*

cccugguacauguaauggcacuggaagaauucacgggauuuuuuucaacauucccguguucucuuaguggcauaccuaguacaggg

.............aauggcUcuggaagaauucacggga..	3	1	OV1
.............aauggcacuggaagaauucacCgga..	7	1	OV1
.............aauggcacuggaagaauucacggUa..	6	1	OV1
.............aauggcacuggaagaauuAacggga..	4	1	OV1
.............aauggcacuggGagaauucacggga..	6	1	OV1
.............aauggcacuggaagaaCucacggga..	8	1	OV1
.............aaugCcacuggaagaauucacggga..	2	1	OV1
.............aauggcacuUgaagaauucacggga..	3	1	OV1
.............aauggcacuggaagaauucacUgga..	5	1	OV1
.............aauUgcacuggaagaauucacggga..	1	1	OV1
.............aauggcacuggaagaauucacgggU..	707	1	OV1
.............aauggcacuggaagaauuUacggga..	10	1	OV1
.............aauggcacuggaagaauucacgUga..	3	1	OV1
.............aauggUacuggaagaauucacgggau...	1	1	OV1
.............aauggcacuggaagaauucacggUau...	1	1	OV1
.............aauggcacuggaagaauucacAggau...	2	1	OV1
.............aauggcacuggaagaauucacgggUu...	30	1	OV1
.............aauggcacuggaagaauucaUgggau...	1	1	OV1
.............aaAggcacuggaagaauucacgggau...	1	1	OV1
.............aauggcacuggaagaauucacgggaC...	64	1	OV1
.............aauggcacuggaagaauucacgggaA...	2533	1	OV1
.............aauggcacuggaagaauucacggAau...	2	1	OV1
.............aauggcacuggaagaauucacgggCu...	6	1	OV1
.............aaCggcacuggaagaauucacgggau...	1	1	OV1
.............aauggcacuggaagaauucacgggaG...	9	1	OV1
.............aauggcacuggaagaauucacgggau...	693	0	OV1
.............aauggcacuggaagaauucacgggauC..	1	1	OV1
.............aauggcacuggaagaauucacgggaAu..	149	1	OV1
.............aauggcacuggaagaauucacgggaCu..	7	1	OV1
.............aauggcacuggaagaauucacgggauu..	47	0	OV1
.............aauggcacuggaagaauucacgggUuu..	15	1	OV1
.............aauggcacuggaagaauucacgggauA..	24	1	OV1
.............aauggcacuggaagaauucacgggauCu...	1	1	OV1
.............aauggcacuggaagaauucacgggauAu...	12	1	OV1
.............aauggcacuggaagaauucacgggauuu...	9	0	OV1
.............aauggcacuggaagaauucacgggaAuu...	6	1	OV1
.............aauggcacuggaagaauucacgggUuuu...	2	1	OV1
.............aauggcacuggaagaauucacgggauAuu..	1	1	OV1
.............aauggcacuAgaagaauucacgggauuuu..	1	1	OV1
.............aauggcacuggaagaauucacgggauuuA..	1	1	OV1
.............aauggcacuggaagaauucacgggauuAu..	2	1	OV1
.............aauggcacuggaagaauucacgggaAuuu..	2	1	OV1
.............aauggcacuggaagaauucacgggUuuuu..	1	1	OV1
.............aauggcacuggaagaauucacgggauuuu..	7	0	OV1
.............aauggcacuggaagaauucacgggaAuuuu...	2	1	OV1
.............aauggcacuggaagaauucacgggauuuuu...	1	0	OV1
.............aauggcacuggaagaauucacgggauuuuuuu...	3	0	OV1
..............auggcacuggaagaauucac..	2	0	OV1
..............auggcacuggaagaauucacgg..	11	0	OV1
..............auggcacuggaagaauucacggg...	20	0	OV1
..............auggcacuggaagaauucacggga..	66	0	OV1
..............auggcacugUaagaauucacggga..	1	1	OV1
..............auggcacuggaagaauucacgggaA...	10	1	OV1
..............auggcacuggaagaauucacgggaC...	2	1	OV1
..............auggcacuggaagaauucacgggauu..	1	0	OV1
...............uggcacuggaagaauucacgg..	2	0	OV1
...............uggcacuggaagaauucacggg...	1	0	OV1
...............uggcacuggaagaauucacggga..	4	0	OV1
...............uggcacuggaagaauucacgggau...	2	0	OV1
.................gcacuggaagaauucacgg..	1	0	OV1
.................Ccacuggaagaauucacggga..	1	1	OV1
.................gcacuggaagaauucacggga..	1	0	OV1
.................gcacuggaagaauucacgggaA...	1	1	OV1
..................cacuggaagaauucacg...	1	0	OV1
..................cacuggaagaauucacgg..	5	0	OV1
..................cacuggaagaauucacggg...	1	0	OV1
..................cacuggaagaauucacggga..	2	0	OV1
...................acuggaagaauucacgg..	1	0	OV1
....................cuggaagaauucacggg...	3	0	OV1

```
             aga-miR-263a
                                                      aga-miR-263a*
cccugguacauguaauggcacuggaagaauucacgggauuuuuuucaacauucccguguucucuuaguggcauaccuaguacaggg

....................cuggaagaauucacgggU................................................    1       1       OV1
....................cuggaagaauucacggga................................................    1       0       OV1
......................................................cguguucucuuaguggcauacc..........    23      0       OV1

...........gGaauggcacuggaagaauucacgg..................................................    1       1       FF1
............Caauggcacuggaagaauucac....................................................    1       1       FF1
............Caauggcacuggaagaauucacg...................................................    2       1       FF1
............Caauggcacuggaagaauucacgg..................................................    14      1       FF1
............uaauggcacuggaagaauucacgg..................................................    2       0       FF1
............uaauggcacuggaagaauucacggg.................................................    1       0       FF1
............Caauggcacuggaagaauucacggg.................................................    49      1       FF1
............Gaauggcacuggaagaauucacggg.................................................    1       1       FF1
............Aaauggcacuggaagaauucacggg.................................................    3       1       FF1
............Caauggcacuggaagaauucacggga................................................    3       1       FF1
.............aauggcacuggaagaau........................................................    1       0       FF1
.............aauggcacuggaagaauu.......................................................    3       0       FF1
.............aauggcacuggaagaauuca.....................................................    60      0       FF1
.............aauggcacuggaagaauucaU....................................................    1       1       FF1
.............aauggcacuggGagaauucac....................................................    1       1       FF1
.............aauggcacuggaagaauucUc....................................................    1       1       FF1
.............aaAggcacuggaagaauucac....................................................    1       1       FF1
.............Gauggcacuggaagaauucac....................................................    1       1       FF1
.............aauggcacuggaagaauucac....................................................    412     0       FF1
.............aauggcacuggaagaauucaA....................................................    2       1       FF1
.............aauggcacCggaagaauucac....................................................    1       1       FF1
.............aauggAacuggaagaauucac....................................................    1       1       FF1
.............aauggcacuggaaAaauucacg...................................................    1       1       FF1
.............aauggcacugAaagaauucacg...................................................    2       1       FF1
.............aauggcaUuggaagaauucacg...................................................    2       1       FF1
.............aauggcacCggaagaauucacg...................................................    1       1       FF1
.............aauUgcacuggaagaauucacg...................................................    1       1       FF1
.............aauggcacuggGagaauucacg...................................................    1       1       FF1
.............aauggcacuggaagaauucaUg...................................................    1       1       FF1
.............aauggcacuggaagaauucacA...................................................    7       1       FF1
.............aGuggcacuggaagaauucacg...................................................    1       1       FF1
.............aauggcacuggaagaauucacg...................................................    1628    0       FF1
.............aauggcacuggaagaauucacU...................................................    3       1       FF1
.............aUuggcacuggaagaauucacg...................................................    1       1       FF1
.............aauggcacuggaagaauuUacg...................................................    2       1       FF1
.............aauggcacugUaagaauucacg...................................................    1       1       FF1
.............aauggcacuggCagaauucacg...................................................    1       1       FF1
.............aauggcacuggaagaauucaGg...................................................    1       1       FF1
.............aaAggcacuggaagaauucacgg..................................................    2       1       FF1
.............aauggcacuggaagaauucaUgg..................................................    8       1       FF1
.............aauggcacuCgaagaauucacgg..................................................    1       1       FF1
.............aauggcUcuggaagaauucacgg..................................................    1       1       FF1
.............aaGggcacuggaagaauucacgg..................................................    3       1       FF1
.............aauggcacuggaaCaauucacgg..................................................    1       1       FF1
.............aauggcacuggaagaauucGcgg..................................................    7       1       FF1
.............aauggcacuggaagaauCcacgg..................................................    6       1       FF1
.............aauggcGcuggaagaauucacgg..................................................    5       1       FF1
.............aauggcacuggaagaauucUcgg..................................................    1       1       FF1
.............aauggcacuggaagaauucacgU..................................................    3       1       FF1
.............aauggcacuggaagaauucacgg..................................................    23348   0       FF1
.............aaugUcacuggaagaauucacgg..................................................    4       1       FF1
.............aauggcacuggaaAaauucacgg..................................................    5       1       FF1
.............aCuggcacuggaagaauucacgg..................................................    2       1       FF1
.............aauggAacuggaagaauucacgg..................................................    4       1       FF1
.............aauggcacugAaagaauucacgg..................................................    32      1       FF1
.............aauggcacuggaagaauAcacgg..................................................    5       1       FF1
.............aauggcacuAgaagaauucacgg..................................................    8       1       FF1
.............Uauggcacuggaagaauucacgg..................................................    1       1       FF1
.............aauggcacugUaagaauucacgg..................................................    7       1       FF1
.............aauggcacuggaagGauucacgg..................................................    2       1       FF1
.............aauggcacuUgaagaauucacgg..................................................    3       1       FF1
.............aauggcacuggaagaGuucacgg..................................................    1       1       FF1
.............aauggcacGggaagaauucacgg..................................................    2       1       FF1
.............aauggcacuggUagaauucacgg..................................................    13      1       FF1
.............aauggcaAuggaagaauucacgg..................................................    2       1       FF1
```

```
             aga-miR-263a
                                                             aga-miR-263a*
cccugguacauguaauggcacuggaagaauucacgggauuuuuuucaacauucccguguucucuuaguggcauaccuaguacaggg

.............aauggcacuggaagaaCucacgg..................................................   6       1    FF1
.............aauggcacCggaagaauucacgg..................................................   3       1    FF1
.............aauggcacuggaagaauuAacgg..................................................   2       1    FF1
.............aauggcacugCaagaauucacgg..................................................   12      1    FF1
.............aGuggcacuggaagaauucacgg..................................................   1       1    FF1
.............aauggGacuggaagaauucacgg..................................................   1       1    FF1
.............aauggcacuggaagaaAucacgg..................................................   1       1    FF1
.............aauggcacuggaagaauucacgA..................................................   32      1    FF1
.............aauggUacuggaagaauucacgg..................................................   6       1    FF1
.............Gauggcacuggaagaauucacgg..................................................   4       1    FF1
.............aUuggcacuggaagaauucacgg..................................................   1       1    FF1
.............aauAgcacuggaagaauucacgg..................................................   11      1    FF1
.............aaugAcacuggaagaauucacgg..................................................   5       1    FF1
.............aauggcacuggaagaauucaAgg..................................................   2       1    FF1
.............aauggcacuggaagaauucacgC..................................................   2       1    FF1
.............aauggcacuggaGgaauucacgg..................................................   1       1    FF1
.............aauggcacuggaagaaGucacgg..................................................   1       1    FF1
.............aauggcacuggGagaauucacgg..................................................   7       1    FF1
.............aaCggcacuggaagaauucacgg..................................................   6       1    FF1
.............aauggcaUuggaagaauucacgg..................................................   5       1    FF1
.............aauggcacuggaUgaauucacgg..................................................   1       1    FF1
.............aauggcacuggaagaauucacAg..................................................   8       1    FF1
.............aauggcacuggaagaauuUacgg..................................................   17      1    FF1
.............aauggcacuggCagaauucacgg..................................................   7       1    FF1
.............aauggcacuggaagaauucacUg..................................................   2       1    FF1
.............aauCgcacuggaagaauucacgg..................................................   1       1    FF1
.............aauggcacuggaagaUuucacgg..................................................   1       1    FF1
.............aauggcacuggaaUaauucacgg..................................................   2       1    FF1
.............aauggcacuggaagaauucacUgg.................................................   6       1    FF1
.............aauggcaAuggaagaauucacggg.................................................   3       1    FF1
.............aauggcacuggaGgaauucacggg.................................................   12      1    FF1
.............aUuggcacuggaagaauucacggg.................................................   7       1    FF1
.............aauggcacuggGagaauucacggg.................................................   30      1    FF1
.............aauggcacAggaagaauucacggg.................................................   10      1    FF1
.............aauggcacuAgaagaauucacggg.................................................   23      1    FF1
.............aauggcacuggaagaauuUacggg.................................................   47      1    FF1
.............aauggcacugAaagaauucacggg.................................................   107     1    FF1
.............aaGggcacuggaagaauucacggg.................................................   3       1    FF1
.............aGuggcacuggaagaauucacggg.................................................   7       1    FF1
.............aauggcacuggaaCaauucacggg.................................................   3       1    FF1
.............aauggcacuggaagaauucCcggg.................................................   6       1    FF1
.............aauggcacuUgaagaauucacggg.................................................   12      1    FF1
.............Gauggcacuggaagaauucacggg.................................................   11      1    FF1
.............aauggcacuggaagGauucacggg.................................................   10      1    FF1
.............aaAggcacuggaagaauucacggg.................................................   7       1    FF1
.............aauggcacuggaagaauucGcggg.................................................   10      1    FF1
.............aauggcacuggaagaauucaAggg.................................................   3       1    FF1
.............Uauggcacuggaagaauucacggg.................................................   10      1    FF1
.............aauCgcacuggaagaauucacggg.................................................   4       1    FF1
.............aauggcacuggaagaauucacgAg.................................................   23      1    FF1
.............aauggUacuggaagaauucacggg.................................................   23      1    FF1
.............aauggcacuggaagaauucUcggg.................................................   6       1    FF1
.............aauggcacugUaagaauucacggg.................................................   38      1    FF1
.............aauggcacuggaagaGuucacggg.................................................   12      1    FF1
.............aauggcacuggaagaauucacggC.................................................   23      1    FF1
.............aauggcacuggaagaauucaGggg.................................................   3       1    FF1
.............aauggcacuggaagaaAucacggg.................................................   6       1    FF1
.............Cauggcacuggaagaauucacggg.................................................   7       1    FF1
.............aauUgcacuggaagaauucacggg.................................................   2       1    FF1
.............aauggcacuggaaUaauucacggg.................................................   5       1    FF1
.............aauggcacuggaagaaCucacggg.................................................   47      1    FF1
.............aauggcacuggaagaauucacggg.................................................   99760   0    FF1
.............aauggcCcuggaagaauucacggg.................................................   1       1    FF1
.............aauggcacuggUagaauucacggg.................................................   26      1    FF1
.............aauggcacuggaagUauucacggg.................................................   1       1    FF1
.............aaugAcacuggaagaauucacggg.................................................   26      1    FF1
.............aauggcacuggaagaCuucacggg.................................................   4       1    FF1
.............aauggcacuggaagaauucacgUg.................................................   3       1    FF1
.............aCuggcacuggaagaauucacggg.................................................   2       1    FF1
```

```
             aga-miR-263a
                                                     aga-miR-263a*
cccugguacauguaauggcacuggaagaauucacgggauuuuuuucaacauucccguguucucuuaguggcauaccuaguacaggg

.............aauggcacuggCagaauucacggg.................................................   33      1   FF1
.............aaugUcacuggaagaauucacggg.................................................   9       1   FF1
.............aauggcacuggaagaauuAacggg.................................................   9       1   FF1
.............aauggcacCggaagaauucacggg.................................................   22      1   FF1
.............aauggcacuggaagaauucacCgg.................................................   5       1   FF1
.............aaugCcacuggaagaauucacggg.................................................   4       1   FF1
.............aauAgcacuggaagaauucacggg.................................................   37      1   FF1
.............aauggcacuggaagaauucacggA.................................................   120     1   FF1
.............aauggcacuggaagaauucacggU.................................................   16      1   FF1
.............aauggcacuCgaagaauucacggg.................................................   4       1   FF1
.............aauggcacuggaUgaauucacggg.................................................   3       1   FF1
.............aauggcacuggaagaauGcacggg.................................................   1       1   FF1
.............aauggcacGggaagaauucacggg.................................................   5       1   FF1
.............aauggcacuggaagaauucacgCg.................................................   2       1   FF1
.............aauggcacuggaagaauAcacggg.................................................   7       1   FF1
.............aauggcacuggaagaauucaUggg.................................................   34      1   FF1
.............aauggcacuggaagaauucacAgg.................................................   24      1   FF1
.............aauggAacuggaagaauucacggg.................................................   9       1   FF1
.............aauggcacuggaagaauuGacggg.................................................   2       1   FF1
.............aauggcacuggaaAaauucacggg.................................................   14      1   FF1
.............aauggcUcuggaagaauucacggg.................................................   2       1   FF1
.............aauggcacuggaagaauCcacggg.................................................   26      1   FF1
.............aaCggcacuggaagaauucacggg.................................................   37      1   FF1
.............aauggcaUuggaagaauucacggg.................................................   12      1   FF1
.............aauggcGcuggaagaauucacggg.................................................   9       1   FF1
.............aauggcacugCaagaauucacggg.................................................   32      1   FF1
.............aauggGacuggaagaauucacggg.................................................   2       1   FF1
.............aaugAcacuggaagaauucacggga................................................   3       1   FF1
.............aauggcacuggaagaauucacgggG................................................   52      1   FF1
.............aauggcUcuggaagaauucacggga................................................   2       1   FF1
.............aauggcacugAaagaauucacggga................................................   10      1   FF1
.............aauggcacuAgaagaauucacggga................................................   5       1   FF1
.............aauggcacuggaagaauucacggga................................................   11130   0   FF1
.............aauggcacuggaagaauuGacggga................................................   1       1   FF1
.............aauggcacuggaagaauucacAgga................................................   2       1   FF1
.............aauggcacuggaagGauucacggga................................................   1       1   FF1
.............aauggcacuggaagaauucacgggC................................................   42      1   FF1
.............aauggcacuggUagaauucacggga................................................   6       1   FF1
.............aauggcacCggaagaauucacggga................................................   2       1   FF1
.............Uauggcacuggaagaauucacggga................................................   1       1   FF1
.............aauAgcacuggaagaauucacggga................................................   11      1   FF1
.............aauggcGcuggaagaauucacggga................................................   3       1   FF1
.............aauggcacuggGagaauucacggga................................................   1       1   FF1
.............aauggcacuggaagaaCucacggga................................................   8       1   FF1
.............aauggAacuggaagaauucacggga................................................   3       1   FF1
.............aauggcacuggaagaGuucacggga................................................   3       1   FF1
.............aauggcaAuggaagaauucacggga................................................   1       1   FF1
.............aauggcacuggaagaauucacgggU................................................   515     1   FF1
.............aauggcacuggaagaauuUacggga................................................   3       1   FF1
.............aauggcacuUgaagaauucacggga................................................   1       1   FF1
.............aUuggcacuggaagaauucacggga................................................   1       1   FF1
.............aauggGacuggaagaauucacggga................................................   2       1   FF1
.............aauCgcacuggaagaauucacggga................................................   1       1   FF1
.............aauggcacuggaagaCuucacggga................................................   1       1   FF1
.............aauggcacuggaagaauucaUggga................................................   7       1   FF1
.............aauggcacuggaagaauucacUgga................................................   1       1   FF1
.............aauggcacuggaaAaauucacggga................................................   1       1   FF1
.............aauggcacuggaagaauucacggCa................................................   2       1   FF1
.............aauggcacugCaagaauucacggga................................................   3       1   FF1
.............aauggcaUuggaagaauucacggga................................................   3       1   FF1
.............aauggcacAggaagaauucacggga................................................   1       1   FF1
.............aaAggcacuggaagaauucacggga................................................   1       1   FF1
.............aauggcacuggaagaauucacggUa................................................   2       1   FF1
.............aaCggcacuggaagaauucacggga................................................   3       1   FF1
.............aauggcacugUaagaauucacggga................................................   6       1   FF1
.............aauggcacuggaagaauuAacggga................................................   1       1   FF1
.............aauggcacuggaagaauucGcggga................................................   6       1   FF1
.............aauggcacuggaagaauucacgAga................................................   1       1   FF1
.............aauggcacuggaagaauGcacggga................................................   1       1   FF1
```

```
             aga-miR-263a
                                                     aga-miR-263a*
cccugguacaugu aauggcacuggaagaauucacggg auuuuuuucaacauucc cguguucucuuaguggcauacc uaguacaggg

.............Gauggcacuggaagaauucacggga................................................    1      1    FF1
.............aauggcacuggaagaauucacggAa................................................    74     1    FF1
.............aauggcacuggaaAaauucacgggau...............................................    1      1    FF1
.............aauggcacuggaagaauucacgggaG...............................................    2      1    FF1
.............aauggcacuggaagaauucacgggCu...............................................    7      1    FF1
.............aauggcacuggaagaauucacgggau...............................................    237    0    FF1
.............aauggcacuggaagaauucacgggUu...............................................    50     1    FF1
.............aaAggcacuggaagaauucacgggau...............................................    1      1    FF1
.............aauggcacCggaagaauucacgggau...............................................    1      1    FF1
.............aauggcacugAaagaauucacgggau...............................................    1      1    FF1
.............aauggcacuggaagaauucacgggaA...............................................    1097   1    FF1
.............aauggcacuggaagaauucacgggaC...............................................    17     1    FF1
.............aauggcacuggaagaauCcacgggau...............................................    1      1    FF1
.............aauggcacuggaagaauucacgggaAu..............................................    70     1    FF1
.............aauggcacuggaagaauucacgggaCu..............................................    2      1    FF1
.............aauggcacuggaagaauucacgggauu..............................................    37     0    FF1
.............aauggcacuggaagaauucacgggauA..............................................    8      1    FF1
.............aauggcacuggaagaauucacgggUuu..............................................    11     1    FF1
.............aauggcacuggaagaauucacgggauAu.............................................    9      1    FF1
.............aauggcacuggaagaauucacgggaAuu.............................................    4      1    FF1
.............aauggcacuggaagaauucacgggUuuu.............................................    5      1    FF1
.............aauggcacuggaagaauucacgggauuu.............................................    17     0    FF1
.............aauggcacuggaagaauucacgggaAuuu............................................    2      1    FF1
.............aauggcacuggaagaauucacgggauuuu............................................    6      0    FF1
.............aauggcacuggaagaauucacgggauuuC............................................    1      1    FF1
.............aauggcacuggaagaauucacgggUuuuu............................................    1      1    FF1
.............aauggcacuggaagaauucacgggauAuu............................................    1      1    FF1
.............aauggcacuggaagaauucacgggauuuA............................................    1      1    FF1
.............aauggcacuggaagaauucacgggauuAu............................................    1      1    FF1
.............aauggcacuggaagaauucacgggauuuuu...........................................    1      0    FF1
.............aauggcacuggaagaauucacgggUuuuuu...........................................    2      1    FF1
.............aauggcacuggaagaauucacgggCuuuuu...........................................    1      1    FF1
.............aauggcacuggaagaauucacgggauuAuu...........................................    1      1    FF1
.............aauggcacuggaagaauucacgggauuuuuu..........................................    2      0    FF1
.............aauggcacuggaagaauucacgggauuuuuuuc........................................    1      0    FF1
.............aauggcacuggaagaauucacgggauuuuuuucaaca....................................    1      0    FF1
.............aauggcacuggaagaauucacgggauuuuuuucaacau...................................    1      0    FF1
.............aauggcacuggaagaauucacgggauuuuuuucaacauucc................................    1      0    FF1
..............auggcacuggaagaauucac....................................................    1      0    FF1
..............auggcacuggaagaauucacg...................................................    2      0    FF1
..............auggcacuggaagaauucacgg..................................................    9      0    FF1
..............auggcacuggaagaauucaUgg..................................................    1      1    FF1
..............auggcacuggaagaauucacggg.................................................    29     0    FF1
..............auggcacGggaagaauucacggg.................................................    1      1    FF1
..............auggcacuggaagaauucacggga................................................    71     0    FF1
..............auggcacuggaagaauucacgggau...............................................    2      0    FF1
..............auggcacuggaagaauucacgggaA...............................................    4      1    FF1
...............uggcacuggaagaauucacggg.................................................    2      0    FF1
...............uggcacuggaagaauucacggga................................................    3      0    FF1
...............uggcacuggaagaauucacgggau...............................................    6      0    FF1
.................gcacuggaagaauucacggg.................................................    1      0    FF1
..................cacuggaagaauucacggga................................................    1      0    FF1
....................cuggaagaauucacggg.................................................    2      0    FF1
.....................................................ccguguucucuuaguggcauacc..........    6      0    FF1
......................................................cguguucucuuagCggcauacc..........    1      1    FF1
......................................................cguguucucuuaguggcauacc..........    88     0    FF1
.......................................................guguucucuuaguggcauacc..........    1      0    FF1

............Caauggcacuggaagaauucacgg..................................................    3      1    MF1
............uaauggcacuggaagaauucacggg.................................................    1      0    MF1
............Caauggcacuggaagaauucacggg.................................................    5      1    MF1
............Caauggcacuggaagaauucacggga................................................    1      1    MF1
.............aauggcacuggaagaau........................................................    1      0    MF1
.............aauggcacuggaagaauu.......................................................    4      0    MF1
.............aauggcacuggaagaauuc......................................................    2      0    MF1
.............aauggcacuggaagaauuca.....................................................    28     0    MF1
.............aaAggcacuggaagaauucac....................................................    1      1    MF1
.............aauggcacuggaagaauucaA....................................................    2      1    MF1
.............aauggcacuggaagaauucac....................................................    385    0    MF1
```

```
             aga-miR-263a
                                                         aga-miR-263a*
cccugguacaugugaauggcacuggaagaauucacgggauuuuuuucaacauucccguguucucuuaguggcauaccuaguacaggg
```

```
.............aauggcacuggaagaauucaU....................................................  1      1   MF1
.............aauggcacuggaagaaAucac....................................................  1      1   MF1
.............aauggcacCggaagaauucacg...................................................  1      1   MF1
.............aauggcaUuggaagaauucacg...................................................  1      1   MF1
.............aauggcCcuggaagaauucacg...................................................  1      1   MF1
.............aauggcacuggaagaauucacg...................................................  976    0   MF1
.............aauggcacuggaagaauucacA...................................................  3      1   MF1
.............aauggcacuggaagaauucacU...................................................  2      1   MF1
.............aauggcacugCaagaauucacgg..................................................  2      1   MF1
.............aauggcacuUgaagaauucacgg..................................................  3      1   MF1
.............aauggcacuggaagaUuucacgg..................................................  2      1   MF1
.............aauggcacuggaagaaGucacgg..................................................  1      1   MF1
.............aaCggcacuggaagaauucacgg..................................................  2      1   MF1
.............aauggcacuggCagaauucacgg..................................................  5      1   MF1
.............aauggcacuggaagaauuUacgg..................................................  4      1   MF1
.............aauggcacuggaagaauucacgC..................................................  1      1   MF1
.............aaugUcacuggaagaauucacgg..................................................  1      1   MF1
.............aauggcacuAgaagaauucacgg..................................................  3      1   MF1
.............aauggcacuggUagaauucacgg..................................................  3      1   MF1
.............aauggUacuggaagaauucacgg..................................................  4      1   MF1
.............aauggcacuggaagaGuucacgg..................................................  1      1   MF1
.............aauggcacuggaagaauucGcgg..................................................  2      1   MF1
.............aauggcacuggaUgaauucacgg..................................................  1      1   MF1
.............aaugAcacuggaagaauucacgg..................................................  4      1   MF1
.............aauggcacuggaagaauucacgA..................................................  10     1   MF1
.............aauggcacAggaagaauucacgg..................................................  1      1   MF1
.............aaugCcacuggaagaauucacgg..................................................  1      1   MF1
.............aauggcacuggaagaauAcacgg..................................................  1      1   MF1
.............aauggcacuggaagaauCcacgg..................................................  2      1   MF1
.............aauggcacuggaagaauucacAg..................................................  6      1   MF1
.............aauggcacuggaagaaCucacgg..................................................  3      1   MF1
.............aauggcacugAaagaauucacgg..................................................  9      1   MF1
.............aauggcacuggaagaauucacUg..................................................  1      1   MF1
.............aauggcaGuggaagaauucacgg..................................................  2      1   MF1
.............aauggcaUuggaagaauucacgg..................................................  2      1   MF1
.............aUuggcacuggaagaauucacgg..................................................  1      1   MF1
.............aaGggcacuggaagaauucacgg..................................................  1      1   MF1
.............aauggcacuggaagUauucacgg..................................................  1      1   MF1
.............aauggcacuggaaUaauucacgg..................................................  1      1   MF1
.............aauggcacuggaGgaauucacgg..................................................  1      1   MF1
.............aauggcacuggaagaCuucacgg..................................................  1      1   MF1
.............aGuggcacuggaagaauucacgg..................................................  1      1   MF1
.............aauggcacuCgaagaauucacgg..................................................  2      1   MF1
.............aauggcacuggaaCaauucacgg..................................................  2      1   MF1
.............aauggcacugUaagaauucacgg..................................................  3      1   MF1
.............aauggcUcuggaagaauucacgg..................................................  1      1   MF1
.............aauggcacCggaagaauucacgg..................................................  2      1   MF1
.............aauggcacuggGagaauucacgg..................................................  7      1   MF1
.............aauAgcacuggaagaauucacgg..................................................  7      1   MF1
.............aauggcaAuggaagaauucacgg..................................................  2      1   MF1
.............Gauggcacuggaagaauucacgg..................................................  3      1   MF1
.............aauggcacuggaagaauucacCg..................................................  3      1   MF1
.............aauggcacuggaaAaauucacgg..................................................  1      1   MF1
.............aauggcacuggaagaauucacgg..................................................  7963   0   MF1
.............aauggcacuggaagaauucaUgg..................................................  5      1   MF1
.............aauggcacuggaagaauucacgU..................................................  1      1   MF1
.............aauggGacuggaagaauucacggg.................................................  1      1   MF1
.............aauggcacuAgaagaauucacggg.................................................  5      1   MF1
.............aauggcacuggaagaauUacggg..................................................  7      1   MF1
.............aauggcacuggaagaauucacgAg.................................................  4      1   MF1
.............aauggcacuggaagaCuucacggg.................................................  1      1   MF1
.............aauggcacCggaagaauucacggg.................................................  5      1   MF1
.............aaGggcacuggaagaauucacggg.................................................  1      1   MF1
.............aauggcacuggaagGauucacggg.................................................  2      1   MF1
.............aauggcGcuggaagaauucacggg.................................................  3      1   MF1
.............aauggcacuggaagaauucacggg.................................................  12741  0   MF1
.............aauggcacuggaagaauucacggC.................................................  7      1   MF1
.............aaAggcacuggaagaauucacggg.................................................  1      1   MF1
.............aauggcacuggaGgaauucacggg.................................................  3      1   MF1
```

```
                aga-miR-263a
                                               aga-miR-263a*
cccugguacaugu aauggcacuggaagaauucacggg auuuuuuucaacauucc cguguucucuuaguggcauacc uaguacaggg

.............aauAgcacuggaagaauucacggg.................................................    7      1    MF1
.............aauggcacuggaagaauucacggU.................................................    8      1    MF1
.............aauggcacuggaagaauuGacggg.................................................    1      1    MF1
.............aauggcacuggUagaauucacggg.................................................    3      1    MF1
.............aaCggcacuggaagaauucacggg.................................................    4      1    MF1
.............aauggcacuggaagaauucacgUg.................................................    2      1    MF1
.............aauggcacugAaagaauucacggg.................................................    12     1    MF1
.............aauggcacuggaagaauucacCgg.................................................    5      1    MF1
.............aauggcacugUaagaauucacggg.................................................    4      1    MF1
.............aauggcacuggCagaauucacggg.................................................    2      1    MF1
.............aauggcacuggaagaaCucacggg.................................................    9      1    MF1
.............aauggcacuggaagaauCcacggg.................................................    1      1    MF1
.............aauggcacuggaagaauAcacggg.................................................    2      1    MF1
.............aauggAacuggaagaauucacggg.................................................    2      1    MF1
.............aUuggcacuggaagaauucacggg.................................................    1      1    MF1
.............aauggcaAuggaagaauucacggg.................................................    1      1    MF1
.............aauggcacuUgaagaauucacggg.................................................    2      1    MF1
.............aauggcacuggaagaauuAacggg.................................................    2      1    MF1
.............aauggcacuggaagaauucCcggg.................................................    3      1    MF1
.............aauggcacuCgaagaauucacggg.................................................    1      1    MF1
.............aauggcacuggGagaauucacggg.................................................    7      1    MF1
.............aauggcacuggaagaauucacggA.................................................    38     1    MF1
.............aauggUacuggaagaauucacggg.................................................    3      1    MF1
.............aauggcacugCaagaauucacggg.................................................    3      1    MF1
.............aauggcacGggaagaauucacggg.................................................    2      1    MF1
.............aaugAcacuggaagaauucacggg.................................................    4      1    MF1
.............aGuggcacuggaagaauucacggg.................................................    1      1    MF1
.............aauggcacuggaagaauucGcggg.................................................    2      1    MF1
.............aauggcacuggaagaauucacAgg.................................................    5      1    MF1
.............aauggcacuggaagaauucaUggg.................................................    2      1    MF1
.............aauggcaUuggaagaauucacggg.................................................    1      1    MF1
.............aauggcacuggaagaauucacUgg.................................................    3      1    MF1
.............aauggcacuggaaAaauucacggg.................................................    1      1    MF1
.............aauggcacuggaagaauucacgggG................................................    6      1    MF1
.............aauggcacCggaagaauucacggga................................................    1      1    MF1
.............aauggcacuggaagaauucacgggC................................................    12     1    MF1
.............aauggcacuggGagaauucacggga................................................    2      1    MF1
.............aauggcacuggaagaauucacgUga................................................    1      1    MF1
.............aauggcacuggUagaauucacggga................................................    1      1    MF1
.............aauggcacuggaagaauucacggga................................................    1636   0    MF1
.............aauggcacuggaaAaauucacggga................................................    1      1    MF1
.............aauggcacuggaagaauucacggAa................................................    8      1    MF1
.............aaugAcacuggaagaauucacggga................................................    1      1    MF1
.............aauggcacuggaagaauucacggUa................................................    2      1    MF1
.............aUuggcacuggaagaauucacggga................................................    1      1    MF1
.............aauggcacugAaagaauucacggga................................................    2      1    MF1
.............aauggcacuAgaagaauucacggga................................................    1      1    MF1
.............aauggcacuggaagaauucGcggga................................................    1      1    MF1
.............aauggcacuggaagaauucacgggU................................................    249    1    MF1
.............aauggcacuggaagaauucaUggga................................................    1      1    MF1
.............aauggcacuggaagaauucacAgga................................................    1      1    MF1
.............aauggcacuggaagaauucacgggaC...............................................    5      1    MF1
.............aauggcacuggaagaauucacgggUu...............................................    20     1    MF1
.............aauggcacuggaagaauucacgggCu...............................................    3      1    MF1
.............aauggcacuggaagaauucacgggaA...............................................    181    1    MF1
.............aauggcacuggaagaauucacgggau...............................................    185    0    MF1
.............aauggcacuggaagaauucacggUau...............................................    1      1    MF1
.............aauggcacuggaagaauucacgggaG...............................................    1      1    MF1
.............aauggcacuggaagaauucacgggaCu..............................................    1      1    MF1
.............aauggcacuggaagaauucacgggauu..............................................    5      0    MF1
.............aauggcacuggaagaauucacgggUuu..............................................    7      1    MF1
.............aauggcacuggaagaauucacgggaAu..............................................    39     1    MF1
.............aauggcacuggaagaauucacgggUuuu.............................................    1      1    MF1
.............aauggcacuggaagaauucacgggaAuu.............................................    4      1    MF1
.............aauggcacuggaagaauucacgggauCu.............................................    1      1    MF1
.............aauggcacuggaagaauucacgggauuu.............................................    4      0    MF1
.............aauggcacuggaagaauucacgggauAu.............................................    7      1    MF1
.............aauggcacuggaagaauucacgggauuuu............................................    5      0    MF1
.............aauggcacuggaagaauucacgggaAuuu............................................    1      1    MF1
```

```
             aga-miR-263a
                                                         aga-miR-263a*
cccugguacauguaauggcacuggaagaauucacgggauuuuuuucaacauucccguguucucuuaguggcauaccuaguacaggg

.............aauggcacuggaagaauucacgggauCuu............................................   1      1   MF1
.............aauggcacuggaagaauucacgggauuAu............................................   2      1   MF1
.............aauggcacuggaagaauucacgggauAuu............................................   1      1   MF1
.............aauggcacuggaagaauucacgggauuuA............................................   1      1   MF1
.............aauggcacuggaagaauucacgggauuuuu...........................................   2      0   MF1
.............aauggcacuggaagaauucacgggauuuuuu..........................................   1      0   MF1
..............auggcacuggaagaauucacg...................................................   1      0   MF1
..............auggcacuggaagaauucacggg.................................................   2      0   MF1
..............auggcacuggaagaauucacggga................................................   3      0   MF1
...............uggcacuggaagaauucacgg..................................................   1      0   MF1
..................cacuggaagaauucacgg..................................................   2      0   MF1
.....................................................ccguguucucuuaguggcauacc..........   1      0   MF1
......................................................cguguucucuuaguggcauacc..........   22     0   MF1

...........gGaauggcacuggaagaauucacggg.................................................   1      1   BF2
............Caauggcacuggaagaauucacgg..................................................   7      1   BF2
............Caauggcacuggaagaauucacggg.................................................   30     1   BF2
............Caauggcacuggaagaauucacggga................................................   2      1   BF2
.............aauggcacuggaagaau........................................................   30     0   BF2
.............aauggcacuggaagaaA........................................................   1      1   BF2
.............aauggcacuggaagaauu.......................................................   23     0   BF2
.............aauggcacuggaagaauuc......................................................   4      0   BF2
.............aauggcacuggaagaauuca.....................................................   48     0   BF2
.............aauggcacuggCagaauucac....................................................   1      1   BF2
.............aauggcacuggaagaauucac....................................................   312    0   BF2
.............aauggcacuggaagaauucacA...................................................   2      1   BF2
.............aauggcacuggaagaaAucacg...................................................   1      1   BF2
.............aauggcacuggaagaauucacg...................................................   991    0   BF2
.............aauggcacugAaagaauucacg...................................................   1      1   BF2
.............aauggcacugUaagaauucacg...................................................   1      1   BF2
.............aaugAcacuggaagaauucacg...................................................   1      1   BF2
.............aaCggcacuggaagaauucacg...................................................   1      1   BF2
.............aauggcacuggaagaGuucacgg..................................................   1      1   BF2
.............aauggcacuggaagaauucaUgg..................................................   1      1   BF2
.............aauggcacuggaagaauCcacgg..................................................   3      1   BF2
.............aauggcacuggaagaauucacAg..................................................   6      1   BF2
.............aauggcacuggaagaauucacCg..................................................   2      1   BF2
.............aauggcacugCaagaauucacgg..................................................   6      1   BF2
.............aauggcacuggaagaauuGacgg..................................................   1      1   BF2
.............aauggcacuggaaAaauucacgg..................................................   1      1   BF2
.............aauggcacuggaagCauucacgg..................................................   1      1   BF2
.............aauggcacuggaagaauucacgg..................................................   11123  0   BF2
.............aauggcacCggaagaauucacgg..................................................   3      1   BF2
.............aauggcacAggaagaauucacgg..................................................   1      1   BF2
.............aauggcacuggaagaauucacgA..................................................   11     1   BF2
.............aauggcacuggaagaauucacUg..................................................   1      1   BF2
.............aauggcacuggCagaauucacgg..................................................   5      1   BF2
.............aauggcacuggUagaauucacgg..................................................   2      1   BF2
.............aauggcacGggaagaauucacgg..................................................   3      1   BF2
.............aauggcacugUaagaauucacgg..................................................   5      1   BF2
.............aauggcGcuggaagaauucacgg..................................................   1      1   BF2
.............aauggcaAuggaagaauucacgg..................................................   1      1   BF2
.............aauggcacuggGagaauucacgg..................................................   6      1   BF2
.............aauggcacuggaagaauuAacgg..................................................   2      1   BF2
.............Uauggcacuggaagaauucacgg..................................................   2      1   BF2
.............aaugAcacuggaagaauucacgg..................................................   1      1   BF2
.............aaCggcacuggaagaauucacgg..................................................   3      1   BF2
.............aauggcUcuggaagaauucacgg..................................................   1      1   BF2
.............aGuggcacuggaagaauucacgg..................................................   1      1   BF2
.............aauggcacuggaagaaAucacgg..................................................   1      1   BF2
.............aauggcacuggaagaauucGcgg..................................................   3      1   BF2
.............aauggcacugAaagaauucacgg..................................................   6      1   BF2
.............aauggcacuggaagaauucacgC..................................................   1      1   BF2
.............aauggcacuggaagaauucaAgg..................................................   1      1   BF2
.............aauggcacuggaagaaCucacgg..................................................   9      1   BF2
.............aauggcaUuggaagaauucacgg..................................................   1      1   BF2
.............aaugGacuggaagaauucacgg...................................................   1      1   BF2
.............aauggcacUgaagaauucacgg...................................................   1      1   BF2
.............aauggcacuAgaagaauucacgg..................................................   2      1   BF2
```

aga-miR-263a

aga-miR-263a*

```
cccugguacauguaauggcacuggaagaauucacgggauuuuuuucaacauucccguguucucuuaguggcauaccuaguacaggg

.............aauggcacuggaagaauuUacgg..................................................  8      1   BF2
.............aauAgcacuggaagaauucacgg..................................................  7      1   BF2
.............aauggcacuCgaagaauucacgg..................................................  1      1   BF2
.............aaAggcacuggaagaauucacgg..................................................  2      1   BF2
.............aauggcacuggaUgaauucacgg..................................................  1      1   BF2
.............aauggcacuggaGgaauucacgg..................................................  1      1   BF2
.............aauggcacuggaaUaauucacgg..................................................  2      1   BF2
.............aauggAacuggaagaauucacgg..................................................  2      1   BF2
.............aauggcacuggaagaauucacgU..................................................  2      1   BF2
.............aaugCcacuggaagaauucacgg..................................................  1      1   BF2
.............aauggcacuggaagUauucacgg..................................................  2      1   BF2
.............aauggUacuggaagaauucacgg..................................................  2      1   BF2
.............aauggUacuggaagaauucacggg.................................................  22     1   BF2
.............aauggcacuggaaAaauucacggg.................................................  14     1   BF2
.............aauggcacuggCagaauucacggg.................................................  38     1   BF2
.............aaugCcacuggaagaauucacggg.................................................  9      1   BF2
.............aauggcacuggaagaauucaAggg.................................................  7      1   BF2
.............aauggcacuggaagaauucacggg.................................................  91482  0   BF2
.............aauggcacuggaagaaGucacggg.................................................  1      1   BF2
.............aaugUcacuggaagaauucacggg.................................................  5      1   BF2
.............aauggcacGggaagaauucacggg.................................................  7      1   BF2
.............aauggcacuggaagaauuCUcggg.................................................  3      1   BF2
.............aauggcacuggaagaauuAacggg.................................................  11     1   BF2
.............aauggcacuggaagaCuucacggg.................................................  4      1   BF2
.............aauUgcacuggaagaauucacggg.................................................  3      1   BF2
.............aauggcacuggaagGauucacggg.................................................  5      1   BF2
.............aaCggcacuggaagaauucacggg.................................................  25     1   BF2
.............aauggcacuggaagaauucacgAg.................................................  16     1   BF2
.............aauggcacuggaagaaAucacggg.................................................  12     1   BF2
.............aauCgcacuggaagaauucacggg.................................................  1      1   BF2
.............aauggAacuggaagaauucacggg.................................................  21     1   BF2
.............aauggcUcuggaagaauucacggg.................................................  3      1   BF2
.............aauggcacuggaagaauucaUggg.................................................  37     1   BF2
.............aauggcacuCgaagaauucacggg.................................................  7      1   BF2
.............aauggcaAuggaagaauucacggg.................................................  2      1   BF2
.............aauggcacuggaagaauucacAgg.................................................  19     1   BF2
.............aauggcacuggaagaauucacggC.................................................  27     1   BF2
.............aauggcacuggaagaauucaCCgg.................................................  7      1   BF2
.............aaGggcacuggaagaauucacggg.................................................  5      1   BF2
.............aauggcacuggaagaauAcacggg.................................................  5      1   BF2
.............aauggcacuggUagaauucacggg.................................................  24     1   BF2
.............aauggcacuggGagaauucacggg.................................................  45     1   BF2
.............aauggcacugUaagaauucacggg.................................................  38     1   BF2
.............aauggcacuggaagaauucacggU.................................................  21     1   BF2
.............aauggcacuggaagaauucacgUg.................................................  5      1   BF2
.............aauggcacugCaagaauucacggg.................................................  27     1   BF2
.............aauggcacuAgaagaauucacggg.................................................  25     1   BF2
.............aauggcaGuggaagaauucacggg.................................................  5      1   BF2
.............aauggcacuggaagaauuUacggg.................................................  37     1   BF2
.............aauggcacuggaUgaauucacggg.................................................  2      1   BF2
.............aauggcacuggaagUauucacggg.................................................  1      1   BF2
.............aauggcaUuggaagaauucacggg.................................................  19     1   BF2
.............aauggcacuggaagaauucacUgg.................................................  6      1   BF2
.............aUuggcacuggaagaauucacggg.................................................  5      1   BF2
.............aGuggcacuggaagaauucacggg.................................................  15     1   BF2
.............aauggcacuggaagaGuucacggg.................................................  5      1   BF2
.............aauggcGcuggaagaauucacggg.................................................  10     1   BF2
.............aauggcacuggaaUaauucacggg.................................................  7      1   BF2
.............aauggcacuggaagaauCcacggg.................................................  16     1   BF2
.............aauggcacuggaagaauucacgCg.................................................  2      1   BF2
.............aauggcacuggaGgaauucacggg.................................................  10     1   BF2
.............aaugAcacuggaagaauucacggg.................................................  26     1   BF2
.............aauggcacugAaagaauucacggg.................................................  85     1   BF2
.............aauggcacuggaagaUuucacggg.................................................  2      1   BF2
.............Gauggcacuggaagaauucacggg.................................................  10     1   BF2
.............aauAgcacuggaagaauucacggg.................................................  31     1   BF2
.............Uauggcacuggaagaauucacggg.................................................  3      1   BF2
.............aaAggcacuggaagaauucacggg.................................................  11     1   BF2
.............aauggcacuggaaCaauucacggg.................................................  2      1   BF2
```

```
             aga-miR-263a
                                                                         aga-miR-263a*
cccugguacauguaauggcacuggaagaauucacgggauuuuuuucaacauucccguguucucuuaguggcauaccuaguacaggg

.............aauggGacuggaagaauucacggg.................................................   5       1   BF2
.............Cauggcacuggaagaauucacggg.................................................   2       1   BF2
.............aauggcacAggaagaauucacggg.................................................   3       1   BF2
.............aauggcCcuggaagaauucacggg.................................................   3       1   BF2
.............aauggcacuggaagaauucaGggg.................................................   4       1   BF2
.............aauggcacuggaagCauucacggg.................................................   5       1   BF2
.............aauggcacuUgaagaauucacggg.................................................   11      1   BF2
.............aauggcacuggaagaaCucacggg.................................................   47      1   BF2
.............aauggcacuggaagaauucacggA.................................................   82      1   BF2
.............aauggcacuggaCgaauucacggg.................................................   1       1   BF2
.............aauggcacuggaagaauucGcggg.................................................   21      1   BF2
.............aauggcacCggaagaauucacggg.................................................   23      1   BF2
.............aauggcacuggaagaauuGacggg.................................................   9       1   BF2
.............aauCgcacuggaagaauucacggga................................................   1       1   BF2
.............aauggGacuggaagaauucacggga................................................   1       1   BF2
.............aauggcacAggaagaauucacggga................................................   2       1   BF2
.............aauggcacuggaagaauucaUggga................................................   4       1   BF2
.............Cauggcacuggaagaauucacggga................................................   1       1   BF2
.............aauggcacuggaagaauucacUgga................................................   1       1   BF2
.............aaugAcacuggaagaauucacggga................................................   1       1   BF2
.............aauggcacGggaagaauucacggga................................................   2       1   BF2
.............aauggcacuggaagaauucacgUga................................................   2       1   BF2
.............aaGggcacuggaagaauucacggga................................................   1       1   BF2
.............Uauggcacuggaagaauucacggga................................................   1       1   BF2
.............aauggcGcuggaagaauucacggga................................................   1       1   BF2
.............aauggUacuggaagaauucacggga................................................   3       1   BF2
.............aauggcaUuggaagaauucacggga................................................   2       1   BF2
.............aauggcacuggaagaaCucacggga................................................   5       1   BF2
.............aauggcacuggGagaauucacggga................................................   4       1   BF2
.............aauggcacuggaagaGuucacggga................................................   2       1   BF2
.............aauggAacuggaagaauucacggga................................................   3       1   BF2
.............aauggcacuggaagaauucacggAa................................................   49      1   BF2
.............aauggcacuUgaagaauucacggga................................................   2       1   BF2
.............aaAggcacuggaagaauucacggga................................................   1       1   BF2
.............aauggcacCggaagaauucacggga................................................   2       1   BF2
.............Gauggcacuggaagaauucacggga................................................   2       1   BF2
.............aauggcacugAaagaauucacggga................................................   11      1   BF2
.............aaugUcacuggaagaauucacggga................................................   2       1   BF2
.............aauggcaGuggaagaauucacggga................................................   1       1   BF2
.............aauggcacuggaagaauucacgggU................................................   296     1   BF2
.............aauggcacuggUagaauucacggga................................................   3       1   BF2
.............aauggcacuggaagaUuucacggga................................................   1       1   BF2
.............aauggcacuggaagaaAucacggga................................................   2       1   BF2
.............aauggcacugCaagaauucacggga................................................   3       1   BF2
.............aauggcacuggaaCaauucacggga................................................   1       1   BF2
.............aauggcacuggaGgaauucacggga................................................   2       1   BF2
.............aauggcacuggaagaauucacgggG................................................   35      1   BF2
.............aauggcacuggCagaauucacggga................................................   4       1   BF2
.............aauggcacuggaagaauCcacggga................................................   4       1   BF2
.............aauggcacuggaagaauucacgAga................................................   1       1   BF2
.............aauggcacuggaaAaauucacggga................................................   1       1   BF2
.............aauggcacuggaagaauucacggUa................................................   1       1   BF2
.............aauAgcacuggaagaauucacggga................................................   2       1   BF2
.............aauggcacugUaagaauucacggga................................................   4       1   BF2
.............aauggcacuggaagaauucUcggga................................................   1       1   BF2
.............aauggcacuggaagaauuUacggga................................................   8       1   BF2
.............aauggcacuggaagaauucacggCa................................................   3       1   BF2
.............aauggcacuggaagaauucacAgga................................................   4       1   BF2
.............aauggcacuggaagaauucacgggC................................................   28      1   BF2
.............aauggcacuggaagaauAcacggga................................................   1       1   BF2
.............aauggcacuggaagaauucacggga................................................   10047   0   BF2
.............aauggcacuggaagaauuGacggga................................................   1       1   BF2
.............aauggcacuggaagGauucacggga................................................   1       1   BF2
.............aauggcacuggaUgaauucacggga................................................   1       1   BF2
.............aGuggcacuggaagaauucacggga................................................   1       1   BF2
.............aauggcacuggaagaauucacgggaC...............................................   9       1   BF2
.............aauggcacuggaagaauucacgggCu...............................................   4       1   BF2
.............aauggcacuggaagaauucacgggaG...............................................   2       1   BF2
.............aaugAcacuggaagaauucacgggau...............................................   1       1   BF2
```

```
             aga-miR-263a
                                                      aga-miR-263a*
cccugguacauguaauggcacuggaagaauucacgggauuuuuuucaacauucccguguucucuuaguggcauaccuaguacaggg

.............aauggcacugCaagaauucacgggau...............................................    1     1    BF2
.............aauggcacuggaagaauucacgggUu...............................................    19    1    BF2
.............aauggcacuggaagaauucacgggau...............................................    184   0    BF2
.............aauggcacuggaagaauucacgggaA...............................................    917   1    BF2
.............aauggcacugAaagaauucacgggau...............................................    1     1    BF2
.............aauggcacuggaagaauucacgggauA..............................................    5     1    BF2
.............aauggcacuggaagaauucacgggaAu..............................................    30    1    BF2
.............aauggcacuggaagaauucacgggUuu..............................................    4     1    BF2
.............aauggcacuggaagaauucacgggauu..............................................    31    0    BF2
.............aauggcacuggaagaauucacgggauuu.............................................    6     0    BF2
.............aauggcacuggaagaauucacgggaAuu.............................................    3     1    BF2
.............aauggcacuggaagaauucacgggUuuu.............................................    1     1    BF2
.............aauggcacuggaagaauucacgggauAu.............................................    1     1    BF2
.............aauggcacuggaagaauucacgggauuAu............................................    1     1    BF2
.............aauggcacuggaagaauucacgggUuuuu............................................    2     1    BF2
.............aauggcacuggaagaauucacgggaAuuu............................................    1     1    BF2
.............aauggcacuggaagaauucacgggauuuu............................................    11    0    BF2
.............aauggcacuggaagaauucacgggauuuA............................................    1     1    BF2
.............aauggcacuggaagaauucacgggauuuuu...........................................    1     0    BF2
.............aauggcacuggaagaauucacgggauuuuuu..........................................    1     0    BF2
.............aauggcacuggaagaauucacgggauuuuuuu.........................................    1     0    BF2
.............aauggcacuggaagaauucacgggauuuuuuucaaAa....................................    1     1    BF2
.............aauggcacuggaagaauucacgggauuuuuuucaacau...................................    1     0    BF2
.............aauggcacuggaagaauucacgggauuuuuuucaacauucc................................    1     0    BF2
..............auggcacuggaagaauucacgg..................................................    4     0    BF2
..............Guggcacuggaagaauucacggg.................................................    2     1    BF2
..............auggcacuggaagaauAcacggg.................................................    1     1    BF2
..............auggcacuggaagaauucacggg.................................................    42    0    BF2
..............auggcacuggaagaauucacggga................................................    57    0    BF2
..............auggcacuggaGgaauucacggga................................................    1     1    BF2
..............auggcacuAgaagaauucacggga................................................    1     1    BF2
..............auggcacuggaagaauucacgggaA...............................................    5     1    BF2
...............uggcacuggaagaauucacggg.................................................    3     0    BF2
...............uggcacuggaagaauucacggga................................................    3     0    BF2
...............uggcacuggaagaauucacgggau...............................................    4     0    BF2
................ggcacuggaagaauucacggg.................................................    4     0    BF2
................ggcacuggaagaauucacggga................................................    1     0    BF2
.................gcacuggaagaauucacgg..................................................    12    0    BF2
.................gcacuggaagaauucacggg.................................................    130   0    BF2
.................gcacuggaagaauucacggA.................................................    1     1    BF2
.................gcacuggaagaauucacAgg.................................................    1     1    BF2
.................gUacuggaagaauucacggg.................................................    1     1    BF2
.................gcacuggaagaauucacggga................................................    13    0    BF2
..................cacuggaagaauucacg...................................................    2     0    BF2
..................cacuggaagaauucacgg..................................................    3     0    BF2
..................cGcuggaagaauucacggg.................................................    1     1    BF2
..................cacuggaagaauucacggg.................................................    11    0    BF2
..................cacuggaagaauucacggga................................................    4     0    BF2
...................acuggaagaauUacggg..................................................    1     1    BF2
...................acuggaagaauucacggg.................................................    2     0    BF2
...................acuggaagaauucacggga................................................    3     0    BF2
...................acuggaagaauucacgggUu...............................................    1     1    BF2
....................cuggaagaauucacggg.................................................    26    0    BF2
....................cuggaagaauucacggga................................................    2     0    BF2
....................cuggaagaauucacgggG................................................    1     1    BF2
.....................................................ccguguucucuuaguggcaua............    1     0    BF2
.....................................................ccguguucucuuaguggcauac...........    3     0    BF2
.....................................................ccguguucucuuaguggcauacc..........    9     0    BF2
.....................................................ccguguucuUuuaguggcauacc..........    1     1    BF2
......................................................cguguucucuuaguggca..............    1     0    BF2
......................................................cguguucucuuaguggcauac...........    2     0    BF2
......................................................cgCguucucuuaguggcauacc..........    1     1    BF2
......................................................cguguucucuCaguggcauacc..........    1     1    BF2
......................................................cguguucucuuaguggcauacU..........    1     1    BF2
......................................................cguguucucuuaguggcauaUc..........    1     1    BF2
......................................................cguguucucuuagAggcauacc..........    1     1    BF2
......................................................cguguucucuuaguAgcauacc..........    1     1    BF2
......................................................cguguucucuuaguggcauacc..........    467   0    BF2
......................................................cguguucucuuaguggcauaccu.........    1     0    BF2
```

```
              aga-miR-263a
                                           aga-miR-263a*
cccugguacauguaauggcacuggaagaauucacgggauuuuuuucaacauucccguguucucuuaguggcauaccuaguacaggg


...........gGaauggcacuggaagaauucacgg..................................................    1     1    BF1
............Caauggcacuggaagaauucac....................................................    1     1    BF1
............Caauggcacuggaagaauucacg...................................................    2     1    BF1
............Caauggcacuggaagaauucacgg..................................................    14    1    BF1
............uaauggcacuggaagaauucacgg..................................................    2     0    BF1
............Aaauggcacuggaagaauucacggg.................................................    2     1    BF1
............Caauggcacuggaagaauucacggg.................................................    48    1    BF1
............Gaauggcacuggaagaauucacggg.................................................    1     1    BF1
............uaauggcacuggaagaauucacggg.................................................    1     0    BF1
............Caauggcacuggaagaauucacggga................................................    3     1    BF1
.............aauggcacuggaagaau........................................................    1     0    BF1
.............aauggcacuggaagaauu.......................................................    3     0    BF1
.............aauggcacuggaagaauuca.....................................................    60    0    BF1
.............aauggcacCggaagaauucac....................................................    1     1    BF1
.............aaAggcacuggaagaauucac....................................................    1     1    BF1
.............Gauggcacuggaagaauucac....................................................    1     1    BF1
.............aauggcacuggaagaauucaA....................................................    2     1    BF1
.............aauggcacuggGagaauucac....................................................    1     1    BF1
.............aauggAacuggaagaauucac....................................................    1     1    BF1
.............aauggcacuggaagaauucac....................................................    412   0    BF1
.............aauggcacuggGagaauucacg...................................................    1     1    BF1
.............aauggcaUuggaagaauucacg...................................................    2     1    BF1
.............aauggcacuggaagaauucacg...................................................    1606  0    BF1
.............aauggcacugAaagaauucacg...................................................    2     1    BF1
.............aauggcacugUaagaauucacg...................................................    1     1    BF1
.............aauggcacuggaagaauucacU...................................................    2     1    BF1
.............aauggcacuggaagaauucacA...................................................    7     1    BF1
.............aauggcacuggaaAaauucacg...................................................    1     1    BF1
.............aauggcacuggaagaauucaUg...................................................    1     1    BF1
.............aGuggcacuggaagaauucacg...................................................    1     1    BF1
.............aauggcacuggCagaauucacg...................................................    1     1    BF1
.............aUuggcacuggaagaauucacg...................................................    1     1    BF1
.............aauggcacuggaagaauuUacg...................................................    2     1    BF1
.............aauUgcacuggaagaauucacg...................................................    1     1    BF1
.............aauggcacCggaagaauucacg...................................................    1     1    BF1
.............aauggcacuggaagaauucaGg...................................................    1     1    BF1
.............aauggcacuggaagaauuAacgg..................................................    2     1    BF1
.............aauggcacuggaagaaCucacgg..................................................    6     1    BF1
.............aauggcacuggUagaauucacgg..................................................    12    1    BF1
.............aauggcUcuggaagaauucacgg..................................................    1     1    BF1
.............aauggcacuggaagaauAcacgg..................................................    5     1    BF1
.............aauCgcacuggaagaauucacgg..................................................    1     1    BF1
.............aaGggcacuggaagaauucacgg..................................................    3     1    BF1
.............aauggcacuggaagaaGucacgg..................................................    1     1    BF1
.............aauggAacuggaagaauucacgg..................................................    3     1    BF1
.............aauggcacGggaagaauucacgg..................................................    2     1    BF1
.............aauggcacuggaagaGuucacgg..................................................    1     1    BF1
.............aauggcacuggaagaauucacgC..................................................    2     1    BF1
.............aauAgcacuggaagaauucacgg..................................................    11    1    BF1
.............Uauggcacuggaagaauucacgg..................................................    1     1    BF1
.............aauggcacuggaagaauucaUgg..................................................    8     1    BF1
.............aaCggcacuggaagaauucacgg..................................................    6     1    BF1
.............aauggcacugCaagaauucacgg..................................................    12    1    BF1
.............aauggcacugAaagaauucacgg..................................................    32    1    BF1
.............aauggcGcuggaagaauucacgg..................................................    5     1    BF1
.............aauggcacuggaUgaauucacgg..................................................    1     1    BF1
.............aauggcacuggaagaUuucacgg..................................................    1     1    BF1
.............aauggcacuggaagaaAucacgg..................................................    1     1    BF1
.............aauggcaUuggaagaauucacgg..................................................    5     1    BF1
.............aauggUacuggaagaauucacgg..................................................    6     1    BF1
.............aauggcacuggaGgaauucacgg..................................................    1     1    BF1
.............aaugAcacuggaagaauucacgg..................................................    5     1    BF1
.............aauggcacuUgaagaauucacgg..................................................    3     1    BF1
.............aauggcacuggaaUaauucacgg..................................................    2     1    BF1
.............aauggcacuggaagaauucacUg..................................................    2     1    BF1
.............aauggcacuggaagaauucGcgg..................................................    6     1    BF1
.............aauggcacuggaagaauucacgU..................................................    3     1    BF1
.............aauggcacugUaagaauucacgg..................................................    7     1    BF1
```

```
             aga-miR-263a
                                             aga-miR-263a*
cccugguacauguaauggcacuggaagaauucacgggauuuuuuucaacauucccguguucucuuaguggcauaccuaguacaggg

.............aauggcacuggaagaauucUcgg..................................................    1       1    BF1
.............aauggcaAuggaagaauucacgg..................................................    2       1    BF1
.............aauggcacuggaagaauucacgA..................................................    32      1    BF1
.............aaAggcacuggaagaauucacgg..................................................    2       1    BF1
.............aauggcacuggaaCaauucacgg..................................................    1       1    BF1
.............aauggcacuggaagaauucacAg..................................................    8       1    BF1
.............aauggcacuggCagaauucacgg..................................................    7       1    BF1
.............aauggcacuAgaagaauucacgg..................................................    8       1    BF1
.............aauggcacuggaagaauuUacgg..................................................    17      1    BF1
.............aCuggcacuggaagaauucacgg..................................................    2       1    BF1
.............aauggcacuggaagaauucacgg..................................................    23196   0    BF1
.............aauggGacuggaagaauucacgg..................................................    1       1    BF1
.............Gauggcacuggaagaauucacgg..................................................    4       1    BF1
.............aauggcacCggaagaauucacgg..................................................    3       1    BF1
.............aauggcacuggaagaauucaAgg..................................................    2       1    BF1
.............aauggcacuggaaAaauucacgg..................................................    4       1    BF1
.............aauggcacuCgaagaauucacgg..................................................    1       1    BF1
.............aauggcacuggGagaauucacgg..................................................    7       1    BF1
.............aaugUcacuggaagaauucacgg..................................................    4       1    BF1
.............aUuggcacuggaagaauucacgg..................................................    1       1    BF1
.............aGuggcacuggaagaauucacgg..................................................    1       1    BF1
.............aauggcacuggaagGauucacgg..................................................    2       1    BF1
.............aauggcacuggaagaauCcacgg..................................................    6       1    BF1
.............aauggcaAuggaagaauucacggg.................................................    3       1    BF1
.............aauggcacuggaagaauucaGggg.................................................    3       1    BF1
.............aauggcacCggaagaauucacggg.................................................    22      1    BF1
.............aauggcacuggaagaauuUacggg.................................................    47      1    BF1
.............aauUgcacuggaagaauucacggg.................................................    2       1    BF1
.............aauggcacuggaUgaauucacggg.................................................    3       1    BF1
.............aauggcacuUgaagaauucacggg.................................................    12      1    BF1
.............aauggcacuggaagaauucacUgg.................................................    6       1    BF1
.............aauggcacuggaagaauucacAgg.................................................    23      1    BF1
.............aauggcacuggaagaauucCcggg.................................................    6       1    BF1
.............aauggcacuggaGgaauucacggg.................................................    12      1    BF1
.............aauggcacuggaagGauucacggg.................................................    10      1    BF1
.............aauggcacuggaagaGuucacggg.................................................    12      1    BF1
.............aauggcacuggaaAaauucacggg.................................................    14      1    BF1
.............aauggcacuggaagaauucUcggg.................................................    6       1    BF1
.............Cauggcacuggaagaauucacggg.................................................    7       1    BF1
.............aUuggcacuggaagaauucacggg.................................................    7       1    BF1
.............aauggcUcuggaagaauucacggg.................................................    2       1    BF1
.............aauggAacuggaagaauucacggg.................................................    9       1    BF1
.............aaugCcacuggaagaauucacggg.................................................    3       1    BF1
.............aauggcacuggaaUaauucacggg.................................................    5       1    BF1
.............aauggcacuggaagaauucacggC.................................................    23      1    BF1
.............aauggcCcuggaagaauucacggg.................................................    1       1    BF1
.............aauggcacuggaagaauucGcggg.................................................    10      1    BF1
.............aaugAcacuggaagaauucacggg.................................................    25      1    BF1
.............aauggcacuggaagaauucacggU.................................................    15      1    BF1
.............aauggcacuggaagaauucaAggg.................................................    3       1    BF1
.............aauggcGcuggaagaauucacggg.................................................    9       1    BF1
.............aCuggcacuggaagaauucacggg.................................................    2       1    BF1
.............Gauggcacuggaagaauucacggg.................................................    11      1    BF1
.............aaugUcacuggaagaauucacggg.................................................    9       1    BF1
.............aauggcacuggaagaauuGacggg.................................................    2       1    BF1
.............aauggcacAggaagaauucacggg.................................................    10      1    BF1
.............aauggcacuggaagaauucacgUg.................................................    3       1    BF1
.............aauggcacuggaagaauucacggA.................................................    120     1    BF1
.............Uauggcacuggaagaauucacggg.................................................    10      1    BF1
.............aauggUacuggaagaauucacggg.................................................    23      1    BF1
.............aauggcacuCgaagaauucacggg.................................................    4       1    BF1
.............aauggcacuggaagaauucacgAg.................................................    23      1    BF1
.............aauggcacuggaagaauucacggg.................................................    99391   0    BF1
.............aauggcacuggaagUauucacggg.................................................    1       1    BF1
.............aGuggcacuggaagaauucacggg.................................................    7       1    BF1
.............aauggcacuggaagaauuAacggg.................................................    8       1    BF1
.............aauggcacuggUagaauucacggg.................................................    26      1    BF1
.............aauAgcacuggaagaauucacggg.................................................    37      1    BF1
.............aauggcacuggaagaauCcacggg.................................................    26      1    BF1
```

```
             aga-miR-263a
                                                        aga-miR-263a*
cccugguacauguaauggcacuggaagaauucacgggauuuuuuucaacauucccguguucucuuaguggcauaccuaguacaggg

.............aauggcacuggCagaauucacggg.................................................   32      1    BF1
.............aauggcacuggaagaaAucacggg.................................................   6       1    BF1
.............aaAggcacuggaagaauucacggg.................................................   7       1    BF1
.............aauCgcacuggaagaauucacggg.................................................   4       1    BF1
.............aauggcacugCaagaauucacggg.................................................   32      1    BF1
.............aauggcacuggaaCaauucacggg.................................................   3       1    BF1
.............aauggcacuggaagaauucacCgg.................................................   5       1    BF1
.............aauggcacuAgaagaauucacggg.................................................   23      1    BF1
.............aauggcacugAaagaauucacggg.................................................   107     1    BF1
.............aaGggcacuggaagaauucacggg.................................................   3       1    BF1
.............aauggcaUuggaagaauucacggg.................................................   12      1    BF1
.............aauggcacGgaagaauucacggg.................................................   5       1    BF1
.............aauggcacuggaagaauAcacggg.................................................   7       1    BF1
.............aauggcacuggaagaCuucacggg.................................................   4       1    BF1
.............aauggcacuggaagaauGcacggg.................................................   1       1    BF1
.............aauggcacugUaagaauucacggg.................................................   38      1    BF1
.............aauggcacuggaagaauucacgCg.................................................   2       1    BF1
.............aauggcacuggaagaaCucacggg.................................................   47      1    BF1
.............aauggcacuggaagaauucaUggg.................................................   34      1    BF1
.............aauggGacuggaagaauucacggg.................................................   2       1    BF1
.............aauggcacuggGagaauucacggg.................................................   30      1    BF1
.............aaCggcacuggaagaauucacggg.................................................   35      1    BF1
.............aauggcacugUaagaauucacggga................................................   6       1    BF1
.............aauggcacuggaagaCuucacggga................................................   1       1    BF1
.............aauggcacCggaagaauucacggga................................................   2       1    BF1
.............aaugAcacuggaagaauucacggga................................................   3       1    BF1
.............Uauggcacuggaagaauucacggga................................................   1       1    BF1
.............aauggcGcuggaagaauucacggga................................................   3       1    BF1
.............aauggcacuggaagaauucacggCa................................................   2       1    BF1
.............aauggcacuggaagaauucacggga................................................   11091   0    BF1
.............aauggcaAuggaagaauucacggga................................................   1       1    BF1
.............aauggcacuggaagaauGcacggga................................................   1       1    BF1
.............aaCggcacuggaagaauucacggga................................................   3       1    BF1
.............aauCgcacuggaagaauucacggga................................................   1       1    BF1
.............aauggcacuggaagaauucacgAga................................................   1       1    BF1
.............aauggcacuggaagaauuGacggga................................................   1       1    BF1
.............aauggcacuUgaagaauucacggga................................................   1       1    BF1
.............aauAgcacuggaagaauucacggga................................................   11      1    BF1
.............aauggcacuggaagaaCucacggga................................................   8       1    BF1
.............aauggcacuggGagaauucacggga................................................   1       1    BF1
.............aauggcacuAgaagaauucacggga................................................   5       1    BF1
.............aUuggcacuggaagaauucacggga................................................   1       1    BF1
.............aauggcacuggaaAaauucacggga................................................   1       1    BF1
.............Gauggcacuggaagaauucacggga................................................   1       1    BF1
.............aauggcacuggaagaauuUacggga................................................   3       1    BF1
.............aauggcacuggaagaauucacgggU................................................   509     1    BF1
.............aauggcacuggaagaauucaUggga................................................   7       1    BF1
.............aauggcacAggaagaauucacggga................................................   1       1    BF1
.............aauggcacuggaagaGuucacggga................................................   3       1    BF1
.............aauggAacuggaagaauucacggga................................................   3       1    BF1
.............aauggcacuggaagaauucacgggG................................................   44      1    BF1
.............aauggcacuggaagaauucacAgga................................................   2       1    BF1
.............aauggcacuggaagaauuAacggga................................................   1       1    BF1
.............aauggcacuggUagaauucacggga................................................   6       1    BF1
.............aauggcacuggaagaauucacUgga................................................   1       1    BF1
.............aauggcacuggaagaauucacggAa................................................   74      1    BF1
.............aaAggcacuggaagaauucacggga................................................   1       1    BF1
.............aauggcacuggaagGauucacggga................................................   1       1    BF1
.............aauggcacuggaagaauucacgggC................................................   42      1    BF1
.............aauggcUcuggaagaauucacggga................................................   2       1    BF1
.............aauggcacuggaagaauucacggUa................................................   2       1    BF1
.............aauggcacuggaagaauucGcggga................................................   6       1    BF1
.............aauggcacugCaagaauucacggga................................................   3       1    BF1
.............aauggcacugAaagaauucacggga................................................   10      1    BF1
.............aauggcaUuggaagaauucacggga................................................   3       1    BF1
.............aauggGacuggaagaauucacggga................................................   2       1    BF1
.............aauggcacuggaagaauucacgggCu...............................................   7       1    BF1
.............aauggcacuggaagaauucacgggUu...............................................   50      1    BF1
.............aauggcacuggaagaauucacgggaG...............................................   2       1    BF1
```

```
             aga-miR-263a
                                                      aga-miR-263a*
cccugguacauguaauggcacuggaagaauucacgggauuuuuuucaacauucccguguucucuuaguggcauaccuaguacaggg

.............aauggcacCggaagaauucacgggau...............................................  1     1  BF1
.............aauggcacuggaagaauucacgggau...............................................  237   0  BF1
.............aauggcacuggaagaauucacgggaA................................................  1094  1  BF1
.............aauggcacuggaagaauucacgggaC................................................  17    1  BF1
.............aauggcacuggaagaauCcacgggau...............................................  1     1  BF1
.............aauggcacugAaagaauucacgggau...............................................  1     1  BF1
.............aaAggcacuggaagaauucacgggau...............................................  1     1  BF1
.............aauggcacuggaaAaauucacgggau...............................................  1     1  BF1
.............aauggcacuggaagaauucacgggauA..............................................  8     1  BF1
.............aauggcacuggaagaauucacgggauu..............................................  36    0  BF1
.............aauggcacuggaagaauucacgggUuu..............................................  10    1  BF1
.............aauggcacuggaagaauucacgggaAu..............................................  70    1  BF1
.............aauggcacuggaagaauucacgggaCu..............................................  2     1  BF1
.............aauggcacuggaagaauucacgggaAuu.............................................  4     1  BF1
.............aauggcacuggaagaauucacgggauAu.............................................  9     1  BF1
.............aauggcacuggaagaauucacgggUuuu.............................................  5     1  BF1
.............aauggcacuggaagaauucacgggauuu.............................................  17    0  BF1
.............aauggcacuggaagaauucacgggauuuA............................................  1     1  BF1
.............aauggcacuggaagaauucacgggauuAu............................................  1     1  BF1
.............aauggcacuggaagaauucacgggaAuuu............................................  2     1  BF1
.............aauggcacuggaagaauucacgggauuuC............................................  1     1  BF1
.............aauggcacuggaagaauucacgggUuuuu............................................  1     1  BF1
.............aauggcacuggaagaauucacgggauuuu............................................  6     0  BF1
.............aauggcacuggaagaauucacgggauAuu............................................  1     1  BF1
.............aauggcacuggaagaauucacgggCuuuuu...........................................  1     1  BF1
.............aauggcacuggaagaauucacgggauuAuu...........................................  1     1  BF1
.............aauggcacuggaagaauucacgggauuuuu...........................................  1     0  BF1
.............aauggcacuggaagaauucacgggUuuuuu...........................................  2     1  BF1
.............aauggcacuggaagaauucacgggauuuuuu..........................................  2     0  BF1
.............aauggcacuggaagaauucacgggauuuuuuuc........................................  1     0  BF1
.............aauggcacuggaagaauucacgggauuuuuuucaaca....................................  1     0  BF1
.............aauggcacuggaagaauucacgggauuuuuuucaacau...................................  1     0  BF1
.............aauggcacuggaagaauucacgggauuuuuuucaacauucc................................  1     0  BF1
..............auggcacuggaagaauucac....................................................  1     0  BF1
..............auggcacuggaagaauucacg...................................................  2     0  BF1
..............auggcacuggaagaauucacgg..................................................  9     0  BF1
..............auggcacuggaagaauucaUgg..................................................  1     1  BF1
..............auggcacuggaagaauucacggg.................................................  29    0  BF1
..............auggcacGggaagaauucacggg.................................................  1     1  BF1
..............auggcacuggaagaauucacggga................................................  71    0  BF1
..............auggcacuggaagaauucacgggau...............................................  2     0  BF1
..............auggcacuggaagaauucacgggaA...............................................  4     1  BF1
...............uggcacuggaagaauucacggg.................................................  2     0  BF1
...............uggcacuggaagaauucacggga................................................  3     0  BF1
...............uggcacuggaagaauucacgggau...............................................  6     0  BF1
.................gcacuggaagaauucacggg.................................................  1     0  BF1
..................cacuggaagaauucacggga................................................  1     0  BF1
....................cuggaagaauucacggg.................................................  2     0  BF1
.....................................................ccguguucucuuaguggcauacc..........  6     0  BF1
......................................................cguguucucuuaguggcauacc..........  88    0  BF1
......................................................cguguucucuuagCggcauacc..........  1     1  BF1
.......................................................guguucucuuaguggcauacc..........  1     0  BF1

............Caauggcacuggaagaauucacgg...................................................  2     1  FW1
............Caauggcacuggaagaauucacggg.................................................  7     1  FW1
............Caauggcacuggaagaauucacggga................................................  1     1  FW1
.............aauggcacuggaagaauu.......................................................  1     0  FW1
.............aauggcacuggaagaauuca.....................................................  18    0  FW1
.............aauAgcacuggaagaauucac....................................................  1     1  FW1
.............aauggcacuggGagaauucac....................................................  1     1  FW1
.............Cauggcacuggaagaauucac....................................................  1     1  FW1
.............aauggcacuggaagaauucac....................................................  219   0  FW1
.............aGuggcacuggaagaauucacg...................................................  1     1  FW1
.............aauggcacuggaagaauAcacg...................................................  1     1  FW1
.............aauggcacuggaagaauucacU...................................................  1     1  FW1
.............aauggcacuggaagaauucacA...................................................  1     1  FW1
.............aauggcacugUaagaauucacg...................................................  2     1  FW1
.............aauggcacuggaagaGuucacg...................................................  1     1  FW1
.............aauggcacuggaagGauucacg...................................................  1     1  FW1
```

```
                  aga-miR-263a
                                                          aga-miR-263a*
cccugguacauguaauggcacuggaagaauucacgggauuuuuuucaacauucccguguucucuuaguggcauaccuaguacaggg

.............aauggcacuggaagaauucacg...................................................  571    0    FW1
.............aaugAcacuggaagaauucacg...................................................  1      1    FW1
.............aauggcacuggaagaauuAacg...................................................  1      1    FW1
.............aauggcacugCaagaauucacgg..................................................  2      1    FW1
.............aauggcacuggaagaauucacgg..................................................  6128   0    FW1
.............aauggUacuggaagaauucacgg..................................................  1      1    FW1
.............aauggcacuggaagaaCucacgg..................................................  5      1    FW1
.............aauggcacuggaagaGuucacgg..................................................  1      1    FW1
.............aaGggcacuggaagaauucacgg..................................................  1      1    FW1
.............aauggcacuggaagaauucacAg..................................................  1      1    FW1
.............aauggcacuggaagaauucacgA..................................................  5      1    FW1
.............aUuggcacuggaagaauucacgg..................................................  1      1    FW1
.............aauggcacuggaagaauCcacgg..................................................  2      1    FW1
.............aauggcacuggaagaauucaUgg..................................................  1      1    FW1
.............aauggcacCggaagaauucacgg..................................................  1      1    FW1
.............aauggcacuggaagaaGucacgg..................................................  1      1    FW1
.............aauggcacuggaagaauucaAgg..................................................  1      1    FW1
.............aGuggcacuggaagaauucacgg..................................................  1      1    FW1
.............aauAgcacuggaagaauucacgg..................................................  4      1    FW1
.............aauggcacuggaagaauucacUg..................................................  2      1    FW1
.............aaAggcacuggaagaauucacgg..................................................  1      1    FW1
.............aauggcacuggaagaauuAacgg..................................................  1      1    FW1
.............Gauggcacuggaagaauucacgg..................................................  2      1    FW1
.............aauggcacuggaagUauucacgg..................................................  1      1    FW1
.............aaCggcacuggaagaauucacgg..................................................  3      1    FW1
.............aauggcacugAaagaauucacgg..................................................  3      1    FW1
.............aauggcacuAgaagaauucacgg..................................................  1      1    FW1
.............aauggcacuggGagaauucacgg..................................................  1      1    FW1
.............aauggcacuggaagaauuUacgg..................................................  5      1    FW1
.............aauggcacuUgaagaauucacgg..................................................  1      1    FW1
.............aaugAcacuggaagaauucacgg..................................................  1      1    FW1
.............aauggcacuggCagaauucacgg..................................................  2      1    FW1
.............aauggcUcuggaagaauucacgg..................................................  1      1    FW1
.............aauggcacugUaagaauucacgg..................................................  3      1    FW1
.............aauggcacuggUagaauucacgg..................................................  1      1    FW1
.............aauggcGcuggaagaauucacggg.................................................  1      1    FW1
.............aauggcacGggaagaauucacggg.................................................  1      1    FW1
.............aauggcacuggaagaauuUacggg.................................................  12     1    FW1
.............aauggcacuggaagaaGucacggg.................................................  1      1    FW1
.............aaAggcacuggaagaauucacggg.................................................  1      1    FW1
.............aauggcacuggaagaauucacAgg.................................................  3      1    FW1
.............aauggcacuggaagaauucGcggg.................................................  1      1    FW1
.............Cauggcacuggaagaauucacggg.................................................  2      1    FW1
.............aauggcaUuggaagaauucacggg.................................................  2      1    FW1
.............aauggcacugCaagaauucacggg.................................................  3      1    FW1
.............aauggcacuggaagaauucacggg.................................................  15222  0    FW1
.............aauggcacugAaagaauucacggg.................................................  15     1    FW1
.............aauggcacuggaagaauucaAggg.................................................  1      1    FW1
.............aauggcacuggaagaauucacggC.................................................  12     1    FW1
.............aaugCcacuggaagaauucacggg.................................................  1      1    FW1
.............aauggcacuggUagaauucacggg.................................................  4      1    FW1
.............aauggcacuggaagaauucacgAg.................................................  2      1    FW1
.............aauggcUcuggaagaauucacggg.................................................  2      1    FW1
.............aauggcacuggaGgaauucacggg.................................................  1      1    FW1
.............aauggcacuggaagaauCcacggg.................................................  7      1    FW1
.............aauggcaGuggaagaauucacggg.................................................  1      1    FW1
.............aauggcacCggaagaauucacggg.................................................  3      1    FW1
.............aauggcacuggaagGauucacggg.................................................  2      1    FW1
.............Gauggcacuggaagaauucacggg.................................................  5      1    FW1
.............aauggcacuggaagaauAcacggg.................................................  2      1    FW1
.............aauUgcacuggaagaauucacggg.................................................  2      1    FW1
.............aauggcacAggaagaauucacggg.................................................  2      1    FW1
.............aauggcacuggaagaauucacgUg.................................................  2      1    FW1
.............aaGggcacuggaagaauucacggg.................................................  1      1    FW1
.............aauggcacuggaagaauucacggA.................................................  18     1    FW1
.............aauggcacuggaagaauucacCgg.................................................  1      1    FW1
.............aauggcacuggaagaaCucacggg.................................................  11     1    FW1
.............Uauggcacuggaagaauucacggg.................................................  1      1    FW1
.............aauggcacuggaaAaauucacggg.................................................  1      1    FW1
```

```
                 aga-miR-263a
                                                   aga-miR-263a*
cccugguacauguaauggcacuggaagaauucacgggauuuuuuucaacauucccguguucucuuaguggcauaccuaguacaggg

.............aauggcacugUaagaauucacggg.................................................    3       1       FW1
.............aauggcacuggaagaauuGacggg.................................................    1       1       FW1
.............aaCggcacuggaagaauucacggg.................................................    5       1       FW1
.............aauggcacuggaagaauucacggU.................................................    6       1       FW1
.............aauggcacuggaaUaauucacggg.................................................    1       1       FW1
.............aauggUacuggaagaauucacggg.................................................    4       1       FW1
.............aauggcacuggaagaauucUcggg.................................................    1       1       FW1
.............aauggGacuggaagaauucacggg.................................................    1       1       FW1
.............aauggcacuggCagaauucacggg.................................................    4       1       FW1
.............aauggcacuggaagaauucaUggg.................................................    7       1       FW1
.............aaugAcacuggaagaauucacggg.................................................    2       1       FW1
.............aUuggcacuggaagaauucacggg.................................................    1       1       FW1
.............aauggcacuggGagaauucacggg.................................................    6       1       FW1
.............aGuggcacuggaagaauucacggg.................................................    1       1       FW1
.............aauggcacuggaagaauucacUgg.................................................    3       1       FW1
.............aauAgcacuggaagaauucacggg.................................................    6       1       FW1
.............aauggcacuAgaagaauucacggg.................................................    3       1       FW1
.............aaCggcacuggaagaauucacggga................................................    1       1       FW1
.............aauggcacuggUagaauucacggga................................................    1       1       FW1
.............aauggcacuggaagaauucaGggga................................................    1       1       FW1
.............aauggcacugAaagaauucacggga................................................    1       1       FW1
.............aauggcacuggaagaaCucacggga................................................    1       1       FW1
.............aauggcacuggCagaauucacggga................................................    1       1       FW1
.............aauggcacuggaagaauucacggAa................................................    6       1       FW1
.............aauggcacuggaagaauucacggga................................................    1526    0       FW1
.............aauggcacuggaagaauuUacggga................................................    1       1       FW1
.............aaugUcacuggaagaauucacggga................................................    1       1       FW1
.............aaAggcacuggaagaauucacggga................................................    1       1       FW1
.............aauAgcacuggaagaauucacggga................................................    1       1       FW1
.............aauggcacuggaagaauucacgggU................................................    181     1       FW1
.............aauggcacuggaagaauucacgggG................................................    9       1       FW1
.............aauggUacuggaagaauucacggga................................................    2       1       FW1
.............aauggcacuggaagaauucacgggC................................................    10      1       FW1
.............aauggcacuggaagaGuucacggga................................................    1       1       FW1
.............aauggcacuggaagaauucacgggau...............................................    54      0       FW1
.............aauggcacuggaagaauucacgggUu...............................................    11      1       FW1
.............aauggcacuggaagaauucacgggaA...............................................    98      1       FW1
.............aauggcacuggaagaauucacgggCu...............................................    1       1       FW1
.............aauggcacuggaagaauucacgggaC...............................................    1       1       FW1
.............aauggcacuggaagaauucacgggUuu..............................................    1       1       FW1
.............aauggcacuggaagaauucacgggaAu..............................................    4       1       FW1
.............aauggcacuggaagaauucacgggaCu..............................................    1       1       FW1
.............aauggcacuggaagaauucacgggauA..............................................    4       1       FW1
.............aauggcacuggaagaauucacgggauu..............................................    1       0       FW1
.............aauggcacuggaagaauucacgggUuuu.............................................    1       1       FW1
.............aauggcacuggaagaauucacgggUuuuu............................................    1       1       FW1
.............aauggcacuggaagaauucacgggauuuu............................................    2       0       FW1
.............aauggcacuggaagaauucacgggauuuuu...........................................    1       0       FW1
..............auggcacuggaagaauucacgg..................................................    3       0       FW1
..............auggcacuggaagaauucacggg.................................................    4       0       FW1
..............auggUacuggaagaauucacggg.................................................    1       1       FW1
..............auggcacuggaagaauucacggga................................................    4       0       FW1
................ggcacuggaagaauucacgg..................................................    1       0       FW1
.................gcacuggaagaauucacgg..................................................    2       0       FW1
...................acuggaagaauucacgg..................................................    1       0       FW1
.....................................................ccguguucucuuaguggcauacc..........    3       0       FW1
......................................................cguguucucuuaguggcauaUc..........    1       1       FW1
......................................................cguguucucuGaguggcauacc..........    1       1       FW1
......................................................cguguucuUuuaguggcauacc..........    1       1       FW1
......................................................cguguucucuuaguggcauacc..........    99      0       FW1
......................................................Gguguucucuuaguggcauacc..........    1       1       FW1

............Caauggcacuggaagaauucacgg..................................................    1       1       MW1
............Aaauggcacuggaagaauucacgg..................................................    1       1       MW1
............Caauggcacuggaagaauucacggg.................................................    5       1       MW1
............Caauggcacuggaagaauucacggga................................................    1       1       MW1
.............aauggcacuggaagaau........................................................    10      0       MW1
.............aauggcacuggaagaauu.......................................................    13      0       MW1
.............aauggcacuggaagaauuc......................................................    1       0       MW1
```

```
             aga-miR-263a
                                                      aga-miR-263a*
cccugguacauguaauggcacuggaagaauucacgggauuuuuuucaacauucccguguucucuuaguggcauaccuaguacaggg

.............aauggcacuggaagaauuca.....................................................  52     0     MW1
.............aauggcacuggaaUaauucac....................................................  1      1     MW1
.............aauggcacuggaagaauucac....................................................  376    0     MW1
.............aauggcacuggaagaaCucac....................................................  1      1     MW1
.............aauggcacuggCagaauucac....................................................  1      1     MW1
.............aauggcacuggaagaauucacU...................................................  1      1     MW1
.............aauAgcacuggaagaauucacg...................................................  2      1     MW1
.............aauggcacuggaagaauucacA...................................................  1      1     MW1
.............aauggcacugCaagaauucacg...................................................  1      1     MW1
.............aauggUacuggaagaauucacg...................................................  1      1     MW1
.............aauggcacuggUagaauucacg...................................................  1      1     MW1
.............aauggcacuggaagaauucacg...................................................  995    0     MW1
.............aauggcacCggaagaauucacg...................................................  1      1     MW1
.............aauggcacuggaagaGuucacg...................................................  1      1     MW1
.............aauggAacuggaagaauucacgg..................................................  3      1     MW1
.............aauggcacuggaGgaauucacgg..................................................  1      1     MW1
.............aauggcacuggaaAaauucacgg..................................................  2      1     MW1
.............aGuggcacuggaagaauucacgg..................................................  2      1     MW1
.............Cauggcacuggaagaauucacgg..................................................  1      1     MW1
.............aauggcacuggaagaauCcacgg..................................................  1      1     MW1
.............aauggcacuggaagaaAucacgg..................................................  1      1     MW1
.............aauggcacuggaagaauucacUg..................................................  1      1     MW1
.............aauAgcacuggaagaauucacgg..................................................  1      1     MW1
.............aauggcacuggaagaauAcacgg..................................................  1      1     MW1
.............aauggcacuggGagaauucacgg..................................................  4      1     MW1
.............aauggcacuggaagaauucGcgg..................................................  4      1     MW1
.............aauggcacuggaagaauucacgU..................................................  2      1     MW1
.............aauggcacuggaagaauucacgC..................................................  5      1     MW1
.............aauggcacuggaagaauuUacgg..................................................  5      1     MW1
.............aauggcacuggaagaGuucacgg..................................................  1      1     MW1
.............aauggcacuggaagaauuAacgg..................................................  1      1     MW1
.............aauggcacugUaagaauucacgg..................................................  4      1     MW1
.............aauggcacuUgaagaauucacgg..................................................  1      1     MW1
.............aauggcacuggaagaaCucacgg..................................................  3      1     MW1
.............aauggcacCggaagaauucacgg..................................................  1      1     MW1
.............aUuggcacuggaagaauucacgg..................................................  1      1     MW1
.............aauggcacugCaagaauucacgg..................................................  3      1     MW1
.............aauggUacuggaagaauucacgg..................................................  1      1     MW1
.............aauggcacuggaagaauucacAg..................................................  3      1     MW1
.............aauggcacuggaagaauucaUgg..................................................  1      1     MW1
.............aauggcaUuggaagaauucacgg..................................................  1      1     MW1
.............aaugAcacuggaagaauucacgg..................................................  1      1     MW1
.............aauggcacuggCagaauucacgg..................................................  2      1     MW1
.............aauggcacuggaagaauucacgA..................................................  4      1     MW1
.............aauggcacuAgaagaauucacgg..................................................  4      1     MW1
.............aauggcacugAaagaauucacgg..................................................  8      1     MW1
.............aauggcacuggaagaauucacgg..................................................  7334   0     MW1
.............aauggcacCggaagaauucacggg.................................................  6      1     MW1
.............aauggcacuggaagaCuucacggg.................................................  1      1     MW1
.............aaGggcacuggaagaauucacggg.................................................  3      1     MW1
.............aauggcacuggaagaauAcacggg.................................................  1      1     MW1
.............aauggcacuggCagaauucacggg.................................................  1      1     MW1
.............aauggAacuggaagaauucacggg.................................................  6      1     MW1
.............aauggcGcuggaagaauucacggg.................................................  3      1     MW1
.............aauggcacuggaagaauCcacggg.................................................  6      1     MW1
.............aauggcacuggaagaGuucacggg.................................................  3      1     MW1
.............aauggcacuggaagaauucacggC.................................................  23     1     MW1
.............aauggcacuggaagaauucacgUg.................................................  1      1     MW1
.............aauggcacuggaagGauucacggg.................................................  2      1     MW1
.............aaAggcacuggaagaauucacggg.................................................  4      1     MW1
.............aauggcacuggGagaauucacggg.................................................  5      1     MW1
.............aauggcacuCgaagaauucacggg.................................................  1      1     MW1
.............aauggcacuggaagaauuUacggg.................................................  14     1     MW1
.............aauggcacuggaagaauucaUggg.................................................  3      1     MW1
.............aauCgcacuggaagaauucacggg.................................................  1      1     MW1
.............aauggcacuggaagaauucaGggg.................................................  3      1     MW1
.............aaugCcacuggaagaauucacggg.................................................  1      1     MW1
.............aauggcacuggaagaauuAacggg.................................................  2      1     MW1
.............aauggcacugCaagaauucacggg.................................................  5      1     MW1
```

```
             aga-miR-263a
                                                                          aga-miR-263a*
cccugguacauguaauggcacuggaagaauucacgggauuuuuuucaacauucccguguucucuuaguggcauaccuaguacaggg

.............aauggcaUuggaagaauucacggg.................................................  2       1   MW1
.............aauggcacuggaagaauucacggU.................................................  8       1   MW1
.............aaugAcacuggaagaauucacggg.................................................  3       1   MW1
.............aauggcacuAgaagaauucacggg.................................................  8       1   MW1
.............aauggcacuggaagaauucacAgg.................................................  3       1   MW1
.............aUuggcacuggaagaauucacggg.................................................  1       1   MW1
.............Cauggcacuggaagaauucacggg.................................................  3       1   MW1
.............aauggcacuggaagaaCucacggg.................................................  12      1   MW1
.............aauggcacuggaagaauucaAggg.................................................  1       1   MW1
.............aauggUacuggaagaauucacggg.................................................  3       1   MW1
.............aauggcacuggaagaauucGcggg.................................................  6       1   MW1
.............aauggcacuggaaAaauucacggg.................................................  2       1   MW1
.............aauggcaAuggaagaauucacggg.................................................  1       1   MW1
.............aauggcacugUaagaauucacggg.................................................  12      1   MW1
.............aauggcacuggaagaauuGacggg.................................................  1       1   MW1
.............Gauggcacuggaagaauucacggg.................................................  2       1   MW1
.............aauggcacuggaagaauucacggg.................................................  22105   0   MW1
.............aauggcacuggaagaauucacgCg.................................................  1       1   MW1
.............aauggGacuggaagaauucacggg.................................................  2       1   MW1
.............aauggcacuggaagaauucacgAg.................................................  8       1   MW1
.............aauUgcacuggaagaauucacggg.................................................  3       1   MW1
.............aauggcacuggaagaaAucacggg.................................................  2       1   MW1
.............aGuggcacuggaagaauucacggg.................................................  1       1   MW1
.............Uauggcacuggaagaauucacggg.................................................  4       1   MW1
.............aauggcacGggaagaauucacggg.................................................  1       1   MW1
.............aauAgcacuggaagaauucacggg.................................................  9       1   MW1
.............aauggcacuggUagaauucacggg.................................................  7       1   MW1
.............aauggcacuggaaUaauucacggg.................................................  2       1   MW1
.............aauggcacuUgaagaauucacggg.................................................  2       1   MW1
.............aauggcacuggaGgaauucacggg.................................................  2       1   MW1
.............aauggcacuggaagaauucacggA.................................................  25      1   MW1
.............aaCggcacuggaagaauucacggg.................................................  10      1   MW1
.............aauggcacugAaagaauucacggg.................................................  23      1   MW1
.............aauggcaGuggaagaauucacggg.................................................  2       1   MW1
.............aauggcacuggaaAaauucacggga................................................  1       1   MW1
.............aauggcacuggaagaauucacggga................................................  2321    0   MW1
.............aauggcacugAaagaauucacggga................................................  1       1   MW1
.............aauggAacuggaagaauucacggga................................................  2       1   MW1
.............aauggcacCggaagaauucacggga................................................  1       1   MW1
.............aauggcacuggaagaaCucacggga................................................  1       1   MW1
.............aauggcacuggaagaauucacgggG................................................  8       1   MW1
.............aauggcacuggaagaauUacggga................................................  3       1   MW1
.............aauggcacuggaagaauucacggAa................................................  6       1   MW1
.............Gauggcacuggaagaauucacggga................................................  1       1   MW1
.............aGuggcacuggaagaauucacggga................................................  1       1   MW1
.............aauUgcacuggaagaauucacggga................................................  1       1   MW1
.............aauggUacuggaagaauucacggga................................................  1       1   MW1
.............aauggcacuggGagaauucacggga................................................  3       1   MW1
.............aauggcacuggaagaauCcacggga................................................  1       1   MW1
.............aaCggcacuggaagaauucacggga................................................  4       1   MW1
.............aauggcacuggaagaauucacgggC................................................  27      1   MW1
.............aauggcacuAgaagaauucacggga................................................  1       1   MW1
.............aauggcacuggaagaauucacgggU................................................  256     1   MW1
.............aauggcCcuggaagaauucacggga................................................  1       1   MW1
.............aauggcacuggaagaauGcacggga................................................  1       1   MW1
.............aauggcacuggaagaauucacgggaC...............................................  2       1   MW1
.............aauggcacuggaagaauucacgggCu...............................................  1       1   MW1
.............aauggcacuggaagaauucacgggUu...............................................  14      1   MW1
.............aauggcacuggaagaauucacgggau...............................................  80      0   MW1
.............aauggcacuggaagaauucacgggaA...............................................  124     1   MW1
.............aauggcacuggaagaauucacgggaAu..............................................  9       1   MW1
.............aauggcacuggaagaauucacgggauC..............................................  2       1   MW1
.............aauggcacuggaagaauucacgggUuu..............................................  1       1   MW1
.............aauggcacuggaagaauucacgggaCu..............................................  1       1   MW1
.............aauggcacuggaagaauucacgggauA..............................................  3       1   MW1
.............aauggcacuggaagaauucacgggauu..............................................  3       0   MW1
.............aauggcacuggaagaauucacgggauuu.............................................  1       0   MW1
.............aauggcacuggaagaauucacgggauAu.............................................  1       1   MW1
.............aauggcacuggaagaauucacgggaAuu.............................................  2       1   MW1
```

```
             aga-miR-263a
                                                      aga-miR-263a*
cccugguacauguaauggcacuggaagaauucacgggauuuuuuucaacauucccguguucucuuaguggcauaccuaguacaggg

.............aauggcacuggaagaauucacgggauuuu............................................   1     0     MW1
.............aauggcacuggaagaauucacgggUuuuu............................................   1     1     MW1
.............aauggcacuggaagaauucacgggauuAu............................................   1     1     MW1
.............aauggcacuggaagaauucacgggaAuuu............................................   1     1     MW1
.............aauggcacuggaagaauucacgggauuuCu...........................................   1     1     MW1
.............aauggcacuggaagaauucacgggauuuuuuucaaca....................................   1     0     MW1
..............auggcacuggaagaauucacgg..................................................   5     0     MW1
..............auggcacuggaagaauucacggg.................................................   16    0     MW1
..............auggcacuggaagaauucacggga................................................   5     0     MW1
..............auggcacuggaagaauucacgggaA...............................................   3     1     MW1
...............uggcacuggaagaauucacgggU................................................   1     1     MW1
...............uggcacuggaagaauucacgggau...............................................   1     0     MW1
.................gcacuggaagaauucacgg..................................................   3     0     MW1
.................gcacuggaagaauucacggg.................................................   5     0     MW1
..................cacuggaagaauucacg...................................................   1     0     MW1
..................cacuggaagaauucacgg..................................................   2     0     MW1
..................cacuggaagaauucacggg.................................................   5     0     MW1
..................cacuggaagaauucacggga................................................   1     0     MW1
...................acuggaagaauucacggg.................................................   1     0     MW1
....................cuggaagaauucacggg.................................................   4     0     MW1
....................cuggaagaauucacgggau...............................................   1     0     MW1
.....................................................ccguguucucuuaguggcauacc..........   3     0     MW1
......................................................cguguucucuuaguggcauac...........   1     0     MW1
......................................................cguguucucuuagugUcauacc..........   1     1     MW1
......................................................cguguucucuuaguggcauacc..........   265   0     MW1
.......................................................guguucucuuaguggcauacc..........   2     0     MW1

............Caauggcacuggaagaauucacg...................................................   1     1     MW2
............Caauggcacuggaagaauucacgg..................................................   4     1     MW2
............Caauggcacuggaagaauucacggg.................................................   1     1     MW2
.............aauggcacuggaagaau........................................................   3     0     MW2
.............aauggcacuggaagaauu.......................................................   4     0     MW2
.............aauggcacuggaagaauuca.....................................................   15    0     MW2
.............aauggcacuggaagaauuUac....................................................   1     1     MW2
.............aauggcacuggaagaauucaU....................................................   1     1     MW2
.............aauggcacuggaagaauucac....................................................   119   0     MW2
.............aauggcacugAaagaauucacg...................................................   1     1     MW2
.............aauggcacuggaagaauucacg...................................................   384   0     MW2
.............aauggcacCggaagaauucacg...................................................   1     1     MW2
.............aauggcGcuggaagaauucacg...................................................   1     1     MW2
.............aauggcacuggaagaauucaUg...................................................   1     1     MW2
.............aauggcacuggUagaauucacg...................................................   1     1     MW2
.............aauggcacuggaagaCuucacg...................................................   1     1     MW2
.............aauggcacuggaagaauucacA...................................................   2     1     MW2
.............aauggcacuggaagaUuucacg...................................................   1     1     MW2
.............aauggcacuggaagaauucGcgg..................................................   1     1     MW2
.............aauggcacuggaagaauucacgU..................................................   1     1     MW2
.............aGuggcacuggaagaauucacgg..................................................   1     1     MW2
.............aauggcacuggaagaaAucacgg..................................................   1     1     MW2
.............aauggcacCggaagaauucacgg..................................................   1     1     MW2
.............aauggcGcuggaagaauucacgg..................................................   1     1     MW2
.............aauggcacuggaagaauucacgA..................................................   3     1     MW2
.............aauggcacuggaGgaauucacgg..................................................   3     1     MW2
.............aauggcacuggaaAaauucacgg..................................................   1     1     MW2
.............aauggcacuUgaagaauucacgg..................................................   1     1     MW2
.............aauggcacuggGagaauucacgg..................................................   1     1     MW2
.............aauggcacuggaagaauuGacgg..................................................   1     1     MW2
.............Uauggcacuggaagaauucacgg..................................................   1     1     MW2
.............aaugAcacuggaagaauucacgg..................................................   3     1     MW2
.............aauggcacuggUagaauucacgg..................................................   1     1     MW2
.............aauggcacuggaagaaCucacgg..................................................   3     1     MW2
.............aauggcacugAaagaauucacgg..................................................   2     1     MW2
.............aauggcacuggaagaauuUacgg..................................................   1     1     MW2
.............aauggcacuggaagaauGcacgg..................................................   1     1     MW2
.............aauggcacuggaagaauucacgg..................................................   3399  0     MW2
.............Cauggcacuggaagaauucacgg..................................................   1     1     MW2
.............aaCggcacuggaagaauucacggg.................................................   1     1     MW2
.............aauggcacuggaagGauucacggg.................................................   2     1     MW2
.............aauggcacuggaagaauucacgAg.................................................   1     1     MW2
```

```
             aga-miR-263a
                                         aga-miR-263a*
cccugguacauguaauggcacuggaagaauucacgggauuuuuuucaacauucccguguucucuuaguggcauaccuaguacaggg

.............Cauggcacuggaagaauucacggg.................................................   1      1    MW2
.............aauggcacuggaagCauucacggg.................................................   1      1    MW2
.............aauggcacugUaagaauucacggg.................................................   3      1    MW2
.............aauggcacuggaGgaauucacggg.................................................   1      1    MW2
.............aauggcacugCaagaauucacggg.................................................   2      1    MW2
.............aauggcacuggaagaauucaUggg.................................................   1      1    MW2
.............aauggcacuAgaagaauucacggg.................................................   2      1    MW2
.............aauggcacuggaaUaauucacggg.................................................   1      1    MW2
.............aauggcacuggaagaauucacggg.................................................   7457   0    MW2
.............aauggcacuggCagaauucacggg.................................................   4      1    MW2
.............aauggcacuggaagaauCcacggg.................................................   1      1    MW2
.............aaugUcacuggaagaauucacggg.................................................   1      1    MW2
.............aauggcacuggaagUauucacggg.................................................   1      1    MW2
.............aauggcacugAaagaauucacggg.................................................   7      1    MW2
.............aauggcacuggaagaauuUacggg.................................................   3      1    MW2
.............aauggcacuggaagaauuGacggg.................................................   1      1    MW2
.............aauggcacuggaagaGuucacggg.................................................   2      1    MW2
.............aauggcacuggaagaauucUcggg.................................................   1      1    MW2
.............aauggcacuggaagaaGucacggg.................................................   1      1    MW2
.............aauggcacuggaagaauuAacggg.................................................   1      1    MW2
.............aauggcacuggaagaauucacggC.................................................   6      1    MW2
.............aauggcacuggaaAaauucacggg.................................................   1      1    MW2
.............aauggcacuggaagaauucacggA.................................................   11     1    MW2
.............aauggcacuggaagaaCucacggg.................................................   4      1    MW2
.............Gauggcacuggaagaauucacggg.................................................   3      1    MW2
.............aauggUacuggaagaauucacggg.................................................   2      1    MW2
.............aauggcacuggaagaauucacggU.................................................   6      1    MW2
.............aGuggcacuggaagaauucacggg.................................................   1      1    MW2
.............aauggcacuggaagaauucacUgg.................................................   1      1    MW2
.............aauAgcacuggaagaauucacggg.................................................   6      1    MW2
.............aauggAacuggaagaauucacggg.................................................   2      1    MW2
.............aauggcacuggaaCaauucacggg.................................................   1      1    MW2
.............aauggcacCggaagaauucacggg.................................................   1      1    MW2
.............aauggcacAggaagaauucacggg.................................................   2      1    MW2
.............aauggcacuggaagaauucaGggg.................................................   1      1    MW2
.............aauggcacuggaagaauucacCgg.................................................   1      1    MW2
.............aaGggcacuggaagaauucacggg.................................................   1      1    MW2
.............aauggcacuggaagaauuUacggga................................................   1      1    MW2
.............aauggcacuggaagaaCucacggga................................................   1      1    MW2
.............aaCggcacuggaagaauucacggga................................................   1      1    MW2
.............aaugAcacuggaagaauucacggga................................................   2      1    MW2
.............aauggcacuggaagaauucacgggG................................................   1      1    MW2
.............aauggcacuggaagaauucacgggC................................................   8      1    MW2
.............aauggcacuggCagaauucacggga................................................   1      1    MW2
.............aaugCcacuggaagaauucacggga................................................   1      1    MW2
.............aauggcacuggaagaauucacggga................................................   879    0    MW2
.............aauggcacuggaagaauucacggAa................................................   2      1    MW2
.............aauggcacuggaagaauucacgggU................................................   70     1    MW2
.............aauggcacuggaagaauucacgggau...............................................   19     0    MW2
.............aauggcacuggaagaauucacgggUu...............................................   10     1    MW2
.............aauggcacuggaagaauucacgggCu...............................................   3      1    MW2
.............aauggcacuggaagaauucacgggaC...............................................   2      1    MW2
.............aauggcacuggaagaauucacgggaA...............................................   41     1    MW2
.............aauggcacuggaagaauucacgggUuu..............................................   1      1    MW2
.............aauggcacuggaagaauucacgggaAu..............................................   3      1    MW2
.............aauggcacuggaagaauucacgggauC..............................................   1      1    MW2
.............aauggcacuggaagaauucacgggauuAu............................................   1      1    MW2
..............auggcacuggaagaauucacgg..................................................   1      0    MW2
..............auggcacuggaagaauucacggg.................................................   7      0    MW2
...............uggcacuggaagaauucacggg.................................................   1      0    MW2
.................gcacuggaagaauucacggg.................................................   2      0    MW2
..................cacuggaagaauucacggg.................................................   1      0    MW2
....................cuggaagaauucacggg.................................................   2      0    MW2
......................................................cguguucucuuaguggcauacc..........   54     0    MW2

............Caauggcacuggaagaauucacgg..................................................   1      1    TE2
............Caauggcacuggaagaauucacggg.................................................   1      1    TE2
.............aauggcacuggaagaau........................................................   3      0    TE2
.............aauggcacuggaagaauu.......................................................   71     0    TE2
```

aga-miR-263a

aga-miR-263a*

cccugguacauguaauggcacuggaagaauucacgggauuuuuuucaacauucccguguucucuuaguggcauaccuaguacaggg

.............aauggcacuggaagaauuc..	2	0	TE2
.............aauggcacuggaagaauuca...	10	0	TE2
.............aauggAacuggaagaauucac..	1	1	TE2
.............aauggcacuggaagaauucac..	152	0	TE2
.............Gauggcacuggaagaauucac..	1	1	TE2
.............aauggcacuggGagaauucac..	1	1	TE2
.............aauggcacuggaagaauucacA...	3	1	TE2
.............aauggcacuggaagaauuUacg...	1	1	TE2
.............aauggcacuggaagaauucacg...	470	0	TE2
.............aauggcacuggaagaauucacU...	1	1	TE2
.............aauggcacuAgaagaauucacg...	1	1	TE2
.............aauggcacuggaagaCuucacg...	1	1	TE2
.............aauggcGcuggaagaauucacg...	1	1	TE2
.............aauggcacuggGagaauucacg...	3	1	TE2
.............aauggcacuggaagaaCucacgg..	2	1	TE2
.............aauggcacGggaagaauucacgg..	1	1	TE2
.............aauCgcacuggaagaauucacgg..	1	1	TE2
.............aauggcacuggaagaauucacgg..	5377	0	TE2
.............aauggcacuggaagaauucaUgg..	3	1	TE2
.............aauggcacuggaagaauucacUg..	2	1	TE2
.............aaCggcacuggaagaauucacgg..	2	1	TE2
.............aGuggcacuggaagaauucacgg..	1	1	TE2
.............aauggcacugUaagaauucacgg..	2	1	TE2
.............aaAggcacuggaagaauucacgg..	2	1	TE2
.............aauggcacuggaagaauCcacgg..	1	1	TE2
.............aauggcacuggCagaauucacgg..	4	1	TE2
.............aauggcacuggaagaauuUacgg..	2	1	TE2
.............aauggcaUuggaagaauucacgg..	2	1	TE2
.............aauggcacuggaagaauucacAg..	2	1	TE2
.............aauggcacuggaagaauuAacgg..	1	1	TE2
.............aauggcacuggUagaauucacgg..	1	1	TE2
.............aauggcacuggaagaauucacgA..	9	1	TE2
.............aaugUcacuggaagaauucacgg..	1	1	TE2
.............aauggcacuUgaagaauucacgg..	1	1	TE2
.............aauggUacuggaagaauucacgg..	3	1	TE2
.............aauggcacuggaaAaauucacgg..	1	1	TE2
.............aUuggcacuggaagaauucacgg..	1	1	TE2
.............aaugAcacuggaagaauucacgg..	1	1	TE2
.............aauggcacugCaagaauucacgg..	1	1	TE2
.............aauggcacuggaagaauucaGgg..	1	1	TE2
.............aauggcacuggaagaauucGcgg..	1	1	TE2
.............aauggcacugAaagaauucacgg..	6	1	TE2
.............aauggAacuggaagaauucacgg..	1	1	TE2
.............aauggcacuggGagaauucacgg..	1	1	TE2
.............aauggcacuAgaagaauucacgg..	2	1	TE2
.............aauggcacuggaagaauGcacggg...	1	1	TE2
.............aauggcacuAgaagaauucacggg...	2	1	TE2
.............aauggcacuggaagaauuUacggg...	6	1	TE2
.............aauggcacugUaagaauucacggg...	4	1	TE2
.............aauggcacuggaagaauucaUggg...	4	1	TE2
.............aauggcacuggaagaauucacAgg...	2	1	TE2
.............aauggcacuggaagaaCucacggg...	2	1	TE2
.............aauggcacuggaagaauucacgAg...	2	1	TE2
.............aauggcacCggaagaauucacggg...	1	1	TE2
.............aauggcacuggGagaauucacggg...	2	1	TE2
.............aauggcacuggaagaauucacggA...	18	1	TE2
.............aauggcacuggaagaauucacggC...	2	1	TE2
.............aauggcacuggaagaauucacggU...	2	1	TE2
.............aauggcacuggaagaCuucacggg...	1	1	TE2
.............aauggcacuggUagaauucacggg...	1	1	TE2
.............aauggcCcuggaagaauucacggg...	1	1	TE2
.............aauggcacuggaagaauucUcggg...	1	1	TE2
.............aauggcacuggaGgaauucacggg...	1	1	TE2
.............aauggcacuCgaagaauucacggg...	1	1	TE2
.............aauggcacuggaagaauucacggg...	3578	0	TE2
.............aaugAcacuggaagaauucacggg...	1	1	TE2
.............aauggcacuggCagaauucacggg...	3	1	TE2
.............aauggAacuggaagaauucacggg...	1	1	TE2
.............aauggcacAggaagaauucacggg...	1	1	TE2

```
               aga-miR-263a
                                                     aga-miR-263a*
cccugguacaugu aauggcacuggaagaauucacggg auuuuuuucaacauucc cguguucucuuaguggcauacc uaguacaggg
```

```
cccugguacauguaauggcacuggaagaauucacgggauuuuuuucaacauucccguguucucuuaguggcauaccuaguacaggg

.............aauAgcacuggaagaauucacggg.................................................   4     1   TE2
.............aauggcacugAaagaauucacggg.................................................   2     1   TE2
.............aauggcacuggaagaauucacCgg.................................................   1     1   TE2
.............aauggcacuggaagaauucacgCga................................................   1     1   TE2
.............aauggcacuggaagaauucacgggG................................................   4     1   TE2
.............aauggcacuUgaagaauucacggga................................................   1     1   TE2
.............aauggcacuggaagaauucacggAa................................................   9     1   TE2
.............aauggcacuggaagaauucacgggU................................................   68    1   TE2
.............aauggcacuggUagaauucacggga................................................   1     1   TE2
.............aauggcacuggaagaauucacggCa................................................   2     1   TE2
.............aauggcacuggaagaaAucacggga................................................   1     1   TE2
.............aauggcacuAgaagaauucacggga................................................   1     1   TE2
.............aauggcacuggaagaauucacgggC................................................   5     1   TE2
.............aauggcacuggaagaauucaUggga................................................   1     1   TE2
.............aauggcacuggaagaauucacggga................................................   702   0   TE2
.............aauggcacuggaagaauucacgggaA...............................................   58    1   TE2
.............aauggcacuggaagaauucacgggaC...............................................   1     1   TE2
.............aauggcacuggaagaauucacgggUu...............................................   6     1   TE2
.............aauggcacuggaagaauucacgggau...............................................   46    0   TE2
.............aauggcacuggaagaauucacgggaAu..............................................   5     1   TE2
.............aauggcacuggaagaauucacgggUuu..............................................   2     1   TE2
.............aauggcacuggaagaauucacgggauu..............................................   2     0   TE2
.............aauggcacuggaagaauucacgggauA..............................................   1     1   TE2
.............aauggcacuggaagaauucacgggauuu.............................................   1     0   TE2
.............aauggcacuggaagaauucacgggaAuu.............................................   1     1   TE2
.............aauggcacuggaagaauucacgggUuuuu............................................   1     1   TE2
.............aauggcacuggaagaauucacgggaAuuu............................................   1     1   TE2
.............aauggcacuggaagaauucacgggauuuu............................................   2     0   TE2
..............auggcacuggaagaauucacgg..................................................   1     0   TE2
..............auggcacuggaagaauucacggga................................................   2     0   TE2
..............auggcacuggaagaauucacgggau...............................................   1     0   TE2
..................cacuggaagaauucacg...................................................   2     0   TE2
..................cacuggaagaauucacgg..................................................   1     0   TE2
......................................................cguguucucuuaguggcauacc..........   5     0   TE2
```

miRBase precursor : aga-mir-263b
Total read count : 334
aga-miR-263b read count : 332
aga-miR-263b* read count : 2
remaining reads : 0

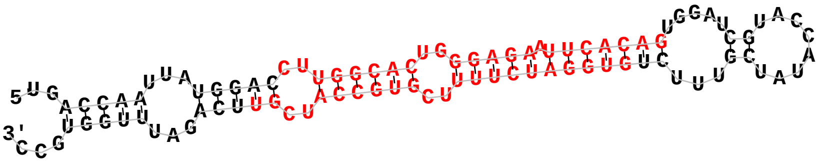

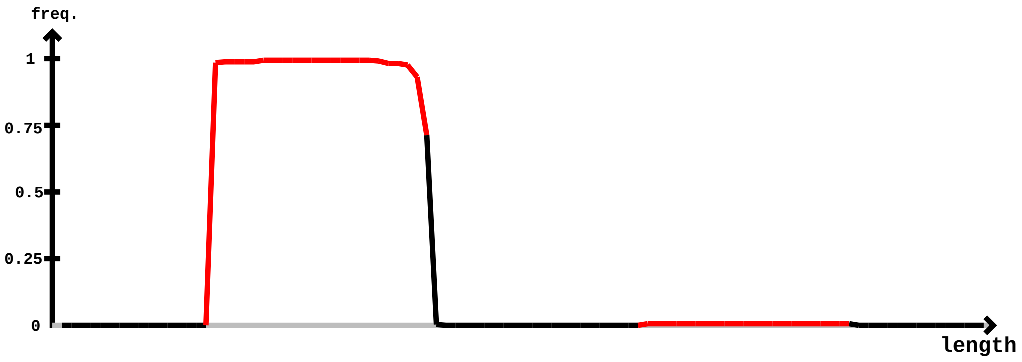

aga-miR-263b

aga-miR-263b*

```
5'- ugaccaauuauggaccuuggcacugggagaauucacaguggaucguaccauaucguuucuguggaucuuuucgugccaucguucagauuuggugcc   -3'   exp
    ..(((((...(((((..((((((..(((((.(((((((.....((........))...))))))))))))..))))))..)))))...)))))...    reads      mm        sample
    ...............cuuggcacugggagaauucac............................................................    2          0         FF2
    ...............cuuggcacugggagaauucaca...........................................................    2          0         FF2
    ...............cuuggcacugggagaauucacag..........................................................    16         0         FF2
    ....................cacugggagaauucacag..........................................................    1          0         FF2

    ...............cuuggcacugggagaauucac............................................................    1          0         TE1
    ...............cuuggcacugggagaauucaca...........................................................    7          0         TE1
    ...............cuugAcacugggagaauucacag..........................................................    1          1         TE1
    ...............cuuggcUcugggagaauucacag..........................................................    1          1         TE1
    ...............cuuggcacugggagaauucacag..........................................................    24         0         TE1

    ...............cuuggcacugggagaauu...............................................................    2          0         MF2
    ...............cuuggcacugggagaauuca.............................................................    2          0         MF2
    ...............cuuggcacugggagaauucac............................................................    9          0         MF2
    ...............cuuggcacugggaAaauucac............................................................    1          1         MF2
    ...............cuuggcacugggagaauucaca...........................................................    44         0         MF2
    ...............cuuggcacugUgagaauucaca...........................................................    1          1         MF2
    ...............cuuggcacugggagaauucacaA..........................................................    1          1         MF2
    ...............cuuggcacugggagaauucacag..........................................................    98         0         MF2
    ...............cuuggcacuggAagaauucacag..........................................................    1          1         MF2
    ...............cuuggcacugggagaauuAacag..........................................................    1          1         MF2

    ...............cuuggcacugggagaauucaca...........................................................    4          0         FW2
    ...............cuuggcacugggagaauucacag..........................................................    22         0         FW2
    ............................................................guggaucuuuucgugccaucgu..............    1          0         FW2

    ...............cuuggcacugggagaauucacag..........................................................    1          0         OV1

    ...............cuuggcacugggagaauucaca...........................................................    1          0         FF1
    ...............cuuggcacugggagaauucacag..........................................................    1          0         FF1

    ...............cuuggcacugggagaauucaca...........................................................    5          0         MF1
    ...............cuuggcacugggagaauucacag..........................................................    18         0         MF1
    ...............cuuggcacugggagaauucacagu.........................................................    1          0         MF1
```

```
               aga-miR-263b
                                                           aga-miR-263b*
ugaccaauuauggaccuuggcacugggagaauucacaguggaucguaccauaucguuucuguggaucuuuucgugccaucguucagauuuggugcc


...............cuuggcacugggagaauucaca...........................................................    1    0    BF1
...............cuuggcacugggagaauucacag..........................................................    1    0    BF1

...............cuuggcacugggagaau................................................................    1    0    MW1
...............cuuggcacugggagaauucaca...........................................................    2    0    MW1
...............cuuggcacugggagaauucacag..........................................................    24   0    MW1
...............cuuggcacugggagaaCucacag..........................................................    1    1    MW1

...............cuuggcacugggagaauucac............................................................    1    0    FW1
...............cuuggcacugggagaauucaca...........................................................    2    0    FW1
...............cuuggcacugggagaauucacag..........................................................    8    0    FW1
............................................................guggaucuuuucgugccaucgu..............    1    0    FW1

...............cuuggcacugggagaauucacag..........................................................    10   0    MW2

...............cuuggcacugggagaauu...............................................................    1    0    TE2
...............cuuggcacugggagaauucac............................................................    1    0    TE2
...............cuuggcacugggagaauucaca...........................................................    3    0    TE2
...............cuuggcacugggagaauucacag..........................................................    5    0    TE2
...............cuuggcacugggagaCuucacag..........................................................    1    1    TE2
................uuggcacugggagaauucaca...........................................................    1    0    TE2
....................cacugggagaauucacag..........................................................    1    0    TE2
```

miRBase precursor : aga-mir-275
Total read count : 33271
aga-miR-275 read count : 31823
aga-miR-275* read count : 1415
remaining reads : 33

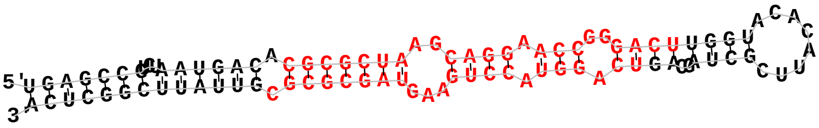

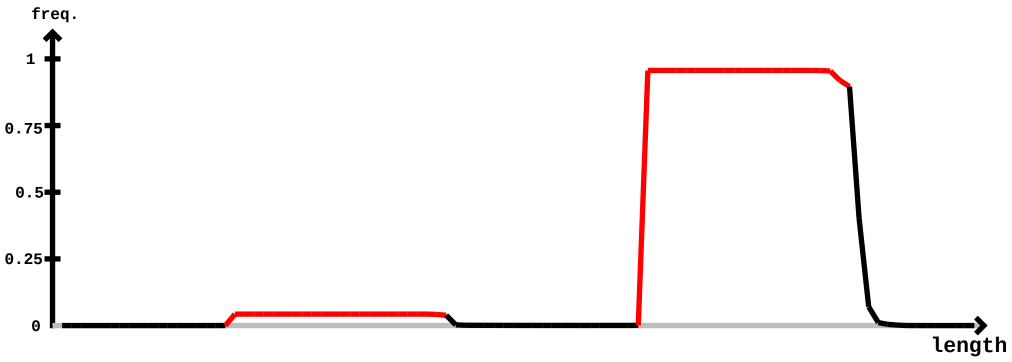

aga-miR-275

aga-miR-275*

```
5'- ugagccgucuaaugacacgcgcuaagcaggaaccgggacuugguacacauucgcuagcagucagguaccugaaguagcgcgcguuauucggcuca  -3'    exp
    .((((((...((((((.(((((((..((((.(((..((((((((........))))..)))).))).))))...))))))).))))))))))).    reads    mm    sample
    .................cgcgcuaagcaggaaccggga.........................................................    2        0     OV2
    .................cgcgcuaagcaggaaccgggac........................................................    2        0     OV2
    .................cgcgcuaagcaAgaaccgggacu.......................................................    1        1     OV2
    .................cgcgcuaagcaggaaccgggacu.......................................................    12       0     OV2
    .................cgcgcuaagcaggaaccgggacuu......................................................    1        0     OV2
    ...........................................................Cucagguaccugaaguagcgcgc.............    1        1     OV2
    ............................................................ucagguaccugaaguagc.................    1        0     OV2
    ............................................................ucagguaccugaaguagcg................    3        0     OV2
    ............................................................ucagguaccuCaaguagcgc...............    1        1     OV2
    ............................................................ucagguaccugaaguagcgc...............    63       0     OV2
    ............................................................ucagguaccugaaguagcgcg..............    27       0     OV2
    ............................................................ucagguaccuAaaguagcgcgc.............    1        1     OV2
    ............................................................ucagguaccugaaguagcgcgc.............    765      0     OV2
    ............................................................ucagguaccugaaguagcgcgU.............    1        1     OV2
    ............................................................ucagguaccugaaguagcgcgG.............    1        1     OV2
    ............................................................ucagguaccugGaguagcgcgc.............    1        1     OV2
    ............................................................ucagguaccuUaaguagcgcgc.............    1        1     OV2
    ............................................................Ccagguaccugaaguagcgcgc.............    1        1     OV2
    ............................................................ucagguaccCgaaguagcgcgc.............    1        1     OV2
    ............................................................ucaggGaccugaaguagcgcgc.............    1        1     OV2
    ............................................................ucagguaccugaaguagcAcgc.............    1        1     OV2
    ............................................................ucagguaccugaaguGgcgcgc.............    1        1     OV2
    ............................................................ucaggCaccugaaguagcgcgc.............    1        1     OV2
    ............................................................ucagguaccugaaguagUgcgc.............    1        1     OV2
    ............................................................ucagguaccugaaguagcgcgcU............    7        1     OV2
    ............................................................ucagguaccugaaguagcgcgcC............    13       1     OV2
    ............................................................ucagguaccugaaguagcgcgcA............    2        1     OV2
    ............................................................ucagguaccugaaguagcgcgcg............    254      0     OV2
    ............................................................ucagguaccugaaguagcgcgcgu...........    20       0     OV2
    ............................................................ucagguaccugaaguagcgcgcUu...........    2        1     OV2
    ............................................................ucagguaccugaaguagcgcgcgA...........    13       1     OV2
    ............................................................ucagguaccugaaguagcgcgcCu...........    1        1     OV2
    ............................................................ucagguaccugaaguagcgcgcgC...........    10       1     OV2
    ............................................................ucagguaccugaaguagcgcgcAu...........    3        1     OV2
```

```
                                                            aga-miR-275
                 aga-miR-275*
ugagccgucuaaugacacgcgcuaagcaggaaccgggacuugguacacauucgcuagcagucagguaccugaaguagcgcgcguuauucggcuca

............................................................ucagguaccugaaguagcgcgcgAu..........    1     1     OV2
............................................................ucagguaccugaaguagcgcgcguu..........    1     0     OV2
............................................................ucagguaccugaaguagcgcgcguuU.........    2     1     OV2
............................................................ucagguaccugaaguagcgcgcguAa.........    1     1     OV2
............................................................ucagguaccugaaguagcgcgcgAuau........    1     1     OV2
............................................................ucagguaccugaaguagcgcgcguAau........    1     1     OV2
............................................................ucagguaccugaaguagcgcgcguuUu........    1     1     OV2

.................cgcgcuaagcaggaaccggga.........................................................    1     0     TE1
.................cgcgcuaagcaggaaccgggacu.......................................................    7     0     TE1
................................................auucgcuagcagucagguaccugaaguag..................    1     0     TE1
............................................................ucagguaccugaaguagcg................    5     0     TE1
............................................................ucagguaccAgaaguagcgc...............    1     1     TE1
............................................................ucagguaccugaaguagcgc...............    63    0     TE1
............................................................ucagguaccCgaaguagcgc...............    1     1     TE1
............................................................ucagguaccugaaguagcgcg..............    30    0     TE1
............................................................uUagguaccugaaguagcgcgc.............    1     1     TE1
............................................................ucagguaccugaaguagcgcgA.............    1     1     TE1
............................................................ucagguaccugaaguCgcgcgc.............    1     1     TE1
............................................................ucagAuaccugaaguagcgcgc.............    1     1     TE1
............................................................ucagguaccuUaaguagcgcgc.............    1     1     TE1
............................................................ucaggAaccugaaguagcgcgc.............    1     1     TE1
............................................................ucagguaccugaaguagcgcgU.............    3     1     TE1
............................................................ucagguaccuAaaguagcgcgc.............    1     1     TE1
............................................................ucagguaccugGaguagcgcgc.............    2     1     TE1
............................................................ucagguaccugaaguagcgcgc.............    631   0     TE1
............................................................ucagguaccugaaguagcgcgcC............    6     1     TE1
............................................................ucagguaccugaaguagcgcgcg............    153   0     TE1
............................................................ucagguaccugaaguagcgcgcU............    3     1     TE1
............................................................ucagguaccugaaguagcCgcg.............    1     1     TE1
............................................................ucagguaccugaaguagcgcgcgu...........    25    0     TE1
............................................................ucagguaccugaaguagcgcgcgA...........    9     1     TE1
............................................................ucagguaccugaaguagcgcgcgC...........    1     1     TE1
............................................................ucagguaccugaaguagcgcgcCu...........    1     1     TE1
............................................................ucagguaccugaaguagcgcgcUu...........    3     1     TE1
............................................................ucagguaccugaaguagcgcgcgAu..........    2     1     TE1
............................................................ucagguaccugaaguagcgcgcguu..........    2     0     TE1
............................................................ucagguaccugaaguagcgcgcguAa.........    1     1     TE1
............................................................ucagguaccugaaguagcgcgcgAuau........    1     1     TE1

.................cgcgcuaagcaggaaccgggG.........................................................    2     1     FF2
.................cgcgcuaagcaggaaccggga.........................................................    6     0     FF2
.................cgcgcuaagcaggaaccgggac........................................................    4     0     FF2
.................cgcgcuaagcaggaaccgggacu.......................................................    92    0     FF2
.................cgcgcuaagcaggaaccgggacC.......................................................    1     1     FF2
.................cgcgcuaagcaggaaccgggacuu......................................................    4     0     FF2
.................cgcgcuaagcaggaaccgggacuuggua..................................................    1     0     FF2
................................ccgggacuugguacacauucgcuagc.....................................    1     0     FF2
...........................................................Cucagguaccugaaguagcgcgc.............    1     1     FF2
...........................................................Cucagguaccugaaguagcgcgcg............    1     1     FF2
............................................................ucagguaccugaaguagcg................    2     0     FF2
............................................................ucagguaccCgaaguagcgc...............    1     1     FF2
............................................................ucagguaccugaaguagcgc...............    54    0     FF2
............................................................ucagguaccugaaguagcgcg..............    21    0     FF2
............................................................ucagguaccCgaaguagcgcg..............    1     1     FF2
............................................................uGagguaccugaaguagcgcg..............    1     1     FF2
............................................................ucagguaccugaaguagAgcgc.............    1     1     FF2
............................................................ucagguaccugaaguagcgcgc.............    811   0     FF2
............................................................uUagguaccugaaguagcgcgc.............    1     1     FF2
............................................................ucagguaccugaagCagcgcgc.............    1     1     FF2
............................................................ucagguaccAgaaguagcgcgc.............    1     1     FF2
............................................................ucagguaccugaagGagcgcgc.............    1     1     FF2
............................................................ucagguaccugaaguagcgcAc.............    2     1     FF2
............................................................ucagguaccugaaguGgcgcgc.............    1     1     FF2
............................................................ucagguaAcugaaguagcgcgc.............    1     1     FF2
............................................................ucagguaccugaaguagcgcgA.............    3     1     FF2
............................................................ucagguaccugaaguagcgcgG.............    2     1     FF2
............................................................ucagguaccugaaguagcgcUc.............    1     1     FF2
............................................................ucagguaccugaaguagcgcgU.............    1     1     FF2
```

```
                                                            aga-miR-275
                 aga-miR-275*
ugagccgucuaaugacacgcgcuaagcaggaaccgggacuugguacacauucgcuagcagucagguaccugaaguagcgcgcguuauucggcuca

............................................................ucagguaccugaaCuagcgcgc.............    1     1    FF2
............................................................ucagguaccugaaguaUcgcgc.............    1     1    FF2
............................................................ucagguaccugaaguagcAcgc.............    1     1    FF2
............................................................ucagguaccugaaguaUcgcgcg............    1     1    FF2
............................................................ucagguaccugaaguagcgcgcg............    564   0    FF2
............................................................ucagguaccugaagCagcgcgcg............    1     1    FF2
............................................................ucagguaccugaUguagcgcgcg............    1     1    FF2
............................................................ucagguaccuUaaguagcgcgcg............    1     1    FF2
............................................................ucagguaccugaaguagcgcgcC............    37    1    FF2
............................................................uUagguaccugaaguagcgcgcg............    2     1    FF2
............................................................ucagguaccugaaguagcgcgcA............    1     1    FF2
............................................................ucagguaccugaaguGgcgcgcg............    1     1    FF2
............................................................ucagguaccugGaguagcgcgcg............    1     1    FF2
............................................................ucagguaccugaaguagcgcgcU............    10    1    FF2
............................................................ucagguaccugaaguagcgcgGg............    1     1    FF2
............................................................ucagAuaccugaaguagcgcgcg............    1     1    FF2
............................................................ucagguaccugaaguagcgcgcgC...........    7     1    FF2
............................................................ucagguaccugaaguagcgcgcCu...........    13    1    FF2
............................................................ucagguaccugaaguagcgcgcgA...........    43    1    FF2
............................................................ucagguaccugaaguagcgcgcgu...........    46    0    FF2
............................................................ucagguaccugaaguagcgcgcUu...........    9     1    FF2
............................................................ucagguaccugaaguagcgcgcgCu..........    1     1    FF2
............................................................ucagguaccugaaguagcgcgcgAu..........    9     1    FF2
............................................................ucagguaccugaaguagcgcgcguu..........    3     0    FF2
............................................................ucagguaccugaaguagcgcgcguuU.........    2     1    FF2
............................................................ucagguaccugaaguagcgcgcguAa.........    4     1    FF2
............................................................ucagguaccugaaguagcgcgcguuUu........    2     1    FF2
............................................................ucagguaccugaaguagcgcgcUuuau........    2     1    FF2
............................................................ucagguaccugaaguagcgcgcguuaG........    1     1    FF2
............................................................ucagguaccugaaguagcgcgcguAau........    2     1    FF2

.................cgcgcuaagcaggaaccggg..........................................................    1     0    MF2
.................cgcgcuaagcaggaaccggga.........................................................    12    0    MF2
.................cgcgcuaagcaggaaccgggac........................................................    46    0    MF2
.................cgcgcuaagcaggaaccgggaA........................................................    3     1    MF2
.................cgcgcuaagcaggaaccCggac........................................................    1     1    MF2
.................cgcgcuaagcaAgaaccgggacu.......................................................    2     1    MF2
.................cgcgcuaagcaggaaccgggacu.......................................................    551   0    MF2
.................cgcAcuaagcaggaaccgggacu.......................................................    1     1    MF2
.................cgcgcuaagcaggaaccgggacA.......................................................    1     1    MF2
.................cgcgcuaagcaggaaccgggaGu.......................................................    1     1    MF2
.................cgcgcuaagcaggaaccgCgacu.......................................................    1     1    MF2
.................Agcgcuaagcaggaaccgggacu.......................................................    1     1    MF2
.................cgcgcuGagcaggaaccgggacu.......................................................    1     1    MF2
.................cgcgcuaagcaggaaccgggacuA......................................................    2     1    MF2
.................cgcgcuaagcaggaaccgggacuu......................................................    33    0    MF2
.................cgcgcuaagcaggaaccgggacuuU.....................................................    2     1    MF2
.................cgcgcuaagcaggaaccgggacuugguac.................................................    2     0    MF2
.................cgcgcuaagcaggaaccgggacuugguacacauuc...........................................    1     0    MF2
.................cgcgcuaagcaggaaccgggacuugguacacauucg..........................................    1     0    MF2
.................cgcgcuaagcaggaaccgggacuugguacacauucgc.........................................    1     0    MF2
..................gcgcuaagcaggaaccggga.........................................................    1     0    MF2
................................ccgggacuugguacacauucgcuagcagucagguaccugaaguagcgcgcC............    1     1    MF2
........................................ugguacacauucgcuagcag...................................    1     0    MF2
.......................................................agcagucagguaccugaaguagcgcgcg............    2     0    MF2
..........................................................aAucagguaccugaaguagcgcgcg............    1     1    MF2
...........................................................Uucagguaccugaaguagcgcgc.............    1     1    MF2
...........................................................Cucagguaccugaaguagcgcgcg............    2     1    MF2
............................................................ucagguaccugaaguagc.................    8     0    MF2
............................................................ucagguaccugaaguagcU................    2     1    MF2
............................................................ucagguaccugaaguagcg................    12    0    MF2
............................................................ucagguaccugaaguagcgc...............    320   0    MF2
............................................................ucagguaccugaaAuagcgc...............    1     1    MF2
............................................................ucagguaccugaaguagcgcg..............    176   0    MF2
............................................................ucagguaccugaaguagcgcU..............    7     1    MF2
............................................................ucagguaccugaaguaAcgcg..............    1     1    MF2
............................................................ucagguaAcugaaguagcgcgc.............    1     1    MF2
............................................................ucagguaccugaaguagcUcgc.............    1     1    MF2
............................................................ucagguaccugaagGagcgcgc.............    1     1    MF2
```

```
                                                            aga-miR-275
                 aga-miR-275*
ugagccgucuaaugacacgcgcuaagcaggaaccgggacuugguacacauucgcuagcagucagguaccugaaguagcgcgcguuauucggcuca

............................................................ucagguaccugaaguGgcgcgc.............    4       1    MF2
............................................................ucagguaccuAaaguagcgcgc.............    3       1    MF2
............................................................ucagguaccugaagCagcgcgc.............    3       1    MF2
............................................................ucagguaccugaaguagcgcgA.............    7       1    MF2
............................................................ucagguaccugaaguagcgAgc.............    1       1    MF2
............................................................ucagguaccCgaaguagcgcgc.............    2       1    MF2
............................................................ucagguGccugaaguagcgcgc.............    1       1    MF2
............................................................ucagguaccugaUguagcgcgc.............    2       1    MF2
............................................................ucagguaccugaaguagcgUgc.............    3       1    MF2
............................................................ucaggCaccugaaguagcgcgc.............    4       1    MF2
............................................................uUagguaccugaaguagcgcgc.............    8       1    MF2
............................................................ucagguaccugUaguagcgcgc.............    1       1    MF2
............................................................ucagguaccugaaguCgcgcgc.............    1       1    MF2
............................................................ucagguaccugaaguagcgcAc.............    6       1    MF2
............................................................ucaggGaccugaaguagcgcgc.............    1       1    MF2
............................................................ucagguaccugaGguagcgcgc.............    1       1    MF2
............................................................ucagAuaccugaaguagcgcgc.............    1       1    MF2
............................................................ucagguaccugaaguagUgcgc.............    2       1    MF2
............................................................ucagguaccGgaaguagcgcgc.............    2       1    MF2
............................................................ucagguaccugaaguaAcgcgc.............    4       1    MF2
............................................................ucagguaccugaaguagcgGgc.............    1       1    MF2
............................................................ucagguaccugaaguagcgcgU.............    21      1    MF2
............................................................ucagguaccugaaguagcgcgG.............    7       1    MF2
............................................................ucagguaccugaaguagAgcgc.............    2       1    MF2
............................................................ucagguaccugaaguagcgcgc.............    4491    0    MF2
............................................................Ccagguaccugaaguagcgcgc.............    1       1    MF2
............................................................ucGgguaccugaaguagcgcgc.............    1       1    MF2
............................................................ucagguaccugCaguagcgcgc.............    1       1    MF2
............................................................ucagguaccugGaguagcgcgc.............    2       1    MF2
............................................................ucaggAaccugaaguagcgcgc.............    1       1    MF2
............................................................ucagguaUcugaaguagcgcgc.............    1       1    MF2
............................................................uAagguaccugaaguagcgcgcg............    1       1    MF2
............................................................ucagguaccugaaguagcgcgcA............    15      1    MF2
............................................................ucaggCaccugaaguagcgcgcg............    3       1    MF2
............................................................ucagguaccugGaguagcgcgcg............    1       1    MF2
............................................................ucagguaccugaaguagcgcgcC............    195     1    MF2
............................................................ucagguaccugaaguagcgUgcg............    1       1    MF2
............................................................ucagguaccugaaguagcgcgGg............    1       1    MF2
............................................................ucagguaccugaaguaCcgcgcg............    1       1    MF2
............................................................ucagguaccugaaguagcgcgcU............    57      1    MF2
............................................................ucagguaccugaaCuagcgcgcg............    2       1    MF2
............................................................ucagguaccugaaguagcAcgcg............    1       1    MF2
............................................................ucagguaccugaaguagAgcgcg............    2       1    MF2
............................................................ucagguaccugaaguagUgcgcg............    1       1    MF2
............................................................ucagguaccGgaaguagcgcgcg............    1       1    MF2
............................................................ucagguGccugaaguagcgcgcg............    2       1    MF2
............................................................ucagguaccugaaguagcgcgcg............    3325    0    MF2
............................................................ucagguaccugaagCagcgcgcg............    2       1    MF2
............................................................ucagguaccugaCguagcgcgcg............    1       1    MF2
............................................................ucaggGaccugaaguagcgcgcg............    1       1    MF2
............................................................ucagguaccugaaguGgcgcgcg............    18      1    MF2
............................................................ucGgguaccugaaguagcgcgcg............    2       1    MF2
............................................................ucagguaccugaaguUgcgcgcg............    1       1    MF2
............................................................ucagguaccugaaguagcgcgUg............    1       1    MF2
............................................................ucagguaccugaagGagcgcgcg............    1       1    MF2
............................................................ucagguaccugUaguagcgcgcg............    4       1    MF2
............................................................ucagguaccAgaaguagcgcgcg............    1       1    MF2
............................................................ucagguaccCgaaguagcgcgcg............    2       1    MF2
............................................................ucagguaccuAaaguagcgcgcg............    3       1    MF2
............................................................ucagguaccugaGguagcgcgcg............    1       1    MF2
............................................................uUagguaccugaaguagcgcgcg............    1       1    MF2
............................................................ucagguaccugaaguagcgcgcgG...........    1       1    MF2
............................................................ucagguaccugaaguagcgcgcgC...........    44      1    MF2
............................................................ucagguaccugaaguagcgcgcCu...........    155     1    MF2
............................................................ucagguaccCgaaguagcgcgcgu...........    1       1    MF2
............................................................ucagguaccugaaguagcgcgcgu...........    309     0    MF2
............................................................ucagguaccugaaguagcgcgcgA...........    390     1    MF2
............................................................ucagguaccugaaguagcgcgcUu...........    65      1    MF2
............................................................ucagguaccugaaguGgcgcgcgu...........    1       1    MF2
```

```
                                                            aga-miR-275
                 aga-miR-275*
ugagccgucuaaugacacgcgcuaagcaggaaccgggacuugguacacauucgcuagcagucagguaccugaaguagcgcgcguuauucggcuca

............................................................ucagguaccugaaUuagcgcgcgu...........   1      1      MF2
............................................................ucagguaccugaagCagcgcgcgu...........   1      1      MF2
............................................................ucagguaccuAaaguagcgcgcgu...........   1      1      MF2
............................................................ucaggGaccugaaguagcgcgcgu...........   1      1      MF2
............................................................ucagguaccugaaguagcgcgcAu...........   7      1      MF2
............................................................ucagguaccugaaguagcgcgcgCu..........   2      1      MF2
............................................................ucagguaccugaaguagcgcgcguA..........   2      1      MF2
............................................................ucagguaccugaaguagcgcgcguC..........   1      1      MF2
............................................................ucagguaccugaaguagcgcgcguu..........   30     0      MF2
............................................................ucagguaccugaaguagcgcgcUuu..........   1      1      MF2
............................................................ucagguaccugaaguagcgcgcgAu..........   102    1      MF2
............................................................ucagguaccugaaguagcgcgcguuU.........   23     1      MF2
............................................................ucagguaccugaaguagcgcgcguGa.........   2      1      MF2
............................................................ucagguaccugaaguagcgcgcguuC.........   2      1      MF2
............................................................ucagguaccugaaguagcgcgcguCa.........   1      1      MF2
............................................................ucagguaccugaaguagcgcgcUuua.........   6      1      MF2
............................................................ucagguaccugaaguagcgcgcguua.........   5      0      MF2
............................................................ucagguaccugaaguagcgcgcguuG.........   1      1      MF2
............................................................ucagguaccugaaguagcgcgcguAa.........   10     1      MF2
............................................................ucagguaccugaaguagcgcgcgAua.........   12     1      MF2
............................................................ucagguaccugaaguagcgcgcguuaA........   2      1      MF2
............................................................ucagguaccugaaguagcgcgcguuUu........   2      1      MF2
............................................................ucagguaccugaaguagcgcgcguuau........   4      0      MF2
............................................................ucagguaccugaaguagcgcgcgAuau........   7      1      MF2
............................................................ucagguaccugaaguagcgcgcguAau........   13     1      MF2
............................................................ucagguaccugaaguagcgcgcguuUuu.......   3      1      MF2
............................................................ucagguaccugaaguagcgcgcguuaAu.......   3      1      MF2
.............................................................cagguaccugaaguagcgcgc.............   2      0      MF2
.............................................................cagguaccugaaguagcgcgcU............   1      1      MF2
.............................................................cagguaccugaaguagcgcgcUu...........   1      1      MF2
..............................................................agguaccugaaguagcgcgc.............   1      0      MF2
..............................................................agguaccugaaguagcgcgcg............   2      0      MF2

ugagccgucuaaugaca..............................................................................   1      0      FW2
................acgcgcuaagcaggaaccgggacu.......................................................   1      0      FW2
................acgcgcuaagcaggaaccgggacuuU.....................................................   1      1      FW2
.................cgcgcuaagcaggaaccggga.........................................................   2      0      FW2
.................cgcgcuaagcaggaaccgggac........................................................   12     0      FW2
.................cgcgcuaagcaggaaccgggacu.......................................................   59     0      FW2
.................cgcgcuaagcaggaaccgggacuu......................................................   1      0      FW2
.................cgcgcuaagcaggaaccgggacuugguacacauucgcuagcag...................................   2      0      FW2
........................................ugguacacauucgcuagcag...................................   1      0      FW2
...........................................................Cucagguaccugaaguagcgcgc.............   1      1      FW2
............................................................ucagguaccugaaguag..................   1      0      FW2
............................................................ucagguaccugaaguagc.................   3      0      FW2
............................................................ucagguaccugaaguagcg................   3      0      FW2
............................................................ucagguaccugaaguagcgc...............   73     0      FW2
............................................................ucagguaccugaaguagcUc...............   1      1      FW2
............................................................ucagguaccugaaguagcgcg..............   55     0      FW2
............................................................ucagguaccugGaguagcgcgc.............   1      1      FW2
............................................................ucagguaccugaaguagcgcCc.............   1      1      FW2
............................................................ucagguaccCgaaguagcgcgc.............   1      1      FW2
............................................................ucagguaccuCaaguagcgcgc.............   1      1      FW2
............................................................ucagguaccugaaguagcgcgU.............   1      1      FW2
............................................................ucagguaccugaaguagcgcAc.............   1      1      FW2
............................................................uUagguaccugaaguagcgcgc.............   1      1      FW2
............................................................Acagguaccugaaguagcgcgc.............   1      1      FW2
............................................................ucagguaccugaaAuagcgcgc.............   1      1      FW2
............................................................ucagguaccugaaguagcgcUc.............   1      1      FW2
............................................................ucagguaccugaaguagAgcgc.............   1      1      FW2
............................................................ucagguaccugaagAagcgcgc.............   1      1      FW2
............................................................ucagguaccugaaguaAcgcgc.............   1      1      FW2
............................................................ucagguaccugaaguagcgcgc.............   1075   0      FW2
............................................................ucagguaccugaaguagcgcgcA............   3      1      FW2
............................................................ucagguaccugaaguagcgcgcU............   2      1      FW2
............................................................ucagguaccugaaguagcgcgcC............   26     1      FW2
............................................................ucagguaccuAaaguagcgcgcg............   1      1      FW2
............................................................ucagguaccugaaguagcgcgcg............   446    0      FW2
............................................................ucagguaccugaaguGgcgcgcg............   1      1      FW2
```

```
                                                            aga-miR-275
                 aga-miR-275*
ugagccgucuaaugacacgcgcuaagcaggaaccgggacuugguacacauucgcuagcagucagguaccugaaguagcgcgcguuauucggcuca

............................................................ucaggCaccugaaguagcgcgcg............    1         1         FW2
............................................................uUagguaccugaaguagcgcgcg............    1         1         FW2
............................................................ucagguaccugaaguagcgcgcgC...........    9         1         FW2
............................................................ucagguaccugaaguagcgcgcgA...........    17        1         FW2
............................................................ucagguaUcugaaguagcgcgcgu...........    1         1         FW2
............................................................ucagguaccugaaguagcgcgcgu...........    29        0         FW2
............................................................ucagguaccugaaguagcgcgcCu...........    7         1         FW2
............................................................ucagguaccugaaguagcgcgcguA..........    1         1         FW2
............................................................ucagguaccugaaguagcgcgcgAu..........    3         1         FW2
............................................................ucagguaccugaaguagcgcgcguu..........    5         0         FW2
............................................................ucagguaccugaaguagcgcgcguAa.........    1         1         FW2
............................................................ucagguaccugaaguagcgcgcguuU.........    1         1         FW2
............................................................ucagguaccugaaguagcgcgcguAau........    1         1         FW2
..............................................................agguaccugaaguagcgcg..............    1         0         FW2
..............................................................agguaccugaaguagcgcgcg............    2         0         FW2
...............................................................Cguaccugaaguagcgc...............    1         1         FW2

.................cgcgcuaagcaggaaccggga.........................................................    1         0         OV1
.................cgcgcuaagcaggaaccgggaA........................................................    1         1         OV1
.................cgcgcuaagcaggaaccgggac........................................................    4         0         OV1
.................cgcgcuaagcaggaaccgggacu.......................................................    24        0         OV1
.................cgcgcuaagcaggaaccgggacuugg....................................................    2         0         OV1
.................cgcgcuaagcaggaaccgggacuuggua..................................................    1         0         OV1
...........................................................Cucagguaccugaaguagcgcgc.............    2         1         OV1
............................................................ucagguaccugaaguagcg................    4         0         OV1
............................................................ucagguaccuAaaguagcgc...............    2         1         OV1
............................................................ucagguaccugaaguagcgc...............    73        0         OV1
............................................................ucagguaccugaaguagcgcg..............    70        0         OV1
............................................................ucagguaccugaaguagcgcC..............    1         1         OV1
............................................................ucagguaccugaaguGgcgcg..............    1         1         OV1
............................................................ucagguaccugaagCagcgcg..............    1         1         OV1
............................................................ucGgguaccugaaguagcgcgc.............    1         1         OV1
............................................................ucagguaccugaaguagcgcgc.............    1284      0         OV1
............................................................ucagguaccuCaaguagcgcgc.............    1         1         OV1
............................................................ucagguaccugUaguagcgcgc.............    1         1         OV1
............................................................ucagguaccugaaguagAgcgc.............    1         1         OV1
............................................................ucUgguaccugaaguagcgcgc.............    1         1         OV1
............................................................Acagguaccugaaguagcgcgc.............    1         1         OV1
............................................................ucagguaccuAaaguagcgcgc.............    3         1         OV1
............................................................ucagguaccugaaguagcgcgG.............    1         1         OV1
............................................................ucagguaccAgaaguagcgcgc.............    1         1         OV1
............................................................ucaggGaccugaaguagcgcgc.............    2         1         OV1
............................................................ucagguaccugaaguagcgcgA.............    4         1         OV1
............................................................ucagguaccugaaguaAcgcgc.............    1         1         OV1
............................................................ucagguaccugaaguGgcgcgc.............    3         1         OV1
............................................................ucagguaccugGaguagcgcgc.............    2         1         OV1
............................................................ucagguaccugaagCagcgcgc.............    1         1         OV1
............................................................ucagguaccugaaguagcAcgc.............    1         1         OV1
............................................................ucaggAaccugaaguagcgcgc.............    1         1         OV1
............................................................ucagguaccugaaguagcgcgU.............    1         1         OV1
............................................................ucaggCaccugaaguagcgcgc.............    2         1         OV1
............................................................ucagguaccugaagGagcgcgc.............    1         1         OV1
............................................................ucCgguaccugaaguagcgcgc.............    1         1         OV1
............................................................ucagguaccugaaguGgcgcgcg............    3         1         OV1
............................................................ucagguaccugaaguagcgcgcg............    527       0         OV1
............................................................ucagguaccugaaguagcgcgcU............    12        1         OV1
............................................................ucagguacUugaaguagcgcgcg............    1         1         OV1
............................................................Acagguaccugaaguagcgcgcg............    1         1         OV1
............................................................ucagguaccugaaguagcgcgGg............    2         1         OV1
............................................................ucagguaccugaaguagcgcgcC............    12        1         OV1
............................................................ucagguaccugaaguagcgcAcg............    1         1         OV1
............................................................ucagguaccugaaguagcgcgAg............    1         1         OV1
............................................................ucagguaccugaaguagcgcgcA............    5         1         OV1
............................................................ucagguaccugaaguagcgcgcUu...........    5         1         OV1
............................................................ucagguaccugaaguagcgcgcgC...........    21        1         OV1
............................................................ucagguaccugaaguagcgcgcgA...........    27        1         OV1
............................................................ucagguaccugaaguagcgcgcCu...........    11        1         OV1
............................................................ucagguaccugaaguagcgcgcgu...........    71        0         OV1
............................................................ucagguaccugaaguagcgcgcguu..........    4         0         OV1
```

```
                                                            aga-miR-275
                 aga-miR-275*
ugagccgucuaaugacacgcgcuaagcaggaaccgggacuugguacacauucgcuagcagucagguaccugaaguagcgcgcguuauucggcuca

............................................................ucagguaccugaaguagcgcgcgAu..........    6      1      OV1
............................................................ucagguaccugaaguagcgcgcguuU.........    1      1      OV1
............................................................ucagguaccugaaguagcgcgcguua.........    1      0      OV1
............................................................ucagguaccugaaguagcgcgcguAau........    2      1      OV1
............................................................ucagguaccugaaguagcgcgcguuau........    1      0      OV1
............................................................ucagguaccugaaguagcgcgcgAuau........    1      1      OV1

.................cgcgcuaagcaggaacc..............................................................    1      0      FF1
.................cgcgcuaagcaggaaccgggac.........................................................    2      0      FF1
.................cgcgcuaagcaggaaccgggacu........................................................    34     0      FF1
.................cgcgcuaagcaggaaccgggacuu.......................................................    2      0      FF1
.................cgcgcuaagcaggaaccgggacuuggua...................................................    1      0      FF1
.................cgcgcuaagcaggaaccgggacuugguaca.................................................    1      0      FF1
.................cgcgcuaagcaggaaccgggacuugguacaca...............................................    1      0      FF1
.................cgcgcuaagcaggaaccgggacuugguacacauucgcuagcag...................................    1      0      FF1
...........................................uacacauucgcuagcagucagguacc..........................    1      0      FF1
............................................................ucagguaccugaaguagc.................    1      0      FF1
............................................................ucagguaccugaaguagcgc...............    18     0      FF1
............................................................ucagguaccugaaguagcgcg..............    39     0      FF1
............................................................ucagguaccugaaguagcgcgc.............    610    0      FF1
............................................................ucagguaccugaaguagcgcgU.............    1      1      FF1
............................................................ucagguaccugCaguagcgcgc.............    1      1      FF1
............................................................ucagguaccugaaguagcgcgA.............    1      1      FF1
............................................................ucagguaccCgaaguagcgcgc.............    1      1      FF1
............................................................ucGgguaccugaaguagcgcgc.............    1      1      FF1
............................................................ucagguaccuAaaguagcgcgc.............    1      1      FF1
............................................................ucaggCaccugaaguagcgcgc.............    1      1      FF1
............................................................ucagguaccugUaguagcgcgc.............    1      1      FF1
............................................................ucagguaccugaagCagcgcgc.............    2      1      FF1
............................................................uUagguaccugaaguagcgcgc.............    1      1      FF1
............................................................ucagguaccugaaguGgcgcgc.............    1      1      FF1
............................................................ucaggGaccugaaguagcgcgc.............    1      1      FF1
............................................................ucGgguaccugaaguagcgcgcg............    1      1      FF1
............................................................ucagguaccuAaaguagcgcgcg............    1      1      FF1
............................................................ucagguaccugaaguagcgcgcg............    1046   0      FF1
............................................................ucagguaccuCaaguagcgcgcg............    1      1      FF1
............................................................uUagguaccugaaguagcgcgcg............    1      1      FF1
............................................................ucagguaccugaaguagcgcgcU............    8      1      FF1
............................................................ucagguaccugaaguagcgcgcA............    2      1      FF1
............................................................ucagguacUugaaguagcgcgcg............    2      1      FF1
............................................................ucagguaccugaaguagcgcAcg............    1      1      FF1
............................................................ucagguaccugaaguagUgcgcg............    1      1      FF1
............................................................ucagguaccugaaguagcgcgcC............    55     1      FF1
............................................................ucagAuaccugaaguagcgcgcg............    1      1      FF1
............................................................ucagguaccugaaguagcgcgcCu...........    4      1      FF1
............................................................ucagguaccugaaguagcgcgcgC...........    22     1      FF1
............................................................ucagguaccugaaguagcgcgcgu...........    39     0      FF1
............................................................ucagguaccugaaguagcgcgcgA...........    16     1      FF1
............................................................ucagguaccugaaguagcgcgcgAu..........    3      1      FF1
............................................................ucagguaccugaaguagcgcgcCuu..........    1      1      FF1
............................................................ucagguaccugaaguagcgcgcguuC.........    1      1      FF1

.................cgcgcuaagcaggaaccggga.........................................................    1      0      MF1
.................cgcgcuaagcaggaaccgggac........................................................    9      0      MF1
.................cgcgcuaagcaggaaccggAacu.......................................................    1      1      MF1
.................cgcgcuaagcaggaaccgggacu.......................................................    97     0      MF1
.................cgcgcuaagcaggaaccgggacuu......................................................    5      0      MF1
.................cgcgcuaagcaggaaccgggacuuA.....................................................    1      1      MF1
.................cgcgcuaagcaggaaccgggacuuggu...................................................    1      0      MF1
........................................ugguacacauucgcuagcag...................................    1      0      MF1
............................................................ucagguaccugaaguagc.................    3      0      MF1
............................................................ucagguaccugaaguagcg................    13     0      MF1
............................................................ucagguaccugaaguagcgc...............    120    0      MF1
............................................................uUagguaccugaaguagcgc...............    1      1      MF1
............................................................ucagguaccugaaguagcgU...............    1      1      MF1
............................................................ucagguaccugaaguagcgcg..............    69     0      MF1
............................................................ucagguaccugaaguagcgcC..............    1      1      MF1
............................................................ucagguaccugaaguagcgcU..............    5      1      MF1
............................................................ucagCuaccugaaguagcgcg..............    1      1      MF1
```

```
                                                            aga-miR-275
                 aga-miR-275*
ugagccgucuaaugacacgcgcuaagcaggaaccgggacuugguacacauucgcuagcagucagguaccugaaguagcgcgcguuauucggcuca

............................................................Ccagguaccugaaguagcgcg..............    1      1      MF1
............................................................ucagguaccugaaguGgcgcgc.............    1      1      MF1
............................................................uUagguaccugaaguagcgcgc.............    2      1      MF1
............................................................ucagguaccugaaguCgcgcgc.............    1      1      MF1
............................................................ucagguaccugaaguagcgcgU.............    4      1      MF1
............................................................ucagAuaccugaaguagcgcgc.............    2      1      MF1
............................................................ucagguaccugaaAuagcgcgc.............    1      1      MF1
............................................................ucagguaccugaaguagcgcgA.............    1      1      MF1
............................................................ucagguaccuAaaguagcgcgc.............    1      1      MF1
............................................................ucagUuaccugaaguagcgcgc.............    1      1      MF1
............................................................ucagguaccugaaCuagcgcgc.............    1      1      MF1
............................................................ucagguaccugaaguagcgcAc.............    2      1      MF1
............................................................ucagguaccugaaguagcgcCc.............    2      1      MF1
............................................................ucagguaccugaaguagAgcgc.............    1      1      MF1
............................................................ucaAguaccugaaguagcgcgc.............    1      1      MF1
............................................................ucGgguaccugaaguagcgcgc.............    1      1      MF1
............................................................ucagguaccugaaguagcgcgc.............    1330   0      MF1
............................................................ucagguaccugaaguagAgcgcg............    1      1      MF1
............................................................ucagguaccugaaguagcgcAcg............    1      1      MF1
............................................................ucagguUccugaaguagcgcgcg............    2      1      MF1
............................................................ucagguaccugaaguagcgcgGg............    1      1      MF1
............................................................ucagguaccugaaguagcgcgcA............    4      1      MF1
............................................................ucagguaccugaaguGgcgcgcg............    2      1      MF1
............................................................ucagguaccugGaguagcgcgcg............    1      1      MF1
............................................................ucagguaccuAaaguagcgcgcg............    1      1      MF1
............................................................ucagguaccugaaguagcgcgcU............    9      1      MF1
............................................................ucagguaccugaagGagcgcgcg............    1      1      MF1
............................................................ucagguaccugaaguagcgcgcg............    722    0      MF1
............................................................ucagguaccugaaguagcgcgcC............    51     1      MF1
............................................................ucaggGaccugaaguagcgcgcg............    1      1      MF1
............................................................ucagguaccugaagCagcgcgcg............    1      1      MF1
............................................................ucagguaccugaaguagcgcgcCu...........    27     1      MF1
............................................................ucagguaccugaaguagcgcgcgA...........    58     1      MF1
............................................................ucagguaccugaaguagcgcgcUu...........    3      1      MF1
............................................................ucagguaccugaaguagcgcgcgu...........    55     0      MF1
............................................................ucagguaccugaaguagcgcgcgC...........    5      1      MF1
............................................................ucaggCaccugaaguagcgcgcgu...........    1      1      MF1
............................................................ucagguaccugaaguagcgcgcguG..........    1      1      MF1
............................................................ucagguaccugaaguagcgcgcUuu..........    2      1      MF1
............................................................ucagguaccugaaguagcgcgcguu..........    7      0      MF1
............................................................ucagguaccugaaguagcgcgcgAu..........    10     1      MF1
............................................................ucagguaccugaaguagcgcgcgAua.........    1      1      MF1
............................................................ucagguaccugaaguagcgcgcguuU.........    3      1      MF1
............................................................ucagguaccugaaguagcgcgcguuC.........    1      1      MF1
............................................................ucagguaccugaaguagcgcgcguAau........    1      1      MF1
............................................................ucagguaccugaaguagcgcgcguuaA........    1      1      MF1

.................cgcgcuaagcaggaaccggga.........................................................    7      0      BF2
.................cgcgcuaagcaggaaccgggac........................................................    4      0      BF2
.................cgcgcuaagcaggaaccgggacu.......................................................    78     0      BF2
.................cgcgcuaagcaggaaccgggacG.......................................................    1      1      BF2
.................Ugcgcuaagcaggaaccgggacu.......................................................    1      1      BF2
.................cgcgcuaagcaggaaccgggacuu......................................................    2      0      BF2
.................cgcgcuaagcaggaaccgggacuugg....................................................    3      0      BF2
.................cgcgcuaagcaggaaccgggacuuggua..................................................    1      0      BF2
.................cgcgcuaagcaggaaccgggacuugguacacauuc...........................................    1      0      BF2
........................................ugguacacauucgcuagcag...................................    1      0      BF2
...........................................................Cucagguaccugaaguagcgcgcg............    1      1      BF2
............................................................ucagguaccugaaguag..................    1      0      BF2
............................................................ucagguaccugaaguagcg................    2      0      BF2
............................................................ucagguaccugaaguagcgc...............    20     0      BF2
............................................................uUagguaccugaaguagcgc...............    1      1      BF2
............................................................ucagguaccugaaguagcgcg..............    43     0      BF2
............................................................ucagguaccCgaaguagcgcgc.............    1      1      BF2
............................................................ucagguaccugaaguagcgUgc.............    1      1      BF2
............................................................ucagguaccuUaaguagcgcgc.............    2      1      BF2
............................................................ucagguaccugaagGagcgcgc.............    1      1      BF2
............................................................ucagguaccugaaguagcgcgc.............    968    0      BF2
............................................................ucagguaccuAaaguagcgcgc.............    1      1      BF2
```

```
                                                            aga-miR-275
                 aga-miR-275*
ugagccgucuaaugacacgcgcuaagcaggaaccgggacuugguacacauucgcuagcagucagguaccugaaguagcgcgcguuauucggcuca

............................................................Ccagguaccugaaguagcgcgc.............    1      1      BF2
............................................................ucGgguaccugaaguagcgcgc.............    1      1      BF2
............................................................ucagguaccugaaguGgcgcgc.............    1      1      BF2
............................................................ucGgguaccugaaguagcgcgcg............    1      1      BF2
............................................................ucagguaccuAaaguagcgcgcg............    2      1      BF2
............................................................ucagguaccugaaguagcgcgcU............    5      1      BF2
............................................................uUagguaccugaaguagcgcgcg............    3      1      BF2
............................................................ucagguaccugaaguGgcgcgcg............    1      1      BF2
............................................................ucagguaccCgaaguagcgcgcg............    1      1      BF2
............................................................ucagguaccugGaguagcgcgcg............    1      1      BF2
............................................................ucagguaccugaaguagcgcgUg............    1      1      BF2
............................................................ucagguaccugaaguagcgcgcg............    985    0      BF2
............................................................ucagguacAugaaguagcgcgcg............    1      1      BF2
............................................................ucagguaccugaaguaCcgcgcg............    1      1      BF2
............................................................ucagguaccuCaaguagcgcgcg............    1      1      BF2
............................................................ucagguaccugaaguagcgcgcC............    43     1      BF2
............................................................ucagguaccugaaguagcgcgcA............    1      1      BF2
............................................................ucagguaccugaaguagcgcAcg............    1      1      BF2
............................................................ucagguaccugaaguagcgcgcUu...........    3      1      BF2
............................................................ucagguaccugaaguagcgcgcgC...........    19     1      BF2
............................................................ucagguaccugaaguagcgcgcgG...........    1      1      BF2
............................................................ucagguaccugaaguagcgcgcgA...........    7      1      BF2
............................................................ucagguaccugaaguagcgcgcCu...........    5      1      BF2
............................................................ucagguaccugaaguagcgcgcgu...........    37     0      BF2
............................................................ucagguaccugaaguagcgcgcguu..........    1      0      BF2
............................................................ucagguaccugaaguagcgcgcgAu..........    2      1      BF2
............................................................ucagguaccugaaguagcgcgcguAau........    1      1      BF2
..............................................................agguaccugaaguagcgcgcgu...........    1      0      BF2
...................................................................ccugaaguagcgcgcgu...........    1      0      BF2

.................cgcgcuaagcaggaacc.............................................................    1      0      BF1
.................cgcgcuaagcaggaaccgggac........................................................    2      0      BF1
.................cgcgcuaagcaggaaccgggacu.......................................................    34     0      BF1
.................cgcgcuaagcaggaaccgggacuu......................................................    2      0      BF1
.................cgcgcuaagcaggaaccgggacuuggua..................................................    1      0      BF1
.................cgcgcuaagcaggaaccgggacuugguaca................................................    1      0      BF1
.................cgcgcuaagcaggaaccgggacuugguacaca..............................................    1      0      BF1
.................cgcgcuaagcaggaaccgggacuugguacacauucgcuagcag...................................    1      0      BF1
...........................................uacacauucgcuagcagucagguacc..........................    1      0      BF1
............................................................ucagguaccugaaguagc.................    1      0      BF1
............................................................ucagguaccugaaguagcgc...............    18     0      BF1
............................................................ucagguaccugaaguagcgcg..............    39     0      BF1
............................................................ucagguaccugaaguagcgcgU.............    1      1      BF1
............................................................ucagguaccCgaaguagcgcgc.............    1      1      BF1
............................................................ucGgguaccugaaguagcgcgc.............    1      1      BF1
............................................................ucagguaccugCaguagcgcgc.............    1      1      BF1
............................................................ucaggCaccugaaguagcgcgc.............    1      1      BF1
............................................................ucaggGaccugaaguagcgcgc.............    1      1      BF1
............................................................ucagguaccugUaguagcgcgc.............    1      1      BF1
............................................................ucagguaccugaaguagcgcgA.............    1      1      BF1
............................................................ucagguaccuAaaguagcgcgc.............    1      1      BF1
............................................................uUagguaccugaaguagcgcgc.............    1      1      BF1
............................................................ucagguaccugaaguagcgcgc.............    608    0      BF1
............................................................ucagguaccugaaguGgcgcgc.............    1      1      BF1
............................................................ucagguaccugaagCagcgcgc.............    2      1      BF1
............................................................ucagguaccuCaaguagcgcgcg............    1      1      BF1
............................................................ucagguaccugaaguagcgcgcU............    8      1      BF1
............................................................ucagAuaccugaaguagcgcgcg............    1      1      BF1
............................................................ucagguacUugaaguagcgcgcg............    2      1      BF1
............................................................ucagguaccugaaguagcgcgcA............    2      1      BF1
............................................................ucagguaccugaaguagcgcAcg............    1      1      BF1
............................................................ucGgguaccugaaguagcgcgcg............    1      1      BF1
............................................................ucagguaccugaaguagcgcgcC............    55     1      BF1
............................................................ucagguaccugaaguagcgcgcg............    1046   0      BF1
............................................................uUagguaccugaaguagcgcgcg............    1      1      BF1
............................................................ucagguaccuAaaguagcgcgcg............    1      1      BF1
............................................................ucagguaccugaaguagcgcgcgC...........    22     1      BF1
............................................................ucagguaccugaaguagcgcgcgA...........    16     1      BF1
............................................................ucagguaccugaaguagcgcgcCu...........    4      1      BF1
```

```
                                                                             aga-miR-275
                 aga-miR-275*
ugagccgucuaaugacacgcgcuaagcaggaaccgggacuugguacacauucgcuagcagucagguaccugaaguagcgcgcguuauucggcuca

............................................................ucagguaccugaaguagcgcgcgu...........    38    0    BF1
............................................................ucagguaccugaaguagcgcgcgAu..........    3     1    BF1
............................................................ucagguaccugaaguagcgcgcCuu..........    1     1    BF1
............................................................ucagguaccugaaguagcgcgcguuC.........    1     1    BF1

.................cgcgcuaagcaggaaccgggac........................................................    5     0    MW1
.................cgcgcuaagcaggaaccgggacu.......................................................    91    0    MW1
.................cgcgcCaagcaggaaccgggacu.......................................................    1     1    MW1
.................cgcgcuaagcaggaaccgggacuugg....................................................    1     0    MW1
............................ggaaccgggacuugguacacauucg..........................................    1     0    MW1
...........................................................Cucagguaccugaaguagcgcgc.............    1     1    MW1
............................................................ucagguaccugaaguagc.................    2     0    MW1
............................................................ucagguaccugaaguagcgc...............    65    0    MW1
............................................................ucagguaccugaaguagcgcg..............    26    0    MW1
............................................................ucaggGaccugaaguagcgcgc.............    1     1    MW1
............................................................ucagguaccugaaguagcgcgc.............    749   0    MW1
............................................................ucagguaccuCaaguagcgcgc.............    1     1    MW1
............................................................ucaUguaccugaaguagcgcgc.............    1     1    MW1
............................................................ucaggAaccugaaguagcgcgc.............    1     1    MW1
............................................................ucagAuaccugaaguagcgcgc.............    1     1    MW1
............................................................ucagguaccugaaguagcgcAc.............    1     1    MW1
............................................................ucagguaccuAaaguagcgcgc.............    2     1    MW1
............................................................ucagguaccugGaguagcgcgcg............    1     1    MW1
............................................................ucagguaccugaaguGgcgcgcg............    2     1    MW1
............................................................Gcagguaccugaaguagcgcgcg............    1     1    MW1
............................................................ucagguaccugaaguagcgcgcC............    5     1    MW1
............................................................ucagguaccugaaguagcgcgcU............    2     1    MW1
............................................................ucagguaccugaaguagcgcAcg............    1     1    MW1
............................................................ucagguaccugaaguagcgUgcg............    1     1    MW1
............................................................ucagguaccugaaguagcgcgcA............    1     1    MW1
............................................................uUagguaccugaaguagcgcgcg............    1     1    MW1
............................................................ucagguaccugaaguagcgcgcg............    353   0    MW1
............................................................ucagguaccuAaaguagcgcgcgu...........    1     1    MW1
............................................................ucagguaccugaaguagcgcgcgu...........    31    0    MW1
............................................................ucagguaccugaaguagcgcgcUu...........    3     1    MW1
............................................................ucagguaccugaaguagcgcgcCu...........    4     1    MW1
............................................................ucagguaccugaaguagcgcgcgC...........    8     1    MW1
............................................................ucagguaccugaaguagcgcgcgA...........    13    1    MW1
............................................................ucagguaccugaaguagcgcgcguuU.........    1     1    MW1

.................cgcgcuGagcaggaaccggga.........................................................    1     1    FW1
.................cgcgcuaagcaggaaccggga.........................................................    2     0    FW1
.................cgcgcuaagcaggaaccgggaU........................................................    1     1    FW1
.................cgcgcuaagcaggaaccgggac........................................................    2     0    FW1
.................cgcgcuaagcaggaaccgggacu.......................................................    42    0    FW1
.................cgcgcuaagcaggaUccgggacu.......................................................    1     1    FW1
............................................................ucagguaccugaaguag..................    1     0    FW1
............................................................ucagguaccugaaguagc.................    3     0    FW1
............................................................ucagguaccugaaguagcg................    1     0    FW1
............................................................ucagguaccugaaguagcgc...............    35    0    FW1
............................................................ucagguaccugaaguagcgcg..............    22    0    FW1
............................................................ucagguaccugaaguagcgcU..............    1     1    FW1
............................................................ucagguaccugaaguagcgcgc.............    442   0    FW1
............................................................uUagguaccugaaguagcgcgc.............    1     1    FW1
............................................................ucagguaccugaaguGgcgcgc.............    1     1    FW1
............................................................ucagAuaccugaaguagcgcgc.............    1     1    FW1
............................................................ucagguaccugaaguagcgcgU.............    1     1    FW1
............................................................ucagguaccugaaguGgcgcgcg............    1     1    FW1
............................................................ucagguaccugaagCagcgcgcg............    1     1    FW1
............................................................ucagguaccugaaguagcgcgcU............    1     1    FW1
............................................................ucagguaccugaaguagcgcgcC............    10    1    FW1
............................................................ucagguaccugaaguagcgcgcg............    183   0    FW1
............................................................ucagguaccugaaguagcCcgcg............    1     1    FW1
............................................................ucagguaccugaaguagcgcgcgu...........    14    0    FW1
............................................................ucagguaccugaaguagcgcgcCu...........    2     1    FW1
............................................................ucagguaccugaaguagcgcgcgA...........    13    1    FW1
............................................................ucagguaccugaaguagcgcgcgC...........    5     1    FW1
............................................................ucagguaccugaaguagcgcgcguu..........    1     0    FW1
............................................................ucagguaccugaaguagcgcgcgAu..........    1     1    FW1
```

```
                                                            aga-miR-275
                 aga-miR-275*
ugagccgucuaaugacacgcgcuaagcaggaaccgggacuugguacacauucgcuagcagucagguaccugaaguagcgcgcguuauucggcuca

............................................................ucagguaccugaaguagcgcgcguAa.........   1     1    FW1
............................................................ucagguaccugaaguagcgcgcguuUu........   1     1    FW1
.............................................................cagguaccugaagCagcgcgcg............   1     1    FW1

.................cgcgcuaagcaggaaccggga.........................................................   1     0    MW2
.................cgcgcuaagcaggaaccgggac........................................................   6     0    MW2
.................cgcgcuaagcaggaaccgggacC.......................................................   1     1    MW2
.................cgcgcuaagcaggaaccgggacu.......................................................   37    0    MW2
............................................................ucagguaccugaaguagc.................   1     0    MW2
............................................................ucagguaccugaaguagcg................   1     0    MW2
............................................................ucagguaccugaaguagcgc...............   21    0    MW2
............................................................ucagguaccugaagGagcgcg..............   1     1    MW2
............................................................ucagguaccugaaguagcgcg..............   15    0    MW2
............................................................ucagguaccugaaguagcgcAc.............   1     1    MW2
............................................................ucagAuaccugaaguagcgcgc.............   1     1    MW2
............................................................uUagguaccugaaguagcgcgc.............   1     1    MW2
............................................................ucagguaccugaaguagcgGgc.............   1     1    MW2
............................................................ucagguaccugaaguagcgcgc.............   268   0    MW2
............................................................ucagguaccugaaguagcgcgcC............   7     1    MW2
............................................................Ccagguaccugaaguagcgcgcg............   1     1    MW2
............................................................ucaggCaccugaaguagcgcgcg............   1     1    MW2
............................................................ucagguaccugaaguagcgcgcg............   117   0    MW2
............................................................ucagguUccugaaguagcgcgcg............   1     1    MW2
............................................................ucagguaccugaaguagcgcgcgC...........   3     1    MW2
............................................................ucagguaccugaaguagcgcgcCu...........   1     1    MW2
............................................................ucagguaccugaaguagcgcgcgu...........   13    0    MW2
............................................................ucagguaccugaaguagcgcgcgAua.........   1     1    MW2
............................................................ucagguaccugaaguagcgcgcguAa.........   1     1    MW2
.............................................................cagguaccugaaguagcgcg..............   1     0    MW2

.................cgcgcuaagcaggaaccggga.........................................................   2     0    TE2
.................cgcgcuaagcaggaaccgggac........................................................   2     0    TE2
.................cgcgcuaagcaggaacUgggacu.......................................................   1     1    TE2
.................cgcgcuaagcaggaaccgggacu.......................................................   18    0    TE2
.................cgcgcuaagcaggaaccgggacC.......................................................   2     1    TE2
.................cgcgcuaagcaggaaccgggacuu......................................................   1     0    TE2
............................................................ucagguaccugaaguagcg................   1     0    TE2
............................................................ucagguaccugaaguagcgc...............   216   0    TE2
............................................................ucagguaccugaaguagcgcA..............   1     1    TE2
............................................................ucaggCaccugaaguagcgcg..............   1     1    TE2
............................................................ucagUuaccugaaguagcgcg..............   1     1    TE2
............................................................ucagguaccugGaguagcgcg..............   1     1    TE2
............................................................ucagguaccugaaguagcgcg..............   109   0    TE2
............................................................ucaggGaccugaaguagcgcg..............   1     1    TE2
............................................................ucGgguaccugaaguagcgcg..............   1     1    TE2
............................................................ucagguaccugaaguagcgcgA.............   2     1    TE2
............................................................ucagguGccugaaguagcgcgc.............   1     1    TE2
............................................................ucagguaccugaaguagcgcgU.............   3     1    TE2
............................................................ucagguaccugaaguaAcgcgc.............   1     1    TE2
............................................................ucagguaccugaaguagcgUgc.............   2     1    TE2
............................................................ucagguaccuAaaguagcgcgc.............   2     1    TE2
............................................................ucaggCaccugaaguagcgcgc.............   3     1    TE2
............................................................ucagguaccugaaguagcgcgG.............   6     1    TE2
............................................................ucagguaccuUaaguagcgcgc.............   1     1    TE2
............................................................ucGgguaccugaaguagcgcgc.............   1     1    TE2
............................................................ucagguaccCgaaguagcgcgc.............   1     1    TE2
............................................................ucagguaccugaaguagcgcgc.............   2121  0    TE2
............................................................ucagguaccugGaguagcgcgc.............   1     1    TE2
............................................................ucagguaccugaagCagcgcgc.............   2     1    TE2
............................................................ucagguaccugaaguagUgcgc.............   1     1    TE2
............................................................ucagguaccugaaguGgcgcgc.............   3     1    TE2
............................................................ucaggAaccugaaguagcgcgcg............   1     1    TE2
............................................................ucagguaccugaaguagcgcgcU............   16    1    TE2
............................................................ucagguaccugaaguagcgcgcA............   1     1    TE2
............................................................ucagguacAugaaguagcgcgcg............   1     1    TE2
............................................................ucagguaccugaaguagcgcgcg............   444   0    TE2
............................................................ucagguaccugCaguagcgcgcg............   1     1    TE2
............................................................ucagguaccugaaguagcgcgUg............   1     1    TE2
............................................................ucagguaccugaaguagUgcgcg............   1     1    TE2
```

```
                                                                                          aga-miR-275
                  aga-miR-275*
ugagccgucuaaugacacgcgcuaagcaggaaccgggacuugguacacauucgcuagcagucagguaccugaaguagcgcgcguuauucggcuca

............................................................ucagguaccugaaguagcgcgcC............   9    1    TE2
............................................................ucagguaccugaaguagcgcgAg............   1    1    TE2
............................................................ucagguaccugUaguagcgcgcg............   1    1    TE2
............................................................ucagguaccugaaguagcgcgcCu...........   7    1    TE2
............................................................ucaggCaccugaaguagcgcgcgu...........   1    1    TE2
............................................................ucagguGccugaaguagcgcgcgu...........   1    1    TE2
............................................................ucagguaccugaaguagcgcgcgG...........   2    1    TE2
............................................................ucagguaccugaaguagcgcgcgC...........   7    1    TE2
............................................................ucagguaccugaaguagcgcgcAu...........   2    1    TE2
............................................................ucagguaccugaaguagcgcgcgu...........   50   0    TE2
............................................................ucagguaccugaaguagcgcgcUu...........   9    1    TE2
............................................................ucagguaccugaaguagcgcgcgA...........   13   1    TE2
............................................................ucagguaccugaaguagcgcgcguu..........   2    0    TE2
............................................................ucagguaccugaaguagcgcgcUuu..........   1    1    TE2
............................................................ucagguaccugaaguagcgcgcgAu..........   8    1    TE2
............................................................ucagguaccugaaguagcgcgcguuG.........   1    1    TE2
............................................................ucagguaccugaaguagcgcgcguuU.........   6    1    TE2
............................................................ucagguaccugaaguagcgcgcUuuau........   1    1    TE2
............................................................ucagguaccugaaguagcgcgcguAau........   1    1    TE2
.............................................................cagguaccugaaguagcgcgc.............   1    0    TE2
```

miRBase precursor	: aga-mir-277
Total read count	: 4528
aga-miR-277 read count	: 4426
aga-miR-277* read count	: 84
remaining reads	: 18

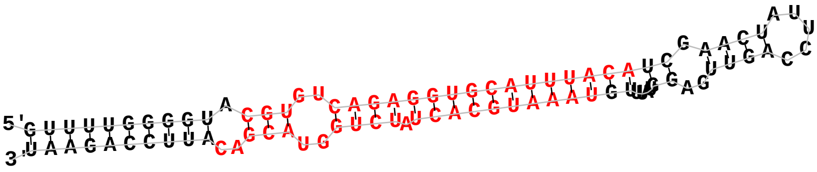

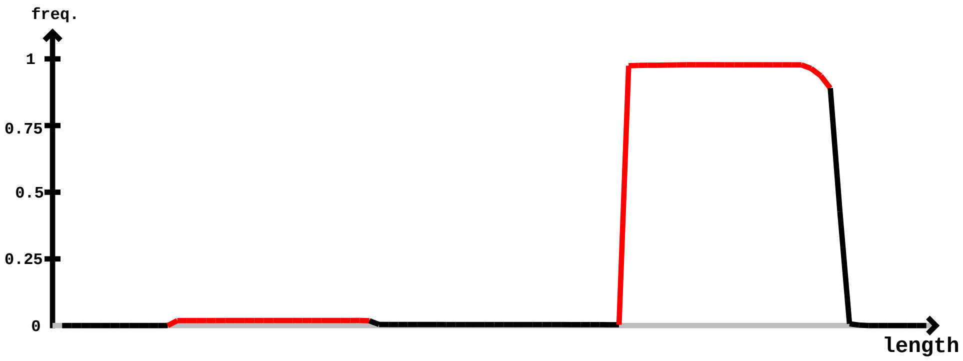


```
                                                                aga-miR-277
          aga-miR-277*
5'- guuuugggguacgugucagaggugcauuuacaucgaacuauuccaguugagguauuuguaaaugcacuaucugguacgacauuccagaau  -3'   exp
    ((((((((((.(((..((((((((((((((((((.((((.....)))).)).)....))))))))))))).)))).)))..)))..))))))))))   reads   mm   sample
    ...........cgugucagaggugcauuuaca..........................................................   4    0    OV2
    ...........cgugucagaggugcauuuacaA.........................................................   1    1    OV2
    ................................ucgaacuauuccaguugagguauuug................................   1    0    OV2
    .........................................................uaaaugcacuaucugguac..............   4    0    OV2
    .........................................................uaaaugcacuaucugguacg.............   10   0    OV2
    .........................................................uaaaugcacuauUugguacga............   1    1    OV2
    .........................................................uaaaugcacuaucugguacga............   22   0    OV2
    .........................................................uaaaugcacuaucugguacgac...........   101  0    OV2
    .........................................................uaaaugcacuauGugguacgac...........   1    1    OV2
    .........................................................uaaaugcacuaucugguacgaca..........   70   0    OV2
    .........................................................uaaaugcacuaucugguacgacU..........   24   1    OV2
    .........................................................uaaaugcaUuaucugguacgaca..........   1    1    OV2
    .........................................................uaaaugcacuaucugguacgacau.........   1    0    OV2
    .........................................................uaaaugcacuaucugguacgacUuu........   1    1    OV2
    ..........................................................aaaugcacuaucugguacgacU..........   1    1    OV2

    ...........cgugucagaggugcauuuaca..........................................................   3    0    FF2
    .........................................................uaaaugcacuaucugguac..............   1    0    FF2
    .........................................................uaaaugcacuaucugguacg.............   8    0    FF2
    .........................................................uaaaugcacuaucugguacga............   10   0    FF2
    .........................................................uaaaugcacuaucugguacgac...........   67   0    FF2
    .........................................................uaaaugcacuaucugguacgaU...........   1    1    FF2
    .........................................................uaaaCgcacuaucugguacgac...........   1    1    FF2
    .........................................................uaaaugcacuaucugguacgaca..........   52   0    FF2
    .........................................................uaaaugcacuaucugguacgacU..........   14   1    FF2
    .........................................................uaaaugcacuaucugguacgacaA.........   2    1    FF2
    .........................................................uaaaugcacuaucugguacgacau.........   1    0    FF2

    ...........cgugucagaggugcauuuac...........................................................   1    0    TE1
    ................................ucgaacuauuccaguugagguauuuguaaaugcacu......................   1    0    TE1
    .........................................................uaaaugcacuaucugguac..............   2    0    TE1
    .........................................................uaaaugcacuaucuggCacga............   1    1    TE1
    .........................................................uaaaugcacuaucugguacga............   9    0    TE1
    .........................................................uaaaugcacuaucugguacgac...........   99   0    TE1
```

```
                                                                aga-miR-277
           aga-miR-277*
guuuuggggua cgugucagaggugcauuuaca ucgaacuauuccaguugagguauuug uaaaugcacuaucugguacgac auuccagaau

..........................................................uaaaugcacAaucugguacgac..........    1     1    TE1
..........................................................uaaaugcacuaucugguacgacU.........    3     1    TE1
..........................................................uaaaugcacuaucuAguacgaca.........    1     1    TE1
..........................................................uaaaugcacuaucugguacgaca.........    88    0    TE1
..........................................................uaaaugcacuaucugguacgacC.........    4     1    TE1

.........acgugucagaggugcauuuaca...........................................................    1     0    MF2
...........cgugucagaggugcauuuac...........................................................    1     0    MF2
...........cgugucagaggGgcauuuaca..........................................................    1     1    MF2
...........cgugucagaggugcauuuaca..........................................................    29    0    MF2
...........cgugucagaggugcauuuacaucgaacu...................................................    1     0    MF2
................cagaggugcauuuacaucgaacuauuccaguugagg......................................    1     0    MF2
................................ucgaacuauuccaguugagguauuug................................    4     0    MF2
..........................................................uaaaugcacuaucugguac.............    27    0    MF2
..........................................................uaaaugcacuaucuggAacg............    1     1    MF2
..........................................................uaaaugcGcuaucugguacg............    1     1    MF2
..........................................................uaaaugcacuaucCgguacg............    1     1    MF2
..........................................................uaaaugcacuaucugguacg............    37    0    MF2
..........................................................uaaaugcacuaucugguacga...........    47    0    MF2
..........................................................uaaaugcacuaucuggGacgac..........    2     1    MF2
..........................................................uaaaugcacuaucugguUcgac..........    1     1    MF2
..........................................................uaaaugcacuaucugguacgac..........    492   0    MF2
..........................................................uaaaugcacuCucugguacgac..........    1     1    MF2
..........................................................uaaaugcacuaucugguaAgac..........    1     1    MF2
..........................................................uaaaugcacuauUugguacgac..........    1     1    MF2
..........................................................uaaaugcacuaucuUguacgac..........    1     1    MF2
..........................................................uaaaugcacuaucugguacAac..........    1     1    MF2
..........................................................uaaaugcacuaucugguaGgac..........    1     1    MF2
..........................................................uaaaugcacuaucugguacgaU..........    5     1    MF2
..........................................................uaaaugcacuaucugguacgUca.........    1     1    MF2
..........................................................uaaaugcacuaAcugguacgaca.........    1     1    MF2
..........................................................uaaaugcacuaucugguacgaUa.........    1     1    MF2
..........................................................uaaaugcacuaucugguacgaca.........    348   0    MF2
..........................................................uaaaugcacuaucugguacgacU.........    148   1    MF2
..........................................................Aaaaugcacuaucugguacgaca.........    1     1    MF2
..........................................................uaaaugcacuaucugguacgaAa.........    1     1    MF2
..........................................................uaaaugcacuaucugguacgacC.........    5     1    MF2
..........................................................uaaaugGacuaucugguacgaca.........    1     1    MF2
..........................................................uaaGugcacuaucugguacgaca.........    1     1    MF2
..........................................................uaaaugcacuaucugguacgacUu........    1     1    MF2
..........................................................uaaaugcacuaucugguacgacaA........    5     1    MF2
..........................................................uaaaugcacuaucugguacgacau........    8     0    MF2
..........................................................uaaaugcacuaucugguacgacaC........    1     1    MF2
..........................................................uaaaugcacuaucugguacgacauA.......    1     1    MF2
..........................................................uaaaugcacuaucugguacgacaCu.......    4     1    MF2
..........................................................uaaaugcacuaucugguacgacauu.......    1     0    MF2
...........................................................aaaugcacuaucugguacg............    1     0    MF2
...........................................................aaaugcacuaucugguacgac..........    2     0    MF2
...........................................................aaaugcacuaucugguacgacU.........    1     1    MF2

...........cgugucagaggugcauuuaca..........................................................    6     0    FW2
..............................caucgaacuauuccaguugagguauuuguaaaugcacuaucuggua..............    1     0    FW2
................................ucgaacuauuccaguugagguauuug................................    1     0    FW2
..........................................................uaaaugcacuaucugguacg............    5     0    FW2
..........................................................uaaaugcacuaucugguacU............    1     1    FW2
..........................................................uaaaugcacuaucugguacga...........    13    0    FW2
..........................................................uaaauUcacuaucugguacgac..........    1     1    FW2
..........................................................uaaaugcaAuaucugguacgac..........    1     1    FW2
..........................................................uaaaugcacuaucugguacgac..........    187   0    FW2
..........................................................uaaaugcacuaucugguacgacU.........    3     1    FW2
..........................................................uaaaugcacuaucugguacgaca.........    133   0    FW2
..........................................................uaaaugcacuaucuggCacgaca.........    1     1    FW2
..........................................................uaaaugcacuaucugguacgacaA........    1     1    FW2
..........................................................uaaaugcacuaucugguacgacau........    1     0    FW2
............................................................aaugcacuaucugguacga...........    1     0    FW2
..............................................................ugcacuaucugguacgac..........    1     0    FW2
...............................................................gcacuaucugguacgac..........    1     0    FW2
................................................................cacuaucugguacgacU.........    1     1    FW2
................................................................cacuaucugguacgaca.........    1     0    FW2
```

```
                                                          aga-miR-277
           aga-miR-277*
guuuuggggguacgugucagaggugcauuuacaucgaacuauuccaguugagguauuuguaaaugcacuaucugguacgacauuccagaau

...........cgugucagaggugcauuuaca..........................................................  4    0    FF1
................................ucgaacuauuccaguugagguauuug................................  2    0    FF1
..........................................................uaaaugcacuaucugguac.............  1    0    FF1
..........................................................uaaaugcacuaucugguacg............  5    0    FF1
..........................................................uaaaugcacuaucCgguacga...........  1    1    FF1
..........................................................uaaaugcacuaucugguacga...........  7    0    FF1
..........................................................uaaaugcacuaucugguacgac..........  39   0    FF1
..........................................................uaaaugcacuaGcugguacgac..........  1    1    FF1
..........................................................uaaaugcacuaucugguacgacC.........  2    1    FF1
..........................................................uaaaugcacuaucugguacgaca.........  65   0    FF1
..........................................................uaaaugcacuaucugguacgacU.........  5    1    FF1

...........cgugucagaggugcauuuaca..........................................................  1    0    OV1
..........................................................uaaaugcacuaucugguac.............  5    0    OV1
..........................................................uaaaugcacuaucugguacg............  13   0    OV1
..........................................................uaaaugcacuaucugguacga...........  29   0    OV1
..........................................................uaaaugcacuaucugguacgaU..........  1    1    OV1
..........................................................uaaaugcacuaucugguaUgac..........  1    1    OV1
..........................................................uaaaugcacuaucugguacgac..........  134  0    OV1
..........................................................uaaaugcacuaucugAuacgac..........  2    1    OV1
..........................................................uUaaugcacuaucugguacgaca.........  1    1    OV1
..........................................................uaaaugcacuaucugguacgacU.........  38   1    OV1
..........................................................uaaaugcacuaucugguacgaca.........  118  0    OV1
...........................................................aaaugcacuaucugguacgac..........  1    0    OV1

...........cgugucagaggugcauuuac...........................................................  1    0    MF1
...........cgugucagaggugcauuuaca..........................................................  6    0    MF1
................................ucgaacuauuccaguugagguauuug................................  1    0    MF1
..........................................................uaaaugcacuaucugguac.............  10   0    MF1
..........................................................uaaaugcacuaucugguacg............  7    0    MF1
..........................................................uaaaugcacuaucugguacgU...........  1    1    MF1
..........................................................uaaaugcacuaucugguacga...........  19   0    MF1
..........................................................uaaauAcacuaucugguacgac..........  1    1    MF1
..........................................................uaaaugcacuaucugguacgac..........  106  0    MF1
..........................................................uaaGugcacuaucugguacgac..........  1    1    MF1
..........................................................uaaaugcacuaucugguacgaU..........  1    1    MF1
..........................................................uaaaugcacuaucugguacgaca.........  35   0    MF1
..........................................................uaaaugcacuaucugguacgacU.........  23   1    MF1

...........cgugucagaggugcauuuac...........................................................  2    0    BF2
...........cgugucagaggugcauuuaca..........................................................  1    0    BF2
................................ucgaacuauuccaguugagguauu..................................  1    0    BF2
................................ucgaacuauuccaguugagguauuugu...............................  1    0    BF2
..........................................................uaaaugcacuaucugguac.............  1    0    BF2
..........................................................uaaaugcacuaucugguacg............  5    0    BF2
..........................................................uaaaugcacuaucugguacga...........  8    0    BF2
..........................................................uaaaugcacuaucugguacgac..........  150  0    BF2
..........................................................uaaaugAacuaucugguacgaca.........  1    1    BF2
..........................................................uaaaugcacuaucugguacgacU.........  15   1    BF2
..........................................................uaaaugcacuaucugguacgaca.........  140  0    BF2
..........................................................uaaaugcacuaucugguacgacauu.......  1    0    BF2

...........cgugucagaggugcauuuaca..........................................................  4    0    BF1
................................ucgaacuauuccaguugagguauuug................................  2    0    BF1
..........................................................uaaaugcacuaucugguac.............  1    0    BF1
..........................................................uaaaugcacuaucugguacg............  5    0    BF1
..........................................................uaaaugcacuaucugguacga...........  7    0    BF1
..........................................................uaaaugcacuaucCgguacga...........  1    1    BF1
..........................................................uaaaugcacuaucugguacgac..........  39   0    BF1
..........................................................uaaaugcacuaGcugguacgac..........  1    1    BF1
..........................................................uaaaugcacuaucugguacgaca.........  65   0    BF1
..........................................................uaaaugcacuaucugguacgacU.........  5    1    BF1
..........................................................uaaaugcacuaucugguacgacC.........  2    1    BF1

..........................................................uaaaugcacuaucugguac.............  1    0    FW1
..........................................................uaaaugcacuaucugguacg............  4    0    FW1
..........................................................uaaaugcacuaucugguacga...........  9    0    FW1
..........................................................uaaaugcacuaucugguacgac..........  73   0    FW1
```

```
                                                          aga-miR-277
           aga-miR-277*
guuuuggguacgugucagaggugcauuuacaucgaacuauuccaguugagguauuuguaaaugcacuaucugguacgacauuccagaau

..........................................................uaUaugcacuaucugguacgac..........    1     1     FW1
..........................................................uaaaugcacuaucugguacgacU.........    3     1     FW1
..........................................................uaaaugcacuaucugguacgaca.........    57    0     FW1
...............................................................gcacuaucugguacgaca.........    1     0     FW1

...........cgugucagaggugcauuuaca..........................................................    6     0     MW1
..........................................................uaaaugcacuaucugguac.............    1     0     MW1
..........................................................uaaaugcacuaucugguacg............    13    0     MW1
..........................................................uaaaugcacuaucugguacga...........    7     0     MW1
..........................................................uaaUugcacuaucugguacgac..........    1     1     MW1
..........................................................uaaauAcacuaucugguacgac..........    1     1     MW1
..........................................................uaaaugcacuaucugguacgac..........    233   0     MW1
..........................................................uaaaugcacuaucugguacgacC.........    4     1     MW1
..........................................................uaaaugcacuaucugguacgacG.........    1     1     MW1
..........................................................uaaaugcacuaucugguacgaca.........    107   0     MW1
..........................................................uaaaugcacuaucugguacgacU.........    8     1     MW1

...........cgugucagaggugcauuuaca..........................................................    3     0     MW2
..........................................................uaaaugcacuaucugguac.............    4     0     MW2
..........................................................uaaaugcacuaucugguacg............    1     0     MW2
..........................................................uaaaugcacuaucugguacgac..........    51    0     MW2
..........................................................uaaaugcacuaucGgguacgac..........    1     1     MW2
..........................................................uaaaugcacuaCcugguacgaca.........    1     1     MW2
..........................................................uaaaugcacuaucugguacgacU.........    5     1     MW2
..........................................................uaaaugcacuaucugguacgacC.........    2     1     MW2
..........................................................uaaaugcacuaucugguacgaca.........    42    0     MW2
............................................................aaugcacuaucugguacgaca.........    1     0     MW2
..............................................................ugcacuaucugguacgaca.........    1     0     MW2
................................................................cacuaucugguacgaca.........    1     0     MW2

...........cgugucagaggugcauuuac...........................................................    1     0     TE2
...........cgugucagaggugcauuuaca..........................................................    7     0     TE2
............gugucagaggugcauuuaca..........................................................    1     0     TE2
................................ucgaacuauuccaguugagguauu..................................    1     0     TE2
..........................................................uaaaugcacuaucugguac.............    3     0     TE2
..........................................................uaaaugcacuaucugguacg............    5     0     TE2
..........................................................uaaaugcacuaucugguacga...........    18    0     TE2
..........................................................uaaaugcacuaucCgguacgac..........    2     1     TE2
..........................................................uaaaugcacuaucAgguacgac..........    1     1     TE2
..........................................................uaaaugcacuaucugguacgac..........    278   0     TE2
..........................................................uaaaugcacCaucugguacgac..........    2     1     TE2
..........................................................uaaaugcacuaucugguaUgac..........    1     1     TE2
..........................................................uaaaugcacuaucugUuacgac..........    1     1     TE2
..........................................................uaaaugcacuaAcugguacgac..........    1     1     TE2
..........................................................uaaaugcacuaucugguacgacC.........    3     1     TE2
..........................................................uaaaugcacuaucugguacgacU.........    25    1     TE2
..........................................................uaaaugcacuaucugguacgaca.........    218   0     TE2
..........................................................uaaaugcacuaucugguacgaUa.........    1     1     TE2
..........................................................uaaaCgcacuaucugguacgaca.........    1     1     TE2
..........................................................uaaaugcacuaucugguacgacaC........    1     1     TE2
...........................................................aaaugcacuaucugguacgac..........    1     0     TE2
............................................................aaugcacuaucugguacgaca.........    1     0     TE2
..............................................................ugcacuaucugguacgaca.........    1     0     TE2
```

miRBase precursor : aga-mir-278
Total read count : 4676
aga-miR-278 read count : 3676
aga-miR-278* read count : 996
remaining reads : 4

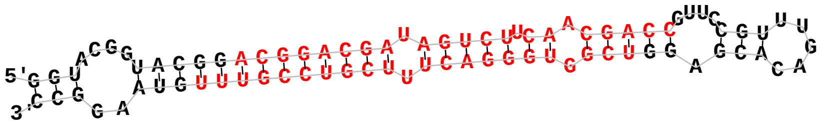

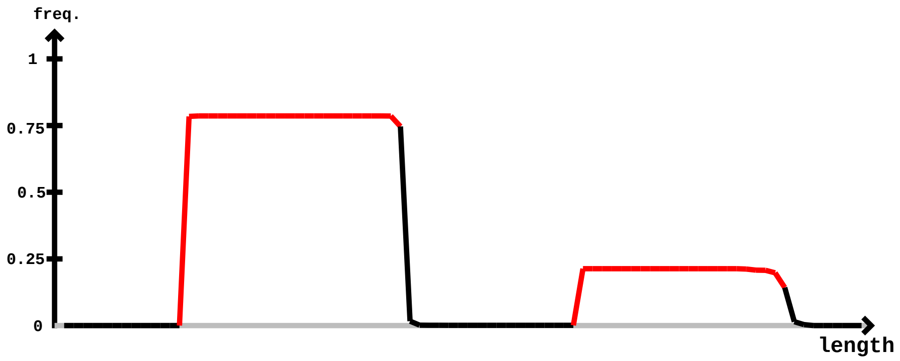


```
                                                     aga-miR-278*
            aga-miR-278
5'- gguacgguacggacggacgauagucuucaacgaccguuccguuugacacgaggucggugggacuuucguccguuuguaaggcc   -3'    exp
    (((....((((((((((((.(((((.((.(((((....(((.....))).))))).))))))).))))))))))))..)))    reads    mm    sample
    ............acggacgauagucuucaacgacU................................................    1        1     OV2
    ............acggacgauagucuucaacgacc................................................    7        0     OV2
    .....................................................ucggugggacuuucguccguu.........    1        0     OV2
    .....................................................ucggugggacuuucguccguuu........    1        0     OV2

    ............acggacgauagucuucaacgac.................................................    14       0     TE1
    ............acggacgauagucuucaacgacU................................................    2        1     TE1
    ............acggacgauagucuucaacgacc................................................    90       0     TE1
    ............acggacgauagucuucaacgaccU...............................................    3        1     TE1
    .....................................................ucggugggacuuucguccguu.........    3        0     TE1
    .....................................................ucggugggacuuucguccguuu........    25       0     TE1
    .....................................................ucggugggacuuucguccguuuU.......    3        1     TE1
    .....................................................ucggugggacuuucguccguuuUu......    1        1     TE1

    ............acggacgauagucuucaacgac.................................................    3        0     FF2
    ............acggacgaCagucuucaacgacc................................................    1        1     FF2
    ............acggacgauagucuCcaacgacc................................................    1        1     FF2
    ............acggacgauagucuucaacgacc................................................    137      0     FF2
    ............acggacgauagucuucaacgacA................................................    2        1     FF2
    ............acggacgauagucuucaacgacU................................................    4        1     FF2
    ............acggacgauagucuucaacgaccU...............................................    2        1     FF2
    .....................................................ucggugggacuuucgucc............    3        0     FF2
    .....................................................ucggugggacuuucguccgu..........    1        0     FF2
    .....................................................ucggugggacuuucguccguu.........    8        0     FF2
    .....................................................ucggugggacuuucguccguuA........    1        1     FF2
    .....................................................ucggugggacuuucUuccguuu........    1        1     FF2
    .....................................................ucggugggacuuucguccguuu........    19       0     FF2

    ............acggacgauagucuucaacgac.................................................    70       0     MF2
    ............acggacgauagCcuucaacgac.................................................    1        1     MF2
    ............Gcggacgauagucuucaacgac.................................................    1        1     MF2
    ............Gcggacgauagucuucaacgacc................................................    1        1     MF2
    ............acggacgauCgucuucaacgacc................................................    1        1     MF2
    ............acggacgauagucuucaacgacU................................................    29       1     MF2
```

```
                                                 aga-miR-278*
            aga-miR-278
gguacgguacggacggacgauagucuucaacgaccguuccguuugacacgaggucggugggacuuucguccguuuguaaggcc

............acggacgauagucuucaacgaAc................................................    2      1    MF2
............acggacgauagucuCcaacgacc................................................    1      1    MF2
............acggacgaCagucuucaacgacc................................................    1      1    MF2
............acggacgauagucuucaacgacc................................................    1535   0    MF2
............acggacgauagucuucaacgacA................................................    10     1    MF2
............acggacgauaCucuucaacgacc................................................    1      1    MF2
............acAgacgauagucuucaacgacc................................................    1      1    MF2
............acggacAauagucuucaacgacc................................................    1      1    MF2
............acggacgauagucuuUaacgacc................................................    2      1    MF2
............acggacgauagucGucaacgacc................................................    1      1    MF2
............acggacgauagAcuucaacgacc................................................    2      1    MF2
............acggacgauagucuucaacgaGc................................................    2      1    MF2
............acUgacgauagucuucaacgacc................................................    1      1    MF2
............acggacgauaUucuucaacgacc................................................    1      1    MF2
............acggacgauagucuucaacgaccg...............................................    3      0    MF2
............acggacgauagucuucaacgaccU...............................................    43     1    MF2
............acggacgauagucuucaacgaccAu..............................................    1      1    MF2
.............cggacgauagucuucaacgac.................................................    1      0    MF2
.............cggacgauagucuucaacgacc................................................    2      0    MF2
.............cggacgauagucuucaacgaccU...............................................    1      1    MF2
...................................guuccguuugacacgagg..............................    1      0    MF2
.....................................................ucggugggacuuucgucc............    5      0    MF2
.....................................................ucggugggacuuucguccg...........    1      0    MF2
.....................................................ucggugggacuuucguccgu..........    13     0    MF2
.....................................................ucggugggacuuucguccgCu.........    1      1    MF2
.....................................................ucggugggacuuucgGccguu.........    1      1    MF2
.....................................................ucggugggacuuucguccguC.........    1      1    MF2
.....................................................ucggugggacuuucguccguu.........    64     0    MF2
.....................................................ucAgugggacuuucguccguu.........    1      1    MF2
.....................................................ucggugggacuuucguccguuu........    175    0    MF2
.....................................................Acggugggacuuucguccguuu........    1      1    MF2
.....................................................ucAgugggacuuucguccguuu........    1      1    MF2
.....................................................ucggugggacuuucAuccguuu........    1      1    MF2
.....................................................ucggugggacuuucguccguuA........    1      1    MF2
.....................................................ucggugggacuuucguccguuuA.......    1      1    MF2
.....................................................ucggugggacuuucguccguuuC.......    4      1    MF2
.....................................................ucggugggacuuucguccguuuU.......    27     1    MF2
.....................................................ucggugggacuuucguccguuugA......    1      1    MF2
.....................................................ucggugggacuuucguccguuuCu......    1      1    MF2
.....................................................ucggugggacuuucguccguuuUu......    13     1    MF2

............acggacgauagucuucaacgac.................................................    15     0    FW2
............acggacgauagucuuUaacgacc................................................    1      1    FW2
............acggacgauaCucuucaacgacc................................................    2      1    FW2
............acggacgauagucuucaacgacU................................................    1      1    FW2
............acggacgauagucuucaacgacc................................................    364    0    FW2
............acggacgauagucuucaacgacA................................................    1      1    FW2
............acggacgauagucuucaacgaccU...............................................    3      1    FW2
............acggacgauagucuucaacgaccUu..............................................    1      1    FW2
............acggacgauagucuucaacgaccguuc............................................    1      0    FW2
...................................guuccguuugacacgaggucggugggacuuucguccguuu........    1      0    FW2
.....................................................ucggugggacuuucguccgu..........    2      0    FW2
.....................................................ucggugggacuuucguccguu.........    39     0    FW2
.....................................................ucggugggacuuucguccguuu........    103    0    FW2
.....................................................ucAgugggacuuucguccguuu........    1      1    FW2
.....................................................ucggugggacuuucguccguuuC.......    1      1    FW2
.....................................................ucggugggacuuucguccguuuU.......    1      1    FW2
.....................................................ucggugggacuuucguccguuuUu......    1      1    FW2

............acggacgauagucuucaacgacc................................................    8      0    OV1
............acggacgauagGcuucaacgacc................................................    1      1    OV1
.....................................................ucggugggacuuucguccguuu........    1      0    OV1

............acggacgauagucuucaacgacc................................................    3      0    FF1

............acggacgauagucuucaacgac.................................................    15     0    MF1
............acggacgauaguUuucaacgacc................................................    1      1    MF1
............acggacgauagucuucaacgacA................................................    2      1    MF1
............acggacgaCagucuucaacgacc................................................    1      1    MF1
```

```
                                                     aga-miR-278*
            aga-miR-278
gguacgguacggacggacgauagucuucaacgaccguuccguuugacacgaggucggugggacuuucguccguuuguaaggcc

............acggacgauagGcuucaacgacc................................................ 1    1    MF1
............acggacgauagucuucaacgacU................................................ 4    1    MF1
............acggaUgauagucuucaacgacc................................................ 1    1    MF1
............acggacgauagucuucaacgaGc................................................ 2    1    MF1
............acggacgauagucuucaacgacc................................................ 235  0    MF1
............acggacgauagucuucaacgaccA............................................... 1    1    MF1
............acggacgauagucuucaacgaccU............................................... 6    1    MF1
.....................................................ucggugggacuuucguc............. 1    0    MF1
.....................................................ucggugggacuuucgucU............ 2    1    MF1
.....................................................ucggugggacuuucgucA............ 1    1    MF1
.....................................................ucggugggacuuucgucc............ 2    0    MF1
.....................................................ucggugggacuuucguccg........... 1    0    MF1
.....................................................ucggugggacuuucguccgG.......... 1    1    MF1
.....................................................ucggugggacuuucguccgu.......... 9    0    MF1
.....................................................ucggugggacuuucguccguu......... 33   0    MF1
.....................................................ucggugggacuuucguccguuu........ 42   0    MF1
.....................................................ucggugggacuuucguccguuA........ 1    1    MF1
.....................................................ucggugggacuuucguccguuuU....... 2    1    MF1
.....................................................ucggugggacuuucguccguuuC....... 1    1    MF1

............acggacgauagucuucaacgacc................................................ 5    0    BF2
.....................................................ucggugggacuuucguccgu.......... 1    0    BF2

............acggacgauagucuucaacgacc................................................ 3    0    BF1

............acggacgauagucuucaacgac................................................. 22   0    MW1
............acggacgauagucuucaacgacU................................................ 1    1    MW1
............acggacgauagucuucaacgacc................................................ 397  0    MW1
............acggacgauagucuucaacgacA................................................ 2    1    MW1
............acggacgauagucuuAaacgacc................................................ 1    1    MW1
............acggacgauagGcuucaacgacc................................................ 1    1    MW1
............acggacgauagucuucaacgaccg............................................... 1    0    MW1
............acggacgauagucuucaacgaccU............................................... 4    1    MW1
.............cggacgauagucuucaacgacc................................................ 1    0    MW1
...................................guuccguuugacacgagg.............................. 2    0    MW1
.....................................................ucggugggacuuucguccgu.......... 6    0    MW1
.....................................................ucggugggacuuucguccguu......... 50   0    MW1
.....................................................ucgguUggacuuucguccguuu........ 1    1    MW1
.....................................................ucggugggacuuucguccguuu........ 96   0    MW1
.....................................................ucggugggacuuucguccguuuA....... 1    1    MW1
.....................................................ucggugggacuuucguccguuuU....... 2    1    MW1
.....................................................ucggugggacuuucguccguuuC....... 1    1    MW1

............acggacgauagucuucaacgac................................................. 13   0    FW1
............acggacgauagucuucaacgacc................................................ 175  0    FW1
............acggacgauagucuucaaAgacc................................................ 1    1    FW1
............acggGcgauagucuucaacgacc................................................ 1    1    FW1
............acggacgauagucGucaacgacc................................................ 1    1    FW1
............acggacgauagucuucaacgaccg............................................... 1    0    FW1
.............cggacgauagucuucaacgacc................................................ 1    0    FW1
.....................................................ucggugggacuuucguc............. 1    0    FW1
.....................................................ucggugggacuuucguccgu.......... 6    0    FW1
.....................................................ucggugggacuuucguccguu......... 18   0    FW1
.....................................................ucggugggacuuucguccguuu........ 46   0    FW1
.....................................................ucggugggacuuucguccguuuU....... 2    1    FW1
.....................................................ucggugggacuuucguccguuuC....... 1    1    FW1

............acgAacgauagucuucaacgac................................................. 1    1    MW2
............acggacgauagucuucaacgac................................................. 7    0    MW2
............acggacgauagucuucaacgacc................................................ 129  0    MW2
............acggacgauagucuucaacgacU................................................ 1    1    MW2
............aUggacgauagucuucaacgacc................................................ 1    1    MW2
............acggacgauGgucuucaacgacc................................................ 1    1    MW2
............acggacgauagucuucaacgaccU............................................... 1    1    MW2
.............cggacgauagucuucaacgac................................................. 1    0    MW2
.............cggacgauagucuucaacgacc................................................ 1    0    MW2
.....................................................ucggugggacuuucguc............. 1    0    MW2
.....................................................ucggugggacuuucgucc............ 2    0    MW2
.....................................................ucggugggacuuucguccguu......... 10   0    MW2
```

```
                                                     aga-miR-278*
            aga-miR-278
gguacgguacggacggacgauagucuucaacgaccguuccguuugacacgaggucggugggacuuucguccguuuguaaggcc

.....................................................ucggugggacuuucguccguuu........    33    0    MW2
.....................................................ucggugggacuuCcguccguuu........    1     1    MW2
.....................................................ucggugggacuuucguccguuuU.......    1     1    MW2
.....................................................ucggugggacuuucguccguuuC.......    1     1    MW2

............acggacgauagucuucaacga..................................................    2     0    TE2
............acggacgauagucuucaacgac.................................................    19    0    TE2
............acggacgauagucuucaacgacU................................................    5     1    TE2
............acggacgaAagucuucaacgacc................................................    1     1    TE2
............acggacgauagucuucaacgacc................................................    218   0    TE2
............acggacgauagucuucaacgacA................................................    2     1    TE2
............acggacgauagAcuucaacgacc................................................    1     1    TE2
............acggacgauagucuucaacgaccU...............................................    1     1    TE2
.............cggacgauagucuucaacgacc................................................    1     0    TE2
.....................................................ucggugggacuuucguc.............    3     0    TE2
.....................................................ucggugggacuuucgucc............    4     0    TE2
.....................................................ucggugggacuuucguccg...........    1     0    TE2
.....................................................ucggugggacuuucguccgu..........    5     0    TE2
.....................................................ucggugggacuuucguccguu.........    28    0    TE2
.....................................................ucggugggacuuucguccguuu........    47    0    TE2
.....................................................ucggugggacuuucguccguuuU.......    2     1    TE2
```

miRBase precursor : aga-mir-279
Total read count : 14475
aga-miR-279 read count : 14361
aga-miR-279* read count : 114
remaining reads : 0

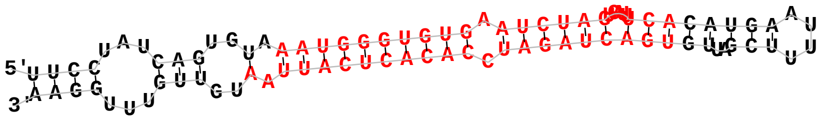

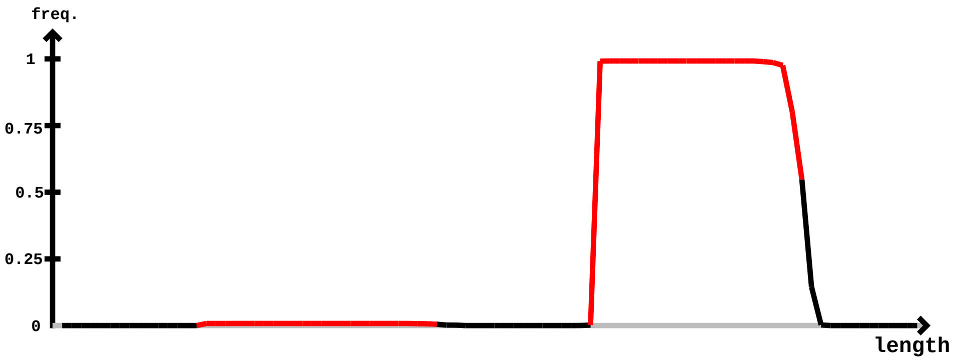


```
                           aga-miR-279*
                                                                   aga-miR-279
5'- uuccuaucaguguaaaugggugugaaucuagugauuucacaugaauuuucgauugugacuagauccacacucauuaauguuguuuggaa   -3'    exp
    .(((...(((..(.((((((((((.((((((.....((((((((....)))..)))))))))))).)))))))))).)..)))...))).    reads     mm        sample
    ..............aaugggugugaaucuagugauuuc...................................................    2         0         FF2
    ..............aaugggugugaaucuagugauuuca..................................................    1         0         FF2
    ......................................................Cugacuagauccacacucauua.............    2         1         FF2
    .......................................................ugacuagauccacacuU.................    1         1         FF2
    .......................................................ugacuagauccacacucau...............    4         0         FF2
    .......................................................ugacuagauccacacucaCu..............    1         1         FF2
    .......................................................ugacuagauccacacucauu..............    40        0         FF2
    .......................................................ugacuagauccacacucauua.............    41        0         FF2
    .......................................................ugacuagauccacacucauuaa............    78        0         FF2
    .......................................................ugacuagauccacacucauuaU............    2         1         FF2
    .......................................................ugacuagauccacacuUauuaa............    1         1         FF2
    .......................................................ugacuagauccacacucauuaau...........    4         0         FF2
    .......................................................ugacuagauccacacucauuaaG...........    1         1         FF2
    .......................................................ugacuagauccacacucauuaaA...........    24        1         FF2

    ..............aaugggugugaaucuagugauuuc...................................................    3         0         TE1
    .....................................................uCugacuagauccacacucauuaa............    1         1         TE1
    ......................................................Cugacuagauccacacucauuaa............    2         1         TE1
    .......................................................ugacuagauccacacuc.................    4         0         TE1
    .......................................................ugacuagauccacacuca................    4         0         TE1
    .......................................................ugacuagauccacacucau...............    11        0         TE1
    .......................................................ugacuagauccacacucauA..............    1         1         TE1
    .......................................................ugacuagauccacacucauu..............    146       0         TE1
    .......................................................ugUcuagauccacacucauu..............    1         1         TE1
    .......................................................ugacuagauccacacucauua.............    233       0         TE1
    .......................................................ugacuagauccCcacucauua.............    1         1         TE1
    .......................................................ugacuagauccacacucauuU.............    1         1         TE1
    .......................................................ugacuagauccacacucauuaU............    4         1         TE1
    .......................................................ugacuagauccacacuAauuaa............    1         1         TE1
    .......................................................ugacuagauccacacucauuaa............    201       0         TE1
    .......................................................ugacuagauccacacucauuaaG...........    1         1         TE1
    .......................................................ugacuagauccacacucauuaaA...........    52        1         TE1
    .......................................................ugacuagauccacacucauuaau...........    6         0         TE1
    ........................................................gacuagauccacacucauua.............    1         0         TE1
```

aga-miR-279*

aga-miR-279

```
uuccuaucaguguaaaugggugugaaucuagugauuucacaugaauuuucgauugugacuagauccacacucauuaauguuguuuggaa

..............aaugggugugaaucuagugauuu....................................................  1    0  OV2
..............aaugggugugaaucuagugauuuc...................................................  1    0  OV2
..............aaugggugugaaucuagugauuuca..................................................  6    0  OV2
..............Gaugggugugaaucuagugauuuca..................................................  1    1  OV2
.....................................................uCugacuagauccacacucauua.............  1    1  OV2
......................................................Cugacuagauccacacucauu..............  1    1  OV2
......................................................Cugacuagauccacacucauua.............  2    1  OV2
......................................................Cugacuagauccacacucauuaa............  2    1  OV2
.......................................................ugacuagauccacacuc.................  3    0  OV2
.......................................................ugacuagauccacacuca................  6    0  OV2
.......................................................ugacuagauccacacucau...............  28   0  OV2
.......................................................ugacuagauccacacucauu..............  391  0  OV2
.......................................................ugacuUgauccacacucauu..............  1    1  OV2
.......................................................ugacuagauccacUcucauu..............  1    1  OV2
.......................................................ugGcuagauccacacucauua.............  1    1  OV2
.......................................................ugacuaAauccacacucauua.............  1    1  OV2
.......................................................Agacuagauccacacucauua.............  1    1  OV2
.......................................................ugacuagaucAacacucauua.............  1    1  OV2
.......................................................ugacuagaucUacacucauua.............  1    1  OV2
.......................................................ugacuagauccCcacucauua.............  1    1  OV2
.......................................................ugacuagauccacacucauuU.............  4    1  OV2
.......................................................ugacuagauccacacucauua.............  551  0  OV2
.......................................................ugacuagauccacacucaCuaa............  1    1  OV2
.......................................................uAacuagauccacacucauuaa............  1    1  OV2
.......................................................ugacuaAauccacacucauuaa............  1    1  OV2
.......................................................ugacuagauccacacucauuaC............  9    1  OV2
.......................................................ugacuagauccacacucauuaU............  10   1  OV2
.......................................................ugacuagauccacacucauuaG............  1    1  OV2
.......................................................ugacuagauccacacucauuGa............  1    1  OV2
.......................................................ugacuagauccacacucauuaa............  594  0  OV2
.......................................................ugaUuagauccacacucauuaa............  1    1  OV2
.......................................................ugacuagauccacacucauuaaG...........  5    1  OV2
.......................................................Agacuagauccacacucauuaau...........  1    1  OV2
.......................................................ugacuagauccacacucauuaaA...........  223  1  OV2
.......................................................ugacuagauccacacucauuaaC...........  8    1  OV2
.......................................................ugacuagauccacacucauuaau...........  54   0  OV2
.......................................................ugacuagauccacacucauuaauA..........  3    1  OV2

..............aaugggugugaaucuagugauuuc...................................................  4    0  MF2
..............aaugggugugaaucuagugauuuca..................................................  7    0  MF2
..............aaugggugugaaucuagugauuucacU................................................  1    1  MF2
..............aaugggugugaaucuagugauuucaca................................................  9    0  MF2
..............aaugggugugaaucuagugauuucaUa................................................  1    1  MF2
.....................................................uCugacuagauccacacucauua.............  2    1  MF2
......................................................Cugacuagauccacacucauu..............  1    1  MF2
.......................................................ugacuagauccacacuc.................  1    0  MF2
.......................................................ugacuagauccacacucau...............  6    0  MF2
.......................................................ugacuagauccGcacucauu..............  1    1  MF2
.......................................................ugacuagauccacacucauu..............  165  0  MF2
.......................................................ugacuagauccacacucauua.............  258  0  MF2
.......................................................ugacuagauccacacucauuU.............  3    1  MF2
.......................................................ugacuagauccacacucauuG.............  1    1  MF2
.......................................................ugacuagauccacacucauuaC............  1    1  MF2
.......................................................ugacuagauccacacuUauuaa............  1    1  MF2
.......................................................ugacuagauccacacucauuaa............  439  0  MF2
.......................................................ugacuagauccacacucauuaG............  2    1  MF2
.......................................................ugacuagauccacacucauuaU............  6    1  MF2
.......................................................ugacuagGuccacacucauuaa............  1    1  MF2
.......................................................ugacuGgauccacacucauuaa............  1    1  MF2
.......................................................ugacuagauccacacucauuaau...........  24   0  MF2
.......................................................ugacuagauccacacucauuaaG...........  3    1  MF2
.......................................................ugacuagauccacacucauuaaC...........  15   1  MF2
.......................................................ugacuagauccacacucauuaaA...........  128  1  MF2
.......................................................ugacuagauccacacucauuaauA..........  1    1  MF2

..............aaugggugugaaucuagugauuuca..................................................  1    0  FW2
..............aaugggugugaaucuagugauuucac.................................................  1    0  FW2
......................................................Cugacuagauccacacucauuaa............  1    1  FW2
```

```
              aga-miR-279*
                                         aga-miR-279
uuccuaucaguguaaaugggugugaaucuagugauuucacaugaauuuucgauugugacuagauccacacucauuaauguuguuuggaa

.......................................................ugacuagauccacacucauu..............  45    0    FW2
.......................................................ugacuagauccacacucauua.............  109   0    FW2
.......................................................ugacuagUuccacacucauua.............  1     1    FW2
.......................................................ugacuagauccacacucauuaa............  291   0    FW2
.......................................................ugacuagauccacacucGuuaa............  1     1    FW2
.......................................................ugacuagauGcacacucauuaa............  1     1    FW2
.......................................................Cgacuagauccacacucauuaa............  1     1    FW2
.......................................................ugacuagauccaUacucauuaa............  1     1    FW2
.......................................................ugacuagauccaAacucauuaa............  1     1    FW2
.......................................................uUacuagauccacacucauuaa............  1     1    FW2
.......................................................ugacuagauccacacucauuaaC...........  4     1    FW2
.......................................................ugacuagauccacacucauuaau...........  4     0    FW2
.......................................................ugacuagauccacacucauuaaA...........  41    1    FW2
.......................................................ugacuagauccacacucauuaauU..........  1     1    FW2
........................................................gacuagauccacacucauuaa............  1     0    FW2

..............aaugggugugaaucuagugauu.....................................................  2     0    FF1
..............aaugggugugaaucuagugauuu....................................................  2     0    FF1
..............aaugggugugaaucuagugauuuc...................................................  4     0    FF1
..............aaugggugugaaucuagugauuuca..................................................  6     0    FF1
..............aaugggugugaaucuagugauuucaca................................................  3     0    FF1
.......................................................ugacuagauccacacucau...............  6     0    FF1
.......................................................ugacuagauccacacucauu..............  65    0    FF1
.......................................................ugacuagauccacacucauua.............  131   0    FF1
.......................................................ugacuagauccacacucauuaU............  2     1    FF1
.......................................................ugacuagauccacacucauuaa............  438   0    FF1
.......................................................ugacuagaucAacacucauuaa............  1     1    FF1
.......................................................ugacuagauccacacucauuaau...........  41    0    FF1
.......................................................ugacuagauccacacucauuaaG...........  1     1    FF1
.......................................................ugacuagauccacacucauuaaC...........  5     1    FF1
.......................................................ugacuagauccacacucauuaaA...........  142   1    FF1
.......................................................ugacuagauccacacucauuaauA..........  6     1    FF1
........................................................gacuagauccacacucauuaa............  1     0    FF1

..............aaugggugugaaucuagugau......................................................  1     0    OV1
..............aaugggugugaaucuagugauu.....................................................  2     0    OV1
..............aaugggugugaaucuagugauuu....................................................  1     0    OV1
..............aaugggugugaaucuagugauuuc...................................................  1     0    OV1
..............aaugggugugaaucuagugauuucU..................................................  1     1    OV1
..............aaugggugugaaucuagugauuuca..................................................  5     0    OV1
..............aaugggugugaaucuagugauuucaca................................................  2     0    OV1
......................................................Cugacuagauccacacucau...............  1     1    OV1
......................................................Cugacuagauccacacucauua.............  1     1    OV1
.......................................................ugacuagauccacacuc.................  4     0    OV1
.......................................................ugacuagauccacacuca................  12    0    OV1
.......................................................ugacuagauccacacuUau...............  1     1    OV1
.......................................................ugacuagauccacacucau...............  24    0    OV1
.......................................................ugacuagauccaUacucauu..............  1     1    OV1
.......................................................ugacuagauccCcacucauu..............  1     1    OV1
.......................................................ugacCagauccacacucauu..............  1     1    OV1
.......................................................ugacuagauccacacCcauu..............  1     1    OV1
.......................................................ugacuagauccacacucauu..............  515   0    OV1
.......................................................ugacuagaucAacacucauua.............  1     1    OV1
.......................................................ugacuagauccacacucauuU.............  2     1    OV1
.......................................................ugacuagaCccacacucauua.............  1     1    OV1
.......................................................ugacuagauccacacucauua.............  648   0    OV1
.......................................................ugacuagauccGcacucauua.............  1     1    OV1
.......................................................ugacuagaucUacacucauua.............  2     1    OV1
.......................................................ugacuagauGcacacucauuaa............  1     1    OV1
.......................................................ugacuagauccGcacucauuaa............  1     1    OV1
.......................................................ugacuagauccacacucauuaU............  11    1    OV1
.......................................................ugacuagauccacacucauuaC............  6     1    OV1
.......................................................ugacuagaucAacacucauuaa............  1     1    OV1
.......................................................ugacuagauccacacuUauuaa............  2     1    OV1
.......................................................ugacuagauccacacucaCuaa............  1     1    OV1
.......................................................ugacuagaucGacacucauuaa............  1     1    OV1
.......................................................ugacuagauccacacucauuaa............  740   0    OV1
.......................................................ugacuagaCccacacucauuaa............  1     1    OV1
.......................................................ugacuagauUcacacucauuaa............  2     1    OV1
```

aga-miR-279*

aga-miR-279

uuccuaucaguguaaaugggugugaaucuagugauuucacaugaauuuucgauugugacuagauccacacucauuaauguuguuuggaa

....ugacuagauccacacucauuaaA...........	321	1	OV1
....ugacuagauccacacucauuaUu...........	4	1	OV1
....ugacuagauccacacucauuaaC...........	14	1	OV1
....ugacuagauccacacucauuaaG...........	5	1	OV1
....ugacuagauccacacucauuaau...........	52	0	OV1
....ugacuagauccacacucauuaauU..........	2	1	OV1
....ugacuagauccacacucauuaauA..........	3	1	OV1
....gacuagauccacacucauuaa............	1	0	OV1
..............aaugggugugaaucuagugauuucaca..	1	0	MF1
....ugacuagauccacacuc.................	1	0	MF1
....ugacuagauccacacuca................	2	0	MF1
....ugacuagauccacacucau...............	2	0	MF1
....ugacuagauccacacucauu..............	90	0	MF1
....ugacuagauccacacucauua.............	75	0	MF1
....ugacuagauUcacacucauua.............	1	1	MF1
....ugacuagaucUacacucauua.............	1	1	MF1
....ugacuagauccacaUucauuaa............	1	1	MF1
....ugacuagauccacacucauuaU............	5	1	MF1
....ugacuagauccacacucauuaa............	76	0	MF1
....ugacuagauccacacucauuaau...........	5	0	MF1
....ugacuagauccacacucauuaaA...........	24	1	MF1
....ugacuagauccacacucauuaaC...........	1	1	MF1
....ugacuagauccacacAcauuaau...........	1	1	MF1
....gacuagauccacacucauua.............	1	0	MF1
..............aaugggugugaaucuagugauu...	3	0	BF2
..............aaugggugugaaucuagugauuu..	1	0	BF2
..............aaugggugugaaucuagugauuuc...	3	0	BF2
..............aaugggugugaaucuagugauuuca..	6	0	BF2
..............aaugggugugaaucuagugauuucac...	2	0	BF2
.................gggugugaaucuagugauuu..	1	0	BF2
....Cugacuagauccacacucauua.............	1	1	BF2
....Cugacuagauccacacucauuaa............	1	1	BF2
....ugacuagauccacacuc.................	5	0	BF2
....ugacuagauccacacuca................	1	0	BF2
....ugacuagauccacacucau...............	15	0	BF2
....ugacuagaucAacacucauu..............	1	1	BF2
....ugacuagauccacacucauu..............	270	0	BF2
....ugacuagauccacacuUauu..............	1	1	BF2
....ugacuagaCccacacucauua.............	1	1	BF2
....ugacuagauccaUacucauua.............	1	1	BF2
....ugacuagauccacacucauuG.............	1	1	BF2
....ugacuagauccacacucauua.............	403	0	BF2
....ugacuagauccacacucauuU.............	1	1	BF2
....ugacuagauccacacuUauua.............	1	1	BF2
....ugacuagauUcacacucauuaa............	1	1	BF2
....ugacuagauccacacucauuaG............	1	1	BF2
....ugacuagauccacacucauuaU............	10	1	BF2
....uAacuagauccacacucauuaa............	2	1	BF2
....ugacuagauccacacucauuaC............	5	1	BF2
....ugacuagauccacacucauGaa............	1	1	BF2
....ugacuagauccacacucauuaa............	1149	0	BF2
....ugacuagauAcacacucauuaa............	1	1	BF2
....Cgacuagauccacacucauuaa............	1	1	BF2
....ugacuaAauccacacucauuaa............	1	1	BF2
....ugacGagauccacacucauuaa............	1	1	BF2
....ugacuagaucUacacucauuaa............	1	1	BF2
....ugacuagauccaUacucauuaa............	2	1	BF2
....ugacuagauccacacucauuaaA...........	333	1	BF2
....ugacuagauUcacacucauuaau...........	1	1	BF2
....ugacuagauAcacacucauuaau...........	1	1	BF2
....ugacuagauccacacucauuaaC...........	14	1	BF2
....ugacuagauccacacucauuaau...........	75	0	BF2
....ugacuagauccacacucauuaaG...........	1	1	BF2
....ugacuagauccacacucauuaaug..........	1	0	BF2
....ugacuagauccacacucauuaauA..........	8	1	BF2
....ugacuagauccacacucauuaauU..........	4	1	BF2
....gacuagauccacacucauua.............	1	0	BF2

```
              aga-miR-279*
                                                       aga-miR-279
uuccuaucaguguaaaugggugugaaucuagugauuucacaugaauuuucgauugugacuagauccacacucauuaauguuguuuggaa

........................................................gacuagauccacacucauuaau...........    1    0    BF2

..............aaugggugugaaucuagugauu.....................................................    2    0    BF1
..............aaugggugugaaucuagugauuu....................................................    2    0    BF1
..............aaugggugugaaucuagugauuuc...................................................    4    0    BF1
..............aaugggugugaaucuagugauuuca..................................................    6    0    BF1
..............aaugggugugaaucuagugauuucaca................................................    3    0    BF1
.......................................................ugacuagauccacacucau...............    6    0    BF1
.......................................................ugacuagauccacacucauu..............    65   0    BF1
.......................................................ugacuagauccacacucauua.............    131  0    BF1
.......................................................ugacuagauccacacucauuaU............    2    1    BF1
.......................................................ugacuagaucAacacucauuaa............    1    1    BF1
.......................................................ugacuagauccacacucauuaa............    437  0    BF1
.......................................................ugacuagauccacacucauuaaG...........    1    1    BF1
.......................................................ugacuagauccacacucauuaaA...........    142  1    BF1
.......................................................ugacuagauccacacucauuaaC...........    5    1    BF1
.......................................................ugacuagauccacacucauuaau...........    41   0    BF1
.......................................................ugacuagauccacacucauuaauA..........    5    1    BF1
........................................................gacuagauccacacucauuaa............    1    0    BF1

..............aaugggugugaaucuagugauuucaca................................................    3    0    MW1
......................................................Cugacuagauccacacucauu..............    1    1    MW1
.......................................................ugacuagauccacacuc.................    2    0    MW1
.......................................................ugacuagauccacacuca................    1    0    MW1
.......................................................ugacuagauccacacucau...............    2    0    MW1
.......................................................ugacuagauccacacucauu..............    76   0    MW1
.......................................................ugacuagauccacacucauua.............    151  0    MW1
.......................................................ugacuagauccacacucauuG.............    1    1    MW1
.......................................................ugacuagauccacacucauuGa............    1    1    MW1
.......................................................ugacuagauccacacucauuaa............    358  0    MW1
.......................................................ugacuagauccacacucauuaU............    3    1    MW1
.......................................................ugacuagauccacacucauuaC............    2    1    MW1
.......................................................ugacuagauccacacucauuaaG...........    1    1    MW1
.......................................................ugacuagauccacacucauuaaA...........    53   1    MW1
.......................................................ugacuagauccacacucauuaau...........    7    0    MW1
.......................................................ugacuagauccacacucauuaaC...........    2    1    MW1
.......................................................ugacuagauccacacucauuaaAg..........    1    1    MW1

..............aaugggugugaaucuagugauuuc...................................................    1    0    FW1
..............aaugggugugaaucuagugauuuca..................................................    1    0    FW1
......................................................Cugacuagauccacacucauua.............    1    1    FW1
.......................................................ugacuagauccacacucauu..............    25   0    FW1
.......................................................ugacuagauccUcacucauua.............    1    1    FW1
.......................................................ugacuagauccacacucauua.............    59   0    FW1
.......................................................ugacuagauccacacCcauuaa............    1    1    FW1
.......................................................ugacuagauccacacucauuaa............    173  0    FW1
.......................................................ugacuagauccacacucauuaau...........    2    0    FW1
.......................................................ugacuagauccacacucauuaaA...........    19   1    FW1

..............aaugggugugaaucuagugauuuca..................................................    1    0    MW2
..............aaugggugugaaucuagugauAucaca................................................    1    1    MW2
.......................................................ugacuagauccacacucau...............    1    0    MW2
.......................................................ugacuagauccacacucauu..............    12   0    MW2
.......................................................ugacuagauccacacucauua.............    22   0    MW2
.......................................................ugacuagauccacacucauuaU............    1    1    MW2
.......................................................ugacuagauccacacucauuaC............    4    1    MW2
.......................................................ugacuagauccacacucauuaa............    71   0    MW2
.......................................................ugacuagauccacacucauuaau...........    1    0    MW2
.......................................................ugacuagauccacacucauuaaA...........    10   1    MW2

..............aaugggugugaaucuagugauu.....................................................    1    0    TE2
..............aaugggugugaaucuagugauuuca..................................................    3    0    TE2
......................................................Cugacuagauccacacucauu..............    2    1    TE2
......................................................Cugacuagauccacacucauua.............    1    1    TE2
......................................................Cugacuagauccacacucauuaa............    1    1    TE2
.......................................................ugacuagauccGcacuc.................    1    1    TE2
.......................................................ugacuagauccacacuc.................    15   0    TE2
.......................................................ugacuagauccacacuca................    22   0    TE2
.......................................................ugacuagauccacacucau...............    45   0    TE2
```

```
              aga-miR-279*
                                                       aga-miR-279
uuccuaucaguguaaaugggugugaaucuagugauuucacaugaauuuucgauugugacuagauccacacucauuaauguuguuuggaa

.......................................................ugUcuagauccacacucauu..............    1      1     TE2
.......................................................ugacuagauccacacucaCu..............    1      1     TE2
.......................................................ugacuagauccacacucauu..............    602    0     TE2
.......................................................ugacuagauGcacacucauu..............    1      1     TE2
.......................................................ugacuagauUcacacucauu..............    1      1     TE2
.......................................................ugacuGgauccacacucauu..............    1      1     TE2
.......................................................ugaUuagauccacacucauu..............    1      1     TE2
.......................................................ugacuagaAccacacucauu..............    1      1     TE2
.......................................................ugacuagauccacacucauua.............    799    0     TE2
.......................................................ugacuagauccacacuUauua.............    1      1     TE2
.......................................................ugacuagauccacacucauuU.............    2      1     TE2
.......................................................ugacuagauAcacacucauua.............    1      1     TE2
.......................................................ugacuagauccacacucGuua.............    1      1     TE2
.......................................................ugacuagaucUacacucauua.............    2      1     TE2
.......................................................Cgacuagauccacacucauua.............    1      1     TE2
.......................................................uAacuagauccacacucauua.............    1      1     TE2
.......................................................ugacuagauccaUacucauua.............    1      1     TE2
.......................................................ugacuagauccacacuAauua.............    1      1     TE2
.......................................................ugacuagauccCcacucauua.............    1      1     TE2
.......................................................ugacuaAauccacacucauua.............    1      1     TE2
.......................................................ugacuagauccacacucauuaU............    6      1     TE2
.......................................................ugacuagauccacacucCuuaa............    1      1     TE2
.......................................................ugacCagauccacacucauuaa............    1      1     TE2
.......................................................ugacuagauccacacucauuaC............    1      1     TE2
.......................................................uAacuagauccacacucauuaa............    1      1     TE2
.......................................................ugUcuagauccacacucauuaa............    1      1     TE2
.......................................................ugacuagauccacacucauuaG............    2      1     TE2
.......................................................ugacuaAauccacacucauuaa............    1      1     TE2
.......................................................ugacuagauccacacucauuaa............    618    0     TE2
.......................................................ugacuagauccacacucauuaau...........    27     0     TE2
.......................................................ugacuagauccacacucauuaaA...........    114    1     TE2
.......................................................ugacuagauccacacucauuaaC...........    3      1     TE2
........................................................gacuagauccacacucauu..............    1      0     TE2
```

miRBase precursor : aga-mir-281
Total read count : 1028753
aga-miR-281 read count : 1004374
aga-miR-281* read count : 24013
remaining reads : 366

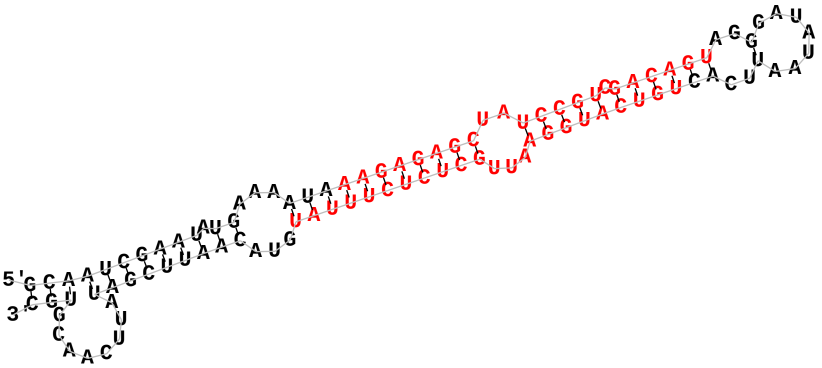

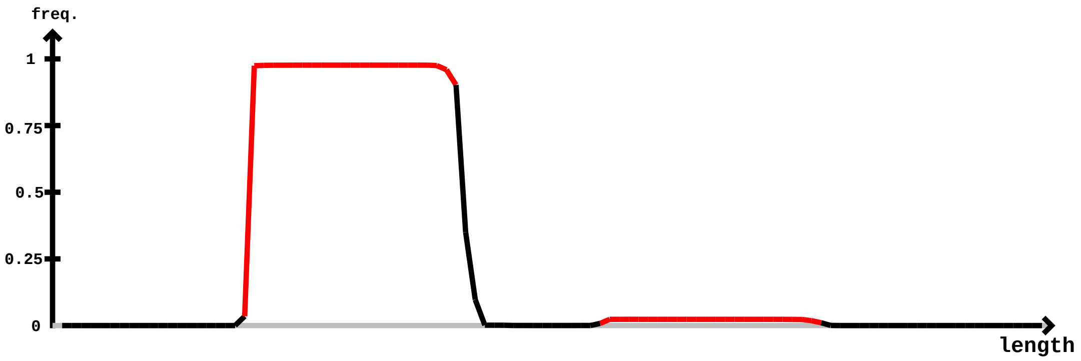

aga-miR-281*
aga-miR-281

```
5'- gcaaucgaauaugaaaauaaagagagcuauccgucgacaguagggauauaauucacugucauggaauugcucucuuuauguacaauucgauauucaacgugc  -3'   exp
    ...((((((.((...((((((((((..(((((.((((((..(.......)..)))))))))))...))))))))))...)))))))))..........   reads   mm   sample
    .................aaagagagcuauccgucgaca...............................................................   5       0    TE1
    .................aaagagagcuauccguUgacag..............................................................   1       1    TE1
    .................aaagaAagcuauccgucgacag..............................................................   1       1    TE1
    .................aaagagagcuauccgucgacag..............................................................   43      0    TE1
    .................aaagagagcuauccgucgacagu.............................................................   87      0    TE1
    .................Caagagagcuauccgucgacagu.............................................................   4       1    TE1
    .................aaagagagcuauccgucgacagG.............................................................   1       1    TE1
    .................aaagagagcuauccgucgacaguU............................................................   3       1    TE1
    .................Caagagagcuauccgucgacagua............................................................   3       1    TE1
    .................aaagagagcuauccgucgacagua............................................................   6       0    TE1
    .................aaagagagcuauccgucgacaguaA...........................................................   1       1    TE1
    ..................aagagagcuauccgucgac................................................................   7       0    TE1
    ..................aagagagcuauccgucgaca...............................................................   54      0    TE1
    ..................aagagagcuauccgucgacU...............................................................   1       1    TE1
    ..................aagagagcuauccAucgacag..............................................................   2       1    TE1
    ..................aagagagcuauccgucAacag..............................................................   1       1    TE1
    ..................aagagagcuauccgucgacaA..............................................................   1       1    TE1
    ..................aagagagcuauccgucgacag..............................................................   177     0    TE1
    ..................aagagagcuauccguUgacagu.............................................................   1       1    TE1
    ..................Uagagagcuauccgucgacagu.............................................................   5       1    TE1
    ..................aagagagcuaucGgucgacagu.............................................................   1       1    TE1
    ..................aagagaUcuauccgucgacagu.............................................................   1       1    TE1
    ..................aagagagcuauccgGcgacagu.............................................................   2       1    TE1
    ..................aagagagcuauUcgucgacagu.............................................................   1       1    TE1
    ..................aagagagUuauccgucgacagu.............................................................   1       1    TE1
    ..................aagagagcuauccgucAacagu.............................................................   1       1    TE1
    ..................aagagagcuauccgucgacagC.............................................................   1       1    TE1
    ..................aagagagcuauccgCcgacagu.............................................................   2       1    TE1
    ..................aagagagcuauAcgucgacagu.............................................................   2       1    TE1
    ..................aagagagcuauccgucgacagu.............................................................   2273    0    TE1
    ..................aagagagcuauccgucgacagG.............................................................   10      1    TE1
    ..................aagagagcuauccgucUacagu.............................................................   1       1    TE1
    ..................aagagagcuauccgucgacagA.............................................................   6       1    TE1
    ..................aagagGgcuauccgucgacagu.............................................................   1       1    TE1
```

```
                                                        aga-miR-281*
                   aga-miR-281
gcaaucgaauaugaaaauaaagagagcuauccgucgacaguagggauauaauucacugucauggaauugcucucuuuauguacaauucgauauucaacgugc

...................aagagagcuauccgucgaUagu.............................................................   3      1   TE1
...................aagagagcuauccgucCacagu.............................................................   1      1   TE1
...................aagagagcuaAccgucgacagu.............................................................   1      1   TE1
...................aagagagcuauccgucgacaguC............................................................   2      1   TE1
...................aagagagcuauccgucgacagCa............................................................   1      1   TE1
...................aagagagcuauccgCcgacagua............................................................   1      1   TE1
...................aagagagcuaucAgucgacagua............................................................   1      1   TE1
...................Uagagagcuauccgucgacagua............................................................   1      1   TE1
...................aagagagcuauccgucgacaguU............................................................   55     1   TE1
...................aagagagcuauccgucgacagua............................................................   1290   0   TE1
...................aagagagcuauUcgucgacagua............................................................   2      1   TE1
...................aagagagcuauAcgucgacagua............................................................   1      1   TE1
...................aagagagcuauccgucgacagGa............................................................   3      1   TE1
...................aagagagcuauccguUgacagua............................................................   1      1   TE1
...................aagagaAcuauccgucgacagua............................................................   1      1   TE1
...................aagagagcuUuccgucgacagua............................................................   1      1   TE1
...................aagagagcuauccUucgacagua............................................................   1      1   TE1
...................aagagagcuauccgucgacaguaU...........................................................   145    1   TE1
...................aagagagcuauccgucgacaguaA...........................................................   152    1   TE1
...................aagagagcuauccgucgacaguaC...........................................................   9      1   TE1
....................agagagcuauccgucgacag..............................................................   1      0   TE1
....................agagagcuauccgucgacagu.............................................................   4      0   TE1
....................agagagcuaucGgucgacagua............................................................   1      1   TE1
....................agagagcuauccgucgacagua............................................................   5      0   TE1
....................agagagcuauccgucgacaguaA...........................................................   1      1   TE1
....................agagagcuauccgucgacaguaU...........................................................   1      1   TE1
.......................................................cugucauggaauugcucucu...........................   1      0   TE1
.......................................................cugucauggaauugcucucuuu.........................   6      0   TE1
.......................................................cugucauggaauugcucucuuua........................   6      0   TE1
.......................................................cugucauggaauugcucucuuuaA.......................   3      1   TE1
.......................................................cugucauggaauugcucucuuuau.......................   4      0   TE1
.......................................................cugucauggaauugcucucuuuauU......................   2      1   TE1
........................................................ugucauggaauugcucucuuu.........................   5      0   TE1
........................................................ugucauggaauugcucucuuua........................   13     0   TE1
........................................................ugucauggaauugcucucuuuU........................   1      1   TE1
........................................................ugucauggaauugcucucuuuau.......................   17     0   TE1
........................................................ugucauggaauugcucucuuuaC.......................   2      1   TE1
........................................................ugucauggaauugcucucuuuUu.......................   1      1   TE1
........................................................ugucauggaauugcucucuuuauU......................   2      1   TE1

.................uaaagagagcuauccgucgaca...............................................................   5      0   OV2
.................uaaagagagcuauccgucgacaA..............................................................   1      1   OV2
.................uaaagagagcuauccgucgacag..............................................................   3      0   OV2
.................Caaagagagcuauccgucgacagu.............................................................   2      1   OV2
..................aaagagagcuauccgucga.................................................................   2      0   OV2
..................aaagagagcuauccgucgac................................................................   15     0   OV2
..................aaagagagcuauccgucCac................................................................   1      1   OV2
..................aaagagagcuauccgucgaca...............................................................   155    0   OV2
..................aaagagagcuauccgucgacGg..............................................................   1      1   OV2
..................aaagagagcuaAccgucgacag..............................................................   1      1   OV2
..................aaagagagcuCuccgucgacag..............................................................   1      1   OV2
..................aaagagagcuauccgucgacaA..............................................................   10     1   OV2
..................aaagagagcuauccgucgacaU..............................................................   1      1   OV2
..................aaagagagcuauccgucgacag..............................................................   431    0   OV2
..................aaagagagcuaGccgucgacagu.............................................................   1      1   OV2
..................aUagagagcuauccgucgacagu.............................................................   1      1   OV2
..................aaagagagcuauccgucgacagu.............................................................   638    0   OV2
..................Uaagagagcuauccgucgacagu.............................................................   1      1   OV2
..................aaagagagcuauccgucgacagG.............................................................   1      1   OV2
..................aaagagagAuauccgucgacagu.............................................................   1      1   OV2
..................Caagagagcuauccgucgacagu.............................................................   10     1   OV2
..................aaagagagcuauccgucAacagu.............................................................   1      1   OV2
..................aaagagagcuauccgucgacagA.............................................................   8      1   OV2
..................aaagagagUuauccgucgacagu.............................................................   1      1   OV2
..................aaagagagcuaCccgucgacagua............................................................   1      1   OV2
..................aaagagagcuauccgucgacaguU............................................................   22     1   OV2
..................aaagagagcuauccgucgacagAa............................................................   2      1   OV2
..................aaagagagcuauccgucgacaguG............................................................   1      1   OV2
..................aaagagagcuauccgucgacagua............................................................   49     0   OV2
```

```
                                                        aga-miR-281*
                   aga-miR-281
gcaaucgaauaugaaaauaaagagagcuauccgucgacaguagggauauaauucacugucauggaauugcucucuuuauguacaauucgauauucaacgugc

..................Caagagagcuauccgucgacagua............................................................   6      1   OV2
..................aaagagagcuauccgucgacaguaC...........................................................   1      1   OV2
..................aaagagagcuauccgucgacaguaA...........................................................   48     1   OV2
..................aaagagagcuauccgucgacaguaU...........................................................   1      1   OV2
..................aaagagagcuauccgucgacaguagg..........................................................   1      0   OV2
..................aaagagagcuauccgucgacaguaggg.........................................................   2      0   OV2
..................aaagagagcuauccgucgacaguaggga........................................................   7      0   OV2
...................aagagagcuauccgucg..................................................................   1      0   OV2
...................aagagagcuauccgucga.................................................................   4      0   OV2
...................aagagagcuauccgucgac................................................................   47     0   OV2
...................aagagagcuauccUucgaca...............................................................   1      1   OV2
...................aagagagcuauccgucgacU...............................................................   3      1   OV2
...................aagagagcuauccguUgaca...............................................................   1      1   OV2
...................aagagagcuaCccgucgaca...............................................................   1      1   OV2
...................aagagagcuauccgucgaUa...............................................................   1      1   OV2
...................aagagagcuauccgucgaca...............................................................   678    0   OV2
...................aagagagcuauccgucAaca...............................................................   2      1   OV2
...................aagagagcuauccgucgacGg..............................................................   4      1   OV2
...................aagagagcuauccgucUacag..............................................................   1      1   OV2
...................aagagagcuauAcgucgacag..............................................................   1      1   OV2
...................aagagagcuauccgucgaUag..............................................................   1      1   OV2
...................aagagagcuauccguAgacag..............................................................   2      1   OV2
...................aagagaAcuauccgucgacag..............................................................   1      1   OV2
...................aagagagcuauccgucgacag..............................................................   2234   0   OV2
...................aagagagcuauccgucAacag..............................................................   2      1   OV2
...................aagagagcuauUcgucgacag..............................................................   5      1   OV2
...................aagagagcuauccgucgacaA..............................................................   21     1   OV2
...................aGgagagcuauccgucgacag..............................................................   1      1   OV2
...................aagagaCcuauccgucgacag..............................................................   1      1   OV2
...................aagagagcuauccgucgacaU..............................................................   2      1   OV2
...................aagaAagcuauccgucgacag..............................................................   1      1   OV2
...................aagagagcuaCccgucgacag..............................................................   1      1   OV2
...................aagagagcuauccAucgacag..............................................................   1      1   OV2
...................aagagagcuauccguUgacag..............................................................   1      1   OV2
...................aagagaAcuauccgucgacagu.............................................................   3      1   OV2
...................aagagagcuauccgGcgacagu.............................................................   5      1   OV2
...................aagagagcuauccgucgaAagu.............................................................   2      1   OV2
...................aagagagcuauccCucgacagu.............................................................   1      1   OV2
...................aagagagcuauccgucgaUagu.............................................................   7      1   OV2
...................aagGgagcuauccgucgacagu.............................................................   1      1   OV2
...................aagagagcuauccUucgacagu.............................................................   3      1   OV2
...................aagagagcuauccgucgacaAu.............................................................   6      1   OV2
...................aagagagcuaCccgucgacagu.............................................................   10     1   OV2
...................aagaAagcuauccgucgacagu.............................................................   3      1   OV2
...................aagagagcuauccgucgacaUu.............................................................   3      1   OV2
...................aaUagagcuauccgucgacagu.............................................................   1      1   OV2
...................aagagagcuauccgCcgacagu.............................................................   11     1   OV2
...................aagagagUuauccgucgacagu.............................................................   5      1   OV2
...................aagagagcuGuccgucgacagu.............................................................   3      1   OV2
...................Gagagagcuauccgucgacagu.............................................................   4      1   OV2
...................aagagagcuauccgucgacagA.............................................................   13     1   OV2
...................aagagagcuauccgucgacCgu.............................................................   2      1   OV2
...................aagagagcuauccgucgacaCu.............................................................   1      1   OV2
...................aagagagcuaAccgucgacagu.............................................................   6      1   OV2
...................aagagagcuauccgucgacagu.............................................................   26446  0   OV2
...................aagagagcuaucUgucgacagu.............................................................   11     1   OV2
...................aUgagagcuauccgucgacagu.............................................................   1      1   OV2
...................aagagagcuauccgucgGcagu.............................................................   2      1   OV2
...................aagagagGuauccgucgacagu.............................................................   1      1   OV2
...................aGgagagcuauccgucgacagu.............................................................   1      1   OV2
...................aaAagagcuauccgucgacagu.............................................................   4      1   OV2
...................aagagagcuauccgucgacGgu.............................................................   3      1   OV2
...................aagaUagcuauccgucgacagu.............................................................   1      1   OV2
...................aagagagcuaGccgucgacagu.............................................................   3      1   OV2
...................aagagagcuauUcgucgacagu.............................................................   19     1   OV2
...................aagagagcuauccgucUacagu.............................................................   1      1   OV2
...................aagagagcuauccgucCacagu.............................................................   2      1   OV2
...................aagagagcuauccguAgacagu.............................................................   4      1   OV2
...................aagagagcuauccgucgacagG.............................................................   36     1   OV2
```

aga-miR-281*

aga-miR-281

gcaaucgaauaugaaaauaaagagagcuauccgucgacaguagggauauaauucacugucauggaauugcucucuuuauguacaauucgauauucaacgugc

(aga-miR-281 = aagagagcuauccgucgacagu; aga-miR-281* = ugucauggaauugcucucuuuau)

...................aagagagcuauccgucAacagu...	12	1	OV2
...................aagagagcuauccAucgacagu...	2	1	OV2
...................aagagagcuaucAgucgacagu...	5	1	OV2
...................aagagaUcuauccgucgacagu...	4	1	OV2
...................aagagagcuauccguUgacagu...	6	1	OV2
...................aagagagcCauccgucgacagu...	4	1	OV2
...................aagUgagcuauccgucgacagu...	1	1	OV2
...................aagagGgcuauccgucgacagu...	3	1	OV2
...................aagagagcuauAcgucgacagu...	9	1	OV2
...................aagagagcuauccgucgacagC...	10	1	OV2
...................aagagagcuauGcgucgacagu...	11	1	OV2
...................aaCagagcuauccgucgacagu...	1	1	OV2
...................Uagagagcuauccgucgacagu...	2	1	OV2
...................aagagagcuauccgAcgacagu...	4	1	OV2
...................aagagaCcuauccgucgacagu...	1	1	OV2
...................aagagagcuauccgucgacaCua..	1	1	OV2
...................aagagUgcuauccgucgacagua..	1	1	OV2
...................aagagagcuauccgucgacaUua..	1	1	OV2
...................aagagaUcuauccgucgacagua..	2	1	OV2
...................Uagagagcuauccgucgacagua..	1	1	OV2
...................aagagagcuauccgucgacaguG..	5	1	OV2
...................aagagagcuauGcgucgacagua..	3	1	OV2
...................aagagagcuauccgucgacaguC..	11	1	OV2
...................aagagagcuaCccgucgacagua..	3	1	OV2
...................aagagagcuauUcgucgacagua..	1	1	OV2
...................aagagagcCauccgucgacagua..	1	1	OV2
...................Cagagagcuauccgucgacagua..	1	1	OV2
...................aagagagAuauccgucgacagua..	1	1	OV2
...................aagagagcuauccguUgacagua..	9	1	OV2
...................aagagagcuauccgucgacaAua..	5	1	OV2
...................aagagagcuauccgucgacagua..	7943	0	OV2
...................aagagagcuauccgucgacagCa..	4	1	OV2
...................aagagagcuauccgucgacagAa..	7	1	OV2
...................aagagagcuauccgucgaUagua..	2	1	OV2
...................aagagagcuauccguGgacagua..	1	1	OV2
...................aagGgagcuauccgucgacagua..	1	1	OV2
...................aaAagagcuauccgucgacagua..	2	1	OV2
...................aagagagcuauccgucAacagua..	4	1	OV2
...................aagagagcuauccgucgacagGa..	1	1	OV2
...................aagagaAcuauccgucgacagua..	2	1	OV2
...................aagagagcuauAcgucgacagua..	8	1	OV2
...................Gagagagcuauccgucgacagua..	2	1	OV2
...................aagagagcuauccgucgacaguU..	217	1	OV2
...................aagagagcuauccgucgaGagua..	1	1	OV2
...................aagagagcuauccgAcgacagua..	1	1	OV2
...................aagagagcuaGccgucgacagua..	3	1	OV2
...................aagagagUuauccgucgacagua..	1	1	OV2
...................aagagagcuaucAgucgacagua..	1	1	OV2
...................aagagagcuaAccgucgacagua..	3	1	OV2
...................aagagagcuauccAucgacagua..	4	1	OV2
...................aagagagcuauccgucgacaguag...	3	0	OV2
...................aagagagcuauccgucgacaguaA...	4567	1	OV2
...................aagagagcuauccgucgacaguaU...	537	1	OV2
...................aagagagcuauccgucgacaguaC...	75	1	OV2
...................aagagagcuauccgucgacaguagg..	3	0	OV2
...................aagagagcuauccgucgacaguaAg..	7	1	OV2
...................aagagagcuauccgucgacaguaggA...	1	1	OV2
...................aagagagcuauccgucgacaguaggg...	25	0	OV2
...................aagagagcuauccgucgacaguCggga..	1	1	OV2
...................aagagagcuauccgucgacaguaggga..	142	0	OV2
...................aagagagcuauccgucgacaguagggaA...	4	1	OV2
...................aagagagcuauccgucgacaguagggau...	2	0	OV2
...................aagagagcuauccgucgacaguagggaua..	2	0	OV2
...................aagagagcuauccgucgacaguagggauauaauuc..	1	0	OV2
....................agagagcuauccgucgacagu...	29	0	OV2
....................agagagcuauccgucUacagua..	1	1	OV2
....................agagagcuauccgucgacagua..	15	0	OV2
....................agagagcuauccgucgacaguaC...	1	1	OV2
....................agagagcuauccgucgacaguaU...	1	1	OV2

```
                                                     aga-miR-281*
                   aga-miR-281
gcaaucgaauaugaaaauaaagagagcuauccgucgacaguagggauauaauucacugucauggaauugcucucuuuauguacaauucgauauucaacgugc

....................agagagcuauccgucgacaguaA...........................................................    3      1      OV2
.....................gagagcuauccgucgacagua............................................................    1      0      OV2
.......................gagcuauccgucgacagua............................................................    1      0      OV2
.........................gcuauccgucgacagua............................................................    1      0      OV2
...................................................uucacugucauggaauugcucucu...........................    1      0      OV2
....................................................ucacugucauggaauugcucucuu..........................    1      0      OV2
......................................................acugucauggaauugcucucuuuau.......................    1      0      OV2
.......................................................cugucauggaauugcucucu...........................    4      0      OV2
.......................................................cugucauggaauugcucucuu..........................    7      0      OV2
.......................................................cugucauggaauugcucucCuu.........................    2      1      OV2
.......................................................cugucauggaauugcucucuuu.........................    35     0      OV2
.......................................................cugucaugAaauugcucucuuua........................    1      1      OV2
.......................................................cugucauggaauugcucucuuua........................    63     0      OV2
.......................................................cugucauggaauugcucucuuuU........................    3      1      OV2
.......................................................cugucauggaauugcucucuuuau.......................    24     0      OV2
.......................................................cugucauggaauugcucucuuuaC.......................    2      1      OV2
.......................................................cugucauggaauugcucucuuuaA.......................    23     1      OV2
.......................................................cugucauggaauugcucucuuuUu.......................    1      1      OV2
.......................................................cugucauggaauugcucucuuuaAg......................    1      1      OV2
........................................................ugucauggaauugcucuc............................    2      0      OV2
........................................................ugucauggaauugcucucu...........................    4      0      OV2
........................................................ugucauggaauugcucucuu..........................    10     0      OV2
........................................................ugucauggaauugcucucuuu.........................    81     0      OV2
........................................................ugucauggaauugcucucCuu.........................    4      1      OV2
........................................................ugucauggaauugcucucuuuU........................    7      1      OV2
........................................................ugucauAgaauugcucucuuua........................    1      1      OV2
........................................................ugucauggaauugcucucuuua........................    169    0      OV2
........................................................uUucauggaauugcucucuuua........................    1      1      OV2
........................................................ugucauggaauugcucucuuuUu.......................    1      1      OV2
........................................................ugucauggaauugcucucuuuaA.......................    102    1      OV2
........................................................ugucauggaauugcucucuuuaG.......................    2      1      OV2
........................................................ugucauggaauugcucucuuuau.......................    140    0      OV2
........................................................ugucauggaauugcucucuuuaC.......................    34     1      OV2
........................................................ugucauggaauugcucucuCuau.......................    1      1      OV2
........................................................ugucauggaauugcucucuuuauU......................    7      1      OV2
........................................................ugucauggaauugcucucuuuauA......................    16     1      OV2

................auCaagagagcuauccgucgacagua............................................................    1      1      FF2
.................uaaagagagcuauccgucgacag..............................................................    1      0      FF2
.................Caaagagagcuauccgucgacagu.............................................................    1      1      FF2
..................aaagagagcuauccgucga.................................................................    1      0      FF2
..................aaagagagcuauccgucgac................................................................    35     0      FF2
..................aaagagagcuauccgucgaU................................................................    2      1      FF2
..................aaagagagcuauccgucgaca...............................................................    302    0      FF2
..................aaagagagcuauccgucgacU...............................................................    5      1      FF2
..................aaagagagcuauUcgucgaca...............................................................    1      1      FF2
..................aaagagagcuauccgucAaca...............................................................    1      1      FF2
..................Caagagagcuauccgucgaca...............................................................    1      1      FF2
..................aaagagagcuauccgucgacaC..............................................................    3      1      FF2
..................aaagagagcuauccgucgacag..............................................................    713    0      FF2
..................aaagagagcuauccgucgacaU..............................................................    9      1      FF2
..................aaagagagcuauUcgucgacag..............................................................    1      1      FF2
..................Caagagagcuauccgucgacag..............................................................    5      1      FF2
..................aaagagagcuauccgucgacaA..............................................................    8      1      FF2
..................aaagagagcuCuccgucgacag..............................................................    1      1      FF2
..................aaagagagcuauccguGgacag..............................................................    2      1      FF2
..................aGagagagcuauccgucgacag..............................................................    1      1      FF2
..................aaagaAagcuauccgucgacag..............................................................    1      1      FF2
..................aaUgagagcuauccgucgacag..............................................................    1      1      FF2
..................aaGgagagcuauccgucgacagu.............................................................    1      1      FF2
..................aaagagagcuauccgucgacagu.............................................................    1129   0      FF2
..................aaagagagcuaGccgucgacagu.............................................................    2      1      FF2
..................aaagagaAcuauccgucgacagu.............................................................    1      1      FF2
..................aaagagagcuauccgucgacagC.............................................................    5      1      FF2
..................aaagagagcuauccAucgacagu.............................................................    1      1      FF2
..................aaagagagcuauccgucgacagG.............................................................    1      1      FF2
..................aaaAagagcuauccgucgacagu.............................................................    1      1      FF2
..................aaagagagcuauccgucgacaCu.............................................................    1      1      FF2
..................aaagagagcuauccgucgacagA.............................................................    2      1      FF2
```

```
                                                    aga-miR-281*
                  aga-miR-281
gcaaucgaauaugaaaauaaagagagcuauccgucgacaguagggauauaauucacugucauggaauugcucuuuauguacaauucgauauucaacgugc

..................aaagagagcuauccgucgUcagu...........................................................   1      1      FF2
..................aaagagagcuauccgCcgacagu...........................................................   1      1      FF2
..................aaagagagcuauccgucgacaAu...........................................................   1      1      FF2
..................Caagagagcuauccgucgacagu...........................................................   31     1      FF2
..................aaagagagcuauccgucAacagu...........................................................   1      1      FF2
..................aaagagagcuauccgucgacaUu...........................................................   1      1      FF2
..................aaagagagcuauAcgucgacagu...........................................................   1      1      FF2
..................aaagagagcuaucUgucgacagu...........................................................   1      1      FF2
..................aaagagagcuauccgucgacaguU..........................................................   50     1      FF2
..................aaagagagcuauccgucgacagAa..........................................................   3      1      FF2
..................Caagagagcuauccgucgacagua..........................................................   10     1      FF2
..................aaagagagcuauccgucgacagua..........................................................   44     0      FF2
..................aaagagagcuauccgucgacaguaU.........................................................   9      1      FF2
..................aaagagagcuauccgucgacaguaA.........................................................   11     1      FF2
..................aaagagagcuauccgucgacaguagg........................................................   1      0      FF2
..................aaagagagcuauccgucgacaguagggauauaauuc..............................................   1      0      FF2
..................aaagagagcuauccgucgacaguagggauauaauucacu...........................................   1      0      FF2
...................aagagagcuauccgucga...............................................................   7      0      FF2
...................aagaCagcuauccgucgac..............................................................   1      1      FF2
...................aagagagcuauccgucgaU..............................................................   2      1      FF2
...................aagagagcuauccgucgac..............................................................   110    0      FF2
...................aagagagAuauccgucgaca.............................................................   1      1      FF2
...................aagagagcuauccgucCaca.............................................................   2      1      FF2
...................aagagCgcuauccgucgaca.............................................................   1      1      FF2
...................aagGgagcuauccgucgaca.............................................................   2      1      FF2
...................aagagagcuauccgucgaAa.............................................................   1      1      FF2
...................aagagagcuauAcgucgaca.............................................................   1      1      FF2
...................aagagagcuauccgucgacU.............................................................   16     1      FF2
...................aagagagcuaucGgucgaca.............................................................   1      1      FF2
...................aagagagcuauccgucgaca.............................................................   1123   0      FF2
...................aagagagcuauccCucgaca.............................................................   1      1      FF2
...................Gagagagcuauccgucgaca.............................................................   1      1      FF2
...................aagagagcuauccguAgaca.............................................................   1      1      FF2
...................aagagagcuauccgucgacG.............................................................   1      1      FF2
...................aagagagcuUuccgucgacag............................................................   1      1      FF2
...................aagagagcuauccgucgacaA............................................................   14     1      FF2
...................aagagagcuauccgucgaUag............................................................   1      1      FF2
...................Gagagagcuauccgucgacag............................................................   2      1      FF2
...................aagagagcuauccgucAacag............................................................   1      1      FF2
...................aagagagcuauGcgucgacag............................................................   2      1      FF2
...................aagagagcuauccgucgacGg............................................................   3      1      FF2
...................aagagagcuauccgucgacag............................................................   3764   0      FF2
...................aagagagUuauccgucgacag............................................................   2      1      FF2
...................aagagagcuauccguGgacag............................................................   1      1      FF2
...................aagagagcuauccguUgacag............................................................   2      1      FF2
...................aagagagcuauccAucgacag............................................................   1      1      FF2
...................aagagagcuauAcgucgacag............................................................   3      1      FF2
...................aagagagcuauUcgucgacag............................................................   5      1      FF2
...................aagagagcuaucAgucgacag............................................................   1      1      FF2
...................aagaAagcuauccgucgacag............................................................   1      1      FF2
...................aagagagcuaGccgucgacag............................................................   1      1      FF2
...................aagagagcuauccgucgacaU............................................................   5      1      FF2
...................aagagagcuGuccgucgacag............................................................   1      1      FF2
...................aagagagcuauccgCcgacag............................................................   1      1      FF2
...................aagagaAcuauccgucgacag............................................................   2      1      FF2
...................aagagagcuaAccgucgacag............................................................   2      1      FF2
...................aagagagcuUuccgucgacagu...........................................................   4      1      FF2
...................aagaUagcuauccgucgacagu...........................................................   5      1      FF2
...................aagagagGuauccgucgacagu...........................................................   1      1      FF2
...................aagagagcuauccgCcgacagu...........................................................   17     1      FF2
...................aagagagcuauccCucgacagu...........................................................   1      1      FF2
...................aagagagcuaCccgucgacagu...........................................................   11     1      FF2
...................aagagagcuauccgucgacGgu...........................................................   6      1      FF2
...................aagagagUuauccgucgacagu...........................................................   8      1      FF2
...................aagagagcuauccguUgacagu...........................................................   20     1      FF2
...................Gagagagcuauccgucgacagu...........................................................   5      1      FF2
...................aagagagcuauccAucgacagu...........................................................   14     1      FF2
...................aagGgagcuauccgucgacagu...........................................................   4      1      FF2
...................aagagagcuaucUgucgacagu...........................................................   12     1      FF2
```

```
                                                       aga-miR-281*
                   aga-miR-281
gcaaucgaauaugaaaauaaagagagcuauccgucgacaguagggauauaauucacugucauggaauugcucucuuuauguacaauucgauauucaacgugc

...................aagagagcuauccgucgGcagu.............................................................   8      1    FF2
...................aagagagcuauccgucUacagu.............................................................   3      1    FF2
...................aagagagcuauccgucgacUgu.............................................................   1      1    FF2
...................aagagagcuauccgucgaUagu.............................................................   14     1    FF2
...................aagagagcuauccguAgacagu.............................................................   13     1    FF2
...................aagagagcuauAcgucgacagu.............................................................   29     1    FF2
...................aagagaUcuauccgucgacagu.............................................................   2      1    FF2
...................aagagagcuauccgucgacagC.............................................................   43     1    FF2
...................Uagagagcuauccgucgacagu.............................................................   5      1    FF2
...................aagagagcuauccgucgacaAu.............................................................   5      1    FF2
...................aagagagcuauccgucgCcagu.............................................................   4      1    FF2
...................aagagagcuauccgucgUcagu.............................................................   4      1    FF2
...................aagagUgcuauccgucgacagu.............................................................   1      1    FF2
...................aagagagcuauccgucgacaCu.............................................................   2      1    FF2
...................aagagagcuauccgucgaAagu.............................................................   5      1    FF2
...................aagagagcuauccgucgacagG.............................................................   79     1    FF2
...................aGgagagcuauccgucgacagu.............................................................   5      1    FF2
...................aagagagcuauccgucgacagu.............................................................   38807  0    FF2
...................aagagagcuauccgAcgacagu.............................................................   6      1    FF2
...................aagagagcuauccgucgaGagu.............................................................   1      1    FF2
...................aagagagcuGuccgucgacagu.............................................................   11     1    FF2
...................aUgagagcuauccgucgacagu.............................................................   9      1    FF2
...................aagagagcuaGccgucgacagu.............................................................   5      1    FF2
...................Cagagagcuauccgucgacagu.............................................................   1      1    FF2
...................aaCagagcuauccgucgacagu.............................................................   1      1    FF2
...................aagagagcAauccgucgacagu.............................................................   1      1    FF2
...................aagagagcuauGcgucgacagu.............................................................   18     1    FF2
...................aagagaAcuauccgucgacagu.............................................................   11     1    FF2
...................aaUagagcuauccgucgacagu.............................................................   4      1    FF2
...................aagagagcuauccgucgacCgu.............................................................   2      1    FF2
...................aagagagcuCuccgucgacagu.............................................................   3      1    FF2
...................aagagagAuauccgucgacagu.............................................................   3      1    FF2
...................aagagagcuauccgucgacaUu.............................................................   4      1    FF2
...................aCgagagcuauccgucgacagu.............................................................   1      1    FF2
...................aagagagcuaucAgucgacagu.............................................................   8      1    FF2
...................aagagagcuauccgucCacagu.............................................................   6      1    FF2
...................aagagagcuauccgucAacagu.............................................................   18     1    FF2
...................aaAagagcuauccgucgacagu.............................................................   5      1    FF2
...................aagagagcuaAccgucgacagu.............................................................   7      1    FF2
...................aagagCgcuauccgucgacagu.............................................................   2      1    FF2
...................aagUgagcuauccgucgacagu.............................................................   3      1    FF2
...................aagagagcuauccgucgacagA.............................................................   24     1    FF2
...................aagagagcuauccguGgacagu.............................................................   3      1    FF2
...................aagagagcCauccgucgacagu.............................................................   10     1    FF2
...................aagagagcuaucGgucgacagu.............................................................   2      1    FF2
...................aagagagcuauUcgucgacagu.............................................................   34     1    FF2
...................aagagagcuauccUucgacagu.............................................................   2      1    FF2
...................aagagGgcuauccgucgacagu.............................................................   9      1    FF2
...................aagaAagcuauccgucgacagu.............................................................   7      1    FF2
...................aagaAagcuauccgucgacagua............................................................   6      1    FF2
...................aagagagcuauccgucgaUagua............................................................   5      1    FF2
...................aagagagcuauccgucgacaUua............................................................   1      1    FF2
...................aagUgagcuauccgucgacagua............................................................   1      1    FF2
...................aagaUagcuauccgucgacagua............................................................   1      1    FF2
...................aagagagcuauccgucAacagua............................................................   11     1    FF2
...................aagagGgcuauccgucgacagua............................................................   4      1    FF2
...................aaUagagcuauccgucgacagua............................................................   1      1    FF2
...................aagagagcuauccgucgacagGa............................................................   31     1    FF2
...................aagagagcuaucUgucgacagua............................................................   6      1    FF2
...................aagagagcuauGcgucgacagua............................................................   8      1    FF2
...................Gagagagcuauccgucgacagua............................................................   2      1    FF2
...................Uagagagcuauccgucgacagua............................................................   1      1    FF2
...................aagagagcuauccgucgacaAua............................................................   6      1    FF2
...................aagagagcuauUcgucgacagua............................................................   11     1    FF2
...................aagagagcuauccgucgacagCa............................................................   22     1    FF2
...................aagagagcuauccgucgacaguU............................................................   680    1    FF2
...................aagagagcuaucAgucgacagua............................................................   1      1    FF2
...................aagagagcuauccgCcgacagua............................................................   7      1    FF2
...................aUgagagcuauccgucgacagua............................................................   2      1    FF2
```

```
                                                  aga-miR-281*
                  aga-miR-281
gcaaucgaauaugaaaauaaagagagcuauccgucgacaguagggauauaauucacugucauggaauugcucucuuuauguacaauucgauauucaacgugc

...................aagagagcuauccgucUacagua............................................................ 1      1   FF2
...................aagGgagcuauccgucgacagua............................................................ 4      1   FF2
...................aagagagcuauccgAcgacagua............................................................ 1      1   FF2
...................aagagagcuCuccgucgacagua............................................................ 1      1   FF2
...................aagagagcuauccgucgacUgua............................................................ 1      1   FF2
...................aagagagcuGuccgucgacagua............................................................ 1      1   FF2
...................aagagagcuauccgucgacGgua............................................................ 2      1   FF2
...................aagagagcuauAcgucgacagua............................................................ 19     1   FF2
...................aagagagcuauccgucgGcagua............................................................ 2      1   FF2
...................aagagagcuauccgucgaAagua............................................................ 1      1   FF2
...................aagagagcuauccguAgacagua............................................................ 4      1   FF2
...................aagagagcuauccCucgacagua............................................................ 1      1   FF2
...................aagagagcuauccgucgacaguC............................................................ 5      1   FF2
...................aagagagAuauccgucgacagua............................................................ 1      1   FF2
...................aagagagcAauccgucgacagua............................................................ 1      1   FF2
...................aagagagcuaucGgucgacagua............................................................ 3      1   FF2
...................aagagagcuaAccgucgacagua............................................................ 3      1   FF2
...................aagagaCcuauccgucgacagua............................................................ 1      1   FF2
...................aagagagcuauccgucgacaguG............................................................ 5      1   FF2
...................aagagagcuauccgucgacagAa............................................................ 4      1   FF2
...................aGgagagcuauccgucgacagua............................................................ 2      1   FF2
...................aagagaAcuauccgucgacagua............................................................ 3      1   FF2
...................aagagagcuauccguGgacagua............................................................ 1      1   FF2
...................aagagagcuauccUucgacagua............................................................ 3      1   FF2
...................aaAagagcuauccgucgacagua............................................................ 2      1   FF2
...................aagagagCauccgucgacagua............................................................. 4      1   FF2
...................aagagagcuUuccgucgacagua............................................................ 1      1   FF2
...................aagagagcuauccguUgacagua............................................................ 4      1   FF2
...................aagagagcuaCccgucgacagua............................................................ 7      1   FF2
...................aagagagcuauccgucCacagua............................................................ 4      1   FF2
...................aagaCagcuauccgucgacagua............................................................ 1      1   FF2
...................aagagagGuauccgucgacagua............................................................ 1      1   FF2
...................aagagagcuauccAucgacagua............................................................ 7      1   FF2
...................aagagagUuauccgucgacagua............................................................ 7      1   FF2
...................aagagagcuauccgucgacagua............................................................ 16938  0   FF2
...................aagagagcuauccgucgacaguaC........................................................... 164    1   FF2
...................aagagagcuauccgucgacaguaA........................................................... 3450   1   FF2
...................aagagagcuauccgucgacaguaU........................................................... 1833   1   FF2
...................aagagagcuauccgucgacaguag........................................................... 3      0   FF2
...................aagagagcuauccgucgacaguagA.......................................................... 1      1   FF2
...................aagagagcuauccgucgacaguaUg.......................................................... 3      1   FF2
...................aagagagcuauccgucgacaguagg.......................................................... 1      0   FF2
...................aagagagcuauccgucgacaguaggga........................................................ 1      0   FF2
...................aagagagcuauccgucgacaguagggau....................................................... 2      0   FF2
...................aagagagcuauccgucgacaguagggauauaauu................................................. 1      0   FF2
...................aagagagcuauccgucgacaguagggauauaauuc................................................ 6      0   FF2
...................aagagagcuauccgucgacaguagggauauaauuca............................................... 2      0   FF2
...................aagagagcuauccgucgacaguagggauauaauucacu............................................. 2      0   FF2
....................agagagcuauccgucgaca............................................................... 2      0   FF2
....................agagagcuauccgucgacag.............................................................. 4      0   FF2
....................agagagcuauccgucgacagu............................................................. 38     0   FF2
....................agagagcuauccgucAacagu............................................................. 1      1   FF2
....................agagagcuauccgucgacaguU............................................................ 3      1   FF2
....................agagagcuauccgucgacagua............................................................ 40     0   FF2
....................agagagcuauccgucgacaguaA........................................................... 4      1   FF2
....................agagagcuauccgucgacaguaU........................................................... 3      1   FF2
.....................gagagcuauccgucgacagu............................................................. 6      0   FF2
.....................gagagcuauccgucgacagua............................................................ 3      0   FF2
.......................gagcuauccgucgacagu............................................................. 4      0   FF2
.......................gagcuauccgucgacagua............................................................ 2      0   FF2
.......................gagcuauccgucgacaguaU........................................................... 1      1   FF2
.........................gcuauccgucgacaguagggauauaauucacugu........................................... 1      0   FF2
........................................uagggauauaauucacu............................................. 1      0   FF2
........................................uagggauauaauucacugucaugga..................................... 1      0   FF2
.........................................agggauauaauucacugucauggaauu.................................. 1      0   FF2
..............................................uauaauucacugucauggaauugcucucu........................... 1      0   FF2
.....................................................cacugucauggaauugcucucuuua........................ 1      0   FF2
.......................................................cugucauggaauugcucuc............................ 2      0   FF2
.......................................................cugucauggaauugcucucu........................... 18     0   FF2
```

aga-miR-281*

aga-miR-281

```
gcaaucgaauaugaaaauaaagagagcuauccgucgacaguagggauauaauucacugucauggaauugcucucuuuauguacaauucgauauucaacgugc

.......................................................cugucauggaauugcucucuA..........................	1	1	FF2
.......................................................cugucauggaauugcucucuu..........................	15	0	FF2
.......................................................cugucauggaauugcucucCu..........................	1	1	FF2
.......................................................cugucauggaauugcucucuuA.........................	1	1	FF2
.......................................................cugucauggaauugcucucCuu.........................	3	1	FF2
.......................................................cugucauggaauugcucucuuu.........................	159	0	FF2
.......................................................cugucauAgaauugcucucuuua........................	1	1	FF2
.......................................................cugucauggaauugcucucuuuU........................	11	1	FF2
.......................................................cuAucauggaauugcucucuuua........................	1	1	FF2
.......................................................cugucauggaauugcucucuuua........................	188	0	FF2
.......................................................cugucauggaauugcucucuuAa........................	1	1	FF2
.......................................................cugucauggaauugcucucuuuaC.......................	5	1	FF2
.......................................................cugucauggaauugcucucuuuau.......................	77	0	FF2
.......................................................cugucauggaauugcucucuuuaA.......................	29	1	FF2
.......................................................cugucauggaauugcucucuuuauA......................	1	1	FF2
........................................................ugucauggaauugcucuc............................	3	0	FF2
........................................................ugucauggaauugcucucu...........................	5	0	FF2
........................................................ugucauggaauugcucucuu..........................	12	0	FF2
........................................................ugucauggaaCugcucucuuu.........................	1	1	FF2
........................................................ugucauggaauugcucucuuu.........................	162	0	FF2
........................................................ugucauggaauugcucucCuu.........................	4	1	FF2
........................................................ugucauggaauugcucCcuuua........................	1	1	FF2
........................................................ugucaugUaauugcucucuuua........................	1	1	FF2
........................................................ugucauggaauugcucucuuua........................	344	0	FF2
........................................................ugucauggaauugcucucuuuU........................	15	1	FF2
........................................................ugucauggaauugcucucuuuaG.......................	3	1	FF2
........................................................ugucauggaauugcucucuuuau.......................	283	0	FF2
........................................................ugucauggaauuAcucucuuuau.......................	1	1	FF2
........................................................ugucauggaauugcucucuuuaC.......................	84	1	FF2
........................................................ugucauggaauugcucucuuuaA.......................	102	1	FF2
........................................................ugucauggaauugcucucuuuauU......................	12	1	FF2
........................................................ugucauggaauugcucucuuuauA......................	2	1	FF2
........................................................ugucauggaauugcucucuuuauAu.....................	3	1	FF2
........................................................ugucauggaauugcucucuuuauUu.....................	1	1	FF2
.........................................................gucauggaauugcucucuuuaC.......................	1	1	FF2
.........................................................gucauggaauugcucucuuuau.......................	5	0	FF2

................auCaagagagcuauccgucgacagu.............................................................	1	1	MF2
.................uaaagagagcuauccgucgac................................................................	1	0	MF2
.................uaaagagagcuauccgucgaca...............................................................	11	0	MF2
.................Caaagagagcuauccgucgacag..............................................................	2	1	MF2
.................uaaagagagcuauccgucgacag..............................................................	4	0	MF2
.................uCaagagagcuauccgucgacagu.............................................................	10	1	MF2
.................uaaagagagcuauccgucgacagu.............................................................	3	0	MF2
.................Caaagagagcuauccgucgacagu.............................................................	5	1	MF2
..................aaagagagcuauccgucg..................................................................	1	0	MF2
..................aaagagagcuauccgucga.................................................................	7	0	MF2
..................Uaagagagcuauccgucgac................................................................	1	1	MF2
..................aaagagagcuauccgucgaU................................................................	3	1	MF2
..................aaagagagcuauccgucgac................................................................	172	0	MF2
..................aaaAagagcuauccgucgaca...............................................................	1	1	MF2
..................aaagagagcuauccguUgaca...............................................................	1	1	MF2
..................aaagagagcuauccgucAaca...............................................................	1	1	MF2
..................aaagagagcuaAccgucgaca...............................................................	1	1	MF2
..................aaagagagcuaucUgucgaca...............................................................	1	1	MF2
..................Caagagagcuauccgucgaca...............................................................	3	1	MF2
..................aaagagagcuauAcgucgaca...............................................................	1	1	MF2
..................aaagagagcuauccguGgaca...............................................................	2	1	MF2
..................aaagGgagcuauccgucgaca...............................................................	1	1	MF2
..................aaagagagcuauccgucgacG...............................................................	3	1	MF2
..................aaagagagcuauccgucgacU...............................................................	57	1	MF2
..................aaagagagUuauccgucgaca...............................................................	1	1	MF2
..................aaagagagcuauUcgucgaca...............................................................	4	1	MF2
..................aaagagagcuaucGgucgaca...............................................................	1	1	MF2
..................aaagagagcuauccgucgaca...............................................................	1664	0	MF2
..................aaGgagagcuauccgucgaca...............................................................	1	1	MF2
..................aaagagagcCauccgucgacag..............................................................	1	1	MF2
..................aaagagagcuauccguGgacag..............................................................	3	1	MF2
..................aaagaAagcuauccgucgacag..............................................................	1	1	MF2
```

```
                                                          aga-miR-281*
                   aga-miR-281
gcaaucgaauaugaaaauaaagagagcuauccgucgacaguagggauauaauucacugucauggaauugcucuuuauguacaauucgauauucaacgugc

..................aaagagagcuauccguUgacag............................................................ 2     1     MF2
..................aaagagagcuauccguAgacag............................................................ 1     1     MF2
..................aaagagagcGauccgucgacag............................................................ 1     1     MF2
..................Caagagagcuauccgucgacag............................................................ 11    1     MF2
..................aaagagagcuauccgucgacGg............................................................ 6     1     MF2
..................aaagagagcuauAcgucgacag............................................................ 1     1     MF2
..................aaagGgagcuauccgucgacag............................................................ 1     1     MF2
..................aaagagagcuCuccgucgacag............................................................ 1     1     MF2
..................aaagagagcuaucUgucgacag............................................................ 2     1     MF2
..................aaagagagcuauUcgucgacag............................................................ 3     1     MF2
..................aaagagagcuaucAgucgacag............................................................ 1     1     MF2
..................aaagagagcuauccgucgacaA............................................................ 34    1     MF2
..................Uaagagagcuauccgucgacag............................................................ 1     1     MF2
..................aaagagagcuauccgucAacag............................................................ 2     1     MF2
..................aaagagagcuauccgucgacaU............................................................ 57    1     MF2
..................aaagagagcuauccgucgacCg............................................................ 1     1     MF2
..................aaagagagcuauccgucgacaC............................................................ 23    1     MF2
..................aaagagagcuauccgAcgacag............................................................ 2     1     MF2
..................aaagagagcuauccgucgacag............................................................ 4368  0     MF2
..................aaagagagcuauccgCcgacag............................................................ 2     1     MF2
..................aaagagagAuauccgucgacag............................................................ 1     1     MF2
..................aaGgagagcuauccgucgacag............................................................ 3     1     MF2
..................aaagagagcAauccgucgacag............................................................ 1     1     MF2
..................aaaAagagcuauccgucgacag............................................................ 1     1     MF2
..................aaagagaAcuauccgucgacag............................................................ 1     1     MF2
..................aaagagagcuaCccgucgacag............................................................ 4     1     MF2
..................aaagagagcuauccgucgaUag............................................................ 1     1     MF2
..................aaagagagcuaCccgucgacagu........................................................... 4     1     MF2
..................aGagagagcuauccgucgacagu........................................................... 1     1     MF2
..................aUagagagcuauccgucgacagu........................................................... 1     1     MF2
..................aaagagagcuaucUgucgacagu........................................................... 2     1     MF2
..................aaagagagcuauccgucCacagu........................................................... 1     1     MF2
..................aaagagagcGauccgucgacagu........................................................... 1     1     MF2
..................aaagagaCcuauccgucgacagu........................................................... 1     1     MF2
..................aaagagagcuauccgucgGcagu........................................................... 1     1     MF2
..................Gaagagagcuauccgucgacagu........................................................... 8     1     MF2
..................aaaUagagcuauccgucgacagu........................................................... 1     1     MF2
..................Uaagagagcuauccgucgacagu........................................................... 3     1     MF2
..................aaagagagcuauccgucgacagC........................................................... 44    1     MF2
..................aaagagagcuauccUucgacagu........................................................... 1     1     MF2
..................aaagaAagcuauccgucgacagu........................................................... 3     1     MF2
..................aaaCagagcuauccgucgacagu........................................................... 1     1     MF2
..................aaagagagcuauccgucgacagA........................................................... 36    1     MF2
..................aaagagagcuauccgucAacagu........................................................... 7     1     MF2
..................aaCgagagcuauccgucgacagu........................................................... 1     1     MF2
..................aaagagagcuCuccgucgacagu........................................................... 1     1     MF2
..................aaagCgagcuauccgucgacagu........................................................... 1     1     MF2
..................aaagagagcuauccgucgacagG........................................................... 13    1     MF2
..................aaagagagcCauccgucgacagu........................................................... 2     1     MF2
..................aaaAagagcuauccgucgacagu........................................................... 2     1     MF2
..................aaagagagcuauccgCcgacagu........................................................... 5     1     MF2
..................aaUgagagcuauccgucgacagu........................................................... 1     1     MF2
..................aaagagagcuGuccgucgacagu........................................................... 1     1     MF2
..................Caagagagcuauccgucgacagu........................................................... 147   1     MF2
..................aaagagUgcuauccgucgacagu........................................................... 1     1     MF2
..................aaagagagcuauccguAgacagu........................................................... 2     1     MF2
..................aaagagagcAauccgucgacagu........................................................... 2     1     MF2
..................aaagagagcuaGccgucgacagu........................................................... 1     1     MF2
..................aaagagagcuauccgucgacagu........................................................... 8540  0     MF2
..................aaagagagcuauAcgucgacagu........................................................... 2     1     MF2
..................aaagagagcuauGcgucgacagu........................................................... 1     1     MF2
..................aaagagagcuauccgucgUcagu........................................................... 1     1     MF2
..................aaagagagUuauccgucgacagu........................................................... 5     1     MF2
..................aaagagagcuauccgucgacaUu........................................................... 8     1     MF2
..................aaagagagcuauccgucgacGgu........................................................... 2     1     MF2
..................aaagagaAcuauccgucgacagu........................................................... 3     1     MF2
..................aaagagagcuauUcgucgacagu........................................................... 7     1     MF2
..................aaagagagcuauccguUgacagu........................................................... 4     1     MF2
..................aaagagagcuauccgucgacCgu........................................................... 1     1     MF2
```

```
                                                       aga-miR-281*
                  aga-miR-281
gcaaucgaauaugaaaauaaagagagcuauccgucgacaguagggauauaauucacugucauggaauugcucucuuuauguacaauucgauauucaacgugc

..................aaagagGgcuauccgucgacagu.............................................................    2      1      MF2
..................aaagagagAuauccgucgacagu.............................................................    2      1      MF2
..................aaagagagcuauccgucgacaCu.............................................................    2      1      MF2
..................aaagagagcuauccgucgaUagu.............................................................    1      1      MF2
..................aaagagagcuauccguGgacagu.............................................................    2      1      MF2
..................aaagagagcuaAccgucgacagu.............................................................    2      1      MF2
..................aaagagagcuauccCucgacagu.............................................................    1      1      MF2
..................aaagGgagcuauccgucgacagu.............................................................    1      1      MF2
..................aaagagagcuauccgGcgacagu.............................................................    1      1      MF2
..................aaagaUagcuauccgucgacagu.............................................................    2      1      MF2
..................aaagagagcuauccAucgacagu.............................................................    1      1      MF2
..................aaGgagagcuauccgucgacagu.............................................................    1      1      MF2
..................aaagagagcuauccgucgacaAu.............................................................    9      1      MF2
..................aaagagagcuauccgAcgacagu.............................................................    2      1      MF2
..................aCagagagcuauccgucgacagu.............................................................    2      1      MF2
..................aaagagagcuauccgucgacaguU............................................................    399    1      MF2
..................aUagagagcuauccgucgacagua............................................................    1      1      MF2
..................aaagagagcuauccgucgacagua............................................................    337    0      MF2
..................aCagagagcuauccgucgacagua............................................................    1      1      MF2
..................aaagaAagcuauccgucgacagua............................................................    1      1      MF2
..................aaagagagcuauccgucgacagAa............................................................    5      1      MF2
..................Caagagagcuauccgucgacagua............................................................    79     1      MF2
..................aaagagagcuauccgucgacaguC............................................................    3      1      MF2
..................aaagagagcuauccgucCacagua............................................................    1      1      MF2
..................aaagagagcuauccgucgacaguaU...........................................................    77     1      MF2
..................aaagagagcuauccgucgacaguaC...........................................................    7      1      MF2
..................aaagagagcuauccgucgacaguaA...........................................................    111    1      MF2
..................aaagagagcuauccgucgacaguagg..........................................................    1      0      MF2
..................aaagagagcuauccgucgacaguaggg.........................................................    2      0      MF2
..................aaagagagcuauccgucgacaguaggga........................................................    5      0      MF2
..................aaagagagcuauccgucgacaguagggau.......................................................    1      0      MF2
..................aaagagagcuauccgucgacaguagggauauaauu.................................................    1      0      MF2
..................aaagagagcuauccgucgacaguagggauauaauuc................................................    1      0      MF2
..................aaagagagcuauccgCcgacaguagggauauaauucacu.............................................    1      1      MF2
..................aaagagagcuauccgucgacaguagggauauaauucacu.............................................    9      0      MF2
...................aagagagcuauccgucga.................................................................    33     0      MF2
...................aagagagcuauccAucgac................................................................    1      1      MF2
...................aagagagcuauccgucgaA................................................................    1      1      MF2
...................aagagagcuauccgucgac................................................................    397    0      MF2
...................aagagagcuaCccgucgac................................................................    1      1      MF2
...................aagagagcuauAcgucgac................................................................    1      1      MF2
...................aagagagcuauccgucgaU................................................................    8      1      MF2
...................aagagagcuauccgucgCca...............................................................    1      1      MF2
...................aagagagcuauccgucCaca...............................................................    1      1      MF2
...................aagagagcuauccgucgacG...............................................................    5      1      MF2
...................aagGgagcuauccgucgaca...............................................................    1      1      MF2
...................aagagagcuauccgucgacU...............................................................    39     1      MF2
...................aagagagcuaGccgucgaca...............................................................    2      1      MF2
...................aagagagcuUuccgucgaca...............................................................    1      1      MF2
...................Gagagagcuauccgucgaca...............................................................    3      1      MF2
...................aagagagcuauccgucgaAa...............................................................    1      1      MF2
...................aagagagcuaCccgucgaca...............................................................    2      1      MF2
...................aaAagagcuauccgucgaca...............................................................    1      1      MF2
...................aagagagcuauccgucgaca...............................................................    5216   0      MF2
...................aagagagcuauAcgucgaca...............................................................    2      1      MF2
...................aagagagcuauccguUgaca...............................................................    1      1      MF2
...................aaUagagcuauccgucgaca...............................................................    1      1      MF2
...................aagagagcuauccUucgaca...............................................................    2      1      MF2
...................aagagaAcuauccgucgaca...............................................................    4      1      MF2
...................aagagagcuauccgucgacC...............................................................    3      1      MF2
...................aagagagcuauUcgucgaca...............................................................    8      1      MF2
...................aagagagcuauGcgucgaca...............................................................    3      1      MF2
...................aagagagcuauccguAgaca...............................................................    4      1      MF2
...................Uagagagcuauccgucgaca...............................................................    1      1      MF2
...................aagagagcuaucUgucgaca...............................................................    1      1      MF2
...................aagagagcCauccgucgaca...............................................................    2      1      MF2
...................aagagaUcuauccgucgaca...............................................................    1      1      MF2
...................aagagagcuauccgucgGca...............................................................    1      1      MF2
...................aagagagcuauccgucAaca...............................................................    7      1      MF2
```

```
                                                    aga-miR-281*
                   aga-miR-281
gcaaucgaauaugaaaauaaagagagcuauccgucgacaguagggauauaauucacugucauggaauugcucuuuauguacaauucgauauucaacgugc

...................aagagagcuauccAucgaca.............................................................  2      1  MF2
...................aagaCagcuauccgucgaca.............................................................  1      1  MF2
...................aagaAagcuauccgucgaca.............................................................  1      1  MF2
...................aagagagcuauccgGcgaca.............................................................  2      1  MF2
...................aagagagcuauccgucgaGag............................................................  1      1  MF2
...................aagagagcuauccgucgacaC............................................................  7      1  MF2
...................aagagagcuauccgucgacag............................................................  20726  0  MF2
...................aagagagcuauccgucUacag............................................................  6      1  MF2
...................aagagagcuaAccgucgacag............................................................  1      1  MF2
...................aagaCagcuauccgucgacag............................................................  1      1  MF2
...................aagagCgcuauccgucgacag............................................................  1      1  MF2
...................aagagagcuauAcgucgacag............................................................  20     1  MF2
...................aagagagcuGuccgucgacag............................................................  2      1  MF2
...................aagagagcuauccguUgacag............................................................  9      1  MF2
...................aaUagagcuauccgucgacag............................................................  1      1  MF2
...................aagagagcuaucUgucgacag............................................................  4      1  MF2
...................aCgagagcuauccgucgacag............................................................  1      1  MF2
...................aagagagcuauccgucgaUag............................................................  11     1  MF2
...................aagagagcuauccgAcgacag............................................................  3      1  MF2
...................aagagagcuauccgucgacGg............................................................  15     1  MF2
...................aagagagcuauccgCcgacag............................................................  3      1  MF2
...................aagagagcuauUcgucgacag............................................................  22     1  MF2
...................aagagaAcuauccgucgacag............................................................  6      1  MF2
...................Cagagagcuauccgucgacag............................................................  1      1  MF2
...................aagagagcuauccgGcgacag............................................................  1      1  MF2
...................aagagagcuauccgucgacCg............................................................  2      1  MF2
...................Uagagagcuauccgucgacag............................................................  1      1  MF2
...................aagagagcuauccguAgacag............................................................  5      1  MF2
...................aaAagagcuauccgucgacag............................................................  5      1  MF2
...................aagagagcuauccAucgacag............................................................  9      1  MF2
...................aagagagcuauccguGgacag............................................................  1      1  MF2
...................aagagagcuauccgucCacag............................................................  3      1  MF2
...................aagagagUuauccgucgacag............................................................  4      1  MF2
...................aagaAagcuauccgucgacag............................................................  4      1  MF2
...................aagagagcuauccUucgacag............................................................  1      1  MF2
...................aagGgagcuauccgucgacag............................................................  7      1  MF2
...................aagagagcuauccgucgacaA............................................................  109    1  MF2
...................Gagagagcuauccgucgacag............................................................  6      1  MF2
...................aagagagcuaucAgucgacag............................................................  1      1  MF2
...................aUgagagcuauccgucgacag............................................................  4      1  MF2
...................aagagagcCauccgucgacag............................................................  8      1  MF2
...................aagUgagcuauccgucgacag............................................................  2      1  MF2
...................aagagagcuauccgucgaAag............................................................  3      1  MF2
...................aagagagcuauccgucAacag............................................................  13     1  MF2
...................aagagagcuauccgucgacaU............................................................  37     1  MF2
...................aagagagcuauGcgucgacag............................................................  7      1  MF2
...................aagagagcuUuccgucgacag............................................................  1      1  MF2
...................aagagaUcuauccgucgacag............................................................  1      1  MF2
...................aGgagagcuauccgucgacag............................................................  3      1  MF2
...................aagagagcuaCccgucgacag............................................................  14     1  MF2
...................aagagGgcuauccgucgacag............................................................  4      1  MF2
...................aagagagcuaucGgucgacag............................................................  1      1  MF2
...................aagagagcuauccgucgacUg............................................................  1      1  MF2
...................aagagagcuauccgucgGcag............................................................  1      1  MF2
...................aagagagcuauccgucgCcag............................................................  1      1  MF2
...................aaAagagcuauccgucgacagu...........................................................  37     1  MF2
...................aagagagcuauccgucAacagu...........................................................  151    1  MF2
...................aagagagcuauccgucgUcagu...........................................................  13     1  MF2
...................aagaAagcuauccgucgacagu...........................................................  43     1  MF2
...................aagagagcuUuccgucgacagu...........................................................  17     1  MF2
...................aagagagcuauUcgucgacagu...........................................................  229    1  MF2
...................aagagagcGauccgucgacagu...........................................................  1      1  MF2
...................aagagagcuauccgucgacGgu...........................................................  36     1  MF2
...................aUgagagcuauccgucgacagu...........................................................  15     1  MF2
...................aagaUagcuauccgucgacagu...........................................................  26     1  MF2
...................aaCagagcuauccgucgacagu...........................................................  2      1  MF2
...................aCgagagcuauccgucgacagu...........................................................  3      1  MF2
...................aagagagcuauccgucgacaAu...........................................................  44     1  MF2
...................aagagagUuauccgucgacagu...........................................................  42     1  MF2
```

aga-miR-281*

aga-miR-281

gcaaucgaauaugaaaauaaagagagcuauccgucgacaguagggauauaauucacugucauggaauugcucucuuuauguacaauucgauauucaacgugc

...................aagagagcuauccgucCacagu...	40	1	MF2
...................aagagagcuauccgucgaGagu...	6	1	MF2
...................aagagUgcuauccgucgacagu...	10	1	MF2
...................Cagagagcuauccgucgacagu...	19	1	MF2
...................aagagagcuauccCucgacagu...	10	1	MF2
...................aagagagcuGuccgucgacagu...	23	1	MF2
...................Gagagagcuauccgucgacagu...	48	1	MF2
...................aagUgagcuauccgucgacagu...	13	1	MF2
...................aagagagcuauGcgucgacagu...	117	1	MF2
...................aagagagcuaucAgucgacagu...	38	1	MF2
...................aagagagcuauccgucgacagC...	163	1	MF2
...................aagagagcuauccgucUacagu...	23	1	MF2
...................aagGgagcuauccgucgacagu...	35	1	MF2
...................aagagaAcuauccgucgacagu...	58	1	MF2
...................aagagagcuauccgucgacagA...	112	1	MF2
...................aagagagcuauccgucgacCgu...	7	1	MF2
...................aagagCgcuauccgucgacagu...	7	1	MF2
...................aagagagcuaCccgucgacagu...	98	1	MF2
...................aGgagagcuauccgucgacagu...	26	1	MF2
...................aagagagcAauccgucgacagu...	7	1	MF2
...................aagagagcuauccgucgaAagu...	17	1	MF2
...................aagagagcuauccgucgacagu...	227629	0	MF2
...................aagagagcuaAccgucgacagu...	25	1	MF2
...................Uagagagcuauccgucgacagu...	16	1	MF2
...................aagagagcuauccguUgacagu...	99	1	MF2
...................aagagagcuaucGgucgacagu...	11	1	MF2
...................aagagagcuauccUucgacagu...	17	1	MF2
...................aagagagcuauccAucgacagu...	87	1	MF2
...................aagagagcuauccguAgacagu...	39	1	MF2
...................aagagagcCauccgucgacagu...	64	1	MF2
...................aagagagcuauccgucgCcagu...	8	1	MF2
...................aagagagcuauccgucgGcagu...	36	1	MF2
...................aagagagcuaucUgucgacagu...	71	1	MF2
...................aagaCagcuauccgucgacagu...	14	1	MF2
...................aaUagagcuauccgucgacagu...	17	1	MF2
...................aagagagcuauAcgucgacagu...	250	1	MF2
...................aagagagcuauccgCcgacagu...	85	1	MF2
...................aagagagcuauccgucgacagG...	530	1	MF2
...................aagagagcuauccgGcgacagu...	20	1	MF2
...................aagagagcuauccgucgacaUu...	12	1	MF2
...................aagagagGuauccgucgacagu...	4	1	MF2
...................aagagagcuauccgucgacaCu...	8	1	MF2
...................aagagaUcuauccgucgacagu...	19	1	MF2
...................aagagagcuauccgucgaUagu...	80	1	MF2
...................aagagagAuauccgucgacagu...	38	1	MF2
...................aagagGgcuauccgucgacagu...	45	1	MF2
...................aagagagcuauccguGgacagu...	19	1	MF2
...................aagagagcuauccgAcgacagu...	19	1	MF2
...................aagagagcuauccgucgacUgu...	14	1	MF2
...................aagagagcuaGccgucgacagu...	9	1	MF2
...................aagagagcuCuccgucgacagu...	15	1	MF2
...................aagCgagcuauccgucgacagu...	4	1	MF2
...................aagagaCcuauccgucgacagu...	5	1	MF2
...................aagagagcuauccguAgacagua..	15	1	MF2
...................aagagagcuCuccgucgacagua..	8	1	MF2
...................aagagagcuaCccgucgacagua..	50	1	MF2
...................aagagagUuauccgucgacagua..	22	1	MF2
...................aagagagcuauccgucgaGagua..	2	1	MF2
...................aagagagcuauccgucgaAagua..	5	1	MF2
...................aagaAagcuauccgucgacagua..	21	1	MF2
...................aagagagcuaGccgucgacagua..	6	1	MF2
...................aagagagcuauAcgucgacagua..	110	1	MF2
...................aagagaAcuauccgucgacagua..	25	1	MF2
...................aagagagcuauccguGgacagua..	11	1	MF2
...................aagagagcuauccCucgacagua..	2	1	MF2
...................aagagagcuauccgucgacCgua..	4	1	MF2
...................aagagGgcuauccgucgacagua..	10	1	MF2
...................Gagagagcuauccgucgacagua..	21	1	MF2
...................aagagagcuauccgucgacagGa..	133	1	MF2

```
                                                        aga-miR-281*
                   aga-miR-281
gcaaucgaauaugaaaauaaagagagcuauccgucgacaguagggauauaauucacugucauggaauugcucuuuauguacaauucgauauucaacgugc

...................aagagagcuauccgucgacaguU..........................................................   4922     1   MF2
...................aagagagcuauccgucgacaguC..........................................................   49       1   MF2
...................aagUgagcuauccgucgacagua..........................................................   3        1   MF2
...................aagagagcuauccgucgacGgua..........................................................   11       1   MF2
...................aagagagcuauccgucCacagua..........................................................   18       1   MF2
...................aagagagcuauUcgucgacagua..........................................................   111      1   MF2
...................aagGgagcuauccgucgacagua..........................................................   14       1   MF2
...................aagagagcuauccgucgacUgua..........................................................   6        1   MF2
...................aaCagagcuauccgucgacagua..........................................................   3        1   MF2
...................aagagagcuauccgucUacagua..........................................................   8        1   MF2
...................aagagagcuauccgAcgacagua..........................................................   13       1   MF2
...................Cagagagcuauccgucgacagua..........................................................   4        1   MF2
...................aagagagcuauccgGcgacagua..........................................................   4        1   MF2
...................aUgagagcuauccgucgacagua..........................................................   10       1   MF2
...................aCgagagcuauccgucgacagua..........................................................   4        1   MF2
...................aagagagcuauccgucgacaCua..........................................................   10       1   MF2
...................aagagagGuauccgucgacagua..........................................................   2        1   MF2
...................aagagaUcuauccgucgacagua..........................................................   9        1   MF2
...................aagagUgcuauccgucgacagua..........................................................   6        1   MF2
...................aagagCgcuauccgucgacagua..........................................................   4        1   MF2
...................aagaUagcuauccgucgacagua..........................................................   13       1   MF2
...................aagagagcuauccguUgacagua..........................................................   41       1   MF2
...................aGgagagcuauccgucgacagua..........................................................   14       1   MF2
...................aagagagcuauGcgucgacagua..........................................................   59       1   MF2
...................aagagagcuauccgucgaUagua..........................................................   42       1   MF2
...................aagagagcuauccgucgCcagua..........................................................   3        1   MF2
...................aagagagcuUuccgucgacagua..........................................................   8        1   MF2
...................aagagagcuaucUgucgacagua..........................................................   39       1   MF2
...................aagagagcuauccgucgGcagua..........................................................   9        1   MF2
...................aagagagcuauccgucgacaUua..........................................................   6        1   MF2
...................aagagagcuaAccgucgacagua..........................................................   17       1   MF2
...................aagagagcuaucAgucgacagua..........................................................   21       1   MF2
...................aagagagcuauccgucgacagCa..........................................................   100      1   MF2
...................aagagaCcuauccgucgacagua..........................................................   2        1   MF2
...................aagagagcCauccgucgacagua..........................................................   28       1   MF2
...................aagaCagcuauccgucgacagua..........................................................   3        1   MF2
...................aagagagcuauccgucgacaAua..........................................................   25       1   MF2
...................aagagagcuauccgucgacagAa..........................................................   51       1   MF2
...................aagCgagcuauccgucgacagua..........................................................   2        1   MF2
...................aagagagcuauccgucgacaguG..........................................................   58       1   MF2
...................aagagagcuaucGgucgacagua..........................................................   4        1   MF2
...................aagagagcuauccgucgUcagua..........................................................   9        1   MF2
...................aagagagcuauccAucgacagua..........................................................   55       1   MF2
...................aagagagcuauccgCcgacagua..........................................................   42       1   MF2
...................Uagagagcuauccgucgacagua..........................................................   8        1   MF2
...................aagagagcuGuccgucgacagua..........................................................   17       1   MF2
...................aagagagcuauccgucgacagua..........................................................   112519   0   MF2
...................aaUagagcuauccgucgacagua..........................................................   7        1   MF2
...................aagagagcuauccUucgacagua..........................................................   8        1   MF2
...................aagagagcuauccgucAacagua..........................................................   62       1   MF2
...................aaAagagcuauccgucgacagua..........................................................   22       1   MF2
...................aagagagAuauccgucgacagua..........................................................   15       1   MF2
...................aagagagcuauccgucgacaguUg.........................................................   3        1   MF2
...................aagagagcuauccgucgacaguaA.........................................................   30445    1   MF2
...................aagagagcuauccgucgacaguCg.........................................................   2        1   MF2
...................aagagagcuauccgucgacaguaC.........................................................   1116     1   MF2
...................aagagagcuauccgucgacaguaU.........................................................   15519    1   MF2
...................aagagagcuauccgucgacaguag.........................................................   23       0   MF2
...................aagagagcuauccgucgacaguagg........................................................   9        0   MF2
...................aagagagcuauccgucgacaguagA........................................................   4        1   MF2
...................aagagagcuauccgucgacaguaAg........................................................   11       1   MF2
...................aagagagcuauccgucgacaguUgg........................................................   2        1   MF2
...................aagagagcuauccgucgacaguaUg........................................................   8        1   MF2
...................aagagagcuauccgucgacaguagU........................................................   6        1   MF2
...................aagagagcuauccgucgacaguaggA.......................................................   1        1   MF2
...................aagagagcuauccgucgacaAuaggg.......................................................   1        1   MF2
...................aagagagcuauccgucgacaguaggg.......................................................   10       0   MF2
...................aagagagcuauccgucgacaguaggga......................................................   4        0   MF2
...................aagagagcuauccgucgacaguagggau.....................................................   26       0   MF2
```

```
                                                       aga-miR-281*
                   aga-miR-281
gcaaucgaauaugaaaauaaagagagcuauccgucgacaguagggauauaauucacugucauggaauugcucucuuuauguacaauucgauauucaacgugc

...................aagagagcuauccgucUacaguagggau.......................................................  1     1     MF2
...................aagagagcuauccgucgacaguagggUu.......................................................  1     1     MF2
...................aagagagcuauccgucgacaguagggaua......................................................  7     0     MF2
...................aagagagcuauccgucgacaguagggauaua....................................................  1     0     MF2
...................aagagagcuauccgucgacaguagggauauaUu..................................................  2     1     MF2
...................aagagagcuauccgucgacaguagggauauaau..................................................  3     0     MF2
...................aagagagcuauccgucgacaguagggauauaauu.................................................  4     0     MF2
...................aagagagcuauccgucgacaguagggauauaauuc................................................  28    0     MF2
...................aagagagcuauccgucgacaguagggauauaauuca...............................................  17    0     MF2
...................aagagagcuauccgucgacaguagggauauaauucacu.............................................  9     0     MF2
...................aagagagcuauccgucgacaguagggauauaauucacugu...........................................  12    0     MF2
...................aagagagcuauccgucgacaguagggauauaauucacugucaug.......................................  1     0     MF2
....................agagagcuauccgucgac................................................................  3     0     MF2
....................agagagcuauccgucgaca...............................................................  4     0     MF2
....................agagagcuauccgucgacU...............................................................  1     1     MF2
....................agagagcuauccgucgacag..............................................................  13    0     MF2
....................agagagcuauccgucgacagu.............................................................  210   0     MF2
....................agagagcuauccgucgacagA.............................................................  1     1     MF2
....................agagagcuauccgucgacaguU............................................................  7     1     MF2
....................agagagcuauccgucgacagua............................................................  234   0     MF2
....................agagagcuauccgGcgacagua............................................................  1     1     MF2
....................agagagcuauccgucgacaguaU...........................................................  18    1     MF2
....................agagagcuauccgucgacaguaA...........................................................  40    1     MF2
....................agagagcuauccgucgacaguagUgau.......................................................  1     1     MF2
.....................gagagcuauccgucgacag..............................................................  1     0     MF2
.....................gagagcuauccgucgacagu.............................................................  21    0     MF2
.....................Aagagcuauccgucgacagu.............................................................  1     1     MF2
.....................gagagcuauccgucgacagua............................................................  8     0     MF2
.....................gagagcuauccgucgacaguagggauauaauu.................................................  1     0     MF2
.....................gagagcuauccgucgacaguagggauauaauucacu.............................................  2     0     MF2
.....................gagagcuauccgucgacaguagggauauaauucacugu...........................................  2     0     MF2
......................agagcuauccgucgacagu.............................................................  4     0     MF2
......................agagcuauccgucgacaguagggauauaauu.................................................  1     0     MF2
......................agagcuauccgucgacaguagggauauaauucacu.............................................  3     0     MF2
......................agagcuauccgucgacaguagggauauaauucacugu...........................................  3     0     MF2
.......................gagcuauccgucgacag..............................................................  2     0     MF2
.......................gagcuauccgucgacagu.............................................................  3     0     MF2
.......................gagcuauccgucgacagua............................................................  1     0     MF2
.......................gagcuauccgucgacaguaggga........................................................  1     0     MF2
.......................gagcuauccgucgacaguagggauauaauu.................................................  1     0     MF2
.......................gagcuauccgucgacaguagggauauaauucacu.............................................  7     0     MF2
........................agcuauccgucgacagu.............................................................  3     0     MF2
........................agcuauccgucgacagua............................................................  3     0     MF2
........................agcuauccgucgacaguaU...........................................................  1     1     MF2
........................agcuauccgucgacaguagggauauaauucacugu...........................................  1     0     MF2
.........................gcuauccgucgacagua............................................................  2     0     MF2
.........................gcuauccgucgacaguagggauauaauucacu.............................................  1     0     MF2
.........................gcuauccgucgacaguagggauauaauucacugu...........................................  1     0     MF2
.........................gcuauccgucgacaguagggauauaaCucacugu...........................................  1     1     MF2
..........................cuauccgucgacaguagggauauaauucacu.............................................  1     0     MF2
..........................cuauccgucgacaguagggauauaauucacugu...........................................  3     0     MF2
............................auccgucgacaguagggauauaauucacu.............................................  2     0     MF2
............................auccgucgacaguagggauauaauucacugu...........................................  1     0     MF2
..................................cgacaguagggauauaauucacu.............................................  1     0     MF2
..................................cgacaguagggauauaauucacugu...........................................  1     0     MF2
........................................uagggauauaauucacu.............................................  1     0     MF2
........................................uagggauauaauucacugucaug.......................................  1     0     MF2
........................................uagggauauaauucacugucauggaa....................................  2     0     MF2
........................................uagggauauaauucacugucauggaau...................................  1     0     MF2
.........................................agggauauaauucacugucauggaau...................................  1     0     MF2
...................................................uucacugucauggaauugcucucuuua........................  3     0     MF2
....................................................ucacugucauggaauugcucuc............................  3     0     MF2
....................................................ucacugucauggaauugcucucu...........................  1     0     MF2
....................................................ucacugucauggaauugcucucuu..........................  4     0     MF2
....................................................ucacugucauggaauugcucucuuu.........................  1     0     MF2
....................................................ucacugucauggaauugcucucuuua........................  1     0     MF2
....................................................ucacugucauggaauugcucucuuuU........................  2     1     MF2
....................................................ucacugucauggaauugcucucuuuau.......................  1     0     MF2
.....................................................cacugucauggaauugcucuc............................  1     0     MF2
```

```
                                                        aga-miR-281*
                   aga-miR-281
gcaauCgaauaugaaaauaaagagagcuauccgucgacaguagggauauaauucacugucauggaauugcucucuuuauguacaauucgauauucaacgugc

.....................................................cacugucauggaauugcucucu...........................    2      0      MF2
.....................................................cacugucauggaauugcucucuuu.........................    1      0      MF2
.....................................................cacugucauggaauugcucucuuuaC.......................    2      1      MF2
.....................................................cacugucauggaauugcucucuuuau.......................    3      0      MF2
......................................................acugucauggaauugcucucuuua........................    1      0      MF2
......................................................Ccugucauggaauugcucucuuua........................    2      1      MF2
......................................................acugucauggaauugcucucuuuau.......................    1      0      MF2
.......................................................cugucauggaauugcuc..............................    1      0      MF2
.......................................................cugucauggaauugcucu.............................    1      0      MF2
.......................................................cugucauggaauugcucuc............................    11     0      MF2
.......................................................cugucauggaauugcucucu...........................    99     0      MF2
.......................................................cugucauggaaAugcucucuu..........................    1      1      MF2
.......................................................cugucauggaauugcucucuu..........................    140    0      MF2
.......................................................cugCcauggaauugcucucuu..........................    1      1      MF2
.......................................................cugucauggaauugcuAucuuu.........................    2      1      MF2
.......................................................cugucauggaauugcucucuuu.........................    1543   0      MF2
.......................................................cugucauggaGuugcucucuuu.........................    1      1      MF2
.......................................................cugAcauggaauugcucucuuu.........................    1      1      MF2
.......................................................cugucauggaaCugcucucuuu.........................    1      1      MF2
.......................................................cugucauggaauugcuGucuuu.........................    2      1      MF2
.......................................................cugucauggaauugcuUucuuu.........................    1      1      MF2
.......................................................cugucauggaaGugcucucuuu.........................    3      1      MF2
.......................................................cugucauggGauugcucucuuu.........................    1      1      MF2
.......................................................cugucauggaUuugcucucuuu.........................    3      1      MF2
.......................................................cuguUauggaauugcucucuuu.........................    1      1      MF2
.......................................................cugucauggaauugcucucuuC.........................    3      1      MF2
.......................................................cugucauggCauugcucucuuu.........................    1      1      MF2
.......................................................cugucauggaCuugcucucuuu.........................    1      1      MF2
.......................................................cugucaCggaauugcucucuuu.........................    5      1      MF2
.......................................................cugucauggaauugcucucCuu.........................    29     1      MF2
.......................................................cuAucauggaauugcucucuuu.........................    1      1      MF2
.......................................................cugucauggaauugcucucCuua........................    3      1      MF2
.......................................................cugucauggGauugcucucuuua........................    1      1      MF2
.......................................................cugucauggaauugcucuUuua........................    1      1      MF2
.......................................................cugucauAgaauugcucucuuua........................    1      1      MF2
.......................................................cugucaCggaauugcucucuuua........................    1      1      MF2
.......................................................cugucauggCauugcucucuuua........................    1      1      MF2
.......................................................cugucauggaauugcuAucuuua........................    1      1      MF2
.......................................................cugCcauggaauugcucucuuua........................    1      1      MF2
.......................................................cugucauggaaCugcucucuuua........................    1      1      MF2
.......................................................cugucauggaauugAucucuuua........................    1      1      MF2
.......................................................cugucauggaauugcucucuuuU........................    150    1      MF2
.......................................................cugucaugAaauugcucucuuua........................    2      1      MF2
.......................................................cuAucauggaauugcucucuuua........................    1      1      MF2
.......................................................cugGcauggaauugcucucuuua........................    1      1      MF2
.......................................................cugucauggaauugcucucuuGa........................    1      1      MF2
.......................................................cugucauggaauugcucucuuCa........................    1      1      MF2
.......................................................cugucauggaauugcucucuuuC........................    3      1      MF2
.......................................................cugucauggaauugcucucuuua........................    1757   0      MF2
.......................................................cugucauggaaCugcucucuuuau.......................    1      1      MF2
.......................................................cugucauggaauugcucucuuuaA.......................    362    1      MF2
.......................................................cugucauggaauugcucucuuuUu.......................    15     1      MF2
.......................................................cugucauggaauugcuGucuuuau.......................    1      1      MF2
.......................................................cugucauggaauAgcucucuuuau.......................    1      1      MF2
.......................................................cugAcauggaauugcucucuuuau.......................    1      1      MF2
.......................................................cugucaCggaauugcucucuuuau.......................    1      1      MF2
.......................................................cugucauggaCuugcucucuuuau.......................    1      1      MF2
.......................................................cugucauggaauugcucucuuGau.......................    2      1      MF2
.......................................................cugucaugAaauugcucucuuuau.......................    3      1      MF2
.......................................................cuUucauggaauugcucucuuuau.......................    1      1      MF2
.......................................................cugucauggaauugcucCcuuuau.......................    1      1      MF2
.......................................................cugucauggGauugcucucuuuau.......................    1      1      MF2
.......................................................cugucauggaaGugcucucuuuau.......................    1      1      MF2
.......................................................cugucauggaauugcucucuuuau.......................    1148   0      MF2
.......................................................cuAucauggaauugcucucuuuau.......................    1      1      MF2
.......................................................cugucauggaauugcucucuuAau.......................    1      1      MF2
.......................................................cugucauggaGuugcucucuuuau.......................    2      1      MF2
```
[truncated: 1,566,921 more chars]
