# Supplementary material for: The germline of the malaria mosquito produces abundant miRNAs, endo-siRNAs, piRNAs and 29-nt small RNAs
Source: BMC Genomics. 2015 Feb 19;16(1):100. doi: 10.1186/s12864-015-1257-2 (PMC4345017; doi:10.1186/s12864-015-1257-2)
Supplement: Additional file 3: — On the top, sequence and predicted RNA secondary structure of annotated aga-mir-133 precursor. On the bottom sequence and predicted RNA secondary structure of aga-mir-133 precursor identified in this study. [file 12864_2015_1257_MOESM3_ESM.pdf]

Annotated aga-miR-133 precursor (http://www.mirbase.org/cgi-bin/mirna_entry.pl?acc=MI0001606)

UGUUGACGCAAUAUUUUCCCUUUGCGAAUGCAUUUGGUCCCCUUCAACCAGCUGUAGCAGUGAUUGCAAUCAACAGU

Annotated aga-miR-133 precursor Hairpin structure

```
UGUUGACGCAAUAUUUUCCCUUUGCGAA    UUUGGUCCCCU    ACCA  U
                            UGCA           UCA    GC G
                            ACGU           AGU    CG U
-------------------UGACAACUA    ----------U    --GA  A
```

aga-miR-133 precursor in our study

AGCUGGUUGACAUCGGGUCAAAUCGUAAUAUUGUUGACGCAAUAUUUUCCCUUUGCGAAUGCAUUUGGUCCCCUUCAACCAGCUGU

aga-miR-133 precursor hairpin structure in our study

```
             CAUC  GU    -----     --------|      U
   AGCUGGUUGA    GG  CAAA     UCGUA         AUAUUGU G
 UGUCGACCAACU    CC  GUUU     AGCGU         UAUAACG A
             UCC-  UG    ACGUA     UUCCCUUU^      C
```

Additional File 2
