# Supplementary material for: The germline of the malaria mosquito produces abundant miRNAs, endo-siRNAs, piRNAs and 29-nt small RNAs
Source: BMC Genomics. 2015 Feb 19;16(1):100. doi: 10.1186/s12864-015-1257-2 (PMC4345017; doi:10.1186/s12864-015-1257-2)
Supplement: Additional file 6: — Small RNA reads distribution and predicted RNA secondary structure of the novel, identified miRNAs in the analysed tissues. [file 12864_2015_1257_MOESM6_ESM.pdf]

miRBase precursor : aga-mir-10355
Total read count : 5218
aga-mir-10355 read count : 5148
aga-mir-10355-star read count : 70
remaining reads : 0

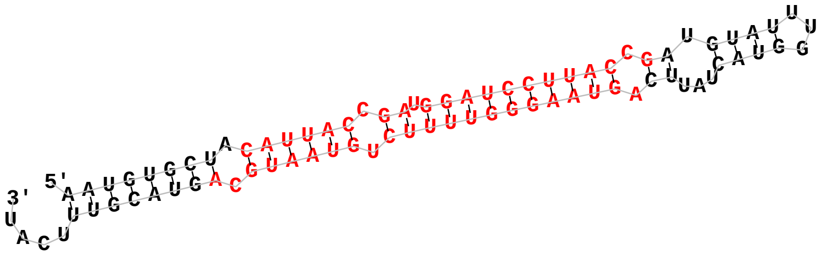

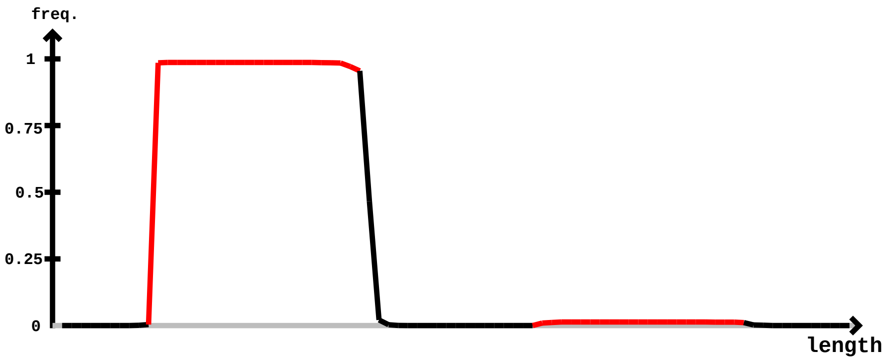

aga-mir-10355

aga-mir-10355-star

5'- aaugugcuacauuaccgauggauccuuaccgauguauuugguacuauucaguaaggguuuucuguaaugcaguacguuucau -3'	exp		
((((((((.((((((.((.((((((((((.((.((((...))))...)).)))))))))))).)))))).))))))))....	reads	mm	sample
.........cauuaccgauggauccuuaccg...	3	0	FF2
.........cauuaccgauggauccuuaccga..	1	0	FF2
....guaaggguuuucuguaaugca...........	1	0	FF2
.........cauuaccgauggauccuuac...	1	0	OV2
.........cauuaccgauggauccuuacc..	1	0	OV2
.........cauuaccgauggauccuuaccg...	20	0	OV2
.........cauuaccgauggauccuuaccga..	26	0	OV2
.........cauuaccgauggauccuuaccgau...	1	0	OV2
....aguaaggguuuucuguaaugc............	3	0	OV2
....aguaaggguuuucuguaaugca...........	4	0	OV2
....guaaggguuuucuguaaugca...........	1	0	OV2
....uaaggguuuucuguaaugcagC.........	1	1	OV2
....uaaggguuuucuguaaugcagu.........	1	0	OV2
.......uacauuaccgauggauccuuacc..	2	0	TE1
........acauuaccgauggauccuuac...	1	0	TE1
........acauuaccgauggauccuuaccg...	1	0	TE1
.........cauuaccgauggauccuua..	2	0	TE1
.........cauuaccgaCggauccuuac...	1	1	TE1
.........cauuaccgauggauccuuac...	19	0	TE1
.........cauuaccgauggauccuuacc..	20	0	TE1
.........cauuaGcgauggauccuuaccg...	1	1	TE1
.........cauuaccgauggauccuuaccg...	588	0	TE1
.........cauuaccgauggauccAuaccg...	1	1	TE1
.........cauuaccgauAgauccuuaccg...	1	1	TE1
.........cauuaccgauggauccuuaccA...	2	1	TE1
.........cauuaccgauggauccuuaccU...	1	1	TE1
.........cGuuaccgauggauccuuaccg...	1	1	TE1
.........cauuaccgauggauccuuacUg...	1	1	TE1
.........cauuaccgauggauccuuaUcg...	1	1	TE1
.........cauuaccgaCggauccuuaccg...	1	1	TE1
.........cauuaccgauggauccuuaccgG..	3	1	TE1
.........cauuaccgauggauccuuaccgU..	1	1	TE1

aga-mir-10355

aga-mir-10355-star

```
aaugugcuacauuaccgauggauccuuaccgauguauuugguacuauucaguaaggguuuucuguaaugcaguacguuucau

.........cauuaccgauggauccuuacGga..................................................   1     1     TE1
.........cauuaccgaCggauccuuaccga..................................................   1     1     TE1
.........cauuaccAauggauccuuaccga..................................................   1     1     TE1
.........cauuaccgauggauccuuaccAa..................................................   1     1     TE1
.........cauuaccgauggauccuuaccga..................................................   614   0     TE1
.........cauuaccgaAggauccuuaccga..................................................   2     1     TE1
.........cauuaccgauggauccCuaccga..................................................   1     1     TE1
.........cauuaccgauggauccuuaccgGu.................................................   1     1     TE1
.........cauuaccgauggauccuuaccgaA.................................................   21    1     TE1
.........cauuaccgauggauccuuaccgau.................................................   8     0     TE1
.........cauuaccgauggauccuuaccgauA................................................   3     1     TE1
.........cauuaccgauggauccuuaccgauAu...............................................   1     1     TE1
..........auuaccgauggauccuuaccg...................................................   2     0     TE1
.................................................aguaaggguuuucuguaaugca...........   1     0     TE1
...................................................uaaggguuuucuguaaugcagu.........   1     0     TE1

........acauuaccgauggauccuuaccg...................................................   2     0     MF2
.........cauuaccgauggauccuuac.....................................................   1     0     MF2
.........cauuaccgauggauccuuacc....................................................   3     0     MF2
.........cauuaccgauAgauccuuaccg...................................................   1     1     MF2
.........cauuaccgauggauccuuaccg...................................................   108   0     MF2
.........cauuacUgauggauccuuaccg...................................................   1     1     MF2
.........cauuGccgauggauccuuaccg...................................................   1     1     MF2
.........cauuaccgauggauccuuaccga..................................................   121   0     MF2
.........cauuaccgauggauccuuaccUa..................................................   1     1     MF2
.........cauuaccgauggauccuuaccgaA.................................................   1     1     MF2
.........cauuaccgauggauccuuaccgauA................................................   5     1     MF2
.................................................aguaaggguuuucuguaaugc............   2     0     MF2
.................................................aguaaggguuuucuguaaugca...........   5     0     MF2
.................................................Gguaaggguuuucuguaaugca...........   1     1     MF2
..................................................guaaggguuuucuguaaugca...........   1     0     MF2
...................................................uaaggguuuucuguaaugcag..........   2     0     MF2

.........cauuaccgauggauccuuaccg...................................................   1     0     FW2

.........cauuaccgauggauccuuac.....................................................   1     0     FF1
.........cauuaccgauggauccuuaccg...................................................   7     0     FF1
.........cauuaccgauggauccuuaccga..................................................   6     0     FF1
..................................................guaaggguuuucuguaaugca...........   1     0     FF1
..................................................guaaggguuuuGuguaaugca...........   1     1     FF1

.......uacauuaccgauggauccuuacc....................................................   1     0     OV1
.........cauuaccgauggauccuuacc....................................................   2     0     OV1
.........cauuaccgauggauccuuaccg...................................................   22    0     OV1
.........cauuaccgauggaucUuuaccga..................................................   1     1     OV1
.........cauuaccgauggauccuuaccga..................................................   29    0     OV1
.........cauuaccgauggauccuuaccgaA.................................................   3     1     OV1
.........cauuaccgauggauccuuaccgau.................................................   1     0     OV1
.................................................aguaaggguuuucuguaa...............   2     0     OV1
.................................................aguaaggguuuucuguaaugc............   1     0     OV1
.................................................aguaaggguuuucuguaaugca...........   8     0     OV1
..................................................guaaggguuuucuguaaugca...........   1     0     OV1

.........cauuaccgauggauccuuaccg...................................................   22    0     MF1
.........cauuaccgauggauccuuaccUa..................................................   1     1     MF1
.........cauuaccgauggauccuuacUga..................................................   1     1     MF1
.........cauuaccgauggauccuuaccga..................................................   18    0     MF1
.........cauuaccgauggauccuuaccgaA.................................................   1     1     MF1

.........cauuaccgauggauccuuac.....................................................   1     0     BF2
.........cauuaccgauggauccuuacc....................................................   1     0     BF2
.........cauuaccgauggauccuuaccg...................................................   3     0     BF2
.........cauuaccgauggauccuuaccga..................................................   8     0     BF2
.........cauuaccgauggauccuuaccAa..................................................   1     1     BF2
.........cauuaccgauggauccuuaccgaC.................................................   1     1     BF2
.................................................aguaaggguuuucuguaa...............   1     0     BF2
.................................................aguaaggguuuucuguaaugc............   1     0     BF2
.................................................aguaaggguuuucuguaaugca...........   4     0     BF2
..................................................guaaggguuuucuguaaugc............   1     0     BF2
```

aga-mir-10355

aga-mir-10355-star

aaugugcuacauuaccgauggauccuuaccgauguauuugguacuauucaguaaggguuuucuguaaugcaguacguuucau

....guaaggguuuucuguaaugca...........	1	0	BF2
....guaaggguuuucuguaaugcaU..........	1	1	BF2
....uaaggguuuucuguaaugcag..........	1	0	BF2
........acauuaccgauggauccuuacc..	1	0	BF1
.........cauuaccgauggauccuuacc..	1	0	BF1
.........cauuaccgauggauccuuaccg...	7	0	BF1
.........cauuaccgauggauccuuaccga..	16	0	BF1
....aguaaggguuuucuguaaugc............	1	0	BF1
....aguaaggguuuucuguaaugca...........	1	0	BF1
....guaaggguuuucuguaaugca...........	1	0	BF1
....guaaggguuuucuguaaugcaA..........	1	1	BF1
....uaaggguuuucuguaaugcagu.........	1	0	BF1
.........cauuaccgauggauccuuaccg...	1	0	MW1
.........cauuaccgauggauccuuaccga..	2	0	MW1
.........cauuaccgauggauccuuaccg...	4	0	MW2
.........cauuaccgauggauccuuaccga..	3	0	MW2
....aguaaggguuuucuguaaugca...........	1	0	MW2
.......uacauuaccgauggauccuuacc..	2	0	TE2
........acauuaccgauggauccuuacc..	1	0	TE2
........acauuaccgauggauccuuaccg...	9	0	TE2
.........cauuaccgauggauccu..	4	0	TE2
.........cauuaccgauggauccuu...	2	0	TE2
.........cauuaccgauggauccuua..	3	0	TE2
.........cauuaccgauggauccuuac...	44	0	TE2
.........cauuaccAauggauccuuacc..	1	1	TE2
.........cauuaccgauggauccuuacc..	49	0	TE2
.........cauuaccgauggauccuuaccU...	1	1	TE2
.........cauuacAgauggauccuuaccg...	1	1	TE2
.........cauuaccgauggauccuuaccA...	1	1	TE2
.........cauuaccgauggauUcuuaccg...	2	1	TE2
.........cauuaccgaGggauccuuaccg...	1	1	TE2
.........Aauuaccgauggauccuuaccg...	1	1	TE2
.........cauuaccgauggaCccuuaccg...	1	1	TE2
.........cauuacGgauggauccuuaccg...	1	1	TE2
.........caCuaccgauggauccuuaccg...	2	1	TE2
.........cauuaccgaugAauccuuaccg...	3	1	TE2
.........cGuuaccgauggauccuuaccg...	1	1	TE2
.........cauuaccgauggauccuuaccg...	1716	0	TE2
.........cauCaccgauggauccuuaccg...	1	1	TE2
.........cauuaccAauggauccuuaccg...	3	1	TE2
.........cauuaccgauAgauccuuaccga..	1	1	TE2
.........cGuuaccgauggauccuuaccga..	1	1	TE2
.........cauuaccgauggauccuuaccgG..	2	1	TE2
.........cauuaccAauggauccuuaccga..	1	1	TE2
.........cauuaccgauggauccCuaccga..	1	1	TE2
.........cauuaccgaugAauccuuaccga..	1	1	TE2
.........cauuacUgauggauccuuaccga..	1	1	TE2
.........cauuaccgauggauccuuaccAa..	2	1	TE2
.........cauuaUcgauggauccuuaccga..	1	1	TE2
.........cauuaccgauggauccuuaccga..	1443	0	TE2
.........cauuaccgauggaCccuuaccga..	3	1	TE2
.........cauCaccgauggauccuuaccga..	2	1	TE2
.........cauuaccgaugCauccuuaccga..	1	1	TE2
.........cauuaccgauggauccuuaccgau...	16	0	TE2
.........cauuaccgauggauccuuaccgaG...	1	1	TE2
.........cauuaccgauggauccuuaccgaA...	39	1	TE2
.........cauuaccgauggauccuuaccgaAg..	3	1	TE2
.........cauuaccgauggauccuuaccgaug..	1	0	TE2
.........cauuaccgauggauccuuaccgauA..	2	1	TE2
.........cauuaccgauggauccuuaccgaugu...	1	0	TE2
..........auuaccgauggauccuuaccg...	1	0	TE2
..........auuaccgauggauccuuaccga..	4	0	TE2
....aguaaggguuuucuguaaugca...........	11	0	TE2
....aguaaggguuuucuguaaugcU...........	2	1	TE2
....uaaggguuuucuguaaugcagC.........	3	1	TE2

aga-mir-10355

aga-mir-10355-star

aaugugcuacauuaccgauggauccuuaccgauguauuugguacuauucaguaaggguuuucuguaaugcaguacguuucau

miRBase precursor : aga-mir-10356-1
Total read count : 5746
aga-mir-10356-1 read count: 2764
aga-mir-10356-1-star read count: 42
aga-mir-10356-2 read count: 2764
aga-mir-10356-2-star read count: 42
remaining reads : 134

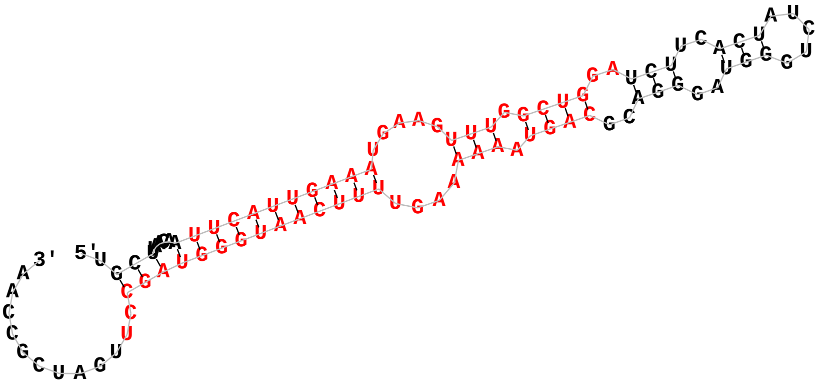

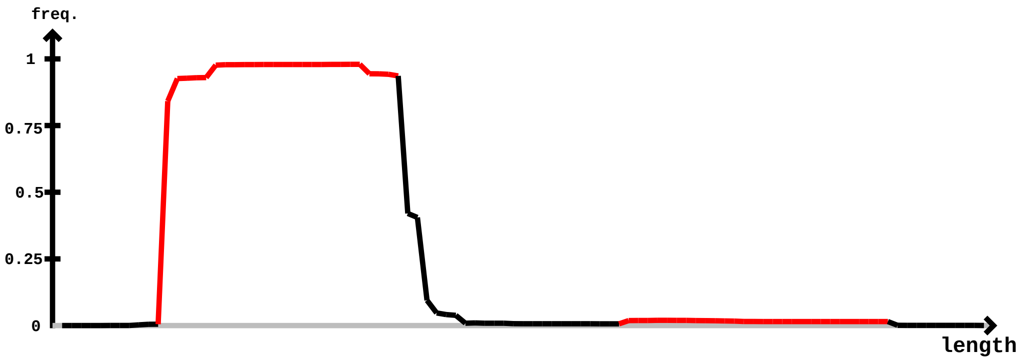

aga-mir-10356-1

aga-mir-10356-1-star

aga-mir-10356-2-star

aga-mir-10356-2

5'- ugcuuaacaauucauugaaaugaaguuuggcuggaucuucacuaucuggguagggacgcaguaaaaaaguuuucaauggguagccuugaucgccaa -3'	exp		
.(((.....((((((((((.....(((.((((..(((..(((.....)))..)))..)))).)))....))))))))))))))..............	reads	mm	sample
.......caauucauugaaaugaaguuuggcugga...	1	0	OV2
........aauucauugaaaugaaguuuggcugga...	2	0	OV2
..........uucauugaaaugaaguuuggc...	20	0	OV2
..........uucauCgaaaugaaguuuggc...	1	1	OV2
..........uucauugaaaugaaguuuggcug...	1	0	OV2
..........uucauugaaaugaaguuuggcugg..	2	0	OV2
..........uucauugaaaugaaguuuggcuggG...	1	1	OV2
..........uucauugaaaugaaguuuggcugAa...	1	1	OV2
..........uucauCgaaaugaaguuuggcugga...	1	1	OV2
..........uucauugaaaugaaguuuggcugga...	313	0	OV2
..........uucauugaaaugaagCuuggcugga...	1	1	OV2
..........uucauugaaaugaaguuuggcuggaA..	1	1	OV2
..........uucauugaaaugaaguuuggcuggau..	5	0	OV2
..........uucauugaaaugaaguuuggcuggauA...	1	1	OV2
..........uucauugaaauAaaguuuggcuggauc...	1	1	OV2
..........uucauugaaaugaagCuuggcuggauc...	1	1	OV2
..........uucauugaaCugaaguuuggcuggauc...	1	1	OV2
..........uucauugaaaugaaguuuggcuggauc...	122	0	OV2
..........uucauugaaaugaaguuuggcuggaucu..	9	0	OV2
..........uucauugaaaugaaguuuggcuggaucuA...	1	1	OV2
..........uucauugaaaugaaguuuggcuggaucuuUacuauc...	1	1	OV2
..........uucauugaaaugaaguuuggcuggaucuuUacuaucuggguagggacg......................................	1	1	OV2
...........ucauugaaaugaaguuuggcugga...	3	0	OV2
...........ucauugaaaugaaguuuggcuggau..	1	0	OV2
...........ucauugaaaugaaguuuggcuggauc...	44	0	OV2
...........ucauugaaaugaaguuuggcuggaucu..	3	0	OV2
...........ucauugaaaugaaguuuggcuggaucuu...	2	0	OV2
...........ucauugaaaugaaguuuggcuggaucuuUacuauc...	1	1	OV2
...........ucauugaaaugaaguuuggcuggaucuucacuauc...	1	0	OV2
............cauugaaaugaaguuuggcuggauc...	1	0	OV2
............cauugaaaugaaguuuggcuggaucu..	1	0	OV2
...............ugaaaugaaguuuggcuggauc...	1	0	OV2

```
          aga-mir-10356-1
                                                          aga-mir-10356-1-star
ugcuuaacaauucauugaaaugaaguuuggcuggaucuucacuaucuggguagggacgcaguaaaaaaguuuucaauggguagccuugaucgccaa
                                                          aga-mir-10356-2-star
          aga-mir-10356-2


...............ugaaaugaaguuuggcuggaucu..........................................................    2    0    OV2
...............ugaaaugaaguuuggcuggaucuu.........................................................    2    0    OV2
...............ugaaaugaaguuuggcuggaucuuc........................................................    2    0    OV2
...............ugaaaugaaguuuggcuggaucuuUa.......................................................    1    1    OV2
...............ugaaaugaaguuuggcuggaucuuca.......................................................    12   0    OV2
...............ugaaaugaaguuuggcuggaucuucaU......................................................    1    1    OV2
................gaaaugaaguuuggcuggauc...........................................................    1    0    OV2
....................ugaaguuuggcuggaucuucacuaucu.................................................    1    0    OV2
..........................................uaucuggguagggacgUaguaaaaaagu..........................    1    1    OV2
..........................................................caguaaaaaaguuuucaauggguagccu..........    3    0    OV2
.............................................................uaaaaaaguuuucaauggguagccu..........    1    0    OV2

....uaacaauucauugaaaugaaguuuggcuggauc...........................................................    1    0    TE1
.......caauucauugaaaugaaguuuggc.................................................................    1    0    TE1
..........uucauugaaaugaaguuuggc.................................................................    6    0    TE1
..........uucauugaaaugaaguuuggcug...............................................................    2    0    TE1
..........uucauuCaaaugaaguuuggcugga.............................................................    1    1    TE1
..........uucauugaaaugaaguuugAcugga.............................................................    1    1    TE1
..........uucauugaaaugaaguuuggcugga.............................................................    82   0    TE1
..........uucauugaaaugaagAuuggcugga.............................................................    1    1    TE1
..........uucauugaaaugaaguuuAgcugga.............................................................    1    1    TE1
..........uucauugaaaugaaguuuggcuggau............................................................    2    0    TE1
..........uucauugaaaugaaguuuggcuggauc...........................................................    53   0    TE1
..........uucauugaaaugaaguuuggcuggauU...........................................................    1    1    TE1
..........uucauugaaaugaaguuuggcuggaucu..........................................................    13   0    TE1
...........ucauugaaaugaaguuuggcugg..............................................................    1    0    TE1
...........ucauugaaaugaaguuuggcuggauc...........................................................    15   0    TE1
...........ucauugaaaugaaguuuggcuggaucu..........................................................    1    0    TE1
...........ucauugaaaugaaguuuggcuggaucuu.........................................................    1    0    TE1
...............ugaaaugaaguuuggcuggaucu..........................................................    1    0    TE1
...............ugaaaugaaguuuggcuggaucuu.........................................................    2    0    TE1
...............ugaaaugaaguuuggcuggaucuuc........................................................    1    0    TE1
...............ugaaaugaaguuuggcuggaucuuca.......................................................    22   0    TE1
...............ugaaaugaaguuuggcuggauUuucac......................................................    1    1    TE1
...............ugaaaugaaguuuggcuggaucuucac......................................................    3    0    TE1
...............ugaaaugaaguuuggcuggaucuucacu.....................................................    3    0    TE1
...............ugaaaugaaguuuggcuggaucuucacuaucuggguagggacg......................................    2    0    TE1
................gaaaugaaguuuggcuggauc...........................................................    1    0    TE1
..........................................uaucuggguagggacgcaguaaaaaag...........................    1    0    TE1
..........................................................caguaaaaaaguuuucaauggguagccu..........    11   0    TE1
...........................................................aguaaaaaaguuuucaauggguagccu..........    1    0    TE1
........................................................................ucaauggguagccuugaucgccaa    1    0    TE1

........aauucauugaaaugaaguuuggcugga.............................................................    1    0    MF2

..........uucauugaaaugaaguuuggcuggau............................................................    1    0    FW2

.......caauucauugaaaugaaguuuggcugga.............................................................    1    0    OV1
.........auucauugaaaugaaguuuggcugga.............................................................    1    0    OV1
..........uucauugaaaugaaguuuggc.................................................................    50   0    OV1
..........uucauugaaaugaaguuuggcug...............................................................    1    0    OV1
..........uucauugaaaugaaguuuggcugg..............................................................    4    0    OV1
..........uucauugaaaugaaguuuggcGgga.............................................................    1    1    OV1
..........uucauugaaaugaagCuuggcugga.............................................................    1    1    OV1
..........uCcauugaaaugaaguuuggcugga.............................................................    1    1    OV1
..........uucauugaaaugaaguuuggAugga.............................................................    2    1    OV1
..........uucauugaaaugaaguuuggcuggG.............................................................    1    1    OV1
..........uucauugaaaugaaguuuggcugga.............................................................    622  0    OV1
..........uucauugaaauUaaguuuggcugga.............................................................    1    1    OV1
..........uucauugaaCugaaguuuggcugga.............................................................    1    1    OV1
..........uucauugaaauCaaguuuggcugga.............................................................    1    1    OV1
..........uucauugaaaugaaguuuggcuggau............................................................    14   0    OV1
..........uucauugaaaugaagCuuggcuggauc...........................................................    1    1    OV1
..........uucauCgaaaugaaguuuggcuggauc...........................................................    1    1    OV1
..........uucauugaaaugaaguuuggUuggauc...........................................................    1    1    OV1
..........uucauuAaaaugaaguuuggcuggauc...........................................................    1    1    OV1
..........Cucauugaaaugaaguuuggcuggauc...........................................................    1    1    OV1
```

```
          aga-mir-10356-1
                                                          aga-mir-10356-1-star
ugcuuaacaauucauugaaaugaaguuuggcuggaucuucacuaucuggguagggacg[illegible]ggguagccuugaucgccaa
          aga-mir-10356-2-star
          aga-mir-10356-2

..........uucauugaaaugaaguuuggcuggauA...........................................................    3      1      OV1
..........uucauugaaaugaaguuuggcuggauc...........................................................    239    0      OV1
..........uucauugaaauAaaguuuggcuggauc...........................................................    1      1      OV1
..........uucauugaaaugaaguuuggcuggaucu..........................................................    40     0      OV1
..........uucauugaaaugaaguuuggcGggaucu..........................................................    1      1      OV1
..........uucauugaaaugaaguuuggcuggaucuu.........................................................    1      0      OV1
..........uucauugaaaugaaguuuggcuggaucuucac......................................................    1      0      OV1
...........ucauugaaaugaaguuuggcugga.............................................................    6      0      OV1
...........ucauugaaaugaaguuuggcuggauA...........................................................    4      1      OV1
...........ucauugaaaugaaguuuggcuggauc...........................................................    71     0      OV1
...........ucauugaaaugaaguuuggcuggaucu..........................................................    10     0      OV1
...........ucauugaaaugaaguuuggcuggaucuu.........................................................    2      0      OV1
...........ucauugaaaugaaguuuggcuggaucuucacuauc..................................................    1      0      OV1
.............auugaaaugaaguuuggcugga.............................................................    2      0      OV1
.............auugaaaugaaguuuggcuggauc...........................................................    1      0      OV1
..............uugaaaugaaguuuggcuggaucu..........................................................    1      0      OV1
...............ugaaaugaaguuuggcuggaucu..........................................................    8      0      OV1
...............ugaaaugaaguuuggcuggaucuu.........................................................    5      0      OV1
...............ugaaaugaaguuuggcuggaucuuca.......................................................    12     0      OV1
...............ugaaaugaaguuuggcuggaucuuUa.......................................................    3      1      OV1
...............ugaaaugaaguuuggcuggaucuucac......................................................    1      0      OV1
................gaaaugaaguuuggcuggauc...........................................................    2      0      OV1
................gaaaugaaguuuggcuggaucu..........................................................    1      0      OV1
..................aaugaaguuuggcuggaucu..........................................................    1      0      OV1
.............................gcCggaucuucacuauc..................................................    1      1      OV1
.....................................uucacuaucuggguagggacgUaguaaaa..............................    1      1      OV1
......................................ucacuaucuggguagggacgUaguaaa...............................    1      1      OV1
..........................................uaucuggguagggacgUaguaaaaaa............................    2      1      OV1
..........................................uaucuggguagggacgUaguaaaaaag...........................    1      1      OV1
..........................................uaucuggguagggacgUaguaaaaaagu..........................    2      1      OV1
..........................................................caguaaaaaaguuuucaauggguagccu..........    3      0      OV1
.....................................................................uuuucaauggguagccuugaucgcca.    1      0      OV1

.......caauucauugaaaugaaguuuggcugg..............................................................    1      0      BF2
........aauucauugaaaugaaguuuggcugga.............................................................    1      0      BF2
..........uucauugaaaugaaguuuggc.................................................................    3      0      BF2
..........uucauugaaaugaaguuuggcugg..............................................................    5      0      BF2
..........uucauugaaaugaaguuuggcugga.............................................................    98     0      BF2
..........uucauugaaauAaaguuuggcugga.............................................................    1      1      BF2
..........uucauugaaauAaaguuuggcuggauc...........................................................    1      1      BF2
..........uucauugaaaugaaguuuggcuggauc...........................................................    36     0      BF2
..........uucauugaaaugaaguuuggcugAauc...........................................................    1      1      BF2
..........uucauugaaaugaaguuuggcuggaCc...........................................................    1      1      BF2
..........uucauugaaaugaaguuuggcuggaucu..........................................................    7      0      BF2
...........ucauugaaaugaaguuuggcuggau............................................................    1      0      BF2
...........ucauugaaaugaaguuuggcuggauA...........................................................    1      1      BF2
...........ucauugaaaugaaguuuggcuggauc...........................................................    18     0      BF2
............cauugaaaugaaguuuggcuggaucu..........................................................    1      0      BF2
.............auugaaaugaaguCuggcuggaucuu.........................................................    1      1      BF2
.............auugaaaugaaguuuggcuggaucuuca.......................................................    1      0      BF2
...............ugaaaugaaguuuggcuggaucu..........................................................    1      0      BF2
...............ugaaaugaaguuuggcuggaucuuc........................................................    2      0      BF2
...............ugaaaugaaguuuggcuggaucuuUa.......................................................    2      1      BF2
...............ugaaaugaaguuuggcuggaucuuca.......................................................    5      0      BF2
...............ugaaaugaaguuuggcuggaucuucac......................................................    1      0      BF2
...........................uggcuggaucuucacuaucuggguaggg.........................................    1      0      BF2
.......................................cacuaucuggguagggacgUaguaaa...............................    1      1      BF2
.......................................cacuaucuggguagggacgUaguaaaaa.............................    1      1      BF2
..........................................uaucuggguagggacgUaguaaaaaag...........................    1      1      BF2

.......caauucauugaaaugaaguuuggcuggaucu..........................................................    1      0      BF1
..........uucauugaaaugaaguuuggc.................................................................    10     0      BF1
..........uucauugaaaugaaguuuggcugg..............................................................    3      0      BF1
..........Cucauugaaaugaaguuuggcugga.............................................................    1      1      BF1
..........uucauugaaaugaaguuuggcugga.............................................................    189    0      BF1
..........uucauugaaaugaaguuuggAugga.............................................................    1      1      BF1
..........uCcauugaaaugaaguuuggcugga.............................................................    1      1      BF1
```

aga-mir-10356-1

aga-mir-10356-1-star

ugcuuaacaauucauugaaaugaaguuuggcuggaucuucacuaucuggguagggacgcaguaaaaaaguuuucaauggguagccuugaucgccaa

aga-mir-10356-2-star

aga-mir-10356-2

..........uucauugaaaugaaguuuggcuggau..	7	0	BF1
..........uucauuAaaaugaaguuuggcuggauc...	1	1	BF1
..........uucauugaaaugaaguuuggcuggauc...	124	0	BF1
..........uucauugaaaugaaguuuggcuggaucu..	17	0	BF1
...........ucauugaaaugaaguuuggcuggauc...	25	0	BF1
...........ucauugaaaugaaguuuggcugAauc...	1	1	BF1
...........ucauugaaaugaaguuuggcuggaucu..	5	0	BF1
...............ugaaaugaaguuuggcuggaucu..	2	0	BF1
...............ugaaaugaaguuuggcuggaucuA...	1	1	BF1
...............ugaaaugaaguuuggcuggaucuuUa...	1	1	BF1
...............ugaaaugaaguuuggcuggaucuuca...	5	0	BF1
....uaucuggguagggacgUaguaaaaaaguuu........................	1	1	BF1
....caguaaaaaaguuuucaauggguagccu..........	1	0	BF1
..........uucauugaaaugaaguuuggcuggauc...	1	0	FW1
..........uucauugaaaugaaguuuggcugga...	1	0	MW2
.......caauucauugaaaugaaguuuggcugga...	2	0	TE2
........aauucauugaaaugaaguuuggcugga...	2	0	TE2
..........uucauugaaaugaaguuuggc...	13	0	TE2
..........uucauugaaaugaaguuuggcu..	1	0	TE2
..........uucauugaaaugaaguuuggcug...	1	0	TE2
..........uucauugaaaugaaguuuggcugg..	2	0	TE2
..........uucauugaaaugaaguuCggcugga...	1	1	TE2
..........Cucauugaaaugaaguuuggcugga...	1	1	TE2
..........uucauugaaaugaaguuuggUugga...	1	1	TE2
..........uucauuCaaaugaaguuuggcugga...	1	1	TE2
..........uucauugaaaugaaguuuggcugga...	159	0	TE2
..........uucauugaaaugaaguuuggcuggau..	9	0	TE2
..........uucauugaaaugaaguuuggAuggauc...	1	1	TE2
..........uucauugaaaugaaguuuggcuggauU...	3	1	TE2
..........uucauugaaGugaaguuuggcuggauc...	1	1	TE2
..........uucauugaaaugaaguuuggcuggauc...	107	0	TE2
..........uCcauugaaaugaaguuuggcuggauc...	1	1	TE2
..........uucauugaaaugaaguuuggcuggaucu..	8	0	TE2
...........ucauugaaaugaaguuuggcugga...	4	0	TE2
...........ucauugaaaugaaguuuggcuggau..	3	0	TE2
...........ucauugaaaugaaguuuggcuggauc...	21	0	TE2
...........ucauugaaaugaaguuuggcuggaucu..	3	0	TE2
............cauugaaaugaaguuuggcuggaucu..	1	0	TE2
..............Cugaaaugaaguuuggcuggaucu..	1	1	TE2
...............ugaaaugaaguuuggcugga...	1	0	TE2
...............ugaaaugaaguuuggcuggauc...	1	0	TE2
...............ugaaaugaaguuuggcuggaucu..	2	0	TE2
...............ugaaaugaaguuuggcuggaucuu...	2	0	TE2
...............ugaaaugaaguuuggcuggauUuc...	1	1	TE2
...............ugaaaugaaguuuggcuggaucuuc..	2	0	TE2
...............ugaaaugaaguGuggcuggaucuuca...	1	1	TE2
...............ugaaaugaaguuuggcuggaucuuca...	15	0	TE2
...............ugaaaugaaguuuggcuggaucuuUa...	7	1	TE2
......................................ucacuaucuggguagggacgUagua.................................	1	1	TE2
....uaucuggguagggacgUaguaaaaaagu..........................	1	1	TE2
....uaucuggguagggacgUaguaaaaaaguuu........................	1	1	TE2
....caguaaaaaagAuuucaauggguagccu..........	1	1	TE2
....caguaaaaaaguuuucaauggguagccu..........	20	0	TE2
....uaaaaaaguuuucaauggguagccuu.........	1	0	TE2

miRBase precursor : aga-mir-10356-2
Total read count : 5746
aga-mir-10356-1 read count: 2764
aga-mir-10356-1-star read count: 42
aga-mir-10356-2 read count: 2764
aga-mir-10356-2-star read count: 42
remaining reads : 134

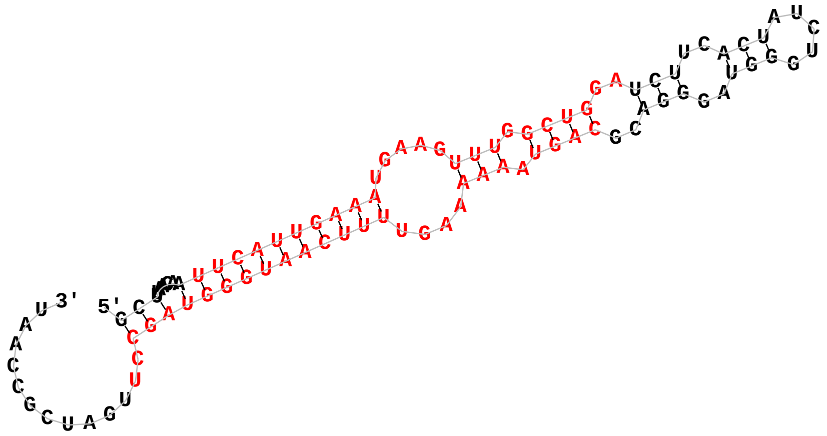

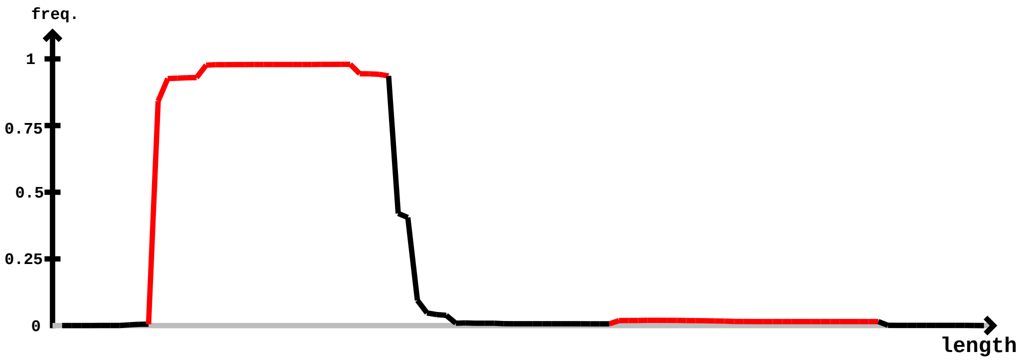

aga-mir-10356-1

aga-mir-10356-1-star

aga-mir-10356-2-star

aga-mir-10356-2

```
5'- gcuuaacaauucauugaaaugaaguuuggcuggaucuucacuaucuggguagggacgcaguaaaaaaguuuucaauggguagccuugaucgccaau   -3'    exp
    (((.....(((((((((((.....(((.((((..(((..(((.....)))..)))..)))).)))....))))))))))))))).............    reads      mm         sample
    ......caauucauugaaaugaaguuuggcugga..............................................................    1          0          OV2
    .......aauucauugaaaugaaguuuggcugga..............................................................    2          0          OV2
    .........uucauugaaaugaaguuuggc..................................................................    20         0          OV2
    .........uucauCgaaaugaaguuuggc..................................................................    1          1          OV2
    .........uucauugaaaugaaguuuggcug................................................................    1          0          OV2
    .........uucauugaaaugaaguuuggcugg...............................................................    2          0          OV2
    .........uucauugaaaugaaguuuggcuggG..............................................................    1          1          OV2
    .........uucauugaaaugaaguuuggcugAa..............................................................    1          1          OV2
    .........uucauCgaaaugaaguuuggcugga..............................................................    1          1          OV2
    .........uucauugaaaugaaguuuggcugga..............................................................    313        0          OV2
    .........uucauugaaaugaagCuuggcugga..............................................................    1          1          OV2
    .........uucauugaaaugaaguuuggcuggaA.............................................................    1          1          OV2
    .........uucauugaaaugaaguuuggcuggau.............................................................    5          0          OV2
    .........uucauugaaaugaaguuuggcuggauA............................................................    1          1          OV2
    .........uucauugaaauAaaguuuggcuggauc............................................................    1          1          OV2
    .........uucauugaaaugaagCuuggcuggauc............................................................    1          1          OV2
    .........uucauugaaCugaaguuuggcuggauc............................................................    1          1          OV2
    .........uucauugaaaugaaguuuggcuggauc............................................................    122        0          OV2
    .........uucauugaaaugaaguuuggcuggaucu...........................................................    9          0          OV2
    .........uucauugaaaugaaguuuggcuggaucuA..........................................................    1          1          OV2
    .........uucauugaaaugaaguuuggcuggaucuuUacuauc...................................................    1          1          OV2
    .........uucauugaaaugaaguuuggcuggaucuuUacuaucuggguagggacg.......................................    1          1          OV2
    ..........ucauugaaaugaaguuuggcugga..............................................................    3          0          OV2
    ..........ucauugaaaugaaguuuggcuggau.............................................................    1          0          OV2
    ..........ucauugaaaugaaguuuggcuggauc............................................................    44         0          OV2
    ..........ucauugaaaugaaguuuggcuggaucu...........................................................    3          0          OV2
    ..........ucauugaaaugaaguuuggcuggaucuu..........................................................    2          0          OV2
    ..........ucauugaaaugaaguuuggcuggaucuuUacuauc...................................................    1          1          OV2
    ..........ucauugaaaugaaguuuggcuggaucuucacuauc...................................................    1          0          OV2
    ...........cauugaaaugaaguuuggcuggauc............................................................    1          0          OV2
    ...........cauugaaaugaaguuuggcuggaucu...........................................................    1          0          OV2
    ..............ugaaaugaaguuuggcuggauc............................................................    1          0          OV2
```

```
         aga-mir-10356-1
                                                         aga-mir-10356-1-star
gcuuaacaauucauugaaaugaaguuuggcuggaucuucacuaucuggguagggacgcaguaaaaaaguuuucaauggguagccuugaucgccaau
                                                         aga-mir-10356-2-star
         aga-mir-10356-2


..............ugaaaugaaguuuggcuggaucu...........................................................   2    0    OV2
..............ugaaaugaaguuuggcuggaucuu..........................................................   2    0    OV2
..............ugaaaugaaguuuggcuggaucuuc.........................................................   2    0    OV2
..............ugaaaugaaguuuggcuggaucuuUa........................................................   1    1    OV2
..............ugaaaugaaguuuggcuggaucuuca........................................................   12   0    OV2
..............ugaaaugaaguuuggcuggaucuucaU.......................................................   1    1    OV2
...............gaaaugaaguuuggcuggauc............................................................   1    0    OV2
...................ugaaguuuggcuggaucuucacuaucu..................................................   1    0    OV2
.........................................uaucuggguagggacgUaguaaaaaagu...........................   1    1    OV2
.........................................................caguaaaaaaguuuucaauggguagccu...........   3    0    OV2
............................................................uaaaaaaguuuucaauggguagccu...........   1    0    OV2

...uaacaauucauugaaaugaaguuuggcuggauc............................................................   1    0    TE1
......caauucauugaaaugaaguuuggc..................................................................   1    0    TE1
.........uucauugaaaugaaguuuggc..................................................................   6    0    TE1
.........uucauugaaaugaaguuuggcug................................................................   2    0    TE1
.........uucauuCaaaugaaguuuggcugga..............................................................   1    1    TE1
.........uucauugaaaugaaguuugAcugga..............................................................   1    1    TE1
.........uucauugaaaugaaguuuggcugga..............................................................   82   0    TE1
.........uucauugaaaugaagAuuggcugga..............................................................   1    1    TE1
.........uucauugaaaugaaguuuAgcugga..............................................................   1    1    TE1
.........uucauugaaaugaaguuuggcuggau.............................................................   2    0    TE1
.........uucauugaaaugaaguuuggcuggauc............................................................   53   0    TE1
.........uucauugaaaugaaguuuggcuggauU............................................................   1    1    TE1
.........uucauugaaaugaaguuuggcuggaucu...........................................................   13   0    TE1
..........ucauugaaaugaaguuuggcugg...............................................................   1    0    TE1
..........ucauugaaaugaaguuuggcuggauc............................................................   15   0    TE1
..........ucauugaaaugaaguuuggcuggaucu...........................................................   1    0    TE1
..........ucauugaaaugaaguuuggcuggaucuu..........................................................   1    0    TE1
..............ugaaaugaaguuuggcuggaucu...........................................................   1    0    TE1
..............ugaaaugaaguuuggcuggaucuu..........................................................   2    0    TE1
..............ugaaaugaaguuuggcuggaucuuc.........................................................   1    0    TE1
..............ugaaaugaaguuuggcuggaucuuca........................................................   22   0    TE1
..............ugaaaugaaguuuggcuggauUuucac.......................................................   1    1    TE1
..............ugaaaugaaguuuggcuggaucuucac.......................................................   3    0    TE1
..............ugaaaugaaguuuggcuggaucuucacu......................................................   3    0    TE1
..............ugaaaugaaguuuggcuggaucuucacuaucuggguagggacg.......................................   2    0    TE1
...............gaaaugaaguuuggcuggauc............................................................   1    0    TE1
.........................................uaucuggguagggacgcaguaaaaaag............................   1    0    TE1
.........................................................caguaaaaaaguuuucaauggguagccu...........   11   0    TE1
..........................................................aguaaaaaaguuuucaauggguagccu...........   1    0    TE1
.......................................................................ucaauggguagccuugaucgccaa.   1    0    TE1

.......aauucauugaaaugaaguuuggcugga..............................................................   1    0    MF2

.........uucauugaaaugaaguuuggcuggau.............................................................   1    0    FW2

......caauucauugaaaugaaguuuggcugga..............................................................   1    0    OV1
........auucauugaaaugaaguuuggcugga..............................................................   1    0    OV1
.........uucauugaaaugaaguuuggc..................................................................   50   0    OV1
.........uucauugaaaugaaguuuggcug................................................................   1    0    OV1
.........uucauugaaaugaaguuuggcugg...............................................................   4    0    OV1
.........uucauugaaaugaaguuuggcGgga..............................................................   1    1    OV1
.........uucauugaaaugaagCuuggcugga..............................................................   1    1    OV1
.........uCcauugaaaugaaguuuggcugga..............................................................   1    1    OV1
.........uucauugaaaugaaguuuggAugga..............................................................   2    1    OV1
.........uucauugaaaugaaguuuggcuggG..............................................................   1    1    OV1
.........uucauugaaaugaaguuuggcugga..............................................................   622  0    OV1
.........uucauugaaauUaaguuuggcugga..............................................................   1    1    OV1
.........uucauugaaCugaaguuuggcugga..............................................................   1    1    OV1
.........uucauugaaauCaaguuuggcugga..............................................................   1    1    OV1
.........uucauugaaaugaaguuuggcuggau.............................................................   14   0    OV1
.........uucauugaaaugaagCuuggcuggauc............................................................   1    1    OV1
.........uucauCgaaaugaaguuuggcuggauc............................................................   1    1    OV1
.........uucauugaaaugaaguuuggUuggauc............................................................   1    1    OV1
.........uucauAaaaugaaguuuggcuggauc.............................................................   1    1    OV1
.........Cucauugaaaugaaguuuggcuggauc............................................................   1    1    OV1
```

aga-mir-10356-1

aga-mir-10356-1-star

gcuuaacaauucauugaaaugaaguuuggcuggaucuucacuaucuggguagggacgcaguaaaaaaguuuucaauggguagccuugaucgccaau

aga-mir-10356-2-star

aga-mir-10356-2

.........uucauugaaaugaaguuuggcuggauA..	3	1	OV1
.........uucauugaaaugaaguuuggcuggauc..	239	0	OV1
.........uucauugaaauAaaguuuggcuggauc..	1	1	OV1
.........uucauugaaaugaaguuuggcuggaucu...	40	0	OV1
.........uucauugaaaugaaguuuggcGggaucu...	1	1	OV1
.........uucauugaaaugaaguuuggcuggaucuu..	1	0	OV1
.........uucauugaaaugaaguuuggcuggaucuucac...	1	0	OV1
..........ucauugaaaugaaguuuggcugga..	6	0	OV1
..........ucauugaaaugaaguuuggcuggauA..	4	1	OV1
..........ucauugaaaugaaguuuggcuggauc..	71	0	OV1
..........ucauugaaaugaaguuuggcuggaucu...	10	0	OV1
..........ucauugaaaugaaguuuggcuggaucuu..	2	0	OV1
..........ucauugaaaugaaguuuggcuggaucuucacuauc...	1	0	OV1
............auugaaaugaaguuuggcugga..	2	0	OV1
............auugaaaugaaguuuggcuggauc..	1	0	OV1
.............uugaaaugaaguuuggcuggaucu...	1	0	OV1
..............ugaaaugaaguuuggcuggaucu...	8	0	OV1
..............ugaaaugaaguuuggcuggaucuu..	5	0	OV1
..............ugaaaugaaguuuggcuggaucuuca..	12	0	OV1
..............ugaaaugaaguuuggcuggaucuuUa..	3	1	OV1
..............ugaaaugaaguuuggcuggaucuucac...	1	0	OV1
...............gaaaugaaguuuggcuggauc..	2	0	OV1
...............gaaaugaaguuuggcuggaucu...	1	0	OV1
.................aaugaaguuuggcuggaucu...	1	0	OV1
............................gcCggaucuucacuauc...	1	1	OV1
....................................uucacuaucuggguagggacgUaguaaaa...............................	1	1	OV1
.....................................ucacuaucuggguagggacgUaguaaa................................	1	1	OV1
....uaucuggguagggacgUaguaaaaaa.............................	2	1	OV1
....uaucuggguagggacgUaguaaaaaag............................	1	1	OV1
....uaucuggguagggacgUaguaaaaaagu...........................	2	1	OV1
....caguaaaaaaguuuucaauggguagccu...........	3	0	OV1
....uuuucaauggguagccuugaucgcca..	1	0	OV1
......caauucauugaaaugaaguuuggcugg...	1	0	BF2
.......aauucauugaaaugaaguuuggcugga..	1	0	BF2
.........uucauugaaaugaaguuuggc..	3	0	BF2
.........uucauugaaaugaaguuuggcugg...	5	0	BF2
.........uucauugaaaugaaguuuggcugga..	98	0	BF2
.........uucauugaaauAaaguuuggcugga..	1	1	BF2
.........uucauugaaauAaaguuuggcuggauc..	1	1	BF2
.........uucauugaaaugaaguuuggcuggauc..	36	0	BF2
.........uucauugaaaugaaguuuggcugAauc..	1	1	BF2
.........uucauugaaaugaaguuuggcuggaCc..	1	1	BF2
.........uucauugaaaugaaguuuggcuggaucu...	7	0	BF2
..........ucauugaaaugaaguuuggcuggau...	1	0	BF2
..........ucauugaaaugaaguuuggcuggauA..	1	1	BF2
..........ucauugaaaugaaguuuggcuggauc..	18	0	BF2
...........cauugaaaugaaguuuggcuggaucu...	1	0	BF2
............auugaaaugaaguCuggcuggaucuu..	1	1	BF2
............auugaaaugaaguuuggcuggaucuuca..	1	0	BF2
..............ugaaaugaaguuuggcuggaucu...	1	0	BF2
..............ugaaaugaaguuuggcuggaucuuc...	2	0	BF2
..............ugaaaugaaguuuggcuggaucuuUa..	2	1	BF2
..............ugaaaugaaguuuggcuggaucuuca..	5	0	BF2
..............ugaaaugaaguuuggcuggaucuucac...	1	0	BF2
..........................uggcuggaucuucacuaucuggguaggg..	1	0	BF2
......................................cacuaucuggguagggacgUaguaaa................................	1	1	BF2
......................................cacuaucuggguagggacgUaguaaaaa..............................	1	1	BF2
....uaucuggguagggacgUaguaaaaaag............................	1	1	BF2
......caauucauugaaaugaaguuuggcuggaucu...	1	0	BF1
.........uucauugaaaugaaguuuggc..	10	0	BF1
.........uucauugaaaugaaguuuggcugg...	3	0	BF1
.........Cucauugaaaugaaguuuggcugga..	1	1	BF1
.........uucauugaaaugaaguuuggcugga..	189	0	BF1
.........uucauugaaaugaaguuuggAugga..	1	1	BF1
.........uCcauugaaaugaaguuuggcugga..	1	1	BF1

```
         aga-mir-10356-1
                                                         aga-mir-10356-1-star
gcuuaacaauucauugaaaugaaguuuggcuggaucuucacuaucuggguagggacgcaguaaaaaaguuuucaauggguagccuugaucgccaau
                                                         aga-mir-10356-2-star
         aga-mir-10356-2


.........uucauugaaaugaaguuuggcuggau.............................................................    7          0          BF1
.........uucauuAaaaugaaguuuggcuggauc............................................................    1          1          BF1
.........uucauugaaaugaaguuuggcuggauc............................................................    124        0          BF1
.........uucauugaaaugaaguuuggcuggaucu...........................................................    17         0          BF1
..........ucauugaaaugaaguuuggcuggauc............................................................    25         0          BF1
..........ucauugaaaugaaguuuggcugAauc............................................................    1          1          BF1
..........ucauugaaaugaaguuuggcuggaucu...........................................................    5          0          BF1
..............ugaaaugaaguuuggcuggaucu...........................................................    2          0          BF1
..............ugaaaugaaguuuggcuggaucuA..........................................................    1          1          BF1
..............ugaaaugaaguuuggcuggaucuuUa........................................................    1          1          BF1
..............ugaaaugaaguuuggcuggaucuuca........................................................    5          0          BF1
.........................................uaucuggguagggacgUaguaaaaaaguuu.........................    1          1          BF1
.........................................................caguaaaaaaguuuucaauggguagccu...........    1          0          BF1

.........uucauugaaaugaaguuuggcuggauc............................................................    1          0          FW1

.........uucauugaaaugaaguuuggcugga..............................................................    1          0          MW2

......caauucauugaaaugaaguuuggcugga..............................................................    2          0          TE2
.......aauucauugaaaugaaguuuggcugga..............................................................    2          0          TE2
.........uucauugaaaugaaguuuggc..................................................................    13         0          TE2
.........uucauugaaaugaaguuuggcu.................................................................    1          0          TE2
.........uucauugaaaugaaguuuggcug................................................................    1          0          TE2
.........uucauugaaaugaaguuuggcugg...............................................................    2          0          TE2
.........uucauugaaaugaaguuCggcugga..............................................................    1          1          TE2
.........Cucauugaaaugaaguuuggcugga..............................................................    1          1          TE2
.........uucauugaaaugaaguuuggUugga..............................................................    1          1          TE2
.........uucauuCaaaugaaguuuggcugga..............................................................    1          1          TE2
.........uucauugaaaugaaguuuggcugga..............................................................    159        0          TE2
.........uucauugaaaugaaguuuggcuggau.............................................................    9          0          TE2
.........uucauugaaaugaaguuuggAuggauc............................................................    1          1          TE2
.........uucauugaaaugaaguuuggcuggauU............................................................    3          1          TE2
.........uucauugaaGugaaguuuggcuggauc............................................................    1          1          TE2
.........uucauugaaaugaaguuuggcuggauc............................................................    107        0          TE2
.........uCcauugaaaugaaguuuggcuggauc............................................................    1          1          TE2
.........uucauugaaaugaaguuuggcuggaucu...........................................................    8          0          TE2
..........ucauugaaaugaaguuuggcugga..............................................................    4          0          TE2
..........ucauugaaaugaaguuuggcuggau.............................................................    3          0          TE2
..........ucauugaaaugaaguuuggcuggauc............................................................    21         0          TE2
..........ucauugaaaugaaguuuggcuggaucu...........................................................    3          0          TE2
...........cauugaaaugaaguuuggcuggaucu...........................................................    1          0          TE2
.............Cugaaaugaaguuuggcuggaucu...........................................................    1          1          TE2
..............ugaaaugaaguuuggcugga..............................................................    1          0          TE2
..............ugaaaugaaguuuggcuggauc............................................................    1          0          TE2
..............ugaaaugaaguuuggcuggaucu...........................................................    2          0          TE2
..............ugaaaugaaguuuggcuggaucuu..........................................................    2          0          TE2
..............ugaaaugaaguuuggcuggauUuuc.........................................................    1          1          TE2
..............ugaaaugaaguuuggcuggaucuuc.........................................................    2          0          TE2
..............ugaaaugaaguGuggcuggaucuuca........................................................    1          1          TE2
..............ugaaaugaaguuuggcuggaucuuca........................................................    15         0          TE2
..............ugaaaugaaguuuggcuggaucuuUa........................................................    7          1          TE2
.....................................ucacuaucuggguagggacgUagua..................................    1          1          TE2
.........................................uaucuggguagggacgUaguaaaaaagu...........................    1          1          TE2
.........................................uaucuggguagggacgUaguaaaaaaguuu.........................    1          1          TE2
.........................................................caguaaaaaagAuuucaauggguagccu...........    1          1          TE2
.........................................................caguaaaaaaguuuucaauggguagccu...........    20         0          TE2
............................................................uaaaaaaguuuucaauggguagccuu..........    1          0          TE2
```

miRBase precursor : aga-mir-10357
Total read count : 2032
aga-mir-10357 read count : 1741
aga-mir-10357-star read count : 239
remaining reads : 52

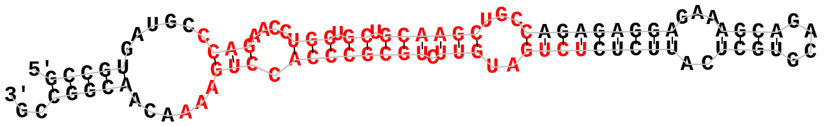

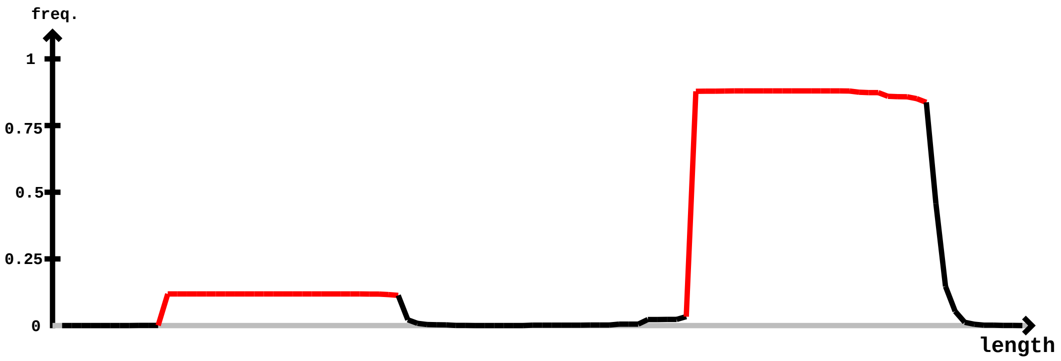


```
                                                                               aga-mir-10357
          aga-mir-10357-star
5'- gccgugaugcccagaaccuggugcugcaagcugccagagaggagaaagcagacgugcucauucucucugauguucugcgcccaccugaaaacaacggccg  -3'    exp
    (((((......(((....(((.((.((((((...(((((((((...((((....))))..)))))))))..))).)))))))).)))......)))))..     reads    mm    sample
    ..........ccagaaccuggugcugcaagcugcc.................................................................     1        0     FF2
    ..........ccagaaccuggCgcugcaagcugcca................................................................     1        1     FF2
    ................................................................cucugauguucugcgcccaccugaaa..........     1        0     FF2
    .................................................................ucugauguucugcgcccaccu..............     1        0     FF2

    ..........ccagaaccuAgugcugcaagcugcc.................................................................     6        1     TE1
    ............................................................uucucucugauguucugcgcccacc...............     1        0     TE1
    ............................................................uucucucugauguucugcgcccaccugaa...........     1        0     TE1
    ................................................................cucugauguucugcgcccaccugaa...........     1        0     TE1
    ................................................................cucugauguucugcgcccaccugaaa..........     1        0     TE1
    .................................................................ucugauguucugcgcccaccugaaa..........     3        0     TE1
    .................................................................ucugauguucugcgcccaccugaaaa.........     7        0     TE1
    .................................................................ucugauguucugcgcccaccugaaaac........     2        0     TE1

    ..........ccagaaccuAgugcugcaagcug...................................................................     1        1     OV2
    ..........ccagaaccuggugcugcaagcugc..................................................................     2        0     OV2
    ..........ccagaaccuAgugcugcaagcugcc.................................................................     3        1     OV2
    ..........ccagaaccuggGgcugcaagcugcc.................................................................     1        1     OV2
    ..........ccagaaccuggugcugcaagcugcc.................................................................     22       0     OV2
    ..........ccagaaccuggugcugcaagcugcca................................................................     2        0     OV2
    ..........ccagaaccuggugcugcaagcugccU................................................................     1        1     OV2
    ..........ccagaaccuggugcugcaagcugUcag...............................................................     2        1     OV2
    ..........ccagaaccuggugcugcaagcugUcaga..............................................................     1        1     OV2
    ..........ccagaaccuAgugcugcaagcugccagag.............................................................     1        1     OV2
    ..........ccagaaccuggugcugcaagcugUcagaga............................................................     1        1     OV2
    ................................................cagacgugcucauCcuc...................................     1        1     OV2
    ............................................................uucucucugauguucugcgcccacc...............     5        0     OV2
    ............................................................uucucucugauguucugcgcccaccuga............     1        0     OV2
    ................................................................cucugauguucugcgcccaccugaa...........     2        0     OV2
    ................................................................cucugauguucugcgcccaccugaaa..........     2        0     OV2
    ................................................................cucugauguucugcgcccaccugaaaa.........     1        0     OV2
    .................................................................ucugauguucugcgcccaccuga............     1        0     OV2
    .................................................................ucugauguucugcgcccaccugaa...........     4        0     OV2
    .................................................................ucugauguucugcgcccaccugaaa..........     133      0     OV2
```

```
                                                                 aga-mir-10357
          aga-mir-10357-star
gccgugaugcccagaaccuggugcugcaagcugccagagaggagaaagcagacgugcucauucucucugauguucugcgcccaccugaaaacaacggccg

.................................................................ucugauguucugcgcccaccugaaU..........    1      1      OV2
.................................................................ucugauguucugUgcccaccugaaaa.........    1      1      OV2
.................................................................ucugauguucugcgcccaccugaaaU.........    1      1      OV2
.................................................................ucugauguucugcgcccaccugaaaa.........    133    0      OV2
.................................................................ucugauguucugcgcccaccugaaaaA........    3      1      OV2
.................................................................ucugauguucugcgcccaccugaaaaG........    1      1      OV2
.................................................................ucugauguucugcgcccaccugaaaac........    35     0      OV2
.................................................................ucugauguucugcgcccaccugaaaaca.......    16     0      OV2
.................................................................ucugauguucugcgcccaccugaaaacaa......    2      0      OV2
.................................................................ucugauguucugcgcccaccugaaaacaac.....    1      0      OV2
.................................................................ucugauguucugcgcccaccugaaaacaacgg...    1      0      OV2
.....................................................................auguucugcgcccaccugaaaacaacgg...    1      0      OV2

..........ccagaaccuggugcugcaagcugcc.................................................................    1      0      MF2
.................................................................ucugauguucugcgcccaccugaaa..........    7      0      MF2
.................................................................ucugauguucugcgcccaccugaaaa.........    6      0      MF2
.................................................................ucugauguucugcgcccaccugaaGac........    1      1      MF2
.................................................................ucugauguucugcgcccaccugaaaac........    1      0      MF2
.................................................................ucugauguucugcgcccaccugaaaaA........    1      1      MF2

............................................................uucucucugauguucugcgcccaccugaa...........    1      0      FF1
............................................................uucGcucugauguucugcgcccaccugaa...........    1      1      FF1
.................................................................ucugauguucugcgccca.................    1      0      FF1
.................................................................ucugauguucugcgcccaccugaaa..........    1      0      FF1
.................................................................ucugauguucugcgcccaccugaaaa.........    2      0      FF1
.................................................................ucuUauguucugcgcccaccugaaaa.........    1      1      FF1

.......ugcccagaaccuggugcugcaagcug...................................................................    1      0      OV1
..........ccagaaccuggugcugcaagcugc..................................................................    2      0      OV1
..........ccagaaccuAgugcugcaagcugcc.................................................................    26     1      OV1
..........ccagaaccuggugcCgcaagcugcc.................................................................    1      1      OV1
..........ccagaaccuggugcugcaagcugcc.................................................................    50     0      OV1
..........Ucagaaccuggugcugcaagcugcc.................................................................    1      1      OV1
..........ccagaaccuggugcugcaagcugUc.................................................................    9      1      OV1
..........ccagaaccuggugcugcaagcugcA.................................................................    1      1      OV1
..........ccagaaccuggugcugcaagcugcca................................................................    10     0      OV1
..........ccagaaccuAgugcugcaagcugcca................................................................    2      1      OV1
..........ccagaaccuAgugcugcaagcugccag...............................................................    1      1      OV1
..........ccagaaccuggugcugcaagcugUcag...............................................................    4      1      OV1
..........ccagaaccuAgugcugcaagcugccaga..............................................................    1      1      OV1
..........ccagaaccuAgugcugcaagcugccagaga............................................................    1      1      OV1
................................................cagacgugcucauucuc...................................    1      0      OV1
......................................................ugcucauCcucucugauguucugcgcc...................    1      1      OV1
.........................................................ucauucucugauguucugcgccc..................    3      0      OV1
............................................................uucucucugauguucugcgccc..................    1      0      OV1
............................................................uucucucugauguucugcgcccacc...............    15     0      OV1
............................................................uCcucucugauguucugcgcccacc...............    2      1      OV1
............................................................uucucucugauguucugcgcccaccug.............    1      0      OV1
............................................................uucucucugauguucugcgcccaccuga............    1      0      OV1
................................................................cucugauguucugcgcccaccugaaa..........    5      0      OV1
................................................................cucugauguucugcgcccaccugaaaa.........    2      0      OV1
................................................................cucugauguucugcgcccaccugaaaac........    1      0      OV1
.................................................................ucugauguucugcgccca.................    1      0      OV1
.................................................................ucugauguucugcgcccaccu..............    1      0      OV1
.................................................................ucugauguucugcgcccaccuga............    6      0      OV1
.................................................................ucugauguucugcgcccaccugaa...........    13     0      OV1
.................................................................ucugauguucugcgcccaccugaaU..........    2      1      OV1
.................................................................ucugauguucugcAcccaccugaaa..........    1      1      OV1
.................................................................ucugauguucAgcgcccaccugaaa..........    1      1      OV1
.................................................................ucugauguucugcgcccaccAgaaa..........    2      1      OV1
.................................................................ucugauguucugcgcccaccugaaa..........    391    0      OV1
.................................................................ucugauguucugcgcccaccuAaaa..........    1      1      OV1
.................................................................ucugauguucugcgcccaccuAaaaa.........    1      1      OV1
.................................................................ucugauguucugcgcccaccugaaGa.........    1      1      OV1
.................................................................ucugauguucugcgcccaccugaaaU.........    1      1      OV1
.................................................................ucugauguucugcgcccaccugaaaa.........    303    0      OV1
.................................................................Gcugauguucugcgcccaccugaaaa.........    1      1      OV1
.................................................................ucugauguucugcgccUaccugaaaa.........    1      1      OV1
.................................................................ucuAauguucugcgcccaccugaaaa.........    1      1      OV1
```

```
                                                                 aga-mir-10357
          aga-mir-10357-star
gccgugaugcccagaaccuggugcugcaagcugccagagaggagaaagcagacgugcucauucucucugauguucugcgcccaccugaaaacaacggccg

.................................................................ucugauguucugcgcccaccugaaaac........    77    0    OV1
.................................................................ucugauguucugcgcccaccugaaaaA........    9     1    OV1
.................................................................ucugauguucugcgcccaccugaaaaca.......    39    0    OV1
.................................................................ucugauguucugcgcccaccugaaaaAa.......    3     1    OV1
.................................................................ucugauguucugcgcccaccugaaaaAaa......    1     1    OV1
.................................................................ucugauguucugcgcccaccugaaaacaa......    3     0    OV1
.................................................................ucugauguucugcgcccaccugaaaacaac.....    2     0    OV1
.................................................................ucugauguucugcgcccaccugaaaacaaA.....    1     1    OV1
.................................................................ucugauguucugcgcccaccugaaaacaacggcc.    1     0    OV1
..................................................................cugauguucugcgcccaccugaaaac........    1     0    OV1
....................................................................gauguucugcgcccaccugaaaacaac.....    1     0    OV1

.................................................................ucugauguucugcgcccaccugaaaa.........    1     0    MF1

..........ccagaaccuggugcugUaagcug...................................................................    1     1    BF2
..........ccagaaccuggugcugcaagcugc..................................................................    1     0    BF2
..........ccagaaccuAgugcugcaagcugcc.................................................................    5     1    BF2
..........ccagaaccuggugcugcaagcugUc.................................................................    1     1    BF2
..........ccagaaccuggugcugUaagcugcc.................................................................    1     1    BF2
..........ccagaaccuggugcugcaagcugcc.................................................................    11    0    BF2
..........ccagaaccuggugcugcaagcugcca................................................................    1     0    BF2
................................................cagacgugcucauucuc...................................    1     0    BF2
.........................................................ucauucucucugauguucugcgAcc..................    1     1    BF2
............................................................uucucucugauguucugcgcccacc...............    2     0    BF2
................................................................cucugauguucugcgcccaccugaaaa.........    2     0    BF2
.................................................................ucugauguucugcgccc..................    1     0    BF2
.................................................................ucugauguucugcgcccacc...............    1     0    BF2
.................................................................ucugauguucugcgcccaccuga............    1     0    BF2
.................................................................ucugauguucugcgcccaccugaa...........    4     0    BF2
.................................................................ucugauguCcugcgcccaccugaaa..........    1     1    BF2
.................................................................ucugauguucugcgcccaccAgaaa..........    1     1    BF2
.................................................................ucugauguucugcgcccaccugaaa..........    97    0    BF2
.................................................................ucugauguucugcAcccaccugaaa..........    1     1    BF2
.................................................................ucugauguucugcgcccaccugaaaU.........    1     1    BF2
.................................................................ucugauguucugcgccAaccugaaaa.........    1     1    BF2
.................................................................ucugauguucugcgcccaccugaaaa.........    83    0    BF2
.................................................................ucugauguucugcgcccaccugaaaaA........    3     1    BF2
.................................................................ucugauguucugcgcccaccugaaaac........    32    0    BF2
.................................................................ucugauguucugcgcccaccugaaaaca.......    18    0    BF2
.................................................................ucugauguucugcgcccaccugaaaaAa.......    1     1    BF2
.................................................................ucugauguucugcgcccaccugaaaacaa......    4     0    BF2
.................................................................ucugauguucugAgcccaccugaaaacaa......    1     1    BF2
.................................................................ucugauguucugcgcccaccugaaaacaac.....    1     0    BF2

..........ccagaaccuggugcugcaagc.....................................................................    1     0    BF1
..........ccagaaccuggugcugcaagcugcc.................................................................    32    0    BF1
..........ccagaaccuAgugcugcaagcugcc.................................................................    3     1    BF1
..........ccagaaccuAgugcugcaagcugcca................................................................    1     1    BF1
..........ccagaaccuggugcugcaagcugcca................................................................    3     0    BF1
..........ccagaaccuggugcugcaagcugccU................................................................    1     1    BF1
..........ccagaaccuAgugcugcaagcugccag...............................................................    1     1    BF1
..........ccagaaccuggugcugcaagcugUcag...............................................................    1     1    BF1
..........ccagaaccuAgugcugcaagcugccagaga............................................................    1     1    BF1
..........ccagaaccuggugcugcaagcugUcagagagg..........................................................    1     1    BF1
............................................................uucucucugauguucugcgcccaccu..............    1     0    BF1
............................................................uucucucugauguucugcgcccaccuga............    1     0    BF1
................................................................cucugauguucugcgcccaccugaaa..........    2     0    BF1
................................................................cucugauguucugcgcccaccugaaaac........    1     0    BF1
.................................................................ucugauguucugcgcccacc...............    1     0    BF1
.................................................................ucugauguucugcgcccaccuga............    2     0    BF1
.................................................................ucugauguucugcgcccaccugaaa..........    102   0    BF1
.................................................................ucugauguucugcgcccaccugaaU..........    1     1    BF1
.................................................................ucugauguucugcgcccaccugaaaa.........    72    0    BF1
.................................................................ucugauguucugcgcccaccuAaaaa.........    1     1    BF1
.................................................................ucugauguucuUcgcccaccugaaaa.........    1     1    BF1
.................................................................ucugauguucugcgcccaccugaaaac........    15    0    BF1
.................................................................ucugauguucugcAcccaccugaaaac........    1     1    BF1
.................................................................ucugauguucugcgcccaccugaaaaA........    1     1    BF1
.................................................................ucugauguucugcgcccaccugaaaaca.......    7     0    BF1
```

```
                                                                 aga-mir-10357
          aga-mir-10357-star
gccgugaugcccagaaccuggugcugcaagcugccagagaggagaaagcagacgugcucauucucucugauguucugcgcccaccugaaaacaacggccg

.................................................................ucugauguucugcgcccaccugaaaacaa......   4   0   BF1
.................................................................ucugauguucugcgcccaccugaaaacaac.....   1   0   BF1

..........ccagaaccuAgugcugcaagcug...................................................................   1   1   TE2
..........ccagaaccuAgugcugcaagcugc..................................................................   1   1   TE2
..........ccagaaccuAgugcugcaagcugcc.................................................................   10  1   TE2
..........ccagaaccuggugcugcaagcugcc.................................................................   1   0   TE2
..........ccagaaccuggugcugcaagcugccU................................................................   2   1   TE2
..........ccagaaccuggugcugcaagcugcca................................................................   2   0   TE2
..........ccagaaccuAgugcugcaagcugcca................................................................   1   1   TE2
..........ccagaaccuAgugcugcaagcugccagaga............................................................   1   1   TE2
.........................................................ucauCcucucugauguucugcgccc..................   3   1   TE2
............................................................uucucucugauguucugcgcccacc...............   1   0   TE2
..............................................................cucucugauguucugcgcccG.................   1   1   TE2
.................................................................ucugauguucugcgcccaccuga............   1   0   TE2
.................................................................ucugauguucugcgcccaccugaa...........   1   0   TE2
.................................................................ucugauguucugcgcccaccAgaaa..........   2   1   TE2
.................................................................ucugauguucugcgcccaccugaaa..........   13  0   TE2
.................................................................ucugauguucugcgcccaccugaaaa.........   6   0   TE2
.................................................................ucugauguucugcgcccaccugaaaac........   3   0   TE2
.................................................................ucugauguucugcgccAaccugaaaac........   1   1   TE2
```

miRBase precursor : aga-mir-10358-1
Total read count : 8587
aga-mir-10358-1 read count: 1689
aga-mir-10358-1-star read count 0
aga-mir-10358-2 read count: 1689
aga-mir-10358-2-star read count 0
aga-mir-10358-3 read count: 1689
aga-mir-10358-3-star read count 0
aga-mir-10358-4 read count: 1689
aga-mir-10358-4-star read count 0
aga-mir-10358-5 read count: 1689
aga-mir-10358-5-star read count 0
remaining reads : 102

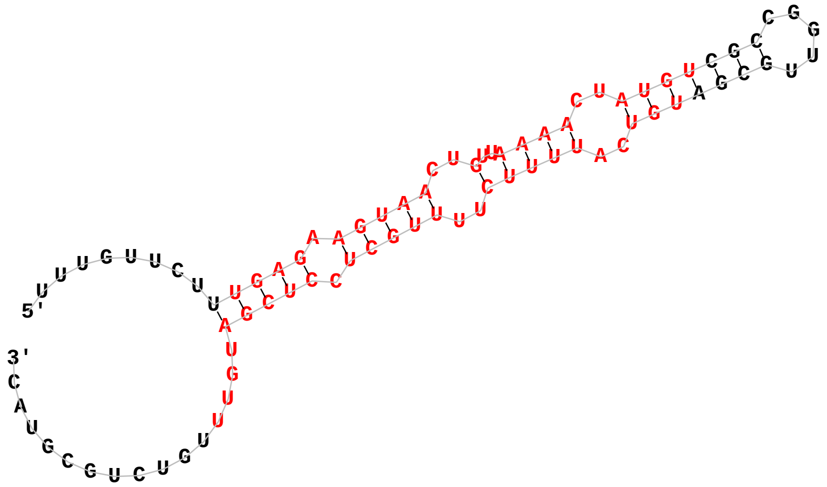

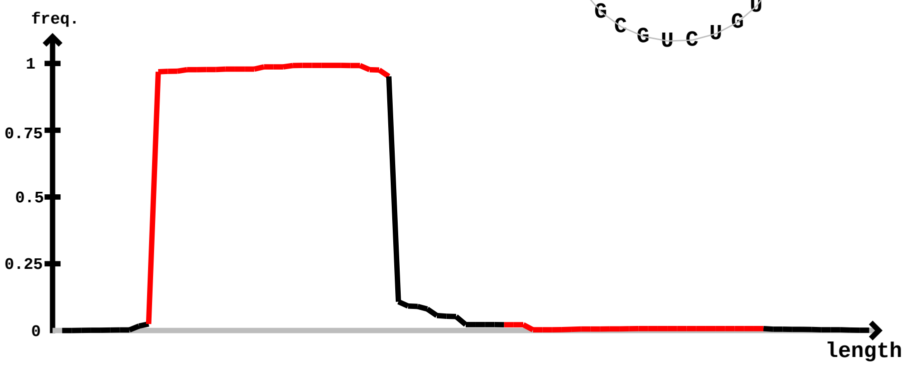


```
                                                  aga-mir-10358-5-star
                                                  aga-mir-10358-3-star
             aga-mir-10358-4
             aga-mir-10358-3
                                                  aga-mir-10358-4-star
                                                  aga-mir-10358-2-star
             aga-mir-10358-5
             aga-mir-10358-2
             aga-mir-10358-1
                                                  aga-mir-10358-1-star
5'- uuuguucuuugagaaguaacuguuaaaacuaugucgccgguugcgaugucauuuucuuuugcuccucgauguuugucugcguac   -3'    exp
    ........(((((.(((((..(..((((..(((((((.....)))))))..))))).)))))..))))).)))))...............   reads    mm    sample
    .........ugagaaguaacuguuaaaacuaugu..................................................   6        0     FF2
    .........ugagaaguaacuguuaaaacuaugucgcc..............................................   1        0     FF2

    .......uuugagaaguaacuguuaaaacuaug...................................................   1        0     OV2
    .......uuugagaaguaacuguuaaaacuaugu..................................................   6        0     OV2
    ........uugagaaguaacuguuaaaacuaugu..................................................   1        0     OV2
    ........uugagaaguaacuguuaaaacuaugucgccgguugcgaug....................................   1        0     OV2
    .........ugagaaguaacuguuaaaacua.....................................................   5        0     OV2
    .........ugagaaguaacuguuaaaacuau....................................................   1        0     OV2
    .........ugagaaguaacuguuaaaacuaug...................................................   5        0     OV2
    .........ugagaaguaacuAuuaaaacuaugu..................................................   1        1     OV2
    .........ugagaaguaacuguuaaaacuaugu..................................................   269      0     OV2
    .........ugagaaguaacuguuaaaacuauguc.................................................   1        0     OV2
    .........ugagaaguaacuguuaaaacuaugucgc...............................................   5        0     OV2
    .........ugagaaguaacuguuaaaacuaugucgcc..............................................   5        0     OV2
    .........ugagaaguaacuguuaaaacuaugucgccggu...........................................   5        0     OV2
    .........ugagaaguaacuguuaaaacuaugucgccgguugcga......................................   2        0     OV2
    .........ugagaaguaacuguuaaaacuaugucgccgguugcgaug....................................   6        0     OV2
    ..........gagaaguaacuguuaaaacuaugu..................................................   1        0     OV2
    ....................uguuaaaacuaugucgccgguugcga......................................   1        0     OV2
    ....................uguuaaaacuaugucgccgguugcgaug....................................   4        0     OV2
    .......................uaaaacuaugucgccgguugcAaug....................................   1        1     OV2
    .......................uaaaacuaugucgccgguugcgaug....................................   4        0     OV2

    .....ucuuugagaaguaacuguuaaaacuaug...................................................   1        0     TE1
    .......uuugagaaguaacuguuaaaacuaug...................................................   1        0     TE1
```

```
                                                aga-mir-10358-5-star
                                                aga-mir-10358-3-star
uuuguucuu[illegible]aaaacuaugucgccgguugcgaugucauuuucuuuugcuccucgauguuugucugcguac
         aga-mir-10358-4
         aga-mir-10358-3
                                                aga-mir-10358-4-star
                                                aga-mir-10358-2-star
         aga-mir-10358-5
         aga-mir-10358-2
         aga-mir-10358-1
                                                aga-mir-10358-1-star


........uugagaaguaacuguuaaaacuaugu..................................................    2     0    TE1
.........ugagaaguaacuguuaaaac.......................................................    1     0    TE1
.........ugagaaguaacuguuaaaacua.....................................................    1     0    TE1
.........ugagaaguaacuguuaaaacuaug...................................................    4     0    TE1
.........ugagaaguaacuguuaaaacuaugu..................................................    146   0    TE1
.........ugagaaguaacuguuaaaacuauguc.................................................    4     0    TE1
.........ugagaaguaacuguuaaaacuaugucgc...............................................    1     0    TE1
.........ugagaaguaacuguuaaaacuaugucgcc..............................................    9     0    TE1
.........ugagaaguaacuguuaaaacuaugucgccU.............................................    2     1    TE1
.........ugagaaguaacuguuaaaacuaugucgccgg............................................    1     0    TE1
.........ugagaagCaacuguuaaaacuaugucgccggu...........................................    1     1    TE1
.........ugagaaguaacuguuaaaacuaugucgccggu...........................................    9     0    TE1
.........ugagaaguaacuguuaaaacuaugucgccgguugcgaug....................................    1     0    TE1
..........gagaaguaacuguuaaaacuaugu..................................................    1     0    TE1
............gaaguaacuguuaaaacuaugucgcc..............................................    4     0    TE1
............gaaguaacuguuaaaacuaugucgccggu...........................................    1     0    TE1
....................uguuaaaacuaugucgccgguugcgaug....................................    1     0    TE1
.......................uaaaacuaugucgccgguugcgaug....................................    1     0    TE1
..............................................ugucauuuucuuuugcuccucgauguu...........    1     0    TE1
....................................................uuucuuuugcuccucgauguuuguc.......    1     0    TE1

........uugagaaguaacuguuaaaacuaugu..................................................    1     0    MF2
.........ugagaaguaacuguuaaaacua.....................................................    3     0    MF2
.........ugagaaguaacuguuaaaacuaug...................................................    1     0    MF2
.........ugagaaguaacuguuaaaacuaugu..................................................    99    0    MF2
.........ugagaaguaacuguuaaaacuauguc.................................................    1     0    MF2
.........ugagaaguaacuguuaaaacuaugucgc...............................................    1     0    MF2
.........ugagaaguaacuguuaaaacuaugucgcc..............................................    2     0    MF2
....................uguuaaaacuaugucgccgguugcgaug....................................    1     0    MF2

.........ugagaaguaacuguuaaaacuaugu..................................................    1     0    FF1
.........ugagaaguaacuguuaaaacuaugucgcc..............................................    1     0    FF1

.uuguucuuugagaaguaacuguuaaaacuaugucgccggu...........................................    1     0    OV1
..uguucuuugagaaguaacuguuaaaacuaugucgccgguugcga......................................    1     0    OV1
....uucuuugagaaguaacuguuaaaacuaugucgccggu...........................................    1     0    OV1
.......uuugagaaguaacuguuaaaacuaug...................................................    2     0    OV1
.......uuugagaaguaacuguuaaaacuaugu..................................................    9     0    OV1
........uugagaaguaacuguuaaaacua.....................................................    1     0    OV1
........uugagaaguaacuguuaaaacuaugu..................................................    3     0    OV1
.........ugagaaguaacuguuaaaacua.....................................................    8     0    OV1
.........ugagaaguaacuguuaaaacuaug...................................................    12    0    OV1
.........ugagaaguaaUuguuaaaacuaugu..................................................    1     1    OV1
.........ugagaaguaacuguuaaaaAuaugu..................................................    1     1    OV1
.........ugagaaguaacuguuaaaacuaugu..................................................    357   0    OV1
.........ugagaaguaacuguuaaaacuauguc.................................................    8     0    OV1
.........ugagaaguaacuguuaaaacuaugucgc...............................................    1     0    OV1
.........ugagaaguaacuguuaaaacuaugucgcc..............................................    6     0    OV1
.........ugagaaguaacuguuaaaacuaugucgccggu...........................................    15    0    OV1
.........ugagaaguaacuguuaaaacuaugucgccgguugcgaug....................................    4     0    OV1
..............aguaacuguuaaaacuaugu..................................................    1     0    OV1
................uaacuguuaaaacuaugucgccggu...........................................    1     0    OV1
....................uguuaaaacuaugucgccgguugcg.......................................    1     0    OV1
....................uguuaaaacuaugucgccgguugcgaug....................................    5     0    OV1
.......................uaaaacuaugucgccgguugcgaug....................................    2     0    OV1
........................aaaacuaugucgccggu...........................................    1     0    OV1
..............................................ugucauuuucuuuugcuccucgauguu...........    3     0    OV1
................................................ucauuuucuuuugcuccucgauguuug.........    1     0    OV1
........................................................uuuugcuccucgauguuugucugcgu..    1     0    OV1

.........ugagaaguaacuguuaaaacuaugu..................................................    8     0    MF1
```

aga-mir-10358-5-star
aga-mir-10358-3-star
uuuguucuuugagaaguaacuguuaaaacuaugucgccgguugcgaugucauuuucuuuugcuccucgauguuugucugcguac
aga-mir-10358-4
aga-mir-10358-3
aga-mir-10358-4-star
aga-mir-10358-2-star
aga-mir-10358-5
aga-mir-10358-2
aga-mir-10358-1
aga-mir-10358-1-star

....................uguuaaaacuaugucgccgguugcgaug....................................	1	0	MF1
......uuugagaaguaacuguuaaaacuaugu...	3	0	BF2
.......uugagaaguaacuguuaaaacuaugu...	1	0	BF2
.........ugagaaguaacuguuaaaacua...	2	0	BF2
.........ugagaaguaacuguuaaaacuau..	1	0	BF2
.........ugagaaguaacuguuaaaacuaug...	4	0	BF2
.........ugagaAuaacuguuaaaacuaugu..	1	1	BF2
.........ugagaaguaacuguuaaaacuaugu..	158	0	BF2
.........ugagaaguaacuguuaaaacuauguc...	5	0	BF2
.........ugagaaguaacuguuaaaacuaugucg..	1	0	BF2
.........ugagaaguaacuguuaaaacuaugucgc...	1	0	BF2
.........ugagaaguaacuguuaaaacuaugucgcc..	1	0	BF2
.........ugagaaguaacuguuaaaacuaugucgccggu...	4	0	BF2
.........ugagaaguaacuguuaaaacuaugucgccgguugcgaug....................................	1	0	BF2
............gaaguaacuguuaaaacuaugucgcc..	2	0	BF2
................uaacuguuaaaacuaugucgccgg..	1	0	BF2
................uaacuguuaaaacuaugucgccggu...	1	0	BF2
....................uguuaaaacuaugucgccgguugcgaug....................................	1	0	BF2
....ugcuccucgauguuugucugcguac	1	0	BF2
......uuugagaaguaacuguuaaaacuaugu...	4	0	BF1
.......uugagaaguaacuguuaaaacuaugu...	1	0	BF1
.......uugagaaguaacuguuaaaacuaugucgcc..	1	0	BF1
.........ugagaaguaacuguuaaaacua...	5	0	BF1
.........ugagaaguaacuguuaaaacuaug...	5	0	BF1
.........ugagaaguaaAuguuaaaacuaugu..	1	1	BF1
.........ugagaaguaacuguCaaaacuaugu..	1	1	BF1
.........ugagaaguaacuguuaaaacuaugu..	153	0	BF1
.........ugagaaguaacuguuaaaacuauguc...	6	0	BF1
.........ugagaaguaacuguuaaaacuaugucg..	1	0	BF1
.........ugagaaguaacuguuaaaacuaugucgc...	1	0	BF1
.........ugagaaguaacuguuaaaacuaugucgcc..	2	0	BF1
.........ugagaaguaacuguuaaaacuaugucgccU...	1	1	BF1
.........ugagaaguaacuguuaaaacuaugucgccggu...	4	0	BF1
...........agaaguaacuguuaaaacuaugucgcc..	1	0	BF1
............gaaguaacuguuaaaacuaugucgc...	1	0	BF1
............gaaguaacuguuaaaacuaugucgcc..	1	0	BF1
....uuuucuuuugcuccucgauguuugucu......	1	0	BF1
....uuucuuuugcuccucgauguuugucu......	1	0	BF1
....uucuuuugcuccucgauguuugucugcg...	2	0	BF1
.........ugagaaguaacuguuaaaacuaugu..	1	0	MW1
.........ugagaaguaacuguuaaaacuaugu..	1	0	FW1
.......uugagaaguaacuguuaaaacuaugu...	2	0	TE2
.........ugagaaguaacuguuaaaacua...	2	0	TE2
.........Cgagaaguaacuguuaaaacuaug...	1	1	TE2
.........ugagaaguaacuguuaaaacuaug...	6	0	TE2
.........ugaCaaguaacuguuaaaacuaugu..	1	1	TE2
.........ugagaaguaacuguuaaaacuaugC..	1	1	TE2
.........ugagaaguaacuguuaaaacuUugu..	1	1	TE2
.........ugagaaguaacuguuaaaacuaugu..	272	0	TE2
.........ugagaaguaacuguuaaaacuaugG..	1	1	TE2
.........ugagaaguaacAguuaaaacuaugu..	1	1	TE2
.........ugagaaguaacuguuaaaacuauguc...	4	0	TE2
.........ugagaaguaacuguuaaaacuaugucg..	1	0	TE2
.........ugagaaguaacuguuaaaacuaugucgc...	6	0	TE2
.........ugagaaguaacuguuaaaacuaugucgcc..	7	0	TE2
.........ugagaaguaacuguuaaaacuaugucgccU...	2	1	TE2

```
                                                aga-mir-10358-5-star
                                                aga-mir-10358-3-star
uuuguucuuaga-mir-10358-4aaaacuaugucgccgguugcgaugucauuuucuuuugcuccucgauguuugucugcguac
         aga-mir-10358-3
                                                aga-mir-10358-4-star
                                                aga-mir-10358-2-star
         aga-mir-10358-5
         aga-mir-10358-2
         aga-mir-10358-1
                                                aga-mir-10358-1-star


.........ugagaaguaacuguuaaaacuaugucgccggu...........................................    10         0         TE2
..........gagaaguaacuguuaaaacua.....................................................    1          0         TE2
............gaaguaacuguuaaaacuaugucgcc..............................................    1          0         TE2
.......................uaaaacuaugucgccgguugcgaug....................................    1          0         TE2
..........................................................uugcuccucgauguuugucugcguac    1          0         TE2
```

miRBase precursor : aga-mir-10358-2
Total read count : 8585
aga-mir-10358-1 read count: 1689
aga-mir-10358-1-star read count: 8
aga-mir-10358-2 read count: 1689
aga-mir-10358-2-star read count: 8
aga-mir-10358-3 read count: 1689
aga-mir-10358-3-star read count: 8
aga-mir-10358-4 read count: 1689
aga-mir-10358-4-star read count: 8
aga-mir-10358-5 read count: 1689
aga-mir-10358-5-star read count: 8
remaining reads : 100

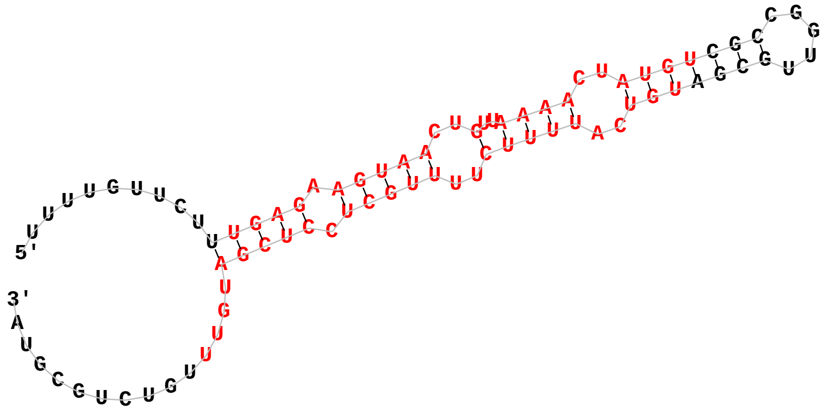

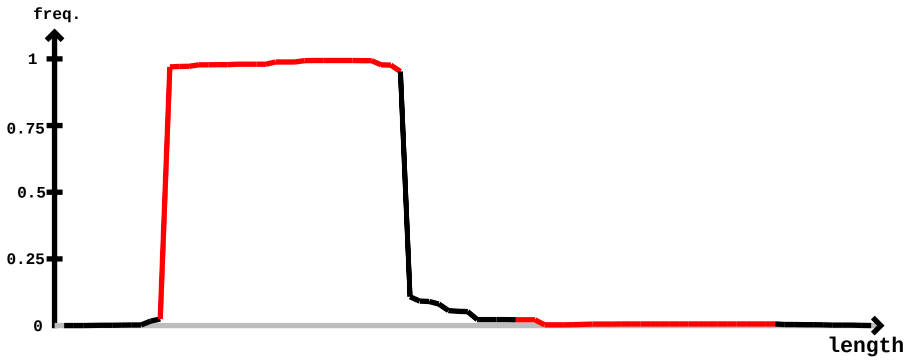


```
                                                aga-mir-10358-5-star
                                                aga-mir-10358-3-star
          aga-mir-10358-4
          aga-mir-10358-3
                                                aga-mir-10358-4-star
                                                aga-mir-10358-2-star
          aga-mir-10358-5
          aga-mir-10358-2
          aga-mir-10358-1
                                                aga-mir-10358-1-star
5'- uuuuguucuuugagaaguaacuguuaaaacuaugucgccgguugcgaugucauuuucuuuugcuccucgauguuugucugcgua  -3'   exp
    .........(((((.(((((..(..((((..(((((((.....)))))))..)))))..))))).)))))..............    reads      mm       sample
    ..........ugagaaguaacuguuaaaacuaugu.................................................    6          0        FF2
    ..........ugagaaguaacuguuaaaacuaugucgcc.............................................    1          0        FF2

    ........uuugagaaguaacuguuaaaacuaug..................................................    1          0        OV2
    ........uuugagaaguaacuguuaaaacuaugu.................................................    6          0        OV2
    .........uugagaaguaacuguuaaaacuaugu.................................................    1          0        OV2
    .........uugagaaguaacuguuaaaacuaugucgccgguugcgaug...................................    1          0        OV2
    ..........ugagaaguaacuguuaaaacua....................................................    5          0        OV2
    ..........ugagaaguaacuguuaaaacuau...................................................    1          0        OV2
    ..........ugagaaguaacuguuaaaacuaug..................................................    5          0        OV2
    ..........ugagaaguaacuAuuaaaacuaugu.................................................    1          1        OV2
    ..........ugagaaguaacuguuaaaacuaugu.................................................    269        0        OV2
    ..........ugagaaguaacuguuaaaacuauguc................................................    1          0        OV2
    ..........ugagaaguaacuguuaaaacuaugucgc..............................................    5          0        OV2
    ..........ugagaaguaacuguuaaaacuaugucgcc.............................................    5          0        OV2
    ..........ugagaaguaacuguuaaaacuaugucgccggu..........................................    5          0        OV2
    ..........ugagaaguaacuguuaaaacuaugucgccgguugcga.....................................    2          0        OV2
    ..........ugagaaguaacuguuaaaacuaugucgccgguugcgaug...................................    6          0        OV2
    ...........gagaaguaacuguuaaaacuaugu.................................................    1          0        OV2
    .....................uguuaaaacuaugucgccgguugcga.....................................    1          0        OV2
    .....................uguuaaaacuaugucgccgguugcgaug...................................    4          0        OV2
    ........................uaaaacuaugucgccgguugcAaug...................................    1          1        OV2
    ........................uaaaacuaugucgccgguugcgaug...................................    4          0        OV2

    ......ucuuugagaaguaacuguuaaaacuaug..................................................    1          0        TE1
    ........uuugagaaguaacuguuaaaacuaug..................................................    1          0        TE1
```

```
                                            aga-mir-10358-5-star
                                            aga-mir-10358-3-star
          aga-mir-10358-4
uuuuguucuuugagaaguaacuguuaaaacuaugucgccgguugcgaugucauuuucuuuugcuccucgauguuugucugcgua
          aga-mir-10358-3
                                            aga-mir-10358-4-star
                                            aga-mir-10358-2-star
          aga-mir-10358-5
          aga-mir-10358-2
          aga-mir-10358-1
                                            aga-mir-10358-1-star


.........uugagaaguaacuguuaaaacuaugu.................................................    2      0      TE1
..........ugagaaguaacuguuaaaac......................................................    1      0      TE1
..........ugagaaguaacuguuaaaacua....................................................    1      0      TE1
..........ugagaaguaacuguuaaaacuaug..................................................    4      0      TE1
..........ugagaaguaacuguuaaaacuaugu.................................................    146    0      TE1
..........ugagaaguaacuguuaaaacuauguc................................................    4      0      TE1
..........ugagaaguaacuguuaaaacuaugucgc..............................................    1      0      TE1
..........ugagaaguaacuguuaaaacuaugucgcc.............................................    9      0      TE1
..........ugagaaguaacuguuaaaacuaugucgccU............................................    2      1      TE1
..........ugagaaguaacuguuaaaacuaugucgccgg...........................................    1      0      TE1
..........ugagaaguaacuguuaaaacuaugucgccggu..........................................    9      0      TE1
..........ugagaagCaacuguuaaaacuaugucgccggu..........................................    1      1      TE1
..........ugagaaguaacuguuaaaacuaugucgccgguugcgaug...................................    1      0      TE1
...........gagaaguaacuguuaaaacuaugu.................................................    1      0      TE1
.............gaaguaacuguuaaaacuaugucgcc.............................................    4      0      TE1
.............gaaguaacuguuaaaacuaugucgccggu..........................................    1      0      TE1
.....................uguuaaaacuaugucgccgguugcgaug...................................    1      0      TE1
........................uaaaacuaugucgccgguugcgaug...................................    1      0      TE1
...............................................ugucauuuucuuuugcuccucgauguu..........    1      0      TE1
.....................................................uuucuuuugcuccucgauguuuguc......    1      0      TE1

.........uugagaaguaacuguuaaaacuaugu.................................................    1      0      MF2
..........ugagaaguaacuguuaaaacua....................................................    3      0      MF2
..........ugagaaguaacuguuaaaacuaug..................................................    1      0      MF2
..........ugagaaguaacuguuaaaacuaugu.................................................    99     0      MF2
..........ugagaaguaacuguuaaaacuauguc................................................    1      0      MF2
..........ugagaaguaacuguuaaaacuaugucgc..............................................    1      0      MF2
..........ugagaaguaacuguuaaaacuaugucgcc.............................................    2      0      MF2
.....................uguuaaaacuaugucgccgguugcgaug...................................    1      0      MF2

..........ugagaaguaacuguuaaaacuaugu.................................................    1      0      FF1
..........ugagaaguaacuguuaaaacuaugucgcc.............................................    1      0      FF1

..uuguucuuugagaaguaacuguuaaaacuaugucgccggu..........................................    1      0      OV1
...uguucuuugagaaguaacuguuaaaacuaugucgccgguugcga.....................................    1      0      OV1
.....uucuuugagaaguaacuguuaaaacuaugucgccggu..........................................    1      0      OV1
........uuugagaaguaacuguuaaaacuaug..................................................    2      0      OV1
........uuugagaaguaacuguuaaaacuaugu.................................................    9      0      OV1
.........uugagaaguaacuguuaaaacua....................................................    1      0      OV1
.........uugagaaguaacuguuaaaacuaugu.................................................    3      0      OV1
..........ugagaaguaacuguuaaaacua....................................................    8      0      OV1
..........ugagaaguaacuguuaaaacuaug..................................................    12     0      OV1
..........ugagaaguaacuguuaaaaAuaugu.................................................    1      1      OV1
..........ugagaaguaacuguuaaaacuaugu.................................................    357    0      OV1
..........ugagaaguaaUuguuaaaacuaugu.................................................    1      1      OV1
..........ugagaaguaacuguuaaaacuauguc................................................    8      0      OV1
..........ugagaaguaacuguuaaaacuaugucgc..............................................    1      0      OV1
..........ugagaaguaacuguuaaaacuaugucgcc.............................................    6      0      OV1
..........ugagaaguaacuguuaaaacuaugucgccggu..........................................    15     0      OV1
..........ugagaaguaacuguuaaaacuaugucgccgguugcgaug...................................    4      0      OV1
...............aguaacuguuaaaacuaugu.................................................    1      0      OV1
.................uaacuguuaaaacuaugucgccggu..........................................    1      0      OV1
.....................uguuaaaacuaugucgccgguugcg......................................    1      0      OV1
.....................uguuaaaacuaugucgccgguugcgaug...................................    5      0      OV1
........................uaaaacuaugucgccgguugcgaug...................................    2      0      OV1
.........................aaaacuaugucgccggu..........................................    1      0      OV1
...............................................ugucauuuucuuuugcuccucgauguu..........    3      0      OV1
.................................................ucauuuucuuuugcuccucgauguuug........    1      0      OV1
.........................................................uuuugcuccucgauguuugucugcgu.    1      0      OV1

..........ugagaaguaacuguuaaaacuaugu.................................................    8      0      MF1
```

aga-mir-10358-5-star
aga-mir-10358-3-star
uuuguucuuugagaaguaacuguuaaaacuaugucgccgguugcgaugucauuuucuuuugcuccucgauguuugucugcgua
aga-mir-10358-4
aga-mir-10358-3
aga-mir-10358-4-star
aga-mir-10358-2-star
aga-mir-10358-5
aga-mir-10358-2
aga-mir-10358-1
aga-mir-10358-1-star

....................uguuaaaacuaugucgccgguugcgaug...................................	1	0	MF1
.......uuugagaaguaacuguuaaaacuaugu...	3	0	BF2
........uugagaaguaacuguuaaaacuaugu...	1	0	BF2
.........ugagaaguaacuguuaaaacua..	2	0	BF2
.........ugagaaguaacuguuaaaacuau...	1	0	BF2
.........ugagaaguaacuguuaaaacuaug..	4	0	BF2
.........ugagaaguaacuguuaaaacuaugu...	158	0	BF2
.........ugagaaAuaacuguuaaaacuaugu...	1	1	BF2
.........ugagaaguaacuguuaaaacuauguc..	5	0	BF2
.........ugagaaguaacuguuaaaacuaugucg...	1	0	BF2
.........ugagaaguaacuguuaaaacuaugucgc..	1	0	BF2
.........ugagaaguaacuguuaaaacuaugucgcc...	1	0	BF2
.........ugagaaguaacuguuaaaacuaugucgccggu..	4	0	BF2
.........ugagaaguaacuguuaaaacuaugucgccgguugcgaug...................................	1	0	BF2
............gaaguaacuguuaaaacuaugucgcc...	2	0	BF2
................uaacuguuaaaacuaugucgccgg...	1	0	BF2
................uaacuguuaaaacuaugucgccggu..	1	0	BF2
....................uguuaaaacuaugucgccgguugcgaug...................................	1	0	BF2
.......uuugagaaguaacuguuaaaacuaugu...	4	0	BF1
........uugagaaguaacuguuaaaacuaugu...	1	0	BF1
........uugagaaguaacuguuaaaacuaugucgcc...	1	0	BF1
.........ugagaaguaacuguuaaaacua..	5	0	BF1
.........ugagaaguaacuguuaaaacuaug..	5	0	BF1
.........ugagaaguaacuguCaaaacuaugu...	1	1	BF1
.........ugagaaguaaAuguuaaaacuaugu...	1	1	BF1
.........ugagaaguaacuguuaaaacuaugu...	153	0	BF1
.........ugagaaguaacuguuaaaacuauguc..	6	0	BF1
.........ugagaaguaacuguuaaaacuaugucg...	1	0	BF1
.........ugagaaguaacuguuaaaacuaugucgc..	1	0	BF1
.........ugagaaguaacuguuaaaacuaugucgcc...	2	0	BF1
.........ugagaaguaacuguuaaaacuaugucgccU..	1	1	BF1
.........ugagaaguaacuguuaaaacuaugucgccggu..	4	0	BF1
...........agaaguaacuguuaaaacuaugucgcc...	1	0	BF1
............gaaguaacuguuaaaacuaugucgc..	1	0	BF1
............gaaguaacuguuaaaacuaugucgcc...	1	0	BF1
....uuuucuuuugcuccucgauguuugucu.....	1	0	BF1
....uuucuuuugcuccucgauguuugucu.....	1	0	BF1
....uucuuuugcuccucgauguuugucugcg..	2	0	BF1
.........ugagaaguaacuguuaaaacuaugu...	1	0	FW1
.........ugagaaguaacuguuaaaacuaugu...	1	0	MW1
........uugagaaguaacuguuaaaacuaugu...	2	0	TE2
.........ugagaaguaacuguuaaaacua..	2	0	TE2
.........Cgagaaguaacuguuaaaacuaug..	1	1	TE2
.........ugagaaguaacuguuaaaacuaug..	6	0	TE2
.........ugagaaguaacuguuaaaacuaugC...	1	1	TE2
.........ugagaaguaacuguuaaaacuaugu...	272	0	TE2
.........ugagaaguaacAguuaaaacuaugu...	1	1	TE2
.........ugagaaguaacuguuaaaacuaugG...	1	1	TE2
.........ugaCaaguaacuguuaaaacuaugu...	1	1	TE2
.........ugagaaguaacuguuaaaacuUugu...	1	1	TE2
.........ugagaaguaacuguuaaaacuauguc..	4	0	TE2
.........ugagaaguaacuguuaaaacuaugucg...	1	0	TE2
.........ugagaaguaacuguuaaaacuaugucgc..	6	0	TE2
.........ugagaaguaacuguuaaaacuaugucgcc...	7	0	TE2
.........ugagaaguaacuguuaaaacuaugucgccU..	2	1	TE2
.........ugagaaguaacuguuaaaacuaugucgccggu..	10	0	TE2

```
                                               aga-mir-10358-5-star
                                               aga-mir-10358-3-star
uuuuguucuu[illegible]aaaacuaugucgccgguugcgaugucauuuucuuuugcuccucgauguuugucugcgua
          aga-mir-10358-4
          aga-mir-10358-3
                                               aga-mir-10358-4-star
                                               aga-mir-10358-2-star
          aga-mir-10358-5
          aga-mir-10358-2
          aga-mir-10358-1
                                               aga-mir-10358-1-star
```

...........gagaaguaacuguuaaaacua..	1	0	TE2
.............gaaguaacuguuaaaacuaugucgcc...	1	0	TE2
........................uaaaacuaugucgccgguugcgaug...................................	1	0	TE2

miRBase precursor : aga-mir-10358-3
Total read count : 8585
aga-mir-10358-1 read count: 1689
aga-mir-10358-1-star read count: 8
aga-mir-10358-2 read count: 1689
aga-mir-10358-2-star read count: 8
aga-mir-10358-3 read count: 1689
aga-mir-10358-3-star read count: 8
aga-mir-10358-4 read count: 1689
aga-mir-10358-4-star read count: 8
aga-mir-10358-5 read count: 1689
aga-mir-10358-5-star read count: 8
remaining reads : 100

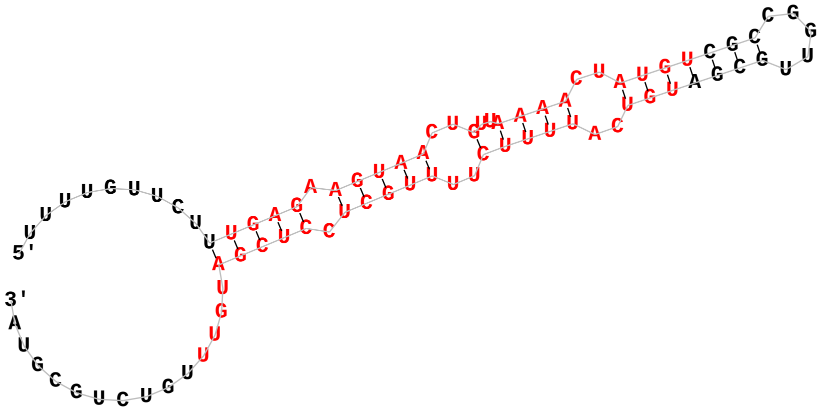

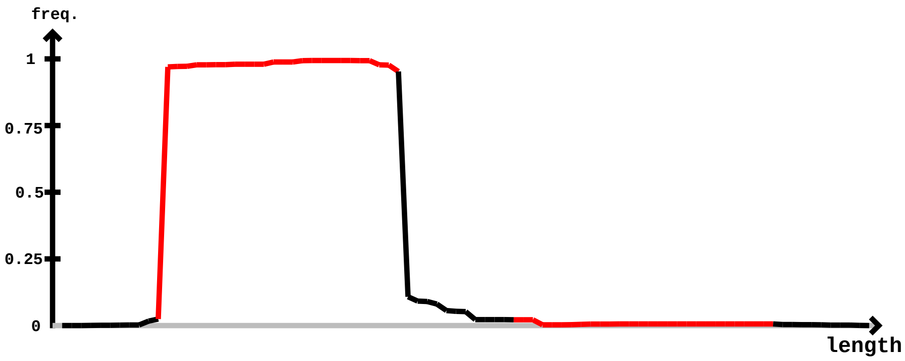

aga-mir-10358-5-star
aga-mir-10358-3-star
aga-mir-10358-4
aga-mir-10358-3
aga-mir-10358-4-star
aga-mir-10358-2-star
aga-mir-10358-5
aga-mir-10358-2
aga-mir-10358-1
aga-mir-10358-1-star

```
5'- uuuuguucuuugagaaguaacuguuaaaacuaugucgccgguugcgaugucauuuucuuuugcuccucgauguuugucugcgua  -3'    exp
    .........(((((.(((((..(..((((..(((((((.....)))))))..))))).))))).))))).............   reads      mm         sample
    .........ugagaaguaacuguuaaaacuaugu.............................................   6          0          FF2
    .........ugagaaguaacuguuaaaacuaugucgcc.........................................   1          0          FF2

    .......uuugagaaguaacuguuaaaacuaug..............................................   1          0          OV2
    .......uuugagaaguaacuguuaaaacuaugu.............................................   6          0          OV2
    ........uugagaaguaacuguuaaaacuaugu.............................................   1          0          OV2
    ........uugagaaguaacuguuaaaacuaugucgccgguugcgaug...............................   1          0          OV2
    .........ugagaaguaacuguuaaaacua................................................   5          0          OV2
    .........ugagaaguaacuguuaaaacuau...............................................   1          0          OV2
    .........ugagaaguaacuguuaaaacuaug..............................................   5          0          OV2
    .........ugagaaguaacuAuuaaaacuaugu.............................................   1          1          OV2
    .........ugagaaguaacuguuaaaacuaugu.............................................   269        0          OV2
    .........ugagaaguaacuguuaaaacuauguc............................................   1          0          OV2
    .........ugagaaguaacuguuaaaacuaugucgc..........................................   5          0          OV2
    .........ugagaaguaacuguuaaaacuaugucgcc.........................................   5          0          OV2
    .........ugagaaguaacuguuaaaacuaugucgccggu......................................   5          0          OV2
    .........ugagaaguaacuguuaaaacuaugucgccgguugcga.................................   2          0          OV2
    .........ugagaaguaacuguuaaaacuaugucgccgguugcgaug...............................   6          0          OV2
    ..........gagaaguaacuguuaaaacuaugu.............................................   1          0          OV2
    ...................uguuaaaacuaugucgccgguugcga..................................   1          0          OV2
    ...................uguuaaaacuaugucgccgguugcgaug................................   4          0          OV2
    ......................uaaaacuaugucgccgguugcAaug................................   1          1          OV2
    ......................uaaaacuaugucgccgguugcgaug................................   4          0          OV2

    .....ucuuugagaaguaacuguuaaaacuaug..............................................   1          0          TE1
    .......uuugagaaguaacuguuaaaacuaug..............................................   1          0          TE1
```

```
                                               aga-mir-10358-5-star
                                               aga-mir-10358-3-star
          aga-mir-10358-4
uuuuguucuuugagaaguaacuguuaaaacuaugucgccgguugcgaugucauuuucuuuugcuccucgauguuugucugcgua
          aga-mir-10358-3
                                               aga-mir-10358-4-star
                                               aga-mir-10358-2-star
          aga-mir-10358-5
          aga-mir-10358-2
          aga-mir-10358-1
                                               aga-mir-10358-1-star


.........uugagaaguaacuguuaaaacuaugu.................................................    2     0    TE1
..........ugagaaguaacuguuaaaac......................................................    1     0    TE1
..........ugagaaguaacuguuaaaacua....................................................    1     0    TE1
..........ugagaaguaacuguuaaaacuaug..................................................    4     0    TE1
..........ugagaaguaacuguuaaaacuaugu.................................................    146   0    TE1
..........ugagaaguaacuguuaaaacuauguc................................................    4     0    TE1
..........ugagaaguaacuguuaaaacuaugucgc..............................................    1     0    TE1
..........ugagaaguaacuguuaaaacuaugucgcc.............................................    9     0    TE1
..........ugagaaguaacuguuaaaacuaugucgccU............................................    2     1    TE1
..........ugagaaguaacuguuaaaacuaugucgccgg...........................................    1     0    TE1
..........ugagaaguaacuguuaaaacuaugucgccggu..........................................    9     0    TE1
..........ugagaagCaacuguuaaaacuaugucgccggu..........................................    1     1    TE1
..........ugagaaguaacuguuaaaacuaugucgccgguugcgaug...................................    1     0    TE1
...........gagaaguaacuguuaaaacuaugu.................................................    1     0    TE1
.............gaaguaacuguuaaaacuaugucgcc.............................................    4     0    TE1
.............gaaguaacuguuaaaacuaugucgccggu..........................................    1     0    TE1
.....................uguuaaaacuaugucgccgguugcgaug...................................    1     0    TE1
........................uaaaacuaugucgccgguugcgaug...................................    1     0    TE1
...............................................ugucauuuucuuuugcuccucgauguu..........    1     0    TE1
.....................................................uuucuuuugcuccucgauguuuguc......    1     0    TE1

.........uugagaaguaacuguuaaaacuaugu.................................................    1     0    MF2
..........ugagaaguaacuguuaaaacua....................................................    3     0    MF2
..........ugagaaguaacuguuaaaacuaug..................................................    1     0    MF2
..........ugagaaguaacuguuaaaacuaugu.................................................    99    0    MF2
..........ugagaaguaacuguuaaaacuauguc................................................    1     0    MF2
..........ugagaaguaacuguuaaaacuaugucgc..............................................    1     0    MF2
..........ugagaaguaacuguuaaaacuaugucgcc.............................................    2     0    MF2
.....................uguuaaaacuaugucgccgguugcgaug...................................    1     0    MF2

..........ugagaaguaacuguuaaaacuaugu.................................................    1     0    FF1
..........ugagaaguaacuguuaaaacuaugucgcc.............................................    1     0    FF1

..uuguucuuugagaaguaacuguuaaaacuaugucgccggu..........................................    1     0    OV1
...uguucuuugagaaguaacuguuaaaacuaugucgccgguugcga.....................................    1     0    OV1
.....uucuuugagaaguaacuguuaaaacuaugucgccggu..........................................    1     0    OV1
........uuugagaaguaacuguuaaaacuaug..................................................    2     0    OV1
........uuugagaaguaacuguuaaaacuaugu.................................................    9     0    OV1
.........uugagaaguaacuguuaaaacua....................................................    1     0    OV1
.........uugagaaguaacuguuaaaacuaugu.................................................    3     0    OV1
..........ugagaaguaacuguuaaaacua....................................................    8     0    OV1
..........ugagaaguaacuguuaaaacuaug..................................................    12    0    OV1
..........ugagaaguaacuguuaaaaAuaugu.................................................    1     1    OV1
..........ugagaaguaacuguuaaaacuaugu.................................................    357   0    OV1
..........ugagaaguaaUuguuaaaacuaugu.................................................    1     1    OV1
..........ugagaaguaacuguuaaaacuauguc................................................    8     0    OV1
..........ugagaaguaacuguuaaaacuaugucgc..............................................    1     0    OV1
..........ugagaaguaacuguuaaaacuaugucgcc.............................................    6     0    OV1
..........ugagaaguaacuguuaaaacuaugucgccggu..........................................    15    0    OV1
..........ugagaaguaacuguuaaaacuaugucgccgguugcgaug...................................    4     0    OV1
...............aguaacuguuaaaacuaugu.................................................    1     0    OV1
.................uaacuguuaaaacuaugucgccggu..........................................    1     0    OV1
.....................uguuaaaacuaugucgccgguugcg......................................    1     0    OV1
.....................uguuaaaacuaugucgccgguugcgaug...................................    5     0    OV1
........................uaaaacuaugucgccgguugcgaug...................................    2     0    OV1
.........................aaaacuaugucgccggu..........................................    1     0    OV1
...............................................ugucauuuucuuuugcuccucgauguu..........    3     0    OV1
.................................................ucauuuucuuuugcuccucgauguuug........    1     0    OV1
.........................................................uuuugcuccucgauguuugucugcgu.    1     0    OV1

..........ugagaaguaacuguuaaaacuaugu.................................................    8     0    MF1
```

aga-mir-10358-5-star
aga-mir-10358-3-star
uuuguucuu[illegible]aaaacuaugucgccgguugcgaugucauuuucuuuugcuccucgauguuugucugcgua
aga-mir-10358-4
aga-mir-10358-3
aga-mir-10358-4-star
aga-mir-10358-2-star
aga-mir-10358-5
aga-mir-10358-2
aga-mir-10358-1
aga-mir-10358-1-star

....................uguuaaaacuaugucgccgguugcgaug...................................	1	0	MF1
.......uuugagaaguaacuguuaaaacuaugu...	3	0	BF2
........uugagaaguaacuguuaaaacuaugu...	1	0	BF2
.........ugagaaguaacuguuaaaacua..	2	0	BF2
.........ugagaaguaacuguuaaaacuau...	1	0	BF2
.........ugagaaguaacuguuaaaacuaug..	4	0	BF2
.........ugagaaguaacuguuaaaacuaugu...	158	0	BF2
.........ugagaAuaacuguuaaaacuaugu...	1	1	BF2
.........ugagaaguaacuguuaaaacuauguc..	5	0	BF2
.........ugagaaguaacuguuaaaacuaugucg...	1	0	BF2
.........ugagaaguaacuguuaaaacuaugucgc..	1	0	BF2
.........ugagaaguaacuguuaaaacuaugucgcc...	1	0	BF2
.........ugagaaguaacuguuaaaacuaugucgccggu..	4	0	BF2
.........ugagaaguaacuguuaaaacuaugucgccgguugcgaug...................................	1	0	BF2
............gaaguaacuguuaaaacuaugucgcc...	2	0	BF2
................uaacuguuaaaacuaugucgccgg...	1	0	BF2
................uaacuguuaaaacuaugucgccggu..	1	0	BF2
....................uguuaaaacuaugucgccgguugcgaug...................................	1	0	BF2
.......uuugagaaguaacuguuaaaacuaugu...	4	0	BF1
........uugagaaguaacuguuaaaacuaugu...	1	0	BF1
........uugagaaguaacuguuaaaacuaugucgcc...	1	0	BF1
.........ugagaaguaacuguuaaaacua..	5	0	BF1
.........ugagaaguaacuguuaaaacuaug..	5	0	BF1
.........ugagaaguaacuguCaaaacuaugu...	1	1	BF1
.........ugagaaguaaAuguuaaaacuaugu...	1	1	BF1
.........ugagaaguaacuguuaaaacuaugu...	153	0	BF1
.........ugagaaguaacuguuaaaacuauguc..	6	0	BF1
.........ugagaaguaacuguuaaaacuaugucg...	1	0	BF1
.........ugagaaguaacuguuaaaacuaugucgc..	1	0	BF1
.........ugagaaguaacuguuaaaacuaugucgcc...	2	0	BF1
.........ugagaaguaacuguuaaaacuaugucgccU..	1	1	BF1
.........ugagaaguaacuguuaaaacuaugucgccggu..	4	0	BF1
...........agaaguaacuguuaaaacuaugucgcc...	1	0	BF1
............gaaguaacuguuaaaacuaugucgc..	1	0	BF1
............gaaguaacuguuaaaacuaugucgcc...	1	0	BF1
....uuuucuuuugcuccucgauguuugucu.....	1	0	BF1
....uuucuuuugcuccucgauguuugucu.....	1	0	BF1
....uucuuuugcuccucgauguuugucugcg..	2	0	BF1
.........ugagaaguaacuguuaaaacuaugu...	1	0	FW1
.........ugagaaguaacuguuaaaacuaugu...	1	0	MW1
........uugagaaguaacuguuaaaacuaugu...	2	0	TE2
.........ugagaaguaacuguuaaaacua..	2	0	TE2
.........Cgagaaguaacuguuaaaacuaug..	1	1	TE2
.........ugagaaguaacuguuaaaacuaug..	6	0	TE2
.........ugagaaguaacuguuaaaacuaugC...	1	1	TE2
.........ugagaaguaacuguuaaaacuaugu...	272	0	TE2
.........ugagaaguaacAguuaaaacuaugu...	1	1	TE2
.........ugagaaguaacuguuaaaacuaugG...	1	1	TE2
.........ugaCaaguaacuguuaaaacuaugu...	1	1	TE2
.........ugagaaguaacuguuaaaacuUugu...	1	1	TE2
.........ugagaaguaacuguuaaaacuauguc..	4	0	TE2
.........ugagaaguaacuguuaaaacuaugucg...	1	0	TE2
.........ugagaaguaacuguuaaaacuaugucgc..	6	0	TE2
.........ugagaaguaacuguuaaaacuaugucgcc...	7	0	TE2
.........ugagaaguaacuguuaaaacuaugucgccU..	2	1	TE2
.........ugagaaguaacuguuaaaacuaugucgccggu..	10	0	TE2

```
                                               aga-mir-10358-5-star
                                               aga-mir-10358-3-star
uuuuguucuuagagaaguaacuguuaaaacuaugucgccgguugcgaugucauuuucuuuugcuccucgauguuugucugcgua
          aga-mir-10358-4
          aga-mir-10358-3
                                               aga-mir-10358-4-star
                                               aga-mir-10358-2-star
          aga-mir-10358-5
          aga-mir-10358-2
          aga-mir-10358-1
                                               aga-mir-10358-1-star

...........gagaaguaacuguuaaaacua....................................................    1    0    TE2
.............gaaguaacuguuaaaacuaugucgcc.............................................    1    0    TE2
........................uaaaacuaugucgccgguugcgaug...................................    1    0    TE2
```

miRBase precursor : aga-mir-10358-4
Total read count : 8587
aga-mir-10358-1 read count: 1689
aga-mir-10358-1-star read count: 0
aga-mir-10358-2 read count: 1689
aga-mir-10358-2-star read count: 0
aga-mir-10358-3 read count: 1689
aga-mir-10358-3-star read count: 0
aga-mir-10358-4 read count: 1689
aga-mir-10358-4-star read count: 0
aga-mir-10358-5 read count: 1689
aga-mir-10358-5-star read count: 0
remaining reads : 102

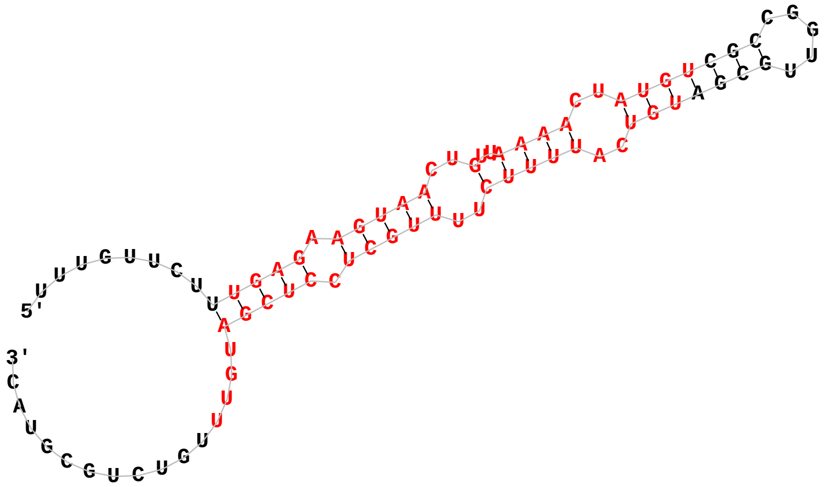

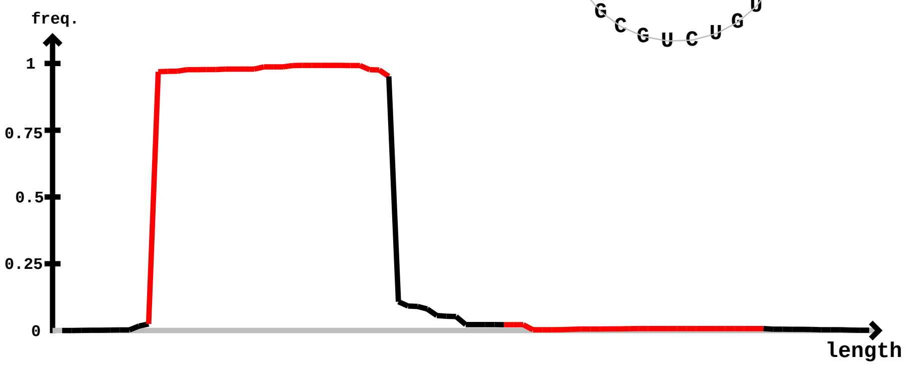


```
                                         aga-mir-10358-5-star
                                         aga-mir-10358-3-star
            aga-mir-10358-4
            aga-mir-10358-3
                                         aga-mir-10358-4-star
                                         aga-mir-10358-2-star
            aga-mir-10358-5
            aga-mir-10358-2
            aga-mir-10358-1
                                         aga-mir-10358-1-star
5'- uuuguucuuugagaagaguaacuguuaaaacuaugucgccgguugcgaugucauuuucuuuugcuccucgauguuugucugcguac  -3'   exp
    ........(((((.(((((..(..((((..(((((((.....)))))))..)))))..))))).)))))..............   reads     mm        sample
    .........ugagaagaguaacuguuaaaacuaugu...............................................   6         0         FF2
    .........ugagaagaguaacuguuaaaacuaugucgcc...........................................   1         0         FF2

    .......uuugagaagaguaacuguuaaaacuaug................................................   1         0         OV2
    .......uuugagaagaguaacuguuaaaacuaugu...............................................   6         0         OV2
    ........uugagaagaguaacuguuaaaacuaugu...............................................   1         0         OV2
    ........uugagaagaguaacuguuaaaacuaugucgccgguugcgaug.................................   1         0         OV2
    .........ugagaagaguaacuguuaaaacua..................................................   5         0         OV2
    .........ugagaagaguaacuguuaaaacuau.................................................   1         0         OV2
    .........ugagaagaguaacuguuaaaacuaug................................................   5         0         OV2
    .........ugagaagaguaacuAuuaaaacuaugu...............................................   1         1         OV2
    .........ugagaagaguaacuguuaaaacuaugu...............................................   269       0         OV2
    .........ugagaagaguaacuguuaaaacuauguc..............................................   1         0         OV2
    .........ugagaagaguaacuguuaaaacuaugucgc............................................   5         0         OV2
    .........ugagaagaguaacuguuaaaacuaugucgcc...........................................   5         0         OV2
    .........ugagaagaguaacuguuaaaacuaugucgccggu........................................   5         0         OV2
    .........ugagaagaguaacuguuaaaacuaugucgccgguugcga...................................   2         0         OV2
    .........ugagaagaguaacuguuaaaacuaugucgccgguugcgaug.................................   6         0         OV2
    ..........gagaagaguaacuguuaaaacuaugu...............................................   1         0         OV2
    ....................uguuaaaacuaugucgccgguugcga.....................................   1         0         OV2
    ....................uguuaaaacuaugucgccgguugcgaug...................................   4         0         OV2
    .......................uaaaacuaugucgccgguugcAaug...................................   1         1         OV2
    .......................uaaaacuaugucgccgguugcgaug...................................   4         0         OV2

    .....ucuuugagaagaguaacuguuaaaacuaug................................................   1         0         TE1
    .......uuugagaagaguaacuguuaaaacuaug................................................   1         0         TE1
```

```
                                                aga-mir-10358-5-star
                                                aga-mir-10358-3-star
uuuguucuu[illegible]aaaacuaugucgccgguugcgaugucauuuucuuuugcuccucgauguuugucugcguac
         aga-mir-10358-4
         aga-mir-10358-3
                                                aga-mir-10358-4-star
                                                aga-mir-10358-2-star
         aga-mir-10358-5
         aga-mir-10358-2
         aga-mir-10358-1
                                                aga-mir-10358-1-star


........uugagaaguaacuguuaaaacuaugu................................................  2    0    TE1
.........ugagaaguaacuguuaaaac.....................................................  1    0    TE1
.........ugagaaguaacuguuaaaacua...................................................  1    0    TE1
.........ugagaaguaacuguuaaaacuaug.................................................  4    0    TE1
.........ugagaaguaacuguuaaaacuaugu................................................  146  0    TE1
.........ugagaaguaacuguuaaaacuauguc...............................................  4    0    TE1
.........ugagaaguaacuguuaaaacuaugucgc.............................................  1    0    TE1
.........ugagaaguaacuguuaaaacuaugucgcc............................................  9    0    TE1
.........ugagaaguaacuguuaaaacuaugucgccU...........................................  2    1    TE1
.........ugagaaguaacuguuaaaacuaugucgccgg..........................................  1    0    TE1
.........ugagaagCaacuguuaaaacuaugucgccggu.........................................  1    1    TE1
.........ugagaaguaacuguuaaaacuaugucgccggu.........................................  9    0    TE1
.........ugagaaguaacuguuaaaacuaugucgccgguugcgaug..................................  1    0    TE1
..........gagaaguaacuguuaaaacuaugu................................................  1    0    TE1
............gaaguaacuguuaaaacuaugucgcc............................................  4    0    TE1
............gaaguaacuguuaaaacuaugucgccggu.........................................  1    0    TE1
....................uguuaaaacuaugucgccgguugcgaug..................................  1    0    TE1
.......................uaaaacuaugucgccgguugcgaug..................................  1    0    TE1
...............................................ugucauuuucuuuugcuccucgauguu.........  1    0    TE1
.....................................................uuucuuuugcuccucgauguuuguc.....  1    0    TE1

........uugagaaguaacuguuaaaacuaugu................................................  1    0    MF2
.........ugagaaguaacuguuaaaacua...................................................  3    0    MF2
.........ugagaaguaacuguuaaaacuaug.................................................  1    0    MF2
.........ugagaaguaacuguuaaaacuaugu................................................  99   0    MF2
.........ugagaaguaacuguuaaaacuauguc...............................................  1    0    MF2
.........ugagaaguaacuguuaaaacuaugucgc.............................................  1    0    MF2
.........ugagaaguaacuguuaaaacuaugucgcc............................................  2    0    MF2
....................uguuaaaacuaugucgccgguugcgaug..................................  1    0    MF2

.........ugagaaguaacuguuaaaacuaugu................................................  1    0    FF1
.........ugagaaguaacuguuaaaacuaugucgcc............................................  1    0    FF1

.uuguucuuugagaaguaacuguuaaaacuaugucgccggu.........................................  1    0    OV1
..uguucuuugagaaguaacuguuaaaacuaugucgccgguugcga....................................  1    0    OV1
....uucuuugagaaguaacuguuaaaacuaugucgccggu.........................................  1    0    OV1
.......uuugagaaguaacuguuaaaacuaug.................................................  2    0    OV1
.......uuugagaaguaacuguuaaaacuaugu................................................  9    0    OV1
........uugagaaguaacuguuaaaacua...................................................  1    0    OV1
........uugagaaguaacuguuaaaacuaugu................................................  3    0    OV1
.........ugagaaguaacuguuaaaacua...................................................  8    0    OV1
.........ugagaaguaacuguuaaaacuaug.................................................  12   0    OV1
.........ugagaaguaaUuguuaaaacuaugu................................................  1    1    OV1
.........ugagaaguaacuguuaaaaAuaugu................................................  1    1    OV1
.........ugagaaguaacuguuaaaacuaugu................................................  357  0    OV1
.........ugagaaguaacuguuaaaacuauguc...............................................  8    0    OV1
.........ugagaaguaacuguuaaaacuaugucgc.............................................  1    0    OV1
.........ugagaaguaacuguuaaaacuaugucgcc............................................  6    0    OV1
.........ugagaaguaacuguuaaaacuaugucgccggu.........................................  15   0    OV1
.........ugagaaguaacuguuaaaacuaugucgccgguugcgaug..................................  4    0    OV1
..............aguaacuguuaaaacuaugu................................................  1    0    OV1
................uaacuguuaaaacuaugucgccggu.........................................  1    0    OV1
....................uguuaaaacuaugucgccgguugcg.....................................  1    0    OV1
....................uguuaaaacuaugucgccgguugcgaug..................................  5    0    OV1
.......................uaaaacuaugucgccgguugcgaug..................................  2    0    OV1
........................aaaacuaugucgccggu.........................................  1    0    OV1
...............................................ugucauuuucuuuugcuccucgauguu.........  3    0    OV1
.................................................ucauuuucuuuugcuccucgauguuug.......  1    0    OV1
.........................................................uuuugcuccucgauguuugucugcgu..  1    0    OV1

.........ugagaaguaacuguuaaaacuaugu................................................  8    0    MF1
```

```
                                                  aga-mir-10358-5-star
                                                  aga-mir-10358-3-star
uuuguucuu[illegible]aaaacuaugucgccgguugcgaugucauuuucuuuugcuccucgauguuugucugcguac
         aga-mir-10358-4
         aga-mir-10358-3
                                                  aga-mir-10358-4-star
                                                  aga-mir-10358-2-star
         aga-mir-10358-5
         aga-mir-10358-2
         aga-mir-10358-1
                                                  aga-mir-10358-1-star


....................uguuaaaacuaugucgccgguugcgaug...................................    1      0      MF1

.......uuugagaaguaacuguuaaaacuaugu.................................................    3      0      BF2
........uugagaaguaacuguuaaaacuaugu.................................................    1      0      BF2
.........ugagaaguaacuguuaaaacua....................................................    2      0      BF2
.........ugagaaguaacuguuaaaacuau...................................................    1      0      BF2
.........ugagaaguaacuguuaaaacuaug..................................................    4      0      BF2
.........ugagaAuaacuguuaaaacuaugu..................................................    1      1      BF2
.........ugagaaguaacuguuaaaacuaugu.................................................    158    0      BF2
.........ugagaaguaacuguuaaaacuauguc................................................    5      0      BF2
.........ugagaaguaacuguuaaaacuaugucg...............................................    1      0      BF2
.........ugagaaguaacuguuaaaacuaugucgc..............................................    1      0      BF2
.........ugagaaguaacuguuaaaacuaugucgcc.............................................    1      0      BF2
.........ugagaaguaacuguuaaaacuaugucgccggu..........................................    4      0      BF2
.........ugagaaguaacuguuaaaacuaugucgccgguugcgaug...................................    1      0      BF2
............gaaguaacuguuaaaacuaugucgcc.............................................    2      0      BF2
................uaacuguuaaaacuaugucgccgg...........................................    1      0      BF2
................uaacuguuaaaacuaugucgccggu..........................................    1      0      BF2
....................uguuaaaacuaugucgccgguugcgaug...................................    1      0      BF2
.........................................................ugcuccucgauguuugucugcguac    1      0      BF2

.......uuugagaaguaacuguuaaaacuaugu.................................................    4      0      BF1
........uugagaaguaacuguuaaaacuaugu.................................................    1      0      BF1
........uugagaaguaacuguuaaaacuaugucgcc.............................................    1      0      BF1
.........ugagaaguaacuguuaaaacua....................................................    5      0      BF1
.........ugagaaguaacuguuaaaacuaug..................................................    5      0      BF1
.........ugagaaguaaAuguuaaaacuaugu.................................................    1      1      BF1
.........ugagaaguaacuguCaaaacuaugu.................................................    1      1      BF1
.........ugagaaguaacuguuaaaacuaugu.................................................    153    0      BF1
.........ugagaaguaacuguuaaaacuauguc................................................    6      0      BF1
.........ugagaaguaacuguuaaaacuaugucg...............................................    1      0      BF1
.........ugagaaguaacuguuaaaacuaugucgc..............................................    1      0      BF1
.........ugagaaguaacuguuaaaacuaugucgcc.............................................    2      0      BF1
.........ugagaaguaacuguuaaaacuaugucgccU............................................    1      1      BF1
.........ugagaaguaacuguuaaaacuaugucgccggu..........................................    4      0      BF1
...........agaaguaacuguuaaaacuaugucgcc.............................................    1      0      BF1
............gaaguaacuguuaaaacuaugucgc..............................................    1      0      BF1
............gaaguaacuguuaaaacuaugucgcc.............................................    1      0      BF1
...................................................uuuucuuuugcuccucgauguuugucu......    1      0      BF1
....................................................uuucuuuugcuccucgauguuugucu......    1      0      BF1
.....................................................uucuuuugcuccucgauguuugucugcg...    2      0      BF1

.........ugagaaguaacuguuaaaacuaugu.................................................    1      0      MW1

.........ugagaaguaacuguuaaaacuaugu.................................................    1      0      FW1

........uugagaaguaacuguuaaaacuaugu.................................................    2      0      TE2
.........ugagaaguaacuguuaaaacua....................................................    2      0      TE2
.........Cgagaaguaacuguuaaaacuaug..................................................    1      1      TE2
.........ugagaaguaacuguuaaaacuaug..................................................    6      0      TE2
.........ugaCaaguaacuguuaaaacuaugu.................................................    1      1      TE2
.........ugagaaguaacuguuaaaacuaugC.................................................    1      1      TE2
.........ugagaaguaacuguuaaaacuUugu.................................................    1      1      TE2
.........ugagaaguaacuguuaaaacuaugu.................................................    272    0      TE2
.........ugagaaguaacuguuaaaacuaugG.................................................    1      1      TE2
.........ugagaaguaacAguuaaaacuaugu.................................................    1      1      TE2
.........ugagaaguaacuguuaaaacuauguc................................................    4      0      TE2
.........ugagaaguaacuguuaaaacuaugucg...............................................    1      0      TE2
.........ugagaaguaacuguuaaaacuaugucgc..............................................    6      0      TE2
.........ugagaaguaacuguuaaaacuaugucgcc.............................................    7      0      TE2
.........ugagaaguaacuguuaaaacuaugucgccU............................................    2      1      TE2
```

aga-mir-10358-5-star
aga-mir-10358-3-star
uuuguucuu[illegible]aaaacuaugucgccgguugcgaugucauuuucuuuugcuccucgauguuugucugcguac
aga-mir-10358-4
aga-mir-10358-3
aga-mir-10358-4-star
aga-mir-10358-2-star
aga-mir-10358-5
aga-mir-10358-2
aga-mir-10358-1
aga-mir-10358-1-star

.........ugagaaguaacuguuaaaacuaugucgccggu...	10	0	TE2
..........gagaaguaacuguuaaaacua..	1	0	TE2
............gaaguaacuguuaaaacuaugucgcc...	1	0	TE2
.......................uaaaacuaugucgccgguugcgaug....................................	1	0	TE2
....uugcuccucgauguuugucugcguac	1	0	TE2

miRBase precursor : aga-mir-10358-5
Total read count : 8546
aga-mir-10358-1 read count: 1689
aga-mir-10358-1-star read co8nt
aga-mir-10358-2 read count: 1689
aga-mir-10358-2-star read co8nt
aga-mir-10358-3 read count: 1689
aga-mir-10358-3-star read co8nt
aga-mir-10358-4 read count: 1689
aga-mir-10358-4-star read co8nt
aga-mir-10358-5 read count: 1689
aga-mir-10358-5-star read co8nt
remaining reads : 61

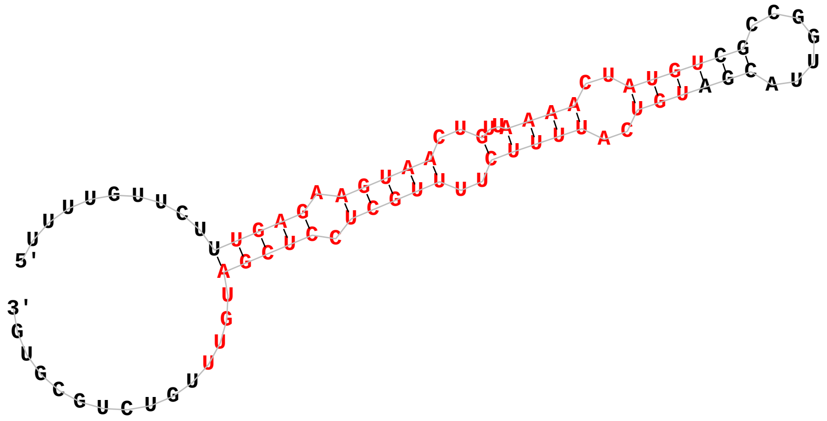

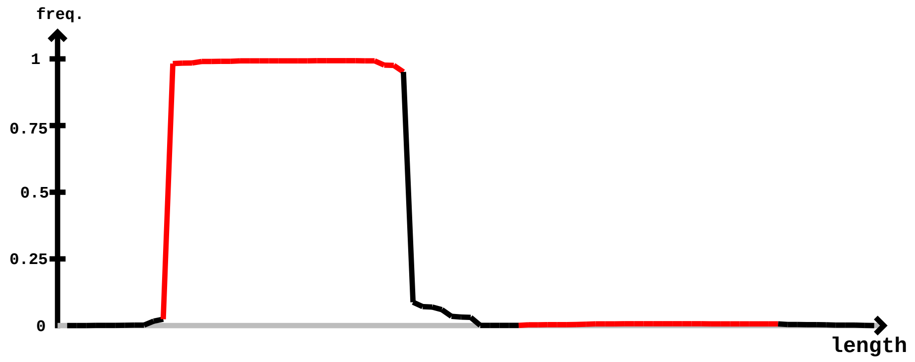


```
                                                    aga-mir-10358-5-star
                                                    aga-mir-10358-3-star
               aga-mir-10358-4
               aga-mir-10358-3
                                                    aga-mir-10358-4-star
                                                    aga-mir-10358-2-star
               aga-mir-10358-5
               aga-mir-10358-2
               aga-mir-10358-1
                                                    aga-mir-10358-1-star
5'- uuuuguucuuugagaaguaacuguuaaaacuaugucgccgguuacgaugucauuuucuuuugcuccucgauguuugucugcgug  -3'   exp
    .........(((((.(((((..(..((((..((((((.......))))))..)))))..))))).))))).............     reads      mm         sample
    .........ugagaaguaacuguuaaaacuaugu...............................................     6          0          FF2
    .........ugagaaguaacuguuaaaacuaugucgcc...........................................     1          0          FF2

    .......uuugagaaguaacuguuaaaacuaug................................................     1          0          OV2
    .......uuugagaaguaacuguuaaaacuaugu...............................................     6          0          OV2
    ........uugagaaguaacuguuaaaacuaugu...............................................     1          0          OV2
    .........ugagaaguaacuguuaaaacua..................................................     5          0          OV2
    .........ugagaaguaacuguuaaaacuau.................................................     1          0          OV2
    .........ugagaaguaacuguuaaaacuaug................................................     5          0          OV2
    .........ugagaaguaacuAuuaaaacuaugu...............................................     1          1          OV2
    .........ugagaaguaacuguuaaaacuaugu...............................................     269        0          OV2
    .........ugagaaguaacuguuaaaacuauguc..............................................     1          0          OV2
    .........ugagaaguaacuguuaaaacuaugucgc............................................     5          0          OV2
    .........ugagaaguaacuguuaaaacuaugucgcc...........................................     5          0          OV2
    .........ugagaaguaacuguuaaaacuaugucgccggu........................................     5          0          OV2
    ..........gagaaguaacuguuaaaacuaugu...............................................     1          0          OV2

    ......ucuuugagaaguaacuguuaaaacuaug...............................................     1          0          TE1
    .......uuugagaaguaacuguuaaaacuaug................................................     1          0          TE1
    ........uugagaaguaacuguuaaaacuaugu...............................................     2          0          TE1
    .........ugagaaguaacuguuaaaac....................................................     1          0          TE1
    .........ugagaaguaacuguuaaaacua..................................................     1          0          TE1
    .........ugagaaguaacuguuaaaacuaug................................................     4          0          TE1
    .........ugagaaguaacuguuaaaacuaugu...............................................     146        0          TE1
    .........ugagaaguaacuguuaaaacuauguc..............................................     4          0          TE1
    .........ugagaaguaacuguuaaaacuaugucgc............................................     1          0          TE1
```

aga-mir-10358-5-star
aga-mir-10358-3-star
uuuuguucuuugagaaguaacuguuaaaacuaugucgccgguuacgaugucauuuucuuuugcuccucgauguuugucugcgug
aga-mir-10358-4
aga-mir-10358-3
aga-mir-10358-4-star
aga-mir-10358-2-star
aga-mir-10358-5
aga-mir-10358-2
aga-mir-10358-1
aga-mir-10358-1-star

..........ugagaaguaacuguuaaaacuaugucgcc...	9	0	TE1
..........ugagaaguaacuguuaaaacuaugucgccU..	2	1	TE1
..........ugagaaguaacuguuaaaacuaugucgccgg...	1	0	TE1
..........ugagaaguaacuguuaaaacuaugucgccggu..	9	0	TE1
..........ugagaagCaacuguuaaaacuaugucgccggu..	1	1	TE1
...........gagaaguaacuguuaaaacuaugu...	1	0	TE1
.............gaaguaacuguuaaaacuaugucgcc...	4	0	TE1
.............gaaguaacuguuaaaacuaugucgccggu..	1	0	TE1
....ugucauuuucuuuugcuccucgauguu..........	1	0	TE1
....uuucuuuugcuccucgauguuuguc......	1	0	TE1
.........uugagaaguaacuguuaaaacuaugu...	1	0	MF2
..........ugagaaguaacuguuaaaacua..	3	0	MF2
..........ugagaaguaacuguuaaaacuaug..	1	0	MF2
..........ugagaaguaacuguuaaaacuaugu...	99	0	MF2
..........ugagaaguaacuguuaaaacuauguc..	1	0	MF2
..........ugagaaguaacuguuaaaacuaugucgc..	1	0	MF2
..........ugagaaguaacuguuaaaacuaugucgcc...	2	0	MF2
..........ugagaaguaacuguuaaaacuaugu...	1	0	FF1
..........ugagaaguaacuguuaaaacuaugucgcc...	1	0	FF1
..uuguucuuugagaaguaacuguuaaaacuaugucgccggu..	1	0	OV1
.....uucuuugagaaguaacuguuaaaacuaugucgccggu..	1	0	OV1
........uuugagaaguaacuguuaaaacuaug..	2	0	OV1
........uuugagaaguaacuguuaaaacuaugu...	9	0	OV1
.........uugagaaguaacuguuaaaacua..	1	0	OV1
.........uugagaaguaacuguuaaaacuaugu...	3	0	OV1
..........ugagaaguaacuguuaaaacua..	8	0	OV1
..........ugagaaguaacuguuaaaacuaug..	12	0	OV1
..........ugagaaguaacuguuaaaaAuaugu...	1	1	OV1
..........ugagaaguaaUuguuaaaacuaugu...	1	1	OV1
..........ugagaaguaacuguuaaaacuaugu...	357	0	OV1
..........ugagaaguaacuguuaaaacuauguc..	8	0	OV1
..........ugagaaguaacuguuaaaacuaugucgc..	1	0	OV1
..........ugagaaguaacuguuaaaacuaugucgcc...	6	0	OV1
..........ugagaaguaacuguuaaaacuaugucgccggu..	15	0	OV1
...............aguaacuguuaaaacuaugu...	1	0	OV1
.................uaacuguuaaaacuaugucgccggu..	1	0	OV1
.........................aaaacuaugucgccggu..	1	0	OV1
....uacgaugucauuuucuuuugcucc..................	1	0	OV1
....ugucauuuucuuuugcuccucgauguu..........	3	0	OV1
....ucauuuucuuuugcuccucgauguuug........	1	0	OV1
....uuuugcuccucgauguuugucugcgu.	1	0	OV1
..........ugagaaguaacuguuaaaacuaugu...	8	0	MF1
........uuugagaaguaacuguuaaaacuaugu...	3	0	BF2
.........uugagaaguaacuguuaaaacuaugu...	1	0	BF2
..........ugagaaguaacuguuaaaacua..	2	0	BF2
..........ugagaaguaacuguuaaaacuau...	1	0	BF2
..........ugagaaguaacuguuaaaacuaug..	4	0	BF2
..........ugagaaAuaacuguuaaaacuaugu...	1	1	BF2
..........ugagaaguaacuguuaaaacuaugu...	158	0	BF2
..........ugagaaguaacuguuaaaacuauguc..	5	0	BF2
..........ugagaaguaacuguuaaaacuaugucg...	1	0	BF2
..........ugagaaguaacuguuaaaacuaugucgc..	1	0	BF2
..........ugagaaguaacuguuaaaacuaugucgcc...	1	0	BF2
..........ugagaaguaacuguuaaaacuaugucgccggu..	4	0	BF2
.............gaaguaacuguuaaaacuaugucgcc...	2	0	BF2
.................uaacuguuaaaacuaugucgccgg...	1	0	BF2

aga-mir-10358-5-star
aga-mir-10358-3-star
uuuuguucuu[illegible]aaaacuaugucgccgguuacgaugucauuuucuuuugcuccucgauguuugucugcgug
aga-mir-10358-4
aga-mir-10358-3
aga-mir-10358-4-star
aga-mir-10358-2-star
aga-mir-10358-5
aga-mir-10358-2
aga-mir-10358-1
aga-mir-10358-1-star

.................uaacuguuaaaacuaugucgccggu..	1	0	BF2
........uuugagaaguaacuguuaaaacuaugu...	4	0	BF1
.........uugagaaguaacuguuaaaacuaugu...	1	0	BF1
.........uugagaaguaacuguuaaaacuaugucgcc...	1	0	BF1
..........ugagaaguaacuguuaaaacua..	5	0	BF1
..........ugagaaguaacuguuaaaacuaug..	5	0	BF1
..........ugagaaguaacuguuaaaacuaugu...	153	0	BF1
..........ugagaaguaacuguCaaaacuaugu...	1	1	BF1
..........ugagaaguaaAuguuaaaacuaugu...	1	1	BF1
..........ugagaaguaacuguuaaaacuauguc..	6	0	BF1
..........ugagaaguaacuguuaaaacuaugucg...	1	0	BF1
..........ugagaaguaacuguuaaaacuaugucgc..	1	0	BF1
..........ugagaaguaacuguuaaaacuaugucgcc...	2	0	BF1
..........ugagaaguaacuguuaaaacuaugucgccU..	1	1	BF1
..........ugagaaguaacuguuaaaacuaugucgccggu..	4	0	BF1
............agaaguaacuguuaaaacuaugucgcc...	1	0	BF1
.............gaaguaacuguuaaaacuaugucgc..	1	0	BF1
.............gaaguaacuguuaaaacuaugucgcc...	1	0	BF1
....uuucuuuugcuccucgauguuugucu.....	1	0	BF1
....uucuuuugcuccucgauguuugucu.....	1	0	BF1
....uucuuuugcuccucgauguuugucugcg..	2	0	BF1
..........ugagaaguaacuguuaaaacuaugu...	1	0	FW1
..........ugagaaguaacuguuaaaacuaugu...	1	0	MW1
.........uugagaaguaacuguuaaaacuaugu...	2	0	TE2
..........ugagaaguaacuguuaaaacua..	2	0	TE2
..........Cgagaaguaacuguuaaaacuaug..	1	1	TE2
..........ugagaaguaacuguuaaaacuaug..	6	0	TE2
..........ugagaaguaacuguuaaaacuUugu...	1	1	TE2
..........ugagaaguaacuguuaaaacuaugC...	1	1	TE2
..........ugagaaguaacuguuaaaacuaugG...	1	1	TE2
..........ugaCaaguaacuguuaaaacuaugu...	1	1	TE2
..........ugagaaguaacuguuaaaacuaugu...	272	0	TE2
..........ugagaaguaacAguuaaaacuaugu...	1	1	TE2
..........ugagaaguaacuguuaaaacuauguc..	4	0	TE2
..........ugagaaguaacuguuaaaacuaugucg...	1	0	TE2
..........ugagaaguaacuguuaaaacuaugucgc..	6	0	TE2
..........ugagaaguaacuguuaaaacuaugucgcc...	7	0	TE2
..........ugagaaguaacuguuaaaacuaugucgccU..	2	1	TE2
..........ugagaaguaacuguuaaaacuaugucgccggu..	10	0	TE2
...........gagaaguaacuguuaaaacua..	1	0	TE2
.............gaaguaacuguuaaaacuaugucgcc...	1	0	TE2

miRBase precursor : aga-mir-10359
Total read count : 1343
aga-mir-10359 read count : 1308
aga-mir-10359-star read count : 19
remaining reads : 16

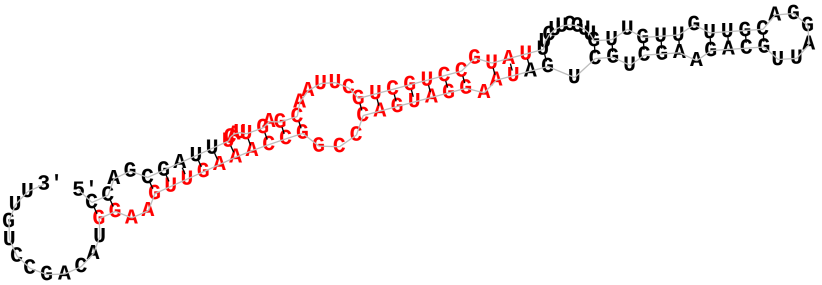

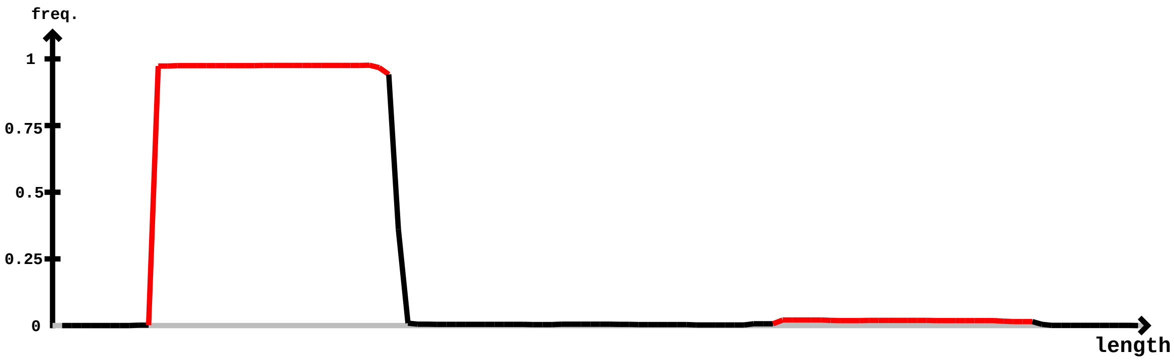


```
                aga-mir-10359
                                                                          aga-mir-10359-star
5'- ccagcgauuucauugagcaauucgucguccguauuucuucguuguuguuguugcaggauugcagaagcugcugauaaggaugacccggccaaaguugaaggguacagccuguu  -3'   exp
    .((((..((((((((.((.....(((((((.((((........((.(((.((((......)))).))).)).)))).)))))))...)))))...))))).....).)))..   reads   mm   sample
    .........ucauugagcaauucgucguccguau..............................................................................   1       0    FF2

    .........ucauugagcaauucgucguccgua...............................................................................   3       0    TE1
    .........ucauugagcaauucgucguccguau..............................................................................   16      0    TE1
    .........ucauugagcaauucgucguccguauu.............................................................................   8       0    TE1
    .........ucauuAagcaauucgucguccguauu.............................................................................   1       1    TE1
    .........ucauugagcaauucgucguccguauuCcu..........................................................................   2       1    TE1

    .........ucauugagcaauucgucguccgu................................................................................   1       0    OV2
    .........ucauugagcaauucgucguccgua...............................................................................   10      0    OV2
    .........ucauugagcaauuUgucguccguau..............................................................................   1       1    OV2
    .........ucauugagcaauucCucguccguau..............................................................................   1       1    OV2
    .........ucauugagcaauucgucguccguau..............................................................................   207     0    OV2
    .........ucauugagcaauucgucgucAguau..............................................................................   1       1    OV2
    .........ucauugagcaauucgucguccguauu.............................................................................   124     0    OV2
    .........ucauugagcaauucgucguccguauA.............................................................................   1       1    OV2
    .........ucauugagcaauucgucguccguauuu............................................................................   1       0    OV2
    .........ucauugagcaauucgucguccguauuC............................................................................   2       1    OV2
    ...........auugagcaauucgucguccguau..............................................................................   1       0    OV2
    .......................................................................ugauaaggaugacccggccaaaguugaagg...........   1       0    OV2
    ..........................................................................uaaggaugacccggccaaaguuga..............   1       0    OV2
    ..........................................................................uaaggaugacUcggccaaaguugaagg...........   1       1    OV2
    ..........................................................................uaaggaugacccggccaaaguugaaggu..........   2       0    OV2

    .........ucauugagcaauucgucguccguau..............................................................................   5       0    MF2
    .........ucauugagcaauucgucguccguauu.............................................................................   2       0    MF2

    .........ucauugagcaauucgucguccguau..............................................................................   1       0    FF1
    ..........................................................................uaaggaugacccggccaaaguugaagg...........   1       0    FF1

    ......uuucauugagcaauucgucguccguau...............................................................................   1       0    OV1
    .........ucauugagcaauucgucguccg.................................................................................   1       0    OV1
    .........ucauugagcaauucgucguccgu................................................................................   2       0    OV1
    .........ucauugagcaauucgucgCccgu................................................................................   1       1    OV1
```

```
         aga-mir-10359
                                                                         aga-mir-10359-star
ccagcgauuucauugagcaauucgucguccguauuucuucguuguuguuguugcaggauugcagaagcugcugauaaggaugacccggccaaaguugaagguacagccuguu

.........ucauugagcaauucgucguccgua...............................................................................   14    0    OV1
.........ucauugagcaauUgucguccguau..............................................................................   1     1    OV1
.........ucauugagcaauucgucAuccguau..............................................................................   1     1    OV1
.........ucauugagcaauucgucguccguau..............................................................................   277   0    OV1
.........ucauuAagcaauucgucguccguau..............................................................................   1     1    OV1
.........ucauCgagcaauucgucguccguau..............................................................................   1     1    OV1
.........ucauugagcaauucgucguccAuau..............................................................................   1     1    OV1
.........ucauugagcaauucgucguccgCau..............................................................................   1     1    OV1
.........ucauugagcaauucgucguccguauu.............................................................................   193   0    OV1
.........ucauugagcaauuAgucguccguauu.............................................................................   1     1    OV1
.........ucauugagcaauucgucguccguauA.............................................................................   3     1    OV1
.........ucauugagcaauucgucguccguauuCcuucguuguuguugu.............................................................   1     1    OV1
......................................ucguuguuguuguugcaggauugcaga...............................................   2     0    OV1
.......................................................................ugauaaggaugUcccggcc......................   1     1    OV1
.......................................................................ugauaaggaugacccggccaaaguug...............   1     0    OV1
..........................................................................uaaggaugacccggccaaaguugaagg...........   7     0    OV1
..........................................................................uaaggaugacccggccaaaguugaaggu..........   1     0    OV1
...................................................................................cccggccaaaguugaagguacagccugu.   1     0    OV1

.......uuucauugagcaauucgucguccguau..............................................................................   1     0    BF2
.........ucauugagcaauucgucguccgu................................................................................   2     0    BF2
.........ucauugagcaauucgucguccgua...............................................................................   2     0    BF2
.........ucauugagcaauucgucguccguau..............................................................................   106   0    BF2
.........ucauugagcaaCucgucguccguau..............................................................................   1     1    BF2
.........ucauugagcaauucgucguccguauu.............................................................................   58    0    BF2
.........ucauugagcaauucgucguccguauA.............................................................................   3     1    BF2
.........ucauugagcaauucgucguccguauuu............................................................................   1     0    BF2
.........ucauugagcaauucgucguccguauuC............................................................................   1     1    BF2
....................uucgucguccguauuCcuucguuguugu................................................................   1     1    BF2
.......................................................................ugauaaggaugacccggccaaaguug...............   2     0    BF2
..........................................................................uaaggaugacccggccaaaguugaagg...........   2     0    BF2
..........................................................................uaaggaugacccggccaaaguugaaggu..........   1     0    BF2

.........ucauugagcaauucgucguccgua...............................................................................   2     0    BF1
.........ucauugagcaauucgucguccgCa...............................................................................   1     1    BF1
.........ucauugagcaauucgucguccguau..............................................................................   113   0    BF1
.........ucauugagcaauucgCcguccguauu.............................................................................   1     1    BF1
.........ucauugagcaauucgucguccguauA.............................................................................   1     1    BF1
.........ucauugagcaauucgucguccguauu.............................................................................   71    0    BF1
.........ucauugagcaauucgucguccguauuCcu..........................................................................   1     1    BF1
...........auugagcaauucgucguccguau..............................................................................   1     0    BF1
...............................uauuCcuucguuguuguuguugcagg.......................................................   1     1    BF1
...............................uauuucuucguuguuguuguugcaggau.....................................................   1     0    BF1
...................................................ugcaggauugcagaagcugcugauaagg.................................   2     0    BF1
...................................................ugcaggauugcagaagcugcugauaagga................................   1     0    BF1
.......................................................................ugauaaggaugacccggccaaaguuga..............   1     0    BF1
..........................................................................uaaggaugacccggccaaaguugaagg...........   2     0    BF1
..........................................................................uaaggaugacccggccaaaguugaaggu..........   1     0    BF1

.........ucauugagcaauucgucguccgu................................................................................   6     0    TE2
.........ucauugagcaauucgucguccgua...............................................................................   2     0    TE2
.........ucauugagcaauucgucguccgCau..............................................................................   1     1    TE2
.........ucauugagcaauucgucguccguau..............................................................................   39    0    TE2
.........ucauugagcaauucgucguccguauu.............................................................................   6     0    TE2
```

miRBase precursor : aga-mir-10360
Total read count : 1217
aga-mir-10360 read count : 1063
aga-mir-10360-star read count : 9
remaining reads : 145

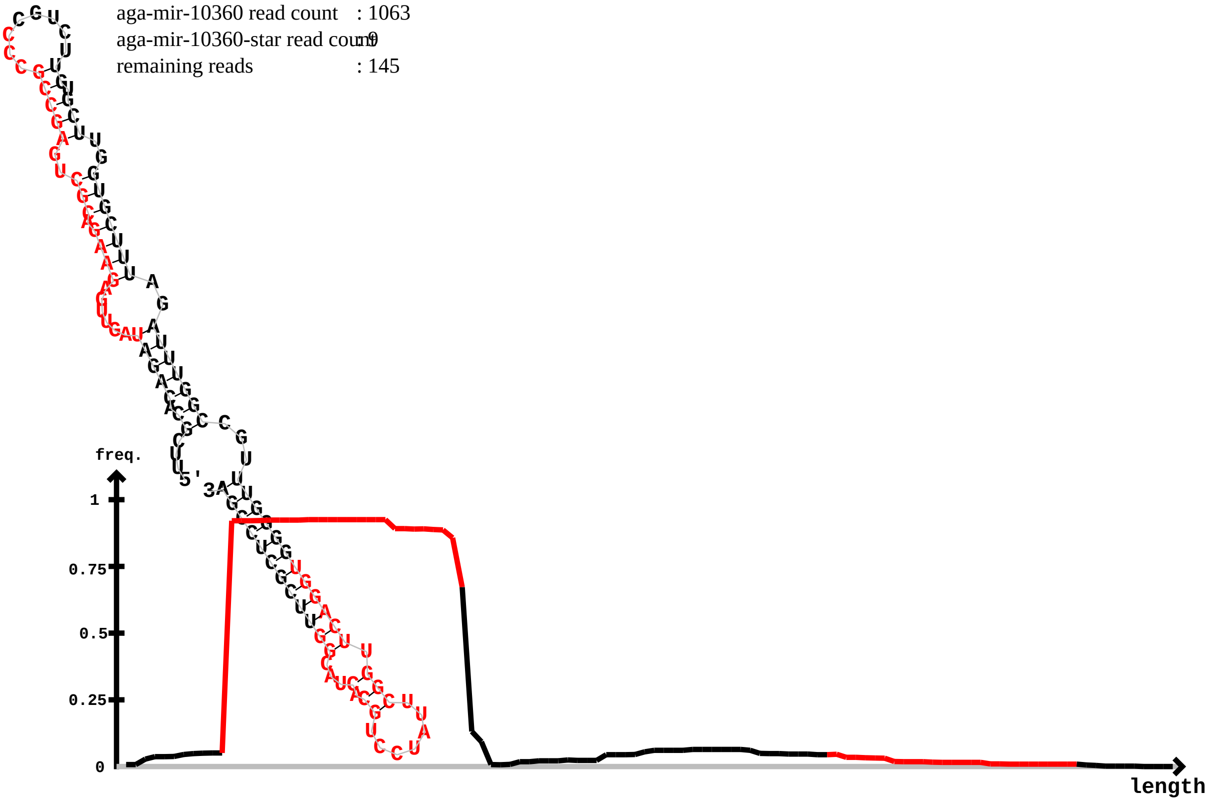


```
                 aga-mir-10360
                                                                 aga-mir-10360-star
5'- uucgcacagauaguugagaagacgcugagccgccccgucuugugcuuggugcuuuagauuuggccguuuggggguggacuuggcuuauccugcacuacgguucgcuccga  -3'  exp
    ...(((..((((((..((...((.(..(((.(((..((((...((.....))...))))..))).))).).))...))..)).)))).)))....(((......))).   reads   mm   sample
    ..........uaguugagaagacgcugagccgcc...........................................................................   1       0    FF2
    ..........uaguugagaagacgcugagccgccc..........................................................................   1       0    FF2

    .ucgcacagauaguugagaagacgcug..................................................................................   1       0    TE1
    ..Ugcacagauaguugagaagacgcugag................................................................................   1       1    TE1
    ......caAauaguugagaagacgcugagccgccc..........................................................................   1       1    TE1
    ..........uaguugagaagacgcugagcc..............................................................................   2       0    TE1
    ..........uaguugagaagacgcugagccgc............................................................................   5       0    TE1
    ..........uaguugagaagacgcCgagccgcc...........................................................................   1       1    TE1
    ..........uaguugagaagacgcugagUcgcc...........................................................................   1       1    TE1
    ..........uaguugagaagacgcugagccgcc...........................................................................   23      0    TE1
    ..........uaguugagaagacgcugagccgccc..........................................................................   55      0    TE1
    ..........uaguugagaagacgcugagUcgccc..........................................................................   1       1    TE1
    ..........uaguugagaagacgcugagccgcccU.........................................................................   1       1    TE1
    ..........uaguugagaagacgcugagccgcccc.........................................................................   5       0    TE1
    ..........uaguugagaagacgcugagccgccccg........................................................................   10      0    TE1
    ........................................ugugcuuggugcuuuagauuuggc.............................................   1       0    TE1
    ..........................................ugcuuggugcuuuagauuuggcc............................................   1       0    TE1
    ..........................................ugcuuggugcuuuagauuuggcUguu.........................................   1       1    TE1
    .................................................ugcuuuagauuuggccguuuggggu...................................   1       0    TE1

    uucgcacagauaguugagaagacgcug..................................................................................   3       0    OV2
    .ucgcacagauaguugagaagacgcug..................................................................................   6       0    OV2
    .ucgcacagauaguugagaagacgcugagccgcccc.........................................................................   1       0    OV2
    ..cgcacagauaguugagaagacgcug..................................................................................   1       0    OV2
    .....acagauaguugagaagacgcugagccgcccc.........................................................................   1       0    OV2
    .......agauaguugagaagacgcugagccgcccc.........................................................................   1       0    OV2
    ........gauaguugagaagacgcugagccgcc...........................................................................   1       0    OV2
    ..........uaguugagaagacgcugagccg.............................................................................   1       0    OV2
    ..........uaguugagaaUacgcugagccgc............................................................................   1       1    OV2
    ..........uaguugagaagacgcugagccgc............................................................................   3       0    OV2
    ..........uaguugagaagacgcugagccgcc...........................................................................   32      0    OV2
    ..........uaguugagaagacgcugagccgccU..........................................................................   1       1    OV2
    ..........uaguugagaagacgcugagccgcccc.........................................................................   132     0    OV2
```

aga-mir-10360

aga-mir-10360-star

uucgcacagauaguugagaagacgcugagccgccccgucuugugcuuggugcuuuagauuuggccguuugggguggacuuggcuuauccugcacuacgguucgcuccga			
..........uaguugUgaagacgcugagccgccc..	1	1	OV2
..........uaguugagaagacgcugagccgcccc...	9	0	OV2
..........uaguugagaagacgcugagccgccccg..	18	0	OV2
..................aagacgcugagccgccccgucGugugcu...	2	1	OV2
....ugugcuuggugcuuuagauuuggcc..	2	0	OV2
....ugcuuggugcuuuagauuuggcc..	1	0	OV2
....ugcuuuagauuuggccguuugggg....................................	1	0	OV2
....ugcuuuagauuuggcUguuugggg....................................	1	1	OV2
....ugcuuuagauuuggccguuuggggu...................................	1	0	OV2
....ugcuuuagauuuggcUguuugggguggacuuggc..........................	1	1	OV2
....uuagauuuggccguuugggguggacu..............................	1	0	OV2
....uagauuuggcUguuugggguggacuuggcuuaucc....................	1	1	OV2
....uggacuuggcuuauccugcacuacgg..........	2	0	OV2
....uggacuuggcuuauccugcacuacgguu........	1	0	OV2
..cgcacagauaguugagaagacgcugag..	1	0	MF2
..........uaguugagaagacgcugagccgc..	1	0	MF2
..........uaguugagaagacgcugagccgcc...	5	0	MF2
..........uaguugagaagacgcugagUcgccc..	1	1	MF2
..........uaguugagaagacgcugagccgccc..	43	0	MF2
..........uaguugagaagacgcugagccgccA..	1	1	MF2
..........uaguugagaagacgcugagccgcccc...	3	0	MF2
..........uaguugagaagacgcugagccgccccU..	1	1	MF2
..........uaguugagaagacgcugagccgccccg..	6	0	MF2
..........uaguugagaagacgcugagUcgccccg..	1	1	MF2
....ugugcuuggugcuuuagauuuggccg...	1	0	MF2
....ugcuuuagauuuggccguuugggguggacu..............................	2	0	MF2
....uagauuuggccguuugggguggacuuggcuuaucc....................	1	0	MF2
..........uaguugagaagacgcugagccgcc...	2	0	FF1
..........uaguugagaagacgcugagccgccc..	1	0	FF1
..........uaguugagaagacUcugagccgccc..	1	1	FF1
..........uaguugagaagacgcugagccgcccc...	1	0	FF1
....ugugcuuggugcuuuagauuuggccguuuggggGggacuuggcuuauccug..................	1	1	FF1
....uuagauuuggcUguuugggguggacuuggcuuaucc....................	1	1	FF1
uucgcacagauaguugagaagacgcug..	4	0	OV1
.ucgcacagauaguugagaagacgcug..	7	0	OV1
.ucgcacagauaguugagaagacgcugagccgccccg..	1	0	OV1
..cgcacagauaguugagaagacgcug..	1	0	OV1
....cacagauaguugagaagacgcugagccgccc..	1	0	OV1
.....acagauaguugagaagacgcugagccgcc...	2	0	OV1
......cagauaguugagaagacgcugagccgccc..	1	0	OV1
.......agauaguugagaagacgcugagccgcc...	1	0	OV1
..........uaguugagaagacgcugagccgc..	11	0	OV1
..........Caguugagaagacgcugagccgcc...	1	1	OV1
..........uaguugagaagacgcugagccgcc...	63	0	OV1
..........uaguugagaagacgcugagccgcAc..	1	1	OV1
..........Caguugagaagacgcugagccgccc..	1	1	OV1
..........uaguugagaagacgcugagccgccc..	170	0	OV1
..........uaguCgagaagacgcugagccgccc..	1	1	OV1
..........uaguugagaagacgcugagccgccA..	1	1	OV1
..........uaguugagaagacgcugagccgccccU..	1	1	OV1
..........uaguugagaagacgcugagccgcccc...	7	0	OV1
..........uaguugagaagacgcugagccgccccg..	27	0	OV1
..........uaguugagaagacgcugagccgccccgucu...	2	0	OV1
.............uugagaagacgcugagccgccccg..	1	0	OV1
..............ugagaagacgcugagccgc..	2	0	OV1
..............................cgccccgucuugugcuuggugcu..	1	0	OV1
.................................cccgucGugugcuuggugcuuuagauu...	1	1	OV1
....ugugcuuggugcuuuagauuuggc...	1	0	OV1
....ugugcuuggugcuuuagauuuggcc..	1	0	OV1
....ugugcuuggugcuuuagauuuggcU..	1	1	OV1
....ugugcuuggugcuuuagauuuggcUguu...	1	1	OV1
....ugcuuggugcuuuagauuuggcc..	1	0	OV1
....ugcuuuagauuuggcUguuuggggu...................................	1	1	OV1
....ugcuuuagauuuggccguuuggggu...................................	3	0	OV1
....uuagauuuggcUguuuggggugg.................................	1	1	OV1
....uuagauuuggccguuugggguggac...............................	1	0	OV1

```
         aga-mir-10360
                                                               aga-mir-10360-star
uucgcacagauaguugagaagacgcugagccgccccgucuugugcuuggugcuuuagauuuggccguuugggguggacuuggcuuauccugcacuacgguucgcuccga

......................................................uuagauuuggccguuugggguggacu..............................   5   0   OV1
......................................................uuagauuuggcUguuugggguggacu..............................   2   1   OV1
.......................................................uagauuuggcUguuugggguggacu..............................   1   1   OV1
.......................................................uagauuuggcUguuugggguggacuuggcuuaucc....................   1   1   OV1
...........................................................uuuggcUguuugggguggacuuggcu.........................   1   1   OV1
.............................................................uggcUguuugggguggacu..............................   1   1   OV1
..........................................................................uggacuuggcuuauccugcacuacgg..........   2   0   OV1
..........................................................................uggacuuggcuuauccugcacuacggu.........   2   0   OV1
..........................................................................uggacuuggcuuauccugcacuacgguu........   2   0   OV1

..........uaguugagaagacgcugagccgcc............................................................................   1   0   MF1
..........uaguugagaagacgcugagccgccc...........................................................................   2   0   MF1
..........uaguugagaagacgcugagccgcccc..........................................................................   1   0   MF1
..........uaguugagaagacgcugagccgccccg.........................................................................   2   0   MF1

uucgcacagauaguugagaagacgcug...................................................................................   1   0   BF2
.ucgcacagauaguugagaagacgcug...................................................................................   5   0   BF2
..cgcacagauaguugagaagacgcug...................................................................................   4   0   BF2
.....acagauaguugagaagacgcugagccgccc...........................................................................   1   0   BF2
......cagauaguugagaagacgcugagccgccc...........................................................................   1   0   BF2
..........uaguugagaagacgcugagccgc.............................................................................   7   0   BF2
..........uaguuAagaagacgcugagccgc.............................................................................   1   1   BF2
..........uaguugagaagacgcugagccgcc............................................................................   14  0   BF2
..........uaguugagaagacgcCgagccgccc...........................................................................   1   1   BF2
..........uaguugagaagacgcugagccgccc...........................................................................   36  0   BF2
..........uaguugagaagacgcugagccgcccc..........................................................................   6   0   BF2
..........uaguugagaagacgcugagccgccccg.........................................................................   8   0   BF2
........................................uugugcuuggugcuuuagauuuggcc............................................   1   0   BF2
.........................................ugugcuuggugcuuuagauuuggcU............................................   1   1   BF2
.........................................ugugcuuggugcuuuagauuuggcc............................................   2   0   BF2
..............................................uuggugcuuuagauuuggcUguuugggg....................................   1   1   BF2
..................................................ugcuuuagauuuggcUguuugggg....................................   1   1   BF2
..................................................ugcuuuagauuuggcUguuuggggu...................................   2   1   BF2
..................................................ugcuuuagauuuggcUguuugggguggg.................................   1   1   BF2
..................................................ugcuuuagauuuggccguuugggguggacu..............................   1   0   BF2
..................................................ugcuuuagauuuggccguuugggguggacuuggcuuaucc....................   1   0   BF2
......................................................uuagauuuggccguuugggguggacuu.............................   1   0   BF2
.......................................................uagauuuggccguuugggguggacu..............................   1   0   BF2
...........................................................uuuggccguuugggguggacuuggc..........................   1   0   BF2
................................................................................uggcuuauccugcacuacgguucgcu....   1   0   BF2

uucgcacagauaguugagaagacgcug...................................................................................   1   0   BF1
.ucgcacagauaguugagaagacgcug...................................................................................   2   0   BF1
..cgcacagauaguugagaagacgcug...................................................................................   2   0   BF1
.....acagauaguugagaagacgcugagccg..............................................................................   1   0   BF1
.....acagauaguugagaagacgcugagccgcc............................................................................   2   0   BF1
.....acagauaguugagaagacgcugagccgccc...........................................................................   1   0   BF1
......cagauaguugagaagacgcugagccgcc............................................................................   1   0   BF1
..........uaguugagaagacgcugagccgc.............................................................................   3   0   BF1
..........uaguugagaagacgcugagccgcc............................................................................   19  0   BF1
..........uaguugagaagacgcugagccgccU...........................................................................   1   1   BF1
..........uaguugagaagacgcugagccgccc...........................................................................   39  0   BF1
..........uaguugagaagacgcugagccgcccU..........................................................................   1   1   BF1
..........uaguugagaagacgcugagccgcccc..........................................................................   3   0   BF1
..........uaguugagaagacgcugagccgccccg.........................................................................   6   0   BF1
......................................ucuugugcuuggugcuuuagauuuggc.............................................   1   0   BF1
.........................................ugugcuuggugcuuuagauuuggc.............................................   1   0   BF1
..............................................uuggugcuuuagauuuggcUguuugg......................................   1   1   BF1
..................................................ugcuuuagauuuggccguuugggg....................................   1   0   BF1
..................................................ugcuuuagauuuggccguuuggggu...................................   2   0   BF1
..................................................ugcuuuagauuuggccguuugggguggacuuggcuuaucc....................   1   0   BF1
...........................................................uuuggccguuugggguggacu..............................   1   0   BF1
...........................................................uuuggcUguuugggguggacuuggcuuaucc....................   1   1   BF1
................................................................................uggcuuauccugcacuacgguucgcu....   1   0   BF1

..........uaguugagaagacgcugagccgc.............................................................................   1   0   MW1

.ucgcacagauaguugagaagacgcug...................................................................................   2   0   TE2
..Ugcacagauaguugagaagacgcug...................................................................................   1   1   TE2
```

```
          aga-mir-10360
                                                                          aga-mir-10360-star
uucgcacagauaguugagaagacgcugagccgcccccgucuugugcuuggugcuuuagauuuggccguuugggguggacuuggcuuauccugcacuacgguucgcuccga

.....acagauaguugagaagacgcugagccgccU...........................................................................    1    1    TE2
..........uaguugagaagacgcugagcc...............................................................................    1    0    TE2
..........uaguugagaagacgcugagccgc.............................................................................    2    0    TE2
..........uaguugagaagacgcugagccgcc............................................................................    55   0    TE2
..........uaguugagaagacgcugagccgccA...........................................................................    1    1    TE2
..........uaguugagaagacgcugagccgccU...........................................................................    1    1    TE2
..........uaguugagaagacgcugagUcgccc...........................................................................    4    1    TE2
..........uaguugagaagacgcugagccgccc...........................................................................    149  0    TE2
..........uaguugagaagGcgcugagccgccc...........................................................................    1    1    TE2
..........uaguugagaagacgcugagccAccc...........................................................................    1    1    TE2
..........uaguugagaagacgcugagccgcccc..........................................................................    8    0    TE2
..........uaguugagaagacgcuAagccgccccg.........................................................................    2    1    TE2
..........uaguugagaagacgcugagccgccccg.........................................................................    23   0    TE2
..........uaguugagaagacgcugagccgccccgu........................................................................    1    0    TE2
..........uaguugagaagacgcugagccgccccgucu......................................................................    1    0    TE2
........................................Cugugcuuggugcuuuagauuuggcc............................................    1    1    TE2
.........................................ugugcuuggugcuuuagauuuggcc............................................    2    0    TE2
..............................................uuggugcuuuagauuuggccguuugg......................................    2    0    TE2
..................................................ugcuuuagauuuggcUguuugggg....................................    1    1    TE2
..................................................ugcuuuagauuuggcUguuuggggu...................................    1    1    TE2
..................................................ugcuuuagauuuggccguuuggggu...................................    2    0    TE2
..................................................ugcuuuagauuuggccguuugggguggacu..............................    1    0    TE2
.....................................................uuuagauuuggcUguuugggguggacu..............................    1    1    TE2
......................................................uuagauuuggcUguuuggggu...................................    1    1    TE2
.......................................................uagauuuggccguuugggg....................................    1    0    TE2
.......................................................uagauuuggcUguuuggggugga................................    1    1    TE2
```

miRBase precursor : aga-mir-10361a-1
Total read count : 2445
aga-mir-10361a-1 read count : 790
aga-mir-10361a-1-star read count : 25
aga-mir-10361a-2 read count : 790
aga-mir-10361a-2-star read count : 25
aga-mir-10361a-3 read count : 790
aga-mir-10361a-3-star read count : 25
remaining reads : 0

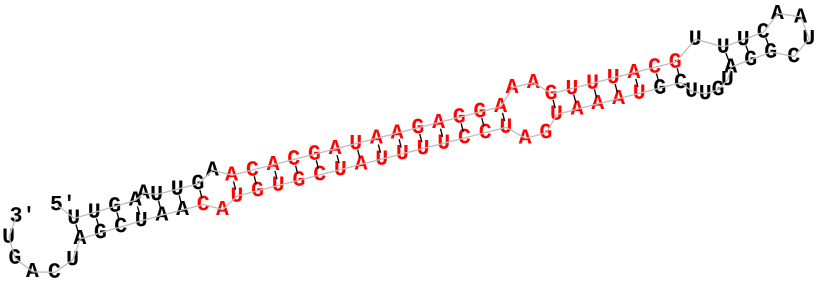

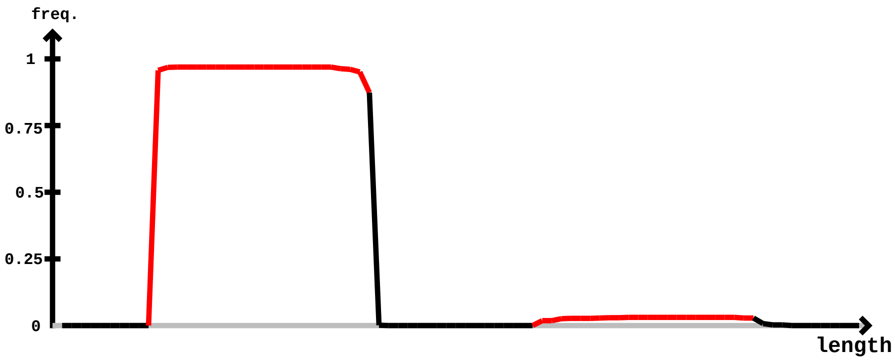

aga-mir-10361a-2-star
aga-mir-10361a-1-star
aga-mir-10361a-3
aga-mir-10361a-3-star
aga-mir-10361a-2
aga-mir-10361a-1

```
5'- uugaauugaacacgauaagaggaaaguuuacguuucaaucggauguucguaaaugauccuuuuaucguguacaaucgaucagu   -3'    exp
    ((((.(((.((((((((((((((..(((((((.(((....)))....)))))))..)))))))))))))).))))))).....    reads    mm    sample
    .........acacgauaagaggaaaguuuac....................................................    1        0     OV2
    .........acacgaCaagaggaaaguuuacg...................................................    1        1     OV2
    .........acacgauaagaggaaaguuuacg...................................................    2        0     OV2
    .................................................uaaaugauccuuuuaucguguac...........    1        0     OV2

    .........acacgauaagaggaaaguuu......................................................    1        0     TE1
    .........acacgauaagaggaaaguuua.....................................................    2        0     TE1
    .........acacgauaagaggaaaguuuac....................................................    20       0     TE1
    .........Gcacgauaagaggaaaguuuac....................................................    1        1     TE1
    .........acacgauaagaggaaaguuuacg...................................................    216      0     TE1
    ..........cacgauaagaggaaaguuuacg...................................................    4        0     TE1
    ...................................................aaugauccuuuuaucguguacU..........    1        1     TE1
    ....................................................augauccuuuuaucguguac...........    1        0     TE1

    .........acacgauaagaggaaaguuuac....................................................    1        0     MF2
    .........acacgauaagCggaaaguuuacg...................................................    1        1     MF2
    .........acacgauaagaggaGaguuuacg...................................................    1        1     MF2
    .........acacgauaagaggaaaguuuacg...................................................    82       0     MF2
    .................................................uaaaugauccuuuuaucguguac...........    1        0     MF2

    .........acacgauaagaggaaaguuuacg...................................................    1        0     FW2

    .........acacgauaagaggaaaguuuacg...................................................    17       0     OV1

    .........acacgauaagaggaaaguuuacg...................................................    1        0     FF1
    ..........cacgauaagaggaaaguuuacg...................................................    1        0     FF1
    .................................................uaaaugauccuuuuaucguguac...........    2        0     FF1
    ........................................................uccuuuuaucguguaca..........    1        0     FF1
    ..........................................................cuuuuaucguguacaUu........    1        1     FF1

    .........acacgauaagaggaaaguu.......................................................    2        0     MF1
```

```
                                                 aga-mir-10361a-2-star
                                                 aga-mir-10361a-1-star
uugaauugaacacgauaagaggaaaguuuacguuucaaucggauguucguaaaugauccuuuuaucguguacaaucgaucagu
         aga-mir-10361a-3
                                                 aga-mir-10361a-3-star
         aga-mir-10361a-2
         aga-mir-10361a-1


.........acacgauaagaggaaaguuuacg...................................................   2     0     MF1
.................................................uaaaugauccuuuuaucguguac...........   1     0     MF1
.......................................................auccuuuuaucguguacaUu........   1     1     MF1

.........acacgauaagaggaaaguuuacg...................................................   2     0     BF2
.................................................uaaaugauccuuuuaucguguacU..........   1     1     BF2

.........acacgauaagaggaaaguuuac....................................................   1     0     BF1
.........acacgauaagaggaaaguuuacg...................................................   4     0     BF1
.................................................uaaaugauccuuuuaucguguac...........   1     0     BF1

.........acacgauaagaggaaaguuuacg...................................................   1     0     MW1

.........acacgauaagaggaaaguuuacg...................................................   1     0     MW2

.........acacgauaagaggaaaguu.......................................................   3     0     TE2
.........acacgauaagaggaaaguuu......................................................   1     0     TE2
.........acacgauaagaggaaaguuua.....................................................   6     0     TE2
.........acacgauaagaAgaaaguuuac....................................................   1     1     TE2
.........acacgauaagaggaaaguuuac....................................................   35    0     TE2
.........acacgauaagaggaUaguuuacg...................................................   2     1     TE2
.........acacgauaagaggaaagCuuacg...................................................   1     1     TE2
.........Gcacgauaagaggaaaguuuacg...................................................   1     1     TE2
.........acacgauaagaAgaaaguuuacg...................................................   2     1     TE2
.........acacgauaaAaggaaaguuuacg...................................................   1     1     TE2
.........acacgauaagaggaaaguuuacg...................................................   365   0     TE2
.........acacgauaagaggaaaguuuacgA..................................................   1     1     TE2
..........cacgauaagaggaaaguuuac....................................................   2     0     TE2
..........cacgauaagaggaaaguuuacg...................................................   2     0     TE2
...........acgauaagaggaaaguuuac....................................................   1     0     TE2
.................................................uaaaugauccuuuuaucgugu.............   2     0     TE2
.................................................uaaaugauccuuuuaucguguac...........   5     0     TE2
.................................................uaaaugauccuuuuaucguguacU..........   1     1     TE2
...................................................aaugauccuuuuaucguguac...........   5     0     TE2
```

miRBase precursor : aga-mir-10361a-2
Total read count : 2445
aga-mir-10361a-1 read count : 790
aga-mir-10361a-1-star read count : 25
aga-mir-10361a-2 read count : 790
aga-mir-10361a-2-star read count : 25
aga-mir-10361a-3 read count : 790
aga-mir-10361a-3-star read count : 25
remaining reads : 0

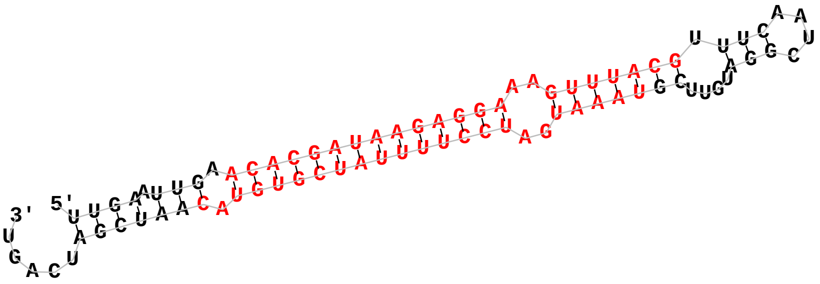

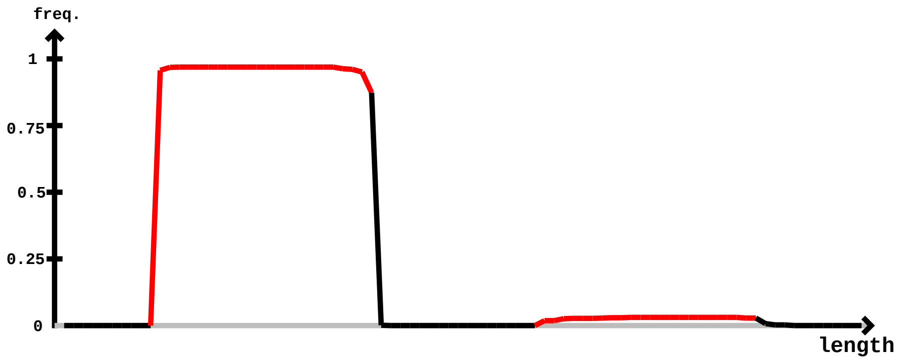


```
                                                  aga-mir-10361a-2-star
                                                  aga-mir-10361a-1-star
         aga-mir-10361a-3
                                                  aga-mir-10361a-3-star
         aga-mir-10361a-2
         aga-mir-10361a-1
5'- uugaauugaacacgauaagaggaaaguuuacguuucaaucggauguucguaaaugauccuuuuaucguguacaaucgaucagu   -3'    exp
    ((((.(((.((((((((((((((..(((((((.(((....)))....)))))))..)))))))))))))).))))))).....    reads   mm   sample
    .........acacgauaagaggaaaguuuac....................................................    1       0    OV2
    .........acacgaCaagaggaaaguuuacg...................................................    1       1    OV2
    .........acacgauaagaggaaaguuuacg...................................................    2       0    OV2
    .................................................uaaaugauccuuuuaucguguac...........    1       0    OV2

    .........acacgauaagaggaaaguuu......................................................    1       0    TE1
    .........acacgauaagaggaaaguuua.....................................................    2       0    TE1
    .........acacgauaagaggaaaguuuac....................................................    20      0    TE1
    .........Gcacgauaagaggaaaguuuac....................................................    1       1    TE1
    .........acacgauaagaggaaaguuuacg...................................................    216     0    TE1
    ..........cacgauaagaggaaaguuuacg...................................................    4       0    TE1
    ...................................................aaugauccuuuuaucguguacU..........    1       1    TE1
    ....................................................augauccuuuuaucguguac...........    1       0    TE1

    .........acacgauaagaggaaaguuuac....................................................    1       0    MF2
    .........acacgauaagCggaaaguuuacg...................................................    1       1    MF2
    .........acacgauaagaggaGaguuuacg...................................................    1       1    MF2
    .........acacgauaagaggaaaguuuacg...................................................    82      0    MF2
    .................................................uaaaugauccuuuuaucguguac...........    1       0    MF2

    .........acacgauaagaggaaaguuuacg...................................................    1       0    FW2

    .........acacgauaagaggaaaguuuacg...................................................    17      0    OV1

    .........acacgauaagaggaaaguuuacg...................................................    1       0    FF1
    ..........cacgauaagaggaaaguuuacg...................................................    1       0    FF1
    .................................................uaaaugauccuuuuaucguguac...........    2       0    FF1
    ........................................................uccuuuuaucguguaca..........    1       0    FF1
    ..........................................................cuuuuaucguguacaUu........    1       1    FF1

    .........acacgauaagaggaaaguu.......................................................    2       0    MF1
```

```
                                                 aga-mir-10361a-2-star
                                                 aga-mir-10361a-1-star
uugaauugaacacgauaagaggaaaguuuacguuucaaucggauguucguaaaugauccuuuuaucguguacaaucgaucagu
         aga-mir-10361a-3
                                                 aga-mir-10361a-3-star
         aga-mir-10361a-2
         aga-mir-10361a-1

.........acacgauaagaggaaaguuuacg...................................................    2     0     MF1
.................................................uaaaugauccuuuuaucguguac...........    1     0     MF1
.......................................................auccuuuuaucguguacaUu........    1     1     MF1

.........acacgauaagaggaaaguuuacg...................................................    2     0     BF2
.................................................uaaaugauccuuuuaucguguacU..........    1     1     BF2

.........acacgauaagaggaaaguuuac....................................................    1     0     BF1
.........acacgauaagaggaaaguuuacg...................................................    4     0     BF1
.................................................uaaaugauccuuuuaucguguac...........    1     0     BF1

.........acacgauaagaggaaaguuuacg...................................................    1     0     MW1

.........acacgauaagaggaaaguuuacg...................................................    1     0     MW2

.........acacgauaagaggaaaguu.......................................................    3     0     TE2
.........acacgauaagaggaaaguuu......................................................    1     0     TE2
.........acacgauaagaggaaaguuua.....................................................    6     0     TE2
.........acacgauaagaAgaaaguuuac....................................................    1     1     TE2
.........acacgauaagaggaaaguuuac....................................................    35    0     TE2
.........acacgauaagaggaUaguuuacg...................................................    2     1     TE2
.........acacgauaagaggaaagCuuacg...................................................    1     1     TE2
.........Gcacgauaagaggaaaguuuacg...................................................    1     1     TE2
.........acacgauaagaAgaaaguuuacg...................................................    2     1     TE2
.........acacgauaaAaggaaaguuuacg...................................................    1     1     TE2
.........acacgauaagaggaaaguuuacg...................................................    365   0     TE2
.........acacgauaagaggaaaguuuacgA..................................................    1     1     TE2
..........cacgauaagaggaaaguuuac....................................................    2     0     TE2
..........cacgauaagaggaaaguuuacg...................................................    2     0     TE2
...........acgauaagaggaaaguuuac....................................................    1     0     TE2
.................................................uaaaugauccuuuuaucgugu.............    2     0     TE2
.................................................uaaaugauccuuuuaucguguac...........    5     0     TE2
.................................................uaaaugauccuuuuaucguguacU..........    1     1     TE2
...................................................aaugauccuuuuaucguguac...........    5     0     TE2
```

miRBase precursor : aga-mir-10361a-3
Total read count : 2445
aga-mir-10361a-1 read count : 790
aga-mir-10361a-1-star read count : 25
aga-mir-10361a-2 read count : 790
aga-mir-10361a-2-star read count : 25
aga-mir-10361a-3 read count : 790
aga-mir-10361a-3-star read count : 25
remaining reads : 0

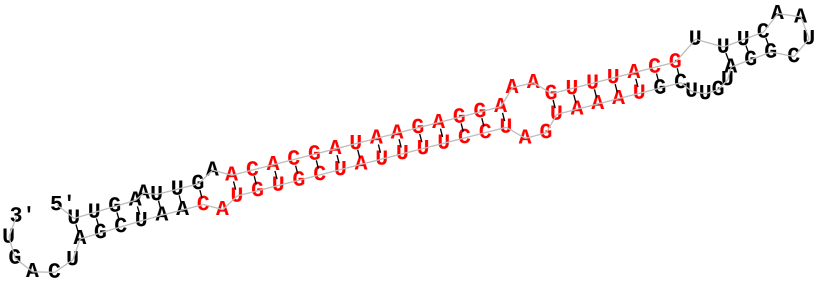

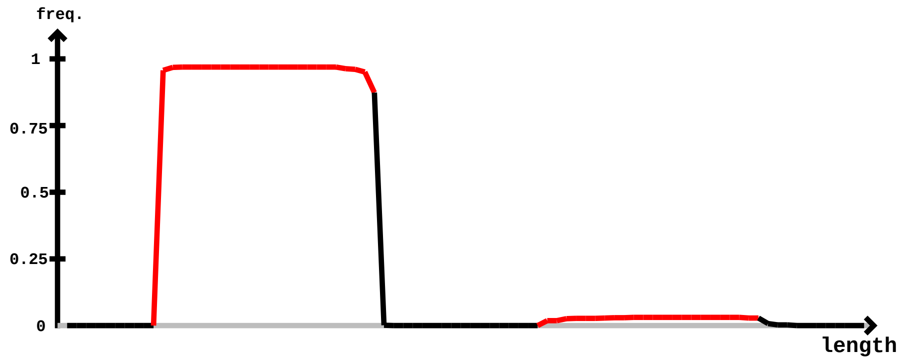


```
                                             aga-mir-10361a-2-star
                                             aga-mir-10361a-1-star
         aga-mir-10361a-3
                                             aga-mir-10361a-3-star
         aga-mir-10361a-2
         aga-mir-10361a-1
5'- uugaauugaacacgauaagaggaaaguuuacguuucaaucggauguucguaaaugauccuuuuaucguguacaaucgaucagu  -3'    exp
    ((((.(((.((((((((((((((..(((((((.(((....)))....)))))))..)))))))))))))).))))))).....   reads   mm   sample
    .........acacgauaagaggaaaguuuac....................................................   1       0    OV2
    .........acacgaCaagaggaaaguuuacg...................................................   1       1    OV2
    .........acacgauaagaggaaaguuuacg...................................................   2       0    OV2
    .................................................uaaaugauccuuuuaucguguac...........   1       0    OV2

    .........acacgauaagaggaaaguuu......................................................   1       0    TE1
    .........acacgauaagaggaaaguuua.....................................................   2       0    TE1
    .........acacgauaagaggaaaguuuac....................................................   20      0    TE1
    .........Gcacgauaagaggaaaguuuac....................................................   1       1    TE1
    .........acacgauaagaggaaaguuuacg...................................................   216     0    TE1
    ..........cacgauaagaggaaaguuuacg...................................................   4       0    TE1
    ...................................................aaugauccuuuuaucguguacU..........   1       1    TE1
    ....................................................augauccuuuuaucguguac...........   1       0    TE1

    .........acacgauaagaggaaaguuuac....................................................   1       0    MF2
    .........acacgauaagCggaaaguuuacg...................................................   1       1    MF2
    .........acacgauaagaggaGaguuuacg...................................................   1       1    MF2
    .........acacgauaagaggaaaguuuacg...................................................   82      0    MF2
    .................................................uaaaugauccuuuuaucguguac...........   1       0    MF2

    .........acacgauaagaggaaaguuuacg...................................................   1       0    FW2

    .........acacgauaagaggaaaguuuacg...................................................   17      0    OV1

    .........acacgauaagaggaaaguuuacg...................................................   1       0    FF1
    ..........cacgauaagaggaaaguuuacg...................................................   1       0    FF1
    .................................................uaaaugauccuuuuaucguguac...........   2       0    FF1
    ........................................................uccuuuuaucguguaca..........   1       0    FF1
    ..........................................................cuuuuaucguguacaUu........   1       1    FF1

    .........acacgauaagaggaaaguu.......................................................   2       0    MF1
```

```
                                                 aga-mir-10361a-2-star
                                                 aga-mir-10361a-1-star
uugaauugaacacgauaagaggaaaguuuacguuucaaucggauguucguaaaugauccuuuuaucguguacaaucgaucagu
         aga-mir-10361a-3
                                                 aga-mir-10361a-3-star
         aga-mir-10361a-2
         aga-mir-10361a-1
```

.........acacgauaagaggaaaguuuacg...	2	0	MF1
....uaaaugauccuuuuaucguguac...........	1	0	MF1
....auccuuuuaucguguacaUu........	1	1	MF1
.........acacgauaagaggaaaguuuacg...	2	0	BF2
....uaaaugauccuuuuaucguguacU..........	1	1	BF2
.........acacgauaagaggaaaguuuac..	1	0	BF1
.........acacgauaagaggaaaguuuacg...	4	0	BF1
....uaaaugauccuuuuaucguguac...........	1	0	BF1
.........acacgauaagaggaaaguuuacg...	1	0	MW1
.........acacgauaagaggaaaguuuacg...	1	0	MW2
.........acacgauaagaggaaaguu...	3	0	TE2
.........acacgauaagaggaaaguuu..	1	0	TE2
.........acacgauaagaggaaaguuua...	6	0	TE2
.........acacgauaagaAgaaaguuuac..	1	1	TE2
.........acacgauaagaggaaaguuuac..	35	0	TE2
.........acacgauaagaggaUaguuuacg...	2	1	TE2
.........acacgauaagaggaaagCuuacg...	1	1	TE2
.........Gcacgauaagaggaaaguuuacg...	1	1	TE2
.........acacgauaagaAgaaaguuuacg...	2	1	TE2
.........acacgauaaAaggaaaguuuacg...	1	1	TE2
.........acacgauaagaggaaaguuuacg...	365	0	TE2
.........acacgauaagaggaaaguuuacgA..	1	1	TE2
..........cacgauaagaggaaaguuuac..	2	0	TE2
..........cacgauaagaggaaaguuuacg...	2	0	TE2
...........acgauaagaggaaaguuuac..	1	0	TE2
....uaaaugauccuuuuaucgugu.............	2	0	TE2
....uaaaugauccuuuuaucguguac...........	5	0	TE2
....uaaaugauccuuuuaucguguacU..........	1	1	TE2
....aaugauccuuuuaucguguac...........	5	0	TE2

miRBase precursor : aga-mir-10361b
Total read count : 4708
aga-mir-10361b read count : 4289
aga-mir-10361b-star read count : 419
remaining reads : 0

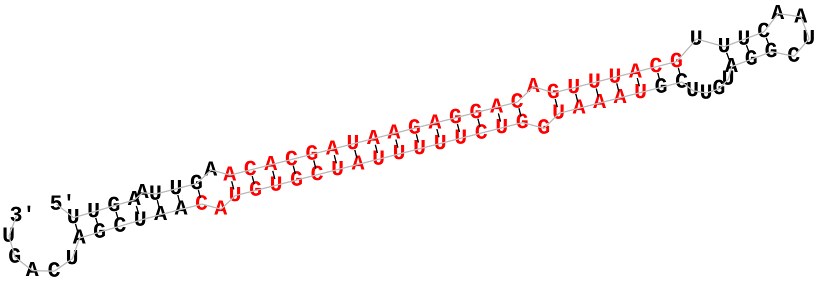

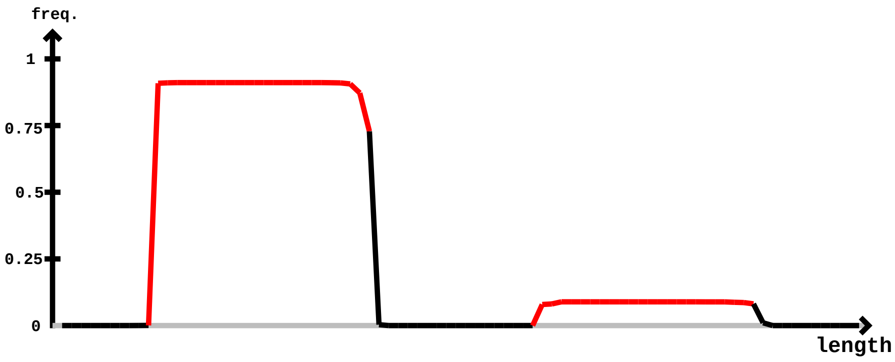


```
                                                  aga-mir-10361b-star
          aga-mir-10361b
5'- uugaauugaacacgauaagaggacaguuuacguuucaaucggauguucguaaauggucuuuuuaucguguacaaucgaucagu  -3'   exp
    ((((.(((.(((((((((((((((.(((((((.(((....)))....))))))).))))))))))))))).))))))).....   reads      mm        sample
    .................................................uaaauggucuuuuuaucgugua............   1          0         FF2

    .........acacgauaagaggacaguuuac....................................................   9          0         OV2
    .........acacgauaagaggacaguuuaA....................................................   1          1         OV2
    .........acacgauaagaggacaguuuacg...................................................   55         0         OV2
    .........acacCauaagaggacaguuuacg...................................................   1          1         OV2
    .................................................uaaauggucuuuuuaucguguac...........   3          0         OV2
    .................................................uaaauggucuuuuuaucguguaca..........   1          0         OV2

    .........acacgauaagaggacaguuu......................................................   2          0         TE1
    .........acacgauaagaggacaguuua.....................................................   30         0         TE1
    .........acacgauaagaggacaguuuaA....................................................   1          1         TE1
    .........acacgauaagaggacaguuuac....................................................   210        0         TE1
    .........acacgauaagaggacaguuuGc....................................................   1          1         TE1
    .........acacgauaaCaggacaguuuac....................................................   1          1         TE1
    .........acacgauaagaUgacaguuuacg...................................................   1          1         TE1
    .........acacgauaagaggacaguuuaAg...................................................   1          1         TE1
    .........acacgauaagaggacaguuuaGg...................................................   2          1         TE1
    .........acacgauaagaggacaguuuacg...................................................   1043       0         TE1
    .........acCcgauaagaggacaguuuacg...................................................   1          1         TE1
    .........acacgaCaagaggacaguuuacg...................................................   2          1         TE1
    .........acacgauaagGggacaguuuacg...................................................   1          1         TE1
    .........acaUgauaagaggacaguuuacg...................................................   1          1         TE1
    .........acacgauaagaggacagCuuacg...................................................   1          1         TE1
    .........aUacgauaagaggacaguuuacg...................................................   1          1         TE1
    .........acacAauaagaggacaguuuacg...................................................   1          1         TE1
    .........acacgauaGgaggacaguuuacg...................................................   1          1         TE1
    .........acGcgauaagaggacaguuuacg...................................................   1          1         TE1
    .........acacUauaagaggacaguuuacg...................................................   1          1         TE1
    .........acacgauaagaggacaguuuacA...................................................   2          1         TE1
    .........acacgauaagaggacaguuuacgA..................................................   5          1         TE1
    ..........cacgauaagaggacaguuuacg...................................................   3          0         TE1
    ...........acgauaagaggacaguuuacg...................................................   1          0         TE1
    .................................................uaaauggucuuuuuGucgug..............   2          1         TE1
```

```
                                                 aga-mir-10361b-star
         aga-mir-10361b
uugaauugaacacgauaagaggacaguuuacguuucaaucggauguucguaaauggucuuuuuaucguguacaaucgaucagu

.................................................uaaauggucuuuuuGucgugua............   6     1     TE1
.................................................uaaauggucuuuuuaucgugua............   3     0     TE1
.................................................uaaauggucuuuuuaucguguac...........   53    0     TE1
.................................................uaaauggucuuuuuGucguguac...........   32    1     TE1
.................................................uaaauggucuuuuuaucguguaca..........   5     0     TE1
.................................................uaaauggucuuuuuaucguguacU..........   5     1     TE1
.................................................uaaauggucuuuuuaucguguacC..........   1     1     TE1
.................................................uaaauggucuuuuuGucguguaca..........   2     1     TE1
..................................................aaauggucuuuuuaucguguacU..........   5     1     TE1
...................................................aauggucuuuuuaucguguac...........   4     0     TE1
...................................................aauggucuuuuuaCcguguac...........   1     1     TE1
...................................................aauggucuuuuuGucguguac...........   2     1     TE1
...................................................aauggucuuuuuaucguguacU..........   3     1     TE1
...................................................aauggucuuuuuGucguguaca..........   1     1     TE1

........Cacacgauaagaggacaguuuacg...................................................   1     1     MF2
.........acacgauaagaggacaguuua.....................................................   10    0     MF2
.........acacgauaagaggacaguuuac....................................................   65    0     MF2
.........acacgauaagaggacaguuuacA...................................................   2     1     MF2
.........acacgauaagaggacCguuuacg...................................................   1     1     MF2
.........acacgauaagaggacaguuuacg...................................................   433   0     MF2
.........acacgauaagaggaGaguuuacg...................................................   1     1     MF2
.........acacgauaagaCgacaguuuacg...................................................   1     1     MF2
.........aUacgauaagaggacaguuuacg...................................................   1     1     MF2
.........acacgauaagaggacaguuuacgA..................................................   5     1     MF2
..........cacgauaagaggacaguuuacg...................................................   2     0     MF2
...........acgauaagaggacaguuuacg...................................................   2     0     MF2
.................................................uaaauggucuuuuuGuc.................   1     1     MF2
.................................................uaaauggucuuuuuGucgug..............   1     1     MF2
.................................................uaaauggucuuuuuGucgugu.............   1     1     MF2
.................................................uaaauggucuuuuuGucgugua............   1     1     MF2
.................................................uaaauggucuuuuuaucgugua............   2     0     MF2
.................................................uaaauggucuuuuuGucguguac...........   12    1     MF2
.................................................uaaauggucuuuuuaucguguac...........   35    0     MF2
.................................................uaaauggucuuuuuGucguguaca..........   3     1     MF2
..................................................aaauggucuuuuuaucguguac...........   1     0     MF2
...................................................aauggucuuuuuGucguguac...........   2     1     MF2
...................................................aauggucuuuuuaucguguacU..........   3     1     MF2

.........acacgauaagaggacaguuua.....................................................   1     0     FF1
.........acacgauaagaggacaguuuacg...................................................   2     0     FF1

.........acacgauaagaggacagu........................................................   1     0     OV1
.........acacgauaagaggacaguuua.....................................................   1     0     OV1
.........acacgauaagaggacaguuuaU....................................................   1     1     OV1
.........acacgauaagaggacaguuuac....................................................   8     0     OV1
.........acacgauaagaggacaguuCacg...................................................   1     1     OV1
.........acaUgauaagaggacaguuuacg...................................................   1     1     OV1
.........acacgauaagaggacaguuuacg...................................................   70    0     OV1
.................................................uaaauggucuuuuuaucguguac...........   1     0     OV1

.........acacgauaagaggacaguuua.....................................................   1     0     MF1
.........acacgauaagaggacaguuuac....................................................   6     0     MF1
.........acacgauaagaggacaguuuacg...................................................   55    0     MF1
.........acacgauaagaggacUguuuacg...................................................   1     1     MF1
.........acacgauaagaggacaguuuaGg...................................................   1     1     MF1
.........acacgauaagagUacaguuuacg...................................................   1     1     MF1
.................................................uaaauggucuuuuuaucgugua............   1     0     MF1
.................................................uaaauggucuuuuuGucguguac...........   1     1     MF1
.................................................uaaauggucuuuuuaucguguac...........   6     0     MF1
..................................................aaauggucuuuuuaucguguac...........   1     0     MF1

.........acacgauaagaggacaguuuacg...................................................   29    0     BF2
.........acacgauaagaggacaguuuacgu..................................................   1     0     BF2
..........cacgauaagaggacaguuuacg...................................................   1     0     BF2
.................................................uaaauggucuuuuuaucguguac...........   6     0     BF2

.........acacgauaagaggacaguuuacg...................................................   51    0     BF1
.................................................uaaauggucuuuuuaucguguac...........   2     0     BF1
```

aga-mir-10361b-star

aga-mir-10361b

uugaauugaacacgauaagaggacaguuuacguuucaaucggauguucguaaauggucuuuuuaucguguacaaucgaucagu

.........acacgauaagaggacaguuuacg...	11	0	MW1
....uaaauggucuuuuuaucguguaU...........	1	1	MW1
.........acacgauaagaggacaguuuacg...	4	0	MW2
.......gaacacgauaagaggacaguuuacg...	1	0	TE2
........Cacacgauaagaggacaguuuacg...	1	1	TE2
.........acacgauaagaggacagu..	1	0	TE2
.........acacgauaagaggacaguu...	3	0	TE2
.........acacgauaagaggacaguuu..	16	0	TE2
.........acacgauaagaggacaguuua...	117	0	TE2
.........acacgauaagaggacaguuuaA..	4	1	TE2
.........acacgauaagagAacaguuuac..	1	1	TE2
.........acacgauaagaggacaguuCac..	1	1	TE2
.........acacgauaagCggacaguuuac..	1	1	TE2
.........acacgauaagaggacaguuuac..	367	0	TE2
.........acacgauaagaggacaguuuaUg...	1	1	TE2
.........acacgauaagaAgacaguuuacg...	2	1	TE2
.........acacgauaagCggacaguuuacg...	1	1	TE2
.........acacgauaagagAacaguuuacg...	1	1	TE2
.........acacgauaagaggaUaguuuacg...	2	1	TE2
.........acacCauaagaggacaguuuacg...	1	1	TE2
.........acacgauaagaggacaguuuacU...	1	1	TE2
.........Gcacgauaagaggacaguuuacg...	1	1	TE2
.........acacgUuaagaggacaguuuacg...	1	1	TE2
.........acacgauaagaggacaguuuacA...	12	1	TE2
.........acacgauaagaggUcaguuuacg...	1	1	TE2
.........acacgauaagaggacaguuuaGg...	1	1	TE2
.........acaUgauaagaggacaguuuacg...	1	1	TE2
.........acacgauGagaggacaguuuacg...	1	1	TE2
.........acacgauaagaggacaguuuacg...	1588	0	TE2
.........acacgauaagaCgacaguuuacg...	1	1	TE2
.........acacgauaagaggacaguuuacgu..	1	0	TE2
.........acacgauaagaggacaguuuacgA..	4	1	TE2
..........cacgauaagaggacaguuuacg...	2	0	TE2
...........acgauaagaggacaguuuacg...	1	0	TE2
....uaaauggucuuuuuaucgug..............	1	0	TE2
....uaaauggucuuuuuGucgug..............	3	1	TE2
....uaaauggucuuuuuaucgugu.............	2	0	TE2
....uaaauggucuuuuuGucgugu.............	2	1	TE2
....uaaauggucuuuuuaucgugua............	3	0	TE2
....uaaauggucuuuuuGucgugua............	6	1	TE2
....uaaauggucuuuuuGucguguac...........	40	1	TE2
....uaaauggucuuuuuaucguguac...........	116	0	TE2
....uaaauggucuuuuuGucguguaca..........	3	1	TE2
....uaaauggucuuuuuaucguguaca..........	3	0	TE2
....uaaauggucuuuuuaucguguacU..........	5	1	TE2
....aaauggucuuuuuaucguguacU..........	3	1	TE2
....aauggucuuuuuGucguguac...........	11	1	TE2
....aauggucuuuuuaucguguac...........	4	0	TE2
....aauggucuuuuuaucguguacU..........	6	1	TE2

miRBase precursor : aga-mir-10362
Total read count : 995
aga-mir-10362 read count : 839
aga-mir-10362-star read count : 52
remaining reads : 104

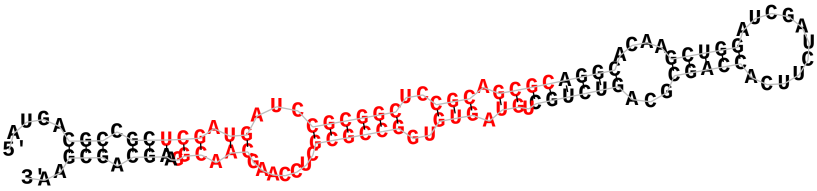

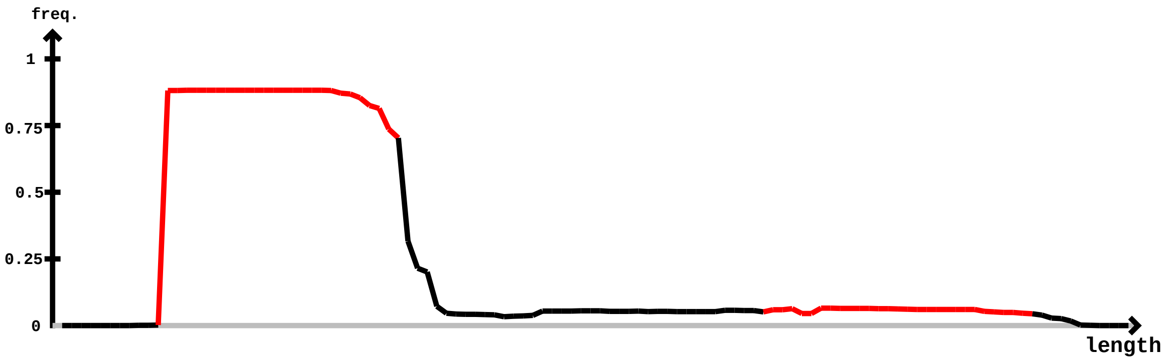


```
                  aga-mir-10362
                                                                    aga-mir-10362-star
5'- augacgccgcucgaugauccgcggcuccgcagcgcaggcacaagcuggaucgaucuucaccagcgcagucugcuguaguguggccgcgcuccaagcaacggaagcagcgaa  -3'   exp
    ....(((.((.........((((((..(((.((((((((....(((((...........)))))...)))))).)).))).)))))).(((.......))).)).)))..    reads   mm   sample
    ..........ucgaugauccgcggcuccgcagcg.............................................................................    1       0    TE1
    ..........ucgaugauccgcggcuccgcagcgc............................................................................    6       0    TE1
    ..........ucgaugauccgcggcuccgcagcgca...........................................................................    1       0    TE1
    ..........ucgaugauccgcggcuccgcaUcgca...........................................................................    1       1    TE1
    ..........ucgaugauccgcAgcuccgcagcgcagg.........................................................................    1       1    TE1
    .................................................ucgaucuucaccagUgcagucugcug....................................    1       1    TE1
    .................................................ucgaucuucaccagcgcagucugcugu...................................    2       0    TE1
    .....................................................ucuucaccagcgcagucugcuguagug...............................    1       0    TE1
    ..............................................................................uguggcUgcgcuccaagcaacggaagc......    1       1    TE1

    ..........ucgaugauccgcggcuccgcagc..............................................................................    1       0    FF2
    ..........ucgaugauccgcggcuccgcagcg.............................................................................    2       0    FF2

    ..........ucgaugauccgcggcucc...................................................................................    1       0    OV2
    ..........ucgaugauccgcggcuccg..................................................................................    1       0    OV2
    ..........ucgaugauccgcggcuccgc.................................................................................    3       0    OV2
    ..........ucgaugauccgcggcuccgca................................................................................    4       0    OV2
    ..........ucgaugauccgcAgcuccgca................................................................................    1       1    OV2
    ..........ucgaugauccgcggcuccgcag...............................................................................    3       0    OV2
    ..........ucgaugauccgcAgcuccgcagc..............................................................................    8       1    OV2
    ..........ucgaugauccgcggcuccgcagc..............................................................................    22      0    OV2
    ..........ucgaugauccgcggcuccgcagcg.............................................................................    6       0    OV2
    ..........ucgaugauccgcAgcuccgcagcg.............................................................................    2       1    OV2
    ..........ucgaugauccgcAgcuccgcagcgc............................................................................    19      1    OV2
    ..........ucgaugauccgcggcCccgcagcgc............................................................................    1       1    OV2
    ..........ucgaugauccgcggcuccgcagcgc............................................................................    78      0    OV2
    ..........ucgaugauccgcggcuccgcagcgca...........................................................................    15      0    OV2
    ..........ucgaugauccgcAgcuccgcagcgca...........................................................................    11      1    OV2
    ..........ucgaugauccgcggcuccgcagcgcaU..........................................................................    2       1    OV2
    ..........ucgaugauccgcAgcuccgcagcgcag..........................................................................    1       1    OV2
    ..........ucgaugauccgcggcuccgcagcgcagU.........................................................................    1       1    OV2
    ..........ucgaugauccgcAgcuccgcagcgcagg.........................................................................    31      1    OV2
    ..........ucgaugauccgcggcuccgcagcgcagg.........................................................................    3       0    OV2
    ..........ucgaugauccgcggcuccgcagcgcaggc........................................................................    1       0    OV2
```

```
          aga-mir-10362
                                                                         aga-mir-10362-star
augacgccgcucgaugauccgcggcuccgcagcgcaggcacaagcuggaucgaucuucaccagcgcagucugcuguaguguggccgcgcuccaagcaacggaagcagcgaa

..........ucgaugauccgcAgcuccgcagcgcaggc........................................................................    4    1    OV2
..........ucgaugauccgcggcuccgcagcgcaggA........................................................................    1    1    OV2
..........ucgaugauccgcggcuccgcagcgcaggcacaa....................................................................    1    0    OV2
..........ucgaugauccgcAgcuccgcagcgcaggcacaagc..................................................................    2    1    OV2
..........ucgaugauccgcggcuccgcagcgcaggcacaagc..................................................................    1    0    OV2
..........ucgaugauccgcAgcuccgcagcgcaggcacaagcugga..............................................................    1    1    OV2
..........ucgaugauccgcggcuccgcagcgcaggcacaagcugga..............................................................    2    0    OV2
.............................................uggaucgaucuucaccagcgcagucug.......................................    1    0    OV2
.................................................ucgaucuucaccagcgcagucugcug....................................    2    0    OV2
.................................................ucgaucuucaccagcgcagucugcAg....................................    1    1    OV2
.................................................ucgaucuucaccagUgcagucugcuguaguguggccgcg.......................    1    1    OV2
....................................................................ucugcuguaguguggcUgcgcuccaag................    3    1    OV2
......................................................................ugcuguaguguggcUgcgcuccaag................    1    1    OV2
......................................................................ugcuguaguguggccgcgcuccaag................    1    0    OV2
.........................................................................uguaguguggcUgcgcuccaagca..............    1    1    OV2
...........................................................................uaguguggcUgcgcuccaagcaac............    1    1    OV2
...........................................................................uaguguggcUgcgcuccaagcaacgg..........    3    1    OV2
...........................................................................uaguguggccgcgcuccaagcaacgg..........    1    0    OV2
...........................................................................uaguguggcUgcgcuccaagcaacgga.........    1    1    OV2
...........................................................................uaguguggccgcgcuccaagcaacggaag.......    1    0    OV2
..............................................................................uguggcUgcgcuccaagcaacggaag.......    2    1    OV2
..............................................................................uguggccgcgcuccaagcaacggaagc......    1    0    OV2

..........ucgaugauccgcggcuccgcagcgc............................................................................    2    0    MF2

.................................................ucgaucuucaccagcgcagucugcugu...................................    1    0    FW2

.......cgcucgaugauccgcggcuccgcag...............................................................................    1    0    OV1
..........ucgaugauccgcAgcuc....................................................................................    1    1    OV1
..........ucgaugauccgcggcucc...................................................................................    5    0    OV1
..........ucgaugauccgcggcuccg..................................................................................    1    0    OV1
..........ucgaugauccgcggcuccgc.................................................................................    8    0    OV1
..........ucgaugauccgcggcuccgca................................................................................    18   0    OV1
..........ucgaugauccUcggcuccgca................................................................................    1    1    OV1
..........ucgaugauccgcAgcuccgcag...............................................................................    3    1    OV1
..........ucgaugauccgcggcuccgcag...............................................................................    3    0    OV1
..........ucgaugauccgcggcuccgcagc..............................................................................    24   0    OV1
..........ucgaugauccgcAgcuccgcagc..............................................................................    7    1    OV1
..........ucgaugauccgcAgcuccgcagcg.............................................................................    1    1    OV1
..........ucgaugauccgcggcuccgcagcg.............................................................................    17   0    OV1
..........ucgaugauccgcAgcuccgcagcgc............................................................................    47   1    OV1
..........ucgGugauccgcggcuccgcagcgc............................................................................    1    1    OV1
..........ucgaugauccgAggcuccgcagcgc............................................................................    1    1    OV1
..........ucgaugauccgcggcuccgcagcgc............................................................................    139  0    OV1
..........ucgaugauccgcggcuccgcagcUc............................................................................    1    1    OV1
..........Gcgaugauccgcggcuccgcagcgc............................................................................    1    1    OV1
..........ucgaugauccgcggcuAcgcagcgc............................................................................    1    1    OV1
..........ucgaugauccgcggcuccgcagcgcU...........................................................................    1    1    OV1
..........ucgaugauccgcAgcuccgcagcgca...........................................................................    20   1    OV1
..........ucgaugauccgcggcuccgcagcgca...........................................................................    29   0    OV1
..........ucgaugauccgcggcuccgcagcgcag..........................................................................    4    0    OV1
..........ucgaugauccgcAgcuccgcagcgcag..........................................................................    1    1    OV1
..........ucgaugauccgcggcuccgcagcgcagg.........................................................................    2    0    OV1
..........ucgaugauccgcAgcuccgcagcgcagg.........................................................................    56   1    OV1
..........ucgaugauccgcAgcuccgcagcgcaggc........................................................................    8    1    OV1
..........ucgaugauccgcggcuccgcagcgcaggc........................................................................    2    0    OV1
..........ucgaugauccgcAgcuccgcagcgcaggca.......................................................................    1    1    OV1
..........ucgaugauccgcggcuccgcagcgcaggcac......................................................................    1    0    OV1
..........ucgaugauccgcggcuccgcagcgcaggcacaag...................................................................    1    0    OV1
..........ucgaugauccgcggcuccgcagcgcaggcacaagc..................................................................    5    0    OV1
..........ucgaugauccgcAgcuccgcagcgcaggcacaagc..................................................................    3    1    OV1
..........ucgaugauccgcggcuccgcagcgcaggcacaagcugga..............................................................    6    0    OV1
..........ucgaugauccgcAgcuccgcagcgcaggcacaagcugga..............................................................    6    1    OV1
............gaugauccgcAgcuccgcagcgcagg.........................................................................    1    1    OV1
.............................cagcgcaggcacaagcuggaucgaucu.......................................................    1    0    OV1
................................cgcaggcacaagcuggaucgaucu.......................................................    1    0    OV1
..................................caggcacaagcuggaucgaucuucac...................................................    1    0    OV1
.............................................uggaucgauUuucaccagcgcaguc.........................................    1    1    OV1
.............................................uggaucgaucuucaccagcgcaguc.........................................    1    0    OV1
```

```
          aga-mir-10362
                                                                        aga-mir-10362-star
augacgccgcucgaugauccgcggcuccgcagcgcaggcacaagcuggaucgaucuucaccagcgcagucugcuguaguguggccgcgcuccaagcaacggaagcagcgaa

.............................................uggaucgaucuucaccagcgcagucug.......................................  4  0  OV1
..............................................ggaucgaucuucaccagcgcagucugc......................................  1  0  OV1
...............................................gaucgaucuucaccagcgcagucugcAguaguguggccgc........................  1  1  OV1
................................................aucgaucuucaccagcgcagucugcAg....................................  1  1  OV1
.................................................ucgaucuucaccagUgcagucugcug....................................  1  1  OV1
.................................................ucgaucuucaccagcgcagucugcug....................................  1  0  OV1
.................................................ucgaucuucaccagcgcagucugcugu...................................  5  0  OV1
....................................................................ucugcuguaguguggcUgcgcuccaag................  2  1  OV1
.........................................................................uguaguguggcUgcgcuccaagc...............  1  1  OV1
.........................................................................uguaguguggcUgcgcuccaagcaac............  1  1  OV1
.........................................................................uguaguguggccgcgcuccaagcaacgg..........  1  0  OV1
.........................................................................uguaguguggcUgcgcuccaagcaacgga.........  2  1  OV1
...........................................................................uaguguggcUgcgcuccaagc...............  1  1  OV1
...........................................................................uaguguggcUgcgcuccaagcaacgga.........  5  1  OV1
...........................................................................uaguguggcUgcgcuccaagcaacggaa........  2  1  OV1
...........................................................................uaguguggcUgcgcuccaagcaacggaagc......  2  1  OV1
..............................................................................uguggcUgcgcuccaagcaacggaag.......  1  1  OV1
..............................................................................uguggccgcgcuccaagcaacggaag.......  1  0  OV1
..............................................................................uguggccgcgcuccaagcaacggaagc......  1  0  OV1
..............................................................................uguggcUgcgcuccaagcaacggaagc......  4  1  OV1

..........ucgaugauccgcggcucc...................................................................................  1  0  BF2
..........ucgaugauccgcggcuccg..................................................................................  2  0  BF2
..........ucgaugauccgcggcuccgc.................................................................................  2  0  BF2
..........ucgaugauccgcggcuccgcag...............................................................................  1  0  BF2
..........ucgaugauccgcAgcuccgcagc..............................................................................  1  1  BF2
..........ucgaugauccgcggcuccgcagcg.............................................................................  1  0  BF2
..........ucgaugauccgcggcuccgcagcgc............................................................................  29  0  BF2
..........ucgaugauccgcAgcuccgcagcgc............................................................................  6  1  BF2
..........ucgaugauccgcggcuccgcagcgca...........................................................................  8  0  BF2
..........ucgaugauccgcAgcuccgcagcgca...........................................................................  3  1  BF2
..........ucgaugauccgcggcuccgcagcgcaU..........................................................................  1  1  BF2
..........ucgaugauccgcAgcuccgcagcgcagg.........................................................................  5  1  BF2
..........ucgaugauccgcAgcuccgcagcgcaggc........................................................................  2  1  BF2
..........ucgaugauccgcggcuccgcagcgcaggca.......................................................................  1  0  BF2
..........ucgaugauccgcAgcuccgcagcgcaggcacaagc..................................................................  1  1  BF2
..........ucgaugauccgcggcuccgcagcgcaggcacaagc..................................................................  1  0  BF2
..........ucgaugauccgcggcuccgcagcgcaggcacaagcugga..............................................................  1  0  BF2
...................................aggcacaagcuggaucgaucuucac...................................................  1  0  BF2
....................................ggcacaagcuggaucgaucuucaccag................................................  1  0  BF2
.............................................uggaucgaucuucaccagcgcaguc.........................................  1  0  BF2
.................................................ucgaucuucaccagUgcagucugc......................................  1  1  BF2
.................................................ucgaucuucaccagcgcagucugcug....................................  3  0  BF2
.................................................ucgaucuucaccagUgcagucugcug....................................  1  1  BF2
.................................................ucgaucuucaccagcgcagucugcugu...................................  4  0  BF2
.................................................ucgaucuucaccagcgcagucugcAgu...................................  1  1  BF2
...........................................................ccagcgcagucugcuguaguguggc...........................  1  0  BF2
.............................................................agcgcagucugcuguaguguggcUg.........................  1  1  BF2
.........................................................................uguaguguggcUgcgcuccaagca..............  1  1  BF2
.........................................................................uguaguguggcUgcgcuccaagcaacg...........  1  1  BF2
.........................................................................uguaguguggcUgcgcuccaagcaacgga.........  2  1  BF2
...........................................................................uaguguggcUgcgcuccaagcaac............  1  1  BF2
...........................................................................uaguguggcUgcgcuccaagcaacggaag.......  1  1  BF2
...........................................................................uaguguggcUgcgcuccaagcaacggaagc......  1  1  BF2
...........................................................................uaguguggccgcgcuccaagcaacggaagcU.....  1  1  BF2
..............................................................................uguggcUgcgcuccaagcaacggaag.......  1  1  BF2
..............................................................................uguggcUgcgcuccaagcaacggaagc......  3  1  BF2
..............................................................................uguggccgcgcuccaagcaacggaagcag....  1  0  BF2

.........cucgaugauccgcAgcuccgcagcgca...........................................................................  1  1  BF1
..........ucgaugauccgcggcucc...................................................................................  1  0  BF1
..........ucgaugauccgcAgcucc...................................................................................  2  1  BF1
..........ucgaugauccgcggcuccgc.................................................................................  1  0  BF1
..........ucgaugauccgcggcuccgca................................................................................  2  0  BF1
..........ucgaugauccgcAgcuccgcag...............................................................................  1  1  BF1
..........ucgaugauccgcggcuccgcagc..............................................................................  9  0  BF1
..........ucgaugauccgcAgcuccgcagc..............................................................................  3  1  BF1
..........ucgaugauccgcAgcuccgcagcg.............................................................................  1  1  BF1
..........ucgaugauccgcggcuccgcagcg.............................................................................  1  0  BF1
```

```
          aga-mir-10362
                                                                        aga-mir-10362-star
augacgccgcucgaugauccgcggcuccgcagcgcaggcacaagcuggaucgaucuucaccagcgcagucugcuguaguguggccgcgcuccaagcaacggaagcagcgaa

..........ucgaugauccgcggcuccgcagcgc............................................................................   35    0    BF1
..........ucgaugauccgcAgcuccgcagcgc............................................................................   7     1    BF1
..........ucgaugauccgcAgcuccgcagcgca...........................................................................   4     1    BF1
..........ucgaugauccgcggcuccgcagcgca...........................................................................   6     0    BF1
..........ucgaugauccgcAgcuccgcagcgcag..........................................................................   2     1    BF1
..........ucgaugauccgcggcuccgcagcgcag..........................................................................   2     0    BF1
..........ucgaugauccgcggcuccgcagcgcaU..........................................................................   1     1    BF1
..........ucgaugauccgcAgcuccgcagcgcagg.........................................................................   20    1    BF1
..........ucgaugauccgcggcuccgcagcgcaggA........................................................................   1     1    BF1
..........ucgaugauccgcggcuccgcagcgcaggc........................................................................   2     0    BF1
..........ucgaugauccgcAgcuccgcagcgcaggc........................................................................   5     1    BF1
..........ucgaugauccgcggcuccgcagcgcaggca.......................................................................   1     0    BF1
..........ucgaugauccgcggcuccgcagcgcaggcacaagc..................................................................   1     0    BF1
..........ucgaugauccgcAgcuccgcagcgcaggcacaagc..................................................................   1     1    BF1
..........ucgaugauccgcggcuccgcagcgcaggcacaagcugga.............................................................   3     0    BF1
..........ucgaugauccgcAgcuccgcagcgcaggcacaagcugga.............................................................   1     1    BF1
..............................................ggaucgaucuucaccagcgcagucugcugu...................................   1     0    BF1
................................................aucgaucuucaccagcgcagucugc......................................   1     0    BF1
.................................................ucgaucuucaccagcgcagucugcug....................................   1     0    BF1
.................................................ucgaucuucaccagUgcagucugcug....................................   1     1    BF1
.................................................ucgaucuucaccagcgcagucugcAg....................................   1     1    BF1
.................................................ucgaucuucaccagcgcagucugcugu...................................   1     0    BF1
.........................................................................uguaguguggcUgcgcuccaagcaacg...........   1     1    BF1
...........................................................................uaguguggcUgcgcuccaagcaacgga.........   1     1    BF1

..........ucgaugauccgcggcuccgcagcgc............................................................................   1     0    FW1

..........ucgaugauccgcggcuccgca................................................................................   3     0    TE2
..........ucgaugauccgcggcuccgcagc..............................................................................   2     0    TE2
..........ucgaugauccgcggcuccgcagcg.............................................................................   2     0    TE2
..........ucgaugauccgcAgcuccgcagcgc............................................................................   3     1    TE2
..........ucgaugauccgcggcuccgcagcgc............................................................................   8     0    TE2
..........ucgaugauccgcAgcuccgcagcgca...........................................................................   1     1    TE2
..........ucgaugauccgcggcuccgcagcgca...........................................................................   1     0    TE2
..........ucgaugauccgcAgcuccgcagcgcagg.........................................................................   7     1    TE2
..........ucgaugauccgcggcuccgcagcgcagg.........................................................................   1     0    TE2
.................................................ucgaucuucaccagcgcagucugcug....................................   4     0    TE2
.................................................ucgaucuucaccagcgcagucugcugu...................................   3     0    TE2
..............................................................................uguggcUgcgcuccaagcaacggaag.......   2     1    TE2
..............................................................................uguggccgcgcuccaagcaacggaagc......   1     0    TE2
..............................................................................uguggcUgcgcuccaagcaacggaagc......   1     1    TE2
```

miRBase precursor : aga-mir-10363
Total read count : 646
aga-mir-10363 read count : 444
aga-mir-10363-star read count : 14
remaining reads : 188

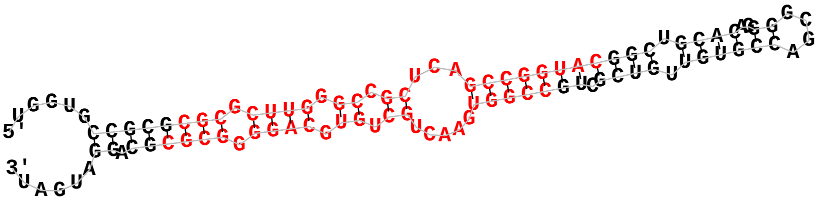

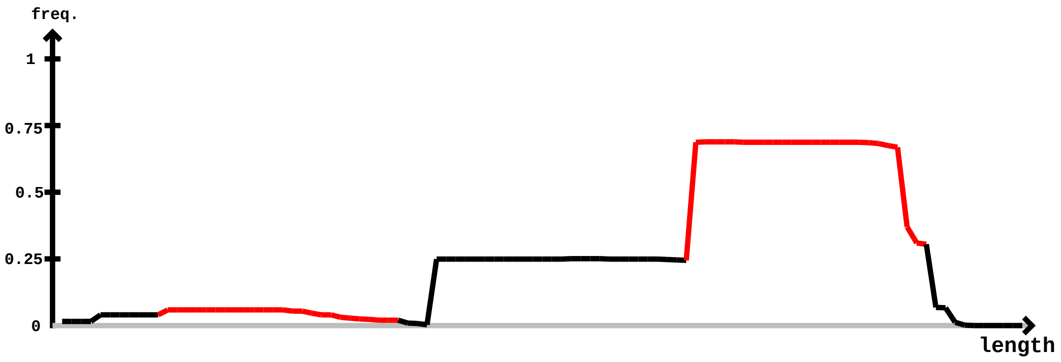


```
                                                                             aga-mir-10363
          aga-mir-10363-star
5'- uggugccgcgcgcgcuugggccgcucagccgguacggcugcacaggggcgaccguguugucgcugccggugaacugcugugcaggggcgcgcaggaugau  -3'   exp
    .....((((((((.((((.((.((...((((((((((.((((..((....)))))).)))).))))))).....)).)).)))).)))))).)).....   reads   mm   sample
    ......................................ugcacaggggcgaccgugAugucgcug...................................   1       1    FF2
    .................................................................ccggugaacugcugugcagggC.............   1       1    FF2
    .................................................................ccggugaacugcugugcaggggcgc..........   2       0    FF2

    ......................................ugcacaggggcgaccguguugucgcug...................................   1       0    TE1
    ......................................ugcacaggggcgaccgugAugucgcug...................................   4       1    TE1
    .................................................................ccggugaacugcugugcagggC.............   19      1    TE1
    .................................................................ccggugaacugcugugcaggggc............   9       0    TE1
    .................................................................ccggugaacugcugugcaggggcgc..........   15      0    TE1
    .................................................................ccggugaacugcugugcaggggcgcgc........   7       0    TE1
    .................................................................ccggugaacugcugugcaggggcgcgca.......   1       0    TE1

    uggugccgcgcgcgcuuggAccgcu...........................................................................   1       1    OV2
    ...ugccgcgcgcgcuuggAccgcuc..........................................................................   1       1    OV2
    ...ugccgcgcgcgcuuggAccgcucag........................................................................   2       1    OV2
    ..........cgcgcuuggAccgcucagccgguac.................................................................   3       1    OV2
    ..........cgcgcuugggccgcucagccgguacgg...............................................................   2       0    OV2
    .....................cgcucagccgguacCgc..............................................................   1       1    OV2
    ......................................ugcacaggggcgaccguguugucgc.....................................   1       0    OV2
    ......................................ugcacaggggcgaccgugAugucgc.....................................   1       1    OV2
    ......................................ugcacaggggcgaccgugAugucgcu....................................   1       1    OV2
    ......................................ugcacaggggcgaccgugAugucgcug...................................   40      1    OV2
    ......................................ugcacaggggcgaccguguugucgcug...................................   4       0    OV2
    ....................................................cgugAugucgcugccggu..............................   1       1    OV2
    .................................................................ccggugaacugcugugca.................   1       0    OV2
    .................................................................ccggugaacCgcugugcaggg..............   1       1    OV2
    .................................................................ccggugaacugcugugcaggg..............   1       0    OV2
    .................................................................ccggugaacugcugugcagggC.............   36      1    OV2
    .................................................................ccggugaacugcugugcaggggc............   7       0    OV2
    .................................................................ccggugaacugcugugcaggggcgc..........   22      0    OV2
    .................................................................ccggugaacugcuguAcaggggcgcgc........   1       1    OV2
    .................................................................ccggugaacugcugugcaggggcgcgc........   2       0    OV2
    .................................................................ccggugaacugcugugcaggggcgcgca.......   2       0    OV2

    ......................................ugcacaggggcgaccgugAugucgcug...................................   2       1    MF2
```

aga-mir-10363

aga-mir-10363-star

uggugccgcgcgcgcuugggccgcucagccgguacggcugcacaggggcgaccguguugucgcugccggugaacugcugugcaggggcgcgcaggaugau

....ccggugaacugcugugcagggC.............	6	1	MF2
....ccggugaacugcugugcaggggc............	3	0	MF2
....ccggugaacugcugugcaggggcg...........	1	0	MF2
....ccggugaacugcugugcaggggcgc..........	11	0	MF2
....ccggugaacugcugugcaggggcgcgc........	2	0	MF2
......................................ugcacaggggcgaccgugAugucgcug...................................	1	1	FW2
......................................ugcacaggggcgaccgug..	1	0	FF1
....ccggugaacugcugugcagg...............	1	0	FF1
....ccggugaacugcugugcagggC.............	2	1	FF1
....ccggugaacugcugugcaggggcgcgc........	2	0	FF1
....ccggugaacugcugugcaggggcgcgcag......	1	0	FF1
uggugccgcgcgcgcuuggAc...	1	1	OV1
uggugccgcgcgcgcuuggAccg...	1	1	OV1
uggugccgcgcgcgcuuggAccgcu...	2	1	OV1
uggugccgcgcgcgcuuggAccgcuc..	2	1	OV1
...ugccgcgcgcgcuuggAccg...	2	1	OV1
...ugccgcgcgcgcuuggAccgcucag...	2	1	OV1
...ugccgcgcgcgcuuggAccgcucagc..	1	1	OV1
..........cgcgcuuggAccgcucagccg...	1	1	OV1
..........cgcgcuuggAccgcucagccgguac...	2	1	OV1
..........cgcgcuugggccgcucagccgguacgg...	1	0	OV1
......................................ugcacaggggcgaccgugAugucg......................................	1	1	OV1
......................................ugcacaggggcgaccgugAugucgcug...................................	46	1	OV1
....ccggugaacCgcugugcag................	2	1	OV1
....ccggugaacugcugugcagg...............	2	0	OV1
....ccggugaacCgcugugcagg...............	1	1	OV1
....ccggugaacugcugugcagggC.............	55	1	OV1
....ccggugaacugcugugcaggggc............	3	0	OV1
....ccggugaacugcugugcaggggcgc..........	39	0	OV1
....ccggugaacugcugugcaggggcgcgc........	1	0	OV1
....ccggugaacugcugugcaggggcgcgca.......	2	0	OV1
....ccggugaacugcugugcagggC.............	1	1	MF1
....ccggugaacugcugugcaggggcgc..........	1	0	MF1
uggugccgcgcgcgcuuggAccgcu...	2	1	BF2
uggugccgcgcgcgcuuggAccgcuc..	1	1	BF2
...ugccgcgcgcgcuuggAccgcucag...	1	1	BF2
...ugccgcgcgcgcuuggAccgcucagcc...	2	1	BF2
..........cgcgcuuggAccgcucagccgguac...	1	1	BF2
..........cgcgcuugggccgcucagccgguacg..	1	0	BF2
......................................ugcacaggggcgaccgugAugucgcug...................................	19	1	BF2
....ccggugaacugcugugcagggC.............	25	1	BF2
....ccggugaacugcugugcaggggc............	1	0	BF2
....ccggugaacugcugugcaggggcgc..........	5	0	BF2
....ccggugaacugcugugcaggggcgcg.........	1	0	BF2
....ccggugaacugcugugcaggggcgcgc........	3	0	BF2
...ugccgcgcgcgcuuggAccgcucag...	1	1	BF1
...ugccgcgcgcgcuuggAccgcucagccgg...	2	1	BF1
..........cgcgcuuggAccgcucagccgguac...	1	1	BF1
......................................ugcacaggggcgaccgugAugucgcug...................................	15	1	BF1
......................................ugcacaggggcgaccguguugucgcug...................................	1	0	BF1
....ccggugaacugcugugcagggC.............	11	1	BF1
....ccggugaacugcugugcaggggcg...........	1	0	BF1
....ccggugaacugcugugcaggggcgc..........	7	0	BF1
....ccggugaacugcugugcaggggcgcgc........	5	0	BF1
....ccggugaacugcugugcaggggcgcgca.......	1	0	BF1
...ugccgcgcgcgcuuggAc...	1	1	TE2
...ugccgcgcgcgcuuggAccgcucagc..	1	1	TE2
.....................cgcucagccgguacCgc..	1	1	TE2
......................................ugcacaggggcgaccguguugucgcug...................................	11	0	TE2
......................................ugcacaggggcgaccgugAugucgcug...................................	11	1	TE2
....ugccggugaacugcugugcagggC.............	1	1	TE2
....ccggugaacugcugugcagg...............	1	0	TE2

```
                                                                 aga-mir-10363
          aga-mir-10363-star
uggugccgcgcgcgcuugggccgcucagccgguacggcugcacaggggcgaccguguugucgcugccggugaacugcugugcaggggcgcgcaggaugau

.................................................................ccggugaacugcugugcaggg..............    1     0     TE2
.................................................................ccggugaacugcugugcagggC.............    36    1     TE2
.................................................................ccggugaacugcugugcaggggc............    16    0     TE2
.................................................................ccggugaacugcugugcaggggcg...........    1     0     TE2
.................................................................ccggugaacugcugugcaggggcgc..........    51    0     TE2
.................................................................ccggugaacugcugugcaggggcgcgc........    12    0     TE2
.................................................................ccggugaacugcugugcaggggcgcgcU.......    1     1     TE2
..................................................................cggugaacugcugugcaggg..............    1     0     TE2
```

miRBase precursor : aga-mir-10364
Total read count : 417
aga-mir-10364 read count : 411
aga-mir-10364-star read count : 1
remaining reads : 5

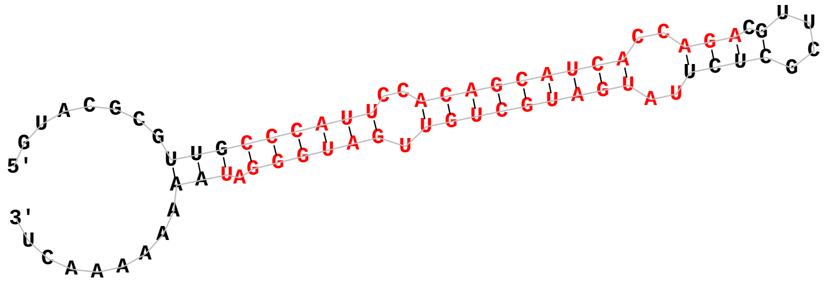

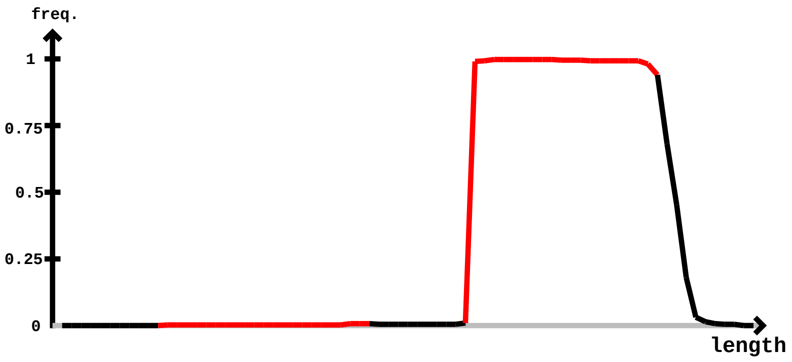


```
                         aga-mir-10364-star
                                                  aga-mir-10364
5'- guacgcguugcccauuccacagcaucaccagacguucgcucuuaugaugcuguugaugggauaaaaaaaacu  -3'   exp
    ..........((((((..(((((((((..(((.(....))))..))))))))).))))))............    reads    mm       sample
    ..........................................uaugaugcuguugaugggau..........    4        0        OV2
    ..........................................uaugaugcuguugaugggaua.........    2        0        OV2
    ..........................................uaugaugcuguugaugggauaa........    3        0        OV2
    ..........................................uaugaugcuguugaugggauaaa.......    1        0        OV2

    .............................agacguucgcucuuaugaugcuguu..................    1        0        TE1
    ..........................................uaugaugcuguugaugggG...........    1        1        TE1
    ..........................................uaugaugcuguugaugggau..........    8        0        TE1
    ..........................................uaugaugcuguugaugggaua.........    13       0        TE1
    ..........................................uaugaugcuguugaugggauaU........    1        1        TE1
    ..........................................uaugaugcuguugaugggauaa........    24       0        TE1
    ..........................................uaugaugcuguugaugggauaaa.......    6        0        TE1
    ..........................................uaugaugcuguugaugggauaaaa......    2        0        TE1
    ............................................ugaugcuguugaugggauaaa.......    1        0        TE1

    ..........................................uaugaugcuguugauggg............    1        0        MF2
    ..........................................uaugaugcuguugaugggau..........    5        0        MF2
    ..........................................uaugaugcuguugaugggaua.........    1        0        MF2
    ..........................................uaugaugcuguugaugggauaa........    8        0        MF2
    ..........................................uaugaugcuguugaugggauaaU.......    1        1        MF2
    ..........................................uaugaugcuguugaugggauaaa.......    4        0        MF2

    ..........................................uaugaugcuguugauggga...........    3        0        OV1
    ..........................................uaugaugcCguugauggga...........    1        1        OV1
    ..........................................uaugaugcuguugaugggau..........    17       0        OV1
    ..........................................uUugaugcuguugaugggau..........    1        1        OV1
    ..........................................uaugaugcuguugaugggaua.........    7        0        OV1
    ..........................................uaugaugcuguugaugggauU.........    1        1        OV1
    ..........................................uaugaugcuguugaugggauaa........    1        0        OV1
    ..........................................uaugaugcuguugaugggauaU........    2        1        OV1
    ..........................................uaugaugcuguugaugggauaaU.......    2        1        OV1
    ..........................................uaugaugcuguugaugggauaGa.......    1        1        OV1
    ..........................................uaugaugcuguugaugggauaaa.......    1        0        OV1
    ..........................................uaugaugcuguugaugggauaaaaU.....    1        1        OV1
```

```
          aga-mir-10364-star
                                          aga-mir-10364
guacgcguugcccauuccacagcaucaccagacguucgcucuuaugaugcuguugaugggauaaaaaaaacu

..........................................uaugaugcuguugaugggauaaaaaaaa..    2      0      OV1

..........................................uauUaugcuguugauggga...........    1      1      FF1
..........................................uaugaugcuguugaugggauaaa.......    1      0      FF1

..........................................uaugaugcuguugauggga...........    1      0      MF1
..........................................uaugaugcuguugaugggauU.........    1      1      MF1
..........................................uaugaugcuguugaugggaua.........    5      0      MF1
..........................................uaugaugcuguugaugggauaa........    1      0      MF1

..........................................uaugaugcuguugaugggau..........    2      0      BF2
..........................................uaugaugcuguugaugggaua.........    3      0      BF2
..........................................uaugaugcuguugaugggauaa........    4      0      BF2
..........................................uaugaugcuguugaugggauaaa.......    2      0      BF2
..........................................uaugaugcuguugaugggauaaaaa.....    1      0      BF2

..........................................uaugaugcuguugaugggaua.........    1      0      BF1
..........................................uaugaugcuguugaugggauaaa.......    1      0      BF1
..........................................uaugaugcuguugaugggauaaaaa.....    1      0      BF1
..........................................uaugaugcuguugaugggauaaaaaa....    1      0      BF1

..........................................uaugaugcuguugaugggauaaa.......    1      0      MW2

..........cccauuccacagcaucaccaga........................................    1      0      TE2
.............................agacguucgcucuuaugaugcu.....................    1      0      TE2
.........................................uuaugaugcuguugaugggau..........    1      0      TE2
.........................................uuaugaugcuguugaugggauaa........    1      0      TE2
..........................................uaugaugcuguugauggg............    4      0      TE2
..........................................uaugaugcuguugauggga...........    10     0      TE2
..........................................uaugaugcuguugaugggau..........    70     0      TE2
..........................................uaugaugcuguugaugggauU.........    2      1      TE2
..........................................uaugaugcuguugaugggaua.........    59     0      TE2
..........................................uaugaugcuguugaugggauaU........    3      1      TE2
..........................................uaugaugcuguugaugggauaa........    66     0      TE2
..........................................uaugaugcuguugaugggauaaU.......    4      1      TE2
..........................................uaugaugAuguugaugggauaaa.......    1      1      TE2
..........................................uaugaugcuguugaugggauaaa.......    33     0      TE2
..........................................uaugaugcuguugaugggauaaaU......    1      1      TE2
..........................................uaugaugcuguugaugggauaaaa......    4      0      TE2
...........................................augaugcuguugaugggauaaa.......    1      0      TE2
............................................ugaugcuguugaugggauaaa.......    1      0      TE2
```

miRBase precursor : aga-mir-10365
Total read count : 350
aga-mir-10365 read count : 266
aga-mir-10365-star read count : 71
remaining reads : 13

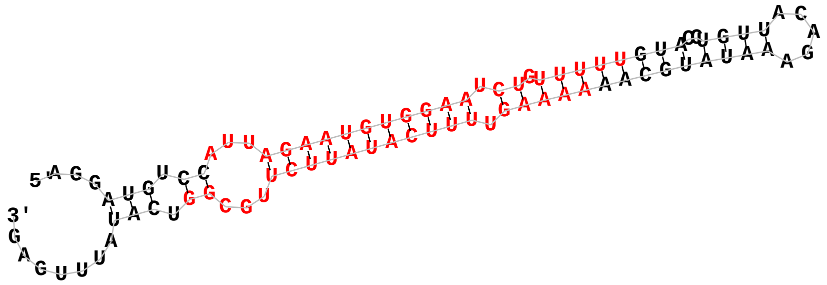

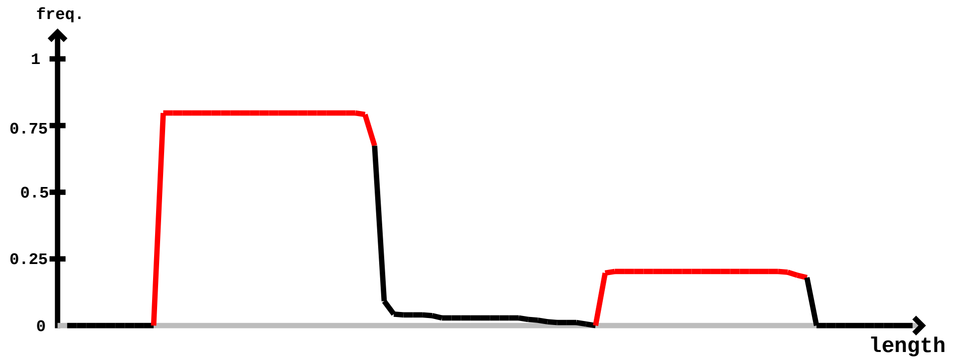


```
                aga-mir-10365
                                                    aga-mir-10365-star
5'- aggaugaccauuagaauguggaaucuguuuuuguaccuguuacagaaauaugcaaaaaaguuuucauauucuugcggucauauuugag   -3'    exp
    ...(((.((...((((((((((.((.((((((((..((((.....)))))))))))))).)))))))))))...)).)))..........     reads     mm      sample
    .........auuagaauguggaaucuguuuu.........................................................     1         0       TE1
    .........auuagaauguggaaucuguuuuu........................................................     3         0       TE1
    .........auuagaauguggaaucuguuuuuguaccu..................................................     1         0       TE1
    .......................................................aaaaguuuucauauucuugcgg...........     3         0       TE1

    .........auuagaauguggaaucuguuuu.........................................................     5         0       OV2
    .........auuagaauguggaaucuguuuuu........................................................     7         0       OV2
    .........auuagaauguggaaucuguuuuugA......................................................     1         1       OV2
    .......................................................aaaaguuuucauauucuugcgg...........     2         0       OV2

    .........auuagaauguggaaucuguuuu.........................................................     1         0       FF2
    .........auuagaauguggaaucuguuuuu........................................................     8         0       FF2
    .........auuagaauguggaaucCguuuuu........................................................     1         1       FF2
    .........auuagaauguggaaucuguuuuuguaccuguuacagaaa........................................     1         0       FF2
    .......................................................aaaaguuuucauauucuugcgg...........     1         0       FF2
    ........................................................aaaguuuucauauucuugcgg...........     1         0       FF2

    .........auuagaauguggaaucuguuuu.........................................................     6         0       MF2
    .........auuagaaugugCaaucuguuuuu........................................................     1         1       MF2
    .........auuagaauguggaaucuguuuuu........................................................     86        0       MF2
    .........auuagaauguggaaucuguuuuuU.......................................................     3         1       MF2
    .........auuagaauguggaaucuguuuuuA.......................................................     2         1       MF2
    .........auuagaauguggaaucuguuuuug.......................................................     1         0       MF2
    .........auuagaauguggaaucuguuuuuguacc...................................................     1         0       MF2
    .........auuagaauguggaaucuguuuuuguaccu..................................................     1         0       MF2
    .........auuagaauguggaaucuguuuuuguaccG..................................................     1         1       MF2
    .........auuagaauguggaaucuguuuuuguaccuguuacagaU.........................................     2         1       MF2
    .........auuagaauguggaaucuguuuuuguaccuguuacagaaau.......................................     1         0       MF2
    .......................................................aaaaguuuucauauucuugc.............     3         0       MF2
    .......................................................aaaaguuuucauauucuugcg............     2         0       MF2
    .......................................................aaaaguuuucauauucuugcgg...........     24        0       MF2
    ........................................................aaaguuuucauauucuugcgg...........     1         0       MF2

    .........auuagaauguggaaucuguuuu.........................................................     2         0       FW2
```

```
         aga-mir-10365
                                                       aga-mir-10365-star
aggauguccauuagaauguggaaucuguuuuuguaccuguuacagaaauaugcaaaaaaguuuucauauucuugcggucauauuugag

.........auuagaauguggaaucuguuuuu........................................................    3    0    FW2
.........auuagaauguggaaucuAuuuuu........................................................    1    1    FW2
.........auuagaauguggaaucuguuuuug.......................................................    1    0    FW2
.......................................................aaaaguuuucauauucuugcgg...........    1    0    FW2

.........auuagaauguggaaucuguuuu.........................................................    3    0    OV1
.........auuagaauguggaaucuguuuuu........................................................    11   0    OV1
.........auuagaaugAggaaucuguuuuu........................................................    1    1    OV1
.........auuagaauguggaaucuguuuuuA.......................................................    1    1    OV1
.......................................................aaaaguuuucauauucuug..............    1    0    OV1
.......................................................aaaaguuuucauauucuugcgg...........    3    0    OV1

.........auuagaauguggaaucuguuu..........................................................    1    0    FF1
.........auuagaauguggaaucuguuuu.........................................................    13   0    FF1
.........auuGgaauguggaaucuguuuuu........................................................    1    1    FF1
.........auuagaauguggaaucuguuuuu........................................................    30   0    FF1
.........auuagaauguggaaucugAuuuu........................................................    1    1    FF1
.........auuagaaugGggaaucuguuuuu........................................................    1    1    FF1
.........auuagaauguggaaucuguuuuuC.......................................................    1    1    FF1
.........auuagaauguggaaucuguuuuuA.......................................................    1    1    FF1
.........auuagaauguggaaucuguuuuuU.......................................................    2    1    FF1
.........auuagaauguggaaucuguuuuuguaccuguuacagaaau.......................................    1    0    FF1
.........auuagaauguggaaucuguuuuuguaccuguuacagaaaua......................................    1    0    FF1
.......................................................aaaaguuuucauauucuugc.............    1    0    FF1
.......................................................aaaaguuuucauauucuugcg............    1    0    FF1
.......................................................aaaaguuuucauauucuugcgg...........    19   0    FF1
.......................................................aaaagGuuucauauucuugcgg...........    1    1    FF1

.........auuagaauguggaaucuguuuu.........................................................    2    0    MF1
.........auuagaauguggaaucuguuuuu........................................................    15   0    MF1
.........auuagaauguggaaucuguuuuuU.......................................................    2    1    MF1
.........auuagaauguggaaucuguuuuuguaccuguuacagaaauaugc...................................    1    0    MF1
.......................................................aaaaguuuucauauucuugcgg...........    1    0    MF1

.........auuagaauguggaaucuguuuu.........................................................    1    0    BF2
.........auuagaauguggaaucuguuuuu........................................................    9    0    BF2
.........auuagaauguggaaucugCuuuu........................................................    1    1    BF2
.........auuagaauguggaaucuguuuuug.......................................................    2    0    BF2
.......................................................aaaaguuuucauauucuugcgg...........    1    0    BF2

.........auuagaauguggaaucuguuuuu........................................................    6    0    BF1
.........auuagaauguggaaucuguuuuuguaccuguuacagaaauaugc...................................    1    0    BF1
.........auuagaauguggaaucuguuuuuguaccuguuacagaaauaugcU..................................    2    1    BF1

.........auuagaauguggaaucuguuu..........................................................    1    0    FW1
.........auuagaauguggaaucuguuuu.........................................................    2    0    FW1
.........auuagaauguggaaucuguuuuu........................................................    6    0    FW1
.......................................................aaaaguuuucauauucuugcgg...........    1    0    FW1

.........auuagaauguggaaucuguuuu.........................................................    2    0    MW1
.........auuagaauguggaaucuguuuuu........................................................    5    0    MW1
.......................................................aaaaguuuucauauucuugcgg...........    2    0    MW1

.........auuagaauguggaaucuguuuuu........................................................    2    0    MW2
.........auuagaauguggaaucuguuuuug.......................................................    1    0    MW2
.......................................................aaaaguuuucauauucuugcgg...........    1    0    MW2

.........auuagaauguggaaucuguuuu.........................................................    3    0    TE2
.........auuagaauguggaaucuguuuuA........................................................    1    1    TE2
.........auuagaauguggaaucuguuuuu........................................................    4    0    TE2
.......................................................aaaaguuuucauauucuugcgg...........    1    0    TE2
```

miRBase precursor : aga-mir-10366a-1
Total read count : 356
aga-mir-10366a-1 read count : 136
aga-mir-10366a-1-star read count : 39
aga-mir-10366a-2 read count : 136
aga-mir-10366a-2-star read count : 28
remaining reads : 17

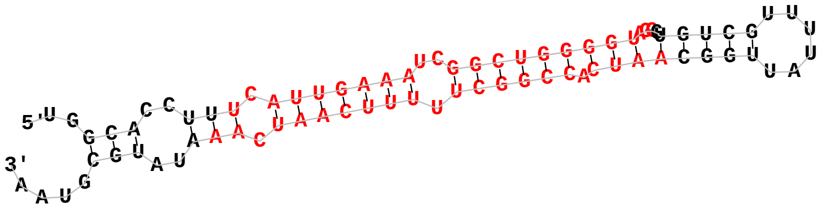

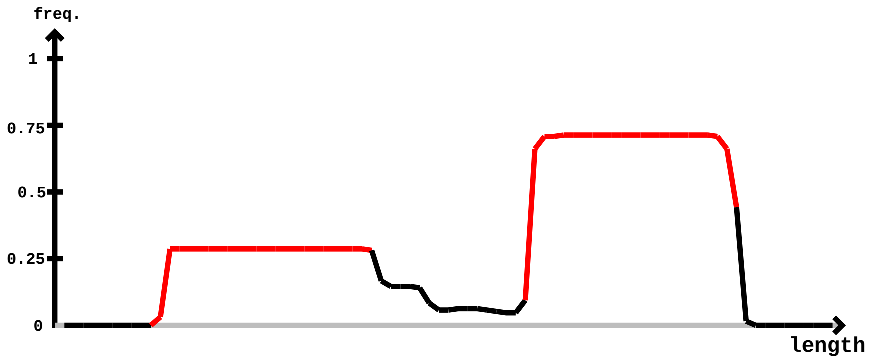


```
                                              aga-mir-10366a-2
          aga-mir-10366a-1-star
         aga-mir-10366a-2-star
                                              aga-mir-10366a-1
5'- uggcaccuuucauugaaaucggcugggguagcgugucguuuuauuggcaaucaccggcuuuuucaaucaaauaugcguaa   -3'    exp
    ..(((..(((.(((((((..((((((((....(((((......)))))))).)))))).))))))).))).)))....    reads      mm         sample
    ................................................aaucaccggcuuuuucaauca...........    3          0          MF1

    .........ucauugaaaucggcugggguagc................................................    1          0          TE1
    .........ucauugaaaucggcugggguagcgugucguuuuauuggca...............................    1          0          TE1
    ..........cauugaaaucggcugggguagc................................................    3          0          TE1
    ..........cauugaaaucggcugggguagcg...............................................    1          0          TE1
    ..........cauugaaaucggcugggguagcA...............................................    1          1          TE1
    ..........cauugaaaucggcugggguagcgugu............................................    1          0          TE1
    ..........cauugaaaucggcugggguagcguguc...........................................    3          0          TE1
    ..........cauugaaaucggcugggguagcgugucU..........................................    2          1          TE1
    ..........cauugaaaucggcugggguagcgugucguuuua.....................................    1          0          TE1
    ..........cauugaaaucggcugggguagcgugucguuuuauu...................................    1          0          TE1
    ..........cauugaaaucggcugggguagcgugucguuuuauugg.................................    2          0          TE1
    ..........cauugaaaucggcugggguagcgugucguuuuauuggc................................    4          0          TE1
    ................................................aaucaccggcuuuuucaauc............    1          0          TE1
    ................................................aaucaccggcuuuuucaauca...........    9          0          TE1
    ................................................aaucaccggcuuuuucaaucaa..........    10         0          TE1
    ................................................aaucaccggcuuuuucaaucaaa.........    1          0          TE1
    .................................................aucaccggcuuuuucaaucaa..........    3          0          TE1

    ..........cauugaaaucggcugggguagcgugucguuuuauuggc................................    1          0          BF2

    ................................................aaucaccggcuuuuucaauc............    1          0          MF2
    ................................................aaucaccggcuuuuucaauca...........    3          0          MF2
    ................................................aaucaccggcuuuuucaaucaa..........    10         0          MF2

    .........ucauugaaaucggcugggguag.................................................    1          0          TE2
    .........ucauugaaaucggcugggguagc................................................    3          0          TE2
    ..........cauugaaaucggcugggguagc................................................    15         0          TE2
    ..........cauugaaaucggcugggguagcg...............................................    2          0          TE2
    ..........cauugaaaucggcugggguagcguguc...........................................    8          0          TE2
    ..........cauugaaaucggcugggguagcgugucU..........................................    3          1          TE2
```

aga-mir-10366a-2

aga-mir-10366a-1-star

uggcaccuu[illegible]gugucguuuuauuggcaaucaccggcuuuuucaaucaaauaugcguaa

aga-mir-10366a-1

..........cauugaaaucggcuggggguagcgugucguuuuaA....................................	1	1	TE2
....uuauuggcaaucaccggcuuuuucaaucaa..........	1	0	TE2
....caaucaccggcuuuuucaauc............	2	0	TE2
....caaucaccggcuuuuucaauca...........	3	0	TE2
....caaucaccggcuuuuucaaucaa..........	6	0	TE2
....aaucaccggcuuuuucaau.............	1	0	TE2
....aaucaccggcuuuuucaauc............	5	0	TE2
....aauUaccggcuuuuucaauca...........	1	1	TE2
....aaucaccggcuuuuucaauca...........	23	0	TE2
....aaucaccggcCuuuucaaucaa..........	1	1	TE2
....aaucaccggcuuuuucaaucaa..........	43	0	TE2
....aaucaccggcuuuuucaaucaaa.........	1	0	TE2
....Gaucaccggcuuuuucaaucaaa.........	1	1	TE2
....aucaccggcuuuuucaaucaa..........	7	0	TE2
....caccggcuuuuucaaucaa..........	1	0	TE2

miRBase precursor : aga-mir-10366a-2
Total read count : 274
aga-mir-10366a-1 read count : 136
aga-mir-10366a-2 read count : 136
aga-mir-10366a-2-star read count : 1
remaining reads : 1

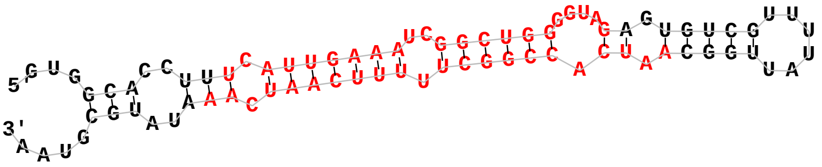

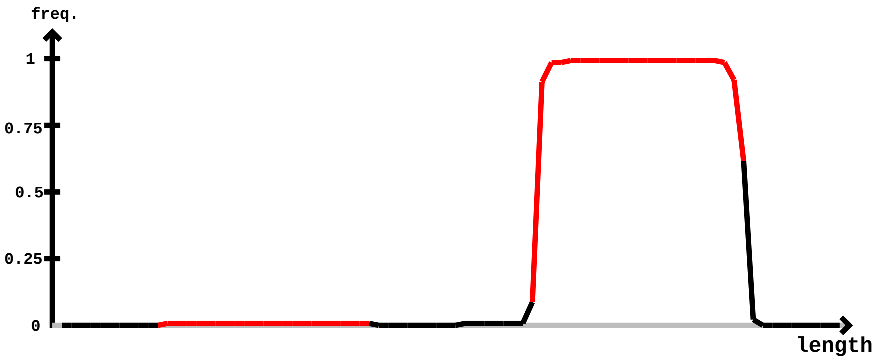


```
                                                aga-mir-10366a-2
            aga-mir-10366a-2-star
                                                aga-mir-10366a-1
5'- guggcaccuuucauugaaaucggcugggguagagugucguuuuauuggcaaucaccggcuuuuucaaucaaauaugcguaa   -3'    exp
    ...(((..(((.(((((((..((((((....((.(((((......))))).)).)))))).))))))).)))..)))....    reads      mm         sample
    .................................................aaucaccggcuuuuucaauca...........    3          0          MF1

    .................................................aaucaccggcuuuuucaauc.............    1          0          TE1
    .................................................aaucaccggcuuuuucaauca...........    9          0          TE1
    .................................................aaucaccggcuuuuucaaucaa..........    10         0          TE1
    .................................................aaucaccggcuuuuucaaucaaa.........    1          0          TE1
    ..................................................aucaccggcuuuuucaaucaa..........    3          0          TE1

    .................................................aaucaccggcuuuuucaauc.............    1          0          MF2
    .................................................aaucaccggcuuuuucaauca...........    3          0          MF2
    .................................................aaucaccggcuuuuucaaucaa..........    10         0          MF2

    ..........ucauugaaaucggcugggguag.................................................    1          0          TE2
    .........................................uuauuggcaaucaccggcuuuuucaaucaa..........    1          0          TE2
    ................................................caaucaccggcuuuuucaauc.............    2          0          TE2
    ................................................caaucaccggcuuuuucaauca............    3          0          TE2
    ................................................caaucaccggcuuuuucaaucaa..........    6          0          TE2
    .................................................aaucaccggcuuuuucaau..............    1          0          TE2
    .................................................aaucaccggcuuuuucaauc.............    5          0          TE2
    .................................................aaucaccggcuuuuucaauca...........    23         0          TE2
    .................................................aauUaccggcuuuuucaauca...........    1          1          TE2
    .................................................aaucaccggcuuuuucaaucaa..........    43         0          TE2
    .................................................aaucaccggcCuuuucaaucaa..........    1          1          TE2
    .................................................aaucaccggcuuuuucaaucaaa.........    1          0          TE2
    .................................................Gaucaccggcuuuuucaaucaaa.........    1          1          TE2
    ..................................................aucaccggcuuuuucaaucaa..........    7          0          TE2
    ....................................................caccggcuuuuucaaucaa..........    1          0          TE2
```

miRBase precursor : aga-mir-10366b
Total read count : 157
aga-mir-10366b read count : 124
aga-mir-10366b-star read count : 32
remaining reads : 1

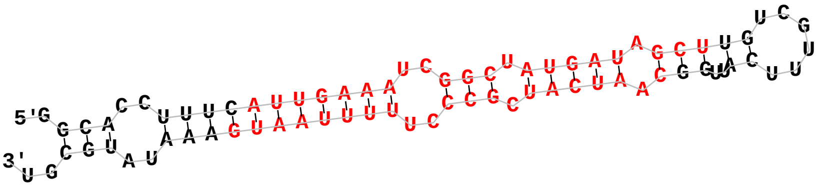

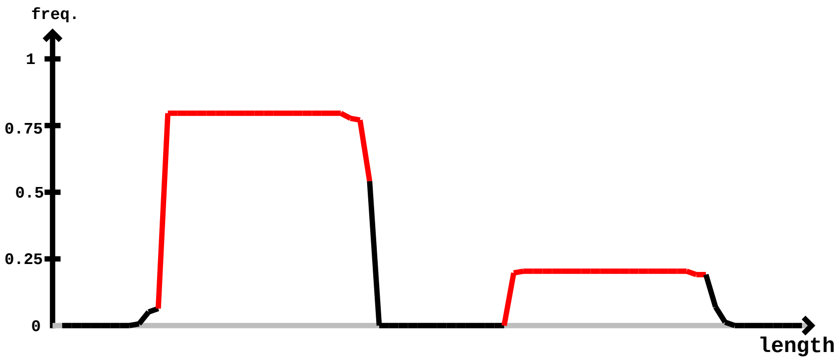


```
                                          aga-mir-10366b-star
            aga-mir-10366b
5'- ggcaccuuucauugaaaucggcuaugauagcuugucguuucauuggcaaucaucgcccuuuuuaaugaaauaugcgu   -3'    exp
    .(((..(((((((((((..(((.(((((.(((((......))..))).))))).))).)))))))))))..)))..    reads     mm        sample
    ..........auugaaaucggcuaugauagc..............................................    1         0         MF1
    ..........auugaaaucggcuaugauagcu.............................................    1         0         MF1

    ........ucauugaaaucggcuaugaua................................................    1         0         TE1
    ........ucauugaaaucggcuaugauagc..............................................    3         0         TE1
    .........cauugaaaucggcuaugauagc..............................................    1         0         TE1
    ..........auugaaaucggcuaugauagc..............................................    5         0         TE1
    ..........auugaaaucggcuaugauagcu.............................................    23        0         TE1
    ..............................................caaucaucgcccuuuuuaa............    1         0         TE1
    ..............................................caaucaucgcccuuuuuaaug..........    8         0         TE1
    ..............................................caaucaucgcccuuuuuaauga.........    6         0         TE1
    ..............................................caaucaucgcccuuuuuaaugaa........    1         0         TE1
    ...............................................aaucaucgcccuuuuuaaug..........    1         0         TE1

    ..............................................caaucaucgcccuuuuuaauga.........    1         0         MW1

    ..........auugaaaucggcuaugauagc..............................................    8         0         MF2
    ..........auugaaaucggcuaugauagcu.............................................    16        0         MF2
    ..............................................caaucaucgcccuuuuuaaug..........    3         0         MF2
    ..............................................caaucaucgcccuuuuuaauga.........    1         0         MF2

    .......uucauugaaaucggcuaugaua................................................    1         0         TE2
    ........ucauugaaaucggcuaugaua................................................    1         0         TE2
    ........ucauugaaaucggcuaugauag...............................................    1         0         TE2
    ........ucauugaaaucggcuaugauagc..............................................    1         0         TE2
    .........cauugaaaucggcuaugauagcu.............................................    1         0         TE2
    ..........auugaaaucggcuaugauagc..............................................    17        0         TE2
    ..........auugaaaucggcuaugauagcG.............................................    2         1         TE2
    ..........auugaaaucggcuaugauagcu.............................................    41        0         TE2
    ..........auugaaauUggcuaugauagcu.............................................    1         1         TE2
    ..............................................caaucaucgcccuuuuuaa............    1         0         TE2
    ..............................................caaucaucgcccuuuuuaaug..........    7         0         TE2
    ..............................................caaucaucgcccuuuuuaauga.........    1         0         TE2
    ..............................................caaucaucgcccuuuuuaaugaa........    1         0         TE2
```

aga-mir-10366b-star

aga-mir-10366b

ggcaccuuucauugaaaucggcuaugauagcuugucguuucauuggcaaucaucgcccuuuuuaaugaaauaugcgu

miRBase precursor : aga-mir-10367
Total read count : 163
aga-mir-10367 read count : 58
aga-mir-10367-star read count : 105
remaining reads : 0

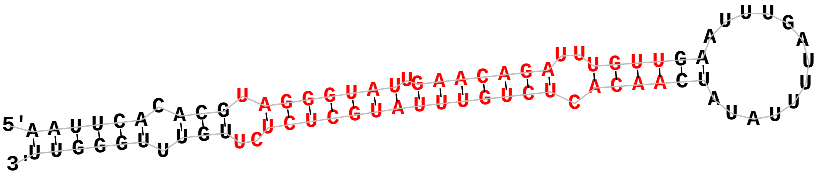

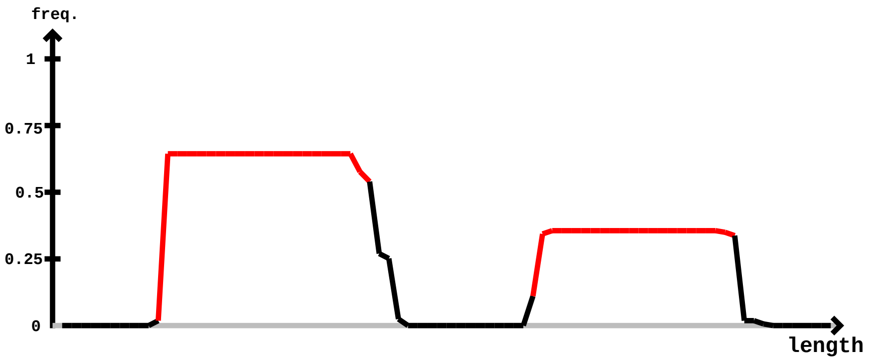

aga-mir-10367-star

aga-mir-10367

```
5'- aauucacacguaggguauugaacagauuuguugaauuugauuuuauaucaacacucuguuuaugcucucuuguuuggguu   -3'    exp
    ((((((.(((.(((((((.(((((((..((((((............)))))).))))))))))))))..))).))))))    reads    mm    sample
    ..........uaggguauugaacagauuug..................................................    4        0     TE1
    ..........uaggguauugaacagauuugu.................................................    1        0     TE1
    ..........uaggguauugaacagauuuguu................................................    15       0     TE1
    ..........uaggguauugaacagauuuguug...............................................    1        0     TE1
    ..........uaggguauugaacagauuuguuga..............................................    13       0     TE1
    ................................................caacacucuguuuaugcucucu..........    6        0     TE1
    .................................................aacacucuguuuaugcucucu..........    5        0     TE1

    ..........uaggguauugaacagauuuguu................................................    3        0     MF2
    ..........uaggguauugaacagauuuguuga..............................................    7        0     MF2
    ................................................caacacucuguuuaugcucucu..........    1        0     MF2

    ..........uaggguauugaacagauuuguuga..............................................    1        0     OV1

    .........guaggguauugaacagauuuguu................................................    1        0     MF1

    ..........uaggguauugaacagauuug..................................................    1        0     BF2

    .........guaggguauugaacagauuuguu................................................    2        0     MW1
    ..........uaggguauugaacagauuuguu................................................    1        0     MW1
    ..........uaggguauugaacagauuuguuga..............................................    1        0     MW1

    ..........uaggguauugaacagauuuguu................................................    1        0     FW1

    ..........uaggguauugaacagauuug..................................................    6        0     TE2
    ..........uaggguauugaacagauuugu.................................................    5        0     TE2
    ..........uaggguauugaacagauuuguu................................................    21       0     TE2
    ..........uaggguauugaacagauuuguug...............................................    2        0     TE2
    ..........uaggguauugaacagauuuguugG..............................................    1        1     TE2
    ..........uaggguauugaacagauuuguuga..............................................    14       0     TE2
    ..........uaggguauugaacagauuuguugaa.............................................    4        0     TE2
    ................................................caacacucuguuuUugcucu............    1        1     TE2
    ................................................caacacucuguuuaugcucuc...........    1        0     TE2
    ................................................caacacucuguuuaugcucucu..........    9        0     TE2
```

```
          aga-mir-10367-star
                                       aga-mir-10367
aauucacacguaggguauugaacagauuuguugaauuugauuuuauaucaacacucuguuuaugcucucuuguuuggguu

................................................aacacucuguuuaugcucuc...........    1          0          TE2
................................................aacacucuguuuaCgcucucu..........    1          1          TE2
................................................aacacucuguuuaugcucucu..........    29         0          TE2
................................................aacacucuguuuaugcucucuug........    2          0          TE2
.................................................acacucuguuuaugcucucu..........    1          0          TE2
.................................................acacucuguuuaugcucucuugu.......    1          0          TE2
```

miRBase precursor : aga-mir-10368
Total read count : 114
aga-mir-10368 read count : 110
aga-mir-10368-star read count : 2
remaining reads : 2

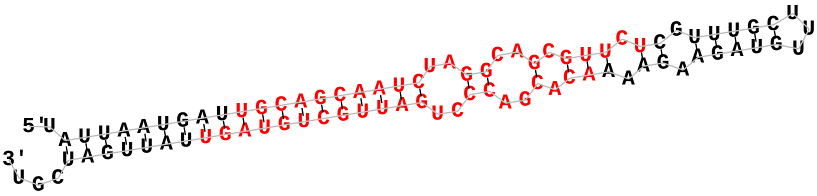

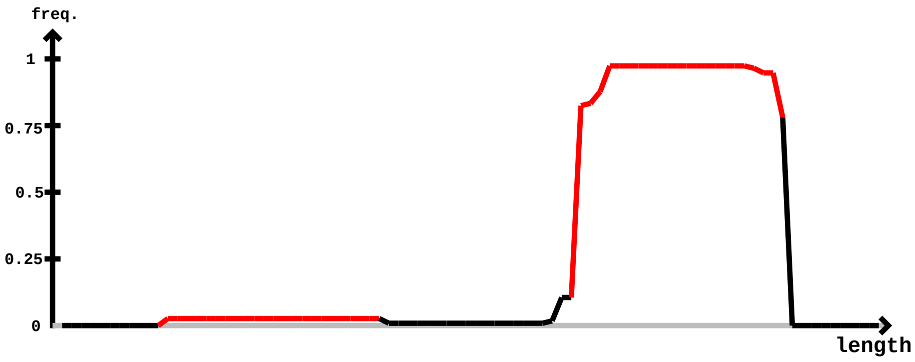


```
                                                                aga-mir-10368
          aga-mir-10368-star
5'- uauuaaugauugcagcaaucuaggcagcguucucguuugcuuuguagaagaaaacacgacccugauugcuguaguuauugaucgu  -3'   exp
    .(((((((((((((((((((..((..(.(((.((.(((((...))))).)).))).)..))..)))))))))))))))))))...    reads    mm    sample
    .....................................................acacgacccugauugcuguagu..........    2        0     OV2

    .....................................................acacgacccugauugcuguag...........    1        0     MF1
    .....................................................acacgacccugauugcuguagu..........    2        0     MF1

    ..........ugcagcaaucuaggcagcguucucguuugcuuuguagaaga..................................    1        0     TE1
    ...................................................aaacacgacccugauugcuguag...........    5        0     TE1
    .....................................................acacgacccugauugcug..............    1        0     TE1
    .....................................................acacgacccugauugcugu.............    1        0     TE1
    .....................................................acacgacccugauugcuguag...........    2        0     TE1
    .....................................................acacgacccugauugcuguagu..........    18       0     TE1
    ........................................................cgacccugauugcuguagu..........    4        0     TE1

    .....................................................acacgacccugauugcuguagu..........    2        0     MW2

    ..................................................aaaacacgacccugauugcuguag...........    1        0     MF2
    ...................................................aaacacgacccugauugcuguag...........    1        0     MF2
    .....................................................acacgacccugauugcuguag...........    1        0     MF2
    .....................................................acacgacccugauugcuguagu..........    7        0     MF2
    .......................................................acgacUcugauugcuguagu..........    1        1     MF2
    .......................................................acgacccugauugcuguagu..........    2        0     MF2

    ..........ugcagcaaucuaggcagcguucu....................................................    2        0     TE2
    ...................................................aaacacgacccugauugcuguag...........    4        0     TE2
    ...................................................Caacacgacccugauugcuguag...........    1        1     TE2
    .....................................................acacgacccugauugcugu.............    1        0     TE2
    .....................................................acacgacccugauugcuguag...........    3        0     TE2
    .....................................................acacgacccugauugcuguagu..........    40       0     TE2
    ......................................................cacgacccugauugcuguagu..........    1        0     TE2
    .......................................................acgacccugauugcuguagu..........    2        0     TE2
    ........................................................cgacccugauugcuguagu..........    7        0     TE2

    .....................................................acacgacccugauugcuguagu..........    1        0     OV1
```

miRBase precursor : aga-mir-10369
Total read count : 114
aga-mir-10369 read count : 111
aga-mir-10369-star read count : 3
remaining reads : 0

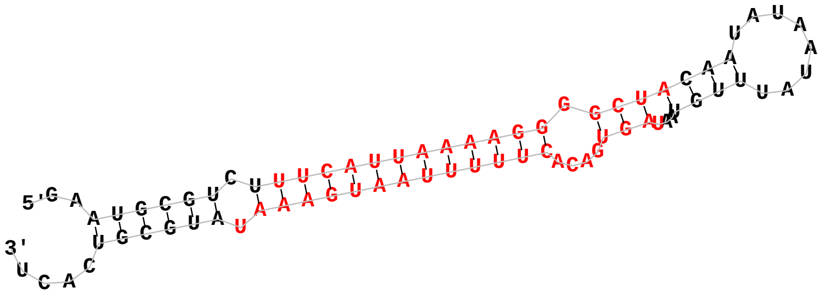

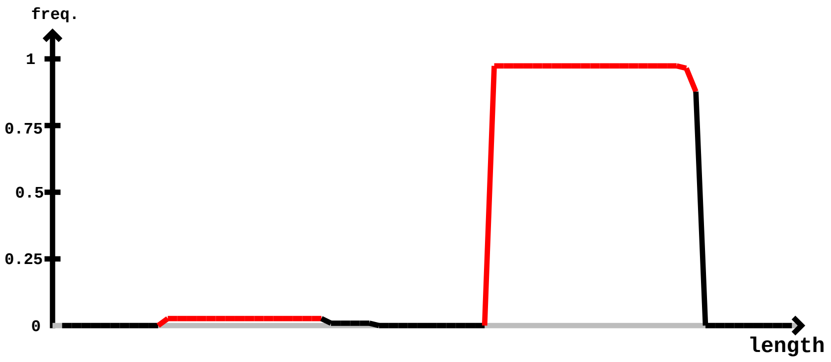


```
                      aga-mir-10369-star
                                                   aga-mir-10369
5'- gaaugcgucuuucauuaaaaggggcuacaauauaauauuuguaauagugacacuuuuuaaugaaauaugcgucacu   -3'    exp
    ((.(((((.((((((((((((.(((((((........))))...)))....))))))))))))).)))))))...    reads      mm         sample
    ...........................................uagugacacuuuuuaaugaaau..........    1          0          MF1

    ..........uucauuaaaaggggcuU................................................    1          1          TE1
    ...........................................uagugacacuuuuuaaugaa............    1          0          TE1
    ...........................................uagugacacuuuuuaaugaaa...........    1          0          TE1
    ...........................................uagugacaUuuuuuaaugaaau..........    1          1          TE1
    ...........................................uagugacacuuuuuaaugaaaG..........    1          1          TE1
    ...........................................uagugacacuuuuuaaugaaau..........    32         0          TE1

    ...........................................uagugacacuuuuuaaugaaau..........    1          0          BF2

    ...........................................uagugacacuuuuuaaugaaau..........    2          0          MW1

    ..........uucauuaaaaggggcuacaaua...........................................    1          0          MF2
    ...........................................uagugacacuuuuuaaugaaa...........    2          0          MF2
    ...........................................uagugacacuuuuuaaugaaau..........    20         0          MF2
    ...........................................uagugacacuuCuuaaugaaau..........    1          1          MF2

    ..........uucauuaaaaggggcuU................................................    1          1          TE2
    ...........................................uagugacacuuuuuaaugaaa...........    7          0          TE2
    ...........................................uagugacacCuuuuaaugaaau..........    1          1          TE2
    ...........................................uagugacacuuuuuaaugaaau..........    40         0          TE2
```

miRBase precursor : aga-mir-10370-1
Total read count : 199
aga-mir-10370-1 read count: 95
aga-mir-10370-1-star read count: 0
aga-mir-10370-2 read count: 95
remaining reads : 0

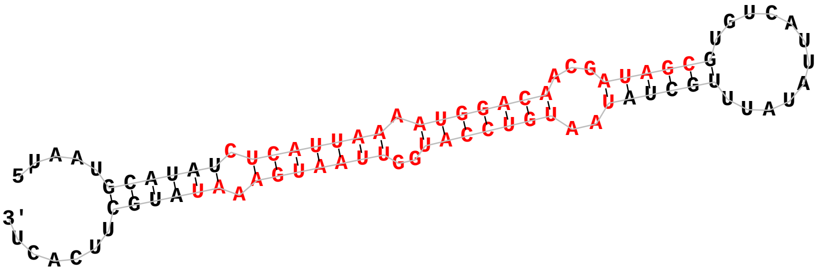

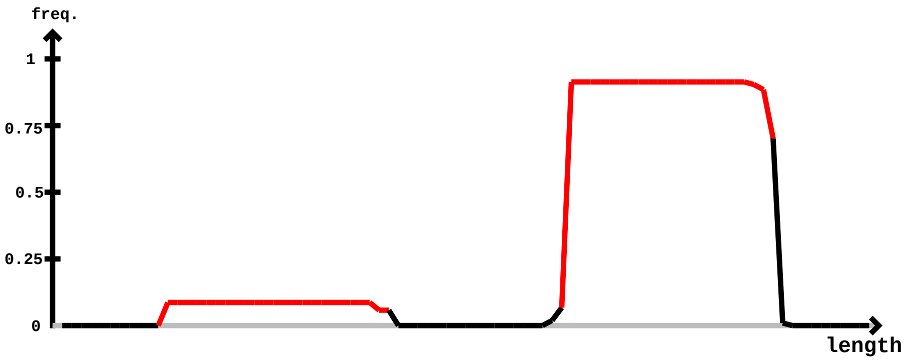


```
                                  aga-mir-10370-1-star
                                                                aga-mir-10370-1
                                                                aga-mir-10370-2
5'- uaaugcauaucucauuaaaauggacaacgauagcgugucauuauauuugcuauaaauguccaugguuaaugaaauaugcuucacu -3'    exp
    ....((((((.(((((((.(((((((...((((((............))))))..)))))))..))))))).))))))......    reads   mm   sample
    ....................................................uaauguccaugguuaaugaaau..........    2       0    MF1

    ..........cucauuaaaauggacaacgauagc..................................................    1       0    TE1
    ...................................................auaauguccaugguuaaugaaau..........    1       0    TE1
    ....................................................uaauguccaugguuaauga.............    1       0    TE1
    ....................................................uaauguccaugguuaaugaaa...........    6       0    TE1
    ....................................................uaaugCccaugguuaaugaaau..........    1       1    TE1
    ....................................................uaauguccaugguuaaugaaau..........    11      0    TE1

    ....................................................uaauguccaugguuaaugaaau..........    1       0    MW1

    ..........cucauuaaaauggacaacgauagc..................................................    5       0    TE2
    ...................................................auaauguccaugguuaaugaaa...........    1       0    TE2
    ...................................................auaauguccaugguuaaugaaau..........    1       0    TE2
    ....................................................uaauguccaugguuaaugaaa...........    11      0    TE2
    ....................................................uaauguccaugguuaaugaaau..........    46      0    TE2
    ....................................................uaauguccaugguuaaugaaaua........    1       0    TE2

    ..........cucauuaaaauggacaacgaua....................................................    3       0    MF2
    ..................................................uauaauguccaugguuaaugaa............    2       0    MF2
    ...................................................auaauguccaugguuaaugaaau..........    2       0    MF2
    ....................................................uaauguccaugguuaaugaaa...........    1       0    MF2
    ....................................................uaauguccaugguuaaugaaau..........    7       0    MF2
```

miRBase precursor : aga-mir-10370-2
Total read count : 203
aga-mir-10370-1 read count: 95
aga-mir-10370-2 read count: 95
aga-mir-10370-2-star read count: 6
remaining reads : 7

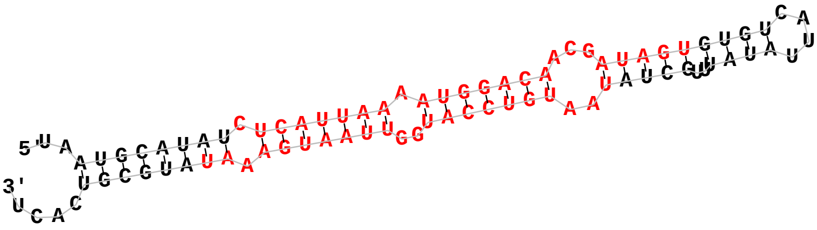

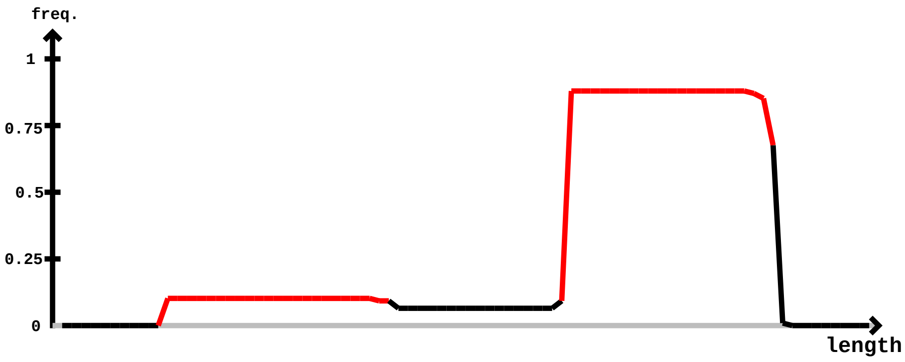


```
           aga-mir-10370-2-star
                                                aga-mir-10370-1
                                                aga-mir-10370-2
5'- uaaugcauaucucauuaaaauggacaacgauagugugucauuauauuugcuauaauguccaugguuaaugaaauaugcgucacu   -3'    exp
    ..((((((((.(((((((.(((((((...((((((((((....))))..)))))..)))))))..))))))).))))))))....    reads      mm        sample
    ..........cucauuaaaauggacaacgauagugugucauuauauuugcuU...............................    1          1         MF1
    ....................................................uaauguccaugguuaaugaaau..........    2          0         MF1

    ..........cucauuaaaauggacaacgauagu..................................................    1          0         TE1
    ..........cucauuaaaauggacaacgauagugugucauuauauuugc..................................    2          0         TE1
    ..........cucauuaaaauggacaacgauagugugucauuauauuugcu.................................    2          0         TE1
    ...................................................auaauguccaugguuaaugaaau..........    1          0         TE1
    ....................................................uaauguccaugguuaauga.............    1          0         TE1
    ....................................................uaauguccaugguuaaugaa............    6          0         TE1
    ....................................................uaauguccaugguuaaugaaau..........    11         0         TE1
    ....................................................uaaugCccaugguuaaugaaau..........    1          1         TE1

    ....................................................uaauguccaugguuaaugaaau..........    1          0         MW1

    ..........cucauuaaaauggacaacgauagu..................................................    1          0         TE2
    ...................................................auaauguccaugguuaaugaa............    1          0         TE2
    ...................................................auaauguccaugguuaaugaaau..........    1          0         TE2
    ....................................................uaauguccaugguuaaugaa............    11         0         TE2
    ....................................................uaauguccaugguuaaugaaau..........    46         0         TE2
    ....................................................uaauguccaugguuaaugaaaua.........    1          0         TE2

    ..........cucauuaaaauggacaacgaua....................................................    3          0         MF2
    ..........cucauuaaaauggacaacgauagu..................................................    1          0         MF2
    ................................gugugucauuauauuugcua................................    2          0         MF2
    ..................................................uauaauguccaugguuaaugaa............    2          0         MF2
    ...................................................auaauguccaugguuaaugaaau..........    2          0         MF2
    ....................................................uaauguccaugguuaaugaa............    1          0         MF2
    ....................................................uaauguccaugguuaaugaaau..........    7          0         MF2
```

miRBase precursor : aga-mir-10371
Total read count : 178
aga-mir-10371 read count : 163
aga-mir-10371-star read count : 6
remaining reads : 9

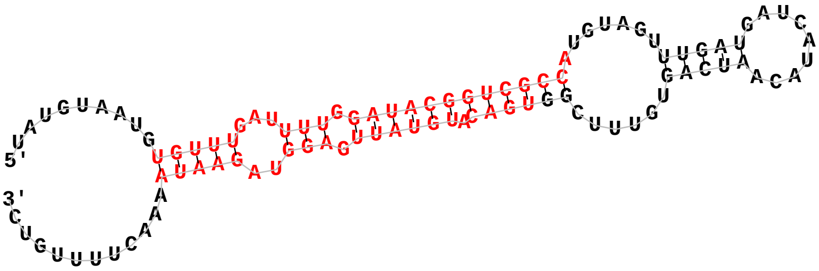

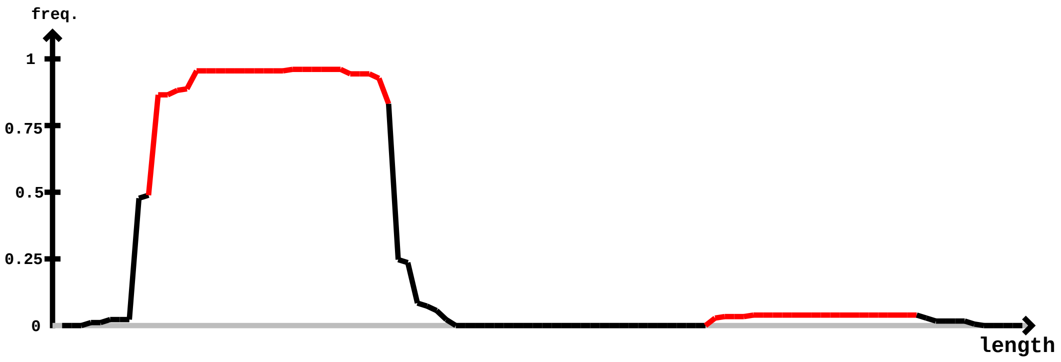


```
                                                                  aga-mir-10371-star
            aga-mir-10371
5'- uauguaaugauguuugauuuuggauacggucgccauguaguuugaugaucauacaaucaguguuucggugacauguauugagguagaauaaaacuuuuguc  -3'   exp
    .........(((((...(((.((((((((((((.......(((((.........)))))......)))))).)))))).)))..))))).........   reads   mm   sample
    ...................................................................ugacauguauCgagguagaauaaaacu......   1       1    FF2

    .......uAuguuugauuuuggauacggucgc....................................................................   3       1    OV2
    .......uAuguuugauuuuggauacggucgcc...................................................................   3       1    OV2
    .......uAuguuugauuuuggauacggucgcca..................................................................   7       1    OV2
    .......uAuguuugauuuuggauacggucgccaug................................................................   1       1    OV2
    ........Auguuugauuuuggauacggucgcca..................................................................   1       1    OV2
    .........uguuugauuuuggauacggucgcca..................................................................   13      0    OV2
    .........uguuugauuuuggauacggucgccaug................................................................   7       0    OV2
    ...........uuugauuuuggauacggucgccaugu...............................................................   1       0    OV2
    .............ugauuuuggauacggucgccaugua..............................................................   1       0    OV2
    .............ugauuuuggauacggucgccauguaA.............................................................   1       1    OV2
    ...................................................................ugacauguauCgagguagaaua...........   1       1    OV2

    ..uguaauAuguuugauuuuggauacggu.......................................................................   2       1    TE1
    ....uaauAuguuugauuuuggauacggucgccau.................................................................   1       1    TE1
    .......uAuguuugauuuuggauacggucgcc...................................................................   1       1    TE1
    .......uAuguuugauuuuggauacggucgcca..................................................................   3       1    TE1
    .......uAuguuugauuuuggauacggucgccaug................................................................   1       1    TE1
    .........uguuugauuuuggauacggucgccaug................................................................   1       0    TE1
    .............ugauuuuggauacggucgccauguaC.............................................................   1       1    TE1
    .............ugauuuuggauacggucgccauguaAu............................................................   1       1    TE1
    .............ugauuuuggauacggucgccauguaCu............................................................   1       1    TE1

    .......uAuguuugauuuuggauacggucgcca..................................................................   1       1    MF2
    .......uAuguuugauuuuggauacggucgccau.................................................................   1       1    MF2
    .......................................................................auguauugagguagaauaaaacu......   1       0    MF2

    .........uguuugauuuuggauacggucgcca..................................................................   1       0    FF1

    .......uAuguuugauuuuggauacggucgcc...................................................................   6       1    OV1
    .......uAuguuugauuuuggauacggucgcca..................................................................   24      1    OV1
    ........Auguuugauuuuggauacggucgcca..................................................................   1       1    OV1
    .........uguuugauuuuggauacggucgcc...................................................................   1       0    OV1
```

```
                                                                   aga-mir-10371-star
         aga-mir-10371
uauguaaugUGUUUGAUUUUGGAUACGGUCGCCAuguaguuugaugaucauacaaucaguguuucggUGACAUGUAUUGAGGUAGAAUAaaacuuuuguc
```

Read	Count	Mismatches	Library
`..............................................................................................`			

```
uauguaauguguuugauuuuggauacggucgccauguaguuugaugaucauacaaucaguguuucggugacauguauugagguagaauaaaacuuuuguc

.........uguuugauuuuggauacggucgcca..................................................................   14   0   OV1
.........uguuugauuuuggauacggucAccaug................................................................   1    1   OV1
.........uguuugauuuuggauacggucgccaug................................................................   7    0   OV1
............uugauuuuggauacggucgccaug................................................................   1    0   OV1
.............ugauuuuggauacggucgccaugua..............................................................   2    0   OV1
.......................uacggucgccauguaAu............................................................   1    1   OV1
...................................................................ugacauguauCgagguagaauaa..........   1    1   OV1

.......uAuguuugauuuuggauacggucgcc...................................................................   5    1   BF2
.......uAuguuugauuuuggauacggucgcca..................................................................   7    1   BF2
.........uguuugauuuuggauacggucgcca..................................................................   7    0   BF2
.........uguuugauuuuggauacggucgccU..................................................................   1    1   BF2
.........uguuugauuuuggauacggucgccaug................................................................   6    0   BF2
.........uguuugauuuuggauacggucgccaugu...............................................................   1    0   BF2
...........uuugauuuuggauacggucgcc...................................................................   1    0   BF2
...................................................................ugacauguauCgagguagaaua...........   1    1   BF2

.......uAuguuugauuuuggauacggucgcca..................................................................   16   1   BF1
.........uguuugauuuuggauacggucgccU..................................................................   1    1   BF1
.........uguuugauuuuggauacggucgcca..................................................................   2    0   BF1
.........uguuugauuuuggauacggucgccaug................................................................   2    0   BF1
...........uuugauuuuggauacggucgcca..................................................................   1    0   BF1
.............ugauuuuggauacggucgccauguaA.............................................................   3    1   BF1
....................................................................gacauguauCgagguagaauaa..........   1    1   BF1

....uaauAuguuugauuuuggauacggu.......................................................................   1    1   TE2
.......uAuguuugauuuuggauacggucgcca..................................................................   2    1   TE2
.........uguuugauuuuggauacggucgcca..................................................................   2    0   TE2
.............ugauuuuggauacggucgccauguaC.............................................................   1    1   TE2
.............ugauuuuggauacggucgccauguaCu............................................................   1    1   TE2
...................................................................ugacauguauCgagguagaauaaaacuu.....   1    1   TE2
```

miRBase precursor : aga-mir-10372a
Total read count : 78
aga-mir-10372a read count : 60
aga-mir-10372a-star read count : 18
remaining reads : 0

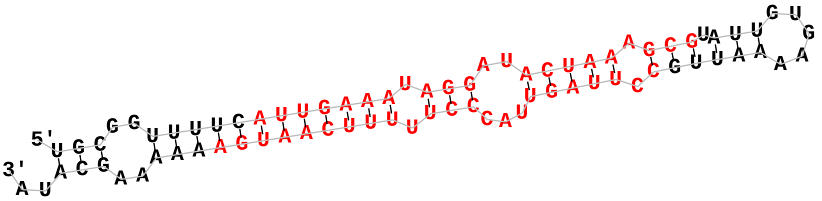

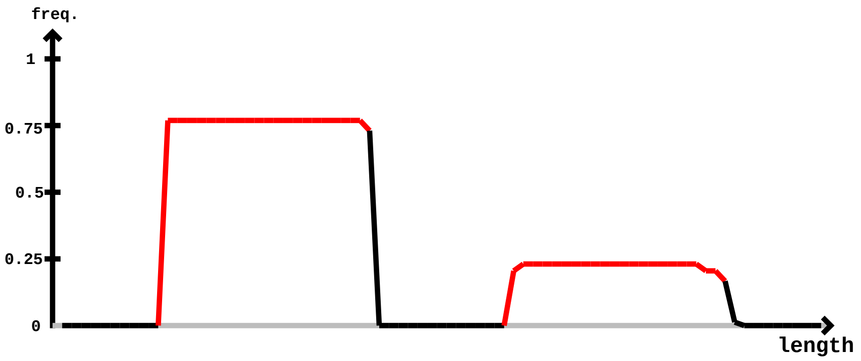


```
                                                                 aga-mir-10372a-star
              aga-mir-10372a
5'- ugcgguuuucauugaaauaggauacuaaagcguauugugaaaauugccuuaguuacccuuuuucaaugaaaaaagcaua   -3'    exp
    (((..((((((((((((.(((..(((((.(((.(((.....)))))).)))))...))).))))))))))))..)))..     reads      mm         sample
    ..........auugaaauaggauacuaaagcg...............................................     9          0          TE1
    ..............................................ccuuaguuacccuuuuucaauga..........     4          0          TE1

    ..........auugaaauaggauacuaaagc................................................     1          0          TE2
    ..........aCugaaauaggauacuaaagcg...............................................     1          1          TE2
    ..........auugaaauaggauacuaaagcg...............................................     31         0          TE2
    ..............................................ccuuaguuacccuuuuucaa.............     2          0          TE2
    ..............................................ccuuaguuacccuuuuucaaug...........     3          0          TE2
    ..............................................ccuuaguuacccuuuuucaauga..........     6          0          TE2
    ...............................................cuuaguuacccuuuuucaauga..........     1          0          TE2
    ...............................................cuuaguuacccuuuuucaaugaU.........     1          1          TE2

    ..........auugaaauaggauacuaaagc................................................     2          0          MF2
    ..........auugaaauaggauacuaaagcg...............................................     16         0          MF2
    ..............................................ccuuaguuacccuuuuucaauga..........     1          0          MF2
```

miRBase precursor : aga-mir-10372b
Total read count : 66
aga-mir-10372b read count : 51
aga-mir-10372b-star read count : 15
remaining reads : 0

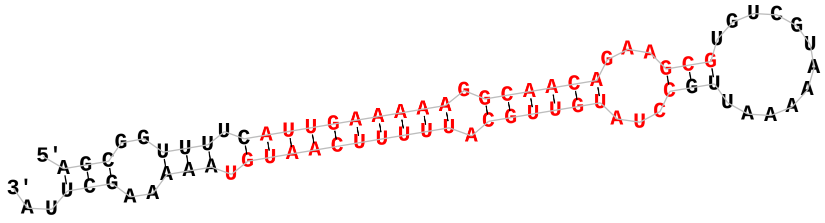

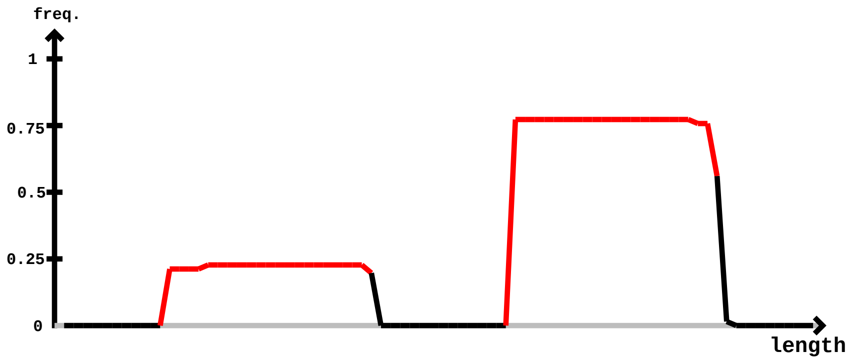


```
                                                     aga-mir-10372b
              aga-mir-10372b-star
5'- agcgguuuucauugaaaaaggcaacagaagcgugucguaaaaauugccuauguugcauuuuucaauguaaaaagcuua   -3'    exp
    (((..(((.((((((((((.((((((...(((............)))...)))))).)))))))))).))).)))..    reads      mm         sample
    ..............................................ccuauguugcauuuuucaaug...........    1          0          MF1

    ..........auugaaaaaggcaacagaagcg..............................................    2          0          TE1
    ..............................................ccuauguugcauuuuucaaug...........    4          0          TE1
    ..............................................ccuauguugcauuuuucaaugu..........    11         0          TE1

    ..........auugaaaaaggcaacagaagcg..............................................    7          0          MF2
    ..............aaaaUggcaacagaagc...............................................    1          1          MF2
    ..............................................ccuauguugcauuuuucaa.............    1          0          MF2
    ..............................................ccuauguugcauuuuucaaug...........    2          0          MF2
    ..............................................ccuauguugcauuuuucaaugu..........    2          0          MF2
    ..............................................ccuauguugcauuuuucaaugua.........    1          0          MF2

    ..........auugaaaaaggcaacagaagc...............................................    1          0          TE2
    ..........auugaaaaaggcaacagaagcg..............................................    4          0          TE2
    ..............................................ccuauguugcauuuuucaaug...........    6          0          TE2
    ..............................................ccuauguugcauuuuucaaugu..........    22         0          TE2

    ..............................................ccuauguugcauuuuucaaugu..........    1          0          FF1
```

miRBase precursor : aga-mir-10373
Total read count : 62
aga-mir-10373 read count : 55
aga-mir-10373-star read count : 4
remaining reads : 3

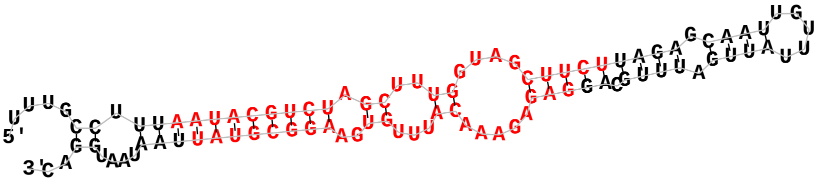

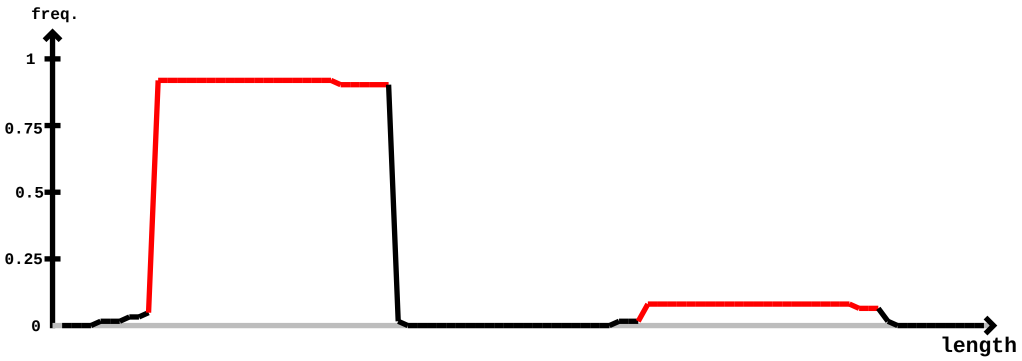


```
                                                        aga-mir-10373-star
           aga-mir-10373
5'- uuugccuuuaauacgucuagcuuugguagcuucuuagagcaauuguuuauugauuugcaggagagaaacauuugugaaggcguauuaauaaauggac   -3'    exp
    ....((.(((((((((((.((..((....((((((((.((((.....)))).)))).))))).....))...))..)))))))))))....))..    reads     mm       sample
    .........aauacgucuagcuuugguagcuucu...............................................................    4         0        TE1

    ...gccuuuaauacgucuagcuuuggua.....................................................................    1         0        OV2
    .........aauacgucuagcuuugguagcuucu...............................................................    16        0        OV2
    ............................................................gagagaaacauuugugaaggcguau............    2         0        OV2

    .........aauacgucuagcuuugguagcuucu...............................................................    9         0        BF2

    ......uuuaauacgucuagcuuugguagcuucu...............................................................    1         0        BF1
    .........aauacgucuagcuuugguagcuucu...............................................................    6         0        BF1
    ............................................................gagagaaacauuugugaaggcguau............    1         0        BF1
    ............................................................gagagaaacauuugugaaggcguauA...........    1         1        BF1

    .........aauacgucuagcuuugguagcuucu...............................................................    8         0        TE2
    .........aauacgucuagcuuugguagcuucuu..............................................................    1         0        TE2
    .........................................................caggagagaaacauuugugaaggcg...............    1         0        TE2

    ........uaauacgucuagcuuugguagcuucu...............................................................    1         0        OV1
    .........aauacgucuagcuuugguagcuucu...............................................................    9         0        OV1
    .........aauacgucuagcuuugguaAcuucu...............................................................    1         1        OV1
```

```
miRBase precursor                : aga-mir-10374
Total read count                 : 153
aga-mir-10374 read count         : 135
aga-mir-10374-star read count    : 14
remaining reads                  : 4
```

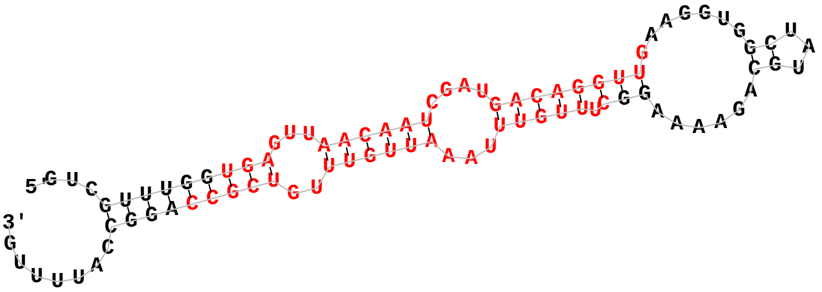

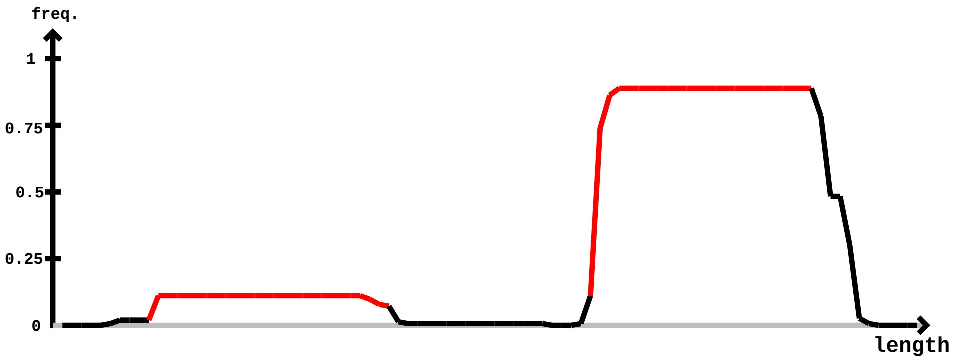


```
                                                              aga-mir-10374
          aga-mir-10374-star
5'- gucguuuggugaguuaacaaucgaugacagguugaagguggcuaugcagaaaaggcuuguuuaaaauuguuugucgccaggccauuuug  -3'   exp
    ((((((..((......))....)))))).((((...((((((...((((...((((...))))...))))..)))))).))))......    reads   mm   sample
    ........................................................uuguuuaaaauuguuugucgccUgg........    1       1    FF2

    .........ugaguuaacaaucgauAacagguug.......................................................    2       1    TE1
    .........ugaguuaacaaucgaugacagguug.......................................................    1       0    TE1
    .....................................................gcuuguuuaaaauuguuugucgcc............    1       0    TE1
    .......................................................cuuguuuaaaauuguuugucgcc...........    1       0    TE1
    .......................................................cuuguuuaaaauuguuugucgccU..........    8       1    TE1
    .......................................................cuuguuuaaaauuguuugucgcca..........    1       0    TE1
    .......................................................cuuguuuaaaauuguuugucgccUgg........    4       1    TE1
    .......................................................cuuguuuaaaauuguuugucgccagg........    1       0    TE1
    .......................................................cuuguuuaaaauuguuugucgccUggc.......    5       1    TE1
    ........................................................uuguuuaaaauuguuugucgccUgg........    1       1    TE1
    ........................................................uuguuuaaaauuguuugucgccUggc.......    4       1    TE1

    ....uuuggAgaguuaacaaucgaugacagguugaagguggcuaugcaga.......................................    1       1    OV2
    .....uuggAgaguuaacaaucgaugacaggu.........................................................    1       1    OV2
    .....................................................gcuuguuuaaaauuguuugucgccU...........    5       1    OV2
    .......................................................cuuguuuaaaauuguuugucgccUggc.......    1       1    OV2
    .......................................................cuuguuuaaaauuguuugucgccaggcc.....    1       0    OV2
    ........................................................uuguuuaaaauuguuugucgccUgg........    1       1    OV2
    ........................................................uuguuuaaaauuguuugucgccUggc.......    2       1    OV2
    ........................................................uuguuuaaaauuguuugucgccaggcc.....    1       0    OV2

    .........Agaguuaacaaucgaugacagguug.......................................................    1       1    MF2
    .......................................................cuuguuuaaaauuguuugucgcc...........    2       0    MF2
    .......................................................cuuguuuaaaauuguuugucgccU..........    4       1    MF2
    .......................................................cuuguuuaaaauuguuugucgccUggc.......    2       1    MF2
    .........................................................uguuuaaaauuguuugucgccUggcc......    1       1    MF2

    .........ugaguuaacaaucgaugacaggu.........................................................    1       0    OV1
    .........ugaguuaacaaucgaugacagguug.......................................................    1       0    OV1
    .........ugaguuaacaaucgauAacagguuga......................................................    1       1    OV1
    ....................................................ggcuuguuuaaaauuguuugucgcc............    1       0    OV1
    .....................................................gcuuguuuaaaauuguuugucgcc............    2       0    OV1
```

```
                                                       aga-mir-10374
         aga-mir-10374-star
gucguuuggugaguuaacaaucgaugacagguugaagguggcuaugcagaaaaggcuuuguuuaaauuguuugucgccaggccauuuug

......................................................gcuuuguuuaaauuguuugucgccU..........   2   1   OV1
......................................................gcuuuguuuaaauuguuugucgccUgg........   1   1   OV1
.......................................................cuuuguuuaaauuguuugucgccU..........   2   1   OV1
.......................................................cuuuguuuaaauuguuugucgccUgg........   2   1   OV1
........................................................uuuguuuaaauuguuugucgccUgg........   1   1   OV1
........................................................uuuguuuaaauuguuugucgccUggc.......   3   1   OV1
.........................................................uuguuuaaauuguuugucgccUggcca.....   1   1   OV1

......................................................gcuuuguuuaaauuguuugucgccU..........   1   1   BF2
......................................................gcuuuguuuaaauuguuugucgccUggc.......   1   1   BF2
........................................................uuuguuuaaauuguuugucgccUgg........   2   1   BF2
.........................................................uuguuuaaauuguuugucgccUggc.......   1   1   BF2

.....uuggugaguuaacaaucgauAacaggu.........................................................   1   1   BF1
.......................................................cuuuguuuaaauuguuugucgcc...........   1   0   BF1
.......................................................cuuuguuuaaauuguuugucgccU..........   1   1   BF1
.......................................................cuuuguuuaaauuguuugucgccUgg........   2   1   BF1
.......................................................cuuuguuuaaauuguuugucgccUggc.......   1   1   BF1
........................................................uuuguuuaaauuguuugucgccUgg........   1   1   BF1

.........Agaguuaacaaucgaugacagg..........................................................   2   1   TE2
.........Agaguuaacaaucgaugacagguu........................................................   1   1   TE2
.........ugaguuaacaaucgaugacagguuA.......................................................   1   1   TE2
.........ugaguuaacaaucgaugacagguug.......................................................   2   0   TE2
.........ugaguuaacaaucgaugGcagguug.......................................................   1   1   TE2
......................................................gcuuuguuuaaauuguuugucgcc...........   1   0   TE2
......................................................gcuuuguuuaaauuguuugucgccU..........   1   1   TE2
......................................................gcuuuguuuaaauuguuugucgccUgg........   1   1   TE2
.......................................................cuuuguuuaaauuguuugucgcc...........   7   0   TE2
.......................................................cuuuguuuaaauuguuugucgccU..........   21  1   TE2
.......................................................cuuuguuuaaauuguuugucgccUgg........   9   1   TE2
.......................................................cuuuguuuaaauuguuugucgccagg........   1   0   TE2
.......................................................cuuuguuuaaauuguuugucgccaggc.......   3   0   TE2
.......................................................cuuuguuuaaauuguuugucgccUggc.......   16  1   TE2
........................................................uuuguuuaaauuguuugucgccUggc.......   2   1   TE2
.........................................................uuguuuaaauuguuugucgccUggc.......   1   1   TE2
```

miRBase precursor : aga-mir-10375a
Total read count : 154
aga-mir-10375a read count : 153
aga-mir-10375a-star read count : 0
remaining reads : 1

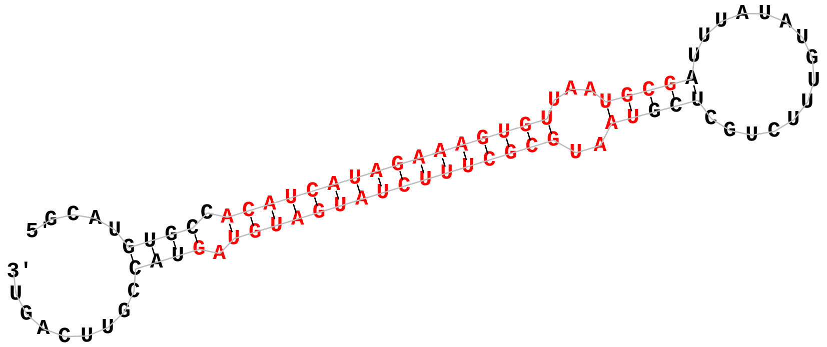

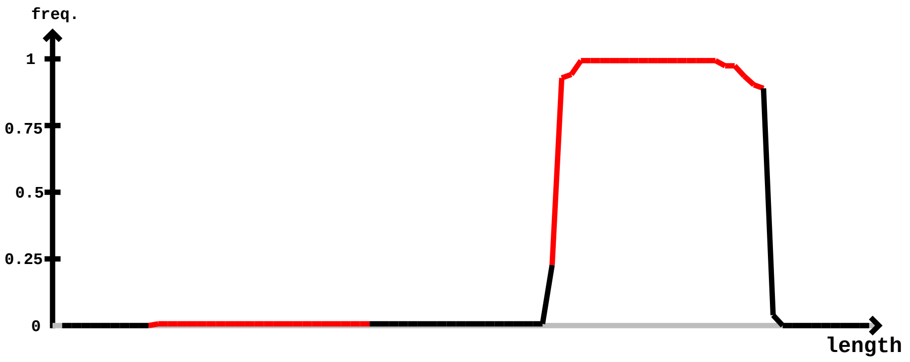


```
aga-mir-10375a-star
                                   aga-mir-10375a
5'- gcaugugccacaucauagaaaguguuaaugcgauuuauauguuucugcucguaaugcgcuuucuaugauguaguaccguucagu  -3'   exp
    ....((((.((((((((((((((((...(((((..............)))))..)))))))))))))))).)))).........   reads   mm   sample
    ...................................................uaaugcgcuuucuaugauguag...........   10      0    OV2
    ...................................................uaaugcgcuuucuaugauguagA..........   1       1    OV2

    .........acaucauagaaaguguuaaugcgauuuauauguuucugcucg.................................   1       0    TE1
    ..................................................guaaugcgcuuucuaugaug..............   1       0    TE1
    ..................................................guaaugcgcuuucuaugaugu.............   1       0    TE1
    ..................................................guaaugcgcuuucuaugaugua............   1       0    TE1
    ..................................................guaaugcgcuuucuaugauguag...........   6       0    TE1
    ..................................................guaaugcgcuuucuaugauguagA..........   1       1    TE1
    ...................................................uaaugcgcuuucuaugauguag...........   18      0    TE1
    ....................................................aaugcgcuuucuaugauguag...........   1       0    TE1
    .....................................................aAgcgcuuucuaugauguag...........   1       1    TE1
    .....................................................augcgcuuucuaugauguag...........   3       0    TE1

    ..................................................guaaugcgcuuucuaugauguag...........   1       0    MF2
    ...................................................uaaugcgcuuucuaugauguag...........   3       0    MF2
    ...................................................uaaugcgcuuucuaugauguagA..........   1       1    MF2

    ...................................................uaaugcgcuuucuaugauguag...........   1       0    FW2

    ...................................................uaaAgcgcuuucuauga................   2       1    OV1
    ...................................................uaaugcgcuuucuaugaugua............   1       0    OV1
    ...................................................uaaugcgcuuucuaugauguag...........   6       0    OV1
    ...................................................uaaugcgcuuucuaugauguGg...........   1       1    OV1

    ..................................................guaaugcgcuuucuaugauguag...........   2       0    MF1
    ...................................................uaaugcgcuuucuaugaugu.............   1       0    MF1
    ...................................................uaaAgcgcuuucuaugauguag...........   1       1    MF1

    ..................................................guaaugcgcuuucuaugauguag...........   2       0    BF2
    ...................................................uaaugcgcuuucuaugauguag...........   8       0    BF2
    ...................................................uaaugcgcuuucuaugauguagA..........   1       1    BF2

    ..................................................guaaugcgcuuucuaugauguag...........   1       0    BF1
```

```
         aga-mir-10375a-star
                                                   aga-mir-10375a
gcaugugccacaucauagaaaguguuaaugcgauuuauauguuucugcucguaaugcgcuuucuaugauguaguaccguucagu

..................................................guaaugcgcuuucuaugaug..............    1    0    TE2
..................................................guaauCcgcuuucuaugaug..............    1    1    TE2
..................................................guaaugcgcuuucuaugauguag...........    15   0    TE2
..................................................guaaugcgcuuucuaugauguagA..........    1    1    TE2
...................................................uaaugcgcuuucuauga................    1    0    TE2
...................................................uaaugcgcuuucuaugaug..............    3    0    TE2
...................................................uaaAgcgcuuucuaugaugu.............    1    1    TE2
...................................................uaaugcgcuuucuaugaugu.............    2    0    TE2
...................................................uaaugcgcuuucuaugauguag...........    43   0    TE2
...................................................uaaugUgcuuucuaugauguag...........    1    1    TE2
...................................................uaaAgcgcuuucuaugauguag...........    2    1    TE2
...................................................uaaugcgcuuucuaugauguagA..........    1    1    TE2
....................................................aaugcgcuuucuaugauguag...........    1    0    TE2
.....................................................augcgcuuucuaugauguag...........    2    0    TE2
.....................................................aAgcgcuuucuaugauguag...........    2    1    TE2
```

miRBase precursor : aga-mir-10375b-1
Total read count : 2094
aga-mir-10375b-1 read count : 926
aga-mir-10375b-1-star read count : 42
aga-mir-10375b-2 read count : 926
aga-mir-10375b-2-star read count : 42
remaining reads : 158

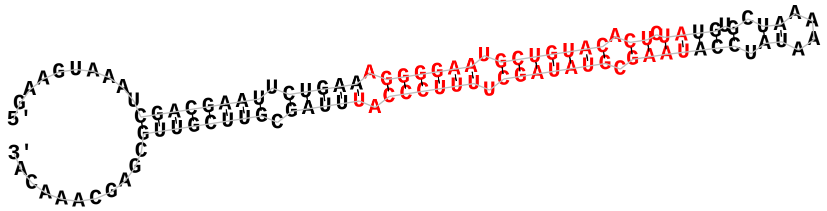

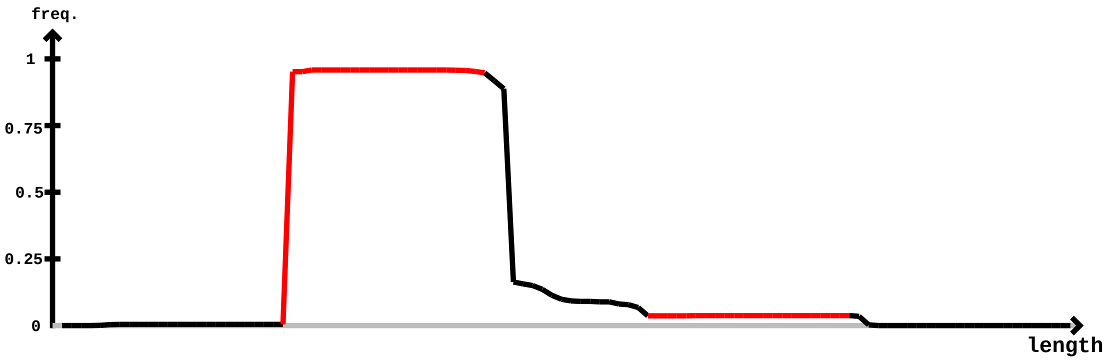


```
                                                           aga-mir-10375b-2-star
                       aga-mir-10375b-2
                                                           aga-mir-10375b-1-star
                       aga-mir-10375b-1
5'- gaaguaaaucgacgaauucugaaaggggaaugcuguacacucuaugugcuaaaaaauauccauaagcguauagcuuuucccauuuagcguucguugcgagcaaaca  -3'   exp
    ...((....((((((((.(((((.((((((.(((((((.((.((((.(.((....)).))))))).))))))).)))))).))))).))))))))...)).....  reads    mm    sample
    .......................aggggaaugcuguacacucua..............................................................  1        0     TE1
    .......................aggggaaugcuguacacucuaGg............................................................  1        1     TE1
    .......................aggggaaugcuguacacucuaug............................................................  13       0     TE1
    .......................aggggaaugcuguacacucuaugA...........................................................  1        1     TE1
    .......................aggggaaugcuguacacucuaugug..........................................................  1        0     TE1
    .......................aggggaaugcuguacacucuaugugc.........................................................  1        0     TE1
    .......................aggggaaugcuguacacucuaugugcu........................................................  1        0     TE1
    .......................aggggaaugcuguacacucuaugugcua.......................................................  1        0     TE1
    .......................aggggaaugcuguacacucuaugugcuaa......................................................  1        0     TE1
    .......................aggggaaugcuguacacucuaugugcuaaa.....................................................  1        0     TE1
    .......................aggggaaugcuguacacucuaugugcuaaaaa...................................................  1        0     TE1
    .......................aggggaaugcuguacacucuaugugcuaaaaaaua.................................................  3        0     TE1
    .......................aggggaaugcuguacacucuaugugcuaaaaaauauc...............................................  1        0     TE1
    .......................aggggaaugcuguacacucuaugugcuaaaaaauaucc..............................................  3        0     TE1
    .......................aggggaaugcuguacacucuaugugcuaaaaaauaucU..............................................  1        1     TE1
    .......................aggggaaugcuguacacucuaugugcuaaaaaauaucca............................................  2        0     TE1
    ..............................................................uaagcguauagcuuuucccau.......................  1        0     TE1

    .......................aggggaaugcuguacacucu...............................................................  1        0     FF2
    .......................aggggaaugcuguacacucua..............................................................  1        0     FF2
    .......................aggggaaugcuguacacucuau.............................................................  2        0     FF2
    .......................Ggggaaugcuguacacucuaug............................................................  1        1     FF2
    .......................aggggaaugcuguacacucuaug............................................................  18       0     FF2
    .......................aggggaaugcuguacacucuaugug..........................................................  1        0     FF2
    .......................aggggaaugcuguacacucuaugugcuaaaaaauaucc..............................................  2        0     FF2

    .......................aggggaaugcuguacacuc................................................................  1        0     OV2
    .......................aggggaaugcuguacacucua..............................................................  1        0     OV2
    .......................aggggaaugcuguacacucuau.............................................................  2        0     OV2
    .......................aggggaaugcuguacacucuaug............................................................  19       0     OV2
    .......................aCgggaaugcuguacacucuaugugc.........................................................  2        1     OV2
    .......................aggggaaugcuguacacucuaugugc.........................................................  4        0     OV2
```

```
                                                             aga-mir-10375b-2-star
                       aga-mir-10375b-2
gaaguaaaucgacgaauucugaaaggggaaugcuguacacucuaugugcuaaaaauauccauaagcguauagcuuuucccauuuuagcguucguugcgagcaaaca
                                                             aga-mir-10375b-1-star
                       aga-mir-10375b-1

.......................aggggaaugcuguacacucuaugugcu........................................................   2   0   OV2
.......................aggggaaugcuguacacucuauAugcuaa......................................................   1   1   OV2
.......................aggggaaugcuguacacucuaugugcuaaaaa...................................................   1   0   OV2
.......................aggggaaugcuguacacucuaugugcuaaaaaua.................................................   1   0   OV2
.......................aggggaaugcuguacacucuaugugcuaaaaauauc...............................................   1   0   OV2
.......................aggggaaugcuguacacucuaugugcuaaaaauaucc..............................................   2   0   OV2
.......................aggggaaugcuguacacucuaugugcuaaaaauaucca.............................................   1   0   OV2
.............................................................uaagcguauagcuuuucccauu.......................   3   0   OV2

....uaaaucgacgaauucugaa...................................................................................   2   0   MF2
.....aaaucgacgaauucugaa...................................................................................   1   0   MF2
.......................aggggaaugcuguacacucua..............................................................   5   0   MF2
.......................aggggaaugcuguacacucuau.............................................................   4   0   MF2
.......................aggggaaugAuguacacucuaug............................................................   1   1   MF2
.......................aggggaaugcuguacacucuauA............................................................   2   1   MF2
.......................aggggaaugcuguacGcucuaug............................................................   1   1   MF2
.......................aggggaaugcuguacacucuaug............................................................   203 0   MF2
.......................aggggaaugcuguacacucuaugA...........................................................   1   1   MF2
.......................aggggaaugcuguacacucuauUu...........................................................   1   1   MF2
.......................aggggaaugcuguacacucuaugug..........................................................   2   0   MF2
.......................aggggaaugcuguacacucuaugugc.........................................................   1   0   MF2
.......................aggggaaugcuguacacucuaugugcu........................................................   3   0   MF2
.......................aggggaaugcuguacacucuaugugcua.......................................................   3   0   MF2
.......................aggggaaugcuguacacucuaugugcuaU......................................................   1   1   MF2
.......................aggggaaugcuguacacucuaugugcuaaaaaua.................................................   2   0   MF2
.......................aggggaaugcuguacacucuaugugcuaaaaauauc...............................................   2   0   MF2
.......................aggggaaugcuguacacucuaugugcuaaaaauaucc..............................................   6   0   MF2
.......................aggggaaugcuguacacucuaugugcuaaaaauauAc..............................................   1   1   MF2
.......................aggggaaugcuguacacucuaugugcuaaaaauaucca.............................................   7   0   MF2
.......................aggggaaugcuguacacucuaugugcuaaaaauauccaua...........................................   1   0   MF2
.............................................................uaagcguauagcuuuucccauu.......................   8   0   MF2

.......................aggggaaugcuguacacucua..............................................................   2   0   FW2
.......................aggUgaaugcuguacacucua..............................................................   1   1   FW2
.......................aggggaaugcuguacacucuau.............................................................   1   0   FW2
.......................aggggaaugcuguacacucuaug............................................................   57  0   FW2
.......................aggggaaugcuguacacucuaugu...........................................................   1   0   FW2
.......................aggggaaugcuguacacucuaugugcuaaaaauaucc..............................................   1   0   FW2
.......................aggggaaugcuguacacucuaugugcuaaaaauaucca.............................................   2   0   FW2
.........................gggaaugcuguacacucuaug............................................................   1   0   FW2
.............................................................uaagcguauagcuuuucccauu.......................   2   0   FW2
.............................................................uaagcguGuagcuuuucccauu.......................   1   1   FW2

.......................aggggaaugcuguacacucua..............................................................   3   0   FF1
.......................aggggaaugcuguacacucuau.............................................................   5   0   FF1
.......................aggggaaugcuguacacAcuaug............................................................   1   1   FF1
.......................aggUgaaugcuguacacucuaug............................................................   7   1   FF1
.......................aggggaaugcuguacaUucuaug............................................................   1   1   FF1
.......................aggggaaugcuguacacucuaug............................................................   104 0   FF1
.......................aggggaaugcuguacacucuauA............................................................   1   1   FF1
.......................aggggaaugcuguacacucuaugugcu........................................................   1   0   FF1
.......................aggggaaugcuguacacucuaugugcua.......................................................   1   0   FF1
.......................aggggaaugcuguacacucuaugugcuaa......................................................   1   0   FF1
.......................aggggaaugcuguacacucuaugugcuaaaaauauA...............................................   1   1   FF1
.......................aggggaaugcuguacacucuaugugcuaaaaauaucca.............................................   1   0   FF1
.............................................................uaagcguauagcuuuucccauu.......................   7   0   FF1

.......................aggggaaugcuguacac..................................................................   1   0   OV1
.......................Gggggaaugcuguacacuc................................................................   1   1   OV1
.......................aggggaaugcuguacacucu...............................................................   1   0   OV1
.......................aggggaaugcuguacacucuau.............................................................   2   0   OV1
.......................aggggaaugcuguacacucuauA............................................................   1   1   OV1
.......................aggggaaugcuguacacucuaug............................................................   28  0   OV1
.......................aggggaaugcuguacacucuaugugc.........................................................   3   0   OV1
.......................aggggaaugcuguacacucuaCgugc.........................................................   1   1   OV1
.......................aggggaaugcuguacacucuaugugcu........................................................   3   0   OV1
.......................aggggaaugcuguacacucuaugugcua.......................................................   5   0   OV1
```

```
                                                             aga-mir-10375b-2-star
                       aga-mir-10375b-2
gaaguaaaucgacgaauucugaaaggggaaugcuguacacucuaugugcuaaaaauauccauaagcguauagcuuuucccauuuuagcguucguugcgagcaaaca
                       aga-mir-10375b-1
```

The overlapping red label printed over the star region of the reference line reads "aga-mir-10375b-1-star".

```
.......................aggggaaugcuguacacucuaugugcuaa......................................................	1	0	OV1
.......................aggggaaugcuguacacucuaugugcuaaaaauauc...............................................	1	0	OV1
.......................aggggaaugcuguacacucuaugugcuaaaaauaucc..............................................	2	0	OV1
.......................aggggaaugcuguacacucuaugugcuaaaaauaucca.............................................	3	0	OV1
.............................................................uaagcguauagcuuuucccauu.......................	2	0	OV1

.......................aggggaaugcuguacacucu...............................................................	1	0	MF1
.......................aggggaaugcuguacacucua..............................................................	1	0	MF1
.......................aggggaaugcuguacacucuau.............................................................	2	0	MF1
.......................aggggaaugcuguacacucuaug............................................................	25	0	MF1
.......................aggggaaugcuguacacucuauC............................................................	1	1	MF1
.......................aggggaaugcuguacacucuaugA...........................................................	1	1	MF1
.......................aggggaaugcuguacacucuaugugcuaaaaauau................................................	1	0	MF1
.......................aggggaaugcuguacacucuaugugcuaaaaauaucca.............................................	1	0	MF1
.........................gggaaugcuguacacucuaug............................................................	1	0	MF1
.............................................................uaagcguauagcuuuucccauu.......................	3	0	MF1
.............................................................uaagcguauagcuuuucccauuu......................	1	0	MF1

.......................aggggaaugcuguacacucu...............................................................	1	0	BF2
.......................aggggaaugcuguacacucua..............................................................	4	0	BF2
.......................aggggaaugcuguacacucuau.............................................................	5	0	BF2
.......................aggggaaugGuguacacucuaug............................................................	1	1	BF2
.......................aggggaGugcuguacacucuaug............................................................	1	1	BF2
.......................aggggaaugAuguacacucuaug............................................................	1	1	BF2
.......................aggggaaugcuguacacucuaug............................................................	123	0	BF2
.......................aggggaaugcuguacacucuaugC...........................................................	2	1	BF2
.......................aggggaaugcuguacacucuaugugc.........................................................	1	0	BF2
.......................aggggaaugcuguacacucuaugugcu........................................................	4	0	BF2
.......................aggggaaugcuguacacucuaugugcua.......................................................	1	0	BF2
.......................aggggaaugcuguacacucuaugugcuaaaaaua.................................................	2	0	BF2
.......................aggggaaugcuguacacucuaugugcuaaaaauau................................................	1	0	BF2
.......................aggggaaugcuguacacucuaugugcuaaaaauauc...............................................	1	0	BF2
.......................aggggaaugcuguacacucuaugugcuaaaaauaucc..............................................	4	0	BF2
.......................aggggaaugcuguacacucuaugugcuaaaaauaucca.............................................	15	0	BF2
.............................................................uaagcguauagcuuuucccauu.......................	1	0	BF2
.............................................................uaagcguauUgcuuuucccauu.......................	1	1	BF2
...............................................................agcguauagcuuuucccauu.......................	1	0	BF2

....uaaaucgacgaauucugaa...................................................................................	1	0	BF1
.......................aggggaaugcuguacacucua..............................................................	2	0	BF1
.......................aggggaaugcuguacacucuaug............................................................	15	0	BF1
.......................aggggaaugcuguacacucuaugug..........................................................	2	0	BF1
.......................aggggaaugcuguacacucuaugugc.........................................................	2	0	BF1
.......................aggggaaugcuguacacucuaugugcu........................................................	4	0	BF1
.......................agggUaaugcuguacacucuaugugcua.......................................................	1	1	BF1
.......................aggggaaugcuguacacucuaugugcua.......................................................	2	0	BF1
.......................aggggaaugcuguacacucuaugugcuaaaaauau................................................	1	0	BF1
.......................aggggaaugcuguacacucuaugugcuaaaaauauc...............................................	3	0	BF1
.......................aggggaaugcuguacacucuaugugcuaaaaauaucc..............................................	11	0	BF1
.......................aggggaaugcuguacacucuaugugcuaaaaauaucca.............................................	8	0	BF1
.............................................................uaagcguauagcuuuucccauu.......................	1	0	BF1

...guaaaucgacgaauucugaa...................................................................................	1	0	MW1
.......................aggggaaugcuguacacucu...............................................................	1	0	MW1
.......................aggggaaugcuguacacucua..............................................................	3	0	MW1
.......................aggggaaugcuguacacucuau.............................................................	4	0	MW1
.......................aggggaaugcuguacacucuauU............................................................	1	1	MW1
.......................aggggaauAcuguacacucuaug............................................................	1	1	MW1
.......................aggggaaugcuguacacucuaug............................................................	75	0	MW1
.......................aggggaaugcuguacacucuaugugcu........................................................	1	0	MW1
.......................aggggaaugcuguacacucuaugugcuaaaaauauc...............................................	1	0	MW1
.......................aggggaaugcuguacacucuaugugcuaaaaauaucU..............................................	1	1	MW1
.........................gggaaugcuguacacucuaug............................................................	2	0	MW1
.............................................................uaagcguauagcuuuucccau........................	2	0	MW1
.............................................................uaagcguauagcuuuucccauu.......................	1	0	MW1
.................................................................cguauagcuuuucccauu.......................	1	0	MW1

.......................aggggaaugcuguacacucu...............................................................	1	0	FW1
```

```
                                                             aga-mir-10375b-2-star
                       aga-mir-10375b-2
gaaguaaaucgacgaauucugaaaggggaaugcuguacacucuaugugcuaaaaauauccauaagcguauagcuuuucccauuuagcguucguugcgagcaaaca
                       aga-mir-10375b-1                      aga-mir-10375b-1-star

.......................aggggaaugcuguacacucua.............................................................    1    0    FW1
.......................aggggaaugcuguacacucuau............................................................    2    0    FW1
.......................aggggaaugcuguacacucuaug...........................................................    30   0    FW1
.......................aggggaaCgcuguacacucuaug...........................................................    1    1    FW1
.......................aggggaaugcuguacacucuaugugcua......................................................    1    0    FW1
.........................gggaaugcuguacacucuaug...........................................................    1    0    FW1
.............................................................uaagcguauagcuuuucccauu......................    3    0    FW1
.............................................................uaagcguauagcuuuucccauuu.....................    1    0    FW1

.......................aggggaaugcuguacacucua.............................................................    1    0    MW2
.......................aggggaaugcuguacacucuau............................................................    1    0    MW2
.......................aggggaaugcuguacacucuaug...........................................................    22   0    MW2
.........................gggaaugcuguacacucua.............................................................    1    0    MW2
.........................gggaaugcuguacacucuaug...........................................................    1    0    MW2

.......................aggggaaugcuguacacu................................................................    1    0    TE2
.......................aggggaaugcuguacacuc...............................................................    2    0    TE2
.......................aggggaaugcuguacacucua.............................................................    6    0    TE2
.......................aggggaaugcuguacacucuau............................................................    4    0    TE2
.......................aggggaaugcuguacacucuaug...........................................................    54   0    TE2
.......................aggggaaugcuguacacucuaugu..........................................................    1    0    TE2
.......................aggggaaugcuguacacucuaugug.........................................................    1    0    TE2
.......................aggggaaugcuguacacucuaugugc........................................................    1    0    TE2
.......................aggggaaugcuguacacucuaugugcA.......................................................    1    1    TE2
.......................aggggaaugcuguacacucuaugugcu.......................................................    5    0    TE2
.......................aggggaaugcuguacacucuaugugcua......................................................    2    0    TE2
.......................aggggaaugcuguacacucuaugugcuaa.....................................................    2    0    TE2
.......................aggggaaugcuguacacucuaugugcuaaa....................................................    1    0    TE2
.......................aggggaaugcuguacacucuaugugcuaaaaaua................................................    1    0    TE2
.......................aggggaaugcuguacacucuaugugcuaaaaauauc..............................................    1    0    TE2
.......................aggggaaugcuguacacucuaugugcuaaaaauaucc.............................................    1    0    TE2
.............................................................uaagcguauagcuuuucccau.......................    1    0    TE2
.............................................................uaagcguauagcuuuucccauuu.....................    1    0    TE2
```

miRBase precursor : aga-mir-10375b-2
Total read count : 2094
aga-mir-10375b-1 read count : 926
aga-mir-10375b-1-star read count : 42
aga-mir-10375b-2 read count : 926
aga-mir-10375b-2-star read count : 42
remaining reads : 158

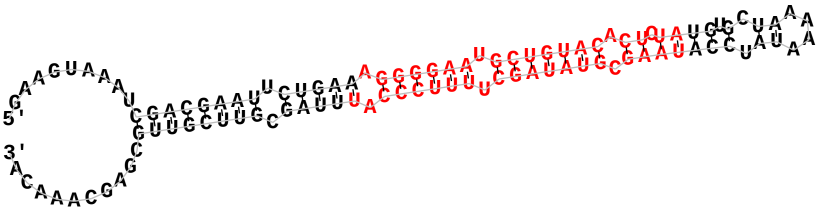

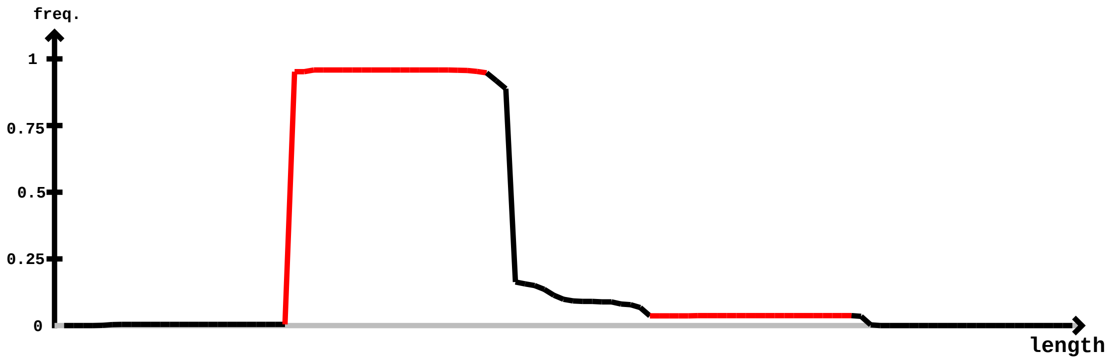


```
                                                            aga-mir-10375b-2-star
                        aga-mir-10375b-2
                                                            aga-mir-10375b-1-star
                        aga-mir-10375b-1
5'- gaaguaaaucgacgaauucugaaaggggaaugcuguacacucuaugugcuaaaaauauccauaagcguauagcuuuucccauuuagcguucguugcgagcaaaca   -3'    exp
    ...((....((((((((.(((((.((((((.(((((((.((.((((.(.((....)).))))))).))))))).)))))).))))).))))))))...)).....   reads   mm   sample
    .......................aggggaaugcuguacacucua.............................................................   1       0    TE1
    .......................aggggaaugcuguacacucuaGg...........................................................   1       1    TE1
    .......................aggggaaugcuguacacucuaug...........................................................   13      0    TE1
    .......................aggggaaugcuguacacucuaugA..........................................................   1       1    TE1
    .......................aggggaaugcuguacacucuaugug.........................................................   1       0    TE1
    .......................aggggaaugcuguacacucuaugugc........................................................   1       0    TE1
    .......................aggggaaugcuguacacucuaugugcu.......................................................   1       0    TE1
    .......................aggggaaugcuguacacucuaugugcua......................................................   1       0    TE1
    .......................aggggaaugcuguacacucuaugugcuaa.....................................................   1       0    TE1
    .......................aggggaaugcuguacacucuaugugcuaaa....................................................   1       0    TE1
    .......................aggggaaugcuguacacucuaugugcuaaaaa..................................................   1       0    TE1
    .......................aggggaaugcuguacacucuaugugcuaaaaaua................................................   3       0    TE1
    .......................aggggaaugcuguacacucuaugugcuaaaaauauc..............................................   1       0    TE1
    .......................aggggaaugcuguacacucuaugugcuaaaaauaucc.............................................   3       0    TE1
    .......................aggggaaugcuguacacucuaugugcuaaaaauaucU.............................................   1       1    TE1
    .......................aggggaaugcuguacacucuaugugcuaaaaauaucca............................................   2       0    TE1
    .............................................................uaagcguauagcuuuucccau.......................   1       0    TE1

    .......................aggggaaugcuguacacucu..............................................................   1       0    FF2
    .......................aggggaaugcuguacacucua.............................................................   1       0    FF2
    .......................aggggaaugcuguacacucuau............................................................   2       0    FF2
    .......................Ggggaaugcuguacacucuaug............................................................   1       1    FF2
    .......................aggggaaugcuguacacucuaug...........................................................   18      0    FF2
    .......................aggggaaugcuguacacucuaugug.........................................................   1       0    FF2
    .......................aggggaaugcuguacacucuaugugcuaaaaauaucc.............................................   2       0    FF2

    .......................aggggaaugcuguacacuc...............................................................   1       0    OV2
    .......................aggggaaugcuguacacucua.............................................................   1       0    OV2
    .......................aggggaaugcuguacacucuau............................................................   2       0    OV2
    .......................aggggaaugcuguacacucuaug...........................................................   19      0    OV2
    .......................aCgggaaugcuguacacucuaugugc........................................................   2       1    OV2
    .......................aggggaaugcuguacacucuaugugc........................................................   4       0    OV2
```

```
                                                             aga-mir-10375b-2-star
                       aga-mir-10375b-2
gaaguaaaucgacgaauucugaaaggggaaugcuguacacucuaugugcuaaaaaauaucc aga-mir-10375b-1-star uuagcguucguugcgagcaaaca
                       aga-mir-10375b-1


.......................aggggaaugcuguacacucuaugugcu.......................................................    2    0    OV2
.......................aggggaaugcuguacacucuauAugcuaa.....................................................    1    1    OV2
.......................aggggaaugcuguacacucuaugugcuaaaaa..................................................    1    0    OV2
.......................aggggaaugcuguacacucuaugugcuaaaaaaua...............................................    1    0    OV2
.......................aggggaaugcuguacacucuaugugcuaaaaaauauc.............................................    1    0    OV2
.......................aggggaaugcuguacacucuaugugcuaaaaaauaucc............................................    2    0    OV2
.......................aggggaaugcuguacacucuaugugcuaaaaaauaucca...........................................    1    0    OV2
.............................................................uaagcguauagcuuuucccauu......................    3    0    OV2

....uaaaucgacgaauucugaa..................................................................................    2    0    MF2
.....aaaucgacgaauucugaa..................................................................................    1    0    MF2
.......................aggggaaugcuguacacucua.............................................................    5    0    MF2
.......................aggggaaugcuguacacucuau............................................................    4    0    MF2
.......................aggggaaugAuguacacucuaug...........................................................    1    1    MF2
.......................aggggaaugcuguacacucuauA...........................................................    2    1    MF2
.......................aggggaaugcuguacGcucuaug...........................................................    1    1    MF2
.......................aggggaaugcuguacacucuaug...........................................................    203  0    MF2
.......................aggggaaugcuguacacucuaugA..........................................................    1    1    MF2
.......................aggggaaugcuguacacucuauUu..........................................................    1    1    MF2
.......................aggggaaugcuguacacucuaugug.........................................................    2    0    MF2
.......................aggggaaugcuguacacucuaugugc........................................................    1    0    MF2
.......................aggggaaugcuguacacucuaugugcu.......................................................    3    0    MF2
.......................aggggaaugcuguacacucuaugugcua......................................................    3    0    MF2
.......................aggggaaugcuguacacucuaugugcuaU.....................................................    1    1    MF2
.......................aggggaaugcuguacacucuaugugcuaaaaaaua...............................................    2    0    MF2
.......................aggggaaugcuguacacucuaugugcuaaaaaauauc.............................................    2    0    MF2
.......................aggggaaugcuguacacucuaugugcuaaaaaauaucc............................................    6    0    MF2
.......................aggggaaugcuguacacucuaugugcuaaaaaauauAc............................................    1    1    MF2
.......................aggggaaugcuguacacucuaugugcuaaaaaauaucca...........................................    7    0    MF2
.......................aggggaaugcuguacacucuaugugcuaaaaaauauccaua.........................................    1    0    MF2
.............................................................uaagcguauagcuuuucccauu......................    8    0    MF2

.......................aggggaaugcuguacacucua.............................................................    2    0    FW2
.......................aggUgaaugcuguacacucua.............................................................    1    1    FW2
.......................aggggaaugcuguacacucuau............................................................    1    0    FW2
.......................aggggaaugcuguacacucuaug...........................................................    57   0    FW2
.......................aggggaaugcuguacacucuaugu..........................................................    1    0    FW2
.......................aggggaaugcuguacacucuaugugcuaaaaaauaucc............................................    1    0    FW2
.......................aggggaaugcuguacacucuaugugcuaaaaaauaucca...........................................    2    0    FW2
.........................gggaaugcuguacacucuaug...........................................................    1    0    FW2
.............................................................uaagcguauagcuuuucccauu......................    2    0    FW2
.............................................................uaagcguGuagcuuuucccauu......................    1    1    FW2

.......................aggggaaugcuguacacucua.............................................................    3    0    FF1
.......................aggggaaugcuguacacucuau............................................................    5    0    FF1
.......................aggggaaugcuguacacAcuaug...........................................................    1    1    FF1
.......................aggUgaaugcuguacacucuaug...........................................................    7    1    FF1
.......................aggggaaugcuguacaUucuaug...........................................................    1    1    FF1
.......................aggggaaugcuguacacucuaug...........................................................    104  0    FF1
.......................aggggaaugcuguacacucuauA...........................................................    1    1    FF1
.......................aggggaaugcuguacacucuaugugcu.......................................................    1    0    FF1
.......................aggggaaugcuguacacucuaugugcua......................................................    1    0    FF1
.......................aggggaaugcuguacacucuaugugcuaa.....................................................    1    0    FF1
.......................aggggaaugcuguacacucuaugugcuaaaaaauauA.............................................    1    1    FF1
.......................aggggaaugcuguacacucuaugugcuaaaaaauaucca...........................................    1    0    FF1
.............................................................uaagcguauagcuuuucccauu......................    7    0    FF1

.......................aggggaaugcuguacac.................................................................    1    0    OV1
.......................Gggggaaugcuguacacuc...............................................................    1    1    OV1
.......................aggggaaugcuguacacucu..............................................................    1    0    OV1
.......................aggggaaugcuguacacucuau............................................................    2    0    OV1
.......................aggggaaugcuguacacucuauA...........................................................    1    1    OV1
.......................aggggaaugcuguacacucuaug...........................................................    28   0    OV1
.......................aggggaaugcuguacacucuaugugc........................................................    3    0    OV1
.......................aggggaaugcuguacacucuaCgugc........................................................    1    1    OV1
.......................aggggaaugcuguacacucuaugugcu.......................................................    3    0    OV1
.......................aggggaaugcuguacacucuaugugcua......................................................    5    0    OV1
```

```
                                                             aga-mir-10375b-2-star
                       aga-mir-10375b-2
gaaguaaaucgacgaauucugaaaggggaaugcuguacacucuaugugcuaaaaauaucca[illegible]uuagcguucguugcgagcaaaca
                       aga-mir-10375b-1                      aga-mir-10375b-1-star

.......................aggggaaugcuguacacucuaugugcuaa.....................................................    1    0    OV1
.......................aggggaaugcuguacacucuaugugcuaaaaauauc..............................................    1    0    OV1
.......................aggggaaugcuguacacucuaugugcuaaaaauaucc.............................................    2    0    OV1
.......................aggggaaugcuguacacucuaugugcuaaaaauaucca............................................    3    0    OV1
.............................................................uaagcguauagcuuuucccauu......................    2    0    OV1

.......................aggggaaugcuguacacucu..............................................................    1    0    MF1
.......................aggggaaugcuguacacucua.............................................................    1    0    MF1
.......................aggggaaugcuguacacucuau............................................................    2    0    MF1
.......................aggggaaugcuguacacucuaug...........................................................    25   0    MF1
.......................aggggaaugcuguacacucuauC...........................................................    1    1    MF1
.......................aggggaaugcuguacacucuaugA..........................................................    1    1    MF1
.......................aggggaaugcuguacacucuaugugcuaaaaauau...............................................    1    0    MF1
.......................aggggaaugcuguacacucuaugugcuaaaaauaucca............................................    1    0    MF1
.........................gggaaugcuguacacucuaug...........................................................    1    0    MF1
.............................................................uaagcguauagcuuuucccauu......................    3    0    MF1
.............................................................uaagcguauagcuuuucccauuu.....................    1    0    MF1

.......................aggggaaugcuguacacucu..............................................................    1    0    BF2
.......................aggggaaugcuguacacucua.............................................................    4    0    BF2
.......................aggggaaugcuguacacucuau............................................................    5    0    BF2
.......................aggggaaugGuguacacucuaug...........................................................    1    1    BF2
.......................aggggaGugcuguacacucuaug...........................................................    1    1    BF2
.......................aggggaaugAuguacacucuaug...........................................................    1    1    BF2
.......................aggggaaugcuguacacucuaug...........................................................    123  0    BF2
.......................aggggaaugcuguacacucuaugC..........................................................    2    1    BF2
.......................aggggaaugcuguacacucuaugugc........................................................    1    0    BF2
.......................aggggaaugcuguacacucuaugugcu.......................................................    4    0    BF2
.......................aggggaaugcuguacacucuaugugcua......................................................    1    0    BF2
.......................aggggaaugcuguacacucuaugugcuaaaaaua................................................    2    0    BF2
.......................aggggaaugcuguacacucuaugugcuaaaaauau...............................................    1    0    BF2
.......................aggggaaugcuguacacucuaugugcuaaaaauauc..............................................    1    0    BF2
.......................aggggaaugcuguacacucuaugugcuaaaaauaucc.............................................    4    0    BF2
.......................aggggaaugcuguacacucuaugugcuaaaaauaucca............................................    15   0    BF2
.............................................................uaagcguauagcuuuucccauu......................    1    0    BF2
.............................................................uaagcguauUgcuuuucccauu......................    1    1    BF2
...............................................................agcguauagcuuuucccauu......................    1    0    BF2

....uaaaucgacgaauucugaa..................................................................................    1    0    BF1
.......................aggggaaugcuguacacucua.............................................................    2    0    BF1
.......................aggggaaugcuguacacucuaug...........................................................    15   0    BF1
.......................aggggaaugcuguacacucuaugug.........................................................    2    0    BF1
.......................aggggaaugcuguacacucuaugugc........................................................    2    0    BF1
.......................aggggaaugcuguacacucuaugugcu.......................................................    4    0    BF1
.......................agggUaaugcuguacacucuaugugcua......................................................    1    1    BF1
.......................aggggaaugcuguacacucuaugugcua......................................................    2    0    BF1
.......................aggggaaugcuguacacucuaugugcuaaaaauau...............................................    1    0    BF1
.......................aggggaaugcuguacacucuaugugcuaaaaauauc..............................................    3    0    BF1
.......................aggggaaugcuguacacucuaugugcuaaaaauaucc.............................................    11   0    BF1
.......................aggggaaugcuguacacucuaugugcuaaaaauaucca............................................    8    0    BF1
.............................................................uaagcguauagcuuuucccauu......................    1    0    BF1

...guaaaucgacgaauucugaa..................................................................................    1    0    MW1
.......................aggggaaugcuguacacucu..............................................................    1    0    MW1
.......................aggggaaugcuguacacucua.............................................................    3    0    MW1
.......................aggggaaugcuguacacucuau............................................................    4    0    MW1
.......................aggggaaugcuguacacucuauU...........................................................    1    1    MW1
.......................aggggaauAcuguacacucuaug...........................................................    1    1    MW1
.......................aggggaaugcuguacacucuaug...........................................................    75   0    MW1
.......................aggggaaugcuguacacucuaugugcu.......................................................    1    0    MW1
.......................aggggaaugcuguacacucuaugugcuaaaaauauc..............................................    1    0    MW1
.......................aggggaaugcuguacacucuaugugcuaaaaauaucU.............................................    1    1    MW1
.........................gggaaugcuguacacucuaug...........................................................    2    0    MW1
.............................................................uaagcguauagcuuuucccau.......................    2    0    MW1
.............................................................uaagcguauagcuuuucccauu......................    1    0    MW1
.................................................................cguauagcuuuucccauu......................    1    0    MW1

.......................aggggaaugcuguacacucu..............................................................    1    0    FW1
```

aga-mir-10375b-2-star

aga-mir-10375b-2

gaaguaaaucgacgaauucugaaaggggaaugcuguacacucuaugugcuaaaaauauccauaagcguauagcuuuucccauuuagcguucguugcgagcaaaca

aga-mir-10375b-1-star

aga-mir-10375b-1

.......................aggggaaugcuguacacucua...	1	0	FW1
.......................aggggaaugcuguacacucuau..	2	0	FW1
.......................aggggaaugcuguacacucuaug...	30	0	FW1
.......................aggggaaCgcuguacacucuaug...	1	1	FW1
.......................aggggaaugcuguacacucuaugugcua..	1	0	FW1
.........................gggaaugcuguacacucuaug...	1	0	FW1
....uaagcguauagcuuuucccauu......................	3	0	FW1
....uaagcguauagcuuuucccauuu.....................	1	0	FW1

.......................aggggaaugcuguacacucua...	1	0	MW2
.......................aggggaaugcuguacacucuau..	1	0	MW2
.......................aggggaaugcuguacacucuaug...	22	0	MW2
.........................gggaaugcuguacacucua...	1	0	MW2
.........................gggaaugcuguacacucuaug...	1	0	MW2

.......................aggggaaugcuguacacu..	1	0	TE2
.......................aggggaaugcuguacacuc...	2	0	TE2
.......................aggggaaugcuguacacucua...	6	0	TE2
.......................aggggaaugcuguacacucuau..	4	0	TE2
.......................aggggaaugcuguacacucuaug...	54	0	TE2
.......................aggggaaugcuguacacucuaugu..	1	0	TE2
.......................aggggaaugcuguacacucuaugug...	1	0	TE2
.......................aggggaaugcuguacacucuaugugc..	1	0	TE2
.......................aggggaaugcuguacacucuaugugcA...	1	1	TE2
.......................aggggaaugcuguacacucuaugugcu...	5	0	TE2
.......................aggggaaugcuguacacucuaugugcua..	2	0	TE2
.......................aggggaaugcuguacacucuaugugcuaa...	2	0	TE2
.......................aggggaaugcuguacacucuaugugcuaaa..	1	0	TE2
.......................aggggaaugcuguacacucuaugugcuaaaaaua..	1	0	TE2
.......................aggggaaugcuguacacucuaugugcuaaaaauauc..	1	0	TE2
.......................aggggaaugcuguacacucuaugugcuaaaaauaucc...	1	0	TE2
....uaagcguauagcuuuucccau.......................	1	0	TE2
....uaagcguauagcuuuucccauuu.....................	1	0	TE2

miRBase precursor : aga-mir-10375b-3
Total read count : 71
aga-mir-10375b-3 read count : 61
aga-mir-10375b-3-star read count : 3
remaining reads : 7

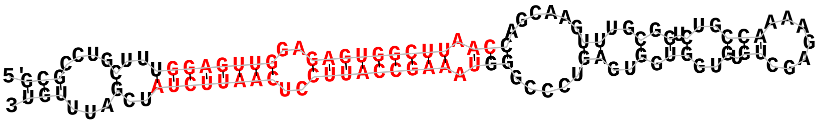

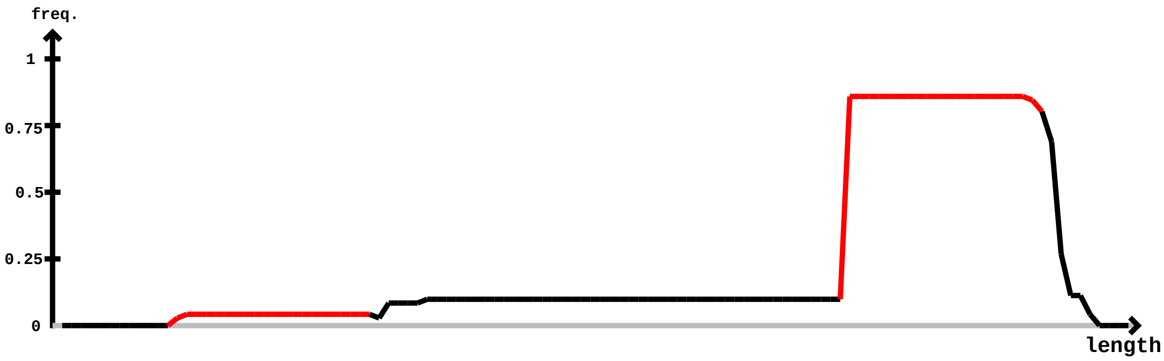


```
                 aga-mir-10375b-3-star
                                                                                  aga-mir-10375b-3
5'- gcgccugcuuuggaguuggagaguggcuuaaccagcaaguuugcggucugccaaaagagcugugugguggugagucccggguaaagccauucccucaauucuaucgauuugu  -3'   exp
    ((....))..((((((((..(((((((((.(((.(((....)))((.((((((..(.(....).)..)))).)).))..))).)))))))))..)))))))).........  reads    mm    sample
    .................................................................................uaaagccauucccucaauucuau........  2        0     OV2
    .................................................................................uaaagccauucccucaauucuauU.......  1        1     OV2

    .................................................................................uaaagccauucccucaauucuau........  1        0     FF2

    ...........ggaguuggagaguggcuuaac................................................................................  1        0     TE1
    .................................agcaaguuugcgAucugccaaaagagcugugugguggugagucccggg...............................  1        1     TE1
    .................................agcaaguuugcggucugccaaaagagcugugugguggugagucccggg...............................  4        0     TE1
    .....................................aAuuugcggucugccaaaagagcugugugguggugagucccggg...............................  1        1     TE1
    .................................................................................uaaagccauucccucaauuc...........  1        0     TE1
    .................................................................................uaaagccauucccucaauucu..........  1        0     TE1
    .................................................................................uaaagccauucccucaauucuau........  9        0     TE1
    .................................................................................uaaagccauucccucaauucuaucga.....  1        0     TE1
    .................................................................................uaaagccauucccucaauucuaucgau....  1        0     TE1

    .................................................................................uaaagccauucccucaauucu..........  1        0     MF2
    .................................................................................uaaagccauucccucaauucua.........  2        0     MF2
    .................................................................................uaaagccauucccucaauucuau........  6        0     MF2
    .................................................................................uaaagccauucccucaauucuauU.......  1        1     MF2
    .................................................................................uaaagccauucccucaauucuaucga.....  1        0     MF2

    .................................................................................uaaagccauucccucaauucuau........  1        0     OV1
    .................................................................................uaaagccauucccucaauucuauU.......  1        1     OV1

    .................................................................................uaaagccauucccucaauucuau........  1        0     MF1

    .................................................................................uaaagccauucccucaauucuau........  2        0     BF2
    .................................................................................uaaagccauucccucaauucuaucgau....  1        0     BF2

    .................................agcaaguuugcggucugccaaaagagcugugugguggugagucccggg...............................  1        0     BF1

    .................................................................................uaaagccauucccucaauucuaucga.....  1        0     FW1

    ...........Agaguuggagaguggcuuaacc...............................................................................  1        1     TE2
```

```
           aga-mir-10375b-3-star
                                                                                 aga-mir-10375b-3
gcgccugcuuuggaguuggagaguggcuuaaccagcaaguuugcggucugccaaaagagcugugugguggugagucccggguaaagccauuccucaauucuaucgauuugu

............gaguuggagaguggcuuaacc..............................................................................     1     0     TE2
.................................................................................uaaagccauuccucaauucu..........     1     0     TE2
.................................................................................uaaagccauuccucaauucua.........     6     0     TE2
.................................................................................uaaagccauuccucaauucuau........     8     0     TE2
.................................................................................uaaagccauuccucaauucuauA.......     5     1     TE2
.................................................................................uaaagccauuccucaauucuauc.......     3     0     TE2
.................................................................................uaaagccauuccucaauucuaucga.....     2     0     TE2
.................................................................................uaaagccauuccucaauucuaucgau....     1     0     TE2
```

miRBase precursor : aga-mir-10376
Total read count : 228
aga-mir-10376 read count : 172
aga-mir-10376-star read count : 56
remaining reads : 0

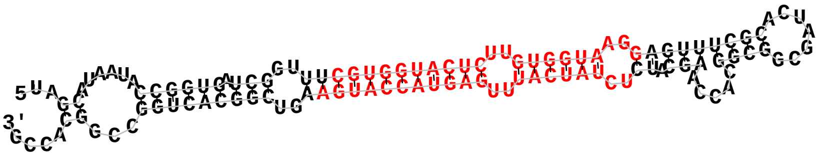

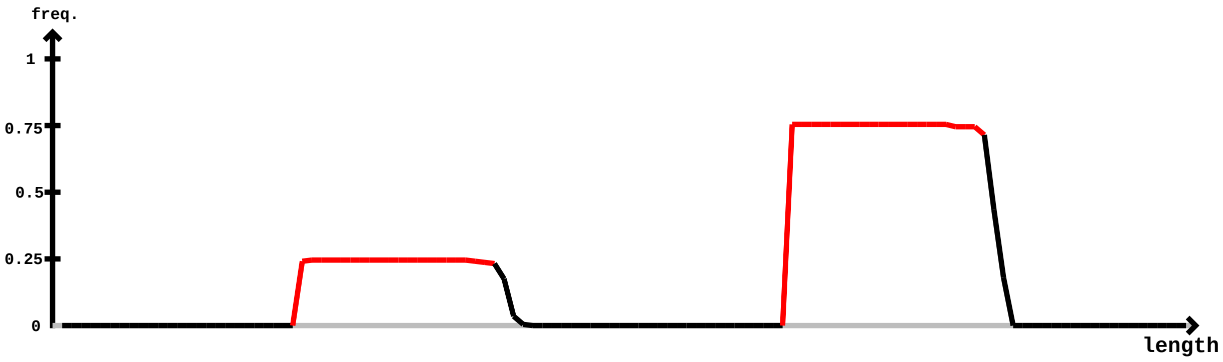


```
                        aga-mir-10376-star
                                                                           aga-mir-10376
5'- uagcauaauaccggugaucgguuucgugguacucuugugguaaggaguuucgcacuagcggcggcaccaagcaucucuaucauuugaguaccaugaagucggcacuggccggcaccg   -3'    exp
    ..((......((((((.(((..((((((((((((..((((((..((...((((....))))..((.....)).)).)).))))))..))))))))))))..)))))))))...))....   reads   mm   sampl
    ...........................................................................ucuaucauuugaguaccauga.....................   2   0   OV2
    ...........................................................................ucuaucauuugaguaccaugaa....................   2   0   OV2
    ...........................................................................ucuaucauuugaguaccaugaaA...................   2   1   OV2
    ...........................................................................ucuaucauuugaguaccaugaaU...................   1   1   OV2

    ........................cgugguacucuugugguaagg........................................................................   1   0   TE1
    ........................cgugguacucuugugguaagga.......................................................................   1   0   TE1

    ........................cgugguacucuugugguaagg........................................................................   1   0   FF2
    ........................cgugguacucuugugguaaggaCu.....................................................................   1   1   FF2
    ...........................................................................ucuaucauuugaguaccauga.....................   3   0   FF2
    ...........................................................................ucuaucauuugaguaccaugaaA...................   1   1   FF2

    ........................cgugguacucuCgugguaa..........................................................................   1   1   MF2
    ........................cgugguacucuugugguaaA.........................................................................   1   1   MF2
    ........................cgugguacucuugugguaagg........................................................................   4   0   MF2
    ........................cgugguacucuugugguaagga.......................................................................   10   0   MF2
    ........................cgugguacucuugugguaaggaA......................................................................   6   1   MF2
    ........................cgugguacucuugugguaaggaU......................................................................   1   1   MF2
    .........................gugguacucuugugguaagga.......................................................................   1   0   MF2
    ...........................................................................ucuaucauuugaguaccaug......................   3   0   MF2
    ...........................................................................ucuaucauuugaguaccauga.....................   14   0   MF2
    ...........................................................................ucuaucauuugaguaccaugaC....................   4   1   MF2
    ...........................................................................ucuaucauuugaguaccaugaa....................   18   0   MF2
    ...........................................................................ucuaucauuugaguaccaugaaA...................   11   1   MF2
    ...........................................................................ucuaucauuugaguaccaugaaC...................   4   1   MF2
    ...........................................................................ucuaucauuugaguaccaugaaU...................   2   1   MF2

    ........................cgugguacucuugugguaagga.......................................................................   2   0   FW2
    ...........................................................................ucuaucauuugaguaccauga.....................   8   0   FW2
    ...........................................................................ucuaucauuugaguaccaugaa....................   3   0   FW2
    ...........................................................................ucuaucauuugaguaccaugaU....................   1   1   FW2
    ...........................................................................ucAaucauuugaguaccaugaa....................   1   1   FW2
    ...........................................................................ucuaucauuugaguaccaugaaA...................   3   1   FW2
```

```
                        aga-mir-10376-star
                                                                           aga-mir-10376
uagcauaauaccggugaucgguuucgugguacucuugugguaaggaguuucgcacuagcggcggcaccaagcaucucuaucauuugaguaccaugaagucggcacuggccggcaccg

...........................................................................ucuaucauuugaguaccauga.....................  4   0   OV1
...........................................................................ucuaucauuugaguaccaugaa....................  4   0   OV1
...........................................................................ucuaucauuugaguaccaugaaA...................  1   1   OV1

........................cgugguacucuugugguaagg........................................................................  2   0   FF1
........................cguUguacucuugugguaagg........................................................................  1   1   FF1
........................cguggGacucuugugguaagg........................................................................  1   1   FF1
........................cgugguacucuugugguaagga.......................................................................  2   0   FF1
........................cguUguacucuugugguaagga.......................................................................  1   1   FF1
...........................................................................ucuaucauuugaguaccaug......................  2   0   FF1
...........................................................................ucuaucauuugaguaccauga.....................  8   0   FF1
...........................................................................ucuaucauuugaguaccaugaa....................  10  0   FF1
...........................................................................ucuaucauuugaguaccaugaaA...................  9   1   FF1

........................cgugguacucuugugguaagga.......................................................................  10  0   MF1
...........................................................................ucuaucauuugaguaccaug......................  2   0   MF1
...........................................................................ucuaucauuugaguaccauga.....................  3   0   MF1
...........................................................................ucuaucauuugaguaccaugaC....................  1   1   MF1
...........................................................................ucuaucauuugaguaccaugaa....................  3   0   MF1
...........................................................................ucuaucauuugaguaccaugaaA...................  3   1   MF1

........................cgugguacucuugugguaagg........................................................................  2   0   BF2
...........................................................................ucuaucauuugaguaccauga.....................  3   0   BF2
...........................................................................ucuaucauuugaguaccaugaa....................  1   0   BF2

...........................................................................ucuaucauuugaguaccauga.....................  3   0   BF1
...........................................................................ucuaucauuugaguaccaugaa....................  3   0   BF1
...........................................................................ucuaucauuugaguaccaugaaA...................  2   1   BF1

........................cgugguacucuuguggua...........................................................................  1   0   FW1
........................cgugguacucuugugguaagga.......................................................................  1   0   FW1
...........................................................................ucuaucauuugaguaccauga.....................  2   0   FW1

........................cgugguacucuugugguaagg........................................................................  1   0   MW1
........................cgugguacucuugugguaagga.......................................................................  4   0   MW1
...........................................................................ucuaucauuugaguacc.........................  2   0   MW1
...........................................................................ucuaucauuugaguaccauga.....................  8   0   MW1
...........................................................................ucuaucauuugaguaccaugaa....................  2   0   MW1

...........................................................................ucuaucauuugaguaccauga.....................  4   0   MW2
...........................................................................ucuaucauuugaguaccaugaaA...................  1   1   MW2

...........................................................................ucuaucauuugaguaccauga.....................  2   0   TE2
...........................................................................ucuaucauuugaguaccaugaa....................  5   0   TE2
...........................................................................ucuaucauuugaguaccaugaaA...................  1   1   TE2
```

miRBase precursor : aga-mir-10377a
Total read count : 36
aga-mir-10377a read count : 1
aga-mir-10377a-star read count : 35
remaining reads : 0

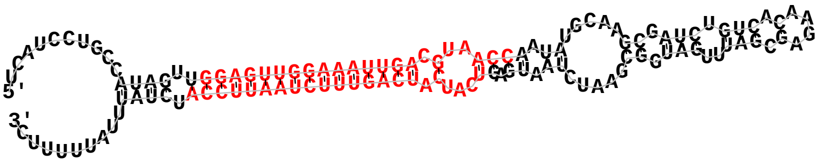

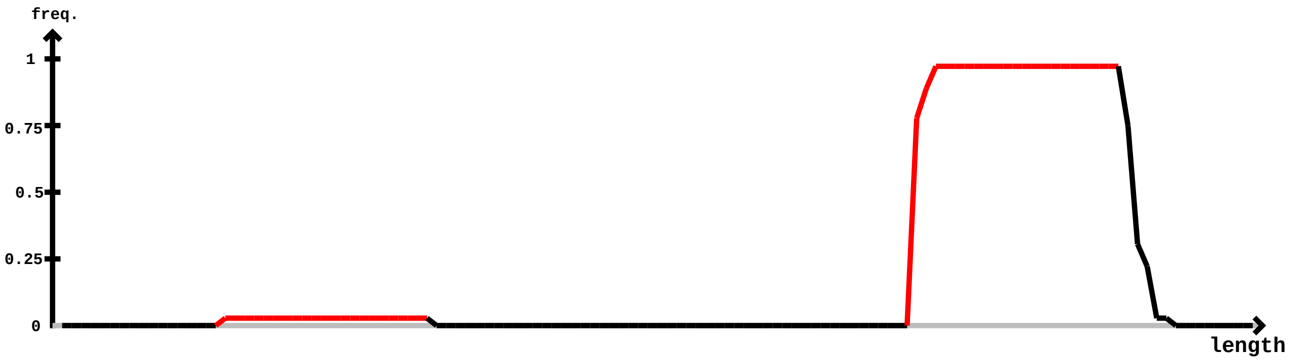


```
                                                                                          aga-mir-10377a-star
                aga-mir-10377a
5'- ucauccugccauaguuggaguuggaaauugacguaaccaauaugcaagcgaucugucacaagagcgauuugauggcgaaucuaaugagucaucaucaguuucuaauuccaucuauuuauuuuuc  -3'   exp
    ..........((((.((((((((((((((((.................((.(((......))).))...(((((((...........))))))))))))))))))))))).)))).........   reads   mm
    ........................................................................................ucaucaucaguuucuaauuccau.............   1       0

    ........................................................................................ucaucaucaguuucuaauuccau.............   1       0

    ................ggaguuggaaauugacguaacc......................................................................................   1       0
    ........................................................................................ucaucaucaguuucuaauucca..............   4       0
    .........................................................................................Uaucaucaguuucuaauuccaucu...........   1       1

    ........................................................................................ucaucaucaguuucuaauucca..............   2       0
    ........................................................................................ucaucaucaguuucuaauuccau.............   5       0
    ........................................................................................ucaucaucaguuucuaauuccaucu...........   1       0
    ........................................................................................ucaucaucaguuucuaauuccaucuau.........   1       0
    ..........................................................................................aucaucaguuucuaauuccau.............   1       0

    ........................................................................................ucaucaucaguuucuaauucca..............   2       0
    ........................................................................................ucaucaucaguuucuaauuccau.............   3       0
    ........................................................................................ucaucaucaguuucuaauuccaucu...........   2       0

    ........................................................................................ucaucaucaguuucuaauuccau.............   1       0
    ........................................................................................ucaucaucaguuucuaauuccaucu...........   1       0

    ........................................................................................ucaucaucaguuucuaauuccaucu...........   1       0

    ........................................................................................ucaucaucaguuucuaauuccau.............   3       0
    .........................................................................................Uaucaucaguuucuaauuccau.............   1       1
    .........................................................................................Uaucaucaguuucuaauuccauc............   1       1
    .........................................................................................Uaucaucaguuucuaauuccaucu...........   1       1
    ..........................................................................................aucaucaguuucuaauuccauc............   1       0
    ..........................................................................................aucaucaguuucuaauuccauU............   1       1
```

miRBase precursor : aga-mir-10377b
Total read count : 25
aga-mir-10377b read count : 23
aga-mir-10377b-star read count : 1
remaining reads : 1

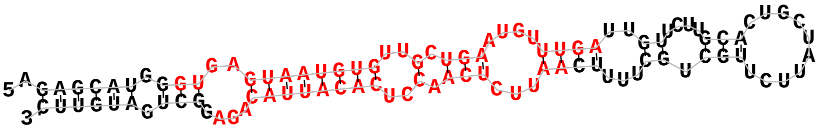

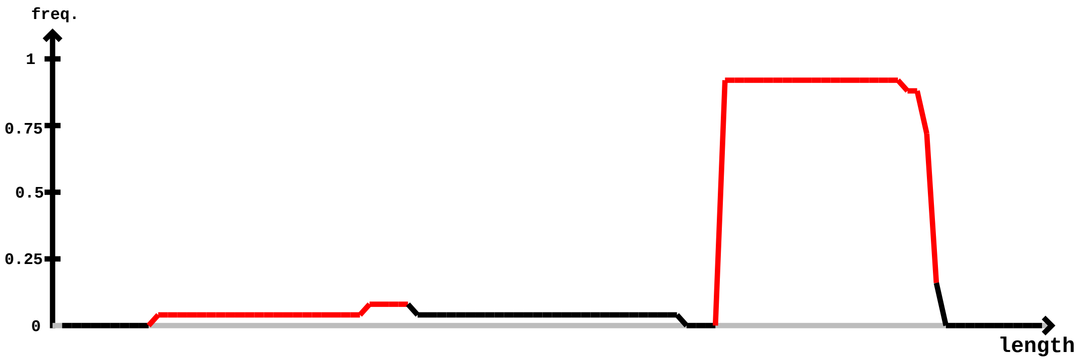

aga-mir-10377b-star

aga-mir-10377b

```
5'- agagcaugggugaguaauguguugcugaauguuugauuguucuugcacugcuauucuugcugcuuuucaauucucaaccucacauuacagaggcugauguuc   -3'    exp
    .((((((.(((..((((((((..(.(((....((((..((....(((..........))).))...))))...))).)..))))))))....))).))))))    reads      mm         sample
    ....................................................................aauucucaaccucaAauuacag............    1          1          FF2

    ....................................................................aauucucaaccucacauuaca.............    1          0          OV2

    ....................................................................aauucucaaccucacauuacag............    5          0          MF2
    ....................................................................aauucucaaccucacauuacagC...........    1          1          MF2
    ....................................................................aauucucaaccucacauuacagU...........    1          1          MF2

    ...............................uuugauuguucuugcacugcuauucuugcugcu.......................................    1          0          FW2

    ....................................................................aauucucaaccucacauuacag............    2          0          FF1
    ....................................................................aauucucaaccucacauuacagU...........    1          1          FF1

    ....................................................................aauucucaaccucacauuaca.............    1          0          OV1
    ....................................................................aauucucaaccucacauuacag............    1          0          OV1
    ....................................................................aauucucaaccucacauuacagC...........    1          1          OV1

    ....................................................................aauucucaaccucacauuacag............    3          0          MF1

    .........gugaguaauguguugcugaauguuuga..................................................................    1          0          BF2
    ....................................................................aauucucaaccucacauuaca.............    1          0          BF2
    ....................................................................aauucucaaccucacauuacag............    1          0          BF2

    ....................................................................aauucucaaccucacauua...............    1          0          BF1

    ....................................................................aauucucaaccucacauuaca.............    1          0          FW1
    ....................................................................aauucucaaccucacauuacag............    1          0          FW1
```

miRBase precursor : aga-mir-10378
Total read count : 18
aga-mir-10378 read count : 17
remaining reads : 1

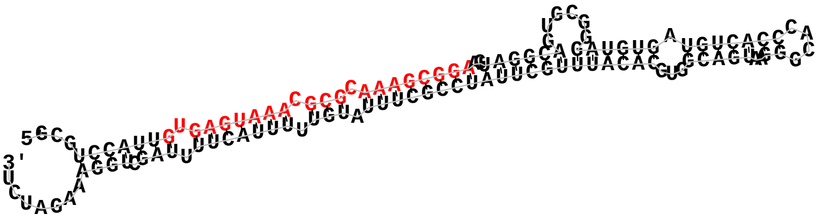

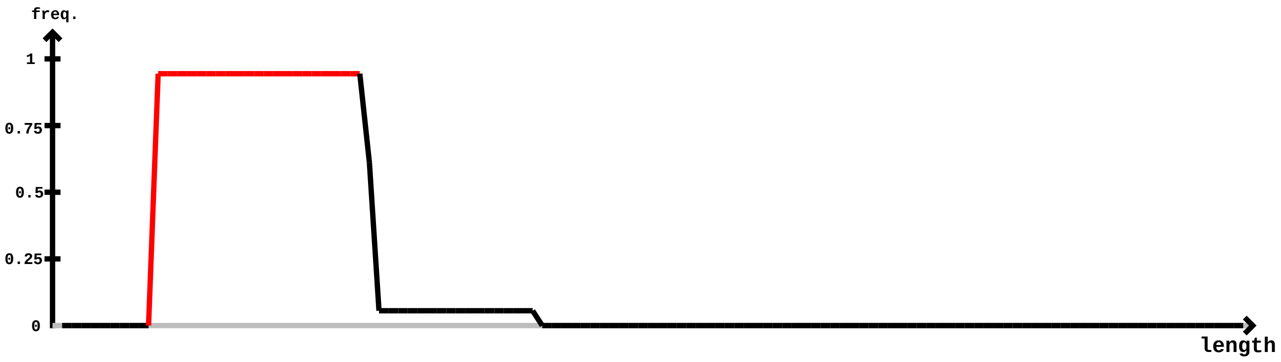


```
            aga-mir-10378
5'- gcguccauugugaguaaacgcgcaaagcggaaguaggcagugcgggaugugaugucacccacgggaaugacggugcacauuugcuuauccgcuuuauguuuuuacuuuuagcuggaaagaucu   -3'    exp
    ...((((((.((((((.(((.((((((((..((((((......((((((.(((((((....))..)))))...)))))))))))))))))))).))).)))))))).))).)))).......    reads      mm
    .........gugaguaaacgcgcaaagcgga............................................................................................    1          0
    .........gugaguaaacgcgcaaagcggaU...........................................................................................    5          1

    .........gugaguaaacgcgcaaagcgga............................................................................................    1          0
    .........gugaguaaacgcgcaaagcggaU...........................................................................................    2          1

    .........gugaguaaacgcgcaaagcgga............................................................................................    1          0

    .........gugaguaaacgcgcaaagcggaU...........................................................................................    1          1

    .........gugaguaaacgcgcaaagcgga............................................................................................    1          0
    .........gugaguaaacgcgcaaagcggaC...........................................................................................    1          1

    .........gugaguaaacgcgcaaagcgga............................................................................................    1          0

    .........gugaguaaacgcgcaaagcgga............................................................................................    2          0
    ...............................aguaggcagugcgggaug..........................................................................    1          0

    .........gugaguaaacgcgcaaagcggaU...........................................................................................    1          1
```

miRBase precursor : aga-mir-10379
Total read count : 801
aga-mir-10379 read count : 729
aga-mir-10379-star read count : 72
remaining reads : 0

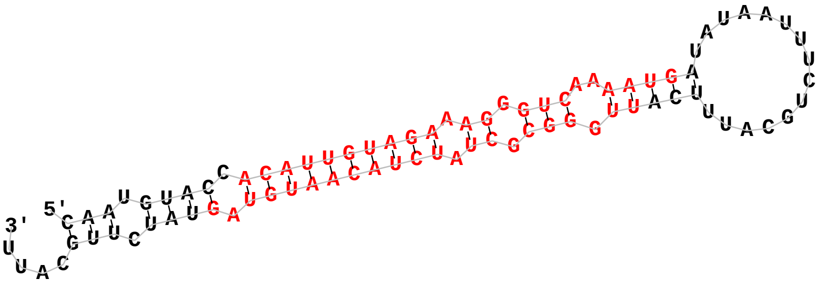

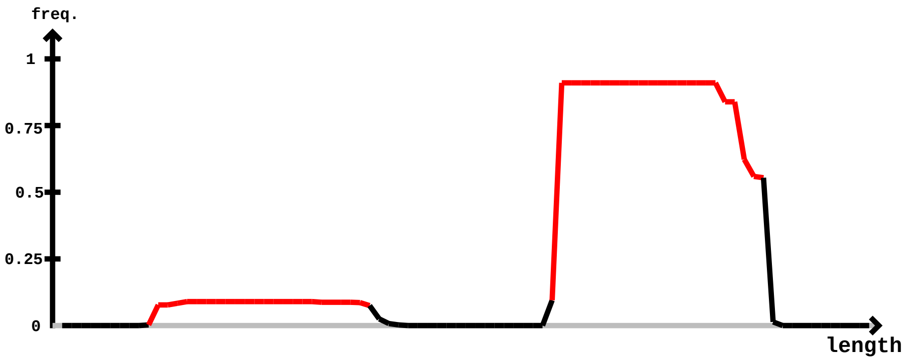

aga-mir-10379-star

aga-mir-10379

```
5'- caauguaccacauuguagaaagggucaaaaugauauaauuucugcauuucauugggcgcuaucuacaauguaguaucuugcauu  -3'   exp
    (((.((((.((((((((((.((.(((..(((((...............))))).))).)).)))))))))).)))).)))....   reads   mm   sample
    ...................................................uugggcgcuaucuacaauguag...........   3       0    FF2

    ..................................................auugggcgcuaucuacaaug..............   3       0    OV2
    ..................................................auugggcgcuaucuacaaugu.............   2       0    OV2
    ..................................................auugggcgcuaucuacaauguag...........   7       0    OV2
    ..................................................auugggcgcuUucuacaauguag...........   1       1    OV2
    ..................................................auugggcgcuaucuacaauguagA..........   1       1    OV2
    ...................................................uugggcgcuaucuacaa................   1       0    OV2
    ...................................................uugggcgcuaucuacaaug..............   8       0    OV2
    ...................................................uugggcgcuUucuacaaugu.............   2       1    OV2
    ...................................................uugggcgcuaucuacaaugu.............   1       0    OV2
    ...................................................uugggcgcuaucuacaauguag...........   23      0    OV2
    ...................................................uugggcgcuUucuacaauguag...........   7       1    OV2

    ........cacauuguagaaagggucaaaaug....................................................   2       0    TE1
    .........acauuguagaaagggucaaaa......................................................   1       0    TE1
    .........acauuguagaaagggucaaaau.....................................................   3       0    TE1
    .........acauuguagaaagggucaaaaug....................................................   17      0    TE1
    .........acauuguagaaagggucaaaauga...................................................   2       0    TE1
    ...........auuguagaaagggucaaaauga...................................................   2       0    TE1
    ............uuguagaaagggucaaaaugaua.................................................   2       0    TE1
    ..................................................auugggcgcuUucuacaaug..............   2       1    TE1
    ..................................................auugggcgcuUucuacaaugu.............   1       1    TE1
    ..................................................auugggcgcuUucuacaauguag...........   2       1    TE1
    ...................................................uugggcgcuUucuacaa................   17      1    TE1
    ...................................................uugggcgcuUucuacaaug..............   28      1    TE1
    ...................................................uugggcgcuaucuacaaug..............   5       0    TE1
    ...................................................uugggcgcuUucuacaaugu.............   6       1    TE1
    ...................................................uugggcgcuaucuacaaugu.............   2       0    TE1
    ...................................................uugggcgcuUucuacaaugua............   1       1    TE1
    ...................................................uugggcgcuaucuacaauguag...........   23      0    TE1
    ...................................................uugggcgcuUucuacaauguag...........   37      1    TE1
    ...................................................uugggcAcuaucuacaauguag...........   1       1    TE1
    ...................................................uugggcgcuUucuacaauguagu..........   2       1    TE1
```

```
         aga-mir-10379-star
                                                   aga-mir-10379
caauguaccacauuguagaaagggucaaaaugauauaauuucugcauuucauugggcgcuaucuacaauguaguaucuugcauu

...................................................uugggcgcuaucuacaauguagu..........    1    0    TE1

.........acauuguagaaagggucaaaau.....................................................    3    0    MF2
.........acauuguagaaagggucaaaaug....................................................    6    0    MF2
.........acauuguagaaagggucaaaauga...................................................    3    0    MF2
...........auuguagaaagggucaaaauga...................................................    1    0    MF2
............uuguagaaagggucaaaaugau..................................................    1    0    MF2
..................................................auugggcgcuaucuacaauguag...........    6    0    MF2
...................................................uugggcgcuUucuacaa................    2    1    MF2
...................................................uugggcgcuUucuacaaug..............    3    1    MF2
...................................................uugggcgcuaucuacaaug..............    1    0    MF2
...................................................uugggcgcuUucuacaaugu.............    1    1    MF2
...................................................uugggcgcuaucuacaauguag...........    8    0    MF2
...................................................uuAggcgcuaucuacaauguag...........    1    1    MF2
...................................................uugggcgcuUucuacaauguag...........    8    1    MF2

.........acauuguagaaagggucaaaaug....................................................    2    0    OV1
..................................................auugggcgcuaucuacaaug..............    4    0    OV1
..................................................auugggcgcuaucuacaaugu.............    2    0    OV1
..................................................auugggcgcuaucuacaauguag...........    17   0    OV1
..................................................auugggcgcuUucuacaauguag...........    1    1    OV1
...................................................uugggcgcuaucuacaaug..............    4    0    OV1
...................................................uugggcgcuUucuacaaug..............    3    1    OV1
...................................................uugggcgcuaucuacaaugu.............    3    0    OV1
...................................................uugggcgcuauGuacaauguag...........    1    1    OV1
...................................................uugggcgcuaucuacaauguag...........    51   0    OV1
...................................................uugggcgcuUucuacaauguag...........    8    1    OV1
...................................................uugggcgcuaucuacaauguaU...........    1    1    OV1
...................................................uugggcgcuaucuacaauguagA..........    1    1    OV1

.........acauuguagaaagggucaaaaug....................................................    2    0    MF1
.........acauuguagaaagggucaaaauga...................................................    1    0    MF1
...........auuguagaaagggucaaaauga...................................................    1    0    MF1
...................................................uugggcgcuUucuacaauguag...........    2    1    MF1

.........acauuguagaaagggucaaaau.....................................................    1    0    BF2
..................................................auugggcgcuaucuacaaug..............    1    0    BF2
..................................................auugggcgcuaucuacaauguag...........    2    0    BF2
..................................................auugggcgcuaucuacaauguagA..........    1    1    BF2
...................................................uugggcgcuaucuacaaug..............    3    0    BF2
...................................................uugggcgcuUucuacaaug..............    1    1    BF2
...................................................uugggcgcuaucuacaaugua............    1    0    BF2
...................................................uugggcgcuaucuacaauguag...........    22   0    BF2
...................................................uugggcgcuaucuacaauguaA...........    1    1    BF2
...................................................uugggcgcuUucuacaauguag...........    3    1    BF2
...................................................uugggcgcuaucuacaauguagu..........    1    0    BF2

..................................................auugggcgcuaucuacaaug..............    1    0    BF1
..................................................auugggcgcuaucuacaauguag...........    4    0    BF1
...................................................uugggcgcuUucuacaaugu.............    1    1    BF1
...................................................uugggcgcuaucuacaauguag...........    18   0    BF1
...................................................uugggcgcuUucuacaauguag...........    1    1    BF1
...................................................uugggcgcuaucuacaauguagA..........    2    1    BF1

...................................................uugggcgcuaucuacaauguag...........    1    0    MW2

.........acauuguagaaaggguc..........................................................    2    0    TE2
.........acauuguagaaagggucaaaau.....................................................    2    0    TE2
.........acauuguagaaagggucaaaaug....................................................    10   0    TE2
.........acauuguagaaagggucaaaaAg....................................................    1    1    TE2
.........acauuguagaaagggucaaaauga...................................................    4    0    TE2
...........auuguagaaagggucaaaaugau..................................................    1    0    TE2
............uuguagaaagggucaaaaugau..................................................    2    0    TE2
..................................................auugggcgcuUucuacaa................    1    1    TE2
..................................................auugggcgcuUucuacaaug..............    7    1    TE2
..................................................auugggcgcuaucuacaaug..............    4    0    TE2
..................................................auugggcgcuUucuacaaugu.............    1    1    TE2
..................................................auugggcgcuaucuacaaugu.............    1    0    TE2
..................................................auugggcgcuaucuacaauguag...........    4    0    TE2
```

aga-mir-10379-star

aga-mir-10379

caauguaccacauuguagaaagggucaaaaugauauaauuucugcauuucauugggcgcuaucuacaauguaguaucuugcauu

....uugggcgcuUucuacaa................	35	1	TE2
....uugggcgcuaucuacaa................	1	0	TE2
....uugggcgcuUucuacaaug..............	87	1	TE2
....uugggcgcuaucuacaaug..............	8	0	TE2
....uugggcgcuUucuacaaugu.............	25	1	TE2
....uugggcgcuaucuacaaugu.............	3	0	TE2
....uugggcgcuUucuacaaugua............	2	1	TE2
....uugggcgcuUucuacaauguag...........	98	1	TE2
....uugggcgcuaucuacaauguag...........	71	0	TE2
....uugggcgcuUucuacaauguagu..........	2	1	TE2

miRBase precursor : aga-mir-10380
Total read count : 801
aga-mir-10380 read count : 729
aga-mir-10380-star read count : 72
remaining reads : 0

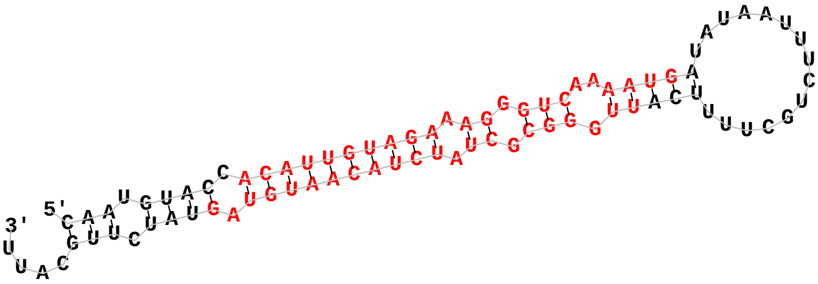

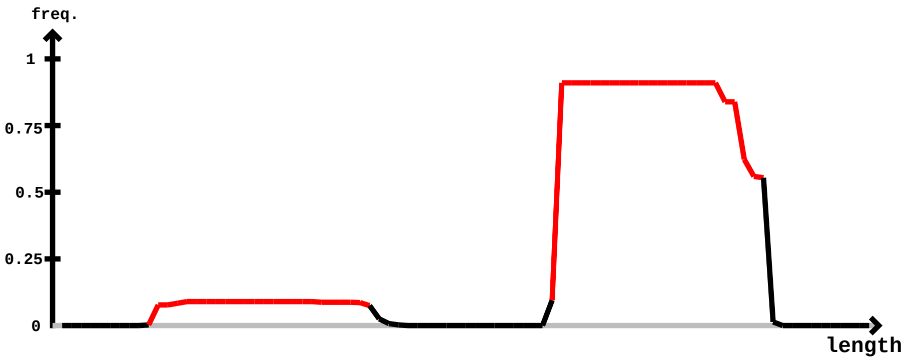


```
         aga-mir-10380-star
                                          aga-mir-10380
5'- caauguaccacauuguagaaagggucaaaaugauauaauuucugcuuuucauugggcgcuaucuacaauguaguaucuugcauu  -3'   exp
    (((.((((.((((((((((.((.(((..(((((...............))))).))).)).)))))))))).)))).)))....   reads   mm   sample
    ...................................................uugggcgcuaucuacaauguag...........   3       0    FF2

    ..................................................auugggcgcuaucuacaaug..............   3       0    OV2
    ..................................................auugggcgcuaucuacaaugu.............   2       0    OV2
    ..................................................auugggcgcuaucuacaauguag...........   7       0    OV2
    ..................................................auugggcgcuUucuacaauguag...........   1       1    OV2
    ..................................................auugggcgcuaucuacaauguagA..........   1       1    OV2
    ...................................................uugggcgcuaucuacaa................   1       0    OV2
    ...................................................uugggcgcuaucuacaaug..............   8       0    OV2
    ...................................................uugggcgcuUucuacaaugu.............   2       1    OV2
    ...................................................uugggcgcuaucuacaaugu.............   1       0    OV2
    ...................................................uugggcgcuaucuacaauguag...........   23      0    OV2
    ...................................................uugggcgcuUucuacaauguag...........   7       1    OV2

    ........cacauuguagaaagggucaaaaug....................................................   2       0    TE1
    .........acauuguagaaagggucaaaa......................................................   1       0    TE1
    .........acauuguagaaagggucaaaau.....................................................   3       0    TE1
    .........acauuguagaaagggucaaaaug....................................................   17      0    TE1
    .........acauuguagaaagggucaaaauga...................................................   2       0    TE1
    ...........auuguagaaagggucaaaauga...................................................   2       0    TE1
    ............uuguagaaagggucaaaaugaua.................................................   2       0    TE1
    ..................................................auugggcgcuUucuacaaug..............   2       1    TE1
    ..................................................auugggcgcuUucuacaaugu.............   1       1    TE1
    ..................................................auugggcgcuUucuacaauguag...........   2       1    TE1
    ...................................................uugggcgcuUucuacaa................   17      1    TE1
    ...................................................uugggcgcuUucuacaaug..............   28      1    TE1
    ...................................................uugggcgcuaucuacaaug..............   5       0    TE1
    ...................................................uugggcgcuUucuacaaugu.............   6       1    TE1
    ...................................................uugggcgcuaucuacaaugu.............   2       0    TE1
    ...................................................uugggcgcuUucuacaaugua............   1       1    TE1
    ...................................................uugggcgcuaucuacaauguag...........   23      0    TE1
    ...................................................uugggcgcuUucuacaauguag...........   37      1    TE1
    ...................................................uugggcAcuaucuacaauguag...........   1       1    TE1
    ...................................................uugggcgcuUucuacaauguagu..........   2       1    TE1
```

```
         aga-mir-10380-star
                                                   aga-mir-10380
caauguaccacauuguagaaagggucaaaaugauauaauuucugcuuuucauugggcgcuaucuacaauguaguaucuugcauu

...................................................uugggcgcuaucuacaauguagu..........    1    0    TE1

.........acauuguagaaagggucaaaau.....................................................    3    0    MF2
.........acauuguagaaagggucaaaaug....................................................    6    0    MF2
.........acauuguagaaagggucaaaauga...................................................    3    0    MF2
...........auuguagaaagggucaaaauga...................................................    1    0    MF2
............uuguagaaagggucaaaaugau..................................................    1    0    MF2
..................................................auugggcgcuaucuacaauguag...........    6    0    MF2
...................................................uugggcgcuUucuacaa................    2    1    MF2
...................................................uugggcgcuUucuacaaug..............    3    1    MF2
...................................................uugggcgcuaucuacaaug..............    1    0    MF2
...................................................uugggcgcuUucuacaaugu.............    1    1    MF2
...................................................uugggcgcuaucuacaauguag...........    8    0    MF2
...................................................uuAggcgcuaucuacaauguag...........    1    1    MF2
...................................................uugggcgcuUucuacaauguag...........    8    1    MF2

.........acauuguagaaagggucaaaaug....................................................    2    0    OV1
..................................................auugggcgcuaucuacaaug..............    4    0    OV1
..................................................auugggcgcuaucuacaaugu.............    2    0    OV1
..................................................auugggcgcuaucuacaauguag...........    17   0    OV1
..................................................auugggcgcuUucuacaauguag...........    1    1    OV1
...................................................uugggcgcuaucuacaaug..............    4    0    OV1
...................................................uugggcgcuUucuacaaug..............    3    1    OV1
...................................................uugggcgcuaucuacaaugu.............    3    0    OV1
...................................................uugggcgcuauGuacaauguag...........    1    1    OV1
...................................................uugggcgcuaucuacaauguag...........    51   0    OV1
...................................................uugggcgcuUucuacaauguag...........    8    1    OV1
...................................................uugggcgcuaucuacaauguaU...........    1    1    OV1
...................................................uugggcgcuaucuacaauguagA..........    1    1    OV1

.........acauuguagaaagggucaaaaug....................................................    2    0    MF1
.........acauuguagaaagggucaaaauga...................................................    1    0    MF1
...........auuguagaaagggucaaaauga...................................................    1    0    MF1
...................................................uugggcgcuUucuacaauguag...........    2    1    MF1

.........acauuguagaaagggucaaaau.....................................................    1    0    BF2
..................................................auugggcgcuaucuacaaug..............    1    0    BF2
..................................................auugggcgcuaucuacaauguag...........    2    0    BF2
..................................................auugggcgcuaucuacaauguagA..........    1    1    BF2
...................................................uugggcgcuaucuacaaug..............    3    0    BF2
...................................................uugggcgcuUucuacaaug..............    1    1    BF2
...................................................uugggcgcuaucuacaaugua............    1    0    BF2
...................................................uugggcgcuaucuacaauguag...........    22   0    BF2
...................................................uugggcgcuaucuacaauguaA...........    1    1    BF2
...................................................uugggcgcuUucuacaauguag...........    3    1    BF2
...................................................uugggcgcuaucuacaauguagu..........    1    0    BF2

..................................................auugggcgcuaucuacaaug..............    1    0    BF1
..................................................auugggcgcuaucuacaauguag...........    4    0    BF1
...................................................uugggcgcuUucuacaaugu.............    1    1    BF1
...................................................uugggcgcuaucuacaauguag...........    18   0    BF1
...................................................uugggcgcuUucuacaauguag...........    1    1    BF1
...................................................uugggcgcuaucuacaauguagA..........    2    1    BF1

...................................................uugggcgcuaucuacaauguag...........    1    0    MW2

.........acauuguagaaaggguc..........................................................    2    0    TE2
.........acauuguagaaagggucaaaau.....................................................    2    0    TE2
.........acauuguagaaagggucaaaaug....................................................    10   0    TE2
.........acauuguagaaagggucaaaaAg....................................................    1    1    TE2
.........acauuguagaaagggucaaaauga...................................................    4    0    TE2
...........auuguagaaagggucaaaaugau..................................................    1    0    TE2
............uuguagaaagggucaaaaugau..................................................    2    0    TE2
..................................................auugggcgcuUucuacaa................    1    1    TE2
..................................................auugggcgcuUucuacaaug..............    7    1    TE2
..................................................auugggcgcuaucuacaaug..............    4    0    TE2
..................................................auugggcgcuUucuacaaugu.............    1    1    TE2
..................................................auugggcgcuaucuacaaugu.............    1    0    TE2
..................................................auugggcgcuaucuacaauguag...........    4    0    TE2
```

```
         aga-mir-10380-star
                                                   aga-mir-10380
caauguaccacauuguagaaagggucaaaaugauauaauuucugcuuuucauugggcgcuaucuacaauguaguaucuugcauu

...................................................uugggcgcuUucuacaa................    35    1    TE2
...................................................uugggcgcuaucuacaa................    1     0    TE2
...................................................uugggcgcuUucuacaaug..............    87    1    TE2
...................................................uugggcgcuaucuacaaug..............    8     0    TE2
...................................................uugggcgcuUucuacaaugu.............    25    1    TE2
...................................................uugggcgcuaucuacaaugu.............    3     0    TE2
...................................................uugggcgcuUucuacaaugua............    2     1    TE2
...................................................uugggcgcuUucuacaauguag...........    98    1    TE2
...................................................uugggcgcuaucuacaauguag...........    71    0    TE2
...................................................uugggcgcuUucuacaauguagu..........    2     1    TE2
```

miRBase precursor : aga-mir-10381
Total read count : 801
aga-mir-10381 read count : 729
aga-mir-10381-star read count : 72
remaining reads : 0

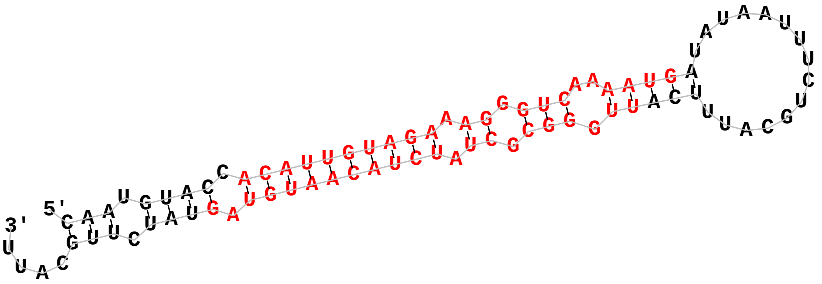

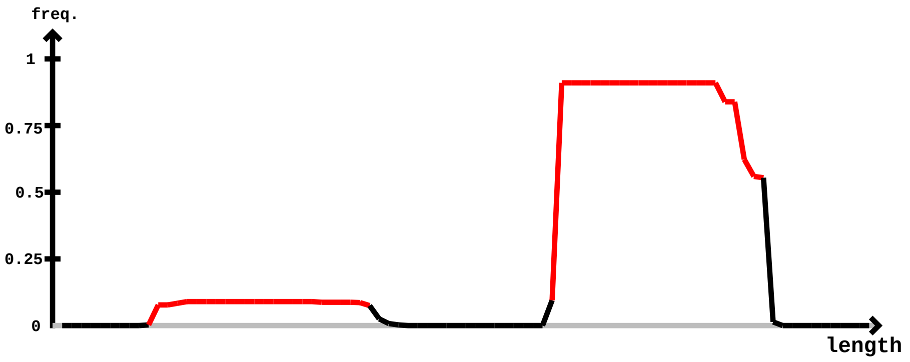


```
                                                 aga-mir-10381
          aga-mir-10381-star
5'- caauguaccacauuguagaaagggucaaaaugauauaauuucugcauuucauugggcgcuaucuacaauguaguaucuugcauu  -3'   exp
    (((.((((.((((((((((.((.(((..(((((...............))))).))).)).)))))))))).)))).)))....   reads   mm   sample
    ...................................................uugggcgcuaucuacaauguag...........   3       0    FF2

    ..................................................auugggcgcuaucuacaaug..............   3       0    OV2
    ..................................................auugggcgcuaucuacaaugu.............   2       0    OV2
    ..................................................auugggcgcuaucuacaauguag...........   7       0    OV2
    ..................................................auugggcgcuUucuacaauguag...........   1       1    OV2
    ..................................................auugggcgcuaucuacaauguagA..........   1       1    OV2
    ...................................................uugggcgcuaucuacaa................   1       0    OV2
    ...................................................uugggcgcuaucuacaaug..............   8       0    OV2
    ...................................................uugggcgcuUucuacaaugu.............   2       1    OV2
    ...................................................uugggcgcuaucuacaaugu.............   1       0    OV2
    ...................................................uugggcgcuaucuacaauguag...........   23      0    OV2
    ...................................................uugggcgcuUucuacaauguag...........   7       1    OV2

    ........cacauuguagaaagggucaaaaug....................................................   2       0    TE1
    .........acauuguagaaagggucaaaa......................................................   1       0    TE1
    .........acauuguagaaagggucaaaau.....................................................   3       0    TE1
    .........acauuguagaaagggucaaaaug....................................................   17      0    TE1
    .........acauuguagaaagggucaaaauga...................................................   2       0    TE1
    ...........auuguagaaagggucaaaauga...................................................   2       0    TE1
    ............uuguagaaagggucaaaaugaua.................................................   2       0    TE1
    ..................................................auugggcgcuUucuacaaug..............   2       1    TE1
    ..................................................auugggcgcuUucuacaaugu.............   1       1    TE1
    ..................................................auugggcgcuUucuacaauguag...........   2       1    TE1
    ...................................................uugggcgcuUucuacaa................   17      1    TE1
    ...................................................uugggcgcuUucuacaaug..............   28      1    TE1
    ...................................................uugggcgcuaucuacaaug..............   5       0    TE1
    ...................................................uugggcgcuUucuacaaugu.............   6       1    TE1
    ...................................................uugggcgcuaucuacaaugu.............   2       0    TE1
    ...................................................uugggcgcuUucuacaaugua............   1       1    TE1
    ...................................................uugggcgcuaucuacaauguag...........   23      0    TE1
    ...................................................uugggcgcuUucuacaauguag...........   37      1    TE1
    ...................................................uugggcAcuaucuacaauguag...........   1       1    TE1
    ...................................................uugggcgcuUucuacaauguagu..........   2       1    TE1
```

```
                                                  aga-mir-10381
         aga-mir-10381-star
caauguaccacauuguagaaagggucaaaaugauauaauuucugcauuucauugggcgcuaucuacaauguaguaucuugcauu

...................................................uugggcgcuaucuacaauguagu..........   1    0    TE1

.........acauuguagaaagggucaaaau.....................................................   3    0    MF2
.........acauuguagaaagggucaaaaug....................................................   6    0    MF2
.........acauuguagaaagggucaaaauga...................................................   3    0    MF2
...........auuguagaaagggucaaaauga...................................................   1    0    MF2
............uuguagaaagggucaaaaugau..................................................   1    0    MF2
..................................................auugggcgcuaucuacaauguag...........   6    0    MF2
...................................................uugggcgcuUucuacaa................   2    1    MF2
...................................................uugggcgcuUucuacaaug..............   3    1    MF2
...................................................uugggcgcuaucuacaaug..............   1    0    MF2
...................................................uugggcgcuUucuacaaugu.............   1    1    MF2
...................................................uugggcgcuaucuacaauguag...........   8    0    MF2
...................................................uuAggcgcuaucuacaauguag...........   1    1    MF2
...................................................uugggcgcuUucuacaauguag...........   8    1    MF2

.........acauuguagaaagggucaaaaug....................................................   2    0    OV1
..................................................auugggcgcuaucuacaaug..............   4    0    OV1
..................................................auugggcgcuaucuacaaugu.............   2    0    OV1
..................................................auugggcgcuaucuacaauguag...........   17   0    OV1
..................................................auugggcgcuUucuacaauguag...........   1    1    OV1
...................................................uugggcgcuaucuacaaug..............   4    0    OV1
...................................................uugggcgcuUucuacaaug..............   3    1    OV1
...................................................uugggcgcuaucuacaaugu.............   3    0    OV1
...................................................uugggcgcuauGuacaauguag...........   1    1    OV1
...................................................uugggcgcuaucuacaauguag...........   51   0    OV1
...................................................uugggcgcuUucuacaauguag...........   8    1    OV1
...................................................uugggcgcuaucuacaauguaU...........   1    1    OV1
...................................................uugggcgcuaucuacaauguagA..........   1    1    OV1

.........acauuguagaaagggucaaaaug....................................................   2    0    MF1
.........acauuguagaaagggucaaaauga...................................................   1    0    MF1
...........auuguagaaagggucaaaauga...................................................   1    0    MF1
...................................................uugggcgcuUucuacaauguag...........   2    1    MF1

.........acauuguagaaagggucaaaau.....................................................   1    0    BF2
..................................................auugggcgcuaucuacaaug..............   1    0    BF2
..................................................auugggcgcuaucuacaauguag...........   2    0    BF2
..................................................auugggcgcuaucuacaauguagA..........   1    1    BF2
...................................................uugggcgcuaucuacaaug..............   3    0    BF2
...................................................uugggcgcuUucuacaaug..............   1    1    BF2
...................................................uugggcgcuaucuacaaugua............   1    0    BF2
...................................................uugggcgcuaucuacaauguag...........   22   0    BF2
...................................................uugggcgcuaucuacaauguaA...........   1    1    BF2
...................................................uugggcgcuUucuacaauguag...........   3    1    BF2
...................................................uugggcgcuaucuacaauguagu..........   1    0    BF2

..................................................auugggcgcuaucuacaaug..............   1    0    BF1
..................................................auugggcgcuaucuacaauguag...........   4    0    BF1
...................................................uugggcgcuUucuacaaugu.............   1    1    BF1
...................................................uugggcgcuaucuacaauguag...........   18   0    BF1
...................................................uugggcgcuUucuacaauguag...........   1    1    BF1
...................................................uugggcgcuaucuacaauguagA..........   2    1    BF1

...................................................uugggcgcuaucuacaauguag...........   1    0    MW2

.........acauuguagaaaggguc..........................................................   2    0    TE2
.........acauuguagaaagggucaaaau.....................................................   2    0    TE2
.........acauuguagaaagggucaaaaug....................................................   10   0    TE2
.........acauuguagaaagggucaaaaAg....................................................   1    1    TE2
.........acauuguagaaagggucaaaauga...................................................   4    0    TE2
...........auuguagaaagggucaaaaugau..................................................   1    0    TE2
............uuguagaaagggucaaaaugau..................................................   2    0    TE2
..................................................auugggcgcuUucuacaa................   1    1    TE2
..................................................auugggcgcuUucuacaaug..............   7    1    TE2
..................................................auugggcgcuaucuacaaug..............   4    0    TE2
..................................................auugggcgcuUucuacaaugu.............   1    1    TE2
..................................................auugggcgcuaucuacaaugu.............   1    0    TE2
..................................................auugggcgcuaucuacaauguag...........   4    0    TE2
```

aga-mir-10381

aga-mir-10381-star

caauguaccacauuguagaaagggucaaaaugauauaauuucugcauuucauugggcgcuaucuacaauguaguaucuugcauu

....uugggcgcuUucuacaa................	35	1	TE2
....uugggcgcuaucuacaa................	1	0	TE2
....uugggcgcuUucuacaaug..............	87	1	TE2
....uugggcgcuaucuacaaug..............	8	0	TE2
....uugggcgcuUucuacaaugu.............	25	1	TE2
....uugggcgcuaucuacaaugu.............	3	0	TE2
....uugggcgcuUucuacaaugua............	2	1	TE2
....uugggcgcuUucuacaauguag...........	98	1	TE2
....uugggcgcuaucuacaauguag...........	71	0	TE2
....uugggcgcuUucuacaauguagu..........	2	1	TE2

miRBase precursor : aga-mir-252
Total read count : 9058
aga-mir-252 read count : 8947
aga-mir-252-star read count: 46
remaining reads : 65

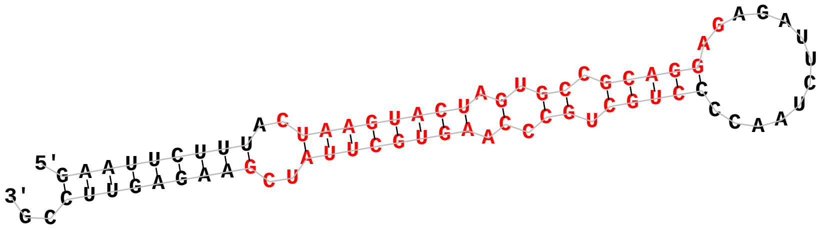

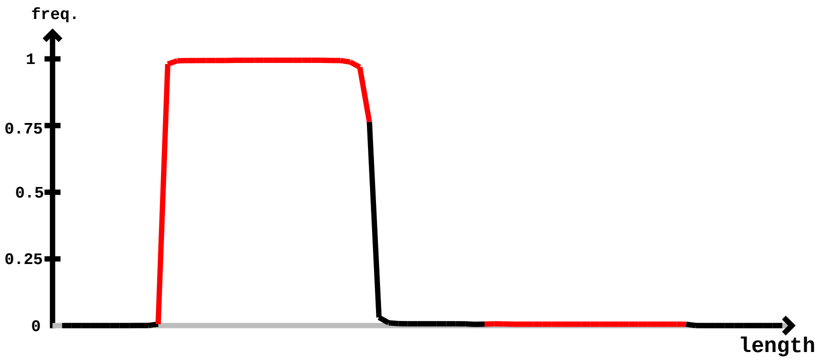


```
                                                aga-mir-252-star
              aga-mir-252
5'- gaauucuuuacuaaguacuagugccgcaggagagauucuaaccccugcugcccaagugcuuaucgaagaguuccg   -3'    exp
    (((((((((..((((((((.(.((.(((((.............))))).)).).))))))))..)))))))))..   reads      mm         sample
    ..........cuaaguacuagugccgcagg.............................................   3          0          OV2
    ..........cuaaguacuagugccgcagga............................................   13         0          OV2
    ..........cuaaguacuagugccgcaggag...........................................   52         0          OV2
    ..........cuaaguacuagugccgcaggaU...........................................   5          1          OV2
    ...........uaaguacuagugccgcaggag...........................................   1          0          OV2

    ..........cuaaguacuagugccgcagg.............................................   2          0          TE1
    ..........cuaaguacuagugccUcagg.............................................   1          1          TE1
    ..........cuaaguacuagugccgcagga............................................   81         0          TE1
    ..........cuUaguacuagugccgcagga............................................   1          1          TE1
    ..........cuaaguacuagugccgGagga............................................   1          1          TE1
    ..........cuaaguacuagGgccgcaggag...........................................   1          1          TE1
    ..........cuaaguaGuagugccgcaggag...........................................   1          1          TE1
    ..........cuaaguacuagugccgcaggaU...........................................   47         1          TE1
    ..........cuaagCacuagugccgcaggag...........................................   1          1          TE1
    ..........cuaaguacuagugccgcaggag...........................................   232        0          TE1
    ..........cuaaguacuagugccgcaggaA...........................................   4          1          TE1
    ..........cuaaguacuagugccgcaggaC...........................................   1          1          TE1
    ..........cuaaguacuagugccgcaggaga..........................................   3          0          TE1
    ..........cuaaguacuagugccgcaggagagauucuaac.................................   1          0          TE1
    ...........uaaguacuagugccgcaggag...........................................   2          0          TE1
    ...........uaaguacuagugccgcaggaga..........................................   1          0          TE1

    .........acuaaguacuagugccgcaggaU...........................................   1          1          FF2
    ..........cuaaguacuagugccgcag..............................................   4          0          FF2
    ..........cuaaguacuagugccgcaU..............................................   1          1          FF2
    ..........cuaaguacuagugccgcagg.............................................   7          0          FF2
    ..........cuaaguacuagugccgcagga............................................   73         0          FF2
    ..........cuaaguacuagugcUgcagga............................................   1          1          FF2
    ..........cuaaguacuagugccgcagAag...........................................   1          1          FF2
    ..........cuaaguacuagugccgcaggaU...........................................   87         1          FF2
    ..........cuaaguacuagugccgcaggaC...........................................   12         1          FF2
    ..........cuaaguacuagugccgcaggaA...........................................   6          1          FF2
    ..........cuaaguacuagugccgcaggag...........................................   306        0          FF2
```

```
                                            aga-mir-252-star
          aga-mir-252
gaauucuuuacuaaguacuagugccgcaggagagauucuaaccccugcugcccaagugcuuaucgaagaguuccg

..........cuaaguacuaguAccgcaggag...........................................   1     1     FF2
..........cuaaguacuagugccCcaggag...........................................   1     1     FF2
..........cuaaguacuagugccgcaggGg...........................................   1     1     FF2
..........cuaaguacuagGgccgcaggag...........................................   1     1     FF2
..........cuaaguacuagCgccgcaggag...........................................   1     1     FF2
..........cuaaguacuagugccgcaggagU..........................................   2     1     FF2
..........cuaaguacuagugccgcaggaga..........................................   7     0     FF2
..........cuaaguacuagugccgcaggagaU.........................................   1     1     FF2
..........cuaaguacuagugccgcaggagagauucuaa..................................   1     0     FF2
..........cuaaguacuagugccgcaggagagauucuaaA.................................   2     1     FF2
..........cuaaguacuagugccgcaggagagauucuaaccc...............................   1     0     FF2
...........uaaguacuagugccgcaggag...........................................   3     0     FF2
...........uaaguacuagugccgcaggaU...........................................   2     1     FF2
...........uaaguacuagugccgcaggagagauucuaacU................................   1     1     FF2
...........................................ccugcugcccaagugcuuaucg..........   2     0     FF2
............................................cugcugcccaagugcuuaucg..........   3     0     FF2

.......uuacuaaguacuagugccgcaggaU...........................................   2     1     MF2
.........acuaaguacuagugccgcag..............................................   1     0     MF2
.........acuaaguacuagugccgcagg.............................................   1     0     MF2
.........acuaaguacuagugccgcagga............................................   5     0     MF2
.........acuaaguacuagugccgcaggaU...........................................   17    1     MF2
.........acuaaguacuagugccgcaggag...........................................   1     0     MF2
..........cuaaguacuagugccgc................................................   3     0     MF2
..........cuaaguacuagugccgca...............................................   1     0     MF2
..........cuaaguacuagugccgcag..............................................   18    0     MF2
..........Uuaaguacuagugccgcag..............................................   1     1     MF2
..........cuaaguacuagugccgcagg.............................................   58    0     MF2
..........cuaaguacuagugccgcagU.............................................   1     1     MF2
..........cuaaguacuagugccCcagg.............................................   1     1     MF2
..........cuaaguacuagugccgcagga............................................   567   0     MF2
..........cuaaguacuagugccgcaggC............................................   1     1     MF2
..........cuaaguacuagugccgcaggU............................................   3     1     MF2
..........cuaaguacuagugccgcagAa............................................   1     1     MF2
..........cuaaguacuagugccgGagga............................................   1     1     MF2
..........cuaaguacuaCugccgcaggag...........................................   1     1     MF2
..........cuaaguacuagugccgcCggag...........................................   1     1     MF2
..........cuaaguacuagAgccgcaggag...........................................   3     1     MF2
..........cuaaguacuaguAccgcaggag...........................................   1     1     MF2
..........Uuaaguacuagugccgcaggag...........................................   1     1     MF2
..........cuaaguacuagugccgcaggaU...........................................   634   1     MF2
..........cuaaguacuagGgccgcaggag...........................................   3     1     MF2
..........cuaagGacuagugccgcaggag...........................................   1     1     MF2
..........cuaaguacuagugccgcaggaA...........................................   33    1     MF2
..........cCaaguacuagugccgcaggag...........................................   2     1     MF2
..........cuaaguacuagugccgcaggaC...........................................   118   1     MF2
..........cuaagCacuagugccgcaggag...........................................   1     1     MF2
..........cuaaAuacuagugccgcaggag...........................................   2     1     MF2
..........cuaaguacuaUugccgcaggag...........................................   2     1     MF2
..........cuaaguacuagugccgcUggag...........................................   1     1     MF2
..........cuaaguacuagCgccgcaggag...........................................   2     1     MF2
..........cuaaguacuagugccgcaggag...........................................   2133  0     MF2
..........cuaaguacuagugccgcagAag...........................................   1     1     MF2
..........cuGaguacuagugccgcaggag...........................................   1     1     MF2
..........cuaaguacuagugccgcaggagU..........................................   21    1     MF2
..........cuaaguacuagugccgcaggaUa..........................................   3     1     MF2
..........cuaaguacuagugccgcaggaAa..........................................   3     1     MF2
..........cuaaguacuagugccgcaggagC..........................................   7     1     MF2
..........cuaaguacuagugccgcaggaga..........................................   61    0     MF2
..........cuaaguacuagugccgcaggagaA.........................................   11    1     MF2
..........cuaaguacuagugccgcaggagaU.........................................   3     1     MF2
..........cuaaguacuagugccgcaggagaAa........................................   2     1     MF2
..........cuaaguacuagugccgcaggagagU........................................   1     1     MF2
..........cuaaguacuagugccgcaggagagauucua...................................   1     0     MF2
..........cuaaguacuagugccgcaggagagauucuaac.................................   1     0     MF2
..........cuaaguacuagugccgcaggagagauucuaaA.................................   1     1     MF2
..........cuaaguacuagugccgcaggagagauucuaacc................................   4     0     MF2
..........cuaaguacuagugccgcaggagagauucuaacU................................   1     1     MF2
..........cuaaguacuagugccgcaggagagauucuaaccU...............................   6     1     MF2
```

```
                                          aga-mir-252-star
          aga-mir-252
gaauucuuuacuaaguacuagugccgcaggagagauucuaaccccugcugcccaagugcuuaucgaagaguuccg

..........cuaaguacuagugccgcaggagagauucuaaccc...............................     2       0       MF2
..........cuaaguacuagugccgcaggagagauucuaacccc..............................     3       0       MF2
..........cuaaguacuagugccgcaggagagauucuaaccccu.............................     1       0       MF2
...........uaaguacuagugccgcagg.............................................     1       0       MF2
...........uaaguacuagugccgcagga............................................     6       0       MF2
...........uaaguacuagugccgcaggag...........................................     16      0       MF2
...........uaaguacuagugccgcaggaU...........................................     9       1       MF2
...........uaaguacuagugccgcaggaC...........................................     1       1       MF2
...........uaaguacuagugccgcaggaga..........................................     10      0       MF2
...........uaaguacuagugccgcaggagagauucuaaU.................................     2       1       MF2
...........uaaguacuagugccgcaggagagauucuaac.................................     5       0       MF2
...........uaaguacuagugccgcaggagagauucuaacU................................     1       1       MF2
...........uaaguacuagugccgcaggagagauucuaaccccu.............................     1       0       MF2
............aaguacuagugccgcaggag...........................................     2       0       MF2
..............guacuagugccgcaggagagauucuaaccccu.............................     1       0       MF2
.................cuagugccgcaggagagauucuaaccc...............................     3       0       MF2
.................cuagugccgcaggagagauucuaacccc..............................     1       0       MF2
.................cuagugccgcaggagagauucuaaccccu.............................     2       0       MF2
...................agugccgcaggagagauucuaaccc...............................     1       0       MF2
..............................agagauucuaaccccugcugcccaagugcuuaucga.........     1       0       MF2
..........................................cccugcugcccaagugcuuaucg..........     1       0       MF2
...........................................ccugcugcccaagugcuuaucg..........     8       0       MF2
............................................cugcugcccaagugcuuauc...........     1       0       MF2
............................................cugcugcccaagugcuuaucg..........     11      0       MF2

.........acuaaguacuagugccgcagg.............................................     1       0       FW2
..........cuaaguacuagugccgc................................................     2       0       FW2
..........cuaaguacuagugccgcag..............................................     3       0       FW2
..........cuaaguacuagugccgcagg.............................................     13      0       FW2
..........cuaaguacuagugccgcaggG............................................     1       1       FW2
..........cuaaguacuagugccgcagga............................................     149     0       FW2
..........cuaaguacuagugccgcaggaU...........................................     19      1       FW2
..........cuaagCacuagugccgcaggag...........................................     1       1       FW2
..........cuaaguacuagugccgcaggag...........................................     186     0       FW2
..........cuaaguacuagugccgcaggaC...........................................     4       1       FW2
..........cuaaguacuagugccgcaggaA...........................................     3       1       FW2
..........cuaaguacuagugccgcaggaga..........................................     1       0       FW2
..........cuaaguacuagugccgcaggagagauucuaacc................................     2       0       FW2
...........uaaguacuagugccgcagga............................................     2       0       FW2
...........uaaguacuagugccgcaggag...........................................     3       0       FW2
...........uaaguacuagugccgcaggaga..........................................     1       0       FW2
...........uaaguacuagugccgcaggagagauucuaac.................................     1       0       FW2
............aaguacuagugccgcaggag...........................................     1       0       FW2
.............aguacuagugccgcaggag...........................................     1       0       FW2
...........................................ccugcugcccaagugcuuaucg..........     1       0       FW2
............................................cugcugcccaagugcuuaucg..........     1       0       FW2

..........cuaaguacuagugccgcagg.............................................     1       0       OV1
..........cuaaguacuagugccgcagga............................................     17      0       OV1
..........cuaaguacuagugccgcaggaC...........................................     1       1       OV1
..........cuaaguacuagugccgUaggag...........................................     1       1       OV1
..........cuaaguacuagugccgcaggag...........................................     59      0       OV1
..........cuaaguacuagugccgcaggaU...........................................     7       1       OV1
..........cuaaguacuagugccgcaggagU..........................................     1       1       OV1
..........cuaaguacuagugccgcaggagagauucuaaccccu.............................     2       0       OV1
...........uaaguacuagugccgcaggag...........................................     3       0       OV1
...........uaaguacuagugccgcaggaga..........................................     1       0       OV1
............aaguacuagugccgcaggag...........................................     1       0       OV1

.......uuacuaaguacuagugccgcag..............................................     1       0       FF1
.........acuaaguacuagugccgcagg.............................................     1       0       FF1
.........acuaaguacuagugccgcagga............................................     1       0       FF1
.........acuaaguacuagugccgcaggaU...........................................     3       1       FF1
..........cuaaguacuagugccgcag..............................................     7       0       FF1
..........cuaaUuacuagugccgcagg.............................................     1       1       FF1
..........cuaagGacuagugccgcagg.............................................     1       1       FF1
..........cuaGguacuagugccgcagg.............................................     2       1       FF1
..........cuaaguacuagugccgcagg.............................................     32      0       FF1
..........cuaaguacuagugccgcagga............................................     351     0       FF1
```

```
                                        aga-mir-252-star
          aga-mir-252
gaauucuuuacuaaguacuagugccgcaggagagauucuaaccccugcugcccaagugcuuaucgaagaguuccg

..........cuaUguacuagugccgcagga............................................    1     1     FF1
..........cuaaguacAagugccgcagga............................................    2     1     FF1
..........cuaaguacuagugccgcaggG............................................    2     1     FF1
..........cuaaguacuagugccgcaggU............................................    1     1     FF1
..........cuaGguacuagugccgcagga............................................    6     1     FF1
..........cuaaUuacuagugccgcagga............................................    8     1     FF1
..........cuaaguacuagugccgAagga............................................    1     1     FF1
..........cuaaguacuagugccgcaggC............................................    4     1     FF1
..........cuaaguacuagugccgcaggaU...........................................    105   1     FF1
..........cuaagCacuagugccgcaggag...........................................    1     1     FF1
..........Guaaguacuagugccgcaggag...........................................    1     1     FF1
..........cuaaguacGagugccgcaggag...........................................    2     1     FF1
..........cuaaguacuagugccgcaggCg...........................................    7     1     FF1
..........cuaaUuacuagugccgcaggag...........................................    18    1     FF1
..........cuaaguacuagugccgcaggaC...........................................    23    1     FF1
..........cGaaguacuagugccgcaggag...........................................    5     1     FF1
..........cuaaguacuagugccUcaggag...........................................    1     1     FF1
..........cuaaguacuagugccgcGggag...........................................    1     1     FF1
..........cuaGguacuagugccgcaggag...........................................    12    1     FF1
..........cuaaguacuagugccgcaggag...........................................    721   0     FF1
..........cuaaguacuagugccgUaggag...........................................    2     1     FF1
..........cuaaguacuagCgccgcaggag...........................................    1     1     FF1
..........cuaaguacuagugccgAaggag...........................................    2     1     FF1
..........cuaaguacuagugccgcaggaA...........................................    10    1     FF1
..........cuaaguacuagugccgcaggaCa..........................................    1     1     FF1
..........cuaaguacuagugccgcaggagC..........................................    2     1     FF1
..........cuaaguacuagugccgcaggaUa..........................................    1     1     FF1
..........cuaaguacuagugccgcaggagU..........................................    4     1     FF1
..........cuaaguacuagugccgcaggaga..........................................    5     0     FF1
..........cuaaUuacuagugccgcaggaga..........................................    1     1     FF1
..........cuaaguacuagugccgcaggagaA.........................................    1     1     FF1
..........cuaaguacuagugccgcaggagaga........................................    1     0     FF1
..........cuaaguacuagugccgcaggagaAa........................................    1     1     FF1
..........cuaaguacuagugccgcaggagagauucuaac.................................    1     0     FF1
..........cuaGguacuagugccgcaggagagauucuaac.................................    1     1     FF1
..........cuaaguacuagugccgcaggagagauucuaacc................................    2     0     FF1
..........cuaaguacuagugccgcaggagagauucuaaccU...............................    1     1     FF1
...........uaaguacuagugccgcagga............................................    1     0     FF1
...........uaaUuacuagugccgcagga............................................    1     1     FF1
...........uaaguacuagugccgcaggaU...........................................    1     1     FF1
...........uaaguacuagugccgcaggag...........................................    3     0     FF1
...........................................ccugcugcccaagugcuuaucg..........    1     0     FF1
............................................cugcugcccaagugcuuaucg..........    5     0     FF1

.........acuaaguacuagugccgcaggaU...........................................    3     1     MF1
..........cuaaguacuagugccgcag..............................................    10    0     MF1
..........cuaaguacuagugccgcagg.............................................    17    0     MF1
..........cuaaguacuagGgccgcagga............................................    1     1     MF1
..........cuaaguacuagugccgcagga............................................    113   0     MF1
..........cuaagCacuagugccgcagga............................................    1     1     MF1
..........cuaaguacuagugUcgcagga............................................    2     1     MF1
..........cuaaguacuagugccgcaggaC...........................................    14    1     MF1
..........cuaaguacuagugccgcaggag...........................................    322   0     MF1
..........cuaaguacuagGgccgcaggag...........................................    1     1     MF1
..........cuaaguacuagugccgcaggaA...........................................    10    1     MF1
..........cuaaguacuagugccgcaggaU...........................................    122   1     MF1
..........cuaaguacuagugccgAaggag...........................................    1     1     MF1
..........cuaaguacuagugccgcaggaga..........................................    11    0     MF1
..........cuaaguacuagugccgcaggagC..........................................    1     1     MF1
..........cuaaguacuagugccgcaggaAa..........................................    1     1     MF1
..........cuaaguacuagugccgcaggaUa..........................................    1     1     MF1
..........cuaaguacuagugccgcaggagU..........................................    7     1     MF1
..........cuaaguacuagugccgcaggagaA.........................................    2     1     MF1
..........cuaaguacuagugccgcaggagaU.........................................    3     1     MF1
..........cuaaguacuagugccgcaggagagauucuaa..................................    1     0     MF1
...........uaaguacuagugccgcagga............................................    1     0     MF1
...........uaaguacuagugccgcaggaU...........................................    4     1     MF1
...........uaaguacuagugccgcaggaA...........................................    1     1     MF1
...........uaaguacuagugccgcaggag...........................................    4     0     MF1
```

```
                                          aga-mir-252-star
          aga-mir-252
gaauucuuuacuaaguacuagugccgcaggagagauucuaacccugcugcccaagugcuuaucgaagaguuccg

...........uaaguacuagugccgcaggaga..........................................     2     0     MF1
...........uaaguacuagugccgcaggagagauucuaac.................................     2     0     MF1
..........................................cccugcugcccaagugcuuaucg..........     1     0     MF1
...........................................ccugcugcccaagugcuuaucg..........     2     0     MF1
............................................cugcugcccaagugcuuaucg..........     1     0     MF1

..........cuaaguacuagugccgcagga............................................     3     0     BF2
..........cuaaguacuagugccgcaggag...........................................     5     0     BF2

.........acuaaguacuagugccgcaggaU...........................................     1     1     BF1
..........cuaaguacuagugccgcagga............................................     8     0     BF1
..........cuaaguacCagugccgcaggag...........................................     1     1     BF1
..........cuaaguacuagugccgcaggag...........................................     21    0     BF1
..........cuaaguacuagugccgcaggaU...........................................     2     1     BF1

.........acuaaguacuagugccgcagga............................................     1     0     MW1
.........Gcuaaguacuagugccgcaggag...........................................     1     1     MW1
.........acuaaguacuagugccgcaggaU...........................................     1     1     MW1
..........cuaaguacuagugccgc................................................     2     0     MW1
..........cuaaguacuagugccgcag..............................................     1     0     MW1
..........cuaaguacuagugccgcagg.............................................     11    0     MW1
..........cuaaguacuagugccgcagga............................................     114   0     MW1
..........cuaaguacuagugccAcaggag...........................................     1     1     MW1
..........cCaaguacuagugccgcaggag...........................................     1     1     MW1
..........cuaaguacCagugccgcaggag...........................................     1     1     MW1
..........cuaaguacuagugccgAaggag...........................................     1     1     MW1
..........cuaaguacuaUugccgcaggag...........................................     1     1     MW1
..........cuaaguacuagugccgcaggaA...........................................     2     1     MW1
..........cuaaguacuagugccgcaggag...........................................     179   0     MW1
..........cuaagCacuagugccgcaggag...........................................     1     1     MW1
..........cuaaguacuagugccgcaggaC...........................................     5     1     MW1
..........cuaaguacuagugccgcaggaU...........................................     17    1     MW1
..........cuaaguacuagugccgcaggagU..........................................     3     1     MW1
...........uaaguacuagugccgcagga............................................     2     0     MW1
...........uaaguacuagugccgcaggaga..........................................     3     0     MW1
............aaguacuagugccgcaggag...........................................     1     0     MW1
...........................................ccugcugcccaagugcuuauc...........     1     0     MW1
............................................cugcugcccaagugcuuaucg..........     2     0     MW1

.........acuaaguacuagugccgcaggaU...........................................     1     1     FW1
..........cuaaguacuagugccgcagg.............................................     2     0     FW1
..........cuaaguacuagugccgcagga............................................     61    0     FW1
..........cuaaguacuagugccgcaggag...........................................     100   0     FW1
..........cuaaguacuGgugccgcaggag...........................................     1     1     FW1
..........cuaaguacuagugccgcaggaU...........................................     14    1     FW1
..........cCaaguacuagugccgcaggag...........................................     1     1     FW1
..........cuaaguacuagugccgcaggaga..........................................     1     0     FW1
..........cuaaguacuagugccgcaggagagauucuaaccccu.............................     1     0     FW1
...........uaaguacuagugccgcagga............................................     2     0     FW1
...........uaaguacuagugccgcaggaU...........................................     1     1     FW1
...........uaaguacuagugccgcaggag...........................................     1     0     FW1
.................cuagugccgcaggagagauucuaacccc..............................     1     0     FW1

.........acuaaguacuagugccgcagga............................................     2     0     MW2
..........cuaaguacuagugccgcag..............................................     1     0     MW2
..........cuaaguacuagugccgcagga............................................     50    0     MW2
..........cuaaguacuagugccgcaggaA...........................................     1     1     MW2
..........cuaaguacuagGgccgcaggag...........................................     1     1     MW2
..........cuaaguacuagugccgcaggaU...........................................     6     1     MW2
..........cuaaguacuagugccgcaggag...........................................     60    0     MW2
..........cuaaguacuagugccgcaggaga..........................................     2     0     MW2
...........uaaguacuagugccgcaggag...........................................     1     0     MW2
............aaguacuagugccgcaggag...........................................     1     0     MW2

.........acuaaguacuagugccgcagg.............................................     1     0     TE2
.........acuaaguacuagugccgcaggaU...........................................     3     1     TE2
..........cuaaguacuagugccgc................................................     1     0     TE2
..........cuaaguacuagugccgcag..............................................     2     0     TE2
..........cuaaguacuagugccgcagg.............................................     17    0     TE2
```

```
                                            aga-mir-252-star
          aga-mir-252
gaauucuuuacuaaguacuagugccgcaggagagauucuaaccccugcugcccaagugcuuaucgaagaguuccg

..........cuaaguacuagCgccgcagg.............................................    1      1      TE2
..........cuaaguacuagCgccgcagga............................................    1      1      TE2
..........cuaaguacuagugccgcagga............................................    196    0      TE2
..........cuaaguacuUgugccgcagga............................................    1      1      TE2
..........cuaaguacuagGgccgcagga............................................    1      1      TE2
..........cuaaguacuagugccgAagga............................................    1      1      TE2
..........cuaaguaUuagugccgcaggag...........................................    1      1      TE2
..........cuaGguacuagugccgcaggag...........................................    1      1      TE2
..........cuGaguacuagugccgcaggag...........................................    1      1      TE2
..........cuaaguacuagugccgcaggaC...........................................    5      1      TE2
..........cuaaguacuagugccgcaggaA...........................................    2      1      TE2
..........cuaaguacuagugccgUaggag...........................................    1      1      TE2
..........cuaaguacuagAgccgcaggag...........................................    1      1      TE2
..........cuaaguacuagugccgcaggag...........................................    553    0      TE2
..........cuaaguacuagugccgcaggaU...........................................    182    1      TE2
..........Uuaaguacuagugccgcaggag...........................................    1      1      TE2
..........cuaaguacuagugccgcaggaga..........................................    13     0      TE2
..........cuaaguacuagugccgcaggagaU.........................................    1      1      TE2
..........cuaaguacuagugccgcaggagaC.........................................    1      1      TE2
..........cuaaguacuagugccgcaggagaA.........................................    3      1      TE2
..........cuaaguacuagugccgcaggagagauucuaac.................................    1      0      TE2
..........cuaaguacuagugccgcaggagagauucuaaccU...............................    1      1      TE2
...........uaaguacuagugccgcaggag...........................................    4      0      TE2
...........uaaguacuagugccgcaggaU...........................................    2      1      TE2
...........uaaguacuagugccgcaggaga..........................................    1      0      TE2
...........uaaguacuagugccgcaggagagauucuaac.................................    1      0      TE2
..............guacuagugccgcaggag...........................................    1      0      TE2
...........................................ccugcugcccaagugcuuaucg..........    3      0      TE2
............................................cugcugcccaagugcuuaucg..........    2      0      TE2
```

miRBase precursor : aga-mir-2796
Total read count : 413
aga-mir-2796 read count : 409
aga-mir-2796-star read count : 4
remaining reads : 0

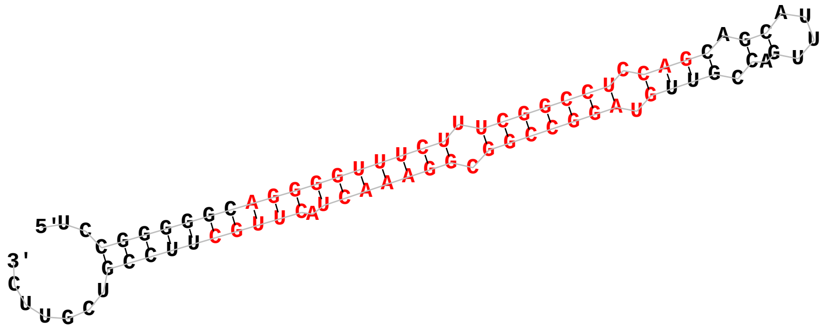

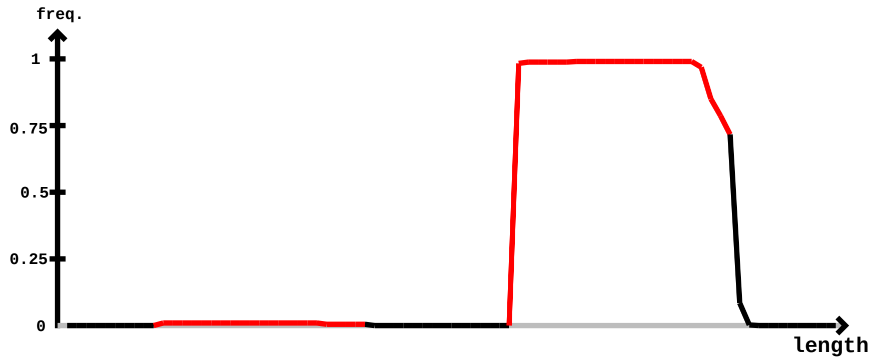

aga-mir-2796-star

aga-mir-2796

```
5'- uccggggcagggguuucuuucggccuccagcagcauuugaccguuguaggccggcggaaacuacuugcuuccgucguuc   -3'    exp
    ..((((((((((((((((.(((((((.((((.((....).).)))).))))))).))))))).)))))))))......     reads      mm         sample
    .............................................guaggccAgcggaaacuacuugc...........     1          1          TE1
    .............................................guaggccggcggaaacuacuugc...........     5          0          TE1

    .............................................guaggccggcggaaacuacu..............     1          0          FF2
    .............................................guaggccggcggaaacuacuu.............     1          0          FF2
    .............................................guaggccggcggaaacuacuugc...........     6          0          FF2
    .............................................guaggccggcggaaacuacuugcu..........     2          0          FF2

    .............................................guaggccggcggaaacuac...............     1          0          OV2
    .............................................guaggccggcggaaacuacu..............     4          0          OV2
    .............................................guaggccggcggaaacuacuu.............     3          0          OV2
    .............................................guaggccggcggaaacuacuug............     2          0          OV2
    .............................................guaggccggcggaaacuacuugU..........     1          1          OV2
    .............................................guaggccggcggaaacuacuugc...........     12         0          OV2
    .............................................guaggccggcggaaacuaUuugc...........     1          1          OV2
    .............................................guaggccggcggaaacuacuugcu..........     1          0          OV2

    .............................................guaggccggcggaaacuac...............     2          0          MF2
    .............................................guaggccggcggaaacuacu..............     15         0          MF2
    .............................................guaggccggcggaaacuacuu.............     5          0          MF2
    .............................................guaggccggcggaaacuacuug............     10         0          MF2
    .............................................guaggccggcggaaacuacuugc...........     44         0          MF2
    .............................................guaggccggcggaaacuacuugA..........     1          1          MF2
    .............................................guaggccggcggaaacuacuugcu..........     6          0          MF2
    .............................................guaggccggcggaaacuacuuUcu..........     1          1          MF2
    .............................................guaggccggcggaaacuacuugcA..........     2          1          MF2
    ..............................................uaggccggcggaaacuacuugc...........     1          0          MF2

    .............................................guaggccggcggaaacuacuu.............     1          0          FW2
    .............................................guaggccggcggaaacuacuug............     4          0          FW2
    .............................................guaggccggcggaaacuacuugc...........     30         0          FW2
    .............................................guaggccggcggaaacuacuugcA..........     2          1          FW2
    .............................................guaggccggcggaaacuacuugcu..........     3          0          FW2

    .............................................guaggccggcggaaacuacu..............     1          0          FF1
```

aga-mir-2796-star

aga-mir-2796

uccgggggcagggguuucuuucggccuccagcagcauuugaccguuguaggccggcggaaacuacuugcuuccgucguuc			
....guaggccggcggaaacuacuu.............	3	0	FF1
....guaggccggcggaaacuacuug............	2	0	FF1
....guaggccggcggaaacuacuugU...........	1	1	FF1
....guaUgccggcggaaacuacuugc...........	1	1	FF1
....guaggccggcggaaacuacuugc...........	15	0	FF1
....guaggccggcggaaacuacuugcu..........	1	0	FF1
....guaggccggcggaaacuac...............	5	0	OV1
....guaggccggcggaaacuacu..............	9	0	OV1
....guaggccggcggaaacuacuu.............	3	0	OV1
....guaggccggcggaaacuacuug............	3	0	OV1
....guaggccggcggaaacuacuugc...........	18	0	OV1
....guaggccggcggaaacuacuugcu..........	1	0	OV1
....guaggccggcggaaacuacu..............	5	0	MF1
....guaggccggcggaaacuacuu.............	3	0	MF1
....guaggccggcggaaacuacuug............	3	0	MF1
....guaggccggcgCaaacuacuugc...........	1	1	MF1
....guaggccggcggaaaUuacuugc...........	1	1	MF1
....guaggccggcggaaacuacuugc...........	9	0	MF1
....guaggccggcggaaacuacuugcu..........	2	0	MF1
....uaggccggcggaaacuacuu.............	1	0	MF1
....guaggccggcggaaacuacu..............	3	0	BF2
....guaggccggcggaaacuacuu.............	1	0	BF2
....guaggccggcggaaacuacuug............	1	0	BF2
....guaggccggcAgaaacuacuugc...........	1	1	BF2
....guaggccggcggaaacuacuugc...........	29	0	BF2
....guaggccggcggaaacuacuugcA..........	1	1	BF2
....guaggccggcggaaacuacu..............	1	0	BF1
....guaggccggcggaaacuacuugc...........	13	0	BF1
....guaggccggcggaaacuacuugU...........	1	1	BF1
....guaggccggcggaaacuacuugcAu.........	1	1	BF1
....guaggccggcggaaacuacu..............	1	0	FW1
....guaggccggcggaaacuacuu.............	4	0	FW1
....guaggccggcggaaacuacuugc...........	11	0	FW1
....guaggccggcggaaacuacuugcA..........	2	1	FW1
....guaggccggcggaaacuacuugcu..........	1	0	FW1
.........agggguuucuuucggcc..	2	0	MW1
.........agggguuucuuucggccuccag...	2	0	MW1
....guaggccggcggaaacuacu..............	5	0	MW1
....guaggccggcggaaacuacuug............	1	0	MW1
....guaggccggcggaaacuacuugc...........	28	0	MW1
....guaggccggcggaaacuacuugcu..........	2	0	MW1
....guaggccggcggaaacuacuugUu..........	1	1	MW1
....guaggccggcggaaacuac...............	1	0	MW2
....guaggccggcggaaacuacu..............	1	0	MW2
....guaggccggcggaaacuacuu.............	1	0	MW2
....guaggccggcggaaacuacuugc...........	7	0	MW2
....guaggccggcggaaacuacuugcu..........	1	0	MW2
....cggcggaaacuacuugc...........	1	0	MW2
....guaggccggcggaaacuacu..............	3	0	TE2
....guaggccggcggaaacuacuug............	3	0	TE2
....guaggccggcgCaaacuacuugc...........	1	1	TE2
....guaggccggcggaaacuacuugc...........	21	0	TE2
....guaggccggcggaaacuacuugcA..........	1	1	TE2
....guaggccggcggaaacuacuugcu..........	4	0	TE2

miRBase precursor : aga-mir-285
Total read count : 1318
aga-mir-285 read count : 1310
aga-mir-285-star read count: 7
remaining reads : 1

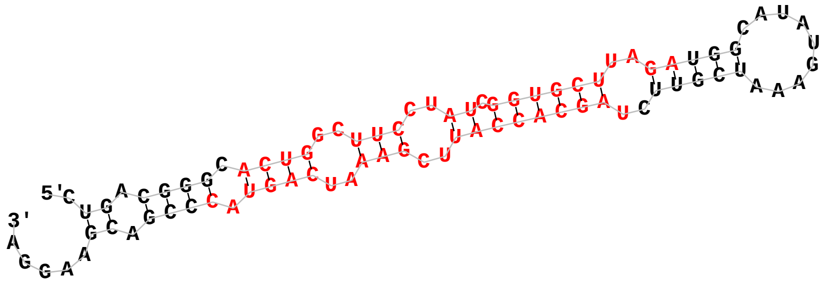

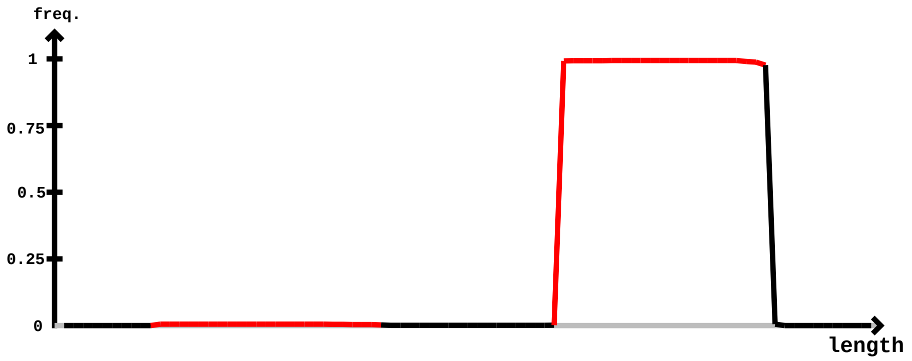

aga-mir-285-star

aga-mir-285

```
5'- cugacgggcacuggcuuccuaucggugcuuagauggcauaugaaaucguucuagcaccauucgaaaucaguacccgacgaagga   -3'    exp
    ....((((.((((..(((..((.((((((..(((((.........)))))..))))))))..))).)))).)))).........   reads      mm         sample
    ...................................................uagcaccauucgaaaucaguac...........   4          0          FF2

    ...................................................uagcaccauucgaaaucagua............   1          0          OV2
    ...................................................uagcaccauucgaaaucaguac...........   204        0          OV2
    ...................................................uagcaccauucgaaaucaguaA...........   1          1          OV2
    ...................................................uGgcaccauucgaaaucaguac...........   1          1          OV2
    ...................................................uagcaccauucgaaaucaguacA..........   2          1          OV2

    ..................................................cuagcaccauucgaaaucaguac...........   1          0          TE1
    ...................................................uagcaccauucgaaaucagua............   3          0          TE1
    ...................................................uagcaccauucgaaaucaguac...........   122        0          TE1
    ...................................................uagcacUauucgaaaucaguac...........   1          1          TE1

    .........acuggcuuccuaucggugcuuag....................................................   1          0          MF2
    ...................................................uagcaccauucgaaaucaguac...........   40         0          MF2

    .........acuggcuuccuaucggugcuuag....................................................   1          0          FW2
    .........acuggcuuccuaucggugcuuaga...................................................   1          0          FW2
    ...................................................uagcaccauucgaaaucag..............   1          0          FW2
    ...................................................uagcaccauucgaaaucaguac...........   97         0          FW2
    ...................................................uagcaccauucgaaaucaguacA..........   1          1          FW2

    ...................................................uagcaccauucgaaaucaguac...........   7          0          FF1
    ...................................................uagcaccauucgaaaucaguCc...........   1          1          FF1

    .........acuggcuuccuaucggugcuuaga...................................................   1          0          OV1
    ...................................................Cagcaccauucgaaaucaguac...........   1          1          OV1
    ...................................................uagcaccauucgaaaucaguac...........   111        0          OV1
    ...................................................uaAcaccauucgaaaucaguac...........   1          1          OV1

    ...................................................uagcaccauucgaaaucaguac...........   10         0          MF1

    ...................................................uagcaccauucgaaaucag..............   1          0          BF2
    ...................................................uagcaccauucgaaaucagu.............   1          0          BF2
```

```
         aga-mir-285-star
                                                   aga-mir-285
cugacgggcacuggcuuccuaucggugcuuagauggcauaugaaaucguucuagcaccauucgaaaucaguacccgacgaagga

...................................................uagcaccauucgaaaucagua............    2     0     BF2
...................................................uagcaccauucgaaaucaguac...........    20    0     BF2

...................................................uagcaccauucgaaaucagu.............    1     0     BF1
...................................................uagcaccauucgaaaucagua............    1     0     BF1
...................................................uagcaccauucgaaaucaguac...........    42    0     BF1
...................................................uagcaccauucgaaaucaguacA..........    1     1     BF1

...................................................uagcaccauucgaaaucagua............    2     0     FW1
...................................................uagcaccauucgaaaucaguaA...........    1     1     FW1
...................................................uagcaccauucUaaaucaguac...........    1     1     FW1
...................................................uagcaccauucgaaaucaguac...........    43    0     FW1

...................................................uagcaccauucgaaaucag..............    1     0     MW1
...................................................uagcaccauucgaaaucaguac...........    84    0     MW1
...................................................Cagcaccauucgaaaucaguac...........    1     1     MW1
...................................................uagcaUcauucgaaaucaguac...........    1     1     MW1
...................................................uagAaccauucgaaaucaguac...........    1     1     MW1
...................................................uagcaccauucgaaaucaguacA..........    1     1     MW1
....................................................Ugcaccauucgaaaucaguac...........    1     1     MW1

.................................uggcauaugaaaucguuc.................................    1     0     MW2
...................................................uagcaccauucgaaaucaguac...........    37    0     MW2

.........acuggcuuccuaucggug.........................................................    1     0     TE2
.........acuggcuuccuaucggugcu.......................................................    1     0     TE2
.........acuggcuuccuaucggugcuuaga...................................................    1     0     TE2
...................................................uagcaccauucgaaaucag..............    2     0     TE2
...................................................uagcaccauucgaaaucagu.............    1     0     TE2
...................................................uagcaccauucgaaaucagua............    6     0     TE2
...................................................uaAcaccauucgaaaucaguac...........    1     1     TE2
...................................................uaUcaccauucgaaaucaguac...........    1     1     TE2
...................................................uagcaccauucgaaaucaguaA...........    1     1     TE2
...................................................uagcaccauucAaaaucaguac...........    1     1     TE2
...................................................uagcaccauucgaaaucaguaU...........    1     1     TE2
...................................................uagcaccauucgaaaCcaguac...........    1     1     TE2
...................................................uagcaccauucgaaaucagGac...........    1     1     TE2
...................................................uagcaccauucgaaaucaguac...........    438   0     TE2
...................................................uagcaccauucgaaaucaguacA..........    1     1     TE2
...................................................uagcaccauucgaaaucaguacU..........    1     1     TE2
........................................................ccauucgaaaucaguac...........    1     0     TE2
```

miRBase precursor : aga-mir-286b
Total read count : 33151
aga-mir-286b read count : 33033
aga-mir-286b-star read count : 110
remaining reads : 8

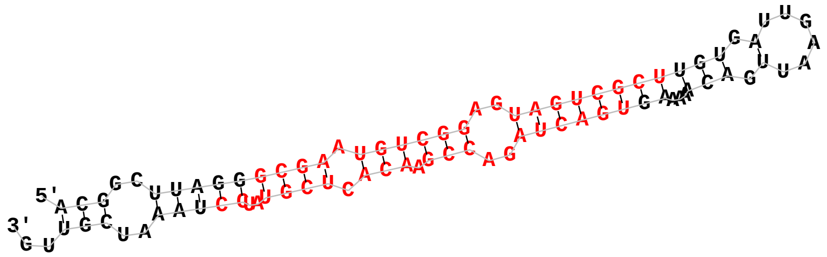

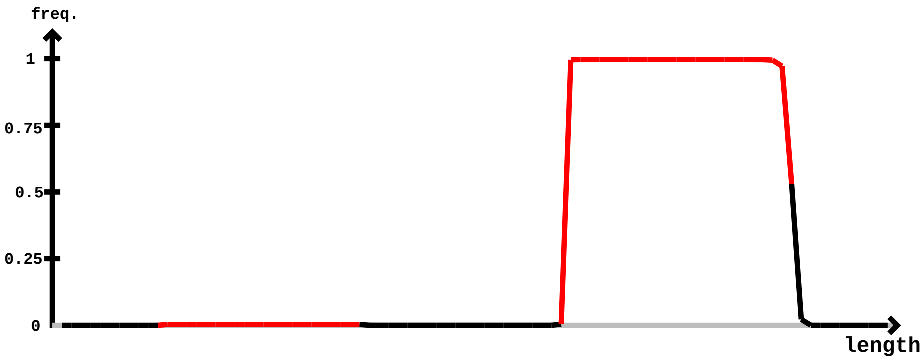


```
                                                   aga-mir-286b
            aga-mir-286b-star
5'- acggcuuagggcgaaugucggaguagucgcuugugauugaauugacaaaaagugacuagaccgaacacucguauccuaaaucguug  -3'   exp
    (((..(((((((((.((((((..(((((((((((.(......).)))...))))))))..))).))).))))..)))))..)))..    reads   mm   sample
    ....................................................ugacuagaccgaacacucguau............    9       0    OV2
    ....................................................ugacuagaccgaacGcucguauc...........    1       1    OV2
    ....................................................ugacuagaccgaacacucguauc...........    32      0    OV2
    ....................................................Agacuagaccgaacacucguaucc..........    1       1    OV2
    ....................................................ugacuagaccgaacacucguaucc..........    13      0    OV2
    .....................................................gacuagaccgaacacucguaucc..........    1       0    OV2

    ....................................................ugacuagaccgaacacucgua.............    7       0    TE1
    ....................................................ugacuagaUcgaacacucgua.............    1       1    TE1
    ....................................................ugacuagaccgaacacucguau............    40      0    TE1
    ....................................................ugacuagaccgaacacuAguauc...........    1       1    TE1
    ....................................................ugacuagaccgaacacucguauc...........    47      0    TE1
    ....................................................ugacuagaccgaacacucguaGcc..........    1       1    TE1
    ....................................................ugacuagaccgaacacucguaucU..........    4       1    TE1
    ....................................................ugacuagaccgaacacucguaucc..........    21      0    TE1
    ....................................................ugacuagaccgaacacucguauccA.........    1       1    TE1
    ....................................................ugacuagaccgaacacucguauccu.........    4       0    TE1

    ...................................................Cugacuagaccgaacacucguauc...........    1       1    FF2
    ....................................................ugacuagaccgaacacucguau............    3       0    FF2
    ....................................................ugacuagaccgaacacucguauc...........    6       0    FF2
    ....................................................ugacuagaccgaacacucguaucU..........    2       1    FF2
    ....................................................ugacuagaccgaacacucguaucc..........    6       0    FF2

    ...................................................Cugacuagaccgaacacucguauc...........    2       1    MF2
    ....................................................ugacuagaccgaacacucgu..............    1       0    MF2
    ....................................................ugacuagaccgaacacucgua.............    2       0    MF2
    ....................................................ugacuagaccgaacacucguau............    14      0    MF2
    ....................................................ugacuagaccgaacacucguaA...........    1       1    MF2
    ....................................................ugacuagaccgaacacucguauc...........    73      0    MF2
    ....................................................ugacuagaccgaacacucguaucU..........    11      1    MF2
    ....................................................ugacuagaccgUacacucguaucc..........    1       1    MF2
    ....................................................ugacuagaccgaacacucguaucc..........    44      0    MF2
    ....................................................ugacuagaccgaacacucguauccu.........    8       0    MF2
```

aga-mir-286b

aga-mir-286b-star

acggcuuagggcgaaugucggaguagucgcuugugauugaauugacaaaaagugacuagaccgaacacucguauccuaaaucguug

....ugacuagaccgaacacucguauc...........	6	0	FW2
....ugacuagaccgaacacucguaucc..........	12	0	FW2
....ugacuagaccgaacacucguaucU..........	2	1	FW2
....ugacuagaccgaacacucguauccu.........	2	0	FW2
....ugacuagaccgaacacucguauccA.........	1	1	FW2
....gugacuagaccgaacacucguauc...........	1	0	OV1
....ugacuagaccgaacacucgu..............	1	0	OV1
....ugacuagaccgaacacucgua.............	2	0	OV1
....ugacuagaccgaacacucguau............	24	0	OV1
....ugacuagaccgaacacucguaA............	1	1	OV1
....ugacuagaccgaacacucguauc...........	34	0	OV1
....ugacuagaccgaacacAcguauc...........	1	1	OV1
....ugacuagaccAaacacucguauc...........	1	1	OV1
....ugacuagaccgaacacucguaucA..........	1	1	OV1
....ugacuagaccgaacacucguaucc..........	14	0	OV1
....ugacuagacAgaacacucguaucc..........	1	1	OV1
....ugacuagaccgaacacucguaucU..........	8	1	OV1
..........gcgaaugucggaguagucg...	1	0	FF1
....Cugacuagaccgaacacucguaucc..........	1	1	FF1
....ugacuagaccgaacacucguCu............	1	1	FF1
....ugacuagaccgaacacucguau............	3	0	FF1
....ugacuagaccgaacacucguauc...........	12	0	FF1
....ugacuagaccgaacacucguaucc..........	6	0	FF1
....ugacuagaccgaacacucguaucU..........	5	1	FF1
....ugacuagaccgaacacucguauccu.........	1	0	FF1
....ugacuagaccgaacacuc................	1	0	MF1
....ugacuagaccgaacacucg...............	1	0	MF1
....ugacuagaccgaacacucguau............	4	0	MF1
....ugacuagaccgaacacucguauc...........	18	0	MF1
....ugacuagaccgaacacucguaucc..........	6	0	MF1
....ugacuagaccgaacacucguaucU..........	2	1	MF1
....ugacuagaccgaacacucguauccu.........	1	0	MF1
.........ggcgaaugucggaguagucgcu...	1	0	BF2
..........gcgaaugucggaguagucg...	1	0	BF2
..........gcgaaugucggaguagucgc..	2	0	BF2
..........gcgaaugucggaguagucgcu...	83	0	BF2
..........gcgaaugucggaguagucgcuu..	6	0	BF2
...........cgaaugucggaguagucgcu...	3	0	BF2
............gaaugucggaguagucgcu...	3	0	BF2
............................gcuugugauugaauugaca.......................................	1	0	BF2
...............................ugugauugaauugacaaaaag..................................	2	0	BF2
...............................ugugauugaauugacaaaaagugacuagaccgaacacucguauc...........	1	0	BF2
....aaaagugacuagaccgaacacu.................	1	0	BF2
....agugacuagaccgaacacucgua.............	1	0	BF2
....gugacuagaccgaacacucgua.............	2	0	BF2
....gugacuagaccgaacacucguau............	4	0	BF2
....Cugacuagaccgaacacucguauc...........	21	1	BF2
....gugacuagaccgaacacucguauc...........	26	0	BF2
....Cugacuagaccgaacacucguaucc..........	17	1	BF2
....gugacuagaccgaacacucguaucc..........	15	0	BF2
....Uugacuagaccgaacacucguaucc..........	1	1	BF2
....gugacuagaccgaacacucguaucU..........	6	1	BF2
....Cugacuagaccgaacacucguauccu.........	2	1	BF2
....gugacuagaccgaacacucguauccu.........	1	0	BF2
....ugacuagaccgaacacu.................	1	0	BF2
....ugacuagaccgaacacucgu..............	1	0	BF2
....ugacuagaccgaacacucgua.............	24	0	BF2
....ugacuagaUcgaacacucgua.............	1	1	BF2
....ugacuagaccgaacacucgCa.............	1	1	BF2
....ugGcuagaccgaacacucguau............	1	1	BF2
....ugacuagaccgaacacucguau............	463	0	BF2
....ugacuagaccgaacacuUguau............	1	1	BF2
....uUacuagaccgaacacucguau............	1	1	BF2
....ugacuagaccgaacacuAguau............	1	1	BF2

```
                                                    aga-mir-286b
          aga-mir-286b-star
acggcuuagggcgaaugucggaguagucgcuugugauugaauugacaaaaagugacuagaccgaacacucguauccuaaaucguug

....................................................ugacuagaccUaacacucguau............	1	1	BF2
....................................................ugacuagacUgaacacucguauc...........	8	1	BF2
....................................................ugacuagaccgaUcacucguauc...........	1	1	BF2
....................................................ugacuagaccgaacacucgAauc...........	1	1	BF2
....................................................ugacuagaccgaacacucCuauc...........	1	1	BF2
....................................................ugacuagaccgaacacucguaCc...........	5	1	BF2
....................................................Cgacuagaccgaacacucguauc...........	3	1	BF2
....................................................ugacuagaccgaacaGucguauc...........	1	1	BF2
....................................................Agacuagaccgaacacucguauc...........	4	1	BF2
....................................................ugacuagaccgGacacucguauc...........	3	1	BF2
....................................................ugacuagaccgaacacucguauU...........	36	1	BF2
....................................................uUacuagaccgaacacucguauc...........	1	1	BF2
....................................................ugacuagaccgaacacucUuauc...........	1	1	BF2
....................................................ugacuagaccgaacacucAuauc...........	3	1	BF2
....................................................ugUcuagaccgaacacucguauc...........	2	1	BF2
....................................................ugacuagaccgaaAacucguauc...........	2	1	BF2
....................................................ugaUuagaccgaacacucguauc...........	1	1	BF2
....................................................ugacAagaccgaacacucguauc...........	2	1	BF2
....................................................ugacuagaccgaacacuGguauc...........	1	1	BF2
....................................................ugacuagaccgaacacucgGauc...........	2	1	BF2
....................................................ugaAuagaccgaacacucguauc...........	1	1	BF2
....................................................uAacuagaccgaacacucguauc...........	3	1	BF2
....................................................ugacuagaGcgaacacucguauc...........	4	1	BF2
....................................................ugacuagaccgaacacCcguauc...........	3	1	BF2
....................................................ugacuaAaccgaacacucguauc...........	1	1	BF2
....................................................ugacuagacAgaacacucguauc...........	2	1	BF2
....................................................ugacuagaccgaGcacucguauc...........	1	1	BF2
....................................................ugacuagaccgaacacucgCauc...........	3	1	BF2
....................................................ugacuagaccgaaUacucguauc...........	2	1	BF2
....................................................ugacuagaccgaacaUucguauc...........	2	1	BF2
....................................................ugacuagaUcgaacacucguauc...........	2	1	BF2
....................................................ugacuagaccgaacacuAguauc...........	1	1	BF2
....................................................ugGcuagaccgaacacucguauc...........	2	1	BF2
....................................................ugacuagaccgaacacucguauA...........	1	1	BF2
....................................................ugacuagaccgaacacucguGuc...........	1	1	BF2
....................................................ugacuagaccgaacacuUguauc...........	5	1	BF2
....................................................ugacuagaccgUacacucguauc...........	1	1	BF2
....................................................ugacCagaccgaacacucguauc...........	3	1	BF2
....................................................ugacuagaccUaacacucguauc...........	2	1	BF2
....................................................ugacuagaAcgaacacucguauc...........	2	1	BF2
....................................................ugacuagGccgaacacucguauc...........	1	1	BF2
....................................................ugacuagaccgaacacucguauc...........	11955	0	BF2
....................................................ugacuagaccgaacacAcguauc...........	1	1	BF2
....................................................ugacuagaccAaacacucguauc...........	23	1	BF2
....................................................ugacuagaccUaacacucguaucc..........	8	1	BF2
....................................................ugacuagaccgaacacucUuaucc..........	2	1	BF2
....................................................ugacuagaccgCacacucguaucc..........	3	1	BF2
....................................................ugacuagaccgaacacuAguaucc..........	1	1	BF2
....................................................ugacuaCaccgaacacucguaucc..........	1	1	BF2
....................................................ugacuUgaccgaacacucguaucc..........	1	1	BF2
....................................................ugacuagaccgaacacCcguaucc..........	2	1	BF2
....................................................ugacuagaccgaacacucgCaucc..........	5	1	BF2
....................................................ugacuagaccgaacacucCuaucc..........	2	1	BF2
....................................................ugacuagaccgaacUcucguaucc..........	1	1	BF2
....................................................Cgacuagaccgaacacucguaucc..........	6	1	BF2
....................................................ugacuagaccAaacacucguaucc..........	11	1	BF2
....................................................ugacuagaccgaacacucgGaucc..........	2	1	BF2
....................................................ugacuagaccgaacacucguaCcc..........	3	1	BF2
....................................................ugacuagaccgaacacucguaucG..........	4	1	BF2
....................................................ugacuagaccgGacacucguaucc..........	3	1	BF2
....................................................ugacuagaccgaacacucguaucc..........	12795	0	BF2
....................................................ugacuagaccCaacacucguaucc..........	4	1	BF2
....................................................ugacuagaccgaacacucguaucU..........	1055	1	BF2
....................................................ugacuagaccgaacacAcguaucc..........	1	1	BF2
....................................................ugaGuagaccgaacacucguaucc..........	1	1	BF2
....................................................ugacuagaccgaacacucAuaucc..........	4	1	BF2
....................................................ugacCagaccgaacacucguaucc..........	1	1	BF2
....................................................ugUcuagaccgaacacucguaucc..........	1	1	BF2
....................................................ugacuagGccgaacacucguaucc..........	1	1	BF2
```

```
                                                    aga-mir-286b
          aga-mir-286b-star
acggcuuagggcgaaugucggaguagucgcuugugauugaauugacaaaaagugacuagaccgaacacucguauccuaaaucguug

....................................................ugaUuagaccgaacacucguaucc..........   1      1      BF2
....................................................ugacuagacUgaacacucguaucc..........   2      1      BF2
....................................................ugacuagaccgaacacucguaucA..........   8      1      BF2
....................................................uCacuagaccgaacacucguaucc..........   1      1      BF2
....................................................ugacuaUaccgaacacucguaucc..........   3      1      BF2
....................................................ugacuagaccgaacacucguauAc..........   1      1      BF2
....................................................ugacuagaccgaacCcucguaucc..........   1      1      BF2
....................................................ugacuagaccgaacacuGguaucc..........   1      1      BF2
....................................................ugacuagaUcgaacacucguaucc..........   2      1      BF2
....................................................ugGcuagaccgaacacucguaucc..........   2      1      BF2
....................................................ugacuGgaccgaacacucguaucc..........   2      1      BF2
....................................................ugacuagaccgaacacuUguaucc..........   3      1      BF2
....................................................uAacuagaccgaacacucguaucc..........   5      1      BF2
....................................................ugacuagaccgaacacucguauUc..........   1      1      BF2
....................................................ugacuagaccgaaUacucguaucc..........   3      1      BF2
....................................................ugacuaAaccgaacacucguaucc..........   3      1      BF2
....................................................ugacuagaccgaacacucguGucc..........   1      1      BF2
....................................................ugacuagaAcgaacacucguaucc..........   2      1      BF2
....................................................ugacuagaccgaacacucguauccu.........   574    0      BF2
....................................................ugacuagGccgaacacucguauccu.........   1      1      BF2
....................................................ugacuagCccgaacacucguauccu.........   1      1      BF2
....................................................ugacuagaccAaacacucguauccu.........   1      1      BF2
....................................................ugacuagaccgaacacucguauccA.........   19     1      BF2
....................................................ugacuagaccgaacacucguauccC.........   3      1      BF2
....................................................ugacuagaccgaacacucguauccG.........   4      1      BF2
....................................................ugacuagaccgUacacucguauccu.........   1      1      BF2
....................................................ugacuagaccgaaAacucguauccu.........   1      1      BF2
....................................................ugacuaAaccgaacacucguauccu.........   1      1      BF2
....................................................ugacuagaccgaacacucguauccuU........   4      1      BF2
.....................................................gacuagaccgaacacucguauc...........   8      0      BF2
.....................................................gacuagaccgaacacucguaucc..........   3      0      BF2
.....................................................gacuagaccgaacacucguauccu.........   1      0      BF2
.......................................................cuagaccgaacacucguaucc..........   2      0      BF2
........................................................uagaccgaacacucguauccu.........   2      0      BF2
.........................................................agaccgaacacucguaucc..........   1      0      BF2

.........ggcgaaugucggaguagucgcu.......................................................   1      0      BF1
..........gcgaaugucggaguagucgcu.......................................................   5      0      BF1
..........gcgaaugucggaguagucgcuugugauugaauugacaaaaag..................................   2      0      BF1
...........cgaaugucggaguagucgcu.......................................................   1      0      BF1
................................gugauugaauugacaaaaagugacuagaccgaacacucguau............   1      0      BF1
...................................................Cugacuagaccgaacacucguau............   1      1      BF1
...................................................gugacuagaccgaacacucguauc...........   7      0      BF1
...................................................Cugacuagaccgaacacucguauc...........   8      1      BF1
...................................................gugacuagaccgaacacucguaucU..........   1      1      BF1
...................................................gugacuagaccgaacacucguaucc..........   5      0      BF1
...................................................Cugacuagaccgaacacucguaucc..........   8      1      BF1
....................................................ugacuagaccgaacacucgua.............   2      0      BF1
....................................................ugacuagaccgaacacucguau............   71     0      BF1
....................................................ugacuagacUgaacacucguauc...........   1      1      BF1
....................................................ugaUuagaccgaacacucguauc...........   1      1      BF1
....................................................ugacuagaUcgaacacucguauc...........   1      1      BF1
....................................................ugacuagaccgUacacucguauc...........   2      1      BF1
....................................................ugUcuagaccgaacacucguauc...........   1      1      BF1
....................................................ugacuagGccgaacacucguauc...........   1      1      BF1
....................................................ugacuagaccgaacacucgCauc...........   1      1      BF1
....................................................ugacuagaccgaacacucguaCc...........   2      1      BF1
....................................................uAacuagaccgaacacucguauc...........   3      1      BF1
....................................................ugacuagaccgaacacucguGuc...........   1      1      BF1
....................................................ugacuagaccgaGcacucguauc...........   1      1      BF1
....................................................ugacuagaccgaacacuUguauc...........   2      1      BF1
....................................................ugacuagaccUaacacucguauc...........   3      1      BF1
....................................................Cgacuagaccgaacacucguauc...........   1      1      BF1
....................................................ugacuagaccgaacUcucguauc...........   1      1      BF1
....................................................ugacCagaccgaacacucguauc...........   1      1      BF1
....................................................ugacuagaccgaacacucguauU...........   7      1      BF1
....................................................ugacuagaccgCacacucguauc...........   2      1      BF1
....................................................ugacuagaccgaacacucguauc...........   2022   0      BF1
....................................................ugacuagaccAaacacucguauc...........   5      1      BF1
```

```
                                                    aga-mir-286b
          aga-mir-286b-star
acggcuuagggcgaaugucggaguagucgcuugugauugaauugacaaaaagugacuagaccgaacacucguaucc uaaaucguug

....................................................ugacuagaccCaacacucguauc...........   2      1     BF1
....................................................ugacuagaccgaacacucgAauc...........   1      1     BF1
....................................................ugacuagaAcgaacacucguauc...........   1      1     BF1
....................................................ugacuagaccAaacacucguaucc..........   6      1     BF1
....................................................ugacuagaccgaacacucguaucU..........   160    1     BF1
....................................................ugacuagaccgaacacucguaucA..........   1      1     BF1
....................................................Cgacuagaccgaacacucguaucc..........   2      1     BF1
....................................................ugaAuagaccgaacacucguaucc..........   1      1     BF1
....................................................ugacuagaccUaacacucguaucc..........   2      1     BF1
....................................................ugacuagaccgaacGcucguaucc..........   1      1     BF1
....................................................ugacuagaccgaacacCcguaucc..........   1      1     BF1
....................................................ugacuagaccgaacacucgCaucc..........   1      1     BF1
....................................................ugacuagaccgaacacucguaCcc..........   2      1     BF1
....................................................ugacuagaccgaacaAucguaucc..........   1      1     BF1
....................................................ugacuagaccgGacacucguaucc..........   1      1     BF1
....................................................ugacuagaccgaaUacucguaucc..........   1      1     BF1
....................................................uAacuagaccgaacacucguaucc..........   1      1     BF1
....................................................ugacuagaccgaacacucguaucc..........   2365   0     BF1
....................................................ugacuagaccgaacacuUguaucc..........   2      1     BF1
....................................................ugacuagaccAaacacucguauccu.........   1      1     BF1
....................................................ugacuagaccgaacacucguauccu.........   99     0     BF1
....................................................ugacuagaccgaacacucguauccG.........   1      1     BF1
....................................................ugacuagaccgaacacucguauccA.........   3      1     BF1
....................................................ugacuagaccgaacacucguauccua........   1      0     BF1
.....................................................gacuagaccgaacacucguauc...........   2      0     BF1

....................................................ugacuagaccgaacacucguau............   1      0     MW1
....................................................ugacuagaccgaacacucguauc...........   7      0     MW1
....................................................ugacuagaccgaacacucguaucU..........   5      1     MW1
....................................................ugacuagaccgaacacucguaucc..........   2      0     MW1
....................................................ugacuagaccgaacacucguauccu.........   1      0     MW1

....................................................ugacuagaccgaacacucguau............   2      0     FW1
....................................................ugacuagaccgaacacucguauc...........   3      0     FW1
....................................................ugacuagaccgaacacucguaucc..........   5      0     FW1
....................................................ugacuagaccgaacacucguaucU..........   1      1     FW1

....................................................ugacuagaccgaacacucguau............   2      0     MW2
....................................................ugacuagaccgaacacucguauc...........   2      0     MW2
....................................................ugacuagaccgaacacucguaucc..........   3      0     MW2

..........gcgaaugucggaguagucg.........................................................   2      0     TE2
..........gcgaaugucggaguagucgcuu......................................................   1      0     TE2
...................................................Cugacuagaccgaacacucguauc...........   2      1     TE2
....................................................ugacuagaccgaacacucg...............   2      0     TE2
....................................................ugacuagaccgaacacucgu..............   2      0     TE2
....................................................ugacuagaccgaacacucgua.............   15     0     TE2
....................................................ugacuagaccgaaUacucguau............   1      1     TE2
....................................................ugacuagaccgaacacucguau............   101    0     TE2
....................................................ugacuagaccgaacacucguauU...........   2      1     TE2
....................................................ugacuagaccgaacacucguauc...........   159    0     TE2
....................................................ugacuagaccgaacacucAuauc...........   1      1     TE2
....................................................ugacuagaccgaacacucguauA...........   1      1     TE2
....................................................ugacuagaccgaacacucguaucU..........   4      1     TE2
....................................................ugacuagaccgaacacucguaucc..........   72     0     TE2
....................................................ugacuagaccgaacacucguauccA.........   1      1     TE2
....................................................ugacuagaccgaacacucguauccu.........   3      0     TE2
```

miRBase precursor : aga-mir-2940
Total read count : 13301
aga-mir-2940 read count : 12649
aga-mir-2940-star read count : 542
remaining reads : 110

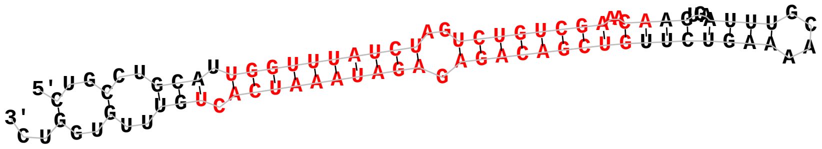

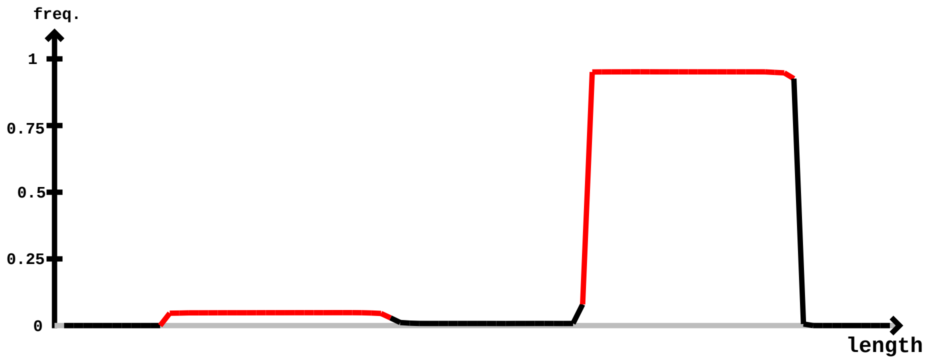


```
                          aga-mir-2940-star
                                                                                  aga-mir-2940
5'- cugccugcauugguuuaucuagucugucgaaacaagugaauuugcaaaagucuugucgacagagagauaaaaucacuguuugugguc   -3'    exp
    (..(..(((.((((((((((..((((((((..((((...((((....)))))))))))))))).)))))))))).))).)..)..)..    reads      mm         sample
    .........ugguuuaucuagucugucgaaac..................................................    6          0          TE1
    .........ugguuuaucuagucugucgaaaca.................................................    1          0          TE1
    .........ugguuuaucuagucugucgaaacaa................................................    2          0          TE1
    .........ugguuuaucuagucugucgaaacaag...............................................    1          0          TE1
    .........ugguuuaucuagucuAucgaaacaagugaauuugcaaaagucuu.............................    1          1          TE1
    .........ugguuuaucuagucugucgaaacaagugaauuugcaaaagucuu.............................    2          0          TE1
    ....................................................ugucgacagagagauaaaauc............    1          0          TE1
    ....................................................ugucgacagagagauaaaaucac..........    3          0          TE1
    ....................................................ugucgacagagagauaaaaucaAu.........    1          1          TE1
    ....................................................ugucgacagagagauaaaaucacu.........    28         0          TE1
    .....................................................gucgacagagagauaaaaucac..........    3          0          TE1
    .....................................................gucgacagagagauaaaaucacG.........    1          1          TE1
    .....................................................Aucgacagagagauaaaaucacu.........    1          1          TE1
    .....................................................gucgacagagagauaaaaucacu.........    216        0          TE1
    .....................................................gucgacagagagauaaCucacu.........    1          1          TE1
    .....................................................gucgacagagagauaaaaucacug........    1          0          TE1
    .....................................................gucgacagagagauaaaaucacuU........    2          1          TE1

    .........ugguuuaucuagucugucgaaa...................................................    3          0          FF2
    .........ugguuuaucuagucugucgaaac..................................................    12         0          FF2
    .........ugguuuaucuagucugucgaaacC.................................................    1          1          FF2
    .........ugguuuaucuagucugucgaaaca.................................................    3          0          FF2
    .........ugguuuaucuagucugucgaaacaa................................................    1          0          FF2
    .........ugguuuaucuagucugucgaaacaag...............................................    1          0          FF2
    ....................................................ugucgacagagagauaaaaucacu.........    19         0          FF2
    ....................................................ugucgacaUagagauaaaaucacu.........    1          1          FF2
    .....................................................gucgacagagagauaaaauca...........    2          0          FF2
    .....................................................Aucgacagagagauaaaaucac..........    1          1          FF2
    .....................................................gucgacagagagauaaaaucac..........    12         0          FF2
    .....................................................gucgacagagagaCaaaucacu.........    1          1          FF2
    .....................................................gucgacagagagauaaaaucacu.........    373        0          FF2
    .....................................................gucgacagaCagauaaaaucacu.........    1          1          FF2
    .....................................................gucAacagagagauaaaaucacu.........    1          1          FF2
    .....................................................gucgacagagagauaaaaucacuU........    1          1          FF2
```

aga-mir-2940-star

aga-mir-2940

cugccugcauugguuuaucuagucugucgaaacaagugaauuugcaaaagucuugucgacagagagauaaaucacuguuugugguc

..........ugguuuaucuagucugucga..	2	0	OV2
..........ugguuuaucuagucugucgaa...	4	0	OV2
..........ugguuuaucuagucugucgaaac...	6	0	OV2
..........ugguuuaucuagucugucgaaaca..	4	0	OV2
..........ugguuuaucuagucugucgaaacaagugaauuugcaaaagucu.................................	1	0	OV2
....ugucgacagagagauaaauc.............	1	0	OV2
....ugucgacagagagauaaauca............	1	0	OV2
....ugucgacagagagauaaaucac...........	1	0	OV2
....ugucgacagagagauaaaucacu..........	17	0	OV2
....ugucgacagagagaAaaaucacu..........	1	1	OV2
....gucgacagagagauaaauca............	2	0	OV2
....gucgacagagagauaaaucac...........	5	0	OV2
....gucgacagagGgauaaaucacu..........	1	1	OV2
....gucgacaCagagauaaaucacu..........	1	1	OV2
....gucgacagagagGuaaaucacu..........	1	1	OV2
....gucgacagagaUauaaaucacu..........	1	1	OV2
....gucgacagagagauaaaucacu..........	174	0	OV2
....gucgacagagagauaaaucacuU.........	1	1	OV2
..........ugguuuaucuagucugucgaa...	5	0	MF2
..........ugguuuaucuagucugucgaaa..	9	0	MF2
..........ugguuuaucuagucugucgaaU..	1	1	MF2
..........ugguuuaucuagucugucgaaac...	85	0	MF2
..........ugguuuaucuagucugucgaaaA...	1	1	MF2
..........uggAuuaucuagucugucgaaac...	1	1	MF2
..........uggCuuaucuagucugucgaaaca..	1	1	MF2
..........ugguuuaucuagucugucgaaacC..	35	1	MF2
..........ugguuuaucuagucugucgaaaca..	79	0	MF2
..........ugguuuaucuagucuguUgaaaca..	1	1	MF2
..........ugguuuaucuagucuAucgaaaca..	1	1	MF2
..........ugguuuaucuagucugucgaaacaa...	15	0	MF2
..........ugguuuaucuagucugucgaaacaag..	7	0	MF2
..........ugguuuaucuagucugucgaaacaagA...	1	1	MF2
..........ugguuuaucuagucugucgaaacaagugaauuugcaa.......................................	1	0	MF2
..........ugguuuaucuagucugucgaaacaagugaauuugcaaaagu...................................	1	0	MF2
..........ugguuuaucuagucugucgaaacaagugaauuugcaaaagucu.................................	5	0	MF2
...........gguuuaucuagucugucgaaa..	1	0	MF2
...........gguuuaucuagucugucgaaaca..	3	0	MF2
...........gguuuaucuagucugucgaaacaagugaauuugcaaaagucu.................................	1	0	MF2
............guuuaucuagucugucgaaa..	1	0	MF2
............guuuaucuagucugucgaaac...	2	0	MF2
............guuuaucuagucugucgaaacC..	2	1	MF2
............guuuaucuagucugucgaaaca..	1	0	MF2
............guuuaucuagucugucgaaacaagugaauuugU...	1	1	MF2
..............uuaucuagucugucgaaaca..	1	0	MF2
................aucuagucugucgaaaca..	1	0	MF2
.......................cugucgaaacaagugaauuugcaaaagucuu................................	1	0	MF2
..................................agugaauuugcaaaagucuugucgaca.........................	1	0	MF2
....aaagucuugucgacagagagauaaaucacu..........	1	0	MF2
....aagucuugucgacagagagauaaaucacu..........	1	0	MF2
....cuugucgacagagagauaaaucacu..........	1	0	MF2
....uugucgacagagagauaaaucacu..........	2	0	MF2
....Cugucgacagagagauaaaucacu..........	1	1	MF2
....ugucgacagagagauaaauc.............	6	0	MF2
....ugucgacagagagauaaauca............	3	0	MF2
....ugucgacagagagauaaaucac...........	20	0	MF2
....uAucgacagagagauaaaucacu..........	1	1	MF2
....ugucgacagagagauaaaucacu..........	272	0	MF2
....ugucgacagagagauaaaucacC..........	2	1	MF2
....ugucgacagaUagauaaaucacu..........	1	1	MF2
....ugucgacagagaAauaaaucacu..........	1	1	MF2
....ugucgacagagagauaaaucacG..........	2	1	MF2
....ugucgacagagagauaaaucacuU.........	1	1	MF2
....gucgacagagagauaaauc.............	4	0	MF2
....gucgacagagagauaaauca............	8	0	MF2
....gucgacagagagauaaaucaA...........	1	1	MF2
....gucgacagagagauaaaucac...........	128	0	MF2
....gucgacagagagaCaaaucac...........	1	1	MF2

```
           aga-mir-2940-star
                                                      aga-mir-2940
cugccugcauugguuuaucuagucugucgaaacaagugaauuugcaaaagucuugucgacagagagauaaaucacuguuugugguc

......................................................Aucgacagagagauaaaucacu..........    3      1    MF2
......................................................gucgacagagagauaaaucacA..........    2      1    MF2
......................................................gucgGcagagagauaaaucacu..........    1      1    MF2
......................................................gucgacagagagauaaaucGcu..........    4      1    MF2
......................................................gucgaUagagagauaaaucacu..........    2      1    MF2
......................................................gucgacagagagauaaGucacu..........    1      1    MF2
......................................................gucgacagaUagauaaaucacu..........    2      1    MF2
......................................................gucgacagagagauGaaucacu..........    1      1    MF2
......................................................gucgacagagagaGaaaucacu..........    1      1    MF2
......................................................gucgacagagUgauaaaucacu..........    1      1    MF2
......................................................guUgacagagagauaaaucacu..........    2      1    MF2
......................................................gucgacagagGgauaaaucacu..........    1      1    MF2
......................................................gucgacagagagaCaaaucacu..........    2      1    MF2
......................................................gucgacagagCgauaaaucacu..........    1      1    MF2
......................................................gucgacagagaAauaaaucacu..........    4      1    MF2
......................................................gucgacagagagauaaaucacu..........    4380   0    MF2
......................................................gucAacagagagauaaaucacu..........    3      1    MF2
......................................................gucgacagagagauaaaCcacu..........    1      1    MF2
......................................................gucgacagagagauaaaucacC..........    8      1    MF2
......................................................gucgacagagagGuaaaucacu..........    1      1    MF2
......................................................gucgacaAagagauaaaucacu..........    2      1    MF2
......................................................gucgacagagagauaaaUacu..........    2      1    MF2
......................................................gucgacaUagagauaaaucacu..........    1      1    MF2
......................................................gucgacagagagauaaaucacG..........    6      1    MF2
......................................................gucgacUgagagauaaaucacu..........    1      1    MF2
......................................................gucgacagGgagauaaaucacu..........    2      1    MF2
......................................................gCcgacagagagauaaaucacu..........    3      1    MF2
......................................................gucgacagagaCauaaaucacu..........    1      1    MF2
......................................................gucgacagaCagauaaaucacu..........    1      1    MF2
......................................................gucgacagagaUauaaaucacu..........    1      1    MF2
......................................................gucgacagagagauaaaucacug.........    1      0    MF2
......................................................gucgacagagagauaaaucacuU.........    21     1    MF2
.......................................................ucgacagagagauaaaucacu..........    2      0    MF2
.........................................................gacagagagauaaaucacu..........    1      0    MF2

..........ugguuuaucuagucugucgaaac.....................................................    8      0    FW2
..........ugguuuaucuagCcugucgaaac.....................................................    1      1    FW2
..........ugguuuaucuagucugucgaaaca....................................................    2      0    FW2
...........gguuuaucuagucugucgaaacaagugaauuugcaaaagucu.................................    1      0    FW2
...................uagucugucgaaacaagugaauuugcaaaagucuu................................    1      0    FW2
....................agucugucgaaacaagugaauuugcaaaagucuu................................    1      0    FW2
...........................cgaaacaagugaauuug..........................................    1      0    FW2
...........................cgaaacaagugaauuugcaaaagucu.................................    1      0    FW2
.................................aagugaauuugcaaaagucuugucgaca.........................    1      0    FW2
...................................gugaauuugcaaaagucuugucgac..........................    1      0    FW2
................................................agucuugucgacagagagauaaaucacu..........    1      0    FW2
.....................................................ugucgacagagagauaaaucac...........    4      0    FW2
.....................................................ugucgacagagagauaaaucacu..........    48     0    FW2
.....................................................ugucgacagagagauaaaucacuU.........    2      1    FW2
......................................................gucgacagagagauaaaucac...........    2      0    FW2
......................................................gucAacagagagauaaaucacu..........    1      1    FW2
......................................................gucgacagagagauaaaucacG..........    1      1    FW2
......................................................gucgacagagagaAaaaucacu..........    1      1    FW2
......................................................gucgacagagagauCaaucacu..........    1      1    FW2
......................................................gucgacagagagauaaaucacu..........    504    0    FW2
......................................................gucgacagagagauaaaucacuU.........    6      1    FW2
......................................................gucgacagagagauaaaucacug.........    1      0    FW2
......................................................gucgacagagagauaaaucacuA.........    1      1    FW2
........................................................cgacagagagauaaaucacu..........    2      0    FW2
...........................................................cagagagauaaaucacu..........    1      0    FW2

..........ugguuuaucuagucugucgaCac.....................................................    1      1    FF1
..........ugguAuaucuagucugucgaaac.....................................................    1      1    FF1
..........uggGuuaucuagucugucgaaac.....................................................    3      1    FF1
..........ugguuuaucuagucugucgaaac.....................................................    27     0    FF1
..........uggCuuaucuagucugucgaaaca....................................................    1      1    FF1
..........uggGuuaucuagucugucgaaaca....................................................    1      1    FF1
..........ugguuuaucuagucugucgaaacC....................................................    2      1    FF1
..........ugguuuaucuagucugucgaaaca....................................................    23     0    FF1
```

aga-mir-2940-star

aga-mir-2940

cugccugcauugguuuaucuagucugucgaaacaagugaauuugcaaaagucuugucgacagagagauaaaucacuguuugugguc

..........ugguuuaucuagucugucgaaacU..	2	1	FF1
..........ugguuuaucuagucugucgaaacaa...	2	0	FF1
..........ugguuuaucuagucugucgaaacaag..	1	0	FF1
..........ugguuuaucuagucugucgaaacaagugaauuugcaaaag....................................	1	0	FF1
..........ugguuuaucuagucugucgaaacaagugaauuugcaaaagucu.................................	2	0	FF1
..........ugguuuaucuagucugucgaaacaagugaauuugcaaaagucuu................................	2	0	FF1
............guuuaucuagucugucgaaacaagugaauuugcaaaagucu.................................	1	0	FF1
...............uaucuagucugucgaaacaagugaauuugcaaaagucu.................................	1	0	FF1
................aucuagucugucgaaac...	1	0	FF1
....aaagucuugucgacagagagauaaaucacu..........	1	0	FF1
....ugucgacagagagauaaau..............	1	0	FF1
....uguGgacagagagauaaauc.............	1	1	FF1
....ugucgacagagagauaaauc.............	2	0	FF1
....ugucgacagagagauaaaucaU...........	1	1	FF1
....ugucgacagagagauaaaucac...........	1	0	FF1
....ugucUacagagagauaaaucacu..........	1	1	FF1
....ugucgacagagagauaaaucacu..........	33	0	FF1
....ugucgacagagagauaaaucacuU.........	1	1	FF1
....gucgacagagagauaaauc.............	3	0	FF1
....gucgacagagagauaaauca............	1	0	FF1
....gucgacagagagauaaaucac...........	14	0	FF1
....gucUacagagagauaaaucac...........	2	1	FF1
....gAcgacagagagauaaaucacu..........	1	1	FF1
....gucgacagagagCuaaaucacu..........	1	1	FF1
....gucgacagagagGuaaaucacu..........	1	1	FF1
....gucgGcagagagauaaaucacu..........	4	1	FF1
....gucgacagagagauaaaucacu..........	622	0	FF1
....gucAacagagagauaaaucacu..........	1	1	FF1
....Uucgacagagagauaaaucacu..........	19	1	FF1
....gucUacagagagauaaaucacu..........	41	1	FF1
....gucCacagagagauaaaucacu..........	1	1	FF1
....gGcgacagagagauaaaucacu..........	2	1	FF1
....gucgacagagagauaaaucacG..........	2	1	FF1
....gucgacagagagauaaCucacu..........	1	1	FF1
....Aucgacagagagauaaaucacu..........	3	1	FF1
....Cucgacagagagauaaaucacu..........	1	1	FF1
....gucgacagagagauaaaGcacu..........	1	1	FF1
....gucgacagagagauaaaucacA..........	1	1	FF1
....gucgacagagaAauaaaucacu..........	1	1	FF1
....gucgacagagagauaaaucCcu..........	1	1	FF1
....gucgacagagagauaaaucacuU.........	3	1	FF1

..........ugguuuaucuagucugucga..	1	0	OV1
..........ugguuuaucuagucugucgaa...	2	0	OV1
..........ugguuuaucuagucugucgaaa..	1	0	OV1
..........ugguuuaucuagucugucgaaac...	6	0	OV1
..........ugguuuaucuagucugucgaaaca..	1	0	OV1
....ugucgacagagagauaaauc.............	2	0	OV1
....ugucgacagagagauaaaucac...........	1	0	OV1
....ugucgacagGgagauaaaucacu..........	1	1	OV1
....ugucgacagagagauaaaucacu..........	20	0	OV1
....gucgacagagagauaaauc.............	1	0	OV1
....gucgacagagagauaaauca............	2	0	OV1
....gucgacagagagauaaaucac...........	5	0	OV1
....Uucgacagagagauaaaucacu..........	1	1	OV1
....gucgacagagagauaaaucacu..........	180	0	OV1
....gucgacagagagauaaaucacuU.........	1	1	OV1

..........ugguuuaucuagucugucgaa...	1	0	MF1
..........ugguuuaucuagucugucgaaa..	3	0	MF1
..........ugguuuaucuagucugucgaaac...	10	0	MF1
..........ugguuuaucuagucugucgaaaca..	9	0	MF1
..........ugguuuaucuagucugucgaaacU..	1	1	MF1
..........ugguuuaucuagucugucgaaacC..	2	1	MF1
..........ugguuuaucuagucugucgaaacaagugaauuugcaaaagucu.................................	1	0	MF1
....ugucgacagagagauaaaucac...........	1	0	MF1
....ugucgacagagagauaaaucacu..........	46	0	MF1
....ugucgacagagagauaaaucacuU.........	1	1	MF1
....gucgacagagagauaaaucac...........	13	0	MF1

aga-mir-2940-star

aga-mir-2940

cugccugcauugguuuaucuagucugucgaaacaagugaauuugcaaaagucuugucgacagagagauaaaucacuguuugugguc

....gucgacagagagaCaaaucacu..........	1	1	MF1
....gucgacaCagagauaaaucacu..........	1	1	MF1
....gucgacagagaAauaaaucacu..........	1	1	MF1
....gucgacagagagauaaaucacG..........	1	1	MF1
....gucgacagagagauaaaucacC..........	2	1	MF1
....gucgaGagagagauaaaucacu..........	1	1	MF1
....gucgacagagagauaaaucacu..........	509	0	MF1
....gucgaUagagagauaaaucacu..........	1	1	MF1
....gucgacagagagauaaauUacu..........	1	1	MF1
....gucgacagagagauaaaucacuU.........	4	1	MF1
....gucgacagagagauaaaucacuA.........	1	1	MF1
....ucgacagagagauaaaucacu..........	2	0	MF1
..........ugguuuaucuagucugucga..	1	0	BF2
..........ugguuuaucuagucugucgaaac...	7	0	BF2
..........ugguuuaucuagucugucgaaacC..	1	1	BF2
..........ugguuuaucuagucugucgaaaca..	20	0	BF2
..........ugguuuaucuagucugucgaaacaa...	6	0	BF2
..........ugguuuaucuagucugucgaaacaag..	2	0	BF2
..........ugguuuaucuagucugucgaaacaagugaauuugcaaaagu....................................	1	0	BF2
..........ugguuuaucuagucugucgaaacaagugaauuugcaaaagucu.................................	5	0	BF2
..........ugguuuaucuagucugucgaaacaagugaauuugcaaaagucuu................................	6	0	BF2
...........gguuuaucuagucugucgaaacaagugaauuugcaaaagucuu................................	1	0	BF2
............guuuaucuagucugucgaaac...	1	0	BF2
............guuuaucuagucugucgaaacaag..	1	0	BF2
....ugucgacagagagauaaaucac...........	8	0	BF2
....ugucgacagagagauaaaucacu..........	215	0	BF2
....ugucAacagagagauaaaucacu..........	1	1	BF2
....ugucgacagagagauaaaucacC..........	2	1	BF2
....ugucgaUagagagauaaaucacu..........	1	1	BF2
....gucgacagagagauaaauc.............	1	0	BF2
....gucgacagagagauaaauca............	2	0	BF2
....gucgacagagagauaaaucac...........	34	0	BF2
....gucgacagagagauaaaucacG..........	2	1	BF2
....gucgacagagagauaaauAacu..........	2	1	BF2
....gucgacagagaUauaaaucacu..........	3	1	BF2
....gucgacagagagauaaauUacu..........	1	1	BF2
....gucgacagagagGuaaaucacu..........	1	1	BF2
....gucgacagagagauaaaucacA..........	1	1	BF2
....gucgacaUagagauaaaucacu..........	1	1	BF2
....gucgacagagGgauaaaucacu..........	1	1	BF2
....guUgacagagagauaaaucacu..........	1	1	BF2
....gucgacagagagaCaaaucacu..........	1	1	BF2
....gucgaUagagagauaaaucacu..........	1	1	BF2
....gucgacaAagagauaaaucacu..........	2	1	BF2
....gucgacagagagauaaaucacu..........	2165	0	BF2
....gucgacagagagauaaaucacug.........	2	0	BF2
....gucgacagagagauaaaucacuU.........	7	1	BF2
..........ugguuuaucuagucugucgaa...	1	0	BF1
..........ugguuuaucuagucugucgaaac...	25	0	BF1
..........ugguuuaucuagucugucgaaaca..	20	0	BF1
..........ugguuuaucuagucugucgaaacaa...	5	0	BF1
..........ugguuuaucuagucugucgaaacaag..	2	0	BF1
..........Cgguuuaucuagucugucgaaacaagugaauuugcaaaaguc..................................	1	1	BF1
..........ugguuuaucuagucugucgaaacaagugaauuugcaaaaguc..................................	1	0	BF1
..........ugguuuaucuagucugucgaaacaagugaauuugcaaaagucu.................................	39	0	BF1
..........ugguuuaucuagucugucgaaacaaguAaauuugcaaaagucuu................................	1	1	BF1
..........ugguuuaucuagucugucgaaacaagugaauuugcaaaagucuu................................	18	0	BF1
............guuuaucuagucugucgaaacaagugaauuugcaaaagucu.................................	2	0	BF1
....ugucgacagagagauaaaucac...........	1	0	BF1
....ugucgacagagagauaaaucacC..........	1	1	BF1
....Cgucgacagagagauaaaucacu..........	1	1	BF1
....ugucgacagagagauaaaucacu..........	112	0	BF1
....gucgacagagagauaaauc.............	2	0	BF1
....gucgacagagagauaaauca............	1	0	BF1
....gucgacagagagauaaaucac...........	9	0	BF1
....gucgacagagGgauaaaucacu..........	1	1	BF1
....gucgacagagagauaaaucacu..........	705	0	BF1

```
         aga-mir-2940-star
                                             aga-mir-2940
cugccugcauugguuuaucuagucugucgaaacaagugaauuugcaaaagucuugucgacagagagauaaaucacuguuugugguc

......................................................gucgacagagagauaaauUacu..........    2    1    BF1
......................................................gucgaUagagagauaaaucacu..........    2    1    BF1
......................................................gucgacagagaAauaaaucacu..........    1    1    BF1
......................................................gucgacagagagauaaaucacC..........    2    1    BF1
......................................................gucgacagagagauaaaucacuU.........    3    1    BF1
......................................................gucgacagagagauaaaucacuC.........    1    1    BF1

..........ugguuuaucuagucugucgaaa......................................................    1    0    FW1
..........ugguuuaucuagucugucgaaac.....................................................    2    0    FW1
..........ugguuuaucuagucugucgaaacC....................................................    1    1    FW1
..........ugguuuaucuagucugucgaaaca....................................................    3    0    FW1
.....................................................ugucgacagagagauaaaucac...........    1    0    FW1
.....................................................ugucgacagagagauaaaucacu..........    18   0    FW1
......................................................gucgacagagagauaaaucac...........    2    0    FW1
......................................................gucgacagagagauaaaucacu..........    140  0    FW1
......................................................gucgGcagagagauaaaucacu..........    1    1    FW1
......................................................gucgacagagagauaaaucacuU.........    2    1    FW1
......................................................gucgacagagagauaaaucacugG........    1    1    FW1
..........................................................acagagagauaaaucacu..........    1    0    FW1

..........ugguuuaucuagucugucgaa.......................................................    1    0    MW1
..........ugguuuaucuagucugucgaaa......................................................    1    0    MW1
..........ugguuuaucuagucugucgaaac.....................................................    1    0    MW1
..........ugguuuaucuagucugucgaaaca....................................................    3    0    MW1
..........ugguuuaucuagucugucgaaacU....................................................    1    1    MW1
.....................................................ugucgacagagagauaaaucac...........    2    0    MW1
.....................................................ugucgacagagagauaaaucacu..........    34   0    MW1
......................................................gucgacagagagauaaauc.............    1    0    MW1
......................................................gucgacagagagauaaaucac...........    4    0    MW1
......................................................guUgacagagagauaaaucacu..........    1    1    MW1
......................................................gucgacagagagauaaaucacu..........    257  0    MW1
......................................................gucgacagagagauaaaucacuU.........    2    1    MW1
........................................................cgacagagagauaaaucacu..........    1    0    MW1
.........................................................gacagagagauaaaucacu..........    1    0    MW1

..........ugguuuaucuagucugucgaaac.....................................................    1    0    MW2
..........ugguuuaucuagucugucgaaaca....................................................    1    0    MW2
.....................................................ugucgacagagagauaaauc.............    1    0    MW2
.....................................................ugucgacagagagauaaaucacu..........    18   0    MW2
.....................................................ugucgacagagagauaaaucacuU.........    1    1    MW2
......................................................gucgacagagagauaaauc.............    1    0    MW2
......................................................gucgacagagagauaaaucac...........    3    0    MW2
......................................................gucgacagagagauaaaucacu..........    318  0    MW2
......................................................gucgacagagUgauaaaucacu..........    1    1    MW2
......................................................gucgacagagagauaaaucacuU.........    4    1    MW2

..........ugguuuaucuagucugucgaaa......................................................    1    0    TE2
..........ugguuuaucuagucugucgaaac.....................................................    15   0    TE2
..........ugguuuaucuagucugucgaaaca....................................................    3    0    TE2
..........ugguuuaucuagucugucgaaacC....................................................    1    1    TE2
.....................................................ugucgacagagagauaaaucac...........    2    0    TE2
.....................................................ugucgacagagagauaaaucacu..........    43   0    TE2
.....................................................ugucgacagagagauaaaucacuC.........    1    1    TE2
......................................................gucgacagagagauaaauca............    1    0    TE2
......................................................gucgacagagagauaaaucac...........    3    0    TE2
......................................................gucgacagagagauaaaucacG..........    1    1    TE2
......................................................gucgacagagUgauaaaucacu..........    1    1    TE2
......................................................gucgacagagagUuaaaucacu..........    1    1    TE2
......................................................gCcgacagagagauaaaucacu..........    2    1    TE2
......................................................gucgacagagagauaaaucacu..........    535  0    TE2
......................................................gucgacagagagauaaaucacuU.........    4    1    TE2
```

miRBase precursor : aga-mir-2944a-1
Total read count : 8884
aga-mir-2944a-1 read count : 4397
aga-mir-2944a-1-star read count : 45
aga-mir-2944a-2 read count : 4397
aga-mir-2944a-2-star read count : 45
remaining reads : 0

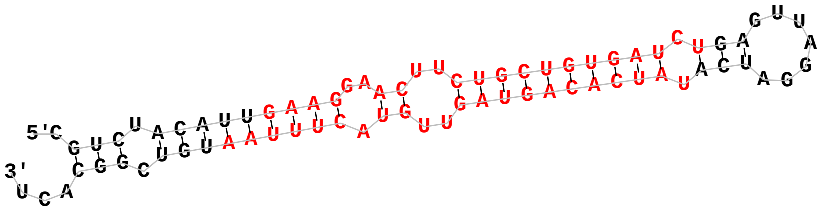

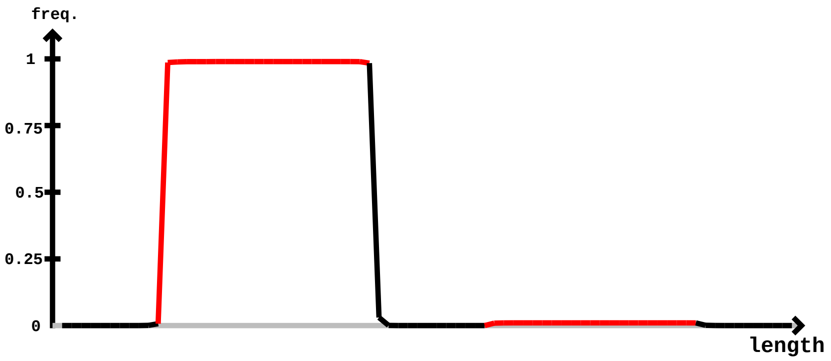


```
                                                aga-mir-2944a-1-star
          aga-mir-2944a-1
          aga-mir-2944a-2
                                                aga-mir-2944a-2-star
5'- cgucuacauugaaggaacuucugcugugaucugaguuaggaucauaucacaguaguuguacuuuaaugucggcacu   -3'    exp
    .(((.(((((((((..((..(((((((((.(((.......))).))))))))))..)).))))))))).)))...    reads      mm         sample
    ..........gaaggaacuucugcugugaucu............................................    8          0          OV2
    ..........gaaggaacuucugcugugaucug...........................................    1          0          OV2

    ..........gaaggaacuucugcugugaucu............................................    6          0          FF2

    ..........gaaggaacuucugcugugaucu............................................    14         0          TE1

    ........uCgaaggaacuucugcugugaucu............................................    1          1          MF2
    .........ugaaggaacuucugcugugaucu............................................    1          0          MF2
    ..........gaaggaacuucugcugugauc.............................................    2          0          MF2
    ..........gaaggaacuucugcugugaucu............................................    51         0          MF2
    ..........gaaggaacuucugcugugaucC............................................    1          1          MF2
    ..........gaaggaacuucugUugugaucu............................................    1          1          MF2
    ..........gaaggaacuucugcugugaucG............................................    1          1          MF2
    ..........gaaggaacuucugcuguAaucu............................................    1          1          MF2
    ..........gaaggaacuucugcugugaucuU...........................................    1          1          MF2
    ............................................uaucacaguaguuguacuuuaa..........    1          0          MF2

    ..........gaaggaacuucugcugugaucu............................................    4          0          FW2

    ..........gaaggaacuucugcugugauc.............................................    1          0          OV1
    ..........gaaggaacuucugcugugaucu............................................    7          0          OV1

    ..........gaaggaacuucugcugugauc.............................................    3          0          FF1
    ..........gaaUgaacuucugcugugaucu............................................    1          1          FF1
    ..........gaaggaacuucugcugugaucu............................................    14         0          FF1

    ..........gaaggaacuucugcugugaucu............................................    13         0          MF1

    ........uCgaaggaacuucugcugugaucu............................................    2          1          BF2
    .........Agaaggaacuucugcugugaucu............................................    4          1          BF2
    .........ugaaggaacuucugcugugaucu............................................    10         0          BF2
```

aga-mir-2944a-1-star

aga-mir-2944a-1

cgucuacauugaaggaacuucugcugugaucugaguuaggaucauaucacaguaguuguacuuuaaugucggcacu

aga-mir-2944a-2

aga-mir-2944a-2-star

.........Cgaaggaacuucugcugugaucu..	10	1	BF2
..........gaaggaacuucugcugugau..	1	0	BF2
..........gaaggaacuucugcugugauc...	13	0	BF2
..........gaaggaacCucugcugugaucu..	1	1	BF2
..........gaaggaacuucugcugugaucG..	10	1	BF2
..........gaaggaacuucCgcugugaucu..	4	1	BF2
..........gaaggaacuuGugcugugaucu..	1	1	BF2
..........gaaggaacuucugcugugauUu..	1	1	BF2
..........gaaggaacuuUugcugugaucu..	1	1	BF2
..........gaaggaacuucugcugugaucA..	1	1	BF2
..........gaaggaacuucGgcugugaucu..	1	1	BF2
..........gaaggaacuucAgcugugaucu..	2	1	BF2
..........gaaggaaUuucugcugugaucu..	2	1	BF2
..........Uaaggaacuucugcugugaucu..	1	1	BF2
..........gaaggaacuucugcugGgaucu..	1	1	BF2
..........gaaggaacuucugcugugaucu..	3486	0	BF2
..........gaaggCacuucugcugugaucu..	1	1	BF2
..........gaagCaacuucugcugugaucu..	1	1	BF2
..........gaaggaacuucugcugugauAu..	1	1	BF2
..........gaGggaacuucugcugugaucu..	1	1	BF2
..........gaaggaacuucugcuguAaucu..	4	1	BF2
..........gaaggaacuucugcugCgaucu..	1	1	BF2
..........Aaaggaacuucugcugugaucu..	2	1	BF2
..........gaaggaacuucugcugugaucug...	97	0	BF2
..........gaaggaacuucugcugugaucuU...	3	1	BF2
..........gaaggaacuucugcugugaucugU..	1	1	BF2
...........aaggaacuucugcugugaucu..	8	0	BF2
...........aaggaacuucugcugugaucug...	1	0	BF2
............aggaacuucugcugugaucu..	3	0	BF2
............aggaacuucugcugugaucuga..	1	0	BF2
...............aacuucugcugugaucu..	1	0	BF2
....uaucacaguaguuguacuuua...........	1	0	BF2
....uaucacaguaguuguacuuuaa..........	30	0	BF2
....uaucacaguaguuguacuuuaC..........	3	1	BF2
....uauUacaguaguuguacuuuaa..........	1	1	BF2
....uaucacaguaguuguacuuuaG..........	1	1	BF2
....uaucacaguaguuguacuuuaau.........	1	0	BF2
....uaucacaguaguuguacuuuaaC.........	1	1	BF2
....aucacaguaguuguacuuuaa..........	1	0	BF2
....aucacaguaguuguacuuuaG..........	1	1	BF2
....aucacaguaguuguacuuuaau.........	1	0	BF2
....aucacaguaguuguacuuuaaA.........	1	1	BF2
....ucacaguaguuguacuuuaaug........	1	0	BF2
.........ugaaggaacuucugcugugaucu..	2	0	BF1
.........Cgaaggaacuucugcugugaucu..	1	1	BF1
..........gaaggaacuucugcugugauc...	2	0	BF1
..........gaaggaacuucugcugugaucu..	499	0	BF1
..........gaaggaacuucugcuAugaucu..	1	1	BF1
..........gaaggaacuucugcugugGucu..	1	1	BF1
..........gaaggaacuuGugcugugaucu..	1	1	BF1
..........gaaggGacuucugcugugaucu..	1	1	BF1
..........gaaggaacuucCgcugugaucu..	1	1	BF1
..........gaagAaacuucugcugugaucu..	1	1	BF1
..........gaaggaacuucAgcugugaucu..	1	1	BF1
..........gaaggaacuucugcugugaucug...	27	0	BF1
..........gaaggaacuucugcugugaucuU...	1	1	BF1
...........aaggaacuucugcugugaucu..	1	0	BF1
............aggaacuucugcugugaucug...	1	0	BF1
....uaucacaguaguuguacuuuaa..........	1	0	BF1
..........gaaggaacuucugcugugaucu..	1	0	FW1
..........gaaggaacuucugcugugaucu..	6	0	MW1
..........gaaggaacuucugcugugaucu..	1	0	MW2
..........gaaggaacuucugcugugauc...	4	0	TE2

aga-mir-2944a-1-star

aga-mir-2944a-1

cgucuacauugaaggaacuucugcugugaucugaguuaggaucauaucacaguaguuguacuuuaaugucggcacu

aga-mir-2944a-2

aga-mir-2944a-2-star

..........gaaggaacuucugcugugaucu.. 34 0 TE2

miRBase precursor : aga-mir-2944a-2
Total read count : 8891
aga-mir-2944a-1 read count: 4397
aga-mir-2944a-1-star read count: 45
aga-mir-2944a-2 read count: 4397
aga-mir-2944a-2-star read count: 45
remaining reads : 7

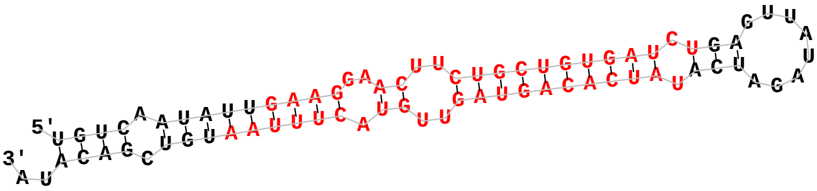

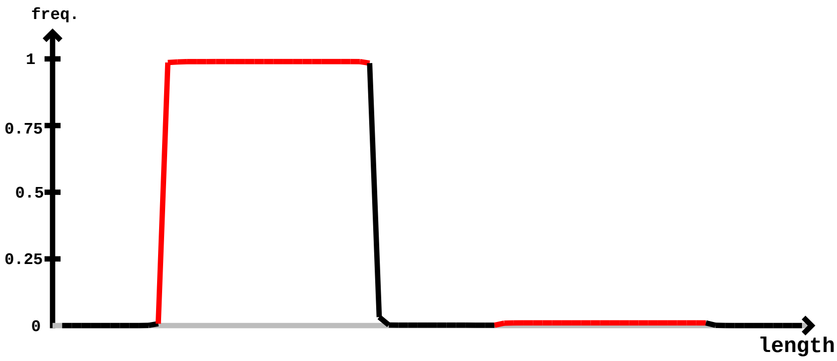


```
                                        aga-mir-2944a-1-star
          aga-mir-2944a-1
          aga-mir-2944a-2
                                        aga-mir-2944a-2-star
5'- ugucaauauugaaggaacuucugcugugaucugaguuauagaucauaucacaguaguuguacuuuaaugucgacaua  -3'    exp
    ((((.(((((((((..((..((((((((((.(((........))).)))))))))))..)).))))))))).)))).. reads      mm         sample
    .........gaaggaacuucugcugugaucu...........................................    8          0          OV2
    .........gaaggaacuucugcugugaucug..........................................    1          0          OV2

    .........gaaggaacuucugcugugaucu...........................................    6          0          FF2

    .........gaaggaacuucugcugugaucu...........................................    14         0          TE1

    ........uCgaaggaacuucugcugugaucu..........................................    1          1          MF2
    ........ugaaggaacuucugcugugaucu...........................................    1          0          MF2
    .........gaaggaacuucugcugugauc............................................    2          0          MF2
    .........gaaggaacuucugcuguAaucu...........................................    1          1          MF2
    .........gaaggaacuucugcugugaucu...........................................    51         0          MF2
    .........gaaggaacuucugcugugaucG...........................................    1          1          MF2
    .........gaaggaacuucugUugugaucu...........................................    1          1          MF2
    .........gaaggaacuucugcugugaucC...........................................    1          1          MF2
    .........gaaggaacuucugcugugaucuU..........................................    1          1          MF2
    ........................................uaucacaguaguuguacuuuaa..........    1          0          MF2

    .........gaaggaacuucugcugugaucu...........................................    4          0          FW2

    .........gaaggaacuucugcugugauc............................................    3          0          FF1
    .........gaaUgaacuucugcugugaucu...........................................    1          1          FF1
    .........gaaggaacuucugcugugaucu...........................................    14         0          FF1

    .........gaaggaacuucugcugugauc............................................    1          0          OV1
    .........gaaggaacuucugcugugaucu...........................................    7          0          OV1

    .........gaaggaacuucugcugugaucu...........................................    13         0          MF1

    ........uCgaaggaacuucugcugugaucu..........................................    2          1          BF2
    ........Cgaaggaacuucugcugugaucu...........................................    10         1          BF2
    ........Agaaggaacuucugcugugaucu...........................................    4          1          BF2
```

aga-mir-2944a-1-star

aga-mir-2944a-1

ugucaauauugaaggaacuucugcugugaucugaguuauagaucauaucacaguaguuguacuuuaaugucgacaua

aga-mir-2944a-2

aga-mir-2944a-2-star

.........ugaaggaacuucugcugugaucu...	10	0	BF2
..........gaaggaacuucugcugugau...	1	0	BF2
..........gaaggaacuucugcugugauc..	13	0	BF2
..........gaaggaacuucugcugugauAu...	1	1	BF2
..........gaaggCacuucugcugugaucu...	1	1	BF2
..........gaGggaacuucugcugugaucu...	1	1	BF2
..........gaaggaacuuGugcugugaucu...	1	1	BF2
..........Uaaggaacuucugcugugaucu...	1	1	BF2
..........gaaggaacuucugcugugaucu...	3486	0	BF2
..........gaaggaaUuucugcugugaucu...	2	1	BF2
..........gaaggaacuucGgcugugaucu...	1	1	BF2
..........gaaggaacuucugcugugauUu...	1	1	BF2
..........gaaggaacuuUugcugugaucu...	1	1	BF2
..........gaaggaacuucugcugCgaucu...	1	1	BF2
..........gaaggaacuucugcuguAaucu...	4	1	BF2
..........gaaggaacCucugcugugaucu...	1	1	BF2
..........gaaggaacuucugcugugaucG...	10	1	BF2
..........gaagCaacuucugcugugaucu...	1	1	BF2
..........gaaggaacuucCgcugugaucu...	4	1	BF2
..........gaaggaacuucAgcugugaucu...	2	1	BF2
..........gaaggaacuucugcugugaucA...	1	1	BF2
..........Aaaggaacuucugcugugaucu...	2	1	BF2
..........gaaggaacuucugcugGgaucu...	1	1	BF2
..........gaaggaacuucugcugugaucug..	97	0	BF2
..........gaaggaacuucugcugugaucuU..	3	1	BF2
..........gaaggaacuucugcugugaucugU...	1	1	BF2
..........gaaggaacuucugcugugaucugaguuauaga...................................	1	0	BF2
..........gaaggaacuucugcugugaucugaguuauagauca................................	2	0	BF2
...........aaggaacuucugcugugaucu...	8	0	BF2
...........aaggaacuucugcugugaucug..	1	0	BF2
............aggaacuucugcugugaucu...	3	0	BF2
............aggaacuucugcugugaucuga...	1	0	BF2
...............aacuucugcugugaucu...	1	0	BF2
....uaucacaguaguuguacuuua...........	1	0	BF2
....uaucacaguaguuguacuuuaC..........	3	1	BF2
....uaucacaguaguuguacuuuaa..........	30	0	BF2
....uaucacaguaguuguacuuuaG..........	1	1	BF2
....uauUacaguaguuguacuuuaa..........	1	1	BF2
....uaucacaguaguuguacuuuaau.........	1	0	BF2
....uaucacaguaguuguacuuuaaC.........	1	1	BF2
....aucacaguaguuguacuuuaG..........	1	1	BF2
....aucacaguaguuguacuuuaa..........	1	0	BF2
....aucacaguaguuguacuuuaaA.........	1	1	BF2
....aucacaguaguuguacuuuaau.........	1	0	BF2
....ucacaguaguuguacuuuaaug........	1	0	BF2
.........ugaaggaacuucugcugugaucu...	2	0	BF1
.........Cgaaggaacuucugcugugaucu...	1	1	BF1
..........gaaggaacuucugcugugauc..	2	0	BF1
..........gaaggaacuucAgcugugaucu...	1	1	BF1
..........gaaggaacuucugcugugaucu...	499	0	BF1
..........gaaggaacuuGugcugugaucu...	1	1	BF1
..........gaaggaacuucugcuAugaucu...	1	1	BF1
..........gaaggGacuucugcugugaucu...	1	1	BF1
..........gaaggaacuucCgcugugaucu...	1	1	BF1
..........gaagAaacuucugcugugaucu...	1	1	BF1
..........gaaggaacuucugcugugGucu...	1	1	BF1
..........gaaggaacuucugcugugaucuU..	1	1	BF1
..........gaaggaacuucugcugugaucug..	27	0	BF1
..........gaaggaacuucugcugugaucugaguuauagauca................................	3	0	BF1
..........gaagAaacuucugcugugaucugaguuauagauca................................	1	1	BF1
...........aaggaacuucugcugugaucu...	1	0	BF1
............aggaacuucugcugugaucug..	1	0	BF1
....uaucacaguaguuguacuuuaa..........	1	0	BF1
..........gaaggaacuucugcugugaucu...	1	0	FW1
..........gaaggaacuucugcugugaucu...	6	0	MW1

aga-mir-2944a-1-star

aga-mir-2944a-1

ugucaauauugaaggaacuucugcugugaucugaguuauagaucauaucacaguaguuguacuuuaaugucgacaua

aga-mir-2944a-2

aga-mir-2944a-2-star

..........gaaggaacuucugcugugaucu...	1	0	MW2
..........gaaggaacuucugcugugauc..	4	0	TE2
..........gaaggaacuucugcugugaucu...	34	0	TE2

miRBase precursor : aga-mir-2944b
Total read count : 3666
aga-mir-2944b read count : 3589
aga-mir-2944b-star read count : 77
remaining reads : 0

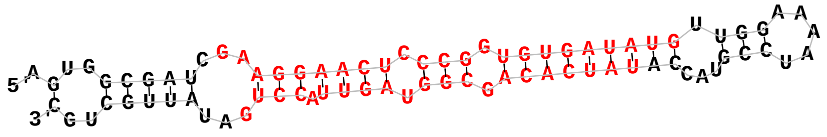

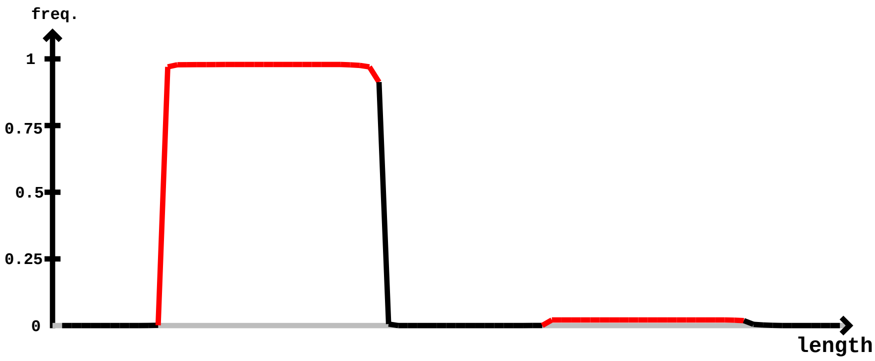


```
                                                  aga-mir-2944b-star
          aga-mir-2944b
5'- aguggcgaucgaaggaacucccggugugauauguuggaaaauccguaccauaucacagcgguaguuaccugauauugcugc  -3'    exp
    .(..(((((...(((((((.(((.(((((((((.(((.....)))...))))))))).))).)))).))).)))...)))))..)   reads    mm    sample
    ..........gaaggaacucccggugugau...................................................   1        0     FF2
    ..........gaaggaacucccggugugauaug................................................   2        0     FF2
    ..................................................uaucacagcgguaguuaccug..........   1        0     FF2
    ..................................................uaucacagcgguaguuaccugau........   2        0     FF2
    ..................................................uaucacagcgguaguuaccugaua.......   1        0     FF2

    ..........gaaggaacucccggugugau...................................................   1        0     OV2
    ..........gaaggaacucccggugugauaug................................................   1        0     OV2

    ..........gaaggaacucccggugugau...................................................   1        0     TE1
    ..........gaaggaacucccggugugauau.................................................   1        0     TE1
    ..........gaaggaacucccggugugauaug................................................   4        0     TE1

    ..........gaaggaacucccggugugau...................................................   2        0     MF2
    ..........gaaggaacucccggugugaua..................................................   1        0     MF2
    ..........gaaggaacucccggugugauau.................................................   1        0     MF2
    ..........gaaggaacucccggugugauaug................................................   3        0     MF2
    ..................................................uaucacagcgguaguuaccug..........   17       0     MF2
    ..................................................uaucacagcgguaguuaccuga.........   3        0     MF2

    ..........gaaggaacucccggugugauau.................................................   2        0     FW2
    ..................................................uaucacagcAguaguuaccu...........   1        1     FW2
    ..................................................uaucacagcgguaguuaccug..........   8        0     FW2

    ..........gaaggaacucccggugugauau.................................................   1        0     FF1
    ..................................................uaucacagcgAuaguuaccug..........   1        1     FF1
    ..................................................uaucacagcgguaguuaccug..........   7        0     FF1
    ..................................................uaucacagcgguaguuaccuga.........   1        0     FF1
    ..................................................uaucacagcgguaguuaccugau........   1        0     FF1

    ..........gaaggaacucccgguguga....................................................   1        0     OV1
    ..........gaaggaacucccggugugau...................................................   2        0     OV1
    ..........gaaggaacucccggugugauU..................................................   1        1     OV1
    ..........gaaggaacucccggugugauau.................................................   1        0     OV1
```

```
                                                  aga-mir-2944b-star
          aga-mir-2944b
aguggcgaucgaaggaacucccggugugauauguuggaaaauccguaccauaucacagcgguaguuaccugauauugcugc

..........gaaggaacucccggugugauaA.................................................    1      1      OV1

..........gaaggaacucccgguguga....................................................    1      0      MF1
..................................................uaucacagcgguaguuaccug..........    6      0      MF1
..................................................uaucacagcgguaguuaccuga.........    2      0      MF1

........Acgaaggaacucccggugugauaug................................................    1      1      BF2
.........Ggaaggaacucccggugugauaug................................................    1      1      BF2
.........cgaaggaacucccggugugauaug................................................    2      0      BF2
..........gaaggaacucccggugugaua..................................................    14     0      BF2
..........gaaggaacucccggugugauaG.................................................    1      1      BF2
..........gaaggaacucccggugCgauau.................................................    1      1      BF2
..........gaaggaacucccggugugauau.................................................    167    0      BF2
..........gaaggaacuccAggugugauaug................................................    1      1      BF2
..........gaaggaacucccggugugauaCg................................................    1      1      BF2
..........gaaggaacucccggugugauauU................................................    1      1      BF2
..........gaaggaacucccggugCgauaug................................................    1      1      BF2
..........gaaggaacucccggugGgauaug................................................    1      1      BF2
..........gaaggaacucccggCgugauaug................................................    1      1      BF2
..........gaaggaacucccggGgugauaug................................................    2      1      BF2
..........Aaaggaacucccggugugauaug................................................    1      1      BF2
..........gaaggaacucccgguUugauaug................................................    1      1      BF2
..........gaaggaUcucccggugugauaug................................................    1      1      BF2
..........gaaggaacuccGggugugauaug................................................    2      1      BF2
..........gaaggaacucAcggugugauaug................................................    1      1      BF2
..........gaaggaacuccUggugugauaug................................................    1      1      BF2
..........gaaggaacucccggugugauaug................................................    2589   0      BF2
..........gaaggaacucccgguguAauaug................................................    1      1      BF2
..........gaaggaacucccgguAugauaug................................................    1      1      BF2
..........gaaggaacCcccggugugauaug................................................    1      1      BF2
..........gaaggaacucccAgugugauaug................................................    1      1      BF2
..........gaaggaacucccgAugugauaug................................................    1      1      BF2
..........gaaggaacucccggugugauauA................................................    4      1      BF2
..........gaaggaacucccggugugauaugC...............................................    5      1      BF2
..........gaaggaacucccggugugauaugu...............................................    9      0      BF2
..........gaaggaacucccggugugauaugA...............................................    6      1      BF2
...........aaggaacucccggugugauaug................................................    25     0      BF2
...........aaggaacucccggugugauauU................................................    1      1      BF2
............aggaacucccggugugauaug................................................    1      0      BF2
.............ggaacucccggugugauaug................................................    1      0      BF2
...............aacucccggugugauaug................................................    1      0      BF2
................acucccggugugauaug................................................    1      0      BF2
................................................cauaucacagcAguaguuaccu...........    1      1      BF2
..................................................uaucacagcAguaguuacc............    3      1      BF2

..........gaaggaacucccggugugaua..................................................    2      0      BF1
..........gaaggaacucccggugugauau.................................................    30     0      BF1
..........gaaggaacucccggugGgauaug................................................    1      1      BF1
..........gaaggaacuUccggugugauaug................................................    1      1      BF1
..........gaaggaacucUcggugugauaug................................................    1      1      BF1
..........gaaggaacucccggugugauaug................................................    659    0      BF1
..........gaaggaGcucccggugugauaug................................................    1      1      BF1
..........gaaggaacucccAgugugauaug................................................    1      1      BF1
..........gaaggaacCcccggugugauaug................................................    1      1      BF1
..........gaaggaacucccggugugauaugA...............................................    1      1      BF1
...........aaggaacucccggugugauaug................................................    3      0      BF1

..................................................uaucacagcgguaguuaccug..........    5      0      MW1
..................................................uaucacagcgguaguuaccuga.........    2      0      MW1
..................................................uaucacagcgguaguuaccugau........    2      0      MW1
..................................................uaucacagcgguaguuaccugaua.......    2      0      MW1

..........gaaggaacucccggugugauau.................................................    1      0      FW1
..................................................uaucacagcgguaguuaccug..........    4      0      FW1

..................................................uaucacagcgguaguuaccug..........    2      0      MW2

..........gaaggaacucccgguguga....................................................    2      0      TE2
..........gaGggaacucccgguguga....................................................    1      1      TE2
```

```
                                                   aga-mir-2944b-star
          aga-mir-2944b
aguggcgaucgaaggaacucccggugugauauguuggaaaauccguaccauaucacagcgguaguuaccugauauugcugc

..........gaaggaacucccggugugau...................................................    1           0           TE2
..........gaaggaacucccggugugaua..................................................    2           0           TE2
..........gaaggaacucccggugugauau.................................................    2           0           TE2
..........gaaggaacucccggugugauaug................................................    1           0           TE2
..................................................uaucacagcAguaguuaccu...........    4           1           TE2
..................................................uaucacagcgguaguuaccuga.........    1           0           TE2
```

miRBase precursor : aga-mir-2945
Total read count : 1571
aga-mir-2945 read count : 1113
aga-mir-2945-star read count : 446
remaining reads : 12

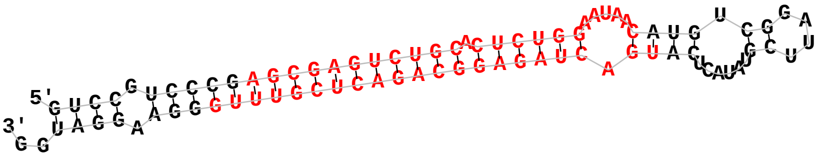

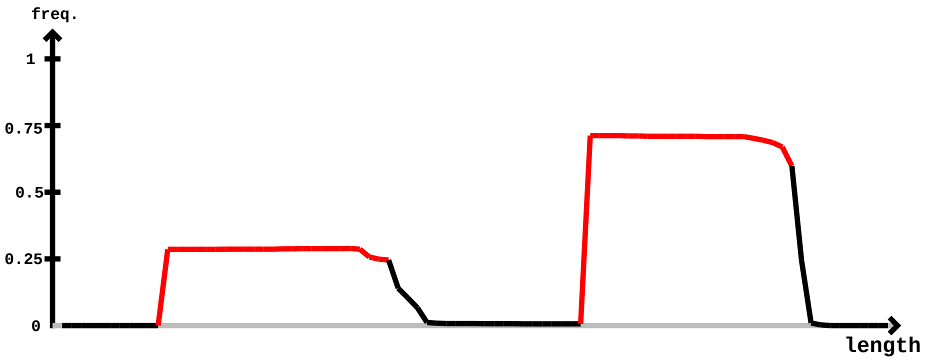


```
                                                                aga-mir-2945
           aga-mir-2945-star
5'- guccgucccgagcgagucugcacucuggaauaacaugucggauucguauacucaugacuagaggcagacucguuugggaaggaugg  -3'    exp
    ((((.((((((((((((((((.((((((.....((((.((....))......)))).)))))))))))))))))))))).)))).. reads      mm         sample
    .........agcgagucugcacucuggaau........................................................  2          0          TE1
    .........agcgagucugcacucuggaauaac.....................................................  2          0          TE1
    .........agcgagucugcacucuggaauaaca....................................................  1          0          TE1
    .........agcgagucugcacucuggaauaacau...................................................  5          0          TE1
    .........agcgagucugcacucuggaauaacaug..................................................  4          0          TE1
    .........agcgagucugcacucuggaauaacauguc................................................  1          0          TE1
    .........agcgagucugcacucuggaauaacaugucggauucguauacuca.................................  1          0          TE1
    .....................................................ugacuagaggcagacucg...............  1          0          TE1
    .....................................................ugacuagaggcagacucgu..............  2          0          TE1
    .....................................................ugacuagaggcagacucguu.............  1          0          TE1
    .....................................................ugacuagaggcagacucguuu............  3          0          TE1
    .....................................................ugacuagaggcagacucguuug...........  31         0          TE1
    .....................................................ugacuagaggcagacucguuuA...........  1          1          TE1
    .....................................................ugacuagaggcagacucguuugC..........  1          1          TE1
    .....................................................ugacuagaggcagacucguuugA..........  16         1          TE1
    .....................................................ugacuagaggcagacucguuugU..........  5          1          TE1
    .....................................................ugacuagaggcagacucguuugAg.........  1          1          TE1

    .........agcgagucugcacucuggaau........................................................  2          0          FF2
    .........agcgagucugcacucuggaauaac.....................................................  5          0          FF2
    .........agcgagucugcacucuggaauaacau...................................................  3          0          FF2
    .........agcgagucugcacucuggaauaacaug..................................................  4          0          FF2
    .....................................................ugacuagaggcagacuc................  1          0          FF2
    .....................................................ugacuagaggcagacucguu.............  2          0          FF2
    .....................................................ugacuagaggcagacucguuu............  2          0          FF2
    .....................................................ugacuagaggcagacucguuuU...........  1          1          FF2
    .....................................................ugacuagaggcagacucguuug...........  16         0          FF2
    .....................................................ugacuagaggcagacucguuugC..........  5          1          FF2
    .....................................................ugacuagaggcagacucguuugU..........  1          1          FF2
    .....................................................ugacuagaggcagacucguuugA..........  4          1          FF2

    .........agcgagucugcacucuggaau........................................................  1          0          OV2
    .........agcgagucugcacucuggaauaac.....................................................  3          0          OV2
    .........agcgagucugcacucuggaauaaca....................................................  2          0          OV2
```

aga-mir-2945

aga-mir-2945-star

```
guccgucccgagcgagucugcacucuggaauaacaugucggauucguauacucaugacuagaggcagacucguuugggaaggaugg

..........agcgagucugcacucuggaauaacau..................................................   2    0    OV2
..........agcgagucugcacucuggaauaacaug.................................................   2    0    OV2
......................................................ugacuagaggcagacucguuu...........   6    0    OV2
......................................................ugacuagaggcagacucguuug..........   30   0    OV2
......................................................ugacuagaggcagacucgCuug..........   1    1    OV2
......................................................ugacuagaggcagacucguuuU..........   1    1    OV2
......................................................ugacuagaggcagacucguuugC.........   4    1    OV2
......................................................ugacuagaggcagacucguuugU.........   6    1    OV2
......................................................ugacuagaggcagacucguuugA.........   6    1    OV2

..........agcgagucugcacucuggaau.......................................................   7    0    MF2
..........agcgagucugcacucuggaaua......................................................   2    0    MF2
..........agcgagucugcacucuggaauaa.....................................................   2    0    MF2
..........agcgagGcugcacucuggaauaac....................................................   1    1    MF2
..........agcgagucugcacucuggaauaac....................................................   68   0    MF2
..........agcgagucugcacucuggaauaaU....................................................   1    1    MF2
..........agcgagucugAacucuggaauaac....................................................   1    1    MF2
..........agcgagucugcacucuggaauaaca...................................................   24   0    MF2
..........agcgagucugcacucuggaauaacU...................................................   1    1    MF2
..........agcgagucugcacucuggaauaacau..................................................   13   0    MF2
..........agcgagucugcacucuggaauaacaA..................................................   1    1    MF2
..........agcgagucugcacucuggaauaacaug.................................................   32   0    MF2
..........agcgagucugcacucuggaauaacaugA................................................   1    1    MF2
......................cucuggaauaacaugucggauucguauacucaugac............................   2    0    MF2
........................cuggaauaacaugucggauucguauacucaugacuagaggca....................   1    0    MF2
......................................................ugacuagaggcagacuA...............   1    1    MF2
......................................................ugacuagaggcagacuc...............   1    0    MF2
......................................................ugacuagaggcagacucgu.............   1    0    MF2
......................................................ugacuagaggcagacucguuu...........   13   0    MF2
......................................................ugacuagaggcagacucguuug..........   99   0    MF2
......................................................ugacuagaggcagacucguuuU..........   1    1    MF2
......................................................ugacuagaggcaUacucguuug..........   1    1    MF2
......................................................ugacuagaggcagacucguuugC.........   22   1    MF2
......................................................ugacuagaggcagacucguuugU.........   55   1    MF2
......................................................ugacuagaggcagacucguuugA.........   20   1    MF2
......................................................ugacuagaggcagacucguuuAg.........   1    1    MF2
......................................................ugacuagaggcagacucguuugCg........   2    1    MF2
......................................................ugacuagaggcagacucguuugggC.......   1    1    MF2
......................................................ugacuagaggcagacucguuugUga.......   1    1    MF2

..........agcgagucugcacucuggaa........................................................   1    0    FW2
..........agcgagGcugcacucuggaau.......................................................   1    1    FW2
..........agcgagucugcacucuggaau.......................................................   5    0    FW2
..........agcgagucugcacucuggaauU......................................................   1    1    FW2
..........agcgagucugcacucuggaaua......................................................   1    0    FW2
..........agcgagucugcacucuggaauaac....................................................   18   0    FW2
..........agcgagucugcacucuggaauaaca...................................................   3    0    FW2
..........agcgagucugcacucuggaauaacau..................................................   11   0    FW2
..........agcgagucugcacucuggaauaacaug.................................................   11   0    FW2
..........agcgagucugcacucuggaauaacaugA................................................   1    1    FW2
..........agcgagucugcacucuggaauaacauguA...............................................   1    1    FW2
................ucugcacucuggaauaacaugucggauucguauacucaugacua..........................   1    0    FW2
......................................................ugacuagaggcagacucguuu...........   9    0    FW2
......................................................ugacuagaggcagacucguuuC..........   2    1    FW2
......................................................ugacuagaggcagacucguuug..........   18   0    FW2
......................................................ugacuagaggcagacucguuugU.........   5    1    FW2
......................................................ugacuagaggcagacucguuugC.........   1    1    FW2
......................................................ugacuagaggcagacucguuugA.........   1    1    FW2

..........agcgagucugcacucuggaauaaca...................................................   1    0    OV1
..........agcgagucugcacucuggaauaacau..................................................   1    0    OV1
..........agcgagucugcacucuggaauaacaug.................................................   1    0    OV1
......................................................ugacuagaggcagacuc...............   1    0    OV1
......................................................ugacuagaggcagacucguu............   4    0    OV1
......................................................ugacuagaggcagacucguuu...........   7    0    OV1
......................................................ugacuagaggcagacuAguuug..........   1    1    OV1
......................................................ugacuagaggAagacucguuug..........   1    1    OV1
......................................................ugacuagaggcagacucguuug..........   44   0    OV1
......................................................ugacuagaggcagacucguuuU..........   2    1    OV1
```

aga-mir-2945

aga-mir-2945-star

guccgucccgagcgagucugcacucuggaauaacaugucggauucguauacucaugacuagaggcagacucguuugggaaggaugg

....ugacuagaggcagacucgGuug..........	1	1	OV1
....ugacuagaggcagacucguuugU.........	22	1	OV1
....ugacuagaggcagacucguuugC.........	18	1	OV1
....ugacuagaggcagacucguuugA.........	11	1	OV1
....ugacuagaggcagacucguuugUg........	1	1	OV1
....ugacuagaggcagacucguuugAga.......	1	1	OV1
..........agcgagucugcacucuggaau...	1	0	FF1
..........agcgagucugcacucuggaauaa...	2	0	FF1
..........agcgagucugcacucuggaauaac..	16	0	FF1
..........agcgagucugcacucuggaauaacU...	1	1	FF1
..........agcgagucugcacucuggaauaaca...	8	0	FF1
..........agcgagucugcacucuggaauaacau..	3	0	FF1
..........agcgagucugcacucuggaauaacaug...	7	0	FF1
..........agcUagucugcacucuggaauaacaug...	1	1	FF1
.............................auaacaugucggauucguauacucaugacuagaggca....................	1	0	FF1
....ugacuagaggcagacucg..............	1	0	FF1
....ugacGagaggcagacucgu.............	1	1	FF1
....ugacuagaggcagacucguuu...........	2	0	FF1
....ugacuagaggcagacucUuuu...........	1	1	FF1
....ugacuagaggcagacucguuug..........	46	0	FF1
....Ggacuagaggcagacucguuug..........	1	1	FF1
....ugacGagaggcagacucguuug..........	3	1	FF1
....ugaGuagaggcagacucguuug..........	1	1	FF1
....ugacuagaggcagacucguuuU..........	1	1	FF1
....ugacuagaggcagacucguuugU.........	5	1	FF1
....ugacuagaggcagacucguuugA.........	2	1	FF1
....ugacuagaggcagacucguuugC.........	1	1	FF1
..........agcgagucugcacucuggaau...	1	0	MF1
..........agcgagucugcacucuggaauaac..	7	0	MF1
..........agcgagucugcacucuggaauaaA..	1	1	MF1
..........agcgagucugcacucuggaauaaca...	2	0	MF1
..........agcgagucugcacucuggaauaacau..	1	0	MF1
..........agcgagucugcacucuggaauaacaug...	3	0	MF1
....ugacuagaggcagacucg..............	1	0	MF1
....ugacuagaggcagacucgu.............	2	0	MF1
....ugacuagaggcagacucguu............	3	0	MF1
....ugacuagaggcagacucguuu...........	5	0	MF1
....ugacuagaggcagacucguuug..........	32	0	MF1
....ugacuagaggcagacucguuugU.........	11	1	MF1
....ugacuagaggcagacucguuugC.........	5	1	MF1
....ugacuagaggcagacucguuugA.........	4	1	MF1
..........agcgagucugcacucuggaau...	2	0	BF2
..........agcgagucugcacucuggaauaac..	5	0	BF2
..........agcgagucugcacucuggaauaacau..	5	0	BF2
..........agcgagucugcacucuggaauaacaug...	2	0	BF2
..........agcgagucugcacucuggaauaacaugucggau...	1	0	BF2
....ugacuagaggcagacuc...............	1	0	BF2
....ugacuagaggcagacucg..............	1	0	BF2
....ugacuagaggcagacucgu.............	1	0	BF2
....ugacuagaggcagacucguu............	2	0	BF2
....ugacuagaggcagacucguuu...........	19	0	BF2
....ugacuagaggcagacucguuug..........	45	0	BF2
....ugacuagaggcagacucguuuU..........	1	1	BF2
....ugacuagaggcagacucguuugA.........	12	1	BF2
....ugacuagaggcagacucguuugC.........	6	1	BF2
....ugacuagaggcagacucguuugU.........	40	1	BF2
....ugacuagaggcagacucguuugAg........	1	1	BF2
..........agcgagucugcacucuggaauaac..	1	0	BF1
..........agcgagucugcacucuggaauaaca...	3	0	BF1
..........agcgagucugcacucuggaauaacaug...	3	0	BF1
.....................................ucggauucguauacuca................................	1	0	BF1
....ugacuagaggcagacucgu.............	1	0	BF1
....ugacuagaggcagacucguu............	1	0	BF1
....ugacuagaggcagacucguuu...........	2	0	BF1
....ugacuagaggcagacucguuug..........	25	0	BF1

```
                                                      aga-mir-2945
          aga-mir-2945-star
guccgucccgagcgagucugcacucuggaauaacaugucggauucguauacucaugacuagaggcagacucguuugggaaggaugg

......................................................ugacuagaggcagacucguuugU.........  5    1    BF1
......................................................ugacuagaggcagacucguuugA.........  5    1    BF1
......................................................ugacuagaggcagacucguuugC.........  4    1    BF1
......................................................ugacuagaggcagacucguuuggU........  1    1    BF1

..........agcgagucugcacucuggaau.......................................................  11   0    FW1
..........agcgagucugcacucuggaaua......................................................  3    0    FW1
..........agcgagucugcacucuggaauaac....................................................  12   0    FW1
..........agcgagucugcacucuggaauaaca...................................................  2    0    FW1
..........agcgagucugcacucuggaauaacau..................................................  7    0    FW1
..........agcgagucugcacucuggaauaacaug.................................................  12   0    FW1
......................................................ugacuagaggcagacucguu............  1    0    FW1
......................................................ugacuagaggcagacucguuu...........  2    0    FW1
......................................................ugacuagaggcagacucguuug..........  16   0    FW1
......................................................ugacuagaggcagacucguuugU.........  3    1    FW1
......................................................ugacuagaggcagacucguuugUga.......  1    1    FW1

..........agcgagucugcacucuggaa........................................................  3    0    MW1
..........agcgagucugcacuUggaau.......................................................  1    1    MW1
..........agcgagucugcacucuggaau.......................................................  5    0    MW1
..........agcgagucugcacucuggaaua......................................................  4    0    MW1
..........agcgagucugcacucuggaauaac....................................................  18   0    MW1
..........agcgagucugcacucuggaauaaca...................................................  3    0    MW1
..........agcgagucugcacucuggaauaaAa...................................................  1    1    MW1
..........agcgagucugcacucuggaauaacau..................................................  4    0    MW1
..........agcgagucugcacucuggaauaacaug.................................................  9    0    MW1
..........agcgagucugcacucuggaauaacaugu................................................  1    0    MW1
..........agcgagucugcacucuggaauaacaugC................................................  1    1    MW1
..........agcgagucugcacucuggaauaacaugucggauucgu.......................................  1    0    MW1
.....................................ucggauucguauacucaugacua..........................  1    0    MW1
......................................................ugacuagaggcagacucgu.............  1    0    MW1
......................................................ugacuagaggcagacucguu............  1    0    MW1
......................................................ugacuagaggcagacucguuu...........  15   0    MW1
......................................................ugacuagaggUagacucguuu...........  1    1    MW1
......................................................ugacuagaggcagacucguuuU..........  1    1    MW1
......................................................ugacuagaggcagacucguuug..........  28   0    MW1
......................................................ugacuagaggcagacucguuugU.........  6    1    MW1
......................................................ugacuagaggcagacucguuugA.........  2    1    MW1
......................................................ugacuagaggcagacucguuugC.........  4    1    MW1
......................................................ugacuagaggcagacucguuugAg........  3    1    MW1
......................................................ugacuagaggcagacucguuuggU........  1    1    MW1

..........agcgagucugcacucuggaau.......................................................  1    0    MW2
..........agcgagucugcacucuggaaua......................................................  2    0    MW2
..........agcgagucugcacucuggaauaac....................................................  4    0    MW2
..........agcgagucugcacucuggaauaaca...................................................  4    0    MW2
..........agcgagucugcacucuggaauaacau..................................................  1    0    MW2
......................................................ugacuagaggcagacucguuu...........  2    0    MW2
......................................................ugacuagaggcagacucguuug..........  17   0    MW2

..........agcgagucugcacucuggaau.......................................................  6    0    TE2
..........agcgagucugcacucuggaauaa.....................................................  1    0    TE2
..........agcgagucugcacucuggaauaac....................................................  3    0    TE2
..........agcgagucugcacucuggaauaaca...................................................  1    0    TE2
..........agcgagucugcacucuggaauaacau..................................................  1    0    TE2
.....................................ucggauucguauacuca................................  2    0    TE2
......................................................ugacuagaggcagacuc...............  5    0    TE2
......................................................uAacuagaggcagacucg..............  1    1    TE2
......................................................ugacuagaggcagacucg..............  6    0    TE2
......................................................ugacuagaggcagacucgu.............  5    0    TE2
......................................................ugacuagaggcagacucguu............  11   0    TE2
......................................................ugacuagaggcagacucguuu...........  23   0    TE2
......................................................ugacuagaggcagacucguuuA..........  1    1    TE2
......................................................ugacuagaggcagacucguuug..........  84   0    TE2
......................................................ugacuagaggcagacucguuugU.........  6    1    TE2
......................................................ugacuagaggcagacucguuugC.........  5    1    TE2
......................................................ugacuagaggcagacucguuugA.........  42   1    TE2
......................................................ugacuagaggcagacucguuugAg........  1    1    TE2
```

miRBase precursor : aga-mir-2b
Total read count : 35831
aga-mir-2b read count : 32922
aga-mir-2b-star read count : 2886
remaining reads : 23

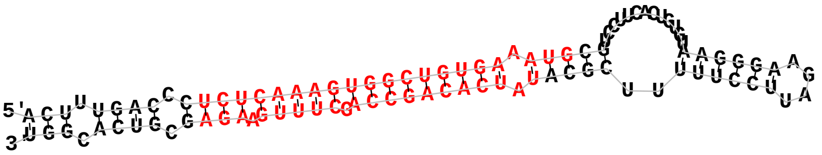

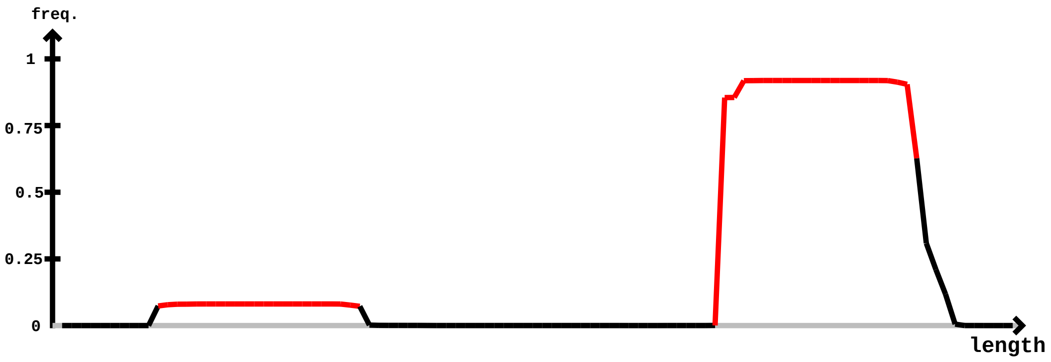

aga-mir-2b-star

aga-mir-2b

```
5'- acuuugacccucucaaaguggcugugaaaugcguccuucacuguguaagggaagauuccuuuuucgcauaucacagccagcuuugaagagcgucacggu   -3'    exp
    (((.((((.((((((((((((((((((.(((((.............((((((....))))))..))))).)))))))))).))))).)))).)))).)))    reads   mm   sample
    .........cucucaaaguggcugugaaau.....................................................................    1       0    TE1
    .........cucucaaaguggcugugaaaug....................................................................    12      0    TE1
    ..........ucucaaaguUgcugugaaaug....................................................................    1       1    TE1
    ..........ucucaaaguggcugugaaaug....................................................................    3       0    TE1
    .............caaaguggcugugaaauA....................................................................    2       1    TE1
    ....................................................................uaucacagccagcuuuga.............    4       0    TE1
    ....................................................................uaucacagccagcuuugaa............    1       0    TE1
    ....................................................................uaucacagccagcuuugGag...........    1       1    TE1
    ....................................................................uaucacagccagcuuugaaA...........    1       1    TE1
    ....................................................................uaucacagccagcuuugaUg...........    7       1    TE1
    ....................................................................uaucacagccagcuuugaag...........    77      0    TE1
    ....................................................................Caucacagccagcuuugaaga..........    2       1    TE1
    ....................................................................uaucacagccagcuuugaaAa..........    1       1    TE1
    ....................................................................uauUacagccagcuuugaaga..........    1       1    TE1
    ....................................................................uaucacagccagcuuugaaga..........    196     0    TE1
    ....................................................................uaucacagccaUcuuugaaga..........    1       1    TE1
    ....................................................................uaucacagccagcuuugaUga..........    10      1    TE1
    ....................................................................uaucacagccagcuuuAaaga..........    1       1    TE1
    ....................................................................uaucacagccagcuuugaUgag.........    15      1    TE1
    ....................................................................uaucacagccagcuuugaagag.........    132     0    TE1
    ....................................................................uaucacagccagcuuugaagaA.........    1       1    TE1
    ....................................................................uaucacagccagcuuugaUgagc........    45      1    TE1
    ....................................................................uaucacagccagcuuugaagagA........    31      1    TE1
    ....................................................................uaucacagccagcuuugaagagc........    76      0    TE1
    ....................................................................uaucacagccagcuuugaagagcU.......    2       1    TE1
    ....................................................................uaucacagccagcuuugaaAagcg.......    1       1    TE1
    ....................................................................uaucacagccagcuuugaagagcg.......    190     0    TE1
    ....................................................................uaucacagccagcuuugaagagcA.......    2       1    TE1
    ....................................................................uaucacagccagcuuugaUgagcg.......    1       1    TE1
    ....................................................................uaucacagccagcuuugaagagcgu......    6       0    TE1
    ....................................................................uaucacagccagcuuugaagagcgA......    3       1    TE1
    .....................................................................aucacagccagcuuugaaga..........    1       0    TE1
    ......................................................................ucacagccagcuuugaUg...........    1       1    TE1
    ......................................................................ucacagccagcuuugaUga..........    1       1    TE1
```

```
          aga-mir-2b-star
                                                            aga-mir-2b
acuuugacccucucaaaguggcugugaaaugcguccuucacuguguaagggaagauuccuuuuucgcauaucacagccagcuuugaagagcgucacggu

......................................................................ucacagccagcuuugaUgag.........    29    1    TE1
......................................................................ucacagccagcuuugaUgagc........    72    1    TE1
........................................................................acagccagcuuugaagagcg.......    1     0    TE1

.........cucucaaaguggcugugaaa......................................................................    8     0    FF2
.........cucucaaaguggcugugaaau.....................................................................    11    0    FF2
.........cucucaaaguggcugugaaauA....................................................................    1     1    FF2
.........cucucaaaguUgcugugaaaug....................................................................    1     1    FF2
.........cucucaaaguggcugugaaaug....................................................................    161   0    FF2
.........cucucaaaguggcuguAaaaug....................................................................    1     1    FF2
.........cucucaaaguggcugugaaauU....................................................................    2     1    FF2
.........cucucaaaguggcugugGaaug....................................................................    1     1    FF2
.........cucucaaaguggcugugaaaugU...................................................................    3     1    FF2
.........cucucaaaguggcugugaaaugA...................................................................    2     1    FF2
..........ucucaaaguggcugugaaaug....................................................................    2     0    FF2
...........cucaaaguggcugugaaaug....................................................................    2     0    FF2
............ucaaaguggcugugaaau.....................................................................    2     0    FF2
...................................................................Cuaucacagccagcuuugaag...........    1     1    FF2
....................................................................uaucacagccagcuuuga.............    4     0    FF2
....................................................................uaucacagccagcuuugaU............    1     1    FF2
....................................................................uaucacagccagcuuugaa............    13    0    FF2
....................................................................uaucacagccagcuuugaaU...........    3     1    FF2
....................................................................uaucacagccGgcuuugaag...........    1     1    FF2
....................................................................uaucacagccagcuuugaag...........    395   0    FF2
....................................................................uaucacagccagcuuugaUg...........    15    1    FF2
....................................................................uaucacaCccagcuuugaag...........    2     1    FF2
....................................................................uaucacagccagcuuugaaA...........    2     1    FF2
....................................................................uauAacagccagcuuugaaga..........    1     1    FF2
....................................................................uaucacagccagcuuugaUga..........    35    1    FF2
....................................................................uaucacagccagcuuugaaAa..........    1     1    FF2
....................................................................uaucacGgccagcuuugaaga..........    1     1    FF2
....................................................................uaucacagccCgcuuugaaga..........    1     1    FF2
....................................................................uaucacagccagcuuugaaga..........    328   0    FF2
....................................................................uaucacagccagcuuugaagaA.........    5     1    FF2
....................................................................uaucacagccagcuuugaagaC.........    1     1    FF2
....................................................................uaucacagccagcuuugaUgag.........    22    1    FF2
....................................................................uaucacagccagcuuugaagag.........    47    0    FF2
....................................................................uaucacagccagcuuugaUgagc........    19    1    FF2
....................................................................uaucacagccagcuuugaagagA........    9     1    FF2
....................................................................uaucacagccagcuuugaagagc........    17    0    FF2
....................................................................uaucacagccagcuuugaagagcU.......    1     1    FF2
....................................................................uaucacagccagcuuugaagagcg.......    129   0    FF2
....................................................................uaucacagccagcuuugaagagcgu......    2     0    FF2
.....................................................................aucacagccagcuuugaag...........    1     0    FF2
......................................................................ucacagccagcuuugaUgag.........    21    1    FF2
......................................................................ucacagccagcuuugaUgagc........    42    1    FF2
......................................................................ucacagccagcuuugaUgagcg.......    2     1    FF2

.........cucucaaaguggcugugaa.......................................................................    1     0    OV2
.........cucucaaaguggcugugaaa......................................................................    6     0    OV2
.........cucucaaaguggcugugaaau.....................................................................    1     0    OV2
.........cucucaaaguggcugugaaaA.....................................................................    1     1    OV2
.........cucucaaaguggcugugaaaug....................................................................    53    0    OV2
..........ucucaaaguggcugugaaa......................................................................    1     0    OV2
..........ucucaaaguggcugugaaau.....................................................................    2     0    OV2
..........ucucaaaguggcugugaaaug....................................................................    8     0    OV2
..........ucucaaaguggcugugaaaugcguccu..............................................................    1     0    OV2
...........cucaaaguggcugugaaa......................................................................    1     0    OV2
...........cucaaaguggcugugaaaug....................................................................    8     0    OV2
...................................................................Cuaucacagccagcuuugaag...........    2     1    OV2
...................................................................Cuaucacagccagcuuugaaga..........    1     1    OV2
...................................................................auaucacagccagcuuugaagagcg.......    1     0    OV2
....................................................................uaucacagccagcuuuga.............    10    0    OV2
....................................................................uaucacagccagcuuugaa............    19    0    OV2
....................................................................uaucacagccagcuuugaU............    1     1    OV2
....................................................................uaucacagccagcuuuUaag...........    1     1    OV2
....................................................................uaucacagUcagcuuugaag...........    4     1    OV2
....................................................................uaucacagccagUuuugaag...........    1     1    OV2
....................................................................uaucacagccagcuuugaaA...........    19    1    OV2
```

aga-mir-2b-star

aga-mir-2b

acuuugacccucucaaaguggcugugaaaugcguccuucacuguguaagggaagauuccuuuuucgcauaucacagccagcuuugaagagcgucacggu

....uauUacagccagcuuugaag...........	1	1	OV2
....uaucacagccagcuuuAaag...........	1	1	OV2
....uaucacagccagcuuugaaU...........	7	1	OV2
....uaucacagccagcuuugaaC...........	2	1	OV2
....uaucacagccagcuuugaag...........	784	0	OV2
....uaucacagccagcuuugaUg...........	57	1	OV2
....uaucGcagccagcuuugaaga..........	1	1	OV2
....uaucacagccagcuuugaaUa..........	1	1	OV2
....uaucacagccagcuuugaagU..........	4	1	OV2
....uaucacagccCgcuuugaaga..........	1	1	OV2
....uaucacagccagcuuugaUga..........	67	1	OV2
....uaucacagccagcuuugaaga..........	1641	0	OV2
....uaucacagccagcuuugaagG..........	1	1	OV2
....uaucacagccUgcuuugaaga..........	1	1	OV2
....uaucacagccagcCuugaaga..........	2	1	OV2
....uaucacagccagcuuuAaaga..........	2	1	OV2
....uaucacagcAagcuuugaaga..........	1	1	OV2
....Caucacagccagcuuugaaga..........	1	1	OV2
....uaucacagccagcuuugaaAa..........	1	1	OV2
....uaucacagccagcuuugaagag.........	99	0	OV2
....uaucacagccagcuuugaagaA.........	44	1	OV2
....uaucacagccagcuuugaagaC.........	2	1	OV2
....uaucacagccagcuCugaagag.........	1	1	OV2
....uaucacagccagcuuugaagaU.........	5	1	OV2
....uaucacagccagcuuugaUgag.........	42	1	OV2
....uaucacagccagcuuugaagagc........	23	0	OV2
....uaucacagccagcuuugaagagA........	17	1	OV2
....uaucacagccagcuuugaUgagc........	35	1	OV2
....uaucacagccagcuuugaagagcg.......	105	0	OV2
....uaucacagccagcuuugaagagcU.......	1	1	OV2
....uaucacagccagcuuugaagagcgu......	17	0	OV2
....uaucacagccagcuuugaagagcgC......	2	1	OV2
....aucacagccagcuuugaUgagc........	2	1	OV2
....ucacagccagcuuugaUg...........	1	1	OV2
....ucacagccagcuuugaaga..........	1	0	OV2
....ucacagccagcuuugaUga..........	1	1	OV2
....ucacagccagcuuugaUgag.........	59	1	OV2
....ucacagccagcuuugaUgagc........	169	1	OV2
....ucacagccagcuuugaagagcg.......	2	0	OV2
....ucacagccagcuuugaUgagcg.......	2	1	OV2
....acagccagcuuugaaga..........	2	0	OV2
....acagccagcuuugaUgagc........	1	1	OV2
.........cucucaaaguggcuguAaaa..	1	1	MF2
.........cucucaaaguggcugugaaa..	60	0	MF2
.........cucucaaaguggcugugaaau...	69	0	MF2
.........cucucaaaguggcugugaaaug..	1195	0	MF2
.........cucucaaaguggcugugaaauC..	1	1	MF2
.........cucucUaaguggcugugaaaug..	1	1	MF2
.........cucucaaagugCcugugaaaug..	2	1	MF2
.........cucucaaagCggcugugaaaug..	1	1	MF2
.........cucCcaaaguggcugugaaaug..	2	1	MF2
.........cucucaaaguggcugugaaauU..	14	1	MF2
.........cucucaaagGggcugugaaaug..	3	1	MF2
.........cucuUaaaguggcugugaaaug..	1	1	MF2
.........cucucaaUguggcugugaaaug..	1	1	MF2
.........cuGucaaaguggcugugaaaug..	1	1	MF2
.........cucucaaaguggcugugaaauA..	1	1	MF2
.........cucucaaaguggcugugaaaugU...	9	1	MF2
.........cucucaaaguggcugugaaaugc...	3	0	MF2
.........cucucaaaguggcugugaaaugA...	8	1	MF2
.........cucucaaaguggcugugaaaugcg..	2	0	MF2
.........cucucaaaguggcugugaaaugcguc..	1	0	MF2
.........cucucaaaguggcugugaaaugcguccu..	3	0	MF2
.........cucucaaaguggcugugaaaugcguccuu...	4	0	MF2
..........ucucaaaguggcuguga..	1	0	MF2
..........ucucaaaguggcugugaaau...	4	0	MF2
..........ucCcaaaguggcugugaaaug..	1	1	MF2
..........ucucaaaguggcugugaaaug..	63	0	MF2

```
          aga-mir-2b-star
                                                                    aga-mir-2b
acuuugacccucucaaaguggcugugaaaugcguccuucacuguguaagggaagauuccuuuuucgcauaucacagccagcuuugaagagcgucacggu

..........ucucaaaguggcugugaaaugU...................................................................   1      1    MF2
..........ucucaaaguggcugugaaaugcg..................................................................   1      0    MF2
...........cucaaaguggcugugaaaug....................................................................   17     0    MF2
...........cucaaaguggcugugaaaugA...................................................................   1      1    MF2
...........cucaaaguggcugugaaaugcguccuucacu.........................................................   1      0    MF2
............ucaaaguggcugugaaaug....................................................................   3      0    MF2
.............caaaguggcugugaaaug....................................................................   3      0    MF2
.............caaaguggcugugaaaugcU..................................................................   1      1    MF2
...................................................................Cuaucacagccagcuuugaag...........   1      1    MF2
...................................................................Cuaucacagccagcuuugaaga..........   1      1    MF2
...................................................................auaucacagccagcuuugaUga..........   1      1    MF2
...................................................................auaucacagccagcuuugaagagcU.......   1      1    MF2
....................................................................uaucacagccagcuuug..............   1      0    MF2
....................................................................uaucacagccagcuuuga.............   34     0    MF2
....................................................................uaucacagccagcuuugaa............   35     0    MF2
....................................................................uaucacagccagcuuugaC............   1      1    MF2
....................................................................uaucacagccagcuuugaU............   11     1    MF2
....................................................................Caucacagccagcuuugaag...........   2      1    MF2
....................................................................uaucacagccagcuuugaaC...........   7      1    MF2
....................................................................uaucacagccagcuuuAaag...........   2      1    MF2
....................................................................uaucacagcUagcuuugaag...........   1      1    MF2
....................................................................uaucacagccagcuuugaaU...........   6      1    MF2
....................................................................uauUacagccagcuuugaag...........   2      1    MF2
....................................................................uaucacagccagcuuugaaA...........   11     1    MF2
....................................................................uaucacagccagAuuugaag...........   1      1    MF2
....................................................................uaucacagccagcuCugaag...........   1      1    MF2
....................................................................uaucacagccagcuuugaag...........   1894   0    MF2
....................................................................uaucacaAccagcuuugaag...........   2      1    MF2
....................................................................uaucacagccagcuuugaUg...........   111    1    MF2
....................................................................uaucacagccagcuuuUaag...........   1      1    MF2
....................................................................uaucacagccagcuuuCaag...........   1      1    MF2
....................................................................uaucacagAcagcuuugaaga..........   1      1    MF2
....................................................................uaucacagccagcuuugaaAa..........   3      1    MF2
....................................................................uauUacagccagcuuugaaga..........   1      1    MF2
....................................................................uaucacagccUgcuuugaaga..........   1      1    MF2
....................................................................uaucacagccagcuuugaagG..........   2      1    MF2
....................................................................uaucacagccagcuuugaCga..........   1      1    MF2
....................................................................uaucacagccagcuuugaUga..........   207    1    MF2
....................................................................uCucacagccagcuuugaaga..........   1      1    MF2
....................................................................uaucacaUccagcuuugaaga..........   1      1    MF2
....................................................................uaucacagccagcuuugaagU..........   2      1    MF2
....................................................................uaucacagcUagcuuugaaga..........   1      1    MF2
....................................................................uaucacagccagcuuuAaaga..........   3      1    MF2
....................................................................uaucacagccagcCuugaaga..........   1      1    MF2
....................................................................uaucacagccaCcuuugaaga..........   2      1    MF2
....................................................................uaucacagccagcuuugaaga..........   1683   0    MF2
....................................................................uaucacagccagcuuCgaaga..........   1      1    MF2
....................................................................uaucacagccGgcuuugaaga..........   1      1    MF2
....................................................................uaucacagccagcuuugaagaU.........   4      1    MF2
....................................................................uaucacagccagcuuugaagaC.........   5      1    MF2
....................................................................uaucacagcUagcuuugaagag.........   1      1    MF2
....................................................................uaucacagccagcuuuAaagag.........   1      1    MF2
....................................................................uaucacagccagcuuugaagag.........   329    0    MF2
....................................................................uaucacagccagcuuugaagaA.........   29     1    MF2
....................................................................uaucacagccagcuuugaUgag.........   127    1    MF2
....................................................................uaCcacagccagcuuugaagag.........   1      1    MF2
....................................................................uaucGcagccagcuuugaagagc........   1      1    MF2
....................................................................uaucacagccagcuuugaagagA........   36     1    MF2
....................................................................uaucacagccagcuuugaagaUc........   2      1    MF2
....................................................................uaucacagccaAcuuugaagagc........   1      1    MF2
....................................................................uaucacagccagcuuugaagagc........   141    0    MF2
....................................................................uaucacagccagcuuugaUgagc........   150    1    MF2
....................................................................uaucacagccagcCuugaagagc........   1      1    MF2
....................................................................uaucacagccagcuuugaagaAc........   1      1    MF2
....................................................................uaucacagccagcuuuCaagagc........   1      1    MF2
....................................................................uaucacagccaUcuuugaagagc........   1      1    MF2
....................................................................uaucacagccagcuuugaUgagcg.......   3      1    MF2
....................................................................uaucacagccagcuuugaagagcU.......   13     1    MF2
....................................................................uaucacagccGgcuuugaagagcg.......   1      1    MF2
```

```
          aga-mir-2b-star
                                                                   aga-mir-2b
acuuugacccucucaaaguggcugugaaaugcguccuucacuguguaagggaagauuccuuuuucgcauaucacagccagcuuugaagagcgucacggu

...................................................................Caucacagccagcuuugaagagcg........   1     1    MF2
...................................................................uaucacagccagcuuugaagagcA........   4     1    MF2
...................................................................uaucacagccagcuuugaagagcg........   940   0    MF2
...................................................................uaucacGgccagcuuugaagagcg........   1     1    MF2
...................................................................uaucacagccagcuuugaagaCcg........   1     1    MF2
...................................................................uGucacagccagcuuugaagagcg........   1     1    MF2
...................................................................uauUacagccagcuuugaagagcg........   1     1    MF2
...................................................................uaucacagccagcuuugaaAagcg........   1     1    MF2
...................................................................uaucacagccagcuuugaagaUcg........   1     1    MF2
...................................................................uaucacagccaUcuuugaagagcg........   1     1    MF2
...................................................................uaucacagccagcuuugaGgagcg........   1     1    MF2
...................................................................uaAcacagccagcuuugaagagcg........   2     1    MF2
...................................................................uaucacagccagcuuugGagagcg........   2     1    MF2
...................................................................uaucaUagccagcuuugaagagcg........   1     1    MF2
...................................................................uaucacagccagcuuugaagaAcg........   1     1    MF2
...................................................................uaucacagccagcuuuAaagagcg........   1     1    MF2
...................................................................uaucacagcUagcuuugaagagcg........   1     1    MF2
...................................................................uaucacagccaCcuuugaagagcg........   1     1    MF2
...................................................................uaucacagUcagcuuugaagagcg........   2     1    MF2
...................................................................uaucacagccagcuuugaagagcgu.......   33    0    MF2
...................................................................uaucacagccagcuuugaagagcgC.......   6     1    MF2
...................................................................uaucacagccagcuuuUaagagcgu.......   1     1    MF2
...................................................................uaucacagccagcuuugaagagcguAa.....   1     1    MF2
...................................................................uaucacagccagcuuugaagagcguUac....   1     1    MF2
....................................................................aucacagccagcuuugaaga...........   1     0    MF2
....................................................................aucacagccagcuuugaUgagc.........   3     1    MF2
.....................................................................ucacagccagcuuugaag............   1     0    MF2
.....................................................................ucacagccagcuuugaUg............   1     1    MF2
.....................................................................ucacagccagcuuugaUga...........   2     1    MF2
.....................................................................ucacagccagcuuugaUgag..........   108   1    MF2
.....................................................................ucacagccagcuuugaUgagc.........   244   1    MF2
.....................................................................ucacagccagcuuugaagagcA........   1     1    MF2
.....................................................................ucacagccagcuuugaUgagcg........   3     1    MF2
......................................................................cacagccagcuuugaUgagc.........   1     1    MF2

.........cucucaaaguggcugugaa.......................................................................   1     0    FW2
.........cucucaaaguggcugugaaa......................................................................   3     0    FW2
.........cucucaaaguggcugugaaau.....................................................................   6     0    FW2
.........cucucaaaguggcugugaaaug....................................................................   93    0    FW2
.........cucucaaaguggcugugaaauA....................................................................   1     1    FW2
.........cucucaaagugAcugugaaaug....................................................................   1     1    FW2
..........ucucaaaguggcugugaaaug....................................................................   1     0    FW2
..........ucucaaaguggcugugaaaugcg..................................................................   1     0    FW2
...........cucaaaguggcugugaaa......................................................................   1     0    FW2
...........cucaaaguggcugugaaau.....................................................................   2     0    FW2
...........cucaaaguggcugugaaaug....................................................................   1     0    FW2
...................................................................uaucacagccagcuuuga..............   4     0    FW2
...................................................................uaucacagccagcuuugaU.............   2     1    FW2
...................................................................uaucacagccagcuuugaa.............   2     0    FW2
...................................................................uaucacagccagcuuugaag............   167   0    FW2
...................................................................uaucacagccagcuuugaUg............   16    1    FW2
...................................................................uaucacagccagcuuugaaU............   2     1    FW2
...................................................................uaucacagccagcuuugaaA............   2     1    FW2
...................................................................uaucacagccagcuuuAaag............   1     1    FW2
...................................................................uaucacagccagcuuuAaaga...........   1     1    FW2
...................................................................uaCcacagccagcuuugaaga...........   1     1    FW2
...................................................................uaucacagccagcuuugaaUa...........   1     1    FW2
...................................................................uaucacagccagcuuugaUga...........   14    1    FW2
...................................................................Caucacagccagcuuugaaga...........   1     1    FW2
...................................................................uaucacagccagcuuugaaga...........   216   0    FW2
...................................................................uaucacagccagcuuugaUgag..........   23    1    FW2
...................................................................uaucacagccagcuCugaagag..........   1     1    FW2
...................................................................uaucacagccagcuuugaagag..........   148   0    FW2
...................................................................uaucacagccagcuuugaagagA.........   9     1    FW2
...................................................................uaucacagccagcuuugaagagc.........   88    0    FW2
...................................................................uaucacagccagcuuugaUgagc.........   67    1    FW2
...................................................................uaucacagccagcuuugaagagcA........   1     1    FW2
...................................................................uaucacaUccagcuuugaagagcg........   1     1    FW2
...................................................................uaucacagccagcuuugaUgagcg........   3     1    FW2
```

```
          aga-mir-2b-star
                                                           aga-mir-2b
acuuugacccucucaaaguggcugugaaaugcguccuucacuguguaagggaagauuccuuuuucgcauaucacagccagcuuugaagagcgucacggu

....................................................................uaucacagccagcuuugaagagcg.......    334    0    FW2
....................................................................uaucacagccagcuuugaagagcU.......    1      1    FW2
....................................................................uaucacagccagcuuugaagagcgA......    1      1    FW2
....................................................................uaucacagccagcuuugaagagcgC......    1      1    FW2
....................................................................uaucacagccagcuuugaagagcCu......    1      1    FW2
....................................................................uaucacagccagcuuugaagagcgu......    15     0    FW2
.....................................................................aucacagccagcuuugaaga..........    1      0    FW2
......................................................................ucacagccagcuuugaUg...........    1      1    FW2
......................................................................ucacagccagcuuugaUga..........    5      1    FW2
......................................................................ucacagccagcuuugaUgag.........    13     1    FW2
......................................................................ucacagccagcuuugaUgagc........    69     1    FW2
......................................................................ucacagccagcuuugaagagcg.......    1      0    FW2
......................................................................ucacagccagcuuugaUgagcg.......    1      1    FW2
.......................................................................cacagccagcuuugaUga..........    1      1    FW2
.......................................................................cacagccagcuuugaagag.........    1      0    FW2

.........cucucaaaguggcugugaa.......................................................................    1      0    FF1
.........cucGcaaaguggcugugaaa......................................................................    1      1    FF1
.........cucucaaaguggcugugaaa......................................................................    12     0    FF1
.........cucucaaaguggcugugaaau.....................................................................    14     0    FF1
.........cucuGaaaguggcugugaaau.....................................................................    1      1    FF1
.........Uucucaaaguggcugugaaaug....................................................................    1      1    FF1
.........cucGcaaaguggcugugaaaug....................................................................    4      1    FF1
.........Gucucaaaguggcugugaaaug....................................................................    1      1    FF1
.........cucuGaaaguggcugugaaaug....................................................................    1      1    FF1
.........cucucaaaguggcugugaUaug....................................................................    1      1    FF1
.........cucucaaaguggcugugaaaug....................................................................    191    0    FF1
.........cAcucaaaguggcugugaaaug....................................................................    1      1    FF1
.........cucucaaagCggcugugaaaug....................................................................    1      1    FF1
.........cucucaaaguggcugugaaaugU...................................................................    3      1    FF1
.........cucucaaaguggcugugaaaugcg..................................................................    2      0    FF1
.........cucucaaaguggcugugaaaugcguccu..............................................................    2      0    FF1
.........cucucaaaguggcugugaaaugcguccuu.............................................................    1      0    FF1
..........ucucGaaguggcugugaaaug....................................................................    1      1    FF1
..........ucucaaaguggcugugaaaug....................................................................    4      0    FF1
...........cucaaaguggcugugaaaug....................................................................    6      0    FF1
...........cucGaaguggcugugaaaug....................................................................    1      1    FF1
...........cucaaaguggcugugaaaugcg..................................................................    1      0    FF1
............ucaaaguggcugugaaaug....................................................................    1      0    FF1
...................................................................auaucacagccagcuuugaag...........    1      0    FF1
...................................................................auaucacagccagcuuugaagag.........    1      0    FF1
...................................................................Cuaucacagccagcuuugaagag.........    1      1    FF1
....................................................................uaucacagccagcuuug..............    3      0    FF1
....................................................................uaucGcagccagcuuuga.............    1      1    FF1
....................................................................uauGacagccagcuuuga.............    2      1    FF1
....................................................................uaucacagccagcuuuga.............    76     0    FF1
....................................................................uaucacagccaCcuuuga.............    1      1    FF1
....................................................................uaucacagccagcCuugaa............    1      1    FF1
....................................................................uaucacagccagcuuugGa............    1      1    FF1
....................................................................uaucacagccagcuuugaC............    1      1    FF1
....................................................................uaucacagccagcuuugaa............    95     0    FF1
....................................................................uauGacagccagcuuugaa............    2      1    FF1
....................................................................uaucacagccagcuuugaU............    18     1    FF1
....................................................................uaucacagAcagcuuugaa............    1      1    FF1
....................................................................Gaucacagccagcuuugaa............    1      1    FF1
....................................................................uaucacagGcagcuuugaag...........    9      1    FF1
....................................................................uaucacagccagcuuuCaag...........    1      1    FF1
....................................................................uaucGcagccagcuuugaag...........    12     1    FF1
....................................................................uaucacagccagcuuugCag...........    3      1    FF1
....................................................................uaucacagccagcuuugaaC...........    1      1    FF1
....................................................................uaucacagcGagcuuugaag...........    1      1    FF1
....................................................................uaCcacagccagcuuugaag...........    1      1    FF1
....................................................................uaucacagccagcuuugaaA...........    19     1    FF1
....................................................................uaucacagAcagcuuugaag...........    12     1    FF1
....................................................................Gaucacagccagcuuugaag...........    12     1    FF1
....................................................................Caucacagccagcuuugaag...........    1      1    FF1
....................................................................uaucacagccagcuuugaGg...........    4      1    FF1
....................................................................uaucacagccagcuuugaaU...........    7      1    FF1
....................................................................uaucacagccagcuuugaUg...........    188    1    FF1
```

aga-mir-2b-star

aga-mir-2b

acuuugacccucucaaaguggcugugaaaugcguccuucacuguguaagggaagauuccuuuuucgcauaucacagccagcuuugaagagcgucacggu

....uaAcacagccagcuuugaag...........	2	1	FF1
....uauUacagccagcuuugaag...........	1	1	FF1
....uaucacagUcagcuuugaag...........	2	1	FF1
....uGucacagccagcuuugaag...........	2	1	FF1
....uaucacagccaUcuuugaag...........	1	1	FF1
....uaucacagccagcuuugGag...........	2	1	FF1
....uaucacaAccagcuuugaag...........	1	1	FF1
....uauGacagccagcuuugaag...........	38	1	FF1
....uaucacagccagcuuugaag...........	2296	0	FF1
....uaucacagccagcuuugaagC..........	15	1	FF1
....uaucacagGcagcuuugaaga..........	2	1	FF1
....uauGacagccagcuuugaaga..........	14	1	FF1
....Caucacagccagcuuugaaga..........	1	1	FF1
....uaucacagccagcuuugaaAa..........	2	1	FF1
....uaucacagccagcuCugaaga..........	1	1	FF1
....uaucacagcGagcuuugaaga..........	2	1	FF1
....uaucacagccagcuuUaaga..........	1	1	FF1
....uaucacagccagcuuugaUga..........	315	1	FF1
....uGucacagccagcuuugaaga..........	1	1	FF1
....Gaucacagccagcuuugaaga..........	5	1	FF1
....uaucacagccagcAuugaaga..........	2	1	FF1
....uaucGcagccagcuuugaaga..........	2	1	FF1
....uaucacagUcagcuuugaaga..........	1	1	FF1
....uaucacCgccagcuuugaaga..........	1	1	FF1
....uaucacagAcagcuuugaaga..........	4	1	FF1
....uaucacagccagcuuugaagU..........	1	1	FF1
....uaucacagccagcuuugGaga..........	2	1	FF1
....uaucacagccagcuuugaaga..........	976	0	FF1
....uauUacagccagcuuugaaga..........	1	1	FF1
....uaucacagccagcuuugaCga..........	1	1	FF1
....uaucacagccagcuuugaagG..........	1	1	FF1
....uaucacagccagcuGugaaga..........	1	1	FF1
....uaucacagccagUuuugaaga..........	1	1	FF1
....uaucacagccagcuuugaGga..........	2	1	FF1
....uaucacagccagcuuugaUgag.........	167	1	FF1
....uaucacagccagcuuugaagCg.........	2	1	FF1
....uaucacagccagcuuugaagaA.........	31	1	FF1
....uaucGcagccagcuuugaagag.........	1	1	FF1
....uaucacagccagcuuugaCgag.........	1	1	FF1
....uaCcacagccagcuuugaagag.........	1	1	FF1
....uaucacagccagcuuugaagaC.........	2	1	FF1
....uaucacagAcagcuuugaagag.........	1	1	FF1
....uaucacagccagcuuugaagaU.........	4	1	FF1
....uauGacagccagcuuugaagag.........	1	1	FF1
....uaucacagccagcuuugaagag.........	173	0	FF1
....uaucacagccagcuuugaagCgc........	1	1	FF1
....uaucacagccagcuuugaagagA........	7	1	FF1
....uaucacagccagcuuugaGgagc........	1	1	FF1
....uaucacagGcagcuuugaagagc........	2	1	FF1
....uaucacagccagcuuugaagagc........	66	0	FF1
....uaucacagccagcuuugaUgagc........	74	1	FF1
....uauGacagccagcuuugaagagcg.......	4	1	FF1
....uaucacagccagcuuugaagagcU.......	5	1	FF1
....uaucacagccagcuuugaagagcg.......	232	0	FF1
....uaucacagccagcuuugaagagcA.......	1	1	FF1
....uCucacagccagcuuugaagagcg.......	1	1	FF1
....uaucacagccagcuuugaGgagcg.......	2	1	FF1
....uaucacagGcagcuuugaagagcg.......	1	1	FF1
....uaucacagccagcuuugaUgagcg.......	2	1	FF1
....uaucacagccagcuuugaCgagcg.......	1	1	FF1
....uaucacagcAagcuuugaagagcg.......	1	1	FF1
....uaucacagccagcuuugaagCgcg.......	1	1	FF1
....uCucacagccagcuuugaagagcgu......	1	1	FF1
....uaucacagccagcuuugaagagcgu......	5	0	FF1
....uaucacagccagcuuugaagagcgA......	1	1	FF1
....uaucacagccagcuuugaagagcgC......	3	1	FF1
....aucacagccagcuuugaag...........	1	0	FF1
....ucacagccagcuuugaU...........	1	1	FF1
....ucacagccagcuuugaUg...........	3	1	FF1

```
            aga-mir-2b-star
                                                                       aga-mir-2b
acuuugacccucucaaaguggcugugaaaugcguccuucacuguguaagggaagauuccuuuuucgcauaucacagccagcuuugaagagcgucacggu

......................................................................ucacagccagcuuugaUga..........   7      1     FF1
......................................................................ucacagccagcuuugaagag.........   1      0     FF1
......................................................................ucacagccagcuuugaUgag.........   100    1     FF1
......................................................................ucacagccagcuuugaUgagc........   265    1     FF1
......................................................................ucacagccagcuuugaUgagcg.......   4      1     FF1
........................................................................acagccagcuuugaagC..........   1      1     FF1
........................................................................acagccagcuuugaUga..........   1      1     FF1

.........cucucaaaguggcugugaa.......................................................................   1      0     OV1
.........cucucaaaguggcugugaaa......................................................................   16     0     OV1
.........cucucaaaguggcugugaaau.....................................................................   11     0     OV1
.........cucucaaaguggcugugaaaug....................................................................   67     0     OV1
.........cucucaaaguggcugugUaaug....................................................................   1      1     OV1
.........cucucaaaguggcugugaaauU....................................................................   2      1     OV1
.........cucucaaaguggcugugaaaugA...................................................................   1      1     OV1
.........cucucaaaguggcugugaaaugU...................................................................   1      1     OV1
.........cucucaaaguggcugugaaaugcguccu..............................................................   1      0     OV1
..........ucucaaaguggcugugaaa......................................................................   3      0     OV1
..........ucucaaaguggcugugaaau.....................................................................   1      0     OV1
..........ucucaaaguggcugugaaaug....................................................................   24     0     OV1
..........ucucaaaguggcugugaaaugA...................................................................   1      1     OV1
...........cucaaaguggcugugaaa......................................................................   1      0     OV1
...........cucaaaguggcugugaaau.....................................................................   1      0     OV1
...........cCcaaaguggcugugaaaug....................................................................   1      1     OV1
...........cucaaaguggcugugaaaug....................................................................   12     0     OV1
...........cucaaaguggcugugaaaugcg..................................................................   1      0     OV1
.............caaaguggcugugaaaug....................................................................   7      0     OV1
..............aaaguggcugugaaaug....................................................................   2      0     OV1
...............................cguccuucacuguguaagggaagauuccuuuuucgca...............................   1      0     OV1
...................................................................Cuaucacagccagcuuugaaga..........   1      1     OV1
....................................................................uaucacagccagcuuug..............   4      0     OV1
....................................................................uaucacagccagcuuuga.............   25     0     OV1
....................................................................uaucacagccagcuuugaU............   4      1     OV1
....................................................................uaucacagccagcuuugaG............   1      1     OV1
....................................................................uaucacagccagcuuugaa............   20     0     OV1
....................................................................uaucacagccagcuuugaag...........   1277   0     OV1
....................................................................uaucacagccagcuuugaaC...........   1      1     OV1
....................................................................uaucacagccagcuuuCaag...........   1      1     OV1
....................................................................uaucacagccagcuuugaUg...........   75     1     OV1
....................................................................uaucacagccagcuuugaaU...........   8      1     OV1
....................................................................uaucacagccagcuuugaaA...........   20     1     OV1
....................................................................uaucGcagccagcuuugaag...........   3      1     OV1
....................................................................uaucacagcUagcuuugaag...........   1      1     OV1
....................................................................uaucacagccagcuuugaCg...........   1      1     OV1
....................................................................uaucacagccagcuuuAaag...........   2      1     OV1
....................................................................uaucacagccagCuugaag............   1      1     OV1
....................................................................uauUacagccagcuuugaag...........   1      1     OV1
....................................................................uaucacagccaCcuuugaag...........   1      1     OV1
....................................................................uaucacagccGgcuuugaag...........   1      1     OV1
....................................................................uaucacagccaUcuuugaaga..........   1      1     OV1
....................................................................uaucacagAcagcuuugaaga..........   1      1     OV1
....................................................................uaucUcagccagcuuugaaga..........   1      1     OV1
....................................................................uaucacagccagcuuugaagU..........   2      1     OV1
....................................................................uaucacagccagcuuugaaga..........   2082   0     OV1
....................................................................uaucacGgccagcuuugaaga..........   1      1     OV1
....................................................................uaucaGagccagcuuugaaga..........   1      1     OV1
....................................................................uaucacagcUagcuuugaaga..........   1      1     OV1
....................................................................Caucacagccagcuuugaaga..........   1      1     OV1
....................................................................uaucacagccagAuuugaaga..........   1      1     OV1
....................................................................uaucacagccGgcuuugaaga..........   3      1     OV1
....................................................................uauUacagccagcuuugaaga..........   2      1     OV1
....................................................................uaucacagccagcuuugaaUa..........   1      1     OV1
....................................................................uaucacagccaAcuuugaaga..........   1      1     OV1
....................................................................uaucacaAccagcuuugaaga..........   2      1     OV1
....................................................................Aaucacagccagcuuugaaga..........   1      1     OV1
....................................................................uaucacagccagcuuuAaaga..........   3      1     OV1
....................................................................uaucGcagccagcuuugaaga..........   1      1     OV1
....................................................................uaucacagccagcuuugaUga..........   80     1     OV1
....................................................................uGucacagccagcuuugaaga..........   1      1     OV1
```

```
          aga-mir-2b-star
                                                                    aga-mir-2b
acuuugacccucucaaaguggcugugaaaugcguccuucacuguguaagggaagauuccuuuuucgcauaucacagccagcuuugaagagcgucacggu

....................................................................uaucacagccagcuuugaaAa..........   8     1    OV1
....................................................................uaucacagccagcuuGgaaga..........   3     1    OV1
....................................................................uaucacagGcagcuuugaaga..........   1     1    OV1
....................................................................uaucacagccagcuuugGaga..........   1     1    OV1
....................................................................uaucaUagccagcuuugaaga..........   1     1    OV1
....................................................................uaucacagcGagcuuugaaga..........   1     1    OV1
....................................................................uaucacagccagcuuugaagaA.........   35    1    OV1
....................................................................uaucacagccagcuuugaagag.........   157   0    OV1
....................................................................uaucacagccagcuuugaagaU.........   13    1    OV1
....................................................................uaucacagccagcuuugaagaC.........   6     1    OV1
....................................................................uaucacagccagcuuugaUgag.........   50    1    OV1
....................................................................uaucacagccagcuuugaagaAc........   1     1    OV1
....................................................................uaucacagccagcuuugaagagc........   13    0    OV1
....................................................................uaucacagccagcuuugaagagA........   26    1    OV1
....................................................................uaucacagccagcuuugaUgagc........   40    1    OV1
....................................................................uaucacagccagcuuugaagagcg.......   138   0    OV1
....................................................................uaucacagAcagcuuugaagagcg.......   1     1    OV1
....................................................................uaucacagccagcuuugaagagcU.......   1     1    OV1
....................................................................uaucacagcAagcuuugaagagcg.......   1     1    OV1
....................................................................uaucacagccagcuuugaagagcgu......   20    0    OV1
....................................................................uaucacagccagcuuugaagagcgA......   1     1    OV1
....................................................................uaucacagccagcuuugaagagcgC......   1     1    OV1
....................................................................Aaucacagccagcuuugaagagcgu......   1     1    OV1
.....................................................................aucacagccagcuuugaagag.........   1     0    OV1
.....................................................................aucacagccagcuuugaagagcg.......   1     0    OV1
......................................................................ucacagccagcuuugaa............   1     0    OV1
......................................................................ucacagccagcuuugaUg...........   3     1    OV1
......................................................................ucacagccagcuuugaUga..........   7     1    OV1
......................................................................ucacagccagcuuugaUgag.........   104   1    OV1
......................................................................ucacagccagcuuugaUgagc........   247   1    OV1
........................................................................acagccagcuuugaaga..........   2     0    OV1
........................................................................acagccagcuuugaUgagc........   2     1    OV1

........Acucucaaaguggcugugaaaug....................................................................   1     1    MF1
.........cucucaaaguggcugugaaa......................................................................   9     0    MF1
.........cucucaaaguggcugugaaau.....................................................................   5     0    MF1
.........cucucaaagGggcugugaaaug....................................................................   1     1    MF1
.........cucucaaaguggcugugaaaug....................................................................   114   0    MF1
.........cucucaaagugUcugugaaaug....................................................................   1     1    MF1
.........cucucaaaguggcugugaaaugU...................................................................   2     1    MF1
.........cucucaaaguggcugugaaaugcguccu..............................................................   3     0    MF1
..........ucucaaaguggcugugaaa......................................................................   2     0    MF1
..........ucucaaaguggcugugaaau.....................................................................   4     0    MF1
..........ucucaaaguggcugugaaaug....................................................................   6     0    MF1
...........cucaaaguggcugugaaaug....................................................................   2     0    MF1
..............aaaguggcugugaaaug....................................................................   1     0    MF1
....................................................................uaucacagccagcuuug..............   2     0    MF1
....................................................................uaucacagccagcuuuga.............   27    0    MF1
....................................................................uaucacagccagcuuugaU............   4     1    MF1
....................................................................uaucacagccagcuuugaa............   20    0    MF1
....................................................................uaucacagccaAcuuugaag...........   1     1    MF1
....................................................................uaucacaAccagcuuugaag...........   1     1    MF1
....................................................................uaucacagccagcuuugaag...........   578   0    MF1
....................................................................uaucUcagccagcuuugaag...........   1     1    MF1
....................................................................uaucacagccCgcuuugaag...........   1     1    MF1
....................................................................uaucacagccagcuuugaUg...........   40    1    MF1
....................................................................uaucacagccagcuCugaag...........   1     1    MF1
....................................................................uaucacagccagcuuugaaU...........   4     1    MF1
....................................................................uaCcacagccagcuuugaag...........   1     1    MF1
....................................................................uaucacagccagcuuugaaA...........   11    1    MF1
....................................................................uaucacagccagcuuugaaC...........   3     1    MF1
....................................................................uaucacagccagcuuugaaUa..........   1     1    MF1
....................................................................uaucacagccagcuuuCaaga..........   1     1    MF1
....................................................................uaucacagccaAcuuugaaga..........   1     1    MF1
....................................................................uaucacagccagcuuugaagU..........   1     1    MF1
....................................................................uaucacagccagcuuuUaaga..........   1     1    MF1
....................................................................uaAcacagccagcuuugaaga..........   1     1    MF1
....................................................................uaucacaAccagcuuugaaga..........   1     1    MF1
....................................................................uaucacagccagcCuugaaga..........   1     1    MF1
```

aga-mir-2b-star

aga-mir-2b

acuuugacccucucaaaguggcugugaaaugcguccuucacuguguaagggaagauuccuuuuucgcauaucacagccagcuuugaagagcgucacggu

....uaucacagccUgcuuugaaga..........	1	1	MF1
....uaucacaCccagcuuugaaga..........	2	1	MF1
....uaucacagccagcuuugaUga..........	64	1	MF1
....uaucacagccagcuCugaaga..........	1	1	MF1
....uaucacagccagcuuugaaga..........	544	0	MF1
....uaucacagcUagcuuugaaga..........	1	1	MF1
....uaucacagccagcuuugaagaC.........	1	1	MF1
....uaucacagccagcuuugaagag.........	91	0	MF1
....uaucacagccagcuuugaUgag.........	35	1	MF1
....uaucacagccagcuuugaagaA.........	10	1	MF1
....uaucacagccagcuuugaagaU.........	1	1	MF1
....uaucacagccagcuuugaUgagc........	24	1	MF1
....uaucacagccagcuuugaagagA........	5	1	MF1
....uaucacagccagcuuugaagagc........	22	0	MF1
....uaucacagccagcuuugaagaAc........	1	1	MF1
....uaucacagccagcuuuAaagagcg.......	1	1	MF1
....uaucacagccagcuuugaagagcU.......	3	1	MF1
....uaucacagccagcuuugaagagcg.......	165	0	MF1
....uaucacagccagcuuugaagagcgu......	2	0	MF1
....ucacagccagcuuugaUg...........	2	1	MF1
....ucacagccagcuuugaUga..........	2	1	MF1
....ucacagccagcuuugaUgag.........	40	1	MF1
....ucacagccagcuuugaUgagc........	68	1	MF1
....ucacagccagcuuugaUgagcg.......	3	1	MF1
.........cucucaaaguggcugugaaa..	2	0	BF2
.........cucucaaaguggcugugaaau...	2	0	BF2
.........cucucaaaguggcugugaaaug..	30	0	BF2
..........ucucaaaguggcugugaaa..	1	0	BF2
..........ucucaaaguggcugugaaaug..	10	0	BF2
...........cucaaaguggcugugaaaug..	5	0	BF2
............ucaaaguggcugugaaaug..	2	0	BF2
.............caaaguggcugugaaaug..	5	0	BF2
...............aaguggcugugaaaugc...	1	0	BF2
....uucgcauaucacagccagcuuugaag...........	1	0	BF2
....uaucacagccagcuuuga.............	4	0	BF2
....uaucacagccagcuuugaa............	6	0	BF2
....uaucacagccagcCuugaag...........	1	1	BF2
....uaucacagccagcuuugaUg...........	43	1	BF2
....uaucacagccagcuuugaaC...........	1	1	BF2
....uaucacagccagcuuugaaU...........	2	1	BF2
....uaucacagccaUcuuugaag...........	1	1	BF2
....uaucaUagccagcuuugaag...........	1	1	BF2
....Caucacagccagcuuugaag...........	1	1	BF2
....uaucacagUcagcuuugaag...........	1	1	BF2
....uaucacagccagcuuugaag...........	737	0	BF2
....uaucacagccagcuuugaaA...........	6	1	BF2
....uaucacagccaCcuuugaag...........	1	1	BF2
....Gaucacagccagcuuugaag...........	1	1	BF2
....uaucacagccagcuuugaGg...........	1	1	BF2
....uaucacagccagcuuuAaaga..........	1	1	BF2
....uaucacagccagcuuugaUga..........	53	1	BF2
....uaucacagccagcuuugaaga..........	1214	0	BF2
....uaucacagAcagcuuugaaga..........	1	1	BF2
....uaucacagccagcuuugaaAa..........	1	1	BF2
....uaucacagccagcuuugaagG..........	1	1	BF2
....uGucacagccagcuuugaaga..........	1	1	BF2
....uaucacagccagcuCugaaga..........	1	1	BF2
....uaucacagccaUcuuugaaga..........	1	1	BF2
....uaucacagcUagcuuugaaga..........	2	1	BF2
....uaucacagccagcuuugaagaU.........	7	1	BF2
....uaucacagccagcuuugaagag.........	220	0	BF2
....uaucacagccagcuuugaUgag.........	64	1	BF2
....uaucacagccagcuuugaagaC.........	3	1	BF2
....uaucacagccagcuuugaagaA.........	12	1	BF2
....uaucaUagccagcuuugaagagc........	1	1	BF2
....uaucacagccagcuuugaUgagc........	41	1	BF2
....uaucacagccagcuuugaagagc........	44	0	BF2
....uaucacagccagcuuugaagaAc........	1	1	BF2

```
            aga-mir-2b-star
                                                           aga-mir-2b
acuuugacccucucaaaguggcugugaaaugcguccuucacuguguaagggaagauuccuuuucgcauaucacagccagcuuugaagagcgucacggu

...................................................................uaucacagccagcuuugaagagA........   24    1    BF2
...................................................................uaucacagccagcuuugaagagcg.......   358   0    BF2
...................................................................uaucacGgccagcuuugaagagcg.......   1     1    BF2
...................................................................uauGacagccagcuuugaagagcg.......   1     1    BF2
...................................................................uaucacagccagcuuugaagagcC.......   1     1    BF2
...................................................................uaucacagccagcuuuAaagagcg.......   1     1    BF2
...................................................................uaucacagccagcuuugaagagcU.......   1     1    BF2
...................................................................uaucacagccaAcuuugaagagcg.......   1     1    BF2
...................................................................Caucacagccagcuuugaagagcg.......   1     1    BF2
...................................................................uaucacagccagcuuugaUgagcg.......   1     1    BF2
...................................................................uaGcacagccagcuuugaagagcg.......   1     1    BF2
...................................................................uaucacagccagcuuugaagagcgA......   2     1    BF2
...................................................................uaucacagccagcuuugaagagcgG......   1     1    BF2
...................................................................uaucacagccagcuuugaagagcgu......   27    0    BF2
....................................................................aucacagccagcuuugaUgagc........   1     1    BF2
....................................................................aucacagccagcuuugaagagcg.......   1     0    BF2
.....................................................................ucacagccagcuuugaUg...........   2     1    BF2
.....................................................................ucacagccagcuuugaUga..........   4     1    BF2
.....................................................................ucacagccagcuuugaUgag.........   48    1    BF2
.....................................................................ucacagccagcuuugaUgagc........   63    1    BF2
.....................................................................ucacagccagcuuugaUgagcg.......   1     1    BF2

.........cucucaaaguggcugugaa......................................................................   1     0    BF1
.........cucucaaaguggcugugaaa.....................................................................   4     0    BF1
.........cucucaaaguggcugugaaau....................................................................   2     0    BF1
.........cucucaaaguCgcugugaaau....................................................................   1     1    BF1
.........cucucaaaguggcugugaaauA...................................................................   1     1    BF1
.........cucucaaaguggcugugaaaug...................................................................   63    0    BF1
..........ucucaaaguggcugugaaa.....................................................................   1     0    BF1
..........ucucaaaguggcugugaaau....................................................................   1     0    BF1
..........ucucaaaguggcugugaaaug...................................................................   4     0    BF1
...........cucaaaguggcugugaaaug...................................................................   4     0    BF1
.............caaaguggcugugaaaug...................................................................   1     0    BF1
..................................................................Cuaucacagccagcuuugaagagcg.......   1     1    BF1
...................................................................uaucacagccagcuuuga.............   2     0    BF1
...................................................................uaucacagccagcuuugaU............   2     1    BF1
...................................................................uaucacagccagcuuugaa............   1     0    BF1
...................................................................uaucacagccagcuuugaaA...........   4     1    BF1
...................................................................uaucacagccagcuuugaag...........   209   0    BF1
...................................................................uaucacagccagcuuugaUg...........   9     1    BF1
...................................................................uaucacagccagcuuugaaC...........   1     1    BF1
...................................................................uaucacagccagcuuugaaU...........   1     1    BF1
...................................................................uaucacagccagcuCugaaga..........   1     1    BF1
...................................................................uaucacagccUgcuuugaaga..........   1     1    BF1
...................................................................uaucacagccagcuuugaagC..........   1     1    BF1
...................................................................uaAcacagccagcuuugaaga..........   1     1    BF1
...................................................................uaucacagccagcuuuAaaga..........   1     1    BF1
...................................................................uaucacagccagcuuugaaAa..........   2     1    BF1
...................................................................uGucacagccagcuuugaaga..........   1     1    BF1
...................................................................uaucacagccagcuuugaaga..........   437   0    BF1
...................................................................uaucacagccagcuuugaUga..........   12    1    BF1
...................................................................uaucacagccagcuuugaagG..........   1     1    BF1
...................................................................uaucacagccagcuuugaagaU.........   1     1    BF1
...................................................................uaucacagccagcuuugaUgag.........   26    1    BF1
...................................................................uaucacagccagcuuugaagaA.........   6     1    BF1
...................................................................uaucacagccagcuuugaagag.........   87    0    BF1
...................................................................uaucacagccagcuuugaagagc........   25    0    BF1
...................................................................uaucacagccagcuuugaagagA........   19    1    BF1
...................................................................uaucacagccagcuuugaUgagc........   26    1    BF1
...................................................................uGucacagccagcuuugaagagcg.......   1     1    BF1
...................................................................uauUacagccagcuuugaagagcg.......   1     1    BF1
...................................................................uaucacagccagcGuugaagagcg.......   1     1    BF1
...................................................................uaucacagccagUuuugaagagcg.......   1     1    BF1
...................................................................uaucacagccagcuuugaUgagcg.......   1     1    BF1
...................................................................uaucacagccagcuuugaagagcU.......   4     1    BF1
...................................................................uaucacagccagcuuugaagagcg.......   292   0    BF1
...................................................................uaucacagccagcuuugaagagcgC......   1     1    BF1
...................................................................uaucacagccagcuuugaagagcgu......   12    0    BF1
.....................................................................ucacagccagcuuugaUga..........   4     1    BF1
```

```
          aga-mir-2b-star
                                                            aga-mir-2b
acuuugacccucucaaaguggcugugaaaugcguccuucacuguguaagggaagauuccuuuuucgcauaucacagccagcuuugaagagcgucacggu

......................................................................ucacagccagcuuugaUgag.........    23     1    BF1
......................................................................ucacagccagcuuugaUgagc........    59     1    BF1
......................................................................ucacagccagcuuugaagagcg.......    1      0    BF1

.........cucucaaaguggcugugaa.......................................................................    1      0    FW1
.........cucucaaaguggcugugaaa......................................................................    4      0    FW1
.........cucucaaaguggcugugaaau.....................................................................    2      0    FW1
.........cucucaaaguggcugugaaaA.....................................................................    1      1    FW1
.........cucucaaaguggcugugaaaug....................................................................    81     0    FW1
...........cucaaaguggcugugaaau.....................................................................    1      0    FW1
...................................................................auaucacagccagcuuugaaga..........    1      0    FW1
....................................................................uaucacagccagcuuugaa............    1      0    FW1
....................................................................uaucacagccagcuuugaag...........    79     0    FW1
....................................................................uaucaUagccagcuuugaag...........    1      1    FW1
....................................................................uaucacagccagcuuugaUg...........    9      1    FW1
....................................................................uaucacagccagcuuugaUga..........    11     1    FW1
....................................................................uaucacagccagcuuugaaga..........    119    0    FW1
....................................................................uaucGcagccagcuuugaaga..........    1      1    FW1
....................................................................uaucacagccagcuuugaagag.........    51     0    FW1
....................................................................uaucacagccagcuuugaagaU.........    1      1    FW1
....................................................................uaucacagccagcuuugaUgag.........    5      1    FW1
....................................................................uaucacagccagcuuugaagaA.........    2      1    FW1
....................................................................uaucacagccagcuuugaagagA........    1      1    FW1
....................................................................uaucacagccagcuuugaagagc........    25     0    FW1
....................................................................uaucacagccagcuuugaUgagc........    29     1    FW1
....................................................................uaucacagccagcuuugaagaUc........    1      1    FW1
....................................................................uaucacagccagcuuugaagagcA.......    1      1    FW1
....................................................................uaAcacagccagcuuugaagagcg.......    1      1    FW1
....................................................................uaucacagccagcuuugaagagcg.......    189    0    FW1
....................................................................uaucacagccagcuuugaUgagcg.......    4      1    FW1
....................................................................uaucacagccaUcuuugaagagcg.......    1      1    FW1
....................................................................uaucacagccagcuCugaagagcg.......    1      1    FW1
....................................................................uaucacagccagcuuugaagagcU.......    2      1    FW1
....................................................................uaucacagccagcuuugaagagcgu......    4      0    FW1
......................................................................ucacagccagcuuugaUg...........    4      1    FW1
......................................................................ucacagccagcuuugaUgag.........    7      1    FW1
......................................................................ucacagccagcuuugaUgagc........    30     1    FW1
.........................................................................cagccagcuuugaagagcg.......    1      0    FW1

.........cucucaaaguggcugugaa.......................................................................    3      0    MW1
.........cucucaaaguggcugugaaa......................................................................    3      0    MW1
.........cucucaaaguggcugugaaau.....................................................................    7      0    MW1
.........cucucaaaguggcugugaaaug....................................................................    128    0    MW1
.........cucuUaaaguggcugugaaaug....................................................................    1      1    MW1
.........cucucaaaguggcugugaaaugcg..................................................................    1      0    MW1
..........ucucaaaguggcugugaaaug....................................................................    7      0    MW1
...........cucaaaguggcugugaaaug....................................................................    1      0    MW1
...........cucaaaguggcugugaaaugcguccuu.............................................................    1      0    MW1
................aguggcugugaaaugcguccuucaUuguguaagggaagauuc.........................................    1      1    MW1
....................................................................uaucacagccagcuuug..............    2      0    MW1
....................................................................uaucacagccagcuuuga.............    1      0    MW1
....................................................................uaucacagccagcuuugaa............    4      0    MW1
....................................................................uaCcacagccagcuuugaag...........    1      1    MW1
....................................................................uaucacagccagcuuugaUg...........    22     1    MW1
....................................................................uaucacagccUgcuuugaag...........    1      1    MW1
....................................................................uaucacagccagcuuugaag...........    233    0    MW1
....................................................................uaucacagccagcuuugaUga..........    25     1    MW1
....................................................................uaucacagccagcCuugaaga..........    1      1    MW1
....................................................................uaucacaAccagcuuugaaga..........    1      1    MW1
....................................................................uaucacagccagcuuugaaga..........    248    0    MW1
....................................................................uaucacagAcagcuuugaaga..........    1      1    MW1
....................................................................Caucacagccagcuuugaagag.........    1      1    MW1
....................................................................uaucacagccagcuuugaUgag.........    31     1    MW1
....................................................................uaucacagccagcuuugaagaA.........    3      1    MW1
....................................................................uaucacagccagcuuugaagag.........    114    0    MW1
....................................................................uaucacagccagcuuugaagagA........    7      1    MW1
....................................................................uaucacagccagcuuugaagagc........    65     0    MW1
....................................................................uaucacagccagcuuugaUgagc........    39     1    MW1
....................................................................uaucacagccagcuuugaagagcg.......    350    0    MW1
```

```
          aga-mir-2b-star
                                                                    aga-mir-2b
acuuugacccucucaaaguggcugugaaaugcguccuucacuguguaagggaagauuccuuuuucgcauaucacagccagcuuugaagagcgucacggu

....................................................................uaucacagcUagcuuugaagagcg.......   1    1    MW1
....................................................................uaucacagccagcuuugaagagcA.......   4    1    MW1
....................................................................uaucacagccagcuuugaUgagcg.......   1    1    MW1
....................................................................uaucacagccagcuuugaagagcU.......   2    1    MW1
....................................................................uaucacagccagcuCugaagagcg.......   1    1    MW1
....................................................................uaucacagccagcuuugaagagUg.......   1    1    MW1
....................................................................uaucacagccagcuuugaagagcgC......   2    1    MW1
....................................................................uaucacagccagcuuugaagagcgu......   3    0    MW1
....................................................................uaucacagccagcuuugaagagcguG.....   1    1    MW1
......................................................................ucacagccagcuuugaUg...........   3    1    MW1
......................................................................ucacagccagcuuugaUga..........   2    1    MW1
......................................................................ucacagccagcuuugaUgag.........   11   1    MW1
......................................................................ucacagccagcuuugaUgagc........   39   1    MW1
......................................................................ucacagccagcuuugaUgagcg.......   1    1    MW1
.......................................................................cacagccagcuuugaUg...........   2    1    MW1
.......................................................................cacagccagcuuugaUga..........   2    1    MW1
.......................................................................cacagccagcuuugaagagcg.......   1    0    MW1
........................................................................acagccagcuuugaagag.........   1    0    MW1
........................................................................acagccagcuuugaagagcg.......   1    0    MW1
.........................................................................cagccagcuuugaagagcg.......   1    0    MW1

.........cucucaaaguggcugugaaau.....................................................................   4    0    MW2
.........cucucaaaguggcugugaaaug....................................................................   38   0    MW2
....................................................................uaucacagccagcuuuga.............   1    0    MW2
....................................................................uaucacagccagcuuugaa............   2    0    MW2
....................................................................uaucacagccagcuuCgaag...........   1    1    MW2
....................................................................uaucacagccagcuuugaUg...........   3    1    MW2
....................................................................uaucacagccagcuuugaag...........   47   0    MW2
....................................................................uaucacagccagcuuugaaga..........   65   0    MW2
....................................................................uaucacagccagcuuugaUga..........   3    1    MW2
....................................................................uaucacagccagcuuugaUgag.........   7    1    MW2
....................................................................uaucacagccagcuuugaagaC.........   2    1    MW2
....................................................................uaucacagccagcuuugaagag.........   36   0    MW2
....................................................................uaucacagccagcuuugaagagc........   22   0    MW2
....................................................................uaucacagccagcuuugaagagA........   7    1    MW2
....................................................................uaucacagccagcuuuAaagagc........   1    1    MW2
....................................................................uaucacagccagcuuugaUgagc........   20   1    MW2
....................................................................uaucacagccagcuuugaagagcA.......   1    1    MW2
....................................................................uauUacagccagcuuugaagagcg.......   1    1    MW2
....................................................................uaucaUagccagcuuugaagagcg.......   1    1    MW2
....................................................................uaucacagccagcuuugaagagcg.......   82   0    MW2
....................................................................uaucacagccagcuuugaagagcgu......   1    0    MW2
......................................................................ucacagccagcuuugaUgag.........   4    1    MW2
......................................................................ucacagccagcuuugaUgagc........   31   1    MW2
......................................................................ucacagccagcuuugaagagcg.......   1    0    MW2
.......................................................................cacagccagcuuugaUga..........   2    1    MW2

.........cucucaaaguggcugugaa.......................................................................   2    0    TE2
.........cucucaaaguggcugugaaa......................................................................   3    0    TE2
.........cucucaaaguggcugugaaau.....................................................................   3    0    TE2
.........cucucaaaguggcugugaaaug....................................................................   13   0    TE2
.........cucucaaaguggcugugaaauA....................................................................   2    1    TE2
.........cucucaaaguggcugugaaaugA...................................................................   1    1    TE2
..........ucucaaaguggcugugaaau.....................................................................   1    0    TE2
..........ucucaaaguggcugugaaaug....................................................................   2    0    TE2
..........ucucaaaguggcugugaaaugA...................................................................   1    1    TE2
...........cucaaaguggcugugaaa......................................................................   1    0    TE2
.............caaaguggcugugaaaug....................................................................   1    0    TE2
....................................................................uaucacagccagcuuug..............   2    0    TE2
....................................................................uaucacagccagcuuuga.............   13   0    TE2
....................................................................uaucacagccagcuuugaa............   12   0    TE2
....................................................................uaucacagccagcuuugaU............   1    1    TE2
....................................................................uaucacagccUgcuuugaag...........   1    1    TE2
....................................................................uaucacagccagcuuugaaA...........   3    1    TE2
....................................................................uaucGcagccagcuuugaag...........   1    1    TE2
....................................................................uaucacagccagcuuugaag...........   212  0    TE2
....................................................................uaucacagccagcuuugaUg...........   18   1    TE2
....................................................................uaucaUagccagcuuugaaga..........   1    1    TE2
....................................................................uaucacGgccagcuuugaaga..........   1    1    TE2
```

aga-mir-2b-star

aga-mir-2b

acuuugacccucucaaaguggcugugaaaugcguccuucacuguguaagggaagauuccuuuuucgcauaucacagccagcuuugaagagcgucacggu

....uGucacagccagcuuugaaga..........	1	1	TE2
....uaucacagccagcuuugaUga..........	23	1	TE2
....uaucacagccagcuuugaaga..........	477	0	TE2
....uaucacagccagcuuugaagU..........	1	1	TE2
....uaucacagccagcuuugaagG..........	1	1	TE2
....uaucacCgccagcuuugaaga..........	1	1	TE2
....uaucacagccagcuuugaagaA.........	3	1	TE2
....uaucacagccagcuuugaaAag.........	1	1	TE2
....uaucacagccagcuuugGagag.........	1	1	TE2
....uaucacagccagcuuugaUgag.........	41	1	TE2
....uaucacagAcagcuuugaagag.........	1	1	TE2
....uaucacagccagcuuugaagag.........	323	0	TE2
....uaucacagccagcuuugaagaU.........	2	1	TE2
....uaucacagccagcuuugaagagU........	1	1	TE2
....uaucacagccagcuuugaUgagc........	124	1	TE2
....uaucUcagccagcuuugaagagc........	1	1	TE2
....uaucacagccGgcuuugaagagc........	1	1	TE2
....uaucacagccagcuuugaagagA........	62	1	TE2
....uaucacagccagcuuugaagagc........	105	0	TE2
....uaucacagcAagcuuugaagagc........	2	1	TE2
....uaucacagccagcuuugaagagcU.......	8	1	TE2
....uaucacagccaUcuuugaagagcg.......	1	1	TE2
....uaucacGgccagcuuugaagagcg.......	1	1	TE2
....uaucacagccagcuuugaUgagcg.......	4	1	TE2
....uaucacagUcagcuuugaagagcg.......	1	1	TE2
....uaucacagccagcuuugaagagcA.......	8	1	TE2
....uaucacagccGgcuuugaagagcg.......	1	1	TE2
....uauUacagccagcuuugaagagcg.......	1	1	TE2
....uaucacagccagcuuugaagagcg.......	341	0	TE2
....uaucacagccagcuuugaaAagcg.......	1	1	TE2
....uaucacagccagcuuugaagagcgu......	11	0	TE2
....uaucacagccagcuuugaagagcgA......	2	1	TE2
....uaucacagccagcuuugaagagcguUa....	1	1	TE2
....ucacagccagcuuugaUg...........	5	1	TE2
....ucacagccagcuuugaUga..........	9	1	TE2
....ucacagccagcuuugaUgag.........	48	1	TE2
....ucacagccagcuuugaUgagc........	141	1	TE2
....ucacagccagUuuugaagagcg.......	1	1	TE2
....ucacagccagcuuugaUgagcg.......	1	1	TE2
....acagccagcuuugaagag.........	1	0	TE2

miRBase precursor : aga-mir-2c
Total read count : 25
aga-mir-2c read count : 14
aga-mir-2c-star read count : 11
remaining reads : 0

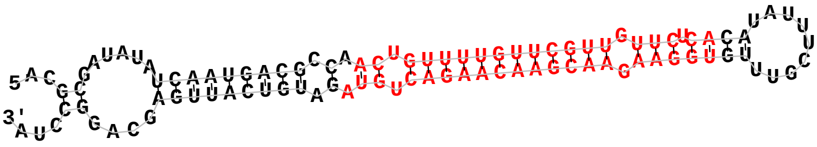

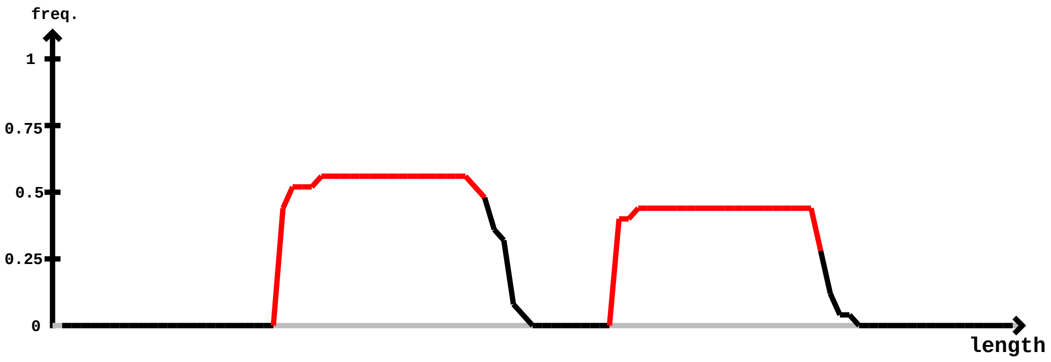


```
                      aga-mir-2c
                                                         aga-mir-2c-star
5'- acgcgauauaucaaugacgccaacuguuuuguucguuguucucacauauuucguuuguggaagaacgaacaagacuguagaugucauugagcaggccua   -3'    exp
    ..((......((((((((.(.((.((((((((((((.(((.((((.........)))))))).)))))))))))).)).).)))))))))....))...     reads    mm    sample
    ......................acuguuuuguucguuguucucacau....................................................     1        0     TE1
    ......................acuguuuuguucguuguucucacaAa...................................................     1        1     TE1
    .......................cuguuuuguucguuguucucaca.....................................................     1        0     TE1
    .........................................................uggaagaacgaacaagacugu.....................     1        0     TE1
    .........................................................uggaagaacgaacaagacugua....................     1        0     TE1

    ......................acuguuuuguucguuguucu.........................................................     1        0     OV2

    ......................acuguuuuguucguuguucucac......................................................     1        0     BF1

    ......................acuguuuuguucguuguucuca.......................................................     1        0     TE2
    ......................acuguuuuguucguuguucucaca.....................................................     2        0     TE2
    .......................cuguuuuguucguuguucucaca.....................................................     1        0     TE2
    .........................................................uggaagaacgaacaagacugu.....................     1        0     TE2
    .........................................................uggaagaacgaacaagacugua....................     3        0     TE2
    .........................................................uggaagaacgaacaagacuguag...................     2        0     TE2
    ...........................................................gaagaacgaacaagacuguagUu.................     1        1     TE2

    ......................acuguuuuguucguuguucuca.......................................................     1        0     MF2
    ......................acuguuuuguucguuguucucaca.....................................................     2        0     MF2
    .........................................................uggaagaacgaacaagacugu.....................     1        0     MF2

    ..........................uuuugCucguuguucuc........................................................     1        1     OV1
    .........................................................uggaagaacgaacaagacugu.....................     1        0     OV1

    ......................acuguuuuguucguuguucucU.......................................................     1        1     FF1
```

miRBase precursor : aga-mir-31
Total read count : 2859
aga-mir-31 read count : 2763
aga-mir-31-star read count : 95
remaining reads : 1

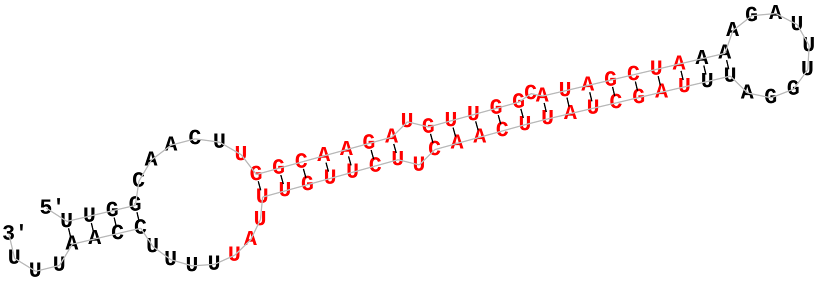

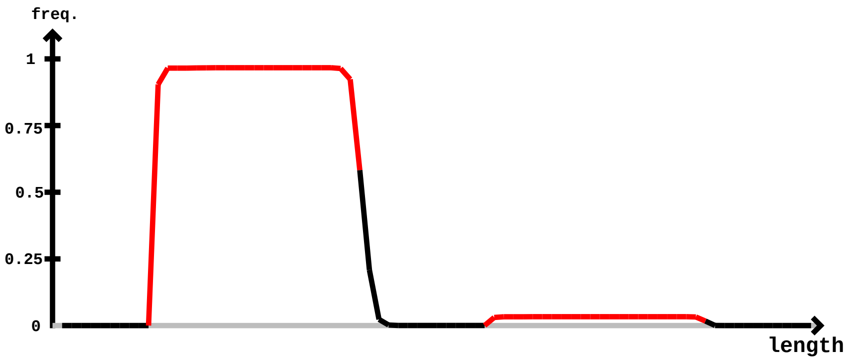

aga-mir-31-star

aga-mir-31

5'- uuggcaacuuggcaagauguuggcauagcuaaaagauuuggauuuagcuauucaacuucuuguuuauuuuuccaauuu -3'	exp		
((((......(((((((.(((((.(((((((((.........)))))))))))))).))))))).......))))...	reads	mm	sample
.........uggcaagauguuggcauagcua...	7	0	TE1
.........uggcaagauguuggcauagcuaU..	1	1	TE1
.........uggcaagauguuggcauagcuaa..	3	0	TE1
..........ggcaagauguuggcauagcuaa..	1	0	TE1
..........ggcaagauguuggAauagcuaaa...	1	1	TE1
.........uggcaagauguuggcauag..	1	0	OV2
.........uggcaagauguuggcauagc...	6	0	OV2
.........uggcaagauguuggcauagcG..	1	1	OV2
.........uggcaagauguuggcauagcu..	47	0	OV2
.........uggcaagauguuggcauagcua...	27	0	OV2
.........uggcaagauguuggcauagcuaa..	14	0	OV2
.........uggcaagauguuggcauagcuaaa...	2	0	OV2
.........uggcaagauguuggcauagcuaaU...	1	1	OV2
..........ggcaagauguuggcauagc...	1	0	OV2
..........ggcaagauguuggcauagcu..	5	0	OV2
..........ggcaagaugCuggcauagcu..	1	1	OV2
..........ggcaagauguuggcauagcua...	2	0	OV2
..........ggcaagauguuggcauagcuaa..	1	0	OV2
....uagcuauucaacuucuuguuua...........	4	0	OV2
.........uggcaagauguuggcauag..	1	0	FF2
.........uggcaagauguuggcauagc...	4	0	FF2
.........uggcaagauguuggcauagcu..	61	0	FF2
.........uggcaagauguuggcauagcua...	69	0	FF2
.........uggcaagauguuggcauagcuU...	2	1	FF2
.........uggcaagauguuggcauagAua...	1	1	FF2
.........uUgcaagauguuggcauagcua...	1	1	FF2
.........uggcaagauguuggcauagcuaC..	1	1	FF2
.........uggcaagauguuggcauagcuaa..	24	0	FF2
.........uggcaagauguuggcauagcuaaa...	1	0	FF2
..........ggcaagauguuggcauagc...	3	0	FF2
..........ggcaagauguuggcauagcu..	6	0	FF2
..........ggcaagauguuggcauagcua...	3	0	FF2

```
                                            aga-mir-31-star
         aga-mir-31
uuggcaacuuggcaagauguuggcauagcuaaaagauuuggauuuagcuauucaacuucuuguuuauuuuccaauuu

..........ggcaagauguuggcauagcuaa..............................................  3     0     FF2
............................................uagcuauucaacuucuuguuua............  1     0     FF2
............................................uagcuauucaacuucuuguuuau...........  1     0     FF2
.............................................agcuauucaacuucuuguuuau...........  2     0     FF2

.........uggcaagauguuggcauagc.................................................  28    0     MF2
.........uggcaaAauguuggcauagcu................................................  1     1     MF2
.........uggcaagauguAggcauagcu................................................  1     1     MF2
.........uggcaagauguuggcaCagcu................................................  1     1     MF2
.........uggcaagauguuggcauagcu................................................  369   0     MF2
.........uggcaagauguuggcauagcG................................................  2     1     MF2
.........uggcaagauguuggcauaAcu................................................  1     1     MF2
.........uggcaagauAuuggcauagcu................................................  1     1     MF2
.........uggcaagauguuggcauagcuU...............................................  4     1     MF2
.........uggcaagauguuggcauagcuC...............................................  1     1     MF2
.........uggcaagauguuggUauagcua...............................................  1     1     MF2
.........uggcaagauguuggcauagcua...............................................  428   0     MF2
.........uggcaagauguuggcauagcuaU..............................................  14    1     MF2
.........uggcaagauguAggcauagcuaa..............................................  1     1     MF2
.........uggcaagauguuggcauagcuaC..............................................  1     1     MF2
.........uggcaagauguuggcauagcuaa..............................................  227   0     MF2
.........uggcaagauguuggcauagcuaaa.............................................  13    0     MF2
.........uggcaagauguuggcauagcuaCa.............................................  2     1     MF2
.........uggcaagauguuggcauagcuaaG.............................................  2     1     MF2
.........uggcaagauguuggcauagcuaaU.............................................  18    1     MF2
.........uggcaagauguuggcauagcuaaaU............................................  2     1     MF2
.........uggcaagauguuggcauagcuaaaaa...........................................  1     0     MF2
..........ggcaagauguuggcauagc.................................................  2     0     MF2
..........ggcaagauguuggcauagcu................................................  17    0     MF2
..........ggcaagauguuggcauagcua...............................................  12    0     MF2
..........ggcaagauguuggcauagcuaa..............................................  6     0     MF2
..........ggcaagauguuggcauagcuaU..............................................  1     1     MF2
..........ggcaagauguuggcauagcuaaU.............................................  1     1     MF2
............................................uagcuauucaacuucuuguuua............  18    0     MF2
............................................uagcuauucaacuucuuguuuaA...........  1     1     MF2
............................................uagcuauucaacuucuuguuuau...........  22    0     MF2
............................................uagcuauucaacuucuuguuuauu..........  1     0     MF2
.............................................agcuauucaacuucuuguuua............  2     0     MF2

.........uggcaagauguuggcauagc.................................................  7     0     FW2
.........uggcaagauguuggcauagcu................................................  16    0     FW2
.........uggcaagauguuggcauagcua...............................................  48    0     FW2
.........uggcaagauUuuggcauagcua...............................................  1     1     FW2
.........uggcaagauguuggcauagcuaa..............................................  23    0     FW2
.........uggcaagauguuggcauagcuaaU.............................................  2     1     FW2
..........ggcaagauguuggcauag..................................................  1     0     FW2
..........ggcaagauguuggcauagcu................................................  4     0     FW2
..........ggcaagauguuggcauagcua...............................................  1     0     FW2
..........ggcaagauguuggcauagcuaa..............................................  7     0     FW2
..........ggcaagauguuggcauagcuaaa.............................................  1     0     FW2
............................................uagcuauucaacuucuuguuu.............  1     0     FW2
............................................uagcuauucaacuucuuguuua............  2     0     FW2
............................................uagcuauucaacuucuuguuuau...........  1     0     FW2

.........uggcaagauguuggcauag..................................................  1     0     OV1
.........uggcaagauguuggcauagc.................................................  7     0     OV1
.........uggcaagauguuggcauagcu................................................  77    0     OV1
.........uggcaagauguuggcaAagcua...............................................  1     1     OV1
.........uggcaagauguuggcauagcua...............................................  43    0     OV1
.........uggcaagauguuggcauagcuU...............................................  2     1     OV1
.........uggcaagauguuggcauagcuaa..............................................  14    0     OV1
.........uggcaagauguuggcauagcuaU..............................................  2     1     OV1
..........ggcaagauguuggcauag..................................................  1     0     OV1
..........ggcaagauguuggcauagc.................................................  2     0     OV1
..........ggcaagauguuggcauagcu................................................  6     0     OV1
..........ggcaagauguuggcauagcua...............................................  2     0     OV1
..........ggcaagauguuggcauagcuaa..............................................  3     0     OV1

.........uggcaagauguuggcauag..................................................  1     0     FF1
```

```
                                            aga-mir-31-star
         aga-mir-31
uuggcaacuuggcaagauguuggcauagcuaaaagauuuggauuuagcuauucaacuucuuguuuauuuuuccaauuu

.........uggcaagauguuggcauagc.................................................  28   0  FF1
.........uggcGagauguuggcauagcu................................................  3    1  FF1
.........uggcaagauguuggcauagcu................................................  154  0  FF1
.........uggGaagauguuggcauagcu................................................  6    1  FF1
.........Gggcaagauguuggcauagcu................................................  1    1  FF1
.........uggcaagauCuuggcauagcu................................................  1    1  FF1
.........uggcaagauguuggcaCagcu................................................  1    1  FF1
.........uggcaagauguuggcaGagcu................................................  1    1  FF1
.........uggcaagauguuggcauagcua...............................................  125  0  FF1
.........uggcaagauguugAcauagcua...............................................  1    1  FF1
.........uggcaagauguuggAauagcua...............................................  1    1  FF1
.........uggcGagauguuggcauagcua...............................................  1    1  FF1
.........uggcaagauguuggcauagcuU...............................................  4    1  FF1
.........uggcaagauguuUgcauagcua...............................................  1    1  FF1
.........uggcaagauguuggGauagcua...............................................  1    1  FF1
.........Gggcaagauguuggcauagcua...............................................  1    1  FF1
.........uggGaagauguuggcauagcua...............................................  2    1  FF1
.........uggGaagauguuggcauagcuaa..............................................  4    1  FF1
.........uggcaagauguuggcauagcuaa..............................................  42   0  FF1
.........uggcaagauguuggcauagcuaU..............................................  3    1  FF1
.........uggcaagauguuggcauagcuaaa.............................................  3    0  FF1
..........ggcaagauguuggcauagc.................................................  1    0  FF1
..........ggcaagauguuggcauagcu................................................  9    0  FF1
..........ggcaagauguuggcauaUcu................................................  1    1  FF1
..........ggcaagauguuggcauagcua...............................................  5    0  FF1
..........ggcaagauguuggcauagcuaa..............................................  1    0  FF1
..........ggcaagauguuggcauagcuCa..............................................  1    1  FF1
..........ggcaagauguuggcauagcuaaa.............................................  1    0  FF1
............................................uagcuauucaacuucuuUuuu.............  1    1  FF1
............................................uagcuauucaacuucuuguuu.............  1    0  FF1
............................................uagcuauucaacuucuuguuua............  7    0  FF1
............................................uagcuauucaacuucuuguuuau...........  6    0  FF1
............................................uagcuauucaacuucuuguuuaA..........  1    1  FF1
............................................uagcuauucaacuucuuUuuuau...........  1    1  FF1

.........uggcaagauguuggcauag..................................................  1    0  MF1
.........uggcaagauguuggcauagc.................................................  5    0  MF1
.........uggcaagauguuggcauaUc.................................................  1    1  MF1
.........uggcaagauguuggcauagcu................................................  36   0  MF1
.........uggcaagauguuggcauagcua...............................................  61   0  MF1
.........uggcaagauguuggcauagcuU...............................................  3    1  MF1
.........uggcaagauguuggcauagcuaa..............................................  19   0  MF1
.........uggcaagauguuggcauagcuaC..............................................  1    1  MF1
.........uggcaagauguuggcauagcuaU..............................................  1    1  MF1
.........uggcaagauguuggcauagcuaaa.............................................  1    0  MF1
..........ggcaagauguuggcauagc.................................................  3    0  MF1
..........ggcaagauguuggcauagcu................................................  4    0  MF1
..........ggcaagauguuggcauagcua...............................................  2    0  MF1
..........ggcaagauguuggcauagcuaa..............................................  1    0  MF1
..........ggcaagauguuggcauagcuaaaagauuuggauu..................................  1    0  MF1
............................................uagcuauucaacuucuuguuua............  3    0  MF1
............................................uagcuauucaacuucuuguuuau...........  1    0  MF1

.........uggcaagauguuggcauagc.................................................  3    0  BF2
.........uggcaagauguuggcauagcu................................................  19   0  BF2
.........uggcaagauguuggcauagcuU...............................................  2    1  BF2
.........uggcaagauguuggcauagcua...............................................  16   0  BF2
.........uggcaagauguuggcauagcuaU..............................................  1    1  BF2
.........uggcaagauguuggcauagcuaa..............................................  5    0  BF2
..........ggcaagauguuggcauagcu................................................  2    0  BF2
..........ggcaagauguuggcauagcua...............................................  3    0  BF2
..........ggcaagauguuggcauagcuaa..............................................  1    0  BF2
............................................uagcuauucaacuucuuguuua............  2    0  BF2
............................................uagcuauucaacuucuuguuuau...........  1    0  BF2
.............................................agcuauucaacuucuuguuuau...........  1    0  BF2

.........uggcaagauguuggcauagcu................................................  7    0  BF1
.........uggcaagauguuggcauagcuU...............................................  1    1  BF1
.........uggcaagauguuggcauagcua...............................................  9    0  BF1
```

```
                          aga-mir-31-star
         aga-mir-31
uuggcaacuuggcaagauguuggcauagcuaaaagauuuggauuuagcuauucaacuucuuguuuauuuuuccaauuu

.........uggcaagauguuggcauagcuaa..............................................  5    0    BF1
.........uggcaagauguuggcauagcuaU..............................................  1    1    BF1
..........ggcaagauguuggcauagcua...............................................  1    0    BF1
..........ggcaagauguuggcauagcuaa..............................................  1    0    BF1

.........uggcaagauguuggcauagc.................................................  5    0    FW1
.........uggcaagauguuggcauagcu................................................  24   0    FW1
.........uggcaagauguuggcauagcua...............................................  37   0    FW1
.........uggcaagauguuggcauagcuaa..............................................  20   0    FW1
.........uggcaagauguuggcUuagcuaa..............................................  1    1    FW1
.........uggcaagauguuggcauagcuaaU.............................................  1    1    FW1
.........uggcaagauguuggcauagcuaaG.............................................  1    1    FW1
..........ggcaagauguuggcauagcu................................................  2    0    FW1
..........ggcaagauguuggcauagcuaa..............................................  6    0    FW1
..........ggcaagauguuggcauagcuaaU.............................................  1    1    FW1
..........ggcaagauguuggcauagcuaaa.............................................  1    0    FW1
.............aagauguuggcauagcuaa..............................................  1    0    FW1
............................................uagcuauucaacuucuuguuua............  1    0    FW1
............................................uagcuauucaacuucuuguuuau...........  2    0    FW1
.............................................agcuauucaacuucuuguuua............  1    0    FW1
.............................................agcuauucaacuucuuguuuau...........  1    0    FW1

.........uggcaagauguuggcauag..................................................  1    0    MW1
.........uggcaagauguuggcauagc.................................................  5    0    MW1
.........uggcaagauguuggcauagcu................................................  62   0    MW1
.........uAgcaagauguuggcauagcua...............................................  1    1    MW1
.........uggcaagauguuggcauagcua...............................................  96   0    MW1
.........uggcaagauguuggcauagcuaa..............................................  47   0    MW1
.........uggcaagauguuggcauagcuaU..............................................  1    1    MW1
.........uggcaagauguuggcauagcuaaa.............................................  1    0    MW1
.........uggcaagauguuggcauagcuaaU.............................................  3    1    MW1
.........uggcaagauguuggcauagcuaaaC............................................  1    1    MW1
..........ggcaagauguuggcauagc.................................................  1    0    MW1
..........ggcaagauguuggcauagcG................................................  1    1    MW1
..........ggcaagauguuggcauagcu................................................  8    0    MW1
..........ggcaagauguuggcauagcua...............................................  4    0    MW1
..........ggcaagauguuggcauagcuaa..............................................  7    0    MW1
..........ggcaagauguuggcauagcuaaa.............................................  1    0    MW1
..........ggcaagauguuggcauagcuaaaU............................................  1    1    MW1
.............aagauguuggcauagcu................................................  1    0    MW1
..............agauguuggcauagcua...............................................  1    0    MW1
............................................uagcuauucaacuucuuguuua............  3    0    MW1
............................................uagcuauucaacuucuuguuuau...........  4    0    MW1
................................................uauucaacuucuuguuuau...........  1    0    MW1

.........uggcaagauguuggcauagcu................................................  2    0    MW2
.........uggcaagauguuggcauagcua...............................................  4    0    MW2
.........uggcaagauguuggcauagcuaa..............................................  2    0    MW2
..........ggcaagauguuggcauagcu................................................  2    0    MW2
..........ggcaagauguuggcauagcua...............................................  2    0    MW2
..........ggcaagauguuggcauagcuaa..............................................  2    0    MW2
...............gauguuggcauagcuaa..............................................  1    0    MW2
............................................uagcuauucaacuucuuguuuau...........  1    0    MW2

.........uggcaagauguuggcauagc.................................................  4    0    TE2
.........uggcaagauguuggcauagcu................................................  9    0    TE2
.........uggcaagauguuggcauagcua...............................................  27   0    TE2
.........uggcaagauguuggcauagcuaa..............................................  7    0    TE2
.........uggcaagauguuggcauagcuaaU.............................................  2    1    TE2
..........ggcaagauguuggcauagcu................................................  1    0    TE2
..........ggcaagauguuggcauagcua...............................................  1    0    TE2
..........ggcaagauguuggcauagcuaa..............................................  2    0    TE2
```

miRBase precursor : aga-mir-33
Total read count : 1027
aga-mir-33 read count : 1009
aga-mir-33-star read count : 18
remaining reads : 0

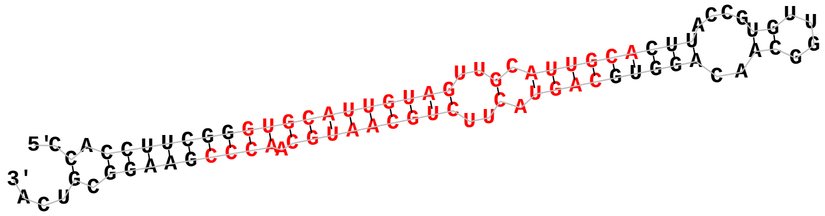

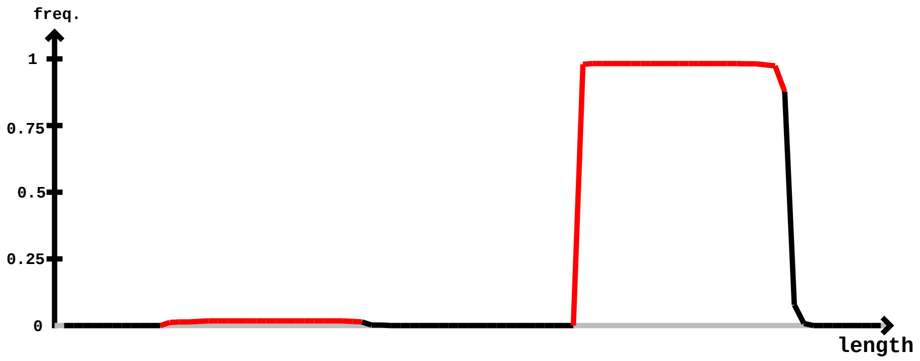


```
                                                     aga-mir-33
          aga-mir-33-star
5'- ccaccuucgggugcauuguaguugcauugcacuuaccguguuggcaacaggugcaguacuucugcaaugcaacccgaaggcguca  -3'    exp
    .(.((((((((((((((((((..(.(((((((((....((....))..))))))))).)..))))))))).))))))))).)...    reads      mm      sample
    .....................................................caguacuucugcaaugcaaccU..........    1          1       TE1
    .....................................................caguacuucugcaaugcaaccc..........    9          0       TE1

    .....................................................caguacuucugcaaugcaacc...........    1          0       FF2
    .....................................................cagGacuucugcaaugcaaccc..........    1          1       FF2
    .....................................................caguacuucugcaaugcaaccU..........    1          1       FF2
    .....................................................caguacuucugcaaugcaaccc..........    44         0       FF2
    .....................................................caguacuucugcaaugcaacccg.........    1          0       FF2
    .....................................................caguacuucugcaaugcaacccU.........    2          1       FF2
    .....................................................caguacuucugcaaugcaacccA.........    2          1       FF2
    ......................................................aguacuucugcaaugcaaccc..........    1          0       FF2

    ..........gugcauuguaguugcauugc.......................................................    1          0       OV2
    .....................................................caguacuucugcaaugcaa.............    1          0       OV2
    .....................................................caguacuucugcaaugcaacc...........    5          0       OV2
    .....................................................caguacuucugcaaugcaaccU..........    4          1       OV2
    .....................................................caguacuucugcaaugcaaccc..........    31         0       OV2
    .....................................................caguacuucugcaaugcaacccg.........    1          0       OV2
    .....................................................caguacuucugcaaugcaacccU.........    1          1       OV2

    .....................................................caguacuucugcaaugcaac............    2          0       MF2
    .....................................................caguacuucugcaaugcaacc...........    36         0       MF2
    .....................................................cagAacuucugcaaugcaaccc..........    1          1       MF2
    .....................................................caguacuucugcaaugcaacUc..........    1          1       MF2
    .....................................................caguacuucugcaaugcaaccc..........    329        0       MF2
    .....................................................caguacuucAgcaaugcaaccc..........    1          1       MF2
    .....................................................caguacuucugcaaugcaaccU..........    14         1       MF2
    .....................................................caguacuucugcaaugcaacccC.........    1          1       MF2
    .....................................................caguacuucugcaaugcaacccg.........    5          0       MF2
    .....................................................caguacuucugcaaugcaacccU.........    9          1       MF2
    .....................................................caguacuucugcaaugcaacccA.........    6          1       MF2
    .....................................................caguacuucugcaaugcaacccgU........    1          1       MF2

    ..........gugcauuguaguugcauug........................................................    1          0       FW2
```

aga-mir-33

aga-mir-33-star

```
ccaccuucgggugcauuguaguugcauugcacuuaccguguuggcaacaggugcaguacuucugcaaugcaacccgaaggcguca

..........gugcauuguaguugcauugca......................................................    2    0    FW2
.............cauuguaguugcauugca......................................................    2    0    FW2
.....................................................caguacuucugcaaugcaacc...........    5    0    FW2
.....................................................caguCcuucugcaaugcaaccc..........    1    1    FW2
.....................................................caguacuucugcaaugcaaccc..........    65   0    FW2
.....................................................caguacuucugcaaugcaaccU..........    1    1    FW2
.....................................................caguacuucugcaaugcaacccg.........    2    0    FW2

.....................................................caguacuucugcaaugcaacc...........    7    0    OV1
.....................................................caguacuucugcaaugcaaccc..........    35   0    OV1
.....................................................caguacuucugcaaugcaaccU..........    1    1    OV1
.....................................................caguacuucugcaaugcaacccA.........    1    1    OV1
.....................................................caguacuucugcaaugcaacccg.........    4    0    OV1
......................................................aguacuucugcaaugcaacccga........    1    0    OV1

..........gugcauuguaguugcauugca......................................................    2    0    FF1
.....................................................cagGacuucugcaaugcaa.............    1    1    FF1
.....................................................caguacuucugcaaugcaa.............    2    0    FF1
.....................................................caguacuucugcaaugcaacc...........    2    0    FF1
.....................................................caguacuucugcaaugcaaccU..........    1    1    FF1
.....................................................cagGacuucugcaaugcaaccc..........    2    1    FF1
.....................................................caguacuucugcaaugcaaccc..........    24   0    FF1
.....................................................caguacuucugcaaugcaacccga........    1    0    FF1
......................................................aguacuucugcaaugcaaccc..........    1    0    FF1

.....................................................caguacuucugcaaugcaacc...........    12   0    MF1
.....................................................caguacuucugcaaugcaaccG..........    1    1    MF1
.....................................................caguacuucugcaaugcaaccU..........    3    1    MF1
.....................................................caguacuucugcaaugcaaccc..........    68   0    MF1
.....................................................caguacuucugcaaugcaacccg.........    1    0    MF1
.....................................................caguacuucugcaaugcaacccU.........    5    1    MF1

..........gugcauuguaguugcauugca......................................................    1    0    BF2
..............auuguaguugcauugca......................................................    1    0    BF2
.....................................................caguacuucugcaaugcaac............    1    0    BF2
.....................................................caguacuucugcaaugcaacc...........    7    0    BF2
.....................................................caguacuucugcaaugcaaccU..........    3    1    BF2
.....................................................caguacuucugcaaugcaaccc..........    43   0    BF2
.....................................................caguacuucugcaaugcaacccg.........    3    0    BF2
.....................................................caguacuucugcaaugcaacccU.........    3    1    BF2
.....................................................caguacuucugcaaugcaacccgU........    2    1    BF2

.....................................................caguacuucugcaaugcaacc...........    2    0    BF1
.....................................................caguacuucugcaaugcaaccc..........    36   0    BF1
.....................................................caguacuucugcaaugcaacccg.........    7    0    BF1
.....................................................caguacuucugcaaugcaacccU.........    5    1    BF1
.....................................................caguacuucugcaaugcaacccgU........    2    1    BF1

..........gugcauuguaguugcauugca......................................................    1    0    FW1
.....................................................caguacuucugcaaugc...............    1    0    FW1
.....................................................caguacuucugcaaugcaac............    1    0    FW1
.....................................................caguacuucugcaaugcaacc...........    3    0    FW1
.....................................................caguacuucugcaaugcaaccU..........    1    1    FW1
.....................................................caguacuucugcaaugcaaccc..........    26   0    FW1
.....................................................caguacuucuCcaaugcaaccc..........    1    1    FW1
.....................................................caguacuucugcaaugcaacccg.........    1    0    FW1
.....................................................caguacuucugcaaugcaacccU.........    1    1    FW1

...........ugcauuguaguugcauugcacu....................................................    2    0    MW1
..............auuguaguugcauugca......................................................    1    0    MW1
.....................................................caguacuucugcaaugcaacc...........    11   0    MW1
.....................................................caguacuucugcaaugAaaccc..........    1    1    MW1
.....................................................caguacuucugcaaugcaaccU..........    1    1    MW1
.....................................................caguacuucugcaaugcaaccc..........    40   0    MW1
.....................................................caguacuucugcaaugcaacccA.........    1    1    MW1
.....................................................caguacuucugcaaugcaacccU.........    1    1    MW1
.....................................................caguacuucugcaaugcaacccg.........    2    0    MW1

..........gugcauuguaguugcauug........................................................    1    0    MW2
```

```
                                                     aga-mir-33
          aga-mir-33-star
ccaccuucgggugcauuguaguugcauugcacuuaccguguuggcaacaggugcaguacuucugcaaugcaacccgaaggcguca

..........gugcauuguaguugcauugc.......................................................   1    0    MW2
..........gugcauuguaguugcauugca......................................................   2    0    MW2
.....................................................caguacuucugcaaugcaacc...........   1    0    MW2
.....................................................caguacuucugcaaugcaaccc..........   12   0    MW2
.....................................................caguacuucugcaaugcaacccg.........   1    0    MW2
.....................................................caguacuucugcaaugcaacccgU........   1    1    MW2

.....................................................caguacuucugcaaugcaacc...........   7    0    TE2
.....................................................caguacuucugcaaugcaaccc..........   14   0    TE2
.....................................................caguacuucugcaaugcaaccU..........   2    1    TE2
.....................................................caguacuucugcaaugcaacccg.........   4    0    TE2
.....................................................caguacuucugcaaugcaacccU.........   2    1    TE2
```

miRBase precursor : aga-mir-932
Total read count : 143
aga-mir-932 read count : 138
aga-mir-932-star read count: 1
remaining reads : 4

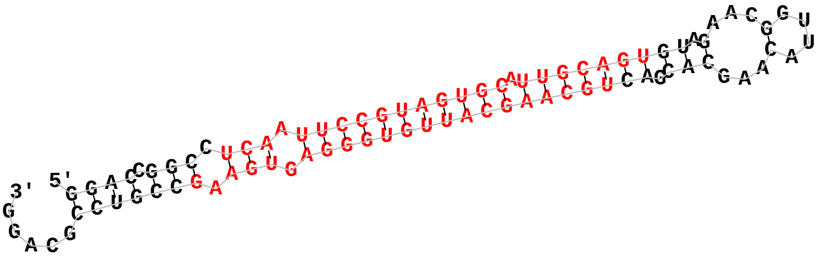

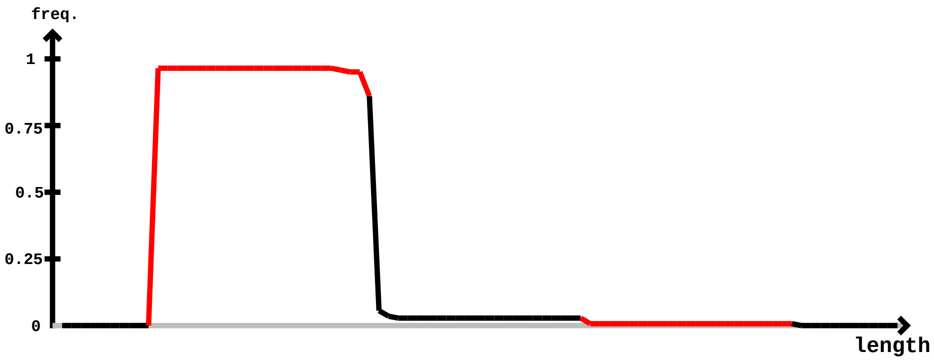


```
                                                                aga-mir-932-star
          aga-mir-932
5'- ggaccggccucaauuccguagugcauugcaguguagaacgguuacaagcacgacugcaagcauugugggagugaagccguccgcagg  -3'    exp
    ((((.(((.(((.(((((((((((.(((((((((.(...(....)...))).))))))))))))))))).))).))))))).....     reads    mm    sample
    .........ucaauuccguagugcauugcagC.......................................................     1        1     TE1
    .........ucaauuccguagugcauugcagu.......................................................     1        0     TE1

    .........ucaauuccguagugcauug...........................................................     1        0     OV2
    .........ucaauuccguagugcauugcag........................................................     4        0     OV2
    .........ucaauuccguagugcauugcagu.......................................................     7        0     OV2
    .........ucaauuccguagugcauugcaguUu.....................................................     1        1     OV2

    .........ucaauuccguagugcauugcagu.......................................................     6        0     MF2

    .........ucaauuccguagugcauugcag........................................................     2        0     FW2
    .........ucaauuccguagugcauugcagu.......................................................     32       0     FW2
    .........ucaauuccguagugcauugcaguU......................................................     1        1     FW2
    ................................guagaacgguuacaagcacgac.................................     2        0     FW2
    ......................................................ugcaagcauugugggagugaag...........     1        0     FW2

    .........ucaauuccguagugcauugcagu.......................................................     1        0     OV1

    .........ucaauuccguagugcauugcagu.......................................................     1        0     FF1

    .........ucaauuccguagugcauugcagu.......................................................     1        0     MF1

    .........ucaauuccguagugcauugcagu.......................................................     2        0     BF1

    .........ucaauuccguagugcauugc..........................................................     1        0     MW1
    .........ucaauuccguagugcauugcag........................................................     3        0     MW1
    .........ucaauuccguagugcauugcagu.......................................................     32       0     MW1
    .........ucaauuccguagugcauugcagG.......................................................     1        1     MW1

    .........ucaauuccguagugcauugcag........................................................     4        0     FW1
    .........ucaauuccguagugcauugcagu.......................................................     24       0     FW1

    .........ucaauuccguagugcauugcagu.......................................................     8        0     MW2
    .........ucaauuccguagugcauugcaguU......................................................     2        1     MW2
```

```
                                                    aga-mir-932-star
         aga-mir-932
ggaccggccucaauuccguagugcauugcaguguagaacgguuacaagcacgacugcaagcauugugggagugaagccguccgcagg

................................guagaacgguuacaagcacgac.................................    2          0          MW2

.........ucaauuccguagugcauugcagu.......................................................    2          0          TE2
```

miRBase precursor : aga-mir-965-1
Total read count : 155
aga-mir-965-1 read count : 128
aga-mir-965-1-star read count : 2
remaining reads : 25

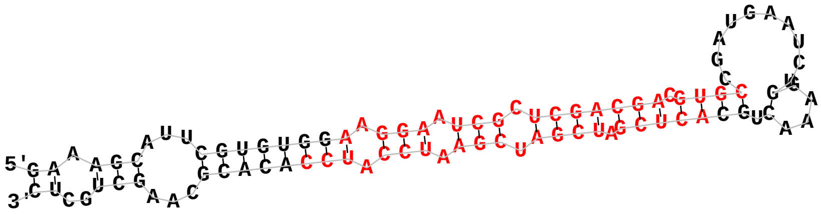

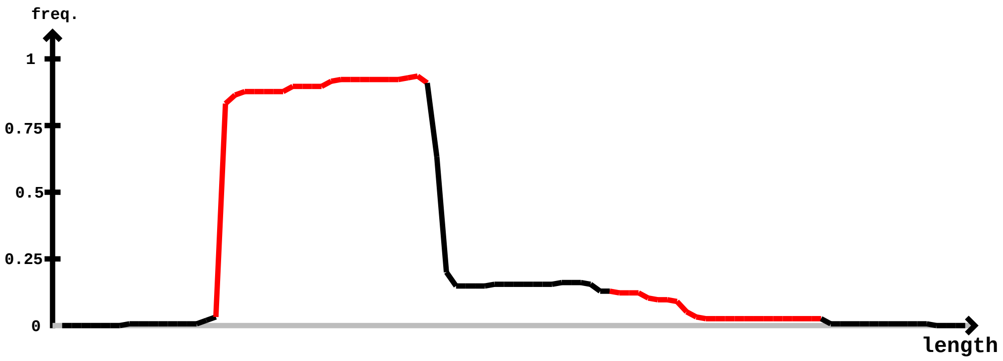

aga-mir-965-1-star

aga-mir-965-1

```
5'- gaaagcauucgugugGaaggaaucgcucgacgacgugccgaugaaucuggaaacugcacucgaucgaucgaauccauccacacgcaagcugcuc   -3'    exp
    ...(((...((((((((.(((.(((.(((((((.((((.((....)).(....).))))))).)))).))).))).))))))))...)))....    reads   mm   sample
    ................aaggaaucgcucgacgacgugc........................................................    1       0    FF2

    ................aaggaaucgcucgacgacgugc........................................................    3       0    OV2
    ................aaggaaucgcucgacgacgugcc.......................................................    3       0    OV2
    ........................................augaaucuggaaacugcacucgaucgaucgaauccaucc...............    1       0    OV2

    ................aaggaaucgcucgacgacgug.........................................................    1       0    TE1
    ................aaggaaucgcucgacgacgugc........................................................    5       0    TE1
    ................aaggaaucgcucgacgacgugcc.......................................................    4       0    TE1
    ...................................ugccgaugaaucuggaaacugcacucgaCcg............................    1       1    TE1

    ..............ggaaggaaucgcucgacgacgugccgaugaaucuggaaacugcacucgaC..............................    1       1    MF2
    ...............gaaggaaucgcucgacgacgugccgaugaaucuggaaacugcacucgaC..............................    1       1    MF2
    ................aaggaaucgcucgacgacg...........................................................    1       0    MF2
    ................aaggaaucgcucgacgacgug.........................................................    2       0    MF2
    ................aaggaaucgcucgacgacgugc........................................................    7       0    MF2
    ................aaggaaucgcucgacgacgugcc.......................................................    14      0    MF2
    ................aaggaaucgcucgacgacgugccU......................................................    4       1    MF2
    ................aaggaaucgcucgacgacgugccgaugaaucuggaaacu.......................................    1       0    MF2
    .................aggaaucgcucgacgacgugccgaugaaucuggaaacu.......................................    1       0    MF2
    ..................ggaaucgcucgacgacgugccgaugaaucuggaaacugcacucgau..............................    2       0    MF2
    .......................cgcucgacgacgugccgaugaaucuggaaacugcacu..................................    1       0    MF2
    ...........................cgacgacgugccgaugaaucuggaaacugc.....................................    1       0    MF2
    ...........................cgacgacgugccgaugaaucuggaaacugcacu..................................    2       0    MF2
    ............................gacgacgugccgaugaaucuggaaacug......................................    1       0    MF2
    ...................................ugccgaugaaucuggaaacugcacucgaCc.............................    2       1    MF2
    ......................................cgaCgaaucuggaaacu.......................................    1       1    MF2
    ............................................aucuggaaacugcacucgaC..............................    1       1    MF2

    ................aaggaaucgcucgacgacgugc........................................................    1       0    FW2
    ................aaggaaucgcucgacgacgugcc.......................................................    1       0    FW2
    .......................cgcucgacgacgugccgaugaaucuggaaacugc.....................................    1       0    FW2
    .......................cgcucgacgacgugccgaugaaucuggaaacugcacuc.................................    1       0    FW2

    ................aaggaaucgcucgacgacgugc........................................................    1       0    OV1
```

```
                                                                aga-mir-965-1-star
                aga-mir-965-1
gaaagcauucguguggaaggaaucgcucgacgacgugccgaugaaucuggaaacugcacucgaucgaucgaauccauccacacgcaagcugcuc

................aaggaaucgcucgacgacgugcc.......................................................    4    0    OV1
.................aggaaucgcucgacgacgugcc.......................................................    1    0    OV1

................aaggaaucgcucgacgacgugc........................................................    10   0    FF1
................aagUaaucgcucgacgacgugc........................................................    1    1    FF1
................aaggaaucgcucgacgacgugcc.......................................................    4    0    FF1
................aagUaaucgcucgacgacgugcc.......................................................    1    1    FF1
................aGggaaucgcucgacgacgugcc.......................................................    1    1    FF1
......................................cgaCgaaucuggaaacu.......................................    1    1    FF1
......................................cgaugaaucuggaaacugcacucgaCc.............................    1    1    FF1

..............ggaaggaaucgcucgacgacgugccgaugaaucuggaaacugcacucgaC..............................    1    1    MF1
................aaggaaucgcucgacgacg...........................................................    1    0    MF1
................aaggaaucgcucgacgacgugc........................................................    1    0    MF1
................aaggaaucgcucgacgacgugccU......................................................    2    1    MF1

......auucguguggaaggaaucgcucgacgacgugccgaugaaucuggaaac........................................    1    0    BF2
................aaggaaucgcucgacgacgugc........................................................    1    0    BF2
................aaggaaucgcucgacgacgugcc.......................................................    10   0    BF2
................aaggaaucgcucgacgacgugccU......................................................    1    1    BF2

................aaggaaucgcucgacgacgugcc.......................................................    4    0    BF1
.................aggaaucgcucgacgacgugcc.......................................................    1    0    BF1
...................................................aacugcacucgaucgaucgaauccauccacCcgcaagcu....    1    1    BF1

................aaggaaucgcucgacgacgugcc.......................................................    1    0    FW1

................aaggaaucgcucgacgacgugc........................................................    1    0    MW1
................aaggaaucgcucgacgacgugcc.......................................................    1    0    MW1

...............Caaggaaucgcucgacgacgugc........................................................    1    1    TE2
................aaggaaucgcucgacgacgug.........................................................    1    0    TE2
................aaggaaucgcucgacgacgugc........................................................    12   0    TE2
................aaggaaucgcucgacgacgugcc.......................................................    16   0    TE2
................aaggaaucgcucgacgacgugccU......................................................    2    1    TE2
.................aggaaucgcucgacgacgugc........................................................    1    0    TE2
.................aggaaucgcucgacgacgugcc.......................................................    1    0    TE2
....................................gccgaugaaucuggaaacugcacucga...............................    1    0    TE2
........................................................cacucgaucgaucgaauccaucc...............    1    0    TE2
.........................................................acucgaucgaucgaauccaucc...............    1    0    TE2
```

miRBase precursor : aga-mir-965-2
Total read count : 3380
aga-mir-965-2 read count : 3202
aga-mir-965-2-star read count : 142
remaining reads : 36

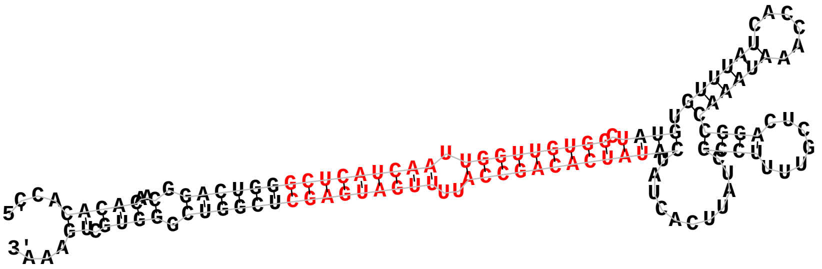

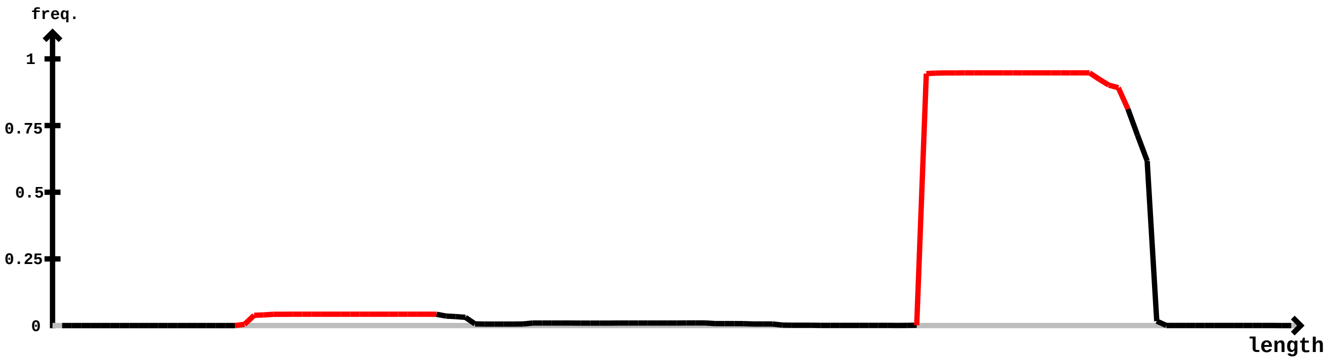

aga-mir-965-2

aga-mir-965-2-star

```
5'- ccacacacaacggacugggcucaucaauugguuguggcuauguguuuaucaccaaauaaaccggacucguuuuccgcuauucacuaucauaucacagccauuuugaugagcucggucgggugcugaaa   -3'    exp
    ...(((((..(.(((((((((((((((.(((((((((.((((.((((((......))))))((((.......))))...........)))))))))))))..)))))))))))))).))))).))...     reads    mm
    ...................cucaucaauugguuguggcuaug......................................................................................     2        0
    .........................................................aaaccggacucguuuuc......................................................     1        0
    .........................................................................................uaucacagccauuuugaC.....................     5        1
    .........................................................................................uaucacagccauuuugaCg....................     1        1
    .........................................................................................uaucacagccauuuugaugag..................     2        0
    .........................................................................................uaucacagccauuuugaCgag..................     10       1
    .........................................................................................uaucacagccauuuugaugagc.................     15       0
    .........................................................................................uaucacagccauuuugaugagcu................     5        0
    .........................................................................................uaucacagccauuuCgaugagcuc...............     1        1
    .........................................................................................uaucacagccauuuugaugagcuc...............     50       0
    .........................................................................................uaucacagccauuuugaugagcucU..............     3        1

    ..................gcucaucaauugguuguggcuau.......................................................................................     1        0
    ..................gcucaucaauugguuguggcuaug......................................................................................     1        0
    ...................cucaucaauugguuguggcu.........................................................................................     2        0
    ...................cucaucaauugCuuguggcuaug......................................................................................     1        1
    ...................cucaucaauugguuguggcuaug......................................................................................     3        0
    ...................cucaucaauugguuguggcuauguguu..................................................................................     1        0
    .....................caucaauugguuguggcuaug......................................................................................     1        0
    ..........................................uguuuaucaccaaauaaaccggacu.............................................................     1        0
    ................................................ucaccaaauaaaccggacucguuuuc......................................................     2        0
    .........................................................................................uaucacagccauuuugaCg....................     1        1
    .........................................................................................uaucacagccauuuugaCga...................     3        1
    .........................................................................................uaucacagccauuuugaugag..................     2        0
    .........................................................................................uaucacagccauuuugaCgag..................     9        1
    .........................................................................................uaucacagccauuuugaugagc.................     37       0
    .........................................................................................uaucacagccauuuugaugagcu................     7        0
    .........................................................................................uaucacagccauuuugaugagcuc...............     77       0
    .........................................................................................uaucacagccUuuuugaugagcuc...............     1        1
    .........................................................................................uaucacagccauuuugaugagcucU..............     1        1

    ..................gcucaucaauugguuguggcu.........................................................................................     1        0
    ...................cucaucaauugguuguggcuaug......................................................................................     1        0
    ...................cucaucaauCgguuguggcuaug......................................................................................     1        1
```

```
                                                                                         aga-mir-965-2
                  aga-mir-965-2-star
ccacacacaacggacugggcucaucaauugguuguggcuauguguuuaucaccaaauaaaccggacucguuuuccgcuauucacuaucauaucacagccauuuugaugagcucggucgggugcugaaa

.........................................................................................uaucacagccauuuugaCg....................  1  1
.........................................................................................uaucacagccauuuugaCgag..................  6  1
.........................................................................................uaucacagccauuuugaugagc.................  12  0
.........................................................................................uaucacagccauuuugaugagcu................  4  0
.........................................................................................uaucacagccauuuugaugagcuc...............  33  0
.........................................................................................uaucacagccauuuugaugagcucU..............  1  1

..................gcucaucaauugguuguggcuaug......................................................................................  2  0
...................cucaucaauugguuguggcu.........................................................................................  3  0
...................cucaucaauugguuguggcua........................................................................................  1  0
...................cucaucaauugguuguggcuau.......................................................................................  2  0
...................cucaucaauugguuguggcuauU......................................................................................  1  1
...................cucaucaauugguuguggcuaug......................................................................................  18  0
...................cucaucaauugguuguggcuaugA.....................................................................................  1  1
...................cucaucaauugguuguggcuauguguuuaucacc...........................................................................  1  0
....................ucaucaauuggGuguggcuaug......................................................................................  1  1
....................ucaucaauugguuguggcuaug......................................................................................  2  0
.....................caucaauugguuguggcuaug......................................................................................  1  0
..........................................uguuuaucaccaaauaaaccggacu.............................................................  4  0
..........................................uguuuaucaccaaauaaaccggacucgu.........................................................  1  0
..........................................uguuuaucaccaaauaaaccggacucguu........................................................  3  0
..........................................uguuuaucaccaaauaaaccggacucguuuuccgcuauucacuauca.......................................  1  0
............................................uuuaucaccaaauaaaccggacucguuuucc.....................................................  1  0
..............................................uaucaccaaauaaaccggacucguuuuc......................................................  1  0
........................................................................................Cuaucacagccauuuugaugagcu................  1  1
.........................................................................................uaucacagccauuuugaC.....................  18  1
.........................................................................................uaucacagccauuuugaCg....................  28  1
.........................................................................................uaucacagccauuuugaug....................  1  0
.........................................................................................uaucacagccauuuugauga...................  1  0
.........................................................................................uaucacagccauuuugaCga...................  14  1
.........................................................................................uaucacagccauuuugaugag..................  10  0
.........................................................................................uaucacagccauuuugaCgag..................  70  1
.........................................................................................uaucacagccauuuugaugagc.................  111  0
.........................................................................................uaucacagccauuuugaCgagc.................  1  1
.........................................................................................uaucacagccauuuugaugagU.................  1  1
.........................................................................................uaucacagccauuuugaugagcu................  69  0
.........................................................................................uaucacagccauuuugaCgagcu................  1  1
.........................................................................................uaucacagcUauuuugaugagcu................  1  1
.........................................................................................uaCcacagccauuuugaugagcuc...............  1  1
.........................................................................................uaucacagccauCuugaugagcuc...............  1  1
.........................................................................................uaucacagccauuuugaugagcuc...............  441  0
.........................................................................................uaucacagccUuuugaugagcuc...............  1  1
.........................................................................................uaucacagccaCuuugaugagcuc...............  1  1
.........................................................................................uaucacGgccauuuugaugagcuc...............  1  1
.........................................................................................uaucacagccauuuugGugagcuc...............  2  1
.........................................................................................uaucacagccauuuugaugagAuc...............  1  1
.........................................................................................uaucacagccauuuugaugagcCc...............  1  1
.........................................................................................uaucacagccauuuugaugagGuc...............  1  1
.........................................................................................uaucacagcAauuuugaugagcuc...............  1  1
.........................................................................................uaucacagccauuuugaugagcuU...............  2  1
.........................................................................................uaucacagccauuuugaugagcucU..............  22  1
.........................................................................................uaucacagccauuuugaugagcucggucgggugcuga..  1  0
..........................................................................................aucacagccauuuugaugagcuc...............  3  0

...................cucaucaauugguuguggcua........................................................................................  2  0
...................cucaucaauugguuguggcuaug......................................................................................  11  0
.........................................................................................uaucacagccauuuugaC.....................  13  1
.........................................................................................uaucacagccauuuugau.....................  1  0
.........................................................................................uaucacagccauuuugaug....................  1  0
.........................................................................................uaucacagccauuuugaCg....................  3  1
.........................................................................................uaucacagccauuuugaCga...................  1  1
.........................................................................................uaucacagccauuuugaCgag..................  6  1
.........................................................................................uaucacagccauuuugaugagc.................  18  0
.........................................................................................uaucacagccauuuAgaugagc.................  1  1
.........................................................................................uaucacagccauuuugaugagcu................  49  0
.........................................................................................uaucacagccauuuugaugagcuc...............  227  0
.........................................................................................Caucacagccauuuugaugagcuc...............  2  1
.........................................................................................uaCcacagccauuuugaugagcuc...............  1  1
.........................................................................................uaucacagccauuuugaugagcuU...............  1  1
```

aga-mir-965-2

aga-mir-965-2-star

ccacacacaacggacugggcucaucaauugguuguggcuauguguuuaucaccaaauaaaccggacucguuuuccgcuauucacuaucauaucacagccauuuugaugagcucggucgggugcugaaa

....uaucacagccauuuugaugagcucU..............	6	1
....uaucacagccauuuugaugagcucC..............	1	1

..................gcucaucaauugguuguggcuaug..	1	0
...................cucaucaauugguuguggcu...	3	0
...................cucaucaauugguuguggcua..	1	0
...................cucaucaauugguuguggcuau...	2	0
...................cucaucaauugguuguggcuaug..	4	0
....................caucaauugguuguggcuaug...	2	0
.......................ucaauugguuguggcuaug..	1	0
....uguuuaucaccaaauaaaccggacu...	1	0
....aucaccaaauaaaccggacucguuuuc..	1	0
....ucaccaaauaaaccggacucguuuuc..	4	0
....ucaccaaauaaaccggacucguuuucc...	1	0
....uaucacagccauuuugaCg....................	1	1
....uaucacagccauuuugaug....................	1	0
....uaucacagccauuuugaCga...................	1	1
....uaucacagccauuuugaugag..................	2	0
....uaucacagccauuuugaCgag..................	17	1
....uaucacagccauuuugaugagc.................	30	0
....uaucacagccauuuugaugagcu................	4	0
....uaucacagccauuuugaugagcuc...............	48	0
....uaucacagccauuuugaugagcucU..............	1	1
....aucacagccauuuugaCgag..................	1	1

..................gcucaucaauugguuguggcu...	1	0
..................gcucaucaauugguuguggcuaug..	1	0
...................cucaucaauugguuguggcu...	2	0
...................cucaucaauugguuguggcuaug..	13	0
...................cucaucaauugguuguggcuCug..	1	1
....................ucaucaauugguuguggcCau...	1	1
....uguuuaucaccaaauaaaccggacucguu...	1	0
....acucguuuuccgcuauucacua..	1	0
....uaucacagccauuuugaC.....................	6	1
....uaucacagccauuuugaCg....................	12	1
....uaucacagccauuuugauga...................	1	0
....uaucacagccauuuugaCga...................	1	1
....uaucacagccauuuugaugag..................	6	0
....uaucacagccauuuugaCgag..................	46	1
....uaucacagAcauuuugaugag..................	1	1
....uaucacagccauuuugaugagc.................	20	0
....uauGacagccauuuugaugagcu................	1	1
....uaucacagccauuuugaugagcu................	20	0
....uGucacagccauuuugaugagcuc...............	1	1
....uCucacagccauuuugaugagcuc...............	1	1
....uauGacagccauuuugaugagcuc...............	1	1
....uaucacagccauuuugaGgagcuc...............	1	1
....uaucacagccauuuugaugagcuc...............	93	0
....uaucacagccauuuugaugagcucU..............	3	1
....uaucacagccauuuugaugagcucA..............	1	1
....acagccauuuugaugagcu................	1	0

...................cucaucaauugguuguggcu...	2	0
...................cucaucaauugguuguggcuau...	1	0
...................cucaucaauugguuguggcuauU..	1	1
....uaucacagccauuuugaC.....................	1	1
....uaucacagccauuuugaCg....................	3	1
....uaucacagccauuuugaCga...................	1	1
....uaucacagccauuuugaCgag..................	19	1
....uaucacagccauuuugaugag..................	1	0
....uaucacagccauuuugaugagc.................	20	0
....uaucacagccauuuugaugagcu................	6	0
....uauUacagccauuuugaugagcu................	1	1
....uaucacagccauuuugaugagcuc...............	73	0
....uaucacagccauuuugaugagcucU..............	2	1
....aucacagccauuuugaugagcuc...............	1	0

...................cucaucaauugguuguggcu...	1	0
...................cucaucaauugguuguggcuau...	1	0

```
                                                                                         aga-mir-965-2
                  aga-mir-965-2-star
ccacacacaacggacugggcucaucaauugguuguggcuauguguuuaucaccaaauaaaccggacucguuuuccgcuauucacuaucauaucacagccauuuugaugagcucggucgggugcugaaa

...................cucaucaauugguuguggcuaug......................................................................................    2    0
....................ucaucaauugguuguggcuaugug....................................................................................    1    0
.....................caucaauugguuguggcuaug......................................................................................    4    0
..........................................uguuuaucaccaaauaaaccggacu.............................................................    1    0
................................................ucaccaaauaaaccggacucguuuuc......................................................    1    0
.........................................................................................uaucacagccauuuugaC.....................    3    1
.........................................................................................uaucacagccauuuugaug....................    1    0
.........................................................................................uaucacagccauuuugaCg....................    4    1
.........................................................................................uaucacagccauuuugaCga...................    2    1
.........................................................................................uaucacagccauuuugaugag..................    6    0
.........................................................................................uaucacagccauuuugaCgag..................    11   1
.........................................................................................uaucacagccauuuugaugagc.................    13   0
.........................................................................................uaucacagccauuuugaugagcu................    14   0
.........................................................................................uaucacagccauuuugaugagcuc...............    186  0
.........................................................................................uaucacagccauuuugaugagcuU...............    1    1
.........................................................................................uUucacagccauuuugaugagcuc...............    1    1
.........................................................................................uaucacagccauuuuCaugagcuc...............    1    1
.........................................................................................uaucacagccauuuugaugagcucU..............    2    1
..........................................................................................aucacagccauuuugaugagcuc...............    1    0
...........................................................................................ucacagccauuuugaugagcuc...............    1    0

...................cucaucaauugguuguggcuaug......................................................................................    1    0
..........................................uguuuaucaccaaauaaaccggacu.............................................................    1    0
..........................................uguuuaucaccaaauaaaccggacucguuuuccgcu..................................................    2    0
................................................ucaccaaauaaaccggacucguuuuc......................................................    2    0
.........................................................................................uaucacagccauuuugaC.....................    1    1
.........................................................................................uaucacagccauuuugaCg....................    3    1
.........................................................................................uaucacagccauuuugaCgag..................    9    1
.........................................................................................uaucacagccauuuugaugagc.................    4    0
.........................................................................................uaucacagccauuuugaugagcu................    5    0
.........................................................................................uaucacagccauuuugaugagcuc...............    32   0
.........................................................................................uaucacagUcauuuugaugagcuc...............    1    1

..................gcucaucaauugguuguggc..........................................................................................    1    0
..................gcucaucaauugguuguggcu.........................................................................................    1    0
..................gcucaucaauugguuguggcuaug......................................................................................    2    0
..................gcucaucaauugguuguggcuaugu.....................................................................................    1    0
...................cucaucaauugguuguggcu.........................................................................................    4    0
...................cucaucaauugguuguggcua........................................................................................    2    0
...................cucaucaauugguuguggcuau.......................................................................................    2    0
...................cucaucaauugguuguggcuaug......................................................................................    8    0
.......................................................................................cauaucacagccauuuugaC.....................    1    1
.........................................................................................uaucacagccauuuugaC.....................    13   1
.........................................................................................uaucacagccauuuugaCg....................    4    1
.........................................................................................uaucacagccauuuugaCga...................    2    1
.........................................................................................uaucacagccauuuugaCgag..................    5    1
.........................................................................................uaucacagccauuuugaugaU..................    1    1
.........................................................................................uaucacagccauuuugaugagc.................    11   0
.........................................................................................uaucacagccauuuugaugagcu................    73   0
.........................................................................................uaucacagccauuuGgaugagcuc...............    1    1
.........................................................................................uaucacagccauCuugaugagcuc...............    1    1
.........................................................................................uaucacagccauuuugaugagcuc...............    420  0
.........................................................................................uaucacagccauuuugauAagcuc...............    1    1
.........................................................................................uaucacagccauuuugaugagcucU..............    7    1
..........................................................................................aucacagccauuuugaugagcuc...............    1    0
...........................................................................................ucacagccauuuugaugagcuc...............    1    0

..................gcucaucaauugguuguggcu.........................................................................................    1    0
..................gcucaucaauugguuguggcuaug......................................................................................    2    0
...................cucaucaauugguuguggcu.........................................................................................    1    0
...................cucaucaauugguuguggcuaug......................................................................................    7    0
........................................................................................Cuaucacagccauuuugaugagcuc...............    1    1
.........................................................................................uaucacagccauuuugaC.....................    11   1
.........................................................................................uaucacagccauuuugaCg....................    4    1
.........................................................................................uaucacagccauuuugaCga...................    1    1
.........................................................................................uaucacagccauuuugauga...................    1    0
.........................................................................................Aaucacagccauuuugaugag..................    1    1
.........................................................................................uaucacagccauuuugaugag..................    2    0
.........................................................................................uaucacagccauuuugaugagc.................    12   0
```

```
                                                                                         aga-mir-965-2
                  aga-mir-965-2-star
ccacacacaacggacugggcucaucaauugguuguggcuauguguuuaucaccaaauaaaccggacucguuuuccgcuauucacuaucauaucacagccauuuugaugagcucggucgggugcugaaa

.........................................................................................uaucacagccauuuugaugagcu................    30     0
.........................................................................................uaucacagccaCuuugaugagcu................    1      1
.........................................................................................uaucacagccaAuuugaugagcu................    1      1
.........................................................................................uaucacagccauuuugaugagcuc...............    171    0
.........................................................................................uaucacagccauuuugaugagcucU..............    2      1

...................cucaucaauugguuguggcu.........................................................................................    1      0
...................cucaucaauugguuguggcuaug......................................................................................    2      0
.........................................................................................uaucacagccauuuugaC.....................    6      1
.........................................................................................uaucacagccauuuugaCg....................    1      1
.........................................................................................uaucacagccauuuugaCgag..................    5      1
.........................................................................................uaucacagccauuuugaugagc.................    4      0
.........................................................................................uaucacagccauuuugaugagcu................    18     0
.........................................................................................uaucacagccauuuugaugagcuc...............    78     0

...................cucaucaauugguuguggcuaug......................................................................................    1      0
................................................ucaccaaauaaaccggacucguuuuc......................................................    2      0
.........................................................................................uaucacagccauuuugaC.....................    2      1
.........................................................................................uaucacagccauuuugaCg....................    2      1
.........................................................................................uaucacagccauuuugaug....................    2      0
.........................................................................................uaucacagccauuuugaCga...................    5      1
.........................................................................................uaucacagccauuuugaugag..................    1      0
.........................................................................................uaucacagccauuuugaCgag..................    21     1
.........................................................................................uaucacagccauuuugaugagc.................    23     0
.........................................................................................uaucacagccauuuugaugagcu................    10     0
.........................................................................................uaucacagccauuuugaugGgcu................    1      1
.........................................................................................uaucacagccauuuugaugagcC................    1      1
.........................................................................................uaucacagccauuuugaugagcuc...............    63     0
.........................................................................................uaucacagccauuuugaugagcucU..............    3      1
```

miRBase precursor : aga-mir-980
Total read count : 263
aga-mir-980 read count : 223
aga-mir-980-star read count: 40
remaining reads : 0

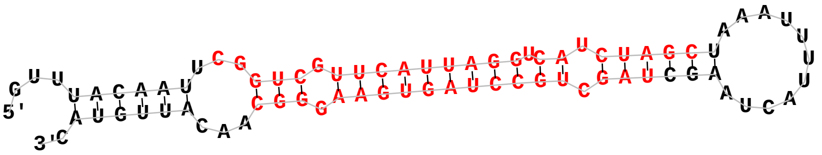

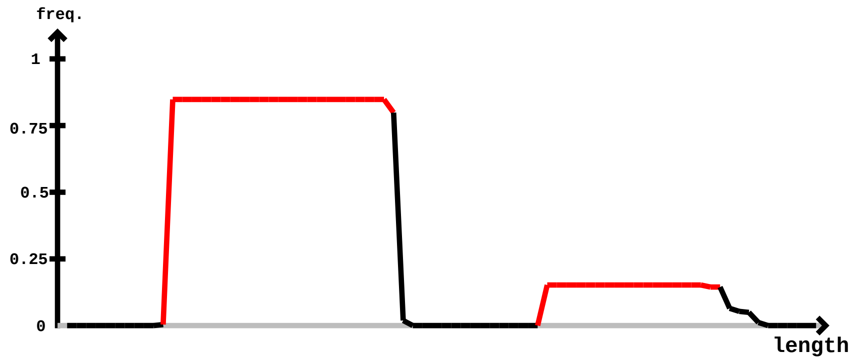


```
                                                 aga-mir-980-star
           aga-mir-980
5'- guuuacaauucggucguucauuaggucaucuagcuaaauuuuacuaagcuagcugccuagugaagggcaacauuguac   -3'    exp
    ...((((((...(((.(((((((((.((.((((((...........)))))).))))))))))).)))...)))))).   reads      mm         sample
    ..........cggucguucauuaggucaucuagc............................................   2          0          TE1
    .................................................uagcugccuagugaagg............   2          0          TE1
    .................................................uagcugccuagugaagggcaa........   1          0          TE1

    ..........cggucguucauuaggucaucuagA............................................   1          1          FF2
    ..........cggucguucauuaggucaucuagc............................................   10         0          FF2
    ..........cggucguucauuaggucaucuagcu...........................................   1          0          FF2
    .................................................uagcugccuagugaagggc..........   2          0          FF2

    ..........cggucguucauuaggucaucuag.............................................   2          0          OV2
    ..........cggucguucauuaggucaucuagc............................................   4          0          OV2
    .................................................uagcugccuagugaagggc..........   1          0          OV2

    ..........cggucguucauuaggucaucuag.............................................   3          0          MF2
    ..........cggucguucauuaggucaucuagc............................................   105        0          MF2
    ..........Uggucguucauuaggucaucuagc............................................   1          1          MF2
    ..........cggucguucauuaggucaucuagcu...........................................   2          0          MF2
    .................................................uagcugccuagugaagggc..........   1          0          MF2
    .................................................uagcugccuagugaagggca.........   1          0          MF2
    .................................................uagcugccuagugaagggcaac.......   2          0          MF2
    .................................................uagcugccuagugaagggcaacU......   1          1          MF2

    ..........cggucguucauuaggucaucuagc............................................   12         0          FW2
    ..........cggucguucauuaggucaucuagcu...........................................   1          0          FW2
    .................................................uagcugccuagugaagggc..........   2          0          FW2
    .................................................uagcugccuagugaagggcaac.......   5          0          FW2

    ..........cUgucguucauuaggucaucuagc............................................   1          1          FF1
    ..........cggucguucauuaggucaucuagc............................................   11         0          FF1
    ..........cggGcguucauuaggucaucuagc............................................   1          1          FF1
    .................................................uagcugccuagugaagggc..........   2          0          FF1
    .................................................uagcugccuagugaagggcaac.......   2          0          FF1
    .................................................uagcugccuagugaagggcaaca......   1          0          FF1

    ..........cggucguucauuaggucaucuag.............................................   2          0          OV1
```

```
                                                 aga-mir-980-star
          aga-mir-980
guuuacaauucggucguucauuaggucaucuagcuaaauuuuacuaagcuagcugccuagugaagggcaacauuguac

..........cggucguucauuaggucaucuagc............................................   8     0     OV1
.................................................uagcugccuagugaagggc..........   1     0     OV1

..........cggucguucauuaggucaucuag.............................................   1     0     MF1
..........cggucguucauuaggucaucuagc............................................   12    0     MF1
.................................................uagcugccuagugaagggc..........   1     0     MF1

..........cggucguucauuaggucaucuag.............................................   1     0     BF2
.................................................uagcugccuagugaagggca.........   1     0     BF2

..........cggucguucauuaggucaucuag.............................................   1     0     BF1
..........cggucguucauuaggucaucuagc............................................   5     0     BF1

..........cggucguucauuaggucaucuagc............................................   5     0     FW1
.................................................uagcugccuagugaagggc..........   2     0     FW1

.........ucggucguucauuaggucaucuag.............................................   1     0     MW1
..........cggucguucauuaggucaucuag.............................................   1     0     MW1
..........cggucguucauuaggucaucuagc............................................   14    0     MW1
..........cggucguucauuaggucaucuagcu...........................................   1     0     MW1
.................................................uagcugccuagugaagggc..........   4     0     MW1
.................................................uagcugccuagugaagggca.........   1     0     MW1
.................................................uagcugccuagugaagggcaac.......   1     0     MW1
.................................................uagcugccuagugaagggcaaca......   1     0     MW1

..........Gggucguucauuaggucaucuagc............................................   1     1     MW2
..........cggucguucauuaggucaucuagc............................................   3     0     MW2
.................................................uagcugccuagugaagggc..........   2     0     MW2

..........cggucguucauuaggucaucuaU.............................................   1     1     TE2
..........cggucguucauuaggucaucuagc............................................   9     0     TE2
.................................................uagcugccuagugaagggc..........   3     0     TE2
```

miRBase precursor : aga-mir-998
Total read count : 5490
aga-mir-998 read count : 5267
aga-mir-998-star read count: 103
remaining reads : 120

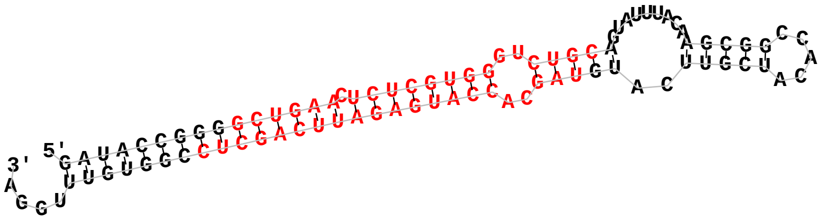

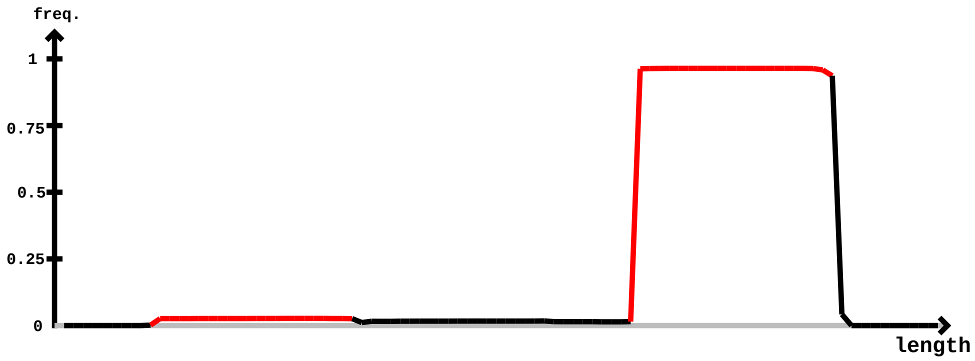

aga-mir-998-star

aga-mir-998

```
5'- gauaccggggcugaacucucgugggucugcaguauuuacaagcggccacaucguucauguagcaccaugagauucagcuccgguguuugga   -3'    exp
    ((((((((((((((.((((((((..(((((.........(((((.....)))))..)))))..)))))))))))))))))))))))....     reads      mm         sample
    .........gcugaacucucgugggucugca............................................................    1          0          TE1
    .........gcugaacucucgugggucugcaguauuuacaagcggccaca.........................................    3          0          TE1
    .........gcugaacucucgugggucugcaguauuuacaagcggccacaucguu....................................    1          0          TE1
    ...............................guauuuacaagcggccacaucguucaug................................    1          0          TE1
    ..................................uuuacaagcggccacaucguucaug................................    1          0          TE1
    ..................................................ucguucauguagcaccaugagauucagcuc...........    1          0          TE1
    ...........................................................uagcaccaugagauucag..............    1          0          TE1
    ...........................................................uagcaccaugagauucagc.............    2          0          TE1
    ...........................................................uagcaccaugagauucagcu............    10         0          TE1
    ...........................................................Cagcaccaugagauucagcuc...........    1          1          TE1
    ...........................................................uagcaccaugagauucagcuc...........    251        0          TE1
    ...........................................................uagcaccaugagauucagUuc...........    1          1          TE1
    ...........................................................uagcaccaugagauucagcCc...........    1          1          TE1
    ...........................................................uagcaccaugagauucagcucU..........    2          1          TE1
    ...........................................................uagcaccaugagauucagcucA..........    2          1          TE1

    .........gcugaacucucgugggucugc.............................................................    3          0          OV2
    .........gcugaacucucgugggucugca............................................................    2          0          OV2
    .........gcugaacucucgugggucugcaguauuuacaagcggccaca.........................................    1          0          OV2
    ...............................guauuuacaagcggccacaucguucaug................................    1          0          OV2
    ..................................................ucguucauguagcaccaugagauucagcuc...........    2          0          OV2
    ..........................................................Cuagcaccaugagauucagcuc...........    1          1          OV2
    ...........................................................uagcaccaugagauucagc.............    5          0          OV2
    ...........................................................uagcaccaugaAauucagcu............    1          1          OV2
    ...........................................................uagcaccaugagauucagcu............    26         0          OV2
    ...........................................................uagcaccaugagauucagcuc...........    925        0          OV2
    ...........................................................uagcaccaugaAauucagcuc...........    1          1          OV2
    ...........................................................uagcaccaugagauuUagcuc...........    1          1          OV2
    ...........................................................uagcaccaugCgauucagcuc...........    1          1          OV2
    ...........................................................uagcaccaugagauucaCcuc...........    1          1          OV2
    ...........................................................uagcaccaugagauucGgcuc...........    1          1          OV2
    ...........................................................uagcaccaugagauucagcucA..........    2          1          OV2
    ...........................................................uagcaccaugagauucagcucc..........    1          0          OV2
    ...........................................................uagcaccaugagauucagcucU..........    53         1          OV2
```

```
         aga-mir-998-star
                                                            aga-mir-998
gauaccggggcugaacucucgugggucugcaguauuuacaagcggccacaucguucauguagcaccaugagauucagcuccgguguuugga

............................................................agcaccaugagauucagcuc...........    1     0     OV2
.............................................................gcaccaugagauucagcuc...........    1     0     OV2

.........gcugaacucucgugggucugc.............................................................    1     0     FF2
.........gcugaacucucgugggucugca............................................................    1     0     FF2
.........gcugaacucucgugggucugcaguauuuacaagcggccaca.........................................    1     0     FF2
...............................guauuuacaagcggccacaucguucaug................................    2     0     FF2
...........................................................uagcaccaugagauucagc.............    2     0     FF2
...........................................................uagcaccaugagauucagcu............    2     0     FF2
...........................................................uagcaccaugagauucagcuc...........    71    0     FF2
...........................................................uagcaccaugagauucagcucU..........    4     1     FF2

........ggcugaacucucguggguc................................................................    1     0     MF2
........ggcugaacucucgugggucugc.............................................................    1     0     MF2
.........gcugaacucucgugggucug..............................................................    1     0     MF2
.........gcugaacucucgugggucugc.............................................................    11    0     MF2
.........gcugaacucucgugggucugca............................................................    1     0     MF2
.........gcugaacucucgugggucugcaguauuuacaagcggccaca.........................................    14    0     MF2
...................cgugggucugcaguauuuacaagc................................................    1     0     MF2
...............................guauuuacaagcggccacaucguucaug................................    6     0     MF2
.................................................aucguucauguagcaccaugagauucagcuc...........    2     0     MF2
..................................................ucguucauguagcaccaugagauucagcuc...........    4     0     MF2
...........................................................uagcaccaugagauucag..............    1     0     MF2
...........................................................uagcaccaugaCauucagc.............    1     1     MF2
...........................................................uagcaccaugagauucagcu............    3     0     MF2
...........................................................uagcaccaugagauucagGuc...........    1     1     MF2
...........................................................uagcaccaugagauucagcuc...........    346   0     MF2
...........................................................uagcaccaugagauucagcucG..........    1     1     MF2
...........................................................uagcaccaugagauucagcucU..........    21    1     MF2
...........................................................uagcaccaugagauucagcucc..........    1     0     MF2
................................................................ccaugagauucagcucU..........    1     1     MF2

........ggcugaacucucgugggucugc.............................................................    3     0     FW2
.........gcugaacucucgugggucugc.............................................................    3     0     FW2
...............................guauuuacaagcggccacaucguucaug................................    2     0     FW2
................................................caucguucauguagcaccaugagauucagcuc...........    1     0     FW2
..................................................ucguucauguagcaccaugagauucagcuc...........    1     0     FW2
...........................................................uagcaccaugagauucagc.............    2     0     FW2
...........................................................uagcaccaugagauucagcu............    5     0     FW2
...........................................................uagcaUcaugagauucagcuc...........    1     1     FW2
...........................................................uagcaccaugagauCcagcuc...........    1     1     FW2
...........................................................uagcaccaugagauucagcuc...........    253   0     FW2
...........................................................Aagcaccaugagauucagcuc...........    1     1     FW2
...........................................................uagcaccaugagauucaUcuc...........    1     1     FW2
...........................................................uagcaccaugaAauucagcuc...........    1     1     FW2
...........................................................uagcaccaugagauucagcucc..........    1     0     FW2
...........................................................uagcaccaugagauucagcucA..........    1     1     FW2
...........................................................uagcaccaugagauucagcucU..........    11    1     FW2

.........gcugaacucucguggguGugc.............................................................    1     1     FF1
.........gcugaacucucgugggucugc.............................................................    4     0     FF1
.........gcuUaacucucgugggucugc.............................................................    1     1     FF1
.........gcugaacucucgugggucugca............................................................    1     0     FF1
.........gcugaacucucgugggucugcaU...........................................................    1     1     FF1
.........gcugaacucucgugggucugcaguauuuacaagcggccaca.........................................    3     0     FF1
................................................caucguucauguagcaccaugagauucagcuc...........    1     0     FF1
...........................................................uagcaGcaugagauucagcuc...........    1     1     FF1
...........................................................uagGaccaugagauucagcuc...........    4     1     FF1
...........................................................uagcaccaugagauucaUcuc...........    2     1     FF1
...........................................................uUgcaccaugagauucagcuc...........    1     1     FF1
...........................................................uagcaccaugagauucagcuc...........    145   0     FF1
...........................................................uagcaccaugagauucagcuA...........    1     1     FF1
...........................................................Gagcaccaugagauucagcuc...........    2     1     FF1
...........................................................uagcaccaugagauucagcucc..........    1     0     FF1
...........................................................uagcaccaugagauucagcucU..........    7     1     FF1

.........gcugaacucucgugggucug..............................................................    1     0     OV1
.........gcugaacucucgugggucugc.............................................................    7     0     OV1
.........gcugaacucucgugggucugcaguauuuacaagcggcca...........................................    1     0     OV1
```

```
         aga-mir-998-star
                                                  aga-mir-998
gauaccggggcugaacucucgugggucugcaguauuuacaagcggccacaucguucauguagcaccaugagauucagcuccgguguuugga

...............................guauuuacaagcggccacaucguucaug................................    3     0     OV1
..................................uuuacaagcggccacaucguucaug................................    1     0     OV1
..................................................ucguucauguagcaccaugagauucagcuc...........    2     0     OV1
..........................................................Cuagcaccaugagauucagcuc...........    2     1     OV1
...........................................................uagcaccaugagauucagc.............    3     0     OV1
...........................................................uagcaccaugagauucagcA............    1     1     OV1
...........................................................uagcaccaugagauucagcu............    19    0     OV1
...........................................................uagcaccaugagauucagcuc...........    851   0     OV1
...........................................................uagcaccaugagaCucagcuc...........    1     1     OV1
...........................................................uaUcaccaugagauucagcuc...........    1     1     OV1
...........................................................uagcaccaugagauucagcuA...........    1     1     OV1
...........................................................uagcaccaugagauucagcuU...........    1     1     OV1
...........................................................uagcaUcaugagauucagcuc...........    1     1     OV1
...........................................................uagcaccaugaAauucagcuc...........    1     1     OV1
...........................................................uagcaccaugagauucagcCc...........    1     1     OV1
...........................................................uagcaccauAagauucagcuc...........    1     1     OV1
...........................................................uagcaccaugUgauucagcuc...........    1     1     OV1
...........................................................uagcaccaugagUuucagcuc...........    1     1     OV1
...........................................................uagcacUaugagauucagcuc...........    1     1     OV1
...........................................................uagUaccaugagauucagcuc...........    1     1     OV1
...........................................................uagcaccaugagauucagcucc..........    2     0     OV1
...........................................................uagcaccaugagauucagcucU..........    47    1     OV1
............................................................agcaccaugagauucagcuc...........    2     0     OV1

.........gcugaacucucgugggucugc.............................................................    6     0     MF1
.........gcugaacucucgugggucugca............................................................    1     0     MF1
...........................................................uagcaccaugagauucagcu............    1     0     MF1
...........................................................uagcaccaugagauucagcuc...........    117   0     MF1
...........................................................uagcaccaugaCauucagcuc...........    1     1     MF1
...........................................................uagcaccaugagauucagcucU..........    4     1     MF1
............................................................agcaccaugagauucagcuc...........    1     0     MF1

........ggcugaacucucgugggucugc.............................................................    2     0     BF2
.........gcugaacucucgugggucugc.............................................................    20    0     BF2
.........gcugaacucucgugggucugca............................................................    5     0     BF2
.........gcugaacucucgugggucugcaguauuuacaagcggccac..........................................    1     0     BF2
.........gcugaacucucgugggucugcaguauuuacaagcggccaca.........................................    5     0     BF2
.........gcugaacucucgugggucugcaguauuuacaagcggccacau........................................    1     0     BF2
.........gcugaacucucgugggucugcaguauuuacaagcggccacaucguu....................................    1     0     BF2
...............................guauuuacaagcggccacaucguucaug................................    13    0     BF2
..................................uuuacaagcggccacaucguucaug................................    1     0     BF2
........................................agcggccacaucguucaug................................    1     0     BF2
..................................................ucguucauguagcaccaugagauucagcuc...........    3     0     BF2
...........................................................uagcaccaugagauucagc.............    4     0     BF2
...........................................................uagcaccaugagauucagcu............    7     0     BF2
...........................................................uagcaccaugagaCucagcuc...........    3     1     BF2
...........................................................uagcaccaugagauucagcuc...........    381   0     BF2
...........................................................uagcacGaugagauucagcuc...........    1     1     BF2
...........................................................uagcaccaugagauuAagcuc...........    1     1     BF2
...........................................................uagcaccaAgagauucagcuc...........    1     1     BF2
...........................................................uagcaccaugagauucagcucU..........    15    1     BF2
...........................................................uagcaccaugagauucagcucc..........    1     0     BF2
...........................................................uagcaccaugagauucagcucA..........    1     1     BF2

........ggcugaacucucgugggucugc.............................................................    1     0     BF1
.........gcugaacucucgugggucugc.............................................................    3     0     BF1
.........gcugaacucucgugggucugca............................................................    1     0     BF1
.........gcugaacucucgugggucugcaguauuuacaagcggccaca.........................................    6     0     BF1
.........gcugaacucucgugggGcugcaguauuuacaagcggccaca.........................................    1     1     BF1
.........gcugaacucucgugggucugcaguauuuacaagcggccacaucguu....................................    1     0     BF1
...............................guauuuacaagcggccacaucguucaug................................    12    0     BF1
....................................uacaagcggccacaucguucaug................................    1     0     BF1
.................................................aucguucauguagcaccaugagauucagcucc..........    1     0     BF1
..................................................ucguucauguagcaccaugagauucagcuc...........    6     0     BF1
..........................................................Cuagcaccaugagauucagcuc...........    1     1     BF1
...........................................................uagcaccaugagauucag..............    1     0     BF1
...........................................................uagcaccaugagauucagcu............    4     0     BF1
...........................................................uagcaccaGgagauucagcuc...........    1     1     BF1
...........................................................uagcaccaugagaCucagcuc...........    1     1     BF1
```

```
         aga-mir-998-star
                                                           aga-mir-998
gauaccggggcugaacucucgugggucugcaguauuuacaagcggccacaucguucauguagcaccaugagauucagcuccgguguuugga

...........................................................Aagcaccaugagauucagcuc...........    1     1     BF1
...........................................................uagcaccaugagauucagcuc...........    399   0     BF1
...........................................................uagcaccaugagauuUagcuc...........    1     1     BF1
...........................................................Cagcaccaugagauucagcuc...........    1     1     BF1
...........................................................uagcaccaCgagauucagcuc...........    1     1     BF1
...........................................................uagcaccaugagauucagcucU..........    13    1     BF1
...........................................................uagcaccaugagauucagcucc..........    1     0     BF1

.........gcugaacucucgugggucugc.............................................................    1     0     FW1
......................gggucugcaguauuuacaagcggccacaucguucauguagca...........................    1     0     FW1
...........................................................uagcaccaugagauucagc.............    1     0     FW1
...........................................................uagcaccaugagauucagcu............    1     0     FW1
...........................................................uagcaccaugagauucagcuc...........    89    0     FW1
...........................................................uagcaccaugagauucagcucU..........    7     1     FW1

.........gcugaacucucgugggucugc.............................................................    6     0     MW1
.........gcugaacucucgugggucugca............................................................    2     0     MW1
.........gcugaacucucgugggucugcaguauuuacaagcggccaca.........................................    2     0     MW1
.............aacucucgugggucugca............................................................    1     0     MW1
...............................guauuuacaagcggccacaucguucaug................................    3     0     MW1
...........................................................uagcaccaugagauucagcu............    1     0     MW1
...........................................................uagcaccaugagauucagcuc...........    136   0     MW1
...........................................................uagcaccaugagauucagcucU..........    7     1     MW1
............................................................agcaccaugagauucagcuc...........    1     0     MW1
.............................................................gcaccaugagauucagcuc...........    1     0     MW1
..............................................................caccaugagauucagcuc...........    1     0     MW1

........ggcugCacucucgugggucugc.............................................................    1     1     MW2
.........gcugaacucucgugggucugc.............................................................    2     0     MW2
.........gcugaacucucgugggucugca............................................................    1     0     MW2
...........................................................uagcaccaugagauucagcuc...........    131   0     MW2
...........................................................uagcaccaugagauucagcucU..........    4     1     MW2

.........gcugaacucucguggguc................................................................    1     0     TE2
.........gcugaacucucgugggucug..............................................................    1     0     TE2
.........gcugaacucucgugggucugc.............................................................    3     0     TE2
..........................................cggccacaucguucaug................................    1     0     TE2
..................................................ucguucauguagcaccaugagauucagcuc...........    2     0     TE2
..................................................ucguucauguagcaUcaugagauucagcuc...........    1     1     TE2
...........................................................uagcaccaugagauucagA.............    1     1     TE2
...........................................................uagcaccaugagauucagc.............    8     0     TE2
...........................................................uagcaccaugagauucagcu............    39    0     TE2
...........................................................uagcaccaugagauucagcuA...........    1     1     TE2
...........................................................uagcacUaugagauucagcuc...........    1     1     TE2
...........................................................uagcaccaugaUauucagcuc...........    1     1     TE2
...........................................................uaUcaccaugagauucagcuc...........    1     1     TE2
...........................................................uagcaccaugGgauucagcuc...........    1     1     TE2
...........................................................uagcaccauAagauucagcuc...........    1     1     TE2
...........................................................uagcaUcaugagauucagcuc...........    2     1     TE2
...........................................................uagcaccaugagauucagcuU...........    1     1     TE2
...........................................................uagcaccaugagauucagcuc...........    719   0     TE2
...........................................................uagcaccaugagauucagcucU..........    18    1     TE2
...........................................................uagcaccaugagauucagcucA..........    1     1     TE2
```

miRBase precursor : aga-mir-999
Total read count : 23840
aga-mir-999 read count : 23831
aga-mir-999-star read count: 6
remaining reads : 3

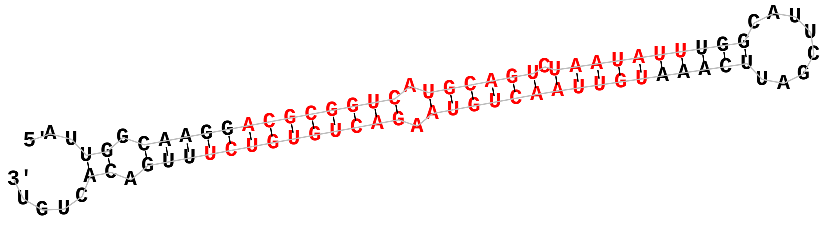

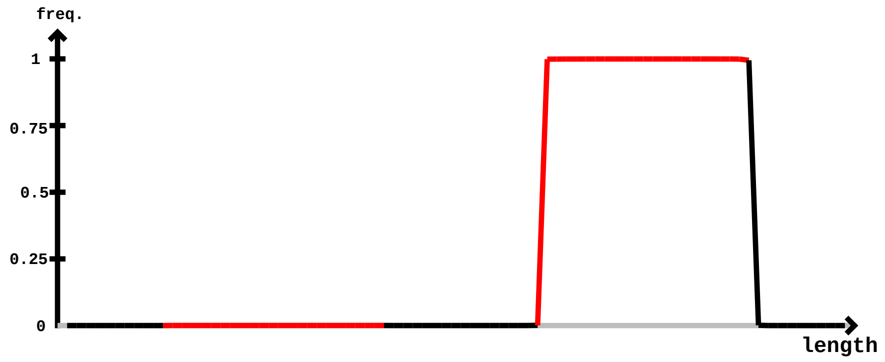


```
                                                 aga-mir-999
           aga-mir-999-star
5'- auuggcaaggacgcggucaugcagucuaauauuuggcauucgauucaaauguuaacuguaagacugugucuuugacacugu   -3'    exp
    ..((.(((((((((((((.((((((.((((((((((........)))))))))))))))).))))))))))))).))....    reads      mm         sample
    .................................................uguuaacuguaagacuguguc...........    9          0          OV2
    .................................................uguuaacuguaagacuAugucu..........    2          1          OV2
    .................................................uguuaacuguaagacugAgucu..........    1          1          OV2
    .................................................uguuaacCguaagacugugucu..........    1          1          OV2
    .................................................uguCaacuguaagacugugucu..........    1          1          OV2
    .................................................ugCuaacuguaagacugugucu..........    2          1          OV2
    .................................................uguuaacuguaagacugugucG..........    1          1          OV2
    .................................................uguuaacuguGagacugugucu..........    1          1          OV2
    .................................................uguuaacuguaagacugugucu..........    1889       0          OV2
    .................................................uguuaacuguaagacuguguUu..........    2          1          OV2
    .................................................uguuaacugCaagacugugucu..........    1          1          OV2
    .................................................uguuaacuguaUgacugugucu..........    1          1          OV2
    .................................................uguuaacuguaagacugugucC..........    3          1          OV2
    .................................................uguuaacuguaagacuguAucu..........    1          1          OV2
    .................................................uAuuaacuguaagacugugucu..........    3          1          OV2
    .................................................uguuaacuguaagacugugucuu.........    1          0          OV2
    ..................................................guuaacuguaagacugugucu..........    1          0          OV2
    ...................................................uuaacuguaagacugugucu..........    1          0          OV2

    .................................................uguuaacuguaagacugugu............    2          0          TE1
    .................................................uguuaacuguaagacuguguc...........    5          0          TE1
    .................................................uguuaacuguaagacuCugucu..........    1          1          TE1
    .................................................uguuaacuguaagacugugucG..........    1          1          TE1
    .................................................uguuaacuguaagacuUugucu..........    1          1          TE1
    .................................................uguuaacuguaagacugugucu..........    641        0          TE1
    .................................................Aguuaacuguaagacugugucu..........    1          1          TE1
    .................................................uguuaacuguaagacuguguAu..........    1          1          TE1
    .................................................uguuaacuguaagGcugugucu..........    2          1          TE1
    .................................................uguuaacuguaagacugugucuu.........    1          0          TE1

    ................................................Cuguuaacuguaagacugugucu..........    1          1          FF2
    .................................................uguuaacuguaagacuguguc...........    5          0          FF2
    .................................................uguuaacuguaagacuAugucu..........    1          1          FF2
    .................................................uguuaacuguaagacugGgucu..........    1          1          FF2
```

```
                                                 aga-mir-999
          aga-mir-999-star
auuggcaaggacgcggucaugcagucuaauauuuggcauucgauucaaauguuaacuguaagacugugucuuugacacugu

.................................................uguuaacuguaagacugugucC..........    1      1      FF2
.................................................uguuaacuguaagacugugAcu..........    1      1      FF2
.................................................Cguuaacuguaagacugugucu..........    2      1      FF2
.................................................uguuaacuguaagacugugucG..........    1      1      FF2
.................................................uguuaacuguaagacugugucu..........    737    0      FF2
.................................................uguuaacuguaagacuCugucu..........    2      1      FF2
.................................................uguuaacuguaagacuguguUu..........    1      1      FF2
.................................................uguuaacuguaagacugugucA..........    1      1      FF2
.................................................uguuaacuguaagaUugugucu..........    2      1      FF2
.................................................uguuaacAguaagacugugucu..........    1      1      FF2
.................................................uAuuaacuguaagacugugucu..........    1      1      FF2
..................................................guuaacuguaagacugugucu..........    1      0      FF2

..........acgcggucaugcagucuaauau.................................................    1      0      MF2
.............................................caaauguuaacuguaagacugugucu..........    1      0      MF2
................................................Cuguuaacuguaagacugugucu..........    7      1      MF2
.................................................uguuaacuguaagacuguguc...........    20     0      MF2
.................................................uguuaacuguaagacuguAuc...........    1      1      MF2
.................................................uguuaaAuguaagacugugucu..........    1      1      MF2
.................................................uguuaacuguaUgacugugucu..........    1      1      MF2
.................................................uguuaacuguaagacugugAcu..........    1      1      MF2
.................................................Cguuaacuguaagacugugucu..........    3      1      MF2
.................................................uguuaacuguaagacuAugucu..........    2      1      MF2
.................................................uguuaacuguGagacugugucu..........    2      1      MF2
.................................................uguuaacuguaCgacugugucu..........    3      1      MF2
.................................................uguuaacuguaagacugugucA..........    3      1      MF2
.................................................uguuaacuguaagacugugucC..........    6      1      MF2
.................................................uguuaacugCaagacugugucu..........    3      1      MF2
.................................................uguuaacuguaagaAugugucu..........    1      1      MF2
.................................................uguuGacuguaagacugugucu..........    3      1      MF2
.................................................uguuaacuguaagacugugucu..........    5505   0      MF2
.................................................uguuaacGguaagacugugucu..........    1      1      MF2
.................................................uAuuaacuguaagacugugucu..........    3      1      MF2
.................................................ugCuaacuguaagacugugucu..........    7      1      MF2
.................................................uguuaacuguaagacugGgucu..........    2      1      MF2
.................................................uguuaacuguaagacuCugucu..........    2      1      MF2
.................................................uguuaacuguaaAacugugucu..........    1      1      MF2
.................................................uguuaacuguaagacuguguAu..........    2      1      MF2
.................................................uguuaacuguaGgacugugucu..........    2      1      MF2
.................................................uguuaacCguaagacugugucu..........    2      1      MF2
.................................................uguuaacuguaagacuguAucu..........    1      1      MF2
.................................................uguuaacuCuaagacugugucu..........    1      1      MF2
.................................................uguuaacuguaagaGugugucu..........    1      1      MF2
.................................................uguuaacuguaagacugAgucu..........    1      1      MF2
.................................................uguuaUcuguaagacugugucu..........    2      1      MF2
.................................................ugAuaacuguaagacugugucu..........    1      1      MF2
.................................................uguuaacuguaagacugugCcu..........    1      1      MF2
.................................................uguuaacugAaagacugugucu..........    1      1      MF2
.................................................uguGaacuguaagacugugucu..........    1      1      MF2
.................................................Aguuaacuguaagacugugucu..........    1      1      MF2
.................................................uguCaacuguaagacugugucu..........    2      1      MF2
.................................................Gguuaacuguaagacugugucu..........    1      1      MF2
.................................................uguuaacuguaagacCgugucu..........    1      1      MF2
.................................................uguuaacuguaagacugCgucu..........    7      1      MF2
.................................................uguuaacuguaagacugugucG..........    11     1      MF2
.................................................uguuaacuguUagacugugucu..........    1      1      MF2
.................................................uguuaacuguaagacugugucuu.........    2      0      MF2
.................................................uguuaacuguaagacugugucuA.........    2      1      MF2
.................................................uguuaacuguaagacugugucuuu........    1      0      MF2
..................................................guuaacuguaagacugugucu..........    2      0      MF2
...................................................uuaacuguaagacugugucu..........    2      0      MF2

................................................auguuGacuguaagacugugucu..........    1      1      FW2
.................................................uguuaacuguaagacuguguc...........    5      0      FW2
.................................................uguuaacuguaagacuUugucu..........    1      1      FW2
.................................................uguuaacugCaagacugugucu..........    1      1      FW2
.................................................uguuaacuguCagacugugucu..........    1      1      FW2
.................................................uguuaacuguaagacugugucu..........    996    0      FW2
.................................................uguuaacuguaGgacugugucu..........    1      1      FW2
```

```
                                                 aga-mir-999
          aga-mir-999-star
auuggcaaggacgcggucaugcagucuaauauuuggcauucgauucaaauguuaacuguaagacugugucuuugacacugu

.................................................uguuaacuguaagacCgugucu..........    1          1          FW2
.................................................uguuaacuguaagacugCgucu..........    1          1          FW2
.................................................uguuaacuguaagacugugucuu.........    2          0          FW2
...................................................uuaacuguaagacugugucu..........    1          0          FW2
....................................................uaacuguaagacugugucu..........    1          0          FW2

................................................Cuguuaacuguaagacugugucu..........    1          1          FF1
.................................................uguuaacuguaagacugugu............    2          0          FF1
.................................................uguuaacuguaagacuguguc...........    21         0          FF1
.................................................uguuaacuguaagacuguCucu..........    2          1          FF1
.................................................uguuaGcuguaagacugugucu..........    1          1          FF1
.................................................uguGaacuguaagacugugucu..........    78         1          FF1
.................................................uguuaacuguaagacugugucA..........    1          1          FF1
.................................................uAuuaacuguaagacugugucu..........    7          1          FF1
.................................................uguuaacugAaagacugugucu..........    1          1          FF1
.................................................uguuaacuguaagacuguAucu..........    4          1          FF1
.................................................uguuaacuCuaagacugugucu..........    6          1          FF1
.................................................uguuaacuguaagacugugucG..........    13         1          FF1
.................................................uguuaacuguaagacugugucC..........    3          1          FF1
.................................................uguuaacuguaagacuguguUu..........    1          1          FF1
.................................................uguAaacuguaagacugugucu..........    1          1          FF1
.................................................Aguuaacuguaagacugugucu..........    2          1          FF1
.................................................uguuaacuguaagacugCgucu..........    1          1          FF1
.................................................ugCuaacuguaagacugugucu..........    5          1          FF1
.................................................uguCaacuguaagacugugucu..........    2          1          FF1
.................................................uCuuaacuguaagacugugucu..........    2          1          FF1
.................................................uguuaacuguaGgacugugucu..........    3          1          FF1
.................................................uguuaacuguaagacugugucu..........    3467       0          FF1
.................................................uguuaCcuguaagacugugucu..........    1          1          FF1
.................................................uguUacuguaagacugugucu...........    1          1          FF1
.................................................Gguuaacuguaagacugugucu..........    17         1          FF1
.................................................uguuaacuguaagacuguUucu..........    1          1          FF1
.................................................uguuaacuguaagaGugugucu..........    1          1          FF1
.................................................uguuaacuguaagacugGgucu..........    26         1          FF1
.................................................uguuaacuguaaCacugugucu..........    1          1          FF1
.................................................uguuaacuUuaagacugugucu..........    1          1          FF1
.................................................uguuaacuguaagacuguguAu..........    1          1          FF1
.................................................uUuuaacuguaagacugugucu..........    4          1          FF1
.................................................uguuaacuguGagacugugucu..........    1          1          FF1
.................................................uguuGacuguaagacugugucu..........    26         1          FF1
.................................................uguuaacuguaaAacugugucu..........    1          1          FF1
.................................................uguuaacuguaagacugAgucu..........    1          1          FF1
.................................................uguuaacuguaCgacugugucu..........    1          1          FF1
.................................................uguuaaAuguaagacugugucu..........    1          1          FF1
.................................................uguuaacCguaagacugugucu..........    2          1          FF1
.................................................uguuaacuAuaagacugugucu..........    5          1          FF1
.................................................uguuaacuguaagacugugucuu.........    5          0          FF1

...............................................aauguuaacuguaagacugugucu..........    1          0          OV1
................................................Cuguuaacuguaagacugugucu..........    1          1          OV1
.................................................uAuuaacuguaagacuguguc...........    1          1          OV1
.................................................uguuaacuguaagacuguguc...........    13         0          OV1
.................................................uguuaacuguaagacugugucA..........    2          1          OV1
.................................................uguuaacuguaagacCgugucu..........    2          1          OV1
.................................................uguuaacuguaagacugugucu..........    2601       0          OV1
.................................................uguUacuguaagacugugucu...........    1          1          OV1
.................................................uguuaacuguaagacuAugucu..........    1          1          OV1
.................................................uguuaacuAuaagacugugucu..........    4          1          OV1
.................................................uguuaacuguaagacuCugucu..........    1          1          OV1
.................................................uguuaacuguGagacugugucu..........    2          1          OV1
.................................................uguuaacuguaagacugugCcu..........    1          1          OV1
.................................................uguuaacuguaUgacugugucu..........    1          1          OV1
.................................................uguuaacugCaagacugugucu..........    1          1          OV1
.................................................ugCuaacuguaagacugugucu..........    1          1          OV1
.................................................uguuaaAuguaagacugugucu..........    1          1          OV1
.................................................uguuaacAguaagacugugucu..........    1          1          OV1
.................................................uguuaacuguaagacuguAucu..........    2          1          OV1
.................................................uguuaacCguaagacugugucu..........    1          1          OV1
.................................................uguCaacuguaagacugugucu..........    1          1          OV1
```

```
                                                 aga-mir-999
          aga-mir-999-star
auuggcaaggacgcggucaugcagucuaauauuuggcauucgauucaaauguuaacuguaagacugugucuuugacacugu

.................................................uguuaacuguaagCcugugucu..........    1       1       OV1
.................................................uguuaacuguaagacugugucG..........    7       1       OV1
.................................................uguuaaUuguaagacugugucu..........    1       1       OV1
.................................................uguuaacuguaagacugugucC..........    2       1       OV1
.................................................uguuaacuguaGgacugugucu..........    1       1       OV1
.................................................uguuaacuguaagacugugucuu.........    1       0       OV1
..................................................guuaacuguaagacugugucu..........    2       0       OV1

................................................Cuguuaacuguaagacugugucu..........    2       1       MF1
.................................................uguuaacuguaagacugugG............    1       1       MF1
.................................................uguuaacuguaagacuguguc...........    7       0       MF1
.................................................uguuaacuguaagacuguguU...........    1       1       MF1
.................................................ugCuaacuguaagacugugucu..........    1       1       MF1
.................................................uAuuaacuguaagacugugucu..........    1       1       MF1
.................................................uguuGacuguaagacugugucu..........    1       1       MF1
.................................................uguCaacuguaagacugugucu..........    1       1       MF1
.................................................uguuaacuguaagacugCgucu..........    1       1       MF1
.................................................uguuaacugCaagacugugucu..........    2       1       MF1
.................................................uguuaacuguaCgacugugucu..........    1       1       MF1
.................................................uguuaacuguaagacugugucu..........    1572    0       MF1
.................................................uguuaacuguaagacuAugucu..........    2       1       MF1
.................................................uguuaacuguaagacuguAucu..........    1       1       MF1
.................................................uguuaacuguCagacugugucu..........    1       1       MF1
.................................................uguuaacuguaagacugugCcu..........    1       1       MF1
.................................................uUuuaacuguaagacugugucu..........    1       1       MF1
.................................................uguuaacuguaagacugugucG..........    1       1       MF1
.................................................uguuaUcuguaagacugugucu..........    1       1       MF1
.................................................uguuaacuguaaAacugugucu..........    1       1       MF1
.................................................uguuaacuguaGgacugugucu..........    1       1       MF1
.................................................uguuaacuguaagacugugucuu.........    1       0       MF1
...................................................uuaacuguaagacugugucu..........    1       0       MF1

..........acgcggucaugcagucuaauau.................................................    1       0       BF2
..........acgcggucaugcagucuaauauu................................................    1       0       BF2
.................................................uguuaacuguaagacuguguc...........    5       0       BF2
.................................................uguuaacuguaagacugCgucu..........    1       1       BF2
.................................................uguuaacuguaagacuAugucu..........    2       1       BF2
.................................................uguuaacuguaagacugugucu..........    1041    0       BF2
.................................................uguuaacuguaagacuUugucu..........    1       1       BF2
.................................................uguuaacCguaagacugugucu..........    1       1       BF2
.................................................uguuaacuguaagacugugAcu..........    1       1       BF2
.................................................uguuaacuguGagacugugucu..........    1       1       BF2
.................................................uguuaacuguaaAacugugucu..........    1       1       BF2
.................................................uguuaacuguaagacCgugucu..........    1       1       BF2
.................................................uguuaacuguaagacugugucC..........    1       1       BF2
.................................................uguuCacuguaagacugugucu..........    1       1       BF2
.................................................uguuaacuguaagacugugucuA.........    1       1       BF2
.................................................uguuaacuguaagacugugucuuu........    1       0       BF2
..................................................guuaacuguaagacugugucu..........    1       0       BF2

.................................................ugCuaacuguaagacugugucu..........    1       1       BF1
.................................................uguuaacuguaagacuUugucu..........    1       1       BF1
.................................................uguuaacuguaGgacugugucu..........    1       1       BF1
.................................................uguuaacugCaagacugugucu..........    2       1       BF1
.................................................uguuaacuguaagacugugucu..........    681     0       BF1
.................................................uguuaacuguaagacugugCcu..........    1       1       BF1
.................................................uguuaacuguaagacugugucuu.........    1       0       BF1

..........acgcggucaugcagucuaauauu................................................    1       0       FW1
...............................................aauguuaacuguaagacugugucu..........    1       0       FW1
................................................Cuguuaacuguaagacugugucu..........    1       1       FW1
.................................................uguuaacuguaagacuguguc...........    3       0       FW1
.................................................uguuaacuguaagacugugucu..........    524     0       FW1
.................................................uguuaacCguaagacugugucu..........    1       1       FW1
.................................................uguuaacuguaagacugugCcu..........    1       1       FW1
.................................................Gguuaacuguaagacugugucu..........    1       1       FW1
.................................................uguuaacuguaagacugugucC..........    2       1       FW1
.................................................uguuaacuguaagacugugucG..........    2       1       FW1
.................................................uguuaacuguaagacugugucuu.........    1       0       FW1
```

```
                                                  aga-mir-999
          aga-mir-999-star
auuggcaaggacgcggucaugcagucuaauauuuggcauucgauucaaauguuaacuguaagacugugucuuugacacugu

.................................................uguuaacuguaagacugugucuuA........   1     1    FW1
...................................................uuaacuguaagacugugucu..........   1     0    FW1
.....................................................aacuguaagacugugucu..........   2     0    FW1

..........acgcggucaugcagucuaauauu................................................   2     0    MW1
..........acgcggucaugcagucuaauauuuggcauucgauuc...................................   1     0    MW1
..........acgcggucaugcagucuaauauuuggcauucgauucaaau...............................   1     0    MW1
...............................................aauguuaacuguaagacugugucu..........   1     0    MW1
.................................................uguuaacuguaagacugug.............   1     0    MW1
.................................................uguuaacuguaagacugugu............   1     0    MW1
.................................................uguuaacuguaagacuguguc...........   2     0    MW1
.................................................uguuaacuguaagacugugucu..........   979   0    MW1
.................................................uguuaacuguaagacugugCcu..........   1     1    MW1
.................................................Cguuaacuguaagacugugucu..........   1     1    MW1
.................................................uAuuaacuguaagacugugucu..........   1     1    MW1
.................................................uguuaacCguaagacugugucu..........   1     1    MW1
.................................................uguuaacuguaagacuUugucu..........   1     1    MW1
.................................................uguuaacuguaGgacugugucu..........   1     1    MW1
.................................................Gguuaacuguaagacugugucu..........   1     1    MW1
.................................................uguuaacuguGagacugugucu..........   1     1    MW1
.................................................uguuaacuguaagacugCgucu..........   1     1    MW1
.................................................uguuaacuguaagacugugucG..........   2     1    MW1
.................................................uguCaacuguaagacugugucu..........   2     1    MW1
.................................................uguuaacuguaagGcugugucu..........   1     1    MW1
.................................................uguuaacuguaagacugugucuu.........   1     0    MW1
...................................................uuaacuguaagacugugucu..........   1     0    MW1
......................................................acuguaagacugugucu..........   1     0    MW1

.................................................uguuaacuguaagacuguguc...........   1     0    MW2
.................................................uguuaGcuguaagacugugucu..........   1     1    MW2
.................................................uguuaacGguaagacugugucu..........   1     1    MW2
.................................................uguuaacuguaGgacugugucu..........   1     1    MW2
.................................................uguuaacuguaagacugugucA..........   1     1    MW2
.................................................uguuaacCguaagacugugucu..........   2     1    MW2
.................................................uguuaacuguaagacugugucu..........   302   0    MW2
.................................................uguCaacuguaagacugugucu..........   1     1    MW2

................................................Cuguuaacuguaagacugugucu..........   1     1    TE2
.................................................uguuaacuguaagacugu..............   1     0    TE2
.................................................uguuaacuguaagacuguguc...........   12    0    TE2
.................................................uguuaacuguaagacugugCcu..........   3     1    TE2
.................................................uguuaacuguaagacuguguUu..........   2     1    TE2
.................................................uguuaacuguaGgacugugucu..........   1     1    TE2
.................................................uguuaacuguaagacuguUucu..........   1     1    TE2
.................................................ugCuaacuguaagacugugucu..........   1     1    TE2
.................................................Cguuaacuguaagacugugucu..........   3     1    TE2
.................................................uguuaacuguaaAacugugucu..........   1     1    TE2
.................................................ugGuaacuguaagacugugucu..........   2     1    TE2
.................................................uguuaacuguaagacCgugucu..........   1     1    TE2
.................................................uguuaacuguaagacugugucA..........   1     1    TE2
.................................................uguuaacuguaagacugugucC..........   3     1    TE2
.................................................uguuaacuguaagacugugucG..........   6     1    TE2
.................................................uguuaacuguCagacugugucu..........   1     1    TE2
.................................................uguuaaUuguaagacugugucu..........   1     1    TE2
.................................................uguuaacuguaaCacugugucu..........   1     1    TE2
.................................................uguuaacuguaaUacugugucu..........   2     1    TE2
.................................................uguuaacuguaagacugugGcu..........   1     1    TE2
.................................................uguuaacugCaagacugugucu..........   2     1    TE2
.................................................uguuaacuguaagacugugucu..........   2224  0    TE2
.................................................Gguuaacuguaagacugugucu..........   1     1    TE2
.................................................uguuaacuguaagacugugucuu.........   4     0    TE2
..................................................guuaacuguaagacugugucu..........   1     0    TE2
```

miRBase precursor : aga-mir-low-compl
Total read count : 1717
aga-mir-low-compl read count : 1645
aga-mir-low-compl-star read count : 2
remaining reads : 70

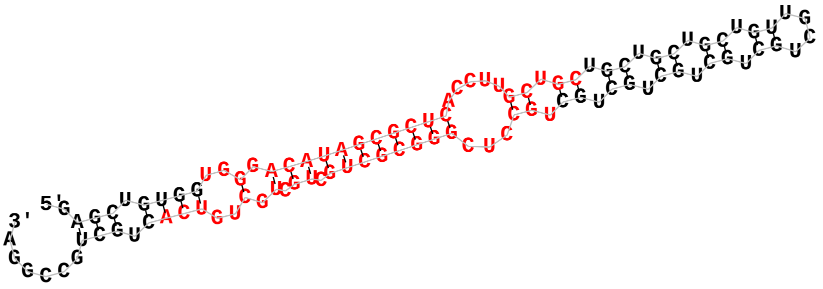

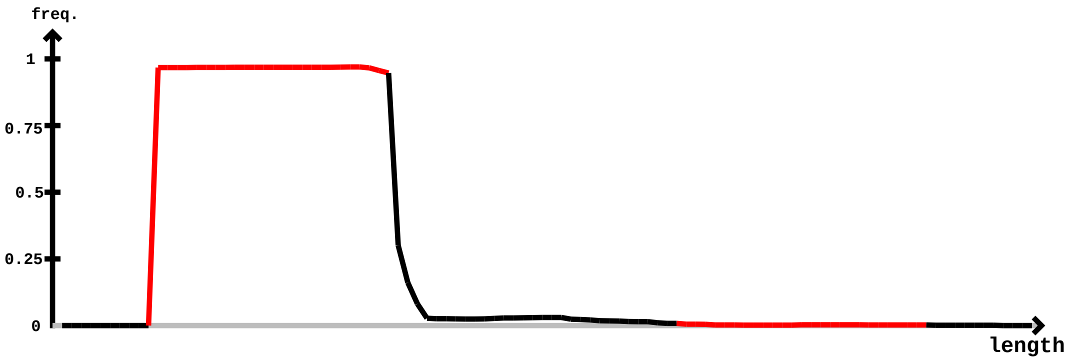


```
                aga-mir-low-compl
                                                           aga-mir-low-compl-star
5'- gagcuguggugggacauagcgcucaccuugcugcugcugcugcuguugcugcugcugcugcugccucgggcgcugcugcugcugucacugcugccgga   -3'    exp
    .(((.((((..(.(((((((((((.....((.((.((.((.((.((....)).)).)).)).)).))...)))))))).)).).)..)))).)))......    reads    mm    sample
    ............................................guugAugcugcugcugcugc.....................................    1        1     FF2

    .........ugggacauaAcgcucaccuugcugcu..................................................................    1        1     TE1
    .......................................cugcuguugcugcugcug............................................    1        0     TE1

    .........ugggacauagcgcucaccuugc......................................................................    2        0     OV2
    .........ugggacauagcgcucaccuugcu.....................................................................    7        0     OV2
    .........ugggacauagcgcucaccuugcug....................................................................    7        0     OV2
    .........ugggacauagAgcucaccuugcugc...................................................................    1        1     OV2
    .........ugggacaCagcgcucaccuugcugc...................................................................    1        1     OV2
    .........ugggacauaAcgcucaccuugcugc...................................................................    34       1     OV2
    .........ugggacauagcgcucaccuugcugc...................................................................    257      0     OV2
    .........ugggacauagUgcucaccuugcugc...................................................................    1        1     OV2
    .........ugggacauagcgcucaccuugcugcu..................................................................    45       0     OV2
    .........ugggacauagcgcucaccuugcugcG..................................................................    1        1     OV2
    .........ugggacauaAcgcucaccuugcugcu..................................................................    10       1     OV2
    .........ugggacauagcgcucaccuugcugcuA.................................................................    9        1     OV2
    .........ugggacauagcgcucaccuugcGgcug.................................................................    1        1     OV2
    .........ugggacauaAcgcucaccuugcugcug.................................................................    4        1     OV2
    .........ugggacauagcgcucaccuugcugcug.................................................................    32       0     OV2
    .........ugggacauaAcgcucaccuugcugcugc................................................................    3        1     OV2
    .........ugggacauagcgcucaccuugcugcugc................................................................    17       0     OV2
    .........ugggacauagcgcucaccuugcCgcugc................................................................    1        1     OV2
    .........ugggacauagcgcucaccuugcugcugcuA..............................................................    1        1     OV2
    .........ugggacauagcgcucaccuugcugcugcugc.............................................................    2        0     OV2
    .........ugggacauagcgcucaccuugcugcugcugcug...........................................................    1        0     OV2
    .........ugggacauagcgcucaccuugcugcugcugcugcu.........................................................    1        0     OV2
    .........ugggacauagcgcucaccuugcugcugcugcugcuguugcugcugcugc..........................................    1        0     OV2
    ................................gcugcugcugcuUuugcugcug..............................................    1        1     OV2
    .................................cugcugcuguAgcugc....................................................    1        1     OV2
    ..................................ugcugcuguAgcugc....................................................    1        1     OV2
    ...................................gcugcuguAgcugc....................................................    1        1     OV2
    ........................................ugcuAuugcugcugcugc...........................................    1        1     OV2
    ........................................ugcuguugGugcugcugcugcu.......................................    1        1     OV2
```

```
         aga-mir-low-compl
                                                                aga-mir-low-compl-star
gagcuguggugggacauagcgcucaccuugcugcugcugcugcuguugcugcugcugcugcugcugccucgggcgcugcugcugcugucacugcugccgga

............................................guugcugcugcGgcugc........................................    1    1    OV2
.............................................................ugcugccuGgggcgcugcugcu..................    1    1    OV2

..............................................ugcugcugcugcuAcugc.....................................    2    1    MF2
..............................................ugcugcugcugcuCcugcug...................................    1    1    MF2
.................................................ugcugcugcugcuAcugc..................................    2    1    MF2

.........ugggacauagcgcucaccuugc......................................................................    6    0    OV1
.........ugggacauagcgcucaccuugcu.....................................................................    7    0    OV1
.........ugggacauaAcgcucaccuugcug....................................................................    1    1    OV1
.........ugggacauagcgcucaccuugcug....................................................................    11   0    OV1
.........ugggacauagcgGucaccuugcugc...................................................................    1    1    OV1
.........ugggacauagcgcucaccuugcugc...................................................................    584  0    OV1
.........ugggacauagcUcucaccuugcugc...................................................................    1    1    OV1
.........ugggacauagcgcucaccuugcuAc...................................................................    1    1    OV1
.........ugggacauaAcgcucaccuugcugc...................................................................    52   1    OV1
.........ugggacGuagcgcucaccuugcugc...................................................................    1    1    OV1
.........ugggacauagcAcucaccuugcugc...................................................................    1    1    OV1
.........ugggacauagcgcucCccuugcugc...................................................................    1    1    OV1
.........ugggacauagcgcCcaccuugcugc...................................................................    1    1    OV1
.........ugggacauagcgcucaccuugcugcC..................................................................    1    1    OV1
.........ugggacauagcgcucaccuugcugcA..................................................................    1    1    OV1
.........ugggacauagcgcucaccuugcugcu..................................................................    118  0    OV1
.........ugggacauaAcgcucaccuugcugcu..................................................................    14   1    OV1
.........ugggacauagcgcucaccuugcugcuA.................................................................    10   1    OV1
.........ugggacauaAcgcucaccuugcugcug.................................................................    7    1    OV1
.........ugggacauagcgcucaccuugcugcug.................................................................    52   0    OV1
.........ugggacauagcgcucaccuugGugcug.................................................................    1    1    OV1
.........ugggacauaAcgcucaccuugcugcugc................................................................    4    1    OV1
.........ugggacauagcgcucaccuugcugcugc................................................................    41   0    OV1
.........ugggacauagcgcucaccuugcugcugcu...............................................................    1    0    OV1
.........ugggacauagcgcucaccuugcugcugcug..............................................................    1    0    OV1
.........ugggacauagcgcucaccuugcugcugcugc.............................................................    2    0    OV1
.........ugggacauagcgcucaccuugcugcugcugcu............................................................    1    0    OV1
.........ugggacauagcgcucaccuugcugcugcugcugcuguugcugc.................................................    1    0    OV1
.........ugggacauagcgcucaccuugcugcugcugcugcuguugcugcugcugc...........................................    1    0    OV1
............................ugcugcugcugcugcuguugcu...................................................    1    0    OV1
...............................ugcugcugcugcuguAgcugc.................................................    1    1    OV1
.................................cugcugcugcuguAgcugc.................................................    2    1    OV1
.................................Uugcugcugcuguugcugc.................................................    1    1    OV1
..................................ugcugcugcuguuAcugcugc..............................................    2    1    OV1
...................................gcugcugcuguAgcugc.................................................    1    1    OV1
....................................cugcugUuguugcugcugcu.............................................    1    1    OV1
.......................................cugcuguugUugcugcugcu..........................................    1    1    OV1
........................................ugUuguugcugcugcugcugc........................................    1    1    OV1
........................................ugcuguugGugcugcugcugc........................................    1    1    OV1
........................................ugcuAuugcugcugcugcugcu.......................................    1    1    OV1
...........................................uguugUugcugcugcugcugc.....................................    1    1    OV1
.............................................uugcugcugcugUugcugc.....................................    1    1    OV1
...............................................Acugcugcugcugcugc.....................................    2    1    OV1
................................................cugcugcugcugcuAcugc..................................    1    1    OV1
..................................................Acugcugcugcugcugc..................................    2    1    OV1
................................................................ugccucgggcgcugcugcugcugucU...........    1    1    OV1
................................................................ugccucgggcgcugcugcugcuguca...........    1    0    OV1
............................................................................ugcugcugcuguUacugcugc....    2    1    OV1

...........................................uguugAugcugcugcugc........................................    2    1    MF1
............................................guugAugcugcugcugc........................................    1    1    MF1

.........ugggacauagcgcucaccuugc......................................................................    1    0    BF2
.........ugggacauagcgcucaccuugcu.....................................................................    2    0    BF2
.........ugggacauagcgcucaccuugcugc...................................................................    56   0    BF2
.........ugggacauagcgcucaccCugcugc...................................................................    1    1    BF2
.........ugggacauaAcgcucaccuugcugc...................................................................    4    1    BF2
.........ugggacauagcgcucaccuugcugcu..................................................................    20   0    BF2
.........ugggacauaAcgcucaccuugcugcu..................................................................    3    1    BF2
.........ugggacauagcgcucaccuugcugcug.................................................................    6    0    BF2
.........ugggacauagcgcucaccuugcugcuA.................................................................    2    1    BF2
.........ugggacauGgcgcucaccuugcugcugc................................................................    1    1    BF2
```

```
         aga-mir-low-compl
                                                                aga-mir-low-compl-star
gagcuguggugggacauagcgcucaccuugcugcugcugcugcuguugcugcugcugcugcugcugccucgggcgcugcugcugcugucacugcugccgga

.........ugggacauagcgcucaccuugcugcugc................................................................   6   0   BF2
.........ugggacauaAcgcucaccuugcugcugc................................................................   5   1   BF2
.........ugggacauagcgcucaccuugcugcugcu...............................................................   1   0   BF2
.........ugggacauagcgcucaccuugcugcugcugc.............................................................   1   0   BF2
.........ugggacauagcgcucaccuugcugcugcugcugc..........................................................   1   0   BF2
.............acauagcgcucaccuugcugcugcugc.............................................................   1   0   BF2
.................agcgcucaccuugcugcugcugcugc..........................................................   1   0   BF2
.............................Ucugcugcugcugcuguugcugcug...............................................   1   1   BF2
...............................ugcugcugcugcuguugcG...................................................   1   1   BF2
...............................ugcugcugcugUuguugcugc.................................................   1   1   BF2
..................................ugcugcugcuguugUugcugc..............................................   1   1   BF2
...................................gcugcugcuguAgcugc.................................................   1   1   BF2
....................................cugcugcuguuUcugcu................................................   1   1   BF2
.....................................ugcugcuguugGugcugcugcugcu.......................................   2   1   BF2
..................................................gcugcugcugcugcugGcuc...............................   1   1   BF2

.........ugggacauagcgcucaccuugc......................................................................   1   0   BF1
.........ugggacauaAcgcucaccuugcu.....................................................................   2   1   BF1
.........ugggacauagcgcucaccuugcug....................................................................   1   0   BF1
.........ugggacauagcgcucaccuugcugc...................................................................  96   0   BF1
.........ugggacauaAcgcucaccuugcugc...................................................................  20   1   BF1
.........ugggacauaAcgcucaccuugcugcu..................................................................   1   1   BF1
.........ugggacauagcgcucaccuugcugcu..................................................................  26   0   BF1
.........ugggacauagcgcucaccuugcugcA..................................................................   1   1   BF1
.........ugggacauagcgcucaccuugcugcug.................................................................  15   0   BF1
.........ugggacauagcgcucaccuugcugcugc................................................................  11   0   BF1
.........ugggacauaAcgcucaccuugcugcugc................................................................   5   1   BF1
.........ugggacauagcgcucaccuugcugcugcu...............................................................   1   0   BF1
.........ugggacauagcgcucaccuugcugcugcugcugcugC.......................................................   2   1   BF1
.........ugggacauagcgcucaccuugcugcugcugcugcuguugcugcu................................................   1   0   BF1
.........ugggacauagcgcucaccuugcugcugcugcugcuguugcugcugc..............................................   1   0   BF1
..................................ugcugcugcuguugcugcugUugc...........................................   1   1   BF1
.............................................uugGugcugcugcugcugcugc..................................   1   1   BF1

..........................................Uuguugcugcugcugcugc........................................   1   1   FW1
.............................................uugUugcugcugcugcugc.....................................   1   1   FW1

.........ugggacauagcgcucaccuugcugc...................................................................   1   0   TE2
.........ugggacauaAcgcucaccuugcugcu..................................................................   1   1   TE2
.........ugggacauaAcgcucaccuugcugcugc................................................................   1   1   TE2
...............................ugcugcugcugcugAugcugcug...............................................   1   1   TE2
...................................gUugcugcuguugcugcugc..............................................   1   1   TE2
............................................guugcugcugUugcugcugc.....................................   1   1   TE2
```
