# Supplementary material for: The germline of the malaria mosquito produces abundant miRNAs, endo-siRNAs, piRNAs and 29-nt small RNAs
Source: BMC Genomics. 2015 Feb 19;16(1):100. doi: 10.1186/s12864-015-1257-2 (PMC4345017; doi:10.1186/s12864-015-1257-2)
Supplement: Additional file 8: — a) comparison of fold changes in mIR expression between male and female gonads (Testes (TE), non-bloodfed ovaries (OV) and bloodfed ovaries (BF)) as calculated from RNA-seq data (DESeq) and qRT-PCR. Two classes of gonad-enriched mIRs were identified, those enriched in the testes only and those enriched in the testes and bloodfed vitellogenic ovaries, but not pre-vitellogenic ovaries, that likely have a role in gametogenesis. (b) Fold changes calculated by the two methods generally showed good correlation. (c) Fold change (log2) increase in testis expression vs carcass expression is shown for two testis-specific miRNAs. [file 12864_2015_1257_MOESM8_ESM.pptx]

## Slide 1
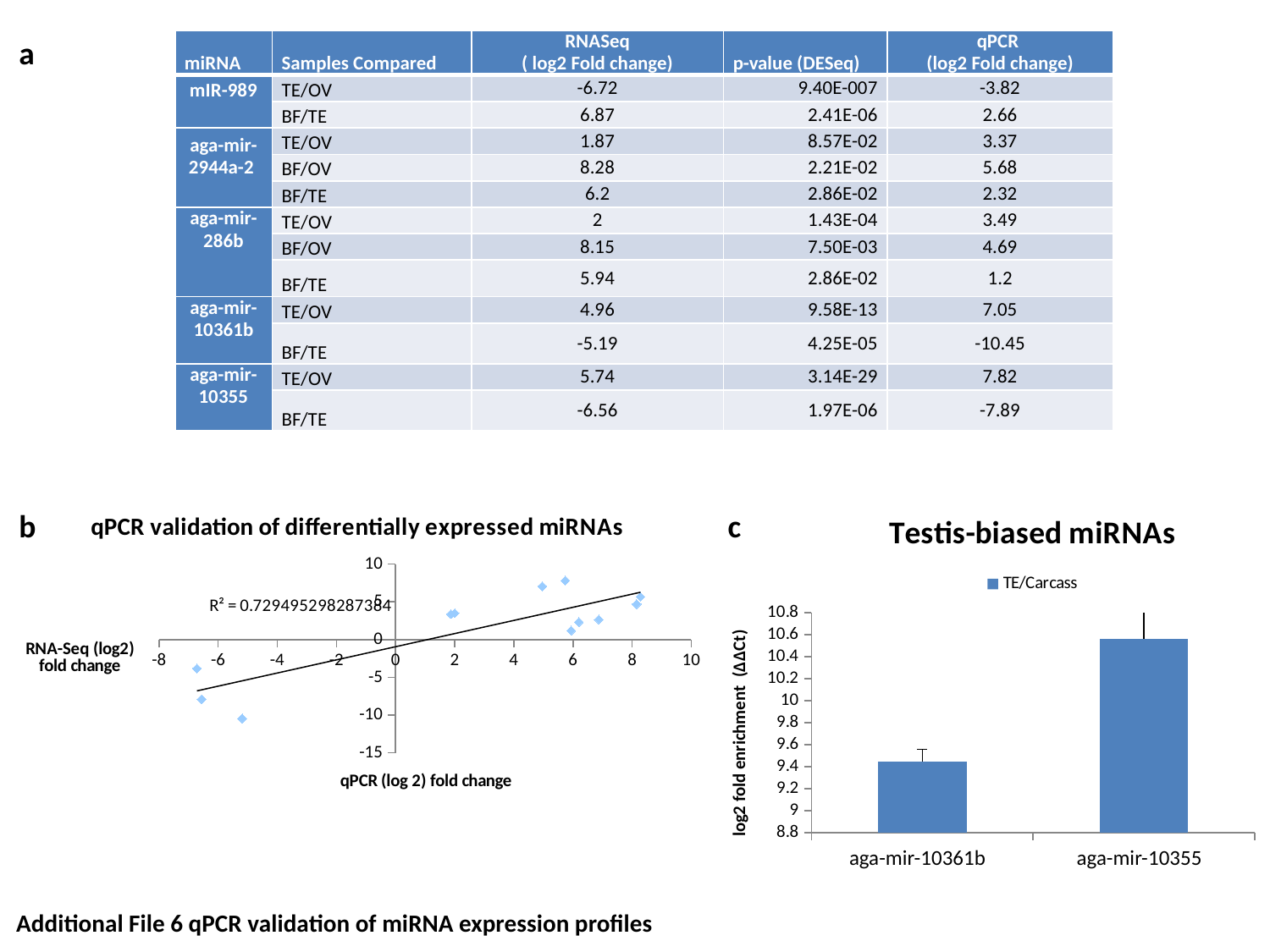

a
| miRNA | Samples Compared | RNASeq ( log2 Fold change) | p-value (DESeq) | qPCR (log2 Fold change) |
| --- | --- | --- | --- | --- |
| mIR-989 | TE/OV | -6.72 | 9.40E-007 | -3.82 |
| | BF/TE | 6.87 | 2.41E-06 | 2.66 |
| aga-mir-2944a-2 | TE/OV | 1.87 | 8.57E-02 | 3.37 |
| | BF/OV | 8.28 | 2.21E-02 | 5.68 |
| | BF/TE | 6.2 | 2.86E-02 | 2.32 |
| aga-mir-286b | TE/OV | 2 | 1.43E-04 | 3.49 |
| | BF/OV | 8.15 | 7.50E-03 | 4.69 |
| | BF/TE | 5.94 | 2.86E-02 | 1.2 |
| aga-mir-10361b | TE/OV | 4.96 | 9.58E-13 | 7.05 |
| | BF/TE | -5.19 | 4.25E-05 | -10.45 |
| aga-mir-10355 | TE/OV | 5.74 | 3.14E-29 | 7.82 |
| | BF/TE | -6.56 | 1.97E-06 | -7.89 |
### Chart: qPCR validation of differentially expressed miRNAs
| Category | qPCR log2 Fold change |
|---|---|
### Chart: Testis-biased miRNAs
| Category | TE/Carcass |
|---|---|
| X_43358 | 9.447006225585938 |
| X_43370 | 10.557308197021484 |aga-mir-10361b
aga-mir-10355
b
c
Additional File 6 qPCR validation of miRNA expression profiles
