# Supplementary material for: The germline of the malaria mosquito produces abundant miRNAs, endo-siRNAs, piRNAs and 29-nt small RNAs
Source: BMC Genomics. 2015 Feb 19;16(1):100. doi: 10.1186/s12864-015-1257-2 (PMC4345017; doi:10.1186/s12864-015-1257-2)
Supplement: Additional file 9: — Read size distribution of small RNAs in total (green) and with the miRNA population removed (red) in each of the various samples (A-G) with and without (red) miRNAs. [file 12864_2015_1257_MOESM9_ESM.pptx]

## Slide 1
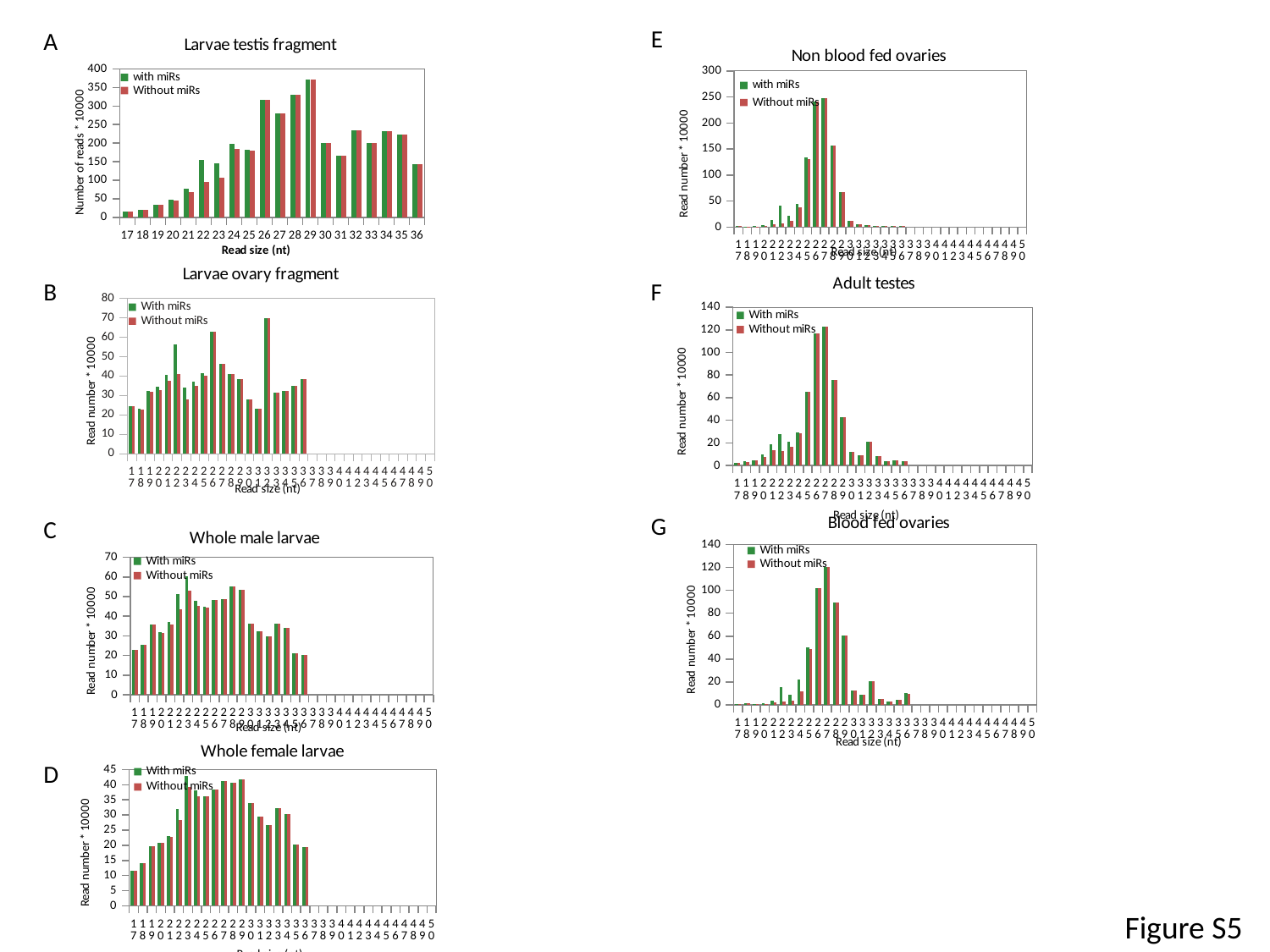

E
A
### Chart: Larvae testis fragment
| Category | with miRs | Without miRs |
|---|---|---|
| 17 | 16.5097 | 16.43209999999998 |
| 18 | 20.6935 | 20.48869999999982 |
| 19 | 34.9229 | 34.4629 |
| 20 | 46.7592 | 44.3454 |
| 21 | 76.35039999999998 | 67.37249999999995 |
| 22 | 154.5485 | 96.7081 |
| 23 | 145.1165 | 107.388 |
| 24 | 197.6182 | 184.0735 |
| 25 | 182.1954 | 179.0906 |
| 26 | 317.1479 | 316.9436999999999 |
| 27 | 280.9286 | 280.908 |
| 28 | 329.22 | 329.2044 |
| 29 | 372.4577 | 372.4468 |
| 30 | 200.8621 | 200.8572 |
| 31 | 167.337 | 167.3342 |
| 32 | 235.5828 | 235.5799 |
| 33 | 201.2183 | 201.2165 |
| 34 | 233.3195 | 233.3092 |
| 35 | 223.5736 | 223.5689 |
| 36 | 144.4496 | 144.4395 |
[unsupported chart]
[unsupported chart]
B
F
[unsupported chart]
G
C
[unsupported chart]
[unsupported chart]
[unsupported chart]
D
Figure S5
