# Supplementary material for: The germline of the malaria mosquito produces abundant miRNAs, endo-siRNAs, piRNAs and 29-nt small RNAs
Source: BMC Genomics. 2015 Feb 19;16(1):100. doi: 10.1186/s12864-015-1257-2 (PMC4345017; doi:10.1186/s12864-015-1257-2)
Supplement: Additional file 10: — cis-NAT-siRNAs size distribution on the various samples (A-G). [file 12864_2015_1257_MOESM10_ESM.pptx]

## Slide 1
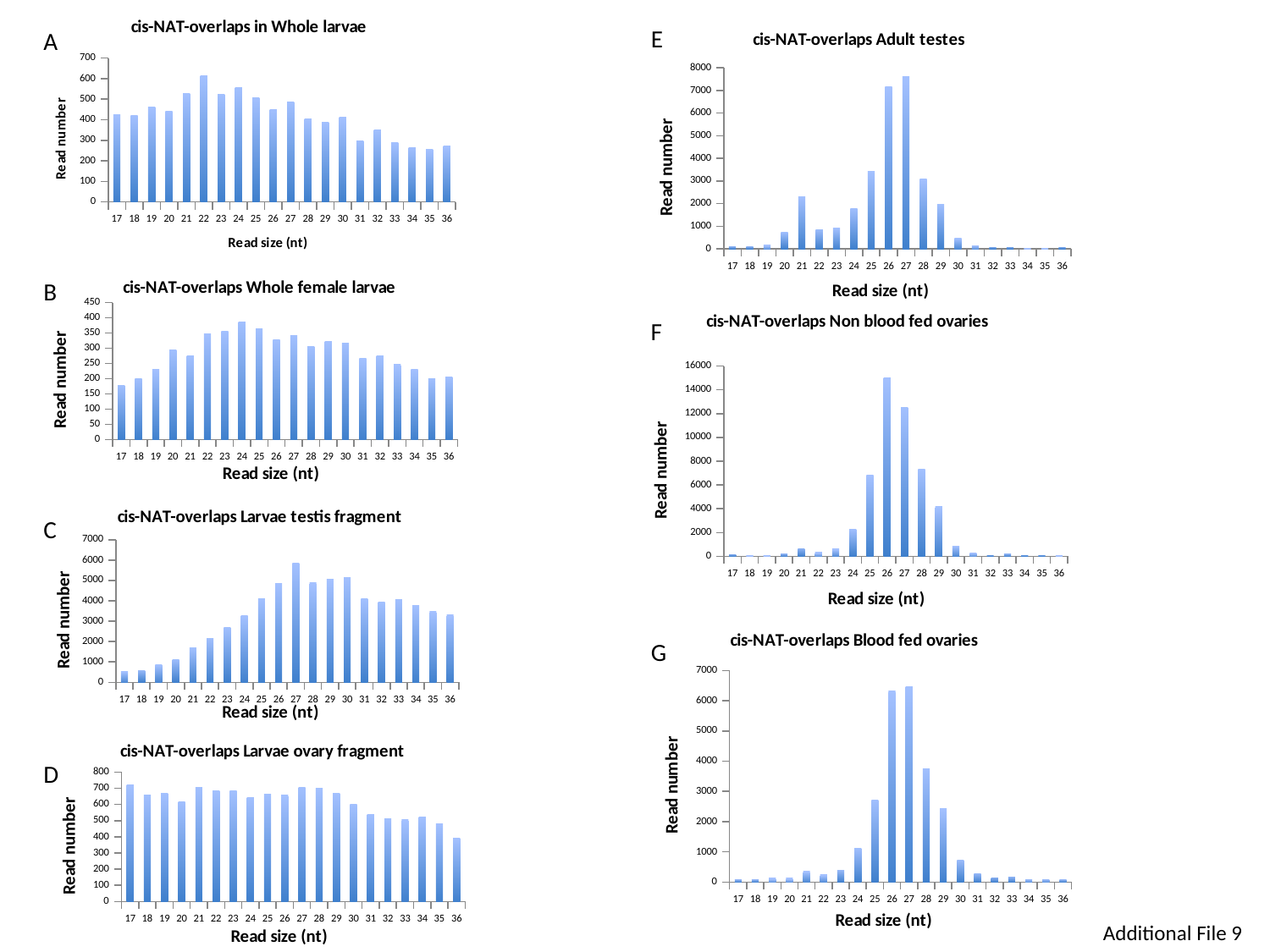

### Chart: cis-NAT-overlaps in Whole larvae
| Category | |
|---|---|
| 17 | 423.0 |
| 18 | 419.0 |
| 19 | 463.0 |
| 20 | 443.0 |
| 21 | 526.0 |
| 22 | 613.0 |
| 23 | 524.0 |
| 24 | 558.0 |
| 25 | 506.0 |
| 26 | 451.0 |
| 27 | 486.0 |
| 28 | 404.0 |
| 29 | 389.0 |
| 30 | 414.0 |
| 31 | 296.0 |
| 32 | 349.0 |
| 33 | 291.0 |
| 34 | 265.0 |
| 35 | 257.0 |
| 36 | 272.0 |E
A
### Chart: cis-NAT-overlaps Adult testes
| Category | |
|---|---|
| 17 | 106.0 |
| 18 | 92.0 |
| 19 | 168.0 |
| 20 | 725.0 |
| 21 | 2317.0 |
| 22 | 859.0 |
| 23 | 906.0 |
| 24 | 1758.0 |
| 25 | 3434.0 |
| 26 | 7177.0 |
| 27 | 7602.0 |
| 28 | 3089.0 |
| 29 | 1955.0 |
| 30 | 455.0 |
| 31 | 120.0 |
| 32 | 54.0 |
| 33 | 57.0 |
| 34 | 30.0 |
| 35 | 21.0 |
| 36 | 42.0 |B
### Chart: cis-NAT-overlaps Whole female larvae
| Category | |
|---|---|
| 17 | 178.0 |
| 18 | 201.0 |
| 19 | 231.0 |
| 20 | 294.0 |
| 21 | 275.0 |
| 22 | 346.0 |
| 23 | 357.0 |
| 24 | 387.0 |
| 25 | 364.0 |
| 26 | 328.0 |
| 27 | 343.0 |
| 28 | 305.0 |
| 29 | 321.0 |
| 30 | 316.0 |
| 31 | 268.0 |
| 32 | 274.0 |
| 33 | 246.0 |
| 34 | 230.0 |
| 35 | 201.0 |
| 36 | 205.0 |
### Chart: cis-NAT-overlaps Non blood fed ovaries
| Category | |
|---|---|
| 17 | 152.0 |
| 18 | 45.0 |
| 19 | 67.0 |
| 20 | 192.0 |
| 21 | 663.0 |
| 22 | 329.0 |
| 23 | 650.0 |
| 24 | 2287.0 |
| 25 | 6828.0 |
| 26 | 15016.0 |
| 27 | 12527.0 |
| 28 | 7307.0 |
| 29 | 4156.0 |
| 30 | 821.0 |
| 31 | 256.0 |
| 32 | 96.0 |
| 33 | 197.0 |
| 34 | 87.0 |
| 35 | 85.0 |
| 36 | 67.0 |F
### Chart: cis-NAT-overlaps Larvae testis fragment
| Category | |
|---|---|
| 17 | 508.0 |
| 18 | 573.0 |
| 19 | 850.0 |
| 20 | 1120.0 |
| 21 | 1709.0 |
| 22 | 2167.0 |
| 23 | 2689.0 |
| 24 | 3278.0 |
| 25 | 4108.0 |
| 26 | 4841.0 |
| 27 | 5864.0 |
| 28 | 4883.0 |
| 29 | 5040.0 |
| 30 | 5125.0 |
| 31 | 4104.0 |
| 32 | 3947.0 |
| 33 | 4052.0 |
| 34 | 3765.0 |
| 35 | 3466.0 |
| 36 | 3322.0 |C
### Chart: cis-NAT-overlaps Blood fed ovaries
| Category | |
|---|---|
| 17 | 84.0 |
| 18 | 77.0 |
| 19 | 142.0 |
| 20 | 140.0 |
| 21 | 375.0 |
| 22 | 251.0 |
| 23 | 399.0 |
| 24 | 1111.0 |
| 25 | 2711.0 |
| 26 | 6310.0 |
| 27 | 6450.0 |
| 28 | 3760.0 |
| 29 | 2443.0 |
| 30 | 739.0 |
| 31 | 267.0 |
| 32 | 150.0 |
| 33 | 156.0 |
| 34 | 90.0 |
| 35 | 95.0 |
| 36 | 83.0 |G
### Chart: cis-NAT-overlaps Larvae ovary fragment
| Category | |
|---|---|
| 17 | 723.0 |
| 18 | 661.0 |
| 19 | 668.0 |
| 20 | 619.0 |
| 21 | 705.0 |
| 22 | 686.0 |
| 23 | 684.0 |
| 24 | 643.0 |
| 25 | 662.0 |
| 26 | 659.0 |
| 27 | 705.0 |
| 28 | 700.0 |
| 29 | 670.0 |
| 30 | 600.0 |
| 31 | 539.0 |
| 32 | 512.0 |
| 33 | 506.0 |
| 34 | 522.0 |
| 35 | 481.0 |
| 36 | 392.0 |D
Additional File 9
