# Supplementary figures and images for: The germline of the malaria mosquito produces abundant miRNAs, endo-siRNAs, piRNAs and 29-nt small RNAs
Source: BMC Genomics. 2015 Feb 19;16(1):100. doi: 10.1186/s12864-015-1257-2 (PMC4345017; doi:10.1186/s12864-015-1257-2)

## Slide 1
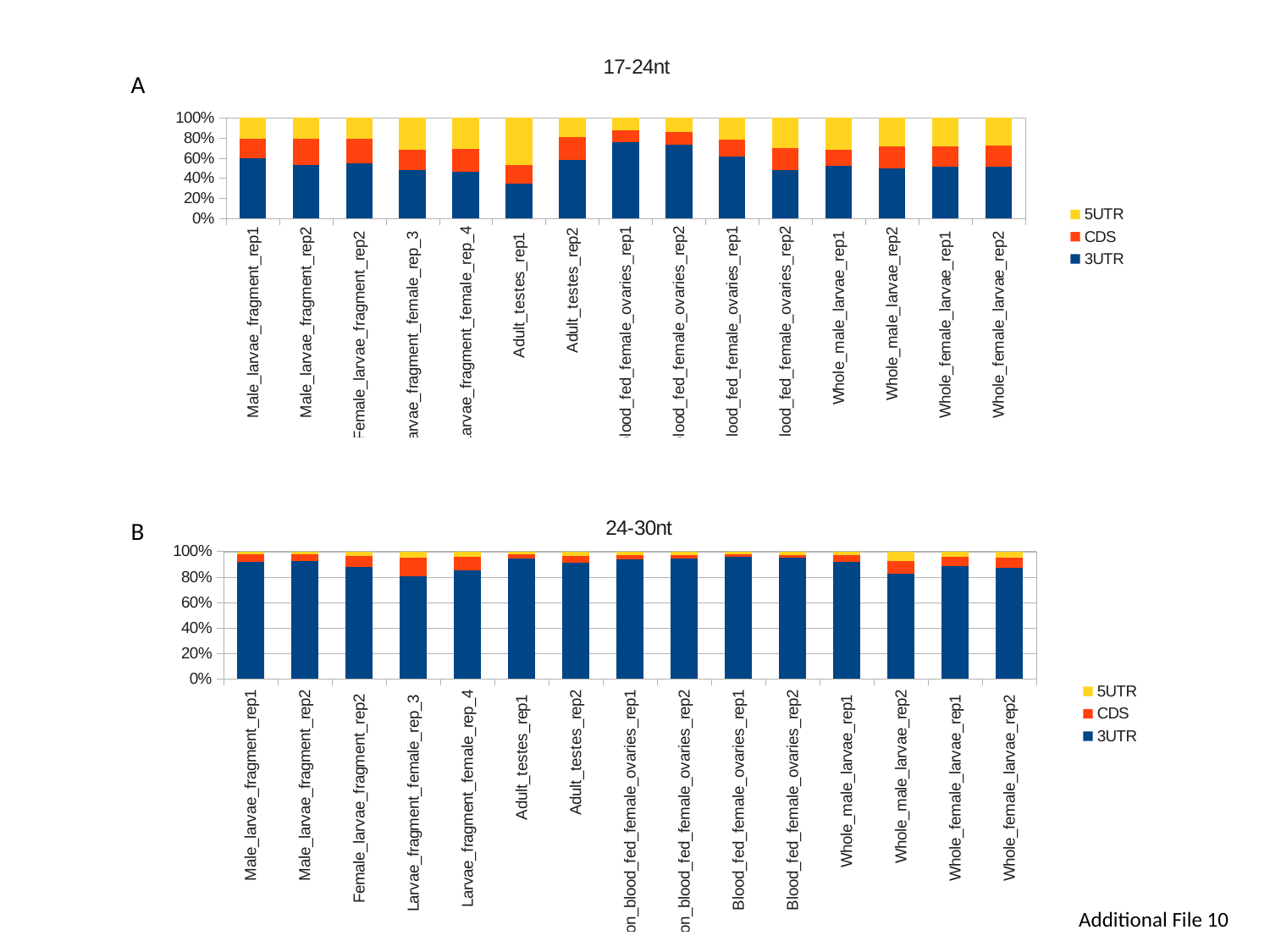

A
B
Additional File 10

Supplement: Additional file 11: — (A) Distribution of the reads from 21 to 24-nt long on the 5UTR, CDS and 3UTR of mRNAs normalized for their genomic coverage. (B) Distribution of the reads from 24 to 30-nt long on the 5UTR, CDS and 3UTR of mRNAs normalized for their genomic coverage. [file 12864_2015_1257_MOESM11_ESM.pptx]

## Slide 1
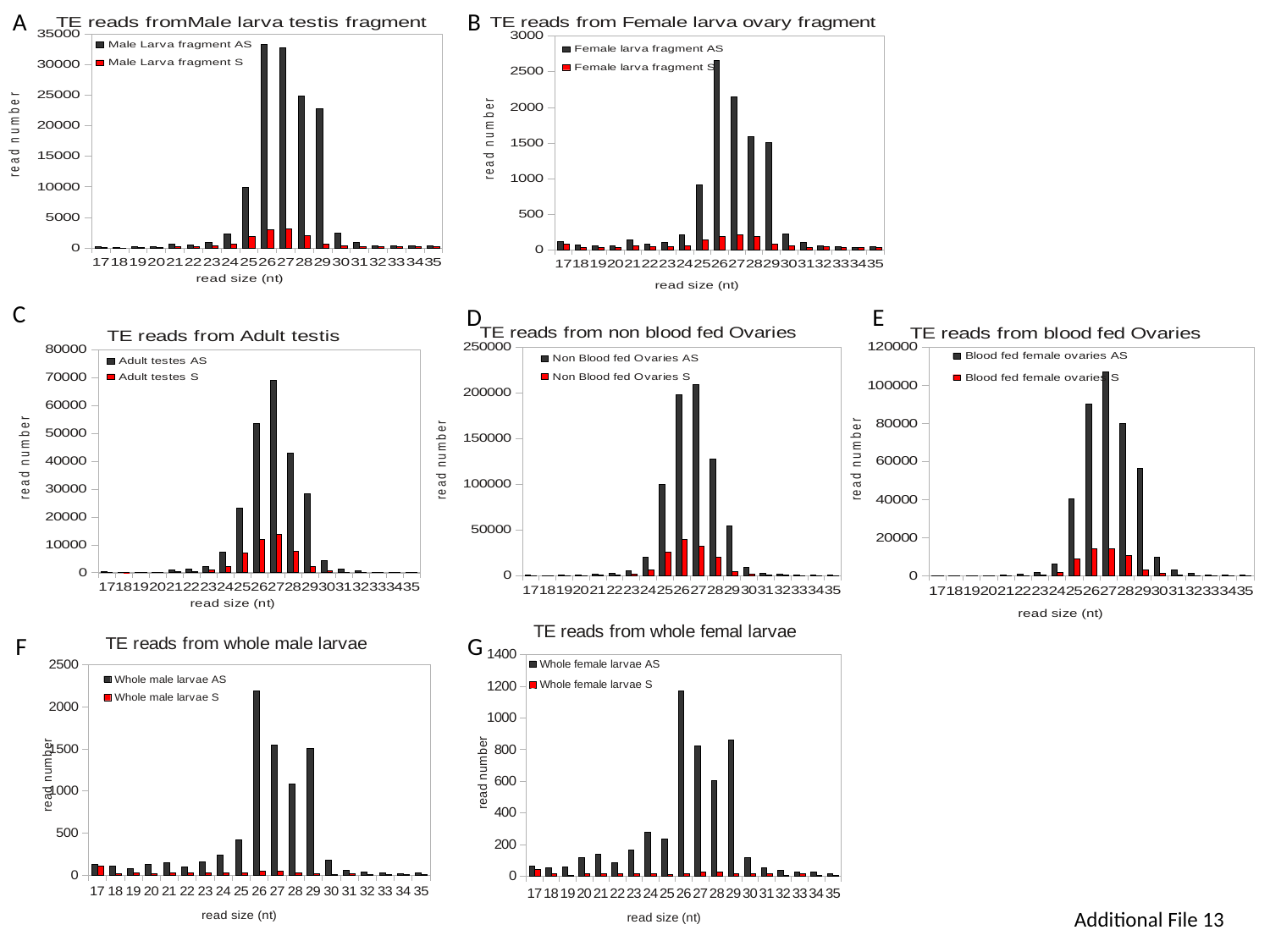

B
A
C
D
E
F
G
Additional File 13

Supplement: Additional file 14: — Nucleotide size distribution of reads from Anopheles antisense (grey) and sense (red) TEs using reads from the various samples (A-G). [file 12864_2015_1257_MOESM14_ESM.pptx]

## Slide 1
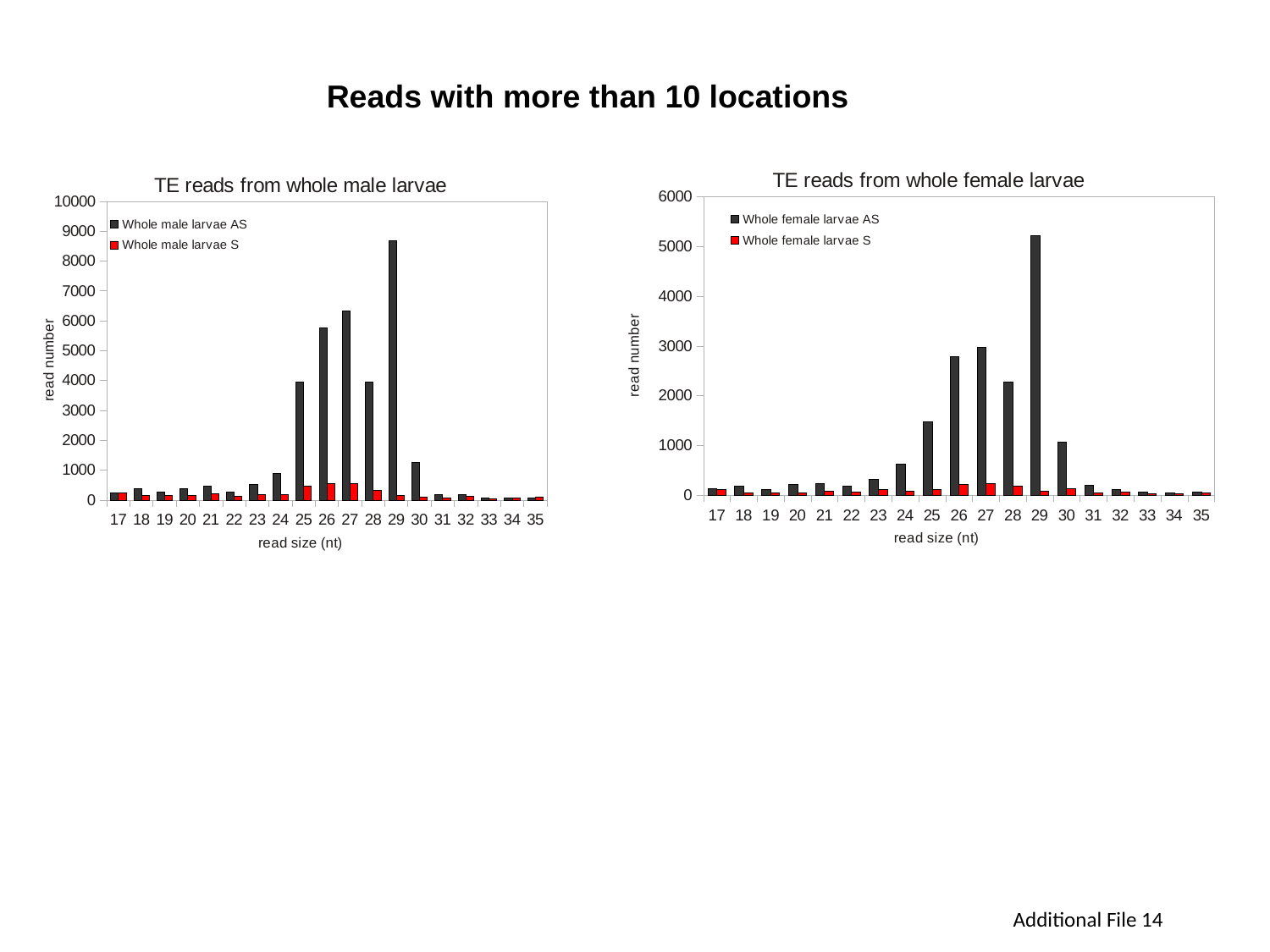

Reads with more than 10 locations
Additional File 14

Supplement: Additional file 15: — Nucleotide size distribution of reads that map on more than 10 locations from Anopheles antisense (grey) and sense (red) TEs, from whole male (left) and female (right) larvae. [file 12864_2015_1257_MOESM15_ESM.pptx]
