# Supplementary material for: The germline of the malaria mosquito produces abundant miRNAs, endo-siRNAs, piRNAs and 29-nt small RNAs
Source: BMC Genomics. 2015 Feb 19;16(1):100. doi: 10.1186/s12864-015-1257-2 (PMC4345017; doi:10.1186/s12864-015-1257-2)
Supplement: Additional file 12: — Size distribution small RNAs mapping to coding transcripts and the relative distribution in the 3’ UTR (red) compared to the entire mRNA (blue) in the following representative samples: (A) whole female larvae, (B) non blood-fed ovaries, (C) larvae ovary fragment and (D) adult testes. [file 12864_2015_1257_MOESM12_ESM.pptx]

## Slide 1
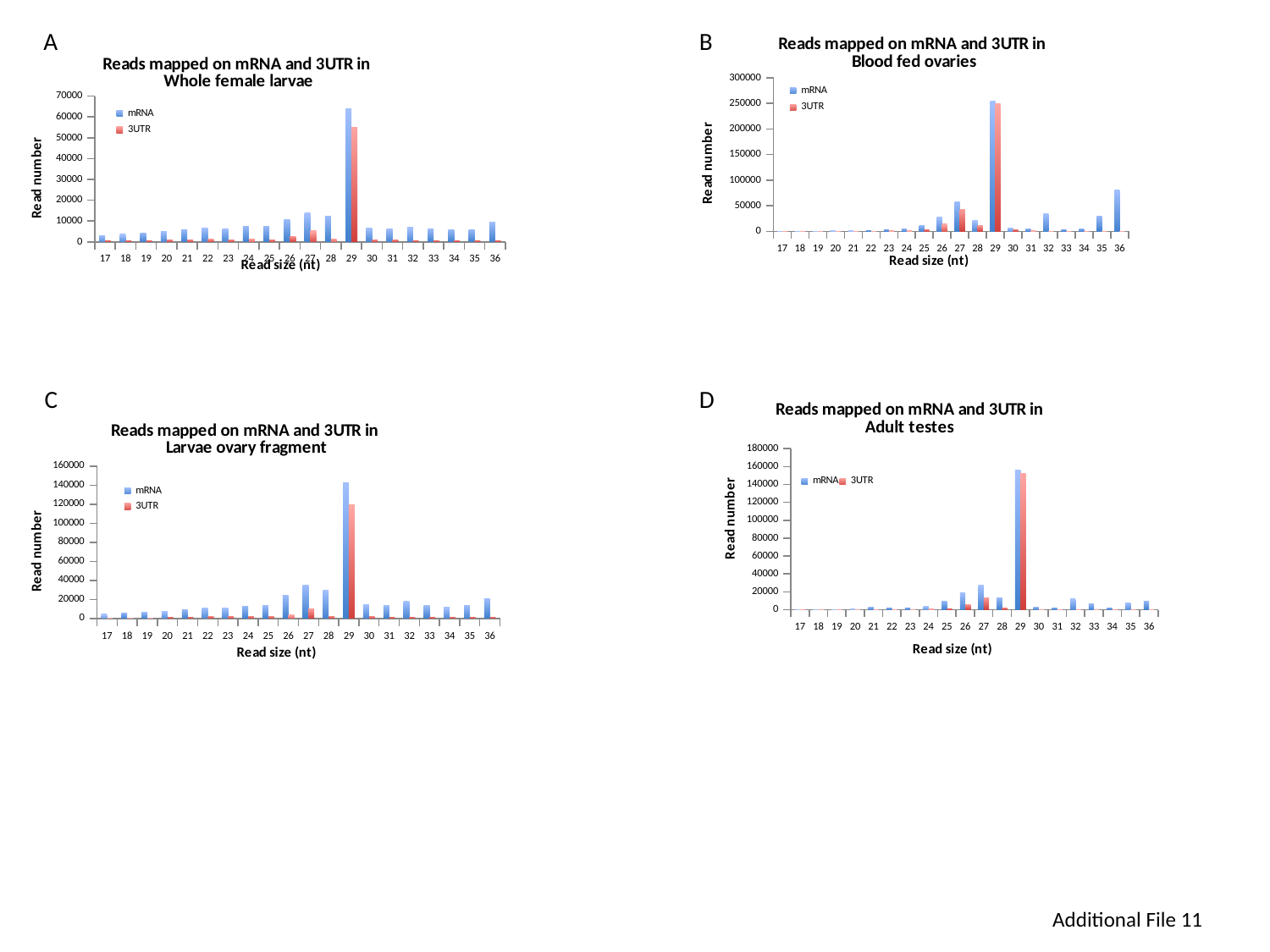

A
B
### Chart: Reads mapped on mRNA and 3UTR in
Blood fed ovaries
| Category | | |
|---|---|---|
| 17 | 208.0 | 36.0 |
| 18 | 332.0 | 48.0 |
| 19 | 489.0 | 73.0 |
| 20 | 809.0 | 140.0 |
| 21 | 1395.0 | 355.0 |
| 22 | 1932.0 | 655.0 |
| 23 | 2433.0 | 759.0 |
| 24 | 4222.0 | 1104.0 |
| 25 | 11893.0 | 2844.0 |
| 26 | 27885.0 | 14604.0 |
| 27 | 57777.0 | 43210.0 |
| 28 | 22054.0 | 10973.0 |
| 29 | 254105.0 | 249010.0 |
| 30 | 6176.0 | 2605.0 |
| 31 | 4100.0 | 774.0 |
| 32 | 34050.0 | 475.0 |
| 33 | 3816.0 | 451.0 |
| 34 | 4100.0 | 435.0 |
| 35 | 29571.0 | 417.0 |
| 36 | 81491.0 | 451.0 |
### Chart: Reads mapped on mRNA and 3UTR in
Whole female larvae
| Category | | |
|---|---|---|
| 17 | 3065.0 | 477.0 |
| 18 | 3671.0 | 583.0 |
| 19 | 4228.0 | 669.0 |
| 20 | 5015.0 | 842.0 |
| 21 | 6029.0 | 860.0 |
| 22 | 6697.0 | 1191.0 |
| 23 | 6377.0 | 952.0 |
| 24 | 7451.0 | 1286.0 |
| 25 | 7457.0 | 1121.0 |
| 26 | 10897.0 | 2673.0 |
| 27 | 13921.0 | 5399.0 |
| 28 | 12547.0 | 1401.0 |
| 29 | 63900.0 | 54889.0 |
| 30 | 6818.0 | 976.0 |
| 31 | 6394.0 | 806.0 |
| 32 | 6855.0 | 730.0 |
| 33 | 6073.0 | 640.0 |
| 34 | 5714.0 | 580.0 |
| 35 | 5657.0 | 510.0 |
| 36 | 9351.0 | 533.0 |C
D
### Chart: Reads mapped on mRNA and 3UTR in
Larvae ovary fragment
| Category | | |
|---|---|---|
| 17 | 4322.0 | 447.0 |
| 18 | 5195.0 | 541.0 |
| 19 | 6199.0 | 641.0 |
| 20 | 7410.0 | 876.0 |
| 21 | 9261.0 | 1103.0 |
| 22 | 10832.0 | 1837.0 |
| 23 | 11107.0 | 1597.0 |
| 24 | 12935.0 | 1779.0 |
| 25 | 13787.0 | 1837.0 |
| 26 | 24038.0 | 3758.0 |
| 27 | 34612.0 | 10067.0 |
| 28 | 29668.0 | 2269.0 |
| 29 | 142646.0 | 119276.0 |
| 30 | 14276.0 | 1643.0 |
| 31 | 13656.0 | 1500.0 |
| 32 | 18395.0 | 1488.0 |
| 33 | 13638.0 | 1360.0 |
| 34 | 11887.0 | 1244.0 |
| 35 | 13852.0 | 1121.0 |
| 36 | 20497.0 | 1108.0 |
### Chart: Reads mapped on mRNA and 3UTR in
Adult testes
| Category | | |
|---|---|---|
| 17 | 396.0 | 62.0 |
| 18 | 430.0 | 31.0 |
| 19 | 491.0 | 42.0 |
| 20 | 698.0 | 56.0 |
| 21 | 2477.0 | 331.0 |
| 22 | 1591.0 | 466.0 |
| 23 | 1945.0 | 366.0 |
| 24 | 3796.0 | 571.0 |
| 25 | 9440.0 | 1220.0 |
| 26 | 19127.0 | 5787.0 |
| 27 | 27712.0 | 13623.0 |
| 28 | 13247.0 | 2095.0 |
| 29 | 156396.0 | 151975.0 |
| 30 | 2521.0 | 295.0 |
| 31 | 1851.0 | 118.0 |
| 32 | 12317.0 | 99.0 |
| 33 | 6255.0 | 75.0 |
| 34 | 2125.0 | 79.0 |
| 35 | 7546.0 | 74.0 |
| 36 | 9470.0 | 69.0 |Additional File 11
