# Supplementary material for: The germline of the malaria mosquito produces abundant miRNAs, endo-siRNAs, piRNAs and 29-nt small RNAs
Source: BMC Genomics. 2015 Feb 19;16(1):100. doi: 10.1186/s12864-015-1257-2 (PMC4345017; doi:10.1186/s12864-015-1257-2)
Supplement: Additional file 16: — Nucleotide bias of each position of 24 and 25-nt long piRNAs obtained from (A) PIWI, (B) AUB and (C) AGO3 Drosophila proteins. [file 12864_2015_1257_MOESM16_ESM.pptx]

## Slide 1
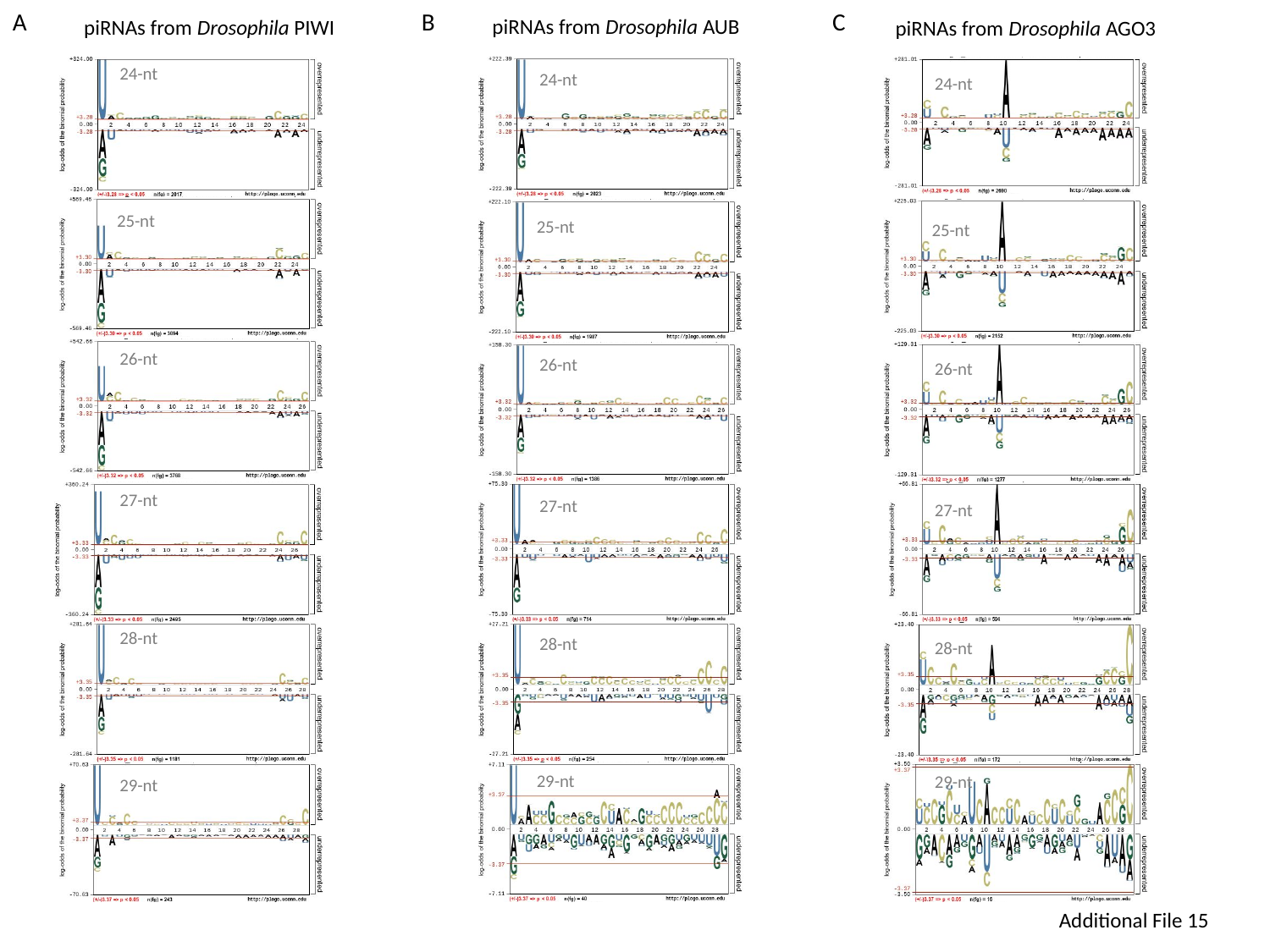

B
C
A
piRNAs from Drosophila AUB
piRNAs from Drosophila PIWI
piRNAs from Drosophila AGO3
24-nt
24-nt
24-nt
25-nt
25-nt
25-nt
26-nt
26-nt
26-nt
27-nt
27-nt
27-nt
28-nt
28-nt
28-nt
29-nt
29-nt
29-nt
Additional File 15
